# Hybrid Quantile-Expectile Error Layers for Technical-Efficiency Recovery in Stochastic Frontier Analysis

**DOI:** 10.3390/e28080881

**Published:** 2026-08-05

**Authors:** Shengming Wang, Yunquan Song, Juan Yu

**Affiliations:** College of Science, China University of Petroleum, Qingdao 266580, China

**Keywords:** stochastic frontier analysis, technical efficiency, HQER-SFA, quantile-expectile loss, robustness analysis

## Abstract

This paper develops and evaluates HQER-SFA, a likelihood-based stochastic frontier specification that embeds a hybrid quantile-expectile error layer into the bilateral noise component while preserving the standard one-sided inefficiency structure. The model nests Quantile-SFA when γ=0 and extends it by normalizing the hybrid loss into a proper bilateral error density, so that likelihood inference, residual decomposition, and technical-efficiency recovery remain in a unified stochastic-frontier framework. We compare HQER-SFA with Traditional-SFA and Quantile-SFA using three processed production modules, Monte Carlo parameter-inversion experiments, an expanded R=300 robustness design, γ sensitivity and ablation checks, convergence diagnostics, and a source-assisted small-sample extension. The results demonstrate clear gains for technical-efficiency recovery: in the R=300 design, HQER-SFA attains win rates of 0.5646 for technical-efficiency RMSE and 0.5578 for technical-efficiency rank correlation, and the agricultural module shows the strongest real-data improvement under the flexible bilateral error layer. The source-assisted analysis further shows that same-domain initialization improves small-sample validation RMSE by about 2.43%, while excessive source-centered penalties should be controlled. Overall, HQER-SFA provides an interpretable and computationally feasible extension for technical-efficiency recovery, especially when bilateral noise is asymmetric, tail-sensitive, or heterogeneous across production modules.

## 1. Introduction

Stochastic frontier analysis was introduced to separate production deviations into bilateral statistical noise and one-sided technical inefficiency [1,2]. Because technical efficiency is inferred rather than directly observed, conditional inefficiency recovery depends on how residual variation is allocated after the deterministic frontier has been fitted [3,4]. The central question in this paper is therefore not only whether a frontier predicts output well, but whether the bilateral error layer helps recover the latent inefficiency component in a stable and interpretable way.

The classical composed-error model provides the structural decomposition that the present paper preserves. Battese and Coelli [5,6] extended inefficiency-effect and panel-data formulations, and Greene [7] emphasized the role of heterogeneity in frontier estimation. Recent work continues to broaden the distributional and computational frontier literature through quantile stochastic frontiers [8,9,10,11], robust and finite-mixture likelihood analysis [12,13,14], semiparametric and regularized inefficiency recovery [15,16], and more flexible frontier specifications for distributional features, endogeneity, skewness, and multivariate settings [17,18,19,20,21]. Nonparametric and robust frontier methods also provide a related background for modeling production boundaries without relying exclusively on a single parametric mean structure [22,23,24,25,26].

Quantile and expectile ideas motivate the error-layer construction. Quantile regression models conditional distributional behavior beyond the conditional mean [27,28], while asymmetric Laplace likelihoods connect quantile loss to likelihood-based estimation [29,30]. Expectile methods, originating from asymmetric least-squares estimation [31,32], provide a complementary quadratic-loss view of tail-sensitive estimation [33,34,35]. The recent hybrid quantile-expectile regression literature [36] supplies a useful loss-function background, but the contribution here is not the proposal of a new loss function per se.

This paper contributes by formulating HQER-SFA as a stochastic-frontier likelihood with an HQER-induced bilateral error density, by clarifying its nesting relationship with Quantile-SFA, and by evaluating its behavior through real-data comparisons, Monte Carlo designs, γ sensitivity, source-assisted checks, and convergence diagnostics. The contribution is therefore the construction and evaluation of a normalized hybrid quantile-expectile bilateral density within the stochastic-frontier likelihood, while the one-sided inefficiency component remains structurally separate. The source-assisted component is motivated by the literature on transfer learning and source-assisted estimation, where external information may help or harm depending on source–target alignment [37,38,39,40,41,42,43]; it is used here to test whether related source estimates can further stabilize small-sample frontier estimation.

The interpretation is targeted rather than overgeneralized. Because HQER-SFA modifies the bilateral error law while retaining the economic frontier and one-sided inefficiency component, its main expected advantage is in residual allocation and posterior technical-efficiency recovery. Prediction RMSE and coefficient recovery are retained as important benchmarks, but the central empirical question is whether the additional error-layer flexibility improves the efficiency-recovery channel that classical and quantile-oriented stochastic frontiers leave more restricted.

## 2. Methodology

The methodological framework begins with the classical stochastic frontier benchmark and then introduces a normalized hybrid bilateral error layer. This section keeps the production-frontier decomposition explicit because the later empirical interpretation depends on distinguishing fitted-frontier accuracy from technical-efficiency recovery.

Because production data contain both bilateral statistical noise and one-sided inefficiency, Traditional-SFA provides the classical reference specification. For production unit *i*, the standard model is

(1) $$y_{i}=x_{i}^{⊤}\beta +v_{i}-u_{i}.$$

Here $y_{i}$ is the output, $x_{i}$ is the input vector, β is the frontier coefficient vector, $v_{i}$ is bilateral noise, and $u_{i}\geq 0$ is inefficiency. The benchmark distributional assumptions are

(2) $$v_{i}∼N\left(0,\sigma_{v}^{2}\right),\;u_{i}∼\left|N\left(0,\sigma_{u}^{2}\right)\right|.$$

Technical efficiency is defined as

(3) $$TE_{i}=\exp\left(-u_{i}\right).$$

In these expressions, $y_{i}$ denotes output, $x_{i}$ the input vector, β the frontier parameters, $v_{i}$ bilateral statistical noise, $u_{i}$ nonnegative inefficiency, and $\sigma_{v}$ and $\sigma_{u}$ the corresponding scale parameters. Technical efficiency is denoted by $TE_{i}$.

This benchmark places HQER-SFA relative to the standard Gaussian-noise stochastic frontier model rather than only relative to a quantile-oriented baseline. Quantile-SFA keeps the same frontier decomposition but specifies the bilateral error with an asymmetric quantile-oriented density. The quantile check loss is

(4) $$\rho_{\tau }\left(s\right)=s\left\{\tau -I\left(s<0\right)\right\},$$

where τ∈(0,1) controls asymmetry and I(·) is the indicator function. In the baseline specification,

(5) $$v_{i}∼ALD\left(0,\sigma_{v},\tau \right).$$

Quantile-SFA is the nested benchmark because it corresponds to the HQER-SFA boundary when the hybrid mixing parameter equals zero. To construct the hybrid layer, let *s* denote a standardized residual and define the asymmetric weight

(6) $$\Psi_{\tau }\left(s\right)=\tau I\left(s\geq 0\right)+\left(1-\tau \right)I\left(s<0\right).$$

The hybrid quantile-expectile loss is

(7) $$\begin{array}{cc} C_{\tau }^{\gamma }\left(s\right) & =\left(1-\gamma \right)\Psi_{\tau }\left(s\right)\left|s\right|+\gamma \Psi_{\tau }\left(s\right)s^{2}, \end{array}$$

where γ∈[0,1] controls the mixture of quantile-like absolute loss and expectile-like quadratic loss. The induced bilateral error density is

(8) $$\begin{array}{cc} f_{v}\left(v;\tau ,\gamma ,\sigma_{v}\right)\; & =\frac{1}{\sigma_{v}Z\left(\tau ,\gamma \right)}\exp\left[-C_{\tau }^{\gamma }\left(\frac{v}{\sigma_{v}}\right)\right], \end{array}$$

with the normalizing constant

(9) $$\begin{array}{cc} Z(\tau ,\gamma )\; & =\int_{-\infty }^{\infty }\exp\left[-C_{\tau }^{\gamma }\left(s\right)\right]\;ds. \end{array}$$

The normalizing constant is required because the loss defines the density shape but not its probability scale. It converts the hybrid quantile-expectile loss from a robust objective into a valid likelihood component, which is the key step that allows HQER-SFA to retain likelihood-based comparison and posterior inefficiency recovery.

The HQER-SFA likelihood embeds this induced bilateral density in the same composed-error structure. Let $\epsilon_{i}=y_{i}-x_{i}^{⊤}\beta$. Because inefficiency is latent, the observation-level likelihood integrates over u≥0:

(10) $$\begin{array}{cc} L_{i}\left(\theta \right) & =\int_{0}^{\infty }f_{v}\left(\epsilon_{i}+u;\tau ,\gamma ,\sigma_{v}\right)f_{u}\left(u;\sigma_{u}\right)\;du. \end{array}$$

The sample log-likelihood is

(11) $$\ell \left(\theta \right)=\sum_{i=1}^{n}\log L_{i}\left(\theta \right).$$

The main distinction from Quantile-SFA is the bilateral density $f_{v}$. The inefficiency distribution and the interpretation of technical efficiency remain unchanged, so the comparison isolates the contribution of the HQER-induced error layer rather than confounding it with a different inefficiency model.

**Figure 1:**
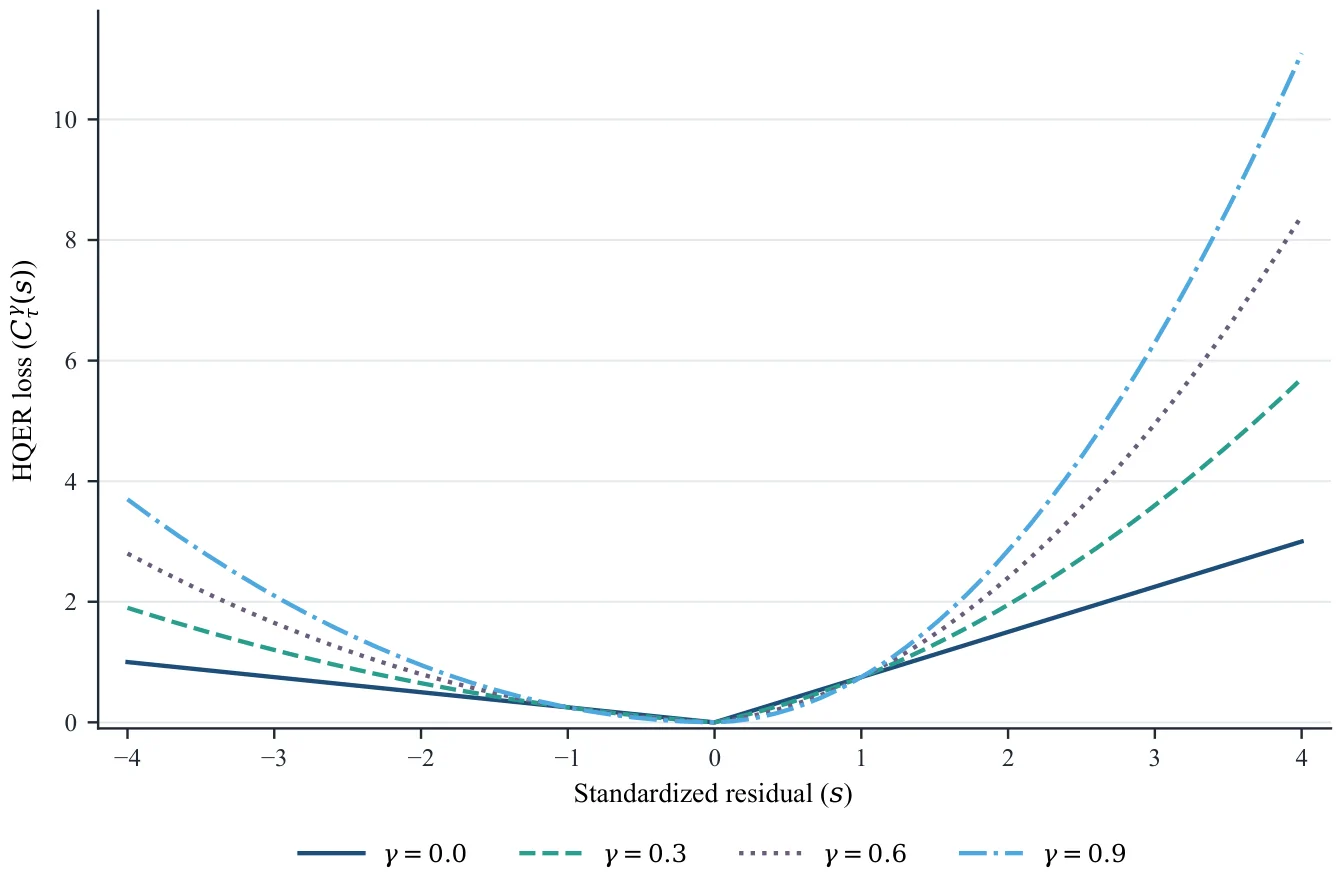
HQER loss curves for the bilateral error layer. Larger γ values increase the expectile-like quadratic component and therefore strengthen tail sensitivity while preserving the asymmetric quantile weight.

Figure 1 illustrates how the mixing parameter changes the shape and tail sensitivity of the HQER loss.

**Figure 2:**
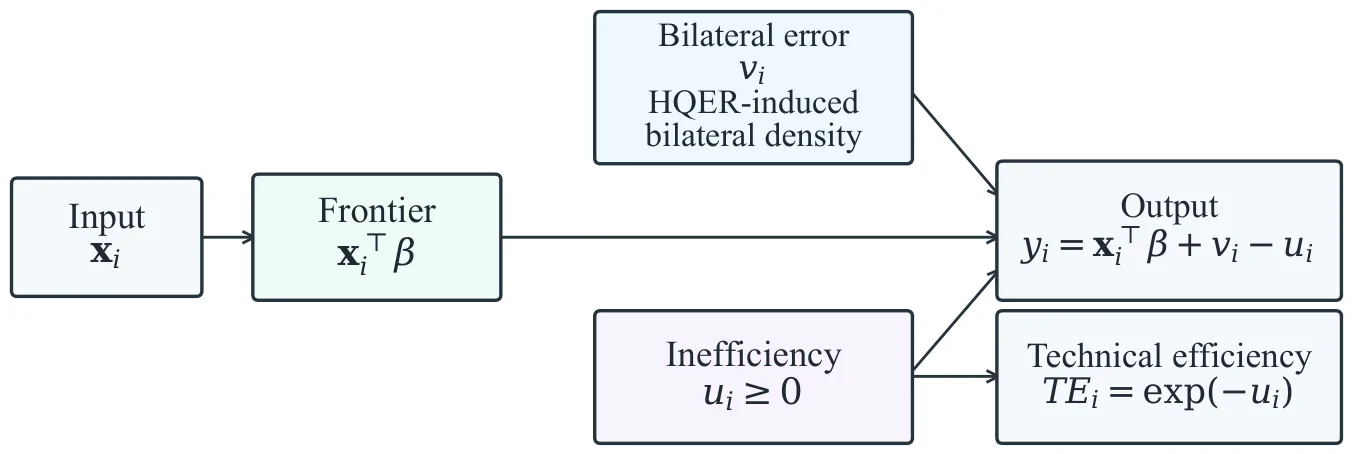
Structure of the HQER-SFA composed-error likelihood. The frontier and one-sided inefficiency structure are preserved, while the bilateral error density is replaced by the normalized HQER-induced layer.

Figure 2 summarizes how the normalized bilateral error layer enters the otherwise standard composed-error likelihood.

The marginal likelihood is evaluated by quadrature over the nonnegative inefficiency component. Positive scale parameters are estimated through transformations, and bounded parameters are constrained to feasible domains. The normalizing constant Z(τ,γ) is evaluated numerically for the relevant parameter values, log-likelihood contributions are computed in a stable form, and multiple starting values are used because the likelihood can be nonconvex. Convergence diagnostics are reported in the supplementary convergence tables and should be read alongside the main estimates. After estimating $\hat{\theta }$, inefficiency is summarized by the posterior mean

(12) $${\hat{u}}_{i}=E\left(u_{i}|\epsilon_{i};\hat{\theta }\right),$$

and technical efficiency is estimated by

(13) $${\hat{TE}}_{i}=\exp\left(-{\hat{u}}_{i}\right).$$

Monte Carlo win rates compare HQER-SFA with a baseline estimator through

(14) $$\begin{array}{cc} {\mathrm{WinRate}}_{m} & =\frac{1}{R}\sum_{r=1}^{R}I\left(M_{\mathrm{HQER},r}\;\mathrm{is}\;\mathrm{better}\;\mathrm{than}\;M_{\mathrm{baseline},r}\right). \end{array}$$

For error metrics, lower values are better; for rank correlation, higher values are better.

Positive paired differences in the statistical checks are defined to favor HQER-SFA. The source-assisted extension evaluates whether external estimates can stabilize a small target sample. Let $\theta_{S}$ denote the source-domain estimate and $\theta_{T}$ denote the target-domain parameter. A source-centered penalized target objective is

(15) $$NLL_{T}\left(\theta \right)+\lambda {∥\theta -\theta_{S}∥}^{2},$$

where $NLL_{T}\left(\theta \right)$ is the target-domain negative log-likelihood, $\theta_{S}$ is the source-domain estimate, and λ controls the penalty strength. This extension evaluates whether external source information can improve small-sample stability when it is introduced through initialization or controlled regularization.

The empirical analysis uses three processed production modules. The small-sample benchmark module, front41Data, is the FRONTIER 4.1 dataset distributed with the R frontier package [44,45] and contains 60 firm-level observations with output, capital, and labor inputs. Its analysis variables are constructed by taking natural logarithms of the corresponding raw production columns. The two large panel modules, WDI_industry and WDI_agriculture, are reconstructed from archived project-level raw JSON and merged source files from the World Bank’s World Development Indicators [46]. The archived reconstruction exactly reproduces the analysis-ready files, including the country/economy-year keys and transformed variables, with full hash checks reported in the Supplementary Materials.

The WDI_industry module uses industrial value added as output, gross fixed capital formation as the main capital proxy, and a labor-count proxy constructed from total labor force and the industry employment share. Where the main capital proxy is unavailable in the archived source, the reconstruction follows the documented fallback rule based on gross capital formation. The final industrial module contains 5546 country/economy-year observations from 203 country/economy codes over 1991–2024. The WDI_agriculture module uses agricultural value added as output, agricultural land as the land input, and a labor-count proxy constructed from total labor force and the agricultural employment share. The final agricultural module contains 6565 country/economy-year observations from 223 country/economy codes over 1991–2023.

For all modules, the dependent variable is retained on the logarithmic scale, while input variables are standardized at the experiment-loading stage after observations with missing or nonpositive core model variables are excluded. Full data-construction details, indicator mappings, archived-source reconstruction scripts, and hash checks are reported in the Supplementary Materials.

The analysis compares three frontier specifications. Traditional-SFA is the classical Gaussian-noise benchmark. Quantile-SFA is the nested quantile-oriented benchmark. HQER-SFA modifies the bilateral error layer through the hybrid quantile-expectile density while preserving the same one-sided inefficiency structure. This design makes it possible to separate prediction-oriented evidence from technical-efficiency-oriented evidence. Table 1 summarizes the datasets, roles, sample sizes, and variables used in the empirical design.

The empirical evaluation reports validation RMSE, log-likelihood, AIC, BIC, mean technical efficiency, median technical efficiency, low-efficiency ratios, and convergence status. The Monte Carlo evaluation reports β error, prediction RMSE, technical-efficiency RMSE, and technical-efficiency rank correlation. These metrics are interpreted separately because they measure different aspects of model behavior.

Table 2 summarizes the evidence design. It links data sources, competing model specifications, empirical and simulation evidence blocks, evaluation metrics, and the final metric-specific interpretation.

## 3. Simulation Study

The simulation study evaluates the same estimators under controlled parameter-inversion designs rather than rerunning or altering the empirical modules. The main Monte Carlo design uses pseudo-true parameters extracted from the locked real-data estimates and then refits the competing estimators on simulated samples. The design crosses dataset-specific pseudo-parameter sources, Quantile-SFA and HQER-SFA pseudo-model sources, normal, Student-*t*, skewed, asymmetric-Laplace, and HQER-induced error scenarios, and sample-size settings. The formal sample sizes are n=60 and n=500 for front41Data, and n=500 and n=2000 for both WDI-derived modules. The R=100 design provides the baseline simulation pattern, and the R=300 design checks whether that pattern remains stable under expanded repetitions. The comparison is metric-specific: lower β error, prediction RMSE, and technical-efficiency RMSE are better, whereas higher technical-efficiency rank correlation is better. Scenario-level design cells and repetition-level summaries are reported in Supplementary Tables S4 and S5.

**Figure 3:**
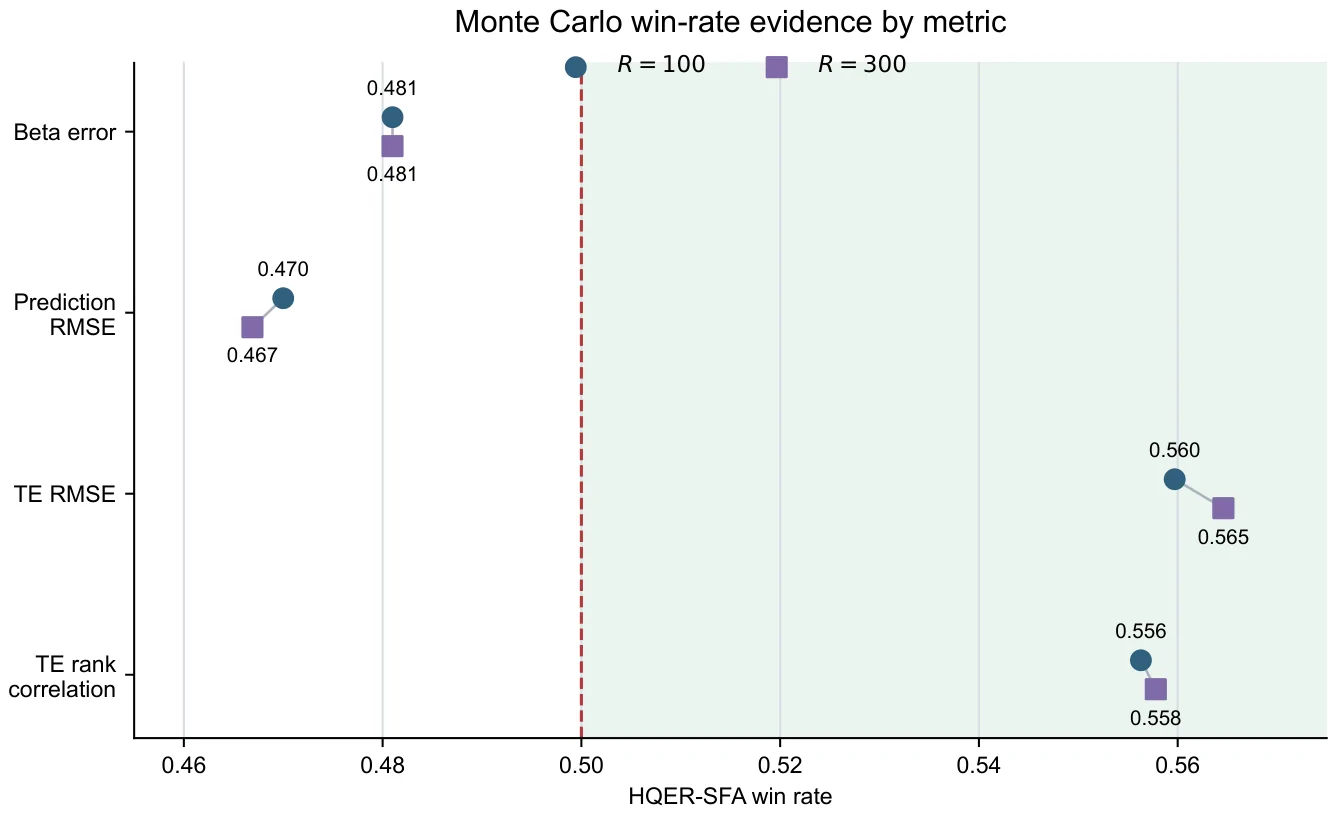
Monte Carlo win-rate comparison across evaluation metrics. The red dashed line marks the 0.5 equal-win reference, and the green-shaded region denotes win rates above 0.5, which favor HQER-SFA. The expanded R=300 design confirms that the clearest gains occur for technical-efficiency RMSE and technical-efficiency rank correlation.

In the R=100 design, the win rates are 0.4810 for β error, 0.4700 for prediction RMSE, 0.5597 for technical-efficiency RMSE, and 0.5563 for technical-efficiency rank correlation. The expanded R=300 experiment preserves and slightly strengthens the same technical-efficiency pattern: the win rates rise to 0.5646 for technical-efficiency RMSE and 0.5578 for technical-efficiency rank correlation. These results indicate that the main benefit of HQER-SFA appears in the residual-decomposition and technical-efficiency-recovery channel, while prediction and coefficient recovery provide complementary benchmark evidence. Figure 3 and Table 3 compare the two repetition designs by metric.

**Figure 4:**
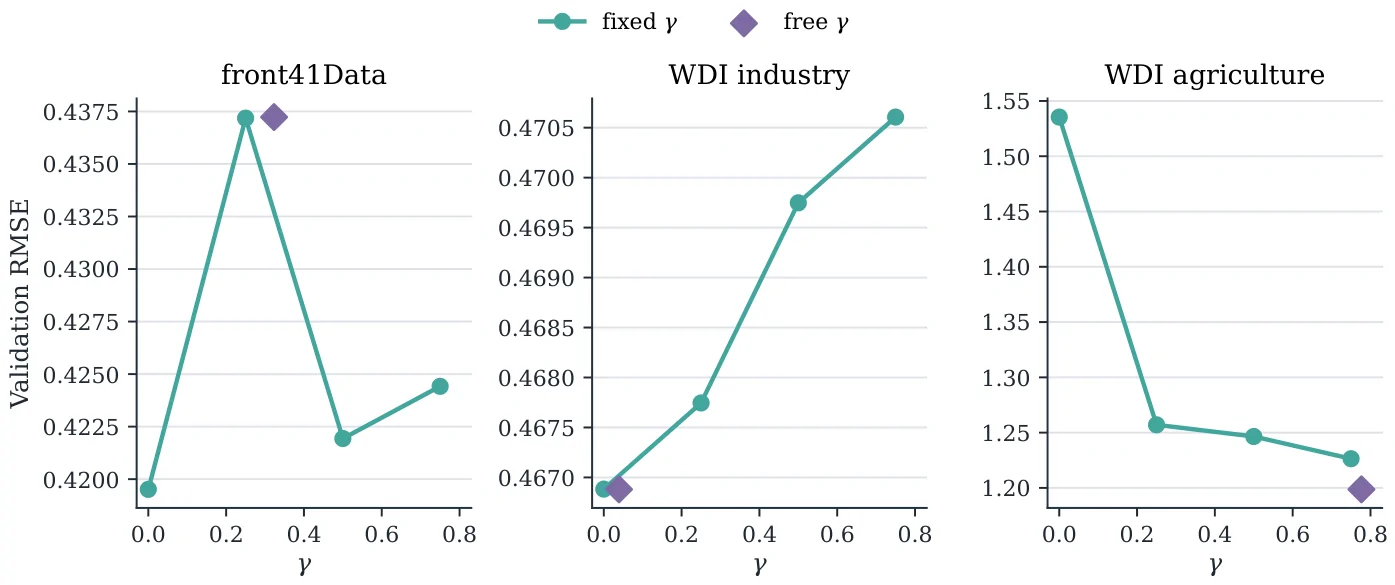
γ sensitivity in real-data validation RMSE. The free-γ estimate is shown separately from fixed settings to make the data-dependent error-layer adaptation visible.

The γ-sensitivity results clarify that γ is not merely an additional tuning parameter. It changes the relative influence of quantile-like absolute loss and expectile-like quadratic loss in the bilateral error layer, thereby controlling how strongly tail-sensitive residuals affect the likelihood. The agricultural module benefits most from a positive and freely estimated γ, while the industrial modules show smaller changes, indicating that the hybrid layer is especially useful when residual allocation is more asymmetric or heterogeneous. Table 4 summarizes the mechanism-level evidence, and Figure 4 visualizes the real-data sensitivity results.

Paired statistical checks compare fitted models under matched design cells and repetitions. Positive paired differences are defined to favor HQER-SFA. The checks show positive average differences for both the R=100 and R=300 designs, including for the technical-efficiency metrics, confirming that the observed gains are not driven by a single aggregate summary. Medians and win rates still reveal heterogeneity across design cells, which is expected because the hybrid error layer is most valuable when residual structure departs from the simpler benchmark densities. Table 5 reports the matched paired-difference tests.

**Figure 5:**
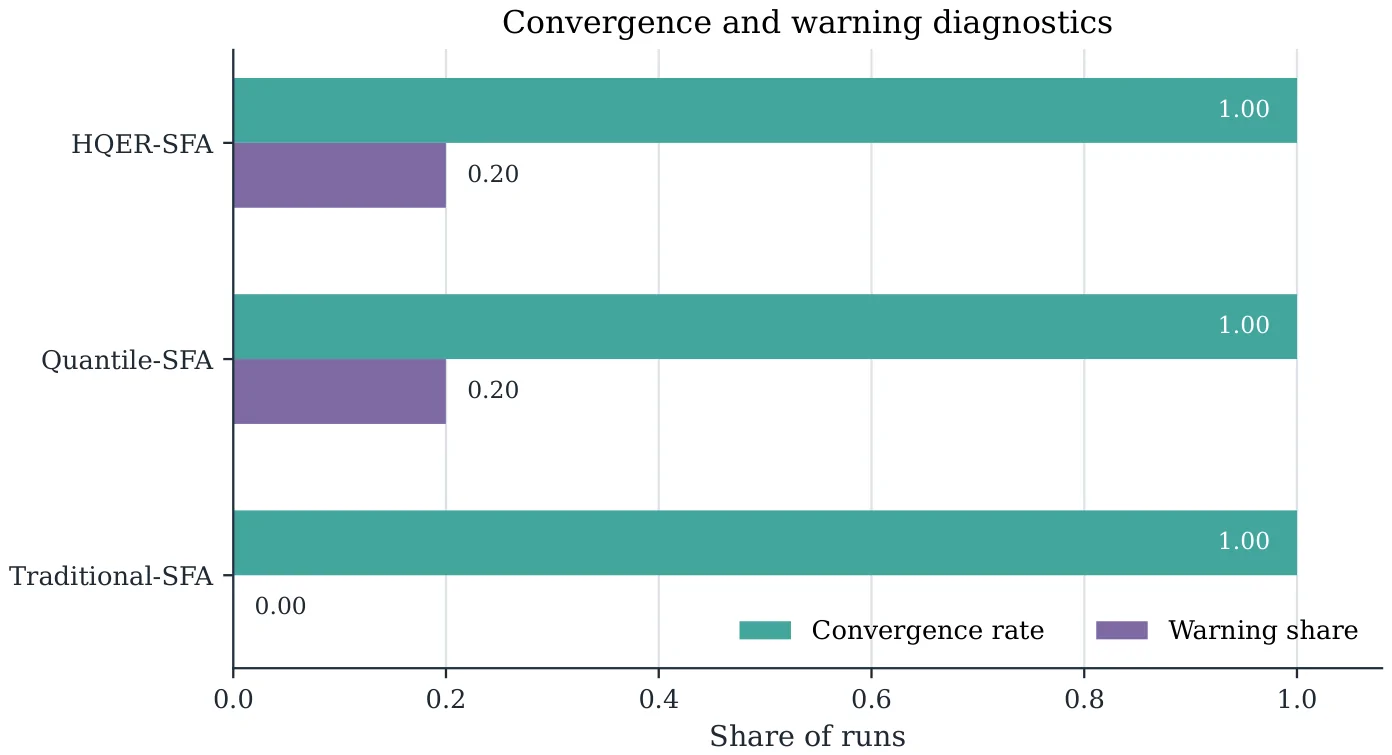
Convergence and warning diagnostics. Convergence rates equal one in the compact summary, while warning shares identify runs requiring numerical caution rather than indicating estimation failures.

Convergence diagnostics were collected across the real-data experiments, the traditional SFA benchmark, the Monte Carlo robustness experiment, the γ-sensitivity analysis, and the source-assisted extension. Missing legacy diagnostic fields are recorded as NA rather than imputed. The diagnostics document numerical feasibility: all reported model classes converge in the compact summary, and warning shares are transparently separated from convergence failures. Table 6 and Figure 5 summarize the convergence and warning results.

Warnings are documented in the Supplementary Diagnostics and do not imply failed convergence unless explicitly flagged.

## 4. Empirical Application

**Figure 6:**
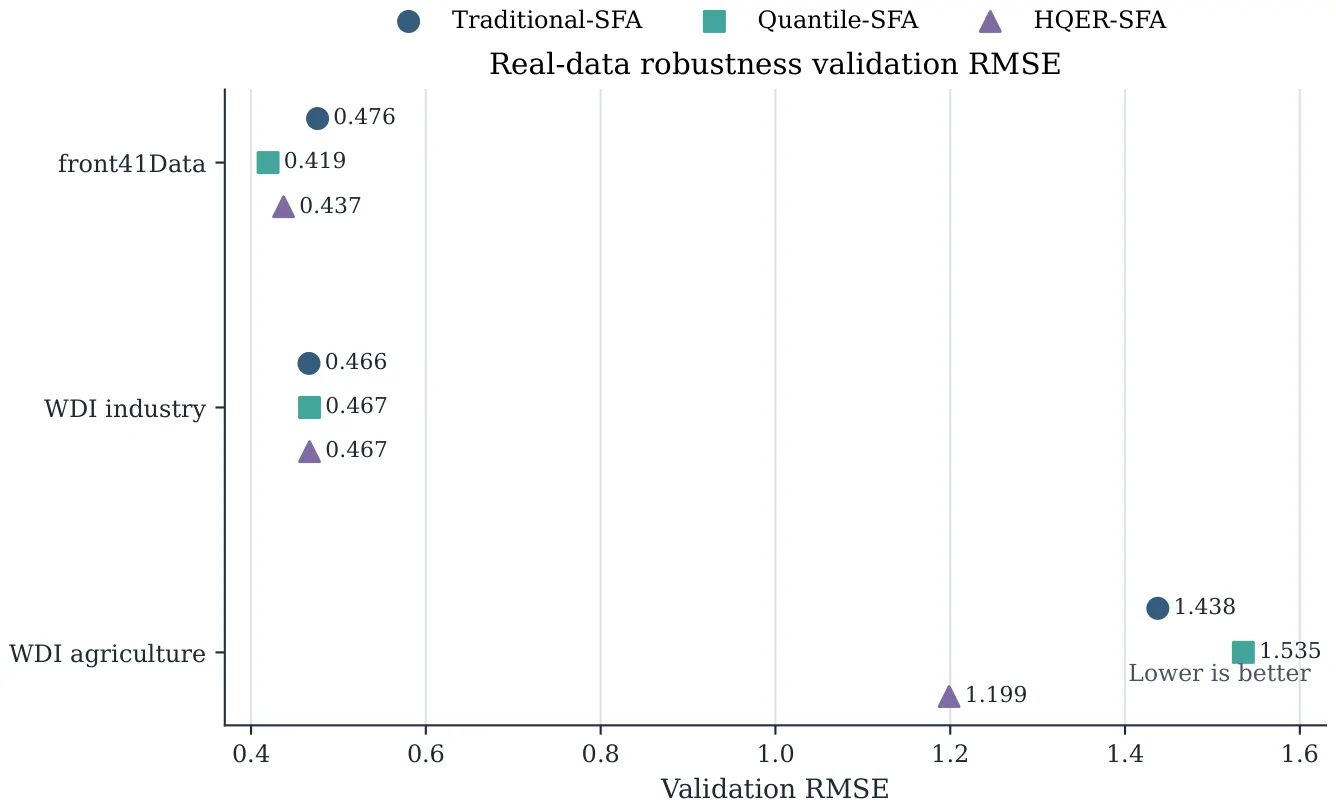
Real-data robustness-validation RMSE comparison across Traditional-SFA, Quantile-SFA, and HQER-SFA. Lower values indicate better prediction under the robustness-validation setting.

The empirical application compares the processed production modules through the same metric-specific lens used in the simulations. Table 7 reports the original real-data comparison between Quantile-SFA and HQER-SFA, and Figure 6 visualizes the three-model robustness-validation RMSE comparison. HQER-SFA attains lower validation RMSE in all three modules in the formal two-model comparison, with the largest gain in the agricultural module. This pattern is consistent with the methodological premise that a more flexible bilateral error density is most useful when residual variation is more asymmetric, tail-sensitive, or heterogeneous.

Table 8 adds the classical Traditional-SFA benchmark. The robustness comparison shows that the industrial modules are close across competing specifications, whereas HQER-SFA gives the lowest validation RMSE in the agricultural case. The result strengthens the interpretation that HQER-SFA is most valuable when the bilateral error layer has a larger role in allocating residual variation.

The validation RMSE entries in Table 7 and Table 8 come from complementary real-data protocols rather than identical re-estimates. Table 7 reports the original two-model comparison between Quantile-SFA and HQER-SFA under the formal real-data estimation protocol, whereas Table 8 reports a separate robustness benchmark that adds Traditional-SFA under a common robustness-validation setting. The two tables are therefore used as complementary evidence on model-ranking stability across protocols.

The empirical technical-efficiency summaries show that the agricultural dataset is most responsive to the error-layer specification. HQER-SFA produces higher mean technical efficiency and a lower low-efficiency ratio than Quantile-SFA in that dataset, indicating that the hybrid bilateral density substantially changes posterior inefficiency allocation. A plausible explanation is that agricultural production data contain stronger tail behavior, measurement variation, or domain heterogeneity than the industrial modules.

Land quality, climate-related shocks, regional heterogeneity, measurement error, and tail disturbances can make the bilateral error specification more consequential for residual allocation and posterior inefficiency.

**Figure 7:**
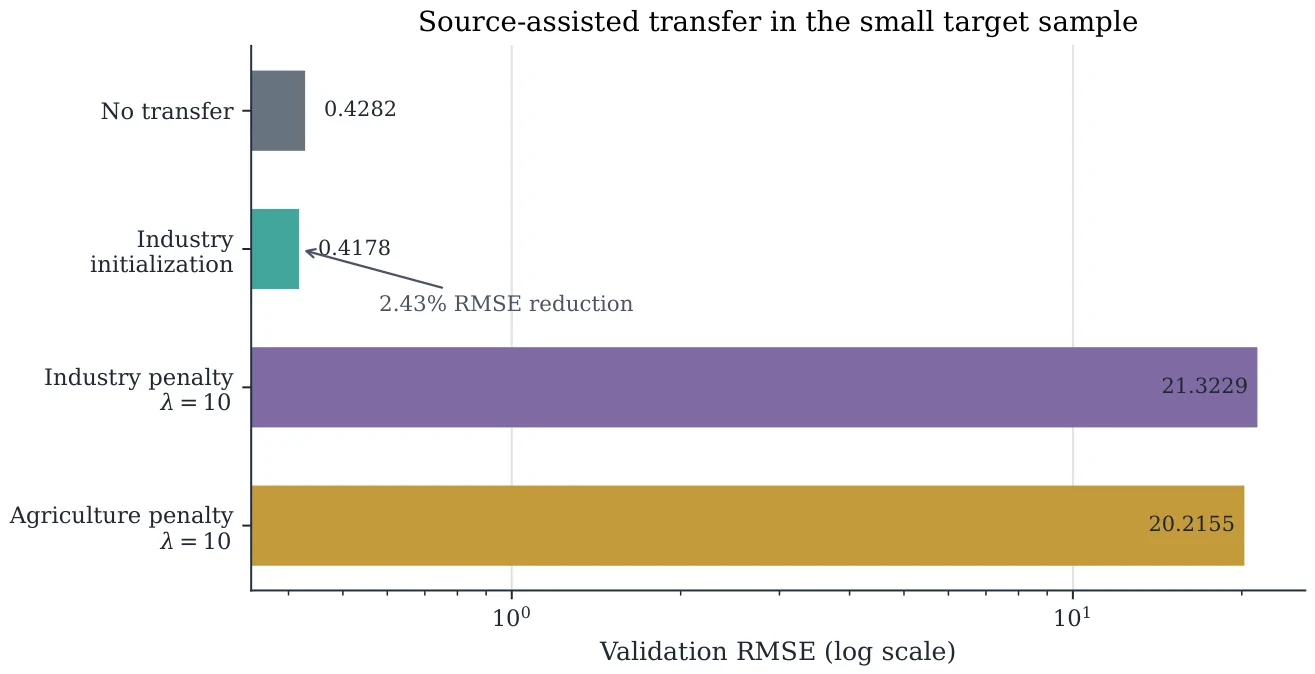
Source-assisted validation RMSE under initialization and penalty transfer. Same-domain initialization improves the small-sample target RMSE, whereas large source-centered penalties illustrate the need to control transfer strength. Note. The *y*-axis is on a logarithmic scale because strong penalty settings produce much larger validation errors than the no-transfer and initialization settings.

The source-assisted extension examines whether external source information can stabilize estimation in the small industrial target sample. Same-domain industrial source initialization reduces validation RMSE from 0.4282 to 0.4178, an improvement of about 2.43%. This result shows that related source information can be useful when it is used for initialization. Strong source-centered penalties, by contrast, over-constrain the target likelihood; the large λ=10 errors are therefore treated as diagnostics for excessive transfer strength rather than as a failure of source-assisted initialization. Figure 7 visualizes these effects, and Table 9 reports the corresponding numerical summary.

## 5. Discussion and Conclusions

Across the empirical and simulation evidence, HQER-SFA delivers its clearest gains for technical-efficiency recovery, especially for TE RMSE and TE rank correlation. The R=300 robustness design reinforces the R=100 pattern, and the agricultural module shows the strongest real-data response to the flexible bilateral error layer. Prediction-oriented metrics remain useful benchmarks, but they are not the primary target of the proposed specification. This pattern is consistent with the recent frontier literature’s emphasis on distributional specification, robustness, mixture behavior, and numerical likelihood care [12,13,14,18].

The strongest evidence appears in technical-efficiency recovery because that target depends directly on decomposing residual variation into bilateral noise and one-sided inefficiency. Prediction RMSE mainly measures the fitted frontier, and beta recovery mainly measures recovery of the frontier parameters. HQER-SFA modifies the bilateral error layer rather than the economic meaning of the frontier itself. This is a strength of the design: it targets the channel through which posterior inefficiency is recovered while leaving the frontier decomposition interpretable and comparable with established SFA benchmarks.

The mixing parameter γ controls the relative weight of quantile-like absolute loss and expectile-like quadratic loss in the bilateral error layer. At γ=0, HQER-SFA reduces to the Quantile-SFA boundary; positive values change tail behavior and residual allocation. The sensitivity and ablation evidence shows that γ is mechanism-relevant and identifies the settings in which additional tail-sensitive flexibility matters most. This interpretation also distinguishes HQER-SFA from broader quantile frontier and semiparametric approaches [10,11,15,16]: the present paper evaluates a likelihood-based bilateral error layer that can be inserted into the composed-error framework without redefining the frontier technology.

The benchmark comparisons are informative because they show where each specification is strongest. Traditional-SFA performs well in the large industrial dataset, while Quantile-SFA performs best on the small industrial robustness benchmark; HQER-SFA is strongest in technical-efficiency recovery and in the agricultural module, where the error-layer specification is more consequential. The contribution is therefore the construction and evaluation of an HQER-induced bilateral error density within a stochastic-frontier likelihood, with empirical evidence showing when this additional flexibility improves residual decomposition.

The model also opens several natural extensions. The HQER-induced density requires numerical normalization and likelihood integration, but the convergence diagnostics show that the reported implementation is feasible for the empirical and simulation designs considered here. Future work can develop identification and asymptotic theory, examine alternative inefficiency distributions and domains, and study the joint behavior of τ, γ, $\sigma_{v}$, and $\sigma_{u}$ in larger frontier systems.

This paper introduces HQER-SFA as a likelihood-based extension of Quantile-SFA for stochastic frontier analysis. It embeds a normalized hybrid quantile-expectile loss into the bilateral error layer while preserving one-sided inefficiency, nests Quantile-SFA when γ=0, and allows the error layer to improve technical-efficiency recovery through more flexible residual allocation. The expanded evidence identifies technical-efficiency recovery as the central advantage: R=300 reinforces the R=100 pattern for TE RMSE and TE rank correlation, the agricultural application shows the strongest real-data improvement, γ sensitivity confirms the role of tail-sensitive adaptation, and same-domain source initialization improves small-sample RMSE. HQER-SFA is therefore a practical and interpretable extension for likelihood-based technical-efficiency recovery in settings where the bilateral error structure matters.

## Figures and Tables

**Table 1:** Processed production modules and model variables used in the empirical design.

Dataset	Role	*N*	Output	Inputs
front41Data	Small industrial target dataset	60	y	x_capital; x_labour
WDI_industry	Large industrial dataset and same-domain source	5546	y	x_capital_proxy; x_industry_labor
WDI_agriculture	Agricultural dataset and cross-domain source	6565	y	x_ag_land; x_ag_labor

**Table 2:** Evidence design linking data sources, model comparisons, evaluation metrics, and interpretation.

Evidence Block	Data/Design	Compared Models	Main Metrics	Role
Real-data comparison	Processed production modules	Traditional-SFA, Quantile-SFA, HQER-SFA	Validation RMSE, AIC/BIC, TE summaries	Empirical behavior
R=100 Monte Carlo	Parameter-inversion design	HQER-SFA vs. baselines	Beta error, prediction RMSE, TE RMSE, TE rank corr.	Baseline simulation pattern
R=300 robustness	Expanded repetitions	HQER-SFA vs. baselines	Win rates by metric	Stability check
γ sensitivity	Fixed/free γ settings	HQER-SFA variants; quantile boundary	Validation RMSE, TE summaries	Error-layer mechanism
Source-assisted extension	Small target with sources	No transfer, initialization, penalty	RMSE, TE rank stability	Small-sample stability
Convergence diagnostics	All estimation blocks	Traditional, Quantile, HQER	Convergence rate, warnings	Numerical feasibility

**Table 3:** Monte Carlo win-rate robustness from R=100 to R=300.

Metric	R=100 HQER-SFA Win Rate	R=300 HQER-SFA Win Rate	Interpretation
Beta error	0.4810	0.4810	Coefficient recovery remains benchmark-comparable.
Prediction RMSE	0.4700	0.4669	Prediction accuracy remains a complementary diagnostic.
TE RMSE	0.5597	0.5646	Technical-efficiency recovery advantage is reinforced.
TE rank correlation	0.5563	0.5578	Technical-efficiency ranking advantage is reinforced.

**Table 4:** γ sensitivity and ablation summary for the HQER-induced bilateral error layer.

Evidence Block	Main Finding	Implication
Real-data γ sensitivity	γ changes validation RMSE and TE summaries across datasets.	The bilateral error layer directly affects residual decomposition.
Monte Carlo γ ablation	Free γ provides additional flexibility across criteria.	The mechanism improves targeted error-layer adaptation.
Boundary case γ=0	γ=0 corresponds to the Quantile-SFA boundary.	This confirms the nesting relationship with Quantile-SFA.
Fixed γ settings	Fixed values reveal how performance changes as the expectile component increases.	The sensitivity profile guides stable specification choices.

**Table 5:** Matched Monte Carlo paired-difference tests favoring HQER-SFA.

Design	Metric	No. of Pairs	Mean Diff.	95% CI	Wilcoxon *p*-Value
R=100	β error	6000	0.0108	[0.0066, 0.0151]	0.00809
R=100	*y* RMSE	6000	0.0092	[0.0068, 0.0116]	7.86 × $10^{-8}$
R=100	TE RMSE	6000	0.0232	[0.0205, 0.0260]	7.53 × $10^{-36}$
R=100	TE rank corr.	6000	0.0018	[0.0012, 0.0025]	2.36 × $10^{-16}$
R=300	β error	18,000	0.0104	[0.0081, 0.0126]	1.61 × $10^{-7}$
R=300	*y* RMSE	18,000	0.0085	[0.0072, 0.0100]	3.10 × $10^{-21}$
R=300	TE RMSE	18,000	0.0231	[0.0216, 0.0245]	1.02 × $10^{-126}$
R=300	TE rank corr.	18,000	0.0022	[0.0018, 0.0026]	1.06 × $10^{-50}$

Note. Positive differences favor HQER-SFA; all listed mean differences are positive.

**Table 6:** Compact convergence and warning diagnostics across estimation blocks.

Model	Runs	Converged	Conv. Rate	Mean Iterations	Warnings	Warning Share
HQER-SFA	24,018	24,018	1.0000	30.8126	4800	0.1999
Quantile-SFA	24,018	24,018	1.0000	26.8643	4800	0.1999
Traditional-SFA	3	3	1.0000	22.6667	0	0.0000

**Table 7:** Formal real-data comparison between Quantile-SFA and HQER-SFA.

Dataset	Model	Val. RMSE	AIC	BIC	Mean TE	Low-Eff. Ratio	γ
front41Data	Quantile-SFA	0.4360	23.1931	35.7592	0.9505	0.0000	0.0000
front41Data	HQER-SFA	0.4354	21.7624	36.4229	0.9505	0.0000	0.3248
WDI_industry	Quantile-SFA	0.4730	3402.3535	3442.0785	0.9505	0.0000	0.0000
WDI_industry	HQER-SFA	0.4711	3447.4393	3493.7851	0.9505	0.0000	0.0386
WDI_agriculture	Quantile-SFA	1.4487	19,045.0900	19,085.8270	0.4141	0.7514	0.0000
WDI_agriculture	HQER-SFA	1.2897	18,778.3079	18,825.8345	0.8492	0.0000	0.9212

**Table 8:** Real-data robustness comparison across Traditional-SFA, Quantile-SFA, and HQER-SFA.

Dataset	Best RMSE Model	Trad. RMSE	Quantile RMSE	HQER RMSE	Interpretation
front41Data	Quantile-SFA	0.4761	0.4195	0.4372	Quantile-SFA is best, with HQER-SFA remaining close.
WDI_industry	Traditional-SFA	0.4663	0.4669	0.4669	Traditional-SFA has a marginal edge; flexible models are nearly tied.
WDI_agriculture	HQER-SFA	1.4375	1.5354	1.1987	HQER-SFA gives the strongest RMSE performance.

**Table 9:** Source-assisted estimation summary for the small target sample.

Method	Source	λ	Val. RMSE	TE Rank Stability	Main Interpretation
No Transfer	none	0	0.4282	0.9764	Target benchmark.
Source Initialization	WDI_industry	0	0.4178	0.9739	Best RMSE; −2.43%.
Penalty Transfer	WDI_industry	10	21.3229	0.2787	Penalty too strong.
Penalty Transfer	WDI_agriculture	10	20.2155	0.2780	Cross-domain penalty too strong.

Note. Full log-likelihood and beta-stability results are reported in Supplementary Table S9.

## Data Availability

Empirical tables, simulation summaries, statistical checks, and figure outputs are provided in the Supplementary Materials; the use of public or third-party data follows the licensing conditions of the original sources.

## Supplementary Materials

The following supporting information can be downloaded at https://www.mdpi.com/article/10.3390/e28080881/s1. Supplementary Tables S1–S13 provide the complete supporting evidence. Table S1 reports descriptive statistics; Tables S2 and S3 report full real-data and Traditional-SFA comparisons; Tables S4 and S5 report R=100 and R=300 Monte Carlo summaries; Tables S6–S8 report γ sensitivity and ablation checks; Tables S9 and S10 report source-assisted results; and Tables S11–S13 report convergence diagnostics, warning cases, and paired statistical checks.

## References

[1] Aigner D., Lovell C.A.K., Schmidt P. (1977). Formulation and Estimation of Stochastic Frontier Production Function Models. J. Econom..

[2] Meeusen W., van den Broeck J. (1977). Efficiency Estimation from Cobb-Douglas Production Functions with Composed Error. Int. Econ. Rev..

[3] Jondrow J., Lovell C.A.K., Materov I.S., Schmidt P. (1982). On the Estimation of Technical Inefficiency in the Stochastic Frontier Production Function Model. J. Econom..

[4] Kumbhakar S.C., Lovell C.A.K. (2000). Stochastic Frontier Analysis.

[5] Battese G.E., Coelli T.J. (1992). Frontier Production Functions, Technical Efficiency and Panel Data: With Application to Paddy Farmers in India. J. Product. Anal..

[6] Battese G.E., Coelli T.J. (1995). A Model for Technical Inefficiency Effects in a Stochastic Frontier Production Function for Panel Data. Empir. Econ..

[7] Greene W. (2005). Reconsidering Heterogeneity in Panel Data Estimators of the Stochastic Frontier Model. J. Econom..

[8] Jradi S., Ruggiero J. (2019). Stochastic Data Envelopment Analysis: A Quantile Regression Approach to Estimate the Production Frontier. Eur. J. Oper. Res..

[9] Tsionas M.G. (2020). Quantile Stochastic Frontiers. Eur. J. Oper. Res..

[10] Jradi S., Parmeter C.F., Ruggiero J. (2021). Quantile Estimation of Stochastic Frontiers with the Normal-Exponential Specification. Eur. J. Oper. Res..

[11] Fusco E., Benedetti R., Vidoli F. (2022). Stochastic Frontier Estimation through Parametric Modelling of Quantile Regression Coefficients. Empir. Econ..

[12] Bělín M., Hanousek J. (2021). SFA Robustness to Violated Distributional Assumptions: Theory, Simulations and Empirical Evidence. Appl. Econ..

[13] Stead A.D., Wheat P., Greene W.H. (2023). Robust Maximum Likelihood Estimation of Stochastic Frontier Models. Eur. J. Oper. Res..

[14] Stead A.D., Wheat P., Greene W.H. (2023). On Hypothesis Testing in Latent Class and Finite Mixture Stochastic Frontier Models, with Application to a Contaminated Normal-Half Normal Model. J. Product. Anal..

[15] Forchini G., Theler R. (2022). Semi-Parametric Modelling of Inefficiencies in Stochastic Frontier Analysis. J. Product. Anal..

[16] Zeebari Z., Månsson K., Sjölander P., Söderberg M. (2022). Regularized Conditional Estimators of Unit Inefficiency in Stochastic Frontier Analysis, with Application to Electricity Distribution Market. J. Product. Anal..

[17] Centorrino S., Pérez-Urdiales M. (2023). Maximum Likelihood Estimation of Stochastic Frontier Models with Endogeneity. J. Econom..

[18] Schmidt R., Kneib T. (2023). Multivariate Distributional Stochastic Frontier Models. Comput. Stat. Data Anal..

[19] Haschka R.E. (2024). Endogeneity in Stochastic Frontier Models with ‘Wrong’ Skewness: Copula Approach without External Instruments. Stat. Methods Appl..

[20] Wei Z., Zhu X., Wang T. (2021). The Extended Skew-Normal-Based Stochastic Frontier Model with a Solution to ‘Wrong Skewness’ Problem. Statistics.

[21] Kumbhakar S.C., Lai H.P. (2021). A Multi-Output Multi-Input Stochastic Frontier System with Input- and Output-Specific Inefficiency. Econ. Lett..

[22] Cazals C., Florens J.P., Simar L. (2002). Nonparametric Frontier Estimation: A Robust Approach. J. Econom..

[23] Aragon Y., Daouia A., Thomas-Agnan C. (2005). Nonparametric Frontier Estimation: A Conditional Quantile-Based Approach. Econom. Theory.

[24] Daouia A., Simar L. (2007). Nonparametric Efficiency Analysis: A Multivariate Conditional Quantile Approach. J. Econom..

[25] Daouia A., Florens J.P., Simar L. (2020). Robustified Expected Maximum Production Frontiers. Econom. Theory.

[26] Tsionas M.G. (2021). Estimating Monotone Concave Stochastic Production Frontiers. J. Bus. Econ. Stat..

[27] Koenker R., Bassett G. (1978). Regression Quantiles. Econometrica.

[28] Koenker R. (2005). Quantile Regression.

[29] Yu K., Moyeed R.A. (2001). Bayesian Quantile Regression. Stat. Probab. Lett..

[30] Kozumi H., Kobayashi G. (2011). Gibbs Sampling Methods for Bayesian Quantile Regression. J. Stat. Comput. Simul..

[31] Newey W.K., Powell J.L. (1987). Asymmetric Least Squares Estimation and Testing. Econometrica.

[32] Efron B. (1991). Regression Percentiles Using Asymmetric Squared Error Loss. Stat. Sin..

[33] Taylor J.W. (2008). Estimating Value at Risk and Expected Shortfall Using Expectiles. J. Financ. Econom..

[34] Schnabel S.K., Eilers P.H.C. (2009). Optimal Expectile Smoothing. Comput. Stat. Data Anal..

[35] Schulze Waltrup L., Sobotka F., Kneib T., Kauermann G. (2015). Expectile and Quantile Regression: David and Goliath?. Stat. Model..

[36] Atanane A., Mkhadri A., Oualkacha K. (2025). An Efficient Hybrid Approach of Quantile and Expectile Regression. Stat. Pap..

[37] Pan S.J., Yang Q. (2010). A Survey on Transfer Learning. IEEE Trans. Knowl. Data Eng..

[38] Ben-David S., Blitzer J., Crammer K., Kulesza A., Pereira F., Vaughan J.W. (2010). A Theory of Learning from Different Domains. Mach. Learn..

[39] Mansour Y., Mohri M., Rostamizadeh A. (2008). Domain Adaptation with Multiple Sources. Advances in Neural Information Processing Systems 21 (NIPS 2008).

[40] Weiss K., Khoshgoftaar T.M., Wang D. (2016). A Survey of Transfer Learning. J. Big Data.

[41] Kouw W.M., Loog M. (2019). A Review of Domain Adaptation without Target Labels. IEEE Trans. Pattern Anal. Mach. Intell..

[42] Li S., Cai T.T., Li H. (2022). Transfer Learning for High-Dimensional Linear Regression: Prediction, Estimation and Minimax Optimality. J. R. Stat. Soc. Ser. B.

[43] Tian Y., Feng Y. (2023). Transfer Learning under High-Dimensional Generalized Linear Models. J. Am. Stat. Assoc..

[44] Coelli T.J. (1996). A Guide to FRONTIER Version 4.1: A Computer Program for Stochastic Frontier Production and Cost Function Estimation.

[45] Coelli T., Henningsen A. (2020). frontier: Stochastic Frontier Analysis.

[46] World Bank (2026). World Development Indicators.

