# Supplementary material for: Hybrid Quantile-Expectile Error Layers for Technical-Efficiency Recovery in Stochastic Frontier Analysis
Source: Entropy (Basel). 2026 Aug 5;28(8):881. doi: 10.3390/e28080881 (PMC13512548; doi:10.3390/e28080881)
Supplement: Supplementary file 1 [file entropy-28-00881-s001.zip › entropy-4421855-Supplementary.pdf]

Supplementary Materials for
Hybrid Quantile-Expectile Error Layers for Technical-Efficiency Recovery in Stochastic Frontier Analysis

Shengming Wang, Yunquan Song and Juan Yu

Supplementary Table Sources

This document displays Supplementary Tables S1–S13 completely. Wide tables are split into sequential panels with key identifier columns repeated, so that no table columns are clipped. The original CSV sources are included in the `tables/` directory.

S1 Full descriptive statistics

Source CSV: `tables/s1_full_descriptive_statistics.csv`. Rows displayed: 9; columns displayed: 13. The table is split into panels to keep all cells inside the page boundary.

Panel 1 of 2

Columns shown: `dataset`, `variable`, `dataset_role`, `variable_role`, `count`, `mean`, `std`, `min`, `q25`.

dataset	variable	dataset_role	variable_role	count	mean	std	min	q25
front41Data	y	small_sample_industry_target	y	60	2.658497064128959	0.613984563054606	0.5068176023684519	2.469562620843125
front41Data	x_capital	small_sample_industry_target	X	60	8.419191270074102e-17	1.0084389681792216	-2.9250887966011097	-0.3695260697698803
front41Data	x_labour	small_sample_industry_target	X	60	3.7886360715333473e-16	1.0084389681792214	-4.4554433851672	-0.2893157262944207
WDI_industry	y	large_sample_industry_source	y	5546	24.112444718601047	2.7244333693334872	17.484139567801236	22.075097301198277
WDI_industry	x_capital_proxy	large_sample_industry_source	X	5546	3.689799998177224e-16	1.000090167260451	-3.002283261374871	-0.7719403452208501
WDI_industry	x_industry_labor	large_sample_industry_source	X	5546	-1.409298610414912e-16	1.000090167260451	-2.1925651227014504	-0.6586684994001905
WDI_agriculture	y	agriculture_external_domain	y	6565	22.534787537629377	2.553348385300609	16.724666209965857	20.77009768556825
WDI_agriculture	x_ag_land	agriculture_external_domain	X	6565	-4.946200003691788e-16	1.000076170164257	-3.145500337787824	-0.554383128341979
WDI_agriculture	x_ag_labor	agriculture_external_domain	X	6565	-7.03507659168416e-17	1.000076170164257	-2.2222989729853424	-0.6912775536552085

Panel 2 of 2

Columns shown: `dataset`, `variable`, `median`, `q75`, `max`, `missing_rate`.

dataset	variable	median	q75	max	missing_rate
front41Data	y	2.7030228244163723	3.0561404459273813	3.614102027156113	0.0
front41Data	x_capital	0.287424366005046	0.7441430543679031	1.057294462885232	0.0
front41Data	x_labour	0.2979694636879092	0.6464388893658128	0.8875093320140558	0.0
WDI_industry	y	24.026530059513835	26.166820570869337	30.880379159441343	0.0
WDI_industry	x_capital_proxy	-0.0642507143048885	0.7434855553093271	2.5757816512731107	0.0
WDI_industry	x_industry_labor	-0.1769917345614084	0.6177133167536113	2.449326093278746	0.0
WDI_agriculture	y	22.13908277594517	24.228739755923584	28.99011689372597	0.0
WDI_agriculture	x_ag_land	0.005668982035361	0.5989633365305942	2.040391901033254	0.0
WDI_agriculture	x_ag_labor	-0.0660589832065201	0.6328384332237309	2.217702629894727	0.0

S2 Full real-data comparison

Source CSV: `tables/s2_full_realdata_quantile_hqer_comparison.csv`. Rows displayed: 6; columns displayed: 25. The table is split into panels to keep all cells inside the page boundary.

Panel 1 of 4

Columns shown: `dataset, model, n, y_col, x_cols, tau_hat, gamma_hat, sigma_v_hat, sigma_u_hat`.

dataset	model	n	y_col	x_cols	tau_hat	gamma_hat	sigma_v_hat	sigma_u_hat
front41Data	Quantile-SFA	60	y	x_capital;x_labour	0.6969051333905386	0.0	0.1078689254853769	0.049830468245088
front41Data	HQER-SFA	60	y	x_capital;x_labour	0.7849700944322544	0.3247900154687536	0.1921439886081905	0.0507529264181521
WDI_industry	Quantile-SFA	5546	y	x_capital_proxy;x_industry_labor	0.4625266099164946	0.0	0.1542332820658772	0.0507401038005439
WDI_industry	HQER-SFA	5546	y	x_capital_proxy;x_industry_labor	0.4468428067972121	0.0386105835857917	0.18664774066355	0.0507308392988696
WDI_agriculture	Quantile-SFA	6565	y	x_ag_land;x_ag_labor	0.5238159965992826	0.0	0.2610017775918883	1.2655760007539676
WDI_agriculture	HQER-SFA	6565	y	x_ag_land;x_ag_labor	0.7142327796494751	0.9211606750577682	0.9120430131952256	0.2015304445363709

Panel 2 of 4

Columns shown: `dataset, model, log_likelihood, AIC, BIC, validation_rmse, mean_TE, median_TE, low_efficiency_ratio`.

dataset	model	log_likelihood	AIC	BIC	validation_rmse	mean_TE	median_TE	low_efficiency_ratio
front41Data	Quantile-SFA	-5.59655193221136	23.19310386442272	35.75917123775532	0.4359803515079578	0.9505303694524264	0.9505301189094344	0.0
front41Data	HQER-SFA	-3.8812235897088265	21.76244717941765	36.422859114972354	0.4353856936710651	0.9505301224522849	0.9505300171739526	0.0
WDI_industry	Quantile-SFA	-1695.1767447321204	3402.3534894642407	3442.078482821295	0.4730038091323133	0.9505301346521912	0.9505305028358558	0.0
WDI_industry	HQER-SFA	-1716.7196604943404	3447.4393209886807	3493.785146571911	0.4711020803238858	0.9505301508190214	0.9505304563904312	0.0
WDI_agriculture	Quantile-SFA	-9516.54497752781	19045.08995505562	19085.82700177604	1.4487271438701574	0.4141296886391253	0.4037377577709754	0.7514089870525514
WDI_agriculture	HQER-SFA	-9382.15396391565	18778.3079278313	18825.83448233846	1.2897252997169235	0.8492424928254257	0.8469137539409048	0.0

Panel 3 of 4

Columns shown: `dataset, model, convergence_status, converged, n_iter, n_quad, max_iter, n_starts, best_start_id`.

dataset	model	convergence_status	converged	n_iter	n_quad	max_iter	n_starts	best_start_id
front41Data	Quantile-SFA	converged	True	27	28	220	5	4
front41Data	HQER-SFA	converged	True	20	28	220	5	4
WDI_industry	Quantile-SFA	converged	True	18	28	220	5	4
WDI_industry	HQER-SFA	converged	True	39	28	220	5	5
WDI_agriculture	Quantile-SFA	converged	True	26	28	220	5	1
WDI_agriculture	HQER-SFA	converged	True	33	28	220	5	4

Panel 4 of 4

Columns shown: `dataset, model, elapsed_time_seconds, warning_message`.

dataset	model	elapsed_time_seconds	warning_message
front41Data	Quantile-SFA	0.660288600018248	
front41Data	HQER-SFA	2.3527707000030205	
WDI_industry	Quantile-SFA	19.960232199984603	
WDI_industry	HQER-SFA	33.79990869999165	
WDI_agriculture	Quantile-SFA	18.001709899981506	
WDI_agriculture	HQER-SFA	34.10950090002734	

S3 Full Traditional-SFA robustness table

Source CSV: `tables/s3_full_traditional_sfa_robustness.csv`. Rows displayed: 9; columns displayed: 21. The table is split into panels to keep all cells inside the page boundary.

Panel 1 of 3

Columns shown: `dataset`, `model`, `n`, `loglik`, `AIC`, `BIC`, `validation_RMSE`, `mean_TE`, `median_TE`.

dataset	model	n	loglik	AIC	BIC	validation_RMSE	mean_TE	median_TE
front41Data	Traditional-SFA	60	-17.027225450396088	44.054450900792176	54.52617371190268	0.4761030020357927	0.7324526858769058	0.7489299734316981
front41Data	Quantile-SFA	60	-5.00881320472594	22.01762640945188	34.58369378278448	0.4195167862143548	0.9505300363644172	0.9505295574211952
front41Data	HQER-SFA	60	-3.823837565424783	21.647675130849567	36.30808706640427	0.4372322056016008	0.9505301456039678	0.9505299950969484
WDI_industry	Traditional-SFA	5546	-3306.4254209147757	6622.850841829551	6655.955002960429	0.4662978799757573	0.987028827285218	0.9870257172943556
WDI_industry	Quantile-SFA	5546	-1695.1828369735003	3402.3656739470007	3442.090667304055	0.4668834420135443	0.950530141828608	0.9505305052726604
WDI_industry	HQER-SFA	5546	-1717.264910646754	3448.529821293508	3494.8756468767383	0.4668811896883829	0.9505301496139272	0.9505304517721544
WDI_agriculture	Traditional-SFA	6565	-9429.319455086084	18868.638910172165	18902.58644910585	1.4374626870384368	0.4194203650123668	0.4264086522148181
WDI_agriculture	Quantile-SFA	6565	-9516.47440276099	19044.94880552198	19085.6858522424	1.535438666351823	0.4138575113094022	0.4041766981090974
WDI_agriculture	HQER-SFA	6565	-9387.313136090328	18788.626272180656	18836.152826687816	1.1987322772035045	0.8488998407873726	0.8463129052697776

Panel 2 of 3

Columns shown: `dataset`, `model`, `low_efficiency_ratio`, `tau_hat`, `gamma_hat`, `sigma_v_hat`, `sigma_u_hat`, `converged`, `n_iter`.

dataset	model	low_efficiency_ratio	tau_hat	gamma_hat	sigma_v_hat	sigma_u_hat	converged	n_iter
front41Data	Traditional-SFA	0.15			0.2097693059219836	0.4159276474021468	True	13
front41Data	Quantile-SFA	0.0	0.7547700626769656	0.0	0.0923463663629484	0.0507797037692787	True	33
front41Data	HQER-SFA	0.0	0.8021253377016503	0.3231480141547307	0.186520435019995	0.0506806124061472	True	52
WDI_industry	Traditional-SFA	0.0			0.4389417650314148	0.0163633409376071	True	38
WDI_industry	Quantile-SFA	0.0	0.4604005645526978	0.0	0.1541097219242705	0.0507233000521415	True	22
WDI_industry	HQER-SFA	0.0	0.4358044452656804	0.0390052714090783	0.1862784476413065	0.0507325080463733	True	46
WDI_agriculture	Traditional-SFA	0.8191926884996192			0.7046777953821455	1.2458638404743958	True	17
WDI_agriculture	Quantile-SFA	0.7509520182787509	0.5212784543827571	0.0	0.2612439142932133	1.265791448193059	True	24
WDI_agriculture	HQER-SFA	0.0	0.6931300284224693	0.7766898931453844	0.8769402452668444	0.2020250733920158	True	24

Panel 3 of 3

Columns shown: `dataset`, `model`, `best_start_id`, `max_iter`, `n_quad`, `n_starts`, `warning_message`.

dataset	model	best_start_id	max_iter	n_quad	n_starts	warning_message
front41Data	Traditional-SFA	3	220	28	5	
front41Data	Quantile-SFA	2	220	28	5	
front41Data	HQER-SFA	2	220	28	5	
WDI_industry	Traditional-SFA	5	220	28	5	
WDI_industry	Quantile-SFA	2	220	28	5	
WDI_industry	HQER-SFA	2	220	28	5	
WDI_agriculture	Traditional-SFA	1	220	28	5	
WDI_agriculture	Quantile-SFA	4	220	28	5	
WDI_agriculture	HQER-SFA	2	220	28	5	

S4 R = 100 Monte Carlo full summary

Source CSV: `tables/s4_r100_monte_carlo_full_summary.csv`. Rows displayed: 60; columns displayed: 12. The table is split into panels to keep all cells inside the page boundary.

Panel 1 of 2

Columns shown: `dataset_source, error_scenario, sample_size_label, fitted_model, mean_beta_error, std_beta_error, mean_y_rmse, std_y_rmse, mean_te_rmse`.

dataset_source	error_scenario	sample_size_label	fitted_model	mean_beta_error	std_beta_error	mean_y_rmse	std_y_rmse	mean_te_rmse
WDI_agriculture	ald	large	HQER-SFA	0.2347737387713872	0.2626258599313636	2.7991234015352635	1.3242746797824598	0.1957567754211884
WDI_agriculture	ald	large	Quantile-SFA	0.1366576078280411	0.1494838346183648	2.7433410850912963	1.2592224267058831	0.1509970332781155
WDI_agriculture	ald	medium	HQER-SFA	0.3417264647390513	0.3254361551539073	2.7842853858901333	1.3268217127386313	0.2191149012973763
WDI_agriculture	ald	medium	Quantile-SFA	0.2237446374572255	0.1691074510405144	2.729240832573134	1.2547773979645087	0.1553281374303944
WDI_agriculture	hqer_induced	large	HQER-SFA	0.0807239328858521	0.0398472337528217	1.314832767986872	0.1651376719851027	0.1440875328490232
WDI_agriculture	hqer_induced	large	Quantile-SFA	0.3454537940447055	0.2635916378860674	1.4981176716650677	0.0686803398903281	0.3413370406712743
WDI_agriculture	hqer_induced	medium	HQER-SFA	0.1620520546106252	0.0916671519887064	1.3089284298051949	0.1767920270061667	0.1504871072797057
WDI_agriculture	hqer_induced	medium	Quantile-SFA	0.374136750879222	0.2272929271734349	1.4779067026115624	0.1146282179478741	0.3462838896257946
WDI_agriculture	normal	large	HQER-SFA	0.1037378916822309	0.0488692618831664	1.1611088414160604	0.2277768713619528	0.1207883904430788
WDI_agriculture	normal	large	Quantile-SFA	0.3270489256263718	0.2129341595043503	1.2649389153057031	0.1317826047869865	0.2848545075717162
WDI_agriculture	normal	medium	HQER-SFA	0.1620446388857303	0.0790088420168936	1.1540659114215135	0.2206304365398705	0.1257523405166425
WDI_agriculture	normal	medium	Quantile-SFA	0.3579801075858866	0.2531911016333376	1.2643455983269771	0.1421073972223524	0.2919735088104528
WDI_agriculture	skewed	large	HQER-SFA	0.444051284908236	0.3715747676690953	1.2276586201339603	0.1150097012481527	0.1335995215726872
WDI_agriculture	skewed	large	Quantile-SFA	0.3108004005631479	0.2389909562020142	1.1659101574714414	0.1767947535608552	0.1726731878395087
WDI_agriculture	skewed	medium	HQER-SFA	0.471007885226265	0.3538911941675823	1.2199095007146818	0.1295523655082547	0.1401039182901716
WDI_agriculture	skewed	medium	Quantile-SFA	0.3253459509849877	0.2389191796622817	1.1588972413791168	0.1829802860024015	0.1817997438691663
WDI_agriculture	student_t	large	HQER-SFA	0.0705488350325463	0.0385056234390862	1.1402053776119898	0.2121315006463904	0.1289283941934107
WDI_agriculture	student_t	large	Quantile-SFA	0.072602751793337	0.0452707512197774	1.137724325589817	0.2117195447732808	0.130424647904835
WDI_agriculture	student_t	medium	HQER-SFA	0.1191830038198623	0.0592650628550682	1.1251483383243672	0.2253240280382799	0.1361627797695923
WDI_agriculture	student_t	medium	Quantile-SFA	0.1196802743221896	0.0596358088563764	1.1257989038971177	0.228651127839076	0.1360543300135742
WDI_industry	ald	large	HQER-SFA	0.0273725855807198	0.0145351541845371	0.4903904907001996	0.050628576035163	0.0307154321714768
WDI_industry	ald	large	Quantile-SFA	0.0273338035116742	0.0147224797562174	0.4903052827504565	0.0505353514874414	0.030715432448654
WDI_industry	ald	medium	HQER-SFA	0.0544172542109112	0.0284367436835557	0.4870670045493562	0.0519353462773213	0.0307274384533139
WDI_industry	ald	medium	Quantile-SFA	0.0541728466367981	0.0287603776831465	0.4869826709639862	0.0518336890897345	0.0307274382752804
WDI_industry	hqer_induced	large	HQER-SFA	0.0267986253159544	0.0148353441171778	0.4177725541733532	0.0104316658315788	0.0306832558690334
WDI_industry	hqer_induced	large	Quantile-SFA	0.0275515247606004	0.0147108042373761	0.4177664391233068	0.0104064811526392	0.0306832561744153
WDI_industry	hqer_induced	medium	HQER-SFA	0.0515094318358729	0.0284049376371744	0.41791082706276	0.0160280044614423	0.0307440074497147
WDI_industry	hqer_induced	medium	Quantile-SFA	0.051158264091336	0.0287610481661473	0.4179383950964325	0.0160303637610638	0.0307440081352362
WDI_industry	normal	large	HQER-SFA	0.019510544432694	0.0106640492155954	0.1815112776306713	0.0157392460275247	0.0306292205393068
WDI_industry	normal	large	Quantile-SFA	0.0203565490298772	0.011481749051553	0.1815243366726419	0.0159181077020009	0.0306292317996192
WDI_industry	normal	medium	HQER-SFA	0.0357810051292848	0.0185677235883375	0.181242234257196	0.0188470149092143	0.030786642256688
WDI_industry	normal	medium	Quantile-SFA	0.0366117180374612	0.0179894263529332	0.1804716503567082	0.0180748841998944	0.030786650860899
WDI_industry	skewed	large	HQER-SFA	0.1287769414760865	0.0265928803520928	0.1948748131197252	0.0259117658767955	0.0306989346955083
WDI_industry	skewed	large	Quantile-SFA	0.1183890424637996	0.0177739746921516	0.1899348476137194	0.0211823821222665	0.0306989517269493
WDI_industry	skewed	medium	HQER-SFA	0.1298853709513132	0.0268070106078834	0.1946967709407905	0.0251598675712864	0.0306487726697495
WDI_industry	skewed	medium	Quantile-SFA	0.117828927939194	0.0196355532793413	0.1892122034441686	0.0197703147628923	0.0306487808116755
WDI_industry	student_t	large	HQER-SFA	0.0157019885292579	0.0061884992903669	0.1785071274850168	0.0202349820225096	0.0306727303547461
WDI_industry	student_t	large	Quantile-SFA	0.0155661446205222	0.0062962262071514	0.1784257165382573	0.0202352880962065	0.0306727269177416
WDI_industry	student_t	medium	HQER-SFA	0.0248625478994648	0.0115912058266511	0.1773356982940042	0.0264012897119068	0.0306883324823868
WDI_industry	student_t	medium	Quantile-SFA	0.0242547314719708	0.0116771622322795	0.1772260196920641	0.0263022041189048	0.0306883280691427
front41Data	ald	medium	HQER-SFA	0.0513685064436209	0.0371541198463823	0.8220213292692904	0.3703955434584102	0.0307244119664674
front41Data	ald	medium	Quantile-SFA	0.0327468064491683	0.0170864334113433	0.8057757003585104	0.3531386985727908	0.0307244120793967
front41Data	ald	small	HQER-SFA	0.1174878811462277	0.0778875076692731	0.807414796132049	0.3925592857922848	0.0303455316097952
front41Data	ald	small	Quantile-SFA	0.0993246957271615	0.0574363746179648	0.7912506630982763	0.3743853576349988	0.0303455322038382
front41Data	hqer_induced	medium	HQER-SFA	0.0264959472160999	0.0140963822214994	0.3888376724877768	0.0291595673992716	0.0306040288882891
front41Data	hqer_induced	medium	Quantile-SFA	0.0302146350304477	0.018029988593165	0.3842796922058471	0.0269287018737153	0.0306040338161566
front41Data	hqer_induced	small	HQER-SFA	0.0853311311556541	0.0534613670480987	0.3942185928712985	0.0619617340237983	0.030650633450253
front41Data	hqer_induced	small	Quantile-SFA	0.0819679567696688	0.0450582462201021	0.3899680716836686	0.0594100939763863	0.0306506409403781
front41Data	normal	medium	HQER-SFA	0.0252858867930943	0.0140816938024311	0.1647595768542825	0.0392435475411361	0.030513239920992
front41Data	normal	medium	Quantile-SFA	0.0289642825147572	0.0191424397333468	0.1662804361453541	0.0410466113548255	0.0305132519668247
front41Data	normal	small	HQER-SFA	0.0649532683524981	0.0391171781449445	0.1706484296545905	0.0471869537748688	0.0305520613244799
front41Data	normal	small	Quantile-SFA	0.0685068223315465	0.0398257381289205	0.1750686170525786	0.0515571931180735	0.0305520871270878
front41Data	skewed	medium	HQER-SFA	0.1062436380867102	0.0473997333057159	0.1693180680294012	0.0550013121871824	0.0306456300314659
front41Data	skewed	medium	Quantile-SFA	0.095381305127218	0.0390807646928537	0.1646854976352488	0.0505275186293662	0.0306456365906227
front41Data	skewed	small	HQER-SFA	0.0940150392781187	0.0543828691879914	0.1683043934245561	0.0518138507523788	0.0305015596086118
front41Data	skewed	small	Quantile-SFA	0.0783397762968545	0.0452510085451657	0.165542350820194	0.0488373878795166	0.0305015727965114
front41Data	student_t	medium	HQER-SFA	0.0171696682467886	0.008883173746022	0.1606639118214132	0.0489969716250184	0.0305914420836395
front41Data	student_t	medium	Quantile-SFA	0.0173889724191039	0.008800339472283	0.160773653976025	0.0489955728820051	0.0305914406353963
front41Data	student_t	small	HQER-SFA	0.0486385957632074	0.0316208318112523	0.161588266486221	0.065580962290792	0.0306851764127229
front41Data	student_t	small	Quantile-SFA	0.0452238010128886	0.0250779012062823	0.1611236316030986	0.0631826516481749	0.0306851684988584

Panel 2 of 2

Columns shown: `dataset_source, error_scenario, sample_size_label, fitted_model, std_te_rmse, mean_te_rank_corr, convergence_rate`.

dataset_source	error_scenario	sample_size_label	fitted_model	std_te_rmse	mean_te_rank_corr	convergence_rate
WDI_agriculture	ald	large	HQER-SFA	0.1424606272432215	0.3756523342255835	1.0
WDI_agriculture	ald	large	Quantile-SFA	0.043809517446759	0.3776834721798137	1.0
WDI_agriculture	ald	medium	HQER-SFA	0.1726937849910758	0.3744173659894639	1.0
WDI_agriculture	ald	medium	Quantile-SFA	0.0572573045768672	0.3766627985446236	1.0
WDI_agriculture	hqer_induced	large	HQER-SFA	0.0460634549273647	0.4147762047140512	1.0
WDI_agriculture	hqer_induced	large	Quantile-SFA	0.1522515695847517	0.4142364210710049	1.0
WDI_agriculture	hqer_induced	medium	HQER-SFA	0.0626603392168316	0.4113307040428161	1.0

dataset_source	error_scenario	sample_size_label	fitted_model	std_te_rmse	mean_te_rank_corr	convergence_rate
WDI_agriculture	hqer_induced	medium	Quantile-SFA	0.1515058525195855	0.4105606154323478	1.0
WDI_agriculture	normal	large	HQER-SFA	0.0254012254468359	0.5231370496042623	1.0
WDI_agriculture	normal	large	Quantile-SFA	0.1447307015819829	0.5222281980968291	1.0
WDI_agriculture	normal	medium	HQER-SFA	0.0390777258466214	0.5239983463933855	1.0
WDI_agriculture	normal	medium	Quantile-SFA	0.1467865720793887	0.5230230050817976	1.0
WDI_agriculture	skewed	large	HQER-SFA	0.0269344303095281	0.5285597727649431	1.0
WDI_agriculture	skewed	large	Quantile-SFA	0.038441569465996	0.5253685173657272	1.0
WDI_agriculture	skewed	medium	HQER-SFA	0.0650479553383827	0.5298519496477986	1.0
WDI_agriculture	skewed	medium	Quantile-SFA	0.0773548510362022	0.5271152078256958	1.0
WDI_agriculture	student_t	large	HQER-SFA	0.0336170308027587	0.5562010960769257	1.0
WDI_agriculture	student_t	large	Quantile-SFA	0.0444361140163979	0.5537699389484938	1.0
WDI_agriculture	student_t	medium	HQER-SFA	0.061705190067417	0.5516936526946108	1.0
WDI_agriculture	student_t	medium	Quantile-SFA	0.0612491503000224	0.5484495947642644	1.0
WDI_industry	ald	large	HQER-SFA	0.0003838487110045	0.0721488102946547	1.0
WDI_industry	ald	large	Quantile-SFA	0.0003838487722812	0.0761011257528783	1.0
WDI_industry	ald	medium	HQER-SFA	0.0008694281774086	0.0803589617638415	1.0
WDI_industry	ald	medium	Quantile-SFA	0.0008694277563583	0.0845794493973502	1.0
WDI_industry	hqer_induced	large	HQER-SFA	0.0003982343952708	0.0797526319461287	1.0
WDI_industry	hqer_induced	large	Quantile-SFA	0.0003982345395408	0.0816341537947344	1.0
WDI_industry	hqer_induced	medium	HQER-SFA	0.0007824780303134	0.0784230137098005	1.0
WDI_industry	hqer_induced	medium	Quantile-SFA	0.000782477427052	0.0801305940534257	1.0
WDI_industry	normal	large	HQER-SFA	0.000411084991511	0.1644389885107951	1.0
WDI_industry	normal	large	Quantile-SFA	0.0004110823719405	0.1502716219294689	1.0
WDI_industry	normal	medium	HQER-SFA	0.0008305359541467	0.1675737559151482	1.0
WDI_industry	normal	medium	Quantile-SFA	0.0008305351125135	0.1528908384924056	1.0
WDI_industry	skewed	large	HQER-SFA	0.0004247216415583	0.1781290009307712	1.0
WDI_industry	skewed	large	Quantile-SFA	0.0004247281917478	0.1851465447555438	1.0
WDI_industry	skewed	medium	HQER-SFA	0.0008145340780829	0.1752550691802766	1.0
WDI_industry	skewed	medium	Quantile-SFA	0.0008145394625175	0.1831626837757399	1.0
WDI_industry	student_t	large	HQER-SFA	0.0003956710958731	0.2274205960522186	1.0
WDI_industry	student_t	large	Quantile-SFA	0.0003956713762659	0.2206806323615315	1.0
WDI_industry	student_t	medium	HQER-SFA	0.0008472080004521	0.2215349309573905	1.0
WDI_industry	student_t	medium	Quantile-SFA	0.0008472082533843	0.2174687230289878	1.0
front41Data	ald	medium	HQER-SFA	0.0008776053293721	0.0681573494293976	1.0
front41Data	ald	medium	Quantile-SFA	0.0008776055147935	0.07074132364573	1.0
front41Data	ald	small	HQER-SFA	0.0023908555265645	0.0510661217714932	1.0
front41Data	ald	small	Quantile-SFA	0.0023908531457898	0.0574836696773382	1.0
front41Data	hqer_induced	medium	HQER-SFA	0.0008396978255005	0.0917475885903543	1.0
front41Data	hqer_induced	medium	Quantile-SFA	0.0008396986959148	0.087530222193629	1.0
front41Data	hqer_induced	small	HQER-SFA	0.0023803354511395	0.0874984687133823	1.0
front41Data	hqer_induced	small	Quantile-SFA	0.0023803312119887	0.0864818092774994	1.0
front41Data	normal	medium	HQER-SFA	0.0008407060669801	0.1934139189441341	1.0
front41Data	normal	medium	Quantile-SFA	0.0008407067978222	0.1803330401352117	1.0
front41Data	normal	small	HQER-SFA	0.0022423115958867	0.1912108919144206	1.0
front41Data	normal	small	Quantile-SFA	0.0022423179384149	0.1750410714070542	1.0
front41Data	skewed	medium	HQER-SFA	0.0009342133396076	0.2103791744203326	1.0
front41Data	skewed	medium	Quantile-SFA	0.0009342123040058	0.2183554140458898	1.0
front41Data	skewed	small	HQER-SFA	0.0022061843483047	0.1898399555432064	1.0
front41Data	skewed	small	Quantile-SFA	0.0022061542580885	0.191319995735119	1.0
front41Data	student_t	medium	HQER-SFA	0.0008015260746696	0.2602360959287317	1.0
front41Data	student_t	medium	Quantile-SFA	0.0008015257986107	0.2533353624765845	1.0
front41Data	student_t	small	HQER-SFA	0.0020933681017818	0.254419314776668	1.0
front41Data	student_t	small	Quantile-SFA	0.0020933773216645	0.2457200427330725	1.0

S5 R = 300 Monte Carlo full summary

Source CSV: `tables/s5_r300_monte_carlo_full_summary.csv`. Rows displayed: 120; columns displayed: 16. The table is split into panels to keep all cells inside the page boundary.

Panel 1 of 3

Columns shown: `dataset_source, pseudo_model_source, error_scenario, sample_size_label, fitted_model, mean_beta_error, std_beta_error, mean_tau_error, mean_gamma_error`.

dataset__source	pseudo__model__source	error__scenario	sample__size__label	fitted__model	mean__beta__error	std__beta__error	mean__tau__error	mean__gamma__error
WDI__agriculture	HQER-SFA	ald	large	HQER-SFA	0.3933125035022774	0.3159809853399491	0.1143619230556261	0.6866572336627574
WDI__agriculture	HQER-SFA	ald	large	Quantile-SFA	0.2109492951972451	0.2337902374413298	0.0082703585930393	
WDI__agriculture	HQER-SFA	ald	medium	HQER-SFA	0.5619016184669897	0.3934649810238707	0.1418391654005707	0.6581362012209332
WDI__agriculture	HQER-SFA	ald	medium	Quantile-SFA	0.2943085612699856	0.2166134498546402	0.0163429823223316	
WDI__agriculture	HQER-SFA	hqer__induced	large	HQER-SFA	0.071636641429653	0.0393283384739211	0.0134557809616028	0.0007722355110054
WDI__agriculture	HQER-SFA	hqer__induced	large	Quantile-SFA	0.5825238117425231	0.0961345648060986	0.1754487261379371	
WDI__agriculture	HQER-SFA	hqer__induced	medium	HQER-SFA	0.1562684645682151	0.0950008489117322	0.0340313491275032	0.0017375081996156
WDI__agriculture	HQER-SFA	hqer__induced	medium	Quantile-SFA	0.5808979615482976	0.1497943485343222	0.1940455489573821	
WDI__agriculture	HQER-SFA	normal	large	HQER-SFA	0.077310781682583	0.0419180059863635	0.2112187876277652	0.0025734491528566
WDI__agriculture	HQER-SFA	normal	large	Quantile-SFA	0.5288788273528379	0.1046898482599449	0.290389318048454	
WDI__agriculture	HQER-SFA	normal	medium	HQER-SFA	0.1555114608314359	0.0844008365900635	0.2126446104784658	0.0023243713797413
WDI__agriculture	HQER-SFA	normal	medium	Quantile-SFA	0.5423904616042141	0.1875582067637103	0.3023523575086449	
WDI__agriculture	HQER-SFA	skewed	large	HQER-SFA	0.7939778056328628	0.0853061672262207	0.6311680099244295	0.0043405388310626
WDI__agriculture	HQER-SFA	skewed	large	Quantile-SFA	0.5369777604680457	0.1113260437939581	0.5178782573275973	
WDI__agriculture	HQER-SFA	skewed	medium	HQER-SFA	0.815650674038588	0.1154559014589845	0.6344871947199809	0.0044171177929778
WDI__agriculture	HQER-SFA	skewed	medium	Quantile-SFA	0.5289699307213669	0.1899195970322986	0.5148015218086439	
WDI__agriculture	HQER-SFA	student__t	large	HQER-SFA	0.0590864303407856	0.0406602587401622	0.214072795322146	0.9152809665836192
WDI__agriculture	HQER-SFA	student__t	large	Quantile-SFA	0.0594357853189171	0.0425541344217637	0.213139931478024	
WDI__agriculture	HQER-SFA	student__t	medium	HQER-SFA	0.1124286363721427	0.0635715696030342	0.215208969680518	0.9026086569160592
WDI__agriculture	HQER-SFA	student__t	medium	Quantile-SFA	0.1139412162036435	0.0741324370635624	0.2123837564619528	
WDI__agriculture	Quantile-SFA	ald	large	HQER-SFA	0.0880787622885883	0.0461951140031621	0.0343330987997305	0.0003286431278571
WDI__agriculture	Quantile-SFA	ald	large	Quantile-SFA	0.088932782599026	0.0457032172447331	0.0336814093755135	
WDI__agriculture	Quantile-SFA	ald	medium	HQER-SFA	0.1739095654039202	0.0950249948503112	0.0598624422202879	0.0003286431278571
WDI__agriculture	Quantile-SFA	ald	medium	Quantile-SFA	0.1785776298397556	0.099737431706424	0.0605165020167833	
WDI__agriculture	Quantile-SFA	hqer__induced	large	HQER-SFA	0.0921658261076641	0.0462966542451634	0.0307971488887757	0.0003286431278571
WDI__agriculture	Quantile-SFA	hqer__induced	large	Quantile-SFA	0.0922806697381156	0.0451531627339981	0.0313143844047981	
WDI__agriculture	Quantile-SFA	hqer__induced	medium	HQER-SFA	0.1776225577504792	0.0988645618840878	0.0522605411294261	0.0003286431278571
WDI__agriculture	Quantile-SFA	hqer__induced	medium	Quantile-SFA	0.1807868352796695	0.1007478377331676	0.0533288336126514	
WDI__agriculture	Quantile-SFA	normal	large	HQER-SFA	0.1338973260846556	0.0471612400205018	0.2512665143562937	0.0003286431278571
WDI__agriculture	Quantile-SFA	normal	large	Quantile-SFA	0.1323555299947999	0.0464526540050959	0.2499113272703275	
WDI__agriculture	Quantile-SFA	normal	medium	HQER-SFA	0.1519537672527095	0.0680756723026937	0.2326621462001774	0.0003286431278571
WDI__agriculture	Quantile-SFA	normal	medium	Quantile-SFA	0.1467307216434512	0.0640591084779486	0.2293758029549787	
WDI__agriculture	Quantile-SFA	skewed	large	HQER-SFA	0.0813675186578511	0.0452871584180062	0.2066639536056514	0.0003286431278571
WDI__agriculture	Quantile-SFA	skewed	large	Quantile-SFA	0.0846051096476884	0.048340642953157	0.2054432268766562	
WDI__agriculture	Quantile-SFA	skewed	medium	HQER-SFA	0.1313976729151506	0.0624457085848685	0.1750964539010851	0.0003286431278571
WDI__agriculture	Quantile-SFA	skewed	medium	Quantile-SFA	0.1326890063430674	0.063094492997835	0.1752101830142422	
WDI__agriculture	Quantile-SFA	student__t	large	HQER-SFA	0.0822482257263944	0.0382953151771384	0.2393824325392035	0.0003286431278571
WDI__agriculture	Quantile-SFA	student__t	large	Quantile-SFA	0.0820844618476306	0.0352006471153672	0.2370045958436047	
WDI__agriculture	Quantile-SFA	student__t	medium	HQER-SFA	0.1157494813094107	0.0564424365889405	0.2214343766532811	0.0003286431278571
WDI__agriculture	Quantile-SFA	student__t	medium	Quantile-SFA	0.1198664441358052	0.0585647295353085	0.2219967287220862	
WDI__industry	HQER-SFA	ald	large	HQER-SFA	0.0293820839994933	0.0157054126112	0.0129392063164744	0.0056137836671778
WDI__industry	HQER-SFA	ald	large	Quantile-SFA	0.0294091277711157	0.0155726705149182	0.0090147616836884	
WDI__industry	HQER-SFA	ald	medium	HQER-SFA	0.0607965957440756	0.0335019855603188	0.023480792885712	0.0051865601017043
WDI__industry	HQER-SFA	ald	medium	Quantile-SFA	0.0609630858260858	0.0333606194993665	0.019611450916907	
WDI__industry	HQER-SFA	hqer__induced	large	HQER-SFA	0.028033708931868	0.0144094694910684	0.011832483659737	0.0044191908696145
WDI__industry	HQER-SFA	hqer__induced	large	Quantile-SFA	0.029747186058594	0.0147599211989364	0.0139671233155712	
WDI__industry	HQER-SFA	hqer__induced	medium	HQER-SFA	0.0562620263544269	0.0289686827651133	0.0223462570117039	0.0074551846423674
WDI__industry	HQER-SFA	hqer__induced	medium	Quantile-SFA	0.0561454318619183	0.0295829043955876	0.0213030297211883	
WDI__industry	HQER-SFA	normal	large	HQER-SFA	0.0202171930578494	0.0103746012888414	0.05664771215004	0.6706297225508075
WDI__industry	HQER-SFA	normal	large	Quantile-SFA	0.0218075182441602	0.0114825895558116	0.0513257268055492	
WDI__industry	HQER-SFA	normal	medium	HQER-SFA	0.0372049940813591	0.0191228562728144	0.0719299235153604	0.4497491665863941
WDI__industry	HQER-SFA	normal	medium	Quantile-SFA	0.0394543724289681	0.0199384299263534	0.0549004471463752	
WDI__industry	HQER-SFA	skewed	large	HQER-SFA	0.1531861412586428	0.0125931901235721	0.2877860738157821	0.0347967696414255
WDI__industry	HQER-SFA	skewed	large	Quantile-SFA	0.1331177033281653	0.0107327142924318	0.2015908441149916	
WDI__industry	HQER-SFA	skewed	medium	HQER-SFA	0.1540983840358784	0.0180349442932943	0.2859301844527613	0.0296778667129403
WDI__industry	HQER-SFA	skewed	medium	Quantile-SFA	0.1311636885950435	0.0199452314645473	0.1958257946540741	
WDI__industry	HQER-SFA	student__t	large	HQER-SFA	0.0162910397200231	0.0068734196744125	0.0548914465076817	0.0207893160174966
WDI__industry	HQER-SFA	student__t	large	Quantile-SFA	0.0159381925889624	0.0068557690410972	0.0531281454130708	
WDI__industry	HQER-SFA	student__t	medium	HQER-SFA	0.0274355384452631	0.0132479185902022	0.0637534562959103	0.0141667115412308
WDI__industry	HQER-SFA	student__t	medium	Quantile-SFA	0.0260730979913912	0.0131809862350419	0.0583612385535007	
WDI__industry	Quantile-SFA	ald	large	HQER-SFA	0.0257289413873077	0.0130749798734048	0.0095633147764967	0.0003286431278571
WDI__industry	Quantile-SFA	ald	large	Quantile-SFA	0.0257457220508376	0.0129981868387153	0.0095404455922413	
WDI__industry	Quantile-SFA	ald	medium	HQER-SFA	0.048547189848283	0.0269502831657264	0.0189404943965869	0.0003286431278571
WDI__industry	Quantile-SFA	ald	medium	Quantile-SFA	0.0486172945392996	0.0271765977142834	0.0189422408076543	
WDI__industry	Quantile-SFA	hqer__induced	large	HQER-SFA	0.0279129618955401	0.0136640324807192	0.0104337165652179	0.0003286431278571
WDI__industry	Quantile-SFA	hqer__induced	large	Quantile-SFA	0.0280423442872774	0.0135978887423208	0.0104773597304505	
WDI__industry	Quantile-SFA	hqer__induced	medium	HQER-SFA	0.0482056249508738	0.0275418924763624	0.0188009100476689	0.0003286431278571
WDI__industry	Quantile-SFA	hqer__induced	medium	Quantile-SFA	0.048589763397402	0.0280579526626025	0.0188629450012644	
WDI__industry	Quantile-SFA	normal	large	HQER-SFA	0.0196583260735307	0.010529498976049	0.0399757397007009	0.0003286431278571
WDI__industry	Quantile-SFA	normal	large	Quantile-SFA	0.0196435137922538	0.0106036589770661	0.0400709122004971	
WDI__industry	Quantile-SFA	normal	medium	HQER-SFA	0.0334687865147513	0.018496380978287	0.0432670497350081	0.0003286431278571
WDI__industry	Quantile-SFA	normal	medium	Quantile-SFA	0.0337706835122905	0.0184933604201208	0.0434222739820162	
WDI__industry	Quantile-SFA	skewed	large	HQER-SFA	0.1055173129949268	0.0085534051922798	0.2103421420003013	0.0003286431278571
WDI__industry	Quantile-SFA	skewed	large	Quantile-SFA	0.1049841407969996	0.0084737126394905	0.2085551184131173	
WDI__industry	Quantile-SFA	skewed	medium	HQER-SFA	0.1059594912651516	0.0158713671568266	0.2063186482276045	0.0003286431278571
WDI__industry	Quantile-SFA	skewed	medium	Quantile-SFA	0.1053883419364875	0.0155301694878372	0.2045902930173188	
WDI__industry	Quantile-SFA	student__t	large	HQER-SFA	0.0151817268835538	0.0060035996052899	0.0421036618704581	0.0003286437513563
WDI__industry	Quantile-SFA	student__t	large	Quantile-SFA	0.0151522335181212	0.0060204103799042	0.0420484735393955	
WDI__industry	Quantile-SFA	student__t	medium	HQER-SFA	0.0229375026410235	0.0113349928194389	0.0480498287371378	0.0003286431278571
WDI__industry	Quantile-SFA	student__t	medium	Quantile-SFA	0.0228603762196791	0.0113396621315777	0.0480601668135289	
front41Data	HQER-SFA	ald	medium	HQER-SFA	0.0852796631867662	0.0409721965386344	0.0830555665586375	0.2392216141022679

dataset_source	pseudo_model_source	error_scenario	sample_size_label	fitted_model	mean_beta_error	std_beta_error	mean_tau_error	mean_gamma_error
front41Data	HQER-SFA	ald	medium	Quantile-SFA	0.0418065498252686	0.0204626569909515	0.0094513451668793	
front41Data	HQER-SFA	ald	small	HQER-SFA	0.1669666058541336	0.08145893192146	0.0998649078656588	0.1938587959269576
front41Data	HQER-SFA	ald	small	Quantile-SFA	0.1311618160928585	0.0691332727349999	0.026787122965801	
front41Data	HQER-SFA	hqer_induced	medium	HQER-SFA	0.0286403924608287	0.0149094635981312	0.0213517796601085	0.0323887193922689
front41Data	HQER-SFA	hqer_induced	medium	Quantile-SFA	0.0352570765850189	0.019812289861152	0.1116154126152684	
front41Data	HQER-SFA	hqer_induced	small	HQER-SFA	0.0971251807866856	0.0588999764405682	0.0898168436224719	0.1443781536327266
front41Data	HQER-SFA	hqer_induced	small	Quantile-SFA	0.0847035491298074	0.0442982198778938	0.0946645883947232	
front41Data	HQER-SFA	normal	medium	HQER-SFA	0.0263230508865551	0.0142082392473279	0.281755503274919	0.2786325660576216
front41Data	HQER-SFA	normal	medium	Quantile-SFA	0.0328205301066065	0.0193344580546019	0.2754915943077944	
front41Data	HQER-SFA	normal	small	HQER-SFA	0.0881398724088516	0.0452152908759729	0.2606720589992072	0.170149008415619
front41Data	HQER-SFA	normal	small	Quantile-SFA	0.0920966921448179	0.0418375672376824	0.2071785639920319	
front41Data	HQER-SFA	skewed	medium	HQER-SFA	0.1520146670067572	0.0144447266147294	0.681140899672767	0.1541688651616401
front41Data	HQER-SFA	skewed	medium	Quantile-SFA	0.1320333720076092	0.0207380987725462	0.5316270696389288	
front41Data	HQER-SFA	skewed	small	HQER-SFA	0.1462872072605329	0.0349111900401015	0.6530406109859315	0.2235303907729104
front41Data	HQER-SFA	skewed	small	Quantile-SFA	0.1136091190552232	0.0446663973887438	0.4460054677015226	
front41Data	HQER-SFA	student_t	medium	HQER-SFA	0.0183157578359398	0.0092984746772204	0.2836973417772067	0.3069000214581338
front41Data	HQER-SFA	student_t	medium	Quantile-SFA	0.0181830333246696	0.0092545612611566	0.2834875627409932	
front41Data	HQER-SFA	student_t	small	HQER-SFA	0.06084143801399	0.0381661894541588	0.2913144703243287	0.1871054238519154
front41Data	HQER-SFA	student_t	small	Quantile-SFA	0.0564221644634948	0.0295618191630211	0.2564653075932678	
front41Data	Quantile-SFA	ald	medium	HQER-SFA	0.0259838641203657	0.0117917260656627	0.0173731668880537	0.0003286431278571
front41Data	Quantile-SFA	ald	medium	Quantile-SFA	0.0259271813409791	0.011561927024613	0.017261078972819	
front41Data	Quantile-SFA	ald	small	HQER-SFA	0.0730420217957219	0.0364116540421934	0.050781808406929	0.0003286431278571
front41Data	Quantile-SFA	ald	small	Quantile-SFA	0.0728993909239977	0.0367140873004853	0.0515891529349559	
front41Data	Quantile-SFA	hqer_induced	medium	HQER-SFA	0.0239367298078438	0.0116956131677784	0.0413158544777804	0.0003286431278571
front41Data	Quantile-SFA	hqer_induced	medium	Quantile-SFA	0.0240160111439327	0.0118928577260039	0.0418753960850322	
front41Data	Quantile-SFA	hqer_induced	small	HQER-SFA	0.0729621115653173	0.0381229302778608	0.0554908341320962	0.0003286431278571
front41Data	Quantile-SFA	hqer_induced	small	Quantile-SFA	0.0735498190617114	0.0391589996844592	0.0571103849605091	
front41Data	Quantile-SFA	normal	medium	HQER-SFA	0.0230489992507161	0.0133198788051089	0.1806363457333972	0.0003286468905685
front41Data	Quantile-SFA	normal	medium	Quantile-SFA	0.0237600974872642	0.0132734274020919	0.1794060986689369	
front41Data	Quantile-SFA	normal	small	HQER-SFA	0.0465202751959076	0.0231291232913233	0.1442343368999523	0.0003286554014769
front41Data	Quantile-SFA	normal	small	Quantile-SFA	0.0465949567787554	0.0226846450671884	0.1448267827758742	
front41Data	Quantile-SFA	skewed	medium	HQER-SFA	0.0614301296346594	0.0116385312616813	0.4121057853191451	0.0003288556141186
front41Data	Quantile-SFA	skewed	medium	Quantile-SFA	0.0614145086092303	0.0117356979012673	0.4115038893384237	
front41Data	Quantile-SFA	skewed	small	HQER-SFA	0.0505280245591415	0.0230836353190327	0.3159503365416863	0.0003286500822943
front41Data	Quantile-SFA	skewed	small	Quantile-SFA	0.0506398674467187	0.0236618679174053	0.3182812133343049	
front41Data	Quantile-SFA	student_t	medium	HQER-SFA	0.015538055134953	0.007682937557305	0.1915096073193216	0.0003286431278571
front41Data	Quantile-SFA	student_t	medium	Quantile-SFA	0.015685132838355	0.0075226520060194	0.1909665859893401	
front41Data	Quantile-SFA	student_t	small	HQER-SFA	0.0315942603444714	0.015577596426807	0.1565243466404682	0.0003286953008101
front41Data	Quantile-SFA	student_t	small	Quantile-SFA	0.0309761613907933	0.0151791469985456	0.1612048776434314	

Panel 2 of 3

Columns shown: `dataset_source`, `pseudo_model_source`, `error_scenario`, `sample_size_label`, `fitted_model`, `mean_sigma_v_error`, `mean_sigma_u_error`, `mean_y_rmse`, `mean_te_rmse`.

dataset_source	pseudo_model_source	error_scenario	sample_size_label	fitted_model	mean_sigma_v_error	mean_sigma_u_error	mean_y_rmse	mean_te_rmse
WDI_agriculture	HQER-SFA	ald	large	HQER-SFA	0.2981503890090201	0.6547727708635049	4.09228486546946	0.2034170824345508
WDI_agriculture	HQER-SFA	ald	large	Quantile-SFA	0.025749323322113	0.0491468517569703	3.978159281220276	0.1113255140864235
WDI_agriculture	HQER-SFA	ald	medium	HQER-SFA	0.2507000257060407	0.9461488644747664	4.143699084338193	0.2453721023798942
WDI_agriculture	HQER-SFA	ald	medium	Quantile-SFA	0.0495756272789865	0.038954535639462	3.991897636103064	0.1101308113882565
WDI_agriculture	HQER-SFA	hqer_induced	large	HQER-SFA	0.0159696145056535	0.000251573522244	1.1589725006311935	0.0981415260251707
WDI_agriculture	HQER-SFA	hqer_induced	large	Quantile-SFA	0.6597208863284943	1.0801467648302308	1.5132134266073392	0.4911910717390793
WDI_agriculture	HQER-SFA	hqer_induced	medium	HQER-SFA	0.0419290021096064	0.006501960317005	1.1548162767349106	0.0999425890191657
WDI_agriculture	HQER-SFA	hqer_induced	medium	Quantile-SFA	0.672548528897812	1.1002520966960327	1.4969189331644026	0.4934786168012249
WDI_agriculture	HQER-SFA	normal	large	HQER-SFA	0.0249559185959959	0.0036061685315691	0.9348021335561696	0.0986686720834874
WDI_agriculture	HQER-SFA	normal	large	Quantile-SFA	0.667396218891767	0.8394745679984793	1.1476962724799948	0.4286995077295468
WDI_agriculture	HQER-SFA	normal	medium	HQER-SFA	0.0344848657056661	0.0056279934053058	0.9400720785063452	0.0995356062938882
WDI_agriculture	HQER-SFA	normal	medium	Quantile-SFA	0.6785440722197118	0.8581716744056312	1.1523900294076592	0.4324253643433572
WDI_agriculture	HQER-SFA	skewed	large	HQER-SFA	0.398744264626349	0.064752181901737	1.1176384608604557	0.1133622991723375
WDI_agriculture	HQER-SFA	skewed	large	Quantile-SFA	0.7227778577601126	0.2479080484279659	0.9978727539110338	0.195045705507548
WDI_agriculture	HQER-SFA	skewed	medium	HQER-SFA	0.4101604909030406	0.0677854456456656	1.127324732331623	0.1139800830488913
WDI_agriculture	HQER-SFA	skewed	medium	Quantile-SFA	0.7233166300319316	0.2564020929454507	1.0010999726167922	0.2013526626951664
WDI_agriculture	HQER-SFA	student_t	large	HQER-SFA	0.6099356979099069	0.0129393247980851	0.930000036000876	0.1005492485729846
WDI_agriculture	HQER-SFA	student_t	large	Quantile-SFA	0.6217062646278277	0.0115055189921591	0.9291478255029392	0.1000028155466798
WDI_agriculture	HQER-SFA	student_t	medium	HQER-SFA	0.6050372460382684	0.0282029096950379	0.9148588366583408	0.1053293514181815
WDI_agriculture	HQER-SFA	student_t	medium	Quantile-SFA	0.6262500209389201	0.0305179295391729	0.9147987222280566	0.1065274580510903
WDI_agriculture	Quantile-SFA	ald	large	HQER-SFA	0.0194313038256426	0.10884074264821	1.4918860872344104	0.1926204635289912
WDI_agriculture	Quantile-SFA	ald	large	Quantile-SFA	0.0185749355247466	0.1058662537682343	1.492237730427859	0.1926164905709541
WDI_agriculture	Quantile-SFA	ald	medium	HQER-SFA	0.0290679025704071	0.1615540671245349	1.4785067532621057	0.2014055539219673
WDI_agriculture	Quantile-SFA	ald	medium	Quantile-SFA	0.029528361734174	0.1608102490109532	1.4806572248136818	0.2025649658299904
WDI_agriculture	Quantile-SFA	hqer_induced	large	HQER-SFA	0.0106243833885333	0.0703560668620365	1.4791426130922385	0.1905593113621572
WDI_agriculture	Quantile-SFA	hqer_induced	large	Quantile-SFA	0.0104491425716351	0.0717837953563396	1.4793551655513808	0.1904954851898014
WDI_agriculture	Quantile-SFA	hqer_induced	medium	HQER-SFA	0.028600720806768	0.1218031969996621	1.46266680294873	0.1964712718808904
WDI_agriculture	Quantile-SFA	hqer_induced	medium	Quantile-SFA	0.0292852221793291	0.1221504449902688	1.4631883847292655	0.1965402797643236
WDI_agriculture	Quantile-SFA	normal	large	HQER-SFA	0.1277330832725848	0.37692401475888	1.386407358349573	0.144856035904721
WDI_agriculture	Quantile-SFA	normal	large	Quantile-SFA	0.128329515561832	0.3719867913266469	1.3868627987882187	0.1428164978779638
WDI_agriculture	Quantile-SFA	normal	medium	HQER-SFA	0.1262766248613861	0.3773834932125143	1.3608442740064008	0.152929277451583
WDI_agriculture	Quantile-SFA	normal	medium	Quantile-SFA	0.1261742369989083	0.3733278974745688	1.3594741826158274	0.1522744504515677
WDI_agriculture	Quantile-SFA	skewed	large	HQER-SFA	0.1023735294586499	0.4101340968214009	1.327035461871847	0.153383971750801
WDI_agriculture	Quantile-SFA	skewed	large	Quantile-SFA	0.103673636550589	0.4032961286122964	1.3288810957857782	0.1524751585632994
WDI_agriculture	Quantile-SFA	skewed	medium	HQER-SFA	0.1072275721589588	0.3730899861555268	1.3042352894469325	0.1596525102958832
WDI_agriculture	Quantile-SFA	skewed	medium	Quantile-SFA	0.1086115762992945	0.3648972247229424	1.3074750011621674	0.1565983360607266
WDI_agriculture	Quantile-SFA	student_t	large	HQER-SFA	0.1178233334082715	0.4456377522662768	1.3413230770141715	0.1580287057836974
WDI_agriculture	Quantile-SFA	student_t	large	Quantile-SFA	0.1181220096519154	0.4366132604818882	1.3416544385263094	0.154645158770284
WDI_agriculture	Quantile-SFA	student_t	medium	HQER-SFA	0.1210770468740193	0.432872473774332	1.3188100315846651	0.1668975143662166
WDI_agriculture	Quantile-SFA	student_t	medium	Quantile-SFA	0.1221404806368129	0.4302462338974561	1.320870287237046	0.16626113191963
WDI_industry	HQER-SFA	ald	large	HQER-SFA	0.0355304299893498	1.7529239830569894e-05	0.5391494074925663	0.0306957588678731
WDI_industry	HQER-SFA	ald	large	Quantile-SFA	0.0036330037772499	1.0980211452172015e-05	0.5389747813941739	0.0306957589373749
WDI_industry	HQER-SFA	ald	medium	HQER-SFA	0.0336793236679128	1.8864634292741395e-05	0.5359555920406889	0.0307271923989861
WDI_industry	HQER-SFA	ald	medium	Quantile-SFA	0.0067248796408069	1.644110830932582e-05	0.5357482673998641	0.0307271914068278
WDI_industry	HQER-SFA	hqer_induced	large	HQER-SFA	0.0039128183018652	1.4563623899345995e-05	0.4243695201726231	0.030669765727135

dataset__source	pseudo__model__source	error__scenario	sample__size__label	fitted__model	mean__sigma__v__error	mean__sigma__u__error	mean__y__rmse	mean__te__rmse
WDI__industry	HQER-SFA	hqer__induced	large	Quantile-SFA	0.0313988393877624	1.0295973483585012e-05	0.4243648844036028	0.0306697664690208
WDI__industry	HQER-SFA	hqer__induced	medium	HQER-SFA	0.0081183826347044	2.07557667263684e-05	0.4236349012597112	0.0306747143395567
WDI__industry	HQER-SFA	hqer__induced	medium	Quantile-SFA	0.032273917659256	1.5970701175890717e-05	0.4236689051661927	0.0306747152685207
WDI__industry	HQER-SFA	normal	large	HQER-SFA	0.0206156684613803	5.493844068912006e-05	0.1965791768656923	0.0306987241889614
WDI__industry	HQER-SFA	normal	large	Quantile-SFA	0.1113305055996872	1.2126763781400404e-05	0.1962875457992303	0.0306987489328461
WDI__industry	HQER-SFA	normal	medium	HQER-SFA	0.0473680011831092	7.181211867395621e-05	0.1973555554957048	0.0307353086069449
WDI__industry	HQER-SFA	normal	medium	Quantile-SFA	0.1122191811562127	2.4486244835529948e-05	0.1967347639025351	0.0307353275319937
WDI__industry	HQER-SFA	skewed	large	HQER-SFA	0.1273037148668821	0.0001004024534707	0.2201933357871945	0.0306570622179176
WDI__industry	HQER-SFA	skewed	large	Quantile-SFA	0.1349449666066797	2.765710213401929e-05	0.2105364900589177	0.0306570913297163
WDI__industry	HQER-SFA	skewed	medium	HQER-SFA	0.1285307502701795	9.213310501682848e-05	0.2200659661460394	0.0306594323871328
WDI__industry	HQER-SFA	skewed	medium	Quantile-SFA	0.1346401669606008	4.352184844528154e-05	0.2093685764136599	0.0306594507896775
WDI__industry	HQER-SFA	student__t	large	HQER-SFA	0.1186708768656196	9.643202475445811e-05	0.1932777949390679	0.0306906296454903
WDI__industry	HQER-SFA	student__t	large	Quantile-SFA	0.1257009056123147	1.4851985628379546e-05	0.1931329993368259	0.0306906235452998
WDI__industry	HQER-SFA	student__t	medium	HQER-SFA	0.1172340293723893	8.055032886303424e-05	0.1923862663364988	0.0306612378565409
WDI__industry	HQER-SFA	student__t	medium	Quantile-SFA	0.1263162782648561	2.72764534059472e-05	0.1920184041579588	0.0306612311393952
WDI__industry	Quantile-SFA	ald	large	HQER-SFA	0.0028326482183993	7.288626810110915e-06	0.4416680622265654	0.0307256746412749
WDI__industry	Quantile-SFA	ald	large	Quantile-SFA	0.0027451796400071	7.758005565981265e-06	0.4416666235386363	0.0307256745192584
WDI__industry	Quantile-SFA	ald	medium	HQER-SFA	0.0062278142062411	1.3899263403057378e-05	0.4409882771316303	0.0305996291125024
WDI__industry	Quantile-SFA	ald	medium	Quantile-SFA	0.0062488038082514	1.3474510853942353e-05	0.4409838515465127	0.0305996294259022
WDI__industry	Quantile-SFA	hqer__induced	large	HQER-SFA	0.0047421140732622	6.203216340831425e-06	0.4123509502558017	0.0306973593458413
WDI__industry	Quantile-SFA	hqer__induced	large	Quantile-SFA	0.0050031544433387	6.499250021035283e-06	0.412353738316858	0.0306973593458512
WDI__industry	Quantile-SFA	hqer__induced	medium	HQER-SFA	0.0061713685207688	1.2695141041942058e-05	0.4135088975388806	0.030695963376266
WDI__industry	Quantile-SFA	hqer__induced	medium	Quantile-SFA	0.0063415095065829	1.3588607126610748e-05	0.4135350199656293	0.0306959635863303
WDI__industry	Quantile-SFA	normal	large	HQER-SFA	0.0915473409864276	1.37325655980382e-05	0.1661024663613569	0.030671181551788
WDI__industry	Quantile-SFA	normal	large	Quantile-SFA	0.0916557704094762	1.45370817493843e-05	0.1661275670537755	0.0306711816392157
WDI__industry	Quantile-SFA	normal	medium	HQER-SFA	0.0921168927000859	2.994662932988044e-05	0.1659965429686067	0.0307755202489669
WDI__industry	Quantile-SFA	normal	medium	Quantile-SFA	0.0922317822084463	2.7489571368513968e-05	0.1659771819333304	0.0307755203985067
WDI__industry	Quantile-SFA	skewed	large	HQER-SFA	0.1102507394903923	3.4473549261395284e-05	0.1700224849165825	0.0306741729937784
WDI__industry	Quantile-SFA	skewed	large	Quantile-SFA	0.1101830681796854	2.9535442051183057e-05	0.1698170436411656	0.0306741752151752
WDI__industry	Quantile-SFA	skewed	medium	HQER-SFA	0.1099346880310067	4.367232114613541e-05	0.1699412496922061	0.0306744936183427
WDI__industry	Quantile-SFA	skewed	medium	Quantile-SFA	0.1098742971050291	5.187334363559952e-05	0.1697235834627495	0.0306744949176937
WDI__industry	Quantile-SFA	student__t	large	HQER-SFA	0.1032615574204352	1.3268327927414489e-05	0.1640619304791448	0.0306612910509949
WDI__industry	Quantile-SFA	student__t	large	Quantile-SFA	0.1034021649473232	1.4635615272769765e-05	0.1640569109937591	0.0306612907451147
WDI__industry	Quantile-SFA	student__t	medium	HQER-SFA	0.103825720370638	2.750402670166344e-05	0.1636828164752427	0.0306692451492832
WDI__industry	Quantile-SFA	student__t	medium	Quantile-SFA	0.1039652024327408	2.7211755405428677e-05	0.1636880843039073	0.0306692448380668
front41Data	HQER-SFA	ald	medium	HQER-SFA	0.0516321037148851	2.401869152017588e-05	1.1962840577289688	0.0307544602359461
front41Data	HQER-SFA	ald	medium	Quantile-SFA	0.0089233020767034	0.0005285076524528	1.1590950289094075	0.0310438333701238
front41Data	HQER-SFA	ald	small	HQER-SFA	0.0832539842633399	4.8517171951144577e-05	1.1777765726757177	0.0304349979751516
front41Data	HQER-SFA	ald	small	Quantile-SFA	0.0283281670612588	3.574206230857496e-05	1.152017362063172	0.0304349987731029
front41Data	HQER-SFA	hqer__induced	medium	HQER-SFA	0.0125950910208849	2.534049110007944e-05	0.4125557492934816	0.030696911261226
front41Data	HQER-SFA	hqer__induced	medium	Quantile-SFA	0.0818174857297801	2.5361441563936165e-05	0.4038003095614502	0.0306969250950265
front41Data	HQER-SFA	hqer__induced	small	HQER-SFA	0.0421245251720334	7.063445703961952e-05	0.4118291036136132	0.0306428069436348
front41Data	HQER-SFA	hqer__induced	small	Quantile-SFA	0.0922988143380638	0.0001116711505042	0.406733952056282	0.0306428248286827
front41Data	HQER-SFA	normal	medium	HQER-SFA	0.029116205747932	4.262511600942048e-05	0.2020977809819449	0.0306263487896344
front41Data	HQER-SFA	normal	medium	Quantile-SFA	0.115680871448882	3.146692317267091e-05	0.2042819007665482	0.0306263741608495
front41Data	HQER-SFA	normal	small	HQER-SFA	0.0604857589948488	7.457588173206538e-05	0.2158704249847074	0.0305728727953108
front41Data	HQER-SFA	normal	small	Quantile-SFA	0.1241447450424702	0.0001409712531783	0.2226075421877095	0.0305729114725327
front41Data	HQER-SFA	skewed	medium	HQER-SFA	0.0989387711337041	5.374460575150831e-05	0.2241948393836869	0.0307284859525467
front41Data	HQER-SFA	skewed	medium	Quantile-SFA	0.1382425871548133	5.184953165956472e-05	0.2154938017731858	0.0307285026561254
front41Data	HQER-SFA	skewed	small	HQER-SFA	0.0991251637415411	0.0001248608268169	0.2218155782126584	0.0305821588506638
front41Data	HQER-SFA	skewed	small	Quantile-SFA	0.1355703794147698	0.000692129598146	0.214715273364586	0.0309505899471404
front41Data	HQER-SFA	student__t	medium	HQER-SFA	0.1239257582893479	0.0001439326375602	0.1971952305941	0.0307947181823473
front41Data	HQER-SFA	student__t	medium	Quantile-SFA	0.1299870640936671	3.113551709089211e-05	0.1972322631001394	0.0307947147977442
front41Data	HQER-SFA	student__t	small	HQER-SFA	0.110720757720144	0.0002584329597922	0.2053989482104172	0.0306347215268199
front41Data	HQER-SFA	student__t	small	Quantile-SFA	0.135488969808002	0.0001440554960402	0.2054483067073611	0.0306347053167912
front41Data	Quantile-SFA	ald	medium	HQER-SFA	0.005384644777443	0.0009060664116445	0.4607131088982952	0.0305245880801124
front41Data	Quantile-SFA	ald	medium	Quantile-SFA	0.0052654657158694	0.0009057721119927	0.4607567991159174	0.0305245881790816
front41Data	Quantile-SFA	ald	small	HQER-SFA	0.0155083149250362	0.0008435709981014	0.4503727391716438	0.0302032843121253
front41Data	Quantile-SFA	ald	small	Quantile-SFA	0.01532711625091	0.0008949596806449	0.4502210999087092	0.0302032866978176
front41Data	Quantile-SFA	hqer__induced	medium	HQER-SFA	0.0065568164674751	0.0009098690152447	0.3652550935712997	0.0304445604782449
front41Data	Quantile-SFA	hqer__induced	medium	Quantile-SFA	0.0066154264741283	0.0009065588853333	0.3651798700805301	0.0304445602112292
front41Data	Quantile-SFA	hqer__induced	small	HQER-SFA	0.0142808066525819	0.0008989044833685	0.366425189932716	0.0304648279686281
front41Data	Quantile-SFA	hqer__induced	small	Quantile-SFA	0.0142554151360686	0.0008853522675429	0.3659612571936232	0.0304648293322806
front41Data	Quantile-SFA	normal	medium	HQER-SFA	0.0638297500247906	0.0009057823631339	0.1261439631731514	0.0304796793835874
front41Data	Quantile-SFA	normal	medium	Quantile-SFA	0.0639396226939317	0.0009027633765528	0.1264007969995651	0.0304796793180467
front41Data	Quantile-SFA	normal	small	HQER-SFA	0.0674273154930535	0.0009237822350321	0.1326492269537738	0.0305183715020994
front41Data	Quantile-SFA	normal	small	Quantile-SFA	0.0674261146781203	0.0009443233091911	0.1324461724763974	0.0305183754610457
front41Data	Quantile-SFA	skewed	medium	HQER-SFA	0.073867461800808	0.000912016930313	0.1143780257816439	0.0304936916977765
front41Data	Quantile-SFA	skewed	medium	Quantile-SFA	0.0739298169481394	0.0009057332664097	0.1143821743152109	0.03049369150648
front41Data	Quantile-SFA	skewed	small	HQER-SFA	0.0719858403410744	0.0009697206209321	0.1188877800804228	0.0303745119649542
front41Data	Quantile-SFA	skewed	small	Quantile-SFA	0.0722981134768986	0.0009070025714991	0.1190994241759239	0.0303745174494635
front41Data	Quantile-SFA	student__t	medium	HQER-SFA	0.0714901946914688	0.000906141178919	0.122435717105367	0.0304229252495117
front41Data	Quantile-SFA	student__t	medium	Quantile-SFA	0.0715816955262928	0.0009091441916843	0.1225008967444721	0.0304229249383949
front41Data	Quantile-SFA	student__t	small	HQER-SFA	0.0740644921786257	0.0009715820113612	0.1244621236649414	0.0304807581178851
front41Data	Quantile-SFA	student__t	small	Quantile-SFA	0.0741533486857563	0.0009346488843359	0.1240157834733608	0.0304807622255245

Panel 3 of 3

Columns shown: `dataset_source, pseudo_model_source, error_scenario, sample_size_label, fitted_model, mean_te_rank_corr, convergence_rate, mean_elapsed_time_seconds`.

dataset__source	pseudo__model__source	error__scenario	sample__size__label	fitted__model	mean__te__rank__corr	convergence__rate	mean__elapsed__time__seconds
WDI__agriculture	HQER-SFA	ald	large	HQER-SFA	0.0437801260000183	1.0	13.937070908001818
WDI__agriculture	HQER-SFA	ald	large	Quantile-SFA	0.0470595770477732	1.0	5.39375686866338
WDI__agriculture	HQER-SFA	ald	medium	HQER-SFA	0.0479358376700255	1.0	6.018344402666553
WDI__agriculture	HQER-SFA	ald	medium	Quantile-SFA	0.0478571636545778	1.0	1.088933300000887
WDI__agriculture	HQER-SFA	hqer__induced	large	HQER-SFA	0.1099016146704036	1.0	5.231172498331968
WDI__agriculture	HQER-SFA	hqer__induced	large	Quantile-SFA	0.1089384813475653	1.0	5.93224178066807
WDI__agriculture	HQER-SFA	hqer__induced	medium	HQER-SFA	0.1104916768467073	1.0	2.3619808069998784
WDI__agriculture	HQER-SFA	hqer__induced	medium	Quantile-SFA	0.1096583715743119	1.0	1.1830220443367336
WDI__agriculture	HQER-SFA	normal	large	HQER-SFA	0.1219306857876714	1.0	5.481616380000681

dataset_source	pseudo_model_source	error_scenario	sample_size_label	fitted_model	mean_te_rank_corr	convergence_rate	mean_elapsed_time_seconds
WDI_agriculture	HQER-SFA	normal	large	Quantile-SFA	0.1198533337987739	1.0	4.478606297665392
WDI_agriculture	HQER-SFA	normal	medium	HQER-SFA	0.1209058557834231	1.0	2.502660770663545
WDI_agriculture	HQER-SFA	normal	medium	Quantile-SFA	0.1182715317426397	1.0	1.118364178998842
WDI_agriculture	HQER-SFA	skewed	large	HQER-SFA	0.1310697332324333	1.0	11.610706107665708
WDI_agriculture	HQER-SFA	skewed	large	Quantile-SFA	0.1258910958659626	1.0	7.047369217332285
WDI_agriculture	HQER-SFA	skewed	medium	HQER-SFA	0.1318907922031688	1.0	4.941650167331682
WDI_agriculture	HQER-SFA	skewed	medium	Quantile-SFA	0.1254172089634924	1.0	1.456891075333988
WDI_agriculture	HQER-SFA	student_t	large	HQER-SFA	0.1678036210315669	1.0	15.915218137660997
WDI_agriculture	HQER-SFA	student_t	large	Quantile-SFA	0.1653635062020647	1.0	4.111694735000201
WDI_agriculture	HQER-SFA	student_t	medium	HQER-SFA	0.1700132102928411	1.0	7.306078237668262
WDI_agriculture	HQER-SFA	student_t	medium	Quantile-SFA	0.1662072687532141	1.0	1.1441479549987708
WDI_agriculture	Quantile-SFA	ald	large	HQER-SFA	0.7091227629106907	1.0	7.776904131665457
WDI_agriculture	Quantile-SFA	ald	large	Quantile-SFA	0.7094526806030693	1.0	5.131103122667312
WDI_agriculture	Quantile-SFA	ald	medium	HQER-SFA	0.7079396666386666	1.0	4.467450002993574
WDI_agriculture	Quantile-SFA	ald	medium	Quantile-SFA	0.707157809615144	1.0	1.1948140083386292
WDI_agriculture	Quantile-SFA	hqer_induced	large	HQER-SFA	0.7181303470575867	1.0	7.503391821001812
WDI_agriculture	Quantile-SFA	hqer_induced	large	Quantile-SFA	0.7179863121065915	1.0	4.868237898669613
WDI_agriculture	Quantile-SFA	hqer_induced	medium	HQER-SFA	0.7182240444161776	1.0	4.385747808666007
WDI_agriculture	Quantile-SFA	hqer_induced	medium	Quantile-SFA	0.7174081134456499	1.0	1.2195043006670312
WDI_agriculture	Quantile-SFA	normal	large	HQER-SFA	0.926763487425872	1.0	10.81282373166711
WDI_agriculture	Quantile-SFA	normal	large	Quantile-SFA	0.9266776957739482	1.0	7.263664760662262
WDI_agriculture	Quantile-SFA	normal	medium	HQER-SFA	0.924578385993544	1.0	5.7390812729998535
WDI_agriculture	Quantile-SFA	normal	medium	Quantile-SFA	0.9227117567923864	1.0	1.5106453889989644
WDI_agriculture	Quantile-SFA	skewed	large	HQER-SFA	0.928150995982749	1.0	10.492891741667894
WDI_agriculture	Quantile-SFA	skewed	large	Quantile-SFA	0.9266470089146224	1.0	6.971597265668291
WDI_agriculture	Quantile-SFA	skewed	medium	HQER-SFA	0.9265487644350576	1.0	5.309009310665812
WDI_agriculture	Quantile-SFA	skewed	medium	Quantile-SFA	0.9257291951541256	1.0	1.4625657356643933
WDI_agriculture	Quantile-SFA	student_t	large	HQER-SFA	0.9414991487597872	1.0	10.746256448000883
WDI_agriculture	Quantile-SFA	student_t	large	Quantile-SFA	0.9413472635244056	1.0	7.3120103956630915
WDI_agriculture	Quantile-SFA	student_t	medium	HQER-SFA	0.9398253807415228	1.0	5.675510672665648
WDI_agriculture	Quantile-SFA	student_t	medium	Quantile-SFA	0.9386693854499656	1.0	1.525625447332471
WDI_industry	HQER-SFA	ald	large	HQER-SFA	0.0647236504009125	1.0	7.993335680670376
WDI_industry	HQER-SFA	ald	large	Quantile-SFA	0.0685065003885426	1.0	4.034991075998308
WDI_industry	HQER-SFA	ald	medium	HQER-SFA	0.0648431406525626	1.0	4.038546926667526
WDI_industry	HQER-SFA	ald	medium	Quantile-SFA	0.0689980657580242	1.0	1.0203059176680591
WDI_industry	HQER-SFA	hqer_induced	large	HQER-SFA	0.0750287216771804	1.0	8.161959278666473
WDI_industry	HQER-SFA	hqer_induced	large	Quantile-SFA	0.0782156794077238	1.0	5.039045540333318
WDI_industry	HQER-SFA	hqer_induced	medium	HQER-SFA	0.0797000301601206	1.0	3.566476004664534
WDI_industry	HQER-SFA	hqer_induced	medium	Quantile-SFA	0.0819739588588278	1.0	1.0516344186660718
WDI_industry	HQER-SFA	normal	large	HQER-SFA	0.1506299931174982	1.0	12.912647906996911
WDI_industry	HQER-SFA	normal	large	Quantile-SFA	0.1365663457212911	1.0	4.957846337336038
WDI_industry	HQER-SFA	normal	medium	HQER-SFA	0.1463827471309885	1.0	5.498097925670639
WDI_industry	HQER-SFA	normal	medium	Quantile-SFA	0.1341093832704343	1.0	1.2611795619988695
WDI_industry	HQER-SFA	skewed	large	HQER-SFA	0.1636564144841036	1.0	9.941550814664806
WDI_industry	HQER-SFA	skewed	large	Quantile-SFA	0.1705003402741592	1.0	5.98212023099768
WDI_industry	HQER-SFA	skewed	medium	HQER-SFA	0.1609060989843959	1.0	5.079819049998187
WDI_industry	HQER-SFA	skewed	medium	Quantile-SFA	0.1672268296413714	1.0	1.512556478670837
WDI_industry	HQER-SFA	student_t	large	HQER-SFA	0.2075275738275615	1.0	13.077210197335614
WDI_industry	HQER-SFA	student_t	large	Quantile-SFA	0.2021786343851992	1.0	5.210488960334236
WDI_industry	HQER-SFA	student_t	medium	HQER-SFA	0.2052578104712418	1.0	5.946168444663829
WDI_industry	HQER-SFA	student_t	medium	Quantile-SFA	0.199181786937789	1.0	1.257372242002554
WDI_industry	Quantile-SFA	ald	large	HQER-SFA	0.0811706306303197	1.0	6.713786354665451
WDI_industry	Quantile-SFA	ald	large	Quantile-SFA	0.0863261969712107	1.0	4.758823932666662
WDI_industry	Quantile-SFA	ald	medium	HQER-SFA	0.0842855606756518	1.0	3.5953792239977824
WDI_industry	Quantile-SFA	ald	medium	Quantile-SFA	0.086860782792532	1.0	1.081300991664854
WDI_industry	Quantile-SFA	hqer_induced	large	HQER-SFA	0.0823271670718672	1.0	6.817873918998909
WDI_industry	Quantile-SFA	hqer_induced	large	Quantile-SFA	0.0851710997411938	1.0	4.848432224666176
WDI_industry	Quantile-SFA	hqer_induced	medium	HQER-SFA	0.0834738916759456	1.0	3.4075628236688984
WDI_industry	Quantile-SFA	hqer_induced	medium	Quantile-SFA	0.0861466195239566	1.0	1.0334845326679836
WDI_industry	Quantile-SFA	normal	large	HQER-SFA	0.1807403389989327	1.0	7.120797629999676
WDI_industry	Quantile-SFA	normal	large	Quantile-SFA	0.1659880089747629	1.0	4.868381893337646
WDI_industry	Quantile-SFA	normal	medium	HQER-SFA	0.1855459876373669	1.0	4.303320579999321
WDI_industry	Quantile-SFA	normal	medium	Quantile-SFA	0.169799821431218	1.0	1.2923113756652067
WDI_industry	Quantile-SFA	skewed	large	HQER-SFA	0.1922706609877561	1.0	9.960796058665435
WDI_industry	Quantile-SFA	skewed	large	Quantile-SFA	0.1995989533369235	1.0	6.973208991333182
WDI_industry	Quantile-SFA	skewed	medium	HQER-SFA	0.1942763390414763	1.0	4.827391095667263
WDI_industry	Quantile-SFA	skewed	medium	Quantile-SFA	0.2011374174156861	1.0	1.4744258219994175
WDI_industry	Quantile-SFA	student_t	large	HQER-SFA	0.2474230797498105	1.0	8.306694330332878
WDI_industry	Quantile-SFA	student_t	large	Quantile-SFA	0.2406916635880925	1.0	5.945634554000377
WDI_industry	Quantile-SFA	student_t	medium	HQER-SFA	0.2432626361390131	1.0	4.322758982333374
WDI_industry	Quantile-SFA	student_t	medium	Quantile-SFA	0.238707680295992	1.0	1.2914054416657503
front41Data	HQER-SFA	ald	medium	HQER-SFA	0.0418126894907579	1.0	5.119786884336015
front41Data	HQER-SFA	ald	medium	Quantile-SFA	0.0493544616835329	1.0	1.170060947665964
front41Data	HQER-SFA	ald	small	HQER-SFA	0.0357460405668241	1.0	4.471103843665914
front41Data	HQER-SFA	ald	small	Quantile-SFA	0.0412378680852909	1.0	0.5076285589987917
front41Data	HQER-SFA	hqer_induced	medium	HQER-SFA	0.0906704782019127	1.0	3.279419796670651
front41Data	HQER-SFA	hqer_induced	medium	Quantile-SFA	0.0840798558645625	1.0	1.4735240073358484
front41Data	HQER-SFA	hqer_induced	small	HQER-SFA	0.0771208669074743	1.0	4.076417586662574
front41Data	HQER-SFA	hqer_induced	small	Quantile-SFA	0.0740424896932648	1.0	0.6219015436670937
front41Data	HQER-SFA	normal	medium	HQER-SFA	0.1474089656358625	1.0	4.493641657662714
front41Data	HQER-SFA	normal	medium	Quantile-SFA	0.1335991420553798	1.0	1.6897297313370898
front41Data	HQER-SFA	normal	small	HQER-SFA	0.1496897286283227	1.0	3.653253593666207
front41Data	HQER-SFA	normal	small	Quantile-SFA	0.1340893070516605	1.0	0.611458055995366
front41Data	HQER-SFA	skewed	medium	HQER-SFA	0.1554280338721354	1.0	4.431361523000329
front41Data	HQER-SFA	skewed	medium	Quantile-SFA	0.1625169594221729	1.0	1.6784096046657455
front41Data	HQER-SFA	skewed	small	HQER-SFA	0.1501409650828934	1.0	4.299031099996452
front41Data	HQER-SFA	skewed	small	Quantile-SFA	0.1441431358991455	1.0	0.7197935440008102
front41Data	HQER-SFA	student_t	medium	HQER-SFA	0.2010992192931091	1.0	7.007959891001228
front41Data	HQER-SFA	student_t	medium	Quantile-SFA	0.1949805505076519	1.0	1.5766617333345736
front41Data	HQER-SFA	student_t	small	HQER-SFA	0.1828602389552653	1.0	4.615613206333268
front41Data	HQER-SFA	student_t	small	Quantile-SFA	0.1766307202057081	1.0	0.5889782976623004
front41Data	Quantile-SFA	ald	medium	HQER-SFA	0.1015706392528147	1.0	4.048710526668389
front41Data	Quantile-SFA	ald	medium	Quantile-SFA	0.1034067681395296	1.0	1.1650128066704686
front41Data	Quantile-SFA	ald	small	HQER-SFA	0.0756791650005749	1.0	3.9240938926655033
front41Data	Quantile-SFA	ald	small	Quantile-SFA	0.0730672206703997	1.0	0.4451472713324862
front41Data	Quantile-SFA	hqer_induced	medium	HQER-SFA	0.0987099858824113	1.0	5.587932566666374
front41Data	Quantile-SFA	hqer_induced	medium	Quantile-SFA	0.0960371003100244	1.0	1.5892092220060294
front41Data	Quantile-SFA	hqer_induced	small	HQER-SFA	0.101739184754925	1.0	4.469511207332446
front41Data	Quantile-SFA	hqer_induced	small	Quantile-SFA	0.1013727280115743	1.0	0.4989445143347257
front41Data	Quantile-SFA	normal	medium	HQER-SFA	0.242337204629627	1.0	3.8677173403307097
front41Data	Quantile-SFA	normal	medium	Quantile-SFA	0.2265044085610306	1.0	1.1561760443328724
front41Data	Quantile-SFA	normal	small	HQER-SFA	0.2447173440442798	1.0	3.645555143998063
front41Data	Quantile-SFA	normal	small	Quantile-SFA	0.2323316402787992	1.0	0.4329463643301278

dataset_source	pseudo_model_source	error_scenario	sample_size_label	fitted_model	mean_te_rank_corr	convergence_rate	mean_elapsed_time_seconds
front41Data	Quantile-SFA	skewed	medium	HQER-SFA	0.2619493510526933	1.0	4.886770000996282
front41Data	Quantile-SFA	skewed	medium	Quantile-SFA	0.2706938092202548	1.0	1.4331609703372428
front41Data	Quantile-SFA	skewed	small	HQER-SFA	0.2490623321292952	1.0	4.341517218331864
front41Data	Quantile-SFA	skewed	small	Quantile-SFA	0.2468142400370734	1.0	0.5023138693355335
front41Data	Quantile-SFA	student_t	medium	HQER-SFA	0.3311008782610689	1.0	3.60190840866766
front41Data	Quantile-SFA	student_t	medium	Quantile-SFA	0.3229205728041439	1.0	1.070508889335809
front41Data	Quantile-SFA	student_t	small	HQER-SFA	0.3207766356861131	1.0	4.059931438003939
front41Data	Quantile-SFA	student_t	small	Quantile-SFA	0.3119033122008671	1.0	0.4712922396687403

S6 R = 300 win rates by design cell

Source CSV: `tables/s6_r300_win_rates_by_design_cell.csv`. Rows displayed: 60; columns displayed: 9.

dataset_source	pseudo_model_source	error_scenario	sample_size_label	beta_error_win_rate	y_rmse_win_rate	te_rmse_win_rate	te_rank_corr_win_rate	n_pairs
WDI_agriculture	HQER-SFA	ald	large	0.2766666666666666	0.33	0.23	0.39	300
WDI_agriculture	HQER-SFA	ald	medium	0.27	0.3033333333333333	0.2066666666666666	0.4766666666666667	300
WDI_agriculture	HQER-SFA	hqer_induced	large	1.0	1.0	1.0	0.72	300
WDI_agriculture	HQER-SFA	hqer_induced	medium	0.99	1.0	0.9966666666666668	0.6166666666666667	300
WDI_agriculture	HQER-SFA	normal	large	1.0	1.0	1.0	0.7266666666666667	300
WDI_agriculture	HQER-SFA	normal	medium	0.9633333333333334	0.9966666666666668	1.0	0.66	300
WDI_agriculture	HQER-SFA	skewed	large	0.02	0.02	0.9533333333333334	0.6166666666666667	300
WDI_agriculture	HQER-SFA	skewed	medium	0.0666666666666666	0.0666666666666666	0.8633333333333333	0.6133333333333333	300
WDI_agriculture	HQER-SFA	student_t	large	0.53	0.4866666666666667	0.6433333333333333	0.5966666666666667	300
WDI_agriculture	HQER-SFA	student_t	medium	0.5366666666666666	0.5066666666666667	0.6133333333333333	0.56	300
WDI_agriculture	Quantile-SFA	ald	large	0.4966666666666666	0.53	0.5433333333333333	0.39	300
WDI_agriculture	Quantile-SFA	ald	medium	0.5633333333333334	0.5166666666666667	0.49	0.4766666666666667	300
WDI_agriculture	Quantile-SFA	hqer_induced	large	0.5233333333333333	0.5533333333333333	0.5166666666666667	0.56	300
WDI_agriculture	Quantile-SFA	hqer_induced	medium	0.5566666666666666	0.5233333333333333	0.5666666666666667	0.5866666666666667	300
WDI_agriculture	Quantile-SFA	normal	large	0.4866666666666667	0.4633333333333333	0.4833333333333333	0.7033333333333334	300
WDI_agriculture	Quantile-SFA	normal	medium	0.4333333333333333	0.4633333333333333	0.44	0.5833333333333334	300
WDI_agriculture	Quantile-SFA	skewed	large	0.5466666666666666	0.51	0.4266666666666667	0.72	300
WDI_agriculture	Quantile-SFA	skewed	medium	0.5166666666666667	0.5166666666666667	0.4633333333333333	0.64	300
WDI_agriculture	Quantile-SFA	student_t	large	0.49	0.5066666666666667	0.4066666666666667	0.2466666666666666	300
WDI_agriculture	Quantile-SFA	student_t	medium	0.5233333333333333	0.5233333333333333	0.4733333333333333	0.48	300
WDI_industry	HQER-SFA	ald	large	0.49	0.2433333333333333	0.5233333333333333	0.3266666666666666	300
WDI_industry	HQER-SFA	ald	medium	0.55	0.4533333333333333	0.4266666666666667	0.41	300
WDI_industry	HQER-SFA	hqer_induced	large	0.69	0.5766666666666667	0.6333333333333333	0.3766666666666666	300
WDI_industry	HQER-SFA	hqer_induced	medium	0.5366666666666666	0.6566666666666666	0.57	0.46	300
WDI_industry	HQER-SFA	normal	large	0.5866666666666667	0.4033333333333333	0.9233333333333332	0.9533333333333334	300
WDI_industry	HQER-SFA	normal	medium	0.5766666666666667	0.47	0.75	0.7533333333333333	300
WDI_industry	HQER-SFA	skewed	large	0.0133333333333333	0.0133333333333333	0.7733333333333333	0.3133333333333333	300
WDI_industry	HQER-SFA	skewed	medium	0.01	0.0133333333333333	0.5266666666666666	0.42	300
WDI_industry	HQER-SFA	student_t	large	0.4533333333333333	0.4366666666666666	0.2466666666666666	0.7333333333333333	300
WDI_industry	HQER-SFA	student_t	medium	0.3966666666666666	0.4233333333333333	0.3466666666666667	0.6333333333333333	300
WDI_industry	Quantile-SFA	ald	large	0.4966666666666666	0.4733333333333333	0.4966666666666666	0.3	300
WDI_industry	Quantile-SFA	ald	medium	0.5	0.49	0.5533333333333333	0.46	300
WDI_industry	Quantile-SFA	hqer_induced	large	0.55	0.5966666666666667	0.5166666666666667	0.3966666666666666	300
WDI_industry	Quantile-SFA	hqer_induced	medium	0.53	0.55	0.5	0.4233333333333333	300
WDI_industry	Quantile-SFA	normal	large	0.48	0.5066666666666667	0.5133333333333333	0.9666666666666668	300
WDI_industry	Quantile-SFA	normal	medium	0.4866666666666667	0.4933333333333333	0.5166666666666667	0.84	300
WDI_industry	Quantile-SFA	skewed	large	0.3666666666666666	0.3633333333333333	0.5633333333333334	0.31	300
WDI_industry	Quantile-SFA	skewed	medium	0.39	0.3766666666666666	0.5133333333333333	0.42	300
WDI_industry	Quantile-SFA	student_t	large	0.48	0.4933333333333333	0.2433333333333333	0.7933333333333333	300
WDI_industry	Quantile-SFA	student_t	medium	0.45	0.47	0.4033333333333333	0.6333333333333333	300
front41Data	HQER-SFA	ald	medium	0.1033333333333333	0.06	0.6033333333333334	0.34	300
front41Data	HQER-SFA	ald	small	0.3233333333333333	0.32	0.51	0.4833333333333333	300
front41Data	HQER-SFA	hqer_induced	medium	0.66	0.13	0.8866666666666667	0.6366666666666667	300
front41Data	HQER-SFA	hqer_induced	small	0.45	0.4033333333333333	0.6566666666666666	0.5633333333333334	300
front41Data	HQER-SFA	normal	medium	0.7233333333333334	0.6533333333333333	0.81	0.82	300
front41Data	HQER-SFA	normal	small	0.58	0.6633333333333333	0.6833333333333333	0.6233333333333333	300
front41Data	HQER-SFA	skewed	medium	0.0833333333333333	0.0833333333333333	0.5066666666666667	0.39	300
front41Data	HQER-SFA	skewed	small	0.22	0.3033333333333333	0.48	0.5266666666666666	300
front41Data	HQER-SFA	student_t	medium	0.46	0.4966666666666666	0.3933333333333333	0.6366666666666667	300
front41Data	HQER-SFA	student_t	small	0.47	0.58	0.47	0.5233333333333333	300
front41Data	Quantile-SFA	ald	medium	0.4566666666666666	0.51	0.53	0.4666666666666667	300
front41Data	Quantile-SFA	ald	small	0.4833333333333333	0.4966666666666666	0.53	0.5233333333333333	300
front41Data	Quantile-SFA	hqer_induced	medium	0.5066666666666667	0.5166666666666667	0.5066666666666667	0.5833333333333334	300
front41Data	Quantile-SFA	hqer_induced	small	0.5066666666666667	0.49	0.4933333333333333	0.5	300
front41Data	Quantile-SFA	normal	medium	0.53	0.54	0.54	0.86	300
front41Data	Quantile-SFA	normal	small	0.5333333333333333	0.5	0.5266666666666666	0.5966666666666667	300
front41Data	Quantile-SFA	skewed	medium	0.45	0.47	0.5133333333333333	0.3766666666666666	300
front41Data	Quantile-SFA	skewed	small	0.4866666666666667	0.4666666666666667	0.47	0.47	300
front41Data	Quantile-SFA	student_t	medium	0.5333333333333333	0.53	0.4333333333333333	0.6866666666666666	300
front41Data	Quantile-SFA	student_t	small	0.4766666666666667	0.48	0.4766666666666667	0.5766666666666667	300

S7 Gamma sensitivity real-data results

Source CSV: `tables/s7_gamma_sensitivity_realdata.csv`. Rows displayed: 15; columns displayed: 14. The table is split into panels to keep all cells inside the page boundary.

Panel 1 of 2

Columns shown: `dataset`, `gamma_setting`, `gamma_value`, `loglik`, `AIC`, `BIC`, `validation_RMSE`, `mean_TE`, `median_TE`.

dataset	gamma_setting	gamma_value	loglik	AIC	BIC	validation_RMSE	mean_TE	median_TE
front41Data	gamma_0	0.0	-5.00881320472594	24.01762640945188	38.67803834500658	0.4195167862143548	0.9505300363644172	0.9505295574211952
front41Data	gamma_0.25	0.25	-3.775603359596047	21.55120671919209	36.21161865474679	0.4371814763103644	0.9505301011285524	0.950529909043184
front41Data	gamma_0.50	0.5	-16.371100710061818	46.74220142012364	61.40261335567834	0.4219351004888945	0.8517111902251474	0.8398718176129418
front41Data	gamma_0.75	0.75	-4.043313911307959	22.08662782261592	36.74703975817062	0.4244191972184034	0.950530151168579	0.950530128137252
front41Data	free_gamma	0.3231480141547307	-3.823837565424783	21.647675130849567	36.30808706640427	0.4372322056016008	0.9505301456039678	0.9505299950969484
WDI_industry	gamma_0	0.0	-1695.1828369735003	3404.3656739470007	3450.711499530231	0.4668834420135443	0.950530141828608	0.9505305052726604
WDI_industry	gamma_0.25	0.25	-1838.663469441896	3691.326938883792	3737.6727644670223	0.4677455909503045	0.9505301581633214	0.9505303613701884
WDI_industry	gamma_0.50	0.5	-1927.2133261295112	3868.4266522590224	3914.772477842253	0.4697482536333948	0.950530155074487	0.9505303147612104
WDI_industry	gamma_0.75	0.75	-1993.8467296177837	4001.693459235567	4048.039284818797	0.4706061795849185	0.9505301512753724	0.950530287241618
WDI_industry	free_gamma	0.0390052714090783	-1717.264910646754	3448.529821293508	3494.8756468767383	0.4668811896883829	0.9505301496139272	0.9505304517721544
WDI_agriculture	gamma_0	0.0	-9516.765067812385	19047.53013562477	19095.05669013193	1.535438666351823	0.4137213741952369	0.4016705302135203
WDI_agriculture	gamma_0.25	0.25	-9441.696303957808	18897.39260791561	18944.91916242277	1.257088459352914	0.8492705774711068	0.8434619862243808
WDI_agriculture	gamma_0.50	0.5	-9400.559885526283	18815.119771052567	18862.646325559726	1.2465335648736855	0.8493599326810404	0.8452676149679509
WDI_agriculture	gamma_0.75	0.75	-9391.7797076262	18797.559415252403	18845.08596975956	1.226434519226416	0.8492048684037666	0.8457503885083049
WDI_agriculture	free_gamma	0.7766898931453844	-9387.313136090328	18788.626272180656	18836.152826687816	1.1987322772035045	0.8488998407873726	0.8463129052697776

Panel 2 of 2

Columns shown: `dataset`, `gamma_setting`, `low_efficiency_ratio`, `converged`, `n_iter`, `best_start_id`, `warning_message`.

dataset	gamma_setting	low_efficiency_ratio	converged	n_iter	best_start_id	warning_message
front41Data	gamma_0	0.0	True	33	2	
front41Data	gamma_0.25	0.0	True	26	2	
front41Data	gamma_0.50	0.0	True	15	1	
front41Data	gamma_0.75	0.0	True	9	2	
front41Data	free_gamma	0.0	True	52	2	
WDI_industry	gamma_0	0.0	True	22	2	
WDI_industry	gamma_0.25	0.0	True	21	2	
WDI_industry	gamma_0.50	0.0	True	21	2	
WDI_industry	gamma_0.75	0.0	True	18	2	
WDI_industry	free_gamma	0.0	True	46	2	
WDI_agriculture	gamma_0	0.7547600913937548	True	16	3	
WDI_agriculture	gamma_0.25	0.0	True	14	2	
WDI_agriculture	gamma_0.50	0.0	True	13	2	
WDI_agriculture	gamma_0.75	0.0	True	17	2	
WDI_agriculture	free_gamma	0.0	True	24	2	

S8 Gamma ablation Monte Carlo results

Source CSV: `tables/s8_gamma_ablation_monte_carlo.csv`. Rows displayed: 90; columns displayed: 12. The table is split into panels to keep all cells inside the page boundary.

Panel 1 of 2

Columns shown: `dataset_source`, `pseudo_model_source`, `error_scenario`, `sample_size_label`, `gamma_setting`, `mean_beta_error`, `mean_prediction_RMSE`, `mean_TE_RMSE`, `mean_TE_rank_corr`.

dataset_source	pseudo_model_source	error_scenario	sample_size_label	gamma_setting	mean_beta_error	mean_prediction_RMSE	mean_TE_RMSE	mean_TE_rank_corr
WDI_agriculture	HQER-SFA	hqer_induced	medium	free_gamma	0.1745607511071962	1.1815661319522417	0.0981265892667169	0.1076834099336397
WDI_agriculture	HQER-SFA	hqer_induced	medium	gamma_0	0.5981971454018575	1.509116441338478	0.4992191064895156	0.1064876961643868
WDI_agriculture	HQER-SFA	hqer_induced	medium	gamma_0.25	0.3127005344344045	1.291930899381555	0.1979836892480505	0.1076074985899943
WDI_agriculture	HQER-SFA	hqer_induced	medium	gamma_0.50	0.1714819605989066	1.1733966014974213	0.0993152365981962	0.1075817762471049
WDI_agriculture	HQER-SFA	hqer_induced	medium	gamma_0.75	0.1528340578950685	1.165206526338129	0.0988063539140555	0.1076679193116772
WDI_agriculture	HQER-SFA	normal	medium	free_gamma	0.1585727829227972	0.9376084807373164	0.0981459618005579	0.1340176487105948
WDI_agriculture	HQER-SFA	normal	medium	gamma_0	0.5628518699025293	1.1613729932546315	0.4367989018240781	0.1301820361565535
WDI_agriculture	HQER-SFA	normal	medium	gamma_0.25	0.2187482337341867	0.967395409815917	0.1367859904629668	0.1339427463309853
WDI_agriculture	HQER-SFA	normal	medium	gamma_0.50	0.1635640626234964	0.9388371042527156	0.0985059170024201	0.1340119693278773
WDI_agriculture	HQER-SFA	normal	medium	gamma_0.75	0.1585230907542727	0.9369530608943608	0.0988718070705964	0.1341471513086052
WDI_agriculture	HQER-SFA	skewed	medium	free_gamma	0.8443743520312296	1.1437733185643753	0.1073181446918084	0.1220900291601166
WDI_agriculture	HQER-SFA	skewed	medium	gamma_0	0.5528757258806835	1.0097722246266772	0.1940647875626181	0.1163445180642715
WDI_agriculture	HQER-SFA	skewed	medium	gamma_0.25	0.857279282939343	1.1517784282117016	0.1051103448105047	0.1221192939171756
WDI_agriculture	HQER-SFA	skewed	medium	gamma_0.50	0.8680055270646634	1.159044037760385	0.1155288853648929	0.1221651197004787
WDI_agriculture	HQER-SFA	skewed	medium	gamma_0.75	0.8386823599108146	1.1396700602088108	0.1170463824486525	0.1221713702854811
WDI_agriculture	Quantile-SFA	hqer_induced	medium	free_gamma	0.4764266239873816	1.1946270929340952	0.4700059846218771	0.7194869835479342
WDI_agriculture	Quantile-SFA	hqer_induced	medium	gamma_0	0.1745482133147899	1.461373827234837	0.1967836743163939	0.7185604184704296
WDI_agriculture	Quantile-SFA	hqer_induced	medium	gamma_0.25	0.4561014471970891	1.2126847970464412	0.4552664840039282	0.7194718049272197
WDI_agriculture	Quantile-SFA	hqer_induced	medium	gamma_0.50	0.490995379280648	1.1861497801227596	0.4621685895073735	0.7195498033192131
WDI_agriculture	Quantile-SFA	hqer_induced	medium	gamma_0.75	0.5196278566324593	1.172443994906156	0.4613626697981074	0.7196017827271309
WDI_agriculture	Quantile-SFA	normal	medium	free_gamma	0.1729601767851195	1.2331292644696252	0.350586047606809	0.9244630050520202
WDI_agriculture	Quantile-SFA	normal	medium	gamma_0	0.1582925918208478	1.3639886465419293	0.1519615721054477	0.9236993437972963
WDI_agriculture	Quantile-SFA	normal	medium	gamma_0.25	0.1796174583941562	1.2487800808268736	0.3589192452784944	0.9239529146916589
WDI_agriculture	Quantile-SFA	normal	medium	gamma_0.50	0.1761226713800673	1.242983352854771	0.3287478910415345	0.9244414971259884
WDI_agriculture	Quantile-SFA	normal	medium	gamma_0.75	0.1981127075518345	1.229636147269776	0.320498045728404	0.9242844030576122
WDI_agriculture	Quantile-SFA	skewed	medium	free_gamma	0.2277217152737327	1.1524574248540318	0.4395791185645962	0.92625224004896
WDI_agriculture	Quantile-SFA	skewed	medium	gamma_0	0.1433262423876724	1.3082990618056431	0.1678896304984492	0.92452774996665
WDI_agriculture	Quantile-SFA	skewed	medium	gamma_0.25	0.2130110798558964	1.1626519300745053	0.4321610128594482	0.9261772119088476
WDI_agriculture	Quantile-SFA	skewed	medium	gamma_0.50	0.2227871772991345	1.1611629350580353	0.4146704498366849	0.9262555856623426
WDI_agriculture	Quantile-SFA	skewed	medium	gamma_0.75	0.2145141249875779	1.1822729761953308	0.3373195139935745	0.9261396200784804
WDI_industry	HQER-SFA	hqer_induced	large	free_gamma	0.0291064219173308	0.4256924956547631	0.0307281268584282	0.076615156318789
WDI_industry	HQER-SFA	hqer_induced	large	gamma_0	0.0299128199849462	0.4255985777164726	0.0307281291384318	0.078698924086101
WDI_industry	HQER-SFA	hqer_induced	large	gamma_0.25	0.0286148846908483	0.4255996666624437	0.0307281302293517	0.0766293423773356
WDI_industry	HQER-SFA	hqer_induced	large	gamma_0.50	0.0306046465073115	0.4255917107811482	0.0307281324197074	0.0766210392152598
WDI_industry	HQER-SFA	hqer_induced	large	gamma_0.75	0.0329462707077983	0.42560089266989	0.0307281342591821	0.0766065694016423
WDI_industry	HQER-SFA	normal	large	free_gamma	0.0210530664716344	0.1969563439512343	0.0306889599748299	0.1476651013662753
WDI_industry	HQER-SFA	normal	large	gamma_0	0.0245378762219342	0.1965641782487459	0.0306889865574832	0.1349944700605149
WDI_industry	HQER-SFA	normal	large	gamma_0.25	0.0229343222316274	0.1968519949217608	0.0306889666627199	0.1476927112281778
WDI_industry	HQER-SFA	normal	large	gamma_0.50	0.021176249119091	0.1966957199462402	0.0306889614199476	0.1476752506838126
WDI_industry	HQER-SFA	normal	large	gamma_0.75	0.0199470116289458	0.1966551674473308	0.0306889580785808	0.1476728839082209
WDI_industry	HQER-SFA	skewed	large	free_gamma	0.1498178030524134	0.2184172697341685	0.0307661581335793	0.1622515625578906
WDI_industry	HQER-SFA	skewed	large	gamma_0	0.1319003428864889	0.2099933072679872	0.0307661960602447	0.1708753541497777
WDI_industry	HQER-SFA	skewed	large	gamma_0.25	0.1525349970074598	0.219766920896054	0.0307661721582373	0.1622574898693724
WDI_industry	HQER-SFA	skewed	large	gamma_0.50	0.1469526079652025	0.2169540078925587	0.0307661571339671	0.1622270963217741
WDI_industry	HQER-SFA	skewed	large	gamma_0.75	0.1408119186586158	0.2140400740164365	0.0307661278141157	0.1622092593373148
WDI_industry	Quantile-SFA	hqer_induced	large	free_gamma	0.0288579088438102	0.4113464653327478	0.030719286665118	0.0828769222470648
WDI_industry	Quantile-SFA	hqer_induced	large	gamma_0	0.0294343815125262	0.4113933022498898	0.030719286705387	0.0858564611615673
WDI_industry	Quantile-SFA	hqer_induced	large	gamma_0.25	0.029624279412896	0.4113494100110466	0.0307192895110599	0.082880808215202
WDI_industry	Quantile-SFA	hqer_induced	large	gamma_0.50	0.0316177843589244	0.4114173915523894	0.0307192926881359	0.0828744084336021
WDI_industry	Quantile-SFA	hqer_induced	large	gamma_0.75	0.034206178776766	0.4115345723954046	0.0307192953011058	0.0828524929931232
WDI_industry	Quantile-SFA	normal	large	free_gamma	0.0203399760531139	0.1661321537736572	0.0307515542639046	0.1815504750057738
WDI_industry	Quantile-SFA	normal	large	gamma_0	0.0197031498027698	0.1655095387266566	0.030751585396557	0.1669077863659631
WDI_industry	Quantile-SFA	normal	large	gamma_0.25	0.0191701857927091	0.1656026685764504	0.0307515649026237	0.1815328931182232
WDI_industry	Quantile-SFA	normal	large	gamma_0.50	0.0187290944248149	0.1656944468934912	0.0307515575193202	0.1815420980955245
WDI_industry	Quantile-SFA	normal	large	gamma_0.75	0.0179922652813044	0.1656335447988974	0.0307515514324948	0.1815562865240716
WDI_industry	Quantile-SFA	skewed	large	free_gamma	0.1201330062470016	0.1762321484580722	0.0306842438982591	0.1935023834855958
WDI_industry	Quantile-SFA	skewed	large	gamma_0	0.1061392296734166	0.1703841958328617	0.030684362332795	0.2009409341370422
WDI_industry	Quantile-SFA	skewed	large	gamma_0.25	0.1222505613731163	0.177203481476129	0.030684278148581	0.1935015312753828
WDI_industry	Quantile-SFA	skewed	large	gamma_0.50	0.1176001107556457	0.1750797209806522	0.0306842070588718	0.1934846524461631
WDI_industry	Quantile-SFA	skewed	large	gamma_0.75	0.1122824053346205	0.1728383083943687	0.0306840982822961	0.1934890266122566
front41Data	HQER-SFA	hqer_induced	small	free_gamma	0.087269124809032	0.4187931337363054	0.0307857700798039	0.0921728257849403
front41Data	HQER-SFA	hqer_induced	small	gamma_0	0.0802565278386289	0.4065838754728086	0.030785780800665	0.0820956370978673
front41Data	HQER-SFA	hqer_induced	small	gamma_0.25	0.0839218286210644	0.4178955340346353	0.0307857665928762	0.0918860794665185
front41Data	HQER-SFA	hqer_induced	small	gamma_0.50	0.0885242809439428	0.4182835425698247	0.0332896814270749	0.092075020839122
front41Data	HQER-SFA	hqer_induced	small	gamma_0.75	0.0888700351701578	0.4155074693027528	0.0316069091742488	0.0912020005557099
front41Data	HQER-SFA	normal	small	free_gamma	0.0835895345405414	0.2129481980636941	0.030793355624176	0.1364073353709364
front41Data	HQER-SFA	normal	small	gamma_0	0.0894063894833391	0.2207603289067869	0.0307933900696209	0.1303165443484948
front41Data	HQER-SFA	normal	small	gamma_0.25	0.0913650021757939	0.2194224364283669	0.0307933655037767	0.1357471519866629
front41Data	HQER-SFA	normal	small	gamma_0.50	0.0821166429295399	0.2124263874304412	0.0307933543434463	0.1357843845512642
front41Data	HQER-SFA	normal	small	gamma_0.75	0.0765145367695161	0.2088229303820482	0.0307933482534282	0.136901917199222
front41Data	HQER-SFA	skewed	small	free_gamma	0.1419992254329399	0.2193781819238745	0.0308529325853247	0.1522856348985829
front41Data	HQER-SFA	skewed	small	gamma_0	0.1134642943377178	0.2138272643119765	0.0308529466165322	0.1454341849090308
front41Data	HQER-SFA	skewed	small	gamma_0.25	0.1421096962785376	0.2214556503735458	0.0308529328311256	0.1527835509863851
front41Data	HQER-SFA	skewed	small	gamma_0.50	0.1455927631708578	0.2210141532338465	0.0308529215032915	0.1522628507918866
front41Data	HQER-SFA	skewed	small	gamma_0.75	0.1448210422785524	0.2197967708924708	0.0308529238715293	0.1524801333703807
front41Data	Quantile-SFA	hqer_induced	small	free_gamma	0.0734806469568116	0.3661424851955087	0.0305696655494735	0.0974131703250903
front41Data	Quantile-SFA	hqer_induced	small	gamma_0	0.0726792186915537	0.3539891956301488	0.0305696717031442	0.0996846927387437
front41Data	Quantile-SFA	hqer_induced	small	gamma_0.25	0.0734410739615143	0.3600295264282044	0.0314157704595943	0.0999672131147541
front41Data	Quantile-SFA	hqer_induced	small	gamma_0.50	0.0756377277796226	0.3604880549211929	0.0305696825756245	0.0996882467352042
front41Data	Quantile-SFA	hqer_induced	small	gamma_0.75	0.0822958562760593	0.3572938891869355	0.0305696707153723	0.099104751319811
front41Data	Quantile-SFA	normal	small	free_gamma	0.0477579268413745	0.1309253226233829	0.0303423529434787	0.2486924145595999
front41Data	Quantile-SFA	normal	small	gamma_0	0.0453520832066597	0.1323390844538686	0.0303423537759605	0.2260621589800447
front41Data	Quantile-SFA	normal	small	gamma_0.25	0.0472165769650499	0.1319050923203432	0.03034235533758	0.2481906085023618

dataset_source	pseudo_model_source	error_scenario	sample_size_label	gamma_setting	mean_beta_error	mean_prediction_RMSE	mean_TE_RMSE	mean_TE_rank_corr
front41Data	Quantile-SFA	normal	small	gamma_0.50	0.0457850348852806	0.1298642601259842	0.0303423495503359	0.2488696860238956
front41Data	Quantile-SFA	normal	small	gamma_0.75	0.0472761545198562	0.1290279806572329	0.0303423525799149	0.249083365119491
front41Data	Quantile-SFA	skewed	small	free_gamma	0.0666782843882607	0.1181550240217149	0.0307233762493563	0.2557288135593221
front41Data	Quantile-SFA	skewed	small	gamma_0	0.0507828265244811	0.1196828084975184	0.0307234015718018	0.2557771323437679
front41Data	Quantile-SFA	skewed	small	gamma_0.25	0.0644423628267708	0.1182400949016459	0.0307233670317582	0.2566207279799945
front41Data	Quantile-SFA	skewed	small	gamma_0.50	0.068672812023659	0.11728491645028	0.0307233820354074	0.2562717421505974
front41Data	Quantile-SFA	skewed	small	gamma_0.75	0.0666151259020093	0.1155561710790374	0.0307233879906699	0.2563689913864963

Panel 2 of 2

Columns shown: `dataset_source`, `pseudo_model_source`, `error_scenario`, `sample_size_label`, `gamma_setting`, `convergence_rate`, `mean_n_iter`, `n_runs`.

dataset_source	pseudo_model_source	error_scenario	sample_size_label	gamma_setting	convergence_rate	mean_n_iter	n_runs
WDI_agriculture	HQER-SFA	hqer_induced	medium	free_gamma	1.0	27.43	100
WDI_agriculture	HQER-SFA	hqer_induced	medium	gamma_0	1.0	28.9	100
WDI_agriculture	HQER-SFA	hqer_induced	medium	gamma_0.25	1.0	18.72	100
WDI_agriculture	HQER-SFA	hqer_induced	medium	gamma_0.50	1.0	14.74	100
WDI_agriculture	HQER-SFA	hqer_induced	medium	gamma_0.75	1.0	14.34	100
WDI_agriculture	HQER-SFA	normal	medium	free_gamma	1.0	33.42	100
WDI_agriculture	HQER-SFA	normal	medium	gamma_0	1.0	25.49	100
WDI_agriculture	HQER-SFA	normal	medium	gamma_0.25	1.0	20.3	100
WDI_agriculture	HQER-SFA	normal	medium	gamma_0.50	1.0	17.22	100
WDI_agriculture	HQER-SFA	normal	medium	gamma_0.75	1.0	17.31	100
WDI_agriculture	HQER-SFA	skewed	medium	free_gamma	1.0	28.9	100
WDI_agriculture	HQER-SFA	skewed	medium	gamma_0	1.0	25.54	100
WDI_agriculture	HQER-SFA	skewed	medium	gamma_0.25	1.0	25.75	100
WDI_agriculture	HQER-SFA	skewed	medium	gamma_0.50	1.0	27.65	100
WDI_agriculture	HQER-SFA	skewed	medium	gamma_0.75	1.0	26.89	100
WDI_agriculture	Quantile-SFA	hqer_induced	medium	free_gamma	1.0	22.16	100
WDI_agriculture	Quantile-SFA	hqer_induced	medium	gamma_0	1.0	28.31	100
WDI_agriculture	Quantile-SFA	hqer_induced	medium	gamma_0.25	1.0	17.91	100
WDI_agriculture	Quantile-SFA	hqer_induced	medium	gamma_0.50	1.0	17.48	100
WDI_agriculture	Quantile-SFA	hqer_induced	medium	gamma_0.75	1.0	15.54	100
WDI_agriculture	Quantile-SFA	normal	medium	free_gamma	1.0	23.38	100
WDI_agriculture	Quantile-SFA	normal	medium	gamma_0	1.0	28.69	100
WDI_agriculture	Quantile-SFA	normal	medium	gamma_0.25	1.0	22.93	100
WDI_agriculture	Quantile-SFA	normal	medium	gamma_0.50	1.0	22.79	100
WDI_agriculture	Quantile-SFA	normal	medium	gamma_0.75	1.0	23.65	100
WDI_agriculture	Quantile-SFA	skewed	medium	free_gamma	1.0	24.31	100
WDI_agriculture	Quantile-SFA	skewed	medium	gamma_0	1.0	26.94	100
WDI_agriculture	Quantile-SFA	skewed	medium	gamma_0.25	1.0	23.14	100
WDI_agriculture	Quantile-SFA	skewed	medium	gamma_0.50	1.0	23.11	100
WDI_agriculture	Quantile-SFA	skewed	medium	gamma_0.75	1.0	23.42	100
WDI_industry	HQER-SFA	hqer_induced	large	free_gamma	1.0	37.53	100
WDI_industry	HQER-SFA	hqer_induced	large	gamma_0	1.0	20.88	100
WDI_industry	HQER-SFA	hqer_induced	large	gamma_0.25	1.0	18.52	100
WDI_industry	HQER-SFA	hqer_induced	large	gamma_0.50	1.0	18.52	100
WDI_industry	HQER-SFA	hqer_induced	large	gamma_0.75	1.0	15.46	100
WDI_industry	HQER-SFA	normal	large	free_gamma	1.0	50.72	100
WDI_industry	HQER-SFA	normal	large	gamma_0	1.0	27.1	100
WDI_industry	HQER-SFA	normal	large	gamma_0.25	1.0	24.49	100
WDI_industry	HQER-SFA	normal	large	gamma_0.50	1.0	22.15	100
WDI_industry	HQER-SFA	normal	large	gamma_0.75	1.0	21.81	100
WDI_industry	HQER-SFA	skewed	large	free_gamma	1.0	25.99	100
WDI_industry	HQER-SFA	skewed	large	gamma_0	1.0	26.33	100
WDI_industry	HQER-SFA	skewed	large	gamma_0.25	1.0	26.84	100
WDI_industry	HQER-SFA	skewed	large	gamma_0.50	1.0	25.4	100
WDI_industry	HQER-SFA	skewed	large	gamma_0.75	1.0	24.57	100
WDI_industry	Quantile-SFA	hqer_induced	large	free_gamma	1.0	36.32	100
WDI_industry	Quantile-SFA	hqer_induced	large	gamma_0	1.0	20.73	100
WDI_industry	Quantile-SFA	hqer_induced	large	gamma_0.25	1.0	18.89	100
WDI_industry	Quantile-SFA	hqer_induced	large	gamma_0.50	1.0	16.86	100
WDI_industry	Quantile-SFA	hqer_induced	large	gamma_0.75	1.0	14.91	100
WDI_industry	Quantile-SFA	normal	large	free_gamma	1.0	46.81	100
WDI_industry	Quantile-SFA	normal	large	gamma_0	1.0	27.53	100
WDI_industry	Quantile-SFA	normal	large	gamma_0.25	1.0	25.69	100
WDI_industry	Quantile-SFA	normal	large	gamma_0.50	1.0	23.36	100
WDI_industry	Quantile-SFA	normal	large	gamma_0.75	1.0	20.12	100
WDI_industry	Quantile-SFA	skewed	large	free_gamma	1.0	27.25	100
WDI_industry	Quantile-SFA	skewed	large	gamma_0	1.0	27.6	100
WDI_industry	Quantile-SFA	skewed	large	gamma_0.25	1.0	26.42	100
WDI_industry	Quantile-SFA	skewed	large	gamma_0.50	1.0	25.23	100
WDI_industry	Quantile-SFA	skewed	large	gamma_0.75	1.0	25.89	100
front41Data	HQER-SFA	hqer_induced	small	free_gamma	1.0	30.89	100
front41Data	HQER-SFA	hqer_induced	small	gamma_0	1.0	24.13	100
front41Data	HQER-SFA	hqer_induced	small	gamma_0.25	1.0	27.82	100
front41Data	HQER-SFA	hqer_induced	small	gamma_0.50	1.0	23.75	100
front41Data	HQER-SFA	hqer_induced	small	gamma_0.75	1.0	22.53	100
front41Data	HQER-SFA	normal	small	free_gamma	1.0	40.61	100
front41Data	HQER-SFA	normal	small	gamma_0	1.0	32.86	100
front41Data	HQER-SFA	normal	small	gamma_0.25	1.0	36.16	100
front41Data	HQER-SFA	normal	small	gamma_0.50	1.0	31.48	100
front41Data	HQER-SFA	normal	small	gamma_0.75	1.0	28.75	100
front41Data	HQER-SFA	skewed	small	free_gamma	1.0	40.18	100
front41Data	HQER-SFA	skewed	small	gamma_0	1.0	41.54	100
front41Data	HQER-SFA	skewed	small	gamma_0.25	1.0	38.99	100
front41Data	HQER-SFA	skewed	small	gamma_0.50	1.0	35.17	100
front41Data	HQER-SFA	skewed	small	gamma_0.75	1.0	32.02	100
front41Data	Quantile-SFA	hqer_induced	small	free_gamma	1.0	40.26	100
front41Data	Quantile-SFA	hqer_induced	small	gamma_0	1.0	27.89	100
front41Data	Quantile-SFA	hqer_induced	small	gamma_0.25	1.0	31.51	100
front41Data	Quantile-SFA	hqer_induced	small	gamma_0.50	1.0	25.76	100

dataset_source	pseudo_model_source	error_scenario	sample_size_label	gamma_setting	convergence_rate	mean_n_iter	n_runs
front41Data	Quantile-SFA	hqer_induced	small	gamma_0.75	1.0	22.07	100
front41Data	Quantile-SFA	normal	small	free_gamma	1.0	39.27	100
front41Data	Quantile-SFA	normal	small	gamma_0	1.0	32.3	100
front41Data	Quantile-SFA	normal	small	gamma_0.25	1.0	35.24	100
front41Data	Quantile-SFA	normal	small	gamma_0.50	1.0	33.19	100
front41Data	Quantile-SFA	normal	small	gamma_0.75	1.0	29.88	100
front41Data	Quantile-SFA	skewed	small	free_gamma	1.0	41.28	100
front41Data	Quantile-SFA	skewed	small	gamma_0	1.0	41.47	100
front41Data	Quantile-SFA	skewed	small	gamma_0.25	1.0	40.72	100
front41Data	Quantile-SFA	skewed	small	gamma_0.50	1.0	37.21	100
front41Data	Quantile-SFA	skewed	small	gamma_0.75	1.0	34.03	100

S9 Source-assisted full penalty grid

Source CSV: `tables/s9_source_assisted_full_penalty_grid.csv`. Rows displayed: 13; columns displayed: 16. The table is split into panels to keep all cells inside the page boundary.

Panel 1 of 3

Columns shown: `transfer_method`, `source_domain`, `lambda_value`, `n_folds`, `mean_validation_rmse`, `std_validation_rmse`, `mean_validation_mae`, `mean_validation_loglikelihood`, `convergence_rate`.

transfer_method	source_domain	lambda_value	n_folds	mean_validation_rmse	std_validation_rmse	mean_validation_mae	mean_validation_loglikelihood	convergence_rate
Negative Penalty Transfer	WDI_agriculture	0.0	100	0.4282396611232928	0.0886439278212841	0.3394777263501518	-3.2448829769555347	1.0
Negative Penalty Transfer	WDI_agriculture	0.01	100	0.4269110553440557	0.0864863117709108	0.3383436514401491	-3.0988622424047976	1.0
Negative Penalty Transfer	WDI_agriculture	0.1	100	0.4336605874469904	0.0896343344592168	0.3433128039809655	-3.155505571014225	1.0
Negative Penalty Transfer	WDI_agriculture	1.0	100	0.5052122490363928	0.0833548729479053	0.4142154633504188	-2.7590802033594906	1.0
Negative Penalty Transfer	WDI_agriculture	10.0	100	20.21554726620588	0.3088101767799763	20.18249036973261	-48.64912455205612	1.0
Negative Source Initialization	WDI_agriculture	0.0	100	0.4282396611232928	0.0886439278212841	0.3394777263501518	-3.2448829769555347	1.0
No Transfer	none	0.0	100	0.4282396611232928	0.0886439278212841	0.3394777263501518	-3.2448829769555347	1.0
Penalty Transfer	WDI_industry	0.0	100	0.4177879804477211	0.0911231398874926	0.3366324048555564	-3.627324557619026	1.0
Penalty Transfer	WDI_industry	0.01	100	0.4188597192649108	0.0920279433109142	0.3380366581533323	-3.5501023818921995	1.0
Penalty Transfer	WDI_industry	0.1	100	0.4310111341374472	0.0959892023059608	0.3499651447770092	-3.424242600302061	1.0
Penalty Transfer	WDI_industry	1.0	100	0.48301035632523	0.0796973367329077	0.3912627825685185	-2.3523825302117727	1.0
Penalty Transfer	WDI_industry	10.0	100	21.322869374489564	0.4967267094087163	21.21126408098392	-50.84056372149927	1.0
Source Initialization Transfer	WDI_industry	0.0	100	0.4177879804477211	0.0911231398874926	0.3366324048555564	-3.627324557619026	1.0

Panel 2 of 3

Columns shown: `transfer_method`, `source_domain`, `lambda_value`, `mean_TE_valid`, `median_TE_valid`, `low_efficiency_ratio_valid`, `mean_TE_stability`, `TE_rank_stability`, `beta_stability`.

transfer_method	source_domain	lambda_value	mean_TE_valid	median_TE_valid	low_efficiency_ratio_valid	mean_TE_stability	TE_rank_stability	beta_stability
Negative Penalty Transfer	WDI_agriculture	0.0	0.9505301061660768	0.9505301109081418	0.0	1.2743079591315265e-07	0.9764335664335664	0.0691207480745605
Negative Penalty Transfer	WDI_agriculture	0.01	0.9505300728786382	0.950530074027546	0.0	1.312171440204235e-07	0.9783916083916084	0.0647894146827068
Negative Penalty Transfer	WDI_agriculture	0.1	0.9481565468662124	0.9488590391698613	0.0033333333333333	0.0001553085779389	0.98	0.0590045871693676
Negative Penalty Transfer	WDI_agriculture	1.0	0.950529098384875	0.950529047951564	0.0	2.0081206121957965e-07	0.9848951048951048	0.1094208776308938
Negative Penalty Transfer	WDI_agriculture	10.0	0.9418034801809504	0.9418029005436686	0.0	1.697878017328236e-06	0.277972027972028	19.93896018599525
Negative Source Initialization	WDI_agriculture	0.0	0.9505301061660768	0.9505301109081418	0.0	1.2743079591315265e-07	0.9764335664335664	0.0691207480745605
No Transfer	none	0.0	0.9505301061660768	0.9505301109081418	0.0	1.2743079591315265e-07	0.9764335664335664	0.0691207480745605
Penalty Transfer	WDI_industry	0.0	0.9505300961437894	0.9505301391211272	0.0	1.3516018206982672e-07	0.973916083916084	0.1051707861206782
Penalty Transfer	WDI_industry	0.01	0.9505300656863884	0.950530099693575	0.0	1.3748252266743144e-07	0.9755944055944056	0.1038239588788011
Penalty Transfer	WDI_industry	0.1	0.9505300596119812	0.9505300505420696	0.0	1.360116880977369e-07	0.9793706293706296	0.104343605398592
Penalty Transfer	WDI_industry	1.0	0.9505300671865669	0.950529908779388	0.0	1.1422166353414199e-07	0.9844055944055944	0.0884184792041563
Penalty Transfer	WDI_industry	10.0	0.9505301466394168	0.9505301465160666	0.0	1.179668507322873e-10	0.2786713286713287	21.048313950864912
Source Initialization Transfer	WDI_industry	0.0	0.9505300961437894	0.9505301391211272	0.0	1.3516018206982672e-07	0.973916083916084	0.1051707861206782

Panel 3 of 3

Columns shown: `transfer_method`, `source_domain`, `lambda_value`, `mean_elapsed_time_seconds`.

transfer_method	source_domain	lambda_value	mean_elapsed_time_seconds
Negative Penalty Transfer	WDI_agriculture	0.0	0.8822214550012722
Negative Penalty Transfer	WDI_agriculture	0.01	4.361701389000518
Negative Penalty Transfer	WDI_agriculture	0.1	3.039500609000679
Negative Penalty Transfer	WDI_agriculture	1.0	3.080618725993554
Negative Penalty Transfer	WDI_agriculture	10.0	1.3482843419950223
Negative Source Initialization	WDI_agriculture	0.0	2.560427244995953
No Transfer	none	0.0	4.219181189993979
Penalty Transfer	WDI_industry	0.0	0.8211411070002942
Penalty Transfer	WDI_industry	0.01	4.72582867600373
Penalty Transfer	WDI_industry	0.1	4.336196349991951
Penalty Transfer	WDI_industry	1.0	3.844122713003308
Penalty Transfer	WDI_industry	10.0	2.5076991059910507
Source Initialization Transfer	WDI_industry	0.0	3.089039422003552

S10 Source-assisted paired checks

Source CSV: `tables/s10_source_assisted_paired_checks.csv`. Rows displayed: 12; columns displayed: 11. The table is split into panels to keep all cells inside the page boundary.

Panel 1 of 2

Columns shown: `comparison, metric, n_pairs, mean_difference, median_difference, bootstrap_ci_lower, bootstrap_ci_upper, wilcoxon_pvalue, sign_test_pvalue`.

comparison	metric	n_pairs	mean_difference	median_difference	bootstrap_ci_lower	bootstrap_ci_upper	wilcoxon_pvalue	sign_test_pvalue
WDI_industry Source Initialization vs No Transfer	validation_rmse	100	0.0104516806755717	0.0	0.0050715821145828	0.0163485926157085	0.0041523485932195	0.175465248990804
WDI_industry Source Initialization vs No Transfer	validation_loglikelihood	100	-0.3824415806634913	0.0	-0.5425638969934375	-0.2268598999261561	6.16519479038218e-06	4.176981747150421e-07
WDI_industry Source Initialization vs No Transfer	beta_stability	100	-0.0360500380461176	0.0	-0.0496984753643977	-0.0244787802701905	3.823644502975134e-07	2.0954757928848267e-09
WDI_industry Source Initialization vs No Transfer	TE_rank_stability	100	-0.0025174825174825	0.0	-0.0059440559440559	7.167832167833065e-05	0.1847621013054982	0.5034446716308594
WDI_industry best Penalty Transfer vs No Transfer	validation_rmse	100	0.0104516806755717	0.0	0.005001519789478	0.0161696716552548	0.0041523485932195	0.175465248990804
WDI_industry best Penalty Transfer vs No Transfer	validation_loglikelihood	100	-0.3824415806634913	0.0	-0.5464527511010085	-0.2190747660935238	6.16519479038218e-06	4.176981747150421e-07
WDI_industry best Penalty Transfer vs No Transfer	beta_stability	100	-0.0360500380461176	0.0	-0.0489100315361306	-0.0239972102900037	3.823644502975134e-07	2.0954757928848267e-09
WDI_industry best Penalty Transfer vs No Transfer	TE_rank_stability	100	-0.0025174825174825	0.0	-0.0059458041958042	0.0001416083916083	0.1847621013054982	0.5034446716308594
WDI_industry Source Initialization vs WDI_agriculture Source Initialization	validation_rmse	100	0.0104516806755717	0.0	0.0050119771860771	0.0159702131724203	0.0041523485932195	0.175465248990804
WDI_industry Source Initialization vs WDI_agriculture Source Initialization	validation_loglikelihood	100	-0.3824415806634913	0.0	-0.5454332735552981	-0.2257148036150446	6.16519479038218e-06	4.176981747150421e-07
WDI_industry Source Initialization vs WDI_agriculture Source Initialization	beta_stability	100	-0.0360500380461176	0.0	-0.0489392127918068	-0.0242285959704482	3.823644502975134e-07	2.0954757928848267e-09
WDI_industry Source Initialization vs WDI_agriculture Source Initialization	TE_rank_stability	100	-0.0025174825174825	0.0	-0.0055262237762237	0.0002097902097902	0.1847621013054982	0.5034446716308594

Panel 2 of 2

Columns shown: `comparison, metric, interpretation, best_industry_penalty_lambda`.

comparison	metric	interpretation	best_industry_penalty_lambda
WDI_industry Source Initialization vs No Transfer	validation_rmse	Paired check provides additional support for WDI_industry Source Initialization under the current formal design.	
WDI_industry Source Initialization vs No Transfer	validation_loglikelihood	Paired check favors No Transfer under the current formal design.	
WDI_industry Source Initialization vs No Transfer	beta_stability	Paired check favors No Transfer under the current formal design.	
WDI_industry Source Initialization vs No Transfer	TE_rank_stability	No clear paired statistical support at the current formal design.	
WDI_industry best Penalty Transfer vs No Transfer	validation_rmse	Paired check provides additional support for WDI_industry best Penalty Transfer under the current formal design.	0.0
WDI_industry best Penalty Transfer vs No Transfer	validation_loglikelihood	Paired check favors No Transfer under the current formal design.	0.0
WDI_industry best Penalty Transfer vs No Transfer	beta_stability	Paired check favors No Transfer under the current formal design.	0.0
WDI_industry best Penalty Transfer vs No Transfer	TE_rank_stability	No clear paired statistical support at the current formal design.	0.0
WDI_industry Source Initialization vs WDI_agriculture Source Initialization	validation_rmse	Paired check provides additional support for WDI_industry Source Initialization under the current formal design.	
WDI_industry Source Initialization vs WDI_agriculture Source Initialization	validation_loglikelihood	Paired check favors WDI_agriculture Source Initialization under the current formal design.	
WDI_industry Source Initialization vs WDI_agriculture Source Initialization	beta_stability	Paired check favors WDI_agriculture Source Initialization under the current formal design.	
WDI_industry Source Initialization vs WDI_agriculture Source Initialization	TE_rank_stability	No clear paired statistical support at the current formal design.	

S11 Convergence diagnostics by model

Source CSV: `tables/s11_convergence_diagnostics_by_model.csv`. Rows displayed: 9; columns displayed: 13. The table is split into panels to keep all cells inside the page boundary.

Panel 1 of 2

Columns shown: `experiment`, `model`, `n_runs`, `n_converged`, `convergence_rate`, `mean_n_iter`, `median_n_iter`, `max_n_iter`, `mean_loglik`.

experiment	model	n__runs	n__converged	convergence__rate	mean__n__iter	median__n__iter	max__n__iter	mean__loglik
	HQER-SFA	24018	24018	1.0	30.812598884170207	28.0	136.0	-444.4710445867176
	Quantile-SFA	24018	24018	1.0	26.86426846531768	26.0	83.0	-455.73228259255336
	Traditional-SFA	3	3	1.0	22.666666666666668	17.0	38.0	-4250.924033817086
	free__gamma	1803	1803	1.0	34.27232390460344	31.0	113.0	-9.393518136554368
	gamma__0	1803	1803	1.0	28.56017748197449	27.0	77.0	-34.370766245211456
	gamma__0.25	1803	1803	1.0	26.65834719911259	25.0	97.0	-14.444218353276916
	gamma__0.50	1803	1803	1.0	24.490293954520244	23.0	68.0	-12.523405214463947
	gamma__0.75	1803	1803	1.0	22.94120909595119	23.0	64.0	-14.14046221524969
		1300	1300	1.0	27.24923076923077	24.0	94.0	

Panel 2 of 2

Columns shown: `experiment`, `model`, `failed_count`, `warning_count`, `best_start_distribution`, `notes`.

experiment	model	failed__count	warning__count	best__start__distribution	notes
	HQER-SFA	0	4800	2.0:7900; 4.0:6775; 1.0:4426; 3.0:3164; 5.0:1738; NA:15	NA fields indicate that the source output did not record that diagnostic.
	Quantile-SFA	0	4800	2.0:7234; 4.0:6249; 1.0:4739; 3.0:3720; 5.0:2061; NA:15	NA fields indicate that the source output did not record that diagnostic.
	Traditional-SFA	0	0	3.0:1; 5.0:1; 1.0:1	NA fields indicate that the source output did not record that diagnostic.
	free__gamma	0	0	2.0:1015; 1.0:307; 5.0:244; 4.0:187; 3.0:50	NA fields indicate that the source output did not record that diagnostic.
	gamma__0	0	0	2.0:655; 1.0:466; 4.0:321; 5.0:205; 3.0:156	NA fields indicate that the source output did not record that diagnostic.
	gamma__0.25	0	0	2.0:952; 1.0:333; 5.0:250; 4.0:194; 3.0:74	NA fields indicate that the source output did not record that diagnostic.
	gamma__0.50	0	0	2.0:986; 1.0:318; 5.0:253; 4.0:165; 3.0:81	NA fields indicate that the source output did not record that diagnostic.
	gamma__0.75	0	0	2.0:987; 1.0:313; 5.0:239; 4.0:170; 3.0:94	NA fields indicate that the source output did not record that diagnostic.
		0	600	4.0:904; 2.0:220; 1.0:131; 3.0:32; 5.0:13	NA fields indicate that the source output did not record that diagnostic.

S12 Warning/failure cases

Source CSV: `tables/s12_warning_failure_cases.csv`. Rows displayed: 10200; columns displayed: 9. This table displays all rows from the source CSV.

experiment	dataset	model	scenario	converged	n_iter	loglik	warning_message	best_start_id
	front41Data	Quantile-SFA	hqer_induced	True	47	-4.9946541692469495	hqer_induced sampler prototype	4.0
	front41Data	HQER-SFA	hqer_induced	True	17	-5.03892546965607	hqer_induced sampler prototype	4.0
	front41Data	Quantile-SFA	hqer_induced	True	45	2.4791224327267307	hqer_induced sampler prototype	4.0
	front41Data	HQER-SFA	hqer_induced	True	47	2.502385836957131	hqer_induced sampler prototype	4.0
	front41Data	Quantile-SFA	hqer_induced	True	23	-0.2706664912887655	hqer_induced sampler prototype	4.0
	front41Data	HQER-SFA	hqer_induced	True	29	-0.2580096111624791	hqer_induced sampler prototype	4.0
	front41Data	Quantile-SFA	hqer_induced	True	16	0.4642418173569262	hqer_induced sampler prototype	4.0
	front41Data	HQER-SFA	hqer_induced	True	20	0.475745767443531	hqer_induced sampler prototype	4.0
	front41Data	Quantile-SFA	hqer_induced	True	34	11.456666356104453	hqer_induced sampler prototype	4.0
	front41Data	HQER-SFA	hqer_induced	True	43	11.481718679318629	hqer_induced sampler prototype	2.0
	front41Data	Quantile-SFA	hqer_induced	True	34	13.560186322931305	hqer_induced sampler prototype	4.0
	front41Data	HQER-SFA	hqer_induced	True	27	13.512895444954465	hqer_induced sampler prototype	4.0
	front41Data	Quantile-SFA	hqer_induced	True	17	10.188334050486016	hqer_induced sampler prototype	4.0
	front41Data	HQER-SFA	hqer_induced	True	19	10.189646335666485	hqer_induced sampler prototype	4.0
	front41Data	Quantile-SFA	hqer_induced	True	30	-1.9190396622093249	hqer_induced sampler prototype	2.0
	front41Data	HQER-SFA	hqer_induced	True	34	-1.9534540340712765	hqer_induced sampler prototype	2.0
	front41Data	Quantile-SFA	hqer_induced	True	27	-7.0737908344639	hqer_induced sampler prototype	4.0
	front41Data	HQER-SFA	hqer_induced	True	10	-7.141198228550425	hqer_induced sampler prototype	2.0
	front41Data	Quantile-SFA	hqer_induced	True	30	3.4958591658621523	hqer_induced sampler prototype	2.0
	front41Data	HQER-SFA	hqer_induced	True	32	3.592232661754436	hqer_induced sampler prototype	4.0
	front41Data	Quantile-SFA	hqer_induced	True	6	1.0971973115246525	hqer_induced sampler prototype	2.0
	front41Data	HQER-SFA	hqer_induced	True	35	1.466600908713313	hqer_induced sampler prototype	4.0
	front41Data	Quantile-SFA	hqer_induced	True	15	2.6868947167194803	hqer_induced sampler prototype	4.0
	front41Data	HQER-SFA	hqer_induced	True	20	2.6871077554215423	hqer_induced sampler prototype	4.0
	front41Data	Quantile-SFA	hqer_induced	True	28	-1.7647479300755646	hqer_induced sampler prototype	4.0
	front41Data	HQER-SFA	hqer_induced	True	45	-1.7465692019431156	hqer_induced sampler prototype	4.0
	front41Data	Quantile-SFA	hqer_induced	True	25	11.3571770738043	hqer_induced sampler prototype	3.0
	front41Data	HQER-SFA	hqer_induced	True	27	11.011911358979525	hqer_induced sampler prototype	4.0
	front41Data	Quantile-SFA	hqer_induced	True	23	0.4260270468978457	hqer_induced sampler prototype	2.0
	front41Data	HQER-SFA	hqer_induced	True	45	0.4814495740131097	hqer_induced sampler prototype	2.0
	front41Data	Quantile-SFA	hqer_induced	True	13	9.85835196555608	hqer_induced sampler prototype	4.0
	front41Data	HQER-SFA	hqer_induced	True	16	9.896838067303465	hqer_induced sampler prototype	4.0
	front41Data	Quantile-SFA	hqer_induced	True	47	-6.261154368696439	hqer_induced sampler prototype	4.0
	front41Data	HQER-SFA	hqer_induced	True	43	-6.267531493910474	hqer_induced sampler prototype	2.0
	front41Data	Quantile-SFA	hqer_induced	True	30	0.599513996798971	hqer_induced sampler prototype	2.0
	front41Data	HQER-SFA	hqer_induced	True	24	0.5926332671366779	hqer_induced sampler prototype	4.0
	front41Data	Quantile-SFA	hqer_induced	True	33	6.244672243780535	hqer_induced sampler prototype	4.0
	front41Data	HQER-SFA	hqer_induced	True	41	6.2741512400233095	hqer_induced sampler prototype	4.0
	front41Data	Quantile-SFA	hqer_induced	True	15	16.108718741628405	hqer_induced sampler prototype	2.0
	front41Data	HQER-SFA	hqer_induced	True	52	18.154348784410114	hqer_induced sampler prototype	4.0
	front41Data	Quantile-SFA	hqer_induced	True	39	9.39103449351324	hqer_induced sampler prototype	2.0
	front41Data	HQER-SFA	hqer_induced	True	17	8.775641002793272	hqer_induced sampler prototype	4.0
	front41Data	Quantile-SFA	hqer_induced	True	47	2.407328006856546	hqer_induced sampler prototype	4.0
	front41Data	HQER-SFA	hqer_induced	True	34	2.41767669937369	hqer_induced sampler prototype	4.0
	front41Data	Quantile-SFA	hqer_induced	True	26	8.131942880286362	hqer_induced sampler prototype	4.0
	front41Data	HQER-SFA	hqer_induced	True	31	8.178701301506758	hqer_induced sampler prototype	4.0
	front41Data	Quantile-SFA	hqer_induced	True	35	11.754844990870753	hqer_induced sampler prototype	4.0
	front41Data	HQER-SFA	hqer_induced	True	17	11.735290466286369	hqer_induced sampler prototype	4.0
	front41Data	Quantile-SFA	hqer_induced	True	26	3.925999876121697	hqer_induced sampler prototype	2.0
	front41Data	HQER-SFA	hqer_induced	True	31	3.9422380782947295	hqer_induced sampler prototype	2.0
	front41Data	Quantile-SFA	hqer_induced	True	27	-2.8703227280468053	hqer_induced sampler prototype	4.0
	front41Data	HQER-SFA	hqer_induced	True	32	-2.864457725289404	hqer_induced sampler prototype	4.0
	front41Data	Quantile-SFA	hqer_induced	True	27	0.8542797180598632	hqer_induced sampler prototype	2.0
	front41Data	HQER-SFA	hqer_induced	True	24	0.7538905170313012	hqer_induced sampler prototype	2.0
	front41Data	Quantile-SFA	hqer_induced	True	42	-6.557672703697791	hqer_induced sampler prototype	4.0
	front41Data	HQER-SFA	hqer_induced	True	44	-6.546312135634077	hqer_induced sampler prototype	4.0
	front41Data	Quantile-SFA	hqer_induced	True	38	4.375077086255859	hqer_induced sampler prototype	4.0
	front41Data	HQER-SFA	hqer_induced	True	19	4.317979947513226	hqer_induced sampler prototype	4.0
	front41Data	Quantile-SFA	hqer_induced	True	20	8.37564627766897	hqer_induced sampler prototype	2.0
	front41Data	HQER-SFA	hqer_induced	True	14	8.378583590319511	hqer_induced sampler prototype	2.0
	front41Data	Quantile-SFA	hqer_induced	True	23	5.343560161604621	hqer_induced sampler prototype	4.0
	front41Data	HQER-SFA	hqer_induced	True	33	5.351592119950007	hqer_induced sampler prototype	4.0
	front41Data	Quantile-SFA	hqer_induced	True	33	-1.1672804651521511	hqer_induced sampler prototype	2.0
	front41Data	HQER-SFA	hqer_induced	True	43	-1.1522053925630775	hqer_induced sampler prototype	4.0
	front41Data	Quantile-SFA	hqer_induced	True	17	12.710325716138332	hqer_induced sampler prototype	2.0
	front41Data	HQER-SFA	hqer_induced	True	13	12.71965489931279	hqer_induced sampler prototype	2.0
	front41Data	Quantile-SFA	hqer_induced	True	35	3.3473552664686883	hqer_induced sampler prototype	2.0
	front41Data	HQER-SFA	hqer_induced	True	36	3.340358050364866	hqer_induced sampler prototype	2.0
	front41Data	Quantile-SFA	hqer_induced	True	24	0.4505286684772712	hqer_induced sampler prototype	4.0
	front41Data	HQER-SFA	hqer_induced	True	30	0.5718318297383516	hqer_induced sampler prototype	4.0
	front41Data	Quantile-SFA	hqer_induced	True	15	0.2902348435396232	hqer_induced sampler prototype	4.0
	front41Data	HQER-SFA	hqer_induced	True	31	0.3667576282539665	hqer_induced sampler prototype	4.0
	front41Data	Quantile-SFA	hqer_induced	True	47	5.7870603668558935	hqer_induced sampler prototype	4.0
	front41Data	HQER-SFA	hqer_induced	True	17	5.800011736315641	hqer_induced sampler prototype	2.0
	front41Data	Quantile-SFA	hqer_induced	True	34	-0.5824798280635197	hqer_induced sampler prototype	2.0
	front41Data	HQER-SFA	hqer_induced	True	36	-0.5533880328312988	hqer_induced sampler prototype	4.0
	front41Data	Quantile-SFA	hqer_induced	True	37	9.783309474143817	hqer_induced sampler prototype	4.0
	front41Data	HQER-SFA	hqer_induced	True	29	9.773621345116863	hqer_induced sampler prototype	4.0
	front41Data	Quantile-SFA	hqer_induced	True	50	-8.744657382428063	hqer_induced sampler prototype	2.0
	front41Data	HQER-SFA	hqer_induced	True	22	-8.839302800486593	hqer_induced sampler prototype	4.0
	front41Data	Quantile-SFA	hqer_induced	True	35	4.480739029035638	hqer_induced sampler prototype	2.0
	front41Data	HQER-SFA	hqer_induced	True	55	4.501720085191634	hqer_induced sampler prototype	2.0
	front41Data	Quantile-SFA	hqer_induced	True	53	1.5905374621267248	hqer_induced sampler prototype	2.0
	front41Data	HQER-SFA	hqer_induced	True	24	1.5940276322698643	hqer_induced sampler prototype	4.0
	front41Data	Quantile-SFA	hqer_induced	True	39	16.186184136839383	hqer_induced sampler prototype	2.0
	front41Data	HQER-SFA	hqer_induced	True	24	16.196827388364433	hqer_induced sampler prototype	4.0
	front41Data	Quantile-SFA	hqer_induced	True	18	3.3341184054592183	hqer_induced sampler prototype	2.0
	front41Data	HQER-SFA	hqer_induced	True	18	3.0807449375741576	hqer_induced sampler prototype	4.0
	front41Data	Quantile-SFA	hqer_induced	True	13	6.747674153683047	hqer_induced sampler prototype	2.0
	front41Data	HQER-SFA	hqer_induced	True	16	6.758182541727201	hqer_induced sampler prototype	2.0
	front41Data	Quantile-SFA	hqer_induced	True	32	6.263460743010446	hqer_induced sampler prototype	4.0
	front41Data	HQER-SFA	hqer_induced	True	46	6.177123001305926	hqer_induced sampler prototype	4.0
	front41Data	Quantile-SFA	hqer_induced	True	42	-3.9369204744091615	hqer_induced sampler prototype	4.0
	front41Data	HQER-SFA	hqer_induced	True	23	-4.001701687030943	hqer_induced sampler prototype	4.0

experiment	dataset	model	scenario	converged	n_iter	loglik	warning_message	best_start_id
	front41Data	Quantile-SFA	hqer_induced	True	32	6.046211293180806	hqer_induced sampler prototype	4.0
	front41Data	HQER-SFA	hqer_induced	True	47	6.090687798189213	hqer_induced sampler prototype	4.0
	front41Data	Quantile-SFA	hqer_induced	True	26	-0.0612736231735678	hqer_induced sampler prototype	4.0
	front41Data	HQER-SFA	hqer_induced	True	42	-0.053438504028457	hqer_induced sampler prototype	2.0
	front41Data	Quantile-SFA	hqer_induced	True	36	4.765199113695056	hqer_induced sampler prototype	2.0
	front41Data	HQER-SFA	hqer_induced	True	59	4.829318020392884	hqer_induced sampler prototype	4.0
	front41Data	Quantile-SFA	hqer_induced	True	8	-0.0176880368577321	hqer_induced sampler prototype	2.0
	front41Data	HQER-SFA	hqer_induced	True	27	0.1458720545631159	hqer_induced sampler prototype	2.0
	front41Data	Quantile-SFA	hqer_induced	True	25	-2.567857406770179	hqer_induced sampler prototype	2.0
	front41Data	HQER-SFA	hqer_induced	True	39	-2.5406576743771865	hqer_induced sampler prototype	2.0
	front41Data	Quantile-SFA	hqer_induced	True	21	7.101225481939336	hqer_induced sampler prototype	2.0
	front41Data	HQER-SFA	hqer_induced	True	18	7.0910139862168	hqer_induced sampler prototype	2.0
	front41Data	Quantile-SFA	hqer_induced	True	65	-1.6876507691438791	hqer_induced sampler prototype	4.0
	front41Data	HQER-SFA	hqer_induced	True	46	-1.6196604209771437	hqer_induced sampler prototype	4.0
	front41Data	Quantile-SFA	hqer_induced	True	18	3.2933090138728027	hqer_induced sampler prototype	4.0
	front41Data	HQER-SFA	hqer_induced	True	23	3.3157843463691496	hqer_induced sampler prototype	4.0
	front41Data	Quantile-SFA	hqer_induced	True	52	-2.1479182998960087	hqer_induced sampler prototype	4.0
	front41Data	HQER-SFA	hqer_induced	True	26	-2.145465973712742	hqer_induced sampler prototype	2.0
	front41Data	Quantile-SFA	hqer_induced	True	31	-3.990057760082733	hqer_induced sampler prototype	2.0
	front41Data	HQER-SFA	hqer_induced	True	35	-4.197457536080146	hqer_induced sampler prototype	4.0
	front41Data	Quantile-SFA	hqer_induced	True	18	3.910905335684516	hqer_induced sampler prototype	4.0
	front41Data	HQER-SFA	hqer_induced	True	24	3.9364728654238657	hqer_induced sampler prototype	4.0
	front41Data	Quantile-SFA	hqer_induced	True	43	-0.4132607512045833	hqer_induced sampler prototype	4.0
	front41Data	HQER-SFA	hqer_induced	True	12	-0.6687761211722464	hqer_induced sampler prototype	2.0
	front41Data	Quantile-SFA	hqer_induced	True	23	1.037996686604076	hqer_induced sampler prototype	2.0
	front41Data	HQER-SFA	hqer_induced	True	53	1.188807419025133	hqer_induced sampler prototype	2.0
	front41Data	Quantile-SFA	hqer_induced	True	32	10.538251262087185	hqer_induced sampler prototype	2.0
	front41Data	HQER-SFA	hqer_induced	True	47	10.54449456814342	hqer_induced sampler prototype	4.0
	front41Data	Quantile-SFA	hqer_induced	True	42	2.948686323062669	hqer_induced sampler prototype	2.0
	front41Data	HQER-SFA	hqer_induced	True	29	2.958094378832253	hqer_induced sampler prototype	2.0
	front41Data	Quantile-SFA	hqer_induced	True	17	-14.914925601450191	hqer_induced sampler prototype	2.0
	front41Data	HQER-SFA	hqer_induced	True	11	-14.931909371585434	hqer_induced sampler prototype	2.0
	front41Data	Quantile-SFA	hqer_induced	True	16	0.284931138228247	hqer_induced sampler prototype	4.0
	front41Data	HQER-SFA	hqer_induced	True	54	0.3036620394654894	hqer_induced sampler prototype	2.0
	front41Data	Quantile-SFA	hqer_induced	True	19	8.70816041519994	hqer_induced sampler prototype	2.0
	front41Data	HQER-SFA	hqer_induced	True	16	8.756828381562828	hqer_induced sampler prototype	2.0
	front41Data	Quantile-SFA	hqer_induced	True	49	9.56509042206555	hqer_induced sampler prototype	1.0
	front41Data	HQER-SFA	hqer_induced	True	45	9.408778785721124	hqer_induced sampler prototype	2.0
	front41Data	Quantile-SFA	hqer_induced	True	23	-4.37090897060167	hqer_induced sampler prototype	2.0
	front41Data	HQER-SFA	hqer_induced	True	42	-3.810740029380786	hqer_induced sampler prototype	4.0
	front41Data	Quantile-SFA	hqer_induced	True	39	-10.705242426288834	hqer_induced sampler prototype	2.0
	front41Data	HQER-SFA	hqer_induced	True	35	-10.71056598087683	hqer_induced sampler prototype	4.0
	front41Data	Quantile-SFA	hqer_induced	True	21	6.485761454291558	hqer_induced sampler prototype	2.0
	front41Data	HQER-SFA	hqer_induced	True	24	6.469949019566753	hqer_induced sampler prototype	4.0
	front41Data	Quantile-SFA	hqer_induced	True	18	11.672571823841157	hqer_induced sampler prototype	2.0
	front41Data	HQER-SFA	hqer_induced	True	48	11.76431492704001	hqer_induced sampler prototype	4.0
	front41Data	Quantile-SFA	hqer_induced	True	29	5.109645588025382	hqer_induced sampler prototype	2.0
	front41Data	HQER-SFA	hqer_induced	True	31	5.103076395765579	hqer_induced sampler prototype	2.0
	front41Data	Quantile-SFA	hqer_induced	True	13	-2.607008880670504	hqer_induced sampler prototype	4.0
	front41Data	HQER-SFA	hqer_induced	True	24	-2.4037984453067014	hqer_induced sampler prototype	4.0
	front41Data	Quantile-SFA	hqer_induced	True	47	8.47794795292176	hqer_induced sampler prototype	4.0
	front41Data	HQER-SFA	hqer_induced	True	31	8.475027210863336	hqer_induced sampler prototype	4.0
	front41Data	Quantile-SFA	hqer_induced	True	22	9.143651316810818	hqer_induced sampler prototype	4.0
	front41Data	HQER-SFA	hqer_induced	True	36	9.206185289271296	hqer_induced sampler prototype	4.0
	front41Data	Quantile-SFA	hqer_induced	True	7	-6.538190452842997	hqer_induced sampler prototype	2.0
	front41Data	HQER-SFA	hqer_induced	True	6	-6.518067858860933	hqer_induced sampler prototype	2.0
	front41Data	Quantile-SFA	hqer_induced	True	39	6.887264922509908	hqer_induced sampler prototype	2.0
	front41Data	HQER-SFA	hqer_induced	True	37	6.8916739939409	hqer_induced sampler prototype	2.0
	front41Data	Quantile-SFA	hqer_induced	True	63	4.303044653622687	hqer_induced sampler prototype	4.0
	front41Data	HQER-SFA	hqer_induced	True	16	4.036238329899857	hqer_induced sampler prototype	4.0
	front41Data	Quantile-SFA	hqer_induced	True	7	-2.494940867270128	hqer_induced sampler prototype	2.0
	front41Data	HQER-SFA	hqer_induced	True	23	-2.347698379526037	hqer_induced sampler prototype	2.0
	front41Data	Quantile-SFA	hqer_induced	True	46	4.701312834237637	hqer_induced sampler prototype	4.0
	front41Data	HQER-SFA	hqer_induced	True	26	4.699681511849043	hqer_induced sampler prototype	4.0
	front41Data	Quantile-SFA	hqer_induced	True	34	3.215269061576157	hqer_induced sampler prototype	2.0
	front41Data	HQER-SFA	hqer_induced	True	63	3.2248762126193253	hqer_induced sampler prototype	4.0
	front41Data	Quantile-SFA	hqer_induced	True	35	-0.1582369303586697	hqer_induced sampler prototype	4.0
	front41Data	HQER-SFA	hqer_induced	True	35	-0.1638198377290528	hqer_induced sampler prototype	4.0
	front41Data	Quantile-SFA	hqer_induced	True	28	-1.0121058190674923	hqer_induced sampler prototype	4.0
	front41Data	HQER-SFA	hqer_induced	True	55	-0.9060945456367704	hqer_induced sampler prototype	2.0
	front41Data	Quantile-SFA	hqer_induced	True	44	2.5185335583618444	hqer_induced sampler prototype	4.0
	front41Data	HQER-SFA	hqer_induced	True	32	2.521099958439568	hqer_induced sampler prototype	4.0
	front41Data	Quantile-SFA	hqer_induced	True	35	-12.13680386790824	hqer_induced sampler prototype	2.0
	front41Data	HQER-SFA	hqer_induced	True	38	-12.135655509940642	hqer_induced sampler prototype	2.0
	front41Data	Quantile-SFA	hqer_induced	True	50	7.1371419923332375	hqer_induced sampler prototype	4.0
	front41Data	HQER-SFA	hqer_induced	True	36	7.148881994540562	hqer_induced sampler prototype	4.0
	front41Data	Quantile-SFA	hqer_induced	True	33	-1.6755029311190115	hqer_induced sampler prototype	2.0
	front41Data	HQER-SFA	hqer_induced	True	57	-1.5979126287230985	hqer_induced sampler prototype	4.0
	front41Data	Quantile-SFA	hqer_induced	True	34	-5.534440524359311	hqer_induced sampler prototype	4.0
	front41Data	HQER-SFA	hqer_induced	True	20	-5.5106774966966	hqer_induced sampler prototype	4.0
	front41Data	Quantile-SFA	hqer_induced	True	42	4.205295670064614	hqer_induced sampler prototype	2.0
	front41Data	HQER-SFA	hqer_induced	True	39	4.230612170925599	hqer_induced sampler prototype	4.0
	front41Data	Quantile-SFA	hqer_induced	True	16	-6.395118157031472	hqer_induced sampler prototype	2.0
	front41Data	HQER-SFA	hqer_induced	True	9	-6.517927080258358	hqer_induced sampler prototype	2.0
	front41Data	Quantile-SFA	hqer_induced	True	25	0.3939459703624632	hqer_induced sampler prototype	2.0
	front41Data	HQER-SFA	hqer_induced	True	15	0.4074615682678427	hqer_induced sampler prototype	2.0
	front41Data	Quantile-SFA	hqer_induced	True	10	-8.175176471747186	hqer_induced sampler prototype	2.0
	front41Data	HQER-SFA	hqer_induced	True	10	-8.170932002834778	hqer_induced sampler prototype	2.0
	front41Data	Quantile-SFA	hqer_induced	True	17	11.615644481816858	hqer_induced sampler prototype	2.0
	front41Data	HQER-SFA	hqer_induced	True	28	11.632953954539364	hqer_induced sampler prototype	2.0
	front41Data	Quantile-SFA	hqer_induced	True	56	6.587960016426439	hqer_induced sampler prototype	4.0
	front41Data	HQER-SFA	hqer_induced	True	19	5.975353276285137	hqer_induced sampler prototype	4.0
	front41Data	Quantile-SFA	hqer_induced	True	20	4.162009552066857	hqer_induced sampler prototype	4.0
	front41Data	HQER-SFA	hqer_induced	True	37	4.531142495257027	hqer_induced sampler prototype	4.0
	front41Data	Quantile-SFA	hqer_induced	True	48	-2.302105661263857	hqer_induced sampler prototype	2.0
	front41Data	HQER-SFA	hqer_induced	True	34	-1.7358499809890218	hqer_induced sampler prototype	4.0
	front41Data	Quantile-SFA	hqer_induced	True	38	-10.227121544908654	hqer_induced sampler prototype	4.0
	front41Data	HQER-SFA	hqer_induced	True	39	-10.215790931684136	hqer_induced sampler prototype	2.0
	front41Data	Quantile-SFA	hqer_induced	True	8	-6.285465706101211	hqer_induced sampler prototype	2.0
	front41Data	HQER-SFA	hqer_induced	True	11	-6.260298986480807	hqer_induced sampler prototype	2.0
	front41Data	Quantile-SFA	hqer_induced	True	22	8.575283473179361	hqer_induced sampler prototype	2.0
	front41Data	HQER-SFA	hqer_induced	True	40	8.622770133020504	hqer_induced sampler prototype	4.0
	front41Data	Quantile-SFA	hqer_induced	True	52	7.79048556975385	hqer_induced sampler prototype	1.0

experiment	dataset	model	scenario	converged	n__iter	loglik	warning__message	best__start__id
	front41Data	HQER-SFA	hqer__induced	True	37	7.77862162267485	hqer__induced sampler prototype	4.0
	front41Data	Quantile-SFA	hqer__induced	True	48	2.421487021699587	hqer__induced sampler prototype	3.0
	front41Data	HQER-SFA	hqer__induced	True	38	2.445000942654935	hqer__induced sampler prototype	2.0
	front41Data	Quantile-SFA	hqer__induced	True	25	1.3189428027807386	hqer__induced sampler prototype	4.0
	front41Data	HQER-SFA	hqer__induced	True	29	1.4259093607701256	hqer__induced sampler prototype	4.0
	front41Data	Quantile-SFA	hqer__induced	True	53	0.9639163763708803	hqer__induced sampler prototype	4.0
	front41Data	HQER-SFA	hqer__induced	True	33	1.0963375274320306	hqer__induced sampler prototype	4.0
	front41Data	Quantile-SFA	hqer__induced	True	38	52.70581237219591	hqer__induced sampler prototype	4.0
	front41Data	HQER-SFA	hqer__induced	True	36	52.84429652862772	hqer__induced sampler prototype	4.0
	front41Data	Quantile-SFA	hqer__induced	True	25	8.384173571889288	hqer__induced sampler prototype	4.0
	front41Data	HQER-SFA	hqer__induced	True	26	8.460603631656276	hqer__induced sampler prototype	4.0
	front41Data	Quantile-SFA	hqer__induced	True	29	-9.51643872384118	hqer__induced sampler prototype	2.0
	front41Data	HQER-SFA	hqer__induced	True	30	-9.375974526552742	hqer__induced sampler prototype	2.0
	front41Data	Quantile-SFA	hqer__induced	True	13	-50.25399593929456	hqer__induced sampler prototype	4.0
	front41Data	HQER-SFA	hqer__induced	True	27	-50.14180717186855	hqer__induced sampler prototype	2.0
	front41Data	Quantile-SFA	hqer__induced	True	30	24.00251155644145	hqer__induced sampler prototype	4.0
	front41Data	HQER-SFA	hqer__induced	True	29	24.086428648976074	hqer__induced sampler prototype	2.0
	front41Data	Quantile-SFA	hqer__induced	True	33	-2.261945427693287	hqer__induced sampler prototype	4.0
	front41Data	HQER-SFA	hqer__induced	True	28	-2.1201474096711355	hqer__induced sampler prototype	4.0
	front41Data	Quantile-SFA	hqer__induced	True	24	-12.675469609499142	hqer__induced sampler prototype	2.0
	front41Data	HQER-SFA	hqer__induced	True	27	-12.601600630604164	hqer__induced sampler prototype	2.0
	front41Data	Quantile-SFA	hqer__induced	True	28	-23.32621527162264	hqer__induced sampler prototype	4.0
	front41Data	HQER-SFA	hqer__induced	True	24	-23.1997606044089	hqer__induced sampler prototype	4.0
	front41Data	Quantile-SFA	hqer__induced	True	49	5.652149471687017	hqer__induced sampler prototype	4.0
	front41Data	HQER-SFA	hqer__induced	True	30	5.756256901352262	hqer__induced sampler prototype	4.0
	front41Data	Quantile-SFA	hqer__induced	True	22	37.03310990464499	hqer__induced sampler prototype	4.0
	front41Data	HQER-SFA	hqer__induced	True	34	37.12388267004167	hqer__induced sampler prototype	4.0
	front41Data	Quantile-SFA	hqer__induced	True	47	-17.163054671322648	hqer__induced sampler prototype	2.0
	front41Data	HQER-SFA	hqer__induced	True	91	-17.136698841687668	hqer__induced sampler prototype	2.0
	front41Data	Quantile-SFA	hqer__induced	True	17	-31.72860866773096	hqer__induced sampler prototype	4.0
	front41Data	HQER-SFA	hqer__induced	True	60	-31.610644328774768	hqer__induced sampler prototype	4.0
	front41Data	Quantile-SFA	hqer__induced	True	22	-0.0344276528818792	hqer__induced sampler prototype	2.0
	front41Data	HQER-SFA	hqer__induced	True	24	0.0690687786175772	hqer__induced sampler prototype	2.0
	front41Data	Quantile-SFA	hqer__induced	True	21	22.25474645048329	hqer__induced sampler prototype	4.0
	front41Data	HQER-SFA	hqer__induced	True	37	22.324534777578165	hqer__induced sampler prototype	4.0
	front41Data	Quantile-SFA	hqer__induced	True	26	-8.626219847422167	hqer__induced sampler prototype	2.0
	front41Data	HQER-SFA	hqer__induced	True	17	-8.565585006830096	hqer__induced sampler prototype	4.0
	front41Data	Quantile-SFA	hqer__induced	True	36	25.85501160650177	hqer__induced sampler prototype	2.0
	front41Data	HQER-SFA	hqer__induced	True	22	25.96798071165761	hqer__induced sampler prototype	4.0
	front41Data	Quantile-SFA	hqer__induced	True	22	6.243120301828845	hqer__induced sampler prototype	4.0
	front41Data	HQER-SFA	hqer__induced	True	31	6.356502280406302	hqer__induced sampler prototype	4.0
	front41Data	Quantile-SFA	hqer__induced	True	33	14.563724023926454	hqer__induced sampler prototype	4.0
	front41Data	HQER-SFA	hqer__induced	True	32	14.654490022615493	hqer__induced sampler prototype	4.0
	front41Data	Quantile-SFA	hqer__induced	True	26	-6.522968225134283	hqer__induced sampler prototype	4.0
	front41Data	HQER-SFA	hqer__induced	True	33	-6.365389351802572	hqer__induced sampler prototype	2.0
	front41Data	Quantile-SFA	hqer__induced	True	28	-1.753870640220371	hqer__induced sampler prototype	2.0
	front41Data	HQER-SFA	hqer__induced	True	25	-1.635447610629107	hqer__induced sampler prototype	2.0
	front41Data	Quantile-SFA	hqer__induced	True	30	-26.20915135564165	hqer__induced sampler prototype	2.0
	front41Data	HQER-SFA	hqer__induced	True	34	-26.12916520638678	hqer__induced sampler prototype	2.0
	front41Data	Quantile-SFA	hqer__induced	True	31	31.159415850940466	hqer__induced sampler prototype	4.0
	front41Data	HQER-SFA	hqer__induced	True	40	31.131032775803607	hqer__induced sampler prototype	2.0
	front41Data	Quantile-SFA	hqer__induced	True	31	35.38599034800382	hqer__induced sampler prototype	4.0
	front41Data	HQER-SFA	hqer__induced	True	48	35.4830889728593	hqer__induced sampler prototype	5.0
	front41Data	Quantile-SFA	hqer__induced	True	30	15.160554999244498	hqer__induced sampler prototype	4.0
	front41Data	HQER-SFA	hqer__induced	True	25	14.964008246627635	hqer__induced sampler prototype	2.0
	front41Data	Quantile-SFA	hqer__induced	True	51	-0.7167136805170706	hqer__induced sampler prototype	2.0
	front41Data	HQER-SFA	hqer__induced	True	72	-0.6974823404160189	hqer__induced sampler prototype	2.0
	front41Data	Quantile-SFA	hqer__induced	True	29	-32.333337961135214	hqer__induced sampler prototype	4.0
	front41Data	HQER-SFA	hqer__induced	True	31	-32.23683122294268	hqer__induced sampler prototype	2.0
	front41Data	Quantile-SFA	hqer__induced	True	36	18.16716895203107	hqer__induced sampler prototype	4.0
	front41Data	HQER-SFA	hqer__induced	True	40	18.25968894059917	hqer__induced sampler prototype	5.0
	front41Data	Quantile-SFA	hqer__induced	True	28	17.28541403935938	hqer__induced sampler prototype	2.0
	front41Data	HQER-SFA	hqer__induced	True	27	17.36493969971058	hqer__induced sampler prototype	2.0
	front41Data	Quantile-SFA	hqer__induced	True	49	1.6419380724571615	hqer__induced sampler prototype	4.0
	front41Data	HQER-SFA	hqer__induced	True	36	1.7536144273833203	hqer__induced sampler prototype	4.0
	front41Data	Quantile-SFA	hqer__induced	True	22	43.87679239425903	hqer__induced sampler prototype	2.0
	front41Data	HQER-SFA	hqer__induced	True	30	43.99810093615464	hqer__induced sampler prototype	4.0
	front41Data	Quantile-SFA	hqer__induced	True	23	29.641438709892	hqer__induced sampler prototype	2.0
	front41Data	HQER-SFA	hqer__induced	True	25	29.721215151856786	hqer__induced sampler prototype	4.0
	front41Data	Quantile-SFA	hqer__induced	True	21	-22.24353965151189	hqer__induced sampler prototype	2.0
	front41Data	HQER-SFA	hqer__induced	True	19	-22.136966106928668	hqer__induced sampler prototype	4.0
	front41Data	Quantile-SFA	hqer__induced	True	47	-30.87606993358781	hqer__induced sampler prototype	4.0
	front41Data	HQER-SFA	hqer__induced	True	54	-30.75968701691039	hqer__induced sampler prototype	4.0
	front41Data	Quantile-SFA	hqer__induced	True	25	-1.363475911540066	hqer__induced sampler prototype	2.0
	front41Data	HQER-SFA	hqer__induced	True	39	-1.257695325193389	hqer__induced sampler prototype	2.0
	front41Data	Quantile-SFA	hqer__induced	True	47	-1.6123083591807983	hqer__induced sampler prototype	2.0
	front41Data	HQER-SFA	hqer__induced	True	24	-1.535059352873703	hqer__induced sampler prototype	4.0
	front41Data	Quantile-SFA	hqer__induced	True	26	-5.352327297952877	hqer__induced sampler prototype	2.0
	front41Data	HQER-SFA	hqer__induced	True	30	-5.288706772728685	hqer__induced sampler prototype	2.0
	front41Data	Quantile-SFA	hqer__induced	True	21	-9.966305200275215	hqer__induced sampler prototype	2.0
	front41Data	HQER-SFA	hqer__induced	True	34	-9.862427866075972	hqer__induced sampler prototype	2.0
	front41Data	Quantile-SFA	hqer__induced	True	24	33.07180864205019	hqer__induced sampler prototype	2.0
	front41Data	HQER-SFA	hqer__induced	True	15	33.12366629444887	hqer__induced sampler prototype	2.0
	front41Data	Quantile-SFA	hqer__induced	True	37	-1.402044201818851	hqer__induced sampler prototype	4.0
	front41Data	HQER-SFA	hqer__induced	True	33	-1.289759994493246	hqer__induced sampler prototype	4.0
	front41Data	Quantile-SFA	hqer__induced	True	39	-0.7459964362546359	hqer__induced sampler prototype	4.0
	front41Data	HQER-SFA	hqer__induced	True	31	-0.6259151701100709	hqer__induced sampler prototype	4.0
	front41Data	Quantile-SFA	hqer__induced	True	21	17.537928681926306	hqer__induced sampler prototype	2.0
	front41Data	HQER-SFA	hqer__induced	True	26	17.607981220958017	hqer__induced sampler prototype	2.0
	front41Data	Quantile-SFA	hqer__induced	True	33	-12.981359163900237	hqer__induced sampler prototype	4.0
	front41Data	HQER-SFA	hqer__induced	True	24	-12.844009237226516	hqer__induced sampler prototype	4.0
	front41Data	Quantile-SFA	hqer__induced	True	39	-2.871714446189418	hqer__induced sampler prototype	4.0
	front41Data	HQER-SFA	hqer__induced	True	33	-2.7825040373432	hqer__induced sampler prototype	4.0
	front41Data	Quantile-SFA	hqer__induced	True	23	-9.45239816096352	hqer__induced sampler prototype	2.0
	front41Data	HQER-SFA	hqer__induced	True	37	-9.346311666155035	hqer__induced sampler prototype	4.0
	front41Data	Quantile-SFA	hqer__induced	True	43	26.26031905031505	hqer__induced sampler prototype	4.0
	front41Data	HQER-SFA	hqer__induced	True	25	26.34652136819157	hqer__induced sampler prototype	2.0
	front41Data	Quantile-SFA	hqer__induced	True	11	5.464822133145196	hqer__induced sampler prototype	2.0
	front41Data	HQER-SFA	hqer__induced	True	22	5.579326294796236	hqer__induced sampler prototype	4.0
	front41Data	Quantile-SFA	hqer__induced	True	24	-16.173546026153474	hqer__induced sampler prototype	2.0
	front41Data	HQER-SFA	hqer__induced	True	28	-16.050700867385252	hqer__induced sampler prototype	2.0
	front41Data	Quantile-SFA	hqer__induced	True	33	-10.949486887119956	hqer__induced sampler prototype	2.0
	front41Data	HQER-SFA	hqer__induced	True	38	-10.807663034669115	hqer__induced sampler prototype	2.0

experiment	dataset	model	scenario	converged	n_iter	loglik	warning_message	best_start_id
	front41Data	Quantile-SFA	hqer_induced	True	27	-37.555055957376126	hqer_induced sampler prototype	4.0
	front41Data	HQER-SFA	hqer_induced	True	31	-37.44428853036696	hqer_induced sampler prototype	4.0
	front41Data	Quantile-SFA	hqer_induced	True	33	-2.313178887709153	hqer_induced sampler prototype	4.0
	front41Data	HQER-SFA	hqer_induced	True	21	-2.284690421427974	hqer_induced sampler prototype	4.0
	front41Data	Quantile-SFA	hqer_induced	True	26	14.08178981579112	hqer_induced sampler prototype	2.0
	front41Data	HQER-SFA	hqer_induced	True	28	14.165013413592815	hqer_induced sampler prototype	2.0
	front41Data	Quantile-SFA	hqer_induced	True	31	-8.722335264303284	hqer_induced sampler prototype	4.0
	front41Data	HQER-SFA	hqer_induced	True	34	-8.61010224369249	hqer_induced sampler prototype	2.0
	front41Data	Quantile-SFA	hqer_induced	True	24	1.5236323807817935	hqer_induced sampler prototype	4.0
	front41Data	HQER-SFA	hqer_induced	True	29	1.6170598098551032	hqer_induced sampler prototype	2.0
	front41Data	Quantile-SFA	hqer_induced	True	22	6.71856331140931	hqer_induced sampler prototype	4.0
	front41Data	HQER-SFA	hqer_induced	True	19	6.79335514750583	hqer_induced sampler prototype	4.0
	front41Data	Quantile-SFA	hqer_induced	True	35	24.35921752959068	hqer_induced sampler prototype	4.0
	front41Data	HQER-SFA	hqer_induced	True	32	24.446889305442948	hqer_induced sampler prototype	4.0
	front41Data	Quantile-SFA	hqer_induced	True	32	-0.4738851727992248	hqer_induced sampler prototype	2.0
	front41Data	HQER-SFA	hqer_induced	True	30	-0.2679470134893744	hqer_induced sampler prototype	2.0
	front41Data	Quantile-SFA	hqer_induced	True	47	-2.505685263226754	hqer_induced sampler prototype	2.0
	front41Data	HQER-SFA	hqer_induced	True	35	-2.412255671961862	hqer_induced sampler prototype	2.0
	front41Data	Quantile-SFA	hqer_induced	True	33	7.323505048562416	hqer_induced sampler prototype	4.0
	front41Data	HQER-SFA	hqer_induced	True	33	7.416724770633085	hqer_induced sampler prototype	4.0
	front41Data	Quantile-SFA	hqer_induced	True	28	13.070800466586284	hqer_induced sampler prototype	4.0
	front41Data	HQER-SFA	hqer_induced	True	37	13.204134692418748	hqer_induced sampler prototype	4.0
	front41Data	Quantile-SFA	hqer_induced	True	22	21.65601272785161	hqer_induced sampler prototype	4.0
	front41Data	HQER-SFA	hqer_induced	True	22	21.754616180725243	hqer_induced sampler prototype	4.0
	front41Data	Quantile-SFA	hqer_induced	True	24	3.3744422537912886	hqer_induced sampler prototype	2.0
	front41Data	HQER-SFA	hqer_induced	True	37	3.598927108778793	hqer_induced sampler prototype	2.0
	front41Data	Quantile-SFA	hqer_induced	True	28	-8.670339818791053	hqer_induced sampler prototype	4.0
	front41Data	HQER-SFA	hqer_induced	True	47	-8.396662923328998	hqer_induced sampler prototype	2.0
	front41Data	Quantile-SFA	hqer_induced	True	37	-4.753204480525193	hqer_induced sampler prototype	4.0
	front41Data	HQER-SFA	hqer_induced	True	41	-4.7598080954958455	hqer_induced sampler prototype	4.0
	front41Data	Quantile-SFA	hqer_induced	True	38	17.62761058432649	hqer_induced sampler prototype	2.0
	front41Data	HQER-SFA	hqer_induced	True	28	17.673648637978282	hqer_induced sampler prototype	2.0
	front41Data	Quantile-SFA	hqer_induced	True	19	-22.74077082911456	hqer_induced sampler prototype	4.0
	front41Data	HQER-SFA	hqer_induced	True	29	-22.698715698098336	hqer_induced sampler prototype	2.0
	front41Data	Quantile-SFA	hqer_induced	True	41	12.639400432837755	hqer_induced sampler prototype	2.0
	front41Data	HQER-SFA	hqer_induced	True	52	12.699117970585636	hqer_induced sampler prototype	2.0
	front41Data	Quantile-SFA	hqer_induced	True	46	1.1224818605621605	hqer_induced sampler prototype	4.0
	front41Data	HQER-SFA	hqer_induced	True	34	1.2685295625225923	hqer_induced sampler prototype	4.0
	front41Data	Quantile-SFA	hqer_induced	True	32	-14.988372570437887	hqer_induced sampler prototype	4.0
	front41Data	HQER-SFA	hqer_induced	True	29	-14.879959722262717	hqer_induced sampler prototype	4.0
	front41Data	Quantile-SFA	hqer_induced	True	15	19.5256656413546	hqer_induced sampler prototype	4.0
	front41Data	HQER-SFA	hqer_induced	True	15	19.5909460038392	hqer_induced sampler prototype	4.0
	front41Data	Quantile-SFA	hqer_induced	True	34	34.26810988434576	hqer_induced sampler prototype	2.0
	front41Data	HQER-SFA	hqer_induced	True	34	34.3769366468118	hqer_induced sampler prototype	2.0
	front41Data	Quantile-SFA	hqer_induced	True	15	11.081943964546744	hqer_induced sampler prototype	2.0
	front41Data	HQER-SFA	hqer_induced	True	36	11.201817472540624	hqer_induced sampler prototype	2.0
	front41Data	Quantile-SFA	hqer_induced	True	36	29.54716968849437	hqer_induced sampler prototype	4.0
	front41Data	HQER-SFA	hqer_induced	True	49	29.64133713026229	hqer_induced sampler prototype	4.0
	front41Data	Quantile-SFA	hqer_induced	True	26	2.0498255527987963	hqer_induced sampler prototype	4.0
	front41Data	HQER-SFA	hqer_induced	True	37	2.158424180406919	hqer_induced sampler prototype	4.0
	front41Data	Quantile-SFA	hqer_induced	True	30	30.32735204530182	hqer_induced sampler prototype	2.0
	front41Data	HQER-SFA	hqer_induced	True	24	30.38572441851513	hqer_induced sampler prototype	2.0
	front41Data	Quantile-SFA	hqer_induced	True	25	-6.950778137147564	hqer_induced sampler prototype	4.0
	front41Data	HQER-SFA	hqer_induced	True	31	-6.867655716865006	hqer_induced sampler prototype	4.0
	front41Data	Quantile-SFA	hqer_induced	True	28	22.70683673956802	hqer_induced sampler prototype	2.0
	front41Data	HQER-SFA	hqer_induced	True	22	22.75653269468379	hqer_induced sampler prototype	2.0
	front41Data	Quantile-SFA	hqer_induced	True	38	7.065225461093731	hqer_induced sampler prototype	2.0
	front41Data	HQER-SFA	hqer_induced	True	33	7.169528135312475	hqer_induced sampler prototype	4.0
	front41Data	Quantile-SFA	hqer_induced	True	18	10.341871399626822	hqer_induced sampler prototype	2.0
	front41Data	HQER-SFA	hqer_induced	True	16	10.429603544967376	hqer_induced sampler prototype	4.0
	front41Data	Quantile-SFA	hqer_induced	True	26	-22.69720833485723	hqer_induced sampler prototype	2.0
	front41Data	HQER-SFA	hqer_induced	True	32	-22.607280545938927	hqer_induced sampler prototype	2.0
	front41Data	Quantile-SFA	hqer_induced	True	22	32.776826986842266	hqer_induced sampler prototype	2.0
	front41Data	HQER-SFA	hqer_induced	True	31	32.828897206641656	hqer_induced sampler prototype	2.0
	front41Data	Quantile-SFA	hqer_induced	True	30	-3.924176067717304	hqer_induced sampler prototype	4.0
	front41Data	HQER-SFA	hqer_induced	True	24	-3.7958217036027633	hqer_induced sampler prototype	2.0
	front41Data	Quantile-SFA	hqer_induced	True	30	12.619995994665686	hqer_induced sampler prototype	2.0
	front41Data	HQER-SFA	hqer_induced	True	25	12.765326621245569	hqer_induced sampler prototype	4.0
	front41Data	Quantile-SFA	hqer_induced	True	23	15.55644060171189	hqer_induced sampler prototype	4.0
	front41Data	HQER-SFA	hqer_induced	True	27	15.619243944875896	hqer_induced sampler prototype	4.0
	front41Data	Quantile-SFA	hqer_induced	True	16	-2.509803682341428	hqer_induced sampler prototype	4.0
	front41Data	HQER-SFA	hqer_induced	True	13	-2.4131784104383662	hqer_induced sampler prototype	4.0
	front41Data	Quantile-SFA	hqer_induced	True	18	-16.09431370330959	hqer_induced sampler prototype	4.0
	front41Data	HQER-SFA	hqer_induced	True	20	-15.943422468570663	hqer_induced sampler prototype	4.0
	front41Data	Quantile-SFA	hqer_induced	True	26	6.4362985390913865	hqer_induced sampler prototype	4.0
	front41Data	HQER-SFA	hqer_induced	True	26	6.567442369571467	hqer_induced sampler prototype	4.0
	front41Data	Quantile-SFA	hqer_induced	True	38	1.8651453475655124	hqer_induced sampler prototype	4.0
	front41Data	HQER-SFA	hqer_induced	True	33	1.9680389852736613	hqer_induced sampler prototype	4.0
	front41Data	Quantile-SFA	hqer_induced	True	46	-17.662532835408626	hqer_induced sampler prototype	4.0
	front41Data	HQER-SFA	hqer_induced	True	28	-17.543871489300066	hqer_induced sampler prototype	4.0
	front41Data	Quantile-SFA	hqer_induced	True	52	-18.03289200658945	hqer_induced sampler prototype	5.0
	front41Data	HQER-SFA	hqer_induced	True	47	-17.951062020260128	hqer_induced sampler prototype	4.0
	front41Data	Quantile-SFA	hqer_induced	True	31	33.29958160916539	hqer_induced sampler prototype	4.0
	front41Data	HQER-SFA	hqer_induced	True	26	33.40071828489442	hqer_induced sampler prototype	4.0
	front41Data	Quantile-SFA	hqer_induced	True	37	9.264681477965071	hqer_induced sampler prototype	2.0
	front41Data	HQER-SFA	hqer_induced	True	23	9.367816078523616	hqer_induced sampler prototype	4.0
	front41Data	Quantile-SFA	hqer_induced	True	21	32.00783326036429	hqer_induced sampler prototype	2.0
	front41Data	HQER-SFA	hqer_induced	True	20	32.12242817067249	hqer_induced sampler prototype	2.0
	front41Data	Quantile-SFA	hqer_induced	True	15	-0.4775422389591512	hqer_induced sampler prototype	4.0
	front41Data	HQER-SFA	hqer_induced	True	51	-0.3767345809793219	hqer_induced sampler prototype	5.0
	front41Data	Quantile-SFA	hqer_induced	True	40	-2.311977601513909	hqer_induced sampler prototype	2.0
	front41Data	HQER-SFA	hqer_induced	True	25	-2.232292929756918	hqer_induced sampler prototype	2.0
	front41Data	Quantile-SFA	hqer_induced	True	21	-13.513843257049848	hqer_induced sampler prototype	2.0
	front41Data	HQER-SFA	hqer_induced	True	15	-13.382745990147402	hqer_induced sampler prototype	2.0
	front41Data	Quantile-SFA	hqer_induced	True	26	-7.609013105944583	hqer_induced sampler prototype	4.0
	front41Data	HQER-SFA	hqer_induced	True	34	-7.6184920092340676	hqer_induced sampler prototype	2.0
	front41Data	Quantile-SFA	hqer_induced	True	20	-3.0698959860551307	hqer_induced sampler prototype	4.0
	front41Data	HQER-SFA	hqer_induced	True	23	-2.943087907085941	hqer_induced sampler prototype	4.0
	front41Data	Quantile-SFA	hqer_induced	True	36	20.06513044615363	hqer_induced sampler prototype	4.0
	front41Data	HQER-SFA	hqer_induced	True	27	20.10598081676154	hqer_induced sampler prototype	2.0
	front41Data	Quantile-SFA	hqer_induced	True	45	-7.987905032051501	hqer_induced sampler prototype	4.0
	front41Data	HQER-SFA	hqer_induced	True	34	-5.944472252489408	hqer_induced sampler prototype	4.0
	front41Data	Quantile-SFA	hqer_induced	True	32	-1.0291772973739537	hqer_induced sampler prototype	2.0

experiment	dataset	model	scenario	converged	n__iter	loglik	warning__message	best__start__id
	front41Data	HQER-SFA	hqer__induced	True	40	0.5129696186894914	hqer__induced sampler prototype	2.0
	front41Data	Quantile-SFA	hqer__induced	True	14	-9.354170310027364	hqer__induced sampler prototype	2.0
	front41Data	HQER-SFA	hqer__induced	True	50	-5.568000939641875	hqer__induced sampler prototype	2.0
	front41Data	Quantile-SFA	hqer__induced	True	24	-7.660430238700328	hqer__induced sampler prototype	4.0
	front41Data	HQER-SFA	hqer__induced	True	38	-9.24159384279173	hqer__induced sampler prototype	4.0
	front41Data	Quantile-SFA	hqer__induced	True	50	-5.772812797555748	hqer__induced sampler prototype	4.0
	front41Data	HQER-SFA	hqer__induced	True	28	-3.535391694005339	hqer__induced sampler prototype	4.0
	front41Data	Quantile-SFA	hqer__induced	True	20	-2.302708399759424	hqer__induced sampler prototype	4.0
	front41Data	HQER-SFA	hqer__induced	True	36	-1.5320001271582115	hqer__induced sampler prototype	4.0
	front41Data	Quantile-SFA	hqer__induced	True	19	-3.8327799551410418	hqer__induced sampler prototype	2.0
	front41Data	HQER-SFA	hqer__induced	True	32	-0.71689465091519	hqer__induced sampler prototype	2.0
	front41Data	Quantile-SFA	hqer__induced	True	13	8.722104341702613	hqer__induced sampler prototype	2.0
	front41Data	HQER-SFA	hqer__induced	True	66	8.385321247442763	hqer__induced sampler prototype	2.0
	front41Data	Quantile-SFA	hqer__induced	True	22	-12.102135557891918	hqer__induced sampler prototype	2.0
	front41Data	HQER-SFA	hqer__induced	True	20	-5.049400753929139	hqer__induced sampler prototype	4.0
	front41Data	Quantile-SFA	hqer__induced	True	16	-7.520815724638856	hqer__induced sampler prototype	2.0
	front41Data	HQER-SFA	hqer__induced	True	28	-4.650791817857211	hqer__induced sampler prototype	4.0
	front41Data	Quantile-SFA	hqer__induced	True	18	-7.869463013974833	hqer__induced sampler prototype	4.0
	front41Data	HQER-SFA	hqer__induced	True	22	-6.0155543536757	hqer__induced sampler prototype	4.0
	front41Data	Quantile-SFA	hqer__induced	True	24	-2.7535072842634585	hqer__induced sampler prototype	2.0
	front41Data	HQER-SFA	hqer__induced	True	38	0.528187040588421	hqer__induced sampler prototype	4.0
	front41Data	Quantile-SFA	hqer__induced	True	17	-6.248959063915398	hqer__induced sampler prototype	4.0
	front41Data	HQER-SFA	hqer__induced	True	15	-4.861067265392708	hqer__induced sampler prototype	2.0
	front41Data	Quantile-SFA	hqer__induced	True	32	2.955254587509879	hqer__induced sampler prototype	2.0
	front41Data	HQER-SFA	hqer__induced	True	50	2.684157271641263	hqer__induced sampler prototype	4.0
	front41Data	Quantile-SFA	hqer__induced	True	14	-11.167989340897416	hqer__induced sampler prototype	4.0
	front41Data	HQER-SFA	hqer__induced	True	27	-8.193785629516357	hqer__induced sampler prototype	2.0
	front41Data	Quantile-SFA	hqer__induced	True	58	4.19427288409252	hqer__induced sampler prototype	3.0
	front41Data	HQER-SFA	hqer__induced	True	24	7.114688560650444	hqer__induced sampler prototype	2.0
	front41Data	Quantile-SFA	hqer__induced	True	23	-3.1442807986761947	hqer__induced sampler prototype	4.0
	front41Data	HQER-SFA	hqer__induced	True	54	-3.5127875135482096	hqer__induced sampler prototype	4.0
	front41Data	Quantile-SFA	hqer__induced	True	20	-7.126765160405128	hqer__induced sampler prototype	2.0
	front41Data	HQER-SFA	hqer__induced	True	62	-7.262537005591465	hqer__induced sampler prototype	2.0
	front41Data	Quantile-SFA	hqer__induced	True	17	-7.417447586120415	hqer__induced sampler prototype	2.0
	front41Data	HQER-SFA	hqer__induced	True	44	-6.986377422041788	hqer__induced sampler prototype	2.0
	front41Data	Quantile-SFA	hqer__induced	True	21	-6.553140247459108	hqer__induced sampler prototype	4.0
	front41Data	HQER-SFA	hqer__induced	True	27	-4.078159368835473	hqer__induced sampler prototype	4.0
	front41Data	Quantile-SFA	hqer__induced	True	32	-2.9135215231038645	hqer__induced sampler prototype	4.0
	front41Data	HQER-SFA	hqer__induced	True	14	-1.054201926456116	hqer__induced sampler prototype	4.0
	front41Data	Quantile-SFA	hqer__induced	True	11	6.289633075493725	hqer__induced sampler prototype	2.0
	front41Data	HQER-SFA	hqer__induced	True	36	7.42225254692729	hqer__induced sampler prototype	2.0
	front41Data	Quantile-SFA	hqer__induced	True	23	5.661036389337396	hqer__induced sampler prototype	4.0
	front41Data	HQER-SFA	hqer__induced	True	36	5.833029089354501	hqer__induced sampler prototype	2.0
	front41Data	Quantile-SFA	hqer__induced	True	12	-3.150808805137361	hqer__induced sampler prototype	2.0
	front41Data	HQER-SFA	hqer__induced	True	28	-0.4789800284472238	hqer__induced sampler prototype	4.0
	front41Data	Quantile-SFA	hqer__induced	True	16	-5.910685445928658	hqer__induced sampler prototype	2.0
	front41Data	HQER-SFA	hqer__induced	True	40	-4.789834872084421	hqer__induced sampler prototype	2.0
	front41Data	Quantile-SFA	hqer__induced	True	16	-15.951032092162285	hqer__induced sampler prototype	2.0
	front41Data	HQER-SFA	hqer__induced	True	55	-13.299218462568534	hqer__induced sampler prototype	2.0
	front41Data	Quantile-SFA	hqer__induced	True	76	0.1046789420024693	hqer__induced sampler prototype	4.0
	front41Data	HQER-SFA	hqer__induced	True	31	-0.4864627348645053	hqer__induced sampler prototype	2.0
	front41Data	Quantile-SFA	hqer__induced	True	13	-6.567583132936915	hqer__induced sampler prototype	2.0
	front41Data	HQER-SFA	hqer__induced	True	21	-5.9042239300879	hqer__induced sampler prototype	4.0
	front41Data	Quantile-SFA	hqer__induced	True	15	-6.225315577632777	hqer__induced sampler prototype	2.0
	front41Data	HQER-SFA	hqer__induced	True	47	-4.714308089379498	hqer__induced sampler prototype	2.0
	front41Data	Quantile-SFA	hqer__induced	True	12	-11.096532799142818	hqer__induced sampler prototype	4.0
	front41Data	HQER-SFA	hqer__induced	True	56	-8.2502604818528	hqer__induced sampler prototype	4.0
	front41Data	Quantile-SFA	hqer__induced	True	14	-13.132746474486323	hqer__induced sampler prototype	4.0
	front41Data	HQER-SFA	hqer__induced	True	65	-12.686646344024416	hqer__induced sampler prototype	4.0
	front41Data	Quantile-SFA	hqer__induced	True	25	-1.530128810648754	hqer__induced sampler prototype	2.0
	front41Data	HQER-SFA	hqer__induced	True	29	-0.4696091294268326	hqer__induced sampler prototype	4.0
	front41Data	Quantile-SFA	hqer__induced	True	22	-16.762592977058876	hqer__induced sampler prototype	2.0
	front41Data	HQER-SFA	hqer__induced	True	60	-11.195130692549032	hqer__induced sampler prototype	4.0
	front41Data	Quantile-SFA	hqer__induced	True	13	-2.993047811292346	hqer__induced sampler prototype	2.0
	front41Data	HQER-SFA	hqer__induced	True	6	-2.394072520429809	hqer__induced sampler prototype	2.0
	front41Data	Quantile-SFA	hqer__induced	True	13	-13.466773167703492	hqer__induced sampler prototype	4.0
	front41Data	HQER-SFA	hqer__induced	True	10	-11.447150998307013	hqer__induced sampler prototype	2.0
	front41Data	Quantile-SFA	hqer__induced	True	44	2.50019936503156	hqer__induced sampler prototype	2.0
	front41Data	HQER-SFA	hqer__induced	True	33	4.060405585777442	hqer__induced sampler prototype	4.0
	front41Data	Quantile-SFA	hqer__induced	True	44	1.6317834788542949	hqer__induced sampler prototype	2.0
	front41Data	HQER-SFA	hqer__induced	True	25	1.214850517933866	hqer__induced sampler prototype	4.0
	front41Data	Quantile-SFA	hqer__induced	True	17	-1.6669836754419265	hqer__induced sampler prototype	4.0
	front41Data	HQER-SFA	hqer__induced	True	49	0.0579803561325996	hqer__induced sampler prototype	4.0
	front41Data	Quantile-SFA	hqer__induced	True	33	-2.0984087240073093	hqer__induced sampler prototype	4.0
	front41Data	HQER-SFA	hqer__induced	True	28	-0.0398855372814067	hqer__induced sampler prototype	3.0
	front41Data	Quantile-SFA	hqer__induced	True	25	2.9380500378302923	hqer__induced sampler prototype	2.0
	front41Data	HQER-SFA	hqer__induced	True	61	8.450468743082155	hqer__induced sampler prototype	4.0
	front41Data	Quantile-SFA	hqer__induced	True	29	-13.96213091943072	hqer__induced sampler prototype	2.0
	front41Data	HQER-SFA	hqer__induced	True	56	-10.362885473912344	hqer__induced sampler prototype	4.0
	front41Data	Quantile-SFA	hqer__induced	True	39	2.368035677331936	hqer__induced sampler prototype	4.0
	front41Data	HQER-SFA	hqer__induced	True	63	3.1309959648549577	hqer__induced sampler prototype	4.0
	front41Data	Quantile-SFA	hqer__induced	True	28	11.335464927555584	hqer__induced sampler prototype	4.0
	front41Data	HQER-SFA	hqer__induced	True	39	10.208139402702864	hqer__induced sampler prototype	1.0
	front41Data	Quantile-SFA	hqer__induced	True	36	3.39010518939371	hqer__induced sampler prototype	2.0
	front41Data	HQER-SFA	hqer__induced	True	68	4.700808911804935	hqer__induced sampler prototype	2.0
	front41Data	Quantile-SFA	hqer__induced	True	20	2.220137261513252	hqer__induced sampler prototype	2.0
	front41Data	HQER-SFA	hqer__induced	True	35	3.780535744787272	hqer__induced sampler prototype	4.0
	front41Data	Quantile-SFA	hqer__induced	True	27	6.824273800801118	hqer__induced sampler prototype	4.0
	front41Data	HQER-SFA	hqer__induced	True	73	6.690705882848127	hqer__induced sampler prototype	2.0
	front41Data	Quantile-SFA	hqer__induced	True	35	6.353232724570861	hqer__induced sampler prototype	2.0
	front41Data	HQER-SFA	hqer__induced	True	73	6.510551128607985	hqer__induced sampler prototype	2.0
	front41Data	Quantile-SFA	hqer__induced	True	19	2.659505423385269	hqer__induced sampler prototype	4.0
	front41Data	HQER-SFA	hqer__induced	True	33	1.9114452621490143	hqer__induced sampler prototype	2.0
	front41Data	Quantile-SFA	hqer__induced	True	36	-1.97405518548152	hqer__induced sampler prototype	4.0
	front41Data	HQER-SFA	hqer__induced	True	49	0.1846654560782901	hqer__induced sampler prototype	4.0
	front41Data	Quantile-SFA	hqer__induced	True	47	-5.5028924861386965	hqer__induced sampler prototype	4.0
	front41Data	HQER-SFA	hqer__induced	True	37	-5.073747742490575	hqer__induced sampler prototype	4.0
	front41Data	Quantile-SFA	hqer__induced	True	15	3.056743197216284	hqer__induced sampler prototype	4.0
	front41Data	HQER-SFA	hqer__induced	True	45	2.931320936759905	hqer__induced sampler prototype	2.0
	front41Data	Quantile-SFA	hqer__induced	True	48	3.60472357627427	hqer__induced sampler prototype	4.0
	front41Data	HQER-SFA	hqer__induced	True	39	5.136311481683042	hqer__induced sampler prototype	4.0
	front41Data	Quantile-SFA	hqer__induced	True	61	0.4717555470684858	hqer__induced sampler prototype	2.0
	front41Data	HQER-SFA	hqer__induced	True	37	1.5397682808321482	hqer__induced sampler prototype	4.0

experiment	dataset	model	scenario	converged	n_iter	loglik	warning_message	best_start_id
	front41Data	Quantile-SFA	hqer_induced	True	35	-2.9156315291497963	hqer_induced sampler prototype	4.0
	front41Data	HQER-SFA	hqer_induced	True	24	-1.2446943452991364	hqer_induced sampler prototype	2.0
	front41Data	Quantile-SFA	hqer_induced	True	27	-14.580820724805871	hqer_induced sampler prototype	4.0
	front41Data	HQER-SFA	hqer_induced	True	35	-11.702206010060172	hqer_induced sampler prototype	4.0
	front41Data	Quantile-SFA	hqer_induced	True	29	3.6332959407426193	hqer_induced sampler prototype	2.0
	front41Data	HQER-SFA	hqer_induced	True	15	2.8957578811630578	hqer_induced sampler prototype	2.0
	front41Data	Quantile-SFA	hqer_induced	True	35	-0.5665064570794152	hqer_induced sampler prototype	4.0
	front41Data	HQER-SFA	hqer_induced	True	36	1.4301638772824845	hqer_induced sampler prototype	2.0
	front41Data	Quantile-SFA	hqer_induced	True	34	-4.916313743204264	hqer_induced sampler prototype	2.0
	front41Data	HQER-SFA	hqer_induced	True	45	-2.520876618034462	hqer_induced sampler prototype	4.0
	front41Data	Quantile-SFA	hqer_induced	True	38	2.9424765726312048	hqer_induced sampler prototype	4.0
	front41Data	HQER-SFA	hqer_induced	True	66	3.483216086712708	hqer_induced sampler prototype	4.0
	front41Data	Quantile-SFA	hqer_induced	True	24	-2.1860389298782827	hqer_induced sampler prototype	4.0
	front41Data	HQER-SFA	hqer_induced	True	34	-0.8044394162165143	hqer_induced sampler prototype	4.0
	front41Data	Quantile-SFA	hqer_induced	True	15	-3.214357277000129	hqer_induced sampler prototype	4.0
	front41Data	HQER-SFA	hqer_induced	True	15	-2.929856443194528	hqer_induced sampler prototype	2.0
	front41Data	Quantile-SFA	hqer_induced	True	14	-10.806754002986793	hqer_induced sampler prototype	2.0
	front41Data	HQER-SFA	hqer_induced	True	47	-7.215716581587432	hqer_induced sampler prototype	4.0
	front41Data	Quantile-SFA	hqer_induced	True	34	-5.30175453559689	hqer_induced sampler prototype	4.0
	front41Data	HQER-SFA	hqer_induced	True	19	-4.088596206414816	hqer_induced sampler prototype	4.0
	front41Data	Quantile-SFA	hqer_induced	True	33	0.1850395599262694	hqer_induced sampler prototype	4.0
	front41Data	HQER-SFA	hqer_induced	True	17	1.5296659630397271	hqer_induced sampler prototype	2.0
	front41Data	Quantile-SFA	hqer_induced	True	17	-13.339334451664543	hqer_induced sampler prototype	2.0
	front41Data	HQER-SFA	hqer_induced	True	31	-9.975738361889176	hqer_induced sampler prototype	4.0
	front41Data	Quantile-SFA	hqer_induced	True	14	-1.6347429595329188	hqer_induced sampler prototype	2.0
	front41Data	HQER-SFA	hqer_induced	True	16	-1.5042956154431746	hqer_induced sampler prototype	2.0
	front41Data	Quantile-SFA	hqer_induced	True	14	3.3196900125274253	hqer_induced sampler prototype	2.0
	front41Data	HQER-SFA	hqer_induced	True	59	3.094766062816089	hqer_induced sampler prototype	2.0
	front41Data	Quantile-SFA	hqer_induced	True	32	0.5244346283032086	hqer_induced sampler prototype	4.0
	front41Data	HQER-SFA	hqer_induced	True	32	0.4629691821523032	hqer_induced sampler prototype	2.0
	front41Data	Quantile-SFA	hqer_induced	True	17	-14.54269851591124	hqer_induced sampler prototype	4.0
	front41Data	HQER-SFA	hqer_induced	True	24	-12.36960988905093	hqer_induced sampler prototype	2.0
	front41Data	Quantile-SFA	hqer_induced	True	52	7.666126123567842	hqer_induced sampler prototype	2.0
	front41Data	HQER-SFA	hqer_induced	True	11	8.928616432689282	hqer_induced sampler prototype	4.0
	front41Data	Quantile-SFA	hqer_induced	True	28	3.0063718882633554	hqer_induced sampler prototype	4.0
	front41Data	HQER-SFA	hqer_induced	True	42	4.369762872071459	hqer_induced sampler prototype	4.0
	front41Data	Quantile-SFA	hqer_induced	True	14	-16.491899213205805	hqer_induced sampler prototype	4.0
	front41Data	HQER-SFA	hqer_induced	True	87	-12.499409525047572	hqer_induced sampler prototype	4.0
	front41Data	Quantile-SFA	hqer_induced	True	19	2.1866437616942025	hqer_induced sampler prototype	4.0
	front41Data	HQER-SFA	hqer_induced	True	27	2.794076390129176	hqer_induced sampler prototype	4.0
	front41Data	Quantile-SFA	hqer_induced	True	15	-4.479255849136085	hqer_induced sampler prototype	4.0
	front41Data	HQER-SFA	hqer_induced	True	23	-3.8328801478926096	hqer_induced sampler prototype	4.0
	front41Data	Quantile-SFA	hqer_induced	True	24	-8.80461824790987	hqer_induced sampler prototype	2.0
	front41Data	HQER-SFA	hqer_induced	True	21	-7.238308527864966	hqer_induced sampler prototype	2.0
	front41Data	Quantile-SFA	hqer_induced	True	25	4.634730994474254	hqer_induced sampler prototype	2.0
	front41Data	HQER-SFA	hqer_induced	True	50	4.50659181608409	hqer_induced sampler prototype	4.0
	front41Data	Quantile-SFA	hqer_induced	True	15	-5.621964891812709	hqer_induced sampler prototype	2.0
	front41Data	HQER-SFA	hqer_induced	True	22	-2.2435085372797925	hqer_induced sampler prototype	4.0
	front41Data	Quantile-SFA	hqer_induced	True	13	-12.184579384989476	hqer_induced sampler prototype	2.0
	front41Data	HQER-SFA	hqer_induced	True	25	-12.404341716341245	hqer_induced sampler prototype	4.0
	front41Data	Quantile-SFA	hqer_induced	True	22	-4.414044465542824	hqer_induced sampler prototype	4.0
	front41Data	HQER-SFA	hqer_induced	True	17	-3.2420184362551554	hqer_induced sampler prototype	4.0
	front41Data	Quantile-SFA	hqer_induced	True	24	-9.531148726897811	hqer_induced sampler prototype	2.0
	front41Data	HQER-SFA	hqer_induced	True	29	-6.239366301760292	hqer_induced sampler prototype	4.0
	front41Data	Quantile-SFA	hqer_induced	True	20	7.444561611418016	hqer_induced sampler prototype	4.0
	front41Data	HQER-SFA	hqer_induced	True	27	11.180013691003069	hqer_induced sampler prototype	4.0
	front41Data	Quantile-SFA	hqer_induced	True	28	-5.52418924260322	hqer_induced sampler prototype	4.0
	front41Data	HQER-SFA	hqer_induced	True	26	-4.266625532118196	hqer_induced sampler prototype	2.0
	front41Data	Quantile-SFA	hqer_induced	True	26	5.009336937331613	hqer_induced sampler prototype	4.0
	front41Data	HQER-SFA	hqer_induced	True	56	4.442249283159258	hqer_induced sampler prototype	4.0
	front41Data	Quantile-SFA	hqer_induced	True	40	-13.493371233160143	hqer_induced sampler prototype	4.0
	front41Data	HQER-SFA	hqer_induced	True	15	-12.454944088806911	hqer_induced sampler prototype	4.0
	front41Data	Quantile-SFA	hqer_induced	True	24	-13.96854501021094	hqer_induced sampler prototype	4.0
	front41Data	HQER-SFA	hqer_induced	True	49	-13.911868912238226	hqer_induced sampler prototype	2.0
	front41Data	Quantile-SFA	hqer_induced	True	19	-7.473139572488317	hqer_induced sampler prototype	4.0
	front41Data	HQER-SFA	hqer_induced	True	26	-7.2981160848624	hqer_induced sampler prototype	4.0
	front41Data	Quantile-SFA	hqer_induced	True	13	0.2924219877773094	hqer_induced sampler prototype	2.0
	front41Data	HQER-SFA	hqer_induced	True	29	1.8725379116714205	hqer_induced sampler prototype	2.0
	front41Data	Quantile-SFA	hqer_induced	True	35	2.052358967109656	hqer_induced sampler prototype	2.0
	front41Data	HQER-SFA	hqer_induced	True	35	4.364148057739561	hqer_induced sampler prototype	4.0
	front41Data	Quantile-SFA	hqer_induced	True	36	-3.578835929776406	hqer_induced sampler prototype	4.0
	front41Data	HQER-SFA	hqer_induced	True	23	-2.5171798394289704	hqer_induced sampler prototype	2.0
	front41Data	Quantile-SFA	hqer_induced	True	15	-1.5351755307063986	hqer_induced sampler prototype	2.0
	front41Data	HQER-SFA	hqer_induced	True	16	-1.4125489041904278	hqer_induced sampler prototype	2.0
	front41Data	Quantile-SFA	hqer_induced	True	25	2.0256549814322984	hqer_induced sampler prototype	2.0
	front41Data	HQER-SFA	hqer_induced	True	42	1.2579723571598764	hqer_induced sampler prototype	2.0
	front41Data	Quantile-SFA	hqer_induced	True	17	1.5385951610615454	hqer_induced sampler prototype	2.0
	front41Data	HQER-SFA	hqer_induced	True	13	1.5075659251563358	hqer_induced sampler prototype	2.0
	front41Data	Quantile-SFA	hqer_induced	True	17	-1.9637926744912264	hqer_induced sampler prototype	2.0
	front41Data	HQER-SFA	hqer_induced	True	51	-2.9815300972615715	hqer_induced sampler prototype	2.0
	front41Data	Quantile-SFA	hqer_induced	True	35	-12.330384318583514	hqer_induced sampler prototype	2.0
	front41Data	HQER-SFA	hqer_induced	True	13	-12.307505920656991	hqer_induced sampler prototype	4.0
	front41Data	Quantile-SFA	hqer_induced	True	49	-4.801889840743642	hqer_induced sampler prototype	4.0
	front41Data	HQER-SFA	hqer_induced	True	28	-2.422754552472393	hqer_induced sampler prototype	4.0
	front41Data	Quantile-SFA	hqer_induced	True	51	0.216318227087839	hqer_induced sampler prototype	4.0
	front41Data	HQER-SFA	hqer_induced	True	11	1.0654664465550596	hqer_induced sampler prototype	2.0
	front41Data	Quantile-SFA	hqer_induced	True	44	-0.7914073934001303	hqer_induced sampler prototype	2.0
	front41Data	HQER-SFA	hqer_induced	True	32	-1.659367543964032	hqer_induced sampler prototype	2.0
	front41Data	Quantile-SFA	hqer_induced	True	25	-7.963077006274792	hqer_induced sampler prototype	4.0
	front41Data	HQER-SFA	hqer_induced	True	33	-6.794784284817949	hqer_induced sampler prototype	2.0
	front41Data	Quantile-SFA	hqer_induced	True	27	10.451345698728304	hqer_induced sampler prototype	4.0
	front41Data	HQER-SFA	hqer_induced	True	44	11.716033635941752	hqer_induced sampler prototype	2.0
	front41Data	Quantile-SFA	hqer_induced	True	35	-3.231487280815705	hqer_induced sampler prototype	4.0
	front41Data	HQER-SFA	hqer_induced	True	30	-0.6481563728112916	hqer_induced sampler prototype	2.0
	front41Data	Quantile-SFA	hqer_induced	True	22	-55.2625491072293	hqer_induced sampler prototype	4.0
	front41Data	HQER-SFA	hqer_induced	True	13	-43.54855119774691	hqer_induced sampler prototype	4.0
	front41Data	Quantile-SFA	hqer_induced	True	16	-79.81151868606827	hqer_induced sampler prototype	4.0
	front41Data	HQER-SFA	hqer_induced	True	12	-69.15534820480207	hqer_induced sampler prototype	2.0
	front41Data	Quantile-SFA	hqer_induced	True	16	-66.49871039393857	hqer_induced sampler prototype	4.0
	front41Data	HQER-SFA	hqer_induced	True	12	-54.35003772590214	hqer_induced sampler prototype	4.0
	front41Data	Quantile-SFA	hqer_induced	True	15	-69.21297685664493	hqer_induced sampler prototype	4.0
	front41Data	HQER-SFA	hqer_induced	True	16	-50.39824803125244	hqer_induced sampler prototype	4.0
	front41Data	Quantile-SFA	hqer_induced	True	16	-25.735635080718367	hqer_induced sampler prototype	4.0

experiment	dataset	model	scenario	converged	n__iter	loglik	warning__message	best__start__id
	front41Data	HQER-SFA	hqer__induced	True	35	-18.269174886409456	hqer__induced sampler prototype	4.0
	front41Data	Quantile-SFA	hqer__induced	True	21	-42.31884000414656	hqer__induced sampler prototype	4.0
	front41Data	HQER-SFA	hqer__induced	True	5	-30.331195965167865	hqer__induced sampler prototype	2.0
	front41Data	Quantile-SFA	hqer__induced	True	22	-50.04874857025462	hqer__induced sampler prototype	2.0
	front41Data	HQER-SFA	hqer__induced	True	29	-28.818180923558835	hqer__induced sampler prototype	2.0
	front41Data	Quantile-SFA	hqer__induced	True	16	-63.64965355379197	hqer__induced sampler prototype	4.0
	front41Data	HQER-SFA	hqer__induced	True	34	-46.52344844314115	hqer__induced sampler prototype	4.0
	front41Data	Quantile-SFA	hqer__induced	True	17	-75.03757874886901	hqer__induced sampler prototype	2.0
	front41Data	HQER-SFA	hqer__induced	True	21	-60.76322341718135	hqer__induced sampler prototype	2.0
	front41Data	Quantile-SFA	hqer__induced	True	36	-56.88127968218571	hqer__induced sampler prototype	4.0
	front41Data	HQER-SFA	hqer__induced	True	21	-42.52935838719367	hqer__induced sampler prototype	4.0
	front41Data	Quantile-SFA	hqer__induced	True	17	-12.807845958634005	hqer__induced sampler prototype	2.0
	front41Data	HQER-SFA	hqer__induced	True	45	-6.354445808148707	hqer__induced sampler prototype	4.0
	front41Data	Quantile-SFA	hqer__induced	True	26	-40.76590300424746	hqer__induced sampler prototype	2.0
	front41Data	HQER-SFA	hqer__induced	True	16	-29.10965733218196	hqer__induced sampler prototype	4.0
	front41Data	Quantile-SFA	hqer__induced	True	31	-51.23543013729751	hqer__induced sampler prototype	4.0
	front41Data	HQER-SFA	hqer__induced	True	19	-36.94657341176514	hqer__induced sampler prototype	4.0
	front41Data	Quantile-SFA	hqer__induced	True	27	-46.35041952428807	hqer__induced sampler prototype	2.0
	front41Data	HQER-SFA	hqer__induced	True	11	-31.166545199660533	hqer__induced sampler prototype	4.0
	front41Data	Quantile-SFA	hqer__induced	True	21	-48.70392812445405	hqer__induced sampler prototype	4.0
	front41Data	HQER-SFA	hqer__induced	True	8	-38.447956665887816	hqer__induced sampler prototype	2.0
	front41Data	Quantile-SFA	hqer__induced	True	42	-50.89478881578123	hqer__induced sampler prototype	2.0
	front41Data	HQER-SFA	hqer__induced	True	21	-38.03695450991918	hqer__induced sampler prototype	2.0
	front41Data	Quantile-SFA	hqer__induced	True	15	-68.90641803040158	hqer__induced sampler prototype	2.0
	front41Data	HQER-SFA	hqer__induced	True	21	-52.68899948868927	hqer__induced sampler prototype	4.0
	front41Data	Quantile-SFA	hqer__induced	True	31	-79.19845551943068	hqer__induced sampler prototype	2.0
	front41Data	HQER-SFA	hqer__induced	True	10	-65.50865053750569	hqer__induced sampler prototype	4.0
	front41Data	Quantile-SFA	hqer__induced	True	37	-29.2323690148752	hqer__induced sampler prototype	4.0
	front41Data	HQER-SFA	hqer__induced	True	47	-24.865266151274085	hqer__induced sampler prototype	4.0
	front41Data	Quantile-SFA	hqer__induced	True	18	-49.37238460041152	hqer__induced sampler prototype	4.0
	front41Data	HQER-SFA	hqer__induced	True	10	-37.68988857953396	hqer__induced sampler prototype	2.0
	front41Data	Quantile-SFA	hqer__induced	True	38	-37.17611200781093	hqer__induced sampler prototype	2.0
	front41Data	HQER-SFA	hqer__induced	True	29	-22.44917864136076	hqer__induced sampler prototype	2.0
	front41Data	Quantile-SFA	hqer__induced	True	30	-76.30603442011092	hqer__induced sampler prototype	2.0
	front41Data	HQER-SFA	hqer__induced	True	14	-66.2316953479718	hqer__induced sampler prototype	2.0
	front41Data	Quantile-SFA	hqer__induced	True	39	-67.28519542236324	hqer__induced sampler prototype	2.0
	front41Data	HQER-SFA	hqer__induced	True	8	-56.82334416592099	hqer__induced sampler prototype	4.0
	front41Data	Quantile-SFA	hqer__induced	True	25	-45.71323903852874	hqer__induced sampler prototype	2.0
	front41Data	HQER-SFA	hqer__induced	True	22	-30.94544137889693	hqer__induced sampler prototype	2.0
	front41Data	Quantile-SFA	hqer__induced	True	25	-40.88042853431762	hqer__induced sampler prototype	2.0
	front41Data	HQER-SFA	hqer__induced	True	13	-27.724163538583703	hqer__induced sampler prototype	4.0
	front41Data	Quantile-SFA	hqer__induced	True	15	-26.512022664801925	hqer__induced sampler prototype	2.0
	front41Data	HQER-SFA	hqer__induced	True	51	-22.287682635972395	hqer__induced sampler prototype	4.0
	front41Data	Quantile-SFA	hqer__induced	True	21	-75.43873053113656	hqer__induced sampler prototype	2.0
	front41Data	HQER-SFA	hqer__induced	True	47	-67.30526924463899	hqer__induced sampler prototype	4.0
	front41Data	Quantile-SFA	hqer__induced	True	27	-54.015178907281935	hqer__induced sampler prototype	2.0
	front41Data	HQER-SFA	hqer__induced	True	12	-37.68987624305955	hqer__induced sampler prototype	4.0
	front41Data	Quantile-SFA	hqer__induced	True	34	-20.25287736207177	hqer__induced sampler prototype	2.0
	front41Data	HQER-SFA	hqer__induced	True	21	-13.08191795604846	hqer__induced sampler prototype	2.0
	front41Data	Quantile-SFA	hqer__induced	True	22	-71.74056411333127	hqer__induced sampler prototype	2.0
	front41Data	HQER-SFA	hqer__induced	True	30	-54.613312988164445	hqer__induced sampler prototype	2.0
	front41Data	Quantile-SFA	hqer__induced	True	33	-35.7922512614068	hqer__induced sampler prototype	2.0
	front41Data	HQER-SFA	hqer__induced	True	8	-18.9606761367428	hqer__induced sampler prototype	2.0
	front41Data	Quantile-SFA	hqer__induced	True	25	4.41930287153866	hqer__induced sampler prototype	2.0
	front41Data	HQER-SFA	hqer__induced	True	26	17.532153443631078	hqer__induced sampler prototype	2.0
	front41Data	Quantile-SFA	hqer__induced	True	17	-18.23987266810788	hqer__induced sampler prototype	4.0
	front41Data	HQER-SFA	hqer__induced	True	33	-10.26845711199593	hqer__induced sampler prototype	4.0
	front41Data	Quantile-SFA	hqer__induced	True	32	-51.05234403001385	hqer__induced sampler prototype	4.0
	front41Data	HQER-SFA	hqer__induced	True	35	-35.777668473028854	hqer__induced sampler prototype	2.0
	front41Data	Quantile-SFA	hqer__induced	True	25	-67.96022606966613	hqer__induced sampler prototype	4.0
	front41Data	HQER-SFA	hqer__induced	True	9	-53.78031588290899	hqer__induced sampler prototype	2.0
	front41Data	Quantile-SFA	hqer__induced	True	21	-27.99659948260146	hqer__induced sampler prototype	4.0
	front41Data	HQER-SFA	hqer__induced	True	13	-9.472480572489062	hqer__induced sampler prototype	2.0
	front41Data	Quantile-SFA	hqer__induced	True	28	-52.92049706794106	hqer__induced sampler prototype	4.0
	front41Data	HQER-SFA	hqer__induced	True	16	-34.87976251931599	hqer__induced sampler prototype	2.0
	front41Data	Quantile-SFA	hqer__induced	True	22	-39.244019627565294	hqer__induced sampler prototype	4.0
	front41Data	HQER-SFA	hqer__induced	True	9	-30.694529914505885	hqer__induced sampler prototype	2.0
	front41Data	Quantile-SFA	hqer__induced	True	35	-38.02429676284864	hqer__induced sampler prototype	4.0
	front41Data	HQER-SFA	hqer__induced	True	27	-27.32449380392788	hqer__induced sampler prototype	4.0
	front41Data	Quantile-SFA	hqer__induced	True	41	-51.14199860474268	hqer__induced sampler prototype	2.0
	front41Data	HQER-SFA	hqer__induced	True	24	-38.53782404098769	hqer__induced sampler prototype	2.0
	front41Data	Quantile-SFA	hqer__induced	True	13	-57.74996610583423	hqer__induced sampler prototype	4.0
	front41Data	HQER-SFA	hqer__induced	True	11	-43.92330034946954	hqer__induced sampler prototype	2.0
	front41Data	Quantile-SFA	hqer__induced	True	13	-57.27341707710754	hqer__induced sampler prototype	4.0
	front41Data	HQER-SFA	hqer__induced	True	11	-51.98098696346493	hqer__induced sampler prototype	2.0
	front41Data	Quantile-SFA	hqer__induced	True	14	-39.38447672157108	hqer__induced sampler prototype	2.0
	front41Data	HQER-SFA	hqer__induced	True	18	-18.501042636322527	hqer__induced sampler prototype	4.0
	front41Data	Quantile-SFA	hqer__induced	True	28	-61.16546566609983	hqer__induced sampler prototype	4.0
	front41Data	HQER-SFA	hqer__induced	True	17	-40.69001120355741	hqer__induced sampler prototype	2.0
	front41Data	Quantile-SFA	hqer__induced	True	26	-43.17969814520941	hqer__induced sampler prototype	2.0
	front41Data	HQER-SFA	hqer__induced	True	29	-21.32707017110657	hqer__induced sampler prototype	2.0
	front41Data	Quantile-SFA	hqer__induced	True	29	-57.97427417305372	hqer__induced sampler prototype	2.0
	front41Data	HQER-SFA	hqer__induced	True	13	-38.54895836233186	hqer__induced sampler prototype	4.0
	front41Data	Quantile-SFA	hqer__induced	True	38	-55.000919937269146	hqer__induced sampler prototype	4.0
	front41Data	HQER-SFA	hqer__induced	True	25	-36.64236636413291	hqer__induced sampler prototype	2.0
	front41Data	Quantile-SFA	hqer__induced	True	25	-72.86704096563294	hqer__induced sampler prototype	2.0
	front41Data	HQER-SFA	hqer__induced	True	17	-64.29947105113118	hqer__induced sampler prototype	2.0
	front41Data	Quantile-SFA	hqer__induced	True	20	-62.99483985803876	hqer__induced sampler prototype	2.0
	front41Data	HQER-SFA	hqer__induced	True	8	-47.98804796653835	hqer__induced sampler prototype	2.0
	front41Data	Quantile-SFA	hqer__induced	True	27	-83.16858011132655	hqer__induced sampler prototype	4.0
	front41Data	HQER-SFA	hqer__induced	True	10	-67.1620327948634	hqer__induced sampler prototype	4.0
	front41Data	Quantile-SFA	hqer__induced	True	29	-15.89350020598443	hqer__induced sampler prototype	4.0
	front41Data	HQER-SFA	hqer__induced	True	20	-10.079446619239857	hqer__induced sampler prototype	4.0
	front41Data	Quantile-SFA	hqer__induced	True	12	-34.84837273382358	hqer__induced sampler prototype	4.0
	front41Data	HQER-SFA	hqer__induced	True	52	-21.797586722237694	hqer__induced sampler prototype	4.0
	front41Data	Quantile-SFA	hqer__induced	True	29	-64.11728237254574	hqer__induced sampler prototype	2.0
	front41Data	HQER-SFA	hqer__induced	True	40	-42.41517470868144	hqer__induced sampler prototype	2.0
	front41Data	Quantile-SFA	hqer__induced	True	37	-37.11694533692098	hqer__induced sampler prototype	4.0
	front41Data	HQER-SFA	hqer__induced	True	22	-25.804564864584314	hqer__induced sampler prototype	4.0
	front41Data	Quantile-SFA	hqer__induced	True	37	-64.15286812997029	hqer__induced sampler prototype	2.0
	front41Data	HQER-SFA	hqer__induced	True	24	-51.66463726904689	hqer__induced sampler prototype	4.0
	front41Data	Quantile-SFA	hqer__induced	True	20	-48.47406769464689	hqer__induced sampler prototype	4.0
	front41Data	HQER-SFA	hqer__induced	True	11	-40.61163234275882	hqer__induced sampler prototype	2.0

experiment	dataset	model	scenario	converged	n_iter	loglik	warning_message	best_start_id
	front41Data	Quantile-SFA	hqer_induced	True	14	-64.51814540695145	hqer_induced sampler prototype	4.0
	front41Data	HQER-SFA	hqer_induced	True	13	-41.9316396536242	hqer_induced sampler prototype	4.0
	front41Data	Quantile-SFA	hqer_induced	True	30	-12.519984168175949	hqer_induced sampler prototype	4.0
	front41Data	HQER-SFA	hqer_induced	True	37	-6.316767867773664	hqer_induced sampler prototype	4.0
	front41Data	Quantile-SFA	hqer_induced	True	27	-33.81478348934159	hqer_induced sampler prototype	4.0
	front41Data	HQER-SFA	hqer_induced	True	15	-22.84263901913081	hqer_induced sampler prototype	4.0
	front41Data	Quantile-SFA	hqer_induced	True	28	-41.66458932960191	hqer_induced sampler prototype	2.0
	front41Data	HQER-SFA	hqer_induced	True	22	-33.18135344532972	hqer_induced sampler prototype	4.0
	front41Data	Quantile-SFA	hqer_induced	True	36	-62.20917952886658	hqer_induced sampler prototype	2.0
	front41Data	HQER-SFA	hqer_induced	True	30	-48.90699873654917	hqer_induced sampler prototype	4.0
	front41Data	Quantile-SFA	hqer_induced	True	12	-99.10146837468008	hqer_induced sampler prototype	4.0
	front41Data	HQER-SFA	hqer_induced	True	13	-82.7230514142478	hqer_induced sampler prototype	4.0
	front41Data	Quantile-SFA	hqer_induced	True	20	-27.088208563614064	hqer_induced sampler prototype	4.0
	front41Data	HQER-SFA	hqer_induced	True	7	-16.89443372452211	hqer_induced sampler prototype	2.0
	front41Data	Quantile-SFA	hqer_induced	True	31	-45.856887684098105	hqer_induced sampler prototype	2.0
	front41Data	HQER-SFA	hqer_induced	True	16	-31.504335625379213	hqer_induced sampler prototype	4.0
	front41Data	Quantile-SFA	hqer_induced	True	31	-56.24756231883688	hqer_induced sampler prototype	4.0
	front41Data	HQER-SFA	hqer_induced	True	19	-31.700077411757924	hqer_induced sampler prototype	2.0
	front41Data	Quantile-SFA	hqer_induced	True	20	-46.31235668351637	hqer_induced sampler prototype	4.0
	front41Data	HQER-SFA	hqer_induced	True	25	-31.373709491518888	hqer_induced sampler prototype	4.0
	front41Data	Quantile-SFA	hqer_induced	True	30	-44.8386254421893	hqer_induced sampler prototype	2.0
	front41Data	HQER-SFA	hqer_induced	True	16	-30.06163065620036	hqer_induced sampler prototype	2.0
	front41Data	Quantile-SFA	hqer_induced	True	20	-58.27198078931938	hqer_induced sampler prototype	4.0
	front41Data	HQER-SFA	hqer_induced	True	25	-40.03353989086996	hqer_induced sampler prototype	4.0
	front41Data	Quantile-SFA	hqer_induced	True	20	-50.51756029269698	hqer_induced sampler prototype	2.0
	front41Data	HQER-SFA	hqer_induced	True	56	-45.06380790684099	hqer_induced sampler prototype	4.0
	front41Data	Quantile-SFA	hqer_induced	True	15	-54.21120127301395	hqer_induced sampler prototype	2.0
	front41Data	HQER-SFA	hqer_induced	True	27	-32.32087019008085	hqer_induced sampler prototype	4.0
	front41Data	Quantile-SFA	hqer_induced	True	32	-32.37683371088039	hqer_induced sampler prototype	2.0
	front41Data	HQER-SFA	hqer_induced	True	61	-25.015668348170983	hqer_induced sampler prototype	4.0
	front41Data	Quantile-SFA	hqer_induced	True	24	-69.22721344439134	hqer_induced sampler prototype	2.0
	front41Data	HQER-SFA	hqer_induced	True	11	-63.1939959233185	hqer_induced sampler prototype	2.0
	front41Data	Quantile-SFA	hqer_induced	True	31	-50.87512548234983	hqer_induced sampler prototype	2.0
	front41Data	HQER-SFA	hqer_induced	True	9	-35.781595825309694	hqer_induced sampler prototype	2.0
	front41Data	Quantile-SFA	hqer_induced	True	30	-52.68473877910718	hqer_induced sampler prototype	4.0
	front41Data	HQER-SFA	hqer_induced	True	16	-37.76825218910129	hqer_induced sampler prototype	2.0
	front41Data	Quantile-SFA	hqer_induced	True	13	-32.88619345749332	hqer_induced sampler prototype	4.0
	front41Data	HQER-SFA	hqer_induced	True	14	-20.58144126317178	hqer_induced sampler prototype	2.0
	front41Data	Quantile-SFA	hqer_induced	True	12	-39.33840009630508	hqer_induced sampler prototype	4.0
	front41Data	HQER-SFA	hqer_induced	True	14	-24.83882685765334	hqer_induced sampler prototype	4.0
	front41Data	Quantile-SFA	hqer_induced	True	28	-37.52673998722848	hqer_induced sampler prototype	4.0
	front41Data	HQER-SFA	hqer_induced	True	11	-25.186881532465183	hqer_induced sampler prototype	4.0
	front41Data	Quantile-SFA	hqer_induced	True	21	-22.59576836887925	hqer_induced sampler prototype	4.0
	front41Data	HQER-SFA	hqer_induced	True	25	-12.056972134355938	hqer_induced sampler prototype	4.0
	front41Data	Quantile-SFA	hqer_induced	True	30	-47.8505574035847	hqer_induced sampler prototype	4.0
	front41Data	HQER-SFA	hqer_induced	True	32	-39.01355554041805	hqer_induced sampler prototype	4.0
	front41Data	Quantile-SFA	hqer_induced	True	32	-53.77724157131138	hqer_induced sampler prototype	2.0
	front41Data	HQER-SFA	hqer_induced	True	39	-38.57653783752218	hqer_induced sampler prototype	4.0
	front41Data	Quantile-SFA	hqer_induced	True	62	-4.314605292857408	hqer_induced sampler prototype	4.0
	front41Data	HQER-SFA	hqer_induced	True	50	4.922436966910034	hqer_induced sampler prototype	2.0
	front41Data	Quantile-SFA	hqer_induced	True	52	-38.83301523130319	hqer_induced sampler prototype	4.0
	front41Data	HQER-SFA	hqer_induced	True	27	-24.89473260007346	hqer_induced sampler prototype	2.0
	front41Data	Quantile-SFA	hqer_induced	True	17	-52.97202734071513	hqer_induced sampler prototype	4.0
	front41Data	HQER-SFA	hqer_induced	True	9	-49.1271772917324	hqer_induced sampler prototype	2.0
	front41Data	Quantile-SFA	hqer_induced	True	38	-63.15953768085342	hqer_induced sampler prototype	2.0
	front41Data	HQER-SFA	hqer_induced	True	11	-52.49255997391081	hqer_induced sampler prototype	4.0
	front41Data	Quantile-SFA	hqer_induced	True	31	-34.60638023498218	hqer_induced sampler prototype	4.0
	front41Data	HQER-SFA	hqer_induced	True	25	-26.78314583155543	hqer_induced sampler prototype	4.0
	front41Data	Quantile-SFA	hqer_induced	True	20	-69.35891480963966	hqer_induced sampler prototype	4.0
	front41Data	HQER-SFA	hqer_induced	True	18	-58.596479283568186	hqer_induced sampler prototype	2.0
	front41Data	Quantile-SFA	hqer_induced	True	17	-67.45931576958202	hqer_induced sampler prototype	4.0
	front41Data	HQER-SFA	hqer_induced	True	28	-50.79326627601952	hqer_induced sampler prototype	2.0
	front41Data	Quantile-SFA	hqer_induced	True	22	-35.10312063669661	hqer_induced sampler prototype	2.0
	front41Data	HQER-SFA	hqer_induced	True	16	-25.806814635201327	hqer_induced sampler prototype	2.0
	front41Data	Quantile-SFA	hqer_induced	True	25	-39.78793057774448	hqer_induced sampler prototype	2.0
	front41Data	HQER-SFA	hqer_induced	True	14	-25.18711920580305	hqer_induced sampler prototype	2.0
	front41Data	Quantile-SFA	hqer_induced	True	16	-12.34764120311026	hqer_induced sampler prototype	2.0
	front41Data	HQER-SFA	hqer_induced	True	32	-4.718614375237794	hqer_induced sampler prototype	2.0
	front41Data	Quantile-SFA	hqer_induced	True	23	-45.2680893371836	hqer_induced sampler prototype	4.0
	front41Data	HQER-SFA	hqer_induced	True	8	-32.173946013921444	hqer_induced sampler prototype	2.0
	front41Data	Quantile-SFA	hqer_induced	True	47	-30.044427102995343	hqer_induced sampler prototype	4.0
	front41Data	HQER-SFA	hqer_induced	True	21	-12.172867667932907	hqer_induced sampler prototype	4.0
	front41Data	Quantile-SFA	hqer_induced	True	15	-57.060978024087774	hqer_induced sampler prototype	4.0
	front41Data	HQER-SFA	hqer_induced	True	11	-40.62528257460607	hqer_induced sampler prototype	2.0
	front41Data	Quantile-SFA	hqer_induced	True	38	-40.507470797398256	hqer_induced sampler prototype	2.0
	front41Data	HQER-SFA	hqer_induced	True	16	-31.32826662424344	hqer_induced sampler prototype	4.0
	front41Data	Quantile-SFA	hqer_induced	True	20	-24.47992860352907	hqer_induced sampler prototype	2.0
	front41Data	HQER-SFA	hqer_induced	True	29	-14.69733597936926	hqer_induced sampler prototype	2.0
	front41Data	Quantile-SFA	hqer_induced	True	38	-66.91331256852258	hqer_induced sampler prototype	2.0
	front41Data	HQER-SFA	hqer_induced	True	29	-47.71492771460501	hqer_induced sampler prototype	4.0
	front41Data	Quantile-SFA	hqer_induced	True	23	-58.947753848410144	hqer_induced sampler prototype	4.0
	front41Data	HQER-SFA	hqer_induced	True	10	-47.07924283278406	hqer_induced sampler prototype	2.0
	front41Data	Quantile-SFA	hqer_induced	True	35	-49.333673272800446	hqer_induced sampler prototype	4.0
	front41Data	HQER-SFA	hqer_induced	True	32	-35.45620704774319	hqer_induced sampler prototype	2.0
	front41Data	Quantile-SFA	hqer_induced	True	33	-45.6846092295531	hqer_induced sampler prototype	2.0
	front41Data	HQER-SFA	hqer_induced	True	32	-30.79065813082736	hqer_induced sampler prototype	2.0
	front41Data	Quantile-SFA	hqer_induced	True	17	-73.82741305461852	hqer_induced sampler prototype	4.0
	front41Data	HQER-SFA	hqer_induced	True	22	-56.94816109803742	hqer_induced sampler prototype	2.0
	WDI_industry	Quantile-SFA	hqer_induced	True	19	-165.6621995592684	hqer_induced sampler prototype	2.0
	WDI_industry	HQER-SFA	hqer_induced	True	24	-165.637135129797	hqer_induced sampler prototype	2.0
	WDI_industry	Quantile-SFA	hqer_induced	True	17	-138.64951807729602	hqer_induced sampler prototype	2.0
	WDI_industry	HQER-SFA	hqer_induced	True	15	-138.60405499057623	hqer_induced sampler prototype	2.0
	WDI_industry	Quantile-SFA	hqer_induced	True	35	-131.42923881587137	hqer_induced sampler prototype	4.0
	WDI_industry	HQER-SFA	hqer_induced	True	26	-131.39470710781734	hqer_induced sampler prototype	4.0
	WDI_industry	Quantile-SFA	hqer_induced	True	26	-114.3925250749536	hqer_induced sampler prototype	4.0
	WDI_industry	HQER-SFA	hqer_induced	True	27	-114.35397202957792	hqer_induced sampler prototype	4.0
	WDI_industry	Quantile-SFA	hqer_induced	True	25	-141.66341142522035	hqer_induced sampler prototype	4.0
	WDI_industry	HQER-SFA	hqer_induced	True	24	-141.6503520845356	hqer_induced sampler prototype	4.0
	WDI_industry	Quantile-SFA	hqer_induced	True	46	-150.0325577858692	hqer_induced sampler prototype	5.0
	WDI_industry	HQER-SFA	hqer_induced	True	19	-149.9793785282685	hqer_induced sampler prototype	4.0
	WDI_industry	Quantile-SFA	hqer_induced	True	19	-133.73362781894338	hqer_induced sampler prototype	2.0
	WDI_industry	HQER-SFA	hqer_induced	True	21	-133.6806016921286	hqer_induced sampler prototype	2.0
	WDI_industry	Quantile-SFA	hqer_induced	True	24	-148.7030161524974	hqer_induced sampler prototype	4.0

experiment	dataset	model	scenario	converged	n__iter	loglik	warning__message	best__start__id
	WDI__industry	HQER-SFA	hqer__induced	True	23	-148.6500409835131	hqer__induced sampler prototype	4.0
	WDI__industry	Quantile-SFA	hqer__induced	True	19	-135.50556506982906	hqer__induced sampler prototype	4.0
	WDI__industry	HQER-SFA	hqer__induced	True	25	-135.45891555288765	hqer__induced sampler prototype	4.0
	WDI__industry	Quantile-SFA	hqer__induced	True	22	-136.20826520051185	hqer__induced sampler prototype	4.0
	WDI__industry	HQER-SFA	hqer__induced	True	23	-136.2065882501225	hqer__induced sampler prototype	4.0
	WDI__industry	Quantile-SFA	hqer__induced	True	29	-147.23783868801144	hqer__induced sampler prototype	2.0
	WDI__industry	HQER-SFA	hqer__induced	True	24	-147.17665785077045	hqer__induced sampler prototype	4.0
	WDI__industry	Quantile-SFA	hqer__induced	True	20	-130.83286333649176	hqer__induced sampler prototype	2.0
	WDI__industry	HQER-SFA	hqer__induced	True	19	-130.77508842975823	hqer__induced sampler prototype	2.0
	WDI__industry	Quantile-SFA	hqer__induced	True	24	-135.21629894039694	hqer__induced sampler prototype	4.0
	WDI__industry	HQER-SFA	hqer__induced	True	17	-135.18806967408312	hqer__induced sampler prototype	2.0
	WDI__industry	Quantile-SFA	hqer__induced	True	25	-141.75389475731322	hqer__induced sampler prototype	2.0
	WDI__industry	HQER-SFA	hqer__induced	True	21	-141.7134999754008	hqer__induced sampler prototype	4.0
	WDI__industry	Quantile-SFA	hqer__induced	True	29	-144.5049048192513	hqer__induced sampler prototype	4.0
	WDI__industry	HQER-SFA	hqer__induced	True	26	-144.45545140532698	hqer__induced sampler prototype	4.0
	WDI__industry	Quantile-SFA	hqer__induced	True	20	-130.81963651639762	hqer__induced sampler prototype	4.0
	WDI__industry	HQER-SFA	hqer__induced	True	30	-130.76663407659908	hqer__induced sampler prototype	4.0
	WDI__industry	Quantile-SFA	hqer__induced	True	20	-144.21882577505892	hqer__induced sampler prototype	4.0
	WDI__industry	HQER-SFA	hqer__induced	True	24	-144.2144650732356	hqer__induced sampler prototype	4.0
	WDI__industry	Quantile-SFA	hqer__induced	True	27	-113.030866572938	hqer__induced sampler prototype	4.0
	WDI__industry	HQER-SFA	hqer__induced	True	23	-113.02713623820254	hqer__induced sampler prototype	4.0
	WDI__industry	Quantile-SFA	hqer__induced	True	28	-144.62810136258597	hqer__induced sampler prototype	2.0
	WDI__industry	HQER-SFA	hqer__induced	True	23	-144.5781922260337	hqer__induced sampler prototype	2.0
	WDI__industry	Quantile-SFA	hqer__induced	True	16	-116.14597274252752	hqer__induced sampler prototype	2.0
	WDI__industry	HQER-SFA	hqer__induced	True	23	-116.12498900656784	hqer__induced sampler prototype	4.0
	WDI__industry	Quantile-SFA	hqer__induced	True	24	-116.04694543772912	hqer__induced sampler prototype	4.0
	WDI__industry	HQER-SFA	hqer__induced	True	28	-116.01092704777692	hqer__induced sampler prototype	4.0
	WDI__industry	Quantile-SFA	hqer__induced	True	22	-145.9237897656552	hqer__induced sampler prototype	4.0
	WDI__industry	HQER-SFA	hqer__induced	True	21	-145.87410066253167	hqer__induced sampler prototype	4.0
	WDI__industry	Quantile-SFA	hqer__induced	True	19	-119.54250743457663	hqer__induced sampler prototype	2.0
	WDI__industry	HQER-SFA	hqer__induced	True	13	-119.52034766118192	hqer__induced sampler prototype	2.0
	WDI__industry	Quantile-SFA	hqer__induced	True	13	-108.71376776325468	hqer__induced sampler prototype	2.0
	WDI__industry	HQER-SFA	hqer__induced	True	18	-108.66854520849962	hqer__induced sampler prototype	2.0
	WDI__industry	Quantile-SFA	hqer__induced	True	29	-175.4968317757366	hqer__induced sampler prototype	4.0
	WDI__industry	HQER-SFA	hqer__induced	True	26	-175.4811878309309	hqer__induced sampler prototype	4.0
	WDI__industry	Quantile-SFA	hqer__induced	True	22	-108.71236097047247	hqer__induced sampler prototype	4.0
	WDI__industry	HQER-SFA	hqer__induced	True	28	-108.6736552484428	hqer__induced sampler prototype	4.0
	WDI__industry	Quantile-SFA	hqer__induced	True	24	-149.87456563981425	hqer__induced sampler prototype	4.0
	WDI__industry	HQER-SFA	hqer__induced	True	29	-149.83267163654827	hqer__induced sampler prototype	4.0
	WDI__industry	Quantile-SFA	hqer__induced	True	22	-144.96780640759482	hqer__induced sampler prototype	4.0
	WDI__industry	HQER-SFA	hqer__induced	True	20	-144.93358240656823	hqer__induced sampler prototype	4.0
	WDI__industry	Quantile-SFA	hqer__induced	True	15	-120.86747575410376	hqer__induced sampler prototype	2.0
	WDI__industry	HQER-SFA	hqer__induced	True	23	-120.80330613751246	hqer__induced sampler prototype	2.0
	WDI__industry	Quantile-SFA	hqer__induced	True	23	-121.91320352971022	hqer__induced sampler prototype	4.0
	WDI__industry	HQER-SFA	hqer__induced	True	21	-121.86114471163552	hqer__induced sampler prototype	4.0
	WDI__industry	Quantile-SFA	hqer__induced	True	16	-129.88374118120615	hqer__induced sampler prototype	2.0
	WDI__industry	HQER-SFA	hqer__induced	True	22	-129.79939388446763	hqer__induced sampler prototype	4.0
	WDI__industry	Quantile-SFA	hqer__induced	True	22	-132.30916148816556	hqer__induced sampler prototype	4.0
	WDI__industry	HQER-SFA	hqer__induced	True	20	-132.2621920609695	hqer__induced sampler prototype	4.0
	WDI__industry	Quantile-SFA	hqer__induced	True	31	-116.31923927463596	hqer__induced sampler prototype	2.0
	WDI__industry	HQER-SFA	hqer__induced	True	40	-116.27767874170802	hqer__induced sampler prototype	4.0
	WDI__industry	Quantile-SFA	hqer__induced	True	32	-110.3284152354505	hqer__induced sampler prototype	4.0
	WDI__industry	HQER-SFA	hqer__induced	True	25	-110.2739430555494	hqer__induced sampler prototype	4.0
	WDI__industry	Quantile-SFA	hqer__induced	True	17	-120.65836821872664	hqer__induced sampler prototype	4.0
	WDI__industry	HQER-SFA	hqer__induced	True	19	-120.62073431909216	hqer__induced sampler prototype	4.0
	WDI__industry	Quantile-SFA	hqer__induced	True	19	-151.07905713760005	hqer__induced sampler prototype	4.0
	WDI__industry	HQER-SFA	hqer__induced	True	21	-151.03250668986655	hqer__induced sampler prototype	2.0
	WDI__industry	Quantile-SFA	hqer__induced	True	24	-158.6747918388341	hqer__induced sampler prototype	4.0
	WDI__industry	HQER-SFA	hqer__induced	True	25	-158.62238111382703	hqer__induced sampler prototype	4.0
	WDI__industry	Quantile-SFA	hqer__induced	True	30	-142.91468433994507	hqer__induced sampler prototype	2.0
	WDI__industry	HQER-SFA	hqer__induced	True	24	-142.84817345473945	hqer__induced sampler prototype	4.0
	WDI__industry	Quantile-SFA	hqer__induced	True	26	-130.74247987908194	hqer__induced sampler prototype	4.0
	WDI__industry	HQER-SFA	hqer__induced	True	21	-130.70870164991808	hqer__induced sampler prototype	2.0
	WDI__industry	Quantile-SFA	hqer__induced	True	20	-150.48487071888945	hqer__induced sampler prototype	2.0
	WDI__industry	HQER-SFA	hqer__induced	True	14	-150.4229856750087	hqer__induced sampler prototype	2.0
	WDI__industry	Quantile-SFA	hqer__induced	True	20	-157.75690711506837	hqer__induced sampler prototype	4.0
	WDI__industry	HQER-SFA	hqer__induced	True	14	-157.69190982119324	hqer__induced sampler prototype	2.0
	WDI__industry	Quantile-SFA	hqer__induced	True	23	-143.6981932513334	hqer__induced sampler prototype	4.0
	WDI__industry	HQER-SFA	hqer__induced	True	27	-143.67058569887286	hqer__induced sampler prototype	4.0
	WDI__industry	Quantile-SFA	hqer__induced	True	16	-147.05047099271815	hqer__induced sampler prototype	4.0
	WDI__industry	HQER-SFA	hqer__induced	True	11	-147.00789809813233	hqer__induced sampler prototype	4.0
	WDI__industry	Quantile-SFA	hqer__induced	True	24	-144.8433487750113	hqer__induced sampler prototype	4.0
	WDI__industry	HQER-SFA	hqer__induced	True	17	-144.86078523304235	hqer__induced sampler prototype	4.0
	WDI__industry	Quantile-SFA	hqer__induced	True	19	-175.4066870322008	hqer__induced sampler prototype	4.0
	WDI__industry	HQER-SFA	hqer__induced	True	19	-175.3349436745064	hqer__induced sampler prototype	2.0
	WDI__industry	Quantile-SFA	hqer__induced	True	29	-131.14331980024286	hqer__induced sampler prototype	4.0
	WDI__industry	HQER-SFA	hqer__induced	True	11	-131.12436925977462	hqer__induced sampler prototype	2.0
	WDI__industry	Quantile-SFA	hqer__induced	True	21	-145.78239095162246	hqer__induced sampler prototype	4.0
	WDI__industry	HQER-SFA	hqer__induced	True	24	-145.73217604854457	hqer__induced sampler prototype	4.0
	WDI__industry	Quantile-SFA	hqer__induced	True	21	-137.670237294427	hqer__induced sampler prototype	4.0
	WDI__industry	HQER-SFA	hqer__induced	True	24	-137.6024423362681	hqer__induced sampler prototype	4.0
	WDI__industry	Quantile-SFA	hqer__induced	True	24	-123.4434280141643	hqer__induced sampler prototype	4.0
	WDI__industry	HQER-SFA	hqer__induced	True	22	-123.41994338167672	hqer__induced sampler prototype	4.0
	WDI__industry	Quantile-SFA	hqer__induced	True	17	-152.86171663121445	hqer__induced sampler prototype	2.0
	WDI__industry	HQER-SFA	hqer__induced	True	32	-152.8392407606636	hqer__induced sampler prototype	4.0
	WDI__industry	Quantile-SFA	hqer__induced	True	20	-115.59336098546028	hqer__induced sampler prototype	2.0
	WDI__industry	HQER-SFA	hqer__induced	True	25	-115.56836909206535	hqer__induced sampler prototype	4.0
	WDI__industry	Quantile-SFA	hqer__induced	True	21	-127.52357061394788	hqer__induced sampler prototype	4.0
	WDI__industry	HQER-SFA	hqer__induced	True	13	-127.4496249780631	hqer__induced sampler prototype	2.0
	WDI__industry	Quantile-SFA	hqer__induced	True	17	-161.70518795929263	hqer__induced sampler prototype	4.0
	WDI__industry	HQER-SFA	hqer__induced	True	11	-161.6907546127207	hqer__induced sampler prototype	2.0
	WDI__industry	Quantile-SFA	hqer__induced	True	24	-126.0706550379016	hqer__induced sampler prototype	2.0
	WDI__industry	HQER-SFA	hqer__induced	True	22	-126.03266804728554	hqer__induced sampler prototype	2.0
	WDI__industry	Quantile-SFA	hqer__induced	True	27	-107.90032153111297	hqer__induced sampler prototype	4.0
	WDI__industry	HQER-SFA	hqer__induced	True	20	-107.90460594071624	hqer__induced sampler prototype	2.0
	WDI__industry	Quantile-SFA	hqer__induced	True	22	-105.49207428899746	hqer__induced sampler prototype	4.0
	WDI__industry	HQER-SFA	hqer__induced	True	21	-105.50497686185253	hqer__induced sampler prototype	2.0
	WDI__industry	Quantile-SFA	hqer__induced	True	26	-129.27801111827927	hqer__induced sampler prototype	4.0
	WDI__industry	HQER-SFA	hqer__induced	True	26	-129.25488154158745	hqer__induced sampler prototype	2.0
	WDI__industry	Quantile-SFA	hqer__induced	True	27	-111.6551649442526	hqer__induced sampler prototype	4.0
	WDI__industry	HQER-SFA	hqer__induced	True	22	-111.6356382053784	hqer__induced sampler prototype	4.0
	WDI__industry	Quantile-SFA	hqer__induced	True	28	-159.37701725857667	hqer__induced sampler prototype	2.0
	WDI__industry	HQER-SFA	hqer__induced	True	27	-159.33017183156852	hqer__induced sampler prototype	2.0

experiment	dataset	model	scenario	converged	n_iter	loglik	warning_message	best_start_id
	WDI_industry	Quantile-SFA	hqer_induced	True	19	-113.7976768316538	hqer_induced sampler prototype	2.0
	WDI_industry	HQER-SFA	hqer_induced	True	20	-113.7560183716763	hqer_induced sampler prototype	2.0
	WDI_industry	Quantile-SFA	hqer_induced	True	28	-132.51507878023128	hqer_induced sampler prototype	4.0
	WDI_industry	HQER-SFA	hqer_induced	True	19	-132.47126442118912	hqer_induced sampler prototype	2.0
	WDI_industry	Quantile-SFA	hqer_induced	True	27	-129.7195266517517	hqer_induced sampler prototype	4.0
	WDI_industry	HQER-SFA	hqer_induced	True	12	-129.6680225741515	hqer_induced sampler prototype	2.0
	WDI_industry	Quantile-SFA	hqer_induced	True	42	-119.16556889300506	hqer_induced sampler prototype	2.0
	WDI_industry	HQER-SFA	hqer_induced	True	24	-119.09915338531609	hqer_induced sampler prototype	2.0
	WDI_industry	Quantile-SFA	hqer_induced	True	24	-141.28396815475242	hqer_induced sampler prototype	2.0
	WDI_industry	HQER-SFA	hqer_induced	True	23	-141.24026190068412	hqer_induced sampler prototype	2.0
	WDI_industry	Quantile-SFA	hqer_induced	True	24	-97.2531165153742	hqer_induced sampler prototype	2.0
	WDI_industry	HQER-SFA	hqer_induced	True	26	-97.22359193541524	hqer_induced sampler prototype	2.0
	WDI_industry	Quantile-SFA	hqer_induced	True	19	-158.42808881990794	hqer_induced sampler prototype	4.0
	WDI_industry	HQER-SFA	hqer_induced	True	19	-158.39969332597104	hqer_induced sampler prototype	4.0
	WDI_industry	Quantile-SFA	hqer_induced	True	28	-171.42025307808848	hqer_induced sampler prototype	4.0
	WDI_industry	HQER-SFA	hqer_induced	True	28	-171.35559405699493	hqer_induced sampler prototype	2.0
	WDI_industry	Quantile-SFA	hqer_induced	True	14	-129.76731990068876	hqer_induced sampler prototype	2.0
	WDI_industry	HQER-SFA	hqer_induced	True	17	-129.73933499928754	hqer_induced sampler prototype	2.0
	WDI_industry	Quantile-SFA	hqer_induced	True	17	-130.4838910686554	hqer_induced sampler prototype	4.0
	WDI_industry	HQER-SFA	hqer_induced	True	27	-130.45559654099543	hqer_induced sampler prototype	2.0
	WDI_industry	Quantile-SFA	hqer_induced	True	24	-124.63672973316216	hqer_induced sampler prototype	4.0
	WDI_industry	HQER-SFA	hqer_induced	True	21	-124.5828418054075	hqer_induced sampler prototype	2.0
	WDI_industry	Quantile-SFA	hqer_induced	True	20	-129.22011450839767	hqer_induced sampler prototype	4.0
	WDI_industry	HQER-SFA	hqer_induced	True	26	-129.167343732537	hqer_induced sampler prototype	2.0
	WDI_industry	Quantile-SFA	hqer_induced	True	20	-156.06920483793655	hqer_induced sampler prototype	4.0
	WDI_industry	HQER-SFA	hqer_induced	True	24	-156.04617975182896	hqer_induced sampler prototype	2.0
	WDI_industry	Quantile-SFA	hqer_induced	True	29	-102.03831772300876	hqer_induced sampler prototype	4.0
	WDI_industry	HQER-SFA	hqer_induced	True	23	-102.03200321179472	hqer_induced sampler prototype	4.0
	WDI_industry	Quantile-SFA	hqer_induced	True	22	-143.67100076506222	hqer_induced sampler prototype	2.0
	WDI_industry	HQER-SFA	hqer_induced	True	23	-143.62747308828574	hqer_induced sampler prototype	4.0
	WDI_industry	Quantile-SFA	hqer_induced	True	24	-126.92808292052884	hqer_induced sampler prototype	4.0
	WDI_industry	HQER-SFA	hqer_induced	True	25	-126.88553867885642	hqer_induced sampler prototype	4.0
	WDI_industry	Quantile-SFA	hqer_induced	True	25	-142.59172402937998	hqer_induced sampler prototype	2.0
	WDI_industry	HQER-SFA	hqer_induced	True	24	-142.52893741682786	hqer_induced sampler prototype	4.0
	WDI_industry	Quantile-SFA	hqer_induced	True	15	-151.2942343361413	hqer_induced sampler prototype	2.0
	WDI_industry	HQER-SFA	hqer_induced	True	21	-151.235407253047	hqer_induced sampler prototype	2.0
	WDI_industry	Quantile-SFA	hqer_induced	True	23	-126.6266422686534	hqer_induced sampler prototype	4.0
	WDI_industry	HQER-SFA	hqer_induced	True	28	-126.57431529439194	hqer_induced sampler prototype	2.0
	WDI_industry	Quantile-SFA	hqer_induced	True	44	-97.63591187037176	hqer_induced sampler prototype	5.0
	WDI_industry	HQER-SFA	hqer_induced	True	22	-97.58964793545124	hqer_induced sampler prototype	4.0
	WDI_industry	Quantile-SFA	hqer_induced	True	19	-144.69492904299878	hqer_induced sampler prototype	4.0
	WDI_industry	HQER-SFA	hqer_induced	True	29	-144.60200136500788	hqer_induced sampler prototype	2.0
	WDI_industry	Quantile-SFA	hqer_induced	True	26	-127.88377266360251	hqer_induced sampler prototype	2.0
	WDI_industry	HQER-SFA	hqer_induced	True	20	-127.83848353835204	hqer_induced sampler prototype	4.0
	WDI_industry	Quantile-SFA	hqer_induced	True	26	-128.5853130319799	hqer_induced sampler prototype	4.0
	WDI_industry	HQER-SFA	hqer_induced	True	19	-128.52501304450684	hqer_induced sampler prototype	2.0
	WDI_industry	Quantile-SFA	hqer_induced	True	28	-132.45130026494144	hqer_induced sampler prototype	4.0
	WDI_industry	HQER-SFA	hqer_induced	True	26	-132.42034176310742	hqer_induced sampler prototype	4.0
	WDI_industry	Quantile-SFA	hqer_induced	True	39	-140.78519594262207	hqer_induced sampler prototype	5.0
	WDI_industry	HQER-SFA	hqer_induced	True	44	-140.76764086205512	hqer_induced sampler prototype	5.0
	WDI_industry	Quantile-SFA	hqer_induced	True	11	-164.1534526067412	hqer_induced sampler prototype	2.0
	WDI_industry	HQER-SFA	hqer_induced	True	30	-164.0905655591082	hqer_induced sampler prototype	4.0
	WDI_industry	Quantile-SFA	hqer_induced	True	23	-143.50572315256	hqer_induced sampler prototype	4.0
	WDI_industry	HQER-SFA	hqer_induced	True	19	-143.4676984239008	hqer_induced sampler prototype	4.0
	WDI_industry	Quantile-SFA	hqer_induced	True	25	-150.60874870402674	hqer_induced sampler prototype	4.0
	WDI_industry	HQER-SFA	hqer_induced	True	21	-150.55947333558896	hqer_induced sampler prototype	4.0
	WDI_industry	Quantile-SFA	hqer_induced	True	38	-159.85554861408357	hqer_induced sampler prototype	4.0
	WDI_industry	HQER-SFA	hqer_induced	True	41	-159.81543951380385	hqer_induced sampler prototype	2.0
	WDI_industry	Quantile-SFA	hqer_induced	True	24	-126.23981485596808	hqer_induced sampler prototype	2.0
	WDI_industry	HQER-SFA	hqer_induced	True	27	-126.20330431718368	hqer_induced sampler prototype	2.0
	WDI_industry	Quantile-SFA	hqer_induced	True	20	-152.32991860340945	hqer_induced sampler prototype	4.0
	WDI_industry	HQER-SFA	hqer_induced	True	23	-152.3037153748005	hqer_induced sampler prototype	4.0
	WDI_industry	Quantile-SFA	hqer_induced	True	20	-127.16370046667896	hqer_induced sampler prototype	4.0
	WDI_industry	HQER-SFA	hqer_induced	True	24	-127.12538592600932	hqer_induced sampler prototype	2.0
	WDI_industry	Quantile-SFA	hqer_induced	True	24	-151.9011686431164	hqer_induced sampler prototype	2.0
	WDI_industry	HQER-SFA	hqer_induced	True	20	-151.8409673648107	hqer_induced sampler prototype	2.0
	WDI_industry	Quantile-SFA	hqer_induced	True	26	-116.55933253715348	hqer_induced sampler prototype	4.0
	WDI_industry	HQER-SFA	hqer_induced	True	23	-116.54712927450116	hqer_induced sampler prototype	4.0
	WDI_industry	Quantile-SFA	hqer_induced	True	23	-111.0357839392552	hqer_induced sampler prototype	4.0
	WDI_industry	HQER-SFA	hqer_induced	True	27	-110.9912434989565	hqer_induced sampler prototype	4.0
	WDI_industry	Quantile-SFA	hqer_induced	True	26	-125.45142099006134	hqer_induced sampler prototype	4.0
	WDI_industry	HQER-SFA	hqer_induced	True	19	-125.42072916052471	hqer_induced sampler prototype	4.0
	WDI_industry	Quantile-SFA	hqer_induced	True	20	-140.4513027578619	hqer_induced sampler prototype	2.0
	WDI_industry	HQER-SFA	hqer_induced	True	12	-140.43037913263933	hqer_induced sampler prototype	4.0
	WDI_industry	Quantile-SFA	hqer_induced	True	24	-139.40843937082377	hqer_induced sampler prototype	4.0
	WDI_industry	HQER-SFA	hqer_induced	True	27	-139.37156648537825	hqer_induced sampler prototype	4.0
	WDI_industry	Quantile-SFA	hqer_induced	True	25	-175.82796201578262	hqer_induced sampler prototype	4.0
	WDI_industry	HQER-SFA	hqer_induced	True	24	-175.79209446181966	hqer_induced sampler prototype	4.0
	WDI_industry	Quantile-SFA	hqer_induced	True	22	-158.87224939739724	hqer_induced sampler prototype	4.0
	WDI_industry	HQER-SFA	hqer_induced	True	17	-158.84193689701584	hqer_induced sampler prototype	4.0
	WDI_industry	Quantile-SFA	hqer_induced	True	21	-123.02660829819824	hqer_induced sampler prototype	2.0
	WDI_industry	HQER-SFA	hqer_induced	True	25	-122.99009092134528	hqer_induced sampler prototype	2.0
	WDI_industry	Quantile-SFA	hqer_induced	True	15	-591.9955985207407	hqer_induced sampler prototype	2.0
	WDI_industry	HQER-SFA	hqer_induced	True	18	-591.776305693444	hqer_induced sampler prototype	2.0
	WDI_industry	Quantile-SFA	hqer_induced	True	14	-587.3922242102008	hqer_induced sampler prototype	2.0
	WDI_industry	HQER-SFA	hqer_induced	True	14	-587.1696813575181	hqer_induced sampler prototype	2.0
	WDI_industry	Quantile-SFA	hqer_induced	True	19	-559.3258474178274	hqer_induced sampler prototype	4.0
	WDI_industry	HQER-SFA	hqer_induced	True	25	-559.1540946023582	hqer_induced sampler prototype	4.0
	WDI_industry	Quantile-SFA	hqer_induced	True	25	-445.9714053249305	hqer_induced sampler prototype	2.0
	WDI_industry	HQER-SFA	hqer_induced	True	19	-445.8617753913138	hqer_induced sampler prototype	2.0
	WDI_industry	Quantile-SFA	hqer_induced	True	25	-527.1436061576267	hqer_induced sampler prototype	4.0
	WDI_industry	HQER-SFA	hqer_induced	True	21	-526.9652287281353	hqer_induced sampler prototype	4.0
	WDI_industry	Quantile-SFA	hqer_induced	True	21	-537.5223446424313	hqer_induced sampler prototype	2.0
	WDI_industry	HQER-SFA	hqer_induced	True	20	-537.3881544278327	hqer_induced sampler prototype	2.0
	WDI_industry	Quantile-SFA	hqer_induced	True	20	-496.3979155329467	hqer_induced sampler prototype	2.0
	WDI_industry	HQER-SFA	hqer_induced	True	18	-496.2860804658086	hqer_induced sampler prototype	4.0
	WDI_industry	Quantile-SFA	hqer_induced	True	14	-524.104740905402	hqer_induced sampler prototype	4.0
	WDI_industry	HQER-SFA	hqer_induced	True	13	-523.9351573269214	hqer_induced sampler prototype	2.0
	WDI_industry	Quantile-SFA	hqer_induced	True	20	-545.1020887748498	hqer_induced sampler prototype	4.0
	WDI_industry	HQER-SFA	hqer_induced	True	14	-544.9035930930419	hqer_induced sampler prototype	2.0
	WDI_industry	Quantile-SFA	hqer_induced	True	23	-517.9434258574715	hqer_induced sampler prototype	4.0
	WDI_industry	HQER-SFA	hqer_induced	True	31	-517.7399036932961	hqer_induced sampler prototype	2.0
	WDI_industry	Quantile-SFA	hqer_induced	True	19	-538.1750839832047	hqer_induced sampler prototype	4.0

experiment	dataset	model	scenario	converged	n_iter	loglik	warning_message	best_start_id
	WDI_industry	HQER-SFA	hqer_induced	True	20	-537.9910440004645	hqer_induced sampler prototype	4.0
	WDI_industry	Quantile-SFA	hqer_induced	True	17	-574.8526337621718	hqer_induced sampler prototype	4.0
	WDI_industry	HQER-SFA	hqer_induced	True	14	-574.692592443187	hqer_induced sampler prototype	2.0
	WDI_industry	Quantile-SFA	hqer_induced	True	19	-483.609063108068	hqer_induced sampler prototype	4.0
	WDI_industry	HQER-SFA	hqer_induced	True	9	-483.3953253971935	hqer_induced sampler prototype	2.0
	WDI_industry	Quantile-SFA	hqer_induced	True	19	-567.3169448243807	hqer_induced sampler prototype	4.0
	WDI_industry	HQER-SFA	hqer_induced	True	16	-567.0863318374943	hqer_induced sampler prototype	4.0
	WDI_industry	Quantile-SFA	hqer_induced	True	11	-593.5385923934559	hqer_induced sampler prototype	2.0
	WDI_industry	HQER-SFA	hqer_induced	True	20	-593.3751906360583	hqer_induced sampler prototype	4.0
	WDI_industry	Quantile-SFA	hqer_induced	True	18	-544.2599457825943	hqer_induced sampler prototype	4.0
	WDI_industry	HQER-SFA	hqer_induced	True	21	-544.1004095510841	hqer_induced sampler prototype	4.0
	WDI_industry	Quantile-SFA	hqer_induced	True	24	-577.6353500311818	hqer_induced sampler prototype	4.0
	WDI_industry	HQER-SFA	hqer_induced	True	21	-577.4638965563726	hqer_induced sampler prototype	4.0
	WDI_industry	Quantile-SFA	hqer_induced	True	16	-582.9803449951814	hqer_induced sampler prototype	4.0
	WDI_industry	HQER-SFA	hqer_induced	True	31	-582.8301133526414	hqer_induced sampler prototype	5.0
	WDI_industry	Quantile-SFA	hqer_induced	True	17	-519.6239267596626	hqer_induced sampler prototype	4.0
	WDI_industry	HQER-SFA	hqer_induced	True	20	-519.4924141183374	hqer_induced sampler prototype	4.0
	WDI_industry	Quantile-SFA	hqer_induced	True	15	-550.1898938398039	hqer_induced sampler prototype	4.0
	WDI_industry	HQER-SFA	hqer_induced	True	16	-550.0836790107641	hqer_induced sampler prototype	4.0
	WDI_industry	Quantile-SFA	hqer_induced	True	17	-607.5012559858565	hqer_induced sampler prototype	2.0
	WDI_industry	HQER-SFA	hqer_induced	True	23	-607.2936067786488	hqer_induced sampler prototype	2.0
	WDI_industry	Quantile-SFA	hqer_induced	True	19	-547.456273512808	hqer_induced sampler prototype	4.0
	WDI_industry	HQER-SFA	hqer_induced	True	20	-547.3581831750267	hqer_induced sampler prototype	2.0
	WDI_industry	Quantile-SFA	hqer_induced	True	21	-493.5105042657736	hqer_induced sampler prototype	4.0
	WDI_industry	HQER-SFA	hqer_induced	True	18	-493.3575431523928	hqer_induced sampler prototype	4.0
	WDI_industry	Quantile-SFA	hqer_induced	True	25	-522.1418564188386	hqer_induced sampler prototype	4.0
	WDI_industry	HQER-SFA	hqer_induced	True	20	-522.0204781795887	hqer_induced sampler prototype	4.0
	WDI_industry	Quantile-SFA	hqer_induced	True	34	-547.7707901806705	hqer_induced sampler prototype	5.0
	WDI_industry	HQER-SFA	hqer_induced	True	20	-547.6276522449709	hqer_induced sampler prototype	4.0
	WDI_industry	Quantile-SFA	hqer_induced	True	27	-527.8335035211526	hqer_induced sampler prototype	4.0
	WDI_industry	HQER-SFA	hqer_induced	True	23	-527.5864439891004	hqer_induced sampler prototype	4.0
	WDI_industry	Quantile-SFA	hqer_induced	True	21	-566.7422883809766	hqer_induced sampler prototype	4.0
	WDI_industry	HQER-SFA	hqer_induced	True	12	-566.5418035450618	hqer_induced sampler prototype	2.0
	WDI_industry	Quantile-SFA	hqer_induced	True	21	-590.6128410185461	hqer_induced sampler prototype	4.0
	WDI_industry	HQER-SFA	hqer_induced	True	24	-590.4748502883241	hqer_induced sampler prototype	4.0
	WDI_industry	Quantile-SFA	hqer_induced	True	21	-608.4129351284853	hqer_induced sampler prototype	2.0
	WDI_industry	HQER-SFA	hqer_induced	True	21	-608.2616092660518	hqer_induced sampler prototype	4.0
	WDI_industry	Quantile-SFA	hqer_induced	True	22	-460.12093291686153	hqer_induced sampler prototype	2.0
	WDI_industry	HQER-SFA	hqer_induced	True	20	-459.9785198817549	hqer_induced sampler prototype	2.0
	WDI_industry	Quantile-SFA	hqer_induced	True	36	-530.6904359250311	hqer_induced sampler prototype	5.0
	WDI_industry	HQER-SFA	hqer_induced	True	20	-530.561789334008	hqer_induced sampler prototype	4.0
	WDI_industry	Quantile-SFA	hqer_induced	True	24	-519.7922554366197	hqer_induced sampler prototype	4.0
	WDI_industry	HQER-SFA	hqer_induced	True	17	-519.5868897803618	hqer_induced sampler prototype	2.0
	WDI_industry	Quantile-SFA	hqer_induced	True	16	-551.3083887040999	hqer_induced sampler prototype	4.0
	WDI_industry	HQER-SFA	hqer_induced	True	16	-551.1369636636516	hqer_induced sampler prototype	2.0
	WDI_industry	Quantile-SFA	hqer_induced	True	13	-542.4729434092819	hqer_induced sampler prototype	2.0
	WDI_industry	HQER-SFA	hqer_induced	True	22	-542.2872031314313	hqer_induced sampler prototype	4.0
	WDI_industry	Quantile-SFA	hqer_induced	True	20	-560.7360106652743	hqer_induced sampler prototype	4.0
	WDI_industry	HQER-SFA	hqer_induced	True	20	-560.557946944496	hqer_induced sampler prototype	4.0
	WDI_industry	Quantile-SFA	hqer_induced	True	23	-513.6610841466401	hqer_induced sampler prototype	2.0
	WDI_industry	HQER-SFA	hqer_induced	True	17	-513.5403781489122	hqer_induced sampler prototype	2.0
	WDI_industry	Quantile-SFA	hqer_induced	True	22	-593.3558333847329	hqer_induced sampler prototype	4.0
	WDI_industry	HQER-SFA	hqer_induced	True	20	-593.2279695376658	hqer_induced sampler prototype	4.0
	WDI_industry	Quantile-SFA	hqer_induced	True	19	-507.6410380995184	hqer_induced sampler prototype	4.0
	WDI_industry	HQER-SFA	hqer_induced	True	21	-507.52854172286345	hqer_induced sampler prototype	2.0
	WDI_industry	Quantile-SFA	hqer_induced	True	21	-577.2977035494587	hqer_induced sampler prototype	4.0
	WDI_industry	HQER-SFA	hqer_induced	True	20	-577.1109008380147	hqer_induced sampler prototype	4.0
	WDI_industry	Quantile-SFA	hqer_induced	True	15	-498.25534717823416	hqer_induced sampler prototype	4.0
	WDI_industry	HQER-SFA	hqer_induced	True	27	-498.0896897074524	hqer_induced sampler prototype	2.0
	WDI_industry	Quantile-SFA	hqer_induced	True	15	-563.3254285414681	hqer_induced sampler prototype	4.0
	WDI_industry	HQER-SFA	hqer_induced	True	32	-563.152227377437	hqer_induced sampler prototype	5.0
	WDI_industry	Quantile-SFA	hqer_induced	True	17	-558.6561763686199	hqer_induced sampler prototype	2.0
	WDI_industry	HQER-SFA	hqer_induced	True	18	-558.4785752242793	hqer_induced sampler prototype	2.0
	WDI_industry	Quantile-SFA	hqer_induced	True	15	-554.8631943526291	hqer_induced sampler prototype	2.0
	WDI_industry	HQER-SFA	hqer_induced	True	24	-554.6929515886807	hqer_induced sampler prototype	4.0
	WDI_industry	Quantile-SFA	hqer_induced	True	13	-602.9024913241356	hqer_induced sampler prototype	2.0
	WDI_industry	HQER-SFA	hqer_induced	True	13	-602.7191269358176	hqer_induced sampler prototype	2.0
	WDI_industry	Quantile-SFA	hqer_induced	True	22	-556.3584899329115	hqer_induced sampler prototype	4.0
	WDI_industry	HQER-SFA	hqer_induced	True	18	-556.2016534217769	hqer_induced sampler prototype	4.0
	WDI_industry	Quantile-SFA	hqer_induced	True	16	-552.1492009408878	hqer_induced sampler prototype	2.0
	WDI_industry	HQER-SFA	hqer_induced	True	19	-552.0287750958004	hqer_induced sampler prototype	2.0
	WDI_industry	Quantile-SFA	hqer_induced	True	12	-522.2213182780381	hqer_induced sampler prototype	2.0
	WDI_industry	HQER-SFA	hqer_induced	True	21	-522.0102098281626	hqer_induced sampler prototype	2.0
	WDI_industry	Quantile-SFA	hqer_induced	True	9	-566.4461609459253	hqer_induced sampler prototype	2.0
	WDI_industry	HQER-SFA	hqer_induced	True	21	-566.2564285755053	hqer_induced sampler prototype	4.0
	WDI_industry	Quantile-SFA	hqer_induced	True	18	-592.3199379972923	hqer_induced sampler prototype	4.0
	WDI_industry	HQER-SFA	hqer_induced	True	16	-592.1382350700778	hqer_induced sampler prototype	2.0
	WDI_industry	Quantile-SFA	hqer_induced	True	20	-575.1043962436843	hqer_induced sampler prototype	2.0
	WDI_industry	HQER-SFA	hqer_induced	True	16	-575.0133835909578	hqer_induced sampler prototype	4.0
	WDI_industry	Quantile-SFA	hqer_induced	True	19	-483.8679197472124	hqer_induced sampler prototype	2.0
	WDI_industry	HQER-SFA	hqer_induced	True	20	-483.72386235489967	hqer_induced sampler prototype	4.0
	WDI_industry	Quantile-SFA	hqer_induced	True	21	-499.6232191600972	hqer_induced sampler prototype	2.0
	WDI_industry	HQER-SFA	hqer_induced	True	17	-499.41546106286114	hqer_induced sampler prototype	4.0
	WDI_industry	Quantile-SFA	hqer_induced	True	17	-570.6857015865928	hqer_induced sampler prototype	4.0
	WDI_industry	HQER-SFA	hqer_induced	True	14	-570.5758804242581	hqer_induced sampler prototype	2.0
	WDI_industry	Quantile-SFA	hqer_induced	True	21	-521.0187388537651	hqer_induced sampler prototype	4.0
	WDI_industry	HQER-SFA	hqer_induced	True	20	-520.8689571927498	hqer_induced sampler prototype	4.0
	WDI_industry	Quantile-SFA	hqer_induced	True	22	-518.1475822170136	hqer_induced sampler prototype	2.0
	WDI_industry	HQER-SFA	hqer_induced	True	19	-518.069824787576	hqer_induced sampler prototype	2.0
	WDI_industry	Quantile-SFA	hqer_induced	True	14	-543.2892131156846	hqer_induced sampler prototype	2.0
	WDI_industry	HQER-SFA	hqer_induced	True	14	-543.0520604064718	hqer_induced sampler prototype	2.0
	WDI_industry	Quantile-SFA	hqer_induced	True	24	-576.8282941315483	hqer_induced sampler prototype	4.0
	WDI_industry	HQER-SFA	hqer_induced	True	26	-576.6658854729278	hqer_induced sampler prototype	4.0
	WDI_industry	Quantile-SFA	hqer_induced	True	20	-530.1140650407256	hqer_induced sampler prototype	4.0
	WDI_industry	HQER-SFA	hqer_induced	True	16	-529.9234738098351	hqer_induced sampler prototype	2.0
	WDI_industry	Quantile-SFA	hqer_induced	True	18	-510.94193583242486	hqer_induced sampler prototype	2.0
	WDI_industry	HQER-SFA	hqer_induced	True	19	-510.8094841950537	hqer_induced sampler prototype	4.0
	WDI_industry	Quantile-SFA	hqer_induced	True	23	-613.0767245977141	hqer_induced sampler prototype	4.0
	WDI_industry	HQER-SFA	hqer_induced	True	18	-612.8236863124346	hqer_induced sampler prototype	4.0
	WDI_industry	Quantile-SFA	hqer_induced	True	18	-616.3535985407118	hqer_induced sampler prototype	2.0
	WDI_industry	HQER-SFA	hqer_induced	True	20	-616.1643358234132	hqer_induced sampler prototype	4.0
	WDI_industry	Quantile-SFA	hqer_induced	True	17	-447.0380854509969	hqer_induced sampler prototype	4.0
	WDI_industry	HQER-SFA	hqer_induced	True	20	-446.9005882246894	hqer_induced sampler prototype	4.0

experiment	dataset	model	scenario	converged	n_iter	loglik	warning_message	best_start_id
	WDI_industry	Quantile-SFA	hqer_induced	True	15	-589.9912882426867	hqer_induced sampler prototype	2.0
	WDI_industry	HQER-SFA	hqer_induced	True	13	-589.8019803658913	hqer_induced sampler prototype	2.0
	WDI_industry	Quantile-SFA	hqer_induced	True	19	-554.4642312528333	hqer_induced sampler prototype	4.0
	WDI_industry	HQER-SFA	hqer_induced	True	15	-554.2859913773415	hqer_induced sampler prototype	2.0
	WDI_industry	Quantile-SFA	hqer_induced	True	16	-578.5674083943609	hqer_induced sampler prototype	2.0
	WDI_industry	HQER-SFA	hqer_induced	True	17	-578.4255740948506	hqer_induced sampler prototype	2.0
	WDI_industry	Quantile-SFA	hqer_induced	True	22	-557.7107105002653	hqer_induced sampler prototype	4.0
	WDI_industry	HQER-SFA	hqer_induced	True	21	-557.6168407773636	hqer_induced sampler prototype	4.0
	WDI_industry	Quantile-SFA	hqer_induced	True	25	-623.3945003919791	hqer_induced sampler prototype	4.0
	WDI_industry	HQER-SFA	hqer_induced	True	22	-623.2964403474489	hqer_induced sampler prototype	4.0
	WDI_industry	Quantile-SFA	hqer_induced	True	18	-522.679540365485	hqer_induced sampler prototype	4.0
	WDI_industry	HQER-SFA	hqer_induced	True	21	-522.5043776794508	hqer_induced sampler prototype	4.0
	WDI_industry	Quantile-SFA	hqer_induced	True	21	-481.348851247012	hqer_induced sampler prototype	4.0
	WDI_industry	HQER-SFA	hqer_induced	True	22	-481.1612575151922	hqer_induced sampler prototype	4.0
	WDI_industry	Quantile-SFA	hqer_induced	True	17	-590.1658491145358	hqer_induced sampler prototype	4.0
	WDI_industry	HQER-SFA	hqer_induced	True	18	-589.9365559561331	hqer_induced sampler prototype	4.0
	WDI_industry	Quantile-SFA	hqer_induced	True	16	-569.4052125611653	hqer_induced sampler prototype	2.0
	WDI_industry	HQER-SFA	hqer_induced	True	19	-569.2071843367025	hqer_induced sampler prototype	2.0
	WDI_industry	Quantile-SFA	hqer_induced	True	17	-526.2659579607068	hqer_induced sampler prototype	4.0
	WDI_industry	HQER-SFA	hqer_induced	True	17	-526.1821343839615	hqer_induced sampler prototype	4.0
	WDI_industry	Quantile-SFA	hqer_induced	True	14	-531.2423666109152	hqer_induced sampler prototype	4.0
	WDI_industry	HQER-SFA	hqer_induced	True	18	-531.1002766652541	hqer_induced sampler prototype	2.0
	WDI_industry	Quantile-SFA	hqer_induced	True	15	-478.0229114049546	hqer_induced sampler prototype	4.0
	WDI_industry	HQER-SFA	hqer_induced	True	23	-477.9097511904789	hqer_induced sampler prototype	2.0
	WDI_industry	Quantile-SFA	hqer_induced	True	25	-524.9736082740152	hqer_induced sampler prototype	4.0
	WDI_industry	HQER-SFA	hqer_induced	True	18	-524.7768055288373	hqer_induced sampler prototype	4.0
	WDI_industry	Quantile-SFA	hqer_induced	True	25	-483.51732974916	hqer_induced sampler prototype	4.0
	WDI_industry	HQER-SFA	hqer_induced	True	19	-483.3638655725786	hqer_induced sampler prototype	4.0
	WDI_industry	Quantile-SFA	hqer_induced	True	11	-506.6175730477282	hqer_induced sampler prototype	4.0
	WDI_industry	HQER-SFA	hqer_induced	True	14	-506.4783350154967	hqer_induced sampler prototype	2.0
	WDI_industry	Quantile-SFA	hqer_induced	True	19	-594.4592855697297	hqer_induced sampler prototype	2.0
	WDI_industry	HQER-SFA	hqer_induced	True	20	-594.2863285087218	hqer_induced sampler prototype	4.0
	WDI_industry	Quantile-SFA	hqer_induced	True	14	-557.593782992074	hqer_induced sampler prototype	2.0
	WDI_industry	HQER-SFA	hqer_induced	True	21	-557.4553583360645	hqer_induced sampler prototype	4.0
	WDI_industry	Quantile-SFA	hqer_induced	True	21	-545.5797148604349	hqer_induced sampler prototype	4.0
	WDI_industry	HQER-SFA	hqer_induced	True	20	-545.4665770477709	hqer_induced sampler prototype	4.0
	WDI_industry	Quantile-SFA	hqer_induced	True	19	-537.9920430115596	hqer_induced sampler prototype	4.0
	WDI_industry	HQER-SFA	hqer_induced	True	17	-537.8471141547806	hqer_induced sampler prototype	4.0
	WDI_industry	Quantile-SFA	hqer_induced	True	21	-521.068304929086	hqer_induced sampler prototype	4.0
	WDI_industry	HQER-SFA	hqer_induced	True	28	-520.8494752801112	hqer_induced sampler prototype	2.0
	WDI_industry	Quantile-SFA	hqer_induced	True	17	-552.3498189645104	hqer_induced sampler prototype	2.0
	WDI_industry	HQER-SFA	hqer_induced	True	25	-552.1684901490898	hqer_induced sampler prototype	4.0
	WDI_industry	Quantile-SFA	hqer_induced	True	10	-506.237068555687	hqer_induced sampler prototype	2.0
	WDI_industry	HQER-SFA	hqer_induced	True	21	-506.0401121337451	hqer_induced sampler prototype	4.0
	WDI_industry	Quantile-SFA	hqer_induced	True	22	-546.7327792606463	hqer_induced sampler prototype	2.0
	WDI_industry	HQER-SFA	hqer_induced	True	20	-546.6054419104444	hqer_induced sampler prototype	4.0
	WDI_industry	Quantile-SFA	hqer_induced	True	20	-496.3351521682595	hqer_induced sampler prototype	4.0
	WDI_industry	HQER-SFA	hqer_induced	True	21	-496.148836213058	hqer_induced sampler prototype	4.0
	WDI_industry	Quantile-SFA	hqer_induced	True	20	-513.1933882964408	hqer_induced sampler prototype	2.0
	WDI_industry	HQER-SFA	hqer_induced	True	19	-513.0375778090003	hqer_induced sampler prototype	2.0
	WDI_industry	Quantile-SFA	hqer_induced	True	19	-546.2621482998725	hqer_induced sampler prototype	4.0
	WDI_industry	HQER-SFA	hqer_induced	True	23	-546.0793807828907	hqer_induced sampler prototype	2.0
	WDI_industry	Quantile-SFA	hqer_induced	True	21	-565.8117462834973	hqer_induced sampler prototype	2.0
	WDI_industry	HQER-SFA	hqer_induced	True	19	-565.6543333413715	hqer_induced sampler prototype	4.0
	WDI_industry	Quantile-SFA	hqer_induced	True	18	-540.1316604678397	hqer_induced sampler prototype	2.0
	WDI_industry	HQER-SFA	hqer_induced	True	19	-540.0232255934028	hqer_induced sampler prototype	2.0
	WDI_industry	Quantile-SFA	hqer_induced	True	20	-607.70284659369	hqer_induced sampler prototype	4.0
	WDI_industry	HQER-SFA	hqer_induced	True	19	-607.5055331897088	hqer_induced sampler prototype	4.0
	WDI_industry	Quantile-SFA	hqer_induced	True	20	-459.0586044192203	hqer_induced sampler prototype	4.0
	WDI_industry	HQER-SFA	hqer_induced	True	39	-458.9542136870957	hqer_induced sampler prototype	5.0
	WDI_industry	Quantile-SFA	hqer_induced	True	20	-474.4400971165575	hqer_induced sampler prototype	4.0
	WDI_industry	HQER-SFA	hqer_induced	True	23	-474.2202342880743	hqer_induced sampler prototype	4.0
	WDI_industry	Quantile-SFA	hqer_induced	True	23	-588.902522719477	hqer_induced sampler prototype	4.0
	WDI_industry	HQER-SFA	hqer_induced	True	23	-588.7115768714305	hqer_induced sampler prototype	4.0
	WDI_industry	Quantile-SFA	hqer_induced	True	17	-475.4594508457475	hqer_induced sampler prototype	2.0
	WDI_industry	HQER-SFA	hqer_induced	True	11	-475.31249996810766	hqer_induced sampler prototype	4.0
	WDI_industry	Quantile-SFA	hqer_induced	True	21	-584.2007955085564	hqer_induced sampler prototype	4.0
	WDI_industry	HQER-SFA	hqer_induced	True	11	-584.0631115397608	hqer_induced sampler prototype	2.0
	WDI_industry	Quantile-SFA	hqer_induced	True	11	-563.6000732753424	hqer_induced sampler prototype	2.0
	WDI_industry	HQER-SFA	hqer_induced	True	9	-563.5293642129055	hqer_induced sampler prototype	2.0
	WDI_industry	Quantile-SFA	hqer_induced	True	18	-563.8231804332627	hqer_induced sampler prototype	4.0
	WDI_industry	HQER-SFA	hqer_induced	True	13	-563.5739005652823	hqer_induced sampler prototype	2.0
	WDI_industry	Quantile-SFA	hqer_induced	True	14	-542.1512748998991	hqer_induced sampler prototype	2.0
	WDI_industry	HQER-SFA	hqer_induced	True	19	-541.9667856155755	hqer_induced sampler prototype	4.0
	WDI_industry	Quantile-SFA	hqer_induced	True	18	-502.6212174471348	hqer_induced sampler prototype	2.0
	WDI_industry	HQER-SFA	hqer_induced	True	23	-502.4245370307808	hqer_induced sampler prototype	2.0
	WDI_industry	Quantile-SFA	hqer_induced	True	25	-131.16156784637337	hqer_induced sampler prototype	4.0
	WDI_industry	HQER-SFA	hqer_induced	True	23	-126.32070383875144	hqer_induced sampler prototype	4.0
	WDI_industry	Quantile-SFA	hqer_induced	True	29	-163.0575476434941	hqer_induced sampler prototype	2.0
	WDI_industry	HQER-SFA	hqer_induced	True	28	-160.28132130145383	hqer_induced sampler prototype	2.0
	WDI_industry	Quantile-SFA	hqer_induced	True	31	-138.97446956445427	hqer_induced sampler prototype	4.0
	WDI_industry	HQER-SFA	hqer_induced	True	20	-137.48957578089176	hqer_induced sampler prototype	2.0
	WDI_industry	Quantile-SFA	hqer_induced	True	18	-135.046327061239	hqer_induced sampler prototype	4.0
	WDI_industry	HQER-SFA	hqer_induced	True	24	-134.47155982672984	hqer_induced sampler prototype	2.0
	WDI_industry	Quantile-SFA	hqer_induced	True	12	-170.57558586946374	hqer_induced sampler prototype	4.0
	WDI_industry	HQER-SFA	hqer_induced	True	19	-168.89304769409398	hqer_induced sampler prototype	2.0
	WDI_industry	Quantile-SFA	hqer_induced	True	24	-155.95607569121267	hqer_induced sampler prototype	4.0
	WDI_industry	HQER-SFA	hqer_induced	True	18	-149.53090322454509	hqer_induced sampler prototype	4.0
	WDI_industry	Quantile-SFA	hqer_induced	True	13	-164.4934283907833	hqer_induced sampler prototype	4.0
	WDI_industry	HQER-SFA	hqer_induced	True	18	-161.81829879740215	hqer_induced sampler prototype	4.0
	WDI_industry	Quantile-SFA	hqer_induced	True	24	-171.19868331325358	hqer_induced sampler prototype	2.0
	WDI_industry	HQER-SFA	hqer_induced	True	19	-168.22228391924966	hqer_induced sampler prototype	4.0
	WDI_industry	Quantile-SFA	hqer_induced	True	22	-123.71441272115672	hqer_induced sampler prototype	2.0
	WDI_industry	HQER-SFA	hqer_induced	True	25	-121.06427609870993	hqer_induced sampler prototype	4.0
	WDI_industry	Quantile-SFA	hqer_induced	True	20	-156.72068598055876	hqer_induced sampler prototype	2.0
	WDI_industry	HQER-SFA	hqer_induced	True	12	-156.1127107281465	hqer_induced sampler prototype	2.0
	WDI_industry	Quantile-SFA	hqer_induced	True	23	-112.95417675003164	hqer_induced sampler prototype	4.0
	WDI_industry	HQER-SFA	hqer_induced	True	24	-110.97831429383892	hqer_induced sampler prototype	4.0
	WDI_industry	Quantile-SFA	hqer_induced	True	25	-151.1572952837329	hqer_induced sampler prototype	2.0
	WDI_industry	HQER-SFA	hqer_induced	True	19	-148.72273026039983	hqer_induced sampler prototype	4.0
	WDI_industry	Quantile-SFA	hqer_induced	True	19	-139.61805932020553	hqer_induced sampler prototype	2.0
	WDI_industry	HQER-SFA	hqer_induced	True	26	-137.8563401984267	hqer_induced sampler prototype	2.0
	WDI_industry	Quantile-SFA	hqer_induced	True	28	-158.94157074379314	hqer_induced sampler prototype	4.0

experiment	dataset	model	scenario	converged	n__iter	loglik	warning__message	best__start__id
	WDI__industry	HQER-SFA	hqer__induced	True	18	-156.43014734678917	hqer__induced sampler prototype	4.0
	WDI__industry	Quantile-SFA	hqer__induced	True	23	-136.6985869490639	hqer__induced sampler prototype	2.0
	WDI__industry	HQER-SFA	hqer__induced	True	19	-136.1230812988881	hqer__induced sampler prototype	2.0
	WDI__industry	Quantile-SFA	hqer__induced	True	29	-188.10506208183745	hqer__induced sampler prototype	4.0
	WDI__industry	HQER-SFA	hqer__induced	True	23	-186.5678285160751	hqer__induced sampler prototype	2.0
	WDI__industry	Quantile-SFA	hqer__induced	True	15	-126.93294256524428	hqer__induced sampler prototype	4.0
	WDI__industry	HQER-SFA	hqer__induced	True	25	-124.41016085148073	hqer__induced sampler prototype	4.0
	WDI__industry	Quantile-SFA	hqer__induced	True	14	-177.5379893238082	hqer__induced sampler prototype	2.0
	WDI__industry	HQER-SFA	hqer__induced	True	26	-174.94649925228998	hqer__induced sampler prototype	4.0
	WDI__industry	Quantile-SFA	hqer__induced	True	23	-124.47175128353352	hqer__induced sampler prototype	4.0
	WDI__industry	HQER-SFA	hqer__induced	True	15	-122.6718079064466	hqer__induced sampler prototype	2.0
	WDI__industry	Quantile-SFA	hqer__induced	True	26	-179.9443623747825	hqer__induced sampler prototype	4.0
	WDI__industry	HQER-SFA	hqer__induced	True	21	-177.02872639046376	hqer__induced sampler prototype	4.0
	WDI__industry	Quantile-SFA	hqer__induced	True	24	-180.5100078857316	hqer__induced sampler prototype	4.0
	WDI__industry	HQER-SFA	hqer__induced	True	26	-176.57740390617568	hqer__induced sampler prototype	4.0
	WDI__industry	Quantile-SFA	hqer__induced	True	29	-177.7717362678539	hqer__induced sampler prototype	4.0
	WDI__industry	HQER-SFA	hqer__induced	True	20	-173.6181418528949	hqer__induced sampler prototype	4.0
	WDI__industry	Quantile-SFA	hqer__induced	True	34	-184.2036671740532	hqer__induced sampler prototype	2.0
	WDI__industry	HQER-SFA	hqer__induced	True	25	-177.23496162046348	hqer__induced sampler prototype	4.0
	WDI__industry	Quantile-SFA	hqer__induced	True	27	-171.6768246415844	hqer__induced sampler prototype	2.0
	WDI__industry	HQER-SFA	hqer__induced	True	19	-169.31432139872277	hqer__induced sampler prototype	4.0
	WDI__industry	Quantile-SFA	hqer__induced	True	18	-108.99878901467478	hqer__induced sampler prototype	2.0
	WDI__industry	HQER-SFA	hqer__induced	True	10	-109.89694409226418	hqer__induced sampler prototype	2.0
	WDI__industry	Quantile-SFA	hqer__induced	True	25	-178.1851016068784	hqer__induced sampler prototype	2.0
	WDI__industry	HQER-SFA	hqer__induced	True	27	-175.01639981522396	hqer__induced sampler prototype	4.0
	WDI__industry	Quantile-SFA	hqer__induced	True	15	-175.94665584557248	hqer__induced sampler prototype	4.0
	WDI__industry	HQER-SFA	hqer__induced	True	13	-173.73164303196057	hqer__induced sampler prototype	4.0
	WDI__industry	Quantile-SFA	hqer__induced	True	19	-134.88216053917768	hqer__induced sampler prototype	4.0
	WDI__industry	HQER-SFA	hqer__induced	True	25	-132.00590126672247	hqer__induced sampler prototype	4.0
	WDI__industry	Quantile-SFA	hqer__induced	True	24	-117.83638757949171	hqer__induced sampler prototype	2.0
	WDI__industry	HQER-SFA	hqer__induced	True	25	-118.71683578986308	hqer__induced sampler prototype	2.0
	WDI__industry	Quantile-SFA	hqer__induced	True	22	-140.1837542373118	hqer__induced sampler prototype	4.0
	WDI__industry	HQER-SFA	hqer__induced	True	9	-138.7381793611673	hqer__induced sampler prototype	2.0
	WDI__industry	Quantile-SFA	hqer__induced	True	23	-137.84810952409583	hqer__induced sampler prototype	4.0
	WDI__industry	HQER-SFA	hqer__induced	True	16	-137.3551221672155	hqer__induced sampler prototype	2.0
	WDI__industry	Quantile-SFA	hqer__induced	True	27	-162.07117174083558	hqer__induced sampler prototype	2.0
	WDI__industry	HQER-SFA	hqer__induced	True	7	-160.38799082749375	hqer__induced sampler prototype	2.0
	WDI__industry	Quantile-SFA	hqer__induced	True	25	-119.1219271374704	hqer__induced sampler prototype	2.0
	WDI__industry	HQER-SFA	hqer__induced	True	13	-118.84100388233684	hqer__induced sampler prototype	2.0
	WDI__industry	Quantile-SFA	hqer__induced	True	28	-146.94752681685148	hqer__induced sampler prototype	4.0
	WDI__industry	HQER-SFA	hqer__induced	True	21	-144.20744281927412	hqer__induced sampler prototype	4.0
	WDI__industry	Quantile-SFA	hqer__induced	True	25	-153.11981273459847	hqer__induced sampler prototype	2.0
	WDI__industry	HQER-SFA	hqer__induced	True	23	-150.40360266184106	hqer__induced sampler prototype	4.0
	WDI__industry	Quantile-SFA	hqer__induced	True	11	-154.75547521369916	hqer__induced sampler prototype	2.0
	WDI__industry	HQER-SFA	hqer__induced	True	8	-152.79794992048127	hqer__induced sampler prototype	2.0
	WDI__industry	Quantile-SFA	hqer__induced	True	17	-181.5782358421665	hqer__induced sampler prototype	2.0
	WDI__industry	HQER-SFA	hqer__induced	True	12	-180.2988175841101	hqer__induced sampler prototype	2.0
	WDI__industry	Quantile-SFA	hqer__induced	True	20	-176.35260401079296	hqer__induced sampler prototype	4.0
	WDI__industry	HQER-SFA	hqer__induced	True	20	-176.1107821911852	hqer__induced sampler prototype	2.0
	WDI__industry	Quantile-SFA	hqer__induced	True	22	-138.1641352490638	hqer__induced sampler prototype	2.0
	WDI__industry	HQER-SFA	hqer__induced	True	29	-132.75152912457378	hqer__induced sampler prototype	4.0
	WDI__industry	Quantile-SFA	hqer__induced	True	17	-170.9125751860534	hqer__induced sampler prototype	2.0
	WDI__industry	HQER-SFA	hqer__induced	True	10	-168.84985116860253	hqer__induced sampler prototype	4.0
	WDI__industry	Quantile-SFA	hqer__induced	True	26	-119.50285837736232	hqer__induced sampler prototype	2.0
	WDI__industry	HQER-SFA	hqer__induced	True	25	-117.19043049556696	hqer__induced sampler prototype	2.0
	WDI__industry	Quantile-SFA	hqer__induced	True	10	-164.47134441722363	hqer__induced sampler prototype	2.0
	WDI__industry	HQER-SFA	hqer__induced	True	26	-160.20297960408635	hqer__induced sampler prototype	4.0
	WDI__industry	Quantile-SFA	hqer__induced	True	26	-178.86324248744083	hqer__induced sampler prototype	2.0
	WDI__industry	HQER-SFA	hqer__induced	True	22	-176.20122948812903	hqer__induced sampler prototype	4.0
	WDI__industry	Quantile-SFA	hqer__induced	True	22	-191.41988164507376	hqer__induced sampler prototype	4.0
	WDI__industry	HQER-SFA	hqer__induced	True	26	-189.8869091309564	hqer__induced sampler prototype	2.0
	WDI__industry	Quantile-SFA	hqer__induced	True	24	-196.8665683801995	hqer__induced sampler prototype	4.0
	WDI__industry	HQER-SFA	hqer__induced	True	26	-192.2086820518539	hqer__induced sampler prototype	4.0
	WDI__industry	Quantile-SFA	hqer__induced	True	16	-144.0411826329948	hqer__induced sampler prototype	2.0
	WDI__industry	HQER-SFA	hqer__induced	True	20	-144.66582575501155	hqer__induced sampler prototype	2.0
	WDI__industry	Quantile-SFA	hqer__induced	True	23	-181.8117058626329	hqer__induced sampler prototype	4.0
	WDI__industry	HQER-SFA	hqer__induced	True	22	-178.5235039171706	hqer__induced sampler prototype	4.0
	WDI__industry	Quantile-SFA	hqer__induced	True	26	-130.90983949480272	hqer__induced sampler prototype	4.0
	WDI__industry	HQER-SFA	hqer__induced	True	27	-129.93586109365992	hqer__induced sampler prototype	2.0
	WDI__industry	Quantile-SFA	hqer__induced	True	20	-166.23725688231792	hqer__induced sampler prototype	4.0
	WDI__industry	HQER-SFA	hqer__induced	True	27	-165.51136319952417	hqer__induced sampler prototype	4.0
	WDI__industry	Quantile-SFA	hqer__induced	True	25	-161.39186011198487	hqer__induced sampler prototype	4.0
	WDI__industry	HQER-SFA	hqer__induced	True	13	-160.22303529716638	hqer__induced sampler prototype	2.0
	WDI__industry	Quantile-SFA	hqer__induced	True	21	-180.16189011440903	hqer__induced sampler prototype	4.0
	WDI__industry	HQER-SFA	hqer__induced	True	14	-176.7054623935563	hqer__induced sampler prototype	4.0
	WDI__industry	Quantile-SFA	hqer__induced	True	25	-113.26172315931908	hqer__induced sampler prototype	4.0
	WDI__industry	HQER-SFA	hqer__induced	True	14	-113.05871760240802	hqer__induced sampler prototype	2.0
	WDI__industry	Quantile-SFA	hqer__induced	True	18	-140.58992218823195	hqer__induced sampler prototype	2.0
	WDI__industry	HQER-SFA	hqer__induced	True	21	-139.10386159674206	hqer__induced sampler prototype	2.0
	WDI__industry	Quantile-SFA	hqer__induced	True	10	-158.9727793912392	hqer__induced sampler prototype	2.0
	WDI__industry	HQER-SFA	hqer__induced	True	10	-157.93154732178934	hqer__induced sampler prototype	2.0
	WDI__industry	Quantile-SFA	hqer__induced	True	27	-111.15241106416886	hqer__induced sampler prototype	2.0
	WDI__industry	HQER-SFA	hqer__induced	True	17	-110.01386404413172	hqer__induced sampler prototype	2.0
	WDI__industry	Quantile-SFA	hqer__induced	True	18	-168.9626383745794	hqer__induced sampler prototype	4.0
	WDI__industry	HQER-SFA	hqer__induced	True	18	-166.73040224251014	hqer__induced sampler prototype	4.0
	WDI__industry	Quantile-SFA	hqer__induced	True	20	-144.3441042070273	hqer__induced sampler prototype	2.0
	WDI__industry	HQER-SFA	hqer__induced	True	22	-142.456195675316	hqer__induced sampler prototype	2.0
	WDI__industry	Quantile-SFA	hqer__induced	True	21	-171.45104843403266	hqer__induced sampler prototype	2.0
	WDI__industry	HQER-SFA	hqer__induced	True	23	-169.36065942537454	hqer__induced sampler prototype	2.0
	WDI__industry	Quantile-SFA	hqer__induced	True	23	-133.15197114345844	hqer__induced sampler prototype	2.0
	WDI__industry	HQER-SFA	hqer__induced	True	25	-130.92073070638548	hqer__induced sampler prototype	4.0
	WDI__industry	Quantile-SFA	hqer__induced	True	19	-153.39326033292065	hqer__induced sampler prototype	2.0
	WDI__industry	HQER-SFA	hqer__induced	True	27	-151.3694729049196	hqer__induced sampler prototype	2.0
	WDI__industry	Quantile-SFA	hqer__induced	True	28	-139.7230978739413	hqer__induced sampler prototype	4.0
	WDI__industry	HQER-SFA	hqer__induced	True	20	-135.34773193034871	hqer__induced sampler prototype	2.0
	WDI__industry	Quantile-SFA	hqer__induced	True	28	-148.5171631264422	hqer__induced sampler prototype	4.0
	WDI__industry	HQER-SFA	hqer__induced	True	32	-145.5466378665726	hqer__induced sampler prototype	4.0
	WDI__industry	Quantile-SFA	hqer__induced	True	20	-164.68156968107667	hqer__induced sampler prototype	2.0
	WDI__industry	HQER-SFA	hqer__induced	True	31	-160.34279409762888	hqer__induced sampler prototype	4.0
	WDI__industry	Quantile-SFA	hqer__induced	True	19	-141.58365866132254	hqer__induced sampler prototype	4.0
	WDI__industry	HQER-SFA	hqer__induced	True	19	-137.8318003280868	hqer__induced sampler prototype	4.0
	WDI__industry	Quantile-SFA	hqer__induced	True	17	-140.30596183657667	hqer__induced sampler prototype	2.0
	WDI__industry	HQER-SFA	hqer__induced	True	24	-140.12892351885478	hqer__induced sampler prototype	2.0

experiment	dataset	model	scenario	converged	n_iter	loglik	warning_message	best_start_id
	WDI_industry	Quantile-SFA	hqer_induced	True	20	-160.9831350454681	hqer_induced sampler prototype	2.0
	WDI_industry	HQER-SFA	hqer_induced	True	25	-158.5093439210629	hqer_induced sampler prototype	4.0
	WDI_industry	Quantile-SFA	hqer_induced	True	26	-166.59258589501331	hqer_induced sampler prototype	2.0
	WDI_industry	HQER-SFA	hqer_induced	True	16	-162.07113882825848	hqer_induced sampler prototype	4.0
	WDI_industry	Quantile-SFA	hqer_induced	True	16	-172.52052099533424	hqer_induced sampler prototype	2.0
	WDI_industry	HQER-SFA	hqer_induced	True	24	-168.62516811073948	hqer_induced sampler prototype	4.0
	WDI_industry	Quantile-SFA	hqer_induced	True	19	-142.5405248613298	hqer_induced sampler prototype	4.0
	WDI_industry	HQER-SFA	hqer_induced	True	24	-137.6545709552916	hqer_induced sampler prototype	4.0
	WDI_industry	Quantile-SFA	hqer_induced	True	15	-138.50108801298066	hqer_induced sampler prototype	2.0
	WDI_industry	HQER-SFA	hqer_induced	True	18	-136.37249412186165	hqer_induced sampler prototype	2.0
	WDI_industry	Quantile-SFA	hqer_induced	True	16	-139.501531615715	hqer_induced sampler prototype	2.0
	WDI_industry	HQER-SFA	hqer_induced	True	25	-137.739017583225	hqer_induced sampler prototype	2.0
	WDI_industry	Quantile-SFA	hqer_induced	True	25	-181.17466161791447	hqer_induced sampler prototype	4.0
	WDI_industry	HQER-SFA	hqer_induced	True	9	-181.57732010167572	hqer_induced sampler prototype	2.0
	WDI_industry	Quantile-SFA	hqer_induced	True	24	-155.73873871914955	hqer_induced sampler prototype	4.0
	WDI_industry	HQER-SFA	hqer_induced	True	24	-153.18583481198888	hqer_induced sampler prototype	4.0
	WDI_industry	Quantile-SFA	hqer_induced	True	25	-148.17331419350083	hqer_induced sampler prototype	4.0
	WDI_industry	HQER-SFA	hqer_induced	True	13	-147.70845545764047	hqer_induced sampler prototype	2.0
	WDI_industry	Quantile-SFA	hqer_induced	True	21	-162.50533989266947	hqer_induced sampler prototype	4.0
	WDI_industry	HQER-SFA	hqer_induced	True	19	-156.7597045482812	hqer_induced sampler prototype	4.0
	WDI_industry	Quantile-SFA	hqer_induced	True	23	-163.59655409784114	hqer_induced sampler prototype	4.0
	WDI_industry	HQER-SFA	hqer_induced	True	19	-160.65745421723423	hqer_induced sampler prototype	4.0
	WDI_industry	Quantile-SFA	hqer_induced	True	21	-162.7707658484051	hqer_induced sampler prototype	2.0
	WDI_industry	HQER-SFA	hqer_induced	True	20	-160.90203539904883	hqer_induced sampler prototype	2.0
	WDI_industry	Quantile-SFA	hqer_induced	True	27	-165.005254004819	hqer_induced sampler prototype	4.0
	WDI_industry	HQER-SFA	hqer_induced	True	24	-163.39934709891332	hqer_induced sampler prototype	2.0
	WDI_industry	Quantile-SFA	hqer_induced	True	22	-148.14739973401444	hqer_induced sampler prototype	2.0
	WDI_industry	HQER-SFA	hqer_induced	True	24	-146.81089532413503	hqer_induced sampler prototype	2.0
	WDI_industry	Quantile-SFA	hqer_induced	True	29	-176.4730475293918	hqer_induced sampler prototype	4.0
	WDI_industry	HQER-SFA	hqer_induced	True	16	-174.30165385749353	hqer_induced sampler prototype	2.0
	WDI_industry	Quantile-SFA	hqer_induced	True	30	-168.38700022914406	hqer_induced sampler prototype	4.0
	WDI_industry	HQER-SFA	hqer_induced	True	26	-166.3184702573364	hqer_induced sampler prototype	4.0
	WDI_industry	Quantile-SFA	hqer_induced	True	30	-171.86044660817427	hqer_induced sampler prototype	4.0
	WDI_industry	HQER-SFA	hqer_induced	True	28	-168.73736019215517	hqer_induced sampler prototype	4.0
	WDI_industry	Quantile-SFA	hqer_induced	True	12	-179.0826954591065	hqer_induced sampler prototype	2.0
	WDI_industry	HQER-SFA	hqer_induced	True	22	-176.33896118694423	hqer_induced sampler prototype	4.0
	WDI_industry	Quantile-SFA	hqer_induced	True	10	-168.89851444969386	hqer_induced sampler prototype	2.0
	WDI_industry	HQER-SFA	hqer_induced	True	15	-168.7411388760938	hqer_induced sampler prototype	2.0
	WDI_industry	Quantile-SFA	hqer_induced	True	28	-153.4224803104143	hqer_induced sampler prototype	4.0
	WDI_industry	HQER-SFA	hqer_induced	True	23	-149.73925401199986	hqer_induced sampler prototype	4.0
	WDI_industry	Quantile-SFA	hqer_induced	True	20	-166.65077118740868	hqer_induced sampler prototype	2.0
	WDI_industry	HQER-SFA	hqer_induced	True	18	-166.31406480142556	hqer_induced sampler prototype	2.0
	WDI_industry	Quantile-SFA	hqer_induced	True	23	-131.1005267089309	hqer_induced sampler prototype	4.0
	WDI_industry	HQER-SFA	hqer_induced	True	34	-128.37984740412216	hqer_induced sampler prototype	4.0
	WDI_industry	Quantile-SFA	hqer_induced	True	35	-141.31452425725345	hqer_induced sampler prototype	4.0
	WDI_industry	HQER-SFA	hqer_induced	True	29	-140.2435959339012	hqer_induced sampler prototype	2.0
	WDI_industry	Quantile-SFA	hqer_induced	True	25	-147.87624084463823	hqer_induced sampler prototype	4.0
	WDI_industry	HQER-SFA	hqer_induced	True	25	-148.0666952236096	hqer_induced sampler prototype	2.0
	WDI_industry	Quantile-SFA	hqer_induced	True	18	-170.65163525791863	hqer_induced sampler prototype	2.0
	WDI_industry	HQER-SFA	hqer_induced	True	34	-167.6914117650854	hqer_induced sampler prototype	4.0
	WDI_industry	Quantile-SFA	hqer_induced	True	23	-136.1872172925943	hqer_induced sampler prototype	2.0
	WDI_industry	HQER-SFA	hqer_induced	True	27	-133.91043311208938	hqer_induced sampler prototype	4.0
	WDI_industry	Quantile-SFA	hqer_induced	True	13	-155.56399490401728	hqer_induced sampler prototype	2.0
	WDI_industry	HQER-SFA	hqer_induced	True	47	-149.05955662429454	hqer_induced sampler prototype	2.0
	WDI_industry	Quantile-SFA	hqer_induced	True	28	-146.6683117288893	hqer_induced sampler prototype	4.0
	WDI_industry	HQER-SFA	hqer_induced	True	29	-141.85872796535492	hqer_induced sampler prototype	4.0
	WDI_industry	Quantile-SFA	hqer_induced	True	21	-144.67302050120193	hqer_induced sampler prototype	4.0
	WDI_industry	HQER-SFA	hqer_induced	True	8	-143.44449010277128	hqer_induced sampler prototype	2.0
	WDI_industry	Quantile-SFA	hqer_induced	True	20	-154.0936866904698	hqer_induced sampler prototype	4.0
	WDI_industry	HQER-SFA	hqer_induced	True	31	-150.6964268229045	hqer_induced sampler prototype	4.0
	WDI_industry	Quantile-SFA	hqer_induced	True	32	-98.56568838747287	hqer_induced sampler prototype	4.0
	WDI_industry	HQER-SFA	hqer_induced	True	30	-97.15794608930766	hqer_induced sampler prototype	2.0
	WDI_industry	Quantile-SFA	hqer_induced	True	23	-128.4895537649675	hqer_induced sampler prototype	4.0
	WDI_industry	HQER-SFA	hqer_induced	True	28	-129.83989232642062	hqer_induced sampler prototype	2.0
	WDI_industry	Quantile-SFA	hqer_induced	True	24	-140.62065146245124	hqer_induced sampler prototype	2.0
	WDI_industry	HQER-SFA	hqer_induced	True	36	-142.39100554490733	hqer_induced sampler prototype	4.0
	WDI_industry	Quantile-SFA	hqer_induced	True	28	-134.0324969867409	hqer_induced sampler prototype	4.0
	WDI_industry	HQER-SFA	hqer_induced	True	26	-133.2458643769735	hqer_induced sampler prototype	2.0
	WDI_industry	Quantile-SFA	hqer_induced	True	20	-147.15538773671113	hqer_induced sampler prototype	2.0
	WDI_industry	HQER-SFA	hqer_induced	True	17	-145.18999870298762	hqer_induced sampler prototype	2.0
	WDI_industry	Quantile-SFA	hqer_induced	True	17	-590.8841914123432	hqer_induced sampler prototype	4.0
	WDI_industry	HQER-SFA	hqer_induced	True	15	-582.2554075412173	hqer_induced sampler prototype	4.0
	WDI_industry	Quantile-SFA	hqer_induced	True	23	-560.5429307607067	hqer_induced sampler prototype	2.0
	WDI_industry	HQER-SFA	hqer_induced	True	12	-550.5980125039813	hqer_induced sampler prototype	4.0
	WDI_industry	Quantile-SFA	hqer_induced	True	18	-611.9548602352297	hqer_induced sampler prototype	2.0
	WDI_industry	HQER-SFA	hqer_induced	True	24	-602.2080559433841	hqer_induced sampler prototype	4.0
	WDI_industry	Quantile-SFA	hqer_induced	True	17	-592.4992803577554	hqer_induced sampler prototype	4.0
	WDI_industry	HQER-SFA	hqer_induced	True	21	-583.0030038894149	hqer_induced sampler prototype	4.0
	WDI_industry	Quantile-SFA	hqer_induced	True	21	-574.792731637706	hqer_induced sampler prototype	4.0
	WDI_industry	HQER-SFA	hqer_induced	True	9	-571.3022536422463	hqer_induced sampler prototype	2.0
	WDI_industry	Quantile-SFA	hqer_induced	True	16	-668.0999962442372	hqer_induced sampler prototype	2.0
	WDI_industry	HQER-SFA	hqer_induced	True	21	-657.5843775998346	hqer_induced sampler prototype	4.0
	WDI_industry	Quantile-SFA	hqer_induced	True	23	-605.8590688100204	hqer_induced sampler prototype	2.0
	WDI_industry	HQER-SFA	hqer_induced	True	20	-590.5363349800713	hqer_induced sampler prototype	4.0
	WDI_industry	Quantile-SFA	hqer_induced	True	21	-649.1434259992518	hqer_induced sampler prototype	4.0
	WDI_industry	HQER-SFA	hqer_induced	True	21	-636.9121661528984	hqer_induced sampler prototype	4.0
	WDI_industry	Quantile-SFA	hqer_induced	True	17	-613.0195592294128	hqer_induced sampler prototype	4.0
	WDI_industry	HQER-SFA	hqer_induced	True	15	-603.7832581625971	hqer_induced sampler prototype	4.0
	WDI_industry	Quantile-SFA	hqer_induced	True	23	-642.8666648609722	hqer_induced sampler prototype	4.0
	WDI_industry	HQER-SFA	hqer_induced	True	19	-634.9512728711294	hqer_induced sampler prototype	4.0
	WDI_industry	Quantile-SFA	hqer_induced	True	23	-584.4044250207628	hqer_induced sampler prototype	2.0
	WDI_industry	HQER-SFA	hqer_induced	True	23	-576.2218855032609	hqer_induced sampler prototype	4.0
	WDI_industry	Quantile-SFA	hqer_induced	True	14	-603.403361129539	hqer_induced sampler prototype	2.0
	WDI_industry	HQER-SFA	hqer_induced	True	29	-591.810467295784	hqer_induced sampler prototype	4.0
	WDI_industry	Quantile-SFA	hqer_induced	True	14	-713.5453405164149	hqer_induced sampler prototype	4.0
	WDI_industry	HQER-SFA	hqer_induced	True	20	-706.776020887839	hqer_induced sampler prototype	2.0
	WDI_industry	Quantile-SFA	hqer_induced	True	16	-645.3621642318408	hqer_induced sampler prototype	4.0
	WDI_industry	HQER-SFA	hqer_induced	True	19	-636.080466085523	hqer_induced sampler prototype	4.0
	WDI_industry	Quantile-SFA	hqer_induced	True	21	-612.9355952214564	hqer_induced sampler prototype	4.0
	WDI_industry	HQER-SFA	hqer_induced	True	21	-600.9661631040395	hqer_induced sampler prototype	4.0
	WDI_industry	Quantile-SFA	hqer_induced	True	23	-624.3859327442119	hqer_induced sampler prototype	2.0
	WDI_industry	HQER-SFA	hqer_induced	True	18	-615.3503655430877	hqer_induced sampler prototype	4.0
	WDI_industry	Quantile-SFA	hqer_induced	True	11	-613.9493554824492	hqer_induced sampler prototype	2.0

experiment	dataset	model	scenario	converged	n__iter	loglik	warning__message	best__start__id
	WDI__industry	HQER-SFA	hqer__induced	True	12	-603.7727786638407	hqer__induced sampler prototype	2.0
	WDI__industry	Quantile-SFA	hqer__induced	True	8	-609.8265997685205	hqer__induced sampler prototype	2.0
	WDI__industry	HQER-SFA	hqer__induced	True	12	-602.2903460979237	hqer__induced sampler prototype	2.0
	WDI__industry	Quantile-SFA	hqer__induced	True	25	-608.2343114275654	hqer__induced sampler prototype	4.0
	WDI__industry	HQER-SFA	hqer__induced	True	22	-597.318241846241	hqer__induced sampler prototype	4.0
	WDI__industry	Quantile-SFA	hqer__induced	True	25	-571.06823234734	hqer__induced sampler prototype	4.0
	WDI__industry	HQER-SFA	hqer__induced	True	19	-564.2500255606554	hqer__induced sampler prototype	2.0
	WDI__industry	Quantile-SFA	hqer__induced	True	23	-667.7773376971345	hqer__induced sampler prototype	4.0
	WDI__industry	HQER-SFA	hqer__induced	True	17	-660.7429585593391	hqer__induced sampler prototype	2.0
	WDI__industry	Quantile-SFA	hqer__induced	True	22	-677.9909128532988	hqer__induced sampler prototype	2.0
	WDI__industry	HQER-SFA	hqer__induced	True	16	-667.3814514749158	hqer__induced sampler prototype	4.0
	WDI__industry	Quantile-SFA	hqer__induced	True	20	-635.0669123747874	hqer__induced sampler prototype	2.0
	WDI__industry	HQER-SFA	hqer__induced	True	11	-621.5489920751604	hqer__induced sampler prototype	4.0
	WDI__industry	Quantile-SFA	hqer__induced	True	25	-607.4232433934592	hqer__induced sampler prototype	4.0
	WDI__industry	HQER-SFA	hqer__induced	True	22	-598.8790191069672	hqer__induced sampler prototype	4.0
	WDI__industry	Quantile-SFA	hqer__induced	True	9	-670.9382306292542	hqer__induced sampler prototype	2.0
	WDI__industry	HQER-SFA	hqer__induced	True	27	-660.2095048954561	hqer__induced sampler prototype	5.0
	WDI__industry	Quantile-SFA	hqer__induced	True	9	-607.7630217129199	hqer__induced sampler prototype	2.0
	WDI__industry	HQER-SFA	hqer__induced	True	24	-598.8907951024996	hqer__induced sampler prototype	4.0
	WDI__industry	Quantile-SFA	hqer__induced	True	12	-627.1041478810216	hqer__induced sampler prototype	2.0
	WDI__industry	HQER-SFA	hqer__induced	True	23	-612.8365355533524	hqer__induced sampler prototype	2.0
	WDI__industry	Quantile-SFA	hqer__induced	True	15	-565.6939643370747	hqer__induced sampler prototype	2.0
	WDI__industry	HQER-SFA	hqer__induced	True	18	-558.3185755228767	hqer__induced sampler prototype	2.0
	WDI__industry	Quantile-SFA	hqer__induced	True	24	-571.247409052633	hqer__induced sampler prototype	2.0
	WDI__industry	HQER-SFA	hqer__induced	True	26	-561.2421201684853	hqer__induced sampler prototype	4.0
	WDI__industry	Quantile-SFA	hqer__induced	True	21	-620.9014345251467	hqer__induced sampler prototype	4.0
	WDI__industry	HQER-SFA	hqer__induced	True	23	-609.0143442186566	hqer__induced sampler prototype	4.0
	WDI__industry	Quantile-SFA	hqer__induced	True	21	-572.6005542192306	hqer__induced sampler prototype	4.0
	WDI__industry	HQER-SFA	hqer__induced	True	21	-564.1234584928984	hqer__induced sampler prototype	4.0
	WDI__industry	Quantile-SFA	hqer__induced	True	14	-676.7125268001838	hqer__induced sampler prototype	4.0
	WDI__industry	HQER-SFA	hqer__induced	True	19	-663.9949113912558	hqer__induced sampler prototype	4.0
	WDI__industry	Quantile-SFA	hqer__induced	True	19	-583.4188842233705	hqer__induced sampler prototype	4.0
	WDI__industry	HQER-SFA	hqer__induced	True	24	-569.2484816345387	hqer__induced sampler prototype	4.0
	WDI__industry	Quantile-SFA	hqer__induced	True	15	-671.8451353247314	hqer__induced sampler prototype	2.0
	WDI__industry	HQER-SFA	hqer__induced	True	18	-661.4422937043361	hqer__induced sampler prototype	4.0
	WDI__industry	Quantile-SFA	hqer__induced	True	14	-627.4778500871695	hqer__induced sampler prototype	4.0
	WDI__industry	HQER-SFA	hqer__induced	True	17	-613.5427909454557	hqer__induced sampler prototype	4.0
	WDI__industry	Quantile-SFA	hqer__induced	True	19	-622.804333121287	hqer__induced sampler prototype	4.0
	WDI__industry	HQER-SFA	hqer__induced	True	17	-619.4181772876135	hqer__induced sampler prototype	2.0
	WDI__industry	Quantile-SFA	hqer__induced	True	21	-648.0244293035778	hqer__induced sampler prototype	2.0
	WDI__industry	HQER-SFA	hqer__induced	True	21	-636.4553695077363	hqer__induced sampler prototype	4.0
	WDI__industry	Quantile-SFA	hqer__induced	True	25	-622.8986600689411	hqer__induced sampler prototype	4.0
	WDI__industry	HQER-SFA	hqer__induced	True	26	-616.1073713982232	hqer__induced sampler prototype	4.0
	WDI__industry	Quantile-SFA	hqer__induced	True	22	-668.1208250068441	hqer__induced sampler prototype	4.0
	WDI__industry	HQER-SFA	hqer__induced	True	17	-657.6221750163857	hqer__induced sampler prototype	4.0
	WDI__industry	Quantile-SFA	hqer__induced	True	20	-650.0988921481305	hqer__induced sampler prototype	4.0
	WDI__industry	HQER-SFA	hqer__induced	True	20	-642.4613987659067	hqer__induced sampler prototype	4.0
	WDI__industry	Quantile-SFA	hqer__induced	True	26	-645.5258354621483	hqer__induced sampler prototype	4.0
	WDI__industry	HQER-SFA	hqer__induced	True	27	-634.1648793538612	hqer__induced sampler prototype	4.0
	WDI__industry	Quantile-SFA	hqer__induced	True	14	-633.3010922367635	hqer__induced sampler prototype	2.0
	WDI__industry	HQER-SFA	hqer__induced	True	20	-624.077820150361	hqer__induced sampler prototype	4.0
	WDI__industry	Quantile-SFA	hqer__induced	True	21	-664.3769873857756	hqer__induced sampler prototype	2.0
	WDI__industry	HQER-SFA	hqer__induced	True	24	-654.723696492937	hqer__induced sampler prototype	4.0
	WDI__industry	Quantile-SFA	hqer__induced	True	17	-665.27217989929	hqer__induced sampler prototype	4.0
	WDI__industry	HQER-SFA	hqer__induced	True	19	-654.5480125154144	hqer__induced sampler prototype	4.0
	WDI__industry	Quantile-SFA	hqer__induced	True	8	-680.5466391916282	hqer__induced sampler prototype	2.0
	WDI__industry	HQER-SFA	hqer__induced	True	24	-665.3600871947378	hqer__induced sampler prototype	4.0
	WDI__industry	Quantile-SFA	hqer__induced	True	26	-579.494253339131	hqer__induced sampler prototype	4.0
	WDI__industry	HQER-SFA	hqer__induced	True	14	-561.7821577531105	hqer__induced sampler prototype	4.0
	WDI__industry	Quantile-SFA	hqer__induced	True	22	-628.3947081576089	hqer__induced sampler prototype	4.0
	WDI__industry	HQER-SFA	hqer__induced	True	24	-621.3933964407348	hqer__induced sampler prototype	2.0
	WDI__industry	Quantile-SFA	hqer__induced	True	23	-653.6708122040885	hqer__induced sampler prototype	4.0
	WDI__industry	HQER-SFA	hqer__induced	True	26	-646.7746696597098	hqer__induced sampler prototype	2.0
	WDI__industry	Quantile-SFA	hqer__induced	True	33	-595.9653012337524	hqer__induced sampler prototype	4.0
	WDI__industry	HQER-SFA	hqer__induced	True	14	-589.1233979825149	hqer__induced sampler prototype	2.0
	WDI__industry	Quantile-SFA	hqer__induced	True	20	-667.2068949157134	hqer__induced sampler prototype	4.0
	WDI__industry	HQER-SFA	hqer__induced	True	14	-660.8944439941478	hqer__induced sampler prototype	2.0
	WDI__industry	Quantile-SFA	hqer__induced	True	28	-567.8762091727165	hqer__induced sampler prototype	4.0
	WDI__industry	HQER-SFA	hqer__induced	True	10	-561.6892864018992	hqer__induced sampler prototype	2.0
	WDI__industry	Quantile-SFA	hqer__induced	True	20	-599.6169839462634	hqer__induced sampler prototype	4.0
	WDI__industry	HQER-SFA	hqer__induced	True	25	-589.3199584885003	hqer__induced sampler prototype	4.0
	WDI__industry	Quantile-SFA	hqer__induced	True	21	-598.963842336949	hqer__induced sampler prototype	4.0
	WDI__industry	HQER-SFA	hqer__induced	True	9	-593.2652822966836	hqer__induced sampler prototype	2.0
	WDI__industry	Quantile-SFA	hqer__induced	True	21	-639.3340080236396	hqer__induced sampler prototype	4.0
	WDI__industry	HQER-SFA	hqer__induced	True	15	-634.8807281993772	hqer__induced sampler prototype	2.0
	WDI__industry	Quantile-SFA	hqer__induced	True	16	-701.452072009218	hqer__induced sampler prototype	4.0
	WDI__industry	HQER-SFA	hqer__induced	True	12	-690.0583037867007	hqer__induced sampler prototype	4.0
	WDI__industry	Quantile-SFA	hqer__induced	True	21	-638.2324194285716	hqer__induced sampler prototype	4.0
	WDI__industry	HQER-SFA	hqer__induced	True	16	-633.9357712061717	hqer__induced sampler prototype	2.0
	WDI__industry	Quantile-SFA	hqer__induced	True	17	-620.9698435264834	hqer__induced sampler prototype	4.0
	WDI__industry	HQER-SFA	hqer__induced	True	17	-615.2976296337076	hqer__induced sampler prototype	2.0
	WDI__industry	Quantile-SFA	hqer__induced	True	19	-632.1301833883474	hqer__induced sampler prototype	2.0
	WDI__industry	HQER-SFA	hqer__induced	True	21	-624.6919952013955	hqer__induced sampler prototype	2.0
	WDI__industry	Quantile-SFA	hqer__induced	True	20	-594.1773701628548	hqer__induced sampler prototype	4.0
	WDI__industry	HQER-SFA	hqer__induced	True	15	-585.3093206069357	hqer__induced sampler prototype	4.0
	WDI__industry	Quantile-SFA	hqer__induced	True	18	-680.7952246523014	hqer__induced sampler prototype	4.0
	WDI__industry	HQER-SFA	hqer__induced	True	20	-669.7729928378019	hqer__induced sampler prototype	4.0
	WDI__industry	Quantile-SFA	hqer__induced	True	20	-577.4315236135864	hqer__induced sampler prototype	2.0
	WDI__industry	HQER-SFA	hqer__induced	True	33	-572.4644765426174	hqer__induced sampler prototype	2.0
	WDI__industry	Quantile-SFA	hqer__induced	True	14	-580.3527247268694	hqer__induced sampler prototype	2.0
	WDI__industry	HQER-SFA	hqer__induced	True	13	-575.6272165081272	hqer__induced sampler prototype	2.0
	WDI__industry	Quantile-SFA	hqer__induced	True	20	-520.2537418859174	hqer__induced sampler prototype	4.0
	WDI__industry	HQER-SFA	hqer__induced	True	15	-513.984782947553	hqer__induced sampler prototype	2.0
	WDI__industry	Quantile-SFA	hqer__induced	True	25	-617.9452763893578	hqer__induced sampler prototype	2.0
	WDI__industry	HQER-SFA	hqer__induced	True	20	-612.4365785357616	hqer__induced sampler prototype	2.0
	WDI__industry	Quantile-SFA	hqer__induced	True	18	-679.15287454171	hqer__induced sampler prototype	2.0
	WDI__industry	HQER-SFA	hqer__induced	True	21	-668.5877264937274	hqer__induced sampler prototype	4.0
	WDI__industry	Quantile-SFA	hqer__induced	True	16	-582.1141202967613	hqer__induced sampler prototype	4.0
	WDI__industry	HQER-SFA	hqer__induced	True	18	-572.9065394647807	hqer__induced sampler prototype	4.0
	WDI__industry	Quantile-SFA	hqer__induced	True	25	-537.7690331610426	hqer__induced sampler prototype	4.0
	WDI__industry	HQER-SFA	hqer__induced	True	24	-526.7191143793112	hqer__induced sampler prototype	4.0
	WDI__industry	Quantile-SFA	hqer__induced	True	34	-581.9450350123875	hqer__induced sampler prototype	5.0
	WDI__industry	HQER-SFA	hqer__induced	True	19	-572.482651481455	hqer__induced sampler prototype	4.0

experiment	dataset	model	scenario	converged	n__iter	loglik	warning__message	best__start__id
	WDI_industry	Quantile-SFA	hqer__induced	True	17	-645.1191541593564	hqer__induced sampler prototype	2.0
	WDI_industry	HQER-SFA	hqer__induced	True	29	-629.520298370199	hqer__induced sampler prototype	4.0
	WDI_industry	Quantile-SFA	hqer__induced	True	20	-635.1267198847395	hqer__induced sampler prototype	4.0
	WDI_industry	HQER-SFA	hqer__induced	True	18	-622.5636482118969	hqer__induced sampler prototype	4.0
	WDI_industry	Quantile-SFA	hqer__induced	True	18	-656.6207727946972	hqer__induced sampler prototype	4.0
	WDI_industry	HQER-SFA	hqer__induced	True	31	-642.9315709977791	hqer__induced sampler prototype	2.0
	WDI_industry	Quantile-SFA	hqer__induced	True	17	-662.5125806789628	hqer__induced sampler prototype	4.0
	WDI_industry	HQER-SFA	hqer__induced	True	14	-651.8075842500871	hqer__induced sampler prototype	4.0
	WDI_industry	Quantile-SFA	hqer__induced	True	20	-627.9056569566752	hqer__induced sampler prototype	4.0
	WDI_industry	HQER-SFA	hqer__induced	True	7	-622.106684262891	hqer__induced sampler prototype	2.0
	WDI_industry	Quantile-SFA	hqer__induced	True	20	-647.7003045719872	hqer__induced sampler prototype	4.0
	WDI_industry	HQER-SFA	hqer__induced	True	16	-640.0171710178186	hqer__induced sampler prototype	4.0
	WDI_industry	Quantile-SFA	hqer__induced	True	22	-609.3877101548962	hqer__induced sampler prototype	4.0
	WDI_industry	HQER-SFA	hqer__induced	True	29	-599.1566421733978	hqer__induced sampler prototype	5.0
	WDI_industry	Quantile-SFA	hqer__induced	True	18	-629.9517561664134	hqer__induced sampler prototype	2.0
	WDI_industry	HQER-SFA	hqer__induced	True	19	-619.5088985987184	hqer__induced sampler prototype	4.0
	WDI_industry	Quantile-SFA	hqer__induced	True	22	-685.038303060838	hqer__induced sampler prototype	4.0
	WDI_industry	HQER-SFA	hqer__induced	True	29	-671.0255746760063	hqer__induced sampler prototype	2.0
	WDI_industry	Quantile-SFA	hqer__induced	True	19	-670.1465408789824	hqer__induced sampler prototype	2.0
	WDI_industry	HQER-SFA	hqer__induced	True	13	-660.9227450945016	hqer__induced sampler prototype	4.0
	WDI_industry	Quantile-SFA	hqer__induced	True	13	-632.6091529630517	hqer__induced sampler prototype	2.0
	WDI_industry	HQER-SFA	hqer__induced	True	13	-627.8296475735318	hqer__induced sampler prototype	2.0
	WDI_industry	Quantile-SFA	hqer__induced	True	25	-628.6087428029687	hqer__induced sampler prototype	4.0
	WDI_industry	HQER-SFA	hqer__induced	True	21	-618.522671106353	hqer__induced sampler prototype	4.0
	WDI_industry	Quantile-SFA	hqer__induced	True	19	-590.2099818788467	hqer__induced sampler prototype	2.0
	WDI_industry	HQER-SFA	hqer__induced	True	11	-578.8815572991914	hqer__induced sampler prototype	4.0
	WDI_industry	Quantile-SFA	hqer__induced	True	20	-588.3390571176087	hqer__induced sampler prototype	4.0
	WDI_industry	HQER-SFA	hqer__induced	True	30	-575.103432747142	hqer__induced sampler prototype	4.0
	WDI_industry	Quantile-SFA	hqer__induced	True	16	-599.158815435304	hqer__induced sampler prototype	4.0
	WDI_industry	HQER-SFA	hqer__induced	True	23	-592.4787325415734	hqer__induced sampler prototype	4.0
	WDI_industry	Quantile-SFA	hqer__induced	True	17	-553.3780369857279	hqer__induced sampler prototype	4.0
	WDI_industry	HQER-SFA	hqer__induced	True	23	-543.1842239080651	hqer__induced sampler prototype	4.0
	WDI_industry	Quantile-SFA	hqer__induced	True	23	-615.6036790851007	hqer__induced sampler prototype	2.0
	WDI_industry	HQER-SFA	hqer__induced	True	15	-606.268682513565	hqer__induced sampler prototype	4.0
	WDI_industry	Quantile-SFA	hqer__induced	True	18	-596.9500228535653	hqer__induced sampler prototype	2.0
	WDI_industry	HQER-SFA	hqer__induced	True	21	-588.3500362744426	hqer__induced sampler prototype	4.0
	WDI_industry	Quantile-SFA	hqer__induced	True	21	-611.8559579358705	hqer__induced sampler prototype	4.0
	WDI_industry	HQER-SFA	hqer__induced	True	25	-601.2327808790554	hqer__induced sampler prototype	4.0
	WDI_industry	Quantile-SFA	hqer__induced	True	7	-594.654052059816	hqer__induced sampler prototype	2.0
	WDI_industry	HQER-SFA	hqer__induced	True	16	-582.5709068891695	hqer__induced sampler prototype	4.0
	WDI_industry	Quantile-SFA	hqer__induced	True	15	-675.9441633949859	hqer__induced sampler prototype	2.0
	WDI_industry	HQER-SFA	hqer__induced	True	24	-668.0328520678718	hqer__induced sampler prototype	2.0
	WDI_industry	Quantile-SFA	hqer__induced	True	19	-597.5771355862664	hqer__induced sampler prototype	2.0
	WDI_industry	HQER-SFA	hqer__induced	True	18	-589.8317778842829	hqer__induced sampler prototype	2.0
	WDI_industry	Quantile-SFA	hqer__induced	True	24	-713.8605086720993	hqer__induced sampler prototype	4.0
	WDI_industry	HQER-SFA	hqer__induced	True	20	-703.8065804841739	hqer__induced sampler prototype	4.0
	WDI_industry	Quantile-SFA	hqer__induced	True	14	-699.4739653839905	hqer__induced sampler prototype	2.0
	WDI_industry	HQER-SFA	hqer__induced	True	21	-688.4863556672492	hqer__induced sampler prototype	4.0
	WDI_industry	Quantile-SFA	hqer__induced	True	15	-592.8715776534062	hqer__induced sampler prototype	2.0
	WDI_industry	HQER-SFA	hqer__induced	True	23	-580.3306602457159	hqer__induced sampler prototype	4.0
	WDI_industry	Quantile-SFA	hqer__induced	True	11	-656.5784204495151	hqer__induced sampler prototype	2.0
	WDI_industry	HQER-SFA	hqer__induced	True	17	-645.3249285484063	hqer__induced sampler prototype	4.0
	WDI_industry	Quantile-SFA	hqer__induced	True	19	-598.1581793093363	hqer__induced sampler prototype	2.0
	WDI_industry	HQER-SFA	hqer__induced	True	22	-592.8185321776145	hqer__induced sampler prototype	2.0
	WDI_industry	Quantile-SFA	hqer__induced	True	22	-536.6738994978107	hqer__induced sampler prototype	4.0
	WDI_industry	HQER-SFA	hqer__induced	True	13	-530.8922606024	hqer__induced sampler prototype	2.0
	WDI_industry	Quantile-SFA	hqer__induced	True	19	-646.7131236573545	hqer__induced sampler prototype	4.0
	WDI_industry	HQER-SFA	hqer__induced	True	17	-636.2581470469017	hqer__induced sampler prototype	4.0
	WDI_industry	Quantile-SFA	hqer__induced	True	17	-627.2598019750094	hqer__induced sampler prototype	4.0
	WDI_industry	HQER-SFA	hqer__induced	True	16	-623.9036935149413	hqer__induced sampler prototype	2.0
	WDI_industry	Quantile-SFA	hqer__induced	True	21	-635.9298296124459	hqer__induced sampler prototype	4.0
	WDI_industry	HQER-SFA	hqer__induced	True	22	-626.2010966289588	hqer__induced sampler prototype	4.0
	WDI_industry	Quantile-SFA	hqer__induced	True	14	-628.8434954291022	hqer__induced sampler prototype	2.0
	WDI_industry	HQER-SFA	hqer__induced	True	4	-621.8045043078689	hqer__induced sampler prototype	2.0
	WDI_agriculture	Quantile-SFA	hqer__induced	True	30	-716.5024820608373	hqer__induced sampler prototype	3.0
	WDI_agriculture	HQER-SFA	hqer__induced	True	26	-716.4790081173292	hqer__induced sampler prototype	1.0
	WDI_agriculture	Quantile-SFA	hqer__induced	True	27	-700.1898951368343	hqer__induced sampler prototype	1.0
	WDI_agriculture	HQER-SFA	hqer__induced	True	29	-700.1847434826575	hqer__induced sampler prototype	3.0
	WDI_agriculture	Quantile-SFA	hqer__induced	True	36	-685.7191101272938	hqer__induced sampler prototype	1.0
	WDI_agriculture	HQER-SFA	hqer__induced	True	24	-685.8270424761699	hqer__induced sampler prototype	3.0
	WDI_agriculture	Quantile-SFA	hqer__induced	True	26	-694.7425826537369	hqer__induced sampler prototype	2.0
	WDI_agriculture	HQER-SFA	hqer__induced	True	30	-694.7239963227078	hqer__induced sampler prototype	3.0
	WDI_agriculture	Quantile-SFA	hqer__induced	True	26	-721.6207259184234	hqer__induced sampler prototype	3.0
	WDI_agriculture	HQER-SFA	hqer__induced	True	31	-721.6131379007611	hqer__induced sampler prototype	3.0
	WDI_agriculture	Quantile-SFA	hqer__induced	True	51	-710.4071824868793	hqer__induced sampler prototype	3.0
	WDI_agriculture	HQER-SFA	hqer__induced	True	39	-710.4474559813125	hqer__induced sampler prototype	3.0
	WDI_agriculture	Quantile-SFA	hqer__induced	True	16	-696.5015770658607	hqer__induced sampler prototype	2.0
	WDI_agriculture	HQER-SFA	hqer__induced	True	34	-696.4809138929677	hqer__induced sampler prototype	3.0
	WDI_agriculture	Quantile-SFA	hqer__induced	True	30	-648.8380265261859	hqer__induced sampler prototype	1.0
	WDI_agriculture	HQER-SFA	hqer__induced	True	23	-648.8173089973554	hqer__induced sampler prototype	3.0
	WDI_agriculture	Quantile-SFA	hqer__induced	True	23	-739.953303296529	hqer__induced sampler prototype	1.0
	WDI_agriculture	HQER-SFA	hqer__induced	True	32	-739.9551968347109	hqer__induced sampler prototype	3.0
	WDI_agriculture	Quantile-SFA	hqer__induced	True	25	-702.0024750011662	hqer__induced sampler prototype	2.0
	WDI_agriculture	HQER-SFA	hqer__induced	True	48	-701.9960060975482	hqer__induced sampler prototype	3.0
	WDI_agriculture	Quantile-SFA	hqer__induced	True	34	-687.3156209713917	hqer__induced sampler prototype	1.0
	WDI_agriculture	HQER-SFA	hqer__induced	True	26	-687.311776689167	hqer__induced sampler prototype	1.0
	WDI_agriculture	Quantile-SFA	hqer__induced	True	33	-703.4086365875029	hqer__induced sampler prototype	1.0
	WDI_agriculture	HQER-SFA	hqer__induced	True	36	-703.3800673805545	hqer__induced sampler prototype	3.0
	WDI_agriculture	Quantile-SFA	hqer__induced	True	14	-693.5996347229603	hqer__induced sampler prototype	2.0
	WDI_agriculture	HQER-SFA	hqer__induced	True	16	-693.5853870534798	hqer__induced sampler prototype	2.0
	WDI_agriculture	Quantile-SFA	hqer__induced	True	20	-706.4890270656429	hqer__induced sampler prototype	3.0
	WDI_agriculture	HQER-SFA	hqer__induced	True	22	-706.4307212435118	hqer__induced sampler prototype	3.0
	WDI_agriculture	Quantile-SFA	hqer__induced	True	47	-712.6930967820279	hqer__induced sampler prototype	1.0
	WDI_agriculture	HQER-SFA	hqer__induced	True	37	-712.6960445073182	hqer__induced sampler prototype	5.0
	WDI_agriculture	Quantile-SFA	hqer__induced	True	21	-735.8870140750786	hqer__induced sampler prototype	5.0
	WDI_agriculture	HQER-SFA	hqer__induced	True	18	-735.8606548772598	hqer__induced sampler prototype	2.0
	WDI_agriculture	Quantile-SFA	hqer__induced	True	25	-711.3832227063479	hqer__induced sampler prototype	1.0
	WDI_agriculture	HQER-SFA	hqer__induced	True	35	-711.2897606536648	hqer__induced sampler prototype	1.0
	WDI_agriculture	Quantile-SFA	hqer__induced	True	32	-750.798004082874	hqer__induced sampler prototype	3.0
	WDI_agriculture	HQER-SFA	hqer__induced	True	34	-750.8089750819234	hqer__induced sampler prototype	3.0
	WDI_agriculture	Quantile-SFA	hqer__induced	True	35	-680.095849743715	hqer__induced sampler prototype	2.0
	WDI_agriculture	HQER-SFA	hqer__induced	True	30	-680.0244777807807	hqer__induced sampler prototype	5.0
	WDI_agriculture	Quantile-SFA	hqer__induced	True	33	-727.2415465947513	hqer__induced sampler prototype	1.0

experiment	dataset	model	scenario	converged	n_iter	loglik	warning_message	best_start_id
	WDI_agriculture	HQER-SFA	hqer_induced	True	26	-727.2075265126534	hqer_induced sampler prototype	1.0
	WDI_agriculture	Quantile-SFA	hqer_induced	True	27	-727.2682955789451	hqer_induced sampler prototype	3.0
	WDI_agriculture	HQER-SFA	hqer_induced	True	17	-727.2639393693058	hqer_induced sampler prototype	2.0
	WDI_agriculture	Quantile-SFA	hqer_induced	True	34	-721.5874774106375	hqer_induced sampler prototype	1.0
	WDI_agriculture	HQER-SFA	hqer_induced	True	23	-721.5689874312728	hqer_induced sampler prototype	5.0
	WDI_agriculture	Quantile-SFA	hqer_induced	True	36	-708.0841003136209	hqer_induced sampler prototype	3.0
	WDI_agriculture	HQER-SFA	hqer_induced	True	23	-708.1127597452694	hqer_induced sampler prototype	3.0
	WDI_agriculture	Quantile-SFA	hqer_induced	True	35	-687.7807885330956	hqer_induced sampler prototype	1.0
	WDI_agriculture	HQER-SFA	hqer_induced	True	32	-687.7643416163133	hqer_induced sampler prototype	3.0
	WDI_agriculture	Quantile-SFA	hqer_induced	True	21	-698.3084479043066	hqer_induced sampler prototype	1.0
	WDI_agriculture	HQER-SFA	hqer_induced	True	29	-698.8449891891883	hqer_induced sampler prototype	1.0
	WDI_agriculture	Quantile-SFA	hqer_induced	True	27	-713.0884997884461	hqer_induced sampler prototype	3.0
	WDI_agriculture	HQER-SFA	hqer_induced	True	22	-713.1489329917888	hqer_induced sampler prototype	5.0
	WDI_agriculture	Quantile-SFA	hqer_induced	True	45	-720.9349686817055	hqer_induced sampler prototype	3.0
	WDI_agriculture	HQER-SFA	hqer_induced	True	38	-720.0715604534171	hqer_induced sampler prototype	1.0
	WDI_agriculture	Quantile-SFA	hqer_induced	True	38	-733.1035451973328	hqer_induced sampler prototype	3.0
	WDI_agriculture	HQER-SFA	hqer_induced	True	31	-733.1413098261027	hqer_induced sampler prototype	1.0
	WDI_agriculture	Quantile-SFA	hqer_induced	True	47	-707.0227793386945	hqer_induced sampler prototype	1.0
	WDI_agriculture	HQER-SFA	hqer_induced	True	46	-708.5110589321182	hqer_induced sampler prototype	3.0
	WDI_agriculture	Quantile-SFA	hqer_induced	True	17	-686.5478371294734	hqer_induced sampler prototype	2.0
	WDI_agriculture	HQER-SFA	hqer_induced	True	19	-686.5349701069088	hqer_induced sampler prototype	2.0
	WDI_agriculture	Quantile-SFA	hqer_induced	True	27	-729.5722744466678	hqer_induced sampler prototype	1.0
	WDI_agriculture	HQER-SFA	hqer_induced	True	23	-729.5760028004299	hqer_induced sampler prototype	1.0
	WDI_agriculture	Quantile-SFA	hqer_induced	True	12	-708.1531992283402	hqer_induced sampler prototype	2.0
	WDI_agriculture	HQER-SFA	hqer_induced	True	9	-708.1581115874413	hqer_induced sampler prototype	2.0
	WDI_agriculture	Quantile-SFA	hqer_induced	True	18	-732.4723239996706	hqer_induced sampler prototype	2.0
	WDI_agriculture	HQER-SFA	hqer_induced	True	28	-732.4367470791075	hqer_induced sampler prototype	1.0
	WDI_agriculture	Quantile-SFA	hqer_induced	True	34	-720.7253992914541	hqer_induced sampler prototype	3.0
	WDI_agriculture	HQER-SFA	hqer_induced	True	16	-720.7036725168155	hqer_induced sampler prototype	5.0
	WDI_agriculture	Quantile-SFA	hqer_induced	True	28	-746.1750230446406	hqer_induced sampler prototype	5.0
	WDI_agriculture	HQER-SFA	hqer_induced	True	34	-746.1630908969194	hqer_induced sampler prototype	5.0
	WDI_agriculture	Quantile-SFA	hqer_induced	True	40	-697.802651914165	hqer_induced sampler prototype	3.0
	WDI_agriculture	HQER-SFA	hqer_induced	True	31	-697.8090336296568	hqer_induced sampler prototype	5.0
	WDI_agriculture	Quantile-SFA	hqer_induced	True	36	-704.3731013646939	hqer_induced sampler prototype	2.0
	WDI_agriculture	HQER-SFA	hqer_induced	True	23	-704.6152233476028	hqer_induced sampler prototype	5.0
	WDI_agriculture	Quantile-SFA	hqer_induced	True	24	-717.0719808745907	hqer_induced sampler prototype	3.0
	WDI_agriculture	HQER-SFA	hqer_induced	True	30	-717.0221405812321	hqer_induced sampler prototype	3.0
	WDI_agriculture	Quantile-SFA	hqer_induced	True	28	-706.4011383368753	hqer_induced sampler prototype	2.0
	WDI_agriculture	HQER-SFA	hqer_induced	True	30	-706.3759254198159	hqer_induced sampler prototype	1.0
	WDI_agriculture	Quantile-SFA	hqer_induced	True	40	-724.7483768334246	hqer_induced sampler prototype	5.0
	WDI_agriculture	HQER-SFA	hqer_induced	True	40	-724.7433705619401	hqer_induced sampler prototype	5.0
	WDI_agriculture	Quantile-SFA	hqer_induced	True	22	-683.8020978244247	hqer_induced sampler prototype	5.0
	WDI_agriculture	HQER-SFA	hqer_induced	True	16	-683.8116981887646	hqer_induced sampler prototype	5.0
	WDI_agriculture	Quantile-SFA	hqer_induced	True	33	-716.35985049265	hqer_induced sampler prototype	3.0
	WDI_agriculture	HQER-SFA	hqer_induced	True	24	-716.289202615566	hqer_induced sampler prototype	1.0
	WDI_agriculture	Quantile-SFA	hqer_induced	True	27	-730.1847554954273	hqer_induced sampler prototype	3.0
	WDI_agriculture	HQER-SFA	hqer_induced	True	33	-730.183672174375	hqer_induced sampler prototype	1.0
	WDI_agriculture	Quantile-SFA	hqer_induced	True	21	-712.6959576121637	hqer_induced sampler prototype	5.0
	WDI_agriculture	HQER-SFA	hqer_induced	True	22	-712.7207673915431	hqer_induced sampler prototype	5.0
	WDI_agriculture	Quantile-SFA	hqer_induced	True	19	-745.5403485139879	hqer_induced sampler prototype	5.0
	WDI_agriculture	HQER-SFA	hqer_induced	True	38	-745.5263490445311	hqer_induced sampler prototype	3.0
	WDI_agriculture	Quantile-SFA	hqer_induced	True	42	-704.7729801419056	hqer_induced sampler prototype	1.0
	WDI_agriculture	HQER-SFA	hqer_induced	True	14	-706.136247037075	hqer_induced sampler prototype	2.0
	WDI_agriculture	Quantile-SFA	hqer_induced	True	25	-716.5336857547404	hqer_induced sampler prototype	3.0
	WDI_agriculture	HQER-SFA	hqer_induced	True	28	-716.509572704157	hqer_induced sampler prototype	1.0
	WDI_agriculture	Quantile-SFA	hqer_induced	True	27	-711.3852048487527	hqer_induced sampler prototype	5.0
	WDI_agriculture	HQER-SFA	hqer_induced	True	41	-711.2541182925008	hqer_induced sampler prototype	5.0
	WDI_agriculture	Quantile-SFA	hqer_induced	True	24	-732.923453163404	hqer_induced sampler prototype	1.0
	WDI_agriculture	HQER-SFA	hqer_induced	True	31	-732.9142638200112	hqer_induced sampler prototype	1.0
	WDI_agriculture	Quantile-SFA	hqer_induced	True	24	-710.1265988242699	hqer_induced sampler prototype	5.0
	WDI_agriculture	HQER-SFA	hqer_induced	True	22	-710.086446666661	hqer_induced sampler prototype	2.0
	WDI_agriculture	Quantile-SFA	hqer_induced	True	32	-717.7838865673018	hqer_induced sampler prototype	5.0
	WDI_agriculture	HQER-SFA	hqer_induced	True	23	-717.7801435416701	hqer_induced sampler prototype	5.0
	WDI_agriculture	Quantile-SFA	hqer_induced	True	34	-697.2292554305734	hqer_induced sampler prototype	2.0
	WDI_agriculture	HQER-SFA	hqer_induced	True	16	-697.5814455400018	hqer_induced sampler prototype	5.0
	WDI_agriculture	Quantile-SFA	hqer_induced	True	37	-702.029287009376	hqer_induced sampler prototype	1.0
	WDI_agriculture	HQER-SFA	hqer_induced	True	26	-702.077779428033	hqer_induced sampler prototype	3.0
	WDI_agriculture	Quantile-SFA	hqer_induced	True	34	-697.0324527020265	hqer_induced sampler prototype	1.0
	WDI_agriculture	HQER-SFA	hqer_induced	True	22	-697.0897951227281	hqer_induced sampler prototype	2.0
	WDI_agriculture	Quantile-SFA	hqer_induced	True	29	-704.4244645447804	hqer_induced sampler prototype	1.0
	WDI_agriculture	HQER-SFA	hqer_induced	True	25	-704.4459874574484	hqer_induced sampler prototype	2.0
	WDI_agriculture	Quantile-SFA	hqer_induced	True	33	-680.3473208330599	hqer_induced sampler prototype	1.0
	WDI_agriculture	HQER-SFA	hqer_induced	True	47	-680.4127333674088	hqer_induced sampler prototype	1.0
	WDI_agriculture	Quantile-SFA	hqer_induced	True	16	-703.4887645881525	hqer_induced sampler prototype	2.0
	WDI_agriculture	HQER-SFA	hqer_induced	True	12	-703.6306733090225	hqer_induced sampler prototype	2.0
	WDI_agriculture	Quantile-SFA	hqer_induced	True	30	-711.2234826343616	hqer_induced sampler prototype	5.0
	WDI_agriculture	HQER-SFA	hqer_induced	True	30	-711.2252631444503	hqer_induced sampler prototype	3.0
	WDI_agriculture	Quantile-SFA	hqer_induced	True	27	-690.4265448129734	hqer_induced sampler prototype	1.0
	WDI_agriculture	HQER-SFA	hqer_induced	True	22	-690.3996846256543	hqer_induced sampler prototype	3.0
	WDI_agriculture	Quantile-SFA	hqer_induced	True	28	-735.5342094077964	hqer_induced sampler prototype	5.0
	WDI_agriculture	HQER-SFA	hqer_induced	True	21	-735.5102338902982	hqer_induced sampler prototype	1.0
	WDI_agriculture	Quantile-SFA	hqer_induced	True	14	-736.3057031645164	hqer_induced sampler prototype	4.0
	WDI_agriculture	HQER-SFA	hqer_induced	True	12	-736.230454993634	hqer_induced sampler prototype	4.0
	WDI_agriculture	Quantile-SFA	hqer_induced	True	20	-732.8870488189393	hqer_induced sampler prototype	1.0
	WDI_agriculture	HQER-SFA	hqer_induced	True	27	-732.8667720387709	hqer_induced sampler prototype	5.0
	WDI_agriculture	Quantile-SFA	hqer_induced	True	15	-679.4626508485328	hqer_induced sampler prototype	4.0
	WDI_agriculture	HQER-SFA	hqer_induced	True	11	-679.3900187475226	hqer_induced sampler prototype	4.0
	WDI_agriculture	Quantile-SFA	hqer_induced	True	34	-669.1977876922573	hqer_induced sampler prototype	1.0
	WDI_agriculture	HQER-SFA	hqer_induced	True	16	-669.2243012913178	hqer_induced sampler prototype	2.0
	WDI_agriculture	Quantile-SFA	hqer_induced	True	25	-706.7279806231343	hqer_induced sampler prototype	2.0
	WDI_agriculture	HQER-SFA	hqer_induced	True	30	-706.7196911594443	hqer_induced sampler prototype	5.0
	WDI_agriculture	Quantile-SFA	hqer_induced	True	31	-736.4465576877702	hqer_induced sampler prototype	1.0
	WDI_agriculture	HQER-SFA	hqer_induced	True	26	-736.4337064484289	hqer_induced sampler prototype	3.0
	WDI_agriculture	Quantile-SFA	hqer_induced	True	15	-699.2787092257919	hqer_induced sampler prototype	5.0
	WDI_agriculture	HQER-SFA	hqer_induced	True	16	-699.2875508734152	hqer_induced sampler prototype	5.0
	WDI_agriculture	Quantile-SFA	hqer_induced	True	32	-732.4733195478804	hqer_induced sampler prototype	3.0
	WDI_agriculture	HQER-SFA	hqer_induced	True	27	-732.4041503758295	hqer_induced sampler prototype	3.0
	WDI_agriculture	Quantile-SFA	hqer_induced	True	28	-726.256889118593	hqer_induced sampler prototype	3.0
	WDI_agriculture	HQER-SFA	hqer_induced	True	22	-726.2363302205138	hqer_induced sampler prototype	1.0
	WDI_agriculture	Quantile-SFA	hqer_induced	True	28	-701.8487945680045	hqer_induced sampler prototype	1.0
	WDI_agriculture	HQER-SFA	hqer_induced	True	29	-701.8151799287027	hqer_induced sampler prototype	1.0
	WDI_agriculture	Quantile-SFA	hqer_induced	True	26	-715.4341929426737	hqer_induced sampler prototype	3.0
	WDI_agriculture	HQER-SFA	hqer_induced	True	30	-715.4277215834409	hqer_induced sampler prototype	3.0

experiment	dataset	model	scenario	converged	n_iter	loglik	warning_message	best_start_id
	WDI_agriculture	Quantile-SFA	hqer_induced	True	45	-684.7630799923695	hqer_induced sampler prototype	1.0
	WDI_agriculture	HQER-SFA	hqer_induced	True	27	-684.7590417434195	hqer_induced sampler prototype	3.0
	WDI_agriculture	Quantile-SFA	hqer_induced	True	29	-696.7707205098884	hqer_induced sampler prototype	1.0
	WDI_agriculture	HQER-SFA	hqer_induced	True	23	-696.7245309748296	hqer_induced sampler prototype	5.0
	WDI_agriculture	Quantile-SFA	hqer_induced	True	27	-712.2157447508914	hqer_induced sampler prototype	2.0
	WDI_agriculture	HQER-SFA	hqer_induced	True	28	-712.1848659767574	hqer_induced sampler prototype	2.0
	WDI_agriculture	Quantile-SFA	hqer_induced	True	15	-705.1020436053401	hqer_induced sampler prototype	5.0
	WDI_agriculture	HQER-SFA	hqer_induced	True	17	-705.0848791393232	hqer_induced sampler prototype	5.0
	WDI_agriculture	Quantile-SFA	hqer_induced	True	26	-708.6713344048728	hqer_induced sampler prototype	3.0
	WDI_agriculture	HQER-SFA	hqer_induced	True	32	-708.6417076979171	hqer_induced sampler prototype	1.0
	WDI_agriculture	Quantile-SFA	hqer_induced	True	39	-734.930379803667	hqer_induced sampler prototype	1.0
	WDI_agriculture	HQER-SFA	hqer_induced	True	35	-734.9085262702026	hqer_induced sampler prototype	3.0
	WDI_agriculture	Quantile-SFA	hqer_induced	True	30	-724.4568646608021	hqer_induced sampler prototype	5.0
	WDI_agriculture	HQER-SFA	hqer_induced	True	45	-723.7085059676351	hqer_induced sampler prototype	1.0
	WDI_agriculture	Quantile-SFA	hqer_induced	True	24	-703.7502474643599	hqer_induced sampler prototype	3.0
	WDI_agriculture	HQER-SFA	hqer_induced	True	18	-703.7403180282372	hqer_induced sampler prototype	3.0
	WDI_agriculture	Quantile-SFA	hqer_induced	True	59	-701.1323396393221	hqer_induced sampler prototype	3.0
	WDI_agriculture	HQER-SFA	hqer_induced	True	37	-701.1177148797115	hqer_induced sampler prototype	5.0
	WDI_agriculture	Quantile-SFA	hqer_induced	True	18	-712.9600808081635	hqer_induced sampler prototype	2.0
	WDI_agriculture	HQER-SFA	hqer_induced	True	18	-712.9459370351627	hqer_induced sampler prototype	5.0
	WDI_agriculture	Quantile-SFA	hqer_induced	True	28	-717.6125272187071	hqer_induced sampler prototype	2.0
	WDI_agriculture	HQER-SFA	hqer_induced	True	25	-718.0791529500962	hqer_induced sampler prototype	3.0
	WDI_agriculture	Quantile-SFA	hqer_induced	True	20	-707.8739631117538	hqer_induced sampler prototype	2.0
	WDI_agriculture	HQER-SFA	hqer_induced	True	23	-707.8542947339191	hqer_induced sampler prototype	1.0
	WDI_agriculture	Quantile-SFA	hqer_induced	True	29	-732.3907059490516	hqer_induced sampler prototype	1.0
	WDI_agriculture	HQER-SFA	hqer_induced	True	23	-732.3752612290734	hqer_induced sampler prototype	1.0
	WDI_agriculture	Quantile-SFA	hqer_induced	True	32	-718.358135868662	hqer_induced sampler prototype	1.0
	WDI_agriculture	HQER-SFA	hqer_induced	True	34	-718.3655356596828	hqer_induced sampler prototype	3.0
	WDI_agriculture	Quantile-SFA	hqer_induced	True	26	-737.5914380847917	hqer_induced sampler prototype	1.0
	WDI_agriculture	HQER-SFA	hqer_induced	True	30	-737.5692670777385	hqer_induced sampler prototype	1.0
	WDI_agriculture	Quantile-SFA	hqer_induced	True	12	-750.2896116423192	hqer_induced sampler prototype	2.0
	WDI_agriculture	HQER-SFA	hqer_induced	True	19	-750.2625845347798	hqer_induced sampler prototype	3.0
	WDI_agriculture	Quantile-SFA	hqer_induced	True	10	-713.6916143404515	hqer_induced sampler prototype	2.0
	WDI_agriculture	HQER-SFA	hqer_induced	True	10	-713.6591239668712	hqer_induced sampler prototype	2.0
	WDI_agriculture	Quantile-SFA	hqer_induced	True	33	-731.0872981298014	hqer_induced sampler prototype	2.0
	WDI_agriculture	HQER-SFA	hqer_induced	True	22	-731.0554139326928	hqer_induced sampler prototype	1.0
	WDI_agriculture	Quantile-SFA	hqer_induced	True	31	-727.0702084466789	hqer_induced sampler prototype	3.0
	WDI_agriculture	HQER-SFA	hqer_induced	True	33	-727.0536776863538	hqer_induced sampler prototype	1.0
	WDI_agriculture	Quantile-SFA	hqer_induced	True	36	-709.005291960052	hqer_induced sampler prototype	2.0
	WDI_agriculture	HQER-SFA	hqer_induced	True	34	-709.0210211467272	hqer_induced sampler prototype	1.0
	WDI_agriculture	Quantile-SFA	hqer_induced	True	28	-731.7115633566325	hqer_induced sampler prototype	3.0
	WDI_agriculture	HQER-SFA	hqer_induced	True	23	-731.7061954730209	hqer_induced sampler prototype	1.0
	WDI_agriculture	Quantile-SFA	hqer_induced	True	28	-689.581156466027	hqer_induced sampler prototype	2.0
	WDI_agriculture	HQER-SFA	hqer_induced	True	32	-689.4733841209921	hqer_induced sampler prototype	2.0
	WDI_agriculture	Quantile-SFA	hqer_induced	True	21	-711.3588379532148	hqer_induced sampler prototype	2.0
	WDI_agriculture	HQER-SFA	hqer_induced	True	14	-711.3634024078723	hqer_induced sampler prototype	5.0
	WDI_agriculture	Quantile-SFA	hqer_induced	True	36	-729.5033016951381	hqer_induced sampler prototype	3.0
	WDI_agriculture	HQER-SFA	hqer_induced	True	29	-729.6755301012782	hqer_induced sampler prototype	2.0
	WDI_agriculture	Quantile-SFA	hqer_induced	True	28	-707.2198717509707	hqer_induced sampler prototype	3.0
	WDI_agriculture	HQER-SFA	hqer_induced	True	28	-707.705134562488	hqer_induced sampler prototype	5.0
	WDI_agriculture	Quantile-SFA	hqer_induced	True	20	-718.5338591034823	hqer_induced sampler prototype	5.0
	WDI_agriculture	HQER-SFA	hqer_induced	True	18	-718.505022740382	hqer_induced sampler prototype	5.0
	WDI_agriculture	Quantile-SFA	hqer_induced	True	23	-706.0112539982272	hqer_induced sampler prototype	3.0
	WDI_agriculture	HQER-SFA	hqer_induced	True	19	-705.9993754002976	hqer_induced sampler prototype	2.0
	WDI_agriculture	Quantile-SFA	hqer_induced	True	26	-708.20406514106	hqer_induced sampler prototype	1.0
	WDI_agriculture	HQER-SFA	hqer_induced	True	20	-708.1997331985804	hqer_induced sampler prototype	3.0
	WDI_agriculture	Quantile-SFA	hqer_induced	True	31	-691.6112589691953	hqer_induced sampler prototype	5.0
	WDI_agriculture	HQER-SFA	hqer_induced	True	29	-691.6716978869864	hqer_induced sampler prototype	2.0
	WDI_agriculture	Quantile-SFA	hqer_induced	True	24	-2875.5089335582115	hqer_induced sampler prototype	5.0
	WDI_agriculture	HQER-SFA	hqer_induced	True	25	-2875.4440496422885	hqer_induced sampler prototype	2.0
	WDI_agriculture	Quantile-SFA	hqer_induced	True	21	-2795.913849676203	hqer_induced sampler prototype	5.0
	WDI_agriculture	HQER-SFA	hqer_induced	True	25	-2795.7176182744406	hqer_induced sampler prototype	1.0
	WDI_agriculture	Quantile-SFA	hqer_induced	True	14	-2836.50395352509	hqer_induced sampler prototype	2.0
	WDI_agriculture	HQER-SFA	hqer_induced	True	27	-2836.438173850201	hqer_induced sampler prototype	1.0
	WDI_agriculture	Quantile-SFA	hqer_induced	True	13	-2813.1075867674763	hqer_induced sampler prototype	2.0
	WDI_agriculture	HQER-SFA	hqer_induced	True	9	-2813.0742203298123	hqer_induced sampler prototype	2.0
	WDI_agriculture	Quantile-SFA	hqer_induced	True	19	-2876.439479705072	hqer_induced sampler prototype	2.0
	WDI_agriculture	HQER-SFA	hqer_induced	True	23	-2876.338784026094	hqer_induced sampler prototype	5.0
	WDI_agriculture	Quantile-SFA	hqer_induced	True	32	-2854.3692010646114	hqer_induced sampler prototype	3.0
	WDI_agriculture	HQER-SFA	hqer_induced	True	21	-2854.3121529917453	hqer_induced sampler prototype	5.0
	WDI_agriculture	Quantile-SFA	hqer_induced	True	26	-2821.164082961081	hqer_induced sampler prototype	3.0
	WDI_agriculture	HQER-SFA	hqer_induced	True	17	-2821.135834581198	hqer_induced sampler prototype	2.0
	WDI_agriculture	Quantile-SFA	hqer_induced	True	26	-2910.069842752542	hqer_induced sampler prototype	1.0
	WDI_agriculture	HQER-SFA	hqer_induced	True	26	-2909.993349275496	hqer_induced sampler prototype	1.0
	WDI_agriculture	Quantile-SFA	hqer_induced	True	30	-2867.508092465787	hqer_induced sampler prototype	1.0
	WDI_agriculture	HQER-SFA	hqer_induced	True	23	-2867.591974965141	hqer_induced sampler prototype	3.0
	WDI_agriculture	Quantile-SFA	hqer_induced	True	27	-2876.8515945200047	hqer_induced sampler prototype	5.0
	WDI_agriculture	HQER-SFA	hqer_induced	True	21	-2876.746643518113	hqer_induced sampler prototype	5.0
	WDI_agriculture	Quantile-SFA	hqer_induced	True	25	-2863.988232270042	hqer_induced sampler prototype	2.0
	WDI_agriculture	HQER-SFA	hqer_induced	True	23	-2863.956534655548	hqer_induced sampler prototype	2.0
	WDI_agriculture	Quantile-SFA	hqer_induced	True	35	-2845.0879509337565	hqer_induced sampler prototype	1.0
	WDI_agriculture	HQER-SFA	hqer_induced	True	21	-2845.063435747592	hqer_induced sampler prototype	3.0
	WDI_agriculture	Quantile-SFA	hqer_induced	True	25	-2818.0982623619816	hqer_induced sampler prototype	3.0
	WDI_agriculture	HQER-SFA	hqer_induced	True	17	-2818.115277141547	hqer_induced sampler prototype	5.0
	WDI_agriculture	Quantile-SFA	hqer_induced	True	19	-2849.317771502904	hqer_induced sampler prototype	2.0
	WDI_agriculture	HQER-SFA	hqer_induced	True	30	-2849.27172211006	hqer_induced sampler prototype	5.0
	WDI_agriculture	Quantile-SFA	hqer_induced	True	28	-2860.7563036211154	hqer_induced sampler prototype	5.0
	WDI_agriculture	HQER-SFA	hqer_induced	True	15	-2860.6700407986827	hqer_induced sampler prototype	2.0
	WDI_agriculture	Quantile-SFA	hqer_induced	True	22	-2836.025194319098	hqer_induced sampler prototype	3.0
	WDI_agriculture	HQER-SFA	hqer_induced	True	21	-2835.902602379151	hqer_induced sampler prototype	1.0
	WDI_agriculture	Quantile-SFA	hqer_induced	True	22	-2927.8723020827542	hqer_induced sampler prototype	3.0
	WDI_agriculture	HQER-SFA	hqer_induced	True	29	-2927.797088806742	hqer_induced sampler prototype	1.0
	WDI_agriculture	Quantile-SFA	hqer_induced	True	19	-2891.4574762146785	hqer_induced sampler prototype	1.0
	WDI_agriculture	HQER-SFA	hqer_induced	True	27	-2891.383170543362	hqer_induced sampler prototype	3.0
	WDI_agriculture	Quantile-SFA	hqer_induced	True	18	-2840.403945566145	hqer_induced sampler prototype	2.0
	WDI_agriculture	HQER-SFA	hqer_induced	True	23	-2840.261067347738	hqer_induced sampler prototype	1.0
	WDI_agriculture	Quantile-SFA	hqer_induced	True	15	-2830.354063264306	hqer_induced sampler prototype	5.0
	WDI_agriculture	HQER-SFA	hqer_induced	True	27	-2830.238319809303	hqer_induced sampler prototype	2.0
	WDI_agriculture	Quantile-SFA	hqer_induced	True	22	-2910.942546763502	hqer_induced sampler prototype	3.0
	WDI_agriculture	HQER-SFA	hqer_induced	True	11	-2910.935156418938	hqer_induced sampler prototype	2.0
	WDI_agriculture	Quantile-SFA	hqer_induced	True	18	-2828.203611457636	hqer_induced sampler prototype	5.0
	WDI_agriculture	HQER-SFA	hqer_induced	True	18	-2828.090005802281	hqer_induced sampler prototype	1.0
	WDI_agriculture	Quantile-SFA	hqer_induced	True	24	-2852.1628556287205	hqer_induced sampler prototype	1.0

experiment	dataset	model	scenario	converged	n_iter	loglik	warning_message	best_start_id
	WDI_agriculture	HQER-SFA	hqer_induced	True	28	-2852.089037399731	hqer_induced sampler prototype	3.0
	WDI_agriculture	Quantile-SFA	hqer_induced	True	12	-2782.962426527218	hqer_induced sampler prototype	2.0
	WDI_agriculture	HQER-SFA	hqer_induced	True	9	-2782.909533237426	hqer_induced sampler prototype	2.0
	WDI_agriculture	Quantile-SFA	hqer_induced	True	26	-2868.1717599171334	hqer_induced sampler prototype	3.0
	WDI_agriculture	HQER-SFA	hqer_induced	True	21	-2868.128194424249	hqer_induced sampler prototype	1.0
	WDI_agriculture	Quantile-SFA	hqer_induced	True	21	-2905.56780107911	hqer_induced sampler prototype	3.0
	WDI_agriculture	HQER-SFA	hqer_induced	True	26	-2905.481967859887	hqer_induced sampler prototype	5.0
	WDI_agriculture	Quantile-SFA	hqer_induced	True	16	-2861.0827413228144	hqer_induced sampler prototype	1.0
	WDI_agriculture	HQER-SFA	hqer_induced	True	20	-2860.7517859479867	hqer_induced sampler prototype	3.0
	WDI_agriculture	Quantile-SFA	hqer_induced	True	19	-2878.046701846989	hqer_induced sampler prototype	2.0
	WDI_agriculture	HQER-SFA	hqer_induced	True	21	-2877.9314172809454	hqer_induced sampler prototype	1.0
	WDI_agriculture	Quantile-SFA	hqer_induced	True	23	-2838.072777689255	hqer_induced sampler prototype	2.0
	WDI_agriculture	HQER-SFA	hqer_induced	True	19	-2837.99197805778	hqer_induced sampler prototype	2.0
	WDI_agriculture	Quantile-SFA	hqer_induced	True	23	-2874.640120494968	hqer_induced sampler prototype	1.0
	WDI_agriculture	HQER-SFA	hqer_induced	True	15	-2874.539557607118	hqer_induced sampler prototype	2.0
	WDI_agriculture	Quantile-SFA	hqer_induced	True	26	-2851.6683387926896	hqer_induced sampler prototype	1.0
	WDI_agriculture	HQER-SFA	hqer_induced	True	30	-2851.594282576117	hqer_induced sampler prototype	1.0
	WDI_agriculture	Quantile-SFA	hqer_induced	True	23	-2878.3543538187164	hqer_induced sampler prototype	1.0
	WDI_agriculture	HQER-SFA	hqer_induced	True	25	-2878.2264507272234	hqer_induced sampler prototype	1.0
	WDI_agriculture	Quantile-SFA	hqer_induced	True	23	-2882.1576671516195	hqer_induced sampler prototype	3.0
	WDI_agriculture	HQER-SFA	hqer_induced	True	30	-2882.086805272522	hqer_induced sampler prototype	3.0
	WDI_agriculture	Quantile-SFA	hqer_induced	True	26	-2885.1284496091275	hqer_induced sampler prototype	2.0
	WDI_agriculture	HQER-SFA	hqer_induced	True	22	-2885.22106103404	hqer_induced sampler prototype	1.0
	WDI_agriculture	Quantile-SFA	hqer_induced	True	20	-2857.023474246768	hqer_induced sampler prototype	1.0
	WDI_agriculture	HQER-SFA	hqer_induced	True	18	-2856.9872411271863	hqer_induced sampler prototype	3.0
	WDI_agriculture	Quantile-SFA	hqer_induced	True	21	-2877.622895074416	hqer_induced sampler prototype	1.0
	WDI_agriculture	HQER-SFA	hqer_induced	True	23	-2877.5613019156963	hqer_induced sampler prototype	3.0
	WDI_agriculture	Quantile-SFA	hqer_induced	True	17	-2862.573189254706	hqer_induced sampler prototype	1.0
	WDI_agriculture	HQER-SFA	hqer_induced	True	38	-2862.460215294625	hqer_induced sampler prototype	5.0
	WDI_agriculture	Quantile-SFA	hqer_induced	True	25	-2865.9361146379333	hqer_induced sampler prototype	3.0
	WDI_agriculture	HQER-SFA	hqer_induced	True	27	-2865.9133169349	hqer_induced sampler prototype	1.0
	WDI_agriculture	Quantile-SFA	hqer_induced	True	19	-2862.288056184506	hqer_induced sampler prototype	1.0
	WDI_agriculture	HQER-SFA	hqer_induced	True	21	-2862.244884586796	hqer_induced sampler prototype	5.0
	WDI_agriculture	Quantile-SFA	hqer_induced	True	18	-2902.7189036278164	hqer_induced sampler prototype	3.0
	WDI_agriculture	HQER-SFA	hqer_induced	True	19	-2902.253434469822	hqer_induced sampler prototype	3.0
	WDI_agriculture	Quantile-SFA	hqer_induced	True	18	-2873.9746584260693	hqer_induced sampler prototype	2.0
	WDI_agriculture	HQER-SFA	hqer_induced	True	22	-2873.8785635166214	hqer_induced sampler prototype	2.0
	WDI_agriculture	Quantile-SFA	hqer_induced	True	23	-2885.544185300587	hqer_induced sampler prototype	1.0
	WDI_agriculture	HQER-SFA	hqer_induced	True	23	-2885.4056868380435	hqer_induced sampler prototype	5.0
	WDI_agriculture	Quantile-SFA	hqer_induced	True	21	-2820.350327955719	hqer_induced sampler prototype	3.0
	WDI_agriculture	HQER-SFA	hqer_induced	True	15	-2820.22437441715	hqer_induced sampler prototype	5.0
	WDI_agriculture	Quantile-SFA	hqer_induced	True	29	-2847.4904129928104	hqer_induced sampler prototype	3.0
	WDI_agriculture	HQER-SFA	hqer_induced	True	15	-2847.432036988682	hqer_induced sampler prototype	5.0
	WDI_agriculture	Quantile-SFA	hqer_induced	True	23	-2831.3421720455945	hqer_induced sampler prototype	3.0
	WDI_agriculture	HQER-SFA	hqer_induced	True	8	-2831.30195263285	hqer_induced sampler prototype	2.0
	WDI_agriculture	Quantile-SFA	hqer_induced	True	24	-2808.651514009085	hqer_induced sampler prototype	3.0
	WDI_agriculture	HQER-SFA	hqer_induced	True	22	-2808.6003966872227	hqer_induced sampler prototype	1.0
	WDI_agriculture	Quantile-SFA	hqer_induced	True	25	-2813.371464424424	hqer_induced sampler prototype	5.0
	WDI_agriculture	HQER-SFA	hqer_induced	True	27	-2813.277017938476	hqer_induced sampler prototype	5.0
	WDI_agriculture	Quantile-SFA	hqer_induced	True	51	-2881.2159235958484	hqer_induced sampler prototype	1.0
	WDI_agriculture	HQER-SFA	hqer_induced	True	13	-2881.1951545381503	hqer_induced sampler prototype	5.0
	WDI_agriculture	Quantile-SFA	hqer_induced	True	19	-2890.9471405894765	hqer_induced sampler prototype	1.0
	WDI_agriculture	HQER-SFA	hqer_induced	True	21	-2890.849946517153	hqer_induced sampler prototype	1.0
	WDI_agriculture	Quantile-SFA	hqer_induced	True	15	-2848.267979991046	hqer_induced sampler prototype	2.0
	WDI_agriculture	HQER-SFA	hqer_induced	True	13	-2848.1889324807225	hqer_induced sampler prototype	2.0
	WDI_agriculture	Quantile-SFA	hqer_induced	True	20	-2851.2631815898103	hqer_induced sampler prototype	2.0
	WDI_agriculture	HQER-SFA	hqer_induced	True	18	-2851.183630690238	hqer_induced sampler prototype	2.0
	WDI_agriculture	Quantile-SFA	hqer_induced	True	26	-2905.5886903964483	hqer_induced sampler prototype	5.0
	WDI_agriculture	HQER-SFA	hqer_induced	True	32	-2905.5185202252032	hqer_induced sampler prototype	1.0
	WDI_agriculture	Quantile-SFA	hqer_induced	True	18	-2913.578558892847	hqer_induced sampler prototype	5.0
	WDI_agriculture	HQER-SFA	hqer_induced	True	24	-2913.4410362083618	hqer_induced sampler prototype	1.0
	WDI_agriculture	Quantile-SFA	hqer_induced	True	24	-2846.656271690361	hqer_induced sampler prototype	3.0
	WDI_agriculture	HQER-SFA	hqer_induced	True	23	-2846.660738460919	hqer_induced sampler prototype	1.0
	WDI_agriculture	Quantile-SFA	hqer_induced	True	27	-2892.75437313411	hqer_induced sampler prototype	3.0
	WDI_agriculture	HQER-SFA	hqer_induced	True	12	-2892.6593478572886	hqer_induced sampler prototype	2.0
	WDI_agriculture	Quantile-SFA	hqer_induced	True	25	-2884.9442362678647	hqer_induced sampler prototype	5.0
	WDI_agriculture	HQER-SFA	hqer_induced	True	25	-2884.861942859762	hqer_induced sampler prototype	1.0
	WDI_agriculture	Quantile-SFA	hqer_induced	True	23	-2864.1789609395314	hqer_induced sampler prototype	3.0
	WDI_agriculture	HQER-SFA	hqer_induced	True	15	-2864.138658385559	hqer_induced sampler prototype	2.0
	WDI_agriculture	Quantile-SFA	hqer_induced	True	10	-2893.439935235364	hqer_induced sampler prototype	2.0
	WDI_agriculture	HQER-SFA	hqer_induced	True	19	-2893.334259111324	hqer_induced sampler prototype	5.0
	WDI_agriculture	Quantile-SFA	hqer_induced	True	21	-2842.268623178161	hqer_induced sampler prototype	1.0
	WDI_agriculture	HQER-SFA	hqer_induced	True	19	-2842.2050153439777	hqer_induced sampler prototype	2.0
	WDI_agriculture	Quantile-SFA	hqer_induced	True	23	-2854.755349632144	hqer_induced sampler prototype	3.0
	WDI_agriculture	HQER-SFA	hqer_induced	True	19	-2854.7479694075564	hqer_induced sampler prototype	5.0
	WDI_agriculture	Quantile-SFA	hqer_induced	True	28	-2849.674249836348	hqer_induced sampler prototype	1.0
	WDI_agriculture	HQER-SFA	hqer_induced	True	23	-2849.6030273353813	hqer_induced sampler prototype	5.0
	WDI_agriculture	Quantile-SFA	hqer_induced	True	30	-2858.5247321742	hqer_induced sampler prototype	3.0
	WDI_agriculture	HQER-SFA	hqer_induced	True	15	-2858.4685669076844	hqer_induced sampler prototype	2.0
	WDI_agriculture	Quantile-SFA	hqer_induced	True	20	-2810.1899865763667	hqer_induced sampler prototype	3.0
	WDI_agriculture	HQER-SFA	hqer_induced	True	27	-2810.153455163144	hqer_induced sampler prototype	3.0
	WDI_agriculture	Quantile-SFA	hqer_induced	True	35	-2821.6594434653707	hqer_induced sampler prototype	1.0
	WDI_agriculture	HQER-SFA	hqer_induced	True	25	-2821.5844948941217	hqer_induced sampler prototype	3.0
	WDI_agriculture	Quantile-SFA	hqer_induced	True	24	-2841.0984939783284	hqer_induced sampler prototype	5.0
	WDI_agriculture	HQER-SFA	hqer_induced	True	23	-2841.006831633592	hqer_induced sampler prototype	3.0
	WDI_agriculture	Quantile-SFA	hqer_induced	True	30	-2866.721258423653	hqer_induced sampler prototype	1.0
	WDI_agriculture	HQER-SFA	hqer_induced	True	20	-2866.758725347049	hqer_induced sampler prototype	1.0
	WDI_agriculture	Quantile-SFA	hqer_induced	True	36	-2851.694771995094	hqer_induced sampler prototype	1.0
	WDI_agriculture	HQER-SFA	hqer_induced	True	17	-2851.632986854338	hqer_induced sampler prototype	5.0
	WDI_agriculture	Quantile-SFA	hqer_induced	True	19	-2878.63873394042	hqer_induced sampler prototype	2.0
	WDI_agriculture	HQER-SFA	hqer_induced	True	21	-2878.915566476937	hqer_induced sampler prototype	2.0
	WDI_agriculture	Quantile-SFA	hqer_induced	True	17	-2827.039528838576	hqer_induced sampler prototype	3.0
	WDI_agriculture	HQER-SFA	hqer_induced	True	20	-2826.9891613373584	hqer_induced sampler prototype	2.0
	WDI_agriculture	Quantile-SFA	hqer_induced	True	16	-2874.1813319668368	hqer_induced sampler prototype	5.0
	WDI_agriculture	HQER-SFA	hqer_induced	True	13	-2874.0951304826567	hqer_induced sampler prototype	5.0
	WDI_agriculture	Quantile-SFA	hqer_induced	True	23	-2891.899980606599	hqer_induced sampler prototype	3.0
	WDI_agriculture	HQER-SFA	hqer_induced	True	21	-2891.8991142423874	hqer_induced sampler prototype	5.0
	WDI_agriculture	Quantile-SFA	hqer_induced	True	27	-2944.360748961448	hqer_induced sampler prototype	1.0
	WDI_agriculture	HQER-SFA	hqer_induced	True	23	-2944.255426083125	hqer_induced sampler prototype	3.0
	WDI_agriculture	Quantile-SFA	hqer_induced	True	16	-2846.8762336354225	hqer_induced sampler prototype	2.0
	WDI_agriculture	HQER-SFA	hqer_induced	True	21	-2846.808196243277	hqer_induced sampler prototype	1.0
	WDI_agriculture	Quantile-SFA	hqer_induced	True	19	-2879.0279167368776	hqer_induced sampler prototype	2.0
	WDI_agriculture	HQER-SFA	hqer_induced	True	29	-2878.965826028348	hqer_induced sampler prototype	1.0

experiment	dataset	model	scenario	converged	n_iter	loglik	warning_message	best_start_id
	WDI_agriculture	Quantile-SFA	hqer_induced	True	16	-2888.5564540241503	hqer_induced sampler prototype	5.0
	WDI_agriculture	HQER-SFA	hqer_induced	True	21	-2888.4698180822315	hqer_induced sampler prototype	5.0
	WDI_agriculture	Quantile-SFA	hqer_induced	True	24	-2875.1272606615835	hqer_induced sampler prototype	1.0
	WDI_agriculture	HQER-SFA	hqer_induced	True	19	-2875.114064348543	hqer_induced sampler prototype	2.0
	WDI_agriculture	Quantile-SFA	hqer_induced	True	21	-2911.0396166100763	hqer_induced sampler prototype	1.0
	WDI_agriculture	HQER-SFA	hqer_induced	True	20	-2910.941775743756	hqer_induced sampler prototype	1.0
	WDI_agriculture	Quantile-SFA	hqer_induced	True	22	-2932.0828257416706	hqer_induced sampler prototype	1.0
	WDI_agriculture	HQER-SFA	hqer_induced	True	23	-2931.976044797493	hqer_induced sampler prototype	1.0
	WDI_agriculture	Quantile-SFA	hqer_induced	True	15	-2807.546377771546	hqer_induced sampler prototype	5.0
	WDI_agriculture	HQER-SFA	hqer_induced	True	21	-2807.5116447756645	hqer_induced sampler prototype	3.0
	WDI_agriculture	Quantile-SFA	hqer_induced	True	15	-2811.4509528050053	hqer_induced sampler prototype	5.0
	WDI_agriculture	HQER-SFA	hqer_induced	True	26	-2811.3476748129788	hqer_induced sampler prototype	3.0
	WDI_agriculture	Quantile-SFA	hqer_induced	True	25	-2821.6848176095987	hqer_induced sampler prototype	1.0
	WDI_agriculture	HQER-SFA	hqer_induced	True	22	-2821.647549969551	hqer_induced sampler prototype	2.0
	WDI_agriculture	Quantile-SFA	hqer_induced	True	35	-2784.062484863426	hqer_induced sampler prototype	1.0
	WDI_agriculture	HQER-SFA	hqer_induced	True	12	-2783.996887575329	hqer_induced sampler prototype	2.0
	WDI_agriculture	Quantile-SFA	hqer_induced	True	27	-2853.920683117935	hqer_induced sampler prototype	5.0
	WDI_agriculture	HQER-SFA	hqer_induced	True	25	-2853.822844562207	hqer_induced sampler prototype	5.0
	WDI_agriculture	Quantile-SFA	hqer_induced	True	10	-2885.706836847767	hqer_induced sampler prototype	5.0
	WDI_agriculture	HQER-SFA	hqer_induced	True	21	-2885.357758817279	hqer_induced sampler prototype	5.0
	WDI_agriculture	Quantile-SFA	hqer_induced	True	27	-2898.152933240321	hqer_induced sampler prototype	5.0
	WDI_agriculture	HQER-SFA	hqer_induced	True	16	-2898.0536130529963	hqer_induced sampler prototype	2.0
	WDI_agriculture	Quantile-SFA	hqer_induced	True	25	-2857.072424521995	hqer_induced sampler prototype	5.0
	WDI_agriculture	HQER-SFA	hqer_induced	True	23	-2856.9797026083	hqer_induced sampler prototype	2.0
	WDI_agriculture	Quantile-SFA	hqer_induced	True	15	-2849.9307777949184	hqer_induced sampler prototype	2.0
	WDI_agriculture	HQER-SFA	hqer_induced	True	20	-2849.8605378462144	hqer_induced sampler prototype	2.0
	WDI_agriculture	Quantile-SFA	hqer_induced	True	24	-2819.73346067685	hqer_induced sampler prototype	5.0
	WDI_agriculture	HQER-SFA	hqer_induced	True	29	-2819.6548739528826	hqer_induced sampler prototype	5.0
	WDI_agriculture	Quantile-SFA	hqer_induced	True	21	-2874.473554837591	hqer_induced sampler prototype	2.0
	WDI_agriculture	HQER-SFA	hqer_induced	True	24	-2874.398179513993	hqer_induced sampler prototype	5.0
	WDI_agriculture	Quantile-SFA	hqer_induced	True	18	-2897.421979375505	hqer_induced sampler prototype	5.0
	WDI_agriculture	HQER-SFA	hqer_induced	True	16	-2897.3724319079547	hqer_induced sampler prototype	5.0
	WDI_agriculture	Quantile-SFA	hqer_induced	True	22	-2822.8002972469053	hqer_induced sampler prototype	1.0
	WDI_agriculture	HQER-SFA	hqer_induced	True	12	-2822.7242054637422	hqer_induced sampler prototype	2.0
	WDI_agriculture	Quantile-SFA	hqer_induced	True	25	-2905.6619141614274	hqer_induced sampler prototype	3.0
	WDI_agriculture	HQER-SFA	hqer_induced	True	23	-2905.590079901298	hqer_induced sampler prototype	2.0
	WDI_agriculture	Quantile-SFA	hqer_induced	True	23	-2865.3773878860284	hqer_induced sampler prototype	1.0
	WDI_agriculture	HQER-SFA	hqer_induced	True	25	-2865.2640987896684	hqer_induced sampler prototype	5.0
	WDI_agriculture	Quantile-SFA	hqer_induced	True	28	-2895.9528561008865	hqer_induced sampler prototype	3.0
	WDI_agriculture	HQER-SFA	hqer_induced	True	21	-2896.145770476948	hqer_induced sampler prototype	1.0
	WDI_agriculture	Quantile-SFA	hqer_induced	True	25	-2832.1886712868327	hqer_induced sampler prototype	1.0
	WDI_agriculture	HQER-SFA	hqer_induced	True	11	-2832.119090092705	hqer_induced sampler prototype	2.0
	WDI_agriculture	Quantile-SFA	hqer_induced	True	24	-2935.479136853732	hqer_induced sampler prototype	3.0
	WDI_agriculture	HQER-SFA	hqer_induced	True	23	-2935.3832477818232	hqer_induced sampler prototype	3.0
	WDI_agriculture	Quantile-SFA	hqer_induced	True	8	-2898.949854603261	hqer_induced sampler prototype	2.0
	WDI_agriculture	HQER-SFA	hqer_induced	True	26	-2898.8128000418783	hqer_induced sampler prototype	1.0
	WDI_agriculture	Quantile-SFA	hqer_induced	True	23	-2871.5333308623376	hqer_induced sampler prototype	1.0
	WDI_agriculture	HQER-SFA	hqer_induced	True	12	-2871.487634046931	hqer_induced sampler prototype	2.0
	WDI_agriculture	Quantile-SFA	hqer_induced	True	24	-2855.1225247829284	hqer_induced sampler prototype	5.0
	WDI_agriculture	HQER-SFA	hqer_induced	True	23	-2855.026910709903	hqer_induced sampler prototype	2.0
	WDI_agriculture	Quantile-SFA	hqer_induced	True	25	-2904.6063252087697	hqer_induced sampler prototype	1.0
	WDI_agriculture	HQER-SFA	hqer_induced	True	19	-2904.543213680796	hqer_induced sampler prototype	1.0
	WDI_agriculture	Quantile-SFA	hqer_induced	True	34	-726.4424375194919	hqer_induced sampler prototype	5.0
	WDI_agriculture	HQER-SFA	hqer_induced	True	7	-717.5938472564712	hqer_induced sampler prototype	2.0
	WDI_agriculture	Quantile-SFA	hqer_induced	True	17	-718.9185174328716	hqer_induced sampler prototype	3.0
	WDI_agriculture	HQER-SFA	hqer_induced	True	8	-707.9290380196879	hqer_induced sampler prototype	2.0
	WDI_agriculture	Quantile-SFA	hqer_induced	True	19	-731.6405773870216	hqer_induced sampler prototype	5.0
	WDI_agriculture	HQER-SFA	hqer_induced	True	10	-723.6308094343003	hqer_induced sampler prototype	4.0
	WDI_agriculture	Quantile-SFA	hqer_induced	True	35	-702.7665857426551	hqer_induced sampler prototype	3.0
	WDI_agriculture	HQER-SFA	hqer_induced	True	18	-695.7521671029295	hqer_induced sampler prototype	2.0
	WDI_agriculture	Quantile-SFA	hqer_induced	True	19	-706.0960630456761	hqer_induced sampler prototype	5.0
	WDI_agriculture	HQER-SFA	hqer_induced	True	15	-696.8124444211444	hqer_induced sampler prototype	4.0
	WDI_agriculture	Quantile-SFA	hqer_induced	True	35	-731.2398780186443	hqer_induced sampler prototype	5.0
	WDI_agriculture	HQER-SFA	hqer_induced	True	15	-723.3609593890076	hqer_induced sampler prototype	2.0
	WDI_agriculture	Quantile-SFA	hqer_induced	True	33	-732.4565375280536	hqer_induced sampler prototype	1.0
	WDI_agriculture	HQER-SFA	hqer_induced	True	10	-725.9158156711294	hqer_induced sampler prototype	4.0
	WDI_agriculture	Quantile-SFA	hqer_induced	True	30	-731.9000268862812	hqer_induced sampler prototype	1.0
	WDI_agriculture	HQER-SFA	hqer_induced	True	8	-724.2222079758981	hqer_induced sampler prototype	4.0
	WDI_agriculture	Quantile-SFA	hqer_induced	True	35	-718.8420785309947	hqer_induced sampler prototype	3.0
	WDI_agriculture	HQER-SFA	hqer_induced	True	9	-709.6731465254178	hqer_induced sampler prototype	2.0
	WDI_agriculture	Quantile-SFA	hqer_induced	True	27	-722.5494619358735	hqer_induced sampler prototype	1.0
	WDI_agriculture	HQER-SFA	hqer_induced	True	39	-718.7139728376987	hqer_induced sampler prototype	5.0
	WDI_agriculture	Quantile-SFA	hqer_induced	True	41	-695.9621517631376	hqer_induced sampler prototype	1.0
	WDI_agriculture	HQER-SFA	hqer_induced	True	14	-692.7421959015244	hqer_induced sampler prototype	2.0
	WDI_agriculture	Quantile-SFA	hqer_induced	True	23	-734.8122382292038	hqer_induced sampler prototype	5.0
	WDI_agriculture	HQER-SFA	hqer_induced	True	10	-727.3199527605306	hqer_induced sampler prototype	4.0
	WDI_agriculture	Quantile-SFA	hqer_induced	True	21	-724.8946004445202	hqer_induced sampler prototype	5.0
	WDI_agriculture	HQER-SFA	hqer_induced	True	13	-719.3332373054686	hqer_induced sampler prototype	2.0
	WDI_agriculture	Quantile-SFA	hqer_induced	True	29	-720.6276752737242	hqer_induced sampler prototype	1.0
	WDI_agriculture	HQER-SFA	hqer_induced	True	20	-710.8456078310808	hqer_induced sampler prototype	2.0
	WDI_agriculture	Quantile-SFA	hqer_induced	True	32	-697.4861883298092	hqer_induced sampler prototype	1.0
	WDI_agriculture	HQER-SFA	hqer_induced	True	28	-690.5720330561812	hqer_induced sampler prototype	4.0
	WDI_agriculture	Quantile-SFA	hqer_induced	True	24	-739.3892040831598	hqer_induced sampler prototype	1.0
	WDI_agriculture	HQER-SFA	hqer_induced	True	23	-735.8699204082352	hqer_induced sampler prototype	2.0
	WDI_agriculture	Quantile-SFA	hqer_induced	True	28	-692.9546222816439	hqer_induced sampler prototype	1.0
	WDI_agriculture	HQER-SFA	hqer_induced	True	15	-688.5504675807448	hqer_induced sampler prototype	2.0
	WDI_agriculture	Quantile-SFA	hqer_induced	True	34	-691.2034676206206	hqer_induced sampler prototype	3.0
	WDI_agriculture	HQER-SFA	hqer_induced	True	10	-680.8295021136937	hqer_induced sampler prototype	4.0
	WDI_agriculture	Quantile-SFA	hqer_induced	True	28	-710.2510983825671	hqer_induced sampler prototype	3.0
	WDI_agriculture	HQER-SFA	hqer_induced	True	22	-702.2356871261516	hqer_induced sampler prototype	2.0
	WDI_agriculture	Quantile-SFA	hqer_induced	True	36	-711.2703810475901	hqer_induced sampler prototype	1.0
	WDI_agriculture	HQER-SFA	hqer_induced	True	8	-706.3495813519066	hqer_induced sampler prototype	2.0
	WDI_agriculture	Quantile-SFA	hqer_induced	True	23	-716.2519748393977	hqer_induced sampler prototype	5.0
	WDI_agriculture	HQER-SFA	hqer_induced	True	6	-709.9045750347175	hqer_induced sampler prototype	4.0
	WDI_agriculture	Quantile-SFA	hqer_induced	True	17	-678.4269330059058	hqer_induced sampler prototype	5.0
	WDI_agriculture	HQER-SFA	hqer_induced	True	13	-671.559962783929	hqer_induced sampler prototype	2.0
	WDI_agriculture	Quantile-SFA	hqer_induced	True	38	-692.6958207969287	hqer_induced sampler prototype	1.0
	WDI_agriculture	HQER-SFA	hqer_induced	True	20	-687.417082784245	hqer_induced sampler prototype	4.0
	WDI_agriculture	Quantile-SFA	hqer_induced	True	26	-733.0168770998131	hqer_induced sampler prototype	5.0
	WDI_agriculture	HQER-SFA	hqer_induced	True	7	-726.1026821384528	hqer_induced sampler prototype	2.0
	WDI_agriculture	Quantile-SFA	hqer_induced	True	33	-724.2753752058617	hqer_induced sampler prototype	1.0
	WDI_agriculture	HQER-SFA	hqer_induced	True	8	-721.68118324412	hqer_induced sampler prototype	2.0
	WDI_agriculture	Quantile-SFA	hqer_induced	True	29	-692.5745231458855	hqer_induced sampler prototype	5.0

experiment	dataset	model	scenario	converged	n__iter	loglik	warning__message	best__start__id
	WDI__agriculture	HQER-SFA	hqer__induced	True	22	-687.3246261259646	hqer__induced sampler prototype	4.0
	WDI__agriculture	Quantile-SFA	hqer__induced	True	25	-727.6302422754093	hqer__induced sampler prototype	3.0
	WDI__agriculture	HQER-SFA	hqer__induced	True	8	-724.7116886009284	hqer__induced sampler prototype	2.0
	WDI__agriculture	Quantile-SFA	hqer__induced	True	29	-713.9208014273377	hqer__induced sampler prototype	1.0
	WDI__agriculture	HQER-SFA	hqer__induced	True	8	-708.1658303386935	hqer__induced sampler prototype	2.0
	WDI__agriculture	Quantile-SFA	hqer__induced	True	24	-710.1225766930338	hqer__induced sampler prototype	1.0
	WDI__agriculture	HQER-SFA	hqer__induced	True	10	-705.1032919223377	hqer__induced sampler prototype	2.0
	WDI__agriculture	Quantile-SFA	hqer__induced	True	29	-703.3579327801519	hqer__induced sampler prototype	3.0
	WDI__agriculture	HQER-SFA	hqer__induced	True	21	-680.0031883013353	hqer__induced sampler prototype	3.0
	WDI__agriculture	Quantile-SFA	hqer__induced	True	28	-710.6454614791135	hqer__induced sampler prototype	5.0
	WDI__agriculture	HQER-SFA	hqer__induced	True	7	-704.608823366242	hqer__induced sampler prototype	2.0
	WDI__agriculture	Quantile-SFA	hqer__induced	True	27	-728.9253962383771	hqer__induced sampler prototype	1.0
	WDI__agriculture	HQER-SFA	hqer__induced	True	10	-719.3761826444157	hqer__induced sampler prototype	4.0
	WDI__agriculture	Quantile-SFA	hqer__induced	True	28	-757.0409724286294	hqer__induced sampler prototype	5.0
	WDI__agriculture	HQER-SFA	hqer__induced	True	12	-752.443333286714	hqer__induced sampler prototype	4.0
	WDI__agriculture	Quantile-SFA	hqer__induced	True	24	-713.1504010654473	hqer__induced sampler prototype	5.0
	WDI__agriculture	HQER-SFA	hqer__induced	True	14	-712.0199431176243	hqer__induced sampler prototype	4.0
	WDI__agriculture	Quantile-SFA	hqer__induced	True	20	-721.4450082911358	hqer__induced sampler prototype	5.0
	WDI__agriculture	HQER-SFA	hqer__induced	True	8	-714.5690846696762	hqer__induced sampler prototype	2.0
	WDI__agriculture	Quantile-SFA	hqer__induced	True	34	-717.725139112735	hqer__induced sampler prototype	1.0
	WDI__agriculture	HQER-SFA	hqer__induced	True	10	-711.582806091793	hqer__induced sampler prototype	2.0
	WDI__agriculture	Quantile-SFA	hqer__induced	True	24	-719.8313614050238	hqer__induced sampler prototype	5.0
	WDI__agriculture	HQER-SFA	hqer__induced	True	12	-713.1681499594583	hqer__induced sampler prototype	4.0
	WDI__agriculture	Quantile-SFA	hqer__induced	True	30	-729.6620839248687	hqer__induced sampler prototype	1.0
	WDI__agriculture	HQER-SFA	hqer__induced	True	16	-721.806709513738	hqer__induced sampler prototype	4.0
	WDI__agriculture	Quantile-SFA	hqer__induced	True	20	-677.2740988176922	hqer__induced sampler prototype	5.0
	WDI__agriculture	HQER-SFA	hqer__induced	True	23	-673.2011948418343	hqer__induced sampler prototype	4.0
	WDI__agriculture	Quantile-SFA	hqer__induced	True	26	-737.7780371421825	hqer__induced sampler prototype	1.0
	WDI__agriculture	HQER-SFA	hqer__induced	True	8	-727.9086937800448	hqer__induced sampler prototype	2.0
	WDI__agriculture	Quantile-SFA	hqer__induced	True	26	-718.1140621445314	hqer__induced sampler prototype	3.0
	WDI__agriculture	HQER-SFA	hqer__induced	True	18	-714.7825649745629	hqer__induced sampler prototype	4.0
	WDI__agriculture	Quantile-SFA	hqer__induced	True	21	-727.5067290664683	hqer__induced sampler prototype	5.0
	WDI__agriculture	HQER-SFA	hqer__induced	True	14	-721.3173566749783	hqer__induced sampler prototype	4.0
	WDI__agriculture	Quantile-SFA	hqer__induced	True	21	-705.4783078161538	hqer__induced sampler prototype	3.0
	WDI__agriculture	HQER-SFA	hqer__induced	True	17	-703.1130388464713	hqer__induced sampler prototype	4.0
	WDI__agriculture	Quantile-SFA	hqer__induced	True	20	-757.0121877481363	hqer__induced sampler prototype	5.0
	WDI__agriculture	HQER-SFA	hqer__induced	True	12	-751.4856953964461	hqer__induced sampler prototype	4.0
	WDI__agriculture	Quantile-SFA	hqer__induced	True	34	-746.7538067040487	hqer__induced sampler prototype	5.0
	WDI__agriculture	HQER-SFA	hqer__induced	True	15	-739.4079996127201	hqer__induced sampler prototype	4.0
	WDI__agriculture	Quantile-SFA	hqer__induced	True	26	-731.7874653441248	hqer__induced sampler prototype	5.0
	WDI__agriculture	HQER-SFA	hqer__induced	True	9	-723.2302517443279	hqer__induced sampler prototype	4.0
	WDI__agriculture	Quantile-SFA	hqer__induced	True	29	-720.8527987606207	hqer__induced sampler prototype	3.0
	WDI__agriculture	HQER-SFA	hqer__induced	True	17	-710.5372034883796	hqer__induced sampler prototype	4.0
	WDI__agriculture	Quantile-SFA	hqer__induced	True	21	-714.1590969220102	hqer__induced sampler prototype	5.0
	WDI__agriculture	HQER-SFA	hqer__induced	True	14	-707.6727543417285	hqer__induced sampler prototype	2.0
	WDI__agriculture	Quantile-SFA	hqer__induced	True	26	-715.583073119853	hqer__induced sampler prototype	5.0
	WDI__agriculture	HQER-SFA	hqer__induced	True	7	-710.2473275727973	hqer__induced sampler prototype	2.0
	WDI__agriculture	Quantile-SFA	hqer__induced	True	22	-737.3397750599186	hqer__induced sampler prototype	1.0
	WDI__agriculture	HQER-SFA	hqer__induced	True	17	-730.8707618973306	hqer__induced sampler prototype	4.0
	WDI__agriculture	Quantile-SFA	hqer__induced	True	32	-711.6971284648132	hqer__induced sampler prototype	5.0
	WDI__agriculture	HQER-SFA	hqer__induced	True	19	-703.8174480450484	hqer__induced sampler prototype	2.0
	WDI__agriculture	Quantile-SFA	hqer__induced	True	26	-717.8433296636297	hqer__induced sampler prototype	1.0
	WDI__agriculture	HQER-SFA	hqer__induced	True	14	-710.122748095542	hqer__induced sampler prototype	4.0
	WDI__agriculture	Quantile-SFA	hqer__induced	True	17	-702.8901501052683	hqer__induced sampler prototype	5.0
	WDI__agriculture	HQER-SFA	hqer__induced	True	9	-700.1522809478138	hqer__induced sampler prototype	2.0
	WDI__agriculture	Quantile-SFA	hqer__induced	True	28	-707.3267955990269	hqer__induced sampler prototype	5.0
	WDI__agriculture	HQER-SFA	hqer__induced	True	13	-701.7660958395313	hqer__induced sampler prototype	2.0
	WDI__agriculture	Quantile-SFA	hqer__induced	True	54	-733.8902025465027	hqer__induced sampler prototype	1.0
	WDI__agriculture	HQER-SFA	hqer__induced	True	14	-725.9528999338362	hqer__induced sampler prototype	4.0
	WDI__agriculture	Quantile-SFA	hqer__induced	True	36	-736.078628556543	hqer__induced sampler prototype	1.0
	WDI__agriculture	HQER-SFA	hqer__induced	True	9	-732.0408382718847	hqer__induced sampler prototype	2.0
	WDI__agriculture	Quantile-SFA	hqer__induced	True	29	-717.1648711168499	hqer__induced sampler prototype	5.0
	WDI__agriculture	HQER-SFA	hqer__induced	True	9	-718.3962438407401	hqer__induced sampler prototype	2.0
	WDI__agriculture	Quantile-SFA	hqer__induced	True	52	-757.4187126926439	hqer__induced sampler prototype	3.0
	WDI__agriculture	HQER-SFA	hqer__induced	True	12	-753.0706131718742	hqer__induced sampler prototype	2.0
	WDI__agriculture	Quantile-SFA	hqer__induced	True	23	-702.9332942303481	hqer__induced sampler prototype	5.0
	WDI__agriculture	HQER-SFA	hqer__induced	True	16	-691.0434680505953	hqer__induced sampler prototype	2.0
	WDI__agriculture	Quantile-SFA	hqer__induced	True	35	-741.6289925368684	hqer__induced sampler prototype	3.0
	WDI__agriculture	HQER-SFA	hqer__induced	True	7	-734.8690877444308	hqer__induced sampler prototype	4.0
	WDI__agriculture	Quantile-SFA	hqer__induced	True	31	-731.1796526083481	hqer__induced sampler prototype	5.0
	WDI__agriculture	HQER-SFA	hqer__induced	True	8	-724.0158382978632	hqer__induced sampler prototype	4.0
	WDI__agriculture	Quantile-SFA	hqer__induced	True	25	-690.400428167807	hqer__induced sampler prototype	1.0
	WDI__agriculture	HQER-SFA	hqer__induced	True	13	-682.4704377224025	hqer__induced sampler prototype	2.0
	WDI__agriculture	Quantile-SFA	hqer__induced	True	21	-724.4095273361069	hqer__induced sampler prototype	3.0
	WDI__agriculture	HQER-SFA	hqer__induced	True	8	-716.5363271645583	hqer__induced sampler prototype	4.0
	WDI__agriculture	Quantile-SFA	hqer__induced	True	31	-718.154953571651	hqer__induced sampler prototype	5.0
	WDI__agriculture	HQER-SFA	hqer__induced	True	10	-716.6266455868882	hqer__induced sampler prototype	4.0
	WDI__agriculture	Quantile-SFA	hqer__induced	True	35	-680.8671080189238	hqer__induced sampler prototype	1.0
	WDI__agriculture	HQER-SFA	hqer__induced	True	10	-681.337029316707	hqer__induced sampler prototype	2.0
	WDI__agriculture	Quantile-SFA	hqer__induced	True	40	-716.9283615630266	hqer__induced sampler prototype	1.0
	WDI__agriculture	HQER-SFA	hqer__induced	True	11	-711.7929660499794	hqer__induced sampler prototype	4.0
	WDI__agriculture	Quantile-SFA	hqer__induced	True	34	-720.7297784977659	hqer__induced sampler prototype	3.0
	WDI__agriculture	HQER-SFA	hqer__induced	True	12	-717.2668508287163	hqer__induced sampler prototype	2.0
	WDI__agriculture	Quantile-SFA	hqer__induced	True	26	-731.0723752429276	hqer__induced sampler prototype	3.0
	WDI__agriculture	HQER-SFA	hqer__induced	True	19	-725.6834460125183	hqer__induced sampler prototype	2.0
	WDI__agriculture	Quantile-SFA	hqer__induced	True	24	-734.2711234731711	hqer__induced sampler prototype	5.0
	WDI__agriculture	HQER-SFA	hqer__induced	True	10	-729.4949762440165	hqer__induced sampler prototype	4.0
	WDI__agriculture	Quantile-SFA	hqer__induced	True	23	-702.2563179685322	hqer__induced sampler prototype	5.0
	WDI__agriculture	HQER-SFA	hqer__induced	True	7	-697.5198563476986	hqer__induced sampler prototype	2.0
	WDI__agriculture	Quantile-SFA	hqer__induced	True	15	-675.6118028317992	hqer__induced sampler prototype	5.0
	WDI__agriculture	HQER-SFA	hqer__induced	True	11	-668.7953196213782	hqer__induced sampler prototype	4.0
	WDI__agriculture	Quantile-SFA	hqer__induced	True	34	-728.2065794462727	hqer__induced sampler prototype	3.0
	WDI__agriculture	HQER-SFA	hqer__induced	True	10	-723.8340701231448	hqer__induced sampler prototype	4.0
	WDI__agriculture	Quantile-SFA	hqer__induced	True	23	-722.973081803502	hqer__induced sampler prototype	3.0
	WDI__agriculture	HQER-SFA	hqer__induced	True	6	-714.5816197127746	hqer__induced sampler prototype	4.0
	WDI__agriculture	Quantile-SFA	hqer__induced	True	43	-731.6636957310278	hqer__induced sampler prototype	1.0
	WDI__agriculture	HQER-SFA	hqer__induced	True	8	-725.8520046222415	hqer__induced sampler prototype	2.0
	WDI__agriculture	Quantile-SFA	hqer__induced	True	29	-694.6266369013946	hqer__induced sampler prototype	1.0
	WDI__agriculture	HQER-SFA	hqer__induced	True	16	-689.2187597394782	hqer__induced sampler prototype	2.0
	WDI__agriculture	Quantile-SFA	hqer__induced	True	25	-704.5714618637918	hqer__induced sampler prototype	5.0
	WDI__agriculture	HQER-SFA	hqer__induced	True	17	-697.058774877353	hqer__induced sampler prototype	2.0
	WDI__agriculture	Quantile-SFA	hqer__induced	True	19	-690.3836951030503	hqer__induced sampler prototype	3.0
	WDI__agriculture	HQER-SFA	hqer__induced	True	11	-682.1318579541564	hqer__induced sampler prototype	4.0

experiment	dataset	model	scenario	converged	n_iter	loglik	warning_message	best_start_id
	WDI_agriculture	Quantile-SFA	hqer_induced	True	28	-742.037146868578	hqer_induced sampler prototype	3.0
	WDI_agriculture	HQER-SFA	hqer_induced	True	9	-738.6109328596075	hqer_induced sampler prototype	2.0
	WDI_agriculture	Quantile-SFA	hqer_induced	True	30	-699.6273951658816	hqer_induced sampler prototype	5.0
	WDI_agriculture	HQER-SFA	hqer_induced	True	12	-696.1259517591678	hqer_induced sampler prototype	2.0
	WDI_agriculture	Quantile-SFA	hqer_induced	True	24	-709.6756564555683	hqer_induced sampler prototype	5.0
	WDI_agriculture	HQER-SFA	hqer_induced	True	6	-701.3923715483713	hqer_induced sampler prototype	4.0
	WDI_agriculture	Quantile-SFA	hqer_induced	True	25	-719.4708727917932	hqer_induced sampler prototype	3.0
	WDI_agriculture	HQER-SFA	hqer_induced	True	21	-710.2037466227983	hqer_induced sampler prototype	4.0
	WDI_agriculture	Quantile-SFA	hqer_induced	True	23	-708.8984005802954	hqer_induced sampler prototype	1.0
	WDI_agriculture	HQER-SFA	hqer_induced	True	12	-701.4213336554413	hqer_induced sampler prototype	4.0
	WDI_agriculture	Quantile-SFA	hqer_induced	True	16	-729.5474272550307	hqer_induced sampler prototype	5.0
	WDI_agriculture	HQER-SFA	hqer_induced	True	16	-718.2529414649499	hqer_induced sampler prototype	2.0
	WDI_agriculture	Quantile-SFA	hqer_induced	True	25	-723.3688027653583	hqer_induced sampler prototype	1.0
	WDI_agriculture	HQER-SFA	hqer_induced	True	14	-714.703583372149	hqer_induced sampler prototype	2.0
	WDI_agriculture	Quantile-SFA	hqer_induced	True	20	-707.4366882905881	hqer_induced sampler prototype	3.0
	WDI_agriculture	HQER-SFA	hqer_induced	True	15	-705.944480996184	hqer_induced sampler prototype	2.0
	WDI_agriculture	Quantile-SFA	hqer_induced	True	23	-708.122642522191	hqer_induced sampler prototype	5.0
	WDI_agriculture	HQER-SFA	hqer_induced	True	6	-704.1791370151896	hqer_induced sampler prototype	4.0
	WDI_agriculture	Quantile-SFA	hqer_induced	True	23	-715.8431212173948	hqer_induced sampler prototype	3.0
	WDI_agriculture	HQER-SFA	hqer_induced	True	11	-706.3714515536712	hqer_induced sampler prototype	2.0
	WDI_agriculture	Quantile-SFA	hqer_induced	True	24	-717.9060712533332	hqer_induced sampler prototype	5.0
	WDI_agriculture	HQER-SFA	hqer_induced	True	24	-709.1981713619664	hqer_induced sampler prototype	4.0
	WDI_agriculture	Quantile-SFA	hqer_induced	True	27	-698.5463496077035	hqer_induced sampler prototype	5.0
	WDI_agriculture	HQER-SFA	hqer_induced	True	9	-690.5077646079953	hqer_induced sampler prototype	4.0
	WDI_agriculture	Quantile-SFA	hqer_induced	True	26	-712.0868852519054	hqer_induced sampler prototype	3.0
	WDI_agriculture	HQER-SFA	hqer_induced	True	19	-704.1444113822826	hqer_induced sampler prototype	4.0
	WDI_agriculture	Quantile-SFA	hqer_induced	True	31	-711.0484269159113	hqer_induced sampler prototype	1.0
	WDI_agriculture	HQER-SFA	hqer_induced	True	15	-704.4722160617795	hqer_induced sampler prototype	2.0
	WDI_agriculture	Quantile-SFA	hqer_induced	True	29	-748.3748533561486	hqer_induced sampler prototype	1.0
	WDI_agriculture	HQER-SFA	hqer_induced	True	11	-744.3240455957352	hqer_induced sampler prototype	2.0
	WDI_agriculture	Quantile-SFA	hqer_induced	True	31	-709.4817868257842	hqer_induced sampler prototype	1.0
	WDI_agriculture	HQER-SFA	hqer_induced	True	5	-706.0710413248631	hqer_induced sampler prototype	2.0
	WDI_agriculture	Quantile-SFA	hqer_induced	True	28	-730.91983375767	hqer_induced sampler prototype	1.0
	WDI_agriculture	HQER-SFA	hqer_induced	True	15	-727.62930213409	hqer_induced sampler prototype	2.0
	WDI_agriculture	Quantile-SFA	hqer_induced	True	33	-738.1123797060168	hqer_induced sampler prototype	1.0
	WDI_agriculture	HQER-SFA	hqer_induced	True	18	-733.5990617404293	hqer_induced sampler prototype	2.0
	WDI_agriculture	Quantile-SFA	hqer_induced	True	32	-677.4702801639482	hqer_induced sampler prototype	5.0
	WDI_agriculture	HQER-SFA	hqer_induced	True	11	-674.534015605102	hqer_induced sampler prototype	2.0
	WDI_agriculture	Quantile-SFA	hqer_induced	True	21	-733.7934040194631	hqer_induced sampler prototype	3.0
	WDI_agriculture	HQER-SFA	hqer_induced	True	17	-722.4899560131581	hqer_induced sampler prototype	2.0
	WDI_agriculture	Quantile-SFA	hqer_induced	True	25	-677.9208228257548	hqer_induced sampler prototype	5.0
	WDI_agriculture	HQER-SFA	hqer_induced	True	38	-673.5392398312745	hqer_induced sampler prototype	3.0
	WDI_agriculture	Quantile-SFA	hqer_induced	True	19	-742.3655192147667	hqer_induced sampler prototype	5.0
	WDI_agriculture	HQER-SFA	hqer_induced	True	14	-733.379681500568	hqer_induced sampler prototype	2.0
	WDI_agriculture	Quantile-SFA	hqer_induced	True	26	-690.9676321464935	hqer_induced sampler prototype	5.0
	WDI_agriculture	HQER-SFA	hqer_induced	True	17	-683.6281488918478	hqer_induced sampler prototype	2.0
	WDI_agriculture	Quantile-SFA	hqer_induced	True	15	-2884.4738274337474	hqer_induced sampler prototype	5.0
	WDI_agriculture	HQER-SFA	hqer_induced	True	12	-2865.774596476232	hqer_induced sampler prototype	2.0
	WDI_agriculture	Quantile-SFA	hqer_induced	True	27	-2863.037486068746	hqer_induced sampler prototype	1.0
	WDI_agriculture	HQER-SFA	hqer_induced	True	13	-2832.8574960719934	hqer_induced sampler prototype	4.0
	WDI_agriculture	Quantile-SFA	hqer_induced	True	24	-2892.6944851668472	hqer_induced sampler prototype	3.0
	WDI_agriculture	HQER-SFA	hqer_induced	True	7	-2864.750654074416	hqer_induced sampler prototype	2.0
	WDI_agriculture	Quantile-SFA	hqer_induced	True	22	-2883.9649860730374	hqer_induced sampler prototype	3.0
	WDI_agriculture	HQER-SFA	hqer_induced	True	4	-2860.659635056216	hqer_induced sampler prototype	2.0
	WDI_agriculture	Quantile-SFA	hqer_induced	True	36	-2891.0480132325856	hqer_induced sampler prototype	3.0
	WDI_agriculture	HQER-SFA	hqer_induced	True	7	-2864.170253779309	hqer_induced sampler prototype	2.0
	WDI_agriculture	Quantile-SFA	hqer_induced	True	22	-2864.970742083251	hqer_induced sampler prototype	5.0
	WDI_agriculture	HQER-SFA	hqer_induced	True	6	-2839.590527619416	hqer_induced sampler prototype	2.0
	WDI_agriculture	Quantile-SFA	hqer_induced	True	24	-2844.216018160188	hqer_induced sampler prototype	1.0
	WDI_agriculture	HQER-SFA	hqer_induced	True	8	-2818.561998074392	hqer_induced sampler prototype	2.0
	WDI_agriculture	Quantile-SFA	hqer_induced	True	20	-2858.0913159748216	hqer_induced sampler prototype	5.0
	WDI_agriculture	HQER-SFA	hqer_induced	True	10	-2831.2025575562307	hqer_induced sampler prototype	2.0
	WDI_agriculture	Quantile-SFA	hqer_induced	True	23	-2883.646094942331	hqer_induced sampler prototype	1.0
	WDI_agriculture	HQER-SFA	hqer_induced	True	12	-2850.9900846970218	hqer_induced sampler prototype	2.0
	WDI_agriculture	Quantile-SFA	hqer_induced	True	18	-2892.9327447200903	hqer_induced sampler prototype	1.0
	WDI_agriculture	HQER-SFA	hqer_induced	True	12	-2863.977373421493	hqer_induced sampler prototype	4.0
	WDI_agriculture	Quantile-SFA	hqer_induced	True	23	-2856.50369306017	hqer_induced sampler prototype	1.0
	WDI_agriculture	HQER-SFA	hqer_induced	True	8	-2833.2665892345344	hqer_induced sampler prototype	4.0
	WDI_agriculture	Quantile-SFA	hqer_induced	True	22	-2891.3143584361296	hqer_induced sampler prototype	5.0
	WDI_agriculture	HQER-SFA	hqer_induced	True	8	-2868.4786354042985	hqer_induced sampler prototype	2.0
	WDI_agriculture	Quantile-SFA	hqer_induced	True	22	-2868.65336237708	hqer_induced sampler prototype	3.0
	WDI_agriculture	HQER-SFA	hqer_induced	True	9	-2842.509027486348	hqer_induced sampler prototype	4.0
	WDI_agriculture	Quantile-SFA	hqer_induced	True	22	-2922.109420652952	hqer_induced sampler prototype	3.0
	WDI_agriculture	HQER-SFA	hqer_induced	True	6	-2892.478905604371	hqer_induced sampler prototype	2.0
	WDI_agriculture	Quantile-SFA	hqer_induced	True	15	-2909.893488963043	hqer_induced sampler prototype	5.0
	WDI_agriculture	HQER-SFA	hqer_induced	True	12	-2882.751269012301	hqer_induced sampler prototype	4.0
	WDI_agriculture	Quantile-SFA	hqer_induced	True	22	-2879.66316209804	hqer_induced sampler prototype	5.0
	WDI_agriculture	HQER-SFA	hqer_induced	True	8	-2846.7181163506693	hqer_induced sampler prototype	4.0
	WDI_agriculture	Quantile-SFA	hqer_induced	True	15	-2886.8173008557405	hqer_induced sampler prototype	5.0
	WDI_agriculture	HQER-SFA	hqer_induced	True	7	-2857.8618830848563	hqer_induced sampler prototype	2.0
	WDI_agriculture	Quantile-SFA	hqer_induced	True	23	-2880.746590752932	hqer_induced sampler prototype	3.0
	WDI_agriculture	HQER-SFA	hqer_induced	True	11	-2847.813265407553	hqer_induced sampler prototype	4.0
	WDI_agriculture	Quantile-SFA	hqer_induced	True	27	-2854.0188288231075	hqer_induced sampler prototype	3.0
	WDI_agriculture	HQER-SFA	hqer_induced	True	11	-2823.304580211154	hqer_induced sampler prototype	4.0
	WDI_agriculture	Quantile-SFA	hqer_induced	True	36	-2838.806326888204	hqer_induced sampler prototype	3.0
	WDI_agriculture	HQER-SFA	hqer_induced	True	7	-2806.423214407377	hqer_induced sampler prototype	4.0
	WDI_agriculture	Quantile-SFA	hqer_induced	True	18	-2866.724688898189	hqer_induced sampler prototype	5.0
	WDI_agriculture	HQER-SFA	hqer_induced	True	8	-2841.79449160922	hqer_induced sampler prototype	2.0
	WDI_agriculture	Quantile-SFA	hqer_induced	True	24	-2941.913010069932	hqer_induced sampler prototype	5.0
	WDI_agriculture	HQER-SFA	hqer_induced	True	13	-2913.3968484851907	hqer_induced sampler prototype	4.0
	WDI_agriculture	Quantile-SFA	hqer_induced	True	30	-2904.36128191532	hqer_induced sampler prototype	1.0
	WDI_agriculture	HQER-SFA	hqer_induced	True	19	-2875.9928207748835	hqer_induced sampler prototype	4.0
	WDI_agriculture	Quantile-SFA	hqer_induced	True	26	-2883.4982795488763	hqer_induced sampler prototype	3.0
	WDI_agriculture	HQER-SFA	hqer_induced	True	12	-2857.8618904852538	hqer_induced sampler prototype	2.0
	WDI_agriculture	Quantile-SFA	hqer_induced	True	23	-2828.244481467934	hqer_induced sampler prototype	5.0
	WDI_agriculture	HQER-SFA	hqer_induced	True	8	-2803.4772005017394	hqer_induced sampler prototype	2.0
	WDI_agriculture	Quantile-SFA	hqer_induced	True	22	-2853.830828262661	hqer_induced sampler prototype	5.0
	WDI_agriculture	HQER-SFA	hqer_induced	True	12	-2827.907790289909	hqer_induced sampler prototype	4.0
	WDI_agriculture	Quantile-SFA	hqer_induced	True	27	-2886.556787169104	hqer_induced sampler prototype	3.0
	WDI_agriculture	HQER-SFA	hqer_induced	True	7	-2868.647757528696	hqer_induced sampler prototype	4.0
	WDI_agriculture	Quantile-SFA	hqer_induced	True	34	-2871.4144394104096	hqer_induced sampler prototype	3.0
	WDI_agriculture	HQER-SFA	hqer_induced	True	11	-2850.218208586837	hqer_induced sampler prototype	4.0
	WDI_agriculture	Quantile-SFA	hqer_induced	True	21	-2843.929143691877	hqer_induced sampler prototype	1.0

experiment	dataset	model	scenario	converged	n_iter	loglik	warning_message	best_start_id
	WDI_agriculture	HQER-SFA	hqer_induced	True	12	-2812.806587540978	hqer_induced sampler prototype	4.0
	WDI_agriculture	Quantile-SFA	hqer_induced	True	20	-2915.7272597265987	hqer_induced sampler prototype	3.0
	WDI_agriculture	HQER-SFA	hqer_induced	True	14	-2886.9318157850867	hqer_induced sampler prototype	2.0
	WDI_agriculture	Quantile-SFA	hqer_induced	True	24	-2870.957290143512	hqer_induced sampler prototype	3.0
	WDI_agriculture	HQER-SFA	hqer_induced	True	6	-2835.6772475608404	hqer_induced sampler prototype	4.0
	WDI_agriculture	Quantile-SFA	hqer_induced	True	22	-2827.2890018980224	hqer_induced sampler prototype	3.0
	WDI_agriculture	HQER-SFA	hqer_induced	True	11	-2798.678221026583	hqer_induced sampler prototype	4.0
	WDI_agriculture	Quantile-SFA	hqer_induced	True	27	-2847.644355012955	hqer_induced sampler prototype	1.0
	WDI_agriculture	HQER-SFA	hqer_induced	True	11	-2831.942547032088	hqer_induced sampler prototype	2.0
	WDI_agriculture	Quantile-SFA	hqer_induced	True	32	-2831.1486460772503	hqer_induced sampler prototype	1.0
	WDI_agriculture	HQER-SFA	hqer_induced	True	4	-2801.5822463629274	hqer_induced sampler prototype	2.0
	WDI_agriculture	Quantile-SFA	hqer_induced	True	25	-2891.334479412348	hqer_induced sampler prototype	3.0
	WDI_agriculture	HQER-SFA	hqer_induced	True	11	-2853.645546959688	hqer_induced sampler prototype	2.0
	WDI_agriculture	Quantile-SFA	hqer_induced	True	23	-2906.533344010107	hqer_induced sampler prototype	3.0
	WDI_agriculture	HQER-SFA	hqer_induced	True	7	-2879.713389644923	hqer_induced sampler prototype	2.0
	WDI_agriculture	Quantile-SFA	hqer_induced	True	28	-2877.05060983068	hqer_induced sampler prototype	3.0
	WDI_agriculture	HQER-SFA	hqer_induced	True	8	-2855.164752546196	hqer_induced sampler prototype	2.0
	WDI_agriculture	Quantile-SFA	hqer_induced	True	19	-2823.6873077332466	hqer_induced sampler prototype	5.0
	WDI_agriculture	HQER-SFA	hqer_induced	True	16	-2784.9468132178645	hqer_induced sampler prototype	2.0
	WDI_agriculture	Quantile-SFA	hqer_induced	True	23	-2857.3616260971207	hqer_induced sampler prototype	5.0
	WDI_agriculture	HQER-SFA	hqer_induced	True	16	-2823.8692781727823	hqer_induced sampler prototype	4.0
	WDI_agriculture	Quantile-SFA	hqer_induced	True	33	-2885.4610907786027	hqer_induced sampler prototype	3.0
	WDI_agriculture	HQER-SFA	hqer_induced	True	9	-2856.461393706741	hqer_induced sampler prototype	4.0
	WDI_agriculture	Quantile-SFA	hqer_induced	True	25	-2945.857211602908	hqer_induced sampler prototype	1.0
	WDI_agriculture	HQER-SFA	hqer_induced	True	8	-2915.877807454038	hqer_induced sampler prototype	4.0
	WDI_agriculture	Quantile-SFA	hqer_induced	True	25	-2894.083078423157	hqer_induced sampler prototype	5.0
	WDI_agriculture	HQER-SFA	hqer_induced	True	7	-2860.062441012184	hqer_induced sampler prototype	2.0
	WDI_agriculture	Quantile-SFA	hqer_induced	True	25	-2935.401161226059	hqer_induced sampler prototype	5.0
	WDI_agriculture	HQER-SFA	hqer_induced	True	8	-2900.5927578315977	hqer_induced sampler prototype	4.0
	WDI_agriculture	Quantile-SFA	hqer_induced	True	29	-2893.49235906098	hqer_induced sampler prototype	3.0
	WDI_agriculture	HQER-SFA	hqer_induced	True	7	-2864.637563503285	hqer_induced sampler prototype	4.0
	WDI_agriculture	Quantile-SFA	hqer_induced	True	28	-2858.668237940453	hqer_induced sampler prototype	5.0
	WDI_agriculture	HQER-SFA	hqer_induced	True	9	-2828.5679077086284	hqer_induced sampler prototype	2.0
	WDI_agriculture	Quantile-SFA	hqer_induced	True	27	-2900.6518518734915	hqer_induced sampler prototype	3.0
	WDI_agriculture	HQER-SFA	hqer_induced	True	8	-2871.536269018862	hqer_induced sampler prototype	4.0
	WDI_agriculture	Quantile-SFA	hqer_induced	True	27	-2965.213886617731	hqer_induced sampler prototype	3.0
	WDI_agriculture	HQER-SFA	hqer_induced	True	14	-2932.972742107224	hqer_induced sampler prototype	4.0
	WDI_agriculture	Quantile-SFA	hqer_induced	True	23	-2943.1505579601253	hqer_induced sampler prototype	1.0
	WDI_agriculture	HQER-SFA	hqer_induced	True	7	-2908.381287726705	hqer_induced sampler prototype	4.0
	WDI_agriculture	Quantile-SFA	hqer_induced	True	30	-2910.143605203108	hqer_induced sampler prototype	5.0
	WDI_agriculture	HQER-SFA	hqer_induced	True	8	-2893.3491882149783	hqer_induced sampler prototype	4.0
	WDI_agriculture	Quantile-SFA	hqer_induced	True	24	-2916.1830672305805	hqer_induced sampler prototype	5.0
	WDI_agriculture	HQER-SFA	hqer_induced	True	13	-2882.289686534547	hqer_induced sampler prototype	4.0
	WDI_agriculture	Quantile-SFA	hqer_induced	True	23	-2910.4186111825884	hqer_induced sampler prototype	1.0
	WDI_agriculture	HQER-SFA	hqer_induced	True	12	-2880.80349056096	hqer_induced sampler prototype	4.0
	WDI_agriculture	Quantile-SFA	hqer_induced	True	22	-2899.5220008950973	hqer_induced sampler prototype	5.0
	WDI_agriculture	HQER-SFA	hqer_induced	True	10	-2865.7199580760694	hqer_induced sampler prototype	4.0
	WDI_agriculture	Quantile-SFA	hqer_induced	True	27	-2863.1521143418613	hqer_induced sampler prototype	1.0
	WDI_agriculture	HQER-SFA	hqer_induced	True	16	-2833.6528197633143	hqer_induced sampler prototype	4.0
	WDI_agriculture	Quantile-SFA	hqer_induced	True	23	-2857.7379062063496	hqer_induced sampler prototype	3.0
	WDI_agriculture	HQER-SFA	hqer_induced	True	10	-2836.423503807534	hqer_induced sampler prototype	2.0
	WDI_agriculture	Quantile-SFA	hqer_induced	True	27	-2901.4386961661244	hqer_induced sampler prototype	3.0
	WDI_agriculture	HQER-SFA	hqer_induced	True	8	-2877.958320694347	hqer_induced sampler prototype	4.0
	WDI_agriculture	Quantile-SFA	hqer_induced	True	26	-2880.998763629825	hqer_induced sampler prototype	5.0
	WDI_agriculture	HQER-SFA	hqer_induced	True	7	-2853.7522897946124	hqer_induced sampler prototype	4.0
	WDI_agriculture	Quantile-SFA	hqer_induced	True	17	-2866.555888300205	hqer_induced sampler prototype	1.0
	WDI_agriculture	HQER-SFA	hqer_induced	True	13	-2835.084071460244	hqer_induced sampler prototype	2.0
	WDI_agriculture	Quantile-SFA	hqer_induced	True	27	-2857.040314387289	hqer_induced sampler prototype	3.0
	WDI_agriculture	HQER-SFA	hqer_induced	True	10	-2824.142692855134	hqer_induced sampler prototype	2.0
	WDI_agriculture	Quantile-SFA	hqer_induced	True	25	-2951.792591968895	hqer_induced sampler prototype	5.0
	WDI_agriculture	HQER-SFA	hqer_induced	True	11	-2930.7331910562884	hqer_induced sampler prototype	4.0
	WDI_agriculture	Quantile-SFA	hqer_induced	True	20	-2817.8203893800087	hqer_induced sampler prototype	5.0
	WDI_agriculture	HQER-SFA	hqer_induced	True	14	-2793.6177365125927	hqer_induced sampler prototype	4.0
	WDI_agriculture	Quantile-SFA	hqer_induced	True	28	-2896.5933554897056	hqer_induced sampler prototype	3.0
	WDI_agriculture	HQER-SFA	hqer_induced	True	8	-2870.308061912845	hqer_induced sampler prototype	2.0
	WDI_agriculture	Quantile-SFA	hqer_induced	True	23	-2853.8858985371817	hqer_induced sampler prototype	3.0
	WDI_agriculture	HQER-SFA	hqer_induced	True	8	-2826.1089475443614	hqer_induced sampler prototype	2.0
	WDI_agriculture	Quantile-SFA	hqer_induced	True	44	-2893.391013335474	hqer_induced sampler prototype	1.0
	WDI_agriculture	HQER-SFA	hqer_induced	True	9	-2871.638944221454	hqer_induced sampler prototype	4.0
	WDI_agriculture	Quantile-SFA	hqer_induced	True	15	-2925.281758429518	hqer_induced sampler prototype	5.0
	WDI_agriculture	HQER-SFA	hqer_induced	True	9	-2896.437851550516	hqer_induced sampler prototype	2.0
	WDI_agriculture	Quantile-SFA	hqer_induced	True	24	-2903.1999871945154	hqer_induced sampler prototype	1.0
	WDI_agriculture	HQER-SFA	hqer_induced	True	8	-2867.639458364576	hqer_induced sampler prototype	2.0
	WDI_agriculture	Quantile-SFA	hqer_induced	True	28	-2939.7436836779834	hqer_induced sampler prototype	3.0
	WDI_agriculture	HQER-SFA	hqer_induced	True	12	-2908.56347125208	hqer_induced sampler prototype	4.0
	WDI_agriculture	Quantile-SFA	hqer_induced	True	24	-2855.663663186309	hqer_induced sampler prototype	5.0
	WDI_agriculture	HQER-SFA	hqer_induced	True	7	-2823.46284861163	hqer_induced sampler prototype	2.0
	WDI_agriculture	Quantile-SFA	hqer_induced	True	26	-2862.106086331195	hqer_induced sampler prototype	1.0
	WDI_agriculture	HQER-SFA	hqer_induced	True	7	-2840.848407395072	hqer_induced sampler prototype	2.0
	WDI_agriculture	Quantile-SFA	hqer_induced	True	20	-2879.578464156905	hqer_induced sampler prototype	1.0
	WDI_agriculture	HQER-SFA	hqer_induced	True	8	-2849.3111924885507	hqer_induced sampler prototype	2.0
	WDI_agriculture	Quantile-SFA	hqer_induced	True	19	-2882.106692379528	hqer_induced sampler prototype	1.0
	WDI_agriculture	HQER-SFA	hqer_induced	True	21	-2847.131821714457	hqer_induced sampler prototype	4.0
	WDI_agriculture	Quantile-SFA	hqer_induced	True	29	-2905.859888348863	hqer_induced sampler prototype	3.0
	WDI_agriculture	HQER-SFA	hqer_induced	True	8	-2878.066403428558	hqer_induced sampler prototype	2.0
	WDI_agriculture	Quantile-SFA	hqer_induced	True	22	-2884.639337968513	hqer_induced sampler prototype	3.0
	WDI_agriculture	HQER-SFA	hqer_induced	True	10	-2848.4385040658613	hqer_induced sampler prototype	4.0
	WDI_agriculture	Quantile-SFA	hqer_induced	True	20	-2888.127883784829	hqer_induced sampler prototype	3.0
	WDI_agriculture	HQER-SFA	hqer_induced	True	6	-2857.3495590806606	hqer_induced sampler prototype	4.0
	WDI_agriculture	Quantile-SFA	hqer_induced	True	32	-2956.7161958752054	hqer_induced sampler prototype	5.0
	WDI_agriculture	HQER-SFA	hqer_induced	True	9	-2928.2209523241936	hqer_induced sampler prototype	2.0
	WDI_agriculture	Quantile-SFA	hqer_induced	True	21	-2869.3691513489766	hqer_induced sampler prototype	1.0
	WDI_agriculture	HQER-SFA	hqer_induced	True	7	-2834.5398523531085	hqer_induced sampler prototype	2.0
	WDI_agriculture	Quantile-SFA	hqer_induced	True	14	-2901.7245630200327	hqer_induced sampler prototype	5.0
	WDI_agriculture	HQER-SFA	hqer_induced	True	9	-2876.426399507924	hqer_induced sampler prototype	2.0
	WDI_agriculture	Quantile-SFA	hqer_induced	True	25	-2901.54307596579	hqer_induced sampler prototype	1.0
	WDI_agriculture	HQER-SFA	hqer_induced	True	11	-2876.943866231014	hqer_induced sampler prototype	4.0
	WDI_agriculture	Quantile-SFA	hqer_induced	True	20	-2835.821698789749	hqer_induced sampler prototype	5.0
	WDI_agriculture	HQER-SFA	hqer_induced	True	13	-2809.0099926394414	hqer_induced sampler prototype	4.0
	WDI_agriculture	Quantile-SFA	hqer_induced	True	17	-2837.905411427834	hqer_induced sampler prototype	3.0
	WDI_agriculture	HQER-SFA	hqer_induced	True	10	-2805.721335623016	hqer_induced sampler prototype	4.0
	WDI_agriculture	Quantile-SFA	hqer_induced	True	22	-2853.239091729412	hqer_induced sampler prototype	1.0
	WDI_agriculture	HQER-SFA	hqer_induced	True	8	-2826.99292191414	hqer_induced sampler prototype	2.0

experiment	dataset	model	scenario	converged	n__iter	loglik	warning__message	best__start__id
	WDI__agriculture	Quantile-SFA	hqer__induced	True	19	-2930.7156817935347	hqer__induced sampler prototype	1.0
	WDI__agriculture	HQER-SFA	hqer__induced	True	9	-2901.505732048762	hqer__induced sampler prototype	2.0
	WDI__agriculture	Quantile-SFA	hqer__induced	True	27	-2899.5289161969245	hqer__induced sampler prototype	1.0
	WDI__agriculture	HQER-SFA	hqer__induced	True	14	-2870.322834696294	hqer__induced sampler prototype	2.0
	WDI__agriculture	Quantile-SFA	hqer__induced	True	24	-2925.058165176501	hqer__induced sampler prototype	3.0
	WDI__agriculture	HQER-SFA	hqer__induced	True	8	-2893.420979417934	hqer__induced sampler prototype	4.0
	WDI__agriculture	Quantile-SFA	hqer__induced	True	15	-2859.4261211040216	hqer__induced sampler prototype	1.0
	WDI__agriculture	HQER-SFA	hqer__induced	True	19	-2838.6223944684007	hqer__induced sampler prototype	4.0
	WDI__agriculture	Quantile-SFA	hqer__induced	True	26	-2885.499828528133	hqer__induced sampler prototype	3.0
	WDI__agriculture	HQER-SFA	hqer__induced	True	8	-2847.86124713462	hqer__induced sampler prototype	4.0
	WDI__agriculture	Quantile-SFA	hqer__induced	True	24	-2884.4340546439653	hqer__induced sampler prototype	5.0
	WDI__agriculture	HQER-SFA	hqer__induced	True	16	-2850.913828671104	hqer__induced sampler prototype	4.0
	WDI__agriculture	Quantile-SFA	hqer__induced	True	19	-2874.248513489808	hqer__induced sampler prototype	3.0
	WDI__agriculture	HQER-SFA	hqer__induced	True	7	-2846.0835902421904	hqer__induced sampler prototype	4.0
	WDI__agriculture	Quantile-SFA	hqer__induced	True	22	-2897.667981310019	hqer__induced sampler prototype	5.0
	WDI__agriculture	HQER-SFA	hqer__induced	True	3	-2868.02268837722	hqer__induced sampler prototype	2.0
	WDI__agriculture	Quantile-SFA	hqer__induced	True	21	-2914.903776306372	hqer__induced sampler prototype	5.0
	WDI__agriculture	HQER-SFA	hqer__induced	True	12	-2881.780650927537	hqer__induced sampler prototype	4.0
	WDI__agriculture	Quantile-SFA	hqer__induced	True	32	-2878.7332093424834	hqer__induced sampler prototype	3.0
	WDI__agriculture	HQER-SFA	hqer__induced	True	10	-2847.394913492584	hqer__induced sampler prototype	4.0
	WDI__agriculture	Quantile-SFA	hqer__induced	True	24	-2878.8878768474224	hqer__induced sampler prototype	1.0
	WDI__agriculture	HQER-SFA	hqer__induced	True	4	-2850.800819340995	hqer__induced sampler prototype	2.0
	WDI__agriculture	Quantile-SFA	hqer__induced	True	19	-2825.6535017471497	hqer__induced sampler prototype	5.0
	WDI__agriculture	HQER-SFA	hqer__induced	True	8	-2800.9074641842235	hqer__induced sampler prototype	2.0
	WDI__agriculture	Quantile-SFA	hqer__induced	True	34	-2865.423650397973	hqer__induced sampler prototype	3.0
	WDI__agriculture	HQER-SFA	hqer__induced	True	4	-2838.5601507182414	hqer__induced sampler prototype	2.0
	WDI__agriculture	Quantile-SFA	hqer__induced	True	25	-2917.283624407919	hqer__induced sampler prototype	3.0
	WDI__agriculture	HQER-SFA	hqer__induced	True	10	-2887.890054652477	hqer__induced sampler prototype	4.0
	WDI__agriculture	Quantile-SFA	hqer__induced	True	23	-2885.6630488813485	hqer__induced sampler prototype	3.0
	WDI__agriculture	HQER-SFA	hqer__induced	True	7	-2852.996029880682	hqer__induced sampler prototype	4.0
	WDI__agriculture	Quantile-SFA	hqer__induced	True	26	-2851.039578088636	hqer__induced sampler prototype	3.0
	WDI__agriculture	HQER-SFA	hqer__induced	True	8	-2821.689672980204	hqer__induced sampler prototype	4.0
	WDI__agriculture	Quantile-SFA	hqer__induced	True	22	-2859.4738645791467	hqer__induced sampler prototype	3.0
	WDI__agriculture	HQER-SFA	hqer__induced	True	11	-2826.543272087012	hqer__induced sampler prototype	4.0
	WDI__agriculture	Quantile-SFA	hqer__induced	True	20	-2931.0201615364253	hqer__induced sampler prototype	5.0
	WDI__agriculture	HQER-SFA	hqer__induced	True	7	-2908.184326281832	hqer__induced sampler prototype	2.0
	WDI__agriculture	Quantile-SFA	hqer__induced	True	19	-2886.3776305834363	hqer__induced sampler prototype	5.0
	WDI__agriculture	HQER-SFA	hqer__induced	True	18	-2854.0357100319698	hqer__induced sampler prototype	4.0
	WDI__agriculture	Quantile-SFA	hqer__induced	True	27	-2833.0280778213632	hqer__induced sampler prototype	1.0
	WDI__agriculture	HQER-SFA	hqer__induced	True	6	-2800.5702505594672	hqer__induced sampler prototype	4.0
	front41Data	Quantile-SFA	hqer__induced	True	47	-4.9946541692469495	hqer__induced sampler prototype	4.0
	front41Data	HQER-SFA	hqer__induced	True	17	-5.03892546965607	hqer__induced sampler prototype	4.0
	front41Data	Quantile-SFA	hqer__induced	True	45	2.4791224327267307	hqer__induced sampler prototype	4.0
	front41Data	HQER-SFA	hqer__induced	True	47	2.502385836957131	hqer__induced sampler prototype	4.0
	front41Data	Quantile-SFA	hqer__induced	True	23	-0.2706664912887655	hqer__induced sampler prototype	4.0
	front41Data	HQER-SFA	hqer__induced	True	29	-0.2580096111624791	hqer__induced sampler prototype	4.0
	front41Data	Quantile-SFA	hqer__induced	True	16	0.4642418173569262	hqer__induced sampler prototype	4.0
	front41Data	HQER-SFA	hqer__induced	True	20	0.475745767443531	hqer__induced sampler prototype	4.0
	front41Data	Quantile-SFA	hqer__induced	True	34	11.456666356104453	hqer__induced sampler prototype	4.0
	front41Data	HQER-SFA	hqer__induced	True	43	11.481718679318629	hqer__induced sampler prototype	2.0
	front41Data	Quantile-SFA	hqer__induced	True	34	13.560186322931305	hqer__induced sampler prototype	4.0
	front41Data	HQER-SFA	hqer__induced	True	27	13.512895444954465	hqer__induced sampler prototype	4.0
	front41Data	Quantile-SFA	hqer__induced	True	17	10.188334050486016	hqer__induced sampler prototype	4.0
	front41Data	HQER-SFA	hqer__induced	True	19	10.189646335666485	hqer__induced sampler prototype	4.0
	front41Data	Quantile-SFA	hqer__induced	True	30	-1.9190396622093249	hqer__induced sampler prototype	2.0
	front41Data	HQER-SFA	hqer__induced	True	34	-1.9534540340712765	hqer__induced sampler prototype	2.0
	front41Data	Quantile-SFA	hqer__induced	True	27	-7.0737908344639	hqer__induced sampler prototype	4.0
	front41Data	HQER-SFA	hqer__induced	True	10	-7.141198228550425	hqer__induced sampler prototype	2.0
	front41Data	Quantile-SFA	hqer__induced	True	30	3.4958591658621523	hqer__induced sampler prototype	2.0
	front41Data	HQER-SFA	hqer__induced	True	32	3.592232661754436	hqer__induced sampler prototype	4.0
	front41Data	Quantile-SFA	hqer__induced	True	6	1.0971973115246525	hqer__induced sampler prototype	2.0
	front41Data	HQER-SFA	hqer__induced	True	35	1.466600908713313	hqer__induced sampler prototype	4.0
	front41Data	Quantile-SFA	hqer__induced	True	15	2.6868947167194803	hqer__induced sampler prototype	4.0
	front41Data	HQER-SFA	hqer__induced	True	20	2.6871077554215423	hqer__induced sampler prototype	4.0
	front41Data	Quantile-SFA	hqer__induced	True	28	-1.7647479300755646	hqer__induced sampler prototype	4.0
	front41Data	HQER-SFA	hqer__induced	True	45	-1.7465692019431156	hqer__induced sampler prototype	4.0
	front41Data	Quantile-SFA	hqer__induced	True	25	11.3571770738043	hqer__induced sampler prototype	3.0
	front41Data	HQER-SFA	hqer__induced	True	27	11.011911358979525	hqer__induced sampler prototype	4.0
	front41Data	Quantile-SFA	hqer__induced	True	23	0.4260270468978457	hqer__induced sampler prototype	2.0
	front41Data	HQER-SFA	hqer__induced	True	45	0.4814495740131097	hqer__induced sampler prototype	2.0
	front41Data	Quantile-SFA	hqer__induced	True	13	9.85835196555608	hqer__induced sampler prototype	4.0
	front41Data	HQER-SFA	hqer__induced	True	16	9.896838067303465	hqer__induced sampler prototype	4.0
	front41Data	Quantile-SFA	hqer__induced	True	47	-6.261154368696439	hqer__induced sampler prototype	4.0
	front41Data	HQER-SFA	hqer__induced	True	43	-6.267531493910474	hqer__induced sampler prototype	2.0
	front41Data	Quantile-SFA	hqer__induced	True	30	0.599513996798971	hqer__induced sampler prototype	2.0
	front41Data	HQER-SFA	hqer__induced	True	24	0.5926332671366779	hqer__induced sampler prototype	4.0
	front41Data	Quantile-SFA	hqer__induced	True	33	6.244672243780535	hqer__induced sampler prototype	4.0
	front41Data	HQER-SFA	hqer__induced	True	41	6.2741512400233095	hqer__induced sampler prototype	4.0
	front41Data	Quantile-SFA	hqer__induced	True	15	16.108718741628405	hqer__induced sampler prototype	2.0
	front41Data	HQER-SFA	hqer__induced	True	52	18.154348784410114	hqer__induced sampler prototype	4.0
	front41Data	Quantile-SFA	hqer__induced	True	39	9.39103449351324	hqer__induced sampler prototype	2.0
	front41Data	HQER-SFA	hqer__induced	True	17	8.775641002793272	hqer__induced sampler prototype	4.0
	front41Data	Quantile-SFA	hqer__induced	True	47	2.407328006856546	hqer__induced sampler prototype	4.0
	front41Data	HQER-SFA	hqer__induced	True	34	2.41767669937369	hqer__induced sampler prototype	4.0
	front41Data	Quantile-SFA	hqer__induced	True	26	8.131942880286362	hqer__induced sampler prototype	4.0
	front41Data	HQER-SFA	hqer__induced	True	31	8.178701301506758	hqer__induced sampler prototype	4.0
	front41Data	Quantile-SFA	hqer__induced	True	35	11.754844990870753	hqer__induced sampler prototype	4.0
	front41Data	HQER-SFA	hqer__induced	True	17	11.735290466286369	hqer__induced sampler prototype	4.0
	front41Data	Quantile-SFA	hqer__induced	True	26	3.925999876121697	hqer__induced sampler prototype	2.0
	front41Data	HQER-SFA	hqer__induced	True	31	3.9422380782947295	hqer__induced sampler prototype	2.0
	front41Data	Quantile-SFA	hqer__induced	True	27	-2.8703227280468053	hqer__induced sampler prototype	4.0
	front41Data	HQER-SFA	hqer__induced	True	32	-2.864457725289404	hqer__induced sampler prototype	4.0
	front41Data	Quantile-SFA	hqer__induced	True	27	0.8542797180598632	hqer__induced sampler prototype	2.0
	front41Data	HQER-SFA	hqer__induced	True	24	0.7538905170313012	hqer__induced sampler prototype	2.0
	front41Data	Quantile-SFA	hqer__induced	True	42	-6.557672703697791	hqer__induced sampler prototype	4.0
	front41Data	HQER-SFA	hqer__induced	True	44	-6.546312135634077	hqer__induced sampler prototype	4.0
	front41Data	Quantile-SFA	hqer__induced	True	38	4.375077086255859	hqer__induced sampler prototype	4.0
	front41Data	HQER-SFA	hqer__induced	True	19	4.317979947513226	hqer__induced sampler prototype	4.0
	front41Data	Quantile-SFA	hqer__induced	True	20	8.37564627766897	hqer__induced sampler prototype	2.0
	front41Data	HQER-SFA	hqer__induced	True	14	8.378583590319511	hqer__induced sampler prototype	2.0
	front41Data	Quantile-SFA	hqer__induced	True	23	5.343560161604621	hqer__induced sampler prototype	4.0
	front41Data	HQER-SFA	hqer__induced	True	33	5.351592119950007	hqer__induced sampler prototype	4.0
	front41Data	Quantile-SFA	hqer__induced	True	33	-1.1672804651521511	hqer__induced sampler prototype	2.0

experiment	dataset	model	scenario	converged	n_iter	loglik	warning_message	best_start_id
	front41Data	HQER-SFA	hqer_induced	True	43	-1.1522053925630775	hqer_induced sampler prototype	4.0
	front41Data	Quantile-SFA	hqer_induced	True	17	12.710325716138332	hqer_induced sampler prototype	2.0
	front41Data	HQER-SFA	hqer_induced	True	13	12.71965489931279	hqer_induced sampler prototype	2.0
	front41Data	Quantile-SFA	hqer_induced	True	35	3.3473552664686883	hqer_induced sampler prototype	2.0
	front41Data	HQER-SFA	hqer_induced	True	36	3.340358050364866	hqer_induced sampler prototype	2.0
	front41Data	Quantile-SFA	hqer_induced	True	24	0.4505286684772712	hqer_induced sampler prototype	4.0
	front41Data	HQER-SFA	hqer_induced	True	30	0.5718318297383516	hqer_induced sampler prototype	4.0
	front41Data	Quantile-SFA	hqer_induced	True	15	0.2902348435396232	hqer_induced sampler prototype	4.0
	front41Data	HQER-SFA	hqer_induced	True	31	0.3667576282539665	hqer_induced sampler prototype	4.0
	front41Data	Quantile-SFA	hqer_induced	True	47	5.7870603668558935	hqer_induced sampler prototype	4.0
	front41Data	HQER-SFA	hqer_induced	True	17	5.800011736315641	hqer_induced sampler prototype	2.0
	front41Data	Quantile-SFA	hqer_induced	True	34	-0.5824798280635197	hqer_induced sampler prototype	2.0
	front41Data	HQER-SFA	hqer_induced	True	36	-0.5533880328312988	hqer_induced sampler prototype	4.0
	front41Data	Quantile-SFA	hqer_induced	True	37	9.783309474143817	hqer_induced sampler prototype	4.0
	front41Data	HQER-SFA	hqer_induced	True	29	9.773621345116863	hqer_induced sampler prototype	4.0
	front41Data	Quantile-SFA	hqer_induced	True	50	-8.744657382428063	hqer_induced sampler prototype	2.0
	front41Data	HQER-SFA	hqer_induced	True	22	-8.839302800486593	hqer_induced sampler prototype	4.0
	front41Data	Quantile-SFA	hqer_induced	True	35	4.480739029035638	hqer_induced sampler prototype	2.0
	front41Data	HQER-SFA	hqer_induced	True	55	4.501720085191634	hqer_induced sampler prototype	2.0
	front41Data	Quantile-SFA	hqer_induced	True	53	1.5905374621267248	hqer_induced sampler prototype	2.0
	front41Data	HQER-SFA	hqer_induced	True	24	1.5940276322698643	hqer_induced sampler prototype	4.0
	front41Data	Quantile-SFA	hqer_induced	True	39	16.186184136839383	hqer_induced sampler prototype	2.0
	front41Data	HQER-SFA	hqer_induced	True	24	16.196827388364433	hqer_induced sampler prototype	4.0
	front41Data	Quantile-SFA	hqer_induced	True	18	3.3341184054592183	hqer_induced sampler prototype	2.0
	front41Data	HQER-SFA	hqer_induced	True	18	3.0807449375741576	hqer_induced sampler prototype	4.0
	front41Data	Quantile-SFA	hqer_induced	True	13	6.747674153683047	hqer_induced sampler prototype	2.0
	front41Data	HQER-SFA	hqer_induced	True	16	6.758182541727201	hqer_induced sampler prototype	2.0
	front41Data	Quantile-SFA	hqer_induced	True	32	6.263460743010446	hqer_induced sampler prototype	4.0
	front41Data	HQER-SFA	hqer_induced	True	46	6.177123001305926	hqer_induced sampler prototype	4.0
	front41Data	Quantile-SFA	hqer_induced	True	42	-3.9369204744091615	hqer_induced sampler prototype	4.0
	front41Data	HQER-SFA	hqer_induced	True	23	-4.001701687030943	hqer_induced sampler prototype	4.0
	front41Data	Quantile-SFA	hqer_induced	True	32	6.046211293180806	hqer_induced sampler prototype	4.0
	front41Data	HQER-SFA	hqer_induced	True	47	6.090687798189213	hqer_induced sampler prototype	4.0
	front41Data	Quantile-SFA	hqer_induced	True	26	-0.0612736231735678	hqer_induced sampler prototype	4.0
	front41Data	HQER-SFA	hqer_induced	True	42	-0.053438504028457	hqer_induced sampler prototype	2.0
	front41Data	Quantile-SFA	hqer_induced	True	36	4.765199113695056	hqer_induced sampler prototype	2.0
	front41Data	HQER-SFA	hqer_induced	True	59	4.829318020392884	hqer_induced sampler prototype	4.0
	front41Data	Quantile-SFA	hqer_induced	True	8	-0.0176880368577321	hqer_induced sampler prototype	2.0
	front41Data	HQER-SFA	hqer_induced	True	27	0.1458720545631159	hqer_induced sampler prototype	2.0
	front41Data	Quantile-SFA	hqer_induced	True	25	-2.567857406770179	hqer_induced sampler prototype	2.0
	front41Data	HQER-SFA	hqer_induced	True	39	-2.5406576743771865	hqer_induced sampler prototype	2.0
	front41Data	Quantile-SFA	hqer_induced	True	21	7.101225481939336	hqer_induced sampler prototype	2.0
	front41Data	HQER-SFA	hqer_induced	True	18	7.0910139862168	hqer_induced sampler prototype	2.0
	front41Data	Quantile-SFA	hqer_induced	True	65	-1.6876507691438791	hqer_induced sampler prototype	4.0
	front41Data	HQER-SFA	hqer_induced	True	46	-1.6196604209771437	hqer_induced sampler prototype	4.0
	front41Data	Quantile-SFA	hqer_induced	True	18	3.2933090138728027	hqer_induced sampler prototype	4.0
	front41Data	HQER-SFA	hqer_induced	True	23	3.3157843463691496	hqer_induced sampler prototype	4.0
	front41Data	Quantile-SFA	hqer_induced	True	52	-2.1479182998960087	hqer_induced sampler prototype	4.0
	front41Data	HQER-SFA	hqer_induced	True	26	-2.145465973712742	hqer_induced sampler prototype	2.0
	front41Data	Quantile-SFA	hqer_induced	True	31	-3.990057760082733	hqer_induced sampler prototype	2.0
	front41Data	HQER-SFA	hqer_induced	True	35	-4.197457536080146	hqer_induced sampler prototype	4.0
	front41Data	Quantile-SFA	hqer_induced	True	18	3.910905335684516	hqer_induced sampler prototype	4.0
	front41Data	HQER-SFA	hqer_induced	True	24	3.9364728654238657	hqer_induced sampler prototype	4.0
	front41Data	Quantile-SFA	hqer_induced	True	43	-0.4132607512045833	hqer_induced sampler prototype	4.0
	front41Data	HQER-SFA	hqer_induced	True	12	-0.6687761211722464	hqer_induced sampler prototype	2.0
	front41Data	Quantile-SFA	hqer_induced	True	23	1.037996686604076	hqer_induced sampler prototype	2.0
	front41Data	HQER-SFA	hqer_induced	True	53	1.188807419025133	hqer_induced sampler prototype	2.0
	front41Data	Quantile-SFA	hqer_induced	True	32	10.538251262087185	hqer_induced sampler prototype	2.0
	front41Data	HQER-SFA	hqer_induced	True	47	10.54449456814342	hqer_induced sampler prototype	4.0
	front41Data	Quantile-SFA	hqer_induced	True	42	2.948686323062669	hqer_induced sampler prototype	2.0
	front41Data	HQER-SFA	hqer_induced	True	29	2.958094378832253	hqer_induced sampler prototype	2.0
	front41Data	Quantile-SFA	hqer_induced	True	17	-14.914925601450191	hqer_induced sampler prototype	2.0
	front41Data	HQER-SFA	hqer_induced	True	11	-14.931909371585434	hqer_induced sampler prototype	2.0
	front41Data	Quantile-SFA	hqer_induced	True	16	0.284931138228247	hqer_induced sampler prototype	4.0
	front41Data	HQER-SFA	hqer_induced	True	54	0.3036620394654894	hqer_induced sampler prototype	2.0
	front41Data	Quantile-SFA	hqer_induced	True	19	8.70816041519994	hqer_induced sampler prototype	2.0
	front41Data	HQER-SFA	hqer_induced	True	16	8.756828381562828	hqer_induced sampler prototype	2.0
	front41Data	Quantile-SFA	hqer_induced	True	49	9.56509042206555	hqer_induced sampler prototype	1.0
	front41Data	HQER-SFA	hqer_induced	True	45	9.408778785721124	hqer_induced sampler prototype	2.0
	front41Data	Quantile-SFA	hqer_induced	True	23	-4.37090897060167	hqer_induced sampler prototype	2.0
	front41Data	HQER-SFA	hqer_induced	True	42	-3.810740029380786	hqer_induced sampler prototype	4.0
	front41Data	Quantile-SFA	hqer_induced	True	39	-10.705242426288834	hqer_induced sampler prototype	2.0
	front41Data	HQER-SFA	hqer_induced	True	35	-10.71056598087683	hqer_induced sampler prototype	4.0
	front41Data	Quantile-SFA	hqer_induced	True	21	6.485761454291558	hqer_induced sampler prototype	2.0
	front41Data	HQER-SFA	hqer_induced	True	24	6.469949019566753	hqer_induced sampler prototype	4.0
	front41Data	Quantile-SFA	hqer_induced	True	18	11.672571823841157	hqer_induced sampler prototype	2.0
	front41Data	HQER-SFA	hqer_induced	True	48	11.76431492704001	hqer_induced sampler prototype	4.0
	front41Data	Quantile-SFA	hqer_induced	True	29	5.109645588025382	hqer_induced sampler prototype	2.0
	front41Data	HQER-SFA	hqer_induced	True	31	5.103076395765579	hqer_induced sampler prototype	2.0
	front41Data	Quantile-SFA	hqer_induced	True	13	-2.607008880670504	hqer_induced sampler prototype	4.0
	front41Data	HQER-SFA	hqer_induced	True	24	-2.4037984453067014	hqer_induced sampler prototype	4.0
	front41Data	Quantile-SFA	hqer_induced	True	47	8.47794795292176	hqer_induced sampler prototype	4.0
	front41Data	HQER-SFA	hqer_induced	True	31	8.475027210863336	hqer_induced sampler prototype	4.0
	front41Data	Quantile-SFA	hqer_induced	True	22	9.143651316810818	hqer_induced sampler prototype	4.0
	front41Data	HQER-SFA	hqer_induced	True	36	9.206185289271296	hqer_induced sampler prototype	4.0
	front41Data	Quantile-SFA	hqer_induced	True	7	-6.538190452842997	hqer_induced sampler prototype	2.0
	front41Data	HQER-SFA	hqer_induced	True	6	-6.518067858860933	hqer_induced sampler prototype	2.0
	front41Data	Quantile-SFA	hqer_induced	True	39	6.887264922509908	hqer_induced sampler prototype	2.0
	front41Data	HQER-SFA	hqer_induced	True	37	6.8916739939409	hqer_induced sampler prototype	2.0
	front41Data	Quantile-SFA	hqer_induced	True	63	4.303044653622687	hqer_induced sampler prototype	4.0
	front41Data	HQER-SFA	hqer_induced	True	16	4.036238329899857	hqer_induced sampler prototype	4.0
	front41Data	Quantile-SFA	hqer_induced	True	7	-2.494940867270128	hqer_induced sampler prototype	2.0
	front41Data	HQER-SFA	hqer_induced	True	23	-2.347698379526037	hqer_induced sampler prototype	2.0
	front41Data	Quantile-SFA	hqer_induced	True	46	4.701312834237637	hqer_induced sampler prototype	4.0
	front41Data	HQER-SFA	hqer_induced	True	26	4.699681511849043	hqer_induced sampler prototype	4.0
	front41Data	Quantile-SFA	hqer_induced	True	34	3.215269061576157	hqer_induced sampler prototype	2.0
	front41Data	HQER-SFA	hqer_induced	True	63	3.2248762126193253	hqer_induced sampler prototype	4.0
	front41Data	Quantile-SFA	hqer_induced	True	35	-0.1582369303586697	hqer_induced sampler prototype	4.0
	front41Data	HQER-SFA	hqer_induced	True	35	-0.1638198377290528	hqer_induced sampler prototype	4.0
	front41Data	Quantile-SFA	hqer_induced	True	28	-1.0121058190674923	hqer_induced sampler prototype	4.0
	front41Data	HQER-SFA	hqer_induced	True	55	-0.9060945456367704	hqer_induced sampler prototype	2.0
	front41Data	Quantile-SFA	hqer_induced	True	44	2.5185335583618444	hqer_induced sampler prototype	4.0
	front41Data	HQER-SFA	hqer_induced	True	32	2.521099958439568	hqer_induced sampler prototype	4.0

experiment	dataset	model	scenario	converged	n__iter	loglik	warning__message	best__start__id
	front41Data	Quantile-SFA	hqer__induced	True	35	-12.13680386790824	hqer__induced sampler prototype	2.0
	front41Data	HQER-SFA	hqer__induced	True	38	-12.135655509940642	hqer__induced sampler prototype	2.0
	front41Data	Quantile-SFA	hqer__induced	True	50	7.1371419923332375	hqer__induced sampler prototype	4.0
	front41Data	HQER-SFA	hqer__induced	True	36	7.148881994540562	hqer__induced sampler prototype	4.0
	front41Data	Quantile-SFA	hqer__induced	True	33	-1.6755029311190115	hqer__induced sampler prototype	2.0
	front41Data	HQER-SFA	hqer__induced	True	57	-1.5979126287230985	hqer__induced sampler prototype	4.0
	front41Data	Quantile-SFA	hqer__induced	True	34	-5.534440524359311	hqer__induced sampler prototype	4.0
	front41Data	HQER-SFA	hqer__induced	True	20	-5.5106774966966	hqer__induced sampler prototype	4.0
	front41Data	Quantile-SFA	hqer__induced	True	42	4.205295670064614	hqer__induced sampler prototype	2.0
	front41Data	HQER-SFA	hqer__induced	True	39	4.230612170925599	hqer__induced sampler prototype	4.0
	front41Data	Quantile-SFA	hqer__induced	True	16	-6.395118157031472	hqer__induced sampler prototype	2.0
	front41Data	HQER-SFA	hqer__induced	True	9	-6.517927080258358	hqer__induced sampler prototype	2.0
	front41Data	Quantile-SFA	hqer__induced	True	25	0.3939459703624632	hqer__induced sampler prototype	2.0
	front41Data	HQER-SFA	hqer__induced	True	15	0.4074615682678427	hqer__induced sampler prototype	2.0
	front41Data	Quantile-SFA	hqer__induced	True	10	-8.175176471747186	hqer__induced sampler prototype	2.0
	front41Data	HQER-SFA	hqer__induced	True	10	-8.170932002834778	hqer__induced sampler prototype	2.0
	front41Data	Quantile-SFA	hqer__induced	True	17	11.615644481816858	hqer__induced sampler prototype	2.0
	front41Data	HQER-SFA	hqer__induced	True	28	11.632953954539364	hqer__induced sampler prototype	2.0
	front41Data	Quantile-SFA	hqer__induced	True	56	6.587960016426439	hqer__induced sampler prototype	4.0
	front41Data	HQER-SFA	hqer__induced	True	19	5.975353276285137	hqer__induced sampler prototype	4.0
	front41Data	Quantile-SFA	hqer__induced	True	20	4.162009552066857	hqer__induced sampler prototype	4.0
	front41Data	HQER-SFA	hqer__induced	True	37	4.531142495257027	hqer__induced sampler prototype	4.0
	front41Data	Quantile-SFA	hqer__induced	True	48	-2.302105661263857	hqer__induced sampler prototype	2.0
	front41Data	HQER-SFA	hqer__induced	True	34	-1.7358499809890218	hqer__induced sampler prototype	4.0
	front41Data	Quantile-SFA	hqer__induced	True	38	-10.227121544908654	hqer__induced sampler prototype	4.0
	front41Data	HQER-SFA	hqer__induced	True	39	-10.215790931684136	hqer__induced sampler prototype	2.0
	front41Data	Quantile-SFA	hqer__induced	True	8	-6.285465706101211	hqer__induced sampler prototype	2.0
	front41Data	HQER-SFA	hqer__induced	True	11	-6.260298986480807	hqer__induced sampler prototype	2.0
	front41Data	Quantile-SFA	hqer__induced	True	22	8.575283473179361	hqer__induced sampler prototype	2.0
	front41Data	HQER-SFA	hqer__induced	True	40	8.622770133020504	hqer__induced sampler prototype	4.0
	front41Data	Quantile-SFA	hqer__induced	True	52	7.79048556975385	hqer__induced sampler prototype	1.0
	front41Data	HQER-SFA	hqer__induced	True	37	7.77862162267485	hqer__induced sampler prototype	4.0
	front41Data	Quantile-SFA	hqer__induced	True	48	2.421487021699587	hqer__induced sampler prototype	3.0
	front41Data	HQER-SFA	hqer__induced	True	38	2.445000942654935	hqer__induced sampler prototype	2.0
	front41Data	Quantile-SFA	hqer__induced	True	25	1.3189428027807386	hqer__induced sampler prototype	4.0
	front41Data	HQER-SFA	hqer__induced	True	29	1.4259093607701256	hqer__induced sampler prototype	4.0
	front41Data	Quantile-SFA	hqer__induced	True	53	0.9639163763708803	hqer__induced sampler prototype	4.0
	front41Data	HQER-SFA	hqer__induced	True	33	1.0963375274320306	hqer__induced sampler prototype	4.0
	front41Data	Quantile-SFA	hqer__induced	True	38	52.70581237219591	hqer__induced sampler prototype	4.0
	front41Data	HQER-SFA	hqer__induced	True	36	52.84429652862772	hqer__induced sampler prototype	4.0
	front41Data	Quantile-SFA	hqer__induced	True	25	8.384173571889288	hqer__induced sampler prototype	4.0
	front41Data	HQER-SFA	hqer__induced	True	26	8.460603631656276	hqer__induced sampler prototype	4.0
	front41Data	Quantile-SFA	hqer__induced	True	29	-9.51643872384118	hqer__induced sampler prototype	2.0
	front41Data	HQER-SFA	hqer__induced	True	30	-9.375974526552742	hqer__induced sampler prototype	2.0
	front41Data	Quantile-SFA	hqer__induced	True	13	-50.25399593929456	hqer__induced sampler prototype	4.0
	front41Data	HQER-SFA	hqer__induced	True	27	-50.14180717186855	hqer__induced sampler prototype	2.0
	front41Data	Quantile-SFA	hqer__induced	True	30	24.00251155644145	hqer__induced sampler prototype	4.0
	front41Data	HQER-SFA	hqer__induced	True	29	24.086428648976074	hqer__induced sampler prototype	2.0
	front41Data	Quantile-SFA	hqer__induced	True	33	-2.261945427693287	hqer__induced sampler prototype	4.0
	front41Data	HQER-SFA	hqer__induced	True	28	-2.1201474096711355	hqer__induced sampler prototype	4.0
	front41Data	Quantile-SFA	hqer__induced	True	24	-12.675469609499142	hqer__induced sampler prototype	2.0
	front41Data	HQER-SFA	hqer__induced	True	27	-12.601600630604164	hqer__induced sampler prototype	2.0
	front41Data	Quantile-SFA	hqer__induced	True	28	-23.32621527162264	hqer__induced sampler prototype	4.0
	front41Data	HQER-SFA	hqer__induced	True	24	-23.1997606044089	hqer__induced sampler prototype	4.0
	front41Data	Quantile-SFA	hqer__induced	True	49	5.652149471687017	hqer__induced sampler prototype	4.0
	front41Data	HQER-SFA	hqer__induced	True	30	5.756256901352262	hqer__induced sampler prototype	4.0
	front41Data	Quantile-SFA	hqer__induced	True	22	37.03310990464499	hqer__induced sampler prototype	4.0
	front41Data	HQER-SFA	hqer__induced	True	34	37.12388267004167	hqer__induced sampler prototype	4.0
	front41Data	Quantile-SFA	hqer__induced	True	47	-17.163054671322648	hqer__induced sampler prototype	2.0
	front41Data	HQER-SFA	hqer__induced	True	91	-17.136698841687668	hqer__induced sampler prototype	2.0
	front41Data	Quantile-SFA	hqer__induced	True	17	-31.72860866773096	hqer__induced sampler prototype	4.0
	front41Data	HQER-SFA	hqer__induced	True	60	-31.610644328774768	hqer__induced sampler prototype	4.0
	front41Data	Quantile-SFA	hqer__induced	True	22	-0.0344276528818792	hqer__induced sampler prototype	2.0
	front41Data	HQER-SFA	hqer__induced	True	24	0.0690687786175772	hqer__induced sampler prototype	2.0
	front41Data	Quantile-SFA	hqer__induced	True	21	22.25474645048329	hqer__induced sampler prototype	4.0
	front41Data	HQER-SFA	hqer__induced	True	37	22.324534777578165	hqer__induced sampler prototype	4.0
	front41Data	Quantile-SFA	hqer__induced	True	26	-8.626219847422167	hqer__induced sampler prototype	2.0
	front41Data	HQER-SFA	hqer__induced	True	17	-8.565585006830096	hqer__induced sampler prototype	4.0
	front41Data	Quantile-SFA	hqer__induced	True	36	25.85501160650177	hqer__induced sampler prototype	2.0
	front41Data	HQER-SFA	hqer__induced	True	22	25.96798071165761	hqer__induced sampler prototype	4.0
	front41Data	Quantile-SFA	hqer__induced	True	22	6.243120301828845	hqer__induced sampler prototype	4.0
	front41Data	HQER-SFA	hqer__induced	True	31	6.356502280406302	hqer__induced sampler prototype	4.0
	front41Data	Quantile-SFA	hqer__induced	True	33	14.563724023926454	hqer__induced sampler prototype	4.0
	front41Data	HQER-SFA	hqer__induced	True	32	14.654490022615493	hqer__induced sampler prototype	4.0
	front41Data	Quantile-SFA	hqer__induced	True	26	-6.522968225134283	hqer__induced sampler prototype	4.0
	front41Data	HQER-SFA	hqer__induced	True	33	-6.365389351802572	hqer__induced sampler prototype	2.0
	front41Data	Quantile-SFA	hqer__induced	True	28	-1.753870640220371	hqer__induced sampler prototype	2.0
	front41Data	HQER-SFA	hqer__induced	True	25	-1.635447610629107	hqer__induced sampler prototype	2.0
	front41Data	Quantile-SFA	hqer__induced	True	30	-26.20915135564165	hqer__induced sampler prototype	2.0
	front41Data	HQER-SFA	hqer__induced	True	34	-26.12916520638678	hqer__induced sampler prototype	2.0
	front41Data	Quantile-SFA	hqer__induced	True	31	31.159415850940466	hqer__induced sampler prototype	4.0
	front41Data	HQER-SFA	hqer__induced	True	40	31.131032775803607	hqer__induced sampler prototype	2.0
	front41Data	Quantile-SFA	hqer__induced	True	31	35.38599034800382	hqer__induced sampler prototype	4.0
	front41Data	HQER-SFA	hqer__induced	True	48	35.4830889728593	hqer__induced sampler prototype	5.0
	front41Data	Quantile-SFA	hqer__induced	True	30	15.160554999244498	hqer__induced sampler prototype	4.0
	front41Data	HQER-SFA	hqer__induced	True	25	14.964008246627635	hqer__induced sampler prototype	2.0
	front41Data	Quantile-SFA	hqer__induced	True	51	-0.7167136805170706	hqer__induced sampler prototype	2.0
	front41Data	HQER-SFA	hqer__induced	True	72	-0.6974823404160189	hqer__induced sampler prototype	2.0
	front41Data	Quantile-SFA	hqer__induced	True	29	-32.333337961135214	hqer__induced sampler prototype	4.0
	front41Data	HQER-SFA	hqer__induced	True	31	-32.23683122294268	hqer__induced sampler prototype	2.0
	front41Data	Quantile-SFA	hqer__induced	True	36	18.16716895203107	hqer__induced sampler prototype	4.0
	front41Data	HQER-SFA	hqer__induced	True	40	18.25968894059917	hqer__induced sampler prototype	5.0
	front41Data	Quantile-SFA	hqer__induced	True	28	17.28541403935938	hqer__induced sampler prototype	2.0
	front41Data	HQER-SFA	hqer__induced	True	27	17.36493969971058	hqer__induced sampler prototype	2.0
	front41Data	Quantile-SFA	hqer__induced	True	49	1.6419380724571615	hqer__induced sampler prototype	4.0
	front41Data	HQER-SFA	hqer__induced	True	36	1.7536144273833203	hqer__induced sampler prototype	4.0
	front41Data	Quantile-SFA	hqer__induced	True	22	43.87679239425903	hqer__induced sampler prototype	2.0
	front41Data	HQER-SFA	hqer__induced	True	30	43.99810093615464	hqer__induced sampler prototype	4.0
	front41Data	Quantile-SFA	hqer__induced	True	23	29.641438709892	hqer__induced sampler prototype	2.0
	front41Data	HQER-SFA	hqer__induced	True	25	29.721215151856786	hqer__induced sampler prototype	4.0
	front41Data	Quantile-SFA	hqer__induced	True	21	-22.24353965151189	hqer__induced sampler prototype	2.0
	front41Data	HQER-SFA	hqer__induced	True	19	-22.136966106928668	hqer__induced sampler prototype	4.0
	front41Data	Quantile-SFA	hqer__induced	True	47	-30.87606993358781	hqer__induced sampler prototype	4.0

experiment	dataset	model	scenario	converged	n__iter	loglik	warning__message	best__start__id
	front41Data	HQER-SFA	hqer__induced	True	54	-30.75968701691039	hqer__induced sampler prototype	4.0
	front41Data	Quantile-SFA	hqer__induced	True	25	-1.363475911540066	hqer__induced sampler prototype	2.0
	front41Data	HQER-SFA	hqer__induced	True	39	-1.257695325193389	hqer__induced sampler prototype	2.0
	front41Data	Quantile-SFA	hqer__induced	True	47	-1.6123083591807983	hqer__induced sampler prototype	2.0
	front41Data	HQER-SFA	hqer__induced	True	24	-1.535059352873703	hqer__induced sampler prototype	4.0
	front41Data	Quantile-SFA	hqer__induced	True	26	-5.352327297952877	hqer__induced sampler prototype	2.0
	front41Data	HQER-SFA	hqer__induced	True	30	-5.288706772728685	hqer__induced sampler prototype	2.0
	front41Data	Quantile-SFA	hqer__induced	True	21	-9.966305200275215	hqer__induced sampler prototype	2.0
	front41Data	HQER-SFA	hqer__induced	True	34	-9.862427866075972	hqer__induced sampler prototype	2.0
	front41Data	Quantile-SFA	hqer__induced	True	24	33.07180864205019	hqer__induced sampler prototype	2.0
	front41Data	HQER-SFA	hqer__induced	True	15	33.12366629444887	hqer__induced sampler prototype	2.0
	front41Data	Quantile-SFA	hqer__induced	True	37	-1.402044201818851	hqer__induced sampler prototype	4.0
	front41Data	HQER-SFA	hqer__induced	True	33	-1.289759994493246	hqer__induced sampler prototype	4.0
	front41Data	Quantile-SFA	hqer__induced	True	39	-0.7459964362546359	hqer__induced sampler prototype	4.0
	front41Data	HQER-SFA	hqer__induced	True	31	-0.6259151701100709	hqer__induced sampler prototype	4.0
	front41Data	Quantile-SFA	hqer__induced	True	21	17.537928681926306	hqer__induced sampler prototype	2.0
	front41Data	HQER-SFA	hqer__induced	True	26	17.607981220958017	hqer__induced sampler prototype	2.0
	front41Data	Quantile-SFA	hqer__induced	True	33	-12.981359163900237	hqer__induced sampler prototype	4.0
	front41Data	HQER-SFA	hqer__induced	True	24	-12.844009237226516	hqer__induced sampler prototype	4.0
	front41Data	Quantile-SFA	hqer__induced	True	39	-2.871714446189418	hqer__induced sampler prototype	4.0
	front41Data	HQER-SFA	hqer__induced	True	33	-2.7825040373432	hqer__induced sampler prototype	4.0
	front41Data	Quantile-SFA	hqer__induced	True	23	-9.45239816096352	hqer__induced sampler prototype	2.0
	front41Data	HQER-SFA	hqer__induced	True	37	-9.346311666155035	hqer__induced sampler prototype	4.0
	front41Data	Quantile-SFA	hqer__induced	True	43	26.26031905031505	hqer__induced sampler prototype	4.0
	front41Data	HQER-SFA	hqer__induced	True	25	26.34652136819157	hqer__induced sampler prototype	2.0
	front41Data	Quantile-SFA	hqer__induced	True	11	5.464822133145196	hqer__induced sampler prototype	2.0
	front41Data	HQER-SFA	hqer__induced	True	22	5.579326294796236	hqer__induced sampler prototype	4.0
	front41Data	Quantile-SFA	hqer__induced	True	24	-16.173546026153474	hqer__induced sampler prototype	2.0
	front41Data	HQER-SFA	hqer__induced	True	28	-16.050700867385252	hqer__induced sampler prototype	2.0
	front41Data	Quantile-SFA	hqer__induced	True	33	-10.949486887119956	hqer__induced sampler prototype	2.0
	front41Data	HQER-SFA	hqer__induced	True	38	-10.807663034669115	hqer__induced sampler prototype	2.0
	front41Data	Quantile-SFA	hqer__induced	True	27	-37.555055957376126	hqer__induced sampler prototype	4.0
	front41Data	HQER-SFA	hqer__induced	True	31	-37.44428853036696	hqer__induced sampler prototype	4.0
	front41Data	Quantile-SFA	hqer__induced	True	33	-2.313178887709153	hqer__induced sampler prototype	4.0
	front41Data	HQER-SFA	hqer__induced	True	21	-2.284690421427974	hqer__induced sampler prototype	4.0
	front41Data	Quantile-SFA	hqer__induced	True	26	14.08178981579112	hqer__induced sampler prototype	2.0
	front41Data	HQER-SFA	hqer__induced	True	28	14.165013413592815	hqer__induced sampler prototype	2.0
	front41Data	Quantile-SFA	hqer__induced	True	31	-8.722335264303284	hqer__induced sampler prototype	4.0
	front41Data	HQER-SFA	hqer__induced	True	34	-8.61010224369249	hqer__induced sampler prototype	2.0
	front41Data	Quantile-SFA	hqer__induced	True	24	1.5236323807817935	hqer__induced sampler prototype	4.0
	front41Data	HQER-SFA	hqer__induced	True	29	1.6170598098551032	hqer__induced sampler prototype	2.0
	front41Data	Quantile-SFA	hqer__induced	True	22	6.71856331140931	hqer__induced sampler prototype	4.0
	front41Data	HQER-SFA	hqer__induced	True	19	6.79335514750583	hqer__induced sampler prototype	4.0
	front41Data	Quantile-SFA	hqer__induced	True	35	24.35921752959068	hqer__induced sampler prototype	4.0
	front41Data	HQER-SFA	hqer__induced	True	32	24.446889305442948	hqer__induced sampler prototype	4.0
	front41Data	Quantile-SFA	hqer__induced	True	32	-0.4738851727992248	hqer__induced sampler prototype	2.0
	front41Data	HQER-SFA	hqer__induced	True	30	-0.2679470134893744	hqer__induced sampler prototype	2.0
	front41Data	Quantile-SFA	hqer__induced	True	47	-2.505685263226754	hqer__induced sampler prototype	2.0
	front41Data	HQER-SFA	hqer__induced	True	35	-2.412255671961862	hqer__induced sampler prototype	2.0
	front41Data	Quantile-SFA	hqer__induced	True	33	7.323505048562416	hqer__induced sampler prototype	4.0
	front41Data	HQER-SFA	hqer__induced	True	33	7.416724770633085	hqer__induced sampler prototype	4.0
	front41Data	Quantile-SFA	hqer__induced	True	28	13.070800466586284	hqer__induced sampler prototype	4.0
	front41Data	HQER-SFA	hqer__induced	True	37	13.204134692418748	hqer__induced sampler prototype	4.0
	front41Data	Quantile-SFA	hqer__induced	True	22	21.65601272785161	hqer__induced sampler prototype	4.0
	front41Data	HQER-SFA	hqer__induced	True	22	21.754616180725243	hqer__induced sampler prototype	4.0
	front41Data	Quantile-SFA	hqer__induced	True	24	3.3744422537912886	hqer__induced sampler prototype	2.0
	front41Data	HQER-SFA	hqer__induced	True	37	3.598927108778793	hqer__induced sampler prototype	2.0
	front41Data	Quantile-SFA	hqer__induced	True	28	-8.670339818791053	hqer__induced sampler prototype	4.0
	front41Data	HQER-SFA	hqer__induced	True	47	-8.396662923328998	hqer__induced sampler prototype	2.0
	front41Data	Quantile-SFA	hqer__induced	True	37	-4.753204480525193	hqer__induced sampler prototype	4.0
	front41Data	HQER-SFA	hqer__induced	True	41	-4.7598080954958455	hqer__induced sampler prototype	4.0
	front41Data	Quantile-SFA	hqer__induced	True	38	17.62761058432649	hqer__induced sampler prototype	2.0
	front41Data	HQER-SFA	hqer__induced	True	28	17.673648637978282	hqer__induced sampler prototype	2.0
	front41Data	Quantile-SFA	hqer__induced	True	19	-22.74077082911456	hqer__induced sampler prototype	4.0
	front41Data	HQER-SFA	hqer__induced	True	29	-22.698715698098336	hqer__induced sampler prototype	2.0
	front41Data	Quantile-SFA	hqer__induced	True	41	12.639400432837755	hqer__induced sampler prototype	2.0
	front41Data	HQER-SFA	hqer__induced	True	52	12.699117970585636	hqer__induced sampler prototype	2.0
	front41Data	Quantile-SFA	hqer__induced	True	46	1.1224818605621605	hqer__induced sampler prototype	4.0
	front41Data	HQER-SFA	hqer__induced	True	34	1.2685295625225923	hqer__induced sampler prototype	4.0
	front41Data	Quantile-SFA	hqer__induced	True	32	-14.988372570437887	hqer__induced sampler prototype	4.0
	front41Data	HQER-SFA	hqer__induced	True	29	-14.879959722262717	hqer__induced sampler prototype	4.0
	front41Data	Quantile-SFA	hqer__induced	True	15	19.5256656413546	hqer__induced sampler prototype	4.0
	front41Data	HQER-SFA	hqer__induced	True	15	19.5909460038392	hqer__induced sampler prototype	4.0
	front41Data	Quantile-SFA	hqer__induced	True	34	34.26810988434576	hqer__induced sampler prototype	2.0
	front41Data	HQER-SFA	hqer__induced	True	34	34.3769366468118	hqer__induced sampler prototype	2.0
	front41Data	Quantile-SFA	hqer__induced	True	15	11.081943964546744	hqer__induced sampler prototype	2.0
	front41Data	HQER-SFA	hqer__induced	True	36	11.201817472540624	hqer__induced sampler prototype	2.0
	front41Data	Quantile-SFA	hqer__induced	True	36	29.54716968849437	hqer__induced sampler prototype	4.0
	front41Data	HQER-SFA	hqer__induced	True	49	29.64133713026229	hqer__induced sampler prototype	4.0
	front41Data	Quantile-SFA	hqer__induced	True	26	2.0498255527987963	hqer__induced sampler prototype	4.0
	front41Data	HQER-SFA	hqer__induced	True	37	2.158424180406919	hqer__induced sampler prototype	4.0
	front41Data	Quantile-SFA	hqer__induced	True	30	30.32735204530182	hqer__induced sampler prototype	2.0
	front41Data	HQER-SFA	hqer__induced	True	24	30.38572441851513	hqer__induced sampler prototype	2.0
	front41Data	Quantile-SFA	hqer__induced	True	25	-6.950778137147564	hqer__induced sampler prototype	4.0
	front41Data	HQER-SFA	hqer__induced	True	31	-6.867655716865006	hqer__induced sampler prototype	4.0
	front41Data	Quantile-SFA	hqer__induced	True	28	22.70683673956802	hqer__induced sampler prototype	2.0
	front41Data	HQER-SFA	hqer__induced	True	22	22.75653269468379	hqer__induced sampler prototype	2.0
	front41Data	Quantile-SFA	hqer__induced	True	38	7.065225461093731	hqer__induced sampler prototype	2.0
	front41Data	HQER-SFA	hqer__induced	True	33	7.169528135312475	hqer__induced sampler prototype	4.0
	front41Data	Quantile-SFA	hqer__induced	True	18	10.341871399626822	hqer__induced sampler prototype	2.0
	front41Data	HQER-SFA	hqer__induced	True	16	10.429603544967376	hqer__induced sampler prototype	4.0
	front41Data	Quantile-SFA	hqer__induced	True	26	-22.69720833485723	hqer__induced sampler prototype	2.0
	front41Data	HQER-SFA	hqer__induced	True	32	-22.607280545938927	hqer__induced sampler prototype	2.0
	front41Data	Quantile-SFA	hqer__induced	True	22	32.776826986842266	hqer__induced sampler prototype	2.0
	front41Data	HQER-SFA	hqer__induced	True	31	32.828897206641656	hqer__induced sampler prototype	2.0
	front41Data	Quantile-SFA	hqer__induced	True	30	-3.924176067717304	hqer__induced sampler prototype	4.0
	front41Data	HQER-SFA	hqer__induced	True	24	-3.7958217036027633	hqer__induced sampler prototype	2.0
	front41Data	Quantile-SFA	hqer__induced	True	30	12.619995994665686	hqer__induced sampler prototype	2.0
	front41Data	HQER-SFA	hqer__induced	True	25	12.765326621245569	hqer__induced sampler prototype	4.0
	front41Data	Quantile-SFA	hqer__induced	True	23	15.55644060171189	hqer__induced sampler prototype	4.0
	front41Data	HQER-SFA	hqer__induced	True	27	15.619243944875896	hqer__induced sampler prototype	4.0
	front41Data	Quantile-SFA	hqer__induced	True	16	-2.509803682341428	hqer__induced sampler prototype	4.0
	front41Data	HQER-SFA	hqer__induced	True	13	-2.4131784104383662	hqer__induced sampler prototype	4.0

experiment	dataset	model	scenario	converged	n__iter	loglik	warning__message	best__start__id
	front41Data	Quantile-SFA	hqer__induced	True	18	-16.09431370330959	hqer__induced sampler prototype	4.0
	front41Data	HQER-SFA	hqer__induced	True	20	-15.943422468570663	hqer__induced sampler prototype	4.0
	front41Data	Quantile-SFA	hqer__induced	True	26	6.4362985390913865	hqer__induced sampler prototype	4.0
	front41Data	HQER-SFA	hqer__induced	True	26	6.567442369571467	hqer__induced sampler prototype	4.0
	front41Data	Quantile-SFA	hqer__induced	True	38	1.8651453475655124	hqer__induced sampler prototype	4.0
	front41Data	HQER-SFA	hqer__induced	True	33	1.9680389852736613	hqer__induced sampler prototype	4.0
	front41Data	Quantile-SFA	hqer__induced	True	46	-17.662532835408626	hqer__induced sampler prototype	4.0
	front41Data	HQER-SFA	hqer__induced	True	28	-17.543871489300066	hqer__induced sampler prototype	4.0
	front41Data	Quantile-SFA	hqer__induced	True	52	-18.03289200658945	hqer__induced sampler prototype	5.0
	front41Data	HQER-SFA	hqer__induced	True	47	-17.951062020260128	hqer__induced sampler prototype	4.0
	front41Data	Quantile-SFA	hqer__induced	True	31	33.29958160916539	hqer__induced sampler prototype	4.0
	front41Data	HQER-SFA	hqer__induced	True	26	33.40071828489442	hqer__induced sampler prototype	4.0
	front41Data	Quantile-SFA	hqer__induced	True	37	9.264681477965071	hqer__induced sampler prototype	2.0
	front41Data	HQER-SFA	hqer__induced	True	23	9.367816078523616	hqer__induced sampler prototype	4.0
	front41Data	Quantile-SFA	hqer__induced	True	21	32.00783326036429	hqer__induced sampler prototype	2.0
	front41Data	HQER-SFA	hqer__induced	True	20	32.12242817067249	hqer__induced sampler prototype	2.0
	front41Data	Quantile-SFA	hqer__induced	True	15	-0.4775422389591512	hqer__induced sampler prototype	4.0
	front41Data	HQER-SFA	hqer__induced	True	51	-0.3767345809793219	hqer__induced sampler prototype	5.0
	front41Data	Quantile-SFA	hqer__induced	True	40	-2.311977601513909	hqer__induced sampler prototype	2.0
	front41Data	HQER-SFA	hqer__induced	True	25	-2.232292929756918	hqer__induced sampler prototype	2.0
	front41Data	Quantile-SFA	hqer__induced	True	21	-13.513843257049848	hqer__induced sampler prototype	2.0
	front41Data	HQER-SFA	hqer__induced	True	15	-13.382745990147402	hqer__induced sampler prototype	2.0
	front41Data	Quantile-SFA	hqer__induced	True	26	-7.609013105944583	hqer__induced sampler prototype	4.0
	front41Data	HQER-SFA	hqer__induced	True	34	-7.6184920092340676	hqer__induced sampler prototype	2.0
	front41Data	Quantile-SFA	hqer__induced	True	20	-3.0698959860551307	hqer__induced sampler prototype	4.0
	front41Data	HQER-SFA	hqer__induced	True	23	-2.943087907085941	hqer__induced sampler prototype	4.0
	front41Data	Quantile-SFA	hqer__induced	True	36	20.06513044615363	hqer__induced sampler prototype	4.0
	front41Data	HQER-SFA	hqer__induced	True	27	20.10598081676154	hqer__induced sampler prototype	2.0
	front41Data	Quantile-SFA	hqer__induced	True	45	-7.987905032051501	hqer__induced sampler prototype	4.0
	front41Data	HQER-SFA	hqer__induced	True	34	-5.944472252489408	hqer__induced sampler prototype	4.0
	front41Data	Quantile-SFA	hqer__induced	True	32	-1.0291772973739537	hqer__induced sampler prototype	2.0
	front41Data	HQER-SFA	hqer__induced	True	40	0.5129696186894914	hqer__induced sampler prototype	2.0
	front41Data	Quantile-SFA	hqer__induced	True	14	-9.354170310027364	hqer__induced sampler prototype	2.0
	front41Data	HQER-SFA	hqer__induced	True	50	-5.568000939641875	hqer__induced sampler prototype	2.0
	front41Data	Quantile-SFA	hqer__induced	True	24	-7.660430238700328	hqer__induced sampler prototype	4.0
	front41Data	HQER-SFA	hqer__induced	True	38	-9.24159384279173	hqer__induced sampler prototype	4.0
	front41Data	Quantile-SFA	hqer__induced	True	50	-5.772812797555748	hqer__induced sampler prototype	4.0
	front41Data	HQER-SFA	hqer__induced	True	28	-3.535391694005339	hqer__induced sampler prototype	4.0
	front41Data	Quantile-SFA	hqer__induced	True	20	-2.302708399759424	hqer__induced sampler prototype	4.0
	front41Data	HQER-SFA	hqer__induced	True	36	-1.5320001271582115	hqer__induced sampler prototype	4.0
	front41Data	Quantile-SFA	hqer__induced	True	19	-3.8327799551410418	hqer__induced sampler prototype	2.0
	front41Data	HQER-SFA	hqer__induced	True	32	-0.71689465091519	hqer__induced sampler prototype	2.0
	front41Data	Quantile-SFA	hqer__induced	True	13	8.722104341702613	hqer__induced sampler prototype	2.0
	front41Data	HQER-SFA	hqer__induced	True	66	8.385321247442763	hqer__induced sampler prototype	2.0
	front41Data	Quantile-SFA	hqer__induced	True	22	-12.102135557891918	hqer__induced sampler prototype	2.0
	front41Data	HQER-SFA	hqer__induced	True	20	-5.049400753929139	hqer__induced sampler prototype	4.0
	front41Data	Quantile-SFA	hqer__induced	True	16	-7.520815724638856	hqer__induced sampler prototype	2.0
	front41Data	HQER-SFA	hqer__induced	True	28	-4.650791817857211	hqer__induced sampler prototype	4.0
	front41Data	Quantile-SFA	hqer__induced	True	18	-7.869463013974833	hqer__induced sampler prototype	4.0
	front41Data	HQER-SFA	hqer__induced	True	22	-6.0155543536757	hqer__induced sampler prototype	4.0
	front41Data	Quantile-SFA	hqer__induced	True	24	-2.7535072842634585	hqer__induced sampler prototype	2.0
	front41Data	HQER-SFA	hqer__induced	True	38	0.528187040588421	hqer__induced sampler prototype	4.0
	front41Data	Quantile-SFA	hqer__induced	True	17	-6.248959063915398	hqer__induced sampler prototype	4.0
	front41Data	HQER-SFA	hqer__induced	True	15	-4.861067265392708	hqer__induced sampler prototype	2.0
	front41Data	Quantile-SFA	hqer__induced	True	32	2.955254587509879	hqer__induced sampler prototype	2.0
	front41Data	HQER-SFA	hqer__induced	True	50	2.684157271641263	hqer__induced sampler prototype	4.0
	front41Data	Quantile-SFA	hqer__induced	True	14	-11.167989340897416	hqer__induced sampler prototype	4.0
	front41Data	HQER-SFA	hqer__induced	True	27	-8.193785629516357	hqer__induced sampler prototype	2.0
	front41Data	Quantile-SFA	hqer__induced	True	58	4.19427288409252	hqer__induced sampler prototype	3.0
	front41Data	HQER-SFA	hqer__induced	True	24	7.114688560650444	hqer__induced sampler prototype	2.0
	front41Data	Quantile-SFA	hqer__induced	True	23	-3.1442807986761947	hqer__induced sampler prototype	4.0
	front41Data	HQER-SFA	hqer__induced	True	54	-3.5127875135482096	hqer__induced sampler prototype	4.0
	front41Data	Quantile-SFA	hqer__induced	True	20	-7.126765160405128	hqer__induced sampler prototype	2.0
	front41Data	HQER-SFA	hqer__induced	True	62	-7.262537005591465	hqer__induced sampler prototype	2.0
	front41Data	Quantile-SFA	hqer__induced	True	17	-7.417447586120415	hqer__induced sampler prototype	2.0
	front41Data	HQER-SFA	hqer__induced	True	44	-6.986377422041788	hqer__induced sampler prototype	2.0
	front41Data	Quantile-SFA	hqer__induced	True	21	-6.553140247459108	hqer__induced sampler prototype	4.0
	front41Data	HQER-SFA	hqer__induced	True	27	-4.078159368835473	hqer__induced sampler prototype	4.0
	front41Data	Quantile-SFA	hqer__induced	True	32	-2.9135215231038645	hqer__induced sampler prototype	4.0
	front41Data	HQER-SFA	hqer__induced	True	14	-1.054201926456116	hqer__induced sampler prototype	4.0
	front41Data	Quantile-SFA	hqer__induced	True	11	6.289633075493725	hqer__induced sampler prototype	2.0
	front41Data	HQER-SFA	hqer__induced	True	36	7.42225254692729	hqer__induced sampler prototype	2.0
	front41Data	Quantile-SFA	hqer__induced	True	23	5.661036389337396	hqer__induced sampler prototype	4.0
	front41Data	HQER-SFA	hqer__induced	True	36	5.833029089354501	hqer__induced sampler prototype	2.0
	front41Data	Quantile-SFA	hqer__induced	True	12	-3.150808805137361	hqer__induced sampler prototype	2.0
	front41Data	HQER-SFA	hqer__induced	True	28	-0.4789800284472238	hqer__induced sampler prototype	4.0
	front41Data	Quantile-SFA	hqer__induced	True	16	-5.910685445928658	hqer__induced sampler prototype	2.0
	front41Data	HQER-SFA	hqer__induced	True	40	-4.789834872084421	hqer__induced sampler prototype	2.0
	front41Data	Quantile-SFA	hqer__induced	True	16	-15.951032092162285	hqer__induced sampler prototype	2.0
	front41Data	HQER-SFA	hqer__induced	True	55	-13.299218462568534	hqer__induced sampler prototype	2.0
	front41Data	Quantile-SFA	hqer__induced	True	76	0.1046789420024693	hqer__induced sampler prototype	4.0
	front41Data	HQER-SFA	hqer__induced	True	31	-0.4864627348645053	hqer__induced sampler prototype	2.0
	front41Data	Quantile-SFA	hqer__induced	True	13	-6.567583132936915	hqer__induced sampler prototype	2.0
	front41Data	HQER-SFA	hqer__induced	True	21	-5.9042239300879	hqer__induced sampler prototype	4.0
	front41Data	Quantile-SFA	hqer__induced	True	15	-6.225315577632777	hqer__induced sampler prototype	2.0
	front41Data	HQER-SFA	hqer__induced	True	47	-4.714308089379498	hqer__induced sampler prototype	2.0
	front41Data	Quantile-SFA	hqer__induced	True	12	-11.096532799142818	hqer__induced sampler prototype	4.0
	front41Data	HQER-SFA	hqer__induced	True	56	-8.2502604818528	hqer__induced sampler prototype	4.0
	front41Data	Quantile-SFA	hqer__induced	True	14	-13.132746474486323	hqer__induced sampler prototype	4.0
	front41Data	HQER-SFA	hqer__induced	True	65	-12.686646344024416	hqer__induced sampler prototype	4.0
	front41Data	Quantile-SFA	hqer__induced	True	25	-1.530128810648754	hqer__induced sampler prototype	2.0
	front41Data	HQER-SFA	hqer__induced	True	29	-0.4696091294268326	hqer__induced sampler prototype	4.0
	front41Data	Quantile-SFA	hqer__induced	True	22	-16.762592977058876	hqer__induced sampler prototype	2.0
	front41Data	HQER-SFA	hqer__induced	True	60	-11.195130692549032	hqer__induced sampler prototype	4.0
	front41Data	Quantile-SFA	hqer__induced	True	13	-2.993047811292346	hqer__induced sampler prototype	2.0
	front41Data	HQER-SFA	hqer__induced	True	6	-2.394072520429809	hqer__induced sampler prototype	2.0
	front41Data	Quantile-SFA	hqer__induced	True	13	-13.466773167703492	hqer__induced sampler prototype	4.0
	front41Data	HQER-SFA	hqer__induced	True	10	-11.447150998307013	hqer__induced sampler prototype	2.0
	front41Data	Quantile-SFA	hqer__induced	True	44	2.50019936503156	hqer__induced sampler prototype	2.0
	front41Data	HQER-SFA	hqer__induced	True	33	4.060405585777442	hqer__induced sampler prototype	4.0
	front41Data	Quantile-SFA	hqer__induced	True	44	1.6317834788542949	hqer__induced sampler prototype	2.0
	front41Data	HQER-SFA	hqer__induced	True	25	1.214850517933866	hqer__induced sampler prototype	4.0
	front41Data	Quantile-SFA	hqer__induced	True	17	-1.6669836754419265	hqer__induced sampler prototype	4.0

experiment	dataset	model	scenario	converged	n__iter	loglik	warning__message	best__start__id
	front41Data	HQER-SFA	hqer__induced	True	49	0.0579803561325996	hqer__induced sampler prototype	4.0
	front41Data	Quantile-SFA	hqer__induced	True	33	-2.0984087240073093	hqer__induced sampler prototype	4.0
	front41Data	HQER-SFA	hqer__induced	True	28	-0.0398855372814067	hqer__induced sampler prototype	3.0
	front41Data	Quantile-SFA	hqer__induced	True	25	2.9380500378302923	hqer__induced sampler prototype	2.0
	front41Data	HQER-SFA	hqer__induced	True	61	8.450468743082155	hqer__induced sampler prototype	4.0
	front41Data	Quantile-SFA	hqer__induced	True	29	-13.96213091943072	hqer__induced sampler prototype	2.0
	front41Data	HQER-SFA	hqer__induced	True	56	-10.362885473912344	hqer__induced sampler prototype	4.0
	front41Data	Quantile-SFA	hqer__induced	True	39	2.368035677331936	hqer__induced sampler prototype	4.0
	front41Data	HQER-SFA	hqer__induced	True	63	3.1309959648549577	hqer__induced sampler prototype	4.0
	front41Data	Quantile-SFA	hqer__induced	True	28	11.335464927555584	hqer__induced sampler prototype	4.0
	front41Data	HQER-SFA	hqer__induced	True	39	10.208139402702864	hqer__induced sampler prototype	1.0
	front41Data	Quantile-SFA	hqer__induced	True	36	3.39010518939371	hqer__induced sampler prototype	2.0
	front41Data	HQER-SFA	hqer__induced	True	68	4.700808911804935	hqer__induced sampler prototype	2.0
	front41Data	Quantile-SFA	hqer__induced	True	20	2.220137261513252	hqer__induced sampler prototype	2.0
	front41Data	HQER-SFA	hqer__induced	True	35	3.780535744787272	hqer__induced sampler prototype	4.0
	front41Data	Quantile-SFA	hqer__induced	True	27	6.824273800801118	hqer__induced sampler prototype	4.0
	front41Data	HQER-SFA	hqer__induced	True	73	6.690705882848127	hqer__induced sampler prototype	2.0
	front41Data	Quantile-SFA	hqer__induced	True	35	6.353232724570861	hqer__induced sampler prototype	2.0
	front41Data	HQER-SFA	hqer__induced	True	73	6.510551128607985	hqer__induced sampler prototype	2.0
	front41Data	Quantile-SFA	hqer__induced	True	19	2.659505423385269	hqer__induced sampler prototype	4.0
	front41Data	HQER-SFA	hqer__induced	True	33	1.9114452621490143	hqer__induced sampler prototype	2.0
	front41Data	Quantile-SFA	hqer__induced	True	36	-1.97405518548152	hqer__induced sampler prototype	4.0
	front41Data	HQER-SFA	hqer__induced	True	49	0.1846654560782901	hqer__induced sampler prototype	4.0
	front41Data	Quantile-SFA	hqer__induced	True	47	-5.5028924861386965	hqer__induced sampler prototype	4.0
	front41Data	HQER-SFA	hqer__induced	True	37	-5.073747742490575	hqer__induced sampler prototype	4.0
	front41Data	Quantile-SFA	hqer__induced	True	15	3.056743197216284	hqer__induced sampler prototype	4.0
	front41Data	HQER-SFA	hqer__induced	True	45	2.931320936759905	hqer__induced sampler prototype	2.0
	front41Data	Quantile-SFA	hqer__induced	True	48	3.60472357627427	hqer__induced sampler prototype	4.0
	front41Data	HQER-SFA	hqer__induced	True	39	5.136311481683042	hqer__induced sampler prototype	4.0
	front41Data	Quantile-SFA	hqer__induced	True	61	0.4717555470684858	hqer__induced sampler prototype	2.0
	front41Data	HQER-SFA	hqer__induced	True	37	1.5397682808321482	hqer__induced sampler prototype	4.0
	front41Data	Quantile-SFA	hqer__induced	True	35	-2.9156315291497963	hqer__induced sampler prototype	4.0
	front41Data	HQER-SFA	hqer__induced	True	24	-1.2446943452991364	hqer__induced sampler prototype	2.0
	front41Data	Quantile-SFA	hqer__induced	True	27	-14.580820724805871	hqer__induced sampler prototype	4.0
	front41Data	HQER-SFA	hqer__induced	True	35	-11.702206010060172	hqer__induced sampler prototype	4.0
	front41Data	Quantile-SFA	hqer__induced	True	29	3.6332959407426193	hqer__induced sampler prototype	2.0
	front41Data	HQER-SFA	hqer__induced	True	15	2.8957578811630578	hqer__induced sampler prototype	2.0
	front41Data	Quantile-SFA	hqer__induced	True	35	-0.5665064570794152	hqer__induced sampler prototype	4.0
	front41Data	HQER-SFA	hqer__induced	True	36	1.4301638772824845	hqer__induced sampler prototype	2.0
	front41Data	Quantile-SFA	hqer__induced	True	34	-4.916313743204264	hqer__induced sampler prototype	2.0
	front41Data	HQER-SFA	hqer__induced	True	45	-2.520876618034462	hqer__induced sampler prototype	4.0
	front41Data	Quantile-SFA	hqer__induced	True	38	2.9424765726312048	hqer__induced sampler prototype	4.0
	front41Data	HQER-SFA	hqer__induced	True	66	3.483216086712708	hqer__induced sampler prototype	4.0
	front41Data	Quantile-SFA	hqer__induced	True	24	-2.1860389298782827	hqer__induced sampler prototype	4.0
	front41Data	HQER-SFA	hqer__induced	True	34	-0.8044394162165143	hqer__induced sampler prototype	4.0
	front41Data	Quantile-SFA	hqer__induced	True	15	-3.214357277000129	hqer__induced sampler prototype	4.0
	front41Data	HQER-SFA	hqer__induced	True	15	-2.929856443194528	hqer__induced sampler prototype	2.0
	front41Data	Quantile-SFA	hqer__induced	True	14	-10.806754002986793	hqer__induced sampler prototype	2.0
	front41Data	HQER-SFA	hqer__induced	True	47	-7.215716581587432	hqer__induced sampler prototype	4.0
	front41Data	Quantile-SFA	hqer__induced	True	34	-5.30175453559689	hqer__induced sampler prototype	4.0
	front41Data	HQER-SFA	hqer__induced	True	19	-4.088596206414816	hqer__induced sampler prototype	4.0
	front41Data	Quantile-SFA	hqer__induced	True	33	0.1850395599262694	hqer__induced sampler prototype	4.0
	front41Data	HQER-SFA	hqer__induced	True	17	1.5296659630397271	hqer__induced sampler prototype	2.0
	front41Data	Quantile-SFA	hqer__induced	True	17	-13.339334451664543	hqer__induced sampler prototype	2.0
	front41Data	HQER-SFA	hqer__induced	True	31	-9.975738361889176	hqer__induced sampler prototype	4.0
	front41Data	Quantile-SFA	hqer__induced	True	14	-1.6347429595329188	hqer__induced sampler prototype	2.0
	front41Data	HQER-SFA	hqer__induced	True	16	-1.5042956154431746	hqer__induced sampler prototype	2.0
	front41Data	Quantile-SFA	hqer__induced	True	14	3.3196900125274253	hqer__induced sampler prototype	2.0
	front41Data	HQER-SFA	hqer__induced	True	59	3.094766062816089	hqer__induced sampler prototype	2.0
	front41Data	Quantile-SFA	hqer__induced	True	32	0.5244346283032086	hqer__induced sampler prototype	4.0
	front41Data	HQER-SFA	hqer__induced	True	32	0.4629691821523032	hqer__induced sampler prototype	2.0
	front41Data	Quantile-SFA	hqer__induced	True	17	-14.54269851591124	hqer__induced sampler prototype	4.0
	front41Data	HQER-SFA	hqer__induced	True	24	-12.36960988905093	hqer__induced sampler prototype	2.0
	front41Data	Quantile-SFA	hqer__induced	True	52	7.666126123567842	hqer__induced sampler prototype	2.0
	front41Data	HQER-SFA	hqer__induced	True	11	8.928616432689282	hqer__induced sampler prototype	4.0
	front41Data	Quantile-SFA	hqer__induced	True	28	3.0063718882633554	hqer__induced sampler prototype	4.0
	front41Data	HQER-SFA	hqer__induced	True	42	4.369762872071459	hqer__induced sampler prototype	4.0
	front41Data	Quantile-SFA	hqer__induced	True	14	-16.491899213205805	hqer__induced sampler prototype	4.0
	front41Data	HQER-SFA	hqer__induced	True	87	-12.499409525047572	hqer__induced sampler prototype	4.0
	front41Data	Quantile-SFA	hqer__induced	True	19	2.1866437616942025	hqer__induced sampler prototype	4.0
	front41Data	HQER-SFA	hqer__induced	True	27	2.794076390129176	hqer__induced sampler prototype	4.0
	front41Data	Quantile-SFA	hqer__induced	True	15	-4.479255849136085	hqer__induced sampler prototype	4.0
	front41Data	HQER-SFA	hqer__induced	True	23	-3.8328801478926096	hqer__induced sampler prototype	4.0
	front41Data	Quantile-SFA	hqer__induced	True	24	-8.80461824790987	hqer__induced sampler prototype	2.0
	front41Data	HQER-SFA	hqer__induced	True	21	-7.238308527864966	hqer__induced sampler prototype	2.0
	front41Data	Quantile-SFA	hqer__induced	True	25	4.634730994474254	hqer__induced sampler prototype	2.0
	front41Data	HQER-SFA	hqer__induced	True	50	4.50659181608409	hqer__induced sampler prototype	4.0
	front41Data	Quantile-SFA	hqer__induced	True	15	-5.621964891812709	hqer__induced sampler prototype	2.0
	front41Data	HQER-SFA	hqer__induced	True	22	-2.2435085372797925	hqer__induced sampler prototype	4.0
	front41Data	Quantile-SFA	hqer__induced	True	13	-12.184579384989476	hqer__induced sampler prototype	2.0
	front41Data	HQER-SFA	hqer__induced	True	25	-12.404341716341245	hqer__induced sampler prototype	4.0
	front41Data	Quantile-SFA	hqer__induced	True	22	-4.414044465542824	hqer__induced sampler prototype	4.0
	front41Data	HQER-SFA	hqer__induced	True	17	-3.2420184362551554	hqer__induced sampler prototype	4.0
	front41Data	Quantile-SFA	hqer__induced	True	24	-9.531148726897811	hqer__induced sampler prototype	2.0
	front41Data	HQER-SFA	hqer__induced	True	29	-6.239366301760292	hqer__induced sampler prototype	4.0
	front41Data	Quantile-SFA	hqer__induced	True	20	7.444561611418016	hqer__induced sampler prototype	4.0
	front41Data	HQER-SFA	hqer__induced	True	27	11.180013691003069	hqer__induced sampler prototype	4.0
	front41Data	Quantile-SFA	hqer__induced	True	28	-5.52418924260322	hqer__induced sampler prototype	4.0
	front41Data	HQER-SFA	hqer__induced	True	26	-4.266625532118196	hqer__induced sampler prototype	2.0
	front41Data	Quantile-SFA	hqer__induced	True	26	5.009336937331613	hqer__induced sampler prototype	4.0
	front41Data	HQER-SFA	hqer__induced	True	56	4.442249283159258	hqer__induced sampler prototype	4.0
	front41Data	Quantile-SFA	hqer__induced	True	40	-13.493371233160143	hqer__induced sampler prototype	4.0
	front41Data	HQER-SFA	hqer__induced	True	15	-12.454944088806911	hqer__induced sampler prototype	4.0
	front41Data	Quantile-SFA	hqer__induced	True	24	-13.96854501021094	hqer__induced sampler prototype	4.0
	front41Data	HQER-SFA	hqer__induced	True	49	-13.911868912238226	hqer__induced sampler prototype	2.0
	front41Data	Quantile-SFA	hqer__induced	True	19	-7.473139572488317	hqer__induced sampler prototype	4.0
	front41Data	HQER-SFA	hqer__induced	True	26	-7.2981160848624	hqer__induced sampler prototype	4.0
	front41Data	Quantile-SFA	hqer__induced	True	13	0.2924219877773094	hqer__induced sampler prototype	2.0
	front41Data	HQER-SFA	hqer__induced	True	29	1.8725379116714205	hqer__induced sampler prototype	2.0
	front41Data	Quantile-SFA	hqer__induced	True	35	2.052358967109656	hqer__induced sampler prototype	2.0
	front41Data	HQER-SFA	hqer__induced	True	35	4.364148057739561	hqer__induced sampler prototype	4.0
	front41Data	Quantile-SFA	hqer__induced	True	36	-3.578835929776406	hqer__induced sampler prototype	4.0
	front41Data	HQER-SFA	hqer__induced	True	23	-2.5171798394289704	hqer__induced sampler prototype	2.0

experiment	dataset	model	scenario	converged	n__iter	loglik	warning__message	best__start__id
	front41Data	Quantile-SFA	hqer__induced	True	15	-1.5351755307063986	hqer__induced sampler prototype	2.0
	front41Data	HQER-SFA	hqer__induced	True	16	-1.4125489041904278	hqer__induced sampler prototype	2.0
	front41Data	Quantile-SFA	hqer__induced	True	25	2.0256549814322984	hqer__induced sampler prototype	2.0
	front41Data	HQER-SFA	hqer__induced	True	42	1.2579723571598764	hqer__induced sampler prototype	2.0
	front41Data	Quantile-SFA	hqer__induced	True	17	1.5385951610615454	hqer__induced sampler prototype	2.0
	front41Data	HQER-SFA	hqer__induced	True	13	1.5075659251563358	hqer__induced sampler prototype	2.0
	front41Data	Quantile-SFA	hqer__induced	True	17	-1.9637926744912264	hqer__induced sampler prototype	2.0
	front41Data	HQER-SFA	hqer__induced	True	51	-2.9815300972615715	hqer__induced sampler prototype	2.0
	front41Data	Quantile-SFA	hqer__induced	True	35	-12.330384318583514	hqer__induced sampler prototype	2.0
	front41Data	HQER-SFA	hqer__induced	True	13	-12.307505920656991	hqer__induced sampler prototype	4.0
	front41Data	Quantile-SFA	hqer__induced	True	49	-4.801889840743642	hqer__induced sampler prototype	4.0
	front41Data	HQER-SFA	hqer__induced	True	28	-2.422754552472393	hqer__induced sampler prototype	4.0
	front41Data	Quantile-SFA	hqer__induced	True	51	0.216318227087839	hqer__induced sampler prototype	4.0
	front41Data	HQER-SFA	hqer__induced	True	11	1.0654664465550596	hqer__induced sampler prototype	2.0
	front41Data	Quantile-SFA	hqer__induced	True	44	-0.7914073934001303	hqer__induced sampler prototype	2.0
	front41Data	HQER-SFA	hqer__induced	True	32	-1.659367543964032	hqer__induced sampler prototype	2.0
	front41Data	Quantile-SFA	hqer__induced	True	25	-7.963077006274792	hqer__induced sampler prototype	4.0
	front41Data	HQER-SFA	hqer__induced	True	33	-6.794784284817949	hqer__induced sampler prototype	2.0
	front41Data	Quantile-SFA	hqer__induced	True	27	10.451345698728304	hqer__induced sampler prototype	4.0
	front41Data	HQER-SFA	hqer__induced	True	44	11.716033635941752	hqer__induced sampler prototype	2.0
	front41Data	Quantile-SFA	hqer__induced	True	35	-3.231487280815705	hqer__induced sampler prototype	4.0
	front41Data	HQER-SFA	hqer__induced	True	30	-0.6481563728112916	hqer__induced sampler prototype	2.0
	front41Data	Quantile-SFA	hqer__induced	True	22	-55.2625491072293	hqer__induced sampler prototype	4.0
	front41Data	HQER-SFA	hqer__induced	True	13	-43.54855119774691	hqer__induced sampler prototype	4.0
	front41Data	Quantile-SFA	hqer__induced	True	16	-79.81151868606827	hqer__induced sampler prototype	4.0
	front41Data	HQER-SFA	hqer__induced	True	12	-69.15534820480207	hqer__induced sampler prototype	2.0
	front41Data	Quantile-SFA	hqer__induced	True	16	-66.49871039393857	hqer__induced sampler prototype	4.0
	front41Data	HQER-SFA	hqer__induced	True	12	-54.35003772590214	hqer__induced sampler prototype	4.0
	front41Data	Quantile-SFA	hqer__induced	True	15	-69.21297685664493	hqer__induced sampler prototype	4.0
	front41Data	HQER-SFA	hqer__induced	True	16	-50.39824803125244	hqer__induced sampler prototype	4.0
	front41Data	Quantile-SFA	hqer__induced	True	16	-25.735635080718367	hqer__induced sampler prototype	4.0
	front41Data	HQER-SFA	hqer__induced	True	35	-18.269174886409456	hqer__induced sampler prototype	4.0
	front41Data	Quantile-SFA	hqer__induced	True	21	-42.31884000414656	hqer__induced sampler prototype	4.0
	front41Data	HQER-SFA	hqer__induced	True	5	-30.331195965167865	hqer__induced sampler prototype	2.0
	front41Data	Quantile-SFA	hqer__induced	True	22	-50.04874857025462	hqer__induced sampler prototype	2.0
	front41Data	HQER-SFA	hqer__induced	True	29	-28.818180923558835	hqer__induced sampler prototype	2.0
	front41Data	Quantile-SFA	hqer__induced	True	16	-63.64965355379197	hqer__induced sampler prototype	4.0
	front41Data	HQER-SFA	hqer__induced	True	34	-46.52344844314115	hqer__induced sampler prototype	4.0
	front41Data	Quantile-SFA	hqer__induced	True	17	-75.03757874886901	hqer__induced sampler prototype	2.0
	front41Data	HQER-SFA	hqer__induced	True	21	-60.76322341718135	hqer__induced sampler prototype	2.0
	front41Data	Quantile-SFA	hqer__induced	True	36	-56.88127968218571	hqer__induced sampler prototype	4.0
	front41Data	HQER-SFA	hqer__induced	True	21	-42.52935838719367	hqer__induced sampler prototype	4.0
	front41Data	Quantile-SFA	hqer__induced	True	17	-12.807845958634005	hqer__induced sampler prototype	2.0
	front41Data	HQER-SFA	hqer__induced	True	45	-6.354445808148707	hqer__induced sampler prototype	4.0
	front41Data	Quantile-SFA	hqer__induced	True	26	-40.76590300424746	hqer__induced sampler prototype	2.0
	front41Data	HQER-SFA	hqer__induced	True	16	-29.10965733218196	hqer__induced sampler prototype	4.0
	front41Data	Quantile-SFA	hqer__induced	True	31	-51.23543013729751	hqer__induced sampler prototype	4.0
	front41Data	HQER-SFA	hqer__induced	True	19	-36.94657341176514	hqer__induced sampler prototype	4.0
	front41Data	Quantile-SFA	hqer__induced	True	27	-46.35041952428807	hqer__induced sampler prototype	2.0
	front41Data	HQER-SFA	hqer__induced	True	11	-31.166545199660533	hqer__induced sampler prototype	4.0
	front41Data	Quantile-SFA	hqer__induced	True	21	-48.70392812445405	hqer__induced sampler prototype	4.0
	front41Data	HQER-SFA	hqer__induced	True	8	-38.447956665887816	hqer__induced sampler prototype	2.0
	front41Data	Quantile-SFA	hqer__induced	True	42	-50.89478881578123	hqer__induced sampler prototype	2.0
	front41Data	HQER-SFA	hqer__induced	True	21	-38.03695450991918	hqer__induced sampler prototype	2.0
	front41Data	Quantile-SFA	hqer__induced	True	15	-68.90641803040158	hqer__induced sampler prototype	2.0
	front41Data	HQER-SFA	hqer__induced	True	21	-52.68899948868927	hqer__induced sampler prototype	4.0
	front41Data	Quantile-SFA	hqer__induced	True	31	-79.19845551943068	hqer__induced sampler prototype	2.0
	front41Data	HQER-SFA	hqer__induced	True	10	-65.50865053750569	hqer__induced sampler prototype	4.0
	front41Data	Quantile-SFA	hqer__induced	True	37	-29.2323690148752	hqer__induced sampler prototype	4.0
	front41Data	HQER-SFA	hqer__induced	True	47	-24.865266151274085	hqer__induced sampler prototype	4.0
	front41Data	Quantile-SFA	hqer__induced	True	18	-49.37238460041152	hqer__induced sampler prototype	4.0
	front41Data	HQER-SFA	hqer__induced	True	10	-37.68988857953396	hqer__induced sampler prototype	2.0
	front41Data	Quantile-SFA	hqer__induced	True	38	-37.17611200781093	hqer__induced sampler prototype	2.0
	front41Data	HQER-SFA	hqer__induced	True	29	-22.44917864136076	hqer__induced sampler prototype	2.0
	front41Data	Quantile-SFA	hqer__induced	True	30	-76.30603442011092	hqer__induced sampler prototype	2.0
	front41Data	HQER-SFA	hqer__induced	True	14	-66.2316953479718	hqer__induced sampler prototype	2.0
	front41Data	Quantile-SFA	hqer__induced	True	39	-67.28519542236324	hqer__induced sampler prototype	2.0
	front41Data	HQER-SFA	hqer__induced	True	8	-56.82334416592099	hqer__induced sampler prototype	4.0
	front41Data	Quantile-SFA	hqer__induced	True	25	-45.71323903852874	hqer__induced sampler prototype	2.0
	front41Data	HQER-SFA	hqer__induced	True	22	-30.94544137889693	hqer__induced sampler prototype	2.0
	front41Data	Quantile-SFA	hqer__induced	True	25	-40.88042853431762	hqer__induced sampler prototype	2.0
	front41Data	HQER-SFA	hqer__induced	True	13	-27.724163538583703	hqer__induced sampler prototype	4.0
	front41Data	Quantile-SFA	hqer__induced	True	15	-26.512022664801925	hqer__induced sampler prototype	2.0
	front41Data	HQER-SFA	hqer__induced	True	51	-22.287682635972395	hqer__induced sampler prototype	4.0
	front41Data	Quantile-SFA	hqer__induced	True	21	-75.43873053113656	hqer__induced sampler prototype	2.0
	front41Data	HQER-SFA	hqer__induced	True	47	-67.30526924463899	hqer__induced sampler prototype	4.0
	front41Data	Quantile-SFA	hqer__induced	True	27	-54.015178907281935	hqer__induced sampler prototype	2.0
	front41Data	HQER-SFA	hqer__induced	True	12	-37.68987624305955	hqer__induced sampler prototype	4.0
	front41Data	Quantile-SFA	hqer__induced	True	34	-20.25287736207177	hqer__induced sampler prototype	2.0
	front41Data	HQER-SFA	hqer__induced	True	21	-13.08191795604846	hqer__induced sampler prototype	2.0
	front41Data	Quantile-SFA	hqer__induced	True	22	-71.74056411333127	hqer__induced sampler prototype	2.0
	front41Data	HQER-SFA	hqer__induced	True	30	-54.613312988164445	hqer__induced sampler prototype	2.0
	front41Data	Quantile-SFA	hqer__induced	True	33	-35.7922512614068	hqer__induced sampler prototype	2.0
	front41Data	HQER-SFA	hqer__induced	True	8	-18.9606761367428	hqer__induced sampler prototype	2.0
	front41Data	Quantile-SFA	hqer__induced	True	25	4.41930287153866	hqer__induced sampler prototype	2.0
	front41Data	HQER-SFA	hqer__induced	True	26	17.532153443631078	hqer__induced sampler prototype	2.0
	front41Data	Quantile-SFA	hqer__induced	True	17	-18.23987266810788	hqer__induced sampler prototype	4.0
	front41Data	HQER-SFA	hqer__induced	True	33	-10.26845711199593	hqer__induced sampler prototype	4.0
	front41Data	Quantile-SFA	hqer__induced	True	32	-51.05234403001385	hqer__induced sampler prototype	4.0
	front41Data	HQER-SFA	hqer__induced	True	35	-35.777668473028854	hqer__induced sampler prototype	2.0
	front41Data	Quantile-SFA	hqer__induced	True	25	-67.96022606966613	hqer__induced sampler prototype	4.0
	front41Data	HQER-SFA	hqer__induced	True	9	-53.78031588290899	hqer__induced sampler prototype	2.0
	front41Data	Quantile-SFA	hqer__induced	True	21	-27.99659948260146	hqer__induced sampler prototype	4.0
	front41Data	HQER-SFA	hqer__induced	True	13	-9.472480572489062	hqer__induced sampler prototype	2.0
	front41Data	Quantile-SFA	hqer__induced	True	28	-52.92049706794106	hqer__induced sampler prototype	4.0
	front41Data	HQER-SFA	hqer__induced	True	16	-34.87976251931599	hqer__induced sampler prototype	2.0
	front41Data	Quantile-SFA	hqer__induced	True	22	-39.244019627565294	hqer__induced sampler prototype	4.0
	front41Data	HQER-SFA	hqer__induced	True	9	-30.694529914505885	hqer__induced sampler prototype	2.0
	front41Data	Quantile-SFA	hqer__induced	True	35	-38.02429676284864	hqer__induced sampler prototype	4.0
	front41Data	HQER-SFA	hqer__induced	True	27	-27.32449380392788	hqer__induced sampler prototype	4.0
	front41Data	Quantile-SFA	hqer__induced	True	41	-51.14199860474268	hqer__induced sampler prototype	2.0
	front41Data	HQER-SFA	hqer__induced	True	24	-38.53782404098769	hqer__induced sampler prototype	2.0
	front41Data	Quantile-SFA	hqer__induced	True	13	-57.74996610583423	hqer__induced sampler prototype	4.0

experiment	dataset	model	scenario	converged	n__iter	loglik	warning__message	best__start__id
	front41Data	HQER-SFA	hqer__induced	True	11	-43.92330034946954	hqer__induced sampler prototype	2.0
	front41Data	Quantile-SFA	hqer__induced	True	13	-57.27341707710754	hqer__induced sampler prototype	4.0
	front41Data	HQER-SFA	hqer__induced	True	11	-51.98098696346493	hqer__induced sampler prototype	2.0
	front41Data	Quantile-SFA	hqer__induced	True	14	-39.38447672157108	hqer__induced sampler prototype	2.0
	front41Data	HQER-SFA	hqer__induced	True	18	-18.501042636322527	hqer__induced sampler prototype	4.0
	front41Data	Quantile-SFA	hqer__induced	True	28	-61.16546566609983	hqer__induced sampler prototype	4.0
	front41Data	HQER-SFA	hqer__induced	True	17	-40.69001120355741	hqer__induced sampler prototype	2.0
	front41Data	Quantile-SFA	hqer__induced	True	26	-43.17969814520941	hqer__induced sampler prototype	2.0
	front41Data	HQER-SFA	hqer__induced	True	29	-21.32707017110657	hqer__induced sampler prototype	2.0
	front41Data	Quantile-SFA	hqer__induced	True	29	-57.97427417305372	hqer__induced sampler prototype	2.0
	front41Data	HQER-SFA	hqer__induced	True	13	-38.54895836233186	hqer__induced sampler prototype	4.0
	front41Data	Quantile-SFA	hqer__induced	True	38	-55.000919937269146	hqer__induced sampler prototype	4.0
	front41Data	HQER-SFA	hqer__induced	True	25	-36.64236636413291	hqer__induced sampler prototype	2.0
	front41Data	Quantile-SFA	hqer__induced	True	25	-72.86704096563294	hqer__induced sampler prototype	2.0
	front41Data	HQER-SFA	hqer__induced	True	17	-64.29947105113118	hqer__induced sampler prototype	2.0
	front41Data	Quantile-SFA	hqer__induced	True	20	-62.99483985803876	hqer__induced sampler prototype	2.0
	front41Data	HQER-SFA	hqer__induced	True	8	-47.98804796653835	hqer__induced sampler prototype	2.0
	front41Data	Quantile-SFA	hqer__induced	True	27	-83.16858011132655	hqer__induced sampler prototype	4.0
	front41Data	HQER-SFA	hqer__induced	True	10	-67.1620327948634	hqer__induced sampler prototype	4.0
	front41Data	Quantile-SFA	hqer__induced	True	29	-15.89350020598443	hqer__induced sampler prototype	4.0
	front41Data	HQER-SFA	hqer__induced	True	20	-10.079446619239857	hqer__induced sampler prototype	4.0
	front41Data	Quantile-SFA	hqer__induced	True	12	-34.84837273382358	hqer__induced sampler prototype	4.0
	front41Data	HQER-SFA	hqer__induced	True	52	-21.79758672237694	hqer__induced sampler prototype	4.0
	front41Data	Quantile-SFA	hqer__induced	True	29	-64.11728237254574	hqer__induced sampler prototype	2.0
	front41Data	HQER-SFA	hqer__induced	True	40	-42.41517470868144	hqer__induced sampler prototype	2.0
	front41Data	Quantile-SFA	hqer__induced	True	37	-37.11694533692098	hqer__induced sampler prototype	4.0
	front41Data	HQER-SFA	hqer__induced	True	22	-25.804564864584314	hqer__induced sampler prototype	4.0
	front41Data	Quantile-SFA	hqer__induced	True	37	-64.15286812997029	hqer__induced sampler prototype	2.0
	front41Data	HQER-SFA	hqer__induced	True	24	-51.66463726904689	hqer__induced sampler prototype	4.0
	front41Data	Quantile-SFA	hqer__induced	True	20	-48.47406769464689	hqer__induced sampler prototype	4.0
	front41Data	HQER-SFA	hqer__induced	True	11	-40.61163234275882	hqer__induced sampler prototype	2.0
	front41Data	Quantile-SFA	hqer__induced	True	14	-64.51814540695145	hqer__induced sampler prototype	4.0
	front41Data	HQER-SFA	hqer__induced	True	13	-41.9316396536242	hqer__induced sampler prototype	4.0
	front41Data	Quantile-SFA	hqer__induced	True	30	-12.519984168175949	hqer__induced sampler prototype	4.0
	front41Data	HQER-SFA	hqer__induced	True	37	-6.316767867773664	hqer__induced sampler prototype	4.0
	front41Data	Quantile-SFA	hqer__induced	True	27	-33.81478348934159	hqer__induced sampler prototype	4.0
	front41Data	HQER-SFA	hqer__induced	True	15	-22.84263901913081	hqer__induced sampler prototype	4.0
	front41Data	Quantile-SFA	hqer__induced	True	28	-41.66458932960191	hqer__induced sampler prototype	2.0
	front41Data	HQER-SFA	hqer__induced	True	22	-33.18135344532972	hqer__induced sampler prototype	4.0
	front41Data	Quantile-SFA	hqer__induced	True	36	-62.20917952886658	hqer__induced sampler prototype	2.0
	front41Data	HQER-SFA	hqer__induced	True	30	-48.90699873654917	hqer__induced sampler prototype	4.0
	front41Data	Quantile-SFA	hqer__induced	True	12	-99.10146837468008	hqer__induced sampler prototype	4.0
	front41Data	HQER-SFA	hqer__induced	True	13	-82.7230514142478	hqer__induced sampler prototype	4.0
	front41Data	Quantile-SFA	hqer__induced	True	20	-27.088208563614064	hqer__induced sampler prototype	4.0
	front41Data	HQER-SFA	hqer__induced	True	7	-16.89443372452211	hqer__induced sampler prototype	2.0
	front41Data	Quantile-SFA	hqer__induced	True	31	-45.856887684098105	hqer__induced sampler prototype	2.0
	front41Data	HQER-SFA	hqer__induced	True	16	-31.504335625379213	hqer__induced sampler prototype	4.0
	front41Data	Quantile-SFA	hqer__induced	True	31	-56.24756231883688	hqer__induced sampler prototype	4.0
	front41Data	HQER-SFA	hqer__induced	True	19	-31.700077411757924	hqer__induced sampler prototype	2.0
	front41Data	Quantile-SFA	hqer__induced	True	20	-46.31235668351637	hqer__induced sampler prototype	4.0
	front41Data	HQER-SFA	hqer__induced	True	25	-31.373709491518888	hqer__induced sampler prototype	4.0
	front41Data	Quantile-SFA	hqer__induced	True	30	-44.8386254421893	hqer__induced sampler prototype	2.0
	front41Data	HQER-SFA	hqer__induced	True	16	-30.06163065620036	hqer__induced sampler prototype	2.0
	front41Data	Quantile-SFA	hqer__induced	True	20	-58.27198078931938	hqer__induced sampler prototype	4.0
	front41Data	HQER-SFA	hqer__induced	True	25	-40.03353989086996	hqer__induced sampler prototype	4.0
	front41Data	Quantile-SFA	hqer__induced	True	20	-50.51756029269698	hqer__induced sampler prototype	2.0
	front41Data	HQER-SFA	hqer__induced	True	56	-45.06380790684099	hqer__induced sampler prototype	4.0
	front41Data	Quantile-SFA	hqer__induced	True	15	-54.21120127301395	hqer__induced sampler prototype	2.0
	front41Data	HQER-SFA	hqer__induced	True	27	-32.32087019008085	hqer__induced sampler prototype	4.0
	front41Data	Quantile-SFA	hqer__induced	True	32	-32.37683371088039	hqer__induced sampler prototype	2.0
	front41Data	HQER-SFA	hqer__induced	True	61	-25.015668348170983	hqer__induced sampler prototype	4.0
	front41Data	Quantile-SFA	hqer__induced	True	24	-69.22721344439134	hqer__induced sampler prototype	2.0
	front41Data	HQER-SFA	hqer__induced	True	11	-63.1939959233185	hqer__induced sampler prototype	2.0
	front41Data	Quantile-SFA	hqer__induced	True	31	-50.87512548234983	hqer__induced sampler prototype	2.0
	front41Data	HQER-SFA	hqer__induced	True	9	-35.781595825309694	hqer__induced sampler prototype	2.0
	front41Data	Quantile-SFA	hqer__induced	True	30	-52.68473877910718	hqer__induced sampler prototype	4.0
	front41Data	HQER-SFA	hqer__induced	True	16	-37.76825218910129	hqer__induced sampler prototype	2.0
	front41Data	Quantile-SFA	hqer__induced	True	13	-32.88619345749332	hqer__induced sampler prototype	4.0
	front41Data	HQER-SFA	hqer__induced	True	14	-20.58144126317178	hqer__induced sampler prototype	2.0
	front41Data	Quantile-SFA	hqer__induced	True	12	-39.33840009630508	hqer__induced sampler prototype	4.0
	front41Data	HQER-SFA	hqer__induced	True	14	-24.83882685765334	hqer__induced sampler prototype	4.0
	front41Data	Quantile-SFA	hqer__induced	True	28	-37.52673998722848	hqer__induced sampler prototype	4.0
	front41Data	HQER-SFA	hqer__induced	True	11	-25.186881532465183	hqer__induced sampler prototype	4.0
	front41Data	Quantile-SFA	hqer__induced	True	21	-22.59576836887925	hqer__induced sampler prototype	4.0
	front41Data	HQER-SFA	hqer__induced	True	25	-12.056972134355938	hqer__induced sampler prototype	4.0
	front41Data	Quantile-SFA	hqer__induced	True	30	-47.8505574035847	hqer__induced sampler prototype	4.0
	front41Data	HQER-SFA	hqer__induced	True	32	-39.01355554041805	hqer__induced sampler prototype	4.0
	front41Data	Quantile-SFA	hqer__induced	True	32	-53.77724157131138	hqer__induced sampler prototype	2.0
	front41Data	HQER-SFA	hqer__induced	True	39	-38.57653783752218	hqer__induced sampler prototype	4.0
	front41Data	Quantile-SFA	hqer__induced	True	62	-4.314605292857408	hqer__induced sampler prototype	4.0
	front41Data	HQER-SFA	hqer__induced	True	50	4.922436966910034	hqer__induced sampler prototype	2.0
	front41Data	Quantile-SFA	hqer__induced	True	52	-38.83301523130319	hqer__induced sampler prototype	4.0
	front41Data	HQER-SFA	hqer__induced	True	27	-24.89473260007346	hqer__induced sampler prototype	2.0
	front41Data	Quantile-SFA	hqer__induced	True	17	-52.97202734071513	hqer__induced sampler prototype	4.0
	front41Data	HQER-SFA	hqer__induced	True	9	-49.1271772917324	hqer__induced sampler prototype	2.0
	front41Data	Quantile-SFA	hqer__induced	True	38	-63.15953768085342	hqer__induced sampler prototype	2.0
	front41Data	HQER-SFA	hqer__induced	True	11	-52.49255997391081	hqer__induced sampler prototype	4.0
	front41Data	Quantile-SFA	hqer__induced	True	31	-34.60638023498218	hqer__induced sampler prototype	4.0
	front41Data	HQER-SFA	hqer__induced	True	25	-26.78314583155543	hqer__induced sampler prototype	4.0
	front41Data	Quantile-SFA	hqer__induced	True	20	-69.35891480963966	hqer__induced sampler prototype	4.0
	front41Data	HQER-SFA	hqer__induced	True	18	-58.596479283568186	hqer__induced sampler prototype	2.0
	front41Data	Quantile-SFA	hqer__induced	True	17	-67.45931576958202	hqer__induced sampler prototype	4.0
	front41Data	HQER-SFA	hqer__induced	True	28	-50.79326627601952	hqer__induced sampler prototype	2.0
	front41Data	Quantile-SFA	hqer__induced	True	22	-35.10312063669661	hqer__induced sampler prototype	2.0
	front41Data	HQER-SFA	hqer__induced	True	16	-25.806814635201327	hqer__induced sampler prototype	2.0
	front41Data	Quantile-SFA	hqer__induced	True	25	-39.78793057774448	hqer__induced sampler prototype	2.0
	front41Data	HQER-SFA	hqer__induced	True	14	-25.18711920580305	hqer__induced sampler prototype	2.0
	front41Data	Quantile-SFA	hqer__induced	True	16	-12.34764120311026	hqer__induced sampler prototype	2.0
	front41Data	HQER-SFA	hqer__induced	True	32	-4.718614375237794	hqer__induced sampler prototype	2.0
	front41Data	Quantile-SFA	hqer__induced	True	23	-45.2680893371836	hqer__induced sampler prototype	4.0
	front41Data	HQER-SFA	hqer__induced	True	8	-32.173946013921444	hqer__induced sampler prototype	2.0
	front41Data	Quantile-SFA	hqer__induced	True	47	-30.044427102995343	hqer__induced sampler prototype	4.0
	front41Data	HQER-SFA	hqer__induced	True	21	-12.172867667932907	hqer__induced sampler prototype	4.0

experiment	dataset	model	scenario	converged	n_iter	loglik	warning_message	best_start_id
	front41Data	Quantile-SFA	hqer_induced	True	15	-57.060978024087774	hqer_induced sampler prototype	4.0
	front41Data	HQER-SFA	hqer_induced	True	11	-40.62528257460607	hqer_induced sampler prototype	2.0
	front41Data	Quantile-SFA	hqer_induced	True	38	-40.507470797398256	hqer_induced sampler prototype	2.0
	front41Data	HQER-SFA	hqer_induced	True	16	-31.32826662424344	hqer_induced sampler prototype	4.0
	front41Data	Quantile-SFA	hqer_induced	True	20	-24.47992860352907	hqer_induced sampler prototype	2.0
	front41Data	HQER-SFA	hqer_induced	True	29	-14.69733597936926	hqer_induced sampler prototype	2.0
	front41Data	Quantile-SFA	hqer_induced	True	38	-66.91331256852258	hqer_induced sampler prototype	2.0
	front41Data	HQER-SFA	hqer_induced	True	29	-47.71492771460501	hqer_induced sampler prototype	4.0
	front41Data	Quantile-SFA	hqer_induced	True	23	-58.947753848410144	hqer_induced sampler prototype	4.0
	front41Data	HQER-SFA	hqer_induced	True	10	-47.07924283278406	hqer_induced sampler prototype	2.0
	front41Data	Quantile-SFA	hqer_induced	True	35	-49.333673272800446	hqer_induced sampler prototype	4.0
	front41Data	HQER-SFA	hqer_induced	True	32	-35.45620704774319	hqer_induced sampler prototype	2.0
	front41Data	Quantile-SFA	hqer_induced	True	33	-45.6846092295531	hqer_induced sampler prototype	2.0
	front41Data	HQER-SFA	hqer_induced	True	32	-30.79065813082736	hqer_induced sampler prototype	2.0
	front41Data	Quantile-SFA	hqer_induced	True	17	-73.82741305461852	hqer_induced sampler prototype	4.0
	front41Data	HQER-SFA	hqer_induced	True	22	-56.94816109803742	hqer_induced sampler prototype	2.0
	WDI_industry	Quantile-SFA	hqer_induced	True	19	-165.6621995592684	hqer_induced sampler prototype	2.0
	WDI_industry	HQER-SFA	hqer_induced	True	24	-165.637135129797	hqer_induced sampler prototype	2.0
	WDI_industry	Quantile-SFA	hqer_induced	True	17	-138.64951807729602	hqer_induced sampler prototype	2.0
	WDI_industry	HQER-SFA	hqer_induced	True	15	-138.60405499057623	hqer_induced sampler prototype	2.0
	WDI_industry	Quantile-SFA	hqer_induced	True	35	-131.42923881587137	hqer_induced sampler prototype	4.0
	WDI_industry	HQER-SFA	hqer_induced	True	26	-131.39470710781734	hqer_induced sampler prototype	4.0
	WDI_industry	Quantile-SFA	hqer_induced	True	26	-114.3925250749536	hqer_induced sampler prototype	4.0
	WDI_industry	HQER-SFA	hqer_induced	True	27	-114.35397202957792	hqer_induced sampler prototype	4.0
	WDI_industry	Quantile-SFA	hqer_induced	True	25	-141.66341142522035	hqer_induced sampler prototype	4.0
	WDI_industry	HQER-SFA	hqer_induced	True	24	-141.6503520845356	hqer_induced sampler prototype	4.0
	WDI_industry	Quantile-SFA	hqer_induced	True	46	-150.0325577858692	hqer_induced sampler prototype	5.0
	WDI_industry	HQER-SFA	hqer_induced	True	19	-149.9793785282685	hqer_induced sampler prototype	4.0
	WDI_industry	Quantile-SFA	hqer_induced	True	19	-133.73362781894338	hqer_induced sampler prototype	2.0
	WDI_industry	HQER-SFA	hqer_induced	True	21	-133.6806016921286	hqer_induced sampler prototype	2.0
	WDI_industry	Quantile-SFA	hqer_induced	True	24	-148.7030161524974	hqer_induced sampler prototype	4.0
	WDI_industry	HQER-SFA	hqer_induced	True	23	-148.6500409835131	hqer_induced sampler prototype	4.0
	WDI_industry	Quantile-SFA	hqer_induced	True	19	-135.50556506982906	hqer_induced sampler prototype	4.0
	WDI_industry	HQER-SFA	hqer_induced	True	25	-135.45891555288765	hqer_induced sampler prototype	4.0
	WDI_industry	Quantile-SFA	hqer_induced	True	22	-136.20826520051185	hqer_induced sampler prototype	4.0
	WDI_industry	HQER-SFA	hqer_induced	True	23	-136.2065882501225	hqer_induced sampler prototype	4.0
	WDI_industry	Quantile-SFA	hqer_induced	True	29	-147.23783868801144	hqer_induced sampler prototype	2.0
	WDI_industry	HQER-SFA	hqer_induced	True	24	-147.17665785077045	hqer_induced sampler prototype	4.0
	WDI_industry	Quantile-SFA	hqer_induced	True	20	-130.83286333649176	hqer_induced sampler prototype	2.0
	WDI_industry	HQER-SFA	hqer_induced	True	19	-130.77508842975823	hqer_induced sampler prototype	2.0
	WDI_industry	Quantile-SFA	hqer_induced	True	24	-135.21629894039694	hqer_induced sampler prototype	4.0
	WDI_industry	HQER-SFA	hqer_induced	True	17	-135.18806967408312	hqer_induced sampler prototype	2.0
	WDI_industry	Quantile-SFA	hqer_induced	True	25	-141.75389475731322	hqer_induced sampler prototype	2.0
	WDI_industry	HQER-SFA	hqer_induced	True	21	-141.7134999754008	hqer_induced sampler prototype	4.0
	WDI_industry	Quantile-SFA	hqer_induced	True	29	-144.5049048192513	hqer_induced sampler prototype	4.0
	WDI_industry	HQER-SFA	hqer_induced	True	26	-144.45545140532698	hqer_induced sampler prototype	4.0
	WDI_industry	Quantile-SFA	hqer_induced	True	20	-130.81963651639762	hqer_induced sampler prototype	4.0
	WDI_industry	HQER-SFA	hqer_induced	True	30	-130.76663407659908	hqer_induced sampler prototype	4.0
	WDI_industry	Quantile-SFA	hqer_induced	True	20	-144.21882577505892	hqer_induced sampler prototype	4.0
	WDI_industry	HQER-SFA	hqer_induced	True	24	-144.2144650732356	hqer_induced sampler prototype	4.0
	WDI_industry	Quantile-SFA	hqer_induced	True	27	-113.030866572938	hqer_induced sampler prototype	4.0
	WDI_industry	HQER-SFA	hqer_induced	True	23	-113.02713623820254	hqer_induced sampler prototype	4.0
	WDI_industry	Quantile-SFA	hqer_induced	True	28	-144.62810136258597	hqer_induced sampler prototype	2.0
	WDI_industry	HQER-SFA	hqer_induced	True	23	-144.5781922260337	hqer_induced sampler prototype	2.0
	WDI_industry	Quantile-SFA	hqer_induced	True	16	-116.14597274252752	hqer_induced sampler prototype	2.0
	WDI_industry	HQER-SFA	hqer_induced	True	23	-116.12498900656784	hqer_induced sampler prototype	4.0
	WDI_industry	Quantile-SFA	hqer_induced	True	24	-116.04694543772912	hqer_induced sampler prototype	4.0
	WDI_industry	HQER-SFA	hqer_induced	True	28	-116.01092704777692	hqer_induced sampler prototype	4.0
	WDI_industry	Quantile-SFA	hqer_induced	True	22	-145.9237897656552	hqer_induced sampler prototype	4.0
	WDI_industry	HQER-SFA	hqer_induced	True	21	-145.87410066253167	hqer_induced sampler prototype	4.0
	WDI_industry	Quantile-SFA	hqer_induced	True	19	-119.54250743457663	hqer_induced sampler prototype	2.0
	WDI_industry	HQER-SFA	hqer_induced	True	13	-119.52034766118192	hqer_induced sampler prototype	2.0
	WDI_industry	Quantile-SFA	hqer_induced	True	13	-108.71376776325468	hqer_induced sampler prototype	2.0
	WDI_industry	HQER-SFA	hqer_induced	True	18	-108.66854520849962	hqer_induced sampler prototype	2.0
	WDI_industry	Quantile-SFA	hqer_induced	True	29	-175.4968317757366	hqer_induced sampler prototype	4.0
	WDI_industry	HQER-SFA	hqer_induced	True	26	-175.4811878309309	hqer_induced sampler prototype	4.0
	WDI_industry	Quantile-SFA	hqer_induced	True	22	-108.71236097047247	hqer_induced sampler prototype	4.0
	WDI_industry	HQER-SFA	hqer_induced	True	28	-108.6736552484428	hqer_induced sampler prototype	4.0
	WDI_industry	Quantile-SFA	hqer_induced	True	24	-149.87456563981425	hqer_induced sampler prototype	4.0
	WDI_industry	HQER-SFA	hqer_induced	True	29	-149.83267163654827	hqer_induced sampler prototype	4.0
	WDI_industry	Quantile-SFA	hqer_induced	True	22	-144.96780640759482	hqer_induced sampler prototype	4.0
	WDI_industry	HQER-SFA	hqer_induced	True	20	-144.93358240656823	hqer_induced sampler prototype	4.0
	WDI_industry	Quantile-SFA	hqer_induced	True	15	-120.86747575410376	hqer_induced sampler prototype	2.0
	WDI_industry	HQER-SFA	hqer_induced	True	23	-120.80330613751246	hqer_induced sampler prototype	2.0
	WDI_industry	Quantile-SFA	hqer_induced	True	23	-121.91320352971022	hqer_induced sampler prototype	4.0
	WDI_industry	HQER-SFA	hqer_induced	True	21	-121.86114471163552	hqer_induced sampler prototype	4.0
	WDI_industry	Quantile-SFA	hqer_induced	True	16	-129.88374118120615	hqer_induced sampler prototype	2.0
	WDI_industry	HQER-SFA	hqer_induced	True	22	-129.79939388446763	hqer_induced sampler prototype	4.0
	WDI_industry	Quantile-SFA	hqer_induced	True	22	-132.30916148816556	hqer_induced sampler prototype	4.0
	WDI_industry	HQER-SFA	hqer_induced	True	20	-132.2621920609695	hqer_induced sampler prototype	4.0
	WDI_industry	Quantile-SFA	hqer_induced	True	31	-116.31923927463596	hqer_induced sampler prototype	2.0
	WDI_industry	HQER-SFA	hqer_induced	True	40	-116.27767874170802	hqer_induced sampler prototype	4.0
	WDI_industry	Quantile-SFA	hqer_induced	True	32	-110.3284152354505	hqer_induced sampler prototype	4.0
	WDI_industry	HQER-SFA	hqer_induced	True	25	-110.2739430555494	hqer_induced sampler prototype	4.0
	WDI_industry	Quantile-SFA	hqer_induced	True	17	-120.65836821872664	hqer_induced sampler prototype	4.0
	WDI_industry	HQER-SFA	hqer_induced	True	19	-120.62073431909216	hqer_induced sampler prototype	4.0
	WDI_industry	Quantile-SFA	hqer_induced	True	19	-151.07905713760005	hqer_induced sampler prototype	4.0
	WDI_industry	HQER-SFA	hqer_induced	True	21	-151.03250668986655	hqer_induced sampler prototype	2.0
	WDI_industry	Quantile-SFA	hqer_induced	True	24	-158.6747918388341	hqer_induced sampler prototype	4.0
	WDI_industry	HQER-SFA	hqer_induced	True	25	-158.62238111382703	hqer_induced sampler prototype	4.0
	WDI_industry	Quantile-SFA	hqer_induced	True	30	-142.91468433994507	hqer_induced sampler prototype	2.0
	WDI_industry	HQER-SFA	hqer_induced	True	24	-142.84817345473945	hqer_induced sampler prototype	4.0
	WDI_industry	Quantile-SFA	hqer_induced	True	26	-130.74247987908194	hqer_induced sampler prototype	4.0
	WDI_industry	HQER-SFA	hqer_induced	True	21	-130.70870164991808	hqer_induced sampler prototype	2.0
	WDI_industry	Quantile-SFA	hqer_induced	True	20	-150.48487071888945	hqer_induced sampler prototype	2.0
	WDI_industry	HQER-SFA	hqer_induced	True	14	-150.4229856750087	hqer_induced sampler prototype	2.0
	WDI_industry	Quantile-SFA	hqer_induced	True	20	-157.75690711506837	hqer_induced sampler prototype	4.0
	WDI_industry	HQER-SFA	hqer_induced	True	14	-157.69190982119324	hqer_induced sampler prototype	2.0
	WDI_industry	Quantile-SFA	hqer_induced	True	23	-143.6981932513334	hqer_induced sampler prototype	4.0
	WDI_industry	HQER-SFA	hqer_induced	True	27	-143.67058569887286	hqer_induced sampler prototype	4.0
	WDI_industry	Quantile-SFA	hqer_induced	True	16	-147.05047099271815	hqer_induced sampler prototype	4.0
	WDI_industry	HQER-SFA	hqer_induced	True	11	-147.00789809813233	hqer_induced sampler prototype	4.0
	WDI_industry	Quantile-SFA	hqer_induced	True	24	-144.8433487750113	hqer_induced sampler prototype	4.0

experiment	dataset	model	scenario	converged	n_iter	loglik	warning_message	best_start_id
	WDI_industry	HQER-SFA	hqer_induced	True	17	-144.86078523304235	hqer_induced sampler prototype	4.0
	WDI_industry	Quantile-SFA	hqer_induced	True	19	-175.4066870322008	hqer_induced sampler prototype	4.0
	WDI_industry	HQER-SFA	hqer_induced	True	19	-175.3349436745064	hqer_induced sampler prototype	2.0
	WDI_industry	Quantile-SFA	hqer_induced	True	29	-131.14331980024286	hqer_induced sampler prototype	4.0
	WDI_industry	HQER-SFA	hqer_induced	True	11	-131.12436925977462	hqer_induced sampler prototype	2.0
	WDI_industry	Quantile-SFA	hqer_induced	True	21	-145.78239095162246	hqer_induced sampler prototype	4.0
	WDI_industry	HQER-SFA	hqer_induced	True	24	-145.73217604854457	hqer_induced sampler prototype	4.0
	WDI_industry	Quantile-SFA	hqer_induced	True	21	-137.670237294427	hqer_induced sampler prototype	4.0
	WDI_industry	HQER-SFA	hqer_induced	True	24	-137.6024423362681	hqer_induced sampler prototype	4.0
	WDI_industry	Quantile-SFA	hqer_induced	True	24	-123.4434280141643	hqer_induced sampler prototype	4.0
	WDI_industry	HQER-SFA	hqer_induced	True	22	-123.41994338167672	hqer_induced sampler prototype	4.0
	WDI_industry	Quantile-SFA	hqer_induced	True	17	-152.86171663121445	hqer_induced sampler prototype	2.0
	WDI_industry	HQER-SFA	hqer_induced	True	32	-152.8392407606636	hqer_induced sampler prototype	4.0
	WDI_industry	Quantile-SFA	hqer_induced	True	20	-115.59336098546028	hqer_induced sampler prototype	2.0
	WDI_industry	HQER-SFA	hqer_induced	True	25	-115.56836909206535	hqer_induced sampler prototype	4.0
	WDI_industry	Quantile-SFA	hqer_induced	True	21	-127.52357061394788	hqer_induced sampler prototype	4.0
	WDI_industry	HQER-SFA	hqer_induced	True	13	-127.4496249780631	hqer_induced sampler prototype	2.0
	WDI_industry	Quantile-SFA	hqer_induced	True	17	-161.70518795929263	hqer_induced sampler prototype	4.0
	WDI_industry	HQER-SFA	hqer_induced	True	11	-161.6907546127207	hqer_induced sampler prototype	2.0
	WDI_industry	Quantile-SFA	hqer_induced	True	24	-126.0706550379016	hqer_induced sampler prototype	2.0
	WDI_industry	HQER-SFA	hqer_induced	True	22	-126.03266804728554	hqer_induced sampler prototype	2.0
	WDI_industry	Quantile-SFA	hqer_induced	True	27	-107.90032153111297	hqer_induced sampler prototype	4.0
	WDI_industry	HQER-SFA	hqer_induced	True	20	-107.90460594071624	hqer_induced sampler prototype	2.0
	WDI_industry	Quantile-SFA	hqer_induced	True	22	-105.49207428899746	hqer_induced sampler prototype	4.0
	WDI_industry	HQER-SFA	hqer_induced	True	21	-105.50497686185253	hqer_induced sampler prototype	2.0
	WDI_industry	Quantile-SFA	hqer_induced	True	26	-129.27801111827927	hqer_induced sampler prototype	4.0
	WDI_industry	HQER-SFA	hqer_induced	True	26	-129.25488154158745	hqer_induced sampler prototype	2.0
	WDI_industry	Quantile-SFA	hqer_induced	True	27	-111.6551649442526	hqer_induced sampler prototype	4.0
	WDI_industry	HQER-SFA	hqer_induced	True	22	-111.6356382053784	hqer_induced sampler prototype	4.0
	WDI_industry	Quantile-SFA	hqer_induced	True	28	-159.37701725857667	hqer_induced sampler prototype	2.0
	WDI_industry	HQER-SFA	hqer_induced	True	27	-159.33017183156852	hqer_induced sampler prototype	2.0
	WDI_industry	Quantile-SFA	hqer_induced	True	19	-113.7976768316538	hqer_induced sampler prototype	2.0
	WDI_industry	HQER-SFA	hqer_induced	True	20	-113.7560183716763	hqer_induced sampler prototype	2.0
	WDI_industry	Quantile-SFA	hqer_induced	True	28	-132.51507878023128	hqer_induced sampler prototype	4.0
	WDI_industry	HQER-SFA	hqer_induced	True	19	-132.47126442118912	hqer_induced sampler prototype	2.0
	WDI_industry	Quantile-SFA	hqer_induced	True	27	-129.7195266517517	hqer_induced sampler prototype	4.0
	WDI_industry	HQER-SFA	hqer_induced	True	12	-129.6680225741515	hqer_induced sampler prototype	2.0
	WDI_industry	Quantile-SFA	hqer_induced	True	42	-119.16556889300506	hqer_induced sampler prototype	2.0
	WDI_industry	HQER-SFA	hqer_induced	True	24	-119.09915338531609	hqer_induced sampler prototype	2.0
	WDI_industry	Quantile-SFA	hqer_induced	True	24	-141.28396815475242	hqer_induced sampler prototype	2.0
	WDI_industry	HQER-SFA	hqer_induced	True	23	-141.24026190068412	hqer_induced sampler prototype	2.0
	WDI_industry	Quantile-SFA	hqer_induced	True	24	-97.2531165153742	hqer_induced sampler prototype	2.0
	WDI_industry	HQER-SFA	hqer_induced	True	26	-97.22359193541524	hqer_induced sampler prototype	2.0
	WDI_industry	Quantile-SFA	hqer_induced	True	19	-158.42808881990794	hqer_induced sampler prototype	4.0
	WDI_industry	HQER-SFA	hqer_induced	True	19	-158.39969332597104	hqer_induced sampler prototype	4.0
	WDI_industry	Quantile-SFA	hqer_induced	True	28	-171.42025307808848	hqer_induced sampler prototype	4.0
	WDI_industry	HQER-SFA	hqer_induced	True	28	-171.35559405699493	hqer_induced sampler prototype	2.0
	WDI_industry	Quantile-SFA	hqer_induced	True	14	-129.76731990068876	hqer_induced sampler prototype	2.0
	WDI_industry	HQER-SFA	hqer_induced	True	17	-129.73933499928754	hqer_induced sampler prototype	2.0
	WDI_industry	Quantile-SFA	hqer_induced	True	17	-130.4838910686554	hqer_induced sampler prototype	4.0
	WDI_industry	HQER-SFA	hqer_induced	True	27	-130.45559654099543	hqer_induced sampler prototype	2.0
	WDI_industry	Quantile-SFA	hqer_induced	True	24	-124.63672973316216	hqer_induced sampler prototype	4.0
	WDI_industry	HQER-SFA	hqer_induced	True	21	-124.5828418054075	hqer_induced sampler prototype	2.0
	WDI_industry	Quantile-SFA	hqer_induced	True	20	-129.22011450839767	hqer_induced sampler prototype	4.0
	WDI_industry	HQER-SFA	hqer_induced	True	26	-129.167343732537	hqer_induced sampler prototype	2.0
	WDI_industry	Quantile-SFA	hqer_induced	True	20	-156.06920483793655	hqer_induced sampler prototype	4.0
	WDI_industry	HQER-SFA	hqer_induced	True	24	-156.04617975182896	hqer_induced sampler prototype	2.0
	WDI_industry	Quantile-SFA	hqer_induced	True	29	-102.03831772300876	hqer_induced sampler prototype	4.0
	WDI_industry	HQER-SFA	hqer_induced	True	23	-102.03200321179472	hqer_induced sampler prototype	4.0
	WDI_industry	Quantile-SFA	hqer_induced	True	22	-143.67100076506222	hqer_induced sampler prototype	2.0
	WDI_industry	HQER-SFA	hqer_induced	True	23	-143.62747308828574	hqer_induced sampler prototype	4.0
	WDI_industry	Quantile-SFA	hqer_induced	True	24	-126.92808292052884	hqer_induced sampler prototype	4.0
	WDI_industry	HQER-SFA	hqer_induced	True	25	-126.88553867885642	hqer_induced sampler prototype	4.0
	WDI_industry	Quantile-SFA	hqer_induced	True	25	-142.59172402937998	hqer_induced sampler prototype	2.0
	WDI_industry	HQER-SFA	hqer_induced	True	24	-142.52893741682786	hqer_induced sampler prototype	4.0
	WDI_industry	Quantile-SFA	hqer_induced	True	15	-151.2942343361413	hqer_induced sampler prototype	2.0
	WDI_industry	HQER-SFA	hqer_induced	True	21	-151.235407253047	hqer_induced sampler prototype	2.0
	WDI_industry	Quantile-SFA	hqer_induced	True	23	-126.6266422686534	hqer_induced sampler prototype	4.0
	WDI_industry	HQER-SFA	hqer_induced	True	28	-126.57431529439194	hqer_induced sampler prototype	2.0
	WDI_industry	Quantile-SFA	hqer_induced	True	44	-97.63591187037176	hqer_induced sampler prototype	5.0
	WDI_industry	HQER-SFA	hqer_induced	True	22	-97.58964793545124	hqer_induced sampler prototype	4.0
	WDI_industry	Quantile-SFA	hqer_induced	True	19	-144.69492904299878	hqer_induced sampler prototype	4.0
	WDI_industry	HQER-SFA	hqer_induced	True	29	-144.60200136500788	hqer_induced sampler prototype	2.0
	WDI_industry	Quantile-SFA	hqer_induced	True	26	-127.88377266360251	hqer_induced sampler prototype	2.0
	WDI_industry	HQER-SFA	hqer_induced	True	20	-127.83848353835204	hqer_induced sampler prototype	4.0
	WDI_industry	Quantile-SFA	hqer_induced	True	26	-128.5853130319799	hqer_induced sampler prototype	4.0
	WDI_industry	HQER-SFA	hqer_induced	True	19	-128.52501304450684	hqer_induced sampler prototype	2.0
	WDI_industry	Quantile-SFA	hqer_induced	True	28	-132.45130026494144	hqer_induced sampler prototype	4.0
	WDI_industry	HQER-SFA	hqer_induced	True	26	-132.42034176310742	hqer_induced sampler prototype	4.0
	WDI_industry	Quantile-SFA	hqer_induced	True	39	-140.78519594262207	hqer_induced sampler prototype	5.0
	WDI_industry	HQER-SFA	hqer_induced	True	44	-140.76764086205512	hqer_induced sampler prototype	5.0
	WDI_industry	Quantile-SFA	hqer_induced	True	11	-164.1534526067412	hqer_induced sampler prototype	2.0
	WDI_industry	HQER-SFA	hqer_induced	True	30	-164.0905655591082	hqer_induced sampler prototype	4.0
	WDI_industry	Quantile-SFA	hqer_induced	True	23	-143.50572315256	hqer_induced sampler prototype	4.0
	WDI_industry	HQER-SFA	hqer_induced	True	19	-143.4676984239008	hqer_induced sampler prototype	4.0
	WDI_industry	Quantile-SFA	hqer_induced	True	25	-150.60874870402674	hqer_induced sampler prototype	4.0
	WDI_industry	HQER-SFA	hqer_induced	True	21	-150.55947333558896	hqer_induced sampler prototype	4.0
	WDI_industry	Quantile-SFA	hqer_induced	True	38	-159.85554861408357	hqer_induced sampler prototype	4.0
	WDI_industry	HQER-SFA	hqer_induced	True	41	-159.81543951380385	hqer_induced sampler prototype	2.0
	WDI_industry	Quantile-SFA	hqer_induced	True	24	-126.23981485596808	hqer_induced sampler prototype	2.0
	WDI_industry	HQER-SFA	hqer_induced	True	27	-126.20330431718368	hqer_induced sampler prototype	2.0
	WDI_industry	Quantile-SFA	hqer_induced	True	20	-152.32991860340945	hqer_induced sampler prototype	4.0
	WDI_industry	HQER-SFA	hqer_induced	True	23	-152.3037153748005	hqer_induced sampler prototype	4.0
	WDI_industry	Quantile-SFA	hqer_induced	True	20	-127.16370046667896	hqer_induced sampler prototype	4.0
	WDI_industry	HQER-SFA	hqer_induced	True	24	-127.12538592600932	hqer_induced sampler prototype	2.0
	WDI_industry	Quantile-SFA	hqer_induced	True	24	-151.9011686431164	hqer_induced sampler prototype	2.0
	WDI_industry	HQER-SFA	hqer_induced	True	20	-151.8409673648107	hqer_induced sampler prototype	2.0
	WDI_industry	Quantile-SFA	hqer_induced	True	26	-116.55933253715348	hqer_induced sampler prototype	4.0
	WDI_industry	HQER-SFA	hqer_induced	True	23	-116.54712927450116	hqer_induced sampler prototype	4.0
	WDI_industry	Quantile-SFA	hqer_induced	True	23	-111.0357839392552	hqer_induced sampler prototype	4.0
	WDI_industry	HQER-SFA	hqer_induced	True	27	-110.9912434989565	hqer_induced sampler prototype	4.0
	WDI_industry	Quantile-SFA	hqer_induced	True	26	-125.45142099006134	hqer_induced sampler prototype	4.0
	WDI_industry	HQER-SFA	hqer_induced	True	19	-125.42072916052471	hqer_induced sampler prototype	4.0

experiment	dataset	model	scenario	converged	n__iter	loglik	warning__message	best__start__id
	WDI_industry	Quantile-SFA	hqer__induced	True	20	-140.4513027578619	hqer__induced sampler prototype	2.0
	WDI_industry	HQER-SFA	hqer__induced	True	12	-140.43037913263933	hqer__induced sampler prototype	4.0
	WDI_industry	Quantile-SFA	hqer__induced	True	24	-139.40843937082377	hqer__induced sampler prototype	4.0
	WDI_industry	HQER-SFA	hqer__induced	True	27	-139.37156648537825	hqer__induced sampler prototype	4.0
	WDI_industry	Quantile-SFA	hqer__induced	True	25	-175.82796201578262	hqer__induced sampler prototype	4.0
	WDI_industry	HQER-SFA	hqer__induced	True	24	-175.79209446181966	hqer__induced sampler prototype	4.0
	WDI_industry	Quantile-SFA	hqer__induced	True	22	-158.87224939739724	hqer__induced sampler prototype	4.0
	WDI_industry	HQER-SFA	hqer__induced	True	17	-158.84193689701584	hqer__induced sampler prototype	4.0
	WDI_industry	Quantile-SFA	hqer__induced	True	21	-123.02660829819824	hqer__induced sampler prototype	2.0
	WDI_industry	HQER-SFA	hqer__induced	True	25	-122.99009092134528	hqer__induced sampler prototype	2.0
	WDI_industry	Quantile-SFA	hqer__induced	True	15	-591.9955985207407	hqer__induced sampler prototype	2.0
	WDI_industry	HQER-SFA	hqer__induced	True	18	-591.776305693444	hqer__induced sampler prototype	2.0
	WDI_industry	Quantile-SFA	hqer__induced	True	14	-587.3922242102008	hqer__induced sampler prototype	2.0
	WDI_industry	HQER-SFA	hqer__induced	True	14	-587.1696813575181	hqer__induced sampler prototype	2.0
	WDI_industry	Quantile-SFA	hqer__induced	True	19	-559.3258474178274	hqer__induced sampler prototype	4.0
	WDI_industry	HQER-SFA	hqer__induced	True	25	-559.1540946023582	hqer__induced sampler prototype	4.0
	WDI_industry	Quantile-SFA	hqer__induced	True	25	-445.9714053249305	hqer__induced sampler prototype	2.0
	WDI_industry	HQER-SFA	hqer__induced	True	19	-445.8617753913138	hqer__induced sampler prototype	2.0
	WDI_industry	Quantile-SFA	hqer__induced	True	25	-527.1436061576267	hqer__induced sampler prototype	4.0
	WDI_industry	HQER-SFA	hqer__induced	True	21	-526.9652287281353	hqer__induced sampler prototype	4.0
	WDI_industry	Quantile-SFA	hqer__induced	True	21	-537.5223446424313	hqer__induced sampler prototype	2.0
	WDI_industry	HQER-SFA	hqer__induced	True	20	-537.3881544278327	hqer__induced sampler prototype	2.0
	WDI_industry	Quantile-SFA	hqer__induced	True	20	-496.3979155329467	hqer__induced sampler prototype	2.0
	WDI_industry	HQER-SFA	hqer__induced	True	18	-496.2860804658086	hqer__induced sampler prototype	4.0
	WDI_industry	Quantile-SFA	hqer__induced	True	14	-524.104740905402	hqer__induced sampler prototype	4.0
	WDI_industry	HQER-SFA	hqer__induced	True	13	-523.9351573269214	hqer__induced sampler prototype	2.0
	WDI_industry	Quantile-SFA	hqer__induced	True	20	-545.1020887748498	hqer__induced sampler prototype	4.0
	WDI_industry	HQER-SFA	hqer__induced	True	14	-544.9035930930419	hqer__induced sampler prototype	2.0
	WDI_industry	Quantile-SFA	hqer__induced	True	23	-517.9434258574715	hqer__induced sampler prototype	4.0
	WDI_industry	HQER-SFA	hqer__induced	True	31	-517.7399036932961	hqer__induced sampler prototype	2.0
	WDI_industry	Quantile-SFA	hqer__induced	True	19	-538.1750839832047	hqer__induced sampler prototype	4.0
	WDI_industry	HQER-SFA	hqer__induced	True	20	-537.9910440004645	hqer__induced sampler prototype	4.0
	WDI_industry	Quantile-SFA	hqer__induced	True	17	-574.8526337621718	hqer__induced sampler prototype	4.0
	WDI_industry	HQER-SFA	hqer__induced	True	14	-574.692592443187	hqer__induced sampler prototype	2.0
	WDI_industry	Quantile-SFA	hqer__induced	True	19	-483.609063108068	hqer__induced sampler prototype	4.0
	WDI_industry	HQER-SFA	hqer__induced	True	9	-483.3953253971935	hqer__induced sampler prototype	2.0
	WDI_industry	Quantile-SFA	hqer__induced	True	19	-567.3169448243807	hqer__induced sampler prototype	4.0
	WDI_industry	HQER-SFA	hqer__induced	True	16	-567.0863318374943	hqer__induced sampler prototype	4.0
	WDI_industry	Quantile-SFA	hqer__induced	True	11	-593.5385923934559	hqer__induced sampler prototype	2.0
	WDI_industry	HQER-SFA	hqer__induced	True	20	-593.3751906360583	hqer__induced sampler prototype	4.0
	WDI_industry	Quantile-SFA	hqer__induced	True	18	-544.2599457825943	hqer__induced sampler prototype	4.0
	WDI_industry	HQER-SFA	hqer__induced	True	21	-544.1004095510841	hqer__induced sampler prototype	4.0
	WDI_industry	Quantile-SFA	hqer__induced	True	24	-577.6353500311818	hqer__induced sampler prototype	4.0
	WDI_industry	HQER-SFA	hqer__induced	True	21	-577.4638965563726	hqer__induced sampler prototype	4.0
	WDI_industry	Quantile-SFA	hqer__induced	True	16	-582.9803449951814	hqer__induced sampler prototype	4.0
	WDI_industry	HQER-SFA	hqer__induced	True	31	-582.8301133526414	hqer__induced sampler prototype	5.0
	WDI_industry	Quantile-SFA	hqer__induced	True	17	-519.6239267596626	hqer__induced sampler prototype	4.0
	WDI_industry	HQER-SFA	hqer__induced	True	20	-519.4924141183374	hqer__induced sampler prototype	4.0
	WDI_industry	Quantile-SFA	hqer__induced	True	15	-550.1898938398039	hqer__induced sampler prototype	4.0
	WDI_industry	HQER-SFA	hqer__induced	True	16	-550.0836790107641	hqer__induced sampler prototype	4.0
	WDI_industry	Quantile-SFA	hqer__induced	True	17	-607.5012559858565	hqer__induced sampler prototype	2.0
	WDI_industry	HQER-SFA	hqer__induced	True	23	-607.2936067786488	hqer__induced sampler prototype	2.0
	WDI_industry	Quantile-SFA	hqer__induced	True	19	-547.456273512808	hqer__induced sampler prototype	4.0
	WDI_industry	HQER-SFA	hqer__induced	True	20	-547.3581831750267	hqer__induced sampler prototype	2.0
	WDI_industry	Quantile-SFA	hqer__induced	True	21	-493.5105042657736	hqer__induced sampler prototype	4.0
	WDI_industry	HQER-SFA	hqer__induced	True	18	-493.3575431523928	hqer__induced sampler prototype	4.0
	WDI_industry	Quantile-SFA	hqer__induced	True	25	-522.1418564188386	hqer__induced sampler prototype	4.0
	WDI_industry	HQER-SFA	hqer__induced	True	20	-522.0204781795887	hqer__induced sampler prototype	4.0
	WDI_industry	Quantile-SFA	hqer__induced	True	34	-547.7707901806705	hqer__induced sampler prototype	5.0
	WDI_industry	HQER-SFA	hqer__induced	True	20	-547.6276522449709	hqer__induced sampler prototype	4.0
	WDI_industry	Quantile-SFA	hqer__induced	True	27	-527.8335035211526	hqer__induced sampler prototype	4.0
	WDI_industry	HQER-SFA	hqer__induced	True	23	-527.5864439891004	hqer__induced sampler prototype	4.0
	WDI_industry	Quantile-SFA	hqer__induced	True	21	-566.7422883809766	hqer__induced sampler prototype	4.0
	WDI_industry	HQER-SFA	hqer__induced	True	12	-566.5418035450618	hqer__induced sampler prototype	2.0
	WDI_industry	Quantile-SFA	hqer__induced	True	21	-590.6128410185461	hqer__induced sampler prototype	4.0
	WDI_industry	HQER-SFA	hqer__induced	True	24	-590.4748502883241	hqer__induced sampler prototype	4.0
	WDI_industry	Quantile-SFA	hqer__induced	True	21	-608.4129351284853	hqer__induced sampler prototype	2.0
	WDI_industry	HQER-SFA	hqer__induced	True	21	-608.2616092660518	hqer__induced sampler prototype	4.0
	WDI_industry	Quantile-SFA	hqer__induced	True	22	-460.12093291686153	hqer__induced sampler prototype	2.0
	WDI_industry	HQER-SFA	hqer__induced	True	20	-459.9785198817549	hqer__induced sampler prototype	2.0
	WDI_industry	Quantile-SFA	hqer__induced	True	36	-530.6904359250311	hqer__induced sampler prototype	5.0
	WDI_industry	HQER-SFA	hqer__induced	True	20	-530.561789334008	hqer__induced sampler prototype	4.0
	WDI_industry	Quantile-SFA	hqer__induced	True	24	-519.7922554366197	hqer__induced sampler prototype	4.0
	WDI_industry	HQER-SFA	hqer__induced	True	17	-519.5868897803618	hqer__induced sampler prototype	2.0
	WDI_industry	Quantile-SFA	hqer__induced	True	16	-551.3083887040999	hqer__induced sampler prototype	4.0
	WDI_industry	HQER-SFA	hqer__induced	True	16	-551.1369636636516	hqer__induced sampler prototype	2.0
	WDI_industry	Quantile-SFA	hqer__induced	True	13	-542.4729434092819	hqer__induced sampler prototype	2.0
	WDI_industry	HQER-SFA	hqer__induced	True	22	-542.2872031314313	hqer__induced sampler prototype	4.0
	WDI_industry	Quantile-SFA	hqer__induced	True	20	-560.7360106652743	hqer__induced sampler prototype	4.0
	WDI_industry	HQER-SFA	hqer__induced	True	20	-560.557946944496	hqer__induced sampler prototype	4.0
	WDI_industry	Quantile-SFA	hqer__induced	True	23	-513.6610841466401	hqer__induced sampler prototype	2.0
	WDI_industry	HQER-SFA	hqer__induced	True	17	-513.5403781489122	hqer__induced sampler prototype	2.0
	WDI_industry	Quantile-SFA	hqer__induced	True	22	-593.3558333847329	hqer__induced sampler prototype	4.0
	WDI_industry	HQER-SFA	hqer__induced	True	20	-593.2279695376658	hqer__induced sampler prototype	4.0
	WDI_industry	Quantile-SFA	hqer__induced	True	19	-507.6410380995184	hqer__induced sampler prototype	4.0
	WDI_industry	HQER-SFA	hqer__induced	True	21	-507.52854172286345	hqer__induced sampler prototype	2.0
	WDI_industry	Quantile-SFA	hqer__induced	True	21	-577.2977035494587	hqer__induced sampler prototype	4.0
	WDI_industry	HQER-SFA	hqer__induced	True	20	-577.1109008380147	hqer__induced sampler prototype	4.0
	WDI_industry	Quantile-SFA	hqer__induced	True	15	-498.25534717823416	hqer__induced sampler prototype	4.0
	WDI_industry	HQER-SFA	hqer__induced	True	27	-498.0896897074524	hqer__induced sampler prototype	2.0
	WDI_industry	Quantile-SFA	hqer__induced	True	15	-563.3254285414681	hqer__induced sampler prototype	4.0
	WDI_industry	HQER-SFA	hqer__induced	True	32	-563.152227377437	hqer__induced sampler prototype	5.0
	WDI_industry	Quantile-SFA	hqer__induced	True	17	-558.6561763686199	hqer__induced sampler prototype	2.0
	WDI_industry	HQER-SFA	hqer__induced	True	18	-558.4785752242793	hqer__induced sampler prototype	2.0
	WDI_industry	Quantile-SFA	hqer__induced	True	15	-554.8631943526291	hqer__induced sampler prototype	2.0
	WDI_industry	HQER-SFA	hqer__induced	True	24	-554.6929515886807	hqer__induced sampler prototype	4.0
	WDI_industry	Quantile-SFA	hqer__induced	True	13	-602.9024913241356	hqer__induced sampler prototype	2.0
	WDI_industry	HQER-SFA	hqer__induced	True	13	-602.7191269358176	hqer__induced sampler prototype	2.0
	WDI_industry	Quantile-SFA	hqer__induced	True	22	-556.3584899329115	hqer__induced sampler prototype	4.0
	WDI_industry	HQER-SFA	hqer__induced	True	18	-556.2016534217769	hqer__induced sampler prototype	4.0
	WDI_industry	Quantile-SFA	hqer__induced	True	16	-552.1492009408878	hqer__induced sampler prototype	2.0
	WDI_industry	HQER-SFA	hqer__induced	True	19	-552.0287750958004	hqer__induced sampler prototype	2.0
	WDI_industry	Quantile-SFA	hqer__induced	True	12	-522.2213182780381	hqer__induced sampler prototype	2.0

experiment	dataset	model	scenario	converged	n__iter	loglik	warning__message	best__start__id
	WDI__industry	HQER-SFA	hqer__induced	True	21	-522.0102098281626	hqer__induced sampler prototype	2.0
	WDI__industry	Quantile-SFA	hqer__induced	True	9	-566.4461609459253	hqer__induced sampler prototype	2.0
	WDI__industry	HQER-SFA	hqer__induced	True	21	-566.2564285755053	hqer__induced sampler prototype	4.0
	WDI__industry	Quantile-SFA	hqer__induced	True	18	-592.3199379972923	hqer__induced sampler prototype	4.0
	WDI__industry	HQER-SFA	hqer__induced	True	16	-592.1382350700778	hqer__induced sampler prototype	2.0
	WDI__industry	Quantile-SFA	hqer__induced	True	20	-575.1043962436843	hqer__induced sampler prototype	2.0
	WDI__industry	HQER-SFA	hqer__induced	True	16	-575.0133835909578	hqer__induced sampler prototype	4.0
	WDI__industry	Quantile-SFA	hqer__induced	True	19	-483.8679197472124	hqer__induced sampler prototype	2.0
	WDI__industry	HQER-SFA	hqer__induced	True	20	-483.72386235489967	hqer__induced sampler prototype	4.0
	WDI__industry	Quantile-SFA	hqer__induced	True	21	-499.6232191600972	hqer__induced sampler prototype	2.0
	WDI__industry	HQER-SFA	hqer__induced	True	17	-499.41546106286114	hqer__induced sampler prototype	4.0
	WDI__industry	Quantile-SFA	hqer__induced	True	17	-570.6857015865928	hqer__induced sampler prototype	4.0
	WDI__industry	HQER-SFA	hqer__induced	True	14	-570.5758804242581	hqer__induced sampler prototype	2.0
	WDI__industry	Quantile-SFA	hqer__induced	True	21	-521.0187388537651	hqer__induced sampler prototype	4.0
	WDI__industry	HQER-SFA	hqer__induced	True	20	-520.8689571927498	hqer__induced sampler prototype	4.0
	WDI__industry	Quantile-SFA	hqer__induced	True	22	-518.1475822170136	hqer__induced sampler prototype	2.0
	WDI__industry	HQER-SFA	hqer__induced	True	19	-518.069824787576	hqer__induced sampler prototype	2.0
	WDI__industry	Quantile-SFA	hqer__induced	True	14	-543.2892131156846	hqer__induced sampler prototype	2.0
	WDI__industry	HQER-SFA	hqer__induced	True	14	-543.0520604064718	hqer__induced sampler prototype	2.0
	WDI__industry	Quantile-SFA	hqer__induced	True	24	-576.8282941315483	hqer__induced sampler prototype	4.0
	WDI__industry	HQER-SFA	hqer__induced	True	26	-576.6658854729278	hqer__induced sampler prototype	4.0
	WDI__industry	Quantile-SFA	hqer__induced	True	20	-530.1140650407256	hqer__induced sampler prototype	4.0
	WDI__industry	HQER-SFA	hqer__induced	True	16	-529.9234738098351	hqer__induced sampler prototype	2.0
	WDI__industry	Quantile-SFA	hqer__induced	True	18	-510.94193583242486	hqer__induced sampler prototype	2.0
	WDI__industry	HQER-SFA	hqer__induced	True	19	-510.8094841950537	hqer__induced sampler prototype	4.0
	WDI__industry	Quantile-SFA	hqer__induced	True	23	-613.0767245977141	hqer__induced sampler prototype	4.0
	WDI__industry	HQER-SFA	hqer__induced	True	18	-612.8236863124346	hqer__induced sampler prototype	4.0
	WDI__industry	Quantile-SFA	hqer__induced	True	18	-616.3535985407118	hqer__induced sampler prototype	2.0
	WDI__industry	HQER-SFA	hqer__induced	True	20	-616.1643358234132	hqer__induced sampler prototype	4.0
	WDI__industry	Quantile-SFA	hqer__induced	True	17	-447.0380854509969	hqer__induced sampler prototype	4.0
	WDI__industry	HQER-SFA	hqer__induced	True	20	-446.9005882246894	hqer__induced sampler prototype	4.0
	WDI__industry	Quantile-SFA	hqer__induced	True	15	-589.9912882426867	hqer__induced sampler prototype	2.0
	WDI__industry	HQER-SFA	hqer__induced	True	13	-589.8019803658913	hqer__induced sampler prototype	2.0
	WDI__industry	Quantile-SFA	hqer__induced	True	19	-554.4642312528333	hqer__induced sampler prototype	4.0
	WDI__industry	HQER-SFA	hqer__induced	True	15	-554.2859913773415	hqer__induced sampler prototype	2.0
	WDI__industry	Quantile-SFA	hqer__induced	True	16	-578.5674083943609	hqer__induced sampler prototype	2.0
	WDI__industry	HQER-SFA	hqer__induced	True	17	-578.4255740948506	hqer__induced sampler prototype	2.0
	WDI__industry	Quantile-SFA	hqer__induced	True	22	-557.7107105002653	hqer__induced sampler prototype	4.0
	WDI__industry	HQER-SFA	hqer__induced	True	21	-557.6168407773636	hqer__induced sampler prototype	4.0
	WDI__industry	Quantile-SFA	hqer__induced	True	25	-623.3945003919791	hqer__induced sampler prototype	4.0
	WDI__industry	HQER-SFA	hqer__induced	True	22	-623.2964403474489	hqer__induced sampler prototype	4.0
	WDI__industry	Quantile-SFA	hqer__induced	True	18	-522.679540365485	hqer__induced sampler prototype	4.0
	WDI__industry	HQER-SFA	hqer__induced	True	21	-522.5043776794508	hqer__induced sampler prototype	4.0
	WDI__industry	Quantile-SFA	hqer__induced	True	21	-481.348851247012	hqer__induced sampler prototype	4.0
	WDI__industry	HQER-SFA	hqer__induced	True	22	-481.1612575151922	hqer__induced sampler prototype	4.0
	WDI__industry	Quantile-SFA	hqer__induced	True	17	-590.1658491145358	hqer__induced sampler prototype	4.0
	WDI__industry	HQER-SFA	hqer__induced	True	18	-589.9365559561331	hqer__induced sampler prototype	4.0
	WDI__industry	Quantile-SFA	hqer__induced	True	16	-569.4052125611653	hqer__induced sampler prototype	2.0
	WDI__industry	HQER-SFA	hqer__induced	True	19	-569.2071843367025	hqer__induced sampler prototype	2.0
	WDI__industry	Quantile-SFA	hqer__induced	True	17	-526.2659579607068	hqer__induced sampler prototype	4.0
	WDI__industry	HQER-SFA	hqer__induced	True	17	-526.1821343839615	hqer__induced sampler prototype	4.0
	WDI__industry	Quantile-SFA	hqer__induced	True	14	-531.2423666109152	hqer__induced sampler prototype	4.0
	WDI__industry	HQER-SFA	hqer__induced	True	18	-531.1002766652541	hqer__induced sampler prototype	2.0
	WDI__industry	Quantile-SFA	hqer__induced	True	15	-478.0229114049546	hqer__induced sampler prototype	4.0
	WDI__industry	HQER-SFA	hqer__induced	True	23	-477.9097511904789	hqer__induced sampler prototype	2.0
	WDI__industry	Quantile-SFA	hqer__induced	True	25	-524.9736082740152	hqer__induced sampler prototype	4.0
	WDI__industry	HQER-SFA	hqer__induced	True	18	-524.7768055288373	hqer__induced sampler prototype	4.0
	WDI__industry	Quantile-SFA	hqer__induced	True	25	-483.51732974916	hqer__induced sampler prototype	4.0
	WDI__industry	HQER-SFA	hqer__induced	True	19	-483.3638655725786	hqer__induced sampler prototype	4.0
	WDI__industry	Quantile-SFA	hqer__induced	True	11	-506.6175730477282	hqer__induced sampler prototype	4.0
	WDI__industry	HQER-SFA	hqer__induced	True	14	-506.4783350154967	hqer__induced sampler prototype	2.0
	WDI__industry	Quantile-SFA	hqer__induced	True	19	-594.4592855697297	hqer__induced sampler prototype	2.0
	WDI__industry	HQER-SFA	hqer__induced	True	20	-594.2863285087218	hqer__induced sampler prototype	4.0
	WDI__industry	Quantile-SFA	hqer__induced	True	14	-557.593782992074	hqer__induced sampler prototype	2.0
	WDI__industry	HQER-SFA	hqer__induced	True	21	-557.4553583360645	hqer__induced sampler prototype	4.0
	WDI__industry	Quantile-SFA	hqer__induced	True	21	-545.5797148604349	hqer__induced sampler prototype	4.0
	WDI__industry	HQER-SFA	hqer__induced	True	20	-545.4665770477709	hqer__induced sampler prototype	4.0
	WDI__industry	Quantile-SFA	hqer__induced	True	19	-537.9920430115596	hqer__induced sampler prototype	4.0
	WDI__industry	HQER-SFA	hqer__induced	True	17	-537.8471141547806	hqer__induced sampler prototype	4.0
	WDI__industry	Quantile-SFA	hqer__induced	True	21	-521.068304929086	hqer__induced sampler prototype	4.0
	WDI__industry	HQER-SFA	hqer__induced	True	28	-520.8494752801112	hqer__induced sampler prototype	2.0
	WDI__industry	Quantile-SFA	hqer__induced	True	17	-552.3498189645104	hqer__induced sampler prototype	2.0
	WDI__industry	HQER-SFA	hqer__induced	True	25	-552.1684901490898	hqer__induced sampler prototype	4.0
	WDI__industry	Quantile-SFA	hqer__induced	True	10	-506.237068555687	hqer__induced sampler prototype	2.0
	WDI__industry	HQER-SFA	hqer__induced	True	21	-506.0401121337451	hqer__induced sampler prototype	4.0
	WDI__industry	Quantile-SFA	hqer__induced	True	22	-546.7327792606463	hqer__induced sampler prototype	2.0
	WDI__industry	HQER-SFA	hqer__induced	True	20	-546.6054419104444	hqer__induced sampler prototype	4.0
	WDI__industry	Quantile-SFA	hqer__induced	True	20	-496.3351521682595	hqer__induced sampler prototype	4.0
	WDI__industry	HQER-SFA	hqer__induced	True	21	-496.148836213058	hqer__induced sampler prototype	4.0
	WDI__industry	Quantile-SFA	hqer__induced	True	20	-513.1933882964408	hqer__induced sampler prototype	2.0
	WDI__industry	HQER-SFA	hqer__induced	True	19	-513.0375778090003	hqer__induced sampler prototype	2.0
	WDI__industry	Quantile-SFA	hqer__induced	True	19	-546.2621482998725	hqer__induced sampler prototype	4.0
	WDI__industry	HQER-SFA	hqer__induced	True	23	-546.0793807828907	hqer__induced sampler prototype	2.0
	WDI__industry	Quantile-SFA	hqer__induced	True	21	-565.8117462834973	hqer__induced sampler prototype	2.0
	WDI__industry	HQER-SFA	hqer__induced	True	19	-565.6543333413715	hqer__induced sampler prototype	4.0
	WDI__industry	Quantile-SFA	hqer__induced	True	18	-540.1316604678397	hqer__induced sampler prototype	2.0
	WDI__industry	HQER-SFA	hqer__induced	True	19	-540.0232255934028	hqer__induced sampler prototype	2.0
	WDI__industry	Quantile-SFA	hqer__induced	True	20	-607.70284659369	hqer__induced sampler prototype	4.0
	WDI__industry	HQER-SFA	hqer__induced	True	19	-607.5055331897088	hqer__induced sampler prototype	4.0
	WDI__industry	Quantile-SFA	hqer__induced	True	20	-459.0586044192203	hqer__induced sampler prototype	4.0
	WDI__industry	HQER-SFA	hqer__induced	True	39	-458.9542136870957	hqer__induced sampler prototype	5.0
	WDI__industry	Quantile-SFA	hqer__induced	True	20	-474.4400971165575	hqer__induced sampler prototype	4.0
	WDI__industry	HQER-SFA	hqer__induced	True	23	-474.2202342880743	hqer__induced sampler prototype	4.0
	WDI__industry	Quantile-SFA	hqer__induced	True	23	-588.902522719477	hqer__induced sampler prototype	4.0
	WDI__industry	HQER-SFA	hqer__induced	True	23	-588.7115768714305	hqer__induced sampler prototype	4.0
	WDI__industry	Quantile-SFA	hqer__induced	True	17	-475.4594508457475	hqer__induced sampler prototype	2.0
	WDI__industry	HQER-SFA	hqer__induced	True	11	-475.31249996810766	hqer__induced sampler prototype	4.0
	WDI__industry	Quantile-SFA	hqer__induced	True	21	-584.2007955085564	hqer__induced sampler prototype	4.0
	WDI__industry	HQER-SFA	hqer__induced	True	11	-584.0631115397608	hqer__induced sampler prototype	2.0
	WDI__industry	Quantile-SFA	hqer__induced	True	11	-563.6000732753424	hqer__induced sampler prototype	2.0
	WDI__industry	HQER-SFA	hqer__induced	True	9	-563.5293642129055	hqer__induced sampler prototype	2.0
	WDI__industry	Quantile-SFA	hqer__induced	True	18	-563.8231804332627	hqer__induced sampler prototype	4.0
	WDI__industry	HQER-SFA	hqer__induced	True	13	-563.5739005652823	hqer__induced sampler prototype	2.0

experiment	dataset	model	scenario	converged	n__iter	loglik	warning__message	best__start__id
	WDI__industry	Quantile-SFA	hqer__induced	True	14	-542.1512748998991	hqer__induced sampler prototype	2.0
	WDI__industry	HQER-SFA	hqer__induced	True	19	-541.9667856155755	hqer__induced sampler prototype	4.0
	WDI__industry	Quantile-SFA	hqer__induced	True	18	-502.6212174471348	hqer__induced sampler prototype	2.0
	WDI__industry	HQER-SFA	hqer__induced	True	23	-502.4245370307808	hqer__induced sampler prototype	2.0
	WDI__industry	Quantile-SFA	hqer__induced	True	25	-131.16156784637337	hqer__induced sampler prototype	4.0
	WDI__industry	HQER-SFA	hqer__induced	True	23	-126.32070383875144	hqer__induced sampler prototype	4.0
	WDI__industry	Quantile-SFA	hqer__induced	True	29	-163.0575476434941	hqer__induced sampler prototype	2.0
	WDI__industry	HQER-SFA	hqer__induced	True	28	-160.28132130145383	hqer__induced sampler prototype	2.0
	WDI__industry	Quantile-SFA	hqer__induced	True	31	-138.97446956445427	hqer__induced sampler prototype	4.0
	WDI__industry	HQER-SFA	hqer__induced	True	20	-137.48957578089176	hqer__induced sampler prototype	2.0
	WDI__industry	Quantile-SFA	hqer__induced	True	18	-135.046327061239	hqer__induced sampler prototype	4.0
	WDI__industry	HQER-SFA	hqer__induced	True	24	-134.47155982672984	hqer__induced sampler prototype	2.0
	WDI__industry	Quantile-SFA	hqer__induced	True	12	-170.57558586946374	hqer__induced sampler prototype	4.0
	WDI__industry	HQER-SFA	hqer__induced	True	19	-168.89304769409398	hqer__induced sampler prototype	2.0
	WDI__industry	Quantile-SFA	hqer__induced	True	24	-155.95607569121267	hqer__induced sampler prototype	4.0
	WDI__industry	HQER-SFA	hqer__induced	True	18	-149.53090322454509	hqer__induced sampler prototype	4.0
	WDI__industry	Quantile-SFA	hqer__induced	True	13	-164.4934283907833	hqer__induced sampler prototype	4.0
	WDI__industry	HQER-SFA	hqer__induced	True	18	-161.81829879740215	hqer__induced sampler prototype	4.0
	WDI__industry	Quantile-SFA	hqer__induced	True	24	-171.19868331325358	hqer__induced sampler prototype	2.0
	WDI__industry	HQER-SFA	hqer__induced	True	19	-168.22228391924966	hqer__induced sampler prototype	4.0
	WDI__industry	Quantile-SFA	hqer__induced	True	22	-123.71441272115672	hqer__induced sampler prototype	2.0
	WDI__industry	HQER-SFA	hqer__induced	True	25	-121.06427609870993	hqer__induced sampler prototype	4.0
	WDI__industry	Quantile-SFA	hqer__induced	True	20	-156.72068598055876	hqer__induced sampler prototype	2.0
	WDI__industry	HQER-SFA	hqer__induced	True	12	-156.1127107281465	hqer__induced sampler prototype	2.0
	WDI__industry	Quantile-SFA	hqer__induced	True	23	-112.95417675003164	hqer__induced sampler prototype	4.0
	WDI__industry	HQER-SFA	hqer__induced	True	24	-110.97831429383892	hqer__induced sampler prototype	4.0
	WDI__industry	Quantile-SFA	hqer__induced	True	25	-151.1572952837329	hqer__induced sampler prototype	2.0
	WDI__industry	HQER-SFA	hqer__induced	True	19	-148.72273026039983	hqer__induced sampler prototype	4.0
	WDI__industry	Quantile-SFA	hqer__induced	True	19	-139.61805932020553	hqer__induced sampler prototype	2.0
	WDI__industry	HQER-SFA	hqer__induced	True	26	-137.8563401984267	hqer__induced sampler prototype	2.0
	WDI__industry	Quantile-SFA	hqer__induced	True	28	-158.94157074379314	hqer__induced sampler prototype	4.0
	WDI__industry	HQER-SFA	hqer__induced	True	18	-156.43014734678917	hqer__induced sampler prototype	4.0
	WDI__industry	Quantile-SFA	hqer__induced	True	23	-136.6985869490639	hqer__induced sampler prototype	2.0
	WDI__industry	HQER-SFA	hqer__induced	True	19	-136.1230812988881	hqer__induced sampler prototype	2.0
	WDI__industry	Quantile-SFA	hqer__induced	True	29	-188.10506208183745	hqer__induced sampler prototype	4.0
	WDI__industry	HQER-SFA	hqer__induced	True	23	-186.5678285160751	hqer__induced sampler prototype	2.0
	WDI__industry	Quantile-SFA	hqer__induced	True	15	-126.93294256524428	hqer__induced sampler prototype	4.0
	WDI__industry	HQER-SFA	hqer__induced	True	25	-124.41016085148073	hqer__induced sampler prototype	4.0
	WDI__industry	Quantile-SFA	hqer__induced	True	14	-177.5379893238082	hqer__induced sampler prototype	2.0
	WDI__industry	HQER-SFA	hqer__induced	True	26	-174.94649925228998	hqer__induced sampler prototype	4.0
	WDI__industry	Quantile-SFA	hqer__induced	True	23	-124.47175128353352	hqer__induced sampler prototype	4.0
	WDI__industry	HQER-SFA	hqer__induced	True	15	-122.6718079064466	hqer__induced sampler prototype	2.0
	WDI__industry	Quantile-SFA	hqer__induced	True	26	-179.9443623747825	hqer__induced sampler prototype	4.0
	WDI__industry	HQER-SFA	hqer__induced	True	21	-177.02872639046376	hqer__induced sampler prototype	4.0
	WDI__industry	Quantile-SFA	hqer__induced	True	24	-180.5100078857316	hqer__induced sampler prototype	4.0
	WDI__industry	HQER-SFA	hqer__induced	True	26	-176.57740390617568	hqer__induced sampler prototype	4.0
	WDI__industry	Quantile-SFA	hqer__induced	True	29	-177.7717362678539	hqer__induced sampler prototype	4.0
	WDI__industry	HQER-SFA	hqer__induced	True	20	-173.6181418528949	hqer__induced sampler prototype	4.0
	WDI__industry	Quantile-SFA	hqer__induced	True	34	-184.2036671740532	hqer__induced sampler prototype	2.0
	WDI__industry	HQER-SFA	hqer__induced	True	25	-177.23496162046348	hqer__induced sampler prototype	4.0
	WDI__industry	Quantile-SFA	hqer__induced	True	27	-171.6768246415844	hqer__induced sampler prototype	2.0
	WDI__industry	HQER-SFA	hqer__induced	True	19	-169.31432139872277	hqer__induced sampler prototype	4.0
	WDI__industry	Quantile-SFA	hqer__induced	True	18	-108.99878901467478	hqer__induced sampler prototype	2.0
	WDI__industry	HQER-SFA	hqer__induced	True	10	-109.89694409226418	hqer__induced sampler prototype	2.0
	WDI__industry	Quantile-SFA	hqer__induced	True	25	-178.1851016068784	hqer__induced sampler prototype	2.0
	WDI__industry	HQER-SFA	hqer__induced	True	27	-175.01639981522396	hqer__induced sampler prototype	4.0
	WDI__industry	Quantile-SFA	hqer__induced	True	15	-175.94665584557248	hqer__induced sampler prototype	4.0
	WDI__industry	HQER-SFA	hqer__induced	True	13	-173.73164303196057	hqer__induced sampler prototype	4.0
	WDI__industry	Quantile-SFA	hqer__induced	True	19	-134.88216053917768	hqer__induced sampler prototype	4.0
	WDI__industry	HQER-SFA	hqer__induced	True	25	-132.00590126672247	hqer__induced sampler prototype	4.0
	WDI__industry	Quantile-SFA	hqer__induced	True	24	-117.83638757949171	hqer__induced sampler prototype	2.0
	WDI__industry	HQER-SFA	hqer__induced	True	25	-118.71683578986308	hqer__induced sampler prototype	2.0
	WDI__industry	Quantile-SFA	hqer__induced	True	22	-140.1837542373118	hqer__induced sampler prototype	4.0
	WDI__industry	HQER-SFA	hqer__induced	True	9	-138.7381793611673	hqer__induced sampler prototype	2.0
	WDI__industry	Quantile-SFA	hqer__induced	True	23	-137.84810952409583	hqer__induced sampler prototype	4.0
	WDI__industry	HQER-SFA	hqer__induced	True	16	-137.3551221672155	hqer__induced sampler prototype	2.0
	WDI__industry	Quantile-SFA	hqer__induced	True	27	-162.07117174083558	hqer__induced sampler prototype	2.0
	WDI__industry	HQER-SFA	hqer__induced	True	7	-160.38799082749375	hqer__induced sampler prototype	2.0
	WDI__industry	Quantile-SFA	hqer__induced	True	25	-119.1219271374704	hqer__induced sampler prototype	2.0
	WDI__industry	HQER-SFA	hqer__induced	True	13	-118.84100388233684	hqer__induced sampler prototype	2.0
	WDI__industry	Quantile-SFA	hqer__induced	True	28	-146.94752681685148	hqer__induced sampler prototype	4.0
	WDI__industry	HQER-SFA	hqer__induced	True	21	-144.20744281927412	hqer__induced sampler prototype	4.0
	WDI__industry	Quantile-SFA	hqer__induced	True	25	-153.11981273459847	hqer__induced sampler prototype	2.0
	WDI__industry	HQER-SFA	hqer__induced	True	23	-150.40360266184106	hqer__induced sampler prototype	4.0
	WDI__industry	Quantile-SFA	hqer__induced	True	11	-154.75547521369916	hqer__induced sampler prototype	2.0
	WDI__industry	HQER-SFA	hqer__induced	True	8	-152.79794992048127	hqer__induced sampler prototype	2.0
	WDI__industry	Quantile-SFA	hqer__induced	True	17	-181.5782358421665	hqer__induced sampler prototype	2.0
	WDI__industry	HQER-SFA	hqer__induced	True	12	-180.2988175841101	hqer__induced sampler prototype	2.0
	WDI__industry	Quantile-SFA	hqer__induced	True	20	-176.35260401079296	hqer__induced sampler prototype	4.0
	WDI__industry	HQER-SFA	hqer__induced	True	20	-176.1107821911852	hqer__induced sampler prototype	2.0
	WDI__industry	Quantile-SFA	hqer__induced	True	22	-138.1641352490638	hqer__induced sampler prototype	2.0
	WDI__industry	HQER-SFA	hqer__induced	True	29	-132.75152912457378	hqer__induced sampler prototype	4.0
	WDI__industry	Quantile-SFA	hqer__induced	True	17	-170.9125751860534	hqer__induced sampler prototype	2.0
	WDI__industry	HQER-SFA	hqer__induced	True	10	-168.84985116860253	hqer__induced sampler prototype	4.0
	WDI__industry	Quantile-SFA	hqer__induced	True	26	-119.50285837736232	hqer__induced sampler prototype	2.0
	WDI__industry	HQER-SFA	hqer__induced	True	25	-117.19043049556696	hqer__induced sampler prototype	2.0
	WDI__industry	Quantile-SFA	hqer__induced	True	10	-164.47134441722363	hqer__induced sampler prototype	2.0
	WDI__industry	HQER-SFA	hqer__induced	True	26	-160.20297960408635	hqer__induced sampler prototype	4.0
	WDI__industry	Quantile-SFA	hqer__induced	True	26	-178.86324248744083	hqer__induced sampler prototype	2.0
	WDI__industry	HQER-SFA	hqer__induced	True	22	-176.20122948812903	hqer__induced sampler prototype	4.0
	WDI__industry	Quantile-SFA	hqer__induced	True	22	-191.41988164507376	hqer__induced sampler prototype	4.0
	WDI__industry	HQER-SFA	hqer__induced	True	26	-189.8869091309564	hqer__induced sampler prototype	2.0
	WDI__industry	Quantile-SFA	hqer__induced	True	24	-196.8665683801995	hqer__induced sampler prototype	4.0
	WDI__industry	HQER-SFA	hqer__induced	True	26	-192.2086820518539	hqer__induced sampler prototype	4.0
	WDI__industry	Quantile-SFA	hqer__induced	True	16	-144.0411826329948	hqer__induced sampler prototype	2.0
	WDI__industry	HQER-SFA	hqer__induced	True	20	-144.66582575501155	hqer__induced sampler prototype	2.0
	WDI__industry	Quantile-SFA	hqer__induced	True	23	-181.8117058626329	hqer__induced sampler prototype	4.0
	WDI__industry	HQER-SFA	hqer__induced	True	22	-178.5235039171706	hqer__induced sampler prototype	4.0
	WDI__industry	Quantile-SFA	hqer__induced	True	26	-130.90983949480272	hqer__induced sampler prototype	4.0
	WDI__industry	HQER-SFA	hqer__induced	True	27	-129.93586109365992	hqer__induced sampler prototype	2.0
	WDI__industry	Quantile-SFA	hqer__induced	True	20	-166.23725688231792	hqer__induced sampler prototype	4.0
	WDI__industry	HQER-SFA	hqer__induced	True	27	-165.51136319952417	hqer__induced sampler prototype	4.0
	WDI__industry	Quantile-SFA	hqer__induced	True	25	-161.39186011198487	hqer__induced sampler prototype	4.0

experiment	dataset	model	scenario	converged	n__iter	loglik	warning__message	best__start__id
	WDI__industry	HQER-SFA	hqer__induced	True	13	-160.22303529716638	hqer__induced sampler prototype	2.0
	WDI__industry	Quantile-SFA	hqer__induced	True	21	-180.16189011440903	hqer__induced sampler prototype	4.0
	WDI__industry	HQER-SFA	hqer__induced	True	14	-176.7054623935563	hqer__induced sampler prototype	4.0
	WDI__industry	Quantile-SFA	hqer__induced	True	25	-113.26172315931908	hqer__induced sampler prototype	4.0
	WDI__industry	HQER-SFA	hqer__induced	True	14	-113.05871760240802	hqer__induced sampler prototype	2.0
	WDI__industry	Quantile-SFA	hqer__induced	True	18	-140.58992218823195	hqer__induced sampler prototype	2.0
	WDI__industry	HQER-SFA	hqer__induced	True	21	-139.10386159674206	hqer__induced sampler prototype	2.0
	WDI__industry	Quantile-SFA	hqer__induced	True	10	-158.9727793912392	hqer__induced sampler prototype	2.0
	WDI__industry	HQER-SFA	hqer__induced	True	10	-157.93154732178934	hqer__induced sampler prototype	2.0
	WDI__industry	Quantile-SFA	hqer__induced	True	27	-111.15241106416886	hqer__induced sampler prototype	2.0
	WDI__industry	HQER-SFA	hqer__induced	True	17	-110.01386404413172	hqer__induced sampler prototype	2.0
	WDI__industry	Quantile-SFA	hqer__induced	True	18	-168.9626383745794	hqer__induced sampler prototype	4.0
	WDI__industry	HQER-SFA	hqer__induced	True	18	-166.73040224251014	hqer__induced sampler prototype	4.0
	WDI__industry	Quantile-SFA	hqer__induced	True	20	-144.3441042070273	hqer__induced sampler prototype	2.0
	WDI__industry	HQER-SFA	hqer__induced	True	22	-142.456195675316	hqer__induced sampler prototype	2.0
	WDI__industry	Quantile-SFA	hqer__induced	True	21	-171.45104843403266	hqer__induced sampler prototype	2.0
	WDI__industry	HQER-SFA	hqer__induced	True	23	-169.36065942537454	hqer__induced sampler prototype	2.0
	WDI__industry	Quantile-SFA	hqer__induced	True	23	-133.15197114345844	hqer__induced sampler prototype	2.0
	WDI__industry	HQER-SFA	hqer__induced	True	25	-130.92073070638548	hqer__induced sampler prototype	4.0
	WDI__industry	Quantile-SFA	hqer__induced	True	19	-153.39326033292065	hqer__induced sampler prototype	2.0
	WDI__industry	HQER-SFA	hqer__induced	True	27	-151.3694729049196	hqer__induced sampler prototype	2.0
	WDI__industry	Quantile-SFA	hqer__induced	True	28	-139.7230978739413	hqer__induced sampler prototype	4.0
	WDI__industry	HQER-SFA	hqer__induced	True	20	-135.34773193034871	hqer__induced sampler prototype	2.0
	WDI__industry	Quantile-SFA	hqer__induced	True	28	-148.5171631264422	hqer__induced sampler prototype	4.0
	WDI__industry	HQER-SFA	hqer__induced	True	32	-145.5466378665726	hqer__induced sampler prototype	4.0
	WDI__industry	Quantile-SFA	hqer__induced	True	20	-164.68156968107667	hqer__induced sampler prototype	2.0
	WDI__industry	HQER-SFA	hqer__induced	True	31	-160.34279409762888	hqer__induced sampler prototype	4.0
	WDI__industry	Quantile-SFA	hqer__induced	True	19	-141.58365866132254	hqer__induced sampler prototype	4.0
	WDI__industry	HQER-SFA	hqer__induced	True	19	-137.8318003280868	hqer__induced sampler prototype	4.0
	WDI__industry	Quantile-SFA	hqer__induced	True	17	-140.30596183657667	hqer__induced sampler prototype	2.0
	WDI__industry	HQER-SFA	hqer__induced	True	24	-140.12892351885478	hqer__induced sampler prototype	2.0
	WDI__industry	Quantile-SFA	hqer__induced	True	20	-160.9831350454681	hqer__induced sampler prototype	2.0
	WDI__industry	HQER-SFA	hqer__induced	True	25	-158.5093439210629	hqer__induced sampler prototype	4.0
	WDI__industry	Quantile-SFA	hqer__induced	True	26	-166.59258589501331	hqer__induced sampler prototype	2.0
	WDI__industry	HQER-SFA	hqer__induced	True	16	-162.07113882825848	hqer__induced sampler prototype	4.0
	WDI__industry	Quantile-SFA	hqer__induced	True	16	-172.52052099533424	hqer__induced sampler prototype	2.0
	WDI__industry	HQER-SFA	hqer__induced	True	24	-168.62516811073948	hqer__induced sampler prototype	4.0
	WDI__industry	Quantile-SFA	hqer__induced	True	19	-142.5405248613298	hqer__induced sampler prototype	4.0
	WDI__industry	HQER-SFA	hqer__induced	True	24	-137.6545709552916	hqer__induced sampler prototype	4.0
	WDI__industry	Quantile-SFA	hqer__induced	True	15	-138.50108801298066	hqer__induced sampler prototype	2.0
	WDI__industry	HQER-SFA	hqer__induced	True	18	-136.37249412186165	hqer__induced sampler prototype	2.0
	WDI__industry	Quantile-SFA	hqer__induced	True	16	-139.501531615715	hqer__induced sampler prototype	2.0
	WDI__industry	HQER-SFA	hqer__induced	True	25	-137.739017583225	hqer__induced sampler prototype	2.0
	WDI__industry	Quantile-SFA	hqer__induced	True	25	-181.17466161791447	hqer__induced sampler prototype	4.0
	WDI__industry	HQER-SFA	hqer__induced	True	9	-181.57732010167567	hqer__induced sampler prototype	2.0
	WDI__industry	Quantile-SFA	hqer__induced	True	24	-155.73873871914955	hqer__induced sampler prototype	4.0
	WDI__industry	HQER-SFA	hqer__induced	True	24	-153.18583481198888	hqer__induced sampler prototype	4.0
	WDI__industry	Quantile-SFA	hqer__induced	True	25	-148.17331419350083	hqer__induced sampler prototype	4.0
	WDI__industry	HQER-SFA	hqer__induced	True	13	-147.70845545764047	hqer__induced sampler prototype	2.0
	WDI__industry	Quantile-SFA	hqer__induced	True	21	-162.50533989266947	hqer__induced sampler prototype	4.0
	WDI__industry	HQER-SFA	hqer__induced	True	19	-156.7597045482812	hqer__induced sampler prototype	4.0
	WDI__industry	Quantile-SFA	hqer__induced	True	23	-163.59655409784114	hqer__induced sampler prototype	4.0
	WDI__industry	HQER-SFA	hqer__induced	True	19	-160.65745421723423	hqer__induced sampler prototype	4.0
	WDI__industry	Quantile-SFA	hqer__induced	True	21	-162.7707658484051	hqer__induced sampler prototype	2.0
	WDI__industry	HQER-SFA	hqer__induced	True	20	-160.90203539904883	hqer__induced sampler prototype	2.0
	WDI__industry	Quantile-SFA	hqer__induced	True	27	-165.005254004819	hqer__induced sampler prototype	4.0
	WDI__industry	HQER-SFA	hqer__induced	True	24	-163.39934709891332	hqer__induced sampler prototype	2.0
	WDI__industry	Quantile-SFA	hqer__induced	True	22	-148.14739973401444	hqer__induced sampler prototype	2.0
	WDI__industry	HQER-SFA	hqer__induced	True	24	-146.81089532413503	hqer__induced sampler prototype	2.0
	WDI__industry	Quantile-SFA	hqer__induced	True	29	-176.4730475293918	hqer__induced sampler prototype	4.0
	WDI__industry	HQER-SFA	hqer__induced	True	16	-174.30165385749353	hqer__induced sampler prototype	2.0
	WDI__industry	Quantile-SFA	hqer__induced	True	30	-168.38700022914406	hqer__induced sampler prototype	4.0
	WDI__industry	HQER-SFA	hqer__induced	True	26	-166.3184702573364	hqer__induced sampler prototype	4.0
	WDI__industry	Quantile-SFA	hqer__induced	True	30	-171.86044660817427	hqer__induced sampler prototype	4.0
	WDI__industry	HQER-SFA	hqer__induced	True	28	-168.73736019215517	hqer__induced sampler prototype	4.0
	WDI__industry	Quantile-SFA	hqer__induced	True	12	-179.0826954591065	hqer__induced sampler prototype	2.0
	WDI__industry	HQER-SFA	hqer__induced	True	22	-176.33896118694423	hqer__induced sampler prototype	4.0
	WDI__industry	Quantile-SFA	hqer__induced	True	10	-168.89851444969386	hqer__induced sampler prototype	2.0
	WDI__industry	HQER-SFA	hqer__induced	True	15	-168.7411388760938	hqer__induced sampler prototype	2.0
	WDI__industry	Quantile-SFA	hqer__induced	True	28	-153.4224803104143	hqer__induced sampler prototype	4.0
	WDI__industry	HQER-SFA	hqer__induced	True	23	-149.73925401199986	hqer__induced sampler prototype	4.0
	WDI__industry	Quantile-SFA	hqer__induced	True	20	-166.65077118740868	hqer__induced sampler prototype	2.0
	WDI__industry	HQER-SFA	hqer__induced	True	18	-166.31406480142556	hqer__induced sampler prototype	2.0
	WDI__industry	Quantile-SFA	hqer__induced	True	23	-131.1005267089309	hqer__induced sampler prototype	4.0
	WDI__industry	HQER-SFA	hqer__induced	True	34	-128.37984740412216	hqer__induced sampler prototype	4.0
	WDI__industry	Quantile-SFA	hqer__induced	True	35	-141.31452425725345	hqer__induced sampler prototype	4.0
	WDI__industry	HQER-SFA	hqer__induced	True	29	-140.2435959339012	hqer__induced sampler prototype	2.0
	WDI__industry	Quantile-SFA	hqer__induced	True	25	-147.87624084463823	hqer__induced sampler prototype	4.0
	WDI__industry	HQER-SFA	hqer__induced	True	25	-148.0666952236096	hqer__induced sampler prototype	2.0
	WDI__industry	Quantile-SFA	hqer__induced	True	18	-170.65163525791863	hqer__induced sampler prototype	2.0
	WDI__industry	HQER-SFA	hqer__induced	True	34	-167.6914117650854	hqer__induced sampler prototype	4.0
	WDI__industry	Quantile-SFA	hqer__induced	True	23	-136.1872172925943	hqer__induced sampler prototype	2.0
	WDI__industry	HQER-SFA	hqer__induced	True	27	-133.91043311208938	hqer__induced sampler prototype	4.0
	WDI__industry	Quantile-SFA	hqer__induced	True	13	-155.56399490401728	hqer__induced sampler prototype	2.0
	WDI__industry	HQER-SFA	hqer__induced	True	47	-149.05955662429454	hqer__induced sampler prototype	2.0
	WDI__industry	Quantile-SFA	hqer__induced	True	28	-146.6683117288893	hqer__induced sampler prototype	4.0
	WDI__industry	HQER-SFA	hqer__induced	True	29	-141.85872796535492	hqer__induced sampler prototype	4.0
	WDI__industry	Quantile-SFA	hqer__induced	True	21	-144.67302050120193	hqer__induced sampler prototype	4.0
	WDI__industry	HQER-SFA	hqer__induced	True	8	-143.44449010277128	hqer__induced sampler prototype	2.0
	WDI__industry	Quantile-SFA	hqer__induced	True	20	-154.0936866904698	hqer__induced sampler prototype	4.0
	WDI__industry	HQER-SFA	hqer__induced	True	31	-150.6964268229045	hqer__induced sampler prototype	4.0
	WDI__industry	Quantile-SFA	hqer__induced	True	32	-98.56568838747287	hqer__induced sampler prototype	4.0
	WDI__industry	HQER-SFA	hqer__induced	True	30	-97.15794608930766	hqer__induced sampler prototype	2.0
	WDI__industry	Quantile-SFA	hqer__induced	True	23	-128.4895537649675	hqer__induced sampler prototype	4.0
	WDI__industry	HQER-SFA	hqer__induced	True	28	-129.83989232642062	hqer__induced sampler prototype	2.0
	WDI__industry	Quantile-SFA	hqer__induced	True	24	-140.62065146245124	hqer__induced sampler prototype	2.0
	WDI__industry	HQER-SFA	hqer__induced	True	36	-142.39100554490733	hqer__induced sampler prototype	4.0
	WDI__industry	Quantile-SFA	hqer__induced	True	28	-134.0324969867409	hqer__induced sampler prototype	4.0
	WDI__industry	HQER-SFA	hqer__induced	True	26	-133.2458643769735	hqer__induced sampler prototype	2.0
	WDI__industry	Quantile-SFA	hqer__induced	True	20	-147.15538773671113	hqer__induced sampler prototype	2.0
	WDI__industry	HQER-SFA	hqer__induced	True	17	-145.18999870298762	hqer__induced sampler prototype	2.0
	WDI__industry	Quantile-SFA	hqer__induced	True	17	-590.8841914123432	hqer__induced sampler prototype	4.0
	WDI__industry	HQER-SFA	hqer__induced	True	15	-582.2554075412173	hqer__induced sampler prototype	4.0

experiment	dataset	model	scenario	converged	n__iter	loglik	warning__message	best__start__id
	WDI__industry	Quantile-SFA	hqer__induced	True	23	-560.5429307607067	hqer__induced sampler prototype	2.0
	WDI__industry	HQER-SFA	hqer__induced	True	12	-550.5980125039813	hqer__induced sampler prototype	4.0
	WDI__industry	Quantile-SFA	hqer__induced	True	18	-611.9548602352297	hqer__induced sampler prototype	2.0
	WDI__industry	HQER-SFA	hqer__induced	True	24	-602.2080559433841	hqer__induced sampler prototype	4.0
	WDI__industry	Quantile-SFA	hqer__induced	True	17	-592.4992803577554	hqer__induced sampler prototype	4.0
	WDI__industry	HQER-SFA	hqer__induced	True	21	-583.0030038894149	hqer__induced sampler prototype	4.0
	WDI__industry	Quantile-SFA	hqer__induced	True	21	-574.792731637706	hqer__induced sampler prototype	4.0
	WDI__industry	HQER-SFA	hqer__induced	True	9	-571.3022536422463	hqer__induced sampler prototype	2.0
	WDI__industry	Quantile-SFA	hqer__induced	True	16	-668.0999962442372	hqer__induced sampler prototype	2.0
	WDI__industry	HQER-SFA	hqer__induced	True	21	-657.5843775998346	hqer__induced sampler prototype	4.0
	WDI__industry	Quantile-SFA	hqer__induced	True	23	-605.8590688100204	hqer__induced sampler prototype	2.0
	WDI__industry	HQER-SFA	hqer__induced	True	20	-590.5363349800713	hqer__induced sampler prototype	4.0
	WDI__industry	Quantile-SFA	hqer__induced	True	21	-649.1434259992518	hqer__induced sampler prototype	4.0
	WDI__industry	HQER-SFA	hqer__induced	True	21	-636.9121661528984	hqer__induced sampler prototype	4.0
	WDI__industry	Quantile-SFA	hqer__induced	True	17	-613.0195592294128	hqer__induced sampler prototype	4.0
	WDI__industry	HQER-SFA	hqer__induced	True	15	-603.7832581625971	hqer__induced sampler prototype	4.0
	WDI__industry	Quantile-SFA	hqer__induced	True	23	-642.8666648609722	hqer__induced sampler prototype	4.0
	WDI__industry	HQER-SFA	hqer__induced	True	19	-634.9512728711294	hqer__induced sampler prototype	4.0
	WDI__industry	Quantile-SFA	hqer__induced	True	23	-584.4044250207628	hqer__induced sampler prototype	2.0
	WDI__industry	HQER-SFA	hqer__induced	True	23	-576.2218855032609	hqer__induced sampler prototype	4.0
	WDI__industry	Quantile-SFA	hqer__induced	True	14	-603.403361129539	hqer__induced sampler prototype	2.0
	WDI__industry	HQER-SFA	hqer__induced	True	29	-591.810467295784	hqer__induced sampler prototype	4.0
	WDI__industry	Quantile-SFA	hqer__induced	True	14	-713.5453405164149	hqer__induced sampler prototype	4.0
	WDI__industry	HQER-SFA	hqer__induced	True	20	-706.776020887839	hqer__induced sampler prototype	2.0
	WDI__industry	Quantile-SFA	hqer__induced	True	16	-645.3621642318408	hqer__induced sampler prototype	4.0
	WDI__industry	HQER-SFA	hqer__induced	True	19	-636.080466085523	hqer__induced sampler prototype	4.0
	WDI__industry	Quantile-SFA	hqer__induced	True	21	-612.9355952214564	hqer__induced sampler prototype	4.0
	WDI__industry	HQER-SFA	hqer__induced	True	21	-600.9661631040395	hqer__induced sampler prototype	4.0
	WDI__industry	Quantile-SFA	hqer__induced	True	23	-624.3859327442119	hqer__induced sampler prototype	2.0
	WDI__industry	HQER-SFA	hqer__induced	True	18	-615.3503655430877	hqer__induced sampler prototype	4.0
	WDI__industry	Quantile-SFA	hqer__induced	True	11	-613.9493554824492	hqer__induced sampler prototype	2.0
	WDI__industry	HQER-SFA	hqer__induced	True	12	-603.7727786638407	hqer__induced sampler prototype	2.0
	WDI__industry	Quantile-SFA	hqer__induced	True	8	-609.8265997685205	hqer__induced sampler prototype	2.0
	WDI__industry	HQER-SFA	hqer__induced	True	12	-602.2903460979237	hqer__induced sampler prototype	2.0
	WDI__industry	Quantile-SFA	hqer__induced	True	25	-608.2343114275654	hqer__induced sampler prototype	4.0
	WDI__industry	HQER-SFA	hqer__induced	True	22	-597.318241846241	hqer__induced sampler prototype	4.0
	WDI__industry	Quantile-SFA	hqer__induced	True	25	-571.06823234734	hqer__induced sampler prototype	4.0
	WDI__industry	HQER-SFA	hqer__induced	True	19	-564.2500255606554	hqer__induced sampler prototype	2.0
	WDI__industry	Quantile-SFA	hqer__induced	True	23	-667.7773376971345	hqer__induced sampler prototype	4.0
	WDI__industry	HQER-SFA	hqer__induced	True	17	-660.7429585593391	hqer__induced sampler prototype	2.0
	WDI__industry	Quantile-SFA	hqer__induced	True	22	-677.9909128532988	hqer__induced sampler prototype	2.0
	WDI__industry	HQER-SFA	hqer__induced	True	16	-667.3814514749158	hqer__induced sampler prototype	4.0
	WDI__industry	Quantile-SFA	hqer__induced	True	20	-635.0669123747874	hqer__induced sampler prototype	2.0
	WDI__industry	HQER-SFA	hqer__induced	True	11	-621.5489920751604	hqer__induced sampler prototype	4.0
	WDI__industry	Quantile-SFA	hqer__induced	True	25	-607.4232433934592	hqer__induced sampler prototype	4.0
	WDI__industry	HQER-SFA	hqer__induced	True	22	-598.8790191069672	hqer__induced sampler prototype	4.0
	WDI__industry	Quantile-SFA	hqer__induced	True	9	-670.9382306292542	hqer__induced sampler prototype	2.0
	WDI__industry	HQER-SFA	hqer__induced	True	27	-660.2095048954561	hqer__induced sampler prototype	5.0
	WDI__industry	Quantile-SFA	hqer__induced	True	9	-607.7630217129199	hqer__induced sampler prototype	2.0
	WDI__industry	HQER-SFA	hqer__induced	True	24	-598.8907951024996	hqer__induced sampler prototype	4.0
	WDI__industry	Quantile-SFA	hqer__induced	True	12	-627.1041478810216	hqer__induced sampler prototype	2.0
	WDI__industry	HQER-SFA	hqer__induced	True	23	-612.8365355533524	hqer__induced sampler prototype	2.0
	WDI__industry	Quantile-SFA	hqer__induced	True	15	-565.6939643370747	hqer__induced sampler prototype	2.0
	WDI__industry	HQER-SFA	hqer__induced	True	18	-558.3185755228767	hqer__induced sampler prototype	2.0
	WDI__industry	Quantile-SFA	hqer__induced	True	24	-571.247409052633	hqer__induced sampler prototype	2.0
	WDI__industry	HQER-SFA	hqer__induced	True	26	-561.2421201684853	hqer__induced sampler prototype	4.0
	WDI__industry	Quantile-SFA	hqer__induced	True	21	-620.9014345251467	hqer__induced sampler prototype	4.0
	WDI__industry	HQER-SFA	hqer__induced	True	23	-609.0143442186566	hqer__induced sampler prototype	4.0
	WDI__industry	Quantile-SFA	hqer__induced	True	21	-572.6005542192306	hqer__induced sampler prototype	4.0
	WDI__industry	HQER-SFA	hqer__induced	True	21	-564.1234584928984	hqer__induced sampler prototype	4.0
	WDI__industry	Quantile-SFA	hqer__induced	True	14	-676.7125268001838	hqer__induced sampler prototype	4.0
	WDI__industry	HQER-SFA	hqer__induced	True	19	-663.9949113912558	hqer__induced sampler prototype	4.0
	WDI__industry	Quantile-SFA	hqer__induced	True	19	-583.4188842233705	hqer__induced sampler prototype	4.0
	WDI__industry	HQER-SFA	hqer__induced	True	24	-569.2484816345387	hqer__induced sampler prototype	4.0
	WDI__industry	Quantile-SFA	hqer__induced	True	15	-671.8451353247314	hqer__induced sampler prototype	2.0
	WDI__industry	HQER-SFA	hqer__induced	True	18	-661.4422937043361	hqer__induced sampler prototype	4.0
	WDI__industry	Quantile-SFA	hqer__induced	True	14	-627.4778500871695	hqer__induced sampler prototype	4.0
	WDI__industry	HQER-SFA	hqer__induced	True	17	-613.5427909454557	hqer__induced sampler prototype	4.0
	WDI__industry	Quantile-SFA	hqer__induced	True	19	-622.804333121287	hqer__induced sampler prototype	4.0
	WDI__industry	HQER-SFA	hqer__induced	True	17	-619.4181772876135	hqer__induced sampler prototype	2.0
	WDI__industry	Quantile-SFA	hqer__induced	True	21	-648.0244293035778	hqer__induced sampler prototype	2.0
	WDI__industry	HQER-SFA	hqer__induced	True	21	-636.4553695077363	hqer__induced sampler prototype	4.0
	WDI__industry	Quantile-SFA	hqer__induced	True	25	-622.8986600689411	hqer__induced sampler prototype	4.0
	WDI__industry	HQER-SFA	hqer__induced	True	26	-616.1073713982232	hqer__induced sampler prototype	4.0
	WDI__industry	Quantile-SFA	hqer__induced	True	22	-668.1208250068441	hqer__induced sampler prototype	4.0
	WDI__industry	HQER-SFA	hqer__induced	True	17	-657.6221750163857	hqer__induced sampler prototype	4.0
	WDI__industry	Quantile-SFA	hqer__induced	True	20	-650.0988921481305	hqer__induced sampler prototype	4.0
	WDI__industry	HQER-SFA	hqer__induced	True	20	-642.4613987659067	hqer__induced sampler prototype	4.0
	WDI__industry	Quantile-SFA	hqer__induced	True	26	-645.5258354621483	hqer__induced sampler prototype	4.0
	WDI__industry	HQER-SFA	hqer__induced	True	27	-634.1648793538612	hqer__induced sampler prototype	4.0
	WDI__industry	Quantile-SFA	hqer__induced	True	14	-633.3010922367635	hqer__induced sampler prototype	2.0
	WDI__industry	HQER-SFA	hqer__induced	True	20	-624.077820150361	hqer__induced sampler prototype	4.0
	WDI__industry	Quantile-SFA	hqer__induced	True	21	-664.3769873857756	hqer__induced sampler prototype	2.0
	WDI__industry	HQER-SFA	hqer__induced	True	24	-654.723696492937	hqer__induced sampler prototype	4.0
	WDI__industry	Quantile-SFA	hqer__induced	True	17	-665.27217989929	hqer__induced sampler prototype	4.0
	WDI__industry	HQER-SFA	hqer__induced	True	19	-654.5480125154144	hqer__induced sampler prototype	4.0
	WDI__industry	Quantile-SFA	hqer__induced	True	8	-680.5466391916282	hqer__induced sampler prototype	2.0
	WDI__industry	HQER-SFA	hqer__induced	True	24	-665.3600871947378	hqer__induced sampler prototype	4.0
	WDI__industry	Quantile-SFA	hqer__induced	True	26	-579.494253339131	hqer__induced sampler prototype	4.0
	WDI__industry	HQER-SFA	hqer__induced	True	14	-561.7821577531105	hqer__induced sampler prototype	4.0
	WDI__industry	Quantile-SFA	hqer__induced	True	22	-628.3947081576089	hqer__induced sampler prototype	4.0
	WDI__industry	HQER-SFA	hqer__induced	True	24	-621.3933964407348	hqer__induced sampler prototype	2.0
	WDI__industry	Quantile-SFA	hqer__induced	True	23	-653.6708122040885	hqer__induced sampler prototype	4.0
	WDI__industry	HQER-SFA	hqer__induced	True	26	-646.7746696597098	hqer__induced sampler prototype	2.0
	WDI__industry	Quantile-SFA	hqer__induced	True	33	-595.9653012337524	hqer__induced sampler prototype	4.0
	WDI__industry	HQER-SFA	hqer__induced	True	14	-589.1233979825149	hqer__induced sampler prototype	2.0
	WDI__industry	Quantile-SFA	hqer__induced	True	20	-667.2068949157134	hqer__induced sampler prototype	4.0
	WDI__industry	HQER-SFA	hqer__induced	True	14	-660.8944439941478	hqer__induced sampler prototype	2.0
	WDI__industry	Quantile-SFA	hqer__induced	True	28	-567.8762091727165	hqer__induced sampler prototype	4.0
	WDI__industry	HQER-SFA	hqer__induced	True	10	-561.6892864018992	hqer__induced sampler prototype	2.0
	WDI__industry	Quantile-SFA	hqer__induced	True	20	-599.6169839462634	hqer__induced sampler prototype	4.0
	WDI__industry	HQER-SFA	hqer__induced	True	25	-589.3199584885003	hqer__induced sampler prototype	4.0
	WDI__industry	Quantile-SFA	hqer__induced	True	21	-598.963842336949	hqer__induced sampler prototype	4.0

experiment	dataset	model	scenario	converged	n_iter	loglik	warning_message	best_start_id
	WDI_industry	HQER-SFA	hqer_induced	True	9	-593.2652822966836	hqer_induced sampler prototype	2.0
	WDI_industry	Quantile-SFA	hqer_induced	True	21	-639.3340080236396	hqer_induced sampler prototype	4.0
	WDI_industry	HQER-SFA	hqer_induced	True	15	-634.8807281993772	hqer_induced sampler prototype	2.0
	WDI_industry	Quantile-SFA	hqer_induced	True	16	-701.452072009218	hqer_induced sampler prototype	4.0
	WDI_industry	HQER-SFA	hqer_induced	True	12	-690.0583037867007	hqer_induced sampler prototype	4.0
	WDI_industry	Quantile-SFA	hqer_induced	True	21	-638.2324194285716	hqer_induced sampler prototype	4.0
	WDI_industry	HQER-SFA	hqer_induced	True	16	-633.9357712061717	hqer_induced sampler prototype	2.0
	WDI_industry	Quantile-SFA	hqer_induced	True	17	-620.9698435264834	hqer_induced sampler prototype	4.0
	WDI_industry	HQER-SFA	hqer_induced	True	17	-615.2976296337076	hqer_induced sampler prototype	2.0
	WDI_industry	Quantile-SFA	hqer_induced	True	19	-632.1301833883474	hqer_induced sampler prototype	2.0
	WDI_industry	HQER-SFA	hqer_induced	True	21	-624.6919952013955	hqer_induced sampler prototype	2.0
	WDI_industry	Quantile-SFA	hqer_induced	True	20	-594.1773701628548	hqer_induced sampler prototype	4.0
	WDI_industry	HQER-SFA	hqer_induced	True	15	-585.3093206069357	hqer_induced sampler prototype	4.0
	WDI_industry	Quantile-SFA	hqer_induced	True	18	-680.7952246523014	hqer_induced sampler prototype	4.0
	WDI_industry	HQER-SFA	hqer_induced	True	20	-669.7729928378019	hqer_induced sampler prototype	4.0
	WDI_industry	Quantile-SFA	hqer_induced	True	20	-577.4315236135864	hqer_induced sampler prototype	2.0
	WDI_industry	HQER-SFA	hqer_induced	True	33	-572.4644765426174	hqer_induced sampler prototype	2.0
	WDI_industry	Quantile-SFA	hqer_induced	True	14	-580.3527247268694	hqer_induced sampler prototype	2.0
	WDI_industry	HQER-SFA	hqer_induced	True	13	-575.6272165081272	hqer_induced sampler prototype	2.0
	WDI_industry	Quantile-SFA	hqer_induced	True	20	-520.2537418859174	hqer_induced sampler prototype	4.0
	WDI_industry	HQER-SFA	hqer_induced	True	15	-513.984782947553	hqer_induced sampler prototype	2.0
	WDI_industry	Quantile-SFA	hqer_induced	True	25	-617.9452763893578	hqer_induced sampler prototype	2.0
	WDI_industry	HQER-SFA	hqer_induced	True	20	-612.4365785357616	hqer_induced sampler prototype	2.0
	WDI_industry	Quantile-SFA	hqer_induced	True	18	-679.15287454171	hqer_induced sampler prototype	2.0
	WDI_industry	HQER-SFA	hqer_induced	True	21	-668.5877264937274	hqer_induced sampler prototype	4.0
	WDI_industry	Quantile-SFA	hqer_induced	True	16	-582.1141202967613	hqer_induced sampler prototype	4.0
	WDI_industry	HQER-SFA	hqer_induced	True	18	-572.9065394647807	hqer_induced sampler prototype	4.0
	WDI_industry	Quantile-SFA	hqer_induced	True	25	-537.7690331610426	hqer_induced sampler prototype	4.0
	WDI_industry	HQER-SFA	hqer_induced	True	24	-526.7191143793112	hqer_induced sampler prototype	4.0
	WDI_industry	Quantile-SFA	hqer_induced	True	34	-581.9450350123875	hqer_induced sampler prototype	5.0
	WDI_industry	HQER-SFA	hqer_induced	True	19	-572.482651481455	hqer_induced sampler prototype	4.0
	WDI_industry	Quantile-SFA	hqer_induced	True	17	-645.1191541593564	hqer_induced sampler prototype	2.0
	WDI_industry	HQER-SFA	hqer_induced	True	29	-629.520298370199	hqer_induced sampler prototype	4.0
	WDI_industry	Quantile-SFA	hqer_induced	True	20	-635.1267198847395	hqer_induced sampler prototype	4.0
	WDI_industry	HQER-SFA	hqer_induced	True	18	-622.5636482118969	hqer_induced sampler prototype	4.0
	WDI_industry	Quantile-SFA	hqer_induced	True	18	-656.6207727946972	hqer_induced sampler prototype	4.0
	WDI_industry	HQER-SFA	hqer_induced	True	31	-642.9315709977791	hqer_induced sampler prototype	2.0
	WDI_industry	Quantile-SFA	hqer_induced	True	17	-662.5125806789628	hqer_induced sampler prototype	4.0
	WDI_industry	HQER-SFA	hqer_induced	True	14	-651.8075842500871	hqer_induced sampler prototype	4.0
	WDI_industry	Quantile-SFA	hqer_induced	True	20	-627.9056569566752	hqer_induced sampler prototype	4.0
	WDI_industry	HQER-SFA	hqer_induced	True	7	-622.106684262891	hqer_induced sampler prototype	2.0
	WDI_industry	Quantile-SFA	hqer_induced	True	20	-647.7003045719872	hqer_induced sampler prototype	4.0
	WDI_industry	HQER-SFA	hqer_induced	True	16	-640.0171710178186	hqer_induced sampler prototype	4.0
	WDI_industry	Quantile-SFA	hqer_induced	True	22	-609.3877101548962	hqer_induced sampler prototype	4.0
	WDI_industry	HQER-SFA	hqer_induced	True	29	-599.1566421733978	hqer_induced sampler prototype	5.0
	WDI_industry	Quantile-SFA	hqer_induced	True	18	-629.9517561664134	hqer_induced sampler prototype	2.0
	WDI_industry	HQER-SFA	hqer_induced	True	19	-619.5088985987184	hqer_induced sampler prototype	4.0
	WDI_industry	Quantile-SFA	hqer_induced	True	22	-685.038303060838	hqer_induced sampler prototype	4.0
	WDI_industry	HQER-SFA	hqer_induced	True	29	-671.0255746760063	hqer_induced sampler prototype	2.0
	WDI_industry	Quantile-SFA	hqer_induced	True	19	-670.1465408789824	hqer_induced sampler prototype	2.0
	WDI_industry	HQER-SFA	hqer_induced	True	13	-660.9227450945016	hqer_induced sampler prototype	4.0
	WDI_industry	Quantile-SFA	hqer_induced	True	13	-632.6091529630517	hqer_induced sampler prototype	2.0
	WDI_industry	HQER-SFA	hqer_induced	True	13	-627.8296475735318	hqer_induced sampler prototype	2.0
	WDI_industry	Quantile-SFA	hqer_induced	True	25	-628.6087428029687	hqer_induced sampler prototype	4.0
	WDI_industry	HQER-SFA	hqer_induced	True	21	-618.522671106353	hqer_induced sampler prototype	4.0
	WDI_industry	Quantile-SFA	hqer_induced	True	19	-590.2099818788467	hqer_induced sampler prototype	2.0
	WDI_industry	HQER-SFA	hqer_induced	True	11	-578.8815572991914	hqer_induced sampler prototype	4.0
	WDI_industry	Quantile-SFA	hqer_induced	True	20	-588.3390571176087	hqer_induced sampler prototype	4.0
	WDI_industry	HQER-SFA	hqer_induced	True	30	-575.103432747142	hqer_induced sampler prototype	4.0
	WDI_industry	Quantile-SFA	hqer_induced	True	16	-599.158815435304	hqer_induced sampler prototype	4.0
	WDI_industry	HQER-SFA	hqer_induced	True	23	-592.4787325415734	hqer_induced sampler prototype	4.0
	WDI_industry	Quantile-SFA	hqer_induced	True	17	-553.3780369857279	hqer_induced sampler prototype	4.0
	WDI_industry	HQER-SFA	hqer_induced	True	23	-543.1842239080651	hqer_induced sampler prototype	4.0
	WDI_industry	Quantile-SFA	hqer_induced	True	23	-615.6036790851007	hqer_induced sampler prototype	2.0
	WDI_industry	HQER-SFA	hqer_induced	True	15	-606.268682513565	hqer_induced sampler prototype	4.0
	WDI_industry	Quantile-SFA	hqer_induced	True	18	-596.9500228535653	hqer_induced sampler prototype	2.0
	WDI_industry	HQER-SFA	hqer_induced	True	21	-588.3500362744426	hqer_induced sampler prototype	4.0
	WDI_industry	Quantile-SFA	hqer_induced	True	21	-611.8559579358705	hqer_induced sampler prototype	4.0
	WDI_industry	HQER-SFA	hqer_induced	True	25	-601.2327808790554	hqer_induced sampler prototype	4.0
	WDI_industry	Quantile-SFA	hqer_induced	True	7	-594.654052059816	hqer_induced sampler prototype	2.0
	WDI_industry	HQER-SFA	hqer_induced	True	16	-582.5709068891695	hqer_induced sampler prototype	4.0
	WDI_industry	Quantile-SFA	hqer_induced	True	15	-675.9441633949859	hqer_induced sampler prototype	2.0
	WDI_industry	HQER-SFA	hqer_induced	True	24	-668.0328520678718	hqer_induced sampler prototype	2.0
	WDI_industry	Quantile-SFA	hqer_induced	True	19	-597.5771355862664	hqer_induced sampler prototype	2.0
	WDI_industry	HQER-SFA	hqer_induced	True	18	-589.8317778842829	hqer_induced sampler prototype	2.0
	WDI_industry	Quantile-SFA	hqer_induced	True	24	-713.8605086720993	hqer_induced sampler prototype	4.0
	WDI_industry	HQER-SFA	hqer_induced	True	20	-703.8065804841739	hqer_induced sampler prototype	4.0
	WDI_industry	Quantile-SFA	hqer_induced	True	14	-699.4739653839905	hqer_induced sampler prototype	2.0
	WDI_industry	HQER-SFA	hqer_induced	True	21	-688.4863556672492	hqer_induced sampler prototype	4.0
	WDI_industry	Quantile-SFA	hqer_induced	True	15	-592.8715776534062	hqer_induced sampler prototype	2.0
	WDI_industry	HQER-SFA	hqer_induced	True	23	-580.3306602457159	hqer_induced sampler prototype	4.0
	WDI_industry	Quantile-SFA	hqer_induced	True	11	-656.5784204495151	hqer_induced sampler prototype	2.0
	WDI_industry	HQER-SFA	hqer_induced	True	17	-645.3249285484063	hqer_induced sampler prototype	4.0
	WDI_industry	Quantile-SFA	hqer_induced	True	19	-598.1581793093363	hqer_induced sampler prototype	2.0
	WDI_industry	HQER-SFA	hqer_induced	True	22	-592.8185321776145	hqer_induced sampler prototype	2.0
	WDI_industry	Quantile-SFA	hqer_induced	True	22	-536.6738994978107	hqer_induced sampler prototype	4.0
	WDI_industry	HQER-SFA	hqer_induced	True	13	-530.8922606024	hqer_induced sampler prototype	2.0
	WDI_industry	Quantile-SFA	hqer_induced	True	19	-646.7131236573545	hqer_induced sampler prototype	4.0
	WDI_industry	HQER-SFA	hqer_induced	True	17	-636.2581470469017	hqer_induced sampler prototype	4.0
	WDI_industry	Quantile-SFA	hqer_induced	True	17	-627.2598019750094	hqer_induced sampler prototype	4.0
	WDI_industry	HQER-SFA	hqer_induced	True	16	-623.9036935149413	hqer_induced sampler prototype	2.0
	WDI_industry	Quantile-SFA	hqer_induced	True	21	-635.9298296124459	hqer_induced sampler prototype	4.0
	WDI_industry	HQER-SFA	hqer_induced	True	22	-626.2010966289588	hqer_induced sampler prototype	4.0
	WDI_industry	Quantile-SFA	hqer_induced	True	14	-628.8434954291022	hqer_induced sampler prototype	2.0
	WDI_industry	HQER-SFA	hqer_induced	True	4	-621.8045043078689	hqer_induced sampler prototype	2.0
	WDI_agriculture	Quantile-SFA	hqer_induced	True	30	-716.5024820608373	hqer_induced sampler prototype	3.0
	WDI_agriculture	HQER-SFA	hqer_induced	True	26	-716.4790081173292	hqer_induced sampler prototype	1.0
	WDI_agriculture	Quantile-SFA	hqer_induced	True	27	-700.1898951368343	hqer_induced sampler prototype	1.0
	WDI_agriculture	HQER-SFA	hqer_induced	True	29	-700.1847434826575	hqer_induced sampler prototype	3.0
	WDI_agriculture	Quantile-SFA	hqer_induced	True	36	-685.7191101272938	hqer_induced sampler prototype	1.0
	WDI_agriculture	HQER-SFA	hqer_induced	True	24	-685.8270424761699	hqer_induced sampler prototype	3.0
	WDI_agriculture	Quantile-SFA	hqer_induced	True	26	-694.7425826537369	hqer_induced sampler prototype	2.0
	WDI_agriculture	HQER-SFA	hqer_induced	True	30	-694.7239963227078	hqer_induced sampler prototype	3.0

experiment	dataset	model	scenario	converged	n_iter	loglik	warning_message	best_start_id
	WDI_agriculture	Quantile-SFA	hqer_induced	True	26	-721.6207259184234	hqer_induced sampler prototype	3.0
	WDI_agriculture	HQER-SFA	hqer_induced	True	31	-721.6131379007611	hqer_induced sampler prototype	3.0
	WDI_agriculture	Quantile-SFA	hqer_induced	True	51	-710.4071824868793	hqer_induced sampler prototype	3.0
	WDI_agriculture	HQER-SFA	hqer_induced	True	39	-710.4474559813125	hqer_induced sampler prototype	3.0
	WDI_agriculture	Quantile-SFA	hqer_induced	True	16	-696.5015770658607	hqer_induced sampler prototype	2.0
	WDI_agriculture	HQER-SFA	hqer_induced	True	34	-696.4809138929677	hqer_induced sampler prototype	3.0
	WDI_agriculture	Quantile-SFA	hqer_induced	True	30	-648.8380265261859	hqer_induced sampler prototype	1.0
	WDI_agriculture	HQER-SFA	hqer_induced	True	23	-648.8173089973554	hqer_induced sampler prototype	3.0
	WDI_agriculture	Quantile-SFA	hqer_induced	True	23	-739.953303296529	hqer_induced sampler prototype	1.0
	WDI_agriculture	HQER-SFA	hqer_induced	True	32	-739.9551968347109	hqer_induced sampler prototype	3.0
	WDI_agriculture	Quantile-SFA	hqer_induced	True	25	-702.0024750011662	hqer_induced sampler prototype	2.0
	WDI_agriculture	HQER-SFA	hqer_induced	True	48	-701.9960060975482	hqer_induced sampler prototype	3.0
	WDI_agriculture	Quantile-SFA	hqer_induced	True	34	-687.3156209713917	hqer_induced sampler prototype	1.0
	WDI_agriculture	HQER-SFA	hqer_induced	True	26	-687.311776689167	hqer_induced sampler prototype	1.0
	WDI_agriculture	Quantile-SFA	hqer_induced	True	33	-703.4086365875029	hqer_induced sampler prototype	1.0
	WDI_agriculture	HQER-SFA	hqer_induced	True	36	-703.3800673805545	hqer_induced sampler prototype	3.0
	WDI_agriculture	Quantile-SFA	hqer_induced	True	14	-693.5996347229603	hqer_induced sampler prototype	2.0
	WDI_agriculture	HQER-SFA	hqer_induced	True	16	-693.5853870534798	hqer_induced sampler prototype	2.0
	WDI_agriculture	Quantile-SFA	hqer_induced	True	20	-706.4890270656429	hqer_induced sampler prototype	3.0
	WDI_agriculture	HQER-SFA	hqer_induced	True	22	-706.4307212435118	hqer_induced sampler prototype	3.0
	WDI_agriculture	Quantile-SFA	hqer_induced	True	47	-712.6930967820279	hqer_induced sampler prototype	1.0
	WDI_agriculture	HQER-SFA	hqer_induced	True	37	-712.6960445073182	hqer_induced sampler prototype	5.0
	WDI_agriculture	Quantile-SFA	hqer_induced	True	21	-735.8870140750786	hqer_induced sampler prototype	5.0
	WDI_agriculture	HQER-SFA	hqer_induced	True	18	-735.8606548772598	hqer_induced sampler prototype	2.0
	WDI_agriculture	Quantile-SFA	hqer_induced	True	25	-711.3832227063479	hqer_induced sampler prototype	1.0
	WDI_agriculture	HQER-SFA	hqer_induced	True	35	-711.2897606536648	hqer_induced sampler prototype	1.0
	WDI_agriculture	Quantile-SFA	hqer_induced	True	32	-750.798004082874	hqer_induced sampler prototype	3.0
	WDI_agriculture	HQER-SFA	hqer_induced	True	34	-750.8089750819234	hqer_induced sampler prototype	3.0
	WDI_agriculture	Quantile-SFA	hqer_induced	True	35	-680.095849743715	hqer_induced sampler prototype	2.0
	WDI_agriculture	HQER-SFA	hqer_induced	True	30	-680.0244777807807	hqer_induced sampler prototype	5.0
	WDI_agriculture	Quantile-SFA	hqer_induced	True	33	-727.2415465947513	hqer_induced sampler prototype	1.0
	WDI_agriculture	HQER-SFA	hqer_induced	True	26	-727.2075265126534	hqer_induced sampler prototype	1.0
	WDI_agriculture	Quantile-SFA	hqer_induced	True	27	-727.2682955789451	hqer_induced sampler prototype	3.0
	WDI_agriculture	HQER-SFA	hqer_induced	True	17	-727.2639393693058	hqer_induced sampler prototype	2.0
	WDI_agriculture	Quantile-SFA	hqer_induced	True	34	-721.5874774106375	hqer_induced sampler prototype	1.0
	WDI_agriculture	HQER-SFA	hqer_induced	True	23	-721.5689874312728	hqer_induced sampler prototype	5.0
	WDI_agriculture	Quantile-SFA	hqer_induced	True	36	-708.0841003136209	hqer_induced sampler prototype	3.0
	WDI_agriculture	HQER-SFA	hqer_induced	True	23	-708.1127597452694	hqer_induced sampler prototype	3.0
	WDI_agriculture	Quantile-SFA	hqer_induced	True	35	-687.7807885330956	hqer_induced sampler prototype	1.0
	WDI_agriculture	HQER-SFA	hqer_induced	True	32	-687.7643416163133	hqer_induced sampler prototype	3.0
	WDI_agriculture	Quantile-SFA	hqer_induced	True	21	-698.3084479043066	hqer_induced sampler prototype	1.0
	WDI_agriculture	HQER-SFA	hqer_induced	True	29	-698.8449891891883	hqer_induced sampler prototype	1.0
	WDI_agriculture	Quantile-SFA	hqer_induced	True	27	-713.0884997884461	hqer_induced sampler prototype	3.0
	WDI_agriculture	HQER-SFA	hqer_induced	True	22	-713.1489329917888	hqer_induced sampler prototype	5.0
	WDI_agriculture	Quantile-SFA	hqer_induced	True	45	-720.9349686817055	hqer_induced sampler prototype	3.0
	WDI_agriculture	HQER-SFA	hqer_induced	True	38	-720.0715604534171	hqer_induced sampler prototype	1.0
	WDI_agriculture	Quantile-SFA	hqer_induced	True	38	-733.1035451973328	hqer_induced sampler prototype	3.0
	WDI_agriculture	HQER-SFA	hqer_induced	True	31	-733.1413098261027	hqer_induced sampler prototype	1.0
	WDI_agriculture	Quantile-SFA	hqer_induced	True	47	-707.0227793386945	hqer_induced sampler prototype	1.0
	WDI_agriculture	HQER-SFA	hqer_induced	True	46	-708.5110589321182	hqer_induced sampler prototype	3.0
	WDI_agriculture	Quantile-SFA	hqer_induced	True	17	-686.5478371294734	hqer_induced sampler prototype	2.0
	WDI_agriculture	HQER-SFA	hqer_induced	True	19	-686.5349701069088	hqer_induced sampler prototype	2.0
	WDI_agriculture	Quantile-SFA	hqer_induced	True	27	-729.5722744466678	hqer_induced sampler prototype	1.0
	WDI_agriculture	HQER-SFA	hqer_induced	True	23	-729.5760028004299	hqer_induced sampler prototype	1.0
	WDI_agriculture	Quantile-SFA	hqer_induced	True	12	-708.1531992283402	hqer_induced sampler prototype	2.0
	WDI_agriculture	HQER-SFA	hqer_induced	True	9	-708.1581115874413	hqer_induced sampler prototype	2.0
	WDI_agriculture	Quantile-SFA	hqer_induced	True	18	-732.4723239996706	hqer_induced sampler prototype	2.0
	WDI_agriculture	HQER-SFA	hqer_induced	True	28	-732.4367470791075	hqer_induced sampler prototype	1.0
	WDI_agriculture	Quantile-SFA	hqer_induced	True	34	-720.7253992914541	hqer_induced sampler prototype	3.0
	WDI_agriculture	HQER-SFA	hqer_induced	True	16	-720.7036725168155	hqer_induced sampler prototype	5.0
	WDI_agriculture	Quantile-SFA	hqer_induced	True	28	-746.1750230446406	hqer_induced sampler prototype	5.0
	WDI_agriculture	HQER-SFA	hqer_induced	True	34	-746.1630908969194	hqer_induced sampler prototype	5.0
	WDI_agriculture	Quantile-SFA	hqer_induced	True	40	-697.802651914165	hqer_induced sampler prototype	3.0
	WDI_agriculture	HQER-SFA	hqer_induced	True	31	-697.8090336296568	hqer_induced sampler prototype	5.0
	WDI_agriculture	Quantile-SFA	hqer_induced	True	36	-704.3731013646939	hqer_induced sampler prototype	2.0
	WDI_agriculture	HQER-SFA	hqer_induced	True	23	-704.6152233476028	hqer_induced sampler prototype	5.0
	WDI_agriculture	Quantile-SFA	hqer_induced	True	24	-717.0719808745907	hqer_induced sampler prototype	3.0
	WDI_agriculture	HQER-SFA	hqer_induced	True	30	-717.0221405812321	hqer_induced sampler prototype	3.0
	WDI_agriculture	Quantile-SFA	hqer_induced	True	28	-706.4011383368753	hqer_induced sampler prototype	2.0
	WDI_agriculture	HQER-SFA	hqer_induced	True	30	-706.3759254198159	hqer_induced sampler prototype	1.0
	WDI_agriculture	Quantile-SFA	hqer_induced	True	40	-724.7483768334246	hqer_induced sampler prototype	5.0
	WDI_agriculture	HQER-SFA	hqer_induced	True	40	-724.7433705619401	hqer_induced sampler prototype	5.0
	WDI_agriculture	Quantile-SFA	hqer_induced	True	22	-683.8020978244247	hqer_induced sampler prototype	5.0
	WDI_agriculture	HQER-SFA	hqer_induced	True	16	-683.8116981887646	hqer_induced sampler prototype	5.0
	WDI_agriculture	Quantile-SFA	hqer_induced	True	33	-716.35985049265	hqer_induced sampler prototype	3.0
	WDI_agriculture	HQER-SFA	hqer_induced	True	24	-716.289202615566	hqer_induced sampler prototype	1.0
	WDI_agriculture	Quantile-SFA	hqer_induced	True	27	-730.1847554954273	hqer_induced sampler prototype	3.0
	WDI_agriculture	HQER-SFA	hqer_induced	True	33	-730.183672174375	hqer_induced sampler prototype	1.0
	WDI_agriculture	Quantile-SFA	hqer_induced	True	21	-712.6959576121637	hqer_induced sampler prototype	5.0
	WDI_agriculture	HQER-SFA	hqer_induced	True	22	-712.7207673915431	hqer_induced sampler prototype	5.0
	WDI_agriculture	Quantile-SFA	hqer_induced	True	19	-745.5403485139879	hqer_induced sampler prototype	5.0
	WDI_agriculture	HQER-SFA	hqer_induced	True	38	-745.5263490445311	hqer_induced sampler prototype	3.0
	WDI_agriculture	Quantile-SFA	hqer_induced	True	42	-704.7729801419056	hqer_induced sampler prototype	1.0
	WDI_agriculture	HQER-SFA	hqer_induced	True	14	-706.136247037075	hqer_induced sampler prototype	2.0
	WDI_agriculture	Quantile-SFA	hqer_induced	True	25	-716.5336857547404	hqer_induced sampler prototype	3.0
	WDI_agriculture	HQER-SFA	hqer_induced	True	28	-716.509572704157	hqer_induced sampler prototype	1.0
	WDI_agriculture	Quantile-SFA	hqer_induced	True	27	-711.3852048487527	hqer_induced sampler prototype	5.0
	WDI_agriculture	HQER-SFA	hqer_induced	True	41	-711.2541182925008	hqer_induced sampler prototype	5.0
	WDI_agriculture	Quantile-SFA	hqer_induced	True	24	-732.923453163404	hqer_induced sampler prototype	1.0
	WDI_agriculture	HQER-SFA	hqer_induced	True	31	-732.9142638200112	hqer_induced sampler prototype	1.0
	WDI_agriculture	Quantile-SFA	hqer_induced	True	24	-710.1265988242699	hqer_induced sampler prototype	5.0
	WDI_agriculture	HQER-SFA	hqer_induced	True	22	-710.086446666661	hqer_induced sampler prototype	2.0
	WDI_agriculture	Quantile-SFA	hqer_induced	True	32	-717.7838865673018	hqer_induced sampler prototype	5.0
	WDI_agriculture	HQER-SFA	hqer_induced	True	23	-717.7801435416701	hqer_induced sampler prototype	5.0
	WDI_agriculture	Quantile-SFA	hqer_induced	True	34	-697.2292554305734	hqer_induced sampler prototype	2.0
	WDI_agriculture	HQER-SFA	hqer_induced	True	16	-697.5814455400018	hqer_induced sampler prototype	5.0
	WDI_agriculture	Quantile-SFA	hqer_induced	True	37	-702.029287009376	hqer_induced sampler prototype	1.0
	WDI_agriculture	HQER-SFA	hqer_induced	True	26	-702.077779428033	hqer_induced sampler prototype	3.0
	WDI_agriculture	Quantile-SFA	hqer_induced	True	34	-697.0324527020265	hqer_induced sampler prototype	1.0
	WDI_agriculture	HQER-SFA	hqer_induced	True	22	-697.0897951227281	hqer_induced sampler prototype	2.0
	WDI_agriculture	Quantile-SFA	hqer_induced	True	29	-704.4244645447804	hqer_induced sampler prototype	1.0
	WDI_agriculture	HQER-SFA	hqer_induced	True	25	-704.4459874574484	hqer_induced sampler prototype	2.0
	WDI_agriculture	Quantile-SFA	hqer_induced	True	33	-680.3473208330599	hqer_induced sampler prototype	1.0

experiment	dataset	model	scenario	converged	n_iter	loglik	warning_message	best_start_id
	WDI_agriculture	HQER-SFA	hqer_induced	True	47	-680.4127333674088	hqer_induced sampler prototype	1.0
	WDI_agriculture	Quantile-SFA	hqer_induced	True	16	-703.4887645881525	hqer_induced sampler prototype	2.0
	WDI_agriculture	HQER-SFA	hqer_induced	True	12	-703.6306733090225	hqer_induced sampler prototype	2.0
	WDI_agriculture	Quantile-SFA	hqer_induced	True	30	-711.2234826343616	hqer_induced sampler prototype	5.0
	WDI_agriculture	HQER-SFA	hqer_induced	True	30	-711.2252631444503	hqer_induced sampler prototype	3.0
	WDI_agriculture	Quantile-SFA	hqer_induced	True	27	-690.4265448129734	hqer_induced sampler prototype	1.0
	WDI_agriculture	HQER-SFA	hqer_induced	True	22	-690.3996846256543	hqer_induced sampler prototype	3.0
	WDI_agriculture	Quantile-SFA	hqer_induced	True	28	-735.5342094077964	hqer_induced sampler prototype	5.0
	WDI_agriculture	HQER-SFA	hqer_induced	True	21	-735.5102338902982	hqer_induced sampler prototype	1.0
	WDI_agriculture	Quantile-SFA	hqer_induced	True	14	-736.3057031645164	hqer_induced sampler prototype	4.0
	WDI_agriculture	HQER-SFA	hqer_induced	True	12	-736.230454993634	hqer_induced sampler prototype	4.0
	WDI_agriculture	Quantile-SFA	hqer_induced	True	20	-732.8870488189393	hqer_induced sampler prototype	1.0
	WDI_agriculture	HQER-SFA	hqer_induced	True	27	-732.8667720387709	hqer_induced sampler prototype	5.0
	WDI_agriculture	Quantile-SFA	hqer_induced	True	15	-679.4626508485328	hqer_induced sampler prototype	4.0
	WDI_agriculture	HQER-SFA	hqer_induced	True	11	-679.3900187475226	hqer_induced sampler prototype	4.0
	WDI_agriculture	Quantile-SFA	hqer_induced	True	34	-669.1977876922573	hqer_induced sampler prototype	1.0
	WDI_agriculture	HQER-SFA	hqer_induced	True	16	-669.2243012913178	hqer_induced sampler prototype	2.0
	WDI_agriculture	Quantile-SFA	hqer_induced	True	25	-706.7279806231343	hqer_induced sampler prototype	2.0
	WDI_agriculture	HQER-SFA	hqer_induced	True	30	-706.7196911594443	hqer_induced sampler prototype	5.0
	WDI_agriculture	Quantile-SFA	hqer_induced	True	31	-736.4465576877702	hqer_induced sampler prototype	1.0
	WDI_agriculture	HQER-SFA	hqer_induced	True	26	-736.4337064484289	hqer_induced sampler prototype	3.0
	WDI_agriculture	Quantile-SFA	hqer_induced	True	15	-699.2787092257919	hqer_induced sampler prototype	5.0
	WDI_agriculture	HQER-SFA	hqer_induced	True	16	-699.2875508734152	hqer_induced sampler prototype	5.0
	WDI_agriculture	Quantile-SFA	hqer_induced	True	32	-732.4733195478804	hqer_induced sampler prototype	3.0
	WDI_agriculture	HQER-SFA	hqer_induced	True	27	-732.4041503758295	hqer_induced sampler prototype	3.0
	WDI_agriculture	Quantile-SFA	hqer_induced	True	28	-726.256889118593	hqer_induced sampler prototype	3.0
	WDI_agriculture	HQER-SFA	hqer_induced	True	22	-726.2363302205138	hqer_induced sampler prototype	1.0
	WDI_agriculture	Quantile-SFA	hqer_induced	True	28	-701.8487945680045	hqer_induced sampler prototype	1.0
	WDI_agriculture	HQER-SFA	hqer_induced	True	29	-701.8151799287027	hqer_induced sampler prototype	1.0
	WDI_agriculture	Quantile-SFA	hqer_induced	True	26	-715.4341929426737	hqer_induced sampler prototype	3.0
	WDI_agriculture	HQER-SFA	hqer_induced	True	30	-715.4277215834409	hqer_induced sampler prototype	3.0
	WDI_agriculture	Quantile-SFA	hqer_induced	True	45	-684.7630799923695	hqer_induced sampler prototype	1.0
	WDI_agriculture	HQER-SFA	hqer_induced	True	27	-684.7590417434195	hqer_induced sampler prototype	3.0
	WDI_agriculture	Quantile-SFA	hqer_induced	True	29	-696.7707205098884	hqer_induced sampler prototype	1.0
	WDI_agriculture	HQER-SFA	hqer_induced	True	23	-696.7245309748296	hqer_induced sampler prototype	5.0
	WDI_agriculture	Quantile-SFA	hqer_induced	True	27	-712.2157447508914	hqer_induced sampler prototype	2.0
	WDI_agriculture	HQER-SFA	hqer_induced	True	28	-712.1848659767574	hqer_induced sampler prototype	2.0
	WDI_agriculture	Quantile-SFA	hqer_induced	True	15	-705.1020436053401	hqer_induced sampler prototype	5.0
	WDI_agriculture	HQER-SFA	hqer_induced	True	17	-705.0848791393232	hqer_induced sampler prototype	5.0
	WDI_agriculture	Quantile-SFA	hqer_induced	True	26	-708.6713344048728	hqer_induced sampler prototype	3.0
	WDI_agriculture	HQER-SFA	hqer_induced	True	32	-708.6417076979171	hqer_induced sampler prototype	1.0
	WDI_agriculture	Quantile-SFA	hqer_induced	True	39	-734.930379803667	hqer_induced sampler prototype	1.0
	WDI_agriculture	HQER-SFA	hqer_induced	True	35	-734.9085262702026	hqer_induced sampler prototype	3.0
	WDI_agriculture	Quantile-SFA	hqer_induced	True	30	-724.4568646608021	hqer_induced sampler prototype	5.0
	WDI_agriculture	HQER-SFA	hqer_induced	True	45	-723.7085059676351	hqer_induced sampler prototype	1.0
	WDI_agriculture	Quantile-SFA	hqer_induced	True	24	-703.7502474643599	hqer_induced sampler prototype	3.0
	WDI_agriculture	HQER-SFA	hqer_induced	True	18	-703.7403180282372	hqer_induced sampler prototype	3.0
	WDI_agriculture	Quantile-SFA	hqer_induced	True	59	-701.1323396393221	hqer_induced sampler prototype	3.0
	WDI_agriculture	HQER-SFA	hqer_induced	True	37	-701.1177148797115	hqer_induced sampler prototype	5.0
	WDI_agriculture	Quantile-SFA	hqer_induced	True	18	-712.9600808081635	hqer_induced sampler prototype	2.0
	WDI_agriculture	HQER-SFA	hqer_induced	True	18	-712.9459370351627	hqer_induced sampler prototype	5.0
	WDI_agriculture	Quantile-SFA	hqer_induced	True	28	-717.6125272187071	hqer_induced sampler prototype	2.0
	WDI_agriculture	HQER-SFA	hqer_induced	True	25	-718.0791529500962	hqer_induced sampler prototype	3.0
	WDI_agriculture	Quantile-SFA	hqer_induced	True	20	-707.8739631117538	hqer_induced sampler prototype	2.0
	WDI_agriculture	HQER-SFA	hqer_induced	True	23	-707.8542947339191	hqer_induced sampler prototype	1.0
	WDI_agriculture	Quantile-SFA	hqer_induced	True	29	-732.3907059490516	hqer_induced sampler prototype	1.0
	WDI_agriculture	HQER-SFA	hqer_induced	True	23	-732.3752612290734	hqer_induced sampler prototype	1.0
	WDI_agriculture	Quantile-SFA	hqer_induced	True	32	-718.358135868662	hqer_induced sampler prototype	1.0
	WDI_agriculture	HQER-SFA	hqer_induced	True	34	-718.3655356596828	hqer_induced sampler prototype	3.0
	WDI_agriculture	Quantile-SFA	hqer_induced	True	26	-737.5914380847917	hqer_induced sampler prototype	1.0
	WDI_agriculture	HQER-SFA	hqer_induced	True	30	-737.5692670777385	hqer_induced sampler prototype	1.0
	WDI_agriculture	Quantile-SFA	hqer_induced	True	12	-750.2896116423192	hqer_induced sampler prototype	2.0
	WDI_agriculture	HQER-SFA	hqer_induced	True	19	-750.2625845347798	hqer_induced sampler prototype	3.0
	WDI_agriculture	Quantile-SFA	hqer_induced	True	10	-713.6916143404515	hqer_induced sampler prototype	2.0
	WDI_agriculture	HQER-SFA	hqer_induced	True	10	-713.6591239668712	hqer_induced sampler prototype	2.0
	WDI_agriculture	Quantile-SFA	hqer_induced	True	33	-731.0872981298014	hqer_induced sampler prototype	2.0
	WDI_agriculture	HQER-SFA	hqer_induced	True	22	-731.0554139326928	hqer_induced sampler prototype	1.0
	WDI_agriculture	Quantile-SFA	hqer_induced	True	31	-727.0702084466789	hqer_induced sampler prototype	3.0
	WDI_agriculture	HQER-SFA	hqer_induced	True	33	-727.0536776863538	hqer_induced sampler prototype	1.0
	WDI_agriculture	Quantile-SFA	hqer_induced	True	36	-709.005291960052	hqer_induced sampler prototype	2.0
	WDI_agriculture	HQER-SFA	hqer_induced	True	34	-709.0210211467272	hqer_induced sampler prototype	1.0
	WDI_agriculture	Quantile-SFA	hqer_induced	True	28	-731.7115633566325	hqer_induced sampler prototype	3.0
	WDI_agriculture	HQER-SFA	hqer_induced	True	23	-731.7061954730209	hqer_induced sampler prototype	1.0
	WDI_agriculture	Quantile-SFA	hqer_induced	True	28	-689.581156466027	hqer_induced sampler prototype	2.0
	WDI_agriculture	HQER-SFA	hqer_induced	True	32	-689.4733841209921	hqer_induced sampler prototype	2.0
	WDI_agriculture	Quantile-SFA	hqer_induced	True	21	-711.3588379532148	hqer_induced sampler prototype	2.0
	WDI_agriculture	HQER-SFA	hqer_induced	True	14	-711.3634024078723	hqer_induced sampler prototype	5.0
	WDI_agriculture	Quantile-SFA	hqer_induced	True	36	-729.5033016951381	hqer_induced sampler prototype	3.0
	WDI_agriculture	HQER-SFA	hqer_induced	True	29	-729.6755301012782	hqer_induced sampler prototype	2.0
	WDI_agriculture	Quantile-SFA	hqer_induced	True	28	-707.2198717509707	hqer_induced sampler prototype	3.0
	WDI_agriculture	HQER-SFA	hqer_induced	True	28	-707.705134562488	hqer_induced sampler prototype	5.0
	WDI_agriculture	Quantile-SFA	hqer_induced	True	20	-718.5338591034823	hqer_induced sampler prototype	5.0
	WDI_agriculture	HQER-SFA	hqer_induced	True	18	-718.505022740382	hqer_induced sampler prototype	5.0
	WDI_agriculture	Quantile-SFA	hqer_induced	True	23	-706.0112539982272	hqer_induced sampler prototype	3.0
	WDI_agriculture	HQER-SFA	hqer_induced	True	19	-705.9993754002976	hqer_induced sampler prototype	2.0
	WDI_agriculture	Quantile-SFA	hqer_induced	True	26	-708.20406514106	hqer_induced sampler prototype	1.0
	WDI_agriculture	HQER-SFA	hqer_induced	True	20	-708.1997331985804	hqer_induced sampler prototype	3.0
	WDI_agriculture	Quantile-SFA	hqer_induced	True	31	-691.6112589691953	hqer_induced sampler prototype	5.0
	WDI_agriculture	HQER-SFA	hqer_induced	True	29	-691.6716978869864	hqer_induced sampler prototype	2.0
	WDI_agriculture	Quantile-SFA	hqer_induced	True	24	-2875.5089335582115	hqer_induced sampler prototype	5.0
	WDI_agriculture	HQER-SFA	hqer_induced	True	25	-2875.4440496422885	hqer_induced sampler prototype	2.0
	WDI_agriculture	Quantile-SFA	hqer_induced	True	21	-2795.913849676203	hqer_induced sampler prototype	5.0
	WDI_agriculture	HQER-SFA	hqer_induced	True	25	-2795.7176182744406	hqer_induced sampler prototype	1.0
	WDI_agriculture	Quantile-SFA	hqer_induced	True	14	-2836.50395352509	hqer_induced sampler prototype	2.0
	WDI_agriculture	HQER-SFA	hqer_induced	True	27	-2836.438173850201	hqer_induced sampler prototype	1.0
	WDI_agriculture	Quantile-SFA	hqer_induced	True	13	-2813.1075867674763	hqer_induced sampler prototype	2.0
	WDI_agriculture	HQER-SFA	hqer_induced	True	9	-2813.0742203298123	hqer_induced sampler prototype	2.0
	WDI_agriculture	Quantile-SFA	hqer_induced	True	19	-2876.439479705072	hqer_induced sampler prototype	2.0
	WDI_agriculture	HQER-SFA	hqer_induced	True	23	-2876.338784026094	hqer_induced sampler prototype	5.0
	WDI_agriculture	Quantile-SFA	hqer_induced	True	32	-2854.3692010646114	hqer_induced sampler prototype	3.0
	WDI_agriculture	HQER-SFA	hqer_induced	True	21	-2854.3121529917453	hqer_induced sampler prototype	5.0
	WDI_agriculture	Quantile-SFA	hqer_induced	True	26	-2821.164082961081	hqer_induced sampler prototype	3.0
	WDI_agriculture	HQER-SFA	hqer_induced	True	17	-2821.135834581198	hqer_induced sampler prototype	2.0

experiment	dataset	model	scenario	converged	n_iter	loglik	warning_message	best_start_id
	WDI_agriculture	Quantile-SFA	hqer_induced	True	26	-2910.069842752542	hqer_induced sampler prototype	1.0
	WDI_agriculture	HQER-SFA	hqer_induced	True	26	-2909.993349275496	hqer_induced sampler prototype	1.0
	WDI_agriculture	Quantile-SFA	hqer_induced	True	30	-2867.508092465787	hqer_induced sampler prototype	1.0
	WDI_agriculture	HQER-SFA	hqer_induced	True	23	-2867.591974965141	hqer_induced sampler prototype	3.0
	WDI_agriculture	Quantile-SFA	hqer_induced	True	27	-2876.8515945200047	hqer_induced sampler prototype	5.0
	WDI_agriculture	HQER-SFA	hqer_induced	True	21	-2876.746643518113	hqer_induced sampler prototype	5.0
	WDI_agriculture	Quantile-SFA	hqer_induced	True	25	-2863.988232270042	hqer_induced sampler prototype	2.0
	WDI_agriculture	HQER-SFA	hqer_induced	True	23	-2863.956534655548	hqer_induced sampler prototype	2.0
	WDI_agriculture	Quantile-SFA	hqer_induced	True	35	-2845.0879509337565	hqer_induced sampler prototype	1.0
	WDI_agriculture	HQER-SFA	hqer_induced	True	21	-2845.063435747592	hqer_induced sampler prototype	3.0
	WDI_agriculture	Quantile-SFA	hqer_induced	True	25	-2818.0982623619816	hqer_induced sampler prototype	3.0
	WDI_agriculture	HQER-SFA	hqer_induced	True	17	-2818.115277141547	hqer_induced sampler prototype	5.0
	WDI_agriculture	Quantile-SFA	hqer_induced	True	19	-2849.317771502904	hqer_induced sampler prototype	2.0
	WDI_agriculture	HQER-SFA	hqer_induced	True	30	-2849.27172211006	hqer_induced sampler prototype	5.0
	WDI_agriculture	Quantile-SFA	hqer_induced	True	28	-2860.7563036211154	hqer_induced sampler prototype	5.0
	WDI_agriculture	HQER-SFA	hqer_induced	True	15	-2860.6700407986827	hqer_induced sampler prototype	2.0
	WDI_agriculture	Quantile-SFA	hqer_induced	True	22	-2836.025194319098	hqer_induced sampler prototype	3.0
	WDI_agriculture	HQER-SFA	hqer_induced	True	21	-2835.902602379151	hqer_induced sampler prototype	1.0
	WDI_agriculture	Quantile-SFA	hqer_induced	True	22	-2927.8723020827542	hqer_induced sampler prototype	3.0
	WDI_agriculture	HQER-SFA	hqer_induced	True	29	-2927.797088806742	hqer_induced sampler prototype	1.0
	WDI_agriculture	Quantile-SFA	hqer_induced	True	19	-2891.4574762146785	hqer_induced sampler prototype	1.0
	WDI_agriculture	HQER-SFA	hqer_induced	True	27	-2891.383170543362	hqer_induced sampler prototype	3.0
	WDI_agriculture	Quantile-SFA	hqer_induced	True	18	-2840.403945566145	hqer_induced sampler prototype	2.0
	WDI_agriculture	HQER-SFA	hqer_induced	True	23	-2840.261067347738	hqer_induced sampler prototype	1.0
	WDI_agriculture	Quantile-SFA	hqer_induced	True	15	-2830.354063264306	hqer_induced sampler prototype	5.0
	WDI_agriculture	HQER-SFA	hqer_induced	True	27	-2830.238319809303	hqer_induced sampler prototype	2.0
	WDI_agriculture	Quantile-SFA	hqer_induced	True	22	-2910.942546763502	hqer_induced sampler prototype	3.0
	WDI_agriculture	HQER-SFA	hqer_induced	True	11	-2910.935156418938	hqer_induced sampler prototype	2.0
	WDI_agriculture	Quantile-SFA	hqer_induced	True	18	-2828.203611457636	hqer_induced sampler prototype	5.0
	WDI_agriculture	HQER-SFA	hqer_induced	True	18	-2828.090005802281	hqer_induced sampler prototype	1.0
	WDI_agriculture	Quantile-SFA	hqer_induced	True	24	-2852.1628556287205	hqer_induced sampler prototype	1.0
	WDI_agriculture	HQER-SFA	hqer_induced	True	28	-2852.089037399731	hqer_induced sampler prototype	3.0
	WDI_agriculture	Quantile-SFA	hqer_induced	True	12	-2782.962426527218	hqer_induced sampler prototype	2.0
	WDI_agriculture	HQER-SFA	hqer_induced	True	9	-2782.909533237426	hqer_induced sampler prototype	2.0
	WDI_agriculture	Quantile-SFA	hqer_induced	True	26	-2868.1717599171334	hqer_induced sampler prototype	3.0
	WDI_agriculture	HQER-SFA	hqer_induced	True	21	-2868.128194424249	hqer_induced sampler prototype	1.0
	WDI_agriculture	Quantile-SFA	hqer_induced	True	21	-2905.56780107911	hqer_induced sampler prototype	3.0
	WDI_agriculture	HQER-SFA	hqer_induced	True	26	-2905.481967859887	hqer_induced sampler prototype	5.0
	WDI_agriculture	Quantile-SFA	hqer_induced	True	16	-2861.0827413228144	hqer_induced sampler prototype	1.0
	WDI_agriculture	HQER-SFA	hqer_induced	True	20	-2860.7517859479867	hqer_induced sampler prototype	3.0
	WDI_agriculture	Quantile-SFA	hqer_induced	True	19	-2878.046701846989	hqer_induced sampler prototype	2.0
	WDI_agriculture	HQER-SFA	hqer_induced	True	21	-2877.9314172809454	hqer_induced sampler prototype	1.0
	WDI_agriculture	Quantile-SFA	hqer_induced	True	23	-2838.072777689255	hqer_induced sampler prototype	2.0
	WDI_agriculture	HQER-SFA	hqer_induced	True	19	-2837.99197805778	hqer_induced sampler prototype	2.0
	WDI_agriculture	Quantile-SFA	hqer_induced	True	23	-2874.640120494968	hqer_induced sampler prototype	1.0
	WDI_agriculture	HQER-SFA	hqer_induced	True	15	-2874.539557607118	hqer_induced sampler prototype	2.0
	WDI_agriculture	Quantile-SFA	hqer_induced	True	26	-2851.6683387926896	hqer_induced sampler prototype	1.0
	WDI_agriculture	HQER-SFA	hqer_induced	True	30	-2851.594282576117	hqer_induced sampler prototype	1.0
	WDI_agriculture	Quantile-SFA	hqer_induced	True	23	-2878.3543538187164	hqer_induced sampler prototype	1.0
	WDI_agriculture	HQER-SFA	hqer_induced	True	25	-2878.2264507272234	hqer_induced sampler prototype	1.0
	WDI_agriculture	Quantile-SFA	hqer_induced	True	23	-2882.1576671516195	hqer_induced sampler prototype	3.0
	WDI_agriculture	HQER-SFA	hqer_induced	True	30	-2882.086805272522	hqer_induced sampler prototype	3.0
	WDI_agriculture	Quantile-SFA	hqer_induced	True	26	-2885.1284496091275	hqer_induced sampler prototype	2.0
	WDI_agriculture	HQER-SFA	hqer_induced	True	22	-2885.22106103404	hqer_induced sampler prototype	1.0
	WDI_agriculture	Quantile-SFA	hqer_induced	True	20	-2857.023474246768	hqer_induced sampler prototype	1.0
	WDI_agriculture	HQER-SFA	hqer_induced	True	18	-2856.9872411271863	hqer_induced sampler prototype	3.0
	WDI_agriculture	Quantile-SFA	hqer_induced	True	21	-2877.622895074416	hqer_induced sampler prototype	1.0
	WDI_agriculture	HQER-SFA	hqer_induced	True	23	-2877.5613019156963	hqer_induced sampler prototype	3.0
	WDI_agriculture	Quantile-SFA	hqer_induced	True	17	-2862.573189254706	hqer_induced sampler prototype	1.0
	WDI_agriculture	HQER-SFA	hqer_induced	True	38	-2862.460215294625	hqer_induced sampler prototype	5.0
	WDI_agriculture	Quantile-SFA	hqer_induced	True	25	-2865.9361146379333	hqer_induced sampler prototype	3.0
	WDI_agriculture	HQER-SFA	hqer_induced	True	27	-2865.9133169349	hqer_induced sampler prototype	1.0
	WDI_agriculture	Quantile-SFA	hqer_induced	True	19	-2862.288056184506	hqer_induced sampler prototype	1.0
	WDI_agriculture	HQER-SFA	hqer_induced	True	21	-2862.244884586796	hqer_induced sampler prototype	5.0
	WDI_agriculture	Quantile-SFA	hqer_induced	True	18	-2902.7189036278164	hqer_induced sampler prototype	3.0
	WDI_agriculture	HQER-SFA	hqer_induced	True	19	-2902.253434469822	hqer_induced sampler prototype	3.0
	WDI_agriculture	Quantile-SFA	hqer_induced	True	18	-2873.9746584260693	hqer_induced sampler prototype	2.0
	WDI_agriculture	HQER-SFA	hqer_induced	True	22	-2873.8785635166214	hqer_induced sampler prototype	2.0
	WDI_agriculture	Quantile-SFA	hqer_induced	True	23	-2885.544185300587	hqer_induced sampler prototype	1.0
	WDI_agriculture	HQER-SFA	hqer_induced	True	23	-2885.4056868380435	hqer_induced sampler prototype	5.0
	WDI_agriculture	Quantile-SFA	hqer_induced	True	21	-2820.350327955719	hqer_induced sampler prototype	3.0
	WDI_agriculture	HQER-SFA	hqer_induced	True	15	-2820.22437441715	hqer_induced sampler prototype	5.0
	WDI_agriculture	Quantile-SFA	hqer_induced	True	29	-2847.4904129928104	hqer_induced sampler prototype	3.0
	WDI_agriculture	HQER-SFA	hqer_induced	True	15	-2847.432036988682	hqer_induced sampler prototype	5.0
	WDI_agriculture	Quantile-SFA	hqer_induced	True	23	-2831.3421720455945	hqer_induced sampler prototype	3.0
	WDI_agriculture	HQER-SFA	hqer_induced	True	8	-2831.30195263285	hqer_induced sampler prototype	2.0
	WDI_agriculture	Quantile-SFA	hqer_induced	True	24	-2808.651514009085	hqer_induced sampler prototype	3.0
	WDI_agriculture	HQER-SFA	hqer_induced	True	22	-2808.6003966872227	hqer_induced sampler prototype	1.0
	WDI_agriculture	Quantile-SFA	hqer_induced	True	25	-2813.371464424424	hqer_induced sampler prototype	5.0
	WDI_agriculture	HQER-SFA	hqer_induced	True	27	-2813.277017938476	hqer_induced sampler prototype	5.0
	WDI_agriculture	Quantile-SFA	hqer_induced	True	51	-2881.2159235958484	hqer_induced sampler prototype	1.0
	WDI_agriculture	HQER-SFA	hqer_induced	True	13	-2881.1951545381503	hqer_induced sampler prototype	5.0
	WDI_agriculture	Quantile-SFA	hqer_induced	True	19	-2890.9471405894765	hqer_induced sampler prototype	1.0
	WDI_agriculture	HQER-SFA	hqer_induced	True	21	-2890.849946517153	hqer_induced sampler prototype	1.0
	WDI_agriculture	Quantile-SFA	hqer_induced	True	15	-2848.267979991046	hqer_induced sampler prototype	2.0
	WDI_agriculture	HQER-SFA	hqer_induced	True	13	-2848.1889324807225	hqer_induced sampler prototype	2.0
	WDI_agriculture	Quantile-SFA	hqer_induced	True	20	-2851.2631815898103	hqer_induced sampler prototype	2.0
	WDI_agriculture	HQER-SFA	hqer_induced	True	18	-2851.183630690238	hqer_induced sampler prototype	2.0
	WDI_agriculture	Quantile-SFA	hqer_induced	True	26	-2905.5886903964483	hqer_induced sampler prototype	5.0
	WDI_agriculture	HQER-SFA	hqer_induced	True	32	-2905.5185202252032	hqer_induced sampler prototype	1.0
	WDI_agriculture	Quantile-SFA	hqer_induced	True	18	-2913.578558892847	hqer_induced sampler prototype	5.0
	WDI_agriculture	HQER-SFA	hqer_induced	True	24	-2913.4410362083618	hqer_induced sampler prototype	1.0
	WDI_agriculture	Quantile-SFA	hqer_induced	True	24	-2846.656271690361	hqer_induced sampler prototype	3.0
	WDI_agriculture	HQER-SFA	hqer_induced	True	23	-2846.660738460919	hqer_induced sampler prototype	1.0
	WDI_agriculture	Quantile-SFA	hqer_induced	True	27	-2892.75437313411	hqer_induced sampler prototype	3.0
	WDI_agriculture	HQER-SFA	hqer_induced	True	12	-2892.6593478572886	hqer_induced sampler prototype	2.0
	WDI_agriculture	Quantile-SFA	hqer_induced	True	25	-2884.9442362678647	hqer_induced sampler prototype	5.0
	WDI_agriculture	HQER-SFA	hqer_induced	True	25	-2884.861942859762	hqer_induced sampler prototype	1.0
	WDI_agriculture	Quantile-SFA	hqer_induced	True	23	-2864.1789609395314	hqer_induced sampler prototype	3.0
	WDI_agriculture	HQER-SFA	hqer_induced	True	15	-2864.138658385559	hqer_induced sampler prototype	2.0
	WDI_agriculture	Quantile-SFA	hqer_induced	True	10	-2893.439935235364	hqer_induced sampler prototype	2.0
	WDI_agriculture	HQER-SFA	hqer_induced	True	19	-2893.334259111324	hqer_induced sampler prototype	5.0
	WDI_agriculture	Quantile-SFA	hqer_induced	True	21	-2842.268623178161	hqer_induced sampler prototype	1.0

experiment	dataset	model	scenario	converged	n__iter	loglik	warning__message	best__start__id
	WDI_agriculture	HQER-SFA	hqer__induced	True	19	-2842.2050153439777	hqer__induced sampler prototype	2.0
	WDI_agriculture	Quantile-SFA	hqer__induced	True	23	-2854.755349632144	hqer__induced sampler prototype	3.0
	WDI_agriculture	HQER-SFA	hqer__induced	True	19	-2854.7479694075564	hqer__induced sampler prototype	5.0
	WDI_agriculture	Quantile-SFA	hqer__induced	True	28	-2849.674249836348	hqer__induced sampler prototype	1.0
	WDI_agriculture	HQER-SFA	hqer__induced	True	23	-2849.6030273353813	hqer__induced sampler prototype	5.0
	WDI_agriculture	Quantile-SFA	hqer__induced	True	30	-2858.5247321742	hqer__induced sampler prototype	3.0
	WDI_agriculture	HQER-SFA	hqer__induced	True	15	-2858.4685669076844	hqer__induced sampler prototype	2.0
	WDI_agriculture	Quantile-SFA	hqer__induced	True	20	-2810.1899865763667	hqer__induced sampler prototype	3.0
	WDI_agriculture	HQER-SFA	hqer__induced	True	27	-2810.153455163144	hqer__induced sampler prototype	3.0
	WDI_agriculture	Quantile-SFA	hqer__induced	True	35	-2821.6594434653707	hqer__induced sampler prototype	1.0
	WDI_agriculture	HQER-SFA	hqer__induced	True	25	-2821.5844948941217	hqer__induced sampler prototype	3.0
	WDI_agriculture	Quantile-SFA	hqer__induced	True	24	-2841.0984939783284	hqer__induced sampler prototype	5.0
	WDI_agriculture	HQER-SFA	hqer__induced	True	23	-2841.006831633592	hqer__induced sampler prototype	3.0
	WDI_agriculture	Quantile-SFA	hqer__induced	True	30	-2866.721258423653	hqer__induced sampler prototype	1.0
	WDI_agriculture	HQER-SFA	hqer__induced	True	20	-2866.758725347049	hqer__induced sampler prototype	1.0
	WDI_agriculture	Quantile-SFA	hqer__induced	True	36	-2851.694771995094	hqer__induced sampler prototype	1.0
	WDI_agriculture	HQER-SFA	hqer__induced	True	17	-2851.632986854338	hqer__induced sampler prototype	5.0
	WDI_agriculture	Quantile-SFA	hqer__induced	True	19	-2878.63873394042	hqer__induced sampler prototype	2.0
	WDI_agriculture	HQER-SFA	hqer__induced	True	21	-2878.915566476937	hqer__induced sampler prototype	2.0
	WDI_agriculture	Quantile-SFA	hqer__induced	True	17	-2827.039528838576	hqer__induced sampler prototype	3.0
	WDI_agriculture	HQER-SFA	hqer__induced	True	20	-2826.9891613373584	hqer__induced sampler prototype	2.0
	WDI_agriculture	Quantile-SFA	hqer__induced	True	16	-2874.1813319668368	hqer__induced sampler prototype	5.0
	WDI_agriculture	HQER-SFA	hqer__induced	True	13	-2874.0951304826567	hqer__induced sampler prototype	5.0
	WDI_agriculture	Quantile-SFA	hqer__induced	True	23	-2891.899980606599	hqer__induced sampler prototype	3.0
	WDI_agriculture	HQER-SFA	hqer__induced	True	21	-2891.8991142423874	hqer__induced sampler prototype	5.0
	WDI_agriculture	Quantile-SFA	hqer__induced	True	27	-2944.360748961448	hqer__induced sampler prototype	1.0
	WDI_agriculture	HQER-SFA	hqer__induced	True	23	-2944.255426083125	hqer__induced sampler prototype	3.0
	WDI_agriculture	Quantile-SFA	hqer__induced	True	16	-2846.8762336354225	hqer__induced sampler prototype	2.0
	WDI_agriculture	HQER-SFA	hqer__induced	True	21	-2846.808196243277	hqer__induced sampler prototype	1.0
	WDI_agriculture	Quantile-SFA	hqer__induced	True	19	-2879.0279167368776	hqer__induced sampler prototype	2.0
	WDI_agriculture	HQER-SFA	hqer__induced	True	29	-2878.965826028348	hqer__induced sampler prototype	1.0
	WDI_agriculture	Quantile-SFA	hqer__induced	True	16	-2888.5564540241503	hqer__induced sampler prototype	5.0
	WDI_agriculture	HQER-SFA	hqer__induced	True	21	-2888.4698180822315	hqer__induced sampler prototype	5.0
	WDI_agriculture	Quantile-SFA	hqer__induced	True	24	-2875.1272606615835	hqer__induced sampler prototype	1.0
	WDI_agriculture	HQER-SFA	hqer__induced	True	19	-2875.114064348543	hqer__induced sampler prototype	2.0
	WDI_agriculture	Quantile-SFA	hqer__induced	True	21	-2911.0396166100763	hqer__induced sampler prototype	1.0
	WDI_agriculture	HQER-SFA	hqer__induced	True	20	-2910.941775743756	hqer__induced sampler prototype	1.0
	WDI_agriculture	Quantile-SFA	hqer__induced	True	22	-2932.0828257416706	hqer__induced sampler prototype	1.0
	WDI_agriculture	HQER-SFA	hqer__induced	True	23	-2931.976044797493	hqer__induced sampler prototype	1.0
	WDI_agriculture	Quantile-SFA	hqer__induced	True	15	-2807.546377771546	hqer__induced sampler prototype	5.0
	WDI_agriculture	HQER-SFA	hqer__induced	True	21	-2807.5116447756645	hqer__induced sampler prototype	3.0
	WDI_agriculture	Quantile-SFA	hqer__induced	True	15	-2811.4509528050053	hqer__induced sampler prototype	5.0
	WDI_agriculture	HQER-SFA	hqer__induced	True	26	-2811.3476748129788	hqer__induced sampler prototype	3.0
	WDI_agriculture	Quantile-SFA	hqer__induced	True	25	-2821.6848176095987	hqer__induced sampler prototype	1.0
	WDI_agriculture	HQER-SFA	hqer__induced	True	22	-2821.647549969551	hqer__induced sampler prototype	2.0
	WDI_agriculture	Quantile-SFA	hqer__induced	True	35	-2784.062484863426	hqer__induced sampler prototype	1.0
	WDI_agriculture	HQER-SFA	hqer__induced	True	12	-2783.996887575329	hqer__induced sampler prototype	2.0
	WDI_agriculture	Quantile-SFA	hqer__induced	True	27	-2853.920683117935	hqer__induced sampler prototype	5.0
	WDI_agriculture	HQER-SFA	hqer__induced	True	25	-2853.822844562207	hqer__induced sampler prototype	5.0
	WDI_agriculture	Quantile-SFA	hqer__induced	True	10	-2885.706836847767	hqer__induced sampler prototype	5.0
	WDI_agriculture	HQER-SFA	hqer__induced	True	21	-2885.357758817279	hqer__induced sampler prototype	5.0
	WDI_agriculture	Quantile-SFA	hqer__induced	True	27	-2898.152933240321	hqer__induced sampler prototype	5.0
	WDI_agriculture	HQER-SFA	hqer__induced	True	16	-2898.0536130529963	hqer__induced sampler prototype	2.0
	WDI_agriculture	Quantile-SFA	hqer__induced	True	25	-2857.072424521995	hqer__induced sampler prototype	5.0
	WDI_agriculture	HQER-SFA	hqer__induced	True	23	-2856.9797026083	hqer__induced sampler prototype	2.0
	WDI_agriculture	Quantile-SFA	hqer__induced	True	15	-2849.9307777949184	hqer__induced sampler prototype	2.0
	WDI_agriculture	HQER-SFA	hqer__induced	True	20	-2849.8605378462144	hqer__induced sampler prototype	2.0
	WDI_agriculture	Quantile-SFA	hqer__induced	True	24	-2819.73346067685	hqer__induced sampler prototype	5.0
	WDI_agriculture	HQER-SFA	hqer__induced	True	29	-2819.6548739528826	hqer__induced sampler prototype	5.0
	WDI_agriculture	Quantile-SFA	hqer__induced	True	21	-2874.473554837591	hqer__induced sampler prototype	2.0
	WDI_agriculture	HQER-SFA	hqer__induced	True	24	-2874.398179513993	hqer__induced sampler prototype	5.0
	WDI_agriculture	Quantile-SFA	hqer__induced	True	18	-2897.421979375505	hqer__induced sampler prototype	5.0
	WDI_agriculture	HQER-SFA	hqer__induced	True	16	-2897.3724319079547	hqer__induced sampler prototype	5.0
	WDI_agriculture	Quantile-SFA	hqer__induced	True	22	-2822.8002972469053	hqer__induced sampler prototype	1.0
	WDI_agriculture	HQER-SFA	hqer__induced	True	12	-2822.7242054637422	hqer__induced sampler prototype	2.0
	WDI_agriculture	Quantile-SFA	hqer__induced	True	25	-2905.6619141614274	hqer__induced sampler prototype	3.0
	WDI_agriculture	HQER-SFA	hqer__induced	True	23	-2905.590079901298	hqer__induced sampler prototype	2.0
	WDI_agriculture	Quantile-SFA	hqer__induced	True	23	-2865.3773878860284	hqer__induced sampler prototype	1.0
	WDI_agriculture	HQER-SFA	hqer__induced	True	25	-2865.2640987896684	hqer__induced sampler prototype	5.0
	WDI_agriculture	Quantile-SFA	hqer__induced	True	28	-2895.9528561008865	hqer__induced sampler prototype	3.0
	WDI_agriculture	HQER-SFA	hqer__induced	True	21	-2896.145770476948	hqer__induced sampler prototype	1.0
	WDI_agriculture	Quantile-SFA	hqer__induced	True	25	-2832.1886712868327	hqer__induced sampler prototype	1.0
	WDI_agriculture	HQER-SFA	hqer__induced	True	11	-2832.119090092705	hqer__induced sampler prototype	2.0
	WDI_agriculture	Quantile-SFA	hqer__induced	True	24	-2935.479136853732	hqer__induced sampler prototype	3.0
	WDI_agriculture	HQER-SFA	hqer__induced	True	23	-2935.3832477818232	hqer__induced sampler prototype	3.0
	WDI_agriculture	Quantile-SFA	hqer__induced	True	8	-2898.949854603261	hqer__induced sampler prototype	2.0
	WDI_agriculture	HQER-SFA	hqer__induced	True	26	-2898.8128000418783	hqer__induced sampler prototype	1.0
	WDI_agriculture	Quantile-SFA	hqer__induced	True	23	-2871.5333308623376	hqer__induced sampler prototype	1.0
	WDI_agriculture	HQER-SFA	hqer__induced	True	12	-2871.487634046931	hqer__induced sampler prototype	2.0
	WDI_agriculture	Quantile-SFA	hqer__induced	True	24	-2855.1225247829284	hqer__induced sampler prototype	5.0
	WDI_agriculture	HQER-SFA	hqer__induced	True	23	-2855.026910709903	hqer__induced sampler prototype	2.0
	WDI_agriculture	Quantile-SFA	hqer__induced	True	25	-2904.6063252087697	hqer__induced sampler prototype	1.0
	WDI_agriculture	HQER-SFA	hqer__induced	True	19	-2904.543213680796	hqer__induced sampler prototype	1.0
	WDI_agriculture	Quantile-SFA	hqer__induced	True	34	-726.4424375194919	hqer__induced sampler prototype	5.0
	WDI_agriculture	HQER-SFA	hqer__induced	True	7	-717.5938472564712	hqer__induced sampler prototype	2.0
	WDI_agriculture	Quantile-SFA	hqer__induced	True	17	-718.9185174328716	hqer__induced sampler prototype	3.0
	WDI_agriculture	HQER-SFA	hqer__induced	True	8	-707.9290380196879	hqer__induced sampler prototype	2.0
	WDI_agriculture	Quantile-SFA	hqer__induced	True	19	-731.6405773870216	hqer__induced sampler prototype	5.0
	WDI_agriculture	HQER-SFA	hqer__induced	True	10	-723.6308094343003	hqer__induced sampler prototype	4.0
	WDI_agriculture	Quantile-SFA	hqer__induced	True	35	-702.7665857426551	hqer__induced sampler prototype	3.0
	WDI_agriculture	HQER-SFA	hqer__induced	True	18	-695.7521671029295	hqer__induced sampler prototype	2.0
	WDI_agriculture	Quantile-SFA	hqer__induced	True	19	-706.0960630456761	hqer__induced sampler prototype	5.0
	WDI_agriculture	HQER-SFA	hqer__induced	True	15	-696.8124444211444	hqer__induced sampler prototype	4.0
	WDI_agriculture	Quantile-SFA	hqer__induced	True	35	-731.2398780186443	hqer__induced sampler prototype	5.0
	WDI_agriculture	HQER-SFA	hqer__induced	True	15	-723.3609593890076	hqer__induced sampler prototype	2.0
	WDI_agriculture	Quantile-SFA	hqer__induced	True	33	-732.4565375280536	hqer__induced sampler prototype	1.0
	WDI_agriculture	HQER-SFA	hqer__induced	True	10	-725.9158156711294	hqer__induced sampler prototype	4.0
	WDI_agriculture	Quantile-SFA	hqer__induced	True	30	-731.9000268862812	hqer__induced sampler prototype	1.0
	WDI_agriculture	HQER-SFA	hqer__induced	True	8	-724.2222079758981	hqer__induced sampler prototype	4.0
	WDI_agriculture	Quantile-SFA	hqer__induced	True	35	-718.8420785309947	hqer__induced sampler prototype	3.0
	WDI_agriculture	HQER-SFA	hqer__induced	True	9	-709.6731465254178	hqer__induced sampler prototype	2.0
	WDI_agriculture	Quantile-SFA	hqer__induced	True	27	-722.5494619358735	hqer__induced sampler prototype	1.0
	WDI_agriculture	HQER-SFA	hqer__induced	True	39	-718.7139728376987	hqer__induced sampler prototype	5.0

experiment	dataset	model	scenario	converged	n__iter	loglik	warning__message	best__start__id
	WDI_agriculture	Quantile-SFA	hqer_induced	True	41	-695.9621517631376	hqer_induced sampler prototype	1.0
	WDI_agriculture	HQER-SFA	hqer_induced	True	14	-692.7421959015244	hqer_induced sampler prototype	2.0
	WDI_agriculture	Quantile-SFA	hqer_induced	True	23	-734.8122382292038	hqer_induced sampler prototype	5.0
	WDI_agriculture	HQER-SFA	hqer_induced	True	10	-727.3199527605306	hqer_induced sampler prototype	4.0
	WDI_agriculture	Quantile-SFA	hqer_induced	True	21	-724.8946004445202	hqer_induced sampler prototype	5.0
	WDI_agriculture	HQER-SFA	hqer_induced	True	13	-719.3332373054686	hqer_induced sampler prototype	2.0
	WDI_agriculture	Quantile-SFA	hqer_induced	True	29	-720.6276752737242	hqer_induced sampler prototype	1.0
	WDI_agriculture	HQER-SFA	hqer_induced	True	20	-710.8456078310808	hqer_induced sampler prototype	2.0
	WDI_agriculture	Quantile-SFA	hqer_induced	True	32	-697.4861883298092	hqer_induced sampler prototype	1.0
	WDI_agriculture	HQER-SFA	hqer_induced	True	28	-690.5720330561812	hqer_induced sampler prototype	4.0
	WDI_agriculture	Quantile-SFA	hqer_induced	True	24	-739.3892040831598	hqer_induced sampler prototype	1.0
	WDI_agriculture	HQER-SFA	hqer_induced	True	23	-735.8699204082352	hqer_induced sampler prototype	2.0
	WDI_agriculture	Quantile-SFA	hqer_induced	True	28	-692.9546222816439	hqer_induced sampler prototype	1.0
	WDI_agriculture	HQER-SFA	hqer_induced	True	15	-688.5504675807448	hqer_induced sampler prototype	2.0
	WDI_agriculture	Quantile-SFA	hqer_induced	True	34	-691.2034676206206	hqer_induced sampler prototype	3.0
	WDI_agriculture	HQER-SFA	hqer_induced	True	10	-680.8295021136937	hqer_induced sampler prototype	4.0
	WDI_agriculture	Quantile-SFA	hqer_induced	True	28	-710.2510983825671	hqer_induced sampler prototype	3.0
	WDI_agriculture	HQER-SFA	hqer_induced	True	22	-702.2356871261516	hqer_induced sampler prototype	2.0
	WDI_agriculture	Quantile-SFA	hqer_induced	True	36	-711.2703810475901	hqer_induced sampler prototype	1.0
	WDI_agriculture	HQER-SFA	hqer_induced	True	8	-706.3495813519066	hqer_induced sampler prototype	2.0
	WDI_agriculture	Quantile-SFA	hqer_induced	True	23	-716.2519748393977	hqer_induced sampler prototype	5.0
	WDI_agriculture	HQER-SFA	hqer_induced	True	6	-709.9045750347175	hqer_induced sampler prototype	4.0
	WDI_agriculture	Quantile-SFA	hqer_induced	True	17	-678.4269330059058	hqer_induced sampler prototype	5.0
	WDI_agriculture	HQER-SFA	hqer_induced	True	13	-671.559962783929	hqer_induced sampler prototype	2.0
	WDI_agriculture	Quantile-SFA	hqer_induced	True	38	-692.6958207969287	hqer_induced sampler prototype	1.0
	WDI_agriculture	HQER-SFA	hqer_induced	True	20	-687.417082784245	hqer_induced sampler prototype	4.0
	WDI_agriculture	Quantile-SFA	hqer_induced	True	26	-733.0168770998131	hqer_induced sampler prototype	5.0
	WDI_agriculture	HQER-SFA	hqer_induced	True	7	-726.1026821384528	hqer_induced sampler prototype	2.0
	WDI_agriculture	Quantile-SFA	hqer_induced	True	33	-724.2753752058617	hqer_induced sampler prototype	1.0
	WDI_agriculture	HQER-SFA	hqer_induced	True	8	-721.68118324412	hqer_induced sampler prototype	2.0
	WDI_agriculture	Quantile-SFA	hqer_induced	True	29	-692.5745231458855	hqer_induced sampler prototype	5.0
	WDI_agriculture	HQER-SFA	hqer_induced	True	22	-687.3246261259646	hqer_induced sampler prototype	4.0
	WDI_agriculture	Quantile-SFA	hqer_induced	True	25	-727.6302422754093	hqer_induced sampler prototype	3.0
	WDI_agriculture	HQER-SFA	hqer_induced	True	8	-724.7116886009284	hqer_induced sampler prototype	2.0
	WDI_agriculture	Quantile-SFA	hqer_induced	True	29	-713.9208014273377	hqer_induced sampler prototype	1.0
	WDI_agriculture	HQER-SFA	hqer_induced	True	8	-708.1658303386935	hqer_induced sampler prototype	2.0
	WDI_agriculture	Quantile-SFA	hqer_induced	True	24	-710.1225766930338	hqer_induced sampler prototype	1.0
	WDI_agriculture	HQER-SFA	hqer_induced	True	10	-705.1032919223377	hqer_induced sampler prototype	2.0
	WDI_agriculture	Quantile-SFA	hqer_induced	True	29	-703.3579327801519	hqer_induced sampler prototype	3.0
	WDI_agriculture	HQER-SFA	hqer_induced	True	21	-680.0031883013353	hqer_induced sampler prototype	3.0
	WDI_agriculture	Quantile-SFA	hqer_induced	True	28	-710.6454614791135	hqer_induced sampler prototype	5.0
	WDI_agriculture	HQER-SFA	hqer_induced	True	7	-704.608823366242	hqer_induced sampler prototype	2.0
	WDI_agriculture	Quantile-SFA	hqer_induced	True	27	-728.9253962383771	hqer_induced sampler prototype	1.0
	WDI_agriculture	HQER-SFA	hqer_induced	True	10	-719.3761826444157	hqer_induced sampler prototype	4.0
	WDI_agriculture	Quantile-SFA	hqer_induced	True	28	-757.0409724286294	hqer_induced sampler prototype	5.0
	WDI_agriculture	HQER-SFA	hqer_induced	True	12	-752.443333286714	hqer_induced sampler prototype	4.0
	WDI_agriculture	Quantile-SFA	hqer_induced	True	24	-713.1504010654473	hqer_induced sampler prototype	5.0
	WDI_agriculture	HQER-SFA	hqer_induced	True	14	-712.0199431176243	hqer_induced sampler prototype	4.0
	WDI_agriculture	Quantile-SFA	hqer_induced	True	20	-721.4450082911358	hqer_induced sampler prototype	5.0
	WDI_agriculture	HQER-SFA	hqer_induced	True	8	-714.5690846696762	hqer_induced sampler prototype	2.0
	WDI_agriculture	Quantile-SFA	hqer_induced	True	34	-717.725139112735	hqer_induced sampler prototype	1.0
	WDI_agriculture	HQER-SFA	hqer_induced	True	10	-711.582806091793	hqer_induced sampler prototype	2.0
	WDI_agriculture	Quantile-SFA	hqer_induced	True	24	-719.8313614050238	hqer_induced sampler prototype	5.0
	WDI_agriculture	HQER-SFA	hqer_induced	True	12	-713.1681499594583	hqer_induced sampler prototype	4.0
	WDI_agriculture	Quantile-SFA	hqer_induced	True	30	-729.6620839248687	hqer_induced sampler prototype	1.0
	WDI_agriculture	HQER-SFA	hqer_induced	True	16	-721.806709513738	hqer_induced sampler prototype	4.0
	WDI_agriculture	Quantile-SFA	hqer_induced	True	20	-677.2740988176922	hqer_induced sampler prototype	5.0
	WDI_agriculture	HQER-SFA	hqer_induced	True	23	-673.2011948418343	hqer_induced sampler prototype	4.0
	WDI_agriculture	Quantile-SFA	hqer_induced	True	26	-737.7780371421825	hqer_induced sampler prototype	1.0
	WDI_agriculture	HQER-SFA	hqer_induced	True	8	-727.9086937800448	hqer_induced sampler prototype	2.0
	WDI_agriculture	Quantile-SFA	hqer_induced	True	26	-718.1140621445314	hqer_induced sampler prototype	3.0
	WDI_agriculture	HQER-SFA	hqer_induced	True	18	-714.7825649745629	hqer_induced sampler prototype	4.0
	WDI_agriculture	Quantile-SFA	hqer_induced	True	21	-727.5067290664683	hqer_induced sampler prototype	5.0
	WDI_agriculture	HQER-SFA	hqer_induced	True	14	-721.3173566749783	hqer_induced sampler prototype	4.0
	WDI_agriculture	Quantile-SFA	hqer_induced	True	21	-705.4783078161538	hqer_induced sampler prototype	3.0
	WDI_agriculture	HQER-SFA	hqer_induced	True	17	-703.1130388464713	hqer_induced sampler prototype	4.0
	WDI_agriculture	Quantile-SFA	hqer_induced	True	20	-757.0121877481363	hqer_induced sampler prototype	5.0
	WDI_agriculture	HQER-SFA	hqer_induced	True	12	-751.4856953964461	hqer_induced sampler prototype	4.0
	WDI_agriculture	Quantile-SFA	hqer_induced	True	34	-746.7538067040487	hqer_induced sampler prototype	5.0
	WDI_agriculture	HQER-SFA	hqer_induced	True	15	-739.4079996127201	hqer_induced sampler prototype	4.0
	WDI_agriculture	Quantile-SFA	hqer_induced	True	26	-731.7874653441248	hqer_induced sampler prototype	5.0
	WDI_agriculture	HQER-SFA	hqer_induced	True	9	-723.2302517443279	hqer_induced sampler prototype	4.0
	WDI_agriculture	Quantile-SFA	hqer_induced	True	29	-720.8527987606207	hqer_induced sampler prototype	3.0
	WDI_agriculture	HQER-SFA	hqer_induced	True	17	-710.5372034883796	hqer_induced sampler prototype	4.0
	WDI_agriculture	Quantile-SFA	hqer_induced	True	21	-714.1590969220102	hqer_induced sampler prototype	5.0
	WDI_agriculture	HQER-SFA	hqer_induced	True	14	-707.6727543417285	hqer_induced sampler prototype	2.0
	WDI_agriculture	Quantile-SFA	hqer_induced	True	26	-715.583073119853	hqer_induced sampler prototype	5.0
	WDI_agriculture	HQER-SFA	hqer_induced	True	7	-710.2473275727973	hqer_induced sampler prototype	2.0
	WDI_agriculture	Quantile-SFA	hqer_induced	True	22	-737.3397750599186	hqer_induced sampler prototype	1.0
	WDI_agriculture	HQER-SFA	hqer_induced	True	17	-730.8707618973306	hqer_induced sampler prototype	4.0
	WDI_agriculture	Quantile-SFA	hqer_induced	True	32	-711.6971284648132	hqer_induced sampler prototype	5.0
	WDI_agriculture	HQER-SFA	hqer_induced	True	19	-703.8174480450484	hqer_induced sampler prototype	2.0
	WDI_agriculture	Quantile-SFA	hqer_induced	True	26	-717.8433296636297	hqer_induced sampler prototype	1.0
	WDI_agriculture	HQER-SFA	hqer_induced	True	14	-710.122748095542	hqer_induced sampler prototype	4.0
	WDI_agriculture	Quantile-SFA	hqer_induced	True	17	-702.8901501052683	hqer_induced sampler prototype	5.0
	WDI_agriculture	HQER-SFA	hqer_induced	True	9	-700.1522809478138	hqer_induced sampler prototype	2.0
	WDI_agriculture	Quantile-SFA	hqer_induced	True	28	-707.3267955990269	hqer_induced sampler prototype	5.0
	WDI_agriculture	HQER-SFA	hqer_induced	True	13	-701.7660958395313	hqer_induced sampler prototype	2.0
	WDI_agriculture	Quantile-SFA	hqer_induced	True	54	-733.8902025465027	hqer_induced sampler prototype	1.0
	WDI_agriculture	HQER-SFA	hqer_induced	True	14	-725.9528999338362	hqer_induced sampler prototype	4.0
	WDI_agriculture	Quantile-SFA	hqer_induced	True	36	-736.078628556543	hqer_induced sampler prototype	1.0
	WDI_agriculture	HQER-SFA	hqer_induced	True	9	-732.0408382718847	hqer_induced sampler prototype	2.0
	WDI_agriculture	Quantile-SFA	hqer_induced	True	29	-717.1648711168499	hqer_induced sampler prototype	5.0
	WDI_agriculture	HQER-SFA	hqer_induced	True	9	-718.3962438407401	hqer_induced sampler prototype	2.0
	WDI_agriculture	Quantile-SFA	hqer_induced	True	52	-757.4187126926439	hqer_induced sampler prototype	3.0
	WDI_agriculture	HQER-SFA	hqer_induced	True	12	-753.0706131718742	hqer_induced sampler prototype	2.0
	WDI_agriculture	Quantile-SFA	hqer_induced	True	23	-702.9332942303481	hqer_induced sampler prototype	5.0
	WDI_agriculture	HQER-SFA	hqer_induced	True	16	-691.0434680505953	hqer_induced sampler prototype	2.0
	WDI_agriculture	Quantile-SFA	hqer_induced	True	35	-741.6289925368684	hqer_induced sampler prototype	3.0
	WDI_agriculture	HQER-SFA	hqer_induced	True	7	-734.8690877444308	hqer_induced sampler prototype	4.0
	WDI_agriculture	Quantile-SFA	hqer_induced	True	31	-731.1796526083481	hqer_induced sampler prototype	5.0
	WDI_agriculture	HQER-SFA	hqer_induced	True	8	-724.0158382978632	hqer_induced sampler prototype	4.0
	WDI_agriculture	Quantile-SFA	hqer_induced	True	25	-690.400428167807	hqer_induced sampler prototype	1.0

experiment	dataset	model	scenario	converged	n__iter	loglik	warning__message	best__start__id
	WDI__agriculture	HQER-SFA	hqer__induced	True	13	-682.4704377224025	hqer__induced sampler prototype	2.0
	WDI__agriculture	Quantile-SFA	hqer__induced	True	21	-724.4095273361069	hqer__induced sampler prototype	3.0
	WDI__agriculture	HQER-SFA	hqer__induced	True	8	-716.5363271645583	hqer__induced sampler prototype	4.0
	WDI__agriculture	Quantile-SFA	hqer__induced	True	31	-718.154953571651	hqer__induced sampler prototype	5.0
	WDI__agriculture	HQER-SFA	hqer__induced	True	10	-716.6266455868882	hqer__induced sampler prototype	4.0
	WDI__agriculture	Quantile-SFA	hqer__induced	True	35	-680.8671080189238	hqer__induced sampler prototype	1.0
	WDI__agriculture	HQER-SFA	hqer__induced	True	10	-681.337029316707	hqer__induced sampler prototype	2.0
	WDI__agriculture	Quantile-SFA	hqer__induced	True	40	-716.9283615630266	hqer__induced sampler prototype	1.0
	WDI__agriculture	HQER-SFA	hqer__induced	True	11	-711.7929660499794	hqer__induced sampler prototype	4.0
	WDI__agriculture	Quantile-SFA	hqer__induced	True	34	-720.7297784977659	hqer__induced sampler prototype	3.0
	WDI__agriculture	HQER-SFA	hqer__induced	True	12	-717.2668508287163	hqer__induced sampler prototype	2.0
	WDI__agriculture	Quantile-SFA	hqer__induced	True	26	-731.0723752429276	hqer__induced sampler prototype	3.0
	WDI__agriculture	HQER-SFA	hqer__induced	True	19	-725.6834460125183	hqer__induced sampler prototype	2.0
	WDI__agriculture	Quantile-SFA	hqer__induced	True	24	-734.2711234731711	hqer__induced sampler prototype	5.0
	WDI__agriculture	HQER-SFA	hqer__induced	True	10	-729.4949762440165	hqer__induced sampler prototype	4.0
	WDI__agriculture	Quantile-SFA	hqer__induced	True	23	-702.2563179685322	hqer__induced sampler prototype	5.0
	WDI__agriculture	HQER-SFA	hqer__induced	True	7	-697.5198563476986	hqer__induced sampler prototype	2.0
	WDI__agriculture	Quantile-SFA	hqer__induced	True	15	-675.6118028317992	hqer__induced sampler prototype	5.0
	WDI__agriculture	HQER-SFA	hqer__induced	True	11	-668.7953196213782	hqer__induced sampler prototype	4.0
	WDI__agriculture	Quantile-SFA	hqer__induced	True	34	-728.2065794462727	hqer__induced sampler prototype	3.0
	WDI__agriculture	HQER-SFA	hqer__induced	True	10	-723.8340701231448	hqer__induced sampler prototype	4.0
	WDI__agriculture	Quantile-SFA	hqer__induced	True	23	-722.973081803502	hqer__induced sampler prototype	3.0
	WDI__agriculture	HQER-SFA	hqer__induced	True	6	-714.5816197127746	hqer__induced sampler prototype	4.0
	WDI__agriculture	Quantile-SFA	hqer__induced	True	43	-731.6636957310278	hqer__induced sampler prototype	1.0
	WDI__agriculture	HQER-SFA	hqer__induced	True	8	-725.8520046222415	hqer__induced sampler prototype	2.0
	WDI__agriculture	Quantile-SFA	hqer__induced	True	29	-694.6266369013946	hqer__induced sampler prototype	1.0
	WDI__agriculture	HQER-SFA	hqer__induced	True	16	-689.2187597394782	hqer__induced sampler prototype	2.0
	WDI__agriculture	Quantile-SFA	hqer__induced	True	25	-704.5714618637918	hqer__induced sampler prototype	5.0
	WDI__agriculture	HQER-SFA	hqer__induced	True	17	-697.058774877353	hqer__induced sampler prototype	2.0
	WDI__agriculture	Quantile-SFA	hqer__induced	True	19	-690.3836951030503	hqer__induced sampler prototype	3.0
	WDI__agriculture	HQER-SFA	hqer__induced	True	11	-682.1318579541564	hqer__induced sampler prototype	4.0
	WDI__agriculture	Quantile-SFA	hqer__induced	True	28	-742.037146868578	hqer__induced sampler prototype	3.0
	WDI__agriculture	HQER-SFA	hqer__induced	True	9	-738.6109328596075	hqer__induced sampler prototype	2.0
	WDI__agriculture	Quantile-SFA	hqer__induced	True	30	-699.6273951658816	hqer__induced sampler prototype	5.0
	WDI__agriculture	HQER-SFA	hqer__induced	True	12	-696.1259517591678	hqer__induced sampler prototype	2.0
	WDI__agriculture	Quantile-SFA	hqer__induced	True	24	-709.6756564555683	hqer__induced sampler prototype	5.0
	WDI__agriculture	HQER-SFA	hqer__induced	True	6	-701.3923715483713	hqer__induced sampler prototype	4.0
	WDI__agriculture	Quantile-SFA	hqer__induced	True	25	-719.4708727917932	hqer__induced sampler prototype	3.0
	WDI__agriculture	HQER-SFA	hqer__induced	True	21	-710.2037466227983	hqer__induced sampler prototype	4.0
	WDI__agriculture	Quantile-SFA	hqer__induced	True	23	-708.8984005802954	hqer__induced sampler prototype	1.0
	WDI__agriculture	HQER-SFA	hqer__induced	True	12	-701.4213336554413	hqer__induced sampler prototype	4.0
	WDI__agriculture	Quantile-SFA	hqer__induced	True	16	-729.5474272550307	hqer__induced sampler prototype	5.0
	WDI__agriculture	HQER-SFA	hqer__induced	True	16	-718.2529414649499	hqer__induced sampler prototype	2.0
	WDI__agriculture	Quantile-SFA	hqer__induced	True	25	-723.3688027653583	hqer__induced sampler prototype	1.0
	WDI__agriculture	HQER-SFA	hqer__induced	True	14	-714.703583372149	hqer__induced sampler prototype	2.0
	WDI__agriculture	Quantile-SFA	hqer__induced	True	20	-707.4366882905881	hqer__induced sampler prototype	3.0
	WDI__agriculture	HQER-SFA	hqer__induced	True	15	-705.944480996184	hqer__induced sampler prototype	2.0
	WDI__agriculture	Quantile-SFA	hqer__induced	True	23	-708.122642522191	hqer__induced sampler prototype	5.0
	WDI__agriculture	HQER-SFA	hqer__induced	True	6	-704.1791370151896	hqer__induced sampler prototype	4.0
	WDI__agriculture	Quantile-SFA	hqer__induced	True	23	-715.8431212173948	hqer__induced sampler prototype	3.0
	WDI__agriculture	HQER-SFA	hqer__induced	True	11	-706.3714515536712	hqer__induced sampler prototype	2.0
	WDI__agriculture	Quantile-SFA	hqer__induced	True	24	-717.9060712533332	hqer__induced sampler prototype	5.0
	WDI__agriculture	HQER-SFA	hqer__induced	True	24	-709.1981713619664	hqer__induced sampler prototype	4.0
	WDI__agriculture	Quantile-SFA	hqer__induced	True	27	-698.5463496077035	hqer__induced sampler prototype	5.0
	WDI__agriculture	HQER-SFA	hqer__induced	True	9	-690.5077646079953	hqer__induced sampler prototype	4.0
	WDI__agriculture	Quantile-SFA	hqer__induced	True	26	-712.0868852519054	hqer__induced sampler prototype	3.0
	WDI__agriculture	HQER-SFA	hqer__induced	True	19	-704.1444113822826	hqer__induced sampler prototype	4.0
	WDI__agriculture	Quantile-SFA	hqer__induced	True	31	-711.0484269159113	hqer__induced sampler prototype	1.0
	WDI__agriculture	HQER-SFA	hqer__induced	True	15	-704.4722160617795	hqer__induced sampler prototype	2.0
	WDI__agriculture	Quantile-SFA	hqer__induced	True	29	-748.3748533561486	hqer__induced sampler prototype	1.0
	WDI__agriculture	HQER-SFA	hqer__induced	True	11	-744.3240455957352	hqer__induced sampler prototype	2.0
	WDI__agriculture	Quantile-SFA	hqer__induced	True	31	-709.4817868257842	hqer__induced sampler prototype	1.0
	WDI__agriculture	HQER-SFA	hqer__induced	True	5	-706.0710413248631	hqer__induced sampler prototype	2.0
	WDI__agriculture	Quantile-SFA	hqer__induced	True	28	-730.91983375767	hqer__induced sampler prototype	1.0
	WDI__agriculture	HQER-SFA	hqer__induced	True	15	-727.62930213409	hqer__induced sampler prototype	2.0
	WDI__agriculture	Quantile-SFA	hqer__induced	True	33	-738.1123797060168	hqer__induced sampler prototype	1.0
	WDI__agriculture	HQER-SFA	hqer__induced	True	18	-733.5990617404293	hqer__induced sampler prototype	2.0
	WDI__agriculture	Quantile-SFA	hqer__induced	True	32	-677.4702801639482	hqer__induced sampler prototype	5.0
	WDI__agriculture	HQER-SFA	hqer__induced	True	11	-674.534015605102	hqer__induced sampler prototype	2.0
	WDI__agriculture	Quantile-SFA	hqer__induced	True	21	-733.7934040194631	hqer__induced sampler prototype	3.0
	WDI__agriculture	HQER-SFA	hqer__induced	True	17	-722.4899560131581	hqer__induced sampler prototype	2.0
	WDI__agriculture	Quantile-SFA	hqer__induced	True	25	-677.9208228257548	hqer__induced sampler prototype	5.0
	WDI__agriculture	HQER-SFA	hqer__induced	True	38	-673.5392398312745	hqer__induced sampler prototype	3.0
	WDI__agriculture	Quantile-SFA	hqer__induced	True	19	-742.3655192147667	hqer__induced sampler prototype	5.0
	WDI__agriculture	HQER-SFA	hqer__induced	True	14	-733.379681500568	hqer__induced sampler prototype	2.0
	WDI__agriculture	Quantile-SFA	hqer__induced	True	26	-690.9676321464935	hqer__induced sampler prototype	5.0
	WDI__agriculture	HQER-SFA	hqer__induced	True	17	-683.6281488918478	hqer__induced sampler prototype	2.0
	WDI__agriculture	Quantile-SFA	hqer__induced	True	15	-2884.4738274337474	hqer__induced sampler prototype	5.0
	WDI__agriculture	HQER-SFA	hqer__induced	True	12	-2865.774596476232	hqer__induced sampler prototype	2.0
	WDI__agriculture	Quantile-SFA	hqer__induced	True	27	-2863.037486068746	hqer__induced sampler prototype	1.0
	WDI__agriculture	HQER-SFA	hqer__induced	True	13	-2832.8574960719934	hqer__induced sampler prototype	4.0
	WDI__agriculture	Quantile-SFA	hqer__induced	True	24	-2892.6944851668472	hqer__induced sampler prototype	3.0
	WDI__agriculture	HQER-SFA	hqer__induced	True	7	-2864.750654074416	hqer__induced sampler prototype	2.0
	WDI__agriculture	Quantile-SFA	hqer__induced	True	22	-2883.9649860730374	hqer__induced sampler prototype	3.0
	WDI__agriculture	HQER-SFA	hqer__induced	True	4	-2860.659635056216	hqer__induced sampler prototype	2.0
	WDI__agriculture	Quantile-SFA	hqer__induced	True	36	-2891.0480132325856	hqer__induced sampler prototype	3.0
	WDI__agriculture	HQER-SFA	hqer__induced	True	7	-2864.170253779309	hqer__induced sampler prototype	2.0
	WDI__agriculture	Quantile-SFA	hqer__induced	True	22	-2864.970742083251	hqer__induced sampler prototype	5.0
	WDI__agriculture	HQER-SFA	hqer__induced	True	6	-2839.590527619416	hqer__induced sampler prototype	2.0
	WDI__agriculture	Quantile-SFA	hqer__induced	True	24	-2844.216018160188	hqer__induced sampler prototype	1.0
	WDI__agriculture	HQER-SFA	hqer__induced	True	8	-2818.561998074392	hqer__induced sampler prototype	2.0
	WDI__agriculture	Quantile-SFA	hqer__induced	True	20	-2858.0913159748216	hqer__induced sampler prototype	5.0
	WDI__agriculture	HQER-SFA	hqer__induced	True	10	-2831.2025575562307	hqer__induced sampler prototype	2.0
	WDI__agriculture	Quantile-SFA	hqer__induced	True	23	-2883.646094942331	hqer__induced sampler prototype	1.0
	WDI__agriculture	HQER-SFA	hqer__induced	True	12	-2850.9900846970218	hqer__induced sampler prototype	2.0
	WDI__agriculture	Quantile-SFA	hqer__induced	True	18	-2892.9327447200903	hqer__induced sampler prototype	1.0
	WDI__agriculture	HQER-SFA	hqer__induced	True	12	-2863.977373421493	hqer__induced sampler prototype	4.0
	WDI__agriculture	Quantile-SFA	hqer__induced	True	23	-2856.50369306017	hqer__induced sampler prototype	1.0
	WDI__agriculture	HQER-SFA	hqer__induced	True	8	-2833.2665892345344	hqer__induced sampler prototype	4.0
	WDI__agriculture	Quantile-SFA	hqer__induced	True	22	-2891.3143584361296	hqer__induced sampler prototype	5.0
	WDI__agriculture	HQER-SFA	hqer__induced	True	8	-2868.4786354042985	hqer__induced sampler prototype	2.0
	WDI__agriculture	Quantile-SFA	hqer__induced	True	22	-2868.65336237708	hqer__induced sampler prototype	3.0
	WDI__agriculture	HQER-SFA	hqer__induced	True	9	-2842.509027486348	hqer__induced sampler prototype	4.0

experiment	dataset	model	scenario	converged	n_iter	loglik	warning_message	best_start_id
	WDI_agriculture	Quantile-SFA	hqer_induced	True	22	-2922.109420652952	hqer_induced sampler prototype	3.0
	WDI_agriculture	HQER-SFA	hqer_induced	True	6	-2892.478905604371	hqer_induced sampler prototype	2.0
	WDI_agriculture	Quantile-SFA	hqer_induced	True	15	-2909.893488963043	hqer_induced sampler prototype	5.0
	WDI_agriculture	HQER-SFA	hqer_induced	True	12	-2882.751269012301	hqer_induced sampler prototype	4.0
	WDI_agriculture	Quantile-SFA	hqer_induced	True	22	-2879.66316209804	hqer_induced sampler prototype	5.0
	WDI_agriculture	HQER-SFA	hqer_induced	True	8	-2846.7181163506693	hqer_induced sampler prototype	4.0
	WDI_agriculture	Quantile-SFA	hqer_induced	True	15	-2886.8173008557405	hqer_induced sampler prototype	5.0
	WDI_agriculture	HQER-SFA	hqer_induced	True	7	-2857.8618830848563	hqer_induced sampler prototype	2.0
	WDI_agriculture	Quantile-SFA	hqer_induced	True	23	-2880.746590752932	hqer_induced sampler prototype	3.0
	WDI_agriculture	HQER-SFA	hqer_induced	True	11	-2847.813265407553	hqer_induced sampler prototype	4.0
	WDI_agriculture	Quantile-SFA	hqer_induced	True	27	-2854.0188288231075	hqer_induced sampler prototype	3.0
	WDI_agriculture	HQER-SFA	hqer_induced	True	11	-2823.304580211154	hqer_induced sampler prototype	4.0
	WDI_agriculture	Quantile-SFA	hqer_induced	True	36	-2838.806326888204	hqer_induced sampler prototype	3.0
	WDI_agriculture	HQER-SFA	hqer_induced	True	7	-2806.423214407377	hqer_induced sampler prototype	4.0
	WDI_agriculture	Quantile-SFA	hqer_induced	True	18	-2866.724688898189	hqer_induced sampler prototype	5.0
	WDI_agriculture	HQER-SFA	hqer_induced	True	8	-2841.79449160922	hqer_induced sampler prototype	2.0
	WDI_agriculture	Quantile-SFA	hqer_induced	True	24	-2941.913010069932	hqer_induced sampler prototype	5.0
	WDI_agriculture	HQER-SFA	hqer_induced	True	13	-2913.3968484851907	hqer_induced sampler prototype	4.0
	WDI_agriculture	Quantile-SFA	hqer_induced	True	30	-2904.36128191532	hqer_induced sampler prototype	1.0
	WDI_agriculture	HQER-SFA	hqer_induced	True	19	-2875.9928207748835	hqer_induced sampler prototype	4.0
	WDI_agriculture	Quantile-SFA	hqer_induced	True	26	-2883.4982795488763	hqer_induced sampler prototype	3.0
	WDI_agriculture	HQER-SFA	hqer_induced	True	12	-2857.8618904852538	hqer_induced sampler prototype	2.0
	WDI_agriculture	Quantile-SFA	hqer_induced	True	23	-2828.244481467934	hqer_induced sampler prototype	5.0
	WDI_agriculture	HQER-SFA	hqer_induced	True	8	-2803.4772005017394	hqer_induced sampler prototype	2.0
	WDI_agriculture	Quantile-SFA	hqer_induced	True	22	-2853.830828262661	hqer_induced sampler prototype	5.0
	WDI_agriculture	HQER-SFA	hqer_induced	True	12	-2827.907790289909	hqer_induced sampler prototype	4.0
	WDI_agriculture	Quantile-SFA	hqer_induced	True	27	-2886.556787169104	hqer_induced sampler prototype	3.0
	WDI_agriculture	HQER-SFA	hqer_induced	True	7	-2868.647757528696	hqer_induced sampler prototype	4.0
	WDI_agriculture	Quantile-SFA	hqer_induced	True	34	-2871.4144394104096	hqer_induced sampler prototype	3.0
	WDI_agriculture	HQER-SFA	hqer_induced	True	11	-2850.218208586837	hqer_induced sampler prototype	4.0
	WDI_agriculture	Quantile-SFA	hqer_induced	True	21	-2843.929143691877	hqer_induced sampler prototype	1.0
	WDI_agriculture	HQER-SFA	hqer_induced	True	12	-2812.806587540978	hqer_induced sampler prototype	4.0
	WDI_agriculture	Quantile-SFA	hqer_induced	True	20	-2915.7272597265987	hqer_induced sampler prototype	3.0
	WDI_agriculture	HQER-SFA	hqer_induced	True	14	-2886.9318157850867	hqer_induced sampler prototype	2.0
	WDI_agriculture	Quantile-SFA	hqer_induced	True	24	-2870.957290143512	hqer_induced sampler prototype	3.0
	WDI_agriculture	HQER-SFA	hqer_induced	True	6	-2835.6772475608404	hqer_induced sampler prototype	4.0
	WDI_agriculture	Quantile-SFA	hqer_induced	True	22	-2827.2890018980224	hqer_induced sampler prototype	3.0
	WDI_agriculture	HQER-SFA	hqer_induced	True	11	-2798.678221026583	hqer_induced sampler prototype	4.0
	WDI_agriculture	Quantile-SFA	hqer_induced	True	27	-2847.644355012955	hqer_induced sampler prototype	1.0
	WDI_agriculture	HQER-SFA	hqer_induced	True	11	-2831.942547032088	hqer_induced sampler prototype	2.0
	WDI_agriculture	Quantile-SFA	hqer_induced	True	32	-2831.1486460772503	hqer_induced sampler prototype	1.0
	WDI_agriculture	HQER-SFA	hqer_induced	True	4	-2801.5822463629274	hqer_induced sampler prototype	2.0
	WDI_agriculture	Quantile-SFA	hqer_induced	True	25	-2891.334479412348	hqer_induced sampler prototype	3.0
	WDI_agriculture	HQER-SFA	hqer_induced	True	11	-2853.645546959688	hqer_induced sampler prototype	2.0
	WDI_agriculture	Quantile-SFA	hqer_induced	True	23	-2906.533344010107	hqer_induced sampler prototype	3.0
	WDI_agriculture	HQER-SFA	hqer_induced	True	7	-2879.713389644923	hqer_induced sampler prototype	2.0
	WDI_agriculture	Quantile-SFA	hqer_induced	True	28	-2877.05060983068	hqer_induced sampler prototype	3.0
	WDI_agriculture	HQER-SFA	hqer_induced	True	8	-2855.164752546196	hqer_induced sampler prototype	2.0
	WDI_agriculture	Quantile-SFA	hqer_induced	True	19	-2823.6873077332466	hqer_induced sampler prototype	5.0
	WDI_agriculture	HQER-SFA	hqer_induced	True	16	-2784.9468132178645	hqer_induced sampler prototype	2.0
	WDI_agriculture	Quantile-SFA	hqer_induced	True	23	-2857.3616260971207	hqer_induced sampler prototype	5.0
	WDI_agriculture	HQER-SFA	hqer_induced	True	16	-2823.8692781727823	hqer_induced sampler prototype	4.0
	WDI_agriculture	Quantile-SFA	hqer_induced	True	33	-2885.4610907786027	hqer_induced sampler prototype	3.0
	WDI_agriculture	HQER-SFA	hqer_induced	True	9	-2856.461393706741	hqer_induced sampler prototype	4.0
	WDI_agriculture	Quantile-SFA	hqer_induced	True	25	-2945.857211602908	hqer_induced sampler prototype	1.0
	WDI_agriculture	HQER-SFA	hqer_induced	True	8	-2915.877807454038	hqer_induced sampler prototype	4.0
	WDI_agriculture	Quantile-SFA	hqer_induced	True	25	-2894.083078423157	hqer_induced sampler prototype	5.0
	WDI_agriculture	HQER-SFA	hqer_induced	True	7	-2860.062441012184	hqer_induced sampler prototype	2.0
	WDI_agriculture	Quantile-SFA	hqer_induced	True	25	-2935.401161226059	hqer_induced sampler prototype	5.0
	WDI_agriculture	HQER-SFA	hqer_induced	True	8	-2900.5927578315977	hqer_induced sampler prototype	4.0
	WDI_agriculture	Quantile-SFA	hqer_induced	True	29	-2893.49235906098	hqer_induced sampler prototype	3.0
	WDI_agriculture	HQER-SFA	hqer_induced	True	7	-2864.637563503285	hqer_induced sampler prototype	4.0
	WDI_agriculture	Quantile-SFA	hqer_induced	True	28	-2858.668237940453	hqer_induced sampler prototype	5.0
	WDI_agriculture	HQER-SFA	hqer_induced	True	9	-2828.5679077086284	hqer_induced sampler prototype	2.0
	WDI_agriculture	Quantile-SFA	hqer_induced	True	27	-2900.6518518734915	hqer_induced sampler prototype	3.0
	WDI_agriculture	HQER-SFA	hqer_induced	True	8	-2871.536269018862	hqer_induced sampler prototype	4.0
	WDI_agriculture	Quantile-SFA	hqer_induced	True	27	-2965.213886617731	hqer_induced sampler prototype	3.0
	WDI_agriculture	HQER-SFA	hqer_induced	True	14	-2932.972742107224	hqer_induced sampler prototype	4.0
	WDI_agriculture	Quantile-SFA	hqer_induced	True	23	-2943.1505579601253	hqer_induced sampler prototype	1.0
	WDI_agriculture	HQER-SFA	hqer_induced	True	7	-2908.381287726705	hqer_induced sampler prototype	4.0
	WDI_agriculture	Quantile-SFA	hqer_induced	True	30	-2910.143605203108	hqer_induced sampler prototype	5.0
	WDI_agriculture	HQER-SFA	hqer_induced	True	8	-2893.3491882149783	hqer_induced sampler prototype	4.0
	WDI_agriculture	Quantile-SFA	hqer_induced	True	24	-2916.1830672305805	hqer_induced sampler prototype	5.0
	WDI_agriculture	HQER-SFA	hqer_induced	True	13	-2882.289686534547	hqer_induced sampler prototype	4.0
	WDI_agriculture	Quantile-SFA	hqer_induced	True	23	-2910.4186111825884	hqer_induced sampler prototype	1.0
	WDI_agriculture	HQER-SFA	hqer_induced	True	12	-2880.80349056096	hqer_induced sampler prototype	4.0
	WDI_agriculture	Quantile-SFA	hqer_induced	True	22	-2899.5220008950973	hqer_induced sampler prototype	5.0
	WDI_agriculture	HQER-SFA	hqer_induced	True	10	-2865.7199580760694	hqer_induced sampler prototype	4.0
	WDI_agriculture	Quantile-SFA	hqer_induced	True	27	-2863.1521143418613	hqer_induced sampler prototype	1.0
	WDI_agriculture	HQER-SFA	hqer_induced	True	16	-2833.6528197633143	hqer_induced sampler prototype	4.0
	WDI_agriculture	Quantile-SFA	hqer_induced	True	23	-2857.7379062063496	hqer_induced sampler prototype	3.0
	WDI_agriculture	HQER-SFA	hqer_induced	True	10	-2836.423503807534	hqer_induced sampler prototype	2.0
	WDI_agriculture	Quantile-SFA	hqer_induced	True	27	-2901.4386961661244	hqer_induced sampler prototype	3.0
	WDI_agriculture	HQER-SFA	hqer_induced	True	8	-2877.958320694347	hqer_induced sampler prototype	4.0
	WDI_agriculture	Quantile-SFA	hqer_induced	True	26	-2880.998763629825	hqer_induced sampler prototype	5.0
	WDI_agriculture	HQER-SFA	hqer_induced	True	7	-2853.7522897946124	hqer_induced sampler prototype	4.0
	WDI_agriculture	Quantile-SFA	hqer_induced	True	17	-2866.555888300205	hqer_induced sampler prototype	1.0
	WDI_agriculture	HQER-SFA	hqer_induced	True	13	-2835.084071460244	hqer_induced sampler prototype	2.0
	WDI_agriculture	Quantile-SFA	hqer_induced	True	27	-2857.040314387289	hqer_induced sampler prototype	3.0
	WDI_agriculture	HQER-SFA	hqer_induced	True	10	-2824.142692855134	hqer_induced sampler prototype	2.0
	WDI_agriculture	Quantile-SFA	hqer_induced	True	25	-2951.792591968895	hqer_induced sampler prototype	5.0
	WDI_agriculture	HQER-SFA	hqer_induced	True	11	-2930.7331910562884	hqer_induced sampler prototype	4.0
	WDI_agriculture	Quantile-SFA	hqer_induced	True	20	-2817.8203893800087	hqer_induced sampler prototype	5.0
	WDI_agriculture	HQER-SFA	hqer_induced	True	14	-2793.6177365125927	hqer_induced sampler prototype	4.0
	WDI_agriculture	Quantile-SFA	hqer_induced	True	28	-2896.5933554897056	hqer_induced sampler prototype	3.0
	WDI_agriculture	HQER-SFA	hqer_induced	True	8	-2870.308061912845	hqer_induced sampler prototype	2.0
	WDI_agriculture	Quantile-SFA	hqer_induced	True	23	-2853.8858985371817	hqer_induced sampler prototype	3.0
	WDI_agriculture	HQER-SFA	hqer_induced	True	8	-2826.1089475443614	hqer_induced sampler prototype	2.0
	WDI_agriculture	Quantile-SFA	hqer_induced	True	44	-2893.391013335474	hqer_induced sampler prototype	1.0
	WDI_agriculture	HQER-SFA	hqer_induced	True	9	-2871.638944221454	hqer_induced sampler prototype	4.0
	WDI_agriculture	Quantile-SFA	hqer_induced	True	15	-2925.281758429518	hqer_induced sampler prototype	5.0
	WDI_agriculture	HQER-SFA	hqer_induced	True	9	-2896.437851550516	hqer_induced sampler prototype	2.0
	WDI_agriculture	Quantile-SFA	hqer_induced	True	24	-2903.1999871945154	hqer_induced sampler prototype	1.0

experiment	dataset	model	scenario	converged	n_iter	loglik	warning_message	best_start_id
	WDI_agriculture	HQER-SFA	hqer_induced	True	8	-2867.639458364576	hqer_induced sampler prototype	2.0
	WDI_agriculture	Quantile-SFA	hqer_induced	True	28	-2939.7436836779834	hqer_induced sampler prototype	3.0
	WDI_agriculture	HQER-SFA	hqer_induced	True	12	-2908.56347125208	hqer_induced sampler prototype	4.0
	WDI_agriculture	Quantile-SFA	hqer_induced	True	24	-2855.663663186309	hqer_induced sampler prototype	5.0
	WDI_agriculture	HQER-SFA	hqer_induced	True	7	-2823.46284861163	hqer_induced sampler prototype	2.0
	WDI_agriculture	Quantile-SFA	hqer_induced	True	26	-2862.106086331195	hqer_induced sampler prototype	1.0
	WDI_agriculture	HQER-SFA	hqer_induced	True	7	-2840.848407395072	hqer_induced sampler prototype	2.0
	WDI_agriculture	Quantile-SFA	hqer_induced	True	20	-2879.578464156905	hqer_induced sampler prototype	1.0
	WDI_agriculture	HQER-SFA	hqer_induced	True	8	-2849.3111924885507	hqer_induced sampler prototype	2.0
	WDI_agriculture	Quantile-SFA	hqer_induced	True	19	-2882.106692379528	hqer_induced sampler prototype	1.0
	WDI_agriculture	HQER-SFA	hqer_induced	True	21	-2847.131821714457	hqer_induced sampler prototype	4.0
	WDI_agriculture	Quantile-SFA	hqer_induced	True	29	-2905.859888348863	hqer_induced sampler prototype	3.0
	WDI_agriculture	HQER-SFA	hqer_induced	True	8	-2878.066403428558	hqer_induced sampler prototype	2.0
	WDI_agriculture	Quantile-SFA	hqer_induced	True	22	-2884.639337968513	hqer_induced sampler prototype	3.0
	WDI_agriculture	HQER-SFA	hqer_induced	True	10	-2848.4385040658613	hqer_induced sampler prototype	4.0
	WDI_agriculture	Quantile-SFA	hqer_induced	True	20	-2888.127883784829	hqer_induced sampler prototype	3.0
	WDI_agriculture	HQER-SFA	hqer_induced	True	6	-2857.3495590806606	hqer_induced sampler prototype	4.0
	WDI_agriculture	Quantile-SFA	hqer_induced	True	32	-2956.7161958752054	hqer_induced sampler prototype	5.0
	WDI_agriculture	HQER-SFA	hqer_induced	True	9	-2928.2209523241936	hqer_induced sampler prototype	2.0
	WDI_agriculture	Quantile-SFA	hqer_induced	True	21	-2869.3691513489766	hqer_induced sampler prototype	1.0
	WDI_agriculture	HQER-SFA	hqer_induced	True	7	-2834.5398523531085	hqer_induced sampler prototype	2.0
	WDI_agriculture	Quantile-SFA	hqer_induced	True	14	-2901.7245630200327	hqer_induced sampler prototype	5.0
	WDI_agriculture	HQER-SFA	hqer_induced	True	9	-2876.426399507924	hqer_induced sampler prototype	2.0
	WDI_agriculture	Quantile-SFA	hqer_induced	True	25	-2901.54307596579	hqer_induced sampler prototype	1.0
	WDI_agriculture	HQER-SFA	hqer_induced	True	11	-2876.943866231014	hqer_induced sampler prototype	4.0
	WDI_agriculture	Quantile-SFA	hqer_induced	True	20	-2835.821698789749	hqer_induced sampler prototype	5.0
	WDI_agriculture	HQER-SFA	hqer_induced	True	13	-2809.0099926394414	hqer_induced sampler prototype	4.0
	WDI_agriculture	Quantile-SFA	hqer_induced	True	17	-2837.905411427834	hqer_induced sampler prototype	3.0
	WDI_agriculture	HQER-SFA	hqer_induced	True	10	-2805.721335623016	hqer_induced sampler prototype	4.0
	WDI_agriculture	Quantile-SFA	hqer_induced	True	22	-2853.239091729412	hqer_induced sampler prototype	1.0
	WDI_agriculture	HQER-SFA	hqer_induced	True	8	-2826.99292191414	hqer_induced sampler prototype	2.0
	WDI_agriculture	Quantile-SFA	hqer_induced	True	19	-2930.7156817935347	hqer_induced sampler prototype	1.0
	WDI_agriculture	HQER-SFA	hqer_induced	True	9	-2901.505732048762	hqer_induced sampler prototype	2.0
	WDI_agriculture	Quantile-SFA	hqer_induced	True	27	-2899.5289161969245	hqer_induced sampler prototype	1.0
	WDI_agriculture	HQER-SFA	hqer_induced	True	14	-2870.322834696294	hqer_induced sampler prototype	2.0
	WDI_agriculture	Quantile-SFA	hqer_induced	True	24	-2925.058165176501	hqer_induced sampler prototype	3.0
	WDI_agriculture	HQER-SFA	hqer_induced	True	8	-2893.420979417934	hqer_induced sampler prototype	4.0
	WDI_agriculture	Quantile-SFA	hqer_induced	True	15	-2859.4261211040216	hqer_induced sampler prototype	1.0
	WDI_agriculture	HQER-SFA	hqer_induced	True	19	-2838.6223944684007	hqer_induced sampler prototype	4.0
	WDI_agriculture	Quantile-SFA	hqer_induced	True	26	-2885.499828528133	hqer_induced sampler prototype	3.0
	WDI_agriculture	HQER-SFA	hqer_induced	True	8	-2847.86124713462	hqer_induced sampler prototype	4.0
	WDI_agriculture	Quantile-SFA	hqer_induced	True	24	-2884.4340546439653	hqer_induced sampler prototype	5.0
	WDI_agriculture	HQER-SFA	hqer_induced	True	16	-2850.913828671104	hqer_induced sampler prototype	4.0
	WDI_agriculture	Quantile-SFA	hqer_induced	True	19	-2874.248513489808	hqer_induced sampler prototype	3.0
	WDI_agriculture	HQER-SFA	hqer_induced	True	7	-2846.0835902421904	hqer_induced sampler prototype	4.0
	WDI_agriculture	Quantile-SFA	hqer_induced	True	22	-2897.667981310019	hqer_induced sampler prototype	5.0
	WDI_agriculture	HQER-SFA	hqer_induced	True	3	-2868.02268837722	hqer_induced sampler prototype	2.0
	WDI_agriculture	Quantile-SFA	hqer_induced	True	21	-2914.903776306372	hqer_induced sampler prototype	5.0
	WDI_agriculture	HQER-SFA	hqer_induced	True	12	-2881.780650927537	hqer_induced sampler prototype	4.0
	WDI_agriculture	Quantile-SFA	hqer_induced	True	32	-2878.7332093424834	hqer_induced sampler prototype	3.0
	WDI_agriculture	HQER-SFA	hqer_induced	True	10	-2847.394913492584	hqer_induced sampler prototype	4.0
	WDI_agriculture	Quantile-SFA	hqer_induced	True	24	-2878.8878768474224	hqer_induced sampler prototype	1.0
	WDI_agriculture	HQER-SFA	hqer_induced	True	4	-2850.800819340995	hqer_induced sampler prototype	2.0
	WDI_agriculture	Quantile-SFA	hqer_induced	True	19	-2825.6535017471497	hqer_induced sampler prototype	5.0
	WDI_agriculture	HQER-SFA	hqer_induced	True	8	-2800.9074641842235	hqer_induced sampler prototype	2.0
	WDI_agriculture	Quantile-SFA	hqer_induced	True	34	-2865.423650397973	hqer_induced sampler prototype	3.0
	WDI_agriculture	HQER-SFA	hqer_induced	True	4	-2838.5601507182414	hqer_induced sampler prototype	2.0
	WDI_agriculture	Quantile-SFA	hqer_induced	True	25	-2917.283624407919	hqer_induced sampler prototype	3.0
	WDI_agriculture	HQER-SFA	hqer_induced	True	10	-2887.890054652477	hqer_induced sampler prototype	4.0
	WDI_agriculture	Quantile-SFA	hqer_induced	True	23	-2885.6630488813485	hqer_induced sampler prototype	3.0
	WDI_agriculture	HQER-SFA	hqer_induced	True	7	-2852.996029880682	hqer_induced sampler prototype	4.0
	WDI_agriculture	Quantile-SFA	hqer_induced	True	26	-2851.039578088636	hqer_induced sampler prototype	3.0
	WDI_agriculture	HQER-SFA	hqer_induced	True	8	-2821.689672980204	hqer_induced sampler prototype	4.0
	WDI_agriculture	Quantile-SFA	hqer_induced	True	22	-2859.4738645791467	hqer_induced sampler prototype	3.0
	WDI_agriculture	HQER-SFA	hqer_induced	True	11	-2826.543272087012	hqer_induced sampler prototype	4.0
	WDI_agriculture	Quantile-SFA	hqer_induced	True	20	-2931.0201615364253	hqer_induced sampler prototype	5.0
	WDI_agriculture	HQER-SFA	hqer_induced	True	7	-2908.184326281832	hqer_induced sampler prototype	2.0
	WDI_agriculture	Quantile-SFA	hqer_induced	True	19	-2886.3776305834363	hqer_induced sampler prototype	5.0
	WDI_agriculture	HQER-SFA	hqer_induced	True	18	-2854.0357100319698	hqer_induced sampler prototype	4.0
	WDI_agriculture	Quantile-SFA	hqer_induced	True	27	-2833.0280778213632	hqer_induced sampler prototype	1.0
	WDI_agriculture	HQER-SFA	hqer_induced	True	6	-2800.5702505594672	hqer_induced sampler prototype	4.0
	front41Data	Quantile-SFA	hqer_induced	True	17	5.118305721673847	hqer_induced sampler prototype	2.0
	front41Data	HQER-SFA	hqer_induced	True	25	5.131500512346182	hqer_induced sampler prototype	2.0
	front41Data	Quantile-SFA	hqer_induced	True	28	-6.318135567145943	hqer_induced sampler prototype	4.0
	front41Data	HQER-SFA	hqer_induced	True	11	-6.396170353434023	hqer_induced sampler prototype	2.0
	front41Data	Quantile-SFA	hqer_induced	True	24	-1.070030542244635	hqer_induced sampler prototype	2.0
	front41Data	HQER-SFA	hqer_induced	True	17	-1.0582716624039263	hqer_induced sampler prototype	2.0
	front41Data	Quantile-SFA	hqer_induced	True	11	-1.7985466515856985	hqer_induced sampler prototype	2.0
	front41Data	HQER-SFA	hqer_induced	True	11	-1.7683544504100888	hqer_induced sampler prototype	2.0
	front41Data	Quantile-SFA	hqer_induced	True	32	-3.604409749739681	hqer_induced sampler prototype	4.0
	front41Data	HQER-SFA	hqer_induced	True	37	-3.5954504529370475	hqer_induced sampler prototype	4.0
	front41Data	Quantile-SFA	hqer_induced	True	33	13.547088947740043	hqer_induced sampler prototype	4.0
	front41Data	HQER-SFA	hqer_induced	True	13	13.109311180777738	hqer_induced sampler prototype	2.0
	front41Data	Quantile-SFA	hqer_induced	True	21	5.552693928490825	hqer_induced sampler prototype	4.0
	front41Data	HQER-SFA	hqer_induced	True	21	5.5544422879076825	hqer_induced sampler prototype	2.0
	front41Data	Quantile-SFA	hqer_induced	True	16	6.36141676502034	hqer_induced sampler prototype	2.0
	front41Data	HQER-SFA	hqer_induced	True	23	6.407860261493041	hqer_induced sampler prototype	2.0
	front41Data	Quantile-SFA	hqer_induced	True	43	-1.5920626598254568	hqer_induced sampler prototype	2.0
	front41Data	HQER-SFA	hqer_induced	True	27	-1.6330796925829556	hqer_induced sampler prototype	4.0
	front41Data	Quantile-SFA	hqer_induced	True	39	-6.486022759876539	hqer_induced sampler prototype	4.0
	front41Data	HQER-SFA	hqer_induced	True	27	-6.561936296989133	hqer_induced sampler prototype	4.0
	front41Data	Quantile-SFA	hqer_induced	True	32	3.451445924165834	hqer_induced sampler prototype	4.0
	front41Data	HQER-SFA	hqer_induced	True	48	3.4723672672522463	hqer_induced sampler prototype	4.0
	front41Data	Quantile-SFA	hqer_induced	True	16	15.203437405708351	hqer_induced sampler prototype	2.0
	front41Data	HQER-SFA	hqer_induced	True	26	15.229092251883475	hqer_induced sampler prototype	4.0
	front41Data	Quantile-SFA	hqer_induced	True	36	0.7316092834779576	hqer_induced sampler prototype	4.0
	front41Data	HQER-SFA	hqer_induced	True	25	0.7217140530284227	hqer_induced sampler prototype	4.0
	front41Data	Quantile-SFA	hqer_induced	True	37	-0.0470497374532499	hqer_induced sampler prototype	2.0
	front41Data	HQER-SFA	hqer_induced	True	40	-0.029545994230252	hqer_induced sampler prototype	4.0
	front41Data	Quantile-SFA	hqer_induced	True	11	-5.470442430402196	hqer_induced sampler prototype	2.0
	front41Data	HQER-SFA	hqer_induced	True	46	-5.474950368431682	hqer_induced sampler prototype	4.0
	front41Data	Quantile-SFA	hqer_induced	True	15	10.05039812401036	hqer_induced sampler prototype	2.0
	front41Data	HQER-SFA	hqer_induced	True	24	10.186304599596667	hqer_induced sampler prototype	2.0

experiment	dataset	model	scenario	converged	n__iter	loglik	warning__message	best__start__id
	front41Data	Quantile-SFA	hqer__induced	True	25	3.477887530571028	hqer__induced sampler prototype	2.0
	front41Data	HQER-SFA	hqer__induced	True	44	3.498711524600965	hqer__induced sampler prototype	4.0
	front41Data	Quantile-SFA	hqer__induced	True	61	-2.3005160326567093	hqer__induced sampler prototype	4.0
	front41Data	HQER-SFA	hqer__induced	True	42	-2.294713442220636	hqer__induced sampler prototype	2.0
	front41Data	Quantile-SFA	hqer__induced	True	13	-0.1520052621476198	hqer__induced sampler prototype	4.0
	front41Data	HQER-SFA	hqer__induced	True	46	0.0608277479496898	hqer__induced sampler prototype	4.0
	front41Data	Quantile-SFA	hqer__induced	True	55	18.141266261257552	hqer__induced sampler prototype	3.0
	front41Data	HQER-SFA	hqer__induced	True	52	18.13345540574668	hqer__induced sampler prototype	1.0
	front41Data	Quantile-SFA	hqer__induced	True	51	4.515119259539133	hqer__induced sampler prototype	4.0
	front41Data	HQER-SFA	hqer__induced	True	49	4.522922472724548	hqer__induced sampler prototype	4.0
	front41Data	Quantile-SFA	hqer__induced	True	26	16.88627561450987	hqer__induced sampler prototype	4.0
	front41Data	HQER-SFA	hqer__induced	True	21	16.89399164653679	hqer__induced sampler prototype	4.0
	front41Data	Quantile-SFA	hqer__induced	True	37	-7.093315707134296	hqer__induced sampler prototype	2.0
	front41Data	HQER-SFA	hqer__induced	True	35	-7.039732887422278	hqer__induced sampler prototype	4.0
	front41Data	Quantile-SFA	hqer__induced	True	15	-2.9730478578963475	hqer__induced sampler prototype	4.0
	front41Data	HQER-SFA	hqer__induced	True	37	-2.940156082747021	hqer__induced sampler prototype	4.0
	front41Data	Quantile-SFA	hqer__induced	True	29	13.34089936576436	hqer__induced sampler prototype	4.0
	front41Data	HQER-SFA	hqer__induced	True	27	13.372052704725384	hqer__induced sampler prototype	4.0
	front41Data	Quantile-SFA	hqer__induced	True	36	-1.3749690003769923	hqer__induced sampler prototype	4.0
	front41Data	HQER-SFA	hqer__induced	True	32	-1.3178815208015506	hqer__induced sampler prototype	4.0
	front41Data	Quantile-SFA	hqer__induced	True	27	6.443517205227123	hqer__induced sampler prototype	2.0
	front41Data	HQER-SFA	hqer__induced	True	29	6.460760526016747	hqer__induced sampler prototype	2.0
	front41Data	Quantile-SFA	hqer__induced	True	16	0.948828052585322	hqer__induced sampler prototype	2.0
	front41Data	HQER-SFA	hqer__induced	True	31	0.998395496328454	hqer__induced sampler prototype	4.0
	front41Data	Quantile-SFA	hqer__induced	True	5	-3.905165561118784	hqer__induced sampler prototype	2.0
	front41Data	HQER-SFA	hqer__induced	True	5	-3.888329902912583	hqer__induced sampler prototype	2.0
	front41Data	Quantile-SFA	hqer__induced	True	26	1.964408481206397	hqer__induced sampler prototype	2.0
	front41Data	HQER-SFA	hqer__induced	True	25	1.9809443116366343	hqer__induced sampler prototype	2.0
	front41Data	Quantile-SFA	hqer__induced	True	30	19.035823661978338	hqer__induced sampler prototype	4.0
	front41Data	HQER-SFA	hqer__induced	True	35	19.033814081653976	hqer__induced sampler prototype	4.0
	front41Data	Quantile-SFA	hqer__induced	True	28	6.418348297679414	hqer__induced sampler prototype	2.0
	front41Data	HQER-SFA	hqer__induced	True	35	6.507867702851683	hqer__induced sampler prototype	4.0
	front41Data	Quantile-SFA	hqer__induced	True	26	7.725394585301387	hqer__induced sampler prototype	4.0
	front41Data	HQER-SFA	hqer__induced	True	35	7.738027539987707	hqer__induced sampler prototype	2.0
	front41Data	Quantile-SFA	hqer__induced	True	14	10.532350252978366	hqer__induced sampler prototype	2.0
	front41Data	HQER-SFA	hqer__induced	True	16	10.553284820039917	hqer__induced sampler prototype	4.0
	front41Data	Quantile-SFA	hqer__induced	True	38	6.869630737273838	hqer__induced sampler prototype	4.0
	front41Data	HQER-SFA	hqer__induced	True	25	6.226164604036412	hqer__induced sampler prototype	4.0
	front41Data	Quantile-SFA	hqer__induced	True	41	2.504181172390425	hqer__induced sampler prototype	2.0
	front41Data	HQER-SFA	hqer__induced	True	36	2.401530419090339	hqer__induced sampler prototype	4.0
	front41Data	Quantile-SFA	hqer__induced	True	35	-7.562504444589055	hqer__induced sampler prototype	4.0
	front41Data	HQER-SFA	hqer__induced	True	42	-7.491484540099895	hqer__induced sampler prototype	4.0
	front41Data	Quantile-SFA	hqer__induced	True	27	-0.3548132057601853	hqer__induced sampler prototype	4.0
	front41Data	HQER-SFA	hqer__induced	True	50	-0.2139960288327866	hqer__induced sampler prototype	4.0
	front41Data	Quantile-SFA	hqer__induced	True	28	-1.2855426866619486	hqer__induced sampler prototype	4.0
	front41Data	HQER-SFA	hqer__induced	True	27	-1.559371973237397	hqer__induced sampler prototype	4.0
	front41Data	Quantile-SFA	hqer__induced	True	16	-3.8071336068611767	hqer__induced sampler prototype	4.0
	front41Data	HQER-SFA	hqer__induced	True	19	-3.790555873950607	hqer__induced sampler prototype	4.0
	front41Data	Quantile-SFA	hqer__induced	True	19	8.564702284161207	hqer__induced sampler prototype	4.0
	front41Data	HQER-SFA	hqer__induced	True	45	8.584026393437686	hqer__induced sampler prototype	4.0
	front41Data	Quantile-SFA	hqer__induced	True	22	0.0178590442617421	hqer__induced sampler prototype	4.0
	front41Data	HQER-SFA	hqer__induced	True	32	0.0297355873431273	hqer__induced sampler prototype	4.0
	front41Data	Quantile-SFA	hqer__induced	True	38	-0.0589610942687188	hqer__induced sampler prototype	4.0
	front41Data	HQER-SFA	hqer__induced	True	25	-0.0591774919860319	hqer__induced sampler prototype	2.0
	front41Data	Quantile-SFA	hqer__induced	True	6	-8.584086191714428	hqer__induced sampler prototype	2.0
	front41Data	HQER-SFA	hqer__induced	True	24	-8.44664047015101	hqer__induced sampler prototype	2.0
	front41Data	Quantile-SFA	hqer__induced	True	9	-9.639061506770402	hqer__induced sampler prototype	2.0
	front41Data	HQER-SFA	hqer__induced	True	17	-9.530494467301086	hqer__induced sampler prototype	2.0
	front41Data	Quantile-SFA	hqer__induced	True	49	-5.838050063854425	hqer__induced sampler prototype	4.0
	front41Data	HQER-SFA	hqer__induced	True	17	-6.01381837174871	hqer__induced sampler prototype	4.0
	front41Data	Quantile-SFA	hqer__induced	True	19	-2.1115029166386816	hqer__induced sampler prototype	4.0
	front41Data	HQER-SFA	hqer__induced	True	19	-2.0965740512664675	hqer__induced sampler prototype	4.0
	front41Data	Quantile-SFA	hqer__induced	True	21	2.4512038677291903	hqer__induced sampler prototype	4.0
	front41Data	HQER-SFA	hqer__induced	True	27	2.4820677007085323	hqer__induced sampler prototype	4.0
	front41Data	Quantile-SFA	hqer__induced	True	52	6.807302492068532	hqer__induced sampler prototype	2.0
	front41Data	HQER-SFA	hqer__induced	True	50	6.15137842497944	hqer__induced sampler prototype	4.0
	front41Data	Quantile-SFA	hqer__induced	True	37	9.457916275880976	hqer__induced sampler prototype	4.0
	front41Data	HQER-SFA	hqer__induced	True	45	9.449161058608478	hqer__induced sampler prototype	4.0
	front41Data	Quantile-SFA	hqer__induced	True	46	-3.2032343765126954	hqer__induced sampler prototype	4.0
	front41Data	HQER-SFA	hqer__induced	True	28	-3.1649598000368813	hqer__induced sampler prototype	4.0
	front41Data	Quantile-SFA	hqer__induced	True	36	4.707390202873288	hqer__induced sampler prototype	4.0
	front41Data	HQER-SFA	hqer__induced	True	23	4.708949392625789	hqer__induced sampler prototype	4.0
	front41Data	Quantile-SFA	hqer__induced	True	33	9.65422009529711	hqer__induced sampler prototype	4.0
	front41Data	HQER-SFA	hqer__induced	True	55	9.655520837972592	hqer__induced sampler prototype	4.0
	front41Data	Quantile-SFA	hqer__induced	True	35	3.323555552474948	hqer__induced sampler prototype	4.0
	front41Data	HQER-SFA	hqer__induced	True	29	3.370254550665923	hqer__induced sampler prototype	2.0
	front41Data	Quantile-SFA	hqer__induced	True	16	1.333514635702279	hqer__induced sampler prototype	2.0
	front41Data	HQER-SFA	hqer__induced	True	49	1.7402751414802664	hqer__induced sampler prototype	2.0
	front41Data	Quantile-SFA	hqer__induced	True	18	-0.3521854498930622	hqer__induced sampler prototype	4.0
	front41Data	HQER-SFA	hqer__induced	True	24	-0.3508676123291835	hqer__induced sampler prototype	2.0
	front41Data	Quantile-SFA	hqer__induced	True	46	-1.4826934802783915	hqer__induced sampler prototype	4.0
	front41Data	HQER-SFA	hqer__induced	True	29	-1.482898580172689	hqer__induced sampler prototype	4.0
	front41Data	Quantile-SFA	hqer__induced	True	23	5.334547952141674	hqer__induced sampler prototype	4.0
	front41Data	HQER-SFA	hqer__induced	True	20	5.327301165666887	hqer__induced sampler prototype	4.0
	front41Data	Quantile-SFA	hqer__induced	True	23	0.2691108127183358	hqer__induced sampler prototype	4.0
	front41Data	HQER-SFA	hqer__induced	True	29	0.2786878581862012	hqer__induced sampler prototype	4.0
	front41Data	Quantile-SFA	hqer__induced	True	25	-4.857292973232514	hqer__induced sampler prototype	4.0
	front41Data	HQER-SFA	hqer__induced	True	34	-4.850002076070672	hqer__induced sampler prototype	3.0
	front41Data	Quantile-SFA	hqer__induced	True	8	-1.0276922998934084	hqer__induced sampler prototype	2.0
	front41Data	HQER-SFA	hqer__induced	True	31	-0.798458603747652	hqer__induced sampler prototype	2.0
	front41Data	Quantile-SFA	hqer__induced	True	24	-1.95103971771824	hqer__induced sampler prototype	4.0
	front41Data	HQER-SFA	hqer__induced	True	15	-1.940942135990676	hqer__induced sampler prototype	4.0
	front41Data	Quantile-SFA	hqer__induced	True	12	-6.285775229754639	hqer__induced sampler prototype	4.0
	front41Data	HQER-SFA	hqer__induced	True	20	-5.896797852569152	hqer__induced sampler prototype	3.0
	front41Data	Quantile-SFA	hqer__induced	True	56	1.6205003685390331	hqer__induced sampler prototype	2.0
	front41Data	HQER-SFA	hqer__induced	True	20	1.6254806997233044	hqer__induced sampler prototype	4.0
	front41Data	Quantile-SFA	hqer__induced	True	20	5.4611087984954825	hqer__induced sampler prototype	2.0
	front41Data	HQER-SFA	hqer__induced	True	22	5.467013851026797	hqer__induced sampler prototype	2.0
	front41Data	Quantile-SFA	hqer__induced	True	51	8.855760903897155	hqer__induced sampler prototype	2.0
	front41Data	HQER-SFA	hqer__induced	True	42	8.722137284526898	hqer__induced sampler prototype	4.0
	front41Data	Quantile-SFA	hqer__induced	True	25	7.679534637276081	hqer__induced sampler prototype	4.0
	front41Data	HQER-SFA	hqer__induced	True	21	7.626729688691393	hqer__induced sampler prototype	4.0
	front41Data	Quantile-SFA	hqer__induced	True	18	6.721932029841812	hqer__induced sampler prototype	4.0

experiment	dataset	model	scenario	converged	n__iter	loglik	warning__message	best__start__id
	front41Data	HQER-SFA	hqer__induced	True	14	6.735136810620033	hqer__induced sampler prototype	4.0
	front41Data	Quantile-SFA	hqer__induced	True	6	-7.776640856778496	hqer__induced sampler prototype	2.0
	front41Data	HQER-SFA	hqer__induced	True	7	-7.769812314415416	hqer__induced sampler prototype	2.0
	front41Data	Quantile-SFA	hqer__induced	True	32	6.740506348647365	hqer__induced sampler prototype	4.0
	front41Data	HQER-SFA	hqer__induced	True	29	6.761817145111224	hqer__induced sampler prototype	4.0
	front41Data	Quantile-SFA	hqer__induced	True	17	2.825971716499874	hqer__induced sampler prototype	2.0
	front41Data	HQER-SFA	hqer__induced	True	18	2.836379867884798	hqer__induced sampler prototype	2.0
	front41Data	Quantile-SFA	hqer__induced	True	16	6.807313789426215	hqer__induced sampler prototype	2.0
	front41Data	HQER-SFA	hqer__induced	True	39	6.972362902793938	hqer__induced sampler prototype	2.0
	front41Data	Quantile-SFA	hqer__induced	True	31	10.030816969203505	hqer__induced sampler prototype	2.0
	front41Data	HQER-SFA	hqer__induced	True	20	9.957450055417906	hqer__induced sampler prototype	2.0
	front41Data	Quantile-SFA	hqer__induced	True	23	-3.5023919330076287	hqer__induced sampler prototype	2.0
	front41Data	HQER-SFA	hqer__induced	True	21	-3.489948983733527	hqer__induced sampler prototype	2.0
	front41Data	Quantile-SFA	hqer__induced	True	27	9.12998769854659	hqer__induced sampler prototype	4.0
	front41Data	HQER-SFA	hqer__induced	True	29	9.134981439781397	hqer__induced sampler prototype	4.0
	front41Data	Quantile-SFA	hqer__induced	True	38	-3.0289558861858352	hqer__induced sampler prototype	4.0
	front41Data	HQER-SFA	hqer__induced	True	17	-3.041848195646573	hqer__induced sampler prototype	4.0
	front41Data	Quantile-SFA	hqer__induced	True	41	-5.034441133971563	hqer__induced sampler prototype	2.0
	front41Data	HQER-SFA	hqer__induced	True	51	-5.0328109427608165	hqer__induced sampler prototype	2.0
	front41Data	Quantile-SFA	hqer__induced	True	38	-1.857016185836412	hqer__induced sampler prototype	4.0
	front41Data	HQER-SFA	hqer__induced	True	26	-1.857098048937288	hqer__induced sampler prototype	4.0
	front41Data	Quantile-SFA	hqer__induced	True	28	3.26678304739172	hqer__induced sampler prototype	4.0
	front41Data	HQER-SFA	hqer__induced	True	42	3.5618256020291565	hqer__induced sampler prototype	4.0
	front41Data	Quantile-SFA	hqer__induced	True	37	5.007740804426935	hqer__induced sampler prototype	4.0
	front41Data	HQER-SFA	hqer__induced	True	31	5.015175882712381	hqer__induced sampler prototype	4.0
	front41Data	Quantile-SFA	hqer__induced	True	15	9.248443787949835	hqer__induced sampler prototype	2.0
	front41Data	HQER-SFA	hqer__induced	True	36	9.371084007947337	hqer__induced sampler prototype	4.0
	front41Data	Quantile-SFA	hqer__induced	True	10	-1.8111164786817744	hqer__induced sampler prototype	4.0
	front41Data	HQER-SFA	hqer__induced	True	22	-1.5913043449194155	hqer__induced sampler prototype	4.0
	front41Data	Quantile-SFA	hqer__induced	True	35	-6.012066474641203	hqer__induced sampler prototype	4.0
	front41Data	HQER-SFA	hqer__induced	True	25	-5.9960721473363074	hqer__induced sampler prototype	4.0
	front41Data	Quantile-SFA	hqer__induced	True	22	7.629027359730061	hqer__induced sampler prototype	2.0
	front41Data	HQER-SFA	hqer__induced	True	23	7.618988350774365	hqer__induced sampler prototype	2.0
	front41Data	Quantile-SFA	hqer__induced	True	20	-4.847977886318386	hqer__induced sampler prototype	4.0
	front41Data	HQER-SFA	hqer__induced	True	15	-4.847537308322289	hqer__induced sampler prototype	4.0
	front41Data	Quantile-SFA	hqer__induced	True	26	1.475297624825052	hqer__induced sampler prototype	2.0
	front41Data	HQER-SFA	hqer__induced	True	22	1.4683826218689728	hqer__induced sampler prototype	2.0
	front41Data	Quantile-SFA	hqer__induced	True	20	2.295529520689229	hqer__induced sampler prototype	4.0
	front41Data	HQER-SFA	hqer__induced	True	23	2.328571440028116	hqer__induced sampler prototype	4.0
	front41Data	Quantile-SFA	hqer__induced	True	29	3.668766040171034	hqer__induced sampler prototype	4.0
	front41Data	HQER-SFA	hqer__induced	True	22	3.698495993663216	hqer__induced sampler prototype	4.0
	front41Data	Quantile-SFA	hqer__induced	True	32	-14.479025394746408	hqer__induced sampler prototype	2.0
	front41Data	HQER-SFA	hqer__induced	True	30	-14.654748585667626	hqer__induced sampler prototype	4.0
	front41Data	Quantile-SFA	hqer__induced	True	27	9.727215524800645	hqer__induced sampler prototype	4.0
	front41Data	HQER-SFA	hqer__induced	True	25	9.74127085632784	hqer__induced sampler prototype	2.0
	front41Data	Quantile-SFA	hqer__induced	True	34	-2.120470570718184	hqer__induced sampler prototype	4.0
	front41Data	HQER-SFA	hqer__induced	True	34	-2.260566615864674	hqer__induced sampler prototype	2.0
	front41Data	Quantile-SFA	hqer__induced	True	21	9.766421691849278	hqer__induced sampler prototype	2.0
	front41Data	HQER-SFA	hqer__induced	True	20	9.757346687852063	hqer__induced sampler prototype	2.0
	front41Data	Quantile-SFA	hqer__induced	True	19	4.206039546919838	hqer__induced sampler prototype	4.0
	front41Data	HQER-SFA	hqer__induced	True	19	4.230927137587883	hqer__induced sampler prototype	4.0
	front41Data	Quantile-SFA	hqer__induced	True	18	-7.038657245657832	hqer__induced sampler prototype	4.0
	front41Data	HQER-SFA	hqer__induced	True	22	-7.045662394717677	hqer__induced sampler prototype	4.0
	front41Data	Quantile-SFA	hqer__induced	True	36	15.49410229852662	hqer__induced sampler prototype	1.0
	front41Data	HQER-SFA	hqer__induced	True	44	15.439985040939842	hqer__induced sampler prototype	3.0
	front41Data	Quantile-SFA	hqer__induced	True	18	3.0197024651499493	hqer__induced sampler prototype	2.0
	front41Data	HQER-SFA	hqer__induced	True	23	3.0409238941211103	hqer__induced sampler prototype	2.0
	front41Data	Quantile-SFA	hqer__induced	True	24	-5.66309240601605	hqer__induced sampler prototype	4.0
	front41Data	HQER-SFA	hqer__induced	True	31	-5.65099829063298	hqer__induced sampler prototype	4.0
	front41Data	Quantile-SFA	hqer__induced	True	23	1.9554435440766884	hqer__induced sampler prototype	4.0
	front41Data	HQER-SFA	hqer__induced	True	14	1.918655612157085	hqer__induced sampler prototype	4.0
	front41Data	Quantile-SFA	hqer__induced	True	27	-9.637090223231388	hqer__induced sampler prototype	4.0
	front41Data	HQER-SFA	hqer__induced	True	32	-9.618943104149556	hqer__induced sampler prototype	2.0
	front41Data	Quantile-SFA	hqer__induced	True	47	6.525819173490534	hqer__induced sampler prototype	4.0
	front41Data	HQER-SFA	hqer__induced	True	29	6.537949276353352	hqer__induced sampler prototype	2.0
	front41Data	Quantile-SFA	hqer__induced	True	7	-4.082538450814358	hqer__induced sampler prototype	2.0
	front41Data	HQER-SFA	hqer__induced	True	46	-3.2832775306411026	hqer__induced sampler prototype	2.0
	front41Data	Quantile-SFA	hqer__induced	True	15	13.15994045580655	hqer__induced sampler prototype	2.0
	front41Data	HQER-SFA	hqer__induced	True	39	15.164297608231326	hqer__induced sampler prototype	2.0
	front41Data	Quantile-SFA	hqer__induced	True	18	5.7489957039640425	hqer__induced sampler prototype	4.0
	front41Data	HQER-SFA	hqer__induced	True	24	5.755962244651636	hqer__induced sampler prototype	4.0
	front41Data	Quantile-SFA	hqer__induced	True	19	-10.286008903982069	hqer__induced sampler prototype	4.0
	front41Data	HQER-SFA	hqer__induced	True	43	-10.114666564529172	hqer__induced sampler prototype	2.0
	front41Data	Quantile-SFA	hqer__induced	True	34	-4.005877411807099	hqer__induced sampler prototype	4.0
	front41Data	HQER-SFA	hqer__induced	True	32	-3.882808345386312	hqer__induced sampler prototype	4.0
	front41Data	Quantile-SFA	hqer__induced	True	37	6.76041184537516	hqer__induced sampler prototype	2.0
	front41Data	HQER-SFA	hqer__induced	True	36	6.759880261975369	hqer__induced sampler prototype	4.0
	front41Data	Quantile-SFA	hqer__induced	True	25	6.987624484055271	hqer__induced sampler prototype	4.0
	front41Data	HQER-SFA	hqer__induced	True	29	6.996801337517078	hqer__induced sampler prototype	4.0
	front41Data	Quantile-SFA	hqer__induced	True	18	6.555112554281795	hqer__induced sampler prototype	2.0
	front41Data	HQER-SFA	hqer__induced	True	17	6.503494237007921	hqer__induced sampler prototype	2.0
	front41Data	Quantile-SFA	hqer__induced	True	19	4.201262917158676	hqer__induced sampler prototype	4.0
	front41Data	HQER-SFA	hqer__induced	True	40	4.288935705954603	hqer__induced sampler prototype	4.0
	front41Data	Quantile-SFA	hqer__induced	True	34	0.9710126496986936	hqer__induced sampler prototype	2.0
	front41Data	HQER-SFA	hqer__induced	True	35	0.9720196802132992	hqer__induced sampler prototype	4.0
	front41Data	Quantile-SFA	hqer__induced	True	19	-3.585417489709496	hqer__induced sampler prototype	4.0
	front41Data	HQER-SFA	hqer__induced	True	22	-3.5595474466581045	hqer__induced sampler prototype	4.0
	front41Data	Quantile-SFA	hqer__induced	True	12	1.4643520552666658	hqer__induced sampler prototype	2.0
	front41Data	HQER-SFA	hqer__induced	True	11	1.4868510206821772	hqer__induced sampler prototype	2.0
	front41Data	Quantile-SFA	hqer__induced	True	34	11.93636944581195	hqer__induced sampler prototype	2.0
	front41Data	HQER-SFA	hqer__induced	True	38	11.96510308148899	hqer__induced sampler prototype	4.0
	front41Data	Quantile-SFA	hqer__induced	True	17	10.17289551190246	hqer__induced sampler prototype	4.0
	front41Data	HQER-SFA	hqer__induced	True	33	10.246665992355403	hqer__induced sampler prototype	4.0
	front41Data	Quantile-SFA	hqer__induced	True	54	-1.4208840042560786	hqer__induced sampler prototype	2.0
	front41Data	HQER-SFA	hqer__induced	True	42	-1.4096524135777408	hqer__induced sampler prototype	2.0
	front41Data	Quantile-SFA	hqer__induced	True	28	0.1055978480641894	hqer__induced sampler prototype	2.0
	front41Data	HQER-SFA	hqer__induced	True	57	0.129464125177116	hqer__induced sampler prototype	2.0
	front41Data	Quantile-SFA	hqer__induced	True	33	-1.8256576654618168	hqer__induced sampler prototype	2.0
	front41Data	HQER-SFA	hqer__induced	True	44	-1.7810872340961454	hqer__induced sampler prototype	2.0
	front41Data	Quantile-SFA	hqer__induced	True	26	-5.564346868419432	hqer__induced sampler prototype	4.0
	front41Data	HQER-SFA	hqer__induced	True	22	-5.550696358158949	hqer__induced sampler prototype	4.0
	front41Data	Quantile-SFA	hqer__induced	True	28	10.841515642680108	hqer__induced sampler prototype	2.0
	front41Data	HQER-SFA	hqer__induced	True	50	10.929851811490444	hqer__induced sampler prototype	4.0

experiment	dataset	model	scenario	converged	n__iter	loglik	warning__message	best__start__id
	front41Data	Quantile-SFA	hqer__induced	True	23	2.013564576710573	hqer__induced sampler prototype	4.0
	front41Data	HQER-SFA	hqer__induced	True	20	2.0434094340781224	hqer__induced sampler prototype	2.0
	front41Data	Quantile-SFA	hqer__induced	True	33	-0.6161671191257817	hqer__induced sampler prototype	2.0
	front41Data	HQER-SFA	hqer__induced	True	42	-0.6049989986767885	hqer__induced sampler prototype	4.0
	front41Data	Quantile-SFA	hqer__induced	True	17	2.786972576799025	hqer__induced sampler prototype	4.0
	front41Data	HQER-SFA	hqer__induced	True	17	2.8022405246318303	hqer__induced sampler prototype	4.0
	front41Data	Quantile-SFA	hqer__induced	True	18	-6.166385828967394	hqer__induced sampler prototype	4.0
	front41Data	HQER-SFA	hqer__induced	True	15	-6.161797625155547	hqer__induced sampler prototype	4.0
	front41Data	Quantile-SFA	hqer__induced	True	40	5.824520657284341	hqer__induced sampler prototype	2.0
	front41Data	HQER-SFA	hqer__induced	True	25	5.770426801996198	hqer__induced sampler prototype	2.0
	front41Data	Quantile-SFA	hqer__induced	True	32	14.563403405674366	hqer__induced sampler prototype	4.0
	front41Data	HQER-SFA	hqer__induced	True	47	14.597423240403735	hqer__induced sampler prototype	2.0
	front41Data	Quantile-SFA	hqer__induced	True	20	-0.9542196790569916	hqer__induced sampler prototype	2.0
	front41Data	HQER-SFA	hqer__induced	True	31	-0.9475274099856033	hqer__induced sampler prototype	4.0
	front41Data	Quantile-SFA	hqer__induced	True	33	6.478911356368388	hqer__induced sampler prototype	4.0
	front41Data	HQER-SFA	hqer__induced	True	31	6.486034121334364	hqer__induced sampler prototype	4.0
	front41Data	Quantile-SFA	hqer__induced	True	44	16.8710067101727	hqer__induced sampler prototype	4.0
	front41Data	HQER-SFA	hqer__induced	True	46	16.870884821603156	hqer__induced sampler prototype	4.0
	front41Data	Quantile-SFA	hqer__induced	True	38	-4.984378828089975	hqer__induced sampler prototype	4.0
	front41Data	HQER-SFA	hqer__induced	True	16	-5.0500685215455565	hqer__induced sampler prototype	4.0
	front41Data	Quantile-SFA	hqer__induced	True	32	1.5169952980262018	hqer__induced sampler prototype	4.0
	front41Data	HQER-SFA	hqer__induced	True	17	1.4683331254058467	hqer__induced sampler prototype	4.0
	front41Data	Quantile-SFA	hqer__induced	True	18	5.386069245903567	hqer__induced sampler prototype	2.0
	front41Data	HQER-SFA	hqer__induced	True	18	5.390251441467097	hqer__induced sampler prototype	2.0
	front41Data	Quantile-SFA	hqer__induced	True	33	15.7507662931935	hqer__induced sampler prototype	2.0
	front41Data	HQER-SFA	hqer__induced	True	17	15.702044749309826	hqer__induced sampler prototype	2.0
	front41Data	Quantile-SFA	hqer__induced	True	39	4.708303124106688	hqer__induced sampler prototype	2.0
	front41Data	HQER-SFA	hqer__induced	True	16	4.488371006727152	hqer__induced sampler prototype	2.0
	front41Data	Quantile-SFA	hqer__induced	True	45	-3.719304180531644	hqer__induced sampler prototype	2.0
	front41Data	HQER-SFA	hqer__induced	True	18	-3.849323737317749	hqer__induced sampler prototype	4.0
	front41Data	Quantile-SFA	hqer__induced	True	33	13.840242975040104	hqer__induced sampler prototype	4.0
	front41Data	HQER-SFA	hqer__induced	True	23	13.707623959806911	hqer__induced sampler prototype	4.0
	front41Data	Quantile-SFA	hqer__induced	True	32	3.863192023472181	hqer__induced sampler prototype	2.0
	front41Data	HQER-SFA	hqer__induced	True	36	3.88367784595262	hqer__induced sampler prototype	2.0
	front41Data	Quantile-SFA	hqer__induced	True	16	3.626279372392443	hqer__induced sampler prototype	4.0
	front41Data	HQER-SFA	hqer__induced	True	18	3.637034585805235	hqer__induced sampler prototype	4.0
	front41Data	Quantile-SFA	hqer__induced	True	15	-0.1676343127316535	hqer__induced sampler prototype	4.0
	front41Data	HQER-SFA	hqer__induced	True	11	-0.2030161696859689	hqer__induced sampler prototype	4.0
	front41Data	Quantile-SFA	hqer__induced	True	6	-1.827141613788372	hqer__induced sampler prototype	2.0
	front41Data	HQER-SFA	hqer__induced	True	6	-1.809012335656478	hqer__induced sampler prototype	2.0
	front41Data	Quantile-SFA	hqer__induced	True	42	3.827452961240065	hqer__induced sampler prototype	2.0
	front41Data	HQER-SFA	hqer__induced	True	13	3.7678569961880095	hqer__induced sampler prototype	2.0
	front41Data	Quantile-SFA	hqer__induced	True	39	1.4446720575924	hqer__induced sampler prototype	2.0
	front41Data	HQER-SFA	hqer__induced	True	39	1.42442808991526	hqer__induced sampler prototype	2.0
	front41Data	Quantile-SFA	hqer__induced	True	43	-6.811077787679047	hqer__induced sampler prototype	4.0
	front41Data	HQER-SFA	hqer__induced	True	6	-6.837826641323158	hqer__induced sampler prototype	2.0
	front41Data	Quantile-SFA	hqer__induced	True	19	4.2355664259332535	hqer__induced sampler prototype	4.0
	front41Data	HQER-SFA	hqer__induced	True	60	4.876941403785505	hqer__induced sampler prototype	4.0
	front41Data	Quantile-SFA	hqer__induced	True	25	-9.532518421992132	hqer__induced sampler prototype	4.0
	front41Data	HQER-SFA	hqer__induced	True	48	-9.434721290833336	hqer__induced sampler prototype	2.0
	front41Data	Quantile-SFA	hqer__induced	True	34	2.949875139421112	hqer__induced sampler prototype	2.0
	front41Data	HQER-SFA	hqer__induced	True	45	2.9984075903182523	hqer__induced sampler prototype	3.0
	front41Data	Quantile-SFA	hqer__induced	True	51	15.733435614118337	hqer__induced sampler prototype	2.0
	front41Data	HQER-SFA	hqer__induced	True	42	15.358396512086593	hqer__induced sampler prototype	4.0
	front41Data	Quantile-SFA	hqer__induced	True	42	-4.508107320289188	hqer__induced sampler prototype	4.0
	front41Data	HQER-SFA	hqer__induced	True	44	-4.505643831940606	hqer__induced sampler prototype	4.0
	front41Data	Quantile-SFA	hqer__induced	True	47	-0.1370945089504442	hqer__induced sampler prototype	4.0
	front41Data	HQER-SFA	hqer__induced	True	40	-0.1157448596543003	hqer__induced sampler prototype	4.0
	front41Data	Quantile-SFA	hqer__induced	True	26	-2.381825416996368	hqer__induced sampler prototype	4.0
	front41Data	HQER-SFA	hqer__induced	True	13	-2.44273491136538	hqer__induced sampler prototype	4.0
	front41Data	Quantile-SFA	hqer__induced	True	37	-1.463152713638184	hqer__induced sampler prototype	4.0
	front41Data	HQER-SFA	hqer__induced	True	36	-1.4451528987840665	hqer__induced sampler prototype	4.0
	front41Data	Quantile-SFA	hqer__induced	True	21	0.2833591456246711	hqer__induced sampler prototype	4.0
	front41Data	HQER-SFA	hqer__induced	True	19	0.2930446726361546	hqer__induced sampler prototype	4.0
	front41Data	Quantile-SFA	hqer__induced	True	28	-2.820226776396753	hqer__induced sampler prototype	4.0
	front41Data	HQER-SFA	hqer__induced	True	44	-2.809484018966497	hqer__induced sampler prototype	4.0
	front41Data	Quantile-SFA	hqer__induced	True	18	-11.639216497230152	hqer__induced sampler prototype	4.0
	front41Data	HQER-SFA	hqer__induced	True	13	-11.645475515304392	hqer__induced sampler prototype	4.0
	front41Data	Quantile-SFA	hqer__induced	True	17	1.6172488509623482	hqer__induced sampler prototype	2.0
	front41Data	HQER-SFA	hqer__induced	True	29	1.6417607733482955	hqer__induced sampler prototype	2.0
	front41Data	Quantile-SFA	hqer__induced	True	39	6.555267932127069	hqer__induced sampler prototype	4.0
	front41Data	HQER-SFA	hqer__induced	True	27	6.479821095372987	hqer__induced sampler prototype	4.0
	front41Data	Quantile-SFA	hqer__induced	True	32	2.724361137120366	hqer__induced sampler prototype	4.0
	front41Data	HQER-SFA	hqer__induced	True	36	2.7263312643429933	hqer__induced sampler prototype	2.0
	front41Data	Quantile-SFA	hqer__induced	True	21	-1.3325540624472816	hqer__induced sampler prototype	2.0
	front41Data	HQER-SFA	hqer__induced	True	30	-1.3077857947311902	hqer__induced sampler prototype	2.0
	front41Data	Quantile-SFA	hqer__induced	True	17	0.9231921344993448	hqer__induced sampler prototype	2.0
	front41Data	HQER-SFA	hqer__induced	True	21	0.9252306559636302	hqer__induced sampler prototype	2.0
	front41Data	Quantile-SFA	hqer__induced	True	29	6.723687698931337	hqer__induced sampler prototype	4.0
	front41Data	HQER-SFA	hqer__induced	True	19	6.451440735666427	hqer__induced sampler prototype	2.0
	front41Data	Quantile-SFA	hqer__induced	True	24	-1.6809511396266743	hqer__induced sampler prototype	4.0
	front41Data	HQER-SFA	hqer__induced	True	31	-1.6357771355185948	hqer__induced sampler prototype	2.0
	front41Data	Quantile-SFA	hqer__induced	True	22	9.5234111529295	hqer__induced sampler prototype	2.0
	front41Data	HQER-SFA	hqer__induced	True	30	9.567419254836064	hqer__induced sampler prototype	2.0
	front41Data	Quantile-SFA	hqer__induced	True	25	-4.955011264187586	hqer__induced sampler prototype	4.0
	front41Data	HQER-SFA	hqer__induced	True	36	-4.936113554724456	hqer__induced sampler prototype	4.0
	front41Data	Quantile-SFA	hqer__induced	True	41	8.792483501234807	hqer__induced sampler prototype	4.0
	front41Data	HQER-SFA	hqer__induced	True	46	8.816235850332733	hqer__induced sampler prototype	4.0
	front41Data	Quantile-SFA	hqer__induced	True	11	-5.70467504671833	hqer__induced sampler prototype	2.0
	front41Data	HQER-SFA	hqer__induced	True	55	-5.675530589635601	hqer__induced sampler prototype	4.0
	front41Data	Quantile-SFA	hqer__induced	True	23	2.752858243904486	hqer__induced sampler prototype	2.0
	front41Data	HQER-SFA	hqer__induced	True	18	2.768458111610701	hqer__induced sampler prototype	2.0
	front41Data	Quantile-SFA	hqer__induced	True	30	-0.2664170517867079	hqer__induced sampler prototype	4.0
	front41Data	HQER-SFA	hqer__induced	True	34	-0.2674979182911554	hqer__induced sampler prototype	2.0
	front41Data	Quantile-SFA	hqer__induced	True	17	-2.7453465763339766	hqer__induced sampler prototype	4.0
	front41Data	HQER-SFA	hqer__induced	True	19	-2.5355564218010325	hqer__induced sampler prototype	2.0
	front41Data	Quantile-SFA	hqer__induced	True	10	-6.278921688847619	hqer__induced sampler prototype	2.0
	front41Data	HQER-SFA	hqer__induced	True	6	-6.272255744117348	hqer__induced sampler prototype	2.0
	front41Data	Quantile-SFA	hqer__induced	True	37	4.637672749058787	hqer__induced sampler prototype	4.0
	front41Data	HQER-SFA	hqer__induced	True	31	4.62922138692723	hqer__induced sampler prototype	4.0
	front41Data	Quantile-SFA	hqer__induced	True	15	9.361884205164504	hqer__induced sampler prototype	2.0
	front41Data	HQER-SFA	hqer__induced	True	15	9.367444907864217	hqer__induced sampler prototype	2.0
	front41Data	Quantile-SFA	hqer__induced	True	37	-5.33161745631693	hqer__induced sampler prototype	4.0

experiment	dataset	model	scenario	converged	n__iter	loglik	warning__message	best__start__id
	front41Data	HQER-SFA	hqer__induced	True	29	-5.398030761284067	hqer__induced sampler prototype	4.0
	front41Data	Quantile-SFA	hqer__induced	True	19	16.880017501993933	hqer__induced sampler prototype	2.0
	front41Data	HQER-SFA	hqer__induced	True	13	16.858807985640254	hqer__induced sampler prototype	2.0
	front41Data	Quantile-SFA	hqer__induced	True	15	-0.7551880498429976	hqer__induced sampler prototype	4.0
	front41Data	HQER-SFA	hqer__induced	True	41	-0.7076871289234746	hqer__induced sampler prototype	4.0
	front41Data	Quantile-SFA	hqer__induced	True	45	2.4109051246790028	hqer__induced sampler prototype	4.0
	front41Data	HQER-SFA	hqer__induced	True	17	2.3973236967210165	hqer__induced sampler prototype	2.0
	front41Data	Quantile-SFA	hqer__induced	True	34	-4.903759189546891	hqer__induced sampler prototype	4.0
	front41Data	HQER-SFA	hqer__induced	True	9	-5.078027376641308	hqer__induced sampler prototype	2.0
	front41Data	Quantile-SFA	hqer__induced	True	21	5.198057730192618	hqer__induced sampler prototype	4.0
	front41Data	HQER-SFA	hqer__induced	True	39	5.208640390242156	hqer__induced sampler prototype	4.0
	front41Data	Quantile-SFA	hqer__induced	True	20	5.197156137300157	hqer__induced sampler prototype	2.0
	front41Data	HQER-SFA	hqer__induced	True	16	5.18161119048158	hqer__induced sampler prototype	2.0
	front41Data	Quantile-SFA	hqer__induced	True	9	-4.10886871037036	hqer__induced sampler prototype	2.0
	front41Data	HQER-SFA	hqer__induced	True	9	-4.099522989663074	hqer__induced sampler prototype	2.0
	front41Data	Quantile-SFA	hqer__induced	True	18	5.727906789538226	hqer__induced sampler prototype	4.0
	front41Data	HQER-SFA	hqer__induced	True	19	5.7144980394658536	hqer__induced sampler prototype	4.0
	front41Data	Quantile-SFA	hqer__induced	True	43	18.673211064157783	hqer__induced sampler prototype	4.0
	front41Data	HQER-SFA	hqer__induced	True	16	18.66253598443117	hqer__induced sampler prototype	2.0
	front41Data	Quantile-SFA	hqer__induced	True	14	-3.689312176071416	hqer__induced sampler prototype	4.0
	front41Data	HQER-SFA	hqer__induced	True	28	-3.652300807743016	hqer__induced sampler prototype	4.0
	front41Data	Quantile-SFA	hqer__induced	True	21	10.997570491307872	hqer__induced sampler prototype	4.0
	front41Data	HQER-SFA	hqer__induced	True	24	11.009116058042832	hqer__induced sampler prototype	4.0
	front41Data	Quantile-SFA	hqer__induced	True	28	-5.812866452222811	hqer__induced sampler prototype	4.0
	front41Data	HQER-SFA	hqer__induced	True	8	-5.9606096277538985	hqer__induced sampler prototype	2.0
	front41Data	Quantile-SFA	hqer__induced	True	46	6.893322249461788	hqer__induced sampler prototype	2.0
	front41Data	HQER-SFA	hqer__induced	True	46	6.891795816352047	hqer__induced sampler prototype	4.0
	front41Data	Quantile-SFA	hqer__induced	True	9	-8.958634670712065	hqer__induced sampler prototype	2.0
	front41Data	HQER-SFA	hqer__induced	True	13	-8.940833192651166	hqer__induced sampler prototype	2.0
	front41Data	Quantile-SFA	hqer__induced	True	19	6.305975299354951	hqer__induced sampler prototype	4.0
	front41Data	HQER-SFA	hqer__induced	True	17	6.323934317534881	hqer__induced sampler prototype	2.0
	front41Data	Quantile-SFA	hqer__induced	True	47	13.22710048905103	hqer__induced sampler prototype	4.0
	front41Data	HQER-SFA	hqer__induced	True	36	13.252559332139242	hqer__induced sampler prototype	2.0
	front41Data	Quantile-SFA	hqer__induced	True	21	2.1575453881182134	hqer__induced sampler prototype	2.0
	front41Data	HQER-SFA	hqer__induced	True	50	2.173018725704955	hqer__induced sampler prototype	4.0
	front41Data	Quantile-SFA	hqer__induced	True	41	12.787070207161516	hqer__induced sampler prototype	4.0
	front41Data	HQER-SFA	hqer__induced	True	39	12.819898912310627	hqer__induced sampler prototype	2.0
	front41Data	Quantile-SFA	hqer__induced	True	24	10.357668692716512	hqer__induced sampler prototype	2.0
	front41Data	HQER-SFA	hqer__induced	True	17	10.372146657115865	hqer__induced sampler prototype	2.0
	front41Data	Quantile-SFA	hqer__induced	True	20	11.1929301898527	hqer__induced sampler prototype	2.0
	front41Data	HQER-SFA	hqer__induced	True	20	11.213385391162896	hqer__induced sampler prototype	2.0
	front41Data	Quantile-SFA	hqer__induced	True	27	2.601898414205836	hqer__induced sampler prototype	2.0
	front41Data	HQER-SFA	hqer__induced	True	23	2.595920769804533	hqer__induced sampler prototype	2.0
	front41Data	Quantile-SFA	hqer__induced	True	36	3.884165653342999	hqer__induced sampler prototype	2.0
	front41Data	HQER-SFA	hqer__induced	True	34	3.883984545379768	hqer__induced sampler prototype	2.0
	front41Data	Quantile-SFA	hqer__induced	True	35	8.092153471810843	hqer__induced sampler prototype	4.0
	front41Data	HQER-SFA	hqer__induced	True	24	8.105042033092282	hqer__induced sampler prototype	2.0
	front41Data	Quantile-SFA	hqer__induced	True	36	3.4071887298684427	hqer__induced sampler prototype	4.0
	front41Data	HQER-SFA	hqer__induced	True	30	3.3952411668489426	hqer__induced sampler prototype	2.0
	front41Data	Quantile-SFA	hqer__induced	True	47	10.144757471177478	hqer__induced sampler prototype	4.0
	front41Data	HQER-SFA	hqer__induced	True	7	5.413953440683594	hqer__induced sampler prototype	2.0
	front41Data	Quantile-SFA	hqer__induced	True	26	11.349953381187868	hqer__induced sampler prototype	2.0
	front41Data	HQER-SFA	hqer__induced	True	33	11.358885297054128	hqer__induced sampler prototype	4.0
	front41Data	Quantile-SFA	hqer__induced	True	13	7.530491563670283	hqer__induced sampler prototype	2.0
	front41Data	HQER-SFA	hqer__induced	True	15	7.532912110620495	hqer__induced sampler prototype	2.0
	front41Data	Quantile-SFA	hqer__induced	True	27	6.272522850495971	hqer__induced sampler prototype	4.0
	front41Data	HQER-SFA	hqer__induced	True	21	6.267675296718187	hqer__induced sampler prototype	4.0
	front41Data	Quantile-SFA	hqer__induced	True	24	10.875504037845229	hqer__induced sampler prototype	4.0
	front41Data	HQER-SFA	hqer__induced	True	12	10.79644620836964	hqer__induced sampler prototype	4.0
	front41Data	Quantile-SFA	hqer__induced	True	23	3.0575214517457305	hqer__induced sampler prototype	2.0
	front41Data	HQER-SFA	hqer__induced	True	27	3.1558143465400823	hqer__induced sampler prototype	2.0
	front41Data	Quantile-SFA	hqer__induced	True	46	-10.253646217590187	hqer__induced sampler prototype	2.0
	front41Data	HQER-SFA	hqer__induced	True	17	-10.38234724147152	hqer__induced sampler prototype	4.0
	front41Data	Quantile-SFA	hqer__induced	True	24	22.53564392769939	hqer__induced sampler prototype	4.0
	front41Data	HQER-SFA	hqer__induced	True	21	22.65033410811178	hqer__induced sampler prototype	4.0
	front41Data	Quantile-SFA	hqer__induced	True	36	9.644849462379751	hqer__induced sampler prototype	2.0
	front41Data	HQER-SFA	hqer__induced	True	20	9.715625265088727	hqer__induced sampler prototype	4.0
	front41Data	Quantile-SFA	hqer__induced	True	40	13.066897918310069	hqer__induced sampler prototype	2.0
	front41Data	HQER-SFA	hqer__induced	True	37	13.186737713287496	hqer__induced sampler prototype	2.0
	front41Data	Quantile-SFA	hqer__induced	True	31	12.920265160202806	hqer__induced sampler prototype	4.0
	front41Data	HQER-SFA	hqer__induced	True	42	12.949009714126046	hqer__induced sampler prototype	4.0
	front41Data	Quantile-SFA	hqer__induced	True	26	21.10747179896044	hqer__induced sampler prototype	4.0
	front41Data	HQER-SFA	hqer__induced	True	32	21.235307629236484	hqer__induced sampler prototype	4.0
	front41Data	Quantile-SFA	hqer__induced	True	25	-1.1830986306158415	hqer__induced sampler prototype	2.0
	front41Data	HQER-SFA	hqer__induced	True	30	-1.0890182983751373	hqer__induced sampler prototype	2.0
	front41Data	Quantile-SFA	hqer__induced	True	21	11.561270033734582	hqer__induced sampler prototype	2.0
	front41Data	HQER-SFA	hqer__induced	True	23	11.699616029445854	hqer__induced sampler prototype	2.0
	front41Data	Quantile-SFA	hqer__induced	True	21	5.032854315156615	hqer__induced sampler prototype	2.0
	front41Data	HQER-SFA	hqer__induced	True	42	5.236415077585367	hqer__induced sampler prototype	4.0
	front41Data	Quantile-SFA	hqer__induced	True	21	-19.654970294183364	hqer__induced sampler prototype	2.0
	front41Data	HQER-SFA	hqer__induced	True	33	-19.602110627557167	hqer__induced sampler prototype	4.0
	front41Data	Quantile-SFA	hqer__induced	True	27	25.51221323080693	hqer__induced sampler prototype	4.0
	front41Data	HQER-SFA	hqer__induced	True	24	25.55902379730463	hqer__induced sampler prototype	4.0
	front41Data	Quantile-SFA	hqer__induced	True	30	-10.036936571457318	hqer__induced sampler prototype	4.0
	front41Data	HQER-SFA	hqer__induced	True	26	-9.936148562389125	hqer__induced sampler prototype	4.0
	front41Data	Quantile-SFA	hqer__induced	True	25	12.090882544786798	hqer__induced sampler prototype	4.0
	front41Data	HQER-SFA	hqer__induced	True	37	12.16876704754202	hqer__induced sampler prototype	4.0
	front41Data	Quantile-SFA	hqer__induced	True	17	20.56774668186957	hqer__induced sampler prototype	4.0
	front41Data	HQER-SFA	hqer__induced	True	12	20.785725891826893	hqer__induced sampler prototype	4.0
	front41Data	Quantile-SFA	hqer__induced	True	29	-24.07836296045935	hqer__induced sampler prototype	4.0
	front41Data	HQER-SFA	hqer__induced	True	23	-24.007515615518805	hqer__induced sampler prototype	2.0
	front41Data	Quantile-SFA	hqer__induced	True	32	21.35237697317434	hqer__induced sampler prototype	4.0
	front41Data	HQER-SFA	hqer__induced	True	21	21.41497803096864	hqer__induced sampler prototype	4.0
	front41Data	Quantile-SFA	hqer__induced	True	36	3.0432023432439053	hqer__induced sampler prototype	2.0
	front41Data	HQER-SFA	hqer__induced	True	44	3.1547156409986767	hqer__induced sampler prototype	2.0
	front41Data	Quantile-SFA	hqer__induced	True	34	-42.766970230713454	hqer__induced sampler prototype	2.0
	front41Data	HQER-SFA	hqer__induced	True	37	-42.66476899563019	hqer__induced sampler prototype	2.0
	front41Data	Quantile-SFA	hqer__induced	True	39	-2.294496902564319	hqer__induced sampler prototype	2.0
	front41Data	HQER-SFA	hqer__induced	True	35	-2.211098150540426	hqer__induced sampler prototype	4.0
	front41Data	Quantile-SFA	hqer__induced	True	34	28.07915003229957	hqer__induced sampler prototype	2.0
	front41Data	HQER-SFA	hqer__induced	True	24	28.17016307106265	hqer__induced sampler prototype	2.0
	front41Data	Quantile-SFA	hqer__induced	True	37	23.249271266093388	hqer__induced sampler prototype	4.0
	front41Data	HQER-SFA	hqer__induced	True	42	23.39715092205025	hqer__induced sampler prototype	4.0

experiment	dataset	model	scenario	converged	n__iter	loglik	warning__message	best__start__id
	front41Data	Quantile-SFA	hqer__induced	True	46	13.12429167376908	hqer__induced sampler prototype	4.0
	front41Data	HQER-SFA	hqer__induced	True	44	13.11336125389897	hqer__induced sampler prototype	4.0
	front41Data	Quantile-SFA	hqer__induced	True	29	-4.907831686355442	hqer__induced sampler prototype	2.0
	front41Data	HQER-SFA	hqer__induced	True	26	-4.826699652722997	hqer__induced sampler prototype	2.0
	front41Data	Quantile-SFA	hqer__induced	True	20	5.4960775590084125	hqer__induced sampler prototype	2.0
	front41Data	HQER-SFA	hqer__induced	True	11	5.59275537280395	hqer__induced sampler prototype	2.0
	front41Data	Quantile-SFA	hqer__induced	True	32	-0.7095590158789111	hqer__induced sampler prototype	4.0
	front41Data	HQER-SFA	hqer__induced	True	32	-0.6377536648534949	hqer__induced sampler prototype	4.0
	front41Data	Quantile-SFA	hqer__induced	True	55	-16.348956808509975	hqer__induced sampler prototype	4.0
	front41Data	HQER-SFA	hqer__induced	True	15	-16.219024239337173	hqer__induced sampler prototype	2.0
	front41Data	Quantile-SFA	hqer__induced	True	29	0.8213065082652973	hqer__induced sampler prototype	4.0
	front41Data	HQER-SFA	hqer__induced	True	25	0.8807745337443773	hqer__induced sampler prototype	2.0
	front41Data	Quantile-SFA	hqer__induced	True	23	27.185361345052577	hqer__induced sampler prototype	4.0
	front41Data	HQER-SFA	hqer__induced	True	25	27.27699172133501	hqer__induced sampler prototype	2.0
	front41Data	Quantile-SFA	hqer__induced	True	44	-4.117206059359716	hqer__induced sampler prototype	5.0
	front41Data	HQER-SFA	hqer__induced	True	60	-4.000124262919577	hqer__induced sampler prototype	2.0
	front41Data	Quantile-SFA	hqer__induced	True	29	-8.755916945836196	hqer__induced sampler prototype	4.0
	front41Data	HQER-SFA	hqer__induced	True	25	-8.63895356515766	hqer__induced sampler prototype	4.0
	front41Data	Quantile-SFA	hqer__induced	True	26	-13.851931345022964	hqer__induced sampler prototype	4.0
	front41Data	HQER-SFA	hqer__induced	True	40	-13.7450802908063	hqer__induced sampler prototype	2.0
	front41Data	Quantile-SFA	hqer__induced	True	26	19.828276188939576	hqer__induced sampler prototype	4.0
	front41Data	HQER-SFA	hqer__induced	True	24	19.906315542272907	hqer__induced sampler prototype	4.0
	front41Data	Quantile-SFA	hqer__induced	True	31	-11.532728009301508	hqer__induced sampler prototype	2.0
	front41Data	HQER-SFA	hqer__induced	True	29	-11.446946758894144	hqer__induced sampler prototype	2.0
	front41Data	Quantile-SFA	hqer__induced	True	28	22.100565866808346	hqer__induced sampler prototype	2.0
	front41Data	HQER-SFA	hqer__induced	True	23	22.210625705401487	hqer__induced sampler prototype	2.0
	front41Data	Quantile-SFA	hqer__induced	True	30	-10.585165509994695	hqer__induced sampler prototype	4.0
	front41Data	HQER-SFA	hqer__induced	True	30	-10.460584327225613	hqer__induced sampler prototype	4.0
	front41Data	Quantile-SFA	hqer__induced	True	30	-2.8765205829393983	hqer__induced sampler prototype	4.0
	front41Data	HQER-SFA	hqer__induced	True	23	-2.888942852999042	hqer__induced sampler prototype	2.0
	front41Data	Quantile-SFA	hqer__induced	True	44	6.537213320147413	hqer__induced sampler prototype	2.0
	front41Data	HQER-SFA	hqer__induced	True	28	6.634166813459004	hqer__induced sampler prototype	4.0
	front41Data	Quantile-SFA	hqer__induced	True	23	-22.635292600655102	hqer__induced sampler prototype	4.0
	front41Data	HQER-SFA	hqer__induced	True	44	-22.59875239471257	hqer__induced sampler prototype	4.0
	front41Data	Quantile-SFA	hqer__induced	True	41	16.497642161292294	hqer__induced sampler prototype	4.0
	front41Data	HQER-SFA	hqer__induced	True	33	16.556499642899492	hqer__induced sampler prototype	2.0
	front41Data	Quantile-SFA	hqer__induced	True	21	32.567352794978035	hqer__induced sampler prototype	4.0
	front41Data	HQER-SFA	hqer__induced	True	24	32.62658505535796	hqer__induced sampler prototype	4.0
	front41Data	Quantile-SFA	hqer__induced	True	22	-13.994235014581188	hqer__induced sampler prototype	4.0
	front41Data	HQER-SFA	hqer__induced	True	34	-13.979191228306728	hqer__induced sampler prototype	4.0
	front41Data	Quantile-SFA	hqer__induced	True	29	-13.518959477281005	hqer__induced sampler prototype	4.0
	front41Data	HQER-SFA	hqer__induced	True	31	-13.435442050314045	hqer__induced sampler prototype	4.0
	front41Data	Quantile-SFA	hqer__induced	True	32	32.03242977865915	hqer__induced sampler prototype	2.0
	front41Data	HQER-SFA	hqer__induced	True	32	32.11395144523701	hqer__induced sampler prototype	2.0
	front41Data	Quantile-SFA	hqer__induced	True	17	-7.743668987615911	hqer__induced sampler prototype	2.0
	front41Data	HQER-SFA	hqer__induced	True	34	-7.635440568670871	hqer__induced sampler prototype	4.0
	front41Data	Quantile-SFA	hqer__induced	True	34	9.689937235631866	hqer__induced sampler prototype	4.0
	front41Data	HQER-SFA	hqer__induced	True	36	9.842388557297587	hqer__induced sampler prototype	4.0
	front41Data	Quantile-SFA	hqer__induced	True	33	12.361449030527131	hqer__induced sampler prototype	2.0
	front41Data	HQER-SFA	hqer__induced	True	31	12.522922646726425	hqer__induced sampler prototype	4.0
	front41Data	Quantile-SFA	hqer__induced	True	32	35.54354098201034	hqer__induced sampler prototype	4.0
	front41Data	HQER-SFA	hqer__induced	True	20	35.64575539875872	hqer__induced sampler prototype	4.0
	front41Data	Quantile-SFA	hqer__induced	True	30	22.10202116259342	hqer__induced sampler prototype	4.0
	front41Data	HQER-SFA	hqer__induced	True	32	22.16961961365515	hqer__induced sampler prototype	4.0
	front41Data	Quantile-SFA	hqer__induced	True	40	-7.598318501030688	hqer__induced sampler prototype	2.0
	front41Data	HQER-SFA	hqer__induced	True	40	-7.479208930493641	hqer__induced sampler prototype	4.0
	front41Data	Quantile-SFA	hqer__induced	True	46	9.08676413765419	hqer__induced sampler prototype	4.0
	front41Data	HQER-SFA	hqer__induced	True	27	9.36843079326754	hqer__induced sampler prototype	2.0
	front41Data	Quantile-SFA	hqer__induced	True	30	11.122271491625511	hqer__induced sampler prototype	2.0
	front41Data	HQER-SFA	hqer__induced	True	34	11.211386434915234	hqer__induced sampler prototype	2.0
	front41Data	Quantile-SFA	hqer__induced	True	20	-44.78333350575734	hqer__induced sampler prototype	4.0
	front41Data	HQER-SFA	hqer__induced	True	32	-44.713767749600336	hqer__induced sampler prototype	2.0
	front41Data	Quantile-SFA	hqer__induced	True	38	-14.526019520287502	hqer__induced sampler prototype	2.0
	front41Data	HQER-SFA	hqer__induced	True	20	-14.426663763541056	hqer__induced sampler prototype	4.0
	front41Data	Quantile-SFA	hqer__induced	True	37	-12.333331310293593	hqer__induced sampler prototype	4.0
	front41Data	HQER-SFA	hqer__induced	True	25	-12.180408994752206	hqer__induced sampler prototype	4.0
	front41Data	Quantile-SFA	hqer__induced	True	31	-6.607654272333045	hqer__induced sampler prototype	2.0
	front41Data	HQER-SFA	hqer__induced	True	38	-6.487771867506556	hqer__induced sampler prototype	2.0
	front41Data	Quantile-SFA	hqer__induced	True	29	17.935559099215105	hqer__induced sampler prototype	2.0
	front41Data	HQER-SFA	hqer__induced	True	22	18.015340614475768	hqer__induced sampler prototype	4.0
	front41Data	Quantile-SFA	hqer__induced	True	35	-2.792690825457428	hqer__induced sampler prototype	4.0
	front41Data	HQER-SFA	hqer__induced	True	32	-2.6881736359453647	hqer__induced sampler prototype	4.0
	front41Data	Quantile-SFA	hqer__induced	True	33	6.810633418485248	hqer__induced sampler prototype	4.0
	front41Data	HQER-SFA	hqer__induced	True	46	6.892961910864438	hqer__induced sampler prototype	2.0
	front41Data	Quantile-SFA	hqer__induced	True	42	0.12383720095350440	hqer__induced sampler prototype	2.0
	front41Data	HQER-SFA	hqer__induced	True	36	0.3469676002617019	hqer__induced sampler prototype	2.0
	front41Data	Quantile-SFA	hqer__induced	True	21	6.249952493872498	hqer__induced sampler prototype	2.0
	front41Data	HQER-SFA	hqer__induced	True	54	6.37006839937033	hqer__induced sampler prototype	2.0
	front41Data	Quantile-SFA	hqer__induced	True	43	-1.6626860517725026	hqer__induced sampler prototype	4.0
	front41Data	HQER-SFA	hqer__induced	True	37	-1.5738334756604535	hqer__induced sampler prototype	4.0
	front41Data	Quantile-SFA	hqer__induced	True	23	-0.4526778336448118	hqer__induced sampler prototype	2.0
	front41Data	HQER-SFA	hqer__induced	True	27	-0.3653096844496621	hqer__induced sampler prototype	2.0
	front41Data	Quantile-SFA	hqer__induced	True	33	-33.904204958274185	hqer__induced sampler prototype	4.0
	front41Data	HQER-SFA	hqer__induced	True	25	-33.827802867539816	hqer__induced sampler prototype	2.0
	front41Data	Quantile-SFA	hqer__induced	True	24	26.986674473185783	hqer__induced sampler prototype	4.0
	front41Data	HQER-SFA	hqer__induced	True	28	27.10162358926916	hqer__induced sampler prototype	2.0
	front41Data	Quantile-SFA	hqer__induced	True	24	-27.67851491064452	hqer__induced sampler prototype	2.0
	front41Data	HQER-SFA	hqer__induced	True	27	-27.5846074248152	hqer__induced sampler prototype	4.0
	front41Data	Quantile-SFA	hqer__induced	True	35	36.04022371312236	hqer__induced sampler prototype	2.0
	front41Data	HQER-SFA	hqer__induced	True	31	36.120788375746415	hqer__induced sampler prototype	4.0
	front41Data	Quantile-SFA	hqer__induced	True	34	14.509651086240698	hqer__induced sampler prototype	4.0
	front41Data	HQER-SFA	hqer__induced	True	35	14.597419527209604	hqer__induced sampler prototype	4.0
	front41Data	Quantile-SFA	hqer__induced	True	42	5.973197899421718	hqer__induced sampler prototype	2.0
	front41Data	HQER-SFA	hqer__induced	True	37	6.074555908549455	hqer__induced sampler prototype	2.0
	front41Data	Quantile-SFA	hqer__induced	True	25	-10.644978494273670	hqer__induced sampler prototype	2.0
	front41Data	HQER-SFA	hqer__induced	True	27	-10.55513073554032	hqer__induced sampler prototype	2.0
	front41Data	Quantile-SFA	hqer__induced	True	38	11.044151148658376	hqer__induced sampler prototype	2.0
	front41Data	HQER-SFA	hqer__induced	True	39	11.117551876493645	hqer__induced sampler prototype	2.0
	front41Data	Quantile-SFA	hqer__induced	True	44	-14.43029244530976	hqer__induced sampler prototype	2.0
	front41Data	HQER-SFA	hqer__induced	True	29	-14.304759768397195	hqer__induced sampler prototype	4.0
	front41Data	Quantile-SFA	hqer__induced	True	35	2.610180760935478	hqer__induced sampler prototype	4.0
	front41Data	HQER-SFA	hqer__induced	True	28	2.6866265700429253	hqer__induced sampler prototype	4.0
	front41Data	Quantile-SFA	hqer__induced	True	31	-35.87868752998503	hqer__induced sampler prototype	4.0

experiment	dataset	model	scenario	converged	n_iter	loglik	warning_message	best_start_id
	front41Data	HQER-SFA	hqer_induced	True	12	-35.81814673799367	hqer_induced sampler prototype	2.0
	front41Data	Quantile-SFA	hqer_induced	True	28	31.29651050508972	hqer_induced sampler prototype	4.0
	front41Data	HQER-SFA	hqer_induced	True	26	31.173684616629835	hqer_induced sampler prototype	4.0
	front41Data	Quantile-SFA	hqer_induced	True	24	-13.7212643646089	hqer_induced sampler prototype	2.0
	front41Data	HQER-SFA	hqer_induced	True	40	-13.600718914197271	hqer_induced sampler prototype	2.0
	front41Data	Quantile-SFA	hqer_induced	True	21	8.301868755572283	hqer_induced sampler prototype	4.0
	front41Data	HQER-SFA	hqer_induced	True	30	8.422195771810081	hqer_induced sampler prototype	4.0
	front41Data	Quantile-SFA	hqer_induced	True	25	-4.697551402839658	hqer_induced sampler prototype	2.0
	front41Data	HQER-SFA	hqer_induced	True	28	-4.61929865104134	hqer_induced sampler prototype	2.0
	front41Data	Quantile-SFA	hqer_induced	True	42	1.19509851355836	hqer_induced sampler prototype	4.0
	front41Data	HQER-SFA	hqer_induced	True	75	1.3014312648879347	hqer_induced sampler prototype	2.0
	front41Data	Quantile-SFA	hqer_induced	True	31	3.279242514516685	hqer_induced sampler prototype	2.0
	front41Data	HQER-SFA	hqer_induced	True	41	3.3479912700547025	hqer_induced sampler prototype	2.0
	front41Data	Quantile-SFA	hqer_induced	True	31	42.18131229984567	hqer_induced sampler prototype	2.0
	front41Data	HQER-SFA	hqer_induced	True	29	42.25003363643013	hqer_induced sampler prototype	2.0
	front41Data	Quantile-SFA	hqer_induced	True	39	-10.516043239666857	hqer_induced sampler prototype	2.0
	front41Data	HQER-SFA	hqer_induced	True	32	-10.441896143108504	hqer_induced sampler prototype	4.0
	front41Data	Quantile-SFA	hqer_induced	True	44	-14.759819498773894	hqer_induced sampler prototype	4.0
	front41Data	HQER-SFA	hqer_induced	True	29	-14.661020487715774	hqer_induced sampler prototype	4.0
	front41Data	Quantile-SFA	hqer_induced	True	29	-2.021004210347864	hqer_induced sampler prototype	2.0
	front41Data	HQER-SFA	hqer_induced	True	44	-1.9029867117179835	hqer_induced sampler prototype	2.0
	front41Data	Quantile-SFA	hqer_induced	True	41	1.700709484679848	hqer_induced sampler prototype	2.0
	front41Data	HQER-SFA	hqer_induced	True	35	1.7697731691298273	hqer_induced sampler prototype	4.0
	front41Data	Quantile-SFA	hqer_induced	True	27	-10.857703616693948	hqer_induced sampler prototype	4.0
	front41Data	HQER-SFA	hqer_induced	True	28	-10.769489628585545	hqer_induced sampler prototype	4.0
	front41Data	Quantile-SFA	hqer_induced	True	30	0.8074981520542917	hqer_induced sampler prototype	4.0
	front41Data	HQER-SFA	hqer_induced	True	38	0.8573703291313439	hqer_induced sampler prototype	4.0
	front41Data	Quantile-SFA	hqer_induced	True	30	14.629757280057436	hqer_induced sampler prototype	4.0
	front41Data	HQER-SFA	hqer_induced	True	42	14.737470321115795	hqer_induced sampler prototype	4.0
	front41Data	Quantile-SFA	hqer_induced	True	35	19.233954438721703	hqer_induced sampler prototype	4.0
	front41Data	HQER-SFA	hqer_induced	True	21	19.34289448354985	hqer_induced sampler prototype	4.0
	front41Data	Quantile-SFA	hqer_induced	True	22	31.702301972135	hqer_induced sampler prototype	2.0
	front41Data	HQER-SFA	hqer_induced	True	26	31.79231527445588	hqer_induced sampler prototype	4.0
	front41Data	Quantile-SFA	hqer_induced	True	28	4.008227134620615	hqer_induced sampler prototype	4.0
	front41Data	HQER-SFA	hqer_induced	True	24	4.101002879119876	hqer_induced sampler prototype	4.0
	front41Data	Quantile-SFA	hqer_induced	True	36	17.088067712703264	hqer_induced sampler prototype	4.0
	front41Data	HQER-SFA	hqer_induced	True	27	17.173351941481	hqer_induced sampler prototype	4.0
	front41Data	Quantile-SFA	hqer_induced	True	26	-21.7504741811976	hqer_induced sampler prototype	2.0
	front41Data	HQER-SFA	hqer_induced	True	22	-21.669687053052243	hqer_induced sampler prototype	4.0
	front41Data	Quantile-SFA	hqer_induced	True	23	42.12465386537957	hqer_induced sampler prototype	4.0
	front41Data	HQER-SFA	hqer_induced	True	32	42.21630843304224	hqer_induced sampler prototype	4.0
	front41Data	Quantile-SFA	hqer_induced	True	30	54.65939934802523	hqer_induced sampler prototype	2.0
	front41Data	HQER-SFA	hqer_induced	True	36	54.728183653043416	hqer_induced sampler prototype	2.0
	front41Data	Quantile-SFA	hqer_induced	True	29	-20.622940161874705	hqer_induced sampler prototype	2.0
	front41Data	HQER-SFA	hqer_induced	True	31	-20.53063793140759	hqer_induced sampler prototype	2.0
	front41Data	Quantile-SFA	hqer_induced	True	25	-13.69984618367148	hqer_induced sampler prototype	4.0
	front41Data	HQER-SFA	hqer_induced	True	26	-13.60722772978102	hqer_induced sampler prototype	4.0
	front41Data	Quantile-SFA	hqer_induced	True	21	-10.659410805655826	hqer_induced sampler prototype	2.0
	front41Data	HQER-SFA	hqer_induced	True	25	-10.537732116147406	hqer_induced sampler prototype	2.0
	front41Data	Quantile-SFA	hqer_induced	True	16	-25.794330870794123	hqer_induced sampler prototype	4.0
	front41Data	HQER-SFA	hqer_induced	True	26	-25.692901870929173	hqer_induced sampler prototype	4.0
	front41Data	Quantile-SFA	hqer_induced	True	18	23.7445655840662	hqer_induced sampler prototype	4.0
	front41Data	HQER-SFA	hqer_induced	True	19	23.727622838114364	hqer_induced sampler prototype	4.0
	front41Data	Quantile-SFA	hqer_induced	True	27	-15.244169559959392	hqer_induced sampler prototype	4.0
	front41Data	HQER-SFA	hqer_induced	True	29	-15.171121574845753	hqer_induced sampler prototype	4.0
	front41Data	Quantile-SFA	hqer_induced	True	32	-4.000460416827949	hqer_induced sampler prototype	4.0
	front41Data	HQER-SFA	hqer_induced	True	60	-3.8846473173542506	hqer_induced sampler prototype	2.0
	front41Data	Quantile-SFA	hqer_induced	True	23	-3.588492636206457	hqer_induced sampler prototype	4.0
	front41Data	HQER-SFA	hqer_induced	True	19	-3.4859081197204382	hqer_induced sampler prototype	4.0
	front41Data	Quantile-SFA	hqer_induced	True	25	20.547774806890807	hqer_induced sampler prototype	4.0
	front41Data	HQER-SFA	hqer_induced	True	27	20.719313536072026	hqer_induced sampler prototype	4.0
	front41Data	Quantile-SFA	hqer_induced	True	29	23.68763668544817	hqer_induced sampler prototype	4.0
	front41Data	HQER-SFA	hqer_induced	True	24	23.79114564208444	hqer_induced sampler prototype	4.0
	front41Data	Quantile-SFA	hqer_induced	True	26	23.226600172881547	hqer_induced sampler prototype	4.0
	front41Data	HQER-SFA	hqer_induced	True	21	23.448313798156065	hqer_induced sampler prototype	4.0
	front41Data	Quantile-SFA	hqer_induced	True	33	-12.525164113409302	hqer_induced sampler prototype	4.0
	front41Data	HQER-SFA	hqer_induced	True	28	-12.418528976639823	hqer_induced sampler prototype	4.0
	front41Data	Quantile-SFA	hqer_induced	True	25	38.048729876448846	hqer_induced sampler prototype	2.0
	front41Data	HQER-SFA	hqer_induced	True	36	38.35130916603085	hqer_induced sampler prototype	2.0
	front41Data	Quantile-SFA	hqer_induced	True	22	31.833486910148	hqer_induced sampler prototype	2.0
	front41Data	HQER-SFA	hqer_induced	True	42	31.890987974765963	hqer_induced sampler prototype	2.0
	front41Data	Quantile-SFA	hqer_induced	True	29	-1.971567915058671	hqer_induced sampler prototype	4.0
	front41Data	HQER-SFA	hqer_induced	True	26	-1.8758529451628831	hqer_induced sampler prototype	4.0
	front41Data	Quantile-SFA	hqer_induced	True	31	30.83589656129359	hqer_induced sampler prototype	4.0
	front41Data	HQER-SFA	hqer_induced	True	16	30.82738951683214	hqer_induced sampler prototype	2.0
	front41Data	Quantile-SFA	hqer_induced	True	30	4.808114747436221	hqer_induced sampler prototype	4.0
	front41Data	HQER-SFA	hqer_induced	True	49	4.914084853662144	hqer_induced sampler prototype	2.0
	front41Data	Quantile-SFA	hqer_induced	True	33	20.883962511894417	hqer_induced sampler prototype	4.0
	front41Data	HQER-SFA	hqer_induced	True	18	20.952774991316726	hqer_induced sampler prototype	2.0
	front41Data	Quantile-SFA	hqer_induced	True	23	-8.261836419521055	hqer_induced sampler prototype	2.0
	front41Data	HQER-SFA	hqer_induced	True	31	-8.15552161009504	hqer_induced sampler prototype	2.0
	front41Data	Quantile-SFA	hqer_induced	True	38	17.482389887429772	hqer_induced sampler prototype	2.0
	front41Data	HQER-SFA	hqer_induced	True	49	17.526432275395678	hqer_induced sampler prototype	2.0
	front41Data	Quantile-SFA	hqer_induced	True	27	21.23689542195143	hqer_induced sampler prototype	4.0
	front41Data	HQER-SFA	hqer_induced	True	30	21.349676783813912	hqer_induced sampler prototype	4.0
	front41Data	Quantile-SFA	hqer_induced	True	43	3.8319978517389472	hqer_induced sampler prototype	2.0
	front41Data	HQER-SFA	hqer_induced	True	17	3.8460770330345415	hqer_induced sampler prototype	4.0
	front41Data	Quantile-SFA	hqer_induced	True	45	2.001231052572388	hqer_induced sampler prototype	2.0
	front41Data	HQER-SFA	hqer_induced	True	30	2.1002271307419305	hqer_induced sampler prototype	2.0
	front41Data	Quantile-SFA	hqer_induced	True	37	-1.3793143928151856	hqer_induced sampler prototype	4.0
	front41Data	HQER-SFA	hqer_induced	True	30	-1.310747953076148	hqer_induced sampler prototype	2.0
	front41Data	Quantile-SFA	hqer_induced	True	23	12.672334849965193	hqer_induced sampler prototype	4.0
	front41Data	HQER-SFA	hqer_induced	True	39	12.727602760716682	hqer_induced sampler prototype	2.0
	front41Data	Quantile-SFA	hqer_induced	True	26	-32.51071512235621	hqer_induced sampler prototype	2.0
	front41Data	HQER-SFA	hqer_induced	True	21	-32.3948712260192	hqer_induced sampler prototype	2.0
	front41Data	Quantile-SFA	hqer_induced	True	49	-13.235831476115628	hqer_induced sampler prototype	4.0
	front41Data	HQER-SFA	hqer_induced	True	18	-13.17999430026871	hqer_induced sampler prototype	4.0
	front41Data	Quantile-SFA	hqer_induced	True	32	-8.150303545314085	hqer_induced sampler prototype	2.0
	front41Data	HQER-SFA	hqer_induced	True	42	-8.062149369441151	hqer_induced sampler prototype	2.0
	front41Data	Quantile-SFA	hqer_induced	True	64	27.023790145031164	hqer_induced sampler prototype	4.0
	front41Data	HQER-SFA	hqer_induced	True	31	27.07458258989024	hqer_induced sampler prototype	4.0
	front41Data	Quantile-SFA	hqer_induced	True	23	-14.4265853318663	hqer_induced sampler prototype	4.0
	front41Data	HQER-SFA	hqer_induced	True	20	-14.320633835423411	hqer_induced sampler prototype	2.0

experiment	dataset	model	scenario	converged	n__iter	loglik	warning__message	best__start__id
	front41Data	Quantile-SFA	hqer__induced	True	31	-25.70698066717944	hqer__induced sampler prototype	2.0
	front41Data	HQER-SFA	hqer__induced	True	26	-25.59187039235937	hqer__induced sampler prototype	2.0
	front41Data	Quantile-SFA	hqer__induced	True	32	-9.606693713889154	hqer__induced sampler prototype	4.0
	front41Data	HQER-SFA	hqer__induced	True	39	-9.64847764413926	hqer__induced sampler prototype	2.0
	front41Data	Quantile-SFA	hqer__induced	True	19	15.700858668980311	hqer__induced sampler prototype	4.0
	front41Data	HQER-SFA	hqer__induced	True	27	15.788648457806577	hqer__induced sampler prototype	2.0
	front41Data	Quantile-SFA	hqer__induced	True	27	-20.917066401706805	hqer__induced sampler prototype	4.0
	front41Data	HQER-SFA	hqer__induced	True	32	-20.83012129857604	hqer__induced sampler prototype	2.0
	front41Data	Quantile-SFA	hqer__induced	True	26	12.24585178813448	hqer__induced sampler prototype	4.0
	front41Data	HQER-SFA	hqer__induced	True	29	12.348854132908246	hqer__induced sampler prototype	4.0
	front41Data	Quantile-SFA	hqer__induced	True	27	-24.7664625525636	hqer__induced sampler prototype	4.0
	front41Data	HQER-SFA	hqer__induced	True	30	-24.664816859313284	hqer__induced sampler prototype	4.0
	front41Data	Quantile-SFA	hqer__induced	True	15	18.93639107768169	hqer__induced sampler prototype	2.0
	front41Data	HQER-SFA	hqer__induced	True	18	19.07695365864896	hqer__induced sampler prototype	2.0
	front41Data	Quantile-SFA	hqer__induced	True	26	9.291217250722482	hqer__induced sampler prototype	4.0
	front41Data	HQER-SFA	hqer__induced	True	35	9.326546689135956	hqer__induced sampler prototype	4.0
	front41Data	Quantile-SFA	hqer__induced	True	35	3.558450211316078	hqer__induced sampler prototype	2.0
	front41Data	HQER-SFA	hqer__induced	True	29	3.6489838472729295	hqer__induced sampler prototype	2.0
	front41Data	Quantile-SFA	hqer__induced	True	54	-33.4065597406716	hqer__induced sampler prototype	2.0
	front41Data	HQER-SFA	hqer__induced	True	34	-33.3928562725095	hqer__induced sampler prototype	2.0
	front41Data	Quantile-SFA	hqer__induced	True	15	-8.463670535958006	hqer__induced sampler prototype	2.0
	front41Data	HQER-SFA	hqer__induced	True	32	-8.291788597141435	hqer__induced sampler prototype	2.0
	front41Data	Quantile-SFA	hqer__induced	True	42	31.562688886059554	hqer__induced sampler prototype	4.0
	front41Data	HQER-SFA	hqer__induced	True	33	31.563616411139343	hqer__induced sampler prototype	4.0
	front41Data	Quantile-SFA	hqer__induced	True	19	1.574541622885267	hqer__induced sampler prototype	2.0
	front41Data	HQER-SFA	hqer__induced	True	33	1.703534848895245	hqer__induced sampler prototype	4.0
	front41Data	Quantile-SFA	hqer__induced	True	19	1.900597522651834	hqer__induced sampler prototype	4.0
	front41Data	HQER-SFA	hqer__induced	True	18	1.9771167622752124	hqer__induced sampler prototype	4.0
	front41Data	Quantile-SFA	hqer__induced	True	19	0.5450942909599683	hqer__induced sampler prototype	2.0
	front41Data	HQER-SFA	hqer__induced	True	24	0.7293958692840432	hqer__induced sampler prototype	2.0
	front41Data	Quantile-SFA	hqer__induced	True	12	-25.50114632040888	hqer__induced sampler prototype	4.0
	front41Data	HQER-SFA	hqer__induced	True	24	-25.41751454111691	hqer__induced sampler prototype	2.0
	front41Data	Quantile-SFA	hqer__induced	True	36	-11.25185871207458	hqer__induced sampler prototype	2.0
	front41Data	HQER-SFA	hqer__induced	True	42	-11.168973511460525	hqer__induced sampler prototype	4.0
	front41Data	Quantile-SFA	hqer__induced	True	28	-15.930040715984966	hqer__induced sampler prototype	2.0
	front41Data	HQER-SFA	hqer__induced	True	26	-15.815775693153636	hqer__induced sampler prototype	2.0
	front41Data	Quantile-SFA	hqer__induced	True	28	-1.904724109285704	hqer__induced sampler prototype	2.0
	front41Data	HQER-SFA	hqer__induced	True	24	-1.8759260756705043	hqer__induced sampler prototype	2.0
	front41Data	Quantile-SFA	hqer__induced	True	28	16.249117412137366	hqer__induced sampler prototype	4.0
	front41Data	HQER-SFA	hqer__induced	True	22	16.31368459724743	hqer__induced sampler prototype	4.0
	front41Data	Quantile-SFA	hqer__induced	True	36	23.324829674588976	hqer__induced sampler prototype	2.0
	front41Data	HQER-SFA	hqer__induced	True	14	23.2513579512388	hqer__induced sampler prototype	4.0
	front41Data	Quantile-SFA	hqer__induced	True	37	20.90556708799065	hqer__induced sampler prototype	4.0
	front41Data	HQER-SFA	hqer__induced	True	29	21.00719455752208	hqer__induced sampler prototype	4.0
	front41Data	Quantile-SFA	hqer__induced	True	39	22.07310315834114	hqer__induced sampler prototype	2.0
	front41Data	HQER-SFA	hqer__induced	True	18	22.170266490493205	hqer__induced sampler prototype	4.0
	front41Data	Quantile-SFA	hqer__induced	True	35	16.478446419611277	hqer__induced sampler prototype	2.0
	front41Data	HQER-SFA	hqer__induced	True	19	16.558762107011482	hqer__induced sampler prototype	4.0
	front41Data	Quantile-SFA	hqer__induced	True	26	-18.84797255223024	hqer__induced sampler prototype	4.0
	front41Data	HQER-SFA	hqer__induced	True	26	-18.735842830933	hqer__induced sampler prototype	4.0
	front41Data	Quantile-SFA	hqer__induced	True	47	31.757854816218348	hqer__induced sampler prototype	4.0
	front41Data	HQER-SFA	hqer__induced	True	41	31.86571096381805	hqer__induced sampler prototype	2.0
	front41Data	Quantile-SFA	hqer__induced	True	31	-0.8414723871323568	hqer__induced sampler prototype	2.0
	front41Data	HQER-SFA	hqer__induced	True	37	-0.7910199862647431	hqer__induced sampler prototype	2.0
	front41Data	Quantile-SFA	hqer__induced	True	32	21.64389030448722	hqer__induced sampler prototype	2.0
	front41Data	HQER-SFA	hqer__induced	True	25	21.774792381037035	hqer__induced sampler prototype	4.0
	front41Data	Quantile-SFA	hqer__induced	True	36	4.807359258911639	hqer__induced sampler prototype	4.0
	front41Data	HQER-SFA	hqer__induced	True	35	4.902855585133574	hqer__induced sampler prototype	2.0
	front41Data	Quantile-SFA	hqer__induced	True	22	19.669272182477187	hqer__induced sampler prototype	4.0
	front41Data	HQER-SFA	hqer__induced	True	24	19.77598048197472	hqer__induced sampler prototype	4.0
	front41Data	Quantile-SFA	hqer__induced	True	50	-5.602487941527516	hqer__induced sampler prototype	2.0
	front41Data	HQER-SFA	hqer__induced	True	35	-5.499173460051398	hqer__induced sampler prototype	2.0
	front41Data	Quantile-SFA	hqer__induced	True	43	1.5623182956339612	hqer__induced sampler prototype	2.0
	front41Data	HQER-SFA	hqer__induced	True	33	1.5933718203975893	hqer__induced sampler prototype	2.0
	front41Data	Quantile-SFA	hqer__induced	True	13	35.13581349579536	hqer__induced sampler prototype	2.0
	front41Data	HQER-SFA	hqer__induced	True	20	35.26392753035644	hqer__induced sampler prototype	2.0
	front41Data	Quantile-SFA	hqer__induced	True	50	6.910791931943278	hqer__induced sampler prototype	2.0
	front41Data	HQER-SFA	hqer__induced	True	18	6.778446365385525	hqer__induced sampler prototype	2.0
	front41Data	Quantile-SFA	hqer__induced	True	19	-10.039138054207193	hqer__induced sampler prototype	4.0
	front41Data	HQER-SFA	hqer__induced	True	29	-9.774815757504118	hqer__induced sampler prototype	4.0
	front41Data	Quantile-SFA	hqer__induced	True	25	-6.544490341548119	hqer__induced sampler prototype	2.0
	front41Data	HQER-SFA	hqer__induced	True	29	-6.45366702396943	hqer__induced sampler prototype	2.0
	front41Data	Quantile-SFA	hqer__induced	True	56	9.624852856853664	hqer__induced sampler prototype	4.0
	front41Data	HQER-SFA	hqer__induced	True	25	9.359699049339042	hqer__induced sampler prototype	2.0
	front41Data	Quantile-SFA	hqer__induced	True	18	-34.32469148473249	hqer__induced sampler prototype	4.0
	front41Data	HQER-SFA	hqer__induced	True	20	-34.23994554203054	hqer__induced sampler prototype	4.0
	front41Data	Quantile-SFA	hqer__induced	True	40	-11.434313291163065	hqer__induced sampler prototype	2.0
	front41Data	HQER-SFA	hqer__induced	True	33	-11.09094628037143	hqer__induced sampler prototype	4.0
	front41Data	Quantile-SFA	hqer__induced	True	31	-0.7272864596144348	hqer__induced sampler prototype	4.0
	front41Data	HQER-SFA	hqer__induced	True	61	-0.5843890653835437	hqer__induced sampler prototype	4.0
	front41Data	Quantile-SFA	hqer__induced	True	31	-6.580145657917475	hqer__induced sampler prototype	4.0
	front41Data	HQER-SFA	hqer__induced	True	22	-6.409756710627598	hqer__induced sampler prototype	4.0
	front41Data	Quantile-SFA	hqer__induced	True	19	-7.481686858519834	hqer__induced sampler prototype	4.0
	front41Data	HQER-SFA	hqer__induced	True	17	-7.384325614058635	hqer__induced sampler prototype	4.0
	front41Data	Quantile-SFA	hqer__induced	True	28	-43.38775212585012	hqer__induced sampler prototype	4.0
	front41Data	HQER-SFA	hqer__induced	True	31	-43.27438056374257	hqer__induced sampler prototype	2.0
	front41Data	Quantile-SFA	hqer__induced	True	25	-32.104120424563305	hqer__induced sampler prototype	4.0
	front41Data	HQER-SFA	hqer__induced	True	27	-31.9787269412186	hqer__induced sampler prototype	4.0
	front41Data	Quantile-SFA	hqer__induced	True	29	41.454765909841576	hqer__induced sampler prototype	2.0
	front41Data	HQER-SFA	hqer__induced	True	35	41.52430506714582	hqer__induced sampler prototype	2.0
	front41Data	Quantile-SFA	hqer__induced	True	23	-10.35269817035425	hqer__induced sampler prototype	4.0
	front41Data	HQER-SFA	hqer__induced	True	25	-10.35262610372908	hqer__induced sampler prototype	4.0
	front41Data	Quantile-SFA	hqer__induced	True	52	-11.312133572140803	hqer__induced sampler prototype	2.0
	front41Data	HQER-SFA	hqer__induced	True	26	-11.187089526370723	hqer__induced sampler prototype	4.0
	front41Data	Quantile-SFA	hqer__induced	True	27	-11.730586820516471	hqer__induced sampler prototype	2.0
	front41Data	HQER-SFA	hqer__induced	True	37	-11.660522894739282	hqer__induced sampler prototype	2.0
	front41Data	Quantile-SFA	hqer__induced	True	42	2.1334149375727343	hqer__induced sampler prototype	4.0
	front41Data	HQER-SFA	hqer__induced	True	28	2.236463913729173	hqer__induced sampler prototype	4.0
	front41Data	Quantile-SFA	hqer__induced	True	23	21.95128953719308	hqer__induced sampler prototype	4.0
	front41Data	HQER-SFA	hqer__induced	True	35	22.07921691704487	hqer__induced sampler prototype	4.0
	front41Data	Quantile-SFA	hqer__induced	True	34	28.298198796713816	hqer__induced sampler prototype	4.0
	front41Data	HQER-SFA	hqer__induced	True	21	28.41590901065835	hqer__induced sampler prototype	4.0
	front41Data	Quantile-SFA	hqer__induced	True	27	-3.3701071609600657	hqer__induced sampler prototype	4.0

experiment	dataset	model	scenario	converged	n__iter	loglik	warning__message	best__start__id
	front41Data	HQER-SFA	hqer__induced	True	16	-3.268977953730154	hqer__induced sampler prototype	4.0
	front41Data	Quantile-SFA	hqer__induced	True	23	59.9024441513143	hqer__induced sampler prototype	2.0
	front41Data	HQER-SFA	hqer__induced	True	27	60.031301069465	hqer__induced sampler prototype	2.0
	front41Data	Quantile-SFA	hqer__induced	True	15	20.671351263838503	hqer__induced sampler prototype	2.0
	front41Data	HQER-SFA	hqer__induced	True	16	20.81051524898061	hqer__induced sampler prototype	4.0
	front41Data	Quantile-SFA	hqer__induced	True	30	26.48253595518532	hqer__induced sampler prototype	2.0
	front41Data	HQER-SFA	hqer__induced	True	29	26.586400678869857	hqer__induced sampler prototype	2.0
	front41Data	Quantile-SFA	hqer__induced	True	40	17.313581686373336	hqer__induced sampler prototype	4.0
	front41Data	HQER-SFA	hqer__induced	True	36	17.378440595805838	hqer__induced sampler prototype	4.0
	front41Data	Quantile-SFA	hqer__induced	True	24	6.858809241417125	hqer__induced sampler prototype	2.0
	front41Data	HQER-SFA	hqer__induced	True	22	7.01612571708792	hqer__induced sampler prototype	2.0
	front41Data	Quantile-SFA	hqer__induced	True	46	-8.811462196682474	hqer__induced sampler prototype	2.0
	front41Data	HQER-SFA	hqer__induced	True	59	-8.692042493495888	hqer__induced sampler prototype	2.0
	front41Data	Quantile-SFA	hqer__induced	True	27	26.92754345318022	hqer__induced sampler prototype	2.0
	front41Data	HQER-SFA	hqer__induced	True	30	27.003178670285823	hqer__induced sampler prototype	4.0
	front41Data	Quantile-SFA	hqer__induced	True	34	13.1609969627429	hqer__induced sampler prototype	4.0
	front41Data	HQER-SFA	hqer__induced	True	32	13.220426407665624	hqer__induced sampler prototype	4.0
	front41Data	Quantile-SFA	hqer__induced	True	36	-4.963779366083186	hqer__induced sampler prototype	2.0
	front41Data	HQER-SFA	hqer__induced	True	33	-4.8846849273807935	hqer__induced sampler prototype	2.0
	front41Data	Quantile-SFA	hqer__induced	True	36	-15.742791705994025	hqer__induced sampler prototype	2.0
	front41Data	HQER-SFA	hqer__induced	True	26	-15.640577062657467	hqer__induced sampler prototype	2.0
	front41Data	Quantile-SFA	hqer__induced	True	29	6.611418393502401	hqer__induced sampler prototype	2.0
	front41Data	HQER-SFA	hqer__induced	True	23	6.685931585635499	hqer__induced sampler prototype	2.0
	front41Data	Quantile-SFA	hqer__induced	True	27	5.117506330417973	hqer__induced sampler prototype	4.0
	front41Data	HQER-SFA	hqer__induced	True	45	5.291680007103925	hqer__induced sampler prototype	2.0
	front41Data	Quantile-SFA	hqer__induced	True	46	-23.40397972892498	hqer__induced sampler prototype	4.0
	front41Data	HQER-SFA	hqer__induced	True	31	-23.325505892135205	hqer__induced sampler prototype	4.0
	front41Data	Quantile-SFA	hqer__induced	True	23	23.01185758525675	hqer__induced sampler prototype	4.0
	front41Data	HQER-SFA	hqer__induced	True	22	23.14668132836798	hqer__induced sampler prototype	2.0
	front41Data	Quantile-SFA	hqer__induced	True	54	7.632076173349453	hqer__induced sampler prototype	4.0
	front41Data	HQER-SFA	hqer__induced	True	36	7.730526779699407	hqer__induced sampler prototype	2.0
	front41Data	Quantile-SFA	hqer__induced	True	15	50.57950941316606	hqer__induced sampler prototype	4.0
	front41Data	HQER-SFA	hqer__induced	True	38	50.75227849765445	hqer__induced sampler prototype	2.0
	front41Data	Quantile-SFA	hqer__induced	True	26	-6.826565204289487	hqer__induced sampler prototype	4.0
	front41Data	HQER-SFA	hqer__induced	True	25	-6.705843211906863	hqer__induced sampler prototype	4.0
	front41Data	Quantile-SFA	hqer__induced	True	31	-5.261703287902421	hqer__induced sampler prototype	4.0
	front41Data	HQER-SFA	hqer__induced	True	49	-5.1453998990928	hqer__induced sampler prototype	4.0
	front41Data	Quantile-SFA	hqer__induced	True	19	-3.581922874946738	hqer__induced sampler prototype	4.0
	front41Data	HQER-SFA	hqer__induced	True	22	-3.529989733059507	hqer__induced sampler prototype	4.0
	front41Data	Quantile-SFA	hqer__induced	True	30	7.952703444363966	hqer__induced sampler prototype	2.0
	front41Data	HQER-SFA	hqer__induced	True	30	8.014935823071772	hqer__induced sampler prototype	2.0
	front41Data	Quantile-SFA	hqer__induced	True	29	14.4225123728918	hqer__induced sampler prototype	2.0
	front41Data	HQER-SFA	hqer__induced	True	32	14.525649845872582	hqer__induced sampler prototype	4.0
	front41Data	Quantile-SFA	hqer__induced	True	52	-24.93433306795947	hqer__induced sampler prototype	4.0
	front41Data	HQER-SFA	hqer__induced	True	28	-24.849523215209185	hqer__induced sampler prototype	4.0
	front41Data	Quantile-SFA	hqer__induced	True	27	-29.971242935403964	hqer__induced sampler prototype	4.0
	front41Data	HQER-SFA	hqer__induced	True	36	-29.872815671741936	hqer__induced sampler prototype	4.0
	front41Data	Quantile-SFA	hqer__induced	True	38	5.1234698064856	hqer__induced sampler prototype	2.0
	front41Data	HQER-SFA	hqer__induced	True	63	7.921402539850762	hqer__induced sampler prototype	4.0
	front41Data	Quantile-SFA	hqer__induced	True	21	-3.209809318392151	hqer__induced sampler prototype	4.0
	front41Data	HQER-SFA	hqer__induced	True	33	-2.7169316204335265	hqer__induced sampler prototype	4.0
	front41Data	Quantile-SFA	hqer__induced	True	17	3.540952431271699	hqer__induced sampler prototype	4.0
	front41Data	HQER-SFA	hqer__induced	True	27	3.006996590180337	hqer__induced sampler prototype	4.0
	front41Data	Quantile-SFA	hqer__induced	True	40	-11.659101764963385	hqer__induced sampler prototype	4.0
	front41Data	HQER-SFA	hqer__induced	True	28	-10.927626052622337	hqer__induced sampler prototype	2.0
	front41Data	Quantile-SFA	hqer__induced	True	33	4.355032383958261	hqer__induced sampler prototype	2.0
	front41Data	HQER-SFA	hqer__induced	True	87	4.554246184757679	hqer__induced sampler prototype	2.0
	front41Data	Quantile-SFA	hqer__induced	True	10	-1.5616556264730712	hqer__induced sampler prototype	4.0
	front41Data	HQER-SFA	hqer__induced	True	46	4.025188448595741	hqer__induced sampler prototype	4.0
	front41Data	Quantile-SFA	hqer__induced	True	32	0.4639345134447515	hqer__induced sampler prototype	4.0
	front41Data	HQER-SFA	hqer__induced	True	31	-0.1796554480011637	hqer__induced sampler prototype	4.0
	front41Data	Quantile-SFA	hqer__induced	True	39	1.1347625850063925	hqer__induced sampler prototype	4.0
	front41Data	HQER-SFA	hqer__induced	True	95	2.483642122546144	hqer__induced sampler prototype	2.0
	front41Data	Quantile-SFA	hqer__induced	True	28	-9.025704515815727	hqer__induced sampler prototype	4.0
	front41Data	HQER-SFA	hqer__induced	True	14	-7.090390403572964	hqer__induced sampler prototype	4.0
	front41Data	Quantile-SFA	hqer__induced	True	39	-8.360850387226737	hqer__induced sampler prototype	4.0
	front41Data	HQER-SFA	hqer__induced	True	85	-8.158469164949864	hqer__induced sampler prototype	4.0
	front41Data	Quantile-SFA	hqer__induced	True	34	-7.454125828042482	hqer__induced sampler prototype	4.0
	front41Data	HQER-SFA	hqer__induced	True	23	-4.678577459510294	hqer__induced sampler prototype	2.0
	front41Data	Quantile-SFA	hqer__induced	True	34	-5.66587920303 78	hqer__induced sampler prototype	4.0
	front41Data	HQER-SFA	hqer__induced	True	22	-4.835191139801282	hqer__induced sampler prototype	2.0
	front41Data	Quantile-SFA	hqer__induced	True	22	-3.843250099102267	hqer__induced sampler prototype	4.0
	front41Data	HQER-SFA	hqer__induced	True	22	-2.314290822553727	hqer__induced sampler prototype	4.0
	front41Data	Quantile-SFA	hqer__induced	True	35	-2.247887729208884	hqer__induced sampler prototype	4.0
	front41Data	HQER-SFA	hqer__induced	True	44	-2.169489404110473	hqer__induced sampler prototype	4.0
	front41Data	Quantile-SFA	hqer__induced	True	18	18.338724157374617	hqer__induced sampler prototype	4.0
	front41Data	HQER-SFA	hqer__induced	True	12	18.61167557833879	hqer__induced sampler prototype	2.0
	front41Data	Quantile-SFA	hqer__induced	True	28	-1.7344453314657335	hqer__induced sampler prototype	4.0
	front41Data	HQER-SFA	hqer__induced	True	26	0.6900503542730858	hqer__induced sampler prototype	2.0
	front41Data	Quantile-SFA	hqer__induced	True	27	4.28314379486868	hqer__induced sampler prototype	2.0
	front41Data	HQER-SFA	hqer__induced	True	12	4.061152100498412	hqer__induced sampler prototype	2.0
	front41Data	Quantile-SFA	hqer__induced	True	65	8.764950859829094	hqer__induced sampler prototype	2.0
	front41Data	HQER-SFA	hqer__induced	True	31	10.948664583153464	hqer__induced sampler prototype	2.0
	front41Data	Quantile-SFA	hqer__induced	True	22	-3.5593087980818416	hqer__induced sampler prototype	4.0
	front41Data	HQER-SFA	hqer__induced	True	48	-2.624806021708172	hqer__induced sampler prototype	4.0
	front41Data	Quantile-SFA	hqer__induced	True	34	-1.9250622545573468	hqer__induced sampler prototype	2.0
	front41Data	HQER-SFA	hqer__induced	True	42	-1.6104436631742078	hqer__induced sampler prototype	4.0
	front41Data	Quantile-SFA	hqer__induced	True	20	3.051799810953311	hqer__induced sampler prototype	2.0
	front41Data	HQER-SFA	hqer__induced	True	38	2.424924957191424	hqer__induced sampler prototype	4.0
	front41Data	Quantile-SFA	hqer__induced	True	29	0.3990250732634477	hqer__induced sampler prototype	4.0
	front41Data	HQER-SFA	hqer__induced	True	38	0.0838444181577411	hqer__induced sampler prototype	4.0
	front41Data	Quantile-SFA	hqer__induced	True	13	-9.432826903056752	hqer__induced sampler prototype	4.0
	front41Data	HQER-SFA	hqer__induced	True	42	-5.584635222062434	hqer__induced sampler prototype	2.0
	front41Data	Quantile-SFA	hqer__induced	True	23	2.803647786708103	hqer__induced sampler prototype	4.0
	front41Data	HQER-SFA	hqer__induced	True	19	3.336064592562745	hqer__induced sampler prototype	4.0
	front41Data	Quantile-SFA	hqer__induced	True	40	-1.2059543636723231	hqer__induced sampler prototype	4.0
	front41Data	HQER-SFA	hqer__induced	True	18	-0.5501156676215098	hqer__induced sampler prototype	2.0
	front41Data	Quantile-SFA	hqer__induced	True	24	-5.834307555717605	hqer__induced sampler prototype	4.0
	front41Data	HQER-SFA	hqer__induced	True	42	-5.5707202678729	hqer__induced sampler prototype	2.0
	front41Data	Quantile-SFA	hqer__induced	True	15	-0.7205253620790469	hqer__induced sampler prototype	2.0
	front41Data	HQER-SFA	hqer__induced	True	16	1.6393227380033668	hqer__induced sampler prototype	2.0
	front41Data	Quantile-SFA	hqer__induced	True	25	4.617525547802108	hqer__induced sampler prototype	4.0
	front41Data	HQER-SFA	hqer__induced	True	29	7.777602692046176	hqer__induced sampler prototype	4.0

experiment	dataset	model	scenario	converged	n__iter	loglik	warning__message	best__start__id
	front41Data	Quantile-SFA	hqer__induced	True	45	8.657785906024928	hqer__induced sampler prototype	4.0
	front41Data	HQER-SFA	hqer__induced	True	18	10.626790354353082	hqer__induced sampler prototype	2.0
	front41Data	Quantile-SFA	hqer__induced	True	18	-8.159042011437023	hqer__induced sampler prototype	2.0
	front41Data	HQER-SFA	hqer__induced	True	36	-7.834633979965189	hqer__induced sampler prototype	4.0
	front41Data	Quantile-SFA	hqer__induced	True	30	-9.47160817259522	hqer__induced sampler prototype	2.0
	front41Data	HQER-SFA	hqer__induced	True	70	-5.044509547169295	hqer__induced sampler prototype	4.0
	front41Data	Quantile-SFA	hqer__induced	True	36	10.498489516698758	hqer__induced sampler prototype	2.0
	front41Data	HQER-SFA	hqer__induced	True	37	10.59029503944733	hqer__induced sampler prototype	2.0
	front41Data	Quantile-SFA	hqer__induced	True	58	3.740964444207173	hqer__induced sampler prototype	1.0
	front41Data	HQER-SFA	hqer__induced	True	25	5.164589077025169	hqer__induced sampler prototype	2.0
	front41Data	Quantile-SFA	hqer__induced	True	13	-2.354972455618161	hqer__induced sampler prototype	2.0
	front41Data	HQER-SFA	hqer__induced	True	16	1.294231287988066	hqer__induced sampler prototype	2.0
	front41Data	Quantile-SFA	hqer__induced	True	12	-13.464437396725534	hqer__induced sampler prototype	4.0
	front41Data	HQER-SFA	hqer__induced	True	13	-11.597177314673273	hqer__induced sampler prototype	4.0
	front41Data	Quantile-SFA	hqer__induced	True	23	-3.1329928104469627	hqer__induced sampler prototype	4.0
	front41Data	HQER-SFA	hqer__induced	True	19	-0.488784155943012	hqer__induced sampler prototype	4.0
	front41Data	Quantile-SFA	hqer__induced	True	37	0.0886681406053354	hqer__induced sampler prototype	2.0
	front41Data	HQER-SFA	hqer__induced	True	54	0.129999969850929	hqer__induced sampler prototype	4.0
	front41Data	Quantile-SFA	hqer__induced	True	28	3.234840068640325	hqer__induced sampler prototype	4.0
	front41Data	HQER-SFA	hqer__induced	True	42	5.104663558071579	hqer__induced sampler prototype	4.0
	front41Data	Quantile-SFA	hqer__induced	True	14	0.1717363850877793	hqer__induced sampler prototype	2.0
	front41Data	HQER-SFA	hqer__induced	True	78	5.258635906174425	hqer__induced sampler prototype	2.0
	front41Data	Quantile-SFA	hqer__induced	True	13	-9.549950249033692	hqer__induced sampler prototype	2.0
	front41Data	HQER-SFA	hqer__induced	True	32	-8.184687590910384	hqer__induced sampler prototype	2.0
	front41Data	Quantile-SFA	hqer__induced	True	31	-4.796605595764893	hqer__induced sampler prototype	2.0
	front41Data	HQER-SFA	hqer__induced	True	30	-4.871966432137894	hqer__induced sampler prototype	2.0
	front41Data	Quantile-SFA	hqer__induced	True	24	13.0631781694684	hqer__induced sampler prototype	4.0
	front41Data	HQER-SFA	hqer__induced	True	9	13.029709223847105	hqer__induced sampler prototype	2.0
	front41Data	Quantile-SFA	hqer__induced	True	26	-6.482876607989879	hqer__induced sampler prototype	4.0
	front41Data	HQER-SFA	hqer__induced	True	29	-4.474551331076823	hqer__induced sampler prototype	2.0
	front41Data	Quantile-SFA	hqer__induced	True	33	-8.487934186148664	hqer__induced sampler prototype	2.0
	front41Data	HQER-SFA	hqer__induced	True	18	-6.489811059743264	hqer__induced sampler prototype	4.0
	front41Data	Quantile-SFA	hqer__induced	True	46	-12.005800253057757	hqer__induced sampler prototype	2.0
	front41Data	HQER-SFA	hqer__induced	True	30	-10.009161175257011	hqer__induced sampler prototype	2.0
	front41Data	Quantile-SFA	hqer__induced	True	24	-17.852075888724002	hqer__induced sampler prototype	4.0
	front41Data	HQER-SFA	hqer__induced	True	29	-17.23908239021932	hqer__induced sampler prototype	4.0
	front41Data	Quantile-SFA	hqer__induced	True	37	1.1046733856160214	hqer__induced sampler prototype	2.0
	front41Data	HQER-SFA	hqer__induced	True	25	2.9227864140730024	hqer__induced sampler prototype	2.0
	front41Data	Quantile-SFA	hqer__induced	True	15	0.9119933672442636	hqer__induced sampler prototype	4.0
	front41Data	HQER-SFA	hqer__induced	True	61	1.3844298932856105	hqer__induced sampler prototype	4.0
	front41Data	Quantile-SFA	hqer__induced	True	31	10.59075136217108	hqer__induced sampler prototype	4.0
	front41Data	HQER-SFA	hqer__induced	True	25	10.59297427604163	hqer__induced sampler prototype	4.0
	front41Data	Quantile-SFA	hqer__induced	True	17	1.4881602244500929	hqer__induced sampler prototype	2.0
	front41Data	HQER-SFA	hqer__induced	True	17	2.353215850954652	hqer__induced sampler prototype	2.0
	front41Data	Quantile-SFA	hqer__induced	True	16	-4.023667080337308	hqer__induced sampler prototype	4.0
	front41Data	HQER-SFA	hqer__induced	True	56	-0.5993357805478448	hqer__induced sampler prototype	4.0
	front41Data	Quantile-SFA	hqer__induced	True	11	4.147408209228828	hqer__induced sampler prototype	2.0
	front41Data	HQER-SFA	hqer__induced	True	42	6.164137041046989	hqer__induced sampler prototype	4.0
	front41Data	Quantile-SFA	hqer__induced	True	36	2.317825054912612	hqer__induced sampler prototype	4.0
	front41Data	HQER-SFA	hqer__induced	True	13	4.024910345006695	hqer__induced sampler prototype	2.0
	front41Data	Quantile-SFA	hqer__induced	True	30	6.118863908110509	hqer__induced sampler prototype	4.0
	front41Data	HQER-SFA	hqer__induced	True	37	7.210050313183475	hqer__induced sampler prototype	4.0
	front41Data	Quantile-SFA	hqer__induced	True	23	-0.0399782360133393	hqer__induced sampler prototype	4.0
	front41Data	HQER-SFA	hqer__induced	True	11	1.1175789331490251	hqer__induced sampler prototype	2.0
	front41Data	Quantile-SFA	hqer__induced	True	22	5.373985818958719	hqer__induced sampler prototype	4.0
	front41Data	HQER-SFA	hqer__induced	True	54	6.045519731009722	hqer__induced sampler prototype	4.0
	front41Data	Quantile-SFA	hqer__induced	True	20	6.662353968550195	hqer__induced sampler prototype	4.0
	front41Data	HQER-SFA	hqer__induced	True	23	6.394288843167652	hqer__induced sampler prototype	4.0
	front41Data	Quantile-SFA	hqer__induced	True	24	-6.995532877514608	hqer__induced sampler prototype	4.0
	front41Data	HQER-SFA	hqer__induced	True	45	-4.429914072932882	hqer__induced sampler prototype	2.0
	front41Data	Quantile-SFA	hqer__induced	True	48	0.2440403763801081	hqer__induced sampler prototype	2.0
	front41Data	HQER-SFA	hqer__induced	True	28	2.113796958967213	hqer__induced sampler prototype	4.0
	front41Data	Quantile-SFA	hqer__induced	True	17	-8.643321408754804	hqer__induced sampler prototype	2.0
	front41Data	HQER-SFA	hqer__induced	True	25	-6.027697641977955	hqer__induced sampler prototype	4.0
	front41Data	Quantile-SFA	hqer__induced	True	33	-11.084333117643556	hqer__induced sampler prototype	2.0
	front41Data	HQER-SFA	hqer__induced	True	55	-8.537883293292788	hqer__induced sampler prototype	2.0
	front41Data	Quantile-SFA	hqer__induced	True	15	-0.9567939862567644	hqer__induced sampler prototype	2.0
	front41Data	HQER-SFA	hqer__induced	True	21	-0.039232955503729	hqer__induced sampler prototype	4.0
	front41Data	Quantile-SFA	hqer__induced	True	39	-2.352221439153537	hqer__induced sampler prototype	4.0
	front41Data	HQER-SFA	hqer__induced	True	44	-0.3208507524356366	hqer__induced sampler prototype	2.0
	front41Data	Quantile-SFA	hqer__induced	True	22	-8.545830543276411	hqer__induced sampler prototype	4.0
	front41Data	HQER-SFA	hqer__induced	True	12	-7.207732577813453	hqer__induced sampler prototype	2.0
	front41Data	Quantile-SFA	hqer__induced	True	14	-2.1489055343395185	hqer__induced sampler prototype	2.0
	front41Data	HQER-SFA	hqer__induced	True	31	3.610831975562334	hqer__induced sampler prototype	2.0
	front41Data	Quantile-SFA	hqer__induced	True	22	-3.21212745801049	hqer__induced sampler prototype	4.0
	front41Data	HQER-SFA	hqer__induced	True	18	-0.6898377957116988	hqer__induced sampler prototype	2.0
	front41Data	Quantile-SFA	hqer__induced	True	12	-7.420086716554438	hqer__induced sampler prototype	4.0
	front41Data	HQER-SFA	hqer__induced	True	19	-5.108705247499226	hqer__induced sampler prototype	4.0
	front41Data	Quantile-SFA	hqer__induced	True	21	13.551147363799249	hqer__induced sampler prototype	4.0
	front41Data	HQER-SFA	hqer__induced	True	85	13.568602548025131	hqer__induced sampler prototype	4.0
	front41Data	Quantile-SFA	hqer__induced	True	42	-5.163106532035607	hqer__induced sampler prototype	4.0
	front41Data	HQER-SFA	hqer__induced	True	36	-4.080578405746547	hqer__induced sampler prototype	4.0
	front41Data	Quantile-SFA	hqer__induced	True	9	-2.7991063530197184	hqer__induced sampler prototype	2.0
	front41Data	HQER-SFA	hqer__induced	True	47	-3.2200048093604696	hqer__induced sampler prototype	2.0
	front41Data	Quantile-SFA	hqer__induced	True	39	-0.8459542568205818	hqer__induced sampler prototype	4.0
	front41Data	HQER-SFA	hqer__induced	True	27	0.61522654121318	hqer__induced sampler prototype	4.0
	front41Data	Quantile-SFA	hqer__induced	True	11	-8.037384054926477	hqer__induced sampler prototype	2.0
	front41Data	HQER-SFA	hqer__induced	True	33	-4.956958238302051	hqer__induced sampler prototype	4.0
	front41Data	Quantile-SFA	hqer__induced	True	25	-5.112781610696632	hqer__induced sampler prototype	2.0
	front41Data	HQER-SFA	hqer__induced	True	27	-4.760804441149273	hqer__induced sampler prototype	2.0
	front41Data	Quantile-SFA	hqer__induced	True	17	-5.340801738962942	hqer__induced sampler prototype	4.0
	front41Data	HQER-SFA	hqer__induced	True	11	-3.228109093482399	hqer__induced sampler prototype	2.0
	front41Data	Quantile-SFA	hqer__induced	True	15	1.5736253643921627	hqer__induced sampler prototype	2.0
	front41Data	HQER-SFA	hqer__induced	True	37	1.5369385793602133	hqer__induced sampler prototype	2.0
	front41Data	Quantile-SFA	hqer__induced	True	25	-0.5625796187627345	hqer__induced sampler prototype	2.0
	front41Data	HQER-SFA	hqer__induced	True	13	1.8056029612584528	hqer__induced sampler prototype	2.0
	front41Data	Quantile-SFA	hqer__induced	True	32	8.399359717567519	hqer__induced sampler prototype	3.0
	front41Data	HQER-SFA	hqer__induced	True	23	9.248632610864796	hqer__induced sampler prototype	4.0
	front41Data	Quantile-SFA	hqer__induced	True	41	4.830394018781366	hqer__induced sampler prototype	2.0
	front41Data	HQER-SFA	hqer__induced	True	15	7.3234511969608755	hqer__induced sampler prototype	4.0
	front41Data	Quantile-SFA	hqer__induced	True	17	-11.862887333588985	hqer__induced sampler prototype	4.0
	front41Data	HQER-SFA	hqer__induced	True	21	-9.479319850997546	hqer__induced sampler prototype	4.0
	front41Data	Quantile-SFA	hqer__induced	True	11	-6.732218266443687	hqer__induced sampler prototype	4.0

experiment	dataset	model	scenario	converged	n_iter	loglik	warning_message	best_start_id
	front41Data	HQER-SFA	hqer_induced	True	18	-3.166285217307506	hqer_induced sampler prototype	4.0
	front41Data	Quantile-SFA	hqer_induced	True	17	-5.288586706515818	hqer_induced sampler prototype	4.0
	front41Data	HQER-SFA	hqer_induced	True	26	-3.76761602760794	hqer_induced sampler prototype	2.0
	front41Data	Quantile-SFA	hqer_induced	True	17	-9.629063944455575	hqer_induced sampler prototype	2.0
	front41Data	HQER-SFA	hqer_induced	True	31	-8.078021818418799	hqer_induced sampler prototype	4.0
	front41Data	Quantile-SFA	hqer_induced	True	68	2.4248045268419416	hqer_induced sampler prototype	4.0
	front41Data	HQER-SFA	hqer_induced	True	85	1.95873734192044	hqer_induced sampler prototype	4.0
	front41Data	Quantile-SFA	hqer_induced	True	56	-3.839461166496658	hqer_induced sampler prototype	2.0
	front41Data	HQER-SFA	hqer_induced	True	8	-4.108778872423819	hqer_induced sampler prototype	2.0
	front41Data	Quantile-SFA	hqer_induced	True	28	-2.510573822996476	hqer_induced sampler prototype	2.0
	front41Data	HQER-SFA	hqer_induced	True	55	-2.734414840004648	hqer_induced sampler prototype	4.0
	front41Data	Quantile-SFA	hqer_induced	True	21	3.303715265578097	hqer_induced sampler prototype	2.0
	front41Data	HQER-SFA	hqer_induced	True	43	8.141462863202237	hqer_induced sampler prototype	2.0
	front41Data	Quantile-SFA	hqer_induced	True	14	-9.456396948947027	hqer_induced sampler prototype	2.0
	front41Data	HQER-SFA	hqer_induced	True	21	-6.526611848002511	hqer_induced sampler prototype	4.0
	front41Data	Quantile-SFA	hqer_induced	True	17	6.2475538991734645	hqer_induced sampler prototype	4.0
	front41Data	HQER-SFA	hqer_induced	True	12	4.183410603893519	hqer_induced sampler prototype	2.0
	front41Data	Quantile-SFA	hqer_induced	True	24	2.602305896785417	hqer_induced sampler prototype	4.0
	front41Data	HQER-SFA	hqer_induced	True	34	2.463109159848925	hqer_induced sampler prototype	2.0
	front41Data	Quantile-SFA	hqer_induced	True	17	-2.9315481408865987	hqer_induced sampler prototype	4.0
	front41Data	HQER-SFA	hqer_induced	True	22	-1.5316383209374718	hqer_induced sampler prototype	4.0
	front41Data	Quantile-SFA	hqer_induced	True	29	5.899565724020329	hqer_induced sampler prototype	4.0
	front41Data	HQER-SFA	hqer_induced	True	50	8.554573579842533	hqer_induced sampler prototype	3.0
	front41Data	Quantile-SFA	hqer_induced	True	19	-5.141065936939682	hqer_induced sampler prototype	4.0
	front41Data	HQER-SFA	hqer_induced	True	11	-3.970228353019788	hqer_induced sampler prototype	2.0
	front41Data	Quantile-SFA	hqer_induced	True	17	-6.95577187576417	hqer_induced sampler prototype	2.0
	front41Data	HQER-SFA	hqer_induced	True	29	-3.249618131703308	hqer_induced sampler prototype	2.0
	front41Data	Quantile-SFA	hqer_induced	True	34	0.8079833273904403	hqer_induced sampler prototype	4.0
	front41Data	HQER-SFA	hqer_induced	True	40	3.148020867782667	hqer_induced sampler prototype	2.0
	front41Data	Quantile-SFA	hqer_induced	True	13	-0.123023680438691	hqer_induced sampler prototype	4.0
	front41Data	HQER-SFA	hqer_induced	True	34	4.521044819028353	hqer_induced sampler prototype	2.0
	front41Data	Quantile-SFA	hqer_induced	True	35	3.528031725177781	hqer_induced sampler prototype	4.0
	front41Data	HQER-SFA	hqer_induced	True	27	3.763447574264129	hqer_induced sampler prototype	2.0
	front41Data	Quantile-SFA	hqer_induced	True	15	2.7443944967625447	hqer_induced sampler prototype	2.0
	front41Data	HQER-SFA	hqer_induced	True	42	2.950377677692429	hqer_induced sampler prototype	4.0
	front41Data	Quantile-SFA	hqer_induced	True	14	-8.171537294564043	hqer_induced sampler prototype	4.0
	front41Data	HQER-SFA	hqer_induced	True	22	-6.793365867221433	hqer_induced sampler prototype	4.0
	front41Data	Quantile-SFA	hqer_induced	True	26	-12.863682200703048	hqer_induced sampler prototype	4.0
	front41Data	HQER-SFA	hqer_induced	True	33	-10.386135851982088	hqer_induced sampler prototype	2.0
	front41Data	Quantile-SFA	hqer_induced	True	21	-4.462265025762451	hqer_induced sampler prototype	4.0
	front41Data	HQER-SFA	hqer_induced	True	43	-4.641853664235867	hqer_induced sampler prototype	4.0
	front41Data	Quantile-SFA	hqer_induced	True	16	-12.775022319565007	hqer_induced sampler prototype	2.0
	front41Data	HQER-SFA	hqer_induced	True	24	-11.875307685654034	hqer_induced sampler prototype	4.0
	front41Data	Quantile-SFA	hqer_induced	True	12	-3.078496167736874	hqer_induced sampler prototype	2.0
	front41Data	HQER-SFA	hqer_induced	True	39	-0.2884340832189033	hqer_induced sampler prototype	5.0
	front41Data	Quantile-SFA	hqer_induced	True	19	-0.9825225544397208	hqer_induced sampler prototype	2.0
	front41Data	HQER-SFA	hqer_induced	True	10	0.7110424142343081	hqer_induced sampler prototype	4.0
	front41Data	Quantile-SFA	hqer_induced	True	10	2.403524505654113	hqer_induced sampler prototype	4.0
	front41Data	HQER-SFA	hqer_induced	True	30	6.673318144986385	hqer_induced sampler prototype	2.0
	front41Data	Quantile-SFA	hqer_induced	True	25	-2.860719139882169	hqer_induced sampler prototype	4.0
	front41Data	HQER-SFA	hqer_induced	True	20	1.5888912738183372	hqer_induced sampler prototype	4.0
	front41Data	Quantile-SFA	hqer_induced	True	35	-2.4227711552565427	hqer_induced sampler prototype	2.0
	front41Data	HQER-SFA	hqer_induced	True	47	-1.1637977840363578	hqer_induced sampler prototype	2.0
	front41Data	Quantile-SFA	hqer_induced	True	13	-8.584745035258337	hqer_induced sampler prototype	2.0
	front41Data	HQER-SFA	hqer_induced	True	25	-6.081524181227119	hqer_induced sampler prototype	2.0
	front41Data	Quantile-SFA	hqer_induced	True	35	-1.8808212720566784	hqer_induced sampler prototype	4.0
	front41Data	HQER-SFA	hqer_induced	True	25	-1.54391333867588	hqer_induced sampler prototype	4.0
	front41Data	Quantile-SFA	hqer_induced	True	19	-16.333662115673068	hqer_induced sampler prototype	4.0
	front41Data	HQER-SFA	hqer_induced	True	53	-10.915159846284585	hqer_induced sampler prototype	2.0
	front41Data	Quantile-SFA	hqer_induced	True	23	-7.201061833848899	hqer_induced sampler prototype	4.0
	front41Data	HQER-SFA	hqer_induced	True	18	-6.529147770313009	hqer_induced sampler prototype	2.0
	front41Data	Quantile-SFA	hqer_induced	True	24	9.40418166921363	hqer_induced sampler prototype	4.0
	front41Data	HQER-SFA	hqer_induced	True	28	10.290284186950393	hqer_induced sampler prototype	4.0
	front41Data	Quantile-SFA	hqer_induced	True	16	-0.9306767770077272	hqer_induced sampler prototype	2.0
	front41Data	HQER-SFA	hqer_induced	True	27	1.0034081250283655	hqer_induced sampler prototype	2.0
	front41Data	Quantile-SFA	hqer_induced	True	26	-2.17240871533342	hqer_induced sampler prototype	4.0
	front41Data	HQER-SFA	hqer_induced	True	51	0.7544762100452072	hqer_induced sampler prototype	4.0
	front41Data	Quantile-SFA	hqer_induced	True	30	-4.228626013859181	hqer_induced sampler prototype	4.0
	front41Data	HQER-SFA	hqer_induced	True	42	-0.9506849919698788	hqer_induced sampler prototype	2.0
	front41Data	Quantile-SFA	hqer_induced	True	24	-7.094189904819015	hqer_induced sampler prototype	4.0
	front41Data	HQER-SFA	hqer_induced	True	18	-5.537712387351096	hqer_induced sampler prototype	2.0
	front41Data	Quantile-SFA	hqer_induced	True	11	5.902160062655932	hqer_induced sampler prototype	2.0
	front41Data	HQER-SFA	hqer_induced	True	58	6.28798508119757	hqer_induced sampler prototype	2.0
	front41Data	Quantile-SFA	hqer_induced	True	23	-7.930520683981278	hqer_induced sampler prototype	4.0
	front41Data	HQER-SFA	hqer_induced	True	49	-1.9678615395958725	hqer_induced sampler prototype	4.0
	front41Data	Quantile-SFA	hqer_induced	True	22	-9.80336767984721	hqer_induced sampler prototype	4.0
	front41Data	HQER-SFA	hqer_induced	True	37	-9.990147304725555	hqer_induced sampler prototype	4.0
	front41Data	Quantile-SFA	hqer_induced	True	17	-8.023236108093696	hqer_induced sampler prototype	2.0
	front41Data	HQER-SFA	hqer_induced	True	29	-7.085472197484273	hqer_induced sampler prototype	2.0
	front41Data	Quantile-SFA	hqer_induced	True	13	-6.489748167203905	hqer_induced sampler prototype	2.0
	front41Data	HQER-SFA	hqer_induced	True	42	-4.730269871934478	hqer_induced sampler prototype	4.0
	front41Data	Quantile-SFA	hqer_induced	True	17	-2.052580617317945	hqer_induced sampler prototype	4.0
	front41Data	HQER-SFA	hqer_induced	True	44	-1.9239573461094543	hqer_induced sampler prototype	2.0
	front41Data	Quantile-SFA	hqer_induced	True	26	7.196472801857007	hqer_induced sampler prototype	4.0
	front41Data	HQER-SFA	hqer_induced	True	63	6.943194197009142	hqer_induced sampler prototype	2.0
	front41Data	Quantile-SFA	hqer_induced	True	10	-11.934092555359822	hqer_induced sampler prototype	2.0
	front41Data	HQER-SFA	hqer_induced	True	40	-8.175703252558446	hqer_induced sampler prototype	2.0
	front41Data	Quantile-SFA	hqer_induced	True	23	-7.65233980620026	hqer_induced sampler prototype	4.0
	front41Data	HQER-SFA	hqer_induced	True	24	-5.611616579917899	hqer_induced sampler prototype	2.0
	front41Data	Quantile-SFA	hqer_induced	True	39	0.1665242769792086	hqer_induced sampler prototype	4.0
	front41Data	HQER-SFA	hqer_induced	True	47	1.4285837512108104	hqer_induced sampler prototype	4.0
	front41Data	Quantile-SFA	hqer_induced	True	31	-2.3149051851504647	hqer_induced sampler prototype	4.0
	front41Data	HQER-SFA	hqer_induced	True	14	-0.7821608047630052	hqer_induced sampler prototype	2.0
	front41Data	Quantile-SFA	hqer_induced	True	16	-8.84829361143042	hqer_induced sampler prototype	4.0
	front41Data	HQER-SFA	hqer_induced	True	52	-6.031859610975893	hqer_induced sampler prototype	2.0
	front41Data	Quantile-SFA	hqer_induced	True	20	-5.459159741875244	hqer_induced sampler prototype	2.0
	front41Data	HQER-SFA	hqer_induced	True	48	-2.701806755334759	hqer_induced sampler prototype	2.0
	front41Data	Quantile-SFA	hqer_induced	True	23	2.46709691672664	hqer_induced sampler prototype	4.0
	front41Data	HQER-SFA	hqer_induced	True	58	6.5958515691572845	hqer_induced sampler prototype	2.0
	front41Data	Quantile-SFA	hqer_induced	True	40	1.5199861851826997	hqer_induced sampler prototype	4.0
	front41Data	HQER-SFA	hqer_induced	True	45	3.501614746566737	hqer_induced sampler prototype	2.0
	front41Data	Quantile-SFA	hqer_induced	True	26	2.873780913332847	hqer_induced sampler prototype	2.0
	front41Data	HQER-SFA	hqer_induced	True	54	7.350510521675397	hqer_induced sampler prototype	4.0

experiment	dataset	model	scenario	converged	n_iter	loglik	warning_message	best_start_id
	front41Data	Quantile-SFA	hqer_induced	True	16	7.962439503169038	hqer_induced sampler prototype	2.0
	front41Data	HQER-SFA	hqer_induced	True	43	13.044419012510993	hqer_induced sampler prototype	4.0
	front41Data	Quantile-SFA	hqer_induced	True	30	5.00529896218448	hqer_induced sampler prototype	4.0
	front41Data	HQER-SFA	hqer_induced	True	66	9.935241362931476	hqer_induced sampler prototype	4.0
	front41Data	Quantile-SFA	hqer_induced	True	15	-7.800774317423408	hqer_induced sampler prototype	2.0
	front41Data	HQER-SFA	hqer_induced	True	13	-6.152975648892564	hqer_induced sampler prototype	2.0
	front41Data	Quantile-SFA	hqer_induced	True	22	-6.164838434853478	hqer_induced sampler prototype	2.0
	front41Data	HQER-SFA	hqer_induced	True	18	-4.376999308788159	hqer_induced sampler prototype	2.0
	front41Data	Quantile-SFA	hqer_induced	True	32	-2.281343842882508	hqer_induced sampler prototype	4.0
	front41Data	HQER-SFA	hqer_induced	True	28	-1.5463108866858402	hqer_induced sampler prototype	2.0
	front41Data	Quantile-SFA	hqer_induced	True	16	-13.215355356997016	hqer_induced sampler prototype	4.0
	front41Data	HQER-SFA	hqer_induced	True	21	-7.718784177422919	hqer_induced sampler prototype	2.0
	front41Data	Quantile-SFA	hqer_induced	True	13	-16.121896762602965	hqer_induced sampler prototype	4.0
	front41Data	HQER-SFA	hqer_induced	True	53	-12.71271263938192	hqer_induced sampler prototype	4.0
	front41Data	Quantile-SFA	hqer_induced	True	32	-6.307584753901869	hqer_induced sampler prototype	2.0
	front41Data	HQER-SFA	hqer_induced	True	45	-5.852232111041258	hqer_induced sampler prototype	2.0
	front41Data	Quantile-SFA	hqer_induced	True	12	-2.6408547289244413	hqer_induced sampler prototype	2.0
	front41Data	HQER-SFA	hqer_induced	True	50	2.6972182249037675	hqer_induced sampler prototype	2.0
	front41Data	Quantile-SFA	hqer_induced	True	13	-8.165862312548407	hqer_induced sampler prototype	2.0
	front41Data	HQER-SFA	hqer_induced	True	14	-5.891437327398629	hqer_induced sampler prototype	2.0
	front41Data	Quantile-SFA	hqer_induced	True	17	-0.7310098743040987	hqer_induced sampler prototype	2.0
	front41Data	HQER-SFA	hqer_induced	True	50	4.021100660813077	hqer_induced sampler prototype	2.0
	front41Data	Quantile-SFA	hqer_induced	True	21	-9.705071734882688	hqer_induced sampler prototype	2.0
	front41Data	HQER-SFA	hqer_induced	True	41	-6.567981433098824	hqer_induced sampler prototype	4.0
	front41Data	Quantile-SFA	hqer_induced	True	11	1.107827873801364	hqer_induced sampler prototype	2.0
	front41Data	HQER-SFA	hqer_induced	True	40	2.1726130371819945	hqer_induced sampler prototype	2.0
	front41Data	Quantile-SFA	hqer_induced	True	44	2.8017281849678213	hqer_induced sampler prototype	4.0
	front41Data	HQER-SFA	hqer_induced	True	50	5.5245250744769	hqer_induced sampler prototype	4.0
	front41Data	Quantile-SFA	hqer_induced	True	15	0.782734763109266	hqer_induced sampler prototype	4.0
	front41Data	HQER-SFA	hqer_induced	True	15	1.5400912578133927	hqer_induced sampler prototype	2.0
	front41Data	Quantile-SFA	hqer_induced	True	29	-4.857528572865558	hqer_induced sampler prototype	4.0
	front41Data	HQER-SFA	hqer_induced	True	11	-3.825414174772813	hqer_induced sampler prototype	4.0
	front41Data	Quantile-SFA	hqer_induced	True	15	-7.060840488712568	hqer_induced sampler prototype	2.0
	front41Data	HQER-SFA	hqer_induced	True	35	-7.612700133500175	hqer_induced sampler prototype	2.0
	front41Data	Quantile-SFA	hqer_induced	True	12	-1.4844593573014642	hqer_induced sampler prototype	4.0
	front41Data	HQER-SFA	hqer_induced	True	23	-0.3310015302672773	hqer_induced sampler prototype	2.0
	front41Data	Quantile-SFA	hqer_induced	True	25	0.4043068881349043	hqer_induced sampler prototype	2.0
	front41Data	HQER-SFA	hqer_induced	True	28	3.8005474661073886	hqer_induced sampler prototype	4.0
	front41Data	Quantile-SFA	hqer_induced	True	20	3.379051553246298	hqer_induced sampler prototype	4.0
	front41Data	HQER-SFA	hqer_induced	True	39	4.769176388068639	hqer_induced sampler prototype	4.0
	front41Data	Quantile-SFA	hqer_induced	True	17	4.238907864448581	hqer_induced sampler prototype	4.0
	front41Data	HQER-SFA	hqer_induced	True	16	5.267244377514112	hqer_induced sampler prototype	2.0
	front41Data	Quantile-SFA	hqer_induced	True	10	-6.442192716997522	hqer_induced sampler prototype	2.0
	front41Data	HQER-SFA	hqer_induced	True	29	-1.7975865435202625	hqer_induced sampler prototype	4.0
	front41Data	Quantile-SFA	hqer_induced	True	20	-9.871981132295438	hqer_induced sampler prototype	4.0
	front41Data	HQER-SFA	hqer_induced	True	21	-9.75857895393352	hqer_induced sampler prototype	4.0
	front41Data	Quantile-SFA	hqer_induced	True	13	1.2976192249276275	hqer_induced sampler prototype	2.0
	front41Data	HQER-SFA	hqer_induced	True	57	2.592789992137182	hqer_induced sampler prototype	4.0
	front41Data	Quantile-SFA	hqer_induced	True	62	-0.4188597712911266	hqer_induced sampler prototype	4.0
	front41Data	HQER-SFA	hqer_induced	True	28	0.2101418453521342	hqer_induced sampler prototype	2.0
	front41Data	Quantile-SFA	hqer_induced	True	23	-0.7106090157518701	hqer_induced sampler prototype	2.0
	front41Data	HQER-SFA	hqer_induced	True	31	0.1629659027088225	hqer_induced sampler prototype	2.0
	front41Data	Quantile-SFA	hqer_induced	True	17	-12.03137458105308	hqer_induced sampler prototype	2.0
	front41Data	HQER-SFA	hqer_induced	True	20	-11.47673987209861	hqer_induced sampler prototype	4.0
	front41Data	Quantile-SFA	hqer_induced	True	23	-6.617928008280684	hqer_induced sampler prototype	4.0
	front41Data	HQER-SFA	hqer_induced	True	24	-4.875402412604125	hqer_induced sampler prototype	4.0
	front41Data	Quantile-SFA	hqer_induced	True	29	-14.47533415642478	hqer_induced sampler prototype	2.0
	front41Data	HQER-SFA	hqer_induced	True	31	-8.82861695491318	hqer_induced sampler prototype	4.0
	front41Data	Quantile-SFA	hqer_induced	True	36	-5.214000815353172	hqer_induced sampler prototype	2.0
	front41Data	HQER-SFA	hqer_induced	True	52	-2.3736322430828523	hqer_induced sampler prototype	2.0
	front41Data	Quantile-SFA	hqer_induced	True	34	6.304208121620656	hqer_induced sampler prototype	3.0
	front41Data	HQER-SFA	hqer_induced	True	31	7.050972387294514	hqer_induced sampler prototype	2.0
	front41Data	Quantile-SFA	hqer_induced	True	23	1.6833896838827416	hqer_induced sampler prototype	2.0
	front41Data	HQER-SFA	hqer_induced	True	39	4.499886811068235	hqer_induced sampler prototype	4.0
	front41Data	Quantile-SFA	hqer_induced	True	20	-2.417683771021587	hqer_induced sampler prototype	2.0
	front41Data	HQER-SFA	hqer_induced	True	22	-0.8079943757914685	hqer_induced sampler prototype	2.0
	front41Data	Quantile-SFA	hqer_induced	True	22	-6.606553150623803	hqer_induced sampler prototype	2.0
	front41Data	HQER-SFA	hqer_induced	True	13	-4.2642262359431	hqer_induced sampler prototype	4.0
	front41Data	Quantile-SFA	hqer_induced	True	20	-2.502237792265289	hqer_induced sampler prototype	2.0
	front41Data	HQER-SFA	hqer_induced	True	40	-2.091008123953848	hqer_induced sampler prototype	2.0
	front41Data	Quantile-SFA	hqer_induced	True	32	-3.45754197616583	hqer_induced sampler prototype	4.0
	front41Data	HQER-SFA	hqer_induced	True	41	-2.7195797824189825	hqer_induced sampler prototype	2.0
	front41Data	Quantile-SFA	hqer_induced	True	10	-1.0415772741191212	hqer_induced sampler prototype	2.0
	front41Data	HQER-SFA	hqer_induced	True	37	2.605517093418136	hqer_induced sampler prototype	4.0
	front41Data	Quantile-SFA	hqer_induced	True	17	-4.008822147166901	hqer_induced sampler prototype	2.0
	front41Data	HQER-SFA	hqer_induced	True	25	-2.264121405902868	hqer_induced sampler prototype	3.0
	front41Data	Quantile-SFA	hqer_induced	True	16	-5.757682494866945	hqer_induced sampler prototype	2.0
	front41Data	HQER-SFA	hqer_induced	True	45	-4.82148855734992	hqer_induced sampler prototype	4.0
	front41Data	Quantile-SFA	hqer_induced	True	11	-7.99014603540787	hqer_induced sampler prototype	2.0
	front41Data	HQER-SFA	hqer_induced	True	38	-5.444119407348875	hqer_induced sampler prototype	4.0
	front41Data	Quantile-SFA	hqer_induced	True	27	-7.440548048611483	hqer_induced sampler prototype	4.0
	front41Data	HQER-SFA	hqer_induced	True	57	-3.3899490213584667	hqer_induced sampler prototype	2.0
	front41Data	Quantile-SFA	hqer_induced	True	19	-1.8717001409823488	hqer_induced sampler prototype	2.0
	front41Data	HQER-SFA	hqer_induced	True	24	-0.4503073588738451	hqer_induced sampler prototype	4.0
	front41Data	Quantile-SFA	hqer_induced	True	26	-1.697166661644239	hqer_induced sampler prototype	4.0
	front41Data	HQER-SFA	hqer_induced	True	34	-0.5399805118193951	hqer_induced sampler prototype	2.0
	front41Data	Quantile-SFA	hqer_induced	True	17	-12.381012064631092	hqer_induced sampler prototype	4.0
	front41Data	HQER-SFA	hqer_induced	True	34	-9.158389309907877	hqer_induced sampler prototype	2.0
	front41Data	Quantile-SFA	hqer_induced	True	36	5.237369927726343	hqer_induced sampler prototype	2.0
	front41Data	HQER-SFA	hqer_induced	True	50	4.77591368098623	hqer_induced sampler prototype	2.0
	front41Data	Quantile-SFA	hqer_induced	True	26	0.7385887588769291	hqer_induced sampler prototype	2.0
	front41Data	HQER-SFA	hqer_induced	True	33	0.9124893303959286	hqer_induced sampler prototype	2.0
	front41Data	Quantile-SFA	hqer_induced	True	35	-8.01495718259215	hqer_induced sampler prototype	2.0
	front41Data	HQER-SFA	hqer_induced	True	36	-8.371486270635037	hqer_induced sampler prototype	4.0
	front41Data	Quantile-SFA	hqer_induced	True	49	0.6207961070537228	hqer_induced sampler prototype	4.0
	front41Data	HQER-SFA	hqer_induced	True	30	4.334085303193923	hqer_induced sampler prototype	4.0
	front41Data	Quantile-SFA	hqer_induced	True	17	2.5222963483912806	hqer_induced sampler prototype	4.0
	front41Data	HQER-SFA	hqer_induced	True	40	6.799756300333306	hqer_induced sampler prototype	2.0
	front41Data	Quantile-SFA	hqer_induced	True	33	5.936495328181968	hqer_induced sampler prototype	4.0
	front41Data	HQER-SFA	hqer_induced	True	76	6.0754693616173245	hqer_induced sampler prototype	4.0
	front41Data	Quantile-SFA	hqer_induced	True	44	-8.903498645038326	hqer_induced sampler prototype	4.0
	front41Data	HQER-SFA	hqer_induced	True	27	-7.728975642677886	hqer_induced sampler prototype	2.0
	front41Data	Quantile-SFA	hqer_induced	True	31	-1.3272197552514098	hqer_induced sampler prototype	4.0

experiment	dataset	model	scenario	converged	n__iter	loglik	warning__message	best__start__id
	front41Data	HQER-SFA	hqer__induced	True	34	-0.4467778344954281	hqer__induced sampler prototype	2.0
	front41Data	Quantile-SFA	hqer__induced	True	33	-10.763470334893402	hqer__induced sampler prototype	2.0
	front41Data	HQER-SFA	hqer__induced	True	32	-8.787439210062084	hqer__induced sampler prototype	4.0
	front41Data	Quantile-SFA	hqer__induced	True	16	-5.738490872522852	hqer__induced sampler prototype	4.0
	front41Data	HQER-SFA	hqer__induced	True	14	-4.0585091398904645	hqer__induced sampler prototype	4.0
	front41Data	Quantile-SFA	hqer__induced	True	51	-1.55943731328346	hqer__induced sampler prototype	4.0
	front41Data	HQER-SFA	hqer__induced	True	19	3.2866463154777734	hqer__induced sampler prototype	2.0
	front41Data	Quantile-SFA	hqer__induced	True	45	2.186929971948397	hqer__induced sampler prototype	4.0
	front41Data	HQER-SFA	hqer__induced	True	34	2.2159234837161663	hqer__induced sampler prototype	4.0
	front41Data	Quantile-SFA	hqer__induced	True	23	-10.938538201090497	hqer__induced sampler prototype	2.0
	front41Data	HQER-SFA	hqer__induced	True	24	-9.093413852385122	hqer__induced sampler prototype	2.0
	front41Data	Quantile-SFA	hqer__induced	True	20	1.0721022384095855	hqer__induced sampler prototype	2.0
	front41Data	HQER-SFA	hqer__induced	True	51	2.776644073955347	hqer__induced sampler prototype	4.0
	front41Data	Quantile-SFA	hqer__induced	True	31	-5.312588299233439	hqer__induced sampler prototype	4.0
	front41Data	HQER-SFA	hqer__induced	True	25	-1.119925270886437	hqer__induced sampler prototype	2.0
	front41Data	Quantile-SFA	hqer__induced	True	9	-4.485318332631652	hqer__induced sampler prototype	2.0
	front41Data	HQER-SFA	hqer__induced	True	23	-3.100198418395017	hqer__induced sampler prototype	4.0
	front41Data	Quantile-SFA	hqer__induced	True	39	-0.2226571512698418	hqer__induced sampler prototype	4.0
	front41Data	HQER-SFA	hqer__induced	True	47	2.315934210616738	hqer__induced sampler prototype	4.0
	front41Data	Quantile-SFA	hqer__induced	True	16	-11.056100924528398	hqer__induced sampler prototype	2.0
	front41Data	HQER-SFA	hqer__induced	True	22	-8.337289744725037	hqer__induced sampler prototype	4.0
	front41Data	Quantile-SFA	hqer__induced	True	48	-0.6920827315842979	hqer__induced sampler prototype	4.0
	front41Data	HQER-SFA	hqer__induced	True	37	-0.67527065361671	hqer__induced sampler prototype	4.0
	front41Data	Quantile-SFA	hqer__induced	True	44	-5.662576419221628	hqer__induced sampler prototype	4.0
	front41Data	HQER-SFA	hqer__induced	True	108	-1.6821496185233094	hqer__induced sampler prototype	4.0
	front41Data	Quantile-SFA	hqer__induced	True	26	2.7161638950703613	hqer__induced sampler prototype	4.0
	front41Data	HQER-SFA	hqer__induced	True	12	4.358097469997787	hqer__induced sampler prototype	2.0
	front41Data	Quantile-SFA	hqer__induced	True	32	-7.461174432574746	hqer__induced sampler prototype	2.0
	front41Data	HQER-SFA	hqer__induced	True	45	-2.5609223717627856	hqer__induced sampler prototype	4.0
	front41Data	Quantile-SFA	hqer__induced	True	18	1.2527891792263688	hqer__induced sampler prototype	2.0
	front41Data	HQER-SFA	hqer__induced	True	37	1.8960377920824925	hqer__induced sampler prototype	4.0
	front41Data	Quantile-SFA	hqer__induced	True	13	5.273813745626919	hqer__induced sampler prototype	2.0
	front41Data	HQER-SFA	hqer__induced	True	37	6.925421784831952	hqer__induced sampler prototype	4.0
	front41Data	Quantile-SFA	hqer__induced	True	33	-4.782475668738419	hqer__induced sampler prototype	2.0
	front41Data	HQER-SFA	hqer__induced	True	82	-4.681381805695791	hqer__induced sampler prototype	2.0
	front41Data	Quantile-SFA	hqer__induced	True	14	-18.66981925037114	hqer__induced sampler prototype	2.0
	front41Data	HQER-SFA	hqer__induced	True	32	-6.409722976313232	hqer__induced sampler prototype	4.0
	front41Data	Quantile-SFA	hqer__induced	True	36	-43.12135979062394	hqer__induced sampler prototype	4.0
	front41Data	HQER-SFA	hqer__induced	True	30	-26.199804277502505	hqer__induced sampler prototype	4.0
	front41Data	Quantile-SFA	hqer__induced	True	11	-65.98267089209331	hqer__induced sampler prototype	4.0
	front41Data	HQER-SFA	hqer__induced	True	10	-49.96250801940249	hqer__induced sampler prototype	2.0
	front41Data	Quantile-SFA	hqer__induced	True	31	-58.29909316519296	hqer__induced sampler prototype	2.0
	front41Data	HQER-SFA	hqer__induced	True	11	-44.5264933014196	hqer__induced sampler prototype	2.0
	front41Data	Quantile-SFA	hqer__induced	True	16	-80.19050150944686	hqer__induced sampler prototype	4.0
	front41Data	HQER-SFA	hqer__induced	True	17	-60.704631323668906	hqer__induced sampler prototype	4.0
	front41Data	Quantile-SFA	hqer__induced	True	29	-49.72094095138175	hqer__induced sampler prototype	2.0
	front41Data	HQER-SFA	hqer__induced	True	7	-41.3550342826407	hqer__induced sampler prototype	2.0
	front41Data	Quantile-SFA	hqer__induced	True	19	-21.130019773994	hqer__induced sampler prototype	2.0
	front41Data	HQER-SFA	hqer__induced	True	34	-5.419926628926603	hqer__induced sampler prototype	4.0
	front41Data	Quantile-SFA	hqer__induced	True	20	-61.0881508076489	hqer__induced sampler prototype	4.0
	front41Data	HQER-SFA	hqer__induced	True	22	-49.4760723371118	hqer__induced sampler prototype	2.0
	front41Data	Quantile-SFA	hqer__induced	True	29	-42.87227063411837	hqer__induced sampler prototype	4.0
	front41Data	HQER-SFA	hqer__induced	True	20	-36.85787033234749	hqer__induced sampler prototype	2.0
	front41Data	Quantile-SFA	hqer__induced	True	11	-47.87347858370667	hqer__induced sampler prototype	2.0
	front41Data	HQER-SFA	hqer__induced	True	10	-26.62507561397168	hqer__induced sampler prototype	2.0
	front41Data	Quantile-SFA	hqer__induced	True	17	-42.80152467372366	hqer__induced sampler prototype	4.0
	front41Data	HQER-SFA	hqer__induced	True	10	-31.2311813396824	hqer__induced sampler prototype	2.0
	front41Data	Quantile-SFA	hqer__induced	True	14	-21.3266758652235	hqer__induced sampler prototype	4.0
	front41Data	HQER-SFA	hqer__induced	True	8	-10.291220499611718	hqer__induced sampler prototype	2.0
	front41Data	Quantile-SFA	hqer__induced	True	18	-40.25428023406333	hqer__induced sampler prototype	2.0
	front41Data	HQER-SFA	hqer__induced	True	16	-23.63752131410648	hqer__induced sampler prototype	2.0
	front41Data	Quantile-SFA	hqer__induced	True	21	-35.657228632066385	hqer__induced sampler prototype	4.0
	front41Data	HQER-SFA	hqer__induced	True	15	-21.831138660764992	hqer__induced sampler prototype	4.0
	front41Data	Quantile-SFA	hqer__induced	True	17	-0.8068935100492505	hqer__induced sampler prototype	4.0
	front41Data	HQER-SFA	hqer__induced	True	25	7.816834372484636	hqer__induced sampler prototype	4.0
	front41Data	Quantile-SFA	hqer__induced	True	43	-24.87443611813137	hqer__induced sampler prototype	4.0
	front41Data	HQER-SFA	hqer__induced	True	38	-17.857632645613236	hqer__induced sampler prototype	2.0
	front41Data	Quantile-SFA	hqer__induced	True	28	-40.41089764268489	hqer__induced sampler prototype	4.0
	front41Data	HQER-SFA	hqer__induced	True	43	-24.416994788575373	hqer__induced sampler prototype	4.0
	front41Data	Quantile-SFA	hqer__induced	True	36	-31.082587090450986	hqer__induced sampler prototype	2.0
	front41Data	HQER-SFA	hqer__induced	True	13	-17.868301908992034	hqer__induced sampler prototype	4.0
	front41Data	Quantile-SFA	hqer__induced	True	34	-59.3853908906719	hqer__induced sampler prototype	2.0
	front41Data	HQER-SFA	hqer__induced	True	11	-44.34480815132	hqer__induced sampler prototype	2.0
	front41Data	Quantile-SFA	hqer__induced	True	80	-28.29728081603897	hqer__induced sampler prototype	5.0
	front41Data	HQER-SFA	hqer__induced	True	17	-20.942431243830207	hqer__induced sampler prototype	2.0
	front41Data	Quantile-SFA	hqer__induced	True	27	-54.95640323664215	hqer__induced sampler prototype	2.0
	front41Data	HQER-SFA	hqer__induced	True	11	-39.65176220214674	hqer__induced sampler prototype	2.0
	front41Data	Quantile-SFA	hqer__induced	True	37	-49.6300388662786	hqer__induced sampler prototype	4.0
	front41Data	HQER-SFA	hqer__induced	True	11	-33.474489925972264	hqer__induced sampler prototype	4.0
	front41Data	Quantile-SFA	hqer__induced	True	29	-58.77800547850798	hqer__induced sampler prototype	4.0
	front41Data	HQER-SFA	hqer__induced	True	14	-54.24281303464463	hqer__induced sampler prototype	2.0
	front41Data	Quantile-SFA	hqer__induced	True	32	-49.15251135500189	hqer__induced sampler prototype	4.0
	front41Data	HQER-SFA	hqer__induced	True	23	-29.98668347459981	hqer__induced sampler prototype	4.0
	front41Data	Quantile-SFA	hqer__induced	True	39	-52.29136986250462	hqer__induced sampler prototype	2.0
	front41Data	HQER-SFA	hqer__induced	True	28	-39.95567340022146	hqer__induced sampler prototype	2.0
	front41Data	Quantile-SFA	hqer__induced	True	33	-47.23138981460573	hqer__induced sampler prototype	2.0
	front41Data	HQER-SFA	hqer__induced	True	14	-36.63437203109952	hqer__induced sampler prototype	4.0
	front41Data	Quantile-SFA	hqer__induced	True	20	-38.05996508128472	hqer__induced sampler prototype	4.0
	front41Data	HQER-SFA	hqer__induced	True	12	-24.210753448031973	hqer__induced sampler prototype	2.0
	front41Data	Quantile-SFA	hqer__induced	True	25	-66.32132641474703	hqer__induced sampler prototype	4.0
	front41Data	HQER-SFA	hqer__induced	True	18	-52.27448588467615	hqer__induced sampler prototype	4.0
	front41Data	Quantile-SFA	hqer__induced	True	31	-70.06743648943045	hqer__induced sampler prototype	2.0
	front41Data	HQER-SFA	hqer__induced	True	24	-46.96266253532333	hqer__induced sampler prototype	2.0
	front41Data	Quantile-SFA	hqer__induced	True	28	-45.2006448273288	hqer__induced sampler prototype	2.0
	front41Data	HQER-SFA	hqer__induced	True	16	-30.84918254375698	hqer__induced sampler prototype	4.0
	front41Data	Quantile-SFA	hqer__induced	True	31	-27.13705913890367	hqer__induced sampler prototype	4.0
	front41Data	HQER-SFA	hqer__induced	True	43	-14.690928574561912	hqer__induced sampler prototype	2.0
	front41Data	Quantile-SFA	hqer__induced	True	49	-38.38128888960483	hqer__induced sampler prototype	4.0
	front41Data	HQER-SFA	hqer__induced	True	12	-26.53095851203177	hqer__induced sampler prototype	4.0
	front41Data	Quantile-SFA	hqer__induced	True	30	-38.853647293965	hqer__induced sampler prototype	2.0
	front41Data	HQER-SFA	hqer__induced	True	12	-22.70159653211773	hqer__induced sampler prototype	4.0
	front41Data	Quantile-SFA	hqer__induced	True	11	-66.65585805323383	hqer__induced sampler prototype	4.0
	front41Data	HQER-SFA	hqer__induced	True	28	-52.41578201552608	hqer__induced sampler prototype	4.0

experiment	dataset	model	scenario	converged	n_iter	loglik	warning_message	best_start_id
	front41Data	Quantile-SFA	hqer_induced	True	29	-67.10923809936288	hqer_induced sampler prototype	4.0
	front41Data	HQER-SFA	hqer_induced	True	11	-52.39730109908433	hqer_induced sampler prototype	2.0
	front41Data	Quantile-SFA	hqer_induced	True	18	-57.90338160195488	hqer_induced sampler prototype	4.0
	front41Data	HQER-SFA	hqer_induced	True	29	-38.11383160297136	hqer_induced sampler prototype	4.0
	front41Data	Quantile-SFA	hqer_induced	True	38	-43.1237838495435	hqer_induced sampler prototype	2.0
	front41Data	HQER-SFA	hqer_induced	True	17	-27.760008374433298	hqer_induced sampler prototype	2.0
	front41Data	Quantile-SFA	hqer_induced	True	27	-49.54119394955293	hqer_induced sampler prototype	4.0
	front41Data	HQER-SFA	hqer_induced	True	22	-42.298437669983926	hqer_induced sampler prototype	4.0
	front41Data	Quantile-SFA	hqer_induced	True	38	-35.48824121339939	hqer_induced sampler prototype	2.0
	front41Data	HQER-SFA	hqer_induced	True	12	-22.57377510630576	hqer_induced sampler prototype	2.0
	front41Data	Quantile-SFA	hqer_induced	True	26	-51.26992295326496	hqer_induced sampler prototype	4.0
	front41Data	HQER-SFA	hqer_induced	True	23	-33.121039418422725	hqer_induced sampler prototype	4.0
	front41Data	Quantile-SFA	hqer_induced	True	32	-28.63628235545079	hqer_induced sampler prototype	2.0
	front41Data	HQER-SFA	hqer_induced	True	12	-15.012916887631231	hqer_induced sampler prototype	2.0
	front41Data	Quantile-SFA	hqer_induced	True	34	-85.51245650423239	hqer_induced sampler prototype	2.0
	front41Data	HQER-SFA	hqer_induced	True	12	-74.4298804287583	hqer_induced sampler prototype	4.0
	front41Data	Quantile-SFA	hqer_induced	True	23	-55.93767201336625	hqer_induced sampler prototype	4.0
	front41Data	HQER-SFA	hqer_induced	True	15	-35.83656299591646	hqer_induced sampler prototype	4.0
	front41Data	Quantile-SFA	hqer_induced	True	16	-26.3553786785318	hqer_induced sampler prototype	4.0
	front41Data	HQER-SFA	hqer_induced	True	15	-12.42016791012296	hqer_induced sampler prototype	2.0
	front41Data	Quantile-SFA	hqer_induced	True	45	-66.0618372282633	hqer_induced sampler prototype	2.0
	front41Data	HQER-SFA	hqer_induced	True	28	-47.2777547734848	hqer_induced sampler prototype	2.0
	front41Data	Quantile-SFA	hqer_induced	True	26	-60.80827795682398	hqer_induced sampler prototype	4.0
	front41Data	HQER-SFA	hqer_induced	True	18	-44.28487704814548	hqer_induced sampler prototype	2.0
	front41Data	Quantile-SFA	hqer_induced	True	27	-38.68095877774433	hqer_induced sampler prototype	4.0
	front41Data	HQER-SFA	hqer_induced	True	17	-22.99013036701248	hqer_induced sampler prototype	4.0
	front41Data	Quantile-SFA	hqer_induced	True	23	-50.8338317689406	hqer_induced sampler prototype	2.0
	front41Data	HQER-SFA	hqer_induced	True	24	-33.97697067189842	hqer_induced sampler prototype	4.0
	front41Data	Quantile-SFA	hqer_induced	True	20	-48.219225408748656	hqer_induced sampler prototype	4.0
	front41Data	HQER-SFA	hqer_induced	True	7	-31.85035248108141	hqer_induced sampler prototype	2.0
	front41Data	Quantile-SFA	hqer_induced	True	15	-58.177528332352935	hqer_induced sampler prototype	4.0
	front41Data	HQER-SFA	hqer_induced	True	14	-43.46364837402593	hqer_induced sampler prototype	2.0
	front41Data	Quantile-SFA	hqer_induced	True	14	-18.905431839103308	hqer_induced sampler prototype	4.0
	front41Data	HQER-SFA	hqer_induced	True	18	-10.928360717227475	hqer_induced sampler prototype	4.0
	front41Data	Quantile-SFA	hqer_induced	True	30	-25.54937969012556	hqer_induced sampler prototype	4.0
	front41Data	HQER-SFA	hqer_induced	True	19	-14.106825264506329	hqer_induced sampler prototype	2.0
	front41Data	Quantile-SFA	hqer_induced	True	16	-44.89583729446941	hqer_induced sampler prototype	4.0
	front41Data	HQER-SFA	hqer_induced	True	32	-23.70663211233586	hqer_induced sampler prototype	4.0
	front41Data	Quantile-SFA	hqer_induced	True	42	-63.876832416409954	hqer_induced sampler prototype	2.0
	front41Data	HQER-SFA	hqer_induced	True	9	-47.46474655793666	hqer_induced sampler prototype	4.0
	front41Data	Quantile-SFA	hqer_induced	True	16	-47.29291887016749	hqer_induced sampler prototype	2.0
	front41Data	HQER-SFA	hqer_induced	True	37	-36.46766143486768	hqer_induced sampler prototype	2.0
	front41Data	Quantile-SFA	hqer_induced	True	33	-53.05346176587869	hqer_induced sampler prototype	4.0
	front41Data	HQER-SFA	hqer_induced	True	13	-35.785183363073045	hqer_induced sampler prototype	2.0
	front41Data	Quantile-SFA	hqer_induced	True	36	-70.32598517025433	hqer_induced sampler prototype	2.0
	front41Data	HQER-SFA	hqer_induced	True	8	-52.62210726219833	hqer_induced sampler prototype	2.0
	front41Data	Quantile-SFA	hqer_induced	True	36	-16.985055617883944	hqer_induced sampler prototype	2.0
	front41Data	HQER-SFA	hqer_induced	True	20	-7.048412145168996	hqer_induced sampler prototype	4.0
	front41Data	Quantile-SFA	hqer_induced	True	14	-40.105053665599954	hqer_induced sampler prototype	2.0
	front41Data	HQER-SFA	hqer_induced	True	46	-28.5647446862878	hqer_induced sampler prototype	2.0
	front41Data	Quantile-SFA	hqer_induced	True	19	-56.20542845428572	hqer_induced sampler prototype	4.0
	front41Data	HQER-SFA	hqer_induced	True	14	-46.41464192196793	hqer_induced sampler prototype	2.0
	front41Data	Quantile-SFA	hqer_induced	True	23	-42.07061577613564	hqer_induced sampler prototype	2.0
	front41Data	HQER-SFA	hqer_induced	True	16	-25.65102250633368	hqer_induced sampler prototype	4.0
	front41Data	Quantile-SFA	hqer_induced	True	33	-47.08971247928375	hqer_induced sampler prototype	2.0
	front41Data	HQER-SFA	hqer_induced	True	30	-37.17233082982101	hqer_induced sampler prototype	4.0
	front41Data	Quantile-SFA	hqer_induced	True	17	-63.350470280031686	hqer_induced sampler prototype	2.0
	front41Data	HQER-SFA	hqer_induced	True	37	-48.86816654719589	hqer_induced sampler prototype	4.0
	front41Data	Quantile-SFA	hqer_induced	True	28	-54.04386062678199	hqer_induced sampler prototype	2.0
	front41Data	HQER-SFA	hqer_induced	True	16	-38.3824259090883	hqer_induced sampler prototype	4.0
	front41Data	Quantile-SFA	hqer_induced	True	15	-68.5189127867718	hqer_induced sampler prototype	4.0
	front41Data	HQER-SFA	hqer_induced	True	31	-52.64182645902533	hqer_induced sampler prototype	4.0
	front41Data	Quantile-SFA	hqer_induced	True	19	-17.04075939622426	hqer_induced sampler prototype	4.0
	front41Data	HQER-SFA	hqer_induced	True	34	-10.22154501397734	hqer_induced sampler prototype	4.0
	front41Data	Quantile-SFA	hqer_induced	True	34	-82.61072427286872	hqer_induced sampler prototype	4.0
	front41Data	HQER-SFA	hqer_induced	True	18	-68.28801848911417	hqer_induced sampler prototype	4.0
	front41Data	Quantile-SFA	hqer_induced	True	33	-39.19117901775597	hqer_induced sampler prototype	2.0
	front41Data	HQER-SFA	hqer_induced	True	29	-29.437507594488416	hqer_induced sampler prototype	4.0
	front41Data	Quantile-SFA	hqer_induced	True	28	-40.67480814958046	hqer_induced sampler prototype	4.0
	front41Data	HQER-SFA	hqer_induced	True	14	-33.966055641003415	hqer_induced sampler prototype	4.0
	front41Data	Quantile-SFA	hqer_induced	True	27	-63.58346759183823	hqer_induced sampler prototype	2.0
	front41Data	HQER-SFA	hqer_induced	True	44	-47.34372016064968	hqer_induced sampler prototype	2.0
	front41Data	Quantile-SFA	hqer_induced	True	24	-57.40636338998413	hqer_induced sampler prototype	4.0
	front41Data	HQER-SFA	hqer_induced	True	8	-47.09370545571044	hqer_induced sampler prototype	2.0
	front41Data	Quantile-SFA	hqer_induced	True	31	-60.55888451436793	hqer_induced sampler prototype	2.0
	front41Data	HQER-SFA	hqer_induced	True	18	-47.591441687004775	hqer_induced sampler prototype	4.0
	front41Data	Quantile-SFA	hqer_induced	True	24	-46.31362865452533	hqer_induced sampler prototype	2.0
	front41Data	HQER-SFA	hqer_induced	True	11	-35.67500844917998	hqer_induced sampler prototype	4.0
	front41Data	Quantile-SFA	hqer_induced	True	33	-15.087937073423058	hqer_induced sampler prototype	4.0
	front41Data	HQER-SFA	hqer_induced	True	10	-7.962290252447065	hqer_induced sampler prototype	2.0
	front41Data	Quantile-SFA	hqer_induced	True	25	-51.7550905546387	hqer_induced sampler prototype	4.0
	front41Data	HQER-SFA	hqer_induced	True	22	-33.103673955637376	hqer_induced sampler prototype	4.0
	front41Data	Quantile-SFA	hqer_induced	True	29	-63.42296082639369	hqer_induced sampler prototype	2.0
	front41Data	HQER-SFA	hqer_induced	True	14	-48.236221545765936	hqer_induced sampler prototype	4.0
	front41Data	Quantile-SFA	hqer_induced	True	33	-83.38302835956131	hqer_induced sampler prototype	2.0
	front41Data	HQER-SFA	hqer_induced	True	14	-69.32455476118105	hqer_induced sampler prototype	4.0
	front41Data	Quantile-SFA	hqer_induced	True	45	-58.91120948744594	hqer_induced sampler prototype	4.0
	front41Data	HQER-SFA	hqer_induced	True	64	-51.85043954381277	hqer_induced sampler prototype	4.0
	front41Data	Quantile-SFA	hqer_induced	True	25	-46.229038431300246	hqer_induced sampler prototype	4.0
	front41Data	HQER-SFA	hqer_induced	True	8	-35.079331523964974	hqer_induced sampler prototype	2.0
	front41Data	Quantile-SFA	hqer_induced	True	29	-22.02267774950583	hqer_induced sampler prototype	2.0
	front41Data	HQER-SFA	hqer_induced	True	21	-12.441602770340513	hqer_induced sampler prototype	2.0
	front41Data	Quantile-SFA	hqer_induced	True	45	-57.10137042190435	hqer_induced sampler prototype	2.0
	front41Data	HQER-SFA	hqer_induced	True	15	-41.99138995148837	hqer_induced sampler prototype	2.0
	front41Data	Quantile-SFA	hqer_induced	True	28	-51.89537907509717	hqer_induced sampler prototype	2.0
	front41Data	HQER-SFA	hqer_induced	True	18	-39.3124578789656	hqer_induced sampler prototype	2.0
	front41Data	Quantile-SFA	hqer_induced	True	16	-78.02531632904928	hqer_induced sampler prototype	4.0
	front41Data	HQER-SFA	hqer_induced	True	28	-57.65477220715957	hqer_induced sampler prototype	4.0
	front41Data	Quantile-SFA	hqer_induced	True	15	-76.33699067583287	hqer_induced sampler prototype	2.0
	front41Data	HQER-SFA	hqer_induced	True	9	-62.729079312543725	hqer_induced sampler prototype	4.0
	front41Data	Quantile-SFA	hqer_induced	True	12	-68.52301987626801	hqer_induced sampler prototype	4.0
	front41Data	HQER-SFA	hqer_induced	True	9	-54.41180637909652	hqer_induced sampler prototype	2.0
	front41Data	Quantile-SFA	hqer_induced	True	47	-54.964830483570765	hqer_induced sampler prototype	4.0

experiment	dataset	model	scenario	converged	n_iter	loglik	warning_message	best_start_id
	front41Data	HQER-SFA	hqer_induced	True	32	-45.10938323960952	hqer_induced sampler prototype	4.0
	front41Data	Quantile-SFA	hqer_induced	True	26	-42.51125193122071	hqer_induced sampler prototype	2.0
	front41Data	HQER-SFA	hqer_induced	True	40	-36.18592519207289	hqer_induced sampler prototype	4.0
	front41Data	Quantile-SFA	hqer_induced	True	13	-52.634525063269145	hqer_induced sampler prototype	2.0
	front41Data	HQER-SFA	hqer_induced	True	11	-42.51832437155765	hqer_induced sampler prototype	2.0
	front41Data	Quantile-SFA	hqer_induced	True	30	-28.15264861335101	hqer_induced sampler prototype	2.0
	front41Data	HQER-SFA	hqer_induced	True	15	-20.44610615839996	hqer_induced sampler prototype	4.0
	front41Data	Quantile-SFA	hqer_induced	True	33	-37.18062040112137	hqer_induced sampler prototype	2.0
	front41Data	HQER-SFA	hqer_induced	True	7	-20.053545249452373	hqer_induced sampler prototype	2.0
	front41Data	Quantile-SFA	hqer_induced	True	33	-32.0512982943429	hqer_induced sampler prototype	2.0
	front41Data	HQER-SFA	hqer_induced	True	24	-19.554841607666773	hqer_induced sampler prototype	4.0
	front41Data	Quantile-SFA	hqer_induced	True	20	-26.755193276024496	hqer_induced sampler prototype	2.0
	front41Data	HQER-SFA	hqer_induced	True	16	-11.3271610989184	hqer_induced sampler prototype	4.0
	front41Data	Quantile-SFA	hqer_induced	True	17	-58.44308955344557	hqer_induced sampler prototype	2.0
	front41Data	HQER-SFA	hqer_induced	True	15	-43.37913698547493	hqer_induced sampler prototype	2.0
	front41Data	Quantile-SFA	hqer_induced	True	15	-17.270948600802228	hqer_induced sampler prototype	2.0
	front41Data	HQER-SFA	hqer_induced	True	29	-8.845527018688733	hqer_induced sampler prototype	2.0
	front41Data	Quantile-SFA	hqer_induced	True	20	-53.36061624834034	hqer_induced sampler prototype	2.0
	front41Data	HQER-SFA	hqer_induced	True	31	-43.318685258615375	hqer_induced sampler prototype	4.0
	front41Data	Quantile-SFA	hqer_induced	True	19	-58.649741882386245	hqer_induced sampler prototype	4.0
	front41Data	HQER-SFA	hqer_induced	True	11	-41.90604996210706	hqer_induced sampler prototype	2.0
	front41Data	Quantile-SFA	hqer_induced	True	36	-13.963343455690111	hqer_induced sampler prototype	4.0
	front41Data	HQER-SFA	hqer_induced	True	30	0.4717801256453473	hqer_induced sampler prototype	4.0
	front41Data	Quantile-SFA	hqer_induced	True	62	-44.04678077411573	hqer_induced sampler prototype	2.0
	front41Data	HQER-SFA	hqer_induced	True	20	-30.42728050385021	hqer_induced sampler prototype	2.0
	front41Data	Quantile-SFA	hqer_induced	True	29	-24.037699826983328	hqer_induced sampler prototype	2.0
	front41Data	HQER-SFA	hqer_induced	True	22	-14.75974880724442	hqer_induced sampler prototype	2.0
	front41Data	Quantile-SFA	hqer_induced	True	20	-60.78796901098026	hqer_induced sampler prototype	4.0
	front41Data	HQER-SFA	hqer_induced	True	10	-42.00004803701268	hqer_induced sampler prototype	2.0
	front41Data	Quantile-SFA	hqer_induced	True	24	-64.81050728967631	hqer_induced sampler prototype	2.0
	front41Data	HQER-SFA	hqer_induced	True	13	-48.18070553487948	hqer_induced sampler prototype	4.0
	front41Data	Quantile-SFA	hqer_induced	True	16	-31.43604430078565	hqer_induced sampler prototype	4.0
	front41Data	HQER-SFA	hqer_induced	True	29	-23.40761753592944	hqer_induced sampler prototype	4.0
	front41Data	Quantile-SFA	hqer_induced	True	25	-40.85213553584627	hqer_induced sampler prototype	4.0
	front41Data	HQER-SFA	hqer_induced	True	16	-19.4805304705555	hqer_induced sampler prototype	4.0
	front41Data	Quantile-SFA	hqer_induced	True	45	-43.32731253583445	hqer_induced sampler prototype	5.0
	front41Data	HQER-SFA	hqer_induced	True	23	-35.767925540161386	hqer_induced sampler prototype	2.0
	front41Data	Quantile-SFA	hqer_induced	True	35	-62.87657438317672	hqer_induced sampler prototype	2.0
	front41Data	HQER-SFA	hqer_induced	True	10	-49.66218467297611	hqer_induced sampler prototype	2.0
	front41Data	Quantile-SFA	hqer_induced	True	20	-47.09292773454598	hqer_induced sampler prototype	2.0
	front41Data	HQER-SFA	hqer_induced	True	13	-36.85854501734664	hqer_induced sampler prototype	4.0
	front41Data	Quantile-SFA	hqer_induced	True	18	-83.18811534369335	hqer_induced sampler prototype	4.0
	front41Data	HQER-SFA	hqer_induced	True	13	-70.82589699400239	hqer_induced sampler prototype	2.0
	front41Data	Quantile-SFA	hqer_induced	True	15	-46.12034497288465	hqer_induced sampler prototype	4.0
	front41Data	HQER-SFA	hqer_induced	True	52	-24.89597273138279	hqer_induced sampler prototype	2.0
	front41Data	Quantile-SFA	hqer_induced	True	38	-33.40459367018564	hqer_induced sampler prototype	2.0
	front41Data	HQER-SFA	hqer_induced	True	20	-23.1648333689118	hqer_induced sampler prototype	4.0
	front41Data	Quantile-SFA	hqer_induced	True	18	-48.47322210920443	hqer_induced sampler prototype	4.0
	front41Data	HQER-SFA	hqer_induced	True	27	-30.018774315703816	hqer_induced sampler prototype	4.0
	front41Data	Quantile-SFA	hqer_induced	True	14	-39.73900321771839	hqer_induced sampler prototype	4.0
	front41Data	HQER-SFA	hqer_induced	True	11	-28.70784036039865	hqer_induced sampler prototype	2.0
	front41Data	Quantile-SFA	hqer_induced	True	19	-58.6438312616352	hqer_induced sampler prototype	4.0
	front41Data	HQER-SFA	hqer_induced	True	14	-45.89398181901352	hqer_induced sampler prototype	4.0
	front41Data	Quantile-SFA	hqer_induced	True	22	-55.22700123563406	hqer_induced sampler prototype	4.0
	front41Data	HQER-SFA	hqer_induced	True	13	-34.48805172561598	hqer_induced sampler prototype	4.0
	front41Data	Quantile-SFA	hqer_induced	True	39	-44.03394494218162	hqer_induced sampler prototype	4.0
	front41Data	HQER-SFA	hqer_induced	True	24	-24.655427741744774	hqer_induced sampler prototype	2.0
	front41Data	Quantile-SFA	hqer_induced	True	13	-60.96808257482365	hqer_induced sampler prototype	4.0
	front41Data	HQER-SFA	hqer_induced	True	13	-46.76810473450051	hqer_induced sampler prototype	4.0
	front41Data	Quantile-SFA	hqer_induced	True	16	-50.28374465376335	hqer_induced sampler prototype	4.0
	front41Data	HQER-SFA	hqer_induced	True	28	-34.682148799813284	hqer_induced sampler prototype	4.0
	front41Data	Quantile-SFA	hqer_induced	True	20	-49.31878629693233	hqer_induced sampler prototype	4.0
	front41Data	HQER-SFA	hqer_induced	True	12	-39.48592488500124	hqer_induced sampler prototype	2.0
	front41Data	Quantile-SFA	hqer_induced	True	17	-51.35292921256452	hqer_induced sampler prototype	4.0
	front41Data	HQER-SFA	hqer_induced	True	8	-33.731444696713226	hqer_induced sampler prototype	2.0
	front41Data	Quantile-SFA	hqer_induced	True	28	-56.30288712189887	hqer_induced sampler prototype	4.0
	front41Data	HQER-SFA	hqer_induced	True	14	-43.67937807496233	hqer_induced sampler prototype	2.0
	front41Data	Quantile-SFA	hqer_induced	True	18	-36.27426574518268	hqer_induced sampler prototype	4.0
	front41Data	HQER-SFA	hqer_induced	True	14	-15.942592187038333	hqer_induced sampler prototype	4.0
	front41Data	Quantile-SFA	hqer_induced	True	17	-50.02999684785216	hqer_induced sampler prototype	4.0
	front41Data	HQER-SFA	hqer_induced	True	5	-34.06558925542656	hqer_induced sampler prototype	2.0
	front41Data	Quantile-SFA	hqer_induced	True	29	-47.73473425936176	hqer_induced sampler prototype	2.0
	front41Data	HQER-SFA	hqer_induced	True	14	-33.81210450877349	hqer_induced sampler prototype	4.0
	front41Data	Quantile-SFA	hqer_induced	True	23	-74.19571934478591	hqer_induced sampler prototype	2.0
	front41Data	HQER-SFA	hqer_induced	True	35	-58.444972580444144	hqer_induced sampler prototype	2.0
	front41Data	Quantile-SFA	hqer_induced	True	17	-60.67122445726112	hqer_induced sampler prototype	4.0
	front41Data	HQER-SFA	hqer_induced	True	39	-51.05927599370776	hqer_induced sampler prototype	4.0
	front41Data	Quantile-SFA	hqer_induced	True	32	-34.61682305155817	hqer_induced sampler prototype	4.0
	front41Data	HQER-SFA	hqer_induced	True	16	-16.980607091022943	hqer_induced sampler prototype	4.0
	front41Data	Quantile-SFA	hqer_induced	True	20	-14.080512194025886	hqer_induced sampler prototype	4.0
	front41Data	HQER-SFA	hqer_induced	True	9	-4.1656627134248	hqer_induced sampler prototype	2.0
	front41Data	Quantile-SFA	hqer_induced	True	12	-42.58762861829029	hqer_induced sampler prototype	4.0
	front41Data	HQER-SFA	hqer_induced	True	18	-21.987930597418888	hqer_induced sampler prototype	4.0
	front41Data	Quantile-SFA	hqer_induced	True	20	-10.239494567060198	hqer_induced sampler prototype	2.0
	front41Data	HQER-SFA	hqer_induced	True	10	-8.346543898177178	hqer_induced sampler prototype	2.0
	front41Data	Quantile-SFA	hqer_induced	True	17	-22.4596772044206	hqer_induced sampler prototype	4.0
	front41Data	HQER-SFA	hqer_induced	True	31	-9.905580506102552	hqer_induced sampler prototype	4.0
	front41Data	Quantile-SFA	hqer_induced	True	17	-95.6722112153882	hqer_induced sampler prototype	4.0
	front41Data	HQER-SFA	hqer_induced	True	25	-78.85672626464768	hqer_induced sampler prototype	4.0
	front41Data	Quantile-SFA	hqer_induced	True	17	-65.50475805174275	hqer_induced sampler prototype	4.0
	front41Data	HQER-SFA	hqer_induced	True	14	-47.98556782925522	hqer_induced sampler prototype	4.0
	front41Data	Quantile-SFA	hqer_induced	True	14	-69.0181728223865	hqer_induced sampler prototype	4.0
	front41Data	HQER-SFA	hqer_induced	True	12	-55.223011602470415	hqer_induced sampler prototype	2.0
	front41Data	Quantile-SFA	hqer_induced	True	29	-71.48540196115367	hqer_induced sampler prototype	4.0
	front41Data	HQER-SFA	hqer_induced	True	36	-42.32014182906927	hqer_induced sampler prototype	2.0
	front41Data	Quantile-SFA	hqer_induced	True	16	-79.09662118102757	hqer_induced sampler prototype	4.0
	front41Data	HQER-SFA	hqer_induced	True	11	-58.93340297250832	hqer_induced sampler prototype	4.0
	front41Data	Quantile-SFA	hqer_induced	True	25	-27.493805854801806	hqer_induced sampler prototype	4.0
	front41Data	HQER-SFA	hqer_induced	True	22	-11.64171816779072	hqer_induced sampler prototype	4.0
	front41Data	Quantile-SFA	hqer_induced	True	28	-29.544984037512087	hqer_induced sampler prototype	2.0
	front41Data	HQER-SFA	hqer_induced	True	36	-16.88278576940857	hqer_induced sampler prototype	2.0
	front41Data	Quantile-SFA	hqer_induced	True	18	-52.61832445271985	hqer_induced sampler prototype	4.0
	front41Data	HQER-SFA	hqer_induced	True	32	-36.54296163753049	hqer_induced sampler prototype	4.0

experiment	dataset	model	scenario	converged	n__iter	loglik	warning__message	best__start__id
	front41Data	Quantile-SFA	hqer__induced	True	39	-88.52104123018056	hqer__induced sampler prototype	2.0
	front41Data	HQER-SFA	hqer__induced	True	25	-70.38484836493302	hqer__induced sampler prototype	4.0
	front41Data	Quantile-SFA	hqer__induced	True	21	-72.61251990001148	hqer__induced sampler prototype	4.0
	front41Data	HQER-SFA	hqer__induced	True	19	-63.71801071358856	hqer__induced sampler prototype	2.0
	front41Data	Quantile-SFA	hqer__induced	True	43	-40.67366682593133	hqer__induced sampler prototype	2.0
	front41Data	HQER-SFA	hqer__induced	True	14	-29.39896687314276	hqer__induced sampler prototype	4.0
	front41Data	Quantile-SFA	hqer__induced	True	28	-35.69385078834786	hqer__induced sampler prototype	4.0
	front41Data	HQER-SFA	hqer__induced	True	38	-22.493798299262103	hqer__induced sampler prototype	4.0
	front41Data	Quantile-SFA	hqer__induced	True	48	-20.87188751911717	hqer__induced sampler prototype	2.0
	front41Data	HQER-SFA	hqer__induced	True	7	-11.883427346063293	hqer__induced sampler prototype	2.0
	front41Data	Quantile-SFA	hqer__induced	True	20	-46.66197246203993	hqer__induced sampler prototype	4.0
	front41Data	HQER-SFA	hqer__induced	True	18	-36.24096622664145	hqer__induced sampler prototype	4.0
	front41Data	Quantile-SFA	hqer__induced	True	42	-37.2076241774823	hqer__induced sampler prototype	4.0
	front41Data	HQER-SFA	hqer__induced	True	18	-22.55240895128682	hqer__induced sampler prototype	2.0
	front41Data	Quantile-SFA	hqer__induced	True	32	-47.87748732706879	hqer__induced sampler prototype	4.0
	front41Data	HQER-SFA	hqer__induced	True	30	-31.05893755018932	hqer__induced sampler prototype	4.0
	front41Data	Quantile-SFA	hqer__induced	True	27	-58.21825108106022	hqer__induced sampler prototype	2.0
	front41Data	HQER-SFA	hqer__induced	True	22	-40.63251789654349	hqer__induced sampler prototype	2.0
	front41Data	Quantile-SFA	hqer__induced	True	28	-36.3635236110114	hqer__induced sampler prototype	2.0
	front41Data	HQER-SFA	hqer__induced	True	13	-27.25943195620403	hqer__induced sampler prototype	2.0
	front41Data	Quantile-SFA	hqer__induced	True	19	-63.49257958146887	hqer__induced sampler prototype	4.0
	front41Data	HQER-SFA	hqer__induced	True	22	-51.335878116363446	hqer__induced sampler prototype	2.0
	front41Data	Quantile-SFA	hqer__induced	True	33	-72.80384187702136	hqer__induced sampler prototype	2.0
	front41Data	HQER-SFA	hqer__induced	True	12	-57.75352973963939	hqer__induced sampler prototype	4.0
	front41Data	Quantile-SFA	hqer__induced	True	31	-27.63723955320078	hqer__induced sampler prototype	4.0
	front41Data	HQER-SFA	hqer__induced	True	32	-19.019469425668525	hqer__induced sampler prototype	4.0
	front41Data	Quantile-SFA	hqer__induced	True	34	-64.62698662454918	hqer__induced sampler prototype	2.0
	front41Data	HQER-SFA	hqer__induced	True	10	-49.81754714713942	hqer__induced sampler prototype	4.0
	front41Data	Quantile-SFA	hqer__induced	True	18	-37.14618606895581	hqer__induced sampler prototype	2.0
	front41Data	HQER-SFA	hqer__induced	True	29	-24.275842082906333	hqer__induced sampler prototype	2.0
	front41Data	Quantile-SFA	hqer__induced	True	36	-44.22737340583587	hqer__induced sampler prototype	4.0
	front41Data	HQER-SFA	hqer__induced	True	15	-39.47865738683198	hqer__induced sampler prototype	2.0
	front41Data	Quantile-SFA	hqer__induced	True	29	-76.63563220689615	hqer__induced sampler prototype	2.0
	front41Data	HQER-SFA	hqer__induced	True	12	-64.39628229961629	hqer__induced sampler prototype	2.0
	front41Data	Quantile-SFA	hqer__induced	True	23	-29.71525766841821	hqer__induced sampler prototype	4.0
	front41Data	HQER-SFA	hqer__induced	True	9	-13.403995125196628	hqer__induced sampler prototype	2.0
	front41Data	Quantile-SFA	hqer__induced	True	19	-62.91659547744083	hqer__induced sampler prototype	2.0
	front41Data	HQER-SFA	hqer__induced	True	20	-43.05357209665088	hqer__induced sampler prototype	2.0
	front41Data	Quantile-SFA	hqer__induced	True	34	-56.695008754172285	hqer__induced sampler prototype	2.0
	front41Data	HQER-SFA	hqer__induced	True	19	-47.00383496366878	hqer__induced sampler prototype	4.0
	front41Data	Quantile-SFA	hqer__induced	True	16	-70.78416735253744	hqer__induced sampler prototype	4.0
	front41Data	HQER-SFA	hqer__induced	True	24	-63.48235685660676	hqer__induced sampler prototype	4.0
	front41Data	Quantile-SFA	hqer__induced	True	16	-40.46986205595392	hqer__induced sampler prototype	4.0
	front41Data	HQER-SFA	hqer__induced	True	6	-32.47392933884937	hqer__induced sampler prototype	2.0
	front41Data	Quantile-SFA	hqer__induced	True	25	-70.864416886124	hqer__induced sampler prototype	4.0
	front41Data	HQER-SFA	hqer__induced	True	12	-58.69357742893434	hqer__induced sampler prototype	2.0
	front41Data	Quantile-SFA	hqer__induced	True	18	-47.421472312263376	hqer__induced sampler prototype	2.0
	front41Data	HQER-SFA	hqer__induced	True	24	-29.321730133761083	hqer__induced sampler prototype	4.0
	front41Data	Quantile-SFA	hqer__induced	True	27	2.315589198519824	hqer__induced sampler prototype	4.0
	front41Data	HQER-SFA	hqer__induced	True	25	12.147535831843092	hqer__induced sampler prototype	2.0
	front41Data	Quantile-SFA	hqer__induced	True	13	-32.563401951843176	hqer__induced sampler prototype	2.0
	front41Data	HQER-SFA	hqer__induced	True	9	-21.58483123834589	hqer__induced sampler prototype	2.0
	front41Data	Quantile-SFA	hqer__induced	True	17	-54.93899827030252	hqer__induced sampler prototype	4.0
	front41Data	HQER-SFA	hqer__induced	True	56	-30.806554154012	hqer__induced sampler prototype	4.0
	front41Data	Quantile-SFA	hqer__induced	True	20	-52.86011779450878	hqer__induced sampler prototype	2.0
	front41Data	HQER-SFA	hqer__induced	True	52	-46.959173910143576	hqer__induced sampler prototype	4.0
	front41Data	Quantile-SFA	hqer__induced	True	30	-52.39859708425104	hqer__induced sampler prototype	2.0
	front41Data	HQER-SFA	hqer__induced	True	23	-42.36003623269043	hqer__induced sampler prototype	2.0
	front41Data	Quantile-SFA	hqer__induced	True	21	-36.1226342432042	hqer__induced sampler prototype	4.0
	front41Data	HQER-SFA	hqer__induced	True	10	-22.933506450398365	hqer__induced sampler prototype	2.0
	front41Data	Quantile-SFA	hqer__induced	True	19	-60.438652978775934	hqer__induced sampler prototype	4.0
	front41Data	HQER-SFA	hqer__induced	True	14	-45.18794167639486	hqer__induced sampler prototype	4.0
	front41Data	Quantile-SFA	hqer__induced	True	33	-36.15897878791141	hqer__induced sampler prototype	4.0
	front41Data	HQER-SFA	hqer__induced	True	37	-19.05295607508569	hqer__induced sampler prototype	4.0
	front41Data	Quantile-SFA	hqer__induced	True	11	-55.714020212956896	hqer__induced sampler prototype	2.0
	front41Data	HQER-SFA	hqer__induced	True	14	-40.90482258125424	hqer__induced sampler prototype	2.0
	front41Data	Quantile-SFA	hqer__induced	True	20	-62.46164219178523	hqer__induced sampler prototype	2.0
	front41Data	HQER-SFA	hqer__induced	True	11	-49.65359441638896	hqer__induced sampler prototype	2.0
	front41Data	Quantile-SFA	hqer__induced	True	25	-48.07485392474019	hqer__induced sampler prototype	2.0
	front41Data	HQER-SFA	hqer__induced	True	60	-45.6834367435532	hqer__induced sampler prototype	4.0
	front41Data	Quantile-SFA	hqer__induced	True	29	-19.79404255661301	hqer__induced sampler prototype	2.0
	front41Data	HQER-SFA	hqer__induced	True	34	-3.7306606990172817	hqer__induced sampler prototype	2.0
	front41Data	Quantile-SFA	hqer__induced	True	25	-54.6340932174503	hqer__induced sampler prototype	2.0
	front41Data	HQER-SFA	hqer__induced	True	19	-39.83313053197615	hqer__induced sampler prototype	2.0
	front41Data	Quantile-SFA	hqer__induced	True	20	-58.25958300043007	hqer__induced sampler prototype	2.0
	front41Data	HQER-SFA	hqer__induced	True	20	-43.45432253551267	hqer__induced sampler prototype	4.0
	front41Data	Quantile-SFA	hqer__induced	True	23	-44.418423389392046	hqer__induced sampler prototype	2.0
	front41Data	HQER-SFA	hqer__induced	True	15	-33.199366261997326	hqer__induced sampler prototype	4.0
	front41Data	Quantile-SFA	hqer__induced	True	22	-73.25706921931567	hqer__induced sampler prototype	4.0
	front41Data	HQER-SFA	hqer__induced	True	14	-57.07114515876948	hqer__induced sampler prototype	4.0
	front41Data	Quantile-SFA	hqer__induced	True	33	-71.03937927084036	hqer__induced sampler prototype	2.0
	front41Data	HQER-SFA	hqer__induced	True	13	-53.71640039710619	hqer__induced sampler prototype	4.0
	front41Data	Quantile-SFA	hqer__induced	True	24	-76.81661047229561	hqer__induced sampler prototype	2.0
	front41Data	HQER-SFA	hqer__induced	True	24	-59.12433281923322	hqer__induced sampler prototype	2.0
	front41Data	Quantile-SFA	hqer__induced	True	19	-21.02006194999484	hqer__induced sampler prototype	4.0
	front41Data	HQER-SFA	hqer__induced	True	44	-11.554706314797327	hqer__induced sampler prototype	4.0
	front41Data	Quantile-SFA	hqer__induced	True	15	-57.32253135076861	hqer__induced sampler prototype	4.0
	front41Data	HQER-SFA	hqer__induced	True	11	-45.336150776795705	hqer__induced sampler prototype	4.0
	front41Data	Quantile-SFA	hqer__induced	True	26	-32.652495842080725	hqer__induced sampler prototype	2.0
	front41Data	HQER-SFA	hqer__induced	True	15	-19.887673595672688	hqer__induced sampler prototype	2.0
	front41Data	Quantile-SFA	hqer__induced	True	18	-38.00428132923104	hqer__induced sampler prototype	4.0
	front41Data	HQER-SFA	hqer__induced	True	21	-28.309850296203173	hqer__induced sampler prototype	2.0
	front41Data	Quantile-SFA	hqer__induced	True	27	-38.58134556345577	hqer__induced sampler prototype	2.0
	front41Data	HQER-SFA	hqer__induced	True	16	-33.005514968088605	hqer__induced sampler prototype	2.0
	front41Data	Quantile-SFA	hqer__induced	True	30	-118.27150728975283	hqer__induced sampler prototype	2.0
	front41Data	HQER-SFA	hqer__induced	True	10	-107.84384899438946	hqer__induced sampler prototype	2.0
	front41Data	Quantile-SFA	hqer__induced	True	32	-16.263853242652868	hqer__induced sampler prototype	2.0
	front41Data	HQER-SFA	hqer__induced	True	42	-6.8611924345435575	hqer__induced sampler prototype	2.0
	front41Data	Quantile-SFA	hqer__induced	True	33	-15.73798920628032	hqer__induced sampler prototype	2.0
	front41Data	HQER-SFA	hqer__induced	True	18	-1.2069936393317011	hqer__induced sampler prototype	4.0
	front41Data	Quantile-SFA	hqer__induced	True	21	-57.15740572200903	hqer__induced sampler prototype	2.0
	front41Data	HQER-SFA	hqer__induced	True	13	-45.848249156008855	hqer__induced sampler prototype	4.0
	front41Data	Quantile-SFA	hqer__induced	True	25	-36.86295919844304	hqer__induced sampler prototype	2.0

experiment	dataset	model	scenario	converged	n__iter	loglik	warning__message	best__start__id
	front41Data	HQER-SFA	hqer__induced	True	46	-19.24398140634221	hqer__induced sampler prototype	2.0
	front41Data	Quantile-SFA	hqer__induced	True	44	-15.739837341477712	hqer__induced sampler prototype	4.0
	front41Data	HQER-SFA	hqer__induced	True	24	-7.254022187230714	hqer__induced sampler prototype	2.0
	front41Data	Quantile-SFA	hqer__induced	True	17	-48.60372761837829	hqer__induced sampler prototype	4.0
	front41Data	HQER-SFA	hqer__induced	True	14	-35.00305626826321	hqer__induced sampler prototype	4.0
	front41Data	Quantile-SFA	hqer__induced	True	30	-37.25421721927872	hqer__induced sampler prototype	2.0
	front41Data	HQER-SFA	hqer__induced	True	10	-18.359152631747435	hqer__induced sampler prototype	2.0
	front41Data	Quantile-SFA	hqer__induced	True	20	-60.96778783417653	hqer__induced sampler prototype	4.0
	front41Data	HQER-SFA	hqer__induced	True	9	-45.85328919193265	hqer__induced sampler prototype	2.0
	front41Data	Quantile-SFA	hqer__induced	True	31	-32.249885594707905	hqer__induced sampler prototype	2.0
	front41Data	HQER-SFA	hqer__induced	True	17	-24.914222560463685	hqer__induced sampler prototype	2.0
	front41Data	Quantile-SFA	hqer__induced	True	51	-45.54531384854332	hqer__induced sampler prototype	4.0
	front41Data	HQER-SFA	hqer__induced	True	11	-28.737193874757203	hqer__induced sampler prototype	4.0
	front41Data	Quantile-SFA	hqer__induced	True	19	-72.92926482288382	hqer__induced sampler prototype	4.0
	front41Data	HQER-SFA	hqer__induced	True	12	-57.968658253540056	hqer__induced sampler prototype	2.0
	front41Data	Quantile-SFA	hqer__induced	True	11	-22.957346781450745	hqer__induced sampler prototype	2.0
	front41Data	HQER-SFA	hqer__induced	True	33	-0.8535976312020295	hqer__induced sampler prototype	4.0
	front41Data	Quantile-SFA	hqer__induced	True	35	-42.531701051241626	hqer__induced sampler prototype	4.0
	front41Data	HQER-SFA	hqer__induced	True	29	-34.95722157986207	hqer__induced sampler prototype	4.0
	front41Data	Quantile-SFA	hqer__induced	True	23	-40.63046974152512	hqer__induced sampler prototype	4.0
	front41Data	HQER-SFA	hqer__induced	True	24	-28.2238433004067	hqer__induced sampler prototype	4.0
	front41Data	Quantile-SFA	hqer__induced	True	29	-38.43239890637864	hqer__induced sampler prototype	2.0
	front41Data	HQER-SFA	hqer__induced	True	37	-19.222268467666293	hqer__induced sampler prototype	2.0
	WDI__industry	Quantile-SFA	hqer__induced	True	39	-129.9928591170535	hqer__induced sampler prototype	4.0
	WDI__industry	HQER-SFA	hqer__induced	True	14	-129.96978327941434	hqer__induced sampler prototype	2.0
	WDI__industry	Quantile-SFA	hqer__induced	True	33	-127.893824845173	hqer__induced sampler prototype	4.0
	WDI__industry	HQER-SFA	hqer__induced	True	25	-127.87512108410282	hqer__induced sampler prototype	4.0
	WDI__industry	Quantile-SFA	hqer__induced	True	19	-119.80053191766594	hqer__induced sampler prototype	4.0
	WDI__industry	HQER-SFA	hqer__induced	True	18	-119.75783939475104	hqer__induced sampler prototype	4.0
	WDI__industry	Quantile-SFA	hqer__induced	True	24	-107.18606713168434	hqer__induced sampler prototype	4.0
	WDI__industry	HQER-SFA	hqer__induced	True	31	-107.17426691413752	hqer__induced sampler prototype	4.0
	WDI__industry	Quantile-SFA	hqer__induced	True	16	-153.28218477175798	hqer__induced sampler prototype	2.0
	WDI__industry	HQER-SFA	hqer__induced	True	22	-153.24341959010007	hqer__induced sampler prototype	2.0
	WDI__industry	Quantile-SFA	hqer__induced	True	17	-113.7603630255336	hqer__induced sampler prototype	2.0
	WDI__industry	HQER-SFA	hqer__induced	True	22	-113.73075535992004	hqer__induced sampler prototype	2.0
	WDI__industry	Quantile-SFA	hqer__induced	True	23	-154.32946599227068	hqer__induced sampler prototype	4.0
	WDI__industry	HQER-SFA	hqer__induced	True	22	-154.29120152322318	hqer__induced sampler prototype	4.0
	WDI__industry	Quantile-SFA	hqer__induced	True	20	-115.75779820711794	hqer__induced sampler prototype	4.0
	WDI__industry	HQER-SFA	hqer__induced	True	24	-115.70841981988514	hqer__induced sampler prototype	4.0
	WDI__industry	Quantile-SFA	hqer__induced	True	16	-166.47065641080508	hqer__induced sampler prototype	2.0
	WDI__industry	HQER-SFA	hqer__induced	True	16	-166.4109434945521	hqer__induced sampler prototype	2.0
	WDI__industry	Quantile-SFA	hqer__induced	True	28	-109.25547702689494	hqer__induced sampler prototype	2.0
	WDI__industry	HQER-SFA	hqer__induced	True	22	-109.2414511508713	hqer__induced sampler prototype	2.0
	WDI__industry	Quantile-SFA	hqer__induced	True	22	-133.44225257775878	hqer__induced sampler prototype	2.0
	WDI__industry	HQER-SFA	hqer__induced	True	24	-133.41380619750544	hqer__induced sampler prototype	2.0
	WDI__industry	Quantile-SFA	hqer__induced	True	19	-148.38168120430157	hqer__induced sampler prototype	4.0
	WDI__industry	HQER-SFA	hqer__induced	True	27	-148.32723326938543	hqer__induced sampler prototype	2.0
	WDI__industry	Quantile-SFA	hqer__induced	True	23	-122.06545827819556	hqer__induced sampler prototype	4.0
	WDI__industry	HQER-SFA	hqer__induced	True	21	-122.07595185586756	hqer__induced sampler prototype	2.0
	WDI__industry	Quantile-SFA	hqer__induced	True	38	-76.97916828884475	hqer__induced sampler prototype	4.0
	WDI__industry	HQER-SFA	hqer__induced	True	26	-76.95095915232758	hqer__induced sampler prototype	2.0
	WDI__industry	Quantile-SFA	hqer__induced	True	29	-150.66439713416605	hqer__induced sampler prototype	4.0
	WDI__industry	HQER-SFA	hqer__induced	True	28	-150.5916478304021	hqer__induced sampler prototype	4.0
	WDI__industry	Quantile-SFA	hqer__induced	True	26	-128.80657500514243	hqer__induced sampler prototype	4.0
	WDI__industry	HQER-SFA	hqer__induced	True	24	-128.7829182029786	hqer__induced sampler prototype	4.0
	WDI__industry	Quantile-SFA	hqer__induced	True	23	-161.20583876112374	hqer__induced sampler prototype	2.0
	WDI__industry	HQER-SFA	hqer__induced	True	22	-161.15423020845932	hqer__induced sampler prototype	2.0
	WDI__industry	Quantile-SFA	hqer__induced	True	21	-170.70922385616706	hqer__induced sampler prototype	4.0
	WDI__industry	HQER-SFA	hqer__induced	True	26	-170.66387612077034	hqer__induced sampler prototype	4.0
	WDI__industry	Quantile-SFA	hqer__induced	True	22	-122.40771722270122	hqer__induced sampler prototype	2.0
	WDI__industry	HQER-SFA	hqer__induced	True	29	-122.3553951344028	hqer__induced sampler prototype	4.0
	WDI__industry	Quantile-SFA	hqer__induced	True	27	-111.73607917313372	hqer__induced sampler prototype	4.0
	WDI__industry	HQER-SFA	hqer__induced	True	22	-111.7024874036936	hqer__induced sampler prototype	2.0
	WDI__industry	Quantile-SFA	hqer__induced	True	37	-158.00404600995302	hqer__induced sampler prototype	5.0
	WDI__industry	HQER-SFA	hqer__induced	True	33	-157.9479530472147	hqer__induced sampler prototype	4.0
	WDI__industry	Quantile-SFA	hqer__induced	True	18	-152.07380151419562	hqer__induced sampler prototype	2.0
	WDI__industry	HQER-SFA	hqer__induced	True	21	-152.03664393303185	hqer__induced sampler prototype	4.0
	WDI__industry	Quantile-SFA	hqer__induced	True	24	-148.1430445977781	hqer__induced sampler prototype	4.0
	WDI__industry	HQER-SFA	hqer__induced	True	30	-148.10730097538976	hqer__induced sampler prototype	2.0
	WDI__industry	Quantile-SFA	hqer__induced	True	16	-135.39403672081897	hqer__induced sampler prototype	2.0
	WDI__industry	HQER-SFA	hqer__induced	True	24	-135.3631547453719	hqer__induced sampler prototype	2.0
	WDI__industry	Quantile-SFA	hqer__induced	True	23	-132.2632294883568	hqer__induced sampler prototype	4.0
	WDI__industry	HQER-SFA	hqer__induced	True	18	-132.23889741128357	hqer__induced sampler prototype	4.0
	WDI__industry	Quantile-SFA	hqer__induced	True	34	-122.04310198671008	hqer__induced sampler prototype	4.0
	WDI__industry	HQER-SFA	hqer__induced	True	31	-121.9903478468044	hqer__induced sampler prototype	4.0
	WDI__industry	Quantile-SFA	hqer__induced	True	29	-128.9759337257002	hqer__induced sampler prototype	2.0
	WDI__industry	HQER-SFA	hqer__induced	True	39	-128.94870632826766	hqer__induced sampler prototype	4.0
	WDI__industry	Quantile-SFA	hqer__induced	True	32	-137.93629365075546	hqer__induced sampler prototype	5.0
	WDI__industry	HQER-SFA	hqer__induced	True	24	-137.8759312433172	hqer__induced sampler prototype	2.0
	WDI__industry	Quantile-SFA	hqer__induced	True	26	-148.4183480030466	hqer__induced sampler prototype	4.0
	WDI__industry	HQER-SFA	hqer__induced	True	18	-148.3868084485482	hqer__induced sampler prototype	2.0
	WDI__industry	Quantile-SFA	hqer__induced	True	25	-163.88996254245026	hqer__induced sampler prototype	4.0
	WDI__industry	HQER-SFA	hqer__induced	True	24	-163.84944411687857	hqer__induced sampler prototype	2.0
	WDI__industry	Quantile-SFA	hqer__induced	True	22	-141.45321718497038	hqer__induced sampler prototype	4.0
	WDI__industry	HQER-SFA	hqer__induced	True	19	-141.42413862969897	hqer__induced sampler prototype	2.0
	WDI__industry	Quantile-SFA	hqer__induced	True	23	-177.39686283126576	hqer__induced sampler prototype	2.0
	WDI__industry	HQER-SFA	hqer__induced	True	22	-177.36017438871664	hqer__induced sampler prototype	2.0
	WDI__industry	Quantile-SFA	hqer__induced	True	25	-155.33648907882315	hqer__induced sampler prototype	4.0
	WDI__industry	HQER-SFA	hqer__induced	True	30	-155.29750509704166	hqer__induced sampler prototype	2.0
	WDI__industry	Quantile-SFA	hqer__induced	True	14	-143.48190853966148	hqer__induced sampler prototype	4.0
	WDI__industry	HQER-SFA	hqer__induced	True	20	-143.4301334418109	hqer__induced sampler prototype	2.0
	WDI__industry	Quantile-SFA	hqer__induced	True	28	-132.2711728690618	hqer__induced sampler prototype	4.0
	WDI__industry	HQER-SFA	hqer__induced	True	26	-132.18149508044337	hqer__induced sampler prototype	4.0
	WDI__industry	Quantile-SFA	hqer__induced	True	32	-124.72417429767803	hqer__induced sampler prototype	4.0
	WDI__industry	HQER-SFA	hqer__induced	True	26	-124.6877242714224	hqer__induced sampler prototype	4.0
	WDI__industry	Quantile-SFA	hqer__induced	True	24	-140.2418684753555	hqer__induced sampler prototype	2.0
	WDI__industry	HQER-SFA	hqer__induced	True	15	-140.1929817365792	hqer__induced sampler prototype	4.0
	WDI__industry	Quantile-SFA	hqer__induced	True	21	-121.408339853467	hqer__induced sampler prototype	4.0
	WDI__industry	HQER-SFA	hqer__induced	True	19	-121.40734981708168	hqer__induced sampler prototype	2.0
	WDI__industry	Quantile-SFA	hqer__induced	True	29	-141.68991616129668	hqer__induced sampler prototype	4.0
	WDI__industry	HQER-SFA	hqer__induced	True	25	-141.64914510663527	hqer__induced sampler prototype	2.0
	WDI__industry	Quantile-SFA	hqer__induced	True	10	-124.74409453466944	hqer__induced sampler prototype	2.0
	WDI__industry	HQER-SFA	hqer__induced	True	26	-124.6788817870594	hqer__induced sampler prototype	4.0

experiment	dataset	model	scenario	converged	n_iter	loglik	warning_message	best_start_id
	WDI_industry	Quantile-SFA	hqer_induced	True	19	-150.29028915444832	hqer_induced sampler prototype	4.0
	WDI_industry	HQER-SFA	hqer_induced	True	21	-150.25329002207002	hqer_induced sampler prototype	4.0
	WDI_industry	Quantile-SFA	hqer_induced	True	31	-145.42026083013872	hqer_induced sampler prototype	4.0
	WDI_industry	HQER-SFA	hqer_induced	True	13	-145.3835341968868	hqer_induced sampler prototype	2.0
	WDI_industry	Quantile-SFA	hqer_induced	True	19	-161.15719696496524	hqer_induced sampler prototype	4.0
	WDI_industry	HQER-SFA	hqer_induced	True	21	-161.10051561504457	hqer_induced sampler prototype	2.0
	WDI_industry	Quantile-SFA	hqer_induced	True	22	-146.93456625993707	hqer_induced sampler prototype	4.0
	WDI_industry	HQER-SFA	hqer_induced	True	21	-146.87142609874385	hqer_induced sampler prototype	4.0
	WDI_industry	Quantile-SFA	hqer_induced	True	17	-158.15234591242483	hqer_induced sampler prototype	4.0
	WDI_industry	HQER-SFA	hqer_induced	True	21	-158.08792833728404	hqer_induced sampler prototype	2.0
	WDI_industry	Quantile-SFA	hqer_induced	True	21	-125.3942082905904	hqer_induced sampler prototype	2.0
	WDI_industry	HQER-SFA	hqer_induced	True	14	-125.38069193073488	hqer_induced sampler prototype	2.0
	WDI_industry	Quantile-SFA	hqer_induced	True	15	-150.317764051963	hqer_induced sampler prototype	2.0
	WDI_industry	HQER-SFA	hqer_induced	True	20	-150.27672684112457	hqer_induced sampler prototype	4.0
	WDI_industry	Quantile-SFA	hqer_induced	True	20	-167.53801397980791	hqer_induced sampler prototype	4.0
	WDI_industry	HQER-SFA	hqer_induced	True	24	-167.51955300212023	hqer_induced sampler prototype	4.0
	WDI_industry	Quantile-SFA	hqer_induced	True	21	-115.0327361467646	hqer_induced sampler prototype	2.0
	WDI_industry	HQER-SFA	hqer_induced	True	21	-114.98671790294333	hqer_induced sampler prototype	4.0
	WDI_industry	Quantile-SFA	hqer_induced	True	24	-130.32779839711145	hqer_induced sampler prototype	4.0
	WDI_industry	HQER-SFA	hqer_induced	True	18	-130.32931250533758	hqer_induced sampler prototype	4.0
	WDI_industry	Quantile-SFA	hqer_induced	True	26	-146.69566804247646	hqer_induced sampler prototype	4.0
	WDI_industry	HQER-SFA	hqer_induced	True	30	-146.69505086116916	hqer_induced sampler prototype	4.0
	WDI_industry	Quantile-SFA	hqer_induced	True	36	-139.1121522517688	hqer_induced sampler prototype	5.0
	WDI_industry	HQER-SFA	hqer_induced	True	7	-139.07228119503756	hqer_induced sampler prototype	2.0
	WDI_industry	Quantile-SFA	hqer_induced	True	17	-143.6394604609471	hqer_induced sampler prototype	2.0
	WDI_industry	HQER-SFA	hqer_induced	True	19	-143.57210615043024	hqer_induced sampler prototype	4.0
	WDI_industry	Quantile-SFA	hqer_induced	True	24	-122.552632514731	hqer_induced sampler prototype	4.0
	WDI_industry	HQER-SFA	hqer_induced	True	28	-122.51674899172914	hqer_induced sampler prototype	4.0
	WDI_industry	Quantile-SFA	hqer_induced	True	21	-145.3784835314068	hqer_induced sampler prototype	2.0
	WDI_industry	HQER-SFA	hqer_induced	True	27	-145.32406240192515	hqer_induced sampler prototype	4.0
	WDI_industry	Quantile-SFA	hqer_induced	True	24	-172.95378968017386	hqer_induced sampler prototype	4.0
	WDI_industry	HQER-SFA	hqer_induced	True	22	-172.91400556351368	hqer_induced sampler prototype	2.0
	WDI_industry	Quantile-SFA	hqer_induced	True	20	-160.95936267202026	hqer_induced sampler prototype	2.0
	WDI_industry	HQER-SFA	hqer_induced	True	15	-160.9214276266547	hqer_induced sampler prototype	4.0
	WDI_industry	Quantile-SFA	hqer_induced	True	25	-152.241073830237	hqer_induced sampler prototype	4.0
	WDI_industry	HQER-SFA	hqer_induced	True	20	-152.20299263176537	hqer_induced sampler prototype	2.0
	WDI_industry	Quantile-SFA	hqer_induced	True	27	-147.720865566686	hqer_induced sampler prototype	4.0
	WDI_industry	HQER-SFA	hqer_induced	True	28	-147.69531630706717	hqer_induced sampler prototype	4.0
	WDI_industry	Quantile-SFA	hqer_induced	True	38	-124.85495144989932	hqer_induced sampler prototype	4.0
	WDI_industry	HQER-SFA	hqer_induced	True	23	-124.7794659010957	hqer_induced sampler prototype	2.0
	WDI_industry	Quantile-SFA	hqer_induced	True	20	-131.4400055004091	hqer_induced sampler prototype	2.0
	WDI_industry	HQER-SFA	hqer_induced	True	25	-131.37120247918	hqer_induced sampler prototype	4.0
	WDI_industry	Quantile-SFA	hqer_induced	True	23	-138.96943944644408	hqer_induced sampler prototype	4.0
	WDI_industry	HQER-SFA	hqer_induced	True	26	-138.94642308999457	hqer_induced sampler prototype	4.0
	WDI_industry	Quantile-SFA	hqer_induced	True	21	-123.41959775955	hqer_induced sampler prototype	2.0
	WDI_industry	HQER-SFA	hqer_induced	True	21	-123.3702371834325	hqer_induced sampler prototype	4.0
	WDI_industry	Quantile-SFA	hqer_induced	True	14	-149.55216882314085	hqer_induced sampler prototype	2.0
	WDI_industry	HQER-SFA	hqer_induced	True	20	-149.5147374559685	hqer_induced sampler prototype	4.0
	WDI_industry	Quantile-SFA	hqer_induced	True	30	-161.8016278379097	hqer_induced sampler prototype	4.0
	WDI_industry	HQER-SFA	hqer_induced	True	18	-161.74338523302967	hqer_induced sampler prototype	4.0
	WDI_industry	Quantile-SFA	hqer_induced	True	14	-124.6739378484762	hqer_induced sampler prototype	2.0
	WDI_industry	HQER-SFA	hqer_induced	True	20	-124.60642621402894	hqer_induced sampler prototype	4.0
	WDI_industry	Quantile-SFA	hqer_induced	True	18	-141.63960412234928	hqer_induced sampler prototype	2.0
	WDI_industry	HQER-SFA	hqer_induced	True	16	-141.60767912930842	hqer_induced sampler prototype	2.0
	WDI_industry	Quantile-SFA	hqer_induced	True	15	-105.7947767012461	hqer_induced sampler prototype	2.0
	WDI_industry	HQER-SFA	hqer_induced	True	17	-105.75831906214763	hqer_induced sampler prototype	2.0
	WDI_industry	Quantile-SFA	hqer_induced	True	22	-114.05315824137556	hqer_induced sampler prototype	2.0
	WDI_industry	HQER-SFA	hqer_induced	True	14	-114.00707785700008	hqer_induced sampler prototype	2.0
	WDI_industry	Quantile-SFA	hqer_induced	True	22	-110.81336814151058	hqer_induced sampler prototype	4.0
	WDI_industry	HQER-SFA	hqer_induced	True	24	-110.80297125401084	hqer_induced sampler prototype	4.0
	WDI_industry	Quantile-SFA	hqer_induced	True	23	-159.38355482832884	hqer_induced sampler prototype	4.0
	WDI_industry	HQER-SFA	hqer_induced	True	23	-159.32554928256144	hqer_induced sampler prototype	4.0
	WDI_industry	Quantile-SFA	hqer_induced	True	27	-95.379655800427	hqer_induced sampler prototype	4.0
	WDI_industry	HQER-SFA	hqer_induced	True	24	-95.38392681410244	hqer_induced sampler prototype	4.0
	WDI_industry	Quantile-SFA	hqer_induced	True	34	-145.1225900219065	hqer_induced sampler prototype	4.0
	WDI_industry	HQER-SFA	hqer_induced	True	23	-145.07948261699477	hqer_induced sampler prototype	4.0
	WDI_industry	Quantile-SFA	hqer_induced	True	20	-93.63906284312264	hqer_induced sampler prototype	4.0
	WDI_industry	HQER-SFA	hqer_induced	True	24	-93.64469191993196	hqer_induced sampler prototype	2.0
	WDI_industry	Quantile-SFA	hqer_induced	True	23	-136.86598631548938	hqer_induced sampler prototype	4.0
	WDI_industry	HQER-SFA	hqer_induced	True	26	-136.82792958184777	hqer_induced sampler prototype	2.0
	WDI_industry	Quantile-SFA	hqer_induced	True	15	-145.81598427967486	hqer_induced sampler prototype	2.0
	WDI_industry	HQER-SFA	hqer_induced	True	12	-145.75824491193333	hqer_induced sampler prototype	2.0
	WDI_industry	Quantile-SFA	hqer_induced	True	18	-146.2917512977907	hqer_induced sampler prototype	2.0
	WDI_industry	HQER-SFA	hqer_induced	True	28	-146.2296009053615	hqer_induced sampler prototype	2.0
	WDI_industry	Quantile-SFA	hqer_induced	True	25	-169.26918430907574	hqer_induced sampler prototype	4.0
	WDI_industry	HQER-SFA	hqer_induced	True	21	-169.21726182154563	hqer_induced sampler prototype	4.0
	WDI_industry	Quantile-SFA	hqer_induced	True	36	-111.64026440722358	hqer_induced sampler prototype	4.0
	WDI_industry	HQER-SFA	hqer_induced	True	27	-111.59758951729356	hqer_induced sampler prototype	4.0
	WDI_industry	Quantile-SFA	hqer_induced	True	20	-132.25647503161426	hqer_induced sampler prototype	4.0
	WDI_industry	HQER-SFA	hqer_induced	True	27	-132.21878720145776	hqer_induced sampler prototype	2.0
	WDI_industry	Quantile-SFA	hqer_induced	True	19	-111.97632271599676	hqer_induced sampler prototype	2.0
	WDI_industry	HQER-SFA	hqer_induced	True	15	-111.95157518317836	hqer_induced sampler prototype	4.0
	WDI_industry	Quantile-SFA	hqer_induced	True	23	-129.9523400179546	hqer_induced sampler prototype	4.0
	WDI_industry	HQER-SFA	hqer_induced	True	38	-129.9005743806577	hqer_induced sampler prototype	5.0
	WDI_industry	Quantile-SFA	hqer_induced	True	26	-102.02868313865542	hqer_induced sampler prototype	4.0
	WDI_industry	HQER-SFA	hqer_induced	True	22	-101.99593831986854	hqer_induced sampler prototype	4.0
	WDI_industry	Quantile-SFA	hqer_induced	True	30	-118.09627543315564	hqer_induced sampler prototype	4.0
	WDI_industry	HQER-SFA	hqer_induced	True	23	-118.0650764651366	hqer_induced sampler prototype	2.0
	WDI_industry	Quantile-SFA	hqer_induced	True	20	-165.43522213768404	hqer_induced sampler prototype	2.0
	WDI_industry	HQER-SFA	hqer_induced	True	21	-165.41107572979934	hqer_induced sampler prototype	2.0
	WDI_industry	Quantile-SFA	hqer_induced	True	24	-145.50212408758608	hqer_induced sampler prototype	4.0
	WDI_industry	HQER-SFA	hqer_induced	True	23	-145.45634711091083	hqer_induced sampler prototype	4.0
	WDI_industry	Quantile-SFA	hqer_induced	True	15	-156.74681726759437	hqer_induced sampler prototype	4.0
	WDI_industry	HQER-SFA	hqer_induced	True	21	-156.6205040042176	hqer_induced sampler prototype	2.0
	WDI_industry	Quantile-SFA	hqer_induced	True	25	-109.4535945902982	hqer_induced sampler prototype	4.0
	WDI_industry	HQER-SFA	hqer_induced	True	21	-109.41312261971478	hqer_induced sampler prototype	2.0
	WDI_industry	Quantile-SFA	hqer_induced	True	26	-129.15956580222664	hqer_induced sampler prototype	4.0
	WDI_industry	HQER-SFA	hqer_induced	True	16	-129.110244123165	hqer_induced sampler prototype	2.0
	WDI_industry	Quantile-SFA	hqer_induced	True	20	-131.0147690513916	hqer_induced sampler prototype	4.0
	WDI_industry	HQER-SFA	hqer_induced	True	17	-130.98735963251104	hqer_induced sampler prototype	4.0
	WDI_industry	Quantile-SFA	hqer_induced	True	16	-127.97767608766324	hqer_induced sampler prototype	2.0
	WDI_industry	HQER-SFA	hqer_induced	True	24	-127.93167357849256	hqer_induced sampler prototype	4.0
	WDI_industry	Quantile-SFA	hqer_induced	True	21	-162.93811431128444	hqer_induced sampler prototype	2.0

experiment	dataset	model	scenario	converged	n_iter	loglik	warning_message	best_start_id
	WDI_industry	HQER-SFA	hqer_induced	True	27	-162.88001432717465	hqer_induced sampler prototype	2.0
	WDI_industry	Quantile-SFA	hqer_induced	True	38	-82.85755205484345	hqer_induced sampler prototype	5.0
	WDI_industry	HQER-SFA	hqer_induced	True	30	-82.83268472727002	hqer_induced sampler prototype	2.0
	WDI_industry	Quantile-SFA	hqer_induced	True	20	-169.93406808230296	hqer_induced sampler prototype	4.0
	WDI_industry	HQER-SFA	hqer_induced	True	17	-169.87870013073714	hqer_induced sampler prototype	4.0
	WDI_industry	Quantile-SFA	hqer_induced	True	25	-139.4853671474156	hqer_induced sampler prototype	4.0
	WDI_industry	HQER-SFA	hqer_induced	True	27	-139.49378733888705	hqer_induced sampler prototype	4.0
	WDI_industry	Quantile-SFA	hqer_induced	True	26	-151.97580842344354	hqer_induced sampler prototype	2.0
	WDI_industry	HQER-SFA	hqer_induced	True	25	-151.91286394240004	hqer_induced sampler prototype	2.0
	WDI_industry	Quantile-SFA	hqer_induced	True	24	-178.73517050865777	hqer_induced sampler prototype	4.0
	WDI_industry	HQER-SFA	hqer_induced	True	24	-178.70048495783573	hqer_induced sampler prototype	4.0
	WDI_industry	Quantile-SFA	hqer_induced	True	23	-162.2253409297499	hqer_induced sampler prototype	4.0
	WDI_industry	HQER-SFA	hqer_induced	True	28	-162.15596011017615	hqer_induced sampler prototype	4.0
	WDI_industry	Quantile-SFA	hqer_induced	True	22	-112.79389572256515	hqer_induced sampler prototype	2.0
	WDI_industry	HQER-SFA	hqer_induced	True	21	-112.78803764550244	hqer_induced sampler prototype	4.0
	WDI_industry	Quantile-SFA	hqer_induced	True	31	-126.68343058517776	hqer_induced sampler prototype	4.0
	WDI_industry	HQER-SFA	hqer_induced	True	34	-126.661816973749	hqer_induced sampler prototype	4.0
	WDI_industry	Quantile-SFA	hqer_induced	True	29	-151.5368378030206	hqer_induced sampler prototype	2.0
	WDI_industry	HQER-SFA	hqer_induced	True	25	-151.463605877438	hqer_induced sampler prototype	4.0
	WDI_industry	Quantile-SFA	hqer_induced	True	23	-91.49816415152682	hqer_induced sampler prototype	4.0
	WDI_industry	HQER-SFA	hqer_induced	True	25	-91.4726858207553	hqer_induced sampler prototype	2.0
	WDI_industry	Quantile-SFA	hqer_induced	True	24	-165.29243144569148	hqer_induced sampler prototype	4.0
	WDI_industry	HQER-SFA	hqer_induced	True	20	-165.24310450295468	hqer_induced sampler prototype	4.0
	WDI_industry	Quantile-SFA	hqer_induced	True	18	-119.73291338393756	hqer_induced sampler prototype	4.0
	WDI_industry	HQER-SFA	hqer_induced	True	20	-119.70889144733655	hqer_induced sampler prototype	4.0
	WDI_industry	Quantile-SFA	hqer_induced	True	21	-134.81279478557684	hqer_induced sampler prototype	4.0
	WDI_industry	HQER-SFA	hqer_induced	True	26	-134.76395985385236	hqer_induced sampler prototype	4.0
	WDI_industry	Quantile-SFA	hqer_induced	True	26	-132.49320606769402	hqer_induced sampler prototype	4.0
	WDI_industry	HQER-SFA	hqer_induced	True	31	-132.41039103420837	hqer_induced sampler prototype	4.0
	WDI_industry	Quantile-SFA	hqer_induced	True	37	-127.83423229242727	hqer_induced sampler prototype	2.0
	WDI_industry	HQER-SFA	hqer_induced	True	36	-127.6730202421025	hqer_induced sampler prototype	4.0
	WDI_industry	Quantile-SFA	hqer_induced	True	25	-100.50515756223946	hqer_induced sampler prototype	2.0
	WDI_industry	HQER-SFA	hqer_induced	True	21	-100.47904917392285	hqer_induced sampler prototype	4.0
	WDI_industry	Quantile-SFA	hqer_induced	True	23	-157.65635797866048	hqer_induced sampler prototype	4.0
	WDI_industry	HQER-SFA	hqer_induced	True	24	-157.62850735021817	hqer_induced sampler prototype	4.0
	WDI_industry	Quantile-SFA	hqer_induced	True	15	-92.58755839364424	hqer_induced sampler prototype	4.0
	WDI_industry	HQER-SFA	hqer_induced	True	41	-92.56684835851196	hqer_induced sampler prototype	5.0
	WDI_industry	Quantile-SFA	hqer_induced	True	22	-122.9787392896496	hqer_induced sampler prototype	2.0
	WDI_industry	HQER-SFA	hqer_induced	True	33	-122.93599493711308	hqer_induced sampler prototype	4.0
	WDI_industry	Quantile-SFA	hqer_induced	True	17	-137.09036209453154	hqer_induced sampler prototype	2.0
	WDI_industry	HQER-SFA	hqer_induced	True	21	-137.02656116408946	hqer_induced sampler prototype	2.0
	WDI_industry	Quantile-SFA	hqer_induced	True	20	-120.27357140335074	hqer_induced sampler prototype	4.0
	WDI_industry	HQER-SFA	hqer_induced	True	21	-120.22982926652172	hqer_induced sampler prototype	4.0
	WDI_industry	Quantile-SFA	hqer_induced	True	23	-135.5620552252771	hqer_induced sampler prototype	4.0
	WDI_industry	HQER-SFA	hqer_induced	True	15	-135.54169955056298	hqer_induced sampler prototype	2.0
	WDI_industry	Quantile-SFA	hqer_induced	True	24	-142.8105335766923	hqer_induced sampler prototype	2.0
	WDI_industry	HQER-SFA	hqer_induced	True	23	-142.78640882511692	hqer_induced sampler prototype	4.0
	WDI_industry	Quantile-SFA	hqer_induced	True	19	-155.4032915446069	hqer_induced sampler prototype	2.0
	WDI_industry	HQER-SFA	hqer_induced	True	24	-155.38523780080538	hqer_induced sampler prototype	4.0
	WDI_industry	Quantile-SFA	hqer_induced	True	22	-150.0829126129118	hqer_induced sampler prototype	4.0
	WDI_industry	HQER-SFA	hqer_induced	True	20	-150.01788155331468	hqer_induced sampler prototype	2.0
	WDI_industry	Quantile-SFA	hqer_induced	True	26	-102.91408962217808	hqer_induced sampler prototype	4.0
	WDI_industry	HQER-SFA	hqer_induced	True	22	-102.90993798521518	hqer_induced sampler prototype	4.0
	WDI_industry	Quantile-SFA	hqer_induced	True	19	-130.94361171876955	hqer_induced sampler prototype	4.0
	WDI_industry	HQER-SFA	hqer_induced	True	21	-130.91105225522625	hqer_induced sampler prototype	4.0
	WDI_industry	Quantile-SFA	hqer_induced	True	30	-141.15620701048965	hqer_induced sampler prototype	4.0
	WDI_industry	HQER-SFA	hqer_induced	True	32	-141.15147180045972	hqer_induced sampler prototype	4.0
	WDI_industry	Quantile-SFA	hqer_induced	True	23	-180.61321340435893	hqer_induced sampler prototype	4.0
	WDI_industry	HQER-SFA	hqer_induced	True	20	-180.5434978201349	hqer_induced sampler prototype	2.0
	WDI_industry	Quantile-SFA	hqer_induced	True	22	-107.69078354957686	hqer_induced sampler prototype	2.0
	WDI_industry	HQER-SFA	hqer_induced	True	23	-107.65795036335632	hqer_induced sampler prototype	4.0
	WDI_industry	Quantile-SFA	hqer_induced	True	30	-111.22699064680477	hqer_induced sampler prototype	4.0
	WDI_industry	HQER-SFA	hqer_induced	True	22	-111.19787626403848	hqer_induced sampler prototype	4.0
	WDI_industry	Quantile-SFA	hqer_induced	True	19	-142.5652424226367	hqer_induced sampler prototype	2.0
	WDI_industry	HQER-SFA	hqer_induced	True	19	-142.51653256644158	hqer_induced sampler prototype	4.0
	WDI_industry	Quantile-SFA	hqer_induced	True	13	-153.13731156770697	hqer_induced sampler prototype	2.0
	WDI_industry	HQER-SFA	hqer_induced	True	22	-153.09473322132322	hqer_induced sampler prototype	4.0
	WDI_industry	Quantile-SFA	hqer_induced	True	17	-135.30006185617867	hqer_induced sampler prototype	4.0
	WDI_industry	HQER-SFA	hqer_induced	True	13	-135.2389625382619	hqer_induced sampler prototype	2.0
	WDI_industry	Quantile-SFA	hqer_induced	True	21	-160.56736784738334	hqer_induced sampler prototype	2.0
	WDI_industry	HQER-SFA	hqer_induced	True	30	-160.5364739879319	hqer_induced sampler prototype	4.0
	WDI_industry	Quantile-SFA	hqer_induced	True	15	-125.14595278591254	hqer_induced sampler prototype	2.0
	WDI_industry	HQER-SFA	hqer_induced	True	28	-125.09095250934702	hqer_induced sampler prototype	4.0
	WDI_industry	Quantile-SFA	hqer_induced	True	22	-147.27258140027098	hqer_induced sampler prototype	4.0
	WDI_industry	HQER-SFA	hqer_induced	True	19	-147.27901573670638	hqer_induced sampler prototype	4.0
	WDI_industry	Quantile-SFA	hqer_induced	True	22	-110.0747353927075	hqer_induced sampler prototype	4.0
	WDI_industry	HQER-SFA	hqer_induced	True	22	-110.04396821283888	hqer_induced sampler prototype	4.0
	WDI_industry	Quantile-SFA	hqer_induced	True	28	-124.24423446394522	hqer_induced sampler prototype	4.0
	WDI_industry	HQER-SFA	hqer_induced	True	26	-124.38487633971476	hqer_induced sampler prototype	4.0
	WDI_industry	Quantile-SFA	hqer_induced	True	29	-142.35565193072185	hqer_induced sampler prototype	4.0
	WDI_industry	HQER-SFA	hqer_induced	True	27	-142.30277937432004	hqer_induced sampler prototype	2.0
	WDI_industry	Quantile-SFA	hqer_induced	True	10	-151.12028798439275	hqer_induced sampler prototype	2.0
	WDI_industry	HQER-SFA	hqer_induced	True	17	-151.0826990234988	hqer_induced sampler prototype	2.0
	WDI_industry	Quantile-SFA	hqer_induced	True	54	-148.4290006726754	hqer_induced sampler prototype	3.0
	WDI_industry	HQER-SFA	hqer_induced	True	23	-148.4681075377111	hqer_induced sampler prototype	4.0
	WDI_industry	Quantile-SFA	hqer_induced	True	10	-140.1575191623524	hqer_induced sampler prototype	2.0
	WDI_industry	HQER-SFA	hqer_induced	True	24	-140.0899852140488	hqer_induced sampler prototype	4.0
	WDI_industry	Quantile-SFA	hqer_induced	True	25	-145.3078722529789	hqer_induced sampler prototype	4.0
	WDI_industry	HQER-SFA	hqer_induced	True	32	-145.25862911859892	hqer_induced sampler prototype	4.0
	WDI_industry	Quantile-SFA	hqer_induced	True	23	-125.35195140324814	hqer_induced sampler prototype	4.0
	WDI_industry	HQER-SFA	hqer_induced	True	34	-125.31078211104104	hqer_induced sampler prototype	5.0
	WDI_industry	Quantile-SFA	hqer_induced	True	12	-110.02691385364685	hqer_induced sampler prototype	2.0
	WDI_industry	HQER-SFA	hqer_induced	True	10	-109.99322952516827	hqer_induced sampler prototype	2.0
	WDI_industry	Quantile-SFA	hqer_induced	True	12	-108.20327189172168	hqer_induced sampler prototype	4.0
	WDI_industry	HQER-SFA	hqer_induced	True	12	-108.158157652436	hqer_induced sampler prototype	4.0
	WDI_industry	Quantile-SFA	hqer_induced	True	19	-136.24529841924965	hqer_induced sampler prototype	2.0
	WDI_industry	HQER-SFA	hqer_induced	True	24	-136.18372989943563	hqer_induced sampler prototype	4.0
	WDI_industry	Quantile-SFA	hqer_induced	True	24	-140.91887545959327	hqer_induced sampler prototype	4.0
	WDI_industry	HQER-SFA	hqer_induced	True	24	-140.86775495390145	hqer_induced sampler prototype	4.0
	WDI_industry	Quantile-SFA	hqer_induced	True	25	-176.63609900843687	hqer_induced sampler prototype	4.0
	WDI_industry	HQER-SFA	hqer_induced	True	27	-176.57584382798615	hqer_induced sampler prototype	2.0
	WDI_industry	Quantile-SFA	hqer_induced	True	44	-158.83903583172653	hqer_induced sampler prototype	5.0
	WDI_industry	HQER-SFA	hqer_induced	True	14	-158.80681547144485	hqer_induced sampler prototype	2.0

experiment	dataset	model	scenario	converged	n__iter	loglik	warning__message	best__start__id
	WDI__industry	Quantile-SFA	hqer__induced	True	30	-103.11152577414238	hqer__induced sampler prototype	4.0
	WDI__industry	HQER-SFA	hqer__induced	True	20	-103.1134507600159	hqer__induced sampler prototype	2.0
	WDI__industry	Quantile-SFA	hqer__induced	True	15	-116.0514422276308	hqer__induced sampler prototype	4.0
	WDI__industry	HQER-SFA	hqer__induced	True	21	-116.02777899323524	hqer__induced sampler prototype	2.0
	WDI__industry	Quantile-SFA	hqer__induced	True	25	-147.50327662503472	hqer__induced sampler prototype	4.0
	WDI__industry	HQER-SFA	hqer__induced	True	19	-147.44922321359746	hqer__induced sampler prototype	2.0
	WDI__industry	Quantile-SFA	hqer__induced	True	47	-170.18477749974494	hqer__induced sampler prototype	5.0
	WDI__industry	HQER-SFA	hqer__induced	True	13	-170.14972430358824	hqer__induced sampler prototype	4.0
	WDI__industry	Quantile-SFA	hqer__induced	True	24	-113.56703299890933	hqer__induced sampler prototype	4.0
	WDI__industry	HQER-SFA	hqer__induced	True	29	-113.50297952169228	hqer__induced sampler prototype	4.0
	WDI__industry	Quantile-SFA	hqer__induced	True	21	-161.1477681007765	hqer__induced sampler prototype	4.0
	WDI__industry	HQER-SFA	hqer__induced	True	28	-161.08769268493035	hqer__induced sampler prototype	4.0
	WDI__industry	Quantile-SFA	hqer__induced	True	22	-162.06583626880655	hqer__induced sampler prototype	4.0
	WDI__industry	HQER-SFA	hqer__induced	True	19	-162.02145876516352	hqer__induced sampler prototype	4.0
	WDI__industry	Quantile-SFA	hqer__induced	True	23	-136.47259922959844	hqer__induced sampler prototype	4.0
	WDI__industry	HQER-SFA	hqer__induced	True	24	-136.44289191556615	hqer__induced sampler prototype	4.0
	WDI__industry	Quantile-SFA	hqer__induced	True	21	-139.2618084296165	hqer__induced sampler prototype	4.0
	WDI__industry	HQER-SFA	hqer__induced	True	21	-139.22586464293852	hqer__induced sampler prototype	4.0
	WDI__industry	Quantile-SFA	hqer__induced	True	21	-133.4045991935275	hqer__induced sampler prototype	4.0
	WDI__industry	HQER-SFA	hqer__induced	True	16	-133.3953505893905	hqer__induced sampler prototype	2.0
	WDI__industry	Quantile-SFA	hqer__induced	True	28	-154.99391156603022	hqer__induced sampler prototype	4.0
	WDI__industry	HQER-SFA	hqer__induced	True	18	-154.959849217321	hqer__induced sampler prototype	4.0
	WDI__industry	Quantile-SFA	hqer__induced	True	21	-142.85079813416496	hqer__induced sampler prototype	2.0
	WDI__industry	HQER-SFA	hqer__induced	True	19	-142.81531508534508	hqer__induced sampler prototype	2.0
	WDI__industry	Quantile-SFA	hqer__induced	True	13	-105.5325192325017	hqer__induced sampler prototype	4.0
	WDI__industry	HQER-SFA	hqer__induced	True	19	-105.50262775600356	hqer__induced sampler prototype	2.0
	WDI__industry	Quantile-SFA	hqer__induced	True	22	-163.02062274556022	hqer__induced sampler prototype	4.0
	WDI__industry	HQER-SFA	hqer__induced	True	26	-162.95062155568743	hqer__induced sampler prototype	4.0
	WDI__industry	Quantile-SFA	hqer__induced	True	31	-131.63671805241006	hqer__induced sampler prototype	2.0
	WDI__industry	HQER-SFA	hqer__induced	True	28	-131.5687229995365	hqer__induced sampler prototype	2.0
	WDI__industry	Quantile-SFA	hqer__induced	True	18	-134.66507636717503	hqer__induced sampler prototype	4.0
	WDI__industry	HQER-SFA	hqer__induced	True	23	-134.6322807430371	hqer__induced sampler prototype	4.0
	WDI__industry	Quantile-SFA	hqer__induced	True	29	-111.25891937355244	hqer__induced sampler prototype	4.0
	WDI__industry	HQER-SFA	hqer__induced	True	34	-111.27066052130706	hqer__induced sampler prototype	4.0
	WDI__industry	Quantile-SFA	hqer__induced	True	27	-127.26060793667251	hqer__induced sampler prototype	4.0
	WDI__industry	HQER-SFA	hqer__induced	True	20	-127.23741911856816	hqer__induced sampler prototype	2.0
	WDI__industry	Quantile-SFA	hqer__induced	True	25	-107.62760549694303	hqer__induced sampler prototype	2.0
	WDI__industry	HQER-SFA	hqer__induced	True	29	-107.57735909139268	hqer__induced sampler prototype	2.0
	WDI__industry	Quantile-SFA	hqer__induced	True	10	-144.99885494668425	hqer__induced sampler prototype	2.0
	WDI__industry	HQER-SFA	hqer__induced	True	14	-144.97351181009418	hqer__induced sampler prototype	2.0
	WDI__industry	Quantile-SFA	hqer__induced	True	26	-122.47849276769465	hqer__induced sampler prototype	4.0
	WDI__industry	HQER-SFA	hqer__induced	True	18	-122.48368110203592	hqer__induced sampler prototype	4.0
	WDI__industry	Quantile-SFA	hqer__induced	True	25	-111.55779602008346	hqer__induced sampler prototype	4.0
	WDI__industry	HQER-SFA	hqer__induced	True	21	-111.50259815293774	hqer__induced sampler prototype	2.0
	WDI__industry	Quantile-SFA	hqer__induced	True	23	-148.99793200904074	hqer__induced sampler prototype	4.0
	WDI__industry	HQER-SFA	hqer__induced	True	27	-148.9597989477766	hqer__induced sampler prototype	2.0
	WDI__industry	Quantile-SFA	hqer__induced	True	9	-117.2042407874956	hqer__induced sampler prototype	2.0
	WDI__industry	HQER-SFA	hqer__induced	True	11	-117.17818876941038	hqer__induced sampler prototype	2.0
	WDI__industry	Quantile-SFA	hqer__induced	True	18	-156.9246829380748	hqer__induced sampler prototype	4.0
	WDI__industry	HQER-SFA	hqer__induced	True	16	-156.88311380620016	hqer__induced sampler prototype	4.0
	WDI__industry	Quantile-SFA	hqer__induced	True	27	-157.66409713555882	hqer__induced sampler prototype	4.0
	WDI__industry	HQER-SFA	hqer__induced	True	31	-157.63977302014607	hqer__induced sampler prototype	4.0
	WDI__industry	Quantile-SFA	hqer__induced	True	19	-134.01955077611737	hqer__induced sampler prototype	2.0
	WDI__industry	HQER-SFA	hqer__induced	True	15	-133.9908018224233	hqer__induced sampler prototype	2.0
	WDI__industry	Quantile-SFA	hqer__induced	True	23	-132.53360578784046	hqer__induced sampler prototype	2.0
	WDI__industry	HQER-SFA	hqer__induced	True	30	-132.4817138821847	hqer__induced sampler prototype	4.0
	WDI__industry	Quantile-SFA	hqer__induced	True	24	-150.68437360951356	hqer__induced sampler prototype	4.0
	WDI__industry	HQER-SFA	hqer__induced	True	18	-150.6671711655802	hqer__induced sampler prototype	4.0
	WDI__industry	Quantile-SFA	hqer__induced	True	13	-111.14941513874376	hqer__induced sampler prototype	2.0
	WDI__industry	HQER-SFA	hqer__induced	True	14	-111.0963563850926	hqer__induced sampler prototype	2.0
	WDI__industry	Quantile-SFA	hqer__induced	True	12	-127.0278214649534	hqer__induced sampler prototype	2.0
	WDI__industry	HQER-SFA	hqer__induced	True	16	-127.00153870557548	hqer__induced sampler prototype	4.0
	WDI__industry	Quantile-SFA	hqer__induced	True	33	-135.63448436738705	hqer__induced sampler prototype	4.0
	WDI__industry	HQER-SFA	hqer__induced	True	27	-135.61546117456098	hqer__induced sampler prototype	4.0
	WDI__industry	Quantile-SFA	hqer__induced	True	34	-151.72482170776385	hqer__induced sampler prototype	4.0
	WDI__industry	HQER-SFA	hqer__induced	True	25	-151.6966395922585	hqer__induced sampler prototype	4.0
	WDI__industry	Quantile-SFA	hqer__induced	True	22	-102.23923808036142	hqer__induced sampler prototype	2.0
	WDI__industry	HQER-SFA	hqer__induced	True	16	-102.20671295016228	hqer__induced sampler prototype	2.0
	WDI__industry	Quantile-SFA	hqer__induced	True	20	-140.37666140573117	hqer__induced sampler prototype	4.0
	WDI__industry	HQER-SFA	hqer__induced	True	19	-140.36349543860285	hqer__induced sampler prototype	4.0
	WDI__industry	Quantile-SFA	hqer__induced	True	28	-140.97112906464832	hqer__induced sampler prototype	2.0
	WDI__industry	HQER-SFA	hqer__induced	True	24	-140.94603366678922	hqer__induced sampler prototype	4.0
	WDI__industry	Quantile-SFA	hqer__induced	True	13	-116.73458171774531	hqer__induced sampler prototype	2.0
	WDI__industry	HQER-SFA	hqer__induced	True	16	-116.6948775167894	hqer__induced sampler prototype	2.0
	WDI__industry	Quantile-SFA	hqer__induced	True	28	-124.60788568749965	hqer__induced sampler prototype	2.0
	WDI__industry	HQER-SFA	hqer__induced	True	28	-124.56216289797716	hqer__induced sampler prototype	2.0
	WDI__industry	Quantile-SFA	hqer__induced	True	15	-129.80995792614942	hqer__induced sampler prototype	2.0
	WDI__industry	HQER-SFA	hqer__induced	True	36	-129.73213260106454	hqer__induced sampler prototype	4.0
	WDI__industry	Quantile-SFA	hqer__induced	True	21	-127.26137764926862	hqer__induced sampler prototype	4.0
	WDI__industry	HQER-SFA	hqer__induced	True	27	-127.23129460383848	hqer__induced sampler prototype	2.0
	WDI__industry	Quantile-SFA	hqer__induced	True	28	-115.54775633815694	hqer__induced sampler prototype	2.0
	WDI__industry	HQER-SFA	hqer__induced	True	19	-115.5257816079899	hqer__induced sampler prototype	4.0
	WDI__industry	Quantile-SFA	hqer__induced	True	29	-147.92999283403535	hqer__induced sampler prototype	4.0
	WDI__industry	HQER-SFA	hqer__induced	True	14	-147.89679708617757	hqer__induced sampler prototype	2.0
	WDI__industry	Quantile-SFA	hqer__induced	True	26	-90.1240611512028	hqer__induced sampler prototype	4.0
	WDI__industry	HQER-SFA	hqer__induced	True	29	-90.06287548764746	hqer__induced sampler prototype	2.0
	WDI__industry	Quantile-SFA	hqer__induced	True	22	-158.74408273029582	hqer__induced sampler prototype	2.0
	WDI__industry	HQER-SFA	hqer__induced	True	24	-158.69244184724684	hqer__induced sampler prototype	4.0
	WDI__industry	Quantile-SFA	hqer__induced	True	29	-141.58056481937268	hqer__induced sampler prototype	4.0
	WDI__industry	HQER-SFA	hqer__induced	True	19	-141.53149313752877	hqer__induced sampler prototype	2.0
	WDI__industry	Quantile-SFA	hqer__induced	True	36	-149.44606750320884	hqer__induced sampler prototype	4.0
	WDI__industry	HQER-SFA	hqer__induced	True	25	-149.43560671751047	hqer__induced sampler prototype	4.0
	WDI__industry	Quantile-SFA	hqer__induced	True	30	-140.9952802190296	hqer__induced sampler prototype	4.0
	WDI__industry	HQER-SFA	hqer__induced	True	28	-140.92842526672564	hqer__induced sampler prototype	4.0
	WDI__industry	Quantile-SFA	hqer__induced	True	27	-134.33650089512133	hqer__induced sampler prototype	2.0
	WDI__industry	HQER-SFA	hqer__induced	True	30	-134.28799739020263	hqer__induced sampler prototype	4.0
	WDI__industry	Quantile-SFA	hqer__induced	True	22	-91.36714105721394	hqer__induced sampler prototype	4.0
	WDI__industry	HQER-SFA	hqer__induced	True	27	-91.36600258894453	hqer__induced sampler prototype	4.0
	WDI__industry	Quantile-SFA	hqer__induced	True	22	-149.62687754545573	hqer__induced sampler prototype	4.0
	WDI__industry	HQER-SFA	hqer__induced	True	24	-149.60272753346763	hqer__induced sampler prototype	4.0
	WDI__industry	Quantile-SFA	hqer__induced	True	15	-159.35111193025642	hqer__induced sampler prototype	2.0
	WDI__industry	HQER-SFA	hqer__induced	True	11	-159.3489966625206	hqer__induced sampler prototype	2.0
	WDI__industry	Quantile-SFA	hqer__induced	True	20	-144.90691865416147	hqer__induced sampler prototype	4.0

experiment	dataset	model	scenario	converged	n_iter	loglik	warning_message	best_start_id
	WDI_industry	HQER-SFA	hqer_induced	True	19	-144.85665982499015	hqer_induced sampler prototype	4.0
	WDI_industry	Quantile-SFA	hqer_induced	True	31	-144.15990125293524	hqer_induced sampler prototype	4.0
	WDI_industry	HQER-SFA	hqer_induced	True	15	-144.1213667793999	hqer_induced sampler prototype	2.0
	WDI_industry	Quantile-SFA	hqer_induced	True	23	-153.74838803373785	hqer_induced sampler prototype	4.0
	WDI_industry	HQER-SFA	hqer_induced	True	22	-153.70071072083346	hqer_induced sampler prototype	4.0
	WDI_industry	Quantile-SFA	hqer_induced	True	23	-142.8376166263579	hqer_induced sampler prototype	2.0
	WDI_industry	HQER-SFA	hqer_induced	True	23	-142.78789214977644	hqer_induced sampler prototype	4.0
	WDI_industry	Quantile-SFA	hqer_induced	True	21	-129.5477465893945	hqer_induced sampler prototype	2.0
	WDI_industry	HQER-SFA	hqer_induced	True	21	-129.51990319422873	hqer_induced sampler prototype	2.0
	WDI_industry	Quantile-SFA	hqer_induced	True	13	-166.22939167441984	hqer_induced sampler prototype	2.0
	WDI_industry	HQER-SFA	hqer_induced	True	14	-166.191468825187	hqer_induced sampler prototype	2.0
	WDI_industry	Quantile-SFA	hqer_induced	True	16	-510.5840556254932	hqer_induced sampler prototype	2.0
	WDI_industry	HQER-SFA	hqer_induced	True	16	-510.5042208724621	hqer_induced sampler prototype	2.0
	WDI_industry	Quantile-SFA	hqer_induced	True	23	-579.3083831029398	hqer_induced sampler prototype	4.0
	WDI_industry	HQER-SFA	hqer_induced	True	21	-579.1677099028489	hqer_induced sampler prototype	4.0
	WDI_industry	Quantile-SFA	hqer_induced	True	19	-549.1305929209922	hqer_induced sampler prototype	2.0
	WDI_industry	HQER-SFA	hqer_induced	True	18	-548.9869510192179	hqer_induced sampler prototype	2.0
	WDI_industry	Quantile-SFA	hqer_induced	True	18	-576.2113256682768	hqer_induced sampler prototype	4.0
	WDI_industry	HQER-SFA	hqer_induced	True	19	-575.9701989094544	hqer_induced sampler prototype	4.0
	WDI_industry	Quantile-SFA	hqer_induced	True	16	-599.5903758778193	hqer_induced sampler prototype	2.0
	WDI_industry	HQER-SFA	hqer_induced	True	19	-599.3730191072347	hqer_induced sampler prototype	2.0
	WDI_industry	Quantile-SFA	hqer_induced	True	14	-554.846046028853	hqer_induced sampler prototype	4.0
	WDI_industry	HQER-SFA	hqer_induced	True	14	-554.6432280450142	hqer_induced sampler prototype	4.0
	WDI_industry	Quantile-SFA	hqer_induced	True	16	-556.5284761101536	hqer_induced sampler prototype	4.0
	WDI_industry	HQER-SFA	hqer_induced	True	14	-556.3832607818451	hqer_induced sampler prototype	2.0
	WDI_industry	Quantile-SFA	hqer_induced	True	31	-460.7750262517802	hqer_induced sampler prototype	5.0
	WDI_industry	HQER-SFA	hqer_induced	True	23	-460.7183202895393	hqer_induced sampler prototype	4.0
	WDI_industry	Quantile-SFA	hqer_induced	True	15	-598.244549168162	hqer_induced sampler prototype	4.0
	WDI_industry	HQER-SFA	hqer_induced	True	18	-598.0510026975231	hqer_induced sampler prototype	4.0
	WDI_industry	Quantile-SFA	hqer_induced	True	30	-587.1754941946033	hqer_induced sampler prototype	5.0
	WDI_industry	HQER-SFA	hqer_induced	True	18	-587.0586917385945	hqer_induced sampler prototype	2.0
	WDI_industry	Quantile-SFA	hqer_induced	True	14	-537.5681015473958	hqer_induced sampler prototype	2.0
	WDI_industry	HQER-SFA	hqer_induced	True	9	-537.3826318360439	hqer_induced sampler prototype	2.0
	WDI_industry	Quantile-SFA	hqer_induced	True	19	-522.6908529014887	hqer_induced sampler prototype	2.0
	WDI_industry	HQER-SFA	hqer_induced	True	14	-522.6035665835038	hqer_induced sampler prototype	2.0
	WDI_industry	Quantile-SFA	hqer_induced	True	24	-577.787229151305	hqer_induced sampler prototype	2.0
	WDI_industry	HQER-SFA	hqer_induced	True	20	-577.6076275125265	hqer_induced sampler prototype	2.0
	WDI_industry	Quantile-SFA	hqer_induced	True	21	-553.4057475536347	hqer_induced sampler prototype	2.0
	WDI_industry	HQER-SFA	hqer_induced	True	18	-553.2892848272054	hqer_induced sampler prototype	2.0
	WDI_industry	Quantile-SFA	hqer_induced	True	20	-561.9460563015523	hqer_induced sampler prototype	2.0
	WDI_industry	HQER-SFA	hqer_induced	True	22	-561.8103390063679	hqer_induced sampler prototype	2.0
	WDI_industry	Quantile-SFA	hqer_induced	True	18	-572.1550319785464	hqer_induced sampler prototype	2.0
	WDI_industry	HQER-SFA	hqer_induced	True	22	-571.972384884195	hqer_induced sampler prototype	4.0
	WDI_industry	Quantile-SFA	hqer_induced	True	34	-521.655762056123	hqer_induced sampler prototype	5.0
	WDI_industry	HQER-SFA	hqer_induced	True	23	-521.5495944424322	hqer_induced sampler prototype	4.0
	WDI_industry	Quantile-SFA	hqer_induced	True	18	-529.1343293362439	hqer_induced sampler prototype	2.0
	WDI_industry	HQER-SFA	hqer_induced	True	15	-528.9317235823387	hqer_induced sampler prototype	2.0
	WDI_industry	Quantile-SFA	hqer_induced	True	25	-505.2596152028393	hqer_induced sampler prototype	4.0
	WDI_industry	HQER-SFA	hqer_induced	True	24	-505.0664120299761	hqer_induced sampler prototype	2.0
	WDI_industry	Quantile-SFA	hqer_induced	True	17	-428.7620649872731	hqer_induced sampler prototype	4.0
	WDI_industry	HQER-SFA	hqer_induced	True	19	-428.5616828826861	hqer_induced sampler prototype	4.0
	WDI_industry	Quantile-SFA	hqer_induced	True	19	-571.5865007209663	hqer_induced sampler prototype	4.0
	WDI_industry	HQER-SFA	hqer_induced	True	15	-571.3844716079125	hqer_induced sampler prototype	2.0
	WDI_industry	Quantile-SFA	hqer_induced	True	19	-559.9960050628088	hqer_induced sampler prototype	4.0
	WDI_industry	HQER-SFA	hqer_induced	True	38	-559.7638932346231	hqer_induced sampler prototype	5.0
	WDI_industry	Quantile-SFA	hqer_induced	True	25	-419.1211777397813	hqer_induced sampler prototype	4.0
	WDI_industry	HQER-SFA	hqer_induced	True	22	-418.9920044811832	hqer_induced sampler prototype	2.0
	WDI_industry	Quantile-SFA	hqer_induced	True	25	-552.3671767567469	hqer_induced sampler prototype	4.0
	WDI_industry	HQER-SFA	hqer_induced	True	22	-552.205993338269	hqer_induced sampler prototype	2.0
	WDI_industry	Quantile-SFA	hqer_induced	True	17	-605.851311379892	hqer_induced sampler prototype	4.0
	WDI_industry	HQER-SFA	hqer_induced	True	16	-605.6439292234029	hqer_induced sampler prototype	4.0
	WDI_industry	Quantile-SFA	hqer_induced	True	21	-550.4805518931342	hqer_induced sampler prototype	4.0
	WDI_industry	HQER-SFA	hqer_induced	True	22	-550.2688404047198	hqer_induced sampler prototype	4.0
	WDI_industry	Quantile-SFA	hqer_induced	True	16	-514.0684616361832	hqer_induced sampler prototype	4.0
	WDI_industry	HQER-SFA	hqer_induced	True	21	-513.9240449970007	hqer_induced sampler prototype	4.0
	WDI_industry	Quantile-SFA	hqer_induced	True	14	-496.2137652386793	hqer_induced sampler prototype	2.0
	WDI_industry	HQER-SFA	hqer_induced	True	17	-496.0685499208416	hqer_induced sampler prototype	4.0
	WDI_industry	Quantile-SFA	hqer_induced	True	23	-600.160321723729	hqer_induced sampler prototype	4.0
	WDI_industry	HQER-SFA	hqer_induced	True	19	-599.9518738010586	hqer_induced sampler prototype	4.0
	WDI_industry	Quantile-SFA	hqer_induced	True	24	-567.3952394584871	hqer_induced sampler prototype	2.0
	WDI_industry	HQER-SFA	hqer_induced	True	16	-567.2334477794645	hqer_induced sampler prototype	2.0
	WDI_industry	Quantile-SFA	hqer_induced	True	13	-585.6146747666199	hqer_induced sampler prototype	2.0
	WDI_industry	HQER-SFA	hqer_induced	True	17	-585.43466636541	hqer_induced sampler prototype	2.0
	WDI_industry	Quantile-SFA	hqer_induced	True	20	-547.7379662673036	hqer_induced sampler prototype	2.0
	WDI_industry	HQER-SFA	hqer_induced	True	20	-547.618421845435	hqer_induced sampler prototype	2.0
	WDI_industry	Quantile-SFA	hqer_induced	True	16	-520.6836250388087	hqer_induced sampler prototype	2.0
	WDI_industry	HQER-SFA	hqer_induced	True	22	-520.5940404020233	hqer_induced sampler prototype	4.0
	WDI_industry	Quantile-SFA	hqer_induced	True	23	-575.4326134881026	hqer_induced sampler prototype	4.0
	WDI_industry	HQER-SFA	hqer_induced	True	20	-575.2431409650436	hqer_induced sampler prototype	4.0
	WDI_industry	Quantile-SFA	hqer_induced	True	20	-518.0425724764896	hqer_induced sampler prototype	4.0
	WDI_industry	HQER-SFA	hqer_induced	True	20	-517.9672183746067	hqer_induced sampler prototype	4.0
	WDI_industry	Quantile-SFA	hqer_induced	True	14	-610.5856534639051	hqer_induced sampler prototype	2.0
	WDI_industry	HQER-SFA	hqer_induced	True	24	-610.3749116413884	hqer_induced sampler prototype	4.0
	WDI_industry	Quantile-SFA	hqer_induced	True	18	-563.6083949136473	hqer_induced sampler prototype	4.0
	WDI_industry	HQER-SFA	hqer_induced	True	38	-563.4596583097077	hqer_induced sampler prototype	3.0
	WDI_industry	Quantile-SFA	hqer_induced	True	24	-544.8767872488811	hqer_induced sampler prototype	4.0
	WDI_industry	HQER-SFA	hqer_induced	True	20	-544.7124188297062	hqer_induced sampler prototype	4.0
	WDI_industry	Quantile-SFA	hqer_induced	True	22	-526.803934013646	hqer_induced sampler prototype	2.0
	WDI_industry	HQER-SFA	hqer_induced	True	21	-526.7411092608498	hqer_induced sampler prototype	2.0
	WDI_industry	Quantile-SFA	hqer_induced	True	23	-605.711445870635	hqer_induced sampler prototype	2.0
	WDI_industry	HQER-SFA	hqer_induced	True	18	-605.5601199761848	hqer_induced sampler prototype	2.0
	WDI_industry	Quantile-SFA	hqer_induced	True	18	-582.9517390279382	hqer_induced sampler prototype	4.0
	WDI_industry	HQER-SFA	hqer_induced	True	18	-582.8231299788564	hqer_induced sampler prototype	4.0
	WDI_industry	Quantile-SFA	hqer_induced	True	17	-578.5589454546239	hqer_induced sampler prototype	4.0
	WDI_industry	HQER-SFA	hqer_induced	True	18	-578.4127244811639	hqer_induced sampler prototype	4.0
	WDI_industry	Quantile-SFA	hqer_induced	True	15	-570.508658594883	hqer_induced sampler prototype	2.0
	WDI_industry	HQER-SFA	hqer_induced	True	17	-570.3220003218767	hqer_induced sampler prototype	4.0
	WDI_industry	Quantile-SFA	hqer_induced	True	11	-589.4276703562443	hqer_induced sampler prototype	4.0
	WDI_industry	HQER-SFA	hqer_induced	True	11	-589.2119722427832	hqer_induced sampler prototype	4.0
	WDI_industry	Quantile-SFA	hqer_induced	True	16	-546.8878220975653	hqer_induced sampler prototype	2.0
	WDI_industry	HQER-SFA	hqer_induced	True	21	-546.7556967668515	hqer_induced sampler prototype	2.0
	WDI_industry	Quantile-SFA	hqer_induced	True	16	-557.9100553844598	hqer_induced sampler prototype	4.0
	WDI_industry	HQER-SFA	hqer_induced	True	22	-557.691350736152	hqer_induced sampler prototype	2.0

experiment	dataset	model	scenario	converged	n__iter	loglik	warning__message	best__start__id
	WDI__industry	Quantile-SFA	hqer__induced	True	22	-488.4385874485951	hqer__induced sampler prototype	4.0
	WDI__industry	HQER-SFA	hqer__induced	True	22	-488.2510935661702	hqer__induced sampler prototype	2.0
	WDI__industry	Quantile-SFA	hqer__induced	True	18	-563.1272131842977	hqer__induced sampler prototype	4.0
	WDI__industry	HQER-SFA	hqer__induced	True	18	-562.9540568392268	hqer__induced sampler prototype	4.0
	WDI__industry	Quantile-SFA	hqer__induced	True	21	-558.7156109488867	hqer__induced sampler prototype	4.0
	WDI__industry	HQER-SFA	hqer__induced	True	16	-558.5340603273781	hqer__induced sampler prototype	4.0
	WDI__industry	Quantile-SFA	hqer__induced	True	13	-469.4748642739581	hqer__induced sampler prototype	2.0
	WDI__industry	HQER-SFA	hqer__induced	True	8	-469.3706995435712	hqer__induced sampler prototype	2.0
	WDI__industry	Quantile-SFA	hqer__induced	True	18	-505.6545707279015	hqer__induced sampler prototype	2.0
	WDI__industry	HQER-SFA	hqer__induced	True	18	-505.5024179726639	hqer__induced sampler prototype	2.0
	WDI__industry	Quantile-SFA	hqer__induced	True	28	-557.8964628225336	hqer__induced sampler prototype	4.0
	WDI__industry	HQER-SFA	hqer__induced	True	20	-557.7507244402163	hqer__induced sampler prototype	4.0
	WDI__industry	Quantile-SFA	hqer__induced	True	25	-511.472146204876	hqer__induced sampler prototype	2.0
	WDI__industry	HQER-SFA	hqer__induced	True	20	-511.306084976881	hqer__induced sampler prototype	2.0
	WDI__industry	Quantile-SFA	hqer__induced	True	19	-592.6093200440555	hqer__induced sampler prototype	4.0
	WDI__industry	HQER-SFA	hqer__induced	True	20	-592.4416610438675	hqer__induced sampler prototype	4.0
	WDI__industry	Quantile-SFA	hqer__induced	True	20	-519.872068768406	hqer__induced sampler prototype	2.0
	WDI__industry	HQER-SFA	hqer__induced	True	19	-519.7475303440287	hqer__induced sampler prototype	4.0
	WDI__industry	Quantile-SFA	hqer__induced	True	22	-510.8761420536253	hqer__induced sampler prototype	4.0
	WDI__industry	HQER-SFA	hqer__induced	True	22	-510.70427067629726	hqer__induced sampler prototype	4.0
	WDI__industry	Quantile-SFA	hqer__induced	True	19	-535.9540293683393	hqer__induced sampler prototype	2.0
	WDI__industry	HQER-SFA	hqer__induced	True	22	-535.7794994714894	hqer__induced sampler prototype	2.0
	WDI__industry	Quantile-SFA	hqer__induced	True	19	-578.4168862889372	hqer__induced sampler prototype	4.0
	WDI__industry	HQER-SFA	hqer__induced	True	20	-578.3292116073483	hqer__induced sampler prototype	4.0
	WDI__industry	Quantile-SFA	hqer__induced	True	17	-634.587988677881	hqer__induced sampler prototype	4.0
	WDI__industry	HQER-SFA	hqer__induced	True	23	-634.4268167649329	hqer__induced sampler prototype	4.0
	WDI__industry	Quantile-SFA	hqer__induced	True	19	-611.4809420289547	hqer__induced sampler prototype	2.0
	WDI__industry	HQER-SFA	hqer__induced	True	16	-611.3228287375182	hqer__induced sampler prototype	2.0
	WDI__industry	Quantile-SFA	hqer__induced	True	16	-537.4411467889743	hqer__induced sampler prototype	2.0
	WDI__industry	HQER-SFA	hqer__induced	True	17	-537.3333359746334	hqer__induced sampler prototype	2.0
	WDI__industry	Quantile-SFA	hqer__induced	True	18	-589.9354528506115	hqer__induced sampler prototype	4.0
	WDI__industry	HQER-SFA	hqer__induced	True	19	-589.7969513476492	hqer__induced sampler prototype	4.0
	WDI__industry	Quantile-SFA	hqer__induced	True	24	-560.9086015389562	hqer__induced sampler prototype	4.0
	WDI__industry	HQER-SFA	hqer__induced	True	25	-560.7818228910692	hqer__induced sampler prototype	4.0
	WDI__industry	Quantile-SFA	hqer__induced	True	29	-486.1048262225575	hqer__induced sampler prototype	4.0
	WDI__industry	HQER-SFA	hqer__induced	True	23	-486.03295190156086	hqer__induced sampler prototype	4.0
	WDI__industry	Quantile-SFA	hqer__induced	True	15	-551.2673944711231	hqer__induced sampler prototype	4.0
	WDI__industry	HQER-SFA	hqer__induced	True	17	-551.0952193737226	hqer__induced sampler prototype	4.0
	WDI__industry	Quantile-SFA	hqer__induced	True	17	-517.4286943509892	hqer__induced sampler prototype	2.0
	WDI__industry	HQER-SFA	hqer__induced	True	25	-517.316251818614	hqer__induced sampler prototype	4.0
	WDI__industry	Quantile-SFA	hqer__induced	True	39	-598.5331983849724	hqer__induced sampler prototype	5.0
	WDI__industry	HQER-SFA	hqer__induced	True	19	-598.3388317942718	hqer__induced sampler prototype	4.0
	WDI__industry	Quantile-SFA	hqer__induced	True	20	-558.959159975632	hqer__induced sampler prototype	2.0
	WDI__industry	HQER-SFA	hqer__induced	True	17	-558.8341125114165	hqer__induced sampler prototype	2.0
	WDI__industry	Quantile-SFA	hqer__induced	True	18	-487.4599372961921	hqer__induced sampler prototype	4.0
	WDI__industry	HQER-SFA	hqer__induced	True	19	-487.2878201636564	hqer__induced sampler prototype	2.0
	WDI__industry	Quantile-SFA	hqer__induced	True	25	-616.6039879563199	hqer__induced sampler prototype	4.0
	WDI__industry	HQER-SFA	hqer__induced	True	21	-616.4359705926497	hqer__induced sampler prototype	4.0
	WDI__industry	Quantile-SFA	hqer__induced	True	17	-534.395081909698	hqer__induced sampler prototype	4.0
	WDI__industry	HQER-SFA	hqer__induced	True	23	-534.2413230923487	hqer__induced sampler prototype	4.0
	WDI__industry	Quantile-SFA	hqer__induced	True	16	-603.0715495737563	hqer__induced sampler prototype	2.0
	WDI__industry	HQER-SFA	hqer__induced	True	15	-602.8987045884855	hqer__induced sampler prototype	2.0
	WDI__industry	Quantile-SFA	hqer__induced	True	21	-505.7824541414083	hqer__induced sampler prototype	4.0
	WDI__industry	HQER-SFA	hqer__induced	True	22	-505.64266049241417	hqer__induced sampler prototype	4.0
	WDI__industry	Quantile-SFA	hqer__induced	True	20	-513.7490011546035	hqer__induced sampler prototype	4.0
	WDI__industry	HQER-SFA	hqer__induced	True	22	-513.593819931575	hqer__induced sampler prototype	4.0
	WDI__industry	Quantile-SFA	hqer__induced	True	17	-506.3197806841382	hqer__induced sampler prototype	2.0
	WDI__industry	HQER-SFA	hqer__induced	True	17	-506.1563966689641	hqer__induced sampler prototype	4.0
	WDI__industry	Quantile-SFA	hqer__induced	True	19	-644.0254238597208	hqer__induced sampler prototype	2.0
	WDI__industry	HQER-SFA	hqer__induced	True	20	-643.8760464045313	hqer__induced sampler prototype	2.0
	WDI__industry	Quantile-SFA	hqer__induced	True	19	-505.3988367028399	hqer__induced sampler prototype	4.0
	WDI__industry	HQER-SFA	hqer__induced	True	22	-505.1997494780306	hqer__induced sampler prototype	4.0
	WDI__industry	Quantile-SFA	hqer__induced	True	20	-620.6488890002877	hqer__induced sampler prototype	4.0
	WDI__industry	HQER-SFA	hqer__induced	True	14	-620.4775034303659	hqer__induced sampler prototype	2.0
	WDI__industry	Quantile-SFA	hqer__induced	True	25	-468.112856386952	hqer__induced sampler prototype	4.0
	WDI__industry	HQER-SFA	hqer__induced	True	19	-467.96920074167446	hqer__induced sampler prototype	2.0
	WDI__industry	Quantile-SFA	hqer__induced	True	18	-573.8595982166952	hqer__induced sampler prototype	2.0
	WDI__industry	HQER-SFA	hqer__induced	True	15	-573.7210696324598	hqer__induced sampler prototype	4.0
	WDI__industry	Quantile-SFA	hqer__induced	True	18	-569.084931528204	hqer__induced sampler prototype	2.0
	WDI__industry	HQER-SFA	hqer__induced	True	12	-568.9058123732993	hqer__induced sampler prototype	2.0
	WDI__industry	Quantile-SFA	hqer__induced	True	15	-520.2070236765235	hqer__induced sampler prototype	2.0
	WDI__industry	HQER-SFA	hqer__induced	True	19	-520.1136965915351	hqer__induced sampler prototype	2.0
	WDI__industry	Quantile-SFA	hqer__induced	True	23	-469.6126508761917	hqer__induced sampler prototype	4.0
	WDI__industry	HQER-SFA	hqer__induced	True	17	-469.4583324762267	hqer__induced sampler prototype	2.0
	WDI__industry	Quantile-SFA	hqer__induced	True	20	-591.1719267075483	hqer__induced sampler prototype	4.0
	WDI__industry	HQER-SFA	hqer__induced	True	13	-591.0087602347512	hqer__induced sampler prototype	2.0
	WDI__industry	Quantile-SFA	hqer__induced	True	19	-662.4182241127935	hqer__induced sampler prototype	2.0
	WDI__industry	HQER-SFA	hqer__induced	True	22	-662.1704299587368	hqer__induced sampler prototype	4.0
	WDI__industry	Quantile-SFA	hqer__induced	True	17	-589.5093624075416	hqer__induced sampler prototype	4.0
	WDI__industry	HQER-SFA	hqer__induced	True	11	-589.3387703471584	hqer__induced sampler prototype	2.0
	WDI__industry	Quantile-SFA	hqer__induced	True	21	-499.1820400732478	hqer__induced sampler prototype	4.0
	WDI__industry	HQER-SFA	hqer__induced	True	20	-499.0703052377127	hqer__induced sampler prototype	4.0
	WDI__industry	Quantile-SFA	hqer__induced	True	17	-520.490027020094	hqer__induced sampler prototype	2.0
	WDI__industry	HQER-SFA	hqer__induced	True	16	-520.2869211699638	hqer__induced sampler prototype	2.0
	WDI__industry	Quantile-SFA	hqer__induced	True	30	-597.1246672737984	hqer__induced sampler prototype	4.0
	WDI__industry	HQER-SFA	hqer__induced	True	21	-596.9670771108899	hqer__induced sampler prototype	4.0
	WDI__industry	Quantile-SFA	hqer__induced	True	24	-557.9435856628636	hqer__induced sampler prototype	4.0
	WDI__industry	HQER-SFA	hqer__induced	True	17	-557.7785438694848	hqer__induced sampler prototype	2.0
	WDI__industry	Quantile-SFA	hqer__induced	True	24	-600.2693187367263	hqer__induced sampler prototype	4.0
	WDI__industry	HQER-SFA	hqer__induced	True	9	-600.16919023527	hqer__induced sampler prototype	2.0
	WDI__industry	Quantile-SFA	hqer__induced	True	16	-654.2001767652499	hqer__induced sampler prototype	2.0
	WDI__industry	HQER-SFA	hqer__induced	True	16	-654.0349034449198	hqer__induced sampler prototype	4.0
	WDI__industry	Quantile-SFA	hqer__induced	True	29	-526.1338778126342	hqer__induced sampler prototype	4.0
	WDI__industry	HQER-SFA	hqer__induced	True	17	-525.9206590729638	hqer__induced sampler prototype	2.0
	WDI__industry	Quantile-SFA	hqer__induced	True	16	-536.5656378465374	hqer__induced sampler prototype	2.0
	WDI__industry	HQER-SFA	hqer__induced	True	20	-536.3303162206087	hqer__induced sampler prototype	2.0
	WDI__industry	Quantile-SFA	hqer__induced	True	19	-567.6733071741038	hqer__induced sampler prototype	4.0
	WDI__industry	HQER-SFA	hqer__induced	True	10	-567.5434940681339	hqer__induced sampler prototype	2.0
	WDI__industry	Quantile-SFA	hqer__induced	True	21	-553.1022772443013	hqer__induced sampler prototype	4.0
	WDI__industry	HQER-SFA	hqer__induced	True	15	-552.8884685313423	hqer__induced sampler prototype	4.0
	WDI__industry	Quantile-SFA	hqer__induced	True	21	-527.2414867551545	hqer__induced sampler prototype	4.0
	WDI__industry	HQER-SFA	hqer__induced	True	11	-527.0716518001511	hqer__induced sampler prototype	2.0
	WDI__industry	Quantile-SFA	hqer__induced	True	27	-509.3423141546303	hqer__induced sampler prototype	2.0

experiment	dataset	model	scenario	converged	n__iter	loglik	warning__message	best__start__id
	WDI__industry	HQER-SFA	hqer__induced	True	23	-509.2155299233045	hqer__induced sampler prototype	2.0
	WDI__industry	Quantile-SFA	hqer__induced	True	17	-594.2372533789246	hqer__induced sampler prototype	2.0
	WDI__industry	HQER-SFA	hqer__induced	True	24	-594.0842232758578	hqer__induced sampler prototype	4.0
	WDI__industry	Quantile-SFA	hqer__induced	True	26	-535.2376007184123	hqer__induced sampler prototype	4.0
	WDI__industry	HQER-SFA	hqer__induced	True	11	-535.1177623344585	hqer__induced sampler prototype	2.0
	WDI__industry	Quantile-SFA	hqer__induced	True	19	-599.7516564967748	hqer__induced sampler prototype	2.0
	WDI__industry	HQER-SFA	hqer__induced	True	10	-599.6389550377871	hqer__induced sampler prototype	4.0
	WDI__industry	Quantile-SFA	hqer__induced	True	19	-665.6769013887048	hqer__induced sampler prototype	2.0
	WDI__industry	HQER-SFA	hqer__induced	True	16	-665.5274343134575	hqer__induced sampler prototype	2.0
	WDI__industry	Quantile-SFA	hqer__induced	True	14	-558.8847783440849	hqer__induced sampler prototype	2.0
	WDI__industry	HQER-SFA	hqer__induced	True	22	-558.7086107752843	hqer__induced sampler prototype	2.0
	WDI__industry	Quantile-SFA	hqer__induced	True	15	-580.6612225150956	hqer__induced sampler prototype	2.0
	WDI__industry	HQER-SFA	hqer__induced	True	16	-580.4266949628227	hqer__induced sampler prototype	2.0
	WDI__industry	Quantile-SFA	hqer__induced	True	24	-487.6334126508351	hqer__induced sampler prototype	4.0
	WDI__industry	HQER-SFA	hqer__induced	True	24	-487.5686699928695	hqer__induced sampler prototype	2.0
	WDI__industry	Quantile-SFA	hqer__induced	True	12	-445.5900418501022	hqer__induced sampler prototype	2.0
	WDI__industry	HQER-SFA	hqer__induced	True	11	-445.4162764960297	hqer__induced sampler prototype	2.0
	WDI__industry	Quantile-SFA	hqer__induced	True	19	-559.4894879745732	hqer__induced sampler prototype	2.0
	WDI__industry	HQER-SFA	hqer__induced	True	24	-559.313399663072	hqer__induced sampler prototype	4.0
	WDI__industry	Quantile-SFA	hqer__induced	True	16	-518.5381178462711	hqer__induced sampler prototype	2.0
	WDI__industry	HQER-SFA	hqer__induced	True	17	-518.404764064288	hqer__induced sampler prototype	2.0
	WDI__industry	Quantile-SFA	hqer__induced	True	22	-532.1765734042431	hqer__induced sampler prototype	2.0
	WDI__industry	HQER-SFA	hqer__induced	True	11	-532.0445440171652	hqer__induced sampler prototype	2.0
	WDI__industry	Quantile-SFA	hqer__induced	True	19	-510.3246606581183	hqer__induced sampler prototype	4.0
	WDI__industry	HQER-SFA	hqer__induced	True	16	-510.182555138413	hqer__induced sampler prototype	2.0
	WDI__industry	Quantile-SFA	hqer__induced	True	30	-592.8211392260262	hqer__induced sampler prototype	5.0
	WDI__industry	HQER-SFA	hqer__induced	True	22	-592.6404092459036	hqer__induced sampler prototype	4.0
	WDI__industry	Quantile-SFA	hqer__induced	True	19	-517.3087418392481	hqer__induced sampler prototype	4.0
	WDI__industry	HQER-SFA	hqer__induced	True	20	-517.2091989267028	hqer__induced sampler prototype	4.0
	WDI__industry	Quantile-SFA	hqer__induced	True	18	-467.2237768044156	hqer__induced sampler prototype	4.0
	WDI__industry	HQER-SFA	hqer__induced	True	18	-467.0509911582218	hqer__induced sampler prototype	2.0
	WDI__industry	Quantile-SFA	hqer__induced	True	17	-676.1866839453241	hqer__induced sampler prototype	2.0
	WDI__industry	HQER-SFA	hqer__induced	True	17	-676.0607650388666	hqer__induced sampler prototype	2.0
	WDI__industry	Quantile-SFA	hqer__induced	True	20	-611.8446583590141	hqer__induced sampler prototype	4.0
	WDI__industry	HQER-SFA	hqer__induced	True	23	-611.6920364006694	hqer__induced sampler prototype	4.0
	WDI__industry	Quantile-SFA	hqer__induced	True	23	-459.378077261208	hqer__induced sampler prototype	4.0
	WDI__industry	HQER-SFA	hqer__induced	True	24	-459.2805071464094	hqer__induced sampler prototype	4.0
	WDI__industry	Quantile-SFA	hqer__induced	True	21	-591.7046522667573	hqer__induced sampler prototype	4.0
	WDI__industry	HQER-SFA	hqer__induced	True	19	-591.5431742529825	hqer__induced sampler prototype	2.0
	WDI__industry	Quantile-SFA	hqer__induced	True	24	-598.9995453691123	hqer__induced sampler prototype	4.0
	WDI__industry	HQER-SFA	hqer__induced	True	22	-598.8361663822943	hqer__induced sampler prototype	4.0
	WDI__industry	Quantile-SFA	hqer__induced	True	21	-555.7901555787316	hqer__induced sampler prototype	2.0
	WDI__industry	HQER-SFA	hqer__induced	True	16	-555.708386279241	hqer__induced sampler prototype	2.0
	WDI__industry	Quantile-SFA	hqer__induced	True	21	-513.088462231059	hqer__induced sampler prototype	2.0
	WDI__industry	HQER-SFA	hqer__induced	True	18	-512.9613821356671	hqer__induced sampler prototype	4.0
	WDI__industry	Quantile-SFA	hqer__induced	True	16	-531.6348592139911	hqer__induced sampler prototype	2.0
	WDI__industry	HQER-SFA	hqer__induced	True	13	-531.5160818682734	hqer__induced sampler prototype	4.0
	WDI__industry	Quantile-SFA	hqer__induced	True	20	-542.3452418740546	hqer__induced sampler prototype	4.0
	WDI__industry	HQER-SFA	hqer__induced	True	17	-542.1869323676342	hqer__induced sampler prototype	2.0
	WDI__industry	Quantile-SFA	hqer__induced	True	17	-539.9284874446903	hqer__induced sampler prototype	4.0
	WDI__industry	HQER-SFA	hqer__induced	True	24	-539.7313042149835	hqer__induced sampler prototype	4.0
	WDI__industry	Quantile-SFA	hqer__induced	True	22	-518.5376779590135	hqer__induced sampler prototype	4.0
	WDI__industry	HQER-SFA	hqer__induced	True	19	-518.3824530552329	hqer__induced sampler prototype	4.0
	WDI__industry	Quantile-SFA	hqer__induced	True	16	-488.40796828295726	hqer__induced sampler prototype	4.0
	WDI__industry	HQER-SFA	hqer__induced	True	12	-488.2780744341189	hqer__induced sampler prototype	2.0
	WDI__industry	Quantile-SFA	hqer__induced	True	21	-570.4751081318246	hqer__induced sampler prototype	4.0
	WDI__industry	HQER-SFA	hqer__induced	True	20	-570.2744635696234	hqer__induced sampler prototype	4.0
	WDI__industry	Quantile-SFA	hqer__induced	True	20	-610.5030799510715	hqer__induced sampler prototype	4.0
	WDI__industry	HQER-SFA	hqer__induced	True	28	-610.326328811027	hqer__induced sampler prototype	4.0
	WDI__industry	Quantile-SFA	hqer__induced	True	22	-507.0608018803469	hqer__induced sampler prototype	4.0
	WDI__industry	HQER-SFA	hqer__induced	True	17	-506.9389443007249	hqer__induced sampler prototype	2.0
	WDI__industry	Quantile-SFA	hqer__induced	True	19	-581.9211028204734	hqer__induced sampler prototype	4.0
	WDI__industry	HQER-SFA	hqer__induced	True	35	-581.7411997235633	hqer__induced sampler prototype	5.0
	WDI__industry	Quantile-SFA	hqer__induced	True	32	-523.760064446571	hqer__induced sampler prototype	4.0
	WDI__industry	HQER-SFA	hqer__induced	True	17	-523.6125330181892	hqer__induced sampler prototype	2.0
	WDI__industry	Quantile-SFA	hqer__induced	True	19	-490.9132173041178	hqer__induced sampler prototype	4.0
	WDI__industry	HQER-SFA	hqer__induced	True	20	-490.7838011198298	hqer__induced sampler prototype	4.0
	WDI__industry	Quantile-SFA	hqer__induced	True	15	-538.1522748493314	hqer__induced sampler prototype	2.0
	WDI__industry	HQER-SFA	hqer__induced	True	18	-538.0305167848301	hqer__induced sampler prototype	2.0
	WDI__industry	Quantile-SFA	hqer__induced	True	26	-484.7626920934771	hqer__induced sampler prototype	4.0
	WDI__industry	HQER-SFA	hqer__induced	True	25	-484.65727139628046	hqer__induced sampler prototype	4.0
	WDI__industry	Quantile-SFA	hqer__induced	True	8	-572.7459022686382	hqer__induced sampler prototype	2.0
	WDI__industry	HQER-SFA	hqer__induced	True	25	-572.5785852321717	hqer__induced sampler prototype	4.0
	WDI__industry	Quantile-SFA	hqer__induced	True	22	-554.3415858352562	hqer__induced sampler prototype	2.0
	WDI__industry	HQER-SFA	hqer__induced	True	28	-554.1829176725595	hqer__induced sampler prototype	4.0
	WDI__industry	Quantile-SFA	hqer__induced	True	16	-500.2420807932602	hqer__induced sampler prototype	2.0
	WDI__industry	HQER-SFA	hqer__induced	True	25	-500.0947879219172	hqer__induced sampler prototype	4.0
	WDI__industry	Quantile-SFA	hqer__induced	True	18	-561.5547624253671	hqer__induced sampler prototype	4.0
	WDI__industry	HQER-SFA	hqer__induced	True	21	-561.380136684057	hqer__induced sampler prototype	2.0
	WDI__industry	Quantile-SFA	hqer__induced	True	21	-522.5212321707301	hqer__induced sampler prototype	2.0
	WDI__industry	HQER-SFA	hqer__induced	True	23	-522.3500825578735	hqer__induced sampler prototype	2.0
	WDI__industry	Quantile-SFA	hqer__induced	True	9	-496.1704645469639	hqer__induced sampler prototype	2.0
	WDI__industry	HQER-SFA	hqer__induced	True	25	-495.98130164859725	hqer__induced sampler prototype	4.0
	WDI__industry	Quantile-SFA	hqer__induced	True	28	-546.5884536120462	hqer__induced sampler prototype	4.0
	WDI__industry	HQER-SFA	hqer__induced	True	23	-546.4071799972564	hqer__induced sampler prototype	4.0
	WDI__industry	Quantile-SFA	hqer__induced	True	22	-545.0781858283794	hqer__induced sampler prototype	4.0
	WDI__industry	HQER-SFA	hqer__induced	True	22	-544.9082060433482	hqer__induced sampler prototype	4.0
	WDI__industry	Quantile-SFA	hqer__induced	True	21	-484.04369223068704	hqer__induced sampler prototype	4.0
	WDI__industry	HQER-SFA	hqer__induced	True	10	-483.86912626110734	hqer__induced sampler prototype	2.0
	WDI__industry	Quantile-SFA	hqer__induced	True	23	-548.0619787330713	hqer__induced sampler prototype	2.0
	WDI__industry	HQER-SFA	hqer__induced	True	18	-547.8691934075284	hqer__induced sampler prototype	4.0
	WDI__industry	Quantile-SFA	hqer__induced	True	18	-503.1099680490787	hqer__induced sampler prototype	4.0
	WDI__industry	HQER-SFA	hqer__induced	True	31	-502.9461027021527	hqer__induced sampler prototype	5.0
	WDI__industry	Quantile-SFA	hqer__induced	True	13	-568.4551952596921	hqer__induced sampler prototype	2.0
	WDI__industry	HQER-SFA	hqer__induced	True	16	-568.3002599832103	hqer__induced sampler prototype	2.0
	WDI__industry	Quantile-SFA	hqer__induced	True	26	-522.9959009674418	hqer__induced sampler prototype	4.0
	WDI__industry	HQER-SFA	hqer__induced	True	28	-522.8436228872424	hqer__induced sampler prototype	4.0
	WDI__industry	Quantile-SFA	hqer__induced	True	20	-579.4062811452014	hqer__induced sampler prototype	4.0
	WDI__industry	HQER-SFA	hqer__induced	True	20	-579.2204660530831	hqer__induced sampler prototype	4.0
	WDI__industry	Quantile-SFA	hqer__induced	True	16	-502.1233985597826	hqer__induced sampler prototype	2.0
	WDI__industry	HQER-SFA	hqer__induced	True	23	-501.9647073725037	hqer__induced sampler prototype	4.0
	WDI__industry	Quantile-SFA	hqer__induced	True	19	-615.5150778696418	hqer__induced sampler prototype	4.0
	WDI__industry	HQER-SFA	hqer__induced	True	23	-615.3179567881073	hqer__induced sampler prototype	2.0

experiment	dataset	model	scenario	converged	n_iter	loglik	warning_message	best_start_id
	WDI_industry	Quantile-SFA	hqer_induced	True	16	-587.3329958971563	hqer_induced sampler prototype	2.0
	WDI_industry	HQER-SFA	hqer_induced	True	15	-587.2175596913565	hqer_induced sampler prototype	2.0
	WDI_industry	Quantile-SFA	hqer_induced	True	20	-567.3213747873855	hqer_induced sampler prototype	4.0
	WDI_industry	HQER-SFA	hqer_induced	True	24	-567.1562090095463	hqer_induced sampler prototype	4.0
	WDI_industry	Quantile-SFA	hqer_induced	True	13	-490.4431491183184	hqer_induced sampler prototype	2.0
	WDI_industry	HQER-SFA	hqer_induced	True	19	-490.2873885556709	hqer_induced sampler prototype	2.0
	WDI_industry	Quantile-SFA	hqer_induced	True	17	-541.4271822909036	hqer_induced sampler prototype	4.0
	WDI_industry	HQER-SFA	hqer_induced	True	25	-541.2490117376085	hqer_induced sampler prototype	4.0
	WDI_industry	Quantile-SFA	hqer_induced	True	21	-545.7408881768195	hqer_induced sampler prototype	4.0
	WDI_industry	HQER-SFA	hqer_induced	True	20	-545.5688371434024	hqer_induced sampler prototype	4.0
	WDI_industry	Quantile-SFA	hqer_induced	True	15	-488.5936005620428	hqer_induced sampler prototype	2.0
	WDI_industry	HQER-SFA	hqer_induced	True	18	-488.3950402219763	hqer_induced sampler prototype	2.0
	WDI_industry	Quantile-SFA	hqer_induced	True	17	-544.0779154778704	hqer_induced sampler prototype	4.0
	WDI_industry	HQER-SFA	hqer_induced	True	21	-543.8694440107972	hqer_induced sampler prototype	4.0
	WDI_industry	Quantile-SFA	hqer_induced	True	13	-512.7933623060235	hqer_induced sampler prototype	2.0
	WDI_industry	HQER-SFA	hqer_induced	True	26	-512.666381315716	hqer_induced sampler prototype	4.0
	WDI_industry	Quantile-SFA	hqer_induced	True	14	-604.4655455680347	hqer_induced sampler prototype	2.0
	WDI_industry	HQER-SFA	hqer_induced	True	17	-604.2681436356362	hqer_induced sampler prototype	2.0
	WDI_industry	Quantile-SFA	hqer_induced	True	20	-523.0882977486851	hqer_induced sampler prototype	4.0
	WDI_industry	HQER-SFA	hqer_induced	True	19	-522.9488017697646	hqer_induced sampler prototype	4.0
	WDI_industry	Quantile-SFA	hqer_induced	True	18	-479.2367362068113	hqer_induced sampler prototype	2.0
	WDI_industry	HQER-SFA	hqer_induced	True	24	-479.07592111448895	hqer_induced sampler prototype	4.0
	WDI_industry	Quantile-SFA	hqer_induced	True	20	-589.7009065311383	hqer_induced sampler prototype	4.0
	WDI_industry	HQER-SFA	hqer_induced	True	19	-589.5388726189292	hqer_induced sampler prototype	2.0
	WDI_industry	Quantile-SFA	hqer_induced	True	19	-524.8242649597332	hqer_induced sampler prototype	4.0
	WDI_industry	HQER-SFA	hqer_induced	True	14	-524.6835454983026	hqer_induced sampler prototype	2.0
	WDI_industry	Quantile-SFA	hqer_induced	True	12	-537.372450855351	hqer_induced sampler prototype	2.0
	WDI_industry	HQER-SFA	hqer_induced	True	15	-537.1917504994134	hqer_induced sampler prototype	2.0
	WDI_industry	Quantile-SFA	hqer_induced	True	14	-469.5751453723849	hqer_induced sampler prototype	4.0
	WDI_industry	HQER-SFA	hqer_induced	True	20	-469.427074002188	hqer_induced sampler prototype	4.0
	WDI_industry	Quantile-SFA	hqer_induced	True	17	-508.892870592461	hqer_induced sampler prototype	4.0
	WDI_industry	HQER-SFA	hqer_induced	True	17	-508.7696508957674	hqer_induced sampler prototype	2.0
	WDI_industry	Quantile-SFA	hqer_induced	True	11	-493.5509856501778	hqer_induced sampler prototype	2.0
	WDI_industry	HQER-SFA	hqer_induced	True	25	-493.36395324974455	hqer_induced sampler prototype	4.0
	WDI_industry	Quantile-SFA	hqer_induced	True	22	-588.6561266222175	hqer_induced sampler prototype	4.0
	WDI_industry	HQER-SFA	hqer_induced	True	21	-588.4979761701983	hqer_induced sampler prototype	4.0
	WDI_industry	Quantile-SFA	hqer_induced	True	22	-498.80194120932026	hqer_induced sampler prototype	2.0
	WDI_industry	HQER-SFA	hqer_induced	True	19	-498.6682992347223	hqer_induced sampler prototype	2.0
	WDI_industry	Quantile-SFA	hqer_induced	True	22	-561.3286827225877	hqer_induced sampler prototype	4.0
	WDI_industry	HQER-SFA	hqer_induced	True	15	-561.2782263335357	hqer_induced sampler prototype	4.0
	WDI_industry	Quantile-SFA	hqer_induced	True	12	-576.5977285964101	hqer_induced sampler prototype	2.0
	WDI_industry	HQER-SFA	hqer_induced	True	15	-576.4050619827576	hqer_induced sampler prototype	2.0
	WDI_industry	Quantile-SFA	hqer_induced	True	40	-577.4976755987822	hqer_induced sampler prototype	5.0
	WDI_industry	HQER-SFA	hqer_induced	True	24	-577.2762975123908	hqer_induced sampler prototype	4.0
	WDI_industry	Quantile-SFA	hqer_induced	True	22	-550.0811115064239	hqer_induced sampler prototype	2.0
	WDI_industry	HQER-SFA	hqer_induced	True	23	-549.9283992264692	hqer_induced sampler prototype	4.0
	WDI_industry	Quantile-SFA	hqer_induced	True	32	-518.1761597688799	hqer_induced sampler prototype	5.0
	WDI_industry	HQER-SFA	hqer_induced	True	20	-517.9607488559636	hqer_induced sampler prototype	4.0
	WDI_industry	Quantile-SFA	hqer_induced	True	18	-606.7222706004252	hqer_induced sampler prototype	2.0
	WDI_industry	HQER-SFA	hqer_induced	True	16	-606.5240865892949	hqer_induced sampler prototype	2.0
	WDI_industry	Quantile-SFA	hqer_induced	True	18	-610.2125092418939	hqer_induced sampler prototype	2.0
	WDI_industry	HQER-SFA	hqer_induced	True	16	-609.9759495758788	hqer_induced sampler prototype	2.0
	WDI_industry	Quantile-SFA	hqer_induced	True	22	-534.3956034591369	hqer_induced sampler prototype	4.0
	WDI_industry	HQER-SFA	hqer_induced	True	22	-534.2294023463876	hqer_induced sampler prototype	4.0
	WDI_industry	Quantile-SFA	hqer_induced	True	19	-534.7193642959365	hqer_induced sampler prototype	4.0
	WDI_industry	HQER-SFA	hqer_induced	True	19	-534.6115656386006	hqer_induced sampler prototype	4.0
	WDI_industry	Quantile-SFA	hqer_induced	True	16	-573.7798362384182	hqer_induced sampler prototype	2.0
	WDI_industry	HQER-SFA	hqer_induced	True	24	-573.7220931562128	hqer_induced sampler prototype	4.0
	WDI_industry	Quantile-SFA	hqer_induced	True	33	-452.3169642551729	hqer_induced sampler prototype	2.0
	WDI_industry	HQER-SFA	hqer_induced	True	20	-452.2237380166282	hqer_induced sampler prototype	4.0
	WDI_industry	Quantile-SFA	hqer_induced	True	18	-480.32568389735536	hqer_induced sampler prototype	4.0
	WDI_industry	HQER-SFA	hqer_induced	True	21	-480.2146884270729	hqer_induced sampler prototype	4.0
	WDI_industry	Quantile-SFA	hqer_induced	True	21	-566.0867680515793	hqer_induced sampler prototype	4.0
	WDI_industry	HQER-SFA	hqer_induced	True	23	-565.9180502868132	hqer_induced sampler prototype	4.0
	WDI_industry	Quantile-SFA	hqer_induced	True	22	-503.7875145758232	hqer_induced sampler prototype	4.0
	WDI_industry	HQER-SFA	hqer_induced	True	22	-503.6225340850861	hqer_induced sampler prototype	2.0
	WDI_industry	Quantile-SFA	hqer_induced	True	17	-561.6025503072447	hqer_induced sampler prototype	4.0
	WDI_industry	HQER-SFA	hqer_induced	True	18	-561.4445428952199	hqer_induced sampler prototype	4.0
	WDI_industry	Quantile-SFA	hqer_induced	True	11	-587.290679871383	hqer_induced sampler prototype	2.0
	WDI_industry	HQER-SFA	hqer_induced	True	15	-587.1012510702911	hqer_induced sampler prototype	2.0
	WDI_industry	Quantile-SFA	hqer_induced	True	19	-530.0380979333288	hqer_induced sampler prototype	4.0
	WDI_industry	HQER-SFA	hqer_induced	True	18	-529.897245582987	hqer_induced sampler prototype	4.0
	WDI_industry	Quantile-SFA	hqer_induced	True	14	-536.4745598271694	hqer_induced sampler prototype	2.0
	WDI_industry	HQER-SFA	hqer_induced	True	24	-536.316277228291	hqer_induced sampler prototype	4.0
	WDI_industry	Quantile-SFA	hqer_induced	True	22	-557.6213028102337	hqer_induced sampler prototype	4.0
	WDI_industry	HQER-SFA	hqer_induced	True	22	-557.4576106990669	hqer_induced sampler prototype	4.0
	WDI_industry	Quantile-SFA	hqer_induced	True	21	-536.4533459386735	hqer_induced sampler prototype	4.0
	WDI_industry	HQER-SFA	hqer_induced	True	24	-536.3128988283231	hqer_induced sampler prototype	4.0
	WDI_industry	Quantile-SFA	hqer_induced	True	17	-498.1692899117989	hqer_induced sampler prototype	4.0
	WDI_industry	HQER-SFA	hqer_induced	True	23	-498.0013376093509	hqer_induced sampler prototype	4.0
	WDI_industry	Quantile-SFA	hqer_induced	True	22	-546.6796849757868	hqer_induced sampler prototype	2.0
	WDI_industry	HQER-SFA	hqer_induced	True	21	-546.55873069437	hqer_induced sampler prototype	2.0
	WDI_industry	Quantile-SFA	hqer_induced	True	13	-525.7243268932319	hqer_induced sampler prototype	4.0
	WDI_industry	HQER-SFA	hqer_induced	True	14	-525.5871269056602	hqer_induced sampler prototype	4.0
	WDI_industry	Quantile-SFA	hqer_induced	True	16	-532.0894956941158	hqer_induced sampler prototype	2.0
	WDI_industry	HQER-SFA	hqer_induced	True	20	-531.9320707508373	hqer_induced sampler prototype	2.0
	WDI_industry	Quantile-SFA	hqer_induced	True	18	-512.1883921283377	hqer_induced sampler prototype	2.0
	WDI_industry	HQER-SFA	hqer_induced	True	11	-512.055651021124	hqer_induced sampler prototype	4.0
	WDI_industry	Quantile-SFA	hqer_induced	True	23	-554.7957652762917	hqer_induced sampler prototype	4.0
	WDI_industry	HQER-SFA	hqer_induced	True	16	-554.6374490068447	hqer_induced sampler prototype	4.0
	WDI_industry	Quantile-SFA	hqer_induced	True	19	-549.0790972369301	hqer_induced sampler prototype	4.0
	WDI_industry	HQER-SFA	hqer_induced	True	17	-548.8115515496964	hqer_induced sampler prototype	4.0
	WDI_industry	Quantile-SFA	hqer_induced	True	18	-516.054623887688	hqer_induced sampler prototype	2.0
	WDI_industry	HQER-SFA	hqer_induced	True	20	-515.9302008345642	hqer_induced sampler prototype	2.0
	WDI_industry	Quantile-SFA	hqer_induced	True	15	-539.9758393577636	hqer_induced sampler prototype	2.0
	WDI_industry	HQER-SFA	hqer_induced	True	20	-539.8125702013868	hqer_induced sampler prototype	4.0
	WDI_industry	Quantile-SFA	hqer_induced	True	18	-532.6049264033454	hqer_induced sampler prototype	2.0
	WDI_industry	HQER-SFA	hqer_induced	True	23	-532.4673976859601	hqer_induced sampler prototype	4.0
	WDI_industry	Quantile-SFA	hqer_induced	True	28	-526.2747562720275	hqer_induced sampler prototype	2.0
	WDI_industry	HQER-SFA	hqer_induced	True	23	-526.1220492648127	hqer_induced sampler prototype	4.0
	WDI_industry	Quantile-SFA	hqer_induced	True	23	-606.5988988917399	hqer_induced sampler prototype	4.0
	WDI_industry	HQER-SFA	hqer_induced	True	22	-606.398348233928	hqer_induced sampler prototype	4.0
	WDI_industry	Quantile-SFA	hqer_induced	True	28	-127.59423800706617	hqer_induced sampler prototype	2.0

experiment	dataset	model	scenario	converged	n_iter	loglik	warning_message	best_start_id
	WDI_industry	HQER-SFA	hqer_induced	True	27	-125.87430142847558	hqer_induced sampler prototype	2.0
	WDI_industry	Quantile-SFA	hqer_induced	True	18	-143.47424046508388	hqer_induced sampler prototype	2.0
	WDI_industry	HQER-SFA	hqer_induced	True	23	-141.67896844733727	hqer_induced sampler prototype	4.0
	WDI_industry	Quantile-SFA	hqer_induced	True	25	-208.37024579281723	hqer_induced sampler prototype	4.0
	WDI_industry	HQER-SFA	hqer_induced	True	26	-204.613813429549	hqer_induced sampler prototype	4.0
	WDI_industry	Quantile-SFA	hqer_induced	True	30	-122.35988591521404	hqer_induced sampler prototype	4.0
	WDI_industry	HQER-SFA	hqer_induced	True	20	-121.29191309854608	hqer_induced sampler prototype	2.0
	WDI_industry	Quantile-SFA	hqer_induced	True	32	-166.90457500821452	hqer_induced sampler prototype	4.0
	WDI_industry	HQER-SFA	hqer_induced	True	41	-161.55342118919674	hqer_induced sampler prototype	4.0
	WDI_industry	Quantile-SFA	hqer_induced	True	20	-177.6198922579577	hqer_induced sampler prototype	2.0
	WDI_industry	HQER-SFA	hqer_induced	True	22	-173.62655748920258	hqer_induced sampler prototype	4.0
	WDI_industry	Quantile-SFA	hqer_induced	True	19	-216.4068485183465	hqer_induced sampler prototype	2.0
	WDI_industry	HQER-SFA	hqer_induced	True	23	-212.7035159635115	hqer_induced sampler prototype	4.0
	WDI_industry	Quantile-SFA	hqer_induced	True	26	-129.3645443303193	hqer_induced sampler prototype	2.0
	WDI_industry	HQER-SFA	hqer_induced	True	20	-125.53412512361358	hqer_induced sampler prototype	2.0
	WDI_industry	Quantile-SFA	hqer_induced	True	19	-173.29587346134738	hqer_induced sampler prototype	2.0
	WDI_industry	HQER-SFA	hqer_induced	True	19	-168.7958740083339	hqer_induced sampler prototype	4.0
	WDI_industry	Quantile-SFA	hqer_induced	True	19	-141.52633968357554	hqer_induced sampler prototype	2.0
	WDI_industry	HQER-SFA	hqer_induced	True	26	-139.08098597673262	hqer_induced sampler prototype	4.0
	WDI_industry	Quantile-SFA	hqer_induced	True	23	-184.8695181816823	hqer_induced sampler prototype	4.0
	WDI_industry	HQER-SFA	hqer_induced	True	22	-182.7978244844908	hqer_induced sampler prototype	4.0
	WDI_industry	Quantile-SFA	hqer_induced	True	18	-139.24244178660427	hqer_induced sampler prototype	2.0
	WDI_industry	HQER-SFA	hqer_induced	True	23	-137.11547343750323	hqer_induced sampler prototype	4.0
	WDI_industry	Quantile-SFA	hqer_induced	True	35	-157.58631205666586	hqer_induced sampler prototype	4.0
	WDI_industry	HQER-SFA	hqer_induced	True	22	-155.92837580299442	hqer_induced sampler prototype	2.0
	WDI_industry	Quantile-SFA	hqer_induced	True	16	-160.2448835499871	hqer_induced sampler prototype	2.0
	WDI_industry	HQER-SFA	hqer_induced	True	26	-157.35282859172736	hqer_induced sampler prototype	4.0
	WDI_industry	Quantile-SFA	hqer_induced	True	28	-137.80357232773594	hqer_induced sampler prototype	2.0
	WDI_industry	HQER-SFA	hqer_induced	True	32	-137.7764257121874	hqer_induced sampler prototype	4.0
	WDI_industry	Quantile-SFA	hqer_induced	True	21	-185.84453161193488	hqer_induced sampler prototype	4.0
	WDI_industry	HQER-SFA	hqer_induced	True	31	-185.7454615476992	hqer_induced sampler prototype	2.0
	WDI_industry	Quantile-SFA	hqer_induced	True	23	-136.6584112037317	hqer_induced sampler prototype	4.0
	WDI_industry	HQER-SFA	hqer_induced	True	16	-135.89108206022087	hqer_induced sampler prototype	2.0
	WDI_industry	Quantile-SFA	hqer_induced	True	19	-174.947194022583	hqer_induced sampler prototype	4.0
	WDI_industry	HQER-SFA	hqer_induced	True	18	-173.42791948743758	hqer_induced sampler prototype	2.0
	WDI_industry	Quantile-SFA	hqer_induced	True	31	-137.5059869453659	hqer_induced sampler prototype	2.0
	WDI_industry	HQER-SFA	hqer_induced	True	26	-136.79268282461845	hqer_induced sampler prototype	3.0
	WDI_industry	Quantile-SFA	hqer_induced	True	22	-164.7168609513305	hqer_induced sampler prototype	4.0
	WDI_industry	HQER-SFA	hqer_induced	True	10	-163.40621069576446	hqer_induced sampler prototype	2.0
	WDI_industry	Quantile-SFA	hqer_induced	True	32	-141.02626871158463	hqer_induced sampler prototype	4.0
	WDI_industry	HQER-SFA	hqer_induced	True	30	-137.6437514826337	hqer_induced sampler prototype	4.0
	WDI_industry	Quantile-SFA	hqer_induced	True	20	-119.24481787659604	hqer_induced sampler prototype	4.0
	WDI_industry	HQER-SFA	hqer_induced	True	31	-117.8214694145364	hqer_induced sampler prototype	5.0
	WDI_industry	Quantile-SFA	hqer_induced	True	22	-151.98056392280833	hqer_induced sampler prototype	4.0
	WDI_industry	HQER-SFA	hqer_induced	True	18	-148.26668574889382	hqer_induced sampler prototype	4.0
	WDI_industry	Quantile-SFA	hqer_induced	True	18	-156.04362840752353	hqer_induced sampler prototype	4.0
	WDI_industry	HQER-SFA	hqer_induced	True	28	-154.32066799001814	hqer_induced sampler prototype	2.0
	WDI_industry	Quantile-SFA	hqer_induced	True	30	-169.50459421629915	hqer_induced sampler prototype	4.0
	WDI_industry	HQER-SFA	hqer_induced	True	27	-166.13928868939684	hqer_induced sampler prototype	4.0
	WDI_industry	Quantile-SFA	hqer_induced	True	25	-147.01360678701616	hqer_induced sampler prototype	4.0
	WDI_industry	HQER-SFA	hqer_induced	True	21	-145.5284436561318	hqer_induced sampler prototype	2.0
	WDI_industry	Quantile-SFA	hqer_induced	True	17	-149.47839096014096	hqer_induced sampler prototype	2.0
	WDI_industry	HQER-SFA	hqer_induced	True	34	-145.8017929116545	hqer_induced sampler prototype	4.0
	WDI_industry	Quantile-SFA	hqer_induced	True	17	-154.4652833758488	hqer_induced sampler prototype	2.0
	WDI_industry	HQER-SFA	hqer_induced	True	16	-152.71290109765533	hqer_induced sampler prototype	2.0
	WDI_industry	Quantile-SFA	hqer_induced	True	20	-159.3328046497315	hqer_induced sampler prototype	4.0
	WDI_industry	HQER-SFA	hqer_induced	True	32	-158.44065916099265	hqer_induced sampler prototype	5.0
	WDI_industry	Quantile-SFA	hqer_induced	True	24	-127.9502703659492	hqer_induced sampler prototype	4.0
	WDI_industry	HQER-SFA	hqer_induced	True	18	-126.89075983863916	hqer_induced sampler prototype	2.0
	WDI_industry	Quantile-SFA	hqer_induced	True	29	-177.2069934416083	hqer_induced sampler prototype	4.0
	WDI_industry	HQER-SFA	hqer_induced	True	22	-174.58591850160883	hqer_induced sampler prototype	4.0
	WDI_industry	Quantile-SFA	hqer_induced	True	22	-146.34411149457435	hqer_induced sampler prototype	4.0
	WDI_industry	HQER-SFA	hqer_induced	True	22	-142.03547526429958	hqer_induced sampler prototype	4.0
	WDI_industry	Quantile-SFA	hqer_induced	True	27	-137.17433765745943	hqer_induced sampler prototype	2.0
	WDI_industry	HQER-SFA	hqer_induced	True	27	-134.37935351894953	hqer_induced sampler prototype	4.0
	WDI_industry	Quantile-SFA	hqer_induced	True	29	-201.69970807464475	hqer_induced sampler prototype	4.0
	WDI_industry	HQER-SFA	hqer_induced	True	38	-198.3144906376973	hqer_induced sampler prototype	4.0
	WDI_industry	Quantile-SFA	hqer_induced	True	27	-166.64725739683536	hqer_induced sampler prototype	2.0
	WDI_industry	HQER-SFA	hqer_induced	True	14	-162.0124427554689	hqer_induced sampler prototype	4.0
	WDI_industry	Quantile-SFA	hqer_induced	True	34	-141.92590323543757	hqer_induced sampler prototype	4.0
	WDI_industry	HQER-SFA	hqer_induced	True	21	-135.59455270203944	hqer_induced sampler prototype	4.0
	WDI_industry	Quantile-SFA	hqer_induced	True	21	-146.42174345647118	hqer_induced sampler prototype	2.0
	WDI_industry	HQER-SFA	hqer_induced	True	31	-146.81962639270748	hqer_induced sampler prototype	5.0
	WDI_industry	Quantile-SFA	hqer_induced	True	23	-144.41295362139758	hqer_induced sampler prototype	2.0
	WDI_industry	HQER-SFA	hqer_induced	True	24	-146.31853641750348	hqer_induced sampler prototype	2.0
	WDI_industry	Quantile-SFA	hqer_induced	True	15	-160.8297547956403	hqer_induced sampler prototype	2.0
	WDI_industry	HQER-SFA	hqer_induced	True	23	-155.64543510141408	hqer_induced sampler prototype	4.0
	WDI_industry	Quantile-SFA	hqer_induced	True	21	-144.0388334777927	hqer_induced sampler prototype	2.0
	WDI_industry	HQER-SFA	hqer_induced	True	23	-137.4376599345776	hqer_induced sampler prototype	4.0
	WDI_industry	Quantile-SFA	hqer_induced	True	32	-134.2658821287622	hqer_induced sampler prototype	4.0
	WDI_industry	HQER-SFA	hqer_induced	True	29	-133.43444279609898	hqer_induced sampler prototype	4.0
	WDI_industry	Quantile-SFA	hqer_induced	True	15	-149.44469399640423	hqer_induced sampler prototype	2.0
	WDI_industry	HQER-SFA	hqer_induced	True	11	-150.67767700569578	hqer_induced sampler prototype	2.0
	WDI_industry	Quantile-SFA	hqer_induced	True	23	-149.7718865194525	hqer_induced sampler prototype	2.0
	WDI_industry	HQER-SFA	hqer_induced	True	16	-146.70784416622493	hqer_induced sampler prototype	4.0
	WDI_industry	Quantile-SFA	hqer_induced	True	19	-147.85073662653724	hqer_induced sampler prototype	2.0
	WDI_industry	HQER-SFA	hqer_induced	True	15	-146.98728763023826	hqer_induced sampler prototype	2.0
	WDI_industry	Quantile-SFA	hqer_induced	True	21	-132.60665008440606	hqer_induced sampler prototype	2.0
	WDI_industry	HQER-SFA	hqer_induced	True	13	-129.66673863316225	hqer_induced sampler prototype	4.0
	WDI_industry	Quantile-SFA	hqer_induced	True	18	-184.12044386651152	hqer_induced sampler prototype	4.0
	WDI_industry	HQER-SFA	hqer_induced	True	16	-181.50159944218208	hqer_induced sampler prototype	4.0
	WDI_industry	Quantile-SFA	hqer_induced	True	39	-142.06725085377386	hqer_induced sampler prototype	4.0
	WDI_industry	HQER-SFA	hqer_induced	True	33	-136.04672780777105	hqer_induced sampler prototype	4.0
	WDI_industry	Quantile-SFA	hqer_induced	True	25	-177.9392590517921	hqer_induced sampler prototype	2.0
	WDI_industry	HQER-SFA	hqer_induced	True	22	-176.80599961979834	hqer_induced sampler prototype	4.0
	WDI_industry	Quantile-SFA	hqer_induced	True	10	-141.11142695128137	hqer_induced sampler prototype	2.0
	WDI_industry	HQER-SFA	hqer_induced	True	33	-137.996684895052	hqer_induced sampler prototype	2.0
	WDI_industry	Quantile-SFA	hqer_induced	True	28	-138.67541507797517	hqer_induced sampler prototype	4.0
	WDI_industry	HQER-SFA	hqer_induced	True	17	-138.24350532350647	hqer_induced sampler prototype	2.0
	WDI_industry	Quantile-SFA	hqer_induced	True	27	-157.65907580285585	hqer_induced sampler prototype	2.0
	WDI_industry	HQER-SFA	hqer_induced	True	21	-154.1214201920059	hqer_induced sampler prototype	4.0
	WDI_industry	Quantile-SFA	hqer_induced	True	12	-167.273658293629	hqer_induced sampler prototype	2.0
	WDI_industry	HQER-SFA	hqer_induced	True	39	-163.7717795524377	hqer_induced sampler prototype	4.0

experiment	dataset	model	scenario	converged	n_iter	loglik	warning_message	best_start_id
	WDI_industry	Quantile-SFA	hqer_induced	True	25	-116.85401308340144	hqer_induced sampler prototype	2.0
	WDI_industry	HQER-SFA	hqer_induced	True	25	-113.5545714822184	hqer_induced sampler prototype	4.0
	WDI_industry	Quantile-SFA	hqer_induced	True	26	-208.6846032772033	hqer_induced sampler prototype	4.0
	WDI_industry	HQER-SFA	hqer_induced	True	20	-204.53002920578209	hqer_induced sampler prototype	4.0
	WDI_industry	Quantile-SFA	hqer_induced	True	22	-122.82965073005634	hqer_induced sampler prototype	4.0
	WDI_industry	HQER-SFA	hqer_induced	True	20	-122.2154008733344	hqer_induced sampler prototype	2.0
	WDI_industry	Quantile-SFA	hqer_induced	True	23	-133.1625349174107	hqer_induced sampler prototype	4.0
	WDI_industry	HQER-SFA	hqer_induced	True	42	-133.64455245985272	hqer_induced sampler prototype	4.0
	WDI_industry	Quantile-SFA	hqer_induced	True	26	-159.2796904170421	hqer_induced sampler prototype	2.0
	WDI_industry	HQER-SFA	hqer_induced	True	23	-154.47231774253603	hqer_induced sampler prototype	4.0
	WDI_industry	Quantile-SFA	hqer_induced	True	31	-111.30834525148882	hqer_induced sampler prototype	4.0
	WDI_industry	HQER-SFA	hqer_induced	True	28	-108.68013090400046	hqer_induced sampler prototype	4.0
	WDI_industry	Quantile-SFA	hqer_induced	True	27	-170.1991660922371	hqer_induced sampler prototype	4.0
	WDI_industry	HQER-SFA	hqer_induced	True	25	-168.21524581086894	hqer_induced sampler prototype	4.0
	WDI_industry	Quantile-SFA	hqer_induced	True	32	-168.53865736848823	hqer_induced sampler prototype	2.0
	WDI_industry	HQER-SFA	hqer_induced	True	23	-166.63461766604732	hqer_induced sampler prototype	2.0
	WDI_industry	Quantile-SFA	hqer_induced	True	22	-175.03813289425034	hqer_induced sampler prototype	4.0
	WDI_industry	HQER-SFA	hqer_induced	True	30	-175.21742433189496	hqer_induced sampler prototype	2.0
	WDI_industry	Quantile-SFA	hqer_induced	True	27	-144.5472880888332	hqer_induced sampler prototype	4.0
	WDI_industry	HQER-SFA	hqer_induced	True	17	-143.0497994145582	hqer_induced sampler prototype	2.0
	WDI_industry	Quantile-SFA	hqer_induced	True	23	-157.4774844803423	hqer_induced sampler prototype	4.0
	WDI_industry	HQER-SFA	hqer_induced	True	21	-156.16222995901137	hqer_induced sampler prototype	2.0
	WDI_industry	Quantile-SFA	hqer_induced	True	19	-138.37935467229923	hqer_induced sampler prototype	2.0
	WDI_industry	HQER-SFA	hqer_induced	True	20	-138.34806016714566	hqer_induced sampler prototype	2.0
	WDI_industry	Quantile-SFA	hqer_induced	True	32	-183.45030615863053	hqer_induced sampler prototype	4.0
	WDI_industry	HQER-SFA	hqer_induced	True	25	-178.94335359295832	hqer_induced sampler prototype	4.0
	WDI_industry	Quantile-SFA	hqer_induced	True	23	-162.20699487239784	hqer_induced sampler prototype	2.0
	WDI_industry	HQER-SFA	hqer_induced	True	24	-159.08967469001163	hqer_induced sampler prototype	4.0
	WDI_industry	Quantile-SFA	hqer_induced	True	27	-185.4461498051896	hqer_induced sampler prototype	4.0
	WDI_industry	HQER-SFA	hqer_induced	True	15	-183.6582702671641	hqer_induced sampler prototype	2.0
	WDI_industry	Quantile-SFA	hqer_induced	True	21	-184.13074172095247	hqer_induced sampler prototype	4.0
	WDI_industry	HQER-SFA	hqer_induced	True	20	-180.94504706515585	hqer_induced sampler prototype	4.0
	WDI_industry	Quantile-SFA	hqer_induced	True	37	-135.1856350147838	hqer_induced sampler prototype	2.0
	WDI_industry	HQER-SFA	hqer_induced	True	22	-128.02428949299753	hqer_induced sampler prototype	4.0
	WDI_industry	Quantile-SFA	hqer_induced	True	20	-173.98280110174272	hqer_induced sampler prototype	4.0
	WDI_industry	HQER-SFA	hqer_induced	True	18	-172.9313973513061	hqer_induced sampler prototype	2.0
	WDI_industry	Quantile-SFA	hqer_induced	True	21	-159.72134882236014	hqer_induced sampler prototype	4.0
	WDI_industry	HQER-SFA	hqer_induced	True	18	-156.855249316899	hqer_induced sampler prototype	4.0
	WDI_industry	Quantile-SFA	hqer_induced	True	19	-136.60984890444814	hqer_induced sampler prototype	2.0
	WDI_industry	HQER-SFA	hqer_induced	True	20	-133.04545665112164	hqer_induced sampler prototype	4.0
	WDI_industry	Quantile-SFA	hqer_induced	True	18	-135.7745629829523	hqer_induced sampler prototype	2.0
	WDI_industry	HQER-SFA	hqer_induced	True	20	-135.7531782140939	hqer_induced sampler prototype	2.0
	WDI_industry	Quantile-SFA	hqer_induced	True	22	-168.051454964848	hqer_induced sampler prototype	4.0
	WDI_industry	HQER-SFA	hqer_induced	True	30	-165.92678822898807	hqer_induced sampler prototype	5.0
	WDI_industry	Quantile-SFA	hqer_induced	True	12	-158.21109405789622	hqer_induced sampler prototype	2.0
	WDI_industry	HQER-SFA	hqer_induced	True	23	-155.83393385185406	hqer_induced sampler prototype	4.0
	WDI_industry	Quantile-SFA	hqer_induced	True	33	-160.19473574747013	hqer_induced sampler prototype	4.0
	WDI_industry	HQER-SFA	hqer_induced	True	30	-157.10120801291112	hqer_induced sampler prototype	4.0
	WDI_industry	Quantile-SFA	hqer_induced	True	20	-135.96200174225044	hqer_induced sampler prototype	2.0
	WDI_industry	HQER-SFA	hqer_induced	True	34	-131.07271889955877	hqer_induced sampler prototype	4.0
	WDI_industry	Quantile-SFA	hqer_induced	True	19	-143.79614658127426	hqer_induced sampler prototype	2.0
	WDI_industry	HQER-SFA	hqer_induced	True	13	-142.70247481010156	hqer_induced sampler prototype	2.0
	WDI_industry	Quantile-SFA	hqer_induced	True	29	-135.7836107289918	hqer_induced sampler prototype	4.0
	WDI_industry	HQER-SFA	hqer_induced	True	24	-133.69029519112394	hqer_induced sampler prototype	4.0
	WDI_industry	Quantile-SFA	hqer_induced	True	27	-148.08968736666984	hqer_induced sampler prototype	4.0
	WDI_industry	HQER-SFA	hqer_induced	True	21	-146.1164327195958	hqer_induced sampler prototype	4.0
	WDI_industry	Quantile-SFA	hqer_induced	True	26	-163.914721822569	hqer_induced sampler prototype	4.0
	WDI_industry	HQER-SFA	hqer_induced	True	25	-158.71311923644322	hqer_induced sampler prototype	4.0
	WDI_industry	Quantile-SFA	hqer_induced	True	17	-134.60784899800782	hqer_induced sampler prototype	2.0
	WDI_industry	HQER-SFA	hqer_induced	True	27	-131.95966162689177	hqer_induced sampler prototype	4.0
	WDI_industry	Quantile-SFA	hqer_induced	True	19	-131.35977921236383	hqer_induced sampler prototype	2.0
	WDI_industry	HQER-SFA	hqer_induced	True	19	-126.43458690669031	hqer_induced sampler prototype	4.0
	WDI_industry	Quantile-SFA	hqer_induced	True	24	-174.8237544415072	hqer_induced sampler prototype	2.0
	WDI_industry	HQER-SFA	hqer_induced	True	18	-170.619685850351	hqer_induced sampler prototype	4.0
	WDI_industry	Quantile-SFA	hqer_induced	True	21	-151.3464436642482	hqer_induced sampler prototype	4.0
	WDI_industry	HQER-SFA	hqer_induced	True	21	-148.1025008450277	hqer_induced sampler prototype	4.0
	WDI_industry	Quantile-SFA	hqer_induced	True	23	-170.41014340072982	hqer_induced sampler prototype	2.0
	WDI_industry	HQER-SFA	hqer_induced	True	12	-168.5826972112507	hqer_induced sampler prototype	2.0
	WDI_industry	Quantile-SFA	hqer_induced	True	9	-168.44526868629907	hqer_induced sampler prototype	2.0
	WDI_industry	HQER-SFA	hqer_induced	True	29	-164.72100798266194	hqer_induced sampler prototype	4.0
	WDI_industry	Quantile-SFA	hqer_induced	True	20	-154.1700576721617	hqer_induced sampler prototype	2.0
	WDI_industry	HQER-SFA	hqer_induced	True	23	-153.73313713669717	hqer_induced sampler prototype	2.0
	WDI_industry	Quantile-SFA	hqer_induced	True	26	-142.12968433929888	hqer_induced sampler prototype	4.0
	WDI_industry	HQER-SFA	hqer_induced	True	23	-139.93928250779527	hqer_induced sampler prototype	4.0
	WDI_industry	Quantile-SFA	hqer_induced	True	33	-141.6101971539379	hqer_induced sampler prototype	4.0
	WDI_industry	HQER-SFA	hqer_induced	True	21	-142.89006842548778	hqer_induced sampler prototype	2.0
	WDI_industry	Quantile-SFA	hqer_induced	True	12	-153.5422739132514	hqer_induced sampler prototype	2.0
	WDI_industry	HQER-SFA	hqer_induced	True	11	-153.1238178801463	hqer_induced sampler prototype	2.0
	WDI_industry	Quantile-SFA	hqer_induced	True	31	-164.22961086866255	hqer_induced sampler prototype	2.0
	WDI_industry	HQER-SFA	hqer_induced	True	14	-162.73600114639038	hqer_induced sampler prototype	2.0
	WDI_industry	Quantile-SFA	hqer_induced	True	29	-111.98989422110556	hqer_induced sampler prototype	4.0
	WDI_industry	HQER-SFA	hqer_induced	True	20	-107.0912598506836	hqer_induced sampler prototype	4.0
	WDI_industry	Quantile-SFA	hqer_induced	True	31	-121.31148208749316	hqer_induced sampler prototype	2.0
	WDI_industry	HQER-SFA	hqer_induced	True	24	-117.47239407971443	hqer_induced sampler prototype	4.0
	WDI_industry	Quantile-SFA	hqer_induced	True	30	-166.30888447699465	hqer_induced sampler prototype	4.0
	WDI_industry	HQER-SFA	hqer_induced	True	26	-164.36888897026972	hqer_induced sampler prototype	4.0
	WDI_industry	Quantile-SFA	hqer_induced	True	28	-127.52285603761015	hqer_induced sampler prototype	4.0
	WDI_industry	HQER-SFA	hqer_induced	True	12	-126.02062629339048	hqer_induced sampler prototype	2.0
	WDI_industry	Quantile-SFA	hqer_induced	True	20	-170.87234464385412	hqer_induced sampler prototype	4.0
	WDI_industry	HQER-SFA	hqer_induced	True	19	-166.75241710056616	hqer_induced sampler prototype	4.0
	WDI_industry	Quantile-SFA	hqer_induced	True	29	-110.21830178848604	hqer_induced sampler prototype	4.0
	WDI_industry	HQER-SFA	hqer_induced	True	22	-107.1913806553257	hqer_induced sampler prototype	4.0
	WDI_industry	Quantile-SFA	hqer_induced	True	27	-193.10519154275977	hqer_induced sampler prototype	4.0
	WDI_industry	HQER-SFA	hqer_induced	True	36	-188.6249929586497	hqer_induced sampler prototype	4.0
	WDI_industry	Quantile-SFA	hqer_induced	True	28	-182.03852866089733	hqer_induced sampler prototype	4.0
	WDI_industry	HQER-SFA	hqer_induced	True	20	-179.27725470832104	hqer_induced sampler prototype	4.0
	WDI_industry	Quantile-SFA	hqer_induced	True	23	-162.28964994479216	hqer_induced sampler prototype	2.0
	WDI_industry	HQER-SFA	hqer_induced	True	22	-158.8388708795415	hqer_induced sampler prototype	4.0
	WDI_industry	Quantile-SFA	hqer_induced	True	25	-138.11183817739987	hqer_induced sampler prototype	2.0
	WDI_industry	HQER-SFA	hqer_induced	True	31	-137.6037041567175	hqer_induced sampler prototype	2.0
	WDI_industry	Quantile-SFA	hqer_induced	True	16	-133.95170612357091	hqer_induced sampler prototype	2.0
	WDI_industry	HQER-SFA	hqer_induced	True	18	-130.9353863463321	hqer_induced sampler prototype	4.0
	WDI_industry	Quantile-SFA	hqer_induced	True	15	-152.92139867534434	hqer_induced sampler prototype	4.0

experiment	dataset	model	scenario	converged	n_iter	loglik	warning_message	best_start_id
	WDI_industry	HQER-SFA	hqer_induced	True	15	-149.8058073627108	hqer_induced sampler prototype	4.0
	WDI_industry	Quantile-SFA	hqer_induced	True	27	-170.5836358026723	hqer_induced sampler prototype	4.0
	WDI_industry	HQER-SFA	hqer_induced	True	8	-171.32661383796113	hqer_induced sampler prototype	2.0
	WDI_industry	Quantile-SFA	hqer_induced	True	18	-151.66961051244863	hqer_induced sampler prototype	2.0
	WDI_industry	HQER-SFA	hqer_induced	True	16	-150.12507357901575	hqer_induced sampler prototype	2.0
	WDI_industry	Quantile-SFA	hqer_induced	True	15	-157.15851540885654	hqer_induced sampler prototype	2.0
	WDI_industry	HQER-SFA	hqer_induced	True	20	-153.12506471142498	hqer_induced sampler prototype	4.0
	WDI_industry	Quantile-SFA	hqer_induced	True	18	-148.00754871667695	hqer_induced sampler prototype	4.0
	WDI_industry	HQER-SFA	hqer_induced	True	23	-144.51277475187248	hqer_induced sampler prototype	4.0
	WDI_industry	Quantile-SFA	hqer_induced	True	27	-143.551939058406	hqer_induced sampler prototype	4.0
	WDI_industry	HQER-SFA	hqer_induced	True	15	-143.2109555870505	hqer_induced sampler prototype	2.0
	WDI_industry	Quantile-SFA	hqer_induced	True	15	-165.48814939730875	hqer_induced sampler prototype	2.0
	WDI_industry	HQER-SFA	hqer_induced	True	15	-163.80104508706364	hqer_induced sampler prototype	2.0
	WDI_industry	Quantile-SFA	hqer_induced	True	26	-182.94916098762275	hqer_induced sampler prototype	4.0
	WDI_industry	HQER-SFA	hqer_induced	True	8	-182.20577657868404	hqer_induced sampler prototype	2.0
	WDI_industry	Quantile-SFA	hqer_induced	True	21	-151.62492209484807	hqer_induced sampler prototype	2.0
	WDI_industry	HQER-SFA	hqer_induced	True	24	-148.14340208651564	hqer_induced sampler prototype	4.0
	WDI_industry	Quantile-SFA	hqer_induced	True	15	-195.32504214947977	hqer_induced sampler prototype	2.0
	WDI_industry	HQER-SFA	hqer_induced	True	25	-190.5938420708611	hqer_induced sampler prototype	4.0
	WDI_industry	Quantile-SFA	hqer_induced	True	25	-153.37615587245108	hqer_induced sampler prototype	4.0
	WDI_industry	HQER-SFA	hqer_induced	True	24	-149.14423520410924	hqer_induced sampler prototype	4.0
	WDI_industry	Quantile-SFA	hqer_induced	True	29	-158.6525678064366	hqer_induced sampler prototype	4.0
	WDI_industry	HQER-SFA	hqer_induced	True	28	-155.54180133158695	hqer_induced sampler prototype	4.0
	WDI_industry	Quantile-SFA	hqer_induced	True	36	-165.1857228264785	hqer_induced sampler prototype	4.0
	WDI_industry	HQER-SFA	hqer_induced	True	10	-164.29949187527032	hqer_induced sampler prototype	2.0
	WDI_industry	Quantile-SFA	hqer_induced	True	21	-157.48059814583974	hqer_induced sampler prototype	2.0
	WDI_industry	HQER-SFA	hqer_induced	True	25	-155.0951155712007	hqer_induced sampler prototype	4.0
	WDI_industry	Quantile-SFA	hqer_induced	True	22	-176.93828236767254	hqer_induced sampler prototype	4.0
	WDI_industry	HQER-SFA	hqer_induced	True	18	-176.69129764110207	hqer_induced sampler prototype	2.0
	WDI_industry	Quantile-SFA	hqer_induced	True	23	-121.78986769540754	hqer_induced sampler prototype	4.0
	WDI_industry	HQER-SFA	hqer_induced	True	20	-120.03064413988804	hqer_induced sampler prototype	2.0
	WDI_industry	Quantile-SFA	hqer_induced	True	32	-138.6686011595382	hqer_induced sampler prototype	4.0
	WDI_industry	HQER-SFA	hqer_induced	True	25	-137.2826970278228	hqer_induced sampler prototype	4.0
	WDI_industry	Quantile-SFA	hqer_induced	True	21	-153.0334790674881	hqer_induced sampler prototype	4.0
	WDI_industry	HQER-SFA	hqer_induced	True	21	-148.87827411520834	hqer_induced sampler prototype	4.0
	WDI_industry	Quantile-SFA	hqer_induced	True	15	-104.46685294190551	hqer_induced sampler prototype	2.0
	WDI_industry	HQER-SFA	hqer_induced	True	32	-102.19425069901776	hqer_induced sampler prototype	4.0
	WDI_industry	Quantile-SFA	hqer_induced	True	30	-189.1498550053309	hqer_induced sampler prototype	2.0
	WDI_industry	HQER-SFA	hqer_induced	True	25	-187.3166346435214	hqer_induced sampler prototype	2.0
	WDI_industry	Quantile-SFA	hqer_induced	True	20	-179.00779319954472	hqer_induced sampler prototype	4.0
	WDI_industry	HQER-SFA	hqer_induced	True	22	-174.642764549829	hqer_induced sampler prototype	4.0
	WDI_industry	Quantile-SFA	hqer_induced	True	18	-153.64181811882835	hqer_induced sampler prototype	4.0
	WDI_industry	HQER-SFA	hqer_induced	True	33	-150.5579702209075	hqer_induced sampler prototype	4.0
	WDI_industry	Quantile-SFA	hqer_induced	True	28	-147.15466758543872	hqer_induced sampler prototype	2.0
	WDI_industry	HQER-SFA	hqer_induced	True	22	-145.5430773272658	hqer_induced sampler prototype	2.0
	WDI_industry	Quantile-SFA	hqer_induced	True	16	-164.30960079056976	hqer_induced sampler prototype	4.0
	WDI_industry	HQER-SFA	hqer_induced	True	17	-163.31025755049328	hqer_induced sampler prototype	2.0
	WDI_industry	Quantile-SFA	hqer_induced	True	24	-147.57101099467593	hqer_induced sampler prototype	2.0
	WDI_industry	HQER-SFA	hqer_induced	True	30	-144.68967402625202	hqer_induced sampler prototype	4.0
	WDI_industry	Quantile-SFA	hqer_induced	True	30	-141.87874107663555	hqer_induced sampler prototype	4.0
	WDI_industry	HQER-SFA	hqer_induced	True	27	-138.2945706757419	hqer_induced sampler prototype	4.0
	WDI_industry	Quantile-SFA	hqer_induced	True	29	-141.854576932626	hqer_induced sampler prototype	2.0
	WDI_industry	HQER-SFA	hqer_induced	True	15	-138.4732400402307	hqer_induced sampler prototype	4.0
	WDI_industry	Quantile-SFA	hqer_induced	True	26	-188.8718935201392	hqer_induced sampler prototype	4.0
	WDI_industry	HQER-SFA	hqer_induced	True	20	-186.7106991343353	hqer_induced sampler prototype	2.0
	WDI_industry	Quantile-SFA	hqer_induced	True	21	-131.29320653851406	hqer_induced sampler prototype	4.0
	WDI_industry	HQER-SFA	hqer_induced	True	37	-131.13548432939083	hqer_induced sampler prototype	4.0
	WDI_industry	Quantile-SFA	hqer_induced	True	20	-174.07457294154648	hqer_induced sampler prototype	4.0
	WDI_industry	HQER-SFA	hqer_induced	True	46	-167.85144246491325	hqer_induced sampler prototype	4.0
	WDI_industry	Quantile-SFA	hqer_induced	True	17	-142.3298619738129	hqer_induced sampler prototype	4.0
	WDI_industry	HQER-SFA	hqer_induced	True	15	-140.30773357356975	hqer_induced sampler prototype	4.0
	WDI_industry	Quantile-SFA	hqer_induced	True	14	-157.79376515418045	hqer_induced sampler prototype	2.0
	WDI_industry	HQER-SFA	hqer_induced	True	21	-154.47718209416243	hqer_induced sampler prototype	4.0
	WDI_industry	Quantile-SFA	hqer_induced	True	13	-157.08655669072607	hqer_induced sampler prototype	2.0
	WDI_industry	HQER-SFA	hqer_induced	True	11	-155.3711021549721	hqer_induced sampler prototype	2.0
	WDI_industry	Quantile-SFA	hqer_induced	True	34	-127.77483015744006	hqer_induced sampler prototype	4.0
	WDI_industry	HQER-SFA	hqer_induced	True	34	-124.80575501457376	hqer_induced sampler prototype	4.0
	WDI_industry	Quantile-SFA	hqer_induced	True	14	-171.79591427233413	hqer_induced sampler prototype	2.0
	WDI_industry	HQER-SFA	hqer_induced	True	22	-166.28786420320654	hqer_induced sampler prototype	4.0
	WDI_industry	Quantile-SFA	hqer_induced	True	11	-188.9572070988258	hqer_induced sampler prototype	2.0
	WDI_industry	HQER-SFA	hqer_induced	True	27	-185.03891185422583	hqer_induced sampler prototype	4.0
	WDI_industry	Quantile-SFA	hqer_induced	True	21	-160.0933497152012	hqer_induced sampler prototype	4.0
	WDI_industry	HQER-SFA	hqer_induced	True	36	-157.31064892031128	hqer_induced sampler prototype	4.0
	WDI_industry	Quantile-SFA	hqer_induced	True	25	-144.87327167625446	hqer_induced sampler prototype	4.0
	WDI_industry	HQER-SFA	hqer_induced	True	24	-144.45752481696564	hqer_induced sampler prototype	2.0
	WDI_industry	Quantile-SFA	hqer_induced	True	35	-177.46779728741808	hqer_induced sampler prototype	2.0
	WDI_industry	HQER-SFA	hqer_induced	True	12	-173.18346878778857	hqer_induced sampler prototype	4.0
	WDI_industry	Quantile-SFA	hqer_induced	True	23	-174.27364534423424	hqer_induced sampler prototype	4.0
	WDI_industry	HQER-SFA	hqer_induced	True	26	-172.71086311909096	hqer_induced sampler prototype	2.0
	WDI_industry	Quantile-SFA	hqer_induced	True	19	-118.48033965532986	hqer_induced sampler prototype	2.0
	WDI_industry	HQER-SFA	hqer_induced	True	15	-120.27109040984269	hqer_induced sampler prototype	2.0
	WDI_industry	Quantile-SFA	hqer_induced	True	32	-157.1026175189654	hqer_induced sampler prototype	4.0
	WDI_industry	HQER-SFA	hqer_induced	True	22	-152.19109624825154	hqer_induced sampler prototype	4.0
	WDI_industry	Quantile-SFA	hqer_induced	True	18	-156.79994487057493	hqer_induced sampler prototype	2.0
	WDI_industry	HQER-SFA	hqer_induced	True	12	-155.49917735683906	hqer_induced sampler prototype	2.0
	WDI_industry	Quantile-SFA	hqer_induced	True	22	-112.1663902668584	hqer_induced sampler prototype	4.0
	WDI_industry	HQER-SFA	hqer_induced	True	23	-111.32597739660692	hqer_induced sampler prototype	2.0
	WDI_industry	Quantile-SFA	hqer_induced	True	20	-157.4512184431161	hqer_induced sampler prototype	4.0
	WDI_industry	HQER-SFA	hqer_induced	True	10	-156.44312139495878	hqer_induced sampler prototype	2.0
	WDI_industry	Quantile-SFA	hqer_induced	True	25	-138.37789108531953	hqer_induced sampler prototype	2.0
	WDI_industry	HQER-SFA	hqer_induced	True	15	-134.55489262870148	hqer_induced sampler prototype	4.0
	WDI_industry	Quantile-SFA	hqer_induced	True	22	-146.93599002910918	hqer_induced sampler prototype	2.0
	WDI_industry	HQER-SFA	hqer_induced	True	31	-144.59180384244166	hqer_induced sampler prototype	4.0
	WDI_industry	Quantile-SFA	hqer_induced	True	27	-143.79951112066266	hqer_induced sampler prototype	4.0
	WDI_industry	HQER-SFA	hqer_induced	True	43	-138.575848612245	hqer_induced sampler prototype	2.0
	WDI_industry	Quantile-SFA	hqer_induced	True	17	-161.68319987107185	hqer_induced sampler prototype	4.0
	WDI_industry	HQER-SFA	hqer_induced	True	17	-159.13627975796797	hqer_induced sampler prototype	4.0
	WDI_industry	Quantile-SFA	hqer_induced	True	27	-165.63032536051944	hqer_induced sampler prototype	4.0
	WDI_industry	HQER-SFA	hqer_induced	True	25	-162.54942599239314	hqer_induced sampler prototype	4.0
	WDI_industry	Quantile-SFA	hqer_induced	True	25	-153.56023514537907	hqer_induced sampler prototype	2.0
	WDI_industry	HQER-SFA	hqer_induced	True	31	-150.73583866035193	hqer_induced sampler prototype	4.0
	WDI_industry	Quantile-SFA	hqer_induced	True	29	-167.81543982374387	hqer_induced sampler prototype	4.0
	WDI_industry	HQER-SFA	hqer_induced	True	21	-163.60098947565058	hqer_induced sampler prototype	4.0

experiment	dataset	model	scenario	converged	n__iter	loglik	warning__message	best__start__id
	WDI__industry	Quantile-SFA	hqer__induced	True	25	-140.9929524656697	hqer__induced sampler prototype	4.0
	WDI__industry	HQER-SFA	hqer__induced	True	24	-137.32152180885322	hqer__induced sampler prototype	4.0
	WDI__industry	Quantile-SFA	hqer__induced	True	14	-179.64765775897268	hqer__induced sampler prototype	4.0
	WDI__industry	HQER-SFA	hqer__induced	True	28	-175.45596253047017	hqer__induced sampler prototype	4.0
	WDI__industry	Quantile-SFA	hqer__induced	True	16	-143.62871278385165	hqer__induced sampler prototype	2.0
	WDI__industry	HQER-SFA	hqer__induced	True	29	-139.42374996099065	hqer__induced sampler prototype	4.0
	WDI__industry	Quantile-SFA	hqer__induced	True	26	-94.67995735648736	hqer__induced sampler prototype	4.0
	WDI__industry	HQER-SFA	hqer__induced	True	25	-92.31470583466098	hqer__induced sampler prototype	4.0
	WDI__industry	Quantile-SFA	hqer__induced	True	16	-128.55978239916342	hqer__induced sampler prototype	2.0
	WDI__industry	HQER-SFA	hqer__induced	True	25	-125.51732537545053	hqer__induced sampler prototype	4.0
	WDI__industry	Quantile-SFA	hqer__induced	True	17	-161.91047116148195	hqer__induced sampler prototype	2.0
	WDI__industry	HQER-SFA	hqer__induced	True	28	-159.46834695815602	hqer__induced sampler prototype	4.0
	WDI__industry	Quantile-SFA	hqer__induced	True	18	-162.55442448040105	hqer__induced sampler prototype	4.0
	WDI__industry	HQER-SFA	hqer__induced	True	19	-161.4247671157687	hqer__induced sampler prototype	2.0
	WDI__industry	Quantile-SFA	hqer__induced	True	26	-124.08788172560088	hqer__induced sampler prototype	2.0
	WDI__industry	HQER-SFA	hqer__induced	True	26	-120.47485074125728	hqer__induced sampler prototype	4.0
	WDI__industry	Quantile-SFA	hqer__induced	True	38	-178.74826844265365	hqer__induced sampler prototype	5.0
	WDI__industry	HQER-SFA	hqer__induced	True	20	-178.4641935398978	hqer__induced sampler prototype	2.0
	WDI__industry	Quantile-SFA	hqer__induced	True	29	-164.93505471365387	hqer__induced sampler prototype	4.0
	WDI__industry	HQER-SFA	hqer__induced	True	18	-164.1638734061337	hqer__induced sampler prototype	2.0
	WDI__industry	Quantile-SFA	hqer__induced	True	16	-184.96323877130823	hqer__induced sampler prototype	2.0
	WDI__industry	HQER-SFA	hqer__induced	True	28	-182.92616476238624	hqer__induced sampler prototype	2.0
	WDI__industry	Quantile-SFA	hqer__induced	True	14	-140.4743143027537	hqer__induced sampler prototype	2.0
	WDI__industry	HQER-SFA	hqer__induced	True	15	-138.71289409566606	hqer__induced sampler prototype	2.0
	WDI__industry	Quantile-SFA	hqer__induced	True	25	-168.34643332425208	hqer__induced sampler prototype	4.0
	WDI__industry	HQER-SFA	hqer__induced	True	19	-168.45294939685277	hqer__induced sampler prototype	2.0
	WDI__industry	Quantile-SFA	hqer__induced	True	19	-185.4460752669802	hqer__induced sampler prototype	4.0
	WDI__industry	HQER-SFA	hqer__induced	True	19	-183.04119530927937	hqer__induced sampler prototype	4.0
	WDI__industry	Quantile-SFA	hqer__induced	True	18	-172.287463972612	hqer__induced sampler prototype	2.0
	WDI__industry	HQER-SFA	hqer__induced	True	21	-167.68419798961332	hqer__induced sampler prototype	4.0
	WDI__industry	Quantile-SFA	hqer__induced	True	19	-190.76589336069287	hqer__induced sampler prototype	4.0
	WDI__industry	HQER-SFA	hqer__induced	True	30	-187.312788675106	hqer__induced sampler prototype	4.0
	WDI__industry	Quantile-SFA	hqer__induced	True	25	-151.8260723082793	hqer__induced sampler prototype	4.0
	WDI__industry	HQER-SFA	hqer__induced	True	24	-149.5659766899688	hqer__induced sampler prototype	4.0
	WDI__industry	Quantile-SFA	hqer__induced	True	36	-137.87976377537768	hqer__induced sampler prototype	2.0
	WDI__industry	HQER-SFA	hqer__induced	True	35	-133.31491264513926	hqer__induced sampler prototype	4.0
	WDI__industry	Quantile-SFA	hqer__induced	True	26	-160.67615243722804	hqer__induced sampler prototype	4.0
	WDI__industry	HQER-SFA	hqer__induced	True	24	-158.74941829400586	hqer__induced sampler prototype	2.0
	WDI__industry	Quantile-SFA	hqer__induced	True	24	-129.17837619099686	hqer__induced sampler prototype	2.0
	WDI__industry	HQER-SFA	hqer__induced	True	33	-126.55556873731864	hqer__induced sampler prototype	4.0
	WDI__industry	Quantile-SFA	hqer__induced	True	11	-169.82110177585434	hqer__induced sampler prototype	2.0
	WDI__industry	HQER-SFA	hqer__induced	True	29	-167.3854744580285	hqer__induced sampler prototype	4.0
	WDI__industry	Quantile-SFA	hqer__induced	True	22	-165.19928454382344	hqer__induced sampler prototype	4.0
	WDI__industry	HQER-SFA	hqer__induced	True	15	-160.67840743157916	hqer__induced sampler prototype	4.0
	WDI__industry	Quantile-SFA	hqer__induced	True	31	-169.07052192917206	hqer__induced sampler prototype	4.0
	WDI__industry	HQER-SFA	hqer__induced	True	22	-167.52012769463852	hqer__induced sampler prototype	2.0
	WDI__industry	Quantile-SFA	hqer__induced	True	13	-130.28293491807716	hqer__induced sampler prototype	2.0
	WDI__industry	HQER-SFA	hqer__induced	True	18	-127.97256386376613	hqer__induced sampler prototype	4.0
	WDI__industry	Quantile-SFA	hqer__induced	True	33	-121.80509070892089	hqer__induced sampler prototype	4.0
	WDI__industry	HQER-SFA	hqer__induced	True	26	-119.27475788140596	hqer__induced sampler prototype	4.0
	WDI__industry	Quantile-SFA	hqer__induced	True	31	-131.00950560403402	hqer__induced sampler prototype	4.0
	WDI__industry	HQER-SFA	hqer__induced	True	24	-125.14184746165812	hqer__induced sampler prototype	4.0
	WDI__industry	Quantile-SFA	hqer__induced	True	21	-165.31662469095275	hqer__induced sampler prototype	4.0
	WDI__industry	HQER-SFA	hqer__induced	True	26	-161.76585811399704	hqer__induced sampler prototype	4.0
	WDI__industry	Quantile-SFA	hqer__induced	True	29	-182.6957343433388	hqer__induced sampler prototype	4.0
	WDI__industry	HQER-SFA	hqer__induced	True	26	-179.07169412752947	hqer__induced sampler prototype	4.0
	WDI__industry	Quantile-SFA	hqer__induced	True	28	-172.01094655783834	hqer__induced sampler prototype	4.0
	WDI__industry	HQER-SFA	hqer__induced	True	25	-169.7513248824921	hqer__induced sampler prototype	4.0
	WDI__industry	Quantile-SFA	hqer__induced	True	24	-181.5184535055729	hqer__induced sampler prototype	2.0
	WDI__industry	HQER-SFA	hqer__induced	True	25	-181.4705341981594	hqer__induced sampler prototype	2.0
	WDI__industry	Quantile-SFA	hqer__induced	True	26	-167.84336661045455	hqer__induced sampler prototype	4.0
	WDI__industry	HQER-SFA	hqer__induced	True	24	-164.48068270196836	hqer__induced sampler prototype	4.0
	WDI__industry	Quantile-SFA	hqer__induced	True	28	-167.3678924535526	hqer__induced sampler prototype	4.0
	WDI__industry	HQER-SFA	hqer__induced	True	25	-162.0785621977392	hqer__induced sampler prototype	4.0
	WDI__industry	Quantile-SFA	hqer__induced	True	28	-152.06505957842666	hqer__induced sampler prototype	4.0
	WDI__industry	HQER-SFA	hqer__induced	True	46	-149.3258904546177	hqer__induced sampler prototype	4.0
	WDI__industry	Quantile-SFA	hqer__induced	True	26	-117.54110048100516	hqer__induced sampler prototype	4.0
	WDI__industry	HQER-SFA	hqer__induced	True	23	-115.20243814210043	hqer__induced sampler prototype	4.0
	WDI__industry	Quantile-SFA	hqer__induced	True	14	-141.26580323908183	hqer__induced sampler prototype	2.0
	WDI__industry	HQER-SFA	hqer__induced	True	26	-137.34625648608767	hqer__induced sampler prototype	4.0
	WDI__industry	Quantile-SFA	hqer__induced	True	21	-156.85289657177958	hqer__induced sampler prototype	2.0
	WDI__industry	HQER-SFA	hqer__induced	True	15	-154.9791236884881	hqer__induced sampler prototype	2.0
	WDI__industry	Quantile-SFA	hqer__induced	True	27	-148.5516348785505	hqer__induced sampler prototype	4.0
	WDI__industry	HQER-SFA	hqer__induced	True	22	-143.51624378146707	hqer__induced sampler prototype	4.0
	WDI__industry	Quantile-SFA	hqer__induced	True	18	-123.00049275535083	hqer__induced sampler prototype	4.0
	WDI__industry	HQER-SFA	hqer__induced	True	20	-122.82628574462684	hqer__induced sampler prototype	2.0
	WDI__industry	Quantile-SFA	hqer__induced	True	19	-188.1090893645476	hqer__induced sampler prototype	4.0
	WDI__industry	HQER-SFA	hqer__induced	True	23	-184.6794705368813	hqer__induced sampler prototype	4.0
	WDI__industry	Quantile-SFA	hqer__induced	True	26	-134.11750298208307	hqer__induced sampler prototype	4.0
	WDI__industry	HQER-SFA	hqer__induced	True	16	-133.36461914027885	hqer__induced sampler prototype	2.0
	WDI__industry	Quantile-SFA	hqer__induced	True	19	-162.88243015958852	hqer__induced sampler prototype	2.0
	WDI__industry	HQER-SFA	hqer__induced	True	22	-162.9682936419187	hqer__induced sampler prototype	2.0
	WDI__industry	Quantile-SFA	hqer__induced	True	26	-170.91543339519194	hqer__induced sampler prototype	4.0
	WDI__industry	HQER-SFA	hqer__induced	True	25	-166.86455308615933	hqer__induced sampler prototype	4.0
	WDI__industry	Quantile-SFA	hqer__induced	True	14	-147.71675912646276	hqer__induced sampler prototype	2.0
	WDI__industry	HQER-SFA	hqer__induced	True	12	-149.13680786213467	hqer__induced sampler prototype	2.0
	WDI__industry	Quantile-SFA	hqer__induced	True	20	-168.2113877581137	hqer__induced sampler prototype	2.0
	WDI__industry	HQER-SFA	hqer__induced	True	20	-166.11041402910092	hqer__induced sampler prototype	4.0
	WDI__industry	Quantile-SFA	hqer__induced	True	22	-192.23409880093408	hqer__induced sampler prototype	4.0
	WDI__industry	HQER-SFA	hqer__induced	True	22	-192.2273033270081	hqer__induced sampler prototype	2.0
	WDI__industry	Quantile-SFA	hqer__induced	True	16	-602.0990115391546	hqer__induced sampler prototype	4.0
	WDI__industry	HQER-SFA	hqer__induced	True	18	-591.2990594392115	hqer__induced sampler prototype	4.0
	WDI__industry	Quantile-SFA	hqer__induced	True	21	-625.141228907064	hqer__induced sampler prototype	2.0
	WDI__industry	HQER-SFA	hqer__induced	True	11	-617.97655916165	hqer__induced sampler prototype	4.0
	WDI__industry	Quantile-SFA	hqer__induced	True	21	-600.5395055330241	hqer__induced sampler prototype	4.0
	WDI__industry	HQER-SFA	hqer__induced	True	20	-589.3474832427455	hqer__induced sampler prototype	4.0
	WDI__industry	Quantile-SFA	hqer__induced	True	20	-668.1378082848723	hqer__induced sampler prototype	4.0
	WDI__industry	HQER-SFA	hqer__induced	True	10	-660.6180616687196	hqer__induced sampler prototype	2.0
	WDI__industry	Quantile-SFA	hqer__induced	True	20	-596.2830908084485	hqer__induced sampler prototype	4.0
	WDI__industry	HQER-SFA	hqer__induced	True	24	-587.7313488750408	hqer__induced sampler prototype	4.0
	WDI__industry	Quantile-SFA	hqer__induced	True	20	-642.4396060680153	hqer__induced sampler prototype	4.0
	WDI__industry	HQER-SFA	hqer__induced	True	22	-638.0073102584529	hqer__induced sampler prototype	2.0
	WDI__industry	Quantile-SFA	hqer__induced	True	22	-681.186460152056	hqer__induced sampler prototype	2.0

experiment	dataset	model	scenario	converged	n_iter	loglik	warning_message	best_start_id
	WDI_industry	HQER-SFA	hqer_induced	True	15	-671.6858769349791	hqer_induced sampler prototype	4.0
	WDI_industry	Quantile-SFA	hqer_induced	True	24	-607.0790078077166	hqer_induced sampler prototype	4.0
	WDI_industry	HQER-SFA	hqer_induced	True	18	-590.7265415184941	hqer_induced sampler prototype	4.0
	WDI_industry	Quantile-SFA	hqer_induced	True	30	-598.9687718384755	hqer_induced sampler prototype	4.0
	WDI_industry	HQER-SFA	hqer_induced	True	21	-588.4564379226772	hqer_induced sampler prototype	4.0
	WDI_industry	Quantile-SFA	hqer_induced	True	20	-615.2117502027788	hqer_induced sampler prototype	2.0
	WDI_industry	HQER-SFA	hqer_induced	True	15	-605.1975497745535	hqer_induced sampler prototype	4.0
	WDI_industry	Quantile-SFA	hqer_induced	True	16	-616.7518910832632	hqer_induced sampler prototype	2.0
	WDI_industry	HQER-SFA	hqer_induced	True	17	-603.0683051543738	hqer_induced sampler prototype	4.0
	WDI_industry	Quantile-SFA	hqer_induced	True	21	-627.9948818185459	hqer_induced sampler prototype	4.0
	WDI_industry	HQER-SFA	hqer_induced	True	18	-618.7785048390167	hqer_induced sampler prototype	4.0
	WDI_industry	Quantile-SFA	hqer_induced	True	17	-600.3742909801965	hqer_induced sampler prototype	2.0
	WDI_industry	HQER-SFA	hqer_induced	True	17	-592.4718449115919	hqer_induced sampler prototype	2.0
	WDI_industry	Quantile-SFA	hqer_induced	True	18	-645.3368950430968	hqer_induced sampler prototype	4.0
	WDI_industry	HQER-SFA	hqer_induced	True	8	-640.7126301807067	hqer_induced sampler prototype	2.0
	WDI_industry	Quantile-SFA	hqer_induced	True	30	-633.194837803116	hqer_induced sampler prototype	5.0
	WDI_industry	HQER-SFA	hqer_induced	True	15	-623.9625230460878	hqer_induced sampler prototype	2.0
	WDI_industry	Quantile-SFA	hqer_induced	True	17	-629.3933348058573	hqer_induced sampler prototype	4.0
	WDI_industry	HQER-SFA	hqer_induced	True	15	-621.2515361575063	hqer_induced sampler prototype	2.0
	WDI_industry	Quantile-SFA	hqer_induced	True	19	-614.0550754002592	hqer_induced sampler prototype	4.0
	WDI_industry	HQER-SFA	hqer_induced	True	14	-601.7084658382425	hqer_induced sampler prototype	4.0
	WDI_industry	Quantile-SFA	hqer_induced	True	26	-678.6599548035098	hqer_induced sampler prototype	4.0
	WDI_industry	HQER-SFA	hqer_induced	True	25	-670.2067009659547	hqer_induced sampler prototype	4.0
	WDI_industry	Quantile-SFA	hqer_induced	True	20	-630.2510974823133	hqer_induced sampler prototype	4.0
	WDI_industry	HQER-SFA	hqer_induced	True	24	-620.1930456396857	hqer_induced sampler prototype	4.0
	WDI_industry	Quantile-SFA	hqer_induced	True	16	-647.6451330498915	hqer_induced sampler prototype	4.0
	WDI_industry	HQER-SFA	hqer_induced	True	9	-641.5390228129764	hqer_induced sampler prototype	2.0
	WDI_industry	Quantile-SFA	hqer_induced	True	21	-595.1225218312196	hqer_induced sampler prototype	4.0
	WDI_industry	HQER-SFA	hqer_induced	True	22	-583.2345807973823	hqer_induced sampler prototype	4.0
	WDI_industry	Quantile-SFA	hqer_induced	True	16	-619.2677510619709	hqer_induced sampler prototype	2.0
	WDI_industry	HQER-SFA	hqer_induced	True	19	-608.2217067447019	hqer_induced sampler prototype	4.0
	WDI_industry	Quantile-SFA	hqer_induced	True	20	-656.1021389926436	hqer_induced sampler prototype	4.0
	WDI_industry	HQER-SFA	hqer_induced	True	21	-637.2931527178328	hqer_induced sampler prototype	2.0
	WDI_industry	Quantile-SFA	hqer_induced	True	21	-598.0767698419177	hqer_induced sampler prototype	4.0
	WDI_industry	HQER-SFA	hqer_induced	True	22	-591.5113697722655	hqer_induced sampler prototype	2.0
	WDI_industry	Quantile-SFA	hqer_induced	True	17	-635.9103231441411	hqer_induced sampler prototype	4.0
	WDI_industry	HQER-SFA	hqer_induced	True	20	-620.8910985881257	hqer_induced sampler prototype	4.0
	WDI_industry	Quantile-SFA	hqer_induced	True	15	-612.7003090307539	hqer_induced sampler prototype	4.0
	WDI_industry	HQER-SFA	hqer_induced	True	17	-604.5797943876332	hqer_induced sampler prototype	4.0
	WDI_industry	Quantile-SFA	hqer_induced	True	15	-611.643451758586	hqer_induced sampler prototype	2.0
	WDI_industry	HQER-SFA	hqer_induced	True	23	-599.1662485519807	hqer_induced sampler prototype	4.0
	WDI_industry	Quantile-SFA	hqer_induced	True	19	-651.8346250617061	hqer_induced sampler prototype	4.0
	WDI_industry	HQER-SFA	hqer_induced	True	28	-638.3863422468173	hqer_induced sampler prototype	4.0
	WDI_industry	Quantile-SFA	hqer_induced	True	9	-636.7696615581299	hqer_induced sampler prototype	2.0
	WDI_industry	HQER-SFA	hqer_induced	True	35	-623.489428705873	hqer_induced sampler prototype	4.0
	WDI_industry	Quantile-SFA	hqer_induced	True	22	-649.1490042642265	hqer_induced sampler prototype	2.0
	WDI_industry	HQER-SFA	hqer_induced	True	20	-636.9805452630982	hqer_induced sampler prototype	4.0
	WDI_industry	Quantile-SFA	hqer_induced	True	23	-634.7574184606035	hqer_induced sampler prototype	2.0
	WDI_industry	HQER-SFA	hqer_induced	True	23	-625.5025917704927	hqer_induced sampler prototype	4.0
	WDI_industry	Quantile-SFA	hqer_induced	True	20	-628.0616396692801	hqer_induced sampler prototype	4.0
	WDI_industry	HQER-SFA	hqer_induced	True	21	-622.0952755943765	hqer_induced sampler prototype	2.0
	WDI_industry	Quantile-SFA	hqer_induced	True	23	-632.8306795204134	hqer_induced sampler prototype	2.0
	WDI_industry	HQER-SFA	hqer_induced	True	18	-621.5328090431572	hqer_induced sampler prototype	4.0
	WDI_industry	Quantile-SFA	hqer_induced	True	29	-627.9434210802432	hqer_induced sampler prototype	4.0
	WDI_industry	HQER-SFA	hqer_induced	True	22	-620.0177917981979	hqer_induced sampler prototype	4.0
	WDI_industry	Quantile-SFA	hqer_induced	True	14	-638.5547476215352	hqer_induced sampler prototype	4.0
	WDI_industry	HQER-SFA	hqer_induced	True	30	-633.1467366430038	hqer_induced sampler prototype	5.0
	WDI_industry	Quantile-SFA	hqer_induced	True	14	-588.6962959846221	hqer_induced sampler prototype	2.0
	WDI_industry	HQER-SFA	hqer_induced	True	20	-585.2645043118866	hqer_induced sampler prototype	2.0
	WDI_industry	Quantile-SFA	hqer_induced	True	26	-604.2092184309573	hqer_induced sampler prototype	4.0
	WDI_industry	HQER-SFA	hqer_induced	True	10	-599.8269632521151	hqer_induced sampler prototype	2.0
	WDI_industry	Quantile-SFA	hqer_induced	True	22	-679.3444683920411	hqer_induced sampler prototype	4.0
	WDI_industry	HQER-SFA	hqer_induced	True	14	-670.4941745804838	hqer_induced sampler prototype	4.0
	WDI_industry	Quantile-SFA	hqer_induced	True	15	-670.0697288736382	hqer_induced sampler prototype	4.0
	WDI_industry	HQER-SFA	hqer_induced	True	19	-661.1851293702814	hqer_induced sampler prototype	4.0
	WDI_industry	Quantile-SFA	hqer_induced	True	21	-532.9587093713646	hqer_induced sampler prototype	4.0
	WDI_industry	HQER-SFA	hqer_induced	True	20	-525.8869288609039	hqer_induced sampler prototype	2.0
	WDI_industry	Quantile-SFA	hqer_induced	True	21	-654.6700159612353	hqer_induced sampler prototype	4.0
	WDI_industry	HQER-SFA	hqer_induced	True	15	-638.9218419226645	hqer_induced sampler prototype	2.0
	WDI_industry	Quantile-SFA	hqer_induced	True	21	-671.4031790808394	hqer_induced sampler prototype	4.0
	WDI_industry	HQER-SFA	hqer_induced	True	20	-664.6744814128961	hqer_induced sampler prototype	2.0
	WDI_industry	Quantile-SFA	hqer_induced	True	24	-558.1310037990623	hqer_induced sampler prototype	4.0
	WDI_industry	HQER-SFA	hqer_induced	True	11	-551.4019230811891	hqer_induced sampler prototype	2.0
	WDI_industry	Quantile-SFA	hqer_induced	True	22	-583.008627503609	hqer_induced sampler prototype	4.0
	WDI_industry	HQER-SFA	hqer_induced	True	15	-573.7005476497254	hqer_induced sampler prototype	4.0
	WDI_industry	Quantile-SFA	hqer_induced	True	33	-608.7509100017483	hqer_induced sampler prototype	4.0
	WDI_industry	HQER-SFA	hqer_induced	True	22	-599.2569701956463	hqer_induced sampler prototype	4.0
	WDI_industry	Quantile-SFA	hqer_induced	True	17	-618.4906812837269	hqer_induced sampler prototype	4.0
	WDI_industry	HQER-SFA	hqer_induced	True	17	-605.544545468802	hqer_induced sampler prototype	4.0
	WDI_industry	Quantile-SFA	hqer_induced	True	12	-653.0097096356941	hqer_induced sampler prototype	4.0
	WDI_industry	HQER-SFA	hqer_induced	True	13	-641.7759255847857	hqer_induced sampler prototype	4.0
	WDI_industry	Quantile-SFA	hqer_induced	True	17	-708.1272593308154	hqer_induced sampler prototype	2.0
	WDI_industry	HQER-SFA	hqer_induced	True	15	-694.5633234548061	hqer_induced sampler prototype	4.0
	WDI_industry	Quantile-SFA	hqer_induced	True	20	-627.7868327092151	hqer_induced sampler prototype	4.0
	WDI_industry	HQER-SFA	hqer_induced	True	15	-619.3983218024774	hqer_induced sampler prototype	4.0
	WDI_industry	Quantile-SFA	hqer_induced	True	18	-651.308264361139	hqer_induced sampler prototype	4.0
	WDI_industry	HQER-SFA	hqer_induced	True	19	-641.3409788011011	hqer_induced sampler prototype	4.0
	WDI_industry	Quantile-SFA	hqer_induced	True	18	-647.6239920628389	hqer_induced sampler prototype	2.0
	WDI_industry	HQER-SFA	hqer_induced	True	15	-636.1684793776571	hqer_induced sampler prototype	4.0
	WDI_industry	Quantile-SFA	hqer_induced	True	46	-646.8839593683258	hqer_induced sampler prototype	5.0
	WDI_industry	HQER-SFA	hqer_induced	True	22	-634.2309045833205	hqer_induced sampler prototype	4.0
	WDI_industry	Quantile-SFA	hqer_induced	True	21	-644.4578422132439	hqer_induced sampler prototype	4.0
	WDI_industry	HQER-SFA	hqer_induced	True	15	-637.3046619070685	hqer_induced sampler prototype	2.0
	WDI_industry	Quantile-SFA	hqer_induced	True	18	-699.3248036716346	hqer_induced sampler prototype	2.0
	WDI_industry	HQER-SFA	hqer_induced	True	18	-682.2676333596557	hqer_induced sampler prototype	4.0
	WDI_industry	Quantile-SFA	hqer_induced	True	11	-636.2177697637896	hqer_induced sampler prototype	2.0
	WDI_industry	HQER-SFA	hqer_induced	True	4	-631.1036286806797	hqer_induced sampler prototype	2.0
	WDI_industry	Quantile-SFA	hqer_induced	True	21	-666.5405287207157	hqer_induced sampler prototype	2.0
	WDI_industry	HQER-SFA	hqer_induced	True	15	-655.1914203769753	hqer_induced sampler prototype	4.0
	WDI_industry	Quantile-SFA	hqer_induced	True	27	-675.5135480123129	hqer_induced sampler prototype	4.0
	WDI_industry	HQER-SFA	hqer_induced	True	24	-664.5637121382827	hqer_induced sampler prototype	4.0
	WDI_industry	Quantile-SFA	hqer_induced	True	26	-628.9779092154312	hqer_induced sampler prototype	4.0
	WDI_industry	HQER-SFA	hqer_induced	True	19	-616.9445405614094	hqer_induced sampler prototype	4.0

experiment	dataset	model	scenario	converged	n_iter	loglik	warning_message	best_start_id
	WDI_industry	Quantile-SFA	hqer_induced	True	25	-631.0240405347829	hqer_induced sampler prototype	2.0
	WDI_industry	HQER-SFA	hqer_induced	True	11	-623.7199461763178	hqer_induced sampler prototype	2.0
	WDI_industry	Quantile-SFA	hqer_induced	True	22	-632.6516308027699	hqer_induced sampler prototype	4.0
	WDI_industry	HQER-SFA	hqer_induced	True	6	-625.1520369872864	hqer_induced sampler prototype	2.0
	WDI_industry	Quantile-SFA	hqer_induced	True	15	-678.5189823045913	hqer_induced sampler prototype	4.0
	WDI_industry	HQER-SFA	hqer_induced	True	15	-665.2081743206901	hqer_induced sampler prototype	4.0
	WDI_industry	Quantile-SFA	hqer_induced	True	19	-616.0456530604652	hqer_induced sampler prototype	4.0
	WDI_industry	HQER-SFA	hqer_induced	True	25	-608.2547674793245	hqer_induced sampler prototype	2.0
	WDI_industry	Quantile-SFA	hqer_induced	True	31	-532.877138608226	hqer_induced sampler prototype	5.0
	WDI_industry	HQER-SFA	hqer_induced	True	15	-522.9723825900139	hqer_induced sampler prototype	4.0
	WDI_industry	Quantile-SFA	hqer_induced	True	18	-614.9096119820372	hqer_induced sampler prototype	4.0
	WDI_industry	HQER-SFA	hqer_induced	True	21	-604.537712496502	hqer_induced sampler prototype	4.0
	WDI_industry	Quantile-SFA	hqer_induced	True	19	-615.5693366116216	hqer_induced sampler prototype	2.0
	WDI_industry	HQER-SFA	hqer_induced	True	13	-604.3201043130333	hqer_induced sampler prototype	4.0
	WDI_industry	Quantile-SFA	hqer_induced	True	13	-618.6233654093448	hqer_induced sampler prototype	2.0
	WDI_industry	HQER-SFA	hqer_induced	True	28	-609.8174751664595	hqer_induced sampler prototype	4.0
	WDI_industry	Quantile-SFA	hqer_induced	True	14	-613.4156668470365	hqer_induced sampler prototype	2.0
	WDI_industry	HQER-SFA	hqer_induced	True	17	-606.7786004925953	hqer_induced sampler prototype	2.0
	WDI_industry	Quantile-SFA	hqer_induced	True	9	-719.6927088870586	hqer_induced sampler prototype	2.0
	WDI_industry	HQER-SFA	hqer_induced	True	19	-700.6532952194293	hqer_induced sampler prototype	4.0
	WDI_industry	Quantile-SFA	hqer_induced	True	20	-656.8294470072389	hqer_induced sampler prototype	2.0
	WDI_industry	HQER-SFA	hqer_induced	True	20	-641.1102766542035	hqer_induced sampler prototype	4.0
	WDI_industry	Quantile-SFA	hqer_induced	True	13	-681.1825195950485	hqer_induced sampler prototype	4.0
	WDI_industry	HQER-SFA	hqer_induced	True	19	-666.8264698952851	hqer_induced sampler prototype	2.0
	WDI_industry	Quantile-SFA	hqer_induced	True	20	-533.3086331473967	hqer_induced sampler prototype	4.0
	WDI_industry	HQER-SFA	hqer_induced	True	23	-520.9175849882624	hqer_induced sampler prototype	4.0
	WDI_industry	Quantile-SFA	hqer_induced	True	19	-662.8336168070126	hqer_induced sampler prototype	2.0
	WDI_industry	HQER-SFA	hqer_induced	True	19	-653.639691875672	hqer_induced sampler prototype	4.0
	WDI_industry	Quantile-SFA	hqer_induced	True	22	-697.5312132189706	hqer_induced sampler prototype	4.0
	WDI_industry	HQER-SFA	hqer_induced	True	29	-686.1285323444615	hqer_induced sampler prototype	5.0
	WDI_industry	Quantile-SFA	hqer_induced	True	29	-630.690457192157	hqer_induced sampler prototype	4.0
	WDI_industry	HQER-SFA	hqer_induced	True	28	-616.0039775370308	hqer_induced sampler prototype	4.0
	WDI_industry	Quantile-SFA	hqer_induced	True	8	-611.7514562851316	hqer_induced sampler prototype	2.0
	WDI_industry	HQER-SFA	hqer_induced	True	19	-599.5008297540711	hqer_induced sampler prototype	2.0
	WDI_industry	Quantile-SFA	hqer_induced	True	18	-694.7225130632778	hqer_induced sampler prototype	4.0
	WDI_industry	HQER-SFA	hqer_induced	True	17	-682.7354142405738	hqer_induced sampler prototype	2.0
	WDI_industry	Quantile-SFA	hqer_induced	True	17	-691.3302331627872	hqer_induced sampler prototype	2.0
	WDI_industry	HQER-SFA	hqer_induced	True	27	-676.779738682854	hqer_induced sampler prototype	4.0
	WDI_industry	Quantile-SFA	hqer_induced	True	21	-595.173909222692	hqer_induced sampler prototype	4.0
	WDI_industry	HQER-SFA	hqer_induced	True	28	-593.3383357297331	hqer_induced sampler prototype	2.0
	WDI_industry	Quantile-SFA	hqer_induced	True	20	-640.1530384731846	hqer_induced sampler prototype	2.0
	WDI_industry	HQER-SFA	hqer_induced	True	16	-625.8139539172535	hqer_induced sampler prototype	4.0
	WDI_industry	Quantile-SFA	hqer_induced	True	21	-622.9520833791231	hqer_induced sampler prototype	2.0
	WDI_industry	HQER-SFA	hqer_induced	True	19	-610.4955077327102	hqer_induced sampler prototype	4.0
	WDI_industry	Quantile-SFA	hqer_induced	True	32	-644.0529347824136	hqer_induced sampler prototype	4.0
	WDI_industry	HQER-SFA	hqer_induced	True	9	-635.1001932774952	hqer_induced sampler prototype	2.0
	WDI_industry	Quantile-SFA	hqer_induced	True	23	-544.1136669556001	hqer_induced sampler prototype	2.0
	WDI_industry	HQER-SFA	hqer_induced	True	16	-538.7499525067121	hqer_induced sampler prototype	2.0
	WDI_industry	Quantile-SFA	hqer_induced	True	23	-642.8194897463895	hqer_induced sampler prototype	4.0
	WDI_industry	HQER-SFA	hqer_induced	True	19	-636.4305473810048	hqer_induced sampler prototype	4.0
	WDI_industry	Quantile-SFA	hqer_induced	True	24	-618.9257691384879	hqer_induced sampler prototype	4.0
	WDI_industry	HQER-SFA	hqer_induced	True	20	-607.8050538887396	hqer_induced sampler prototype	4.0
	WDI_industry	Quantile-SFA	hqer_induced	True	22	-603.9670370449114	hqer_induced sampler prototype	4.0
	WDI_industry	HQER-SFA	hqer_induced	True	21	-593.1326249856877	hqer_induced sampler prototype	4.0
	WDI_industry	Quantile-SFA	hqer_induced	True	18	-695.051985014546	hqer_induced sampler prototype	4.0
	WDI_industry	HQER-SFA	hqer_induced	True	22	-680.5020465402079	hqer_induced sampler prototype	4.0
	WDI_industry	Quantile-SFA	hqer_induced	True	15	-679.6354604087958	hqer_induced sampler prototype	2.0
	WDI_industry	HQER-SFA	hqer_induced	True	15	-672.9588376276783	hqer_induced sampler prototype	2.0
	WDI_industry	Quantile-SFA	hqer_induced	True	13	-604.7698043582809	hqer_induced sampler prototype	4.0
	WDI_industry	HQER-SFA	hqer_induced	True	20	-590.7003084591324	hqer_induced sampler prototype	4.0
	WDI_industry	Quantile-SFA	hqer_induced	True	15	-622.5287002260724	hqer_induced sampler prototype	4.0
	WDI_industry	HQER-SFA	hqer_induced	True	20	-609.3543087523146	hqer_induced sampler prototype	4.0
	WDI_industry	Quantile-SFA	hqer_induced	True	18	-614.1624347562779	hqer_induced sampler prototype	4.0
	WDI_industry	HQER-SFA	hqer_induced	True	17	-602.958652341654	hqer_induced sampler prototype	4.0
	WDI_industry	Quantile-SFA	hqer_induced	True	8	-565.9286518791381	hqer_induced sampler prototype	2.0
	WDI_industry	HQER-SFA	hqer_induced	True	30	-556.6851747221443	hqer_induced sampler prototype	5.0
	WDI_industry	Quantile-SFA	hqer_induced	True	13	-619.1283543874065	hqer_induced sampler prototype	2.0
	WDI_industry	HQER-SFA	hqer_induced	True	8	-611.4649405194076	hqer_induced sampler prototype	2.0
	WDI_industry	Quantile-SFA	hqer_induced	True	33	-617.7233561192268	hqer_induced sampler prototype	4.0
	WDI_industry	HQER-SFA	hqer_induced	True	17	-611.384107517978	hqer_induced sampler prototype	2.0
	WDI_industry	Quantile-SFA	hqer_induced	True	10	-642.640106130063	hqer_induced sampler prototype	2.0
	WDI_industry	HQER-SFA	hqer_induced	True	16	-630.4133882513281	hqer_induced sampler prototype	4.0
	WDI_industry	Quantile-SFA	hqer_induced	True	17	-642.6103091028208	hqer_induced sampler prototype	2.0
	WDI_industry	HQER-SFA	hqer_induced	True	25	-628.2225903062156	hqer_induced sampler prototype	4.0
	WDI_industry	Quantile-SFA	hqer_induced	True	22	-608.2965548228765	hqer_induced sampler prototype	4.0
	WDI_industry	HQER-SFA	hqer_induced	True	14	-599.3905902045309	hqer_induced sampler prototype	2.0
	WDI_industry	Quantile-SFA	hqer_induced	True	12	-636.4563105315774	hqer_induced sampler prototype	2.0
	WDI_industry	HQER-SFA	hqer_induced	True	11	-629.2735862476334	hqer_induced sampler prototype	2.0
	WDI_industry	Quantile-SFA	hqer_induced	True	17	-618.6500436362807	hqer_induced sampler prototype	2.0
	WDI_industry	HQER-SFA	hqer_induced	True	12	-604.7146136379555	hqer_induced sampler prototype	4.0
	WDI_industry	Quantile-SFA	hqer_induced	True	19	-722.8396994228572	hqer_induced sampler prototype	2.0
	WDI_industry	HQER-SFA	hqer_induced	True	25	-709.0948430336441	hqer_induced sampler prototype	4.0
	WDI_industry	Quantile-SFA	hqer_induced	True	23	-643.1531041986834	hqer_induced sampler prototype	4.0
	WDI_industry	HQER-SFA	hqer_induced	True	23	-634.9653715712521	hqer_induced sampler prototype	4.0
	WDI_industry	Quantile-SFA	hqer_induced	True	22	-590.6699174276719	hqer_induced sampler prototype	2.0
	WDI_industry	HQER-SFA	hqer_induced	True	21	-581.3553230031459	hqer_induced sampler prototype	4.0
	WDI_industry	Quantile-SFA	hqer_induced	True	27	-592.8368954821283	hqer_induced sampler prototype	4.0
	WDI_industry	HQER-SFA	hqer_induced	True	11	-586.6492447286971	hqer_induced sampler prototype	2.0
	WDI_industry	Quantile-SFA	hqer_induced	True	21	-659.7692309428162	hqer_induced sampler prototype	4.0
	WDI_industry	HQER-SFA	hqer_induced	True	22	-650.413728619177	hqer_induced sampler prototype	4.0
	WDI_industry	Quantile-SFA	hqer_induced	True	25	-634.9217681977233	hqer_induced sampler prototype	4.0
	WDI_industry	HQER-SFA	hqer_induced	True	26	-625.4038384370613	hqer_induced sampler prototype	4.0
	WDI_industry	Quantile-SFA	hqer_induced	True	10	-611.9569793346518	hqer_induced sampler prototype	2.0
	WDI_industry	HQER-SFA	hqer_induced	True	23	-602.1965855804174	hqer_induced sampler prototype	4.0
	WDI_industry	Quantile-SFA	hqer_induced	True	11	-624.026467034528	hqer_induced sampler prototype	2.0
	WDI_industry	HQER-SFA	hqer_induced	True	22	-615.5007475939569	hqer_induced sampler prototype	4.0
	WDI_industry	Quantile-SFA	hqer_induced	True	20	-609.7864243744405	hqer_induced sampler prototype	2.0
	WDI_industry	HQER-SFA	hqer_induced	True	19	-603.368602777525	hqer_induced sampler prototype	2.0
	WDI_industry	Quantile-SFA	hqer_induced	True	13	-613.6583139804496	hqer_induced sampler prototype	4.0
	WDI_industry	HQER-SFA	hqer_induced	True	16	-605.0961514593938	hqer_induced sampler prototype	4.0
	WDI_industry	Quantile-SFA	hqer_induced	True	25	-630.9922838156148	hqer_induced sampler prototype	2.0
	WDI_industry	HQER-SFA	hqer_induced	True	26	-623.5896014280128	hqer_induced sampler prototype	4.0
	WDI_industry	Quantile-SFA	hqer_induced	True	21	-624.2909223250939	hqer_induced sampler prototype	4.0

experiment	dataset	model	scenario	converged	n__iter	loglik	warning__message	best__start__id
	WDI__industry	HQER-SFA	hqer__induced	True	23	-616.2789724565152	hqer__induced sampler prototype	4.0
	WDI__industry	Quantile-SFA	hqer__induced	True	13	-643.4758343497078	hqer__induced sampler prototype	4.0
	WDI__industry	HQER-SFA	hqer__induced	True	15	-631.6680750764917	hqer__induced sampler prototype	4.0
	WDI__industry	Quantile-SFA	hqer__induced	True	22	-684.2254256569436	hqer__induced sampler prototype	4.0
	WDI__industry	HQER-SFA	hqer__induced	True	21	-673.1709260816165	hqer__induced sampler prototype	4.0
	WDI__industry	Quantile-SFA	hqer__induced	True	9	-592.1262090384769	hqer__induced sampler prototype	2.0
	WDI__industry	HQER-SFA	hqer__induced	True	20	-579.6481794067913	hqer__induced sampler prototype	4.0
	WDI__industry	Quantile-SFA	hqer__induced	True	20	-651.5484840244678	hqer__induced sampler prototype	4.0
	WDI__industry	HQER-SFA	hqer__induced	True	27	-641.5968980844189	hqer__induced sampler prototype	4.0
	WDI__industry	Quantile-SFA	hqer__induced	True	20	-626.8888653972617	hqer__induced sampler prototype	2.0
	WDI__industry	HQER-SFA	hqer__induced	True	32	-612.1779950661148	hqer__induced sampler prototype	4.0
	WDI__industry	Quantile-SFA	hqer__induced	True	10	-604.4526353277022	hqer__induced sampler prototype	2.0
	WDI__industry	HQER-SFA	hqer__induced	True	9	-598.2937363259508	hqer__induced sampler prototype	2.0
	WDI__industry	Quantile-SFA	hqer__induced	True	21	-685.9065947356328	hqer__induced sampler prototype	4.0
	WDI__industry	HQER-SFA	hqer__induced	True	15	-673.2277604388416	hqer__induced sampler prototype	4.0
	WDI__industry	Quantile-SFA	hqer__induced	True	21	-610.6661229779958	hqer__induced sampler prototype	4.0
	WDI__industry	HQER-SFA	hqer__induced	True	24	-600.4408724087871	hqer__induced sampler prototype	4.0
	WDI__industry	Quantile-SFA	hqer__induced	True	12	-566.9302978192959	hqer__induced sampler prototype	2.0
	WDI__industry	HQER-SFA	hqer__induced	True	14	-559.3511726225619	hqer__induced sampler prototype	2.0
	WDI__industry	Quantile-SFA	hqer__induced	True	16	-598.1178879980841	hqer__induced sampler prototype	2.0
	WDI__industry	HQER-SFA	hqer__induced	True	32	-584.162871339448	hqer__induced sampler prototype	4.0
	WDI__industry	Quantile-SFA	hqer__induced	True	22	-691.942318441886	hqer__induced sampler prototype	4.0
	WDI__industry	HQER-SFA	hqer__induced	True	17	-681.2237779105329	hqer__induced sampler prototype	4.0
	WDI__industry	Quantile-SFA	hqer__induced	True	19	-651.3318015326039	hqer__induced sampler prototype	2.0
	WDI__industry	HQER-SFA	hqer__induced	True	18	-640.9856841740217	hqer__induced sampler prototype	4.0
	WDI__industry	Quantile-SFA	hqer__induced	True	20	-565.2872615581139	hqer__induced sampler prototype	4.0
	WDI__industry	HQER-SFA	hqer__induced	True	14	-554.9158304803898	hqer__induced sampler prototype	4.0
	WDI__industry	Quantile-SFA	hqer__induced	True	18	-574.9403498911029	hqer__induced sampler prototype	2.0
	WDI__industry	HQER-SFA	hqer__induced	True	28	-566.4909420718641	hqer__induced sampler prototype	4.0
	WDI__industry	Quantile-SFA	hqer__induced	True	20	-640.7627507755233	hqer__induced sampler prototype	2.0
	WDI__industry	HQER-SFA	hqer__induced	True	22	-631.45090933694	hqer__induced sampler prototype	4.0
	WDI__industry	Quantile-SFA	hqer__induced	True	10	-606.6930517533443	hqer__induced sampler prototype	2.0
	WDI__industry	HQER-SFA	hqer__induced	True	37	-595.1582013030661	hqer__induced sampler prototype	4.0
	WDI__industry	Quantile-SFA	hqer__induced	True	20	-671.0090323154027	hqer__induced sampler prototype	2.0
	WDI__industry	HQER-SFA	hqer__induced	True	19	-657.0869002336403	hqer__induced sampler prototype	4.0
	WDI__industry	Quantile-SFA	hqer__induced	True	20	-676.0263072742575	hqer__induced sampler prototype	4.0
	WDI__industry	HQER-SFA	hqer__induced	True	24	-667.1094923185804	hqer__induced sampler prototype	4.0
	WDI__industry	Quantile-SFA	hqer__induced	True	25	-565.275281271423	hqer__induced sampler prototype	2.0
	WDI__industry	HQER-SFA	hqer__induced	True	11	-553.8441626256272	hqer__induced sampler prototype	4.0
	WDI__industry	Quantile-SFA	hqer__induced	True	13	-608.4631706201878	hqer__induced sampler prototype	2.0
	WDI__industry	HQER-SFA	hqer__induced	True	17	-601.2521502491524	hqer__induced sampler prototype	4.0
	WDI__industry	Quantile-SFA	hqer__induced	True	23	-650.0496578451233	hqer__induced sampler prototype	4.0
	WDI__industry	HQER-SFA	hqer__induced	True	18	-640.2324855821439	hqer__induced sampler prototype	4.0
	WDI__industry	Quantile-SFA	hqer__induced	True	15	-620.2857602801415	hqer__induced sampler prototype	2.0
	WDI__industry	HQER-SFA	hqer__induced	True	27	-612.6079260715323	hqer__induced sampler prototype	4.0
	WDI__industry	Quantile-SFA	hqer__induced	True	14	-562.2532147696272	hqer__induced sampler prototype	2.0
	WDI__industry	HQER-SFA	hqer__induced	True	10	-558.2348622047647	hqer__induced sampler prototype	2.0
	WDI__industry	Quantile-SFA	hqer__induced	True	22	-707.218657659315	hqer__induced sampler prototype	2.0
	WDI__industry	HQER-SFA	hqer__induced	True	17	-698.5700951726474	hqer__induced sampler prototype	4.0
	WDI__industry	Quantile-SFA	hqer__induced	True	18	-635.948455976579	hqer__induced sampler prototype	4.0
	WDI__industry	HQER-SFA	hqer__induced	True	25	-627.7819941486193	hqer__induced sampler prototype	4.0
	WDI__industry	Quantile-SFA	hqer__induced	True	24	-625.2989946379864	hqer__induced sampler prototype	4.0
	WDI__industry	HQER-SFA	hqer__induced	True	21	-614.3568922261431	hqer__induced sampler prototype	4.0
	WDI__industry	Quantile-SFA	hqer__induced	True	18	-610.7103872954212	hqer__induced sampler prototype	4.0
	WDI__industry	HQER-SFA	hqer__induced	True	18	-599.27818360076	hqer__induced sampler prototype	4.0
	WDI__industry	Quantile-SFA	hqer__induced	True	16	-637.6560976264386	hqer__induced sampler prototype	4.0
	WDI__industry	HQER-SFA	hqer__induced	True	17	-631.0561909313185	hqer__induced sampler prototype	2.0
	WDI__industry	Quantile-SFA	hqer__induced	True	18	-550.3804375613935	hqer__induced sampler prototype	4.0
	WDI__industry	HQER-SFA	hqer__induced	True	23	-541.3476100516694	hqer__induced sampler prototype	4.0
	WDI__industry	Quantile-SFA	hqer__induced	True	20	-705.8015958040489	hqer__induced sampler prototype	4.0
	WDI__industry	HQER-SFA	hqer__induced	True	21	-697.2029090247214	hqer__induced sampler prototype	4.0
	WDI__industry	Quantile-SFA	hqer__induced	True	20	-596.2525162872033	hqer__induced sampler prototype	4.0
	WDI__industry	HQER-SFA	hqer__induced	True	20	-582.3837406696214	hqer__induced sampler prototype	4.0
	WDI__industry	Quantile-SFA	hqer__induced	True	34	-572.8446147150223	hqer__induced sampler prototype	4.0
	WDI__industry	HQER-SFA	hqer__induced	True	27	-560.6369547816082	hqer__induced sampler prototype	5.0
	WDI__industry	Quantile-SFA	hqer__induced	True	12	-666.0693017922183	hqer__induced sampler prototype	2.0
	WDI__industry	HQER-SFA	hqer__induced	True	19	-654.2305029509126	hqer__induced sampler prototype	4.0
	WDI__industry	Quantile-SFA	hqer__induced	True	18	-570.9175327839371	hqer__induced sampler prototype	4.0
	WDI__industry	HQER-SFA	hqer__induced	True	11	-565.3294166950315	hqer__induced sampler prototype	2.0
	WDI__industry	Quantile-SFA	hqer__induced	True	20	-673.4392328351944	hqer__induced sampler prototype	4.0
	WDI__industry	HQER-SFA	hqer__induced	True	19	-660.1109402875047	hqer__induced sampler prototype	4.0
	WDI__industry	Quantile-SFA	hqer__induced	True	18	-597.0843703126195	hqer__induced sampler prototype	4.0
	WDI__industry	HQER-SFA	hqer__induced	True	31	-583.4821140329598	hqer__induced sampler prototype	4.0
	WDI__industry	Quantile-SFA	hqer__induced	True	19	-685.9888778039524	hqer__induced sampler prototype	4.0
	WDI__industry	HQER-SFA	hqer__induced	True	35	-676.9784762871911	hqer__induced sampler prototype	5.0
	WDI__industry	Quantile-SFA	hqer__induced	True	20	-622.9520619692828	hqer__induced sampler prototype	2.0
	WDI__industry	HQER-SFA	hqer__induced	True	16	-610.1515998981288	hqer__induced sampler prototype	4.0
	WDI__industry	Quantile-SFA	hqer__induced	True	13	-646.3081367398275	hqer__induced sampler prototype	4.0
	WDI__industry	HQER-SFA	hqer__induced	True	14	-638.4037198239475	hqer__induced sampler prototype	2.0
	WDI__industry	Quantile-SFA	hqer__induced	True	21	-661.6906818741484	hqer__induced sampler prototype	4.0
	WDI__industry	HQER-SFA	hqer__induced	True	17	-650.0829950580849	hqer__induced sampler prototype	4.0
	WDI__industry	Quantile-SFA	hqer__induced	True	20	-568.9870129804336	hqer__induced sampler prototype	4.0
	WDI__industry	HQER-SFA	hqer__induced	True	17	-561.034909135883	hqer__induced sampler prototype	2.0
	WDI__industry	Quantile-SFA	hqer__induced	True	17	-614.0417358218608	hqer__induced sampler prototype	4.0
	WDI__industry	HQER-SFA	hqer__induced	True	30	-604.1078707054974	hqer__induced sampler prototype	4.0
	WDI__industry	Quantile-SFA	hqer__induced	True	23	-679.2606777608361	hqer__induced sampler prototype	4.0
	WDI__industry	HQER-SFA	hqer__induced	True	17	-667.5427850174243	hqer__induced sampler prototype	4.0
	WDI__industry	Quantile-SFA	hqer__induced	True	19	-651.8092863589243	hqer__induced sampler prototype	4.0
	WDI__industry	HQER-SFA	hqer__induced	True	20	-643.5220982343378	hqer__induced sampler prototype	4.0
	WDI__industry	Quantile-SFA	hqer__induced	True	21	-560.3089175356334	hqer__induced sampler prototype	4.0
	WDI__industry	HQER-SFA	hqer__induced	True	25	-548.4931638565172	hqer__induced sampler prototype	4.0
	WDI__industry	Quantile-SFA	hqer__induced	True	14	-606.9259298718159	hqer__induced sampler prototype	2.0
	WDI__industry	HQER-SFA	hqer__induced	True	8	-604.5248454827424	hqer__induced sampler prototype	2.0
	WDI__industry	Quantile-SFA	hqer__induced	True	8	-684.8512674668889	hqer__induced sampler prototype	2.0
	WDI__industry	HQER-SFA	hqer__induced	True	29	-665.8944512173987	hqer__induced sampler prototype	4.0
	WDI__industry	Quantile-SFA	hqer__induced	True	19	-645.9803761230688	hqer__induced sampler prototype	2.0
	WDI__industry	HQER-SFA	hqer__induced	True	23	-632.7680764435028	hqer__induced sampler prototype	4.0
	WDI__industry	Quantile-SFA	hqer__induced	True	24	-636.0088503963377	hqer__induced sampler prototype	4.0
	WDI__industry	HQER-SFA	hqer__induced	True	8	-628.9911776955832	hqer__induced sampler prototype	2.0
	WDI__industry	Quantile-SFA	hqer__induced	True	13	-681.7463179621359	hqer__induced sampler prototype	2.0
	WDI__industry	HQER-SFA	hqer__induced	True	22	-671.1077310908279	hqer__induced sampler prototype	4.0
	WDI__industry	Quantile-SFA	hqer__induced	True	21	-603.3788074106599	hqer__induced sampler prototype	4.0
	WDI__industry	HQER-SFA	hqer__induced	True	24	-595.5510686055586	hqer__induced sampler prototype	4.0

experiment	dataset	model	scenario	converged	n_iter	loglik	warning_message	best_start_id
	WDI_industry	Quantile-SFA	hqer_induced	True	16	-572.5510536564148	hqer_induced sampler prototype	4.0
	WDI_industry	HQER-SFA	hqer_induced	True	21	-562.3840790629434	hqer_induced sampler prototype	4.0
	WDI_industry	Quantile-SFA	hqer_induced	True	18	-629.0580836672489	hqer_induced sampler prototype	2.0
	WDI_industry	HQER-SFA	hqer_induced	True	26	-618.0534578426102	hqer_induced sampler prototype	4.0
	WDI_industry	Quantile-SFA	hqer_induced	True	36	-642.5166837894797	hqer_induced sampler prototype	5.0
	WDI_industry	HQER-SFA	hqer_induced	True	20	-636.7764057147674	hqer_induced sampler prototype	2.0
	WDI_industry	Quantile-SFA	hqer_induced	True	24	-563.9894365284259	hqer_induced sampler prototype	4.0
	WDI_industry	HQER-SFA	hqer_induced	True	8	-557.5972363091987	hqer_induced sampler prototype	2.0
	WDI_industry	Quantile-SFA	hqer_induced	True	14	-663.9011648059305	hqer_induced sampler prototype	4.0
	WDI_industry	HQER-SFA	hqer_induced	True	19	-654.5772064178461	hqer_induced sampler prototype	4.0
	WDI_industry	Quantile-SFA	hqer_induced	True	18	-627.2380191282894	hqer_induced sampler prototype	4.0
	WDI_industry	HQER-SFA	hqer_induced	True	16	-613.4495022345363	hqer_induced sampler prototype	4.0
	WDI_industry	Quantile-SFA	hqer_induced	True	31	-672.7102754468357	hqer_induced sampler prototype	5.0
	WDI_industry	HQER-SFA	hqer_induced	True	11	-664.8062457947706	hqer_induced sampler prototype	4.0
	WDI_industry	Quantile-SFA	hqer_induced	True	20	-604.9611625140388	hqer_induced sampler prototype	4.0
	WDI_industry	HQER-SFA	hqer_induced	True	11	-601.0026829271342	hqer_induced sampler prototype	2.0
	WDI_industry	Quantile-SFA	hqer_induced	True	18	-551.0749612006825	hqer_induced sampler prototype	2.0
	WDI_industry	HQER-SFA	hqer_induced	True	16	-547.448318737391	hqer_induced sampler prototype	2.0
	WDI_industry	Quantile-SFA	hqer_induced	True	20	-639.5117188638033	hqer_induced sampler prototype	4.0
	WDI_industry	HQER-SFA	hqer_induced	True	16	-625.2841042584141	hqer_induced sampler prototype	4.0
	WDI_industry	Quantile-SFA	hqer_induced	True	22	-620.0454431608869	hqer_induced sampler prototype	4.0
	WDI_industry	HQER-SFA	hqer_induced	True	20	-614.9067881835144	hqer_induced sampler prototype	2.0
	WDI_industry	Quantile-SFA	hqer_induced	True	22	-720.7049837346872	hqer_induced sampler prototype	4.0
	WDI_industry	HQER-SFA	hqer_induced	True	15	-712.0163720747821	hqer_induced sampler prototype	4.0
	WDI_industry	Quantile-SFA	hqer_induced	True	20	-661.7973498877997	hqer_induced sampler prototype	4.0
	WDI_industry	HQER-SFA	hqer_induced	True	23	-652.6308706957955	hqer_induced sampler prototype	4.0
	WDI_industry	Quantile-SFA	hqer_induced	True	25	-668.5777491276175	hqer_induced sampler prototype	2.0
	WDI_industry	HQER-SFA	hqer_induced	True	25	-658.0177320417789	hqer_induced sampler prototype	4.0
	WDI_industry	Quantile-SFA	hqer_induced	True	19	-669.7593246328465	hqer_induced sampler prototype	4.0
	WDI_industry	HQER-SFA	hqer_induced	True	24	-664.2248594584075	hqer_induced sampler prototype	2.0
	WDI_industry	Quantile-SFA	hqer_induced	True	12	-634.4647241333078	hqer_induced sampler prototype	2.0
	WDI_industry	HQER-SFA	hqer_induced	True	19	-614.0595691988327	hqer_induced sampler prototype	2.0
	WDI_industry	Quantile-SFA	hqer_induced	True	26	-711.0565770683437	hqer_induced sampler prototype	4.0
	WDI_industry	HQER-SFA	hqer_induced	True	15	-695.957948675013	hqer_induced sampler prototype	4.0
	WDI_industry	Quantile-SFA	hqer_induced	True	15	-662.3160057353005	hqer_induced sampler prototype	2.0
	WDI_industry	HQER-SFA	hqer_induced	True	16	-647.0411278052246	hqer_induced sampler prototype	4.0
	WDI_industry	Quantile-SFA	hqer_induced	True	18	-676.1798406410508	hqer_induced sampler prototype	4.0
	WDI_industry	HQER-SFA	hqer_induced	True	19	-664.0789037557596	hqer_induced sampler prototype	4.0
	WDI_industry	Quantile-SFA	hqer_induced	True	9	-614.2309225387341	hqer_induced sampler prototype	2.0
	WDI_industry	HQER-SFA	hqer_induced	True	18	-601.5615415650504	hqer_induced sampler prototype	4.0
	WDI_industry	Quantile-SFA	hqer_induced	True	21	-664.1384168135131	hqer_induced sampler prototype	2.0
	WDI_industry	HQER-SFA	hqer_induced	True	11	-648.20733701939	hqer_induced sampler prototype	4.0
	WDI_industry	Quantile-SFA	hqer_induced	True	25	-608.1931056358329	hqer_induced sampler prototype	4.0
	WDI_industry	HQER-SFA	hqer_induced	True	14	-603.4658847471679	hqer_induced sampler prototype	2.0
	WDI_industry	Quantile-SFA	hqer_induced	True	34	-537.3792552952671	hqer_induced sampler prototype	5.0
	WDI_industry	HQER-SFA	hqer_induced	True	19	-533.0323213025996	hqer_induced sampler prototype	2.0
	WDI_industry	Quantile-SFA	hqer_induced	True	18	-535.7289600237151	hqer_induced sampler prototype	2.0
	WDI_industry	HQER-SFA	hqer_induced	True	20	-525.7521124593817	hqer_induced sampler prototype	4.0
	WDI_industry	Quantile-SFA	hqer_induced	True	19	-595.3312570704936	hqer_induced sampler prototype	2.0
	WDI_industry	HQER-SFA	hqer_induced	True	10	-586.8574173712312	hqer_induced sampler prototype	4.0
	WDI_industry	Quantile-SFA	hqer_induced	True	23	-671.4872735886099	hqer_induced sampler prototype	4.0
	WDI_industry	HQER-SFA	hqer_induced	True	14	-663.7251905730427	hqer_induced sampler prototype	2.0
	WDI_industry	Quantile-SFA	hqer_induced	True	16	-688.0114510224295	hqer_induced sampler prototype	2.0
	WDI_industry	HQER-SFA	hqer_induced	True	16	-677.4771901909836	hqer_induced sampler prototype	4.0
	WDI_industry	Quantile-SFA	hqer_induced	True	18	-622.5254020391598	hqer_induced sampler prototype	2.0
	WDI_industry	HQER-SFA	hqer_induced	True	23	-608.8023601484589	hqer_induced sampler prototype	4.0
	WDI_industry	Quantile-SFA	hqer_induced	True	15	-629.2082235476078	hqer_induced sampler prototype	2.0
	WDI_industry	HQER-SFA	hqer_induced	True	23	-618.4026124628258	hqer_induced sampler prototype	4.0
	WDI_industry	Quantile-SFA	hqer_induced	True	26	-579.0430359650566	hqer_induced sampler prototype	2.0
	WDI_industry	HQER-SFA	hqer_induced	True	8	-570.6382586798338	hqer_induced sampler prototype	2.0
	WDI_industry	Quantile-SFA	hqer_induced	True	8	-624.30258680529	hqer_induced sampler prototype	2.0
	WDI_industry	HQER-SFA	hqer_induced	True	17	-616.3440584812961	hqer_induced sampler prototype	4.0
	WDI_industry	Quantile-SFA	hqer_induced	True	27	-642.129650591637	hqer_induced sampler prototype	4.0
	WDI_industry	HQER-SFA	hqer_induced	True	13	-635.4475206432628	hqer_induced sampler prototype	2.0
	WDI_industry	Quantile-SFA	hqer_induced	True	18	-600.5549056713431	hqer_induced sampler prototype	2.0
	WDI_industry	HQER-SFA	hqer_induced	True	17	-597.0031990826326	hqer_induced sampler prototype	2.0
	WDI_industry	Quantile-SFA	hqer_induced	True	16	-629.2559221861674	hqer_induced sampler prototype	4.0
	WDI_industry	HQER-SFA	hqer_induced	True	23	-617.6711826256011	hqer_induced sampler prototype	4.0
	WDI_industry	Quantile-SFA	hqer_induced	True	14	-665.478215045951	hqer_induced sampler prototype	2.0
	WDI_industry	HQER-SFA	hqer_induced	True	15	-650.2146545494552	hqer_induced sampler prototype	4.0
	WDI_industry	Quantile-SFA	hqer_induced	True	17	-692.3809608627978	hqer_induced sampler prototype	2.0
	WDI_industry	HQER-SFA	hqer_induced	True	13	-678.6651733589975	hqer_induced sampler prototype	4.0
	WDI_industry	Quantile-SFA	hqer_induced	True	9	-646.5945589940563	hqer_induced sampler prototype	2.0
	WDI_industry	HQER-SFA	hqer_induced	True	20	-636.5337474126036	hqer_induced sampler prototype	4.0
	WDI_industry	Quantile-SFA	hqer_induced	True	18	-567.0217427420009	hqer_induced sampler prototype	2.0
	WDI_industry	HQER-SFA	hqer_induced	True	13	-561.530296035047	hqer_induced sampler prototype	2.0
	WDI_industry	Quantile-SFA	hqer_induced	True	20	-593.859055006615	hqer_induced sampler prototype	4.0
	WDI_industry	HQER-SFA	hqer_induced	True	7	-588.6745116382276	hqer_induced sampler prototype	2.0
	WDI_agriculture	Quantile-SFA	hqer_induced	True	10	-727.0569802577457	hqer_induced sampler prototype	2.0
	WDI_agriculture	HQER-SFA	hqer_induced	True	26	-726.9639774553409	hqer_induced sampler prototype	1.0
	WDI_agriculture	Quantile-SFA	hqer_induced	True	26	-725.8677928945099	hqer_induced sampler prototype	5.0
	WDI_agriculture	HQER-SFA	hqer_induced	True	24	-725.9773891002508	hqer_induced sampler prototype	3.0
	WDI_agriculture	Quantile-SFA	hqer_induced	True	31	-685.8604406925504	hqer_induced sampler prototype	1.0
	WDI_agriculture	HQER-SFA	hqer_induced	True	25	-685.8504544321182	hqer_induced sampler prototype	3.0
	WDI_agriculture	Quantile-SFA	hqer_induced	True	33	-718.4607021183416	hqer_induced sampler prototype	3.0
	WDI_agriculture	HQER-SFA	hqer_induced	True	43	-718.4982674995722	hqer_induced sampler prototype	5.0
	WDI_agriculture	Quantile-SFA	hqer_induced	True	12	-680.8832027485425	hqer_induced sampler prototype	2.0
	WDI_agriculture	HQER-SFA	hqer_induced	True	11	-680.8563410276454	hqer_induced sampler prototype	2.0
	WDI_agriculture	Quantile-SFA	hqer_induced	True	28	-678.6735383040518	hqer_induced sampler prototype	3.0
	WDI_agriculture	HQER-SFA	hqer_induced	True	40	-678.6636458822079	hqer_induced sampler prototype	3.0
	WDI_agriculture	Quantile-SFA	hqer_induced	True	30	-692.2980684388231	hqer_induced sampler prototype	3.0
	WDI_agriculture	HQER-SFA	hqer_induced	True	30	-692.2948700282034	hqer_induced sampler prototype	3.0
	WDI_agriculture	Quantile-SFA	hqer_induced	True	25	-697.0116553469263	hqer_induced sampler prototype	1.0
	WDI_agriculture	HQER-SFA	hqer_induced	True	30	-696.937591348521	hqer_induced sampler prototype	3.0
	WDI_agriculture	Quantile-SFA	hqer_induced	True	12	-689.2216351114075	hqer_induced sampler prototype	2.0
	WDI_agriculture	HQER-SFA	hqer_induced	True	15	-689.1859410406578	hqer_induced sampler prototype	2.0
	WDI_agriculture	Quantile-SFA	hqer_induced	True	26	-725.4795598629419	hqer_induced sampler prototype	1.0
	WDI_agriculture	HQER-SFA	hqer_induced	True	34	-725.4666821928622	hqer_induced sampler prototype	3.0
	WDI_agriculture	Quantile-SFA	hqer_induced	True	28	-715.6280090045782	hqer_induced sampler prototype	2.0
	WDI_agriculture	HQER-SFA	hqer_induced	True	38	-715.5999160893087	hqer_induced sampler prototype	2.0
	WDI_agriculture	Quantile-SFA	hqer_induced	True	20	-733.1050977158318	hqer_induced sampler prototype	1.0
	WDI_agriculture	HQER-SFA	hqer_induced	True	15	-733.1107529226463	hqer_induced sampler prototype	2.0
	WDI_agriculture	Quantile-SFA	hqer_induced	True	39	-714.8345474788616	hqer_induced sampler prototype	1.0

experiment	dataset	model	scenario	converged	n_iter	loglik	warning_message	best_start_id
	WDI_agriculture	HQER-SFA	hqer_induced	True	35	-714.8338309033628	hqer_induced sampler prototype	1.0
	WDI_agriculture	Quantile-SFA	hqer_induced	True	31	-744.3838923455194	hqer_induced sampler prototype	3.0
	WDI_agriculture	HQER-SFA	hqer_induced	True	23	-744.36474162038	hqer_induced sampler prototype	3.0
	WDI_agriculture	Quantile-SFA	hqer_induced	True	40	-720.0397135258067	hqer_induced sampler prototype	5.0
	WDI_agriculture	HQER-SFA	hqer_induced	True	37	-720.0308575201792	hqer_induced sampler prototype	1.0
	WDI_agriculture	Quantile-SFA	hqer_induced	True	31	-703.3775216349609	hqer_induced sampler prototype	1.0
	WDI_agriculture	HQER-SFA	hqer_induced	True	33	-703.33302246379	hqer_induced sampler prototype	3.0
	WDI_agriculture	Quantile-SFA	hqer_induced	True	35	-719.2718425324126	hqer_induced sampler prototype	3.0
	WDI_agriculture	HQER-SFA	hqer_induced	True	25	-719.2032965486068	hqer_induced sampler prototype	2.0
	WDI_agriculture	Quantile-SFA	hqer_induced	True	27	-692.0065317454475	hqer_induced sampler prototype	5.0
	WDI_agriculture	HQER-SFA	hqer_induced	True	36	-691.9949057004574	hqer_induced sampler prototype	3.0
	WDI_agriculture	Quantile-SFA	hqer_induced	True	29	-724.5026337295665	hqer_induced sampler prototype	1.0
	WDI_agriculture	HQER-SFA	hqer_induced	True	29	-724.4827785460401	hqer_induced sampler prototype	1.0
	WDI_agriculture	Quantile-SFA	hqer_induced	True	24	-723.6298773919	hqer_induced sampler prototype	1.0
	WDI_agriculture	HQER-SFA	hqer_induced	True	32	-723.6016812674313	hqer_induced sampler prototype	1.0
	WDI_agriculture	Quantile-SFA	hqer_induced	True	37	-694.3602392798305	hqer_induced sampler prototype	2.0
	WDI_agriculture	HQER-SFA	hqer_induced	True	28	-694.3597580333156	hqer_induced sampler prototype	1.0
	WDI_agriculture	Quantile-SFA	hqer_induced	True	35	-702.1482790210782	hqer_induced sampler prototype	1.0
	WDI_agriculture	HQER-SFA	hqer_induced	True	15	-702.1395065012174	hqer_induced sampler prototype	2.0
	WDI_agriculture	Quantile-SFA	hqer_induced	True	36	-735.3364204927225	hqer_induced sampler prototype	5.0
	WDI_agriculture	HQER-SFA	hqer_induced	True	23	-735.4839799352817	hqer_induced sampler prototype	2.0
	WDI_agriculture	Quantile-SFA	hqer_induced	True	27	-720.6974756559682	hqer_induced sampler prototype	1.0
	WDI_agriculture	HQER-SFA	hqer_induced	True	24	-720.6773686044239	hqer_induced sampler prototype	1.0
	WDI_agriculture	Quantile-SFA	hqer_induced	True	24	-724.5768686934085	hqer_induced sampler prototype	2.0
	WDI_agriculture	HQER-SFA	hqer_induced	True	26	-724.5469878223035	hqer_induced sampler prototype	1.0
	WDI_agriculture	Quantile-SFA	hqer_induced	True	30	-714.6709793227252	hqer_induced sampler prototype	2.0
	WDI_agriculture	HQER-SFA	hqer_induced	True	27	-714.5719214030594	hqer_induced sampler prototype	3.0
	WDI_agriculture	Quantile-SFA	hqer_induced	True	30	-745.0469986957233	hqer_induced sampler prototype	1.0
	WDI_agriculture	HQER-SFA	hqer_induced	True	12	-745.039939650509	hqer_induced sampler prototype	2.0
	WDI_agriculture	Quantile-SFA	hqer_induced	True	27	-698.9668903642894	hqer_induced sampler prototype	2.0
	WDI_agriculture	HQER-SFA	hqer_induced	True	19	-698.9433478866986	hqer_induced sampler prototype	2.0
	WDI_agriculture	Quantile-SFA	hqer_induced	True	26	-706.4561585523843	hqer_induced sampler prototype	3.0
	WDI_agriculture	HQER-SFA	hqer_induced	True	21	-706.4373837551911	hqer_induced sampler prototype	2.0
	WDI_agriculture	Quantile-SFA	hqer_induced	True	25	-728.930704340454	hqer_induced sampler prototype	5.0
	WDI_agriculture	HQER-SFA	hqer_induced	True	17	-728.919354113832	hqer_induced sampler prototype	2.0
	WDI_agriculture	Quantile-SFA	hqer_induced	True	21	-711.6688434617744	hqer_induced sampler prototype	2.0
	WDI_agriculture	HQER-SFA	hqer_induced	True	20	-711.6672869069928	hqer_induced sampler prototype	2.0
	WDI_agriculture	Quantile-SFA	hqer_induced	True	47	-697.3856772717429	hqer_induced sampler prototype	1.0
	WDI_agriculture	HQER-SFA	hqer_induced	True	29	-697.3985254937669	hqer_induced sampler prototype	3.0
	WDI_agriculture	Quantile-SFA	hqer_induced	True	32	-710.1248638502706	hqer_induced sampler prototype	1.0
	WDI_agriculture	HQER-SFA	hqer_induced	True	26	-710.1023902680438	hqer_induced sampler prototype	1.0
	WDI_agriculture	Quantile-SFA	hqer_induced	True	28	-683.0259605652736	hqer_induced sampler prototype	1.0
	WDI_agriculture	HQER-SFA	hqer_induced	True	26	-682.9979422783183	hqer_induced sampler prototype	1.0
	WDI_agriculture	Quantile-SFA	hqer_induced	True	31	-690.9798038404664	hqer_induced sampler prototype	1.0
	WDI_agriculture	HQER-SFA	hqer_induced	True	22	-690.9640277717593	hqer_induced sampler prototype	1.0
	WDI_agriculture	Quantile-SFA	hqer_induced	True	20	-729.2294776086933	hqer_induced sampler prototype	5.0
	WDI_agriculture	HQER-SFA	hqer_induced	True	38	-728.8524717994911	hqer_induced sampler prototype	2.0
	WDI_agriculture	Quantile-SFA	hqer_induced	True	12	-725.0721415622879	hqer_induced sampler prototype	5.0
	WDI_agriculture	HQER-SFA	hqer_induced	True	20	-725.0451898663407	hqer_induced sampler prototype	5.0
	WDI_agriculture	Quantile-SFA	hqer_induced	True	32	-698.8875486696229	hqer_induced sampler prototype	3.0
	WDI_agriculture	HQER-SFA	hqer_induced	True	38	-698.8674106812316	hqer_induced sampler prototype	3.0
	WDI_agriculture	Quantile-SFA	hqer_induced	True	25	-702.019754626978	hqer_induced sampler prototype	2.0
	WDI_agriculture	HQER-SFA	hqer_induced	True	44	-702.0142044299298	hqer_induced sampler prototype	3.0
	WDI_agriculture	Quantile-SFA	hqer_induced	True	34	-696.3184502057145	hqer_induced sampler prototype	3.0
	WDI_agriculture	HQER-SFA	hqer_induced	True	29	-696.3138269487472	hqer_induced sampler prototype	5.0
	WDI_agriculture	Quantile-SFA	hqer_induced	True	9	-706.3517854971037	hqer_induced sampler prototype	2.0
	WDI_agriculture	HQER-SFA	hqer_induced	True	30	-706.2162662169015	hqer_induced sampler prototype	5.0
	WDI_agriculture	Quantile-SFA	hqer_induced	True	13	-727.1636293211186	hqer_induced sampler prototype	2.0
	WDI_agriculture	HQER-SFA	hqer_induced	True	10	-727.1507042158139	hqer_induced sampler prototype	2.0
	WDI_agriculture	Quantile-SFA	hqer_induced	True	44	-695.9930023049726	hqer_induced sampler prototype	3.0
	WDI_agriculture	HQER-SFA	hqer_induced	True	37	-695.9937008579769	hqer_induced sampler prototype	1.0
	WDI_agriculture	Quantile-SFA	hqer_induced	True	41	-719.2574276894411	hqer_induced sampler prototype	3.0
	WDI_agriculture	HQER-SFA	hqer_induced	True	38	-719.2024588996561	hqer_induced sampler prototype	3.0
	WDI_agriculture	Quantile-SFA	hqer_induced	True	11	-723.0377831883235	hqer_induced sampler prototype	2.0
	WDI_agriculture	HQER-SFA	hqer_induced	True	33	-723.0201787103579	hqer_induced sampler prototype	3.0
	WDI_agriculture	Quantile-SFA	hqer_induced	True	20	-714.5462026777009	hqer_induced sampler prototype	2.0
	WDI_agriculture	HQER-SFA	hqer_induced	True	21	-714.5334536963255	hqer_induced sampler prototype	2.0
	WDI_agriculture	Quantile-SFA	hqer_induced	True	28	-722.5375249520885	hqer_induced sampler prototype	3.0
	WDI_agriculture	HQER-SFA	hqer_induced	True	24	-722.5176721990766	hqer_induced sampler prototype	5.0
	WDI_agriculture	Quantile-SFA	hqer_induced	True	13	-703.9040243925763	hqer_induced sampler prototype	2.0
	WDI_agriculture	HQER-SFA	hqer_induced	True	9	-703.8857104454675	hqer_induced sampler prototype	2.0
	WDI_agriculture	Quantile-SFA	hqer_induced	True	31	-716.4424913565296	hqer_induced sampler prototype	3.0
	WDI_agriculture	HQER-SFA	hqer_induced	True	26	-716.4057541631878	hqer_induced sampler prototype	3.0
	WDI_agriculture	Quantile-SFA	hqer_induced	True	28	-723.9132526620675	hqer_induced sampler prototype	1.0
	WDI_agriculture	HQER-SFA	hqer_induced	True	20	-723.8934197860833	hqer_induced sampler prototype	2.0
	WDI_agriculture	Quantile-SFA	hqer_induced	True	37	-692.5736206157576	hqer_induced sampler prototype	3.0
	WDI_agriculture	HQER-SFA	hqer_induced	True	34	-692.5896264089636	hqer_induced sampler prototype	1.0
	WDI_agriculture	Quantile-SFA	hqer_induced	True	31	-722.4093984928635	hqer_induced sampler prototype	3.0
	WDI_agriculture	HQER-SFA	hqer_induced	True	36	-722.3836378506322	hqer_induced sampler prototype	1.0
	WDI_agriculture	Quantile-SFA	hqer_induced	True	33	-721.8227912063717	hqer_induced sampler prototype	5.0
	WDI_agriculture	HQER-SFA	hqer_induced	True	29	-721.8014946433411	hqer_induced sampler prototype	1.0
	WDI_agriculture	Quantile-SFA	hqer_induced	True	56	-714.9170900340032	hqer_induced sampler prototype	2.0
	WDI_agriculture	HQER-SFA	hqer_induced	True	39	-715.3598328792011	hqer_induced sampler prototype	1.0
	WDI_agriculture	Quantile-SFA	hqer_induced	True	35	-741.9341273635348	hqer_induced sampler prototype	5.0
	WDI_agriculture	HQER-SFA	hqer_induced	True	31	-741.8489656273396	hqer_induced sampler prototype	3.0
	WDI_agriculture	Quantile-SFA	hqer_induced	True	33	-700.03042734164	hqer_induced sampler prototype	3.0
	WDI_agriculture	HQER-SFA	hqer_induced	True	25	-700.1713359424151	hqer_induced sampler prototype	5.0
	WDI_agriculture	Quantile-SFA	hqer_induced	True	41	-703.7589868811904	hqer_induced sampler prototype	1.0
	WDI_agriculture	HQER-SFA	hqer_induced	True	25	-703.9855614437126	hqer_induced sampler prototype	3.0
	WDI_agriculture	Quantile-SFA	hqer_induced	True	38	-718.6298763049265	hqer_induced sampler prototype	3.0
	WDI_agriculture	HQER-SFA	hqer_induced	True	30	-718.612025389078	hqer_induced sampler prototype	2.0
	WDI_agriculture	Quantile-SFA	hqer_induced	True	32	-717.8311841959954	hqer_induced sampler prototype	5.0
	WDI_agriculture	HQER-SFA	hqer_induced	True	24	-717.8465777676568	hqer_induced sampler prototype	3.0
	WDI_agriculture	Quantile-SFA	hqer_induced	True	36	-717.8375791138607	hqer_induced sampler prototype	3.0
	WDI_agriculture	HQER-SFA	hqer_induced	True	30	-717.8027163109809	hqer_induced sampler prototype	3.0
	WDI_agriculture	Quantile-SFA	hqer_induced	True	38	-742.1101398471733	hqer_induced sampler prototype	1.0
	WDI_agriculture	HQER-SFA	hqer_induced	True	28	-742.0927306443799	hqer_induced sampler prototype	3.0
	WDI_agriculture	Quantile-SFA	hqer_induced	True	37	-727.0188317088323	hqer_induced sampler prototype	5.0
	WDI_agriculture	HQER-SFA	hqer_induced	True	43	-726.3962501995586	hqer_induced sampler prototype	5.0
	WDI_agriculture	Quantile-SFA	hqer_induced	True	32	-704.656929235784	hqer_induced sampler prototype	2.0
	WDI_agriculture	HQER-SFA	hqer_induced	True	29	-704.5850326674798	hqer_induced sampler prototype	2.0
	WDI_agriculture	Quantile-SFA	hqer_induced	True	29	-709.9271251034128	hqer_induced sampler prototype	2.0
	WDI_agriculture	HQER-SFA	hqer_induced	True	29	-709.8843693023966	hqer_induced sampler prototype	5.0

experiment	dataset	model	scenario	converged	n__iter	loglik	warning__message	best__start__id
	WDI_agriculture	Quantile-SFA	hqer_induced	True	57	-701.142859175613	hqer_induced sampler prototype	2.0
	WDI_agriculture	HQER-SFA	hqer_induced	True	48	-701.103808770481	hqer_induced sampler prototype	1.0
	WDI_agriculture	Quantile-SFA	hqer_induced	True	31	-728.9980485709957	hqer_induced sampler prototype	1.0
	WDI_agriculture	HQER-SFA	hqer_induced	True	30	-728.9448204142652	hqer_induced sampler prototype	1.0
	WDI_agriculture	Quantile-SFA	hqer_induced	True	32	-714.0417373026659	hqer_induced sampler prototype	3.0
	WDI_agriculture	HQER-SFA	hqer_induced	True	26	-714.0261446344496	hqer_induced sampler prototype	5.0
	WDI_agriculture	Quantile-SFA	hqer_induced	True	38	-694.6586129861164	hqer_induced sampler prototype	3.0
	WDI_agriculture	HQER-SFA	hqer_induced	True	28	-694.6519339208728	hqer_induced sampler prototype	1.0
	WDI_agriculture	Quantile-SFA	hqer_induced	True	37	-693.2056926771497	hqer_induced sampler prototype	1.0
	WDI_agriculture	HQER-SFA	hqer_induced	True	37	-693.2068242587566	hqer_induced sampler prototype	1.0
	WDI_agriculture	Quantile-SFA	hqer_induced	True	33	-739.7109045616468	hqer_induced sampler prototype	3.0
	WDI_agriculture	HQER-SFA	hqer_induced	True	25	-739.6951943927907	hqer_induced sampler prototype	2.0
	WDI_agriculture	Quantile-SFA	hqer_induced	True	30	-696.6837102627888	hqer_induced sampler prototype	2.0
	WDI_agriculture	HQER-SFA	hqer_induced	True	26	-696.6981464764292	hqer_induced sampler prototype	2.0
	WDI_agriculture	Quantile-SFA	hqer_induced	True	40	-734.322162662948	hqer_induced sampler prototype	3.0
	WDI_agriculture	HQER-SFA	hqer_induced	True	47	-734.2815835311044	hqer_induced sampler prototype	1.0
	WDI_agriculture	Quantile-SFA	hqer_induced	True	22	-714.7720805476126	hqer_induced sampler prototype	1.0
	WDI_agriculture	HQER-SFA	hqer_induced	True	23	-714.7695203035039	hqer_induced sampler prototype	1.0
	WDI_agriculture	Quantile-SFA	hqer_induced	True	23	-729.9991104718703	hqer_induced sampler prototype	2.0
	WDI_agriculture	HQER-SFA	hqer_induced	True	25	-729.9722444271782	hqer_induced sampler prototype	2.0
	WDI_agriculture	Quantile-SFA	hqer_induced	True	16	-723.6915346557105	hqer_induced sampler prototype	3.0
	WDI_agriculture	HQER-SFA	hqer_induced	True	22	-723.6125610555105	hqer_induced sampler prototype	1.0
	WDI_agriculture	Quantile-SFA	hqer_induced	True	11	-705.3706307639329	hqer_induced sampler prototype	2.0
	WDI_agriculture	HQER-SFA	hqer_induced	True	37	-704.569449370295	hqer_induced sampler prototype	1.0
	WDI_agriculture	Quantile-SFA	hqer_induced	True	23	-719.8859611700747	hqer_induced sampler prototype	2.0
	WDI_agriculture	HQER-SFA	hqer_induced	True	32	-719.8711853241066	hqer_induced sampler prototype	1.0
	WDI_agriculture	Quantile-SFA	hqer_induced	True	40	-728.4240111781471	hqer_induced sampler prototype	3.0
	WDI_agriculture	HQER-SFA	hqer_induced	True	34	-728.4087460629643	hqer_induced sampler prototype	1.0
	WDI_agriculture	Quantile-SFA	hqer_induced	True	36	-713.0123237633851	hqer_induced sampler prototype	5.0
	WDI_agriculture	HQER-SFA	hqer_induced	True	36	-713.0624459282074	hqer_induced sampler prototype	1.0
	WDI_agriculture	Quantile-SFA	hqer_induced	True	32	-745.5630928885987	hqer_induced sampler prototype	2.0
	WDI_agriculture	HQER-SFA	hqer_induced	True	23	-745.5477727523239	hqer_induced sampler prototype	5.0
	WDI_agriculture	Quantile-SFA	hqer_induced	True	24	-695.8852174126364	hqer_induced sampler prototype	3.0
	WDI_agriculture	HQER-SFA	hqer_induced	True	27	-695.8546132572342	hqer_induced sampler prototype	3.0
	WDI_agriculture	Quantile-SFA	hqer_induced	True	27	-717.1819929957026	hqer_induced sampler prototype	2.0
	WDI_agriculture	HQER-SFA	hqer_induced	True	19	-717.2328361623145	hqer_induced sampler prototype	2.0
	WDI_agriculture	Quantile-SFA	hqer_induced	True	15	-718.083652521024	hqer_induced sampler prototype	5.0
	WDI_agriculture	HQER-SFA	hqer_induced	True	26	-718.0528834665482	hqer_induced sampler prototype	3.0
	WDI_agriculture	Quantile-SFA	hqer_induced	True	25	-722.024693037002	hqer_induced sampler prototype	5.0
	WDI_agriculture	HQER-SFA	hqer_induced	True	21	-722.0316888621787	hqer_induced sampler prototype	2.0
	WDI_agriculture	Quantile-SFA	hqer_induced	True	28	-713.4507659668236	hqer_induced sampler prototype	1.0
	WDI_agriculture	HQER-SFA	hqer_induced	True	26	-713.4824387655822	hqer_induced sampler prototype	1.0
	WDI_agriculture	Quantile-SFA	hqer_induced	True	17	-706.5557106653689	hqer_induced sampler prototype	2.0
	WDI_agriculture	HQER-SFA	hqer_induced	True	22	-706.543223679362	hqer_induced sampler prototype	2.0
	WDI_agriculture	Quantile-SFA	hqer_induced	True	26	-708.8961812097302	hqer_induced sampler prototype	3.0
	WDI_agriculture	HQER-SFA	hqer_induced	True	27	-708.9263143437439	hqer_induced sampler prototype	1.0
	WDI_agriculture	Quantile-SFA	hqer_induced	True	14	-726.5684162757558	hqer_induced sampler prototype	4.0
	WDI_agriculture	HQER-SFA	hqer_induced	True	16	-726.4476748950804	hqer_induced sampler prototype	4.0
	WDI_agriculture	Quantile-SFA	hqer_induced	True	28	-701.5990219573914	hqer_induced sampler prototype	1.0
	WDI_agriculture	HQER-SFA	hqer_induced	True	27	-701.5465401820918	hqer_induced sampler prototype	3.0
	WDI_agriculture	Quantile-SFA	hqer_induced	True	26	-686.9134258438396	hqer_induced sampler prototype	3.0
	WDI_agriculture	HQER-SFA	hqer_induced	True	25	-686.9183557002852	hqer_induced sampler prototype	1.0
	WDI_agriculture	Quantile-SFA	hqer_induced	True	29	-692.0576416760974	hqer_induced sampler prototype	1.0
	WDI_agriculture	HQER-SFA	hqer_induced	True	35	-692.0781850016094	hqer_induced sampler prototype	3.0
	WDI_agriculture	Quantile-SFA	hqer_induced	True	25	-713.1636197063492	hqer_induced sampler prototype	1.0
	WDI_agriculture	HQER-SFA	hqer_induced	True	35	-713.1185876102545	hqer_induced sampler prototype	3.0
	WDI_agriculture	Quantile-SFA	hqer_induced	True	22	-714.2187846998834	hqer_induced sampler prototype	3.0
	WDI_agriculture	HQER-SFA	hqer_induced	True	29	-714.1797691052132	hqer_induced sampler prototype	1.0
	WDI_agriculture	Quantile-SFA	hqer_induced	True	32	-716.0072741784388	hqer_induced sampler prototype	5.0
	WDI_agriculture	HQER-SFA	hqer_induced	True	20	-716.1146601140301	hqer_induced sampler prototype	5.0
	WDI_agriculture	Quantile-SFA	hqer_induced	True	33	-695.8721725086882	hqer_induced sampler prototype	1.0
	WDI_agriculture	HQER-SFA	hqer_induced	True	29	-695.8485336106966	hqer_induced sampler prototype	1.0
	WDI_agriculture	Quantile-SFA	hqer_induced	True	39	-732.117413416195	hqer_induced sampler prototype	5.0
	WDI_agriculture	HQER-SFA	hqer_induced	True	11	-732.4822106798886	hqer_induced sampler prototype	3.0
	WDI_agriculture	Quantile-SFA	hqer_induced	True	30	-725.6516958026361	hqer_induced sampler prototype	2.0
	WDI_agriculture	HQER-SFA	hqer_induced	True	38	-725.6385029707346	hqer_induced sampler prototype	5.0
	WDI_agriculture	Quantile-SFA	hqer_induced	True	32	-701.2339015303824	hqer_induced sampler prototype	2.0
	WDI_agriculture	HQER-SFA	hqer_induced	True	25	-701.2144204542506	hqer_induced sampler prototype	5.0
	WDI_agriculture	Quantile-SFA	hqer_induced	True	19	-717.7598607828406	hqer_induced sampler prototype	1.0
	WDI_agriculture	HQER-SFA	hqer_induced	True	18	-717.7519276680799	hqer_induced sampler prototype	2.0
	WDI_agriculture	Quantile-SFA	hqer_induced	True	32	-711.0720363474102	hqer_induced sampler prototype	2.0
	WDI_agriculture	HQER-SFA	hqer_induced	True	29	-711.1562413255417	hqer_induced sampler prototype	3.0
	WDI_agriculture	Quantile-SFA	hqer_induced	True	20	-711.8599625210534	hqer_induced sampler prototype	5.0
	WDI_agriculture	HQER-SFA	hqer_induced	True	23	-711.861237633759	hqer_induced sampler prototype	5.0
	WDI_agriculture	Quantile-SFA	hqer_induced	True	30	-681.4814749051567	hqer_induced sampler prototype	5.0
	WDI_agriculture	HQER-SFA	hqer_induced	True	21	-681.4280266383455	hqer_induced sampler prototype	5.0
	WDI_agriculture	Quantile-SFA	hqer_induced	True	25	-712.3340248207521	hqer_induced sampler prototype	1.0
	WDI_agriculture	HQER-SFA	hqer_induced	True	25	-712.3077269419409	hqer_induced sampler prototype	1.0
	WDI_agriculture	Quantile-SFA	hqer_induced	True	18	-714.272744477213	hqer_induced sampler prototype	2.0
	WDI_agriculture	HQER-SFA	hqer_induced	True	14	-714.2531870314135	hqer_induced sampler prototype	2.0
	WDI_agriculture	Quantile-SFA	hqer_induced	True	28	-675.8540002548066	hqer_induced sampler prototype	2.0
	WDI_agriculture	HQER-SFA	hqer_induced	True	27	-675.8414254122484	hqer_induced sampler prototype	1.0
	WDI_agriculture	Quantile-SFA	hqer_induced	True	21	-723.5040140524388	hqer_induced sampler prototype	5.0
	WDI_agriculture	HQER-SFA	hqer_induced	True	9	-723.4855692584688	hqer_induced sampler prototype	2.0
	WDI_agriculture	Quantile-SFA	hqer_induced	True	27	-713.7937736542005	hqer_induced sampler prototype	3.0
	WDI_agriculture	HQER-SFA	hqer_induced	True	29	-713.798624522925	hqer_induced sampler prototype	3.0
	WDI_agriculture	Quantile-SFA	hqer_induced	True	42	-688.6747336616703	hqer_induced sampler prototype	3.0
	WDI_agriculture	HQER-SFA	hqer_induced	True	35	-688.6698626109198	hqer_induced sampler prototype	1.0
	WDI_agriculture	Quantile-SFA	hqer_induced	True	31	-736.8308397522853	hqer_induced sampler prototype	3.0
	WDI_agriculture	HQER-SFA	hqer_induced	True	27	-736.8249327439021	hqer_induced sampler prototype	2.0
	WDI_agriculture	Quantile-SFA	hqer_induced	True	26	-737.6896779479337	hqer_induced sampler prototype	1.0
	WDI_agriculture	HQER-SFA	hqer_induced	True	30	-737.6676764805223	hqer_induced sampler prototype	3.0
	WDI_agriculture	Quantile-SFA	hqer_induced	True	25	-724.335047675188	hqer_induced sampler prototype	1.0
	WDI_agriculture	HQER-SFA	hqer_induced	True	11	-724.2890748522055	hqer_induced sampler prototype	2.0
	WDI_agriculture	Quantile-SFA	hqer_induced	True	43	-700.3653880522581	hqer_induced sampler prototype	1.0
	WDI_agriculture	HQER-SFA	hqer_induced	True	41	-700.25698362706	hqer_induced sampler prototype	3.0
	WDI_agriculture	Quantile-SFA	hqer_induced	True	30	-709.6772393273691	hqer_induced sampler prototype	3.0
	WDI_agriculture	HQER-SFA	hqer_induced	True	33	-709.7040606761103	hqer_induced sampler prototype	1.0
	WDI_agriculture	Quantile-SFA	hqer_induced	True	17	-700.6307665831629	hqer_induced sampler prototype	5.0
	WDI_agriculture	HQER-SFA	hqer_induced	True	43	-700.6140811401962	hqer_induced sampler prototype	3.0
	WDI_agriculture	Quantile-SFA	hqer_induced	True	40	-707.8462388430105	hqer_induced sampler prototype	5.0
	WDI_agriculture	HQER-SFA	hqer_induced	True	34	-707.7493308498588	hqer_induced sampler prototype	2.0
	WDI_agriculture	Quantile-SFA	hqer_induced	True	25	-746.5374057527908	hqer_induced sampler prototype	3.0

experiment	dataset	model	scenario	converged	n__iter	loglik	warning__message	best__start__id
	WDI_agriculture	HQER-SFA	hqer_induced	True	30	-746.5201115595128	hqer_induced sampler prototype	3.0
	WDI_agriculture	Quantile-SFA	hqer_induced	True	30	-719.0033290663321	hqer_induced sampler prototype	3.0
	WDI_agriculture	HQER-SFA	hqer_induced	True	16	-718.9881873472477	hqer_induced sampler prototype	2.0
	WDI_agriculture	Quantile-SFA	hqer_induced	True	12	-709.5116590714895	hqer_induced sampler prototype	2.0
	WDI_agriculture	HQER-SFA	hqer_induced	True	12	-709.492224567924	hqer_induced sampler prototype	2.0
	WDI_agriculture	Quantile-SFA	hqer_induced	True	17	-695.7224951671469	hqer_induced sampler prototype	5.0
	WDI_agriculture	HQER-SFA	hqer_induced	True	20	-695.70081648088	hqer_induced sampler prototype	5.0
	WDI_agriculture	Quantile-SFA	hqer_induced	True	41	-726.0368487449139	hqer_induced sampler prototype	1.0
	WDI_agriculture	HQER-SFA	hqer_induced	True	19	-726.0207287246648	hqer_induced sampler prototype	5.0
	WDI_agriculture	Quantile-SFA	hqer_induced	True	30	-686.5665235870949	hqer_induced sampler prototype	1.0
	WDI_agriculture	HQER-SFA	hqer_induced	True	25	-686.5542686675976	hqer_induced sampler prototype	1.0
	WDI_agriculture	Quantile-SFA	hqer_induced	True	30	-714.9029996068452	hqer_induced sampler prototype	1.0
	WDI_agriculture	HQER-SFA	hqer_induced	True	23	-715.0249179865119	hqer_induced sampler prototype	3.0
	WDI_agriculture	Quantile-SFA	hqer_induced	True	28	-691.627309350804	hqer_induced sampler prototype	3.0
	WDI_agriculture	HQER-SFA	hqer_induced	True	23	-691.6265298238767	hqer_induced sampler prototype	2.0
	WDI_agriculture	Quantile-SFA	hqer_induced	True	24	-725.0766726007225	hqer_induced sampler prototype	5.0
	WDI_agriculture	HQER-SFA	hqer_induced	True	20	-725.0754770337057	hqer_induced sampler prototype	5.0
	WDI_agriculture	Quantile-SFA	hqer_induced	True	33	-715.0461247006122	hqer_induced sampler prototype	3.0
	WDI_agriculture	HQER-SFA	hqer_induced	True	29	-715.0302243768599	hqer_induced sampler prototype	3.0
	WDI_agriculture	Quantile-SFA	hqer_induced	True	29	-710.1274094326948	hqer_induced sampler prototype	1.0
	WDI_agriculture	HQER-SFA	hqer_induced	True	32	-710.1329640020745	hqer_induced sampler prototype	2.0
	WDI_agriculture	Quantile-SFA	hqer_induced	True	20	-748.3467609497354	hqer_induced sampler prototype	2.0
	WDI_agriculture	HQER-SFA	hqer_induced	True	26	-748.3715435435439	hqer_induced sampler prototype	2.0
	WDI_agriculture	Quantile-SFA	hqer_induced	True	27	-733.2680392732689	hqer_induced sampler prototype	3.0
	WDI_agriculture	HQER-SFA	hqer_induced	True	29	-733.2638600986651	hqer_induced sampler prototype	3.0
	WDI_agriculture	Quantile-SFA	hqer_induced	True	25	-720.414806728475	hqer_induced sampler prototype	5.0
	WDI_agriculture	HQER-SFA	hqer_induced	True	20	-720.411522855158	hqer_induced sampler prototype	2.0
	WDI_agriculture	Quantile-SFA	hqer_induced	True	28	-723.1328702542178	hqer_induced sampler prototype	3.0
	WDI_agriculture	HQER-SFA	hqer_induced	True	17	-723.1284808445652	hqer_induced sampler prototype	1.0
	WDI_agriculture	Quantile-SFA	hqer_induced	True	16	-712.160729339648	hqer_induced sampler prototype	4.0
	WDI_agriculture	HQER-SFA	hqer_induced	True	13	-712.1036032284819	hqer_induced sampler prototype	4.0
	WDI_agriculture	Quantile-SFA	hqer_induced	True	16	-744.2062649635598	hqer_induced sampler prototype	3.0
	WDI_agriculture	HQER-SFA	hqer_induced	True	28	-744.1893595336776	hqer_induced sampler prototype	5.0
	WDI_agriculture	Quantile-SFA	hqer_induced	True	29	-726.5133562592779	hqer_induced sampler prototype	5.0
	WDI_agriculture	HQER-SFA	hqer_induced	True	37	-726.4918415014058	hqer_induced sampler prototype	3.0
	WDI_agriculture	Quantile-SFA	hqer_induced	True	26	-708.4471057688214	hqer_induced sampler prototype	2.0
	WDI_agriculture	HQER-SFA	hqer_induced	True	31	-708.4308475951374	hqer_induced sampler prototype	2.0
	WDI_agriculture	Quantile-SFA	hqer_induced	True	57	-714.8277451907002	hqer_induced sampler prototype	3.0
	WDI_agriculture	HQER-SFA	hqer_induced	True	46	-714.8465066370854	hqer_induced sampler prototype	3.0
	WDI_agriculture	Quantile-SFA	hqer_induced	True	20	-725.744522843787	hqer_induced sampler prototype	5.0
	WDI_agriculture	HQER-SFA	hqer_induced	True	17	-725.69489749069	hqer_induced sampler prototype	5.0
	WDI_agriculture	Quantile-SFA	hqer_induced	True	38	-690.3381570545328	hqer_induced sampler prototype	3.0
	WDI_agriculture	HQER-SFA	hqer_induced	True	24	-690.3161696483367	hqer_induced sampler prototype	3.0
	WDI_agriculture	Quantile-SFA	hqer_induced	True	42	-709.0507475482076	hqer_induced sampler prototype	3.0
	WDI_agriculture	HQER-SFA	hqer_induced	True	32	-709.434677462562	hqer_induced sampler prototype	1.0
	WDI_agriculture	Quantile-SFA	hqer_induced	True	22	-671.5176497167636	hqer_induced sampler prototype	3.0
	WDI_agriculture	HQER-SFA	hqer_induced	True	25	-671.4925464095005	hqer_induced sampler prototype	3.0
	WDI_agriculture	Quantile-SFA	hqer_induced	True	19	-699.9801160437357	hqer_induced sampler prototype	5.0
	WDI_agriculture	HQER-SFA	hqer_induced	True	17	-699.9785314506191	hqer_induced sampler prototype	5.0
	WDI_agriculture	Quantile-SFA	hqer_induced	True	46	-739.5021489191137	hqer_induced sampler prototype	1.0
	WDI_agriculture	HQER-SFA	hqer_induced	True	45	-739.4970580009714	hqer_induced sampler prototype	3.0
	WDI_agriculture	Quantile-SFA	hqer_induced	True	29	-708.2302006456105	hqer_induced sampler prototype	1.0
	WDI_agriculture	HQER-SFA	hqer_induced	True	23	-708.2658495266685	hqer_induced sampler prototype	1.0
	WDI_agriculture	Quantile-SFA	hqer_induced	True	17	-716.1575999680538	hqer_induced sampler prototype	2.0
	WDI_agriculture	HQER-SFA	hqer_induced	True	16	-716.1392912850798	hqer_induced sampler prototype	2.0
	WDI_agriculture	Quantile-SFA	hqer_induced	True	21	-714.0547188289438	hqer_induced sampler prototype	5.0
	WDI_agriculture	HQER-SFA	hqer_induced	True	21	-713.97282038863	hqer_induced sampler prototype	2.0
	WDI_agriculture	Quantile-SFA	hqer_induced	True	28	-737.2572513492452	hqer_induced sampler prototype	3.0
	WDI_agriculture	HQER-SFA	hqer_induced	True	23	-737.2264370635978	hqer_induced sampler prototype	3.0
	WDI_agriculture	Quantile-SFA	hqer_induced	True	25	-703.7036793468196	hqer_induced sampler prototype	1.0
	WDI_agriculture	HQER-SFA	hqer_induced	True	15	-703.6953022136767	hqer_induced sampler prototype	2.0
	WDI_agriculture	Quantile-SFA	hqer_induced	True	20	-729.616285936494	hqer_induced sampler prototype	5.0
	WDI_agriculture	HQER-SFA	hqer_induced	True	30	-729.5803073420107	hqer_induced sampler prototype	1.0
	WDI_agriculture	Quantile-SFA	hqer_induced	True	25	-711.1580136758566	hqer_induced sampler prototype	2.0
	WDI_agriculture	HQER-SFA	hqer_induced	True	38	-711.1506861209017	hqer_induced sampler prototype	1.0
	WDI_agriculture	Quantile-SFA	hqer_induced	True	20	-722.3003324798608	hqer_induced sampler prototype	2.0
	WDI_agriculture	HQER-SFA	hqer_induced	True	25	-722.2843038188971	hqer_induced sampler prototype	2.0
	WDI_agriculture	Quantile-SFA	hqer_induced	True	32	-678.723823539389	hqer_induced sampler prototype	3.0
	WDI_agriculture	HQER-SFA	hqer_induced	True	21	-678.6865915007622	hqer_induced sampler prototype	2.0
	WDI_agriculture	Quantile-SFA	hqer_induced	True	33	-696.7941805332182	hqer_induced sampler prototype	1.0
	WDI_agriculture	HQER-SFA	hqer_induced	True	43	-696.7720636361707	hqer_induced sampler prototype	1.0
	WDI_agriculture	Quantile-SFA	hqer_induced	True	26	-709.1725549962093	hqer_induced sampler prototype	5.0
	WDI_agriculture	HQER-SFA	hqer_induced	True	20	-709.165067126004	hqer_induced sampler prototype	2.0
	WDI_agriculture	Quantile-SFA	hqer_induced	True	18	-708.7245038431986	hqer_induced sampler prototype	2.0
	WDI_agriculture	HQER-SFA	hqer_induced	True	13	-708.7051899466385	hqer_induced sampler prototype	2.0
	WDI_agriculture	Quantile-SFA	hqer_induced	True	32	-731.4030972104088	hqer_induced sampler prototype	1.0
	WDI_agriculture	HQER-SFA	hqer_induced	True	34	-731.3866146651054	hqer_induced sampler prototype	1.0
	WDI_agriculture	Quantile-SFA	hqer_induced	True	27	-721.0694947915466	hqer_induced sampler prototype	1.0
	WDI_agriculture	HQER-SFA	hqer_induced	True	22	-721.071928271264	hqer_induced sampler prototype	5.0
	WDI_agriculture	Quantile-SFA	hqer_induced	True	13	-684.735617195307	hqer_induced sampler prototype	5.0
	WDI_agriculture	HQER-SFA	hqer_induced	True	13	-684.7211602812961	hqer_induced sampler prototype	5.0
	WDI_agriculture	Quantile-SFA	hqer_induced	True	18	-734.8535011443748	hqer_induced sampler prototype	5.0
	WDI_agriculture	HQER-SFA	hqer_induced	True	20	-734.8441213240583	hqer_induced sampler prototype	5.0
	WDI_agriculture	Quantile-SFA	hqer_induced	True	17	-697.2903946369574	hqer_induced sampler prototype	2.0
	WDI_agriculture	HQER-SFA	hqer_induced	True	11	-697.3087831545862	hqer_induced sampler prototype	2.0
	WDI_agriculture	Quantile-SFA	hqer_induced	True	32	-704.3519222987679	hqer_induced sampler prototype	3.0
	WDI_agriculture	HQER-SFA	hqer_induced	True	31	-704.3230033219501	hqer_induced sampler prototype	1.0
	WDI_agriculture	Quantile-SFA	hqer_induced	True	19	-671.6211732595727	hqer_induced sampler prototype	2.0
	WDI_agriculture	HQER-SFA	hqer_induced	True	37	-671.4512302052947	hqer_induced sampler prototype	2.0
	WDI_agriculture	Quantile-SFA	hqer_induced	True	15	-756.4559193749396	hqer_induced sampler prototype	2.0
	WDI_agriculture	HQER-SFA	hqer_induced	True	33	-756.4346815479189	hqer_induced sampler prototype	1.0
	WDI_agriculture	Quantile-SFA	hqer_induced	True	20	-729.9325390868321	hqer_induced sampler prototype	5.0
	WDI_agriculture	HQER-SFA	hqer_induced	True	15	-729.9322690324734	hqer_induced sampler prototype	2.0
	WDI_agriculture	Quantile-SFA	hqer_induced	True	36	-702.380099872802	hqer_induced sampler prototype	1.0
	WDI_agriculture	HQER-SFA	hqer_induced	True	25	-702.361771215381	hqer_induced sampler prototype	2.0
	WDI_agriculture	Quantile-SFA	hqer_induced	True	25	-732.9077845314271	hqer_induced sampler prototype	3.0
	WDI_agriculture	HQER-SFA	hqer_induced	True	29	-732.8977393300528	hqer_induced sampler prototype	3.0
	WDI_agriculture	Quantile-SFA	hqer_induced	True	38	-731.178939339193	hqer_induced sampler prototype	1.0
	WDI_agriculture	HQER-SFA	hqer_induced	True	47	-731.6990507705002	hqer_induced sampler prototype	3.0
	WDI_agriculture	Quantile-SFA	hqer_induced	True	26	-742.4054365717135	hqer_induced sampler prototype	1.0
	WDI_agriculture	HQER-SFA	hqer_induced	True	30	-742.38458069324	hqer_induced sampler prototype	1.0
	WDI_agriculture	Quantile-SFA	hqer_induced	True	31	-731.3123979528141	hqer_induced sampler prototype	3.0
	WDI_agriculture	HQER-SFA	hqer_induced	True	22	-731.6657387424267	hqer_induced sampler prototype	2.0

experiment	dataset	model	scenario	converged	n__iter	loglik	warning__message	best__start__id
	WDI__agriculture	Quantile-SFA	hqer__induced	True	24	-717.097425002492	hqer__induced sampler prototype	1.0
	WDI__agriculture	HQER-SFA	hqer__induced	True	25	-717.0857813169384	hqer__induced sampler prototype	2.0
	WDI__agriculture	Quantile-SFA	hqer__induced	True	17	-719.1181008877652	hqer__induced sampler prototype	2.0
	WDI__agriculture	HQER-SFA	hqer__induced	True	22	-719.1166136280473	hqer__induced sampler prototype	5.0
	WDI__agriculture	Quantile-SFA	hqer__induced	True	23	-706.2208882983211	hqer__induced sampler prototype	1.0
	WDI__agriculture	HQER-SFA	hqer__induced	True	11	-706.378219669245	hqer__induced sampler prototype	2.0
	WDI__agriculture	Quantile-SFA	hqer__induced	True	36	-692.1881091143741	hqer__induced sampler prototype	1.0
	WDI__agriculture	HQER-SFA	hqer__induced	True	29	-692.1840408779516	hqer__induced sampler prototype	3.0
	WDI__agriculture	Quantile-SFA	hqer__induced	True	31	-736.7951344967777	hqer__induced sampler prototype	1.0
	WDI__agriculture	HQER-SFA	hqer__induced	True	29	-736.7801906325597	hqer__induced sampler prototype	3.0
	WDI__agriculture	Quantile-SFA	hqer__induced	True	33	-726.6025634326077	hqer__induced sampler prototype	3.0
	WDI__agriculture	HQER-SFA	hqer__induced	True	20	-726.5135786350955	hqer__induced sampler prototype	1.0
	WDI__agriculture	Quantile-SFA	hqer__induced	True	40	-699.4832304029453	hqer__induced sampler prototype	3.0
	WDI__agriculture	HQER-SFA	hqer__induced	True	34	-699.4620371099753	hqer__induced sampler prototype	3.0
	WDI__agriculture	Quantile-SFA	hqer__induced	True	30	-712.885242415857	hqer__induced sampler prototype	5.0
	WDI__agriculture	HQER-SFA	hqer__induced	True	26	-712.8516356762918	hqer__induced sampler prototype	2.0
	WDI__agriculture	Quantile-SFA	hqer__induced	True	11	-699.2873353231639	hqer__induced sampler prototype	2.0
	WDI__agriculture	HQER-SFA	hqer__induced	True	14	-699.2764763175037	hqer__induced sampler prototype	2.0
	WDI__agriculture	Quantile-SFA	hqer__induced	True	36	-720.533539298088	hqer__induced sampler prototype	1.0
	WDI__agriculture	HQER-SFA	hqer__induced	True	31	-720.6584451593219	hqer__induced sampler prototype	1.0
	WDI__agriculture	Quantile-SFA	hqer__induced	True	23	-718.113731407954	hqer__induced sampler prototype	3.0
	WDI__agriculture	HQER-SFA	hqer__induced	True	21	-718.0782382869577	hqer__induced sampler prototype	3.0
	WDI__agriculture	Quantile-SFA	hqer__induced	True	10	-737.2273914109189	hqer__induced sampler prototype	2.0
	WDI__agriculture	HQER-SFA	hqer__induced	True	16	-737.2193111905272	hqer__induced sampler prototype	2.0
	WDI__agriculture	Quantile-SFA	hqer__induced	True	43	-699.4076110897656	hqer__induced sampler prototype	3.0
	WDI__agriculture	HQER-SFA	hqer__induced	True	44	-699.3987516351594	hqer__induced sampler prototype	1.0
	WDI__agriculture	Quantile-SFA	hqer__induced	True	37	-711.2247319439696	hqer__induced sampler prototype	3.0
	WDI__agriculture	HQER-SFA	hqer__induced	True	29	-711.1505064024336	hqer__induced sampler prototype	1.0
	WDI__agriculture	Quantile-SFA	hqer__induced	True	21	-708.147716463088	hqer__induced sampler prototype	1.0
	WDI__agriculture	HQER-SFA	hqer__induced	True	24	-708.142463797903	hqer__induced sampler prototype	3.0
	WDI__agriculture	Quantile-SFA	hqer__induced	True	28	-724.5783732459872	hqer__induced sampler prototype	3.0
	WDI__agriculture	HQER-SFA	hqer__induced	True	24	-724.553747445856	hqer__induced sampler prototype	5.0
	WDI__agriculture	Quantile-SFA	hqer__induced	True	28	-727.8186578587408	hqer__induced sampler prototype	3.0
	WDI__agriculture	HQER-SFA	hqer__induced	True	31	-727.7754364724408	hqer__induced sampler prototype	3.0
	WDI__agriculture	Quantile-SFA	hqer__induced	True	28	-758.0033013231451	hqer__induced sampler prototype	3.0
	WDI__agriculture	HQER-SFA	hqer__induced	True	25	-758.2688610637937	hqer__induced sampler prototype	3.0
	WDI__agriculture	Quantile-SFA	hqer__induced	True	24	-723.6961387326796	hqer__induced sampler prototype	5.0
	WDI__agriculture	HQER-SFA	hqer__induced	True	26	-723.5575195720211	hqer__induced sampler prototype	2.0
	WDI__agriculture	Quantile-SFA	hqer__induced	True	43	-675.1096271886071	hqer__induced sampler prototype	3.0
	WDI__agriculture	HQER-SFA	hqer__induced	True	11	-675.2406256928964	hqer__induced sampler prototype	2.0
	WDI__agriculture	Quantile-SFA	hqer__induced	True	19	-694.0251214434093	hqer__induced sampler prototype	2.0
	WDI__agriculture	HQER-SFA	hqer__induced	True	14	-694.0204879124132	hqer__induced sampler prototype	2.0
	WDI__agriculture	Quantile-SFA	hqer__induced	True	22	-708.0146591712694	hqer__induced sampler prototype	5.0
	WDI__agriculture	HQER-SFA	hqer__induced	True	33	-707.7747343832801	hqer__induced sampler prototype	5.0
	WDI__agriculture	Quantile-SFA	hqer__induced	True	12	-708.1005300773928	hqer__induced sampler prototype	2.0
	WDI__agriculture	HQER-SFA	hqer__induced	True	20	-708.0500296529763	hqer__induced sampler prototype	2.0
	WDI__agriculture	Quantile-SFA	hqer__induced	True	23	-711.2111810427709	hqer__induced sampler prototype	2.0
	WDI__agriculture	HQER-SFA	hqer__induced	True	23	-711.1974707767761	hqer__induced sampler prototype	5.0
	WDI__agriculture	Quantile-SFA	hqer__induced	True	28	-731.8198916654579	hqer__induced sampler prototype	3.0
	WDI__agriculture	HQER-SFA	hqer__induced	True	32	-731.8261952691308	hqer__induced sampler prototype	1.0
	WDI__agriculture	Quantile-SFA	hqer__induced	True	24	-716.6469423693832	hqer__induced sampler prototype	3.0
	WDI__agriculture	HQER-SFA	hqer__induced	True	27	-716.6324033103915	hqer__induced sampler prototype	1.0
	WDI__agriculture	Quantile-SFA	hqer__induced	True	33	-692.3791635894636	hqer__induced sampler prototype	3.0
	WDI__agriculture	HQER-SFA	hqer__induced	True	36	-692.347754155581	hqer__induced sampler prototype	5.0
	WDI__agriculture	Quantile-SFA	hqer__induced	True	52	-724.4658544884982	hqer__induced sampler prototype	1.0
	WDI__agriculture	HQER-SFA	hqer__induced	True	22	-724.4493439266711	hqer__induced sampler prototype	2.0
	WDI__agriculture	Quantile-SFA	hqer__induced	True	30	-706.0192780902547	hqer__induced sampler prototype	3.0
	WDI__agriculture	HQER-SFA	hqer__induced	True	26	-706.0064901719245	hqer__induced sampler prototype	5.0
	WDI__agriculture	Quantile-SFA	hqer__induced	True	30	-728.3150066400427	hqer__induced sampler prototype	1.0
	WDI__agriculture	HQER-SFA	hqer__induced	True	25	-728.2880287897658	hqer__induced sampler prototype	3.0
	WDI__agriculture	Quantile-SFA	hqer__induced	True	20	-702.1618432505724	hqer__induced sampler prototype	5.0
	WDI__agriculture	HQER-SFA	hqer__induced	True	32	-702.1370913996576	hqer__induced sampler prototype	3.0
	WDI__agriculture	Quantile-SFA	hqer__induced	True	31	-694.1166082500872	hqer__induced sampler prototype	1.0
	WDI__agriculture	HQER-SFA	hqer__induced	True	33	-694.1159849125272	hqer__induced sampler prototype	5.0
	WDI__agriculture	Quantile-SFA	hqer__induced	True	28	-718.038986880134	hqer__induced sampler prototype	3.0
	WDI__agriculture	HQER-SFA	hqer__induced	True	26	-718.004408127729	hqer__induced sampler prototype	1.0
	WDI__agriculture	Quantile-SFA	hqer__induced	True	18	-2839.943456547283	hqer__induced sampler prototype	5.0
	WDI__agriculture	HQER-SFA	hqer__induced	True	8	-2839.892625104957	hqer__induced sampler prototype	2.0
	WDI__agriculture	Quantile-SFA	hqer__induced	True	12	-2857.2472774668145	hqer__induced sampler prototype	5.0
	WDI__agriculture	HQER-SFA	hqer__induced	True	16	-2857.114563899386	hqer__induced sampler prototype	2.0
	WDI__agriculture	Quantile-SFA	hqer__induced	True	20	-2930.5531585991657	hqer__induced sampler prototype	5.0
	WDI__agriculture	HQER-SFA	hqer__induced	True	27	-2930.007386011158	hqer__induced sampler prototype	1.0
	WDI__agriculture	Quantile-SFA	hqer__induced	True	25	-2793.503033971057	hqer__induced sampler prototype	2.0
	WDI__agriculture	HQER-SFA	hqer__induced	True	25	-2793.4014335776915	hqer__induced sampler prototype	3.0
	WDI__agriculture	Quantile-SFA	hqer__induced	True	17	-2939.274962861738	hqer__induced sampler prototype	2.0
	WDI__agriculture	HQER-SFA	hqer__induced	True	23	-2939.1959888455303	hqer__induced sampler prototype	5.0
	WDI__agriculture	Quantile-SFA	hqer__induced	True	27	-2868.80644582284	hqer__induced sampler prototype	5.0
	WDI__agriculture	HQER-SFA	hqer__induced	True	23	-2868.7759764191483	hqer__induced sampler prototype	5.0
	WDI__agriculture	Quantile-SFA	hqer__induced	True	20	-2884.8562737778384	hqer__induced sampler prototype	5.0
	WDI__agriculture	HQER-SFA	hqer__induced	True	22	-2884.819872152479	hqer__induced sampler prototype	1.0
	WDI__agriculture	Quantile-SFA	hqer__induced	True	21	-2809.7365212740256	hqer__induced sampler prototype	5.0
	WDI__agriculture	HQER-SFA	hqer__induced	True	22	-2809.6756925238	hqer__induced sampler prototype	1.0
	WDI__agriculture	Quantile-SFA	hqer__induced	True	20	-2889.7861296081537	hqer__induced sampler prototype	2.0
	WDI__agriculture	HQER-SFA	hqer__induced	True	32	-2889.5829337885293	hqer__induced sampler prototype	1.0
	WDI__agriculture	Quantile-SFA	hqer__induced	True	21	-2901.724722253879	hqer__induced sampler prototype	3.0
	WDI__agriculture	HQER-SFA	hqer__induced	True	20	-2901.660363348813	hqer__induced sampler prototype	2.0
	WDI__agriculture	Quantile-SFA	hqer__induced	True	29	-2834.473509544763	hqer__induced sampler prototype	1.0
	WDI__agriculture	HQER-SFA	hqer__induced	True	15	-2834.405169066209	hqer__induced sampler prototype	2.0
	WDI__agriculture	Quantile-SFA	hqer__induced	True	26	-2885.143705370062	hqer__induced sampler prototype	3.0
	WDI__agriculture	HQER-SFA	hqer__induced	True	14	-2885.138901806867	hqer__induced sampler prototype	5.0
	WDI__agriculture	Quantile-SFA	hqer__induced	True	25	-2821.6915452060925	hqer__induced sampler prototype	1.0
	WDI__agriculture	HQER-SFA	hqer__induced	True	13	-2821.6311982601696	hqer__induced sampler prototype	3.0
	WDI__agriculture	Quantile-SFA	hqer__induced	True	24	-2895.910393497544	hqer__induced sampler prototype	3.0
	WDI__agriculture	HQER-SFA	hqer__induced	True	28	-2895.825828619075	hqer__induced sampler prototype	1.0
	WDI__agriculture	Quantile-SFA	hqer__induced	True	24	-2822.706515389864	hqer__induced sampler prototype	1.0
	WDI__agriculture	HQER-SFA	hqer__induced	True	25	-2822.70583486163	hqer__induced sampler prototype	1.0
	WDI__agriculture	Quantile-SFA	hqer__induced	True	23	-2884.718622562699	hqer__induced sampler prototype	1.0
	WDI__agriculture	HQER-SFA	hqer__induced	True	30	-2884.6514608606035	hqer__induced sampler prototype	1.0
	WDI__agriculture	Quantile-SFA	hqer__induced	True	27	-2901.9638047029275	hqer__induced sampler prototype	3.0
	WDI__agriculture	HQER-SFA	hqer__induced	True	21	-2901.9081786320567	hqer__induced sampler prototype	1.0
	WDI__agriculture	Quantile-SFA	hqer__induced	True	25	-2891.923256363439	hqer__induced sampler prototype	2.0
	WDI__agriculture	HQER-SFA	hqer__induced	True	26	-2891.550159201581	hqer__induced sampler prototype	3.0
	WDI__agriculture	Quantile-SFA	hqer__induced	True	25	-2888.61906572856	hqer__induced sampler prototype	1.0

experiment	dataset	model	scenario	converged	n_iter	loglik	warning_message	best_start_id
	WDI_agriculture	HQER-SFA	hqer_induced	True	23	-2888.516912073429	hqer_induced sampler prototype	2.0
	WDI_agriculture	Quantile-SFA	hqer_induced	True	16	-2866.758281482677	hqer_induced sampler prototype	5.0
	WDI_agriculture	HQER-SFA	hqer_induced	True	19	-2866.6012799774385	hqer_induced sampler prototype	2.0
	WDI_agriculture	Quantile-SFA	hqer_induced	True	23	-2878.769622847816	hqer_induced sampler prototype	3.0
	WDI_agriculture	HQER-SFA	hqer_induced	True	19	-2878.695901632562	hqer_induced sampler prototype	2.0
	WDI_agriculture	Quantile-SFA	hqer_induced	True	35	-2865.7019300739976	hqer_induced sampler prototype	1.0
	WDI_agriculture	HQER-SFA	hqer_induced	True	22	-2865.7886255511485	hqer_induced sampler prototype	3.0
	WDI_agriculture	Quantile-SFA	hqer_induced	True	22	-2900.831890528557	hqer_induced sampler prototype	2.0
	WDI_agriculture	HQER-SFA	hqer_induced	True	23	-2900.759918302437	hqer_induced sampler prototype	5.0
	WDI_agriculture	Quantile-SFA	hqer_induced	True	19	-2879.812245137032	hqer_induced sampler prototype	3.0
	WDI_agriculture	HQER-SFA	hqer_induced	True	19	-2879.7877269319897	hqer_induced sampler prototype	1.0
	WDI_agriculture	Quantile-SFA	hqer_induced	True	24	-2913.4232874327427	hqer_induced sampler prototype	3.0
	WDI_agriculture	HQER-SFA	hqer_induced	True	23	-2913.321378559848	hqer_induced sampler prototype	1.0
	WDI_agriculture	Quantile-SFA	hqer_induced	True	22	-2815.859320508389	hqer_induced sampler prototype	5.0
	WDI_agriculture	HQER-SFA	hqer_induced	True	19	-2815.990289983806	hqer_induced sampler prototype	5.0
	WDI_agriculture	Quantile-SFA	hqer_induced	True	22	-2893.6376151830127	hqer_induced sampler prototype	1.0
	WDI_agriculture	HQER-SFA	hqer_induced	True	16	-2893.6090776811725	hqer_induced sampler prototype	2.0
	WDI_agriculture	Quantile-SFA	hqer_induced	True	26	-2863.351841276215	hqer_induced sampler prototype	1.0
	WDI_agriculture	HQER-SFA	hqer_induced	True	13	-2863.2786513246574	hqer_induced sampler prototype	2.0
	WDI_agriculture	Quantile-SFA	hqer_induced	True	16	-2836.979826832061	hqer_induced sampler prototype	5.0
	WDI_agriculture	HQER-SFA	hqer_induced	True	15	-2836.885193591632	hqer_induced sampler prototype	5.0
	WDI_agriculture	Quantile-SFA	hqer_induced	True	30	-2859.912665636872	hqer_induced sampler prototype	3.0
	WDI_agriculture	HQER-SFA	hqer_induced	True	32	-2859.8598065975484	hqer_induced sampler prototype	3.0
	WDI_agriculture	Quantile-SFA	hqer_induced	True	37	-2862.919380470473	hqer_induced sampler prototype	1.0
	WDI_agriculture	HQER-SFA	hqer_induced	True	25	-2862.9195945050924	hqer_induced sampler prototype	3.0
	WDI_agriculture	Quantile-SFA	hqer_induced	True	26	-2857.34971376732	hqer_induced sampler prototype	3.0
	WDI_agriculture	HQER-SFA	hqer_induced	True	25	-2857.239713481993	hqer_induced sampler prototype	1.0
	WDI_agriculture	Quantile-SFA	hqer_induced	True	17	-2802.582614327664	hqer_induced sampler prototype	2.0
	WDI_agriculture	HQER-SFA	hqer_induced	True	28	-2802.502553495916	hqer_induced sampler prototype	1.0
	WDI_agriculture	Quantile-SFA	hqer_induced	True	18	-2842.2089835746565	hqer_induced sampler prototype	1.0
	WDI_agriculture	HQER-SFA	hqer_induced	True	24	-2842.1155979615414	hqer_induced sampler prototype	1.0
	WDI_agriculture	Quantile-SFA	hqer_induced	True	23	-2807.1675983091336	hqer_induced sampler prototype	3.0
	WDI_agriculture	HQER-SFA	hqer_induced	True	28	-2807.129463970514	hqer_induced sampler prototype	3.0
	WDI_agriculture	Quantile-SFA	hqer_induced	True	23	-2846.5300972965406	hqer_induced sampler prototype	1.0
	WDI_agriculture	HQER-SFA	hqer_induced	True	26	-2846.4535123271303	hqer_induced sampler prototype	5.0
	WDI_agriculture	Quantile-SFA	hqer_induced	True	17	-2862.764844439444	hqer_induced sampler prototype	5.0
	WDI_agriculture	HQER-SFA	hqer_induced	True	31	-2862.7266373763305	hqer_induced sampler prototype	3.0
	WDI_agriculture	Quantile-SFA	hqer_induced	True	26	-2855.567197088193	hqer_induced sampler prototype	1.0
	WDI_agriculture	HQER-SFA	hqer_induced	True	19	-2855.471120126721	hqer_induced sampler prototype	1.0
	WDI_agriculture	Quantile-SFA	hqer_induced	True	22	-2832.2614692713	hqer_induced sampler prototype	1.0
	WDI_agriculture	HQER-SFA	hqer_induced	True	29	-2832.191012684437	hqer_induced sampler prototype	1.0
	WDI_agriculture	Quantile-SFA	hqer_induced	True	22	-2841.401602514926	hqer_induced sampler prototype	5.0
	WDI_agriculture	HQER-SFA	hqer_induced	True	19	-2841.340945822092	hqer_induced sampler prototype	1.0
	WDI_agriculture	Quantile-SFA	hqer_induced	True	21	-2887.1831278051764	hqer_induced sampler prototype	3.0
	WDI_agriculture	HQER-SFA	hqer_induced	True	19	-2887.1200976792325	hqer_induced sampler prototype	5.0
	WDI_agriculture	Quantile-SFA	hqer_induced	True	8	-2834.8047251362486	hqer_induced sampler prototype	2.0
	WDI_agriculture	HQER-SFA	hqer_induced	True	5	-2834.7915819837363	hqer_induced sampler prototype	2.0
	WDI_agriculture	Quantile-SFA	hqer_induced	True	25	-2831.1029203424578	hqer_induced sampler prototype	1.0
	WDI_agriculture	HQER-SFA	hqer_induced	True	28	-2831.0642183004115	hqer_induced sampler prototype	1.0
	WDI_agriculture	Quantile-SFA	hqer_induced	True	18	-2869.508325881443	hqer_induced sampler prototype	5.0
	WDI_agriculture	HQER-SFA	hqer_induced	True	29	-2869.4605169821416	hqer_induced sampler prototype	1.0
	WDI_agriculture	Quantile-SFA	hqer_induced	True	26	-2888.310528841687	hqer_induced sampler prototype	2.0
	WDI_agriculture	HQER-SFA	hqer_induced	True	29	-2888.1788661832416	hqer_induced sampler prototype	5.0
	WDI_agriculture	Quantile-SFA	hqer_induced	True	25	-2856.8537056138384	hqer_induced sampler prototype	3.0
	WDI_agriculture	HQER-SFA	hqer_induced	True	20	-2856.82245492478	hqer_induced sampler prototype	3.0
	WDI_agriculture	Quantile-SFA	hqer_induced	True	25	-2844.9492971198506	hqer_induced sampler prototype	1.0
	WDI_agriculture	HQER-SFA	hqer_induced	True	20	-2844.919090139821	hqer_induced sampler prototype	3.0
	WDI_agriculture	Quantile-SFA	hqer_induced	True	23	-2877.395197564582	hqer_induced sampler prototype	1.0
	WDI_agriculture	HQER-SFA	hqer_induced	True	27	-2877.323266200815	hqer_induced sampler prototype	1.0
	WDI_agriculture	Quantile-SFA	hqer_induced	True	24	-2881.063112063008	hqer_induced sampler prototype	1.0
	WDI_agriculture	HQER-SFA	hqer_induced	True	25	-2880.9984372490685	hqer_induced sampler prototype	3.0
	WDI_agriculture	Quantile-SFA	hqer_induced	True	20	-2798.6335088004803	hqer_induced sampler prototype	3.0
	WDI_agriculture	HQER-SFA	hqer_induced	True	25	-2798.57458674605	hqer_induced sampler prototype	2.0
	WDI_agriculture	Quantile-SFA	hqer_induced	True	23	-2858.080921429008	hqer_induced sampler prototype	3.0
	WDI_agriculture	HQER-SFA	hqer_induced	True	23	-2858.126932319329	hqer_induced sampler prototype	5.0
	WDI_agriculture	Quantile-SFA	hqer_induced	True	13	-2898.800022840184	hqer_induced sampler prototype	2.0
	WDI_agriculture	HQER-SFA	hqer_induced	True	27	-2898.691649608836	hqer_induced sampler prototype	1.0
	WDI_agriculture	Quantile-SFA	hqer_induced	True	18	-2813.697076960761	hqer_induced sampler prototype	3.0
	WDI_agriculture	HQER-SFA	hqer_induced	True	33	-2813.259819757357	hqer_induced sampler prototype	2.0
	WDI_agriculture	Quantile-SFA	hqer_induced	True	24	-2862.1669114566434	hqer_induced sampler prototype	1.0
	WDI_agriculture	HQER-SFA	hqer_induced	True	11	-2862.105482614132	hqer_induced sampler prototype	2.0
	WDI_agriculture	Quantile-SFA	hqer_induced	True	13	-2883.693139803509	hqer_induced sampler prototype	2.0
	WDI_agriculture	HQER-SFA	hqer_induced	True	30	-2883.5809157438484	hqer_induced sampler prototype	1.0
	WDI_agriculture	Quantile-SFA	hqer_induced	True	19	-2914.610271971465	hqer_induced sampler prototype	1.0
	WDI_agriculture	HQER-SFA	hqer_induced	True	32	-2914.527392897261	hqer_induced sampler prototype	1.0
	WDI_agriculture	Quantile-SFA	hqer_induced	True	26	-2833.377086455828	hqer_induced sampler prototype	1.0
	WDI_agriculture	HQER-SFA	hqer_induced	True	32	-2833.2557781938267	hqer_induced sampler prototype	3.0
	WDI_agriculture	Quantile-SFA	hqer_induced	True	22	-2889.3932293112894	hqer_induced sampler prototype	5.0
	WDI_agriculture	HQER-SFA	hqer_induced	True	31	-2889.282038264796	hqer_induced sampler prototype	3.0
	WDI_agriculture	Quantile-SFA	hqer_induced	True	31	-2862.4959588425995	hqer_induced sampler prototype	5.0
	WDI_agriculture	HQER-SFA	hqer_induced	True	20	-2862.456016464618	hqer_induced sampler prototype	3.0
	WDI_agriculture	Quantile-SFA	hqer_induced	True	18	-2809.8840101649384	hqer_induced sampler prototype	5.0
	WDI_agriculture	HQER-SFA	hqer_induced	True	25	-2809.7231046421	hqer_induced sampler prototype	2.0
	WDI_agriculture	Quantile-SFA	hqer_induced	True	18	-2778.953399989006	hqer_induced sampler prototype	2.0
	WDI_agriculture	HQER-SFA	hqer_induced	True	19	-2778.9751960868	hqer_induced sampler prototype	2.0
	WDI_agriculture	Quantile-SFA	hqer_induced	True	14	-2867.5851380376407	hqer_induced sampler prototype	2.0
	WDI_agriculture	HQER-SFA	hqer_induced	True	25	-2867.4924613702487	hqer_induced sampler prototype	3.0
	WDI_agriculture	Quantile-SFA	hqer_induced	True	10	-2846.375553343436	hqer_induced sampler prototype	2.0
	WDI_agriculture	HQER-SFA	hqer_induced	True	9	-2846.227609912868	hqer_induced sampler prototype	2.0
	WDI_agriculture	Quantile-SFA	hqer_induced	True	11	-2874.930584170018	hqer_induced sampler prototype	2.0
	WDI_agriculture	HQER-SFA	hqer_induced	True	24	-2874.86542316792	hqer_induced sampler prototype	5.0
	WDI_agriculture	Quantile-SFA	hqer_induced	True	20	-2890.662356046622	hqer_induced sampler prototype	3.0
	WDI_agriculture	HQER-SFA	hqer_induced	True	16	-2890.583461036461	hqer_induced sampler prototype	2.0
	WDI_agriculture	Quantile-SFA	hqer_induced	True	30	-2850.616633257673	hqer_induced sampler prototype	1.0
	WDI_agriculture	HQER-SFA	hqer_induced	True	19	-2850.552374067362	hqer_induced sampler prototype	3.0
	WDI_agriculture	Quantile-SFA	hqer_induced	True	25	-2910.388207227291	hqer_induced sampler prototype	2.0
	WDI_agriculture	HQER-SFA	hqer_induced	True	22	-2910.337556208276	hqer_induced sampler prototype	5.0
	WDI_agriculture	Quantile-SFA	hqer_induced	True	21	-2951.2932844314064	hqer_induced sampler prototype	3.0
	WDI_agriculture	HQER-SFA	hqer_induced	True	23	-2951.2328441181844	hqer_induced sampler prototype	3.0
	WDI_agriculture	Quantile-SFA	hqer_induced	True	8	-2836.429249721046	hqer_induced sampler prototype	2.0
	WDI_agriculture	HQER-SFA	hqer_induced	True	9	-2836.3690985144367	hqer_induced sampler prototype	2.0
	WDI_agriculture	Quantile-SFA	hqer_induced	True	22	-2878.998871457908	hqer_induced sampler prototype	3.0
	WDI_agriculture	HQER-SFA	hqer_induced	True	18	-2879.080287534109	hqer_induced sampler prototype	5.0

experiment	dataset	model	scenario	converged	n__iter	loglik	warning__message	best__start__id
	WDI_agriculture	Quantile-SFA	hqer_induced	True	20	-2853.574657262704	hqer_induced sampler prototype	1.0
	WDI_agriculture	HQER-SFA	hqer_induced	True	8	-2853.4866602966204	hqer_induced sampler prototype	2.0
	WDI_agriculture	Quantile-SFA	hqer_induced	True	23	-2906.3457835031504	hqer_induced sampler prototype	3.0
	WDI_agriculture	HQER-SFA	hqer_induced	True	17	-2906.360192653086	hqer_induced sampler prototype	2.0
	WDI_agriculture	Quantile-SFA	hqer_induced	True	15	-2818.7428907003728	hqer_induced sampler prototype	2.0
	WDI_agriculture	HQER-SFA	hqer_induced	True	21	-2818.6968211876906	hqer_induced sampler prototype	3.0
	WDI_agriculture	Quantile-SFA	hqer_induced	True	19	-2813.7732512172097	hqer_induced sampler prototype	3.0
	WDI_agriculture	HQER-SFA	hqer_induced	True	10	-2813.685812545824	hqer_induced sampler prototype	2.0
	WDI_agriculture	Quantile-SFA	hqer_induced	True	16	-2861.1284490306184	hqer_induced sampler prototype	3.0
	WDI_agriculture	HQER-SFA	hqer_induced	True	29	-2860.9877117640217	hqer_induced sampler prototype	5.0
	WDI_agriculture	Quantile-SFA	hqer_induced	True	8	-2885.680876169163	hqer_induced sampler prototype	2.0
	WDI_agriculture	HQER-SFA	hqer_induced	True	11	-2885.5965834732287	hqer_induced sampler prototype	2.0
	WDI_agriculture	Quantile-SFA	hqer_induced	True	29	-2864.614243745834	hqer_induced sampler prototype	3.0
	WDI_agriculture	HQER-SFA	hqer_induced	True	21	-2864.465738594641	hqer_induced sampler prototype	1.0
	WDI_agriculture	Quantile-SFA	hqer_induced	True	22	-2893.1847002638297	hqer_induced sampler prototype	1.0
	WDI_agriculture	HQER-SFA	hqer_induced	True	25	-2893.142070818403	hqer_induced sampler prototype	5.0
	WDI_agriculture	Quantile-SFA	hqer_induced	True	16	-2840.718964388655	hqer_induced sampler prototype	5.0
	WDI_agriculture	HQER-SFA	hqer_induced	True	30	-2840.661383853087	hqer_induced sampler prototype	3.0
	WDI_agriculture	Quantile-SFA	hqer_induced	True	22	-2870.625896323848	hqer_induced sampler prototype	1.0
	WDI_agriculture	HQER-SFA	hqer_induced	True	23	-2870.581901205202	hqer_induced sampler prototype	3.0
	WDI_agriculture	Quantile-SFA	hqer_induced	True	10	-2867.124717108317	hqer_induced sampler prototype	2.0
	WDI_agriculture	HQER-SFA	hqer_induced	True	19	-2867.0943338644565	hqer_induced sampler prototype	5.0
	WDI_agriculture	Quantile-SFA	hqer_induced	True	33	-2912.190771517755	hqer_induced sampler prototype	1.0
	WDI_agriculture	HQER-SFA	hqer_induced	True	26	-2912.0917909095806	hqer_induced sampler prototype	1.0
	WDI_agriculture	Quantile-SFA	hqer_induced	True	29	-2833.09845837007	hqer_induced sampler prototype	1.0
	WDI_agriculture	HQER-SFA	hqer_induced	True	21	-2833.011133506886	hqer_induced sampler prototype	1.0
	WDI_agriculture	Quantile-SFA	hqer_induced	True	27	-2888.1240556896028	hqer_induced sampler prototype	1.0
	WDI_agriculture	HQER-SFA	hqer_induced	True	23	-2887.9606243915146	hqer_induced sampler prototype	1.0
	WDI_agriculture	Quantile-SFA	hqer_induced	True	26	-2875.267614104697	hqer_induced sampler prototype	3.0
	WDI_agriculture	HQER-SFA	hqer_induced	True	22	-2875.2203004606386	hqer_induced sampler prototype	3.0
	WDI_agriculture	Quantile-SFA	hqer_induced	True	9	-2883.613725276716	hqer_induced sampler prototype	2.0
	WDI_agriculture	HQER-SFA	hqer_induced	True	9	-2883.5412297573976	hqer_induced sampler prototype	2.0
	WDI_agriculture	Quantile-SFA	hqer_induced	True	24	-2897.1062375915426	hqer_induced sampler prototype	3.0
	WDI_agriculture	HQER-SFA	hqer_induced	True	25	-2897.042791987489	hqer_induced sampler prototype	3.0
	WDI_agriculture	Quantile-SFA	hqer_induced	True	18	-2861.147560374494	hqer_induced sampler prototype	2.0
	WDI_agriculture	HQER-SFA	hqer_induced	True	21	-2861.0652598991715	hqer_induced sampler prototype	3.0
	WDI_agriculture	Quantile-SFA	hqer_induced	True	16	-2872.713030749952	hqer_induced sampler prototype	2.0
	WDI_agriculture	HQER-SFA	hqer_induced	True	16	-2872.8466230775166	hqer_induced sampler prototype	5.0
	WDI_agriculture	Quantile-SFA	hqer_induced	True	22	-2859.4509852290503	hqer_induced sampler prototype	5.0
	WDI_agriculture	HQER-SFA	hqer_induced	True	32	-2859.3832893332656	hqer_induced sampler prototype	1.0
	WDI_agriculture	Quantile-SFA	hqer_induced	True	19	-2864.4441008011536	hqer_induced sampler prototype	3.0
	WDI_agriculture	HQER-SFA	hqer_induced	True	24	-2864.1982486841944	hqer_induced sampler prototype	3.0
	WDI_agriculture	Quantile-SFA	hqer_induced	True	24	-2868.6267646599817	hqer_induced sampler prototype	1.0
	WDI_agriculture	HQER-SFA	hqer_induced	True	28	-2868.529984928008	hqer_induced sampler prototype	1.0
	WDI_agriculture	Quantile-SFA	hqer_induced	True	8	-2902.1570990692267	hqer_induced sampler prototype	2.0
	WDI_agriculture	HQER-SFA	hqer_induced	True	21	-2902.06710557557	hqer_induced sampler prototype	1.0
	WDI_agriculture	Quantile-SFA	hqer_induced	True	26	-2895.9114360807107	hqer_induced sampler prototype	5.0
	WDI_agriculture	HQER-SFA	hqer_induced	True	19	-2895.850372152925	hqer_induced sampler prototype	2.0
	WDI_agriculture	Quantile-SFA	hqer_induced	True	24	-2889.6649582327773	hqer_induced sampler prototype	1.0
	WDI_agriculture	HQER-SFA	hqer_induced	True	27	-2889.6243551059747	hqer_induced sampler prototype	2.0
	WDI_agriculture	Quantile-SFA	hqer_induced	True	23	-2875.2054496529963	hqer_induced sampler prototype	3.0
	WDI_agriculture	HQER-SFA	hqer_induced	True	19	-2875.359919273066	hqer_induced sampler prototype	3.0
	WDI_agriculture	Quantile-SFA	hqer_induced	True	21	-2887.1274421106045	hqer_induced sampler prototype	3.0
	WDI_agriculture	HQER-SFA	hqer_induced	True	21	-2887.0650547230425	hqer_induced sampler prototype	1.0
	WDI_agriculture	Quantile-SFA	hqer_induced	True	21	-2811.9593533798416	hqer_induced sampler prototype	3.0
	WDI_agriculture	HQER-SFA	hqer_induced	True	26	-2811.891020295874	hqer_induced sampler prototype	3.0
	WDI_agriculture	Quantile-SFA	hqer_induced	True	25	-2781.519579124434	hqer_induced sampler prototype	1.0
	WDI_agriculture	HQER-SFA	hqer_induced	True	31	-2781.66500426368	hqer_induced sampler prototype	1.0
	WDI_agriculture	Quantile-SFA	hqer_induced	True	12	-2861.03100719914	hqer_induced sampler prototype	5.0
	WDI_agriculture	HQER-SFA	hqer_induced	True	28	-2860.898900034048	hqer_induced sampler prototype	1.0
	WDI_agriculture	Quantile-SFA	hqer_induced	True	22	-2825.548558878677	hqer_induced sampler prototype	5.0
	WDI_agriculture	HQER-SFA	hqer_induced	True	24	-2825.4834443129907	hqer_induced sampler prototype	3.0
	WDI_agriculture	Quantile-SFA	hqer_induced	True	14	-2841.874031076168	hqer_induced sampler prototype	2.0
	WDI_agriculture	HQER-SFA	hqer_induced	True	15	-2841.7994204921024	hqer_induced sampler prototype	2.0
	WDI_agriculture	Quantile-SFA	hqer_induced	True	18	-2836.3885035243147	hqer_induced sampler prototype	5.0
	WDI_agriculture	HQER-SFA	hqer_induced	True	16	-2836.313920462708	hqer_induced sampler prototype	5.0
	WDI_agriculture	Quantile-SFA	hqer_induced	True	24	-2789.5195830104244	hqer_induced sampler prototype	3.0
	WDI_agriculture	HQER-SFA	hqer_induced	True	31	-2789.407692121888	hqer_induced sampler prototype	3.0
	WDI_agriculture	Quantile-SFA	hqer_induced	True	21	-2845.182705011165	hqer_induced sampler prototype	3.0
	WDI_agriculture	HQER-SFA	hqer_induced	True	22	-2845.16507055859	hqer_induced sampler prototype	1.0
	WDI_agriculture	Quantile-SFA	hqer_induced	True	29	-2883.0647949421987	hqer_induced sampler prototype	3.0
	WDI_agriculture	HQER-SFA	hqer_induced	True	22	-2882.979781523383	hqer_induced sampler prototype	2.0
	WDI_agriculture	Quantile-SFA	hqer_induced	True	28	-2849.143877790336	hqer_induced sampler prototype	3.0
	WDI_agriculture	HQER-SFA	hqer_induced	True	22	-2849.043061958536	hqer_induced sampler prototype	3.0
	WDI_agriculture	Quantile-SFA	hqer_induced	True	24	-2862.0751716972763	hqer_induced sampler prototype	1.0
	WDI_agriculture	HQER-SFA	hqer_induced	True	27	-2861.9671880110086	hqer_induced sampler prototype	5.0
	WDI_agriculture	Quantile-SFA	hqer_induced	True	29	-2897.197455679036	hqer_induced sampler prototype	5.0
	WDI_agriculture	HQER-SFA	hqer_induced	True	27	-2896.647022031194	hqer_induced sampler prototype	3.0
	WDI_agriculture	Quantile-SFA	hqer_induced	True	22	-2883.9374853467843	hqer_induced sampler prototype	1.0
	WDI_agriculture	HQER-SFA	hqer_induced	True	24	-2883.872659552254	hqer_induced sampler prototype	3.0
	WDI_agriculture	Quantile-SFA	hqer_induced	True	24	-2863.1550407781547	hqer_induced sampler prototype	3.0
	WDI_agriculture	HQER-SFA	hqer_induced	True	21	-2863.1656213686783	hqer_induced sampler prototype	5.0
	WDI_agriculture	Quantile-SFA	hqer_induced	True	24	-2920.446959938974	hqer_induced sampler prototype	3.0
	WDI_agriculture	HQER-SFA	hqer_induced	True	12	-2920.344354031244	hqer_induced sampler prototype	2.0
	WDI_agriculture	Quantile-SFA	hqer_induced	True	22	-2919.537533118775	hqer_induced sampler prototype	3.0
	WDI_agriculture	HQER-SFA	hqer_induced	True	24	-2919.4691637206583	hqer_induced sampler prototype	3.0
	WDI_agriculture	Quantile-SFA	hqer_induced	True	23	-2903.3199426437995	hqer_induced sampler prototype	3.0
	WDI_agriculture	HQER-SFA	hqer_induced	True	23	-2903.531120404628	hqer_induced sampler prototype	5.0
	WDI_agriculture	Quantile-SFA	hqer_induced	True	24	-2873.3065729778605	hqer_induced sampler prototype	5.0
	WDI_agriculture	HQER-SFA	hqer_induced	True	28	-2873.2451228358764	hqer_induced sampler prototype	3.0
	WDI_agriculture	Quantile-SFA	hqer_induced	True	24	-2900.2289602722403	hqer_induced sampler prototype	5.0
	WDI_agriculture	HQER-SFA	hqer_induced	True	17	-2900.09932136648	hqer_induced sampler prototype	1.0
	WDI_agriculture	Quantile-SFA	hqer_induced	True	21	-2889.644959826808	hqer_induced sampler prototype	3.0
	WDI_agriculture	HQER-SFA	hqer_induced	True	31	-2889.5449094398155	hqer_induced sampler prototype	1.0
	WDI_agriculture	Quantile-SFA	hqer_induced	True	32	-2875.35828365418	hqer_induced sampler prototype	3.0
	WDI_agriculture	HQER-SFA	hqer_induced	True	35	-2875.2943089834093	hqer_induced sampler prototype	1.0
	WDI_agriculture	Quantile-SFA	hqer_induced	True	21	-2861.76834915215	hqer_induced sampler prototype	3.0
	WDI_agriculture	HQER-SFA	hqer_induced	True	21	-2861.6769448768937	hqer_induced sampler prototype	5.0
	WDI_agriculture	Quantile-SFA	hqer_induced	True	18	-2925.3509020696915	hqer_induced sampler prototype	2.0
	WDI_agriculture	HQER-SFA	hqer_induced	True	25	-2925.2530903103952	hqer_induced sampler prototype	3.0
	WDI_agriculture	Quantile-SFA	hqer_induced	True	17	-2924.114348417099	hqer_induced sampler prototype	2.0
	WDI_agriculture	HQER-SFA	hqer_induced	True	15	-2924.0125288987333	hqer_induced sampler prototype	2.0
	WDI_agriculture	Quantile-SFA	hqer_induced	True	24	-2904.9665430407754	hqer_induced sampler prototype	1.0

experiment	dataset	model	scenario	converged	n__iter	loglik	warning__message	best__start__id
	WDI_agriculture	HQER-SFA	hqer__induced	True	24	-2904.8934300766496	hqer__induced sampler prototype	3.0
	WDI_agriculture	Quantile-SFA	hqer__induced	True	19	-2802.9051897100667	hqer__induced sampler prototype	3.0
	WDI_agriculture	HQER-SFA	hqer__induced	True	30	-2802.825488057615	hqer__induced sampler prototype	5.0
	WDI_agriculture	Quantile-SFA	hqer__induced	True	26	-2824.93542329262	hqer__induced sampler prototype	1.0
	WDI_agriculture	HQER-SFA	hqer__induced	True	21	-2824.83684237656	hqer__induced sampler prototype	1.0
	WDI_agriculture	Quantile-SFA	hqer__induced	True	22	-2899.056722130377	hqer__induced sampler prototype	1.0
	WDI_agriculture	HQER-SFA	hqer__induced	True	22	-2898.9938518089066	hqer__induced sampler prototype	3.0
	WDI_agriculture	Quantile-SFA	hqer__induced	True	13	-2860.304387652787	hqer__induced sampler prototype	2.0
	WDI_agriculture	HQER-SFA	hqer__induced	True	27	-2860.216691772761	hqer__induced sampler prototype	3.0
	WDI_agriculture	Quantile-SFA	hqer__induced	True	26	-2843.223287216493	hqer__induced sampler prototype	3.0
	WDI_agriculture	HQER-SFA	hqer__induced	True	22	-2843.14470650056	hqer__induced sampler prototype	5.0
	WDI_agriculture	Quantile-SFA	hqer__induced	True	23	-2789.4499543185057	hqer__induced sampler prototype	3.0
	WDI_agriculture	HQER-SFA	hqer__induced	True	27	-2789.348231104596	hqer__induced sampler prototype	3.0
	WDI_agriculture	Quantile-SFA	hqer__induced	True	22	-2855.419295490865	hqer__induced sampler prototype	1.0
	WDI_agriculture	HQER-SFA	hqer__induced	True	10	-2855.5625133018684	hqer__induced sampler prototype	2.0
	WDI_agriculture	Quantile-SFA	hqer__induced	True	21	-2903.639200193328	hqer__induced sampler prototype	3.0
	WDI_agriculture	HQER-SFA	hqer__induced	True	23	-2903.553612700353	hqer__induced sampler prototype	2.0
	WDI_agriculture	Quantile-SFA	hqer__induced	True	11	-2839.849391631448	hqer__induced sampler prototype	2.0
	WDI_agriculture	HQER-SFA	hqer__induced	True	11	-2839.7683136981364	hqer__induced sampler prototype	2.0
	WDI_agriculture	Quantile-SFA	hqer__induced	True	18	-2821.468677236934	hqer__induced sampler prototype	2.0
	WDI_agriculture	HQER-SFA	hqer__induced	True	27	-2821.4283888083783	hqer__induced sampler prototype	1.0
	WDI_agriculture	Quantile-SFA	hqer__induced	True	27	-2843.033862735896	hqer__induced sampler prototype	1.0
	WDI_agriculture	HQER-SFA	hqer__induced	True	15	-2842.9965155596187	hqer__induced sampler prototype	2.0
	WDI_agriculture	Quantile-SFA	hqer__induced	True	28	-2823.9085414215506	hqer__induced sampler prototype	1.0
	WDI_agriculture	HQER-SFA	hqer__induced	True	12	-2823.883770588021	hqer__induced sampler prototype	2.0
	WDI_agriculture	Quantile-SFA	hqer__induced	True	29	-2788.4879015332403	hqer__induced sampler prototype	3.0
	WDI_agriculture	HQER-SFA	hqer__induced	True	22	-2788.4231329619806	hqer__induced sampler prototype	5.0
	WDI_agriculture	Quantile-SFA	hqer__induced	True	23	-2794.0604217599875	hqer__induced sampler prototype	1.0
	WDI_agriculture	HQER-SFA	hqer__induced	True	17	-2793.9570723268475	hqer__induced sampler prototype	3.0
	WDI_agriculture	Quantile-SFA	hqer__induced	True	31	-2877.469586043395	hqer__induced sampler prototype	1.0
	WDI_agriculture	HQER-SFA	hqer__induced	True	20	-2877.4562772338977	hqer__induced sampler prototype	1.0
	WDI_agriculture	Quantile-SFA	hqer__induced	True	24	-2878.593892908997	hqer__induced sampler prototype	1.0
	WDI_agriculture	HQER-SFA	hqer__induced	True	22	-2878.465010992152	hqer__induced sampler prototype	1.0
	WDI_agriculture	Quantile-SFA	hqer__induced	True	21	-2853.478649996538	hqer__induced sampler prototype	1.0
	WDI_agriculture	HQER-SFA	hqer__induced	True	13	-2853.37495285577	hqer__induced sampler prototype	5.0
	WDI_agriculture	Quantile-SFA	hqer__induced	True	26	-2864.14657907964	hqer__induced sampler prototype	1.0
	WDI_agriculture	HQER-SFA	hqer__induced	True	25	-2863.946710714692	hqer__induced sampler prototype	5.0
	WDI_agriculture	Quantile-SFA	hqer__induced	True	30	-2839.5217731762013	hqer__induced sampler prototype	1.0
	WDI_agriculture	HQER-SFA	hqer__induced	True	35	-2839.413361108771	hqer__induced sampler prototype	1.0
	WDI_agriculture	Quantile-SFA	hqer__induced	True	13	-2836.580393013652	hqer__induced sampler prototype	2.0
	WDI_agriculture	HQER-SFA	hqer__induced	True	26	-2836.5006431169013	hqer__induced sampler prototype	5.0
	WDI_agriculture	Quantile-SFA	hqer__induced	True	29	-2841.77498020977	hqer__induced sampler prototype	3.0
	WDI_agriculture	HQER-SFA	hqer__induced	True	16	-2841.684555105209	hqer__induced sampler prototype	2.0
	WDI_agriculture	Quantile-SFA	hqer__induced	True	19	-2908.481571174923	hqer__induced sampler prototype	2.0
	WDI_agriculture	HQER-SFA	hqer__induced	True	22	-2908.337998143912	hqer__induced sampler prototype	3.0
	WDI_agriculture	Quantile-SFA	hqer__induced	True	21	-2826.190181029121	hqer__induced sampler prototype	3.0
	WDI_agriculture	HQER-SFA	hqer__induced	True	25	-2826.133335790973	hqer__induced sampler prototype	2.0
	WDI_agriculture	Quantile-SFA	hqer__induced	True	26	-2888.6430158653648	hqer__induced sampler prototype	1.0
	WDI_agriculture	HQER-SFA	hqer__induced	True	22	-2888.58555599582	hqer__induced sampler prototype	1.0
	WDI_agriculture	Quantile-SFA	hqer__induced	True	21	-2871.707410609902	hqer__induced sampler prototype	1.0
	WDI_agriculture	HQER-SFA	hqer__induced	True	27	-2871.616722751653	hqer__induced sampler prototype	1.0
	WDI_agriculture	Quantile-SFA	hqer__induced	True	23	-2842.901586652096	hqer__induced sampler prototype	5.0
	WDI_agriculture	HQER-SFA	hqer__induced	True	23	-2842.7649094219896	hqer__induced sampler prototype	5.0
	WDI_agriculture	Quantile-SFA	hqer__induced	True	25	-2922.740879709306	hqer__induced sampler prototype	1.0
	WDI_agriculture	HQER-SFA	hqer__induced	True	30	-2922.655335425243	hqer__induced sampler prototype	1.0
	WDI_agriculture	Quantile-SFA	hqer__induced	True	26	-2834.64503391719	hqer__induced sampler prototype	3.0
	WDI_agriculture	HQER-SFA	hqer__induced	True	30	-2834.501451442046	hqer__induced sampler prototype	5.0
	WDI_agriculture	Quantile-SFA	hqer__induced	True	28	-2891.8941416713187	hqer__induced sampler prototype	3.0
	WDI_agriculture	HQER-SFA	hqer__induced	True	22	-2891.8758437755178	hqer__induced sampler prototype	3.0
	WDI_agriculture	Quantile-SFA	hqer__induced	True	31	-2847.96205224575	hqer__induced sampler prototype	3.0
	WDI_agriculture	HQER-SFA	hqer__induced	True	26	-2847.907548031856	hqer__induced sampler prototype	3.0
	WDI_agriculture	Quantile-SFA	hqer__induced	True	27	-2825.280227131295	hqer__induced sampler prototype	3.0
	WDI_agriculture	HQER-SFA	hqer__induced	True	29	-2825.205435985539	hqer__induced sampler prototype	3.0
	WDI_agriculture	Quantile-SFA	hqer__induced	True	20	-2832.52526643146	hqer__induced sampler prototype	1.0
	WDI_agriculture	HQER-SFA	hqer__induced	True	24	-2832.5138559912	hqer__induced sampler prototype	3.0
	WDI_agriculture	Quantile-SFA	hqer__induced	True	26	-2864.700769763021	hqer__induced sampler prototype	1.0
	WDI_agriculture	HQER-SFA	hqer__induced	True	28	-2864.5770886074392	hqer__induced sampler prototype	3.0
	WDI_agriculture	Quantile-SFA	hqer__induced	True	24	-2815.3290691411503	hqer__induced sampler prototype	3.0
	WDI_agriculture	HQER-SFA	hqer__induced	True	20	-2815.078105191272	hqer__induced sampler prototype	2.0
	WDI_agriculture	Quantile-SFA	hqer__induced	True	30	-2813.347332797448	hqer__induced sampler prototype	1.0
	WDI_agriculture	HQER-SFA	hqer__induced	True	35	-2813.3021226866763	hqer__induced sampler prototype	1.0
	WDI_agriculture	Quantile-SFA	hqer__induced	True	32	-2869.5090013964264	hqer__induced sampler prototype	3.0
	WDI_agriculture	HQER-SFA	hqer__induced	True	19	-2869.55304652342	hqer__induced sampler prototype	5.0
	WDI_agriculture	Quantile-SFA	hqer__induced	True	20	-2807.965755434266	hqer__induced sampler prototype	2.0
	WDI_agriculture	HQER-SFA	hqer__induced	True	15	-2807.8933009879383	hqer__induced sampler prototype	2.0
	WDI_agriculture	Quantile-SFA	hqer__induced	True	26	-2933.008986515704	hqer__induced sampler prototype	3.0
	WDI_agriculture	HQER-SFA	hqer__induced	True	27	-2932.969962149596	hqer__induced sampler prototype	3.0
	WDI_agriculture	Quantile-SFA	hqer__induced	True	17	-2856.9968017797214	hqer__induced sampler prototype	2.0
	WDI_agriculture	HQER-SFA	hqer__induced	True	22	-2856.7384175941866	hqer__induced sampler prototype	3.0
	WDI_agriculture	Quantile-SFA	hqer__induced	True	38	-2858.133345793219	hqer__induced sampler prototype	5.0
	WDI_agriculture	HQER-SFA	hqer__induced	True	24	-2858.060273326928	hqer__induced sampler prototype	3.0
	WDI_agriculture	Quantile-SFA	hqer__induced	True	27	-2834.3437311099183	hqer__induced sampler prototype	3.0
	WDI_agriculture	HQER-SFA	hqer__induced	True	25	-2834.308694991724	hqer__induced sampler prototype	3.0
	WDI_agriculture	Quantile-SFA	hqer__induced	True	21	-2843.0642738761644	hqer__induced sampler prototype	1.0
	WDI_agriculture	HQER-SFA	hqer__induced	True	15	-2843.0479958383075	hqer__induced sampler prototype	2.0
	WDI_agriculture	Quantile-SFA	hqer__induced	True	24	-2890.8268109818137	hqer__induced sampler prototype	3.0
	WDI_agriculture	HQER-SFA	hqer__induced	True	30	-2890.784355766873	hqer__induced sampler prototype	3.0
	WDI_agriculture	Quantile-SFA	hqer__induced	True	31	-2873.791040820216	hqer__induced sampler prototype	3.0
	WDI_agriculture	HQER-SFA	hqer__induced	True	19	-2873.812092932064	hqer__induced sampler prototype	5.0
	WDI_agriculture	Quantile-SFA	hqer__induced	True	19	-2846.1241637486546	hqer__induced sampler prototype	3.0
	WDI_agriculture	HQER-SFA	hqer__induced	True	22	-2846.117516409581	hqer__induced sampler prototype	3.0
	WDI_agriculture	Quantile-SFA	hqer__induced	True	27	-2849.664409408786	hqer__induced sampler prototype	5.0
	WDI_agriculture	HQER-SFA	hqer__induced	True	17	-2849.5745336365185	hqer__induced sampler prototype	2.0
	WDI_agriculture	Quantile-SFA	hqer__induced	True	28	-2866.423612967838	hqer__induced sampler prototype	5.0
	WDI_agriculture	HQER-SFA	hqer__induced	True	16	-2866.36959734323	hqer__induced sampler prototype	2.0
	WDI_agriculture	Quantile-SFA	hqer__induced	True	25	-2867.941669208759	hqer__induced sampler prototype	1.0
	WDI_agriculture	HQER-SFA	hqer__induced	True	26	-2867.914391566519	hqer__induced sampler prototype	1.0
	WDI_agriculture	Quantile-SFA	hqer__induced	True	20	-2840.5805745360585	hqer__induced sampler prototype	3.0
	WDI_agriculture	HQER-SFA	hqer__induced	True	24	-2840.50467576783	hqer__induced sampler prototype	1.0
	WDI_agriculture	Quantile-SFA	hqer__induced	True	28	-2861.1653705358094	hqer__induced sampler prototype	5.0
	WDI_agriculture	HQER-SFA	hqer__induced	True	33	-2861.0344529305967	hqer__induced sampler prototype	3.0
	WDI_agriculture	Quantile-SFA	hqer__induced	True	29	-2810.3525070955216	hqer__induced sampler prototype	3.0
	WDI_agriculture	HQER-SFA	hqer__induced	True	24	-2810.2458166958136	hqer__induced sampler prototype	5.0

experiment	dataset	model	scenario	converged	n_iter	loglik	warning_message	best_start_id
	WDI_agriculture	Quantile-SFA	hqer_induced	True	28	-2884.9523287807897	hqer_induced sampler prototype	1.0
	WDI_agriculture	HQER-SFA	hqer_induced	True	28	-2884.8673783508248	hqer_induced sampler prototype	5.0
	WDI_agriculture	Quantile-SFA	hqer_induced	True	31	-2838.2550831744093	hqer_induced sampler prototype	1.0
	WDI_agriculture	HQER-SFA	hqer_induced	True	10	-2838.204925139671	hqer_induced sampler prototype	2.0
	WDI_agriculture	Quantile-SFA	hqer_induced	True	22	-2916.89993539596	hqer_induced sampler prototype	3.0
	WDI_agriculture	HQER-SFA	hqer_induced	True	26	-2916.808762979465	hqer_induced sampler prototype	1.0
	WDI_agriculture	Quantile-SFA	hqer_induced	True	19	-2874.2713229471624	hqer_induced sampler prototype	5.0
	WDI_agriculture	HQER-SFA	hqer_induced	True	22	-2874.18448373438	hqer_induced sampler prototype	3.0
	WDI_agriculture	Quantile-SFA	hqer_induced	True	15	-2880.974853353974	hqer_induced sampler prototype	5.0
	WDI_agriculture	HQER-SFA	hqer_induced	True	23	-2880.8731871609507	hqer_induced sampler prototype	3.0
	WDI_agriculture	Quantile-SFA	hqer_induced	True	27	-2829.947397596756	hqer_induced sampler prototype	3.0
	WDI_agriculture	HQER-SFA	hqer_induced	True	18	-2829.8070162917124	hqer_induced sampler prototype	3.0
	WDI_agriculture	Quantile-SFA	hqer_induced	True	20	-2887.198312655444	hqer_induced sampler prototype	3.0
	WDI_agriculture	HQER-SFA	hqer_induced	True	16	-2887.200935826731	hqer_induced sampler prototype	3.0
	WDI_agriculture	Quantile-SFA	hqer_induced	True	23	-2810.879928608466	hqer_induced sampler prototype	5.0
	WDI_agriculture	HQER-SFA	hqer_induced	True	19	-2810.821593720998	hqer_induced sampler prototype	5.0
	WDI_agriculture	Quantile-SFA	hqer_induced	True	19	-2835.7544191544107	hqer_induced sampler prototype	3.0
	WDI_agriculture	HQER-SFA	hqer_induced	True	22	-2835.671371734469	hqer_induced sampler prototype	3.0
	WDI_agriculture	Quantile-SFA	hqer_induced	True	16	-2804.1477871879506	hqer_induced sampler prototype	2.0
	WDI_agriculture	HQER-SFA	hqer_induced	True	27	-2803.9170908176334	hqer_induced sampler prototype	5.0
	WDI_agriculture	Quantile-SFA	hqer_induced	True	20	-2853.574410974304	hqer_induced sampler prototype	5.0
	WDI_agriculture	HQER-SFA	hqer_induced	True	15	-2853.5535020894804	hqer_induced sampler prototype	5.0
	WDI_agriculture	Quantile-SFA	hqer_induced	True	26	-2867.134262816965	hqer_induced sampler prototype	3.0
	WDI_agriculture	HQER-SFA	hqer_induced	True	25	-2867.11013566526	hqer_induced sampler prototype	5.0
	WDI_agriculture	Quantile-SFA	hqer_induced	True	17	-2805.1970961183424	hqer_induced sampler prototype	3.0
	WDI_agriculture	HQER-SFA	hqer_induced	True	23	-2804.814270125152	hqer_induced sampler prototype	5.0
	WDI_agriculture	Quantile-SFA	hqer_induced	True	22	-2826.1526859190444	hqer_induced sampler prototype	3.0
	WDI_agriculture	HQER-SFA	hqer_induced	True	13	-2826.093398942787	hqer_induced sampler prototype	5.0
	WDI_agriculture	Quantile-SFA	hqer_induced	True	13	-2862.419183080467	hqer_induced sampler prototype	2.0
	WDI_agriculture	HQER-SFA	hqer_induced	True	19	-2862.309945873228	hqer_induced sampler prototype	3.0
	WDI_agriculture	Quantile-SFA	hqer_induced	True	28	-2854.638284615886	hqer_induced sampler prototype	3.0
	WDI_agriculture	HQER-SFA	hqer_induced	True	39	-2854.5842141634066	hqer_induced sampler prototype	1.0
	WDI_agriculture	Quantile-SFA	hqer_induced	True	21	-2889.067251344486	hqer_induced sampler prototype	3.0
	WDI_agriculture	HQER-SFA	hqer_induced	True	21	-2888.9718528205726	hqer_induced sampler prototype	3.0
	WDI_agriculture	Quantile-SFA	hqer_induced	True	16	-2909.450606011037	hqer_induced sampler prototype	5.0
	WDI_agriculture	HQER-SFA	hqer_induced	True	28	-2909.3735963871613	hqer_induced sampler prototype	1.0
	WDI_agriculture	Quantile-SFA	hqer_induced	True	24	-2856.7676042849507	hqer_induced sampler prototype	1.0
	WDI_agriculture	HQER-SFA	hqer_induced	True	24	-2856.801890018557	hqer_induced sampler prototype	5.0
	WDI_agriculture	Quantile-SFA	hqer_induced	True	27	-2858.9115898294094	hqer_induced sampler prototype	1.0
	WDI_agriculture	HQER-SFA	hqer_induced	True	18	-2858.7904958450818	hqer_induced sampler prototype	2.0
	WDI_agriculture	Quantile-SFA	hqer_induced	True	17	-2873.733088331302	hqer_induced sampler prototype	5.0
	WDI_agriculture	HQER-SFA	hqer_induced	True	22	-2873.638085536193	hqer_induced sampler prototype	5.0
	WDI_agriculture	Quantile-SFA	hqer_induced	True	27	-2916.199619470907	hqer_induced sampler prototype	5.0
	WDI_agriculture	HQER-SFA	hqer_induced	True	22	-2916.141795649578	hqer_induced sampler prototype	5.0
	WDI_agriculture	Quantile-SFA	hqer_induced	True	29	-2816.9799945195623	hqer_induced sampler prototype	3.0
	WDI_agriculture	HQER-SFA	hqer_induced	True	19	-2816.942028659348	hqer_induced sampler prototype	2.0
	WDI_agriculture	Quantile-SFA	hqer_induced	True	20	-2854.579058825296	hqer_induced sampler prototype	1.0
	WDI_agriculture	HQER-SFA	hqer_induced	True	21	-2854.505439438177	hqer_induced sampler prototype	1.0
	WDI_agriculture	Quantile-SFA	hqer_induced	True	16	-2864.73447051916	hqer_induced sampler prototype	2.0
	WDI_agriculture	HQER-SFA	hqer_induced	True	17	-2864.647202564741	hqer_induced sampler prototype	5.0
	WDI_agriculture	Quantile-SFA	hqer_induced	True	19	-2885.6993012923763	hqer_induced sampler prototype	2.0
	WDI_agriculture	HQER-SFA	hqer_induced	True	18	-2885.622269867697	hqer_induced sampler prototype	2.0
	WDI_agriculture	Quantile-SFA	hqer_induced	True	28	-2830.5472050623	hqer_induced sampler prototype	5.0
	WDI_agriculture	HQER-SFA	hqer_induced	True	19	-2830.559692448127	hqer_induced sampler prototype	2.0
	WDI_agriculture	Quantile-SFA	hqer_induced	True	38	-735.2397499507338	hqer_induced sampler prototype	3.0
	WDI_agriculture	HQER-SFA	hqer_induced	True	7	-730.7159221475727	hqer_induced sampler prototype	4.0
	WDI_agriculture	Quantile-SFA	hqer_induced	True	24	-698.4660409136342	hqer_induced sampler prototype	5.0
	WDI_agriculture	HQER-SFA	hqer_induced	True	12	-692.1569205670912	hqer_induced sampler prototype	2.0
	WDI_agriculture	Quantile-SFA	hqer_induced	True	29	-735.6703776746975	hqer_induced sampler prototype	3.0
	WDI_agriculture	HQER-SFA	hqer_induced	True	10	-727.3318312933518	hqer_induced sampler prototype	4.0
	WDI_agriculture	Quantile-SFA	hqer_induced	True	28	-687.6513519138847	hqer_induced sampler prototype	5.0
	WDI_agriculture	HQER-SFA	hqer_induced	True	9	-683.1262479988447	hqer_induced sampler prototype	2.0
	WDI_agriculture	Quantile-SFA	hqer_induced	True	31	-712.764010938565	hqer_induced sampler prototype	1.0
	WDI_agriculture	HQER-SFA	hqer_induced	True	11	-704.7943706430408	hqer_induced sampler prototype	2.0
	WDI_agriculture	Quantile-SFA	hqer_induced	True	37	-716.6450606819833	hqer_induced sampler prototype	1.0
	WDI_agriculture	HQER-SFA	hqer_induced	True	4	-708.1273477382575	hqer_induced sampler prototype	2.0
	WDI_agriculture	Quantile-SFA	hqer_induced	True	23	-717.3674636101537	hqer_induced sampler prototype	5.0
	WDI_agriculture	HQER-SFA	hqer_induced	True	9	-709.8305859150269	hqer_induced sampler prototype	2.0
	WDI_agriculture	Quantile-SFA	hqer_induced	True	16	-743.5369380895089	hqer_induced sampler prototype	5.0
	WDI_agriculture	HQER-SFA	hqer_induced	True	10	-738.6304551143606	hqer_induced sampler prototype	4.0
	WDI_agriculture	Quantile-SFA	hqer_induced	True	29	-738.7562416788965	hqer_induced sampler prototype	3.0
	WDI_agriculture	HQER-SFA	hqer_induced	True	11	-735.5673273301413	hqer_induced sampler prototype	4.0
	WDI_agriculture	Quantile-SFA	hqer_induced	True	18	-718.0694648899789	hqer_induced sampler prototype	5.0
	WDI_agriculture	HQER-SFA	hqer_induced	True	14	-712.5032340538505	hqer_induced sampler prototype	4.0
	WDI_agriculture	Quantile-SFA	hqer_induced	True	33	-693.730072813777	hqer_induced sampler prototype	3.0
	WDI_agriculture	HQER-SFA	hqer_induced	True	15	-685.2741247902625	hqer_induced sampler prototype	4.0
	WDI_agriculture	Quantile-SFA	hqer_induced	True	17	-716.6559236686069	hqer_induced sampler prototype	5.0
	WDI_agriculture	HQER-SFA	hqer_induced	True	12	-708.6444211398276	hqer_induced sampler prototype	4.0
	WDI_agriculture	Quantile-SFA	hqer_induced	True	27	-727.0793568673456	hqer_induced sampler prototype	3.0
	WDI_agriculture	HQER-SFA	hqer_induced	True	8	-718.5700299361239	hqer_induced sampler prototype	2.0
	WDI_agriculture	Quantile-SFA	hqer_induced	True	31	-731.2411095998018	hqer_induced sampler prototype	3.0
	WDI_agriculture	HQER-SFA	hqer_induced	True	7	-725.3337542476877	hqer_induced sampler prototype	2.0
	WDI_agriculture	Quantile-SFA	hqer_induced	True	29	-735.1025711077453	hqer_induced sampler prototype	5.0
	WDI_agriculture	HQER-SFA	hqer_induced	True	10	-730.2829383899873	hqer_induced sampler prototype	2.0
	WDI_agriculture	Quantile-SFA	hqer_induced	True	21	-730.6072979859271	hqer_induced sampler prototype	3.0
	WDI_agriculture	HQER-SFA	hqer_induced	True	10	-722.276685715543	hqer_induced sampler prototype	4.0
	WDI_agriculture	Quantile-SFA	hqer_induced	True	21	-701.6751329953298	hqer_induced sampler prototype	3.0
	WDI_agriculture	HQER-SFA	hqer_induced	True	11	-696.1549317828425	hqer_induced sampler prototype	4.0
	WDI_agriculture	Quantile-SFA	hqer_induced	True	30	-697.7347655923909	hqer_induced sampler prototype	5.0
	WDI_agriculture	HQER-SFA	hqer_induced	True	9	-693.5095350477502	hqer_induced sampler prototype	4.0
	WDI_agriculture	Quantile-SFA	hqer_induced	True	25	-719.9313913354101	hqer_induced sampler prototype	1.0
	WDI_agriculture	HQER-SFA	hqer_induced	True	28	-712.7597786784413	hqer_induced sampler prototype	4.0
	WDI_agriculture	Quantile-SFA	hqer_induced	True	37	-709.8993734962794	hqer_induced sampler prototype	3.0
	WDI_agriculture	HQER-SFA	hqer_induced	True	12	-704.2973166247164	hqer_induced sampler prototype	2.0
	WDI_agriculture	Quantile-SFA	hqer_induced	True	28	-716.9037259041551	hqer_induced sampler prototype	1.0
	WDI_agriculture	HQER-SFA	hqer_induced	True	17	-712.0304406211831	hqer_induced sampler prototype	4.0
	WDI_agriculture	Quantile-SFA	hqer_induced	True	24	-727.6729431555415	hqer_induced sampler prototype	5.0
	WDI_agriculture	HQER-SFA	hqer_induced	True	10	-720.6346057253269	hqer_induced sampler prototype	4.0
	WDI_agriculture	Quantile-SFA	hqer_induced	True	35	-722.6672856120997	hqer_induced sampler prototype	3.0
	WDI_agriculture	HQER-SFA	hqer_induced	True	6	-714.4866516254356	hqer_induced sampler prototype	4.0
	WDI_agriculture	Quantile-SFA	hqer_induced	True	27	-701.7558125729727	hqer_induced sampler prototype	1.0
	WDI_agriculture	HQER-SFA	hqer_induced	True	16	-690.2719481242982	hqer_induced sampler prototype	2.0
	WDI_agriculture	Quantile-SFA	hqer_induced	True	18	-734.9023230073144	hqer_induced sampler prototype	5.0

experiment	dataset	model	scenario	converged	n__iter	loglik	warning__message	best__start__id
	WDI__agriculture	HQER-SFA	hqer__induced	True	8	-727.354117776981	hqer__induced sampler prototype	2.0
	WDI__agriculture	Quantile-SFA	hqer__induced	True	40	-730.8374617688197	hqer__induced sampler prototype	5.0
	WDI__agriculture	HQER-SFA	hqer__induced	True	8	-728.830680690328	hqer__induced sampler prototype	2.0
	WDI__agriculture	Quantile-SFA	hqer__induced	True	39	-706.4009163414752	hqer__induced sampler prototype	3.0
	WDI__agriculture	HQER-SFA	hqer__induced	True	7	-704.5674059769481	hqer__induced sampler prototype	2.0
	WDI__agriculture	Quantile-SFA	hqer__induced	True	31	-731.3296666280196	hqer__induced sampler prototype	3.0
	WDI__agriculture	HQER-SFA	hqer__induced	True	10	-723.9263920825747	hqer__induced sampler prototype	4.0
	WDI__agriculture	Quantile-SFA	hqer__induced	True	25	-682.4747945519721	hqer__induced sampler prototype	3.0
	WDI__agriculture	HQER-SFA	hqer__induced	True	14	-677.0322358527196	hqer__induced sampler prototype	4.0
	WDI__agriculture	Quantile-SFA	hqer__induced	True	19	-743.297751003048	hqer__induced sampler prototype	5.0
	WDI__agriculture	HQER-SFA	hqer__induced	True	14	-741.7805453135098	hqer__induced sampler prototype	2.0
	WDI__agriculture	Quantile-SFA	hqer__induced	True	38	-696.9929594165478	hqer__induced sampler prototype	5.0
	WDI__agriculture	HQER-SFA	hqer__induced	True	7	-693.8813821315425	hqer__induced sampler prototype	2.0
	WDI__agriculture	Quantile-SFA	hqer__induced	True	29	-744.6441943105158	hqer__induced sampler prototype	5.0
	WDI__agriculture	HQER-SFA	hqer__induced	True	9	-738.8654327149377	hqer__induced sampler prototype	2.0
	WDI__agriculture	Quantile-SFA	hqer__induced	True	24	-704.8174525767536	hqer__induced sampler prototype	3.0
	WDI__agriculture	HQER-SFA	hqer__induced	True	11	-700.767778793019	hqer__induced sampler prototype	2.0
	WDI__agriculture	Quantile-SFA	hqer__induced	True	20	-754.2805666950562	hqer__induced sampler prototype	5.0
	WDI__agriculture	HQER-SFA	hqer__induced	True	11	-747.0657956956886	hqer__induced sampler prototype	2.0
	WDI__agriculture	Quantile-SFA	hqer__induced	True	25	-710.6035862379742	hqer__induced sampler prototype	5.0
	WDI__agriculture	HQER-SFA	hqer__induced	True	20	-702.3484748509229	hqer__induced sampler prototype	2.0
	WDI__agriculture	Quantile-SFA	hqer__induced	True	29	-742.2769127822469	hqer__induced sampler prototype	5.0
	WDI__agriculture	HQER-SFA	hqer__induced	True	9	-732.3764806377607	hqer__induced sampler prototype	2.0
	WDI__agriculture	Quantile-SFA	hqer__induced	True	25	-715.7406802899942	hqer__induced sampler prototype	5.0
	WDI__agriculture	HQER-SFA	hqer__induced	True	11	-711.2653829224837	hqer__induced sampler prototype	2.0
	WDI__agriculture	Quantile-SFA	hqer__induced	True	24	-728.8514002566251	hqer__induced sampler prototype	1.0
	WDI__agriculture	HQER-SFA	hqer__induced	True	7	-720.1241576876298	hqer__induced sampler prototype	2.0
	WDI__agriculture	Quantile-SFA	hqer__induced	True	51	-717.3246368694765	hqer__induced sampler prototype	3.0
	WDI__agriculture	HQER-SFA	hqer__induced	True	14	-710.5619095406529	hqer__induced sampler prototype	2.0
	WDI__agriculture	Quantile-SFA	hqer__induced	True	29	-691.3739164685039	hqer__induced sampler prototype	3.0
	WDI__agriculture	HQER-SFA	hqer__induced	True	9	-687.4126727004443	hqer__induced sampler prototype	2.0
	WDI__agriculture	Quantile-SFA	hqer__induced	True	27	-692.6748623616245	hqer__induced sampler prototype	5.0
	WDI__agriculture	HQER-SFA	hqer__induced	True	18	-683.6756390929237	hqer__induced sampler prototype	4.0
	WDI__agriculture	Quantile-SFA	hqer__induced	True	25	-739.3370730366186	hqer__induced sampler prototype	1.0
	WDI__agriculture	HQER-SFA	hqer__induced	True	11	-732.9881885209932	hqer__induced sampler prototype	2.0
	WDI__agriculture	Quantile-SFA	hqer__induced	True	21	-725.2590272866544	hqer__induced sampler prototype	5.0
	WDI__agriculture	HQER-SFA	hqer__induced	True	16	-718.3940217646534	hqer__induced sampler prototype	4.0
	WDI__agriculture	Quantile-SFA	hqer__induced	True	44	-716.4637426491486	hqer__induced sampler prototype	1.0
	WDI__agriculture	HQER-SFA	hqer__induced	True	14	-713.4162123874019	hqer__induced sampler prototype	2.0
	WDI__agriculture	Quantile-SFA	hqer__induced	True	20	-719.993901639134	hqer__induced sampler prototype	1.0
	WDI__agriculture	HQER-SFA	hqer__induced	True	15	-712.9883431446715	hqer__induced sampler prototype	2.0
	WDI__agriculture	Quantile-SFA	hqer__induced	True	28	-717.1575446652516	hqer__induced sampler prototype	1.0
	WDI__agriculture	HQER-SFA	hqer__induced	True	14	-711.635244843784	hqer__induced sampler prototype	2.0
	WDI__agriculture	Quantile-SFA	hqer__induced	True	32	-721.7566032175596	hqer__induced sampler prototype	3.0
	WDI__agriculture	HQER-SFA	hqer__induced	True	8	-716.3254425731232	hqer__induced sampler prototype	4.0
	WDI__agriculture	Quantile-SFA	hqer__induced	True	31	-740.2980592164178	hqer__induced sampler prototype	3.0
	WDI__agriculture	HQER-SFA	hqer__induced	True	10	-737.9600940069636	hqer__induced sampler prototype	4.0
	WDI__agriculture	Quantile-SFA	hqer__induced	True	28	-733.6958320489391	hqer__induced sampler prototype	1.0
	WDI__agriculture	HQER-SFA	hqer__induced	True	7	-728.3704707190982	hqer__induced sampler prototype	2.0
	WDI__agriculture	Quantile-SFA	hqer__induced	True	28	-707.3145157571155	hqer__induced sampler prototype	5.0
	WDI__agriculture	HQER-SFA	hqer__induced	True	11	-700.5703228125506	hqer__induced sampler prototype	4.0
	WDI__agriculture	Quantile-SFA	hqer__induced	True	20	-744.0178292705054	hqer__induced sampler prototype	5.0
	WDI__agriculture	HQER-SFA	hqer__induced	True	5	-736.6123207758883	hqer__induced sampler prototype	2.0
	WDI__agriculture	Quantile-SFA	hqer__induced	True	30	-694.2941584844912	hqer__induced sampler prototype	3.0
	WDI__agriculture	HQER-SFA	hqer__induced	True	8	-687.2339885844672	hqer__induced sampler prototype	2.0
	WDI__agriculture	Quantile-SFA	hqer__induced	True	32	-702.6738204686033	hqer__induced sampler prototype	3.0
	WDI__agriculture	HQER-SFA	hqer__induced	True	15	-696.4363177535757	hqer__induced sampler prototype	2.0
	WDI__agriculture	Quantile-SFA	hqer__induced	True	21	-704.010356285057	hqer__induced sampler prototype	5.0
	WDI__agriculture	HQER-SFA	hqer__induced	True	12	-696.0083383392248	hqer__induced sampler prototype	2.0
	WDI__agriculture	Quantile-SFA	hqer__induced	True	31	-691.8720593522492	hqer__induced sampler prototype	1.0
	WDI__agriculture	HQER-SFA	hqer__induced	True	12	-687.7902084252062	hqer__induced sampler prototype	4.0
	WDI__agriculture	Quantile-SFA	hqer__induced	True	36	-727.4162004345776	hqer__induced sampler prototype	3.0
	WDI__agriculture	HQER-SFA	hqer__induced	True	7	-722.319678412272	hqer__induced sampler prototype	2.0
	WDI__agriculture	Quantile-SFA	hqer__induced	True	40	-702.9272051429581	hqer__induced sampler prototype	3.0
	WDI__agriculture	HQER-SFA	hqer__induced	True	10	-694.5510812719181	hqer__induced sampler prototype	4.0
	WDI__agriculture	Quantile-SFA	hqer__induced	True	29	-719.901872604737	hqer__induced sampler prototype	5.0
	WDI__agriculture	HQER-SFA	hqer__induced	True	14	-714.6220110625591	hqer__induced sampler prototype	2.0
	WDI__agriculture	Quantile-SFA	hqer__induced	True	27	-725.5403451812849	hqer__induced sampler prototype	3.0
	WDI__agriculture	HQER-SFA	hqer__induced	True	17	-721.0283413927631	hqer__induced sampler prototype	4.0
	WDI__agriculture	Quantile-SFA	hqer__induced	True	31	-746.9511807959104	hqer__induced sampler prototype	1.0
	WDI__agriculture	HQER-SFA	hqer__induced	True	9	-735.6492872175028	hqer__induced sampler prototype	4.0
	WDI__agriculture	Quantile-SFA	hqer__induced	True	28	-723.371156028586	hqer__induced sampler prototype	5.0
	WDI__agriculture	HQER-SFA	hqer__induced	True	14	-716.7236361205406	hqer__induced sampler prototype	2.0
	WDI__agriculture	Quantile-SFA	hqer__induced	True	23	-686.0215823697077	hqer__induced sampler prototype	3.0
	WDI__agriculture	HQER-SFA	hqer__induced	True	12	-680.0385792873385	hqer__induced sampler prototype	2.0
	WDI__agriculture	Quantile-SFA	hqer__induced	True	18	-718.601181143998	hqer__induced sampler prototype	5.0
	WDI__agriculture	HQER-SFA	hqer__induced	True	17	-712.2521025630435	hqer__induced sampler prototype	2.0
	WDI__agriculture	Quantile-SFA	hqer__induced	True	29	-695.4330227973247	hqer__induced sampler prototype	3.0
	WDI__agriculture	HQER-SFA	hqer__induced	True	8	-690.7719067818696	hqer__induced sampler prototype	2.0
	WDI__agriculture	Quantile-SFA	hqer__induced	True	32	-723.7054554012773	hqer__induced sampler prototype	1.0
	WDI__agriculture	HQER-SFA	hqer__induced	True	18	-715.7325118516087	hqer__induced sampler prototype	2.0
	WDI__agriculture	Quantile-SFA	hqer__induced	True	31	-724.8249162779063	hqer__induced sampler prototype	5.0
	WDI__agriculture	HQER-SFA	hqer__induced	True	34	-617.4429493252514	hqer__induced sampler prototype	3.0
	WDI__agriculture	Quantile-SFA	hqer__induced	True	20	-713.196645822037	hqer__induced sampler prototype	5.0
	WDI__agriculture	HQER-SFA	hqer__induced	True	15	-702.8909459500237	hqer__induced sampler prototype	4.0
	WDI__agriculture	Quantile-SFA	hqer__induced	True	34	-755.2605475325645	hqer__induced sampler prototype	1.0
	WDI__agriculture	HQER-SFA	hqer__induced	True	13	-748.2937600282079	hqer__induced sampler prototype	4.0
	WDI__agriculture	Quantile-SFA	hqer__induced	True	25	-722.0084791360024	hqer__induced sampler prototype	1.0
	WDI__agriculture	HQER-SFA	hqer__induced	True	7	-712.8476783245549	hqer__induced sampler prototype	2.0
	WDI__agriculture	Quantile-SFA	hqer__induced	True	26	-745.6402740888806	hqer__induced sampler prototype	5.0
	WDI__agriculture	HQER-SFA	hqer__induced	True	11	-742.0856757918144	hqer__induced sampler prototype	4.0
	WDI__agriculture	Quantile-SFA	hqer__induced	True	30	-715.974107124212	hqer__induced sampler prototype	5.0
	WDI__agriculture	HQER-SFA	hqer__induced	True	11	-710.4780828942023	hqer__induced sampler prototype	2.0
	WDI__agriculture	Quantile-SFA	hqer__induced	True	25	-719.5194319616139	hqer__induced sampler prototype	1.0
	WDI__agriculture	HQER-SFA	hqer__induced	True	15	-709.7734857383366	hqer__induced sampler prototype	4.0
	WDI__agriculture	Quantile-SFA	hqer__induced	True	38	-703.3685240693715	hqer__induced sampler prototype	5.0
	WDI__agriculture	HQER-SFA	hqer__induced	True	17	-698.4157296157941	hqer__induced sampler prototype	4.0
	WDI__agriculture	Quantile-SFA	hqer__induced	True	28	-716.2561821047736	hqer__induced sampler prototype	5.0
	WDI__agriculture	HQER-SFA	hqer__induced	True	19	-713.8592046802833	hqer__induced sampler prototype	2.0
	WDI__agriculture	Quantile-SFA	hqer__induced	True	28	-727.3238254630205	hqer__induced sampler prototype	5.0
	WDI__agriculture	HQER-SFA	hqer__induced	True	12	-721.0731247321586	hqer__induced sampler prototype	4.0
	WDI__agriculture	Quantile-SFA	hqer__induced	True	36	-710.2911201993988	hqer__induced sampler prototype	1.0
	WDI__agriculture	HQER-SFA	hqer__induced	True	9	-702.6569660595073	hqer__induced sampler prototype	4.0

experiment	dataset	model	scenario	converged	n_iter	loglik	warning_message	best_start_id
	WDI_agriculture	Quantile-SFA	hqer_induced	True	35	-697.1251941138428	hqer_induced sampler prototype	5.0
	WDI_agriculture	HQER-SFA	hqer_induced	True	9	-696.3772162790962	hqer_induced sampler prototype	2.0
	WDI_agriculture	Quantile-SFA	hqer_induced	True	20	-702.387093048475	hqer_induced sampler prototype	1.0
	WDI_agriculture	HQER-SFA	hqer_induced	True	14	-695.5653243039277	hqer_induced sampler prototype	2.0
	WDI_agriculture	Quantile-SFA	hqer_induced	True	21	-704.1301444527915	hqer_induced sampler prototype	5.0
	WDI_agriculture	HQER-SFA	hqer_induced	True	9	-697.6259091423763	hqer_induced sampler prototype	2.0
	WDI_agriculture	Quantile-SFA	hqer_induced	True	23	-703.9329891505057	hqer_induced sampler prototype	3.0
	WDI_agriculture	HQER-SFA	hqer_induced	True	14	-697.5622089591075	hqer_induced sampler prototype	2.0
	WDI_agriculture	Quantile-SFA	hqer_induced	True	30	-728.0782782503597	hqer_induced sampler prototype	3.0
	WDI_agriculture	HQER-SFA	hqer_induced	True	16	-724.5234940655346	hqer_induced sampler prototype	4.0
	WDI_agriculture	Quantile-SFA	hqer_induced	True	28	-702.8207357703126	hqer_induced sampler prototype	1.0
	WDI_agriculture	HQER-SFA	hqer_induced	True	12	-699.2543254254745	hqer_induced sampler prototype	4.0
	WDI_agriculture	Quantile-SFA	hqer_induced	True	33	-719.2569376611391	hqer_induced sampler prototype	3.0
	WDI_agriculture	HQER-SFA	hqer_induced	True	12	-714.3406813685074	hqer_induced sampler prototype	4.0
	WDI_agriculture	Quantile-SFA	hqer_induced	True	32	-697.721901382022	hqer_induced sampler prototype	3.0
	WDI_agriculture	HQER-SFA	hqer_induced	True	21	-690.0881582355257	hqer_induced sampler prototype	2.0
	WDI_agriculture	Quantile-SFA	hqer_induced	True	28	-694.1192653054503	hqer_induced sampler prototype	3.0
	WDI_agriculture	HQER-SFA	hqer_induced	True	9	-688.8717062537413	hqer_induced sampler prototype	2.0
	WDI_agriculture	Quantile-SFA	hqer_induced	True	32	-751.0874404470658	hqer_induced sampler prototype	3.0
	WDI_agriculture	HQER-SFA	hqer_induced	True	13	-743.221949865293	hqer_induced sampler prototype	2.0
	WDI_agriculture	Quantile-SFA	hqer_induced	True	22	-690.3026410604839	hqer_induced sampler prototype	1.0
	WDI_agriculture	HQER-SFA	hqer_induced	True	14	-690.3271636709801	hqer_induced sampler prototype	4.0
	WDI_agriculture	Quantile-SFA	hqer_induced	True	21	-724.5584523601218	hqer_induced sampler prototype	1.0
	WDI_agriculture	HQER-SFA	hqer_induced	True	8	-716.996626028061	hqer_induced sampler prototype	2.0
	WDI_agriculture	Quantile-SFA	hqer_induced	True	15	-699.5766372398641	hqer_induced sampler prototype	5.0
	WDI_agriculture	HQER-SFA	hqer_induced	True	20	-692.0802033279232	hqer_induced sampler prototype	4.0
	WDI_agriculture	Quantile-SFA	hqer_induced	True	24	-709.9078139294194	hqer_induced sampler prototype	1.0
	WDI_agriculture	HQER-SFA	hqer_induced	True	15	-706.008865235651	hqer_induced sampler prototype	4.0
	WDI_agriculture	Quantile-SFA	hqer_induced	True	34	-701.5031704086405	hqer_induced sampler prototype	3.0
	WDI_agriculture	HQER-SFA	hqer_induced	True	13	-697.1199141252164	hqer_induced sampler prototype	4.0
	WDI_agriculture	Quantile-SFA	hqer_induced	True	31	-720.3625967598491	hqer_induced sampler prototype	5.0
	WDI_agriculture	HQER-SFA	hqer_induced	True	30	-687.016885325891	hqer_induced sampler prototype	5.0
	WDI_agriculture	Quantile-SFA	hqer_induced	True	23	-705.685808332463	hqer_induced sampler prototype	3.0
	WDI_agriculture	HQER-SFA	hqer_induced	True	13	-698.6201997573734	hqer_induced sampler prototype	2.0
	WDI_agriculture	Quantile-SFA	hqer_induced	True	38	-724.2994888725411	hqer_induced sampler prototype	3.0
	WDI_agriculture	HQER-SFA	hqer_induced	True	14	-716.2876942114913	hqer_induced sampler prototype	4.0
	WDI_agriculture	Quantile-SFA	hqer_induced	True	19	-731.4706730071148	hqer_induced sampler prototype	5.0
	WDI_agriculture	HQER-SFA	hqer_induced	True	5	-723.1546435600592	hqer_induced sampler prototype	2.0
	WDI_agriculture	Quantile-SFA	hqer_induced	True	23	-725.5416309587447	hqer_induced sampler prototype	3.0
	WDI_agriculture	HQER-SFA	hqer_induced	True	8	-718.2221440197686	hqer_induced sampler prototype	2.0
	WDI_agriculture	Quantile-SFA	hqer_induced	True	31	-701.6472615493794	hqer_induced sampler prototype	1.0
	WDI_agriculture	HQER-SFA	hqer_induced	True	17	-699.953274434499	hqer_induced sampler prototype	4.0
	WDI_agriculture	Quantile-SFA	hqer_induced	True	28	-696.1521230356635	hqer_induced sampler prototype	5.0
	WDI_agriculture	HQER-SFA	hqer_induced	True	8	-688.0855061737802	hqer_induced sampler prototype	2.0
	WDI_agriculture	Quantile-SFA	hqer_induced	True	20	-702.6802591241437	hqer_induced sampler prototype	5.0
	WDI_agriculture	HQER-SFA	hqer_induced	True	18	-696.9962907331103	hqer_induced sampler prototype	2.0
	WDI_agriculture	Quantile-SFA	hqer_induced	True	28	-731.3890973085972	hqer_induced sampler prototype	5.0
	WDI_agriculture	HQER-SFA	hqer_induced	True	7	-723.2281864174022	hqer_induced sampler prototype	2.0
	WDI_agriculture	Quantile-SFA	hqer_induced	True	27	-733.6225998036149	hqer_induced sampler prototype	5.0
	WDI_agriculture	HQER-SFA	hqer_induced	True	14	-730.8302303338345	hqer_induced sampler prototype	4.0
	WDI_agriculture	Quantile-SFA	hqer_induced	True	37	-679.2233983051582	hqer_induced sampler prototype	1.0
	WDI_agriculture	HQER-SFA	hqer_induced	True	18	-675.3864921304312	hqer_induced sampler prototype	2.0
	WDI_agriculture	Quantile-SFA	hqer_induced	True	33	-695.6970882159042	hqer_induced sampler prototype	1.0
	WDI_agriculture	HQER-SFA	hqer_induced	True	7	-688.7671847552751	hqer_induced sampler prototype	2.0
	WDI_agriculture	Quantile-SFA	hqer_induced	True	24	-741.4662490207271	hqer_induced sampler prototype	5.0
	WDI_agriculture	HQER-SFA	hqer_induced	True	9	-731.5610989880915	hqer_induced sampler prototype	2.0
	WDI_agriculture	Quantile-SFA	hqer_induced	True	25	-727.5340896518492	hqer_induced sampler prototype	3.0
	WDI_agriculture	HQER-SFA	hqer_induced	True	8	-722.9432140141369	hqer_induced sampler prototype	2.0
	WDI_agriculture	Quantile-SFA	hqer_induced	True	20	-736.4929518785337	hqer_induced sampler prototype	1.0
	WDI_agriculture	HQER-SFA	hqer_induced	True	10	-728.8916358682011	hqer_induced sampler prototype	4.0
	WDI_agriculture	Quantile-SFA	hqer_induced	True	32	-722.5983651789834	hqer_induced sampler prototype	5.0
	WDI_agriculture	HQER-SFA	hqer_induced	True	13	-715.7047489428446	hqer_induced sampler prototype	4.0
	WDI_agriculture	Quantile-SFA	hqer_induced	True	31	-675.5996994317485	hqer_induced sampler prototype	1.0
	WDI_agriculture	HQER-SFA	hqer_induced	True	20	-674.1338518378336	hqer_induced sampler prototype	4.0
	WDI_agriculture	Quantile-SFA	hqer_induced	True	29	-712.0369583780325	hqer_induced sampler prototype	3.0
	WDI_agriculture	HQER-SFA	hqer_induced	True	11	-709.804729699801	hqer_induced sampler prototype	4.0
	WDI_agriculture	Quantile-SFA	hqer_induced	True	20	-730.1377076277079	hqer_induced sampler prototype	5.0
	WDI_agriculture	HQER-SFA	hqer_induced	True	11	-721.3370953099823	hqer_induced sampler prototype	4.0
	WDI_agriculture	Quantile-SFA	hqer_induced	True	31	-695.2721740694464	hqer_induced sampler prototype	5.0
	WDI_agriculture	HQER-SFA	hqer_induced	True	10	-692.6822599608906	hqer_induced sampler prototype	4.0
	WDI_agriculture	Quantile-SFA	hqer_induced	True	23	-699.0490388207998	hqer_induced sampler prototype	3.0
	WDI_agriculture	HQER-SFA	hqer_induced	True	25	-693.4841138654174	hqer_induced sampler prototype	2.0
	WDI_agriculture	Quantile-SFA	hqer_induced	True	28	-686.1793322530415	hqer_induced sampler prototype	5.0
	WDI_agriculture	HQER-SFA	hqer_induced	True	13	-680.7964581440252	hqer_induced sampler prototype	2.0
	WDI_agriculture	Quantile-SFA	hqer_induced	True	24	-747.3125545574571	hqer_induced sampler prototype	3.0
	WDI_agriculture	HQER-SFA	hqer_induced	True	6	-740.2614712492082	hqer_induced sampler prototype	2.0
	WDI_agriculture	Quantile-SFA	hqer_induced	True	30	-711.6882875581182	hqer_induced sampler prototype	1.0
	WDI_agriculture	HQER-SFA	hqer_induced	True	12	-706.8998576491715	hqer_induced sampler prototype	4.0
	WDI_agriculture	Quantile-SFA	hqer_induced	True	25	-709.0312968262688	hqer_induced sampler prototype	5.0
	WDI_agriculture	HQER-SFA	hqer_induced	True	7	-710.3586407958488	hqer_induced sampler prototype	2.0
	WDI_agriculture	Quantile-SFA	hqer_induced	True	32	-727.8557175559421	hqer_induced sampler prototype	1.0
	WDI_agriculture	HQER-SFA	hqer_induced	True	16	-723.3986305753841	hqer_induced sampler prototype	4.0
	WDI_agriculture	Quantile-SFA	hqer_induced	True	26	-728.9917640188798	hqer_induced sampler prototype	3.0
	WDI_agriculture	HQER-SFA	hqer_induced	True	8	-723.4413710030359	hqer_induced sampler prototype	2.0
	WDI_agriculture	Quantile-SFA	hqer_induced	True	29	-725.5173809310321	hqer_induced sampler prototype	3.0
	WDI_agriculture	HQER-SFA	hqer_induced	True	14	-718.7524789369938	hqer_induced sampler prototype	4.0
	WDI_agriculture	Quantile-SFA	hqer_induced	True	50	-710.1467882077243	hqer_induced sampler prototype	3.0
	WDI_agriculture	HQER-SFA	hqer_induced	True	10	-702.2412058711068	hqer_induced sampler prototype	4.0
	WDI_agriculture	Quantile-SFA	hqer_induced	True	24	-729.4526005853638	hqer_induced sampler prototype	5.0
	WDI_agriculture	HQER-SFA	hqer_induced	True	8	-719.901128256444	hqer_induced sampler prototype	2.0
	WDI_agriculture	Quantile-SFA	hqer_induced	True	30	-706.2989249834404	hqer_induced sampler prototype	1.0
	WDI_agriculture	HQER-SFA	hqer_induced	True	12	-694.6862552905183	hqer_induced sampler prototype	2.0
	WDI_agriculture	Quantile-SFA	hqer_induced	True	35	-703.68672430031	hqer_induced sampler prototype	1.0
	WDI_agriculture	HQER-SFA	hqer_induced	True	7	-699.8109317902267	hqer_induced sampler prototype	2.0
	WDI_agriculture	Quantile-SFA	hqer_induced	True	28	-718.078279750519	hqer_induced sampler prototype	5.0
	WDI_agriculture	HQER-SFA	hqer_induced	True	11	-715.8005928794732	hqer_induced sampler prototype	2.0
	WDI_agriculture	Quantile-SFA	hqer_induced	True	31	-703.2499045967311	hqer_induced sampler prototype	1.0
	WDI_agriculture	HQER-SFA	hqer_induced	True	17	-696.2083062175933	hqer_induced sampler prototype	4.0
	WDI_agriculture	Quantile-SFA	hqer_induced	True	33	-713.4994778430417	hqer_induced sampler prototype	3.0
	WDI_agriculture	HQER-SFA	hqer_induced	True	8	-706.6743514963014	hqer_induced sampler prototype	2.0
	WDI_agriculture	Quantile-SFA	hqer_induced	True	22	-701.3925988684706	hqer_induced sampler prototype	1.0
	WDI_agriculture	HQER-SFA	hqer_induced	True	11	-694.8365437261352	hqer_induced sampler prototype	4.0
	WDI_agriculture	Quantile-SFA	hqer_induced	True	25	-731.8163002478504	hqer_induced sampler prototype	5.0

experiment	dataset	model	scenario	converged	n_iter	loglik	warning_message	best_start_id
	WDI_agriculture	HQER-SFA	hqer_induced	True	15	-728.1015143669538	hqer_induced sampler prototype	2.0
	WDI_agriculture	Quantile-SFA	hqer_induced	True	22	-686.3118911688637	hqer_induced sampler prototype	5.0
	WDI_agriculture	HQER-SFA	hqer_induced	True	18	-676.4524519538431	hqer_induced sampler prototype	2.0
	WDI_agriculture	Quantile-SFA	hqer_induced	True	30	-707.4508804452036	hqer_induced sampler prototype	5.0
	WDI_agriculture	HQER-SFA	hqer_induced	True	11	-700.999605436325	hqer_induced sampler prototype	4.0
	WDI_agriculture	Quantile-SFA	hqer_induced	True	25	-710.6592088921959	hqer_induced sampler prototype	1.0
	WDI_agriculture	HQER-SFA	hqer_induced	True	13	-705.157788992952	hqer_induced sampler prototype	2.0
	WDI_agriculture	Quantile-SFA	hqer_induced	True	31	-730.5714922071032	hqer_induced sampler prototype	5.0
	WDI_agriculture	HQER-SFA	hqer_induced	True	18	-724.9216476596698	hqer_induced sampler prototype	4.0
	WDI_agriculture	Quantile-SFA	hqer_induced	True	45	-717.8407456470487	hqer_induced sampler prototype	1.0
	WDI_agriculture	HQER-SFA	hqer_induced	True	16	-710.3186952345905	hqer_induced sampler prototype	2.0
	WDI_agriculture	Quantile-SFA	hqer_induced	True	22	-728.5700889539168	hqer_induced sampler prototype	1.0
	WDI_agriculture	HQER-SFA	hqer_induced	True	15	-723.3511979583147	hqer_induced sampler prototype	2.0
	WDI_agriculture	Quantile-SFA	hqer_induced	True	29	-710.0786953097556	hqer_induced sampler prototype	3.0
	WDI_agriculture	HQER-SFA	hqer_induced	True	12	-704.8876836696559	hqer_induced sampler prototype	2.0
	WDI_agriculture	Quantile-SFA	hqer_induced	True	16	-712.4952073206878	hqer_induced sampler prototype	5.0
	WDI_agriculture	HQER-SFA	hqer_induced	True	7	-706.1129062650491	hqer_induced sampler prototype	2.0
	WDI_agriculture	Quantile-SFA	hqer_induced	True	42	-712.5708588534449	hqer_induced sampler prototype	3.0
	WDI_agriculture	HQER-SFA	hqer_induced	True	13	-712.6713859022111	hqer_induced sampler prototype	2.0
	WDI_agriculture	Quantile-SFA	hqer_induced	True	19	-737.5430432938897	hqer_induced sampler prototype	5.0
	WDI_agriculture	HQER-SFA	hqer_induced	True	13	-731.7530003782529	hqer_induced sampler prototype	4.0
	WDI_agriculture	Quantile-SFA	hqer_induced	True	21	-740.4086020401749	hqer_induced sampler prototype	5.0
	WDI_agriculture	HQER-SFA	hqer_induced	True	23	-681.3261634098704	hqer_induced sampler prototype	1.0
	WDI_agriculture	Quantile-SFA	hqer_induced	True	31	-691.7607577384072	hqer_induced sampler prototype	3.0
	WDI_agriculture	HQER-SFA	hqer_induced	True	16	-684.6007377136149	hqer_induced sampler prototype	2.0
	WDI_agriculture	Quantile-SFA	hqer_induced	True	29	-729.8643215793663	hqer_induced sampler prototype	5.0
	WDI_agriculture	HQER-SFA	hqer_induced	True	7	-725.7674028575702	hqer_induced sampler prototype	2.0
	WDI_agriculture	Quantile-SFA	hqer_induced	True	22	-711.3410346213898	hqer_induced sampler prototype	3.0
	WDI_agriculture	HQER-SFA	hqer_induced	True	10	-705.0748932082056	hqer_induced sampler prototype	2.0
	WDI_agriculture	Quantile-SFA	hqer_induced	True	25	-722.3922640019827	hqer_induced sampler prototype	1.0
	WDI_agriculture	HQER-SFA	hqer_induced	True	12	-716.043017124284	hqer_induced sampler prototype	2.0
	WDI_agriculture	Quantile-SFA	hqer_induced	True	33	-740.2836025004623	hqer_induced sampler prototype	1.0
	WDI_agriculture	HQER-SFA	hqer_induced	True	17	-736.2952748359651	hqer_induced sampler prototype	2.0
	WDI_agriculture	Quantile-SFA	hqer_induced	True	25	-709.0934315274955	hqer_induced sampler prototype	3.0
	WDI_agriculture	HQER-SFA	hqer_induced	True	10	-704.4586350021966	hqer_induced sampler prototype	2.0
	WDI_agriculture	Quantile-SFA	hqer_induced	True	32	-722.5750295428521	hqer_induced sampler prototype	1.0
	WDI_agriculture	HQER-SFA	hqer_induced	True	12	-715.5578625075898	hqer_induced sampler prototype	4.0
	WDI_agriculture	Quantile-SFA	hqer_induced	True	24	-707.2430097503974	hqer_induced sampler prototype	1.0
	WDI_agriculture	HQER-SFA	hqer_induced	True	7	-697.123405397717	hqer_induced sampler prototype	4.0
	WDI_agriculture	Quantile-SFA	hqer_induced	True	19	-725.2335717983125	hqer_induced sampler prototype	5.0
	WDI_agriculture	HQER-SFA	hqer_induced	True	12	-717.992130966177	hqer_induced sampler prototype	4.0
	WDI_agriculture	Quantile-SFA	hqer_induced	True	35	-709.2618527029813	hqer_induced sampler prototype	3.0
	WDI_agriculture	HQER-SFA	hqer_induced	True	3	-704.645869866413	hqer_induced sampler prototype	2.0
	WDI_agriculture	Quantile-SFA	hqer_induced	True	20	-754.6566203040279	hqer_induced sampler prototype	1.0
	WDI_agriculture	HQER-SFA	hqer_induced	True	10	-748.5906306360762	hqer_induced sampler prototype	2.0
	WDI_agriculture	Quantile-SFA	hqer_induced	True	24	-724.4018536422201	hqer_induced sampler prototype	1.0
	WDI_agriculture	HQER-SFA	hqer_induced	True	16	-719.7827440810495	hqer_induced sampler prototype	4.0
	WDI_agriculture	Quantile-SFA	hqer_induced	True	25	-775.7858089038975	hqer_induced sampler prototype	5.0
	WDI_agriculture	HQER-SFA	hqer_induced	True	10	-766.6066663188226	hqer_induced sampler prototype	4.0
	WDI_agriculture	Quantile-SFA	hqer_induced	True	30	-731.017336530618	hqer_induced sampler prototype	3.0
	WDI_agriculture	HQER-SFA	hqer_induced	True	11	-722.0790184711594	hqer_induced sampler prototype	2.0
	WDI_agriculture	Quantile-SFA	hqer_induced	True	35	-695.6779831826823	hqer_induced sampler prototype	1.0
	WDI_agriculture	HQER-SFA	hqer_induced	True	8	-688.1425738434764	hqer_induced sampler prototype	4.0
	WDI_agriculture	Quantile-SFA	hqer_induced	True	26	-734.4432901646336	hqer_induced sampler prototype	5.0
	WDI_agriculture	HQER-SFA	hqer_induced	True	10	-729.6003925943901	hqer_induced sampler prototype	4.0
	WDI_agriculture	Quantile-SFA	hqer_induced	True	33	-695.8053763050303	hqer_induced sampler prototype	1.0
	WDI_agriculture	HQER-SFA	hqer_induced	True	14	-687.8495560156207	hqer_induced sampler prototype	2.0
	WDI_agriculture	Quantile-SFA	hqer_induced	True	27	-700.7991072521733	hqer_induced sampler prototype	3.0
	WDI_agriculture	HQER-SFA	hqer_induced	True	11	-692.6236246424844	hqer_induced sampler prototype	2.0
	WDI_agriculture	Quantile-SFA	hqer_induced	True	23	-717.6389377976184	hqer_induced sampler prototype	3.0
	WDI_agriculture	HQER-SFA	hqer_induced	True	15	-712.6104318605564	hqer_induced sampler prototype	4.0
	WDI_agriculture	Quantile-SFA	hqer_induced	True	30	-709.0604577913859	hqer_induced sampler prototype	5.0
	WDI_agriculture	HQER-SFA	hqer_induced	True	12	-702.8915879921974	hqer_induced sampler prototype	2.0
	WDI_agriculture	Quantile-SFA	hqer_induced	True	31	-684.041097382157	hqer_induced sampler prototype	1.0
	WDI_agriculture	HQER-SFA	hqer_induced	True	9	-676.1959093883364	hqer_induced sampler prototype	2.0
	WDI_agriculture	Quantile-SFA	hqer_induced	True	22	-730.5649833416614	hqer_induced sampler prototype	1.0
	WDI_agriculture	HQER-SFA	hqer_induced	True	7	-722.1099179714108	hqer_induced sampler prototype	4.0
	WDI_agriculture	Quantile-SFA	hqer_induced	True	28	-733.9020647332455	hqer_induced sampler prototype	1.0
	WDI_agriculture	HQER-SFA	hqer_induced	True	11	-728.3532790462691	hqer_induced sampler prototype	4.0
	WDI_agriculture	Quantile-SFA	hqer_induced	True	33	-716.674323847684	hqer_induced sampler prototype	1.0
	WDI_agriculture	HQER-SFA	hqer_induced	True	16	-710.9293074107895	hqer_induced sampler prototype	2.0
	WDI_agriculture	Quantile-SFA	hqer_induced	True	24	-749.4124866515017	hqer_induced sampler prototype	1.0
	WDI_agriculture	HQER-SFA	hqer_induced	True	8	-745.3475871980373	hqer_induced sampler prototype	2.0
	WDI_agriculture	Quantile-SFA	hqer_induced	True	25	-732.8890644107481	hqer_induced sampler prototype	5.0
	WDI_agriculture	HQER-SFA	hqer_induced	True	15	-727.2263697596239	hqer_induced sampler prototype	4.0
	WDI_agriculture	Quantile-SFA	hqer_induced	True	35	-697.5298347619851	hqer_induced sampler prototype	3.0
	WDI_agriculture	HQER-SFA	hqer_induced	True	22	-698.3160538287616	hqer_induced sampler prototype	2.0
	WDI_agriculture	Quantile-SFA	hqer_induced	True	35	-682.8443409803156	hqer_induced sampler prototype	1.0
	WDI_agriculture	HQER-SFA	hqer_induced	True	11	-674.4840984789626	hqer_induced sampler prototype	2.0
	WDI_agriculture	Quantile-SFA	hqer_induced	True	22	-743.15646109218	hqer_induced sampler prototype	3.0
	WDI_agriculture	HQER-SFA	hqer_induced	True	22	-736.374560845295	hqer_induced sampler prototype	2.0
	WDI_agriculture	Quantile-SFA	hqer_induced	True	31	-719.9350149660493	hqer_induced sampler prototype	3.0
	WDI_agriculture	HQER-SFA	hqer_induced	True	16	-713.7325187853362	hqer_induced sampler prototype	2.0
	WDI_agriculture	Quantile-SFA	hqer_induced	True	26	-702.5592625778708	hqer_induced sampler prototype	5.0
	WDI_agriculture	HQER-SFA	hqer_induced	True	5	-694.2409658549677	hqer_induced sampler prototype	2.0
	WDI_agriculture	Quantile-SFA	hqer_induced	True	30	-709.7593767313916	hqer_induced sampler prototype	3.0
	WDI_agriculture	HQER-SFA	hqer_induced	True	14	-704.9867662575413	hqer_induced sampler prototype	2.0
	WDI_agriculture	Quantile-SFA	hqer_induced	True	23	-714.0395531589506	hqer_induced sampler prototype	3.0
	WDI_agriculture	HQER-SFA	hqer_induced	True	13	-704.3973066027482	hqer_induced sampler prototype	4.0
	WDI_agriculture	Quantile-SFA	hqer_induced	True	19	-710.9901969491086	hqer_induced sampler prototype	1.0
	WDI_agriculture	HQER-SFA	hqer_induced	True	8	-702.5285738402747	hqer_induced sampler prototype	2.0
	WDI_agriculture	Quantile-SFA	hqer_induced	True	28	-715.3772172729655	hqer_induced sampler prototype	5.0
	WDI_agriculture	HQER-SFA	hqer_induced	True	14	-708.102376970631	hqer_induced sampler prototype	4.0
	WDI_agriculture	Quantile-SFA	hqer_induced	True	27	-737.515235881589	hqer_induced sampler prototype	5.0
	WDI_agriculture	HQER-SFA	hqer_induced	True	21	-728.272373277239	hqer_induced sampler prototype	4.0
	WDI_agriculture	Quantile-SFA	hqer_induced	True	33	-745.1892983980752	hqer_induced sampler prototype	3.0
	WDI_agriculture	HQER-SFA	hqer_induced	True	7	-737.7606227838512	hqer_induced sampler prototype	2.0
	WDI_agriculture	Quantile-SFA	hqer_induced	True	29	-711.7700844421405	hqer_induced sampler prototype	1.0
	WDI_agriculture	HQER-SFA	hqer_induced	True	8	-708.464205866705	hqer_induced sampler prototype	2.0
	WDI_agriculture	Quantile-SFA	hqer_induced	True	20	-702.9744649596125	hqer_induced sampler prototype	3.0
	WDI_agriculture	HQER-SFA	hqer_induced	True	11	-695.4754928780517	hqer_induced sampler prototype	4.0
	WDI_agriculture	Quantile-SFA	hqer_induced	True	28	-729.5605277609869	hqer_induced sampler prototype	1.0
	WDI_agriculture	HQER-SFA	hqer_induced	True	10	-720.2221138316256	hqer_induced sampler prototype	2.0

experiment	dataset	model	scenario	converged	n_iter	loglik	warning_message	best_start_id
	WDI_agriculture	Quantile-SFA	hqer_induced	True	24	-718.5299340765534	hqer_induced sampler prototype	1.0
	WDI_agriculture	HQER-SFA	hqer_induced	True	21	-712.3591111185337	hqer_induced sampler prototype	4.0
	WDI_agriculture	Quantile-SFA	hqer_induced	True	25	-734.5320379066181	hqer_induced sampler prototype	1.0
	WDI_agriculture	HQER-SFA	hqer_induced	True	12	-725.1060032464407	hqer_induced sampler prototype	2.0
	WDI_agriculture	Quantile-SFA	hqer_induced	True	31	-716.8080802636637	hqer_induced sampler prototype	1.0
	WDI_agriculture	HQER-SFA	hqer_induced	True	10	-709.2813543119369	hqer_induced sampler prototype	4.0
	WDI_agriculture	Quantile-SFA	hqer_induced	True	44	-719.2566238068978	hqer_induced sampler prototype	3.0
	WDI_agriculture	HQER-SFA	hqer_induced	True	18	-713.8926013873952	hqer_induced sampler prototype	2.0
	WDI_agriculture	Quantile-SFA	hqer_induced	True	26	-740.0142654258561	hqer_induced sampler prototype	1.0
	WDI_agriculture	HQER-SFA	hqer_induced	True	13	-731.1314440579201	hqer_induced sampler prototype	2.0
	WDI_agriculture	Quantile-SFA	hqer_induced	True	21	-713.9806413043644	hqer_induced sampler prototype	3.0
	WDI_agriculture	HQER-SFA	hqer_induced	True	8	-704.2230783826013	hqer_induced sampler prototype	2.0
	WDI_agriculture	Quantile-SFA	hqer_induced	True	24	-737.8589839076221	hqer_induced sampler prototype	1.0
	WDI_agriculture	HQER-SFA	hqer_induced	True	11	-732.8281436661568	hqer_induced sampler prototype	2.0
	WDI_agriculture	Quantile-SFA	hqer_induced	True	23	-712.196252988083	hqer_induced sampler prototype	1.0
	WDI_agriculture	HQER-SFA	hqer_induced	True	18	-703.8910090331296	hqer_induced sampler prototype	2.0
	WDI_agriculture	Quantile-SFA	hqer_induced	True	27	-700.4542917484239	hqer_induced sampler prototype	5.0
	WDI_agriculture	HQER-SFA	hqer_induced	True	11	-693.1297745609932	hqer_induced sampler prototype	4.0
	WDI_agriculture	Quantile-SFA	hqer_induced	True	18	-705.7187119141063	hqer_induced sampler prototype	5.0
	WDI_agriculture	HQER-SFA	hqer_induced	True	8	-696.033373112941	hqer_induced sampler prototype	2.0
	WDI_agriculture	Quantile-SFA	hqer_induced	True	39	-718.0815260825489	hqer_induced sampler prototype	1.0
	WDI_agriculture	HQER-SFA	hqer_induced	True	13	-712.5837095830173	hqer_induced sampler prototype	2.0
	WDI_agriculture	Quantile-SFA	hqer_induced	True	16	-724.5730793231446	hqer_induced sampler prototype	5.0
	WDI_agriculture	HQER-SFA	hqer_induced	True	12	-717.467496718602	hqer_induced sampler prototype	2.0
	WDI_agriculture	Quantile-SFA	hqer_induced	True	26	-701.6690674621465	hqer_induced sampler prototype	5.0
	WDI_agriculture	HQER-SFA	hqer_induced	True	18	-696.765821277937	hqer_induced sampler prototype	2.0
	WDI_agriculture	Quantile-SFA	hqer_induced	True	30	-703.1780059065759	hqer_induced sampler prototype	1.0
	WDI_agriculture	HQER-SFA	hqer_induced	True	26	-698.6671930646527	hqer_induced sampler prototype	4.0
	WDI_agriculture	Quantile-SFA	hqer_induced	True	32	-746.29828618596	hqer_induced sampler prototype	5.0
	WDI_agriculture	HQER-SFA	hqer_induced	True	9	-741.6906722821457	hqer_induced sampler prototype	2.0
	WDI_agriculture	Quantile-SFA	hqer_induced	True	22	-718.6028002353848	hqer_induced sampler prototype	5.0
	WDI_agriculture	HQER-SFA	hqer_induced	True	6	-710.6106733235388	hqer_induced sampler prototype	4.0
	WDI_agriculture	Quantile-SFA	hqer_induced	True	23	-732.9291421164677	hqer_induced sampler prototype	3.0
	WDI_agriculture	HQER-SFA	hqer_induced	True	10	-722.9990561956392	hqer_induced sampler prototype	2.0
	WDI_agriculture	Quantile-SFA	hqer_induced	True	31	-694.8493567812175	hqer_induced sampler prototype	3.0
	WDI_agriculture	HQER-SFA	hqer_induced	True	15	-690.0105657438452	hqer_induced sampler prototype	2.0
	WDI_agriculture	Quantile-SFA	hqer_induced	True	27	-716.2330946674515	hqer_induced sampler prototype	5.0
	WDI_agriculture	HQER-SFA	hqer_induced	True	7	-707.5546734548907	hqer_induced sampler prototype	4.0
	WDI_agriculture	Quantile-SFA	hqer_induced	True	28	-732.0926526081196	hqer_induced sampler prototype	1.0
	WDI_agriculture	HQER-SFA	hqer_induced	True	12	-728.4162552739037	hqer_induced sampler prototype	4.0
	WDI_agriculture	Quantile-SFA	hqer_induced	True	41	-713.1065316558313	hqer_induced sampler prototype	5.0
	WDI_agriculture	HQER-SFA	hqer_induced	True	7	-714.0140903908322	hqer_induced sampler prototype	2.0
	WDI_agriculture	Quantile-SFA	hqer_induced	True	26	-2869.048399242045	hqer_induced sampler prototype	5.0
	WDI_agriculture	HQER-SFA	hqer_induced	True	10	-2839.997878610848	hqer_induced sampler prototype	2.0
	WDI_agriculture	Quantile-SFA	hqer_induced	True	22	-2913.835178510752	hqer_induced sampler prototype	1.0
	WDI_agriculture	HQER-SFA	hqer_induced	True	12	-2884.172449279908	hqer_induced sampler prototype	2.0
	WDI_agriculture	Quantile-SFA	hqer_induced	True	26	-2862.574170262852	hqer_induced sampler prototype	1.0
	WDI_agriculture	HQER-SFA	hqer_induced	True	15	-2833.984002101065	hqer_induced sampler prototype	4.0
	WDI_agriculture	Quantile-SFA	hqer_induced	True	20	-2881.8626994764063	hqer_induced sampler prototype	1.0
	WDI_agriculture	HQER-SFA	hqer_induced	True	8	-2851.938321113974	hqer_induced sampler prototype	4.0
	WDI_agriculture	Quantile-SFA	hqer_induced	True	25	-2843.08474745945	hqer_induced sampler prototype	3.0
	WDI_agriculture	HQER-SFA	hqer_induced	True	12	-2811.3229391693367	hqer_induced sampler prototype	2.0
	WDI_agriculture	Quantile-SFA	hqer_induced	True	30	-2910.9960274918976	hqer_induced sampler prototype	1.0
	WDI_agriculture	HQER-SFA	hqer_induced	True	10	-2894.636493280525	hqer_induced sampler prototype	2.0
	WDI_agriculture	Quantile-SFA	hqer_induced	True	19	-2898.557347350048	hqer_induced sampler prototype	1.0
	WDI_agriculture	HQER-SFA	hqer_induced	True	16	-2870.93556690898	hqer_induced sampler prototype	4.0
	WDI_agriculture	Quantile-SFA	hqer_induced	True	28	-2901.5766405137365	hqer_induced sampler prototype	3.0
	WDI_agriculture	HQER-SFA	hqer_induced	True	9	-2867.359817843665	hqer_induced sampler prototype	4.0
	WDI_agriculture	Quantile-SFA	hqer_induced	True	32	-2901.419856575933	hqer_induced sampler prototype	1.0
	WDI_agriculture	HQER-SFA	hqer_induced	True	9	-2878.8848383475624	hqer_induced sampler prototype	4.0
	WDI_agriculture	Quantile-SFA	hqer_induced	True	24	-2896.250785258801	hqer_induced sampler prototype	5.0
	WDI_agriculture	HQER-SFA	hqer_induced	True	10	-2859.830258271969	hqer_induced sampler prototype	4.0
	WDI_agriculture	Quantile-SFA	hqer_induced	True	25	-2834.687809789936	hqer_induced sampler prototype	5.0
	WDI_agriculture	HQER-SFA	hqer_induced	True	10	-2809.151595172467	hqer_induced sampler prototype	2.0
	WDI_agriculture	Quantile-SFA	hqer_induced	True	40	-2898.845260832746	hqer_induced sampler prototype	3.0
	WDI_agriculture	HQER-SFA	hqer_induced	True	13	-2866.6051724566805	hqer_induced sampler prototype	2.0
	WDI_agriculture	Quantile-SFA	hqer_induced	True	18	-2925.4900551417486	hqer_induced sampler prototype	3.0
	WDI_agriculture	HQER-SFA	hqer_induced	True	7	-2894.703076804097	hqer_induced sampler prototype	2.0
	WDI_agriculture	Quantile-SFA	hqer_induced	True	22	-2878.2423774652325	hqer_induced sampler prototype	5.0
	WDI_agriculture	HQER-SFA	hqer_induced	True	10	-2853.461293459124	hqer_induced sampler prototype	4.0
	WDI_agriculture	Quantile-SFA	hqer_induced	True	17	-2892.4750254737173	hqer_induced sampler prototype	5.0
	WDI_agriculture	HQER-SFA	hqer_induced	True	10	-2867.0858530027485	hqer_induced sampler prototype	2.0
	WDI_agriculture	Quantile-SFA	hqer_induced	True	23	-2917.3702304284016	hqer_induced sampler prototype	1.0
	WDI_agriculture	HQER-SFA	hqer_induced	True	10	-2885.3425398104982	hqer_induced sampler prototype	4.0
	WDI_agriculture	Quantile-SFA	hqer_induced	True	26	-2889.7758927781806	hqer_induced sampler prototype	1.0
	WDI_agriculture	HQER-SFA	hqer_induced	True	9	-2863.4065973055467	hqer_induced sampler prototype	4.0
	WDI_agriculture	Quantile-SFA	hqer_induced	True	30	-2894.798761056228	hqer_induced sampler prototype	3.0
	WDI_agriculture	HQER-SFA	hqer_induced	True	10	-2855.5293800630934	hqer_induced sampler prototype	2.0
	WDI_agriculture	Quantile-SFA	hqer_induced	True	28	-2821.817168352868	hqer_induced sampler prototype	1.0
	WDI_agriculture	HQER-SFA	hqer_induced	True	23	-2793.435443515431	hqer_induced sampler prototype	4.0
	WDI_agriculture	Quantile-SFA	hqer_induced	True	34	-2861.471600044633	hqer_induced sampler prototype	3.0
	WDI_agriculture	HQER-SFA	hqer_induced	True	8	-2837.30526921316	hqer_induced sampler prototype	4.0
	WDI_agriculture	Quantile-SFA	hqer_induced	True	20	-2913.3375580804945	hqer_induced sampler prototype	3.0
	WDI_agriculture	HQER-SFA	hqer_induced	True	11	-2887.4522566425103	hqer_induced sampler prototype	2.0
	WDI_agriculture	Quantile-SFA	hqer_induced	True	16	-2864.791154603361	hqer_induced sampler prototype	5.0
	WDI_agriculture	HQER-SFA	hqer_induced	True	12	-2833.4564962661243	hqer_induced sampler prototype	2.0
	WDI_agriculture	Quantile-SFA	hqer_induced	True	17	-2849.342924320392	hqer_induced sampler prototype	5.0
	WDI_agriculture	HQER-SFA	hqer_induced	True	8	-2813.042008990101	hqer_induced sampler prototype	2.0
	WDI_agriculture	Quantile-SFA	hqer_induced	True	29	-2920.846025057473	hqer_induced sampler prototype	1.0
	WDI_agriculture	HQER-SFA	hqer_induced	True	7	-2890.024841299822	hqer_induced sampler prototype	4.0
	WDI_agriculture	Quantile-SFA	hqer_induced	True	22	-2922.3318500700507	hqer_induced sampler prototype	5.0
	WDI_agriculture	HQER-SFA	hqer_induced	True	13	-2886.6641510159416	hqer_induced sampler prototype	4.0
	WDI_agriculture	Quantile-SFA	hqer_induced	True	31	-2831.8432399482126	hqer_induced sampler prototype	3.0
	WDI_agriculture	HQER-SFA	hqer_induced	True	10	-2813.778462525668	hqer_induced sampler prototype	2.0
	WDI_agriculture	Quantile-SFA	hqer_induced	True	29	-2902.909922575022	hqer_induced sampler prototype	3.0
	WDI_agriculture	HQER-SFA	hqer_induced	True	17	-2873.574111275936	hqer_induced sampler prototype	2.0
	WDI_agriculture	Quantile-SFA	hqer_induced	True	22	-2888.6031571640024	hqer_induced sampler prototype	5.0
	WDI_agriculture	HQER-SFA	hqer_induced	True	11	-2856.2328414950744	hqer_induced sampler prototype	4.0
	WDI_agriculture	Quantile-SFA	hqer_induced	True	27	-2872.764666593236	hqer_induced sampler prototype	5.0
	WDI_agriculture	HQER-SFA	hqer_induced	True	9	-2854.215458097946	hqer_induced sampler prototype	4.0
	WDI_agriculture	Quantile-SFA	hqer_induced	True	26	-2830.356089927268	hqer_induced sampler prototype	5.0
	WDI_agriculture	HQER-SFA	hqer_induced	True	6	-2793.9060008836022	hqer_induced sampler prototype	4.0
	WDI_agriculture	Quantile-SFA	hqer_induced	True	22	-2931.5626902500944	hqer_induced sampler prototype	5.0

experiment	dataset	model	scenario	converged	n_iter	loglik	warning_message	best_start_id
	WDI_agriculture	HQER-SFA	hqer_induced	True	14	-2897.4458187618143	hqer_induced sampler prototype	4.0
	WDI_agriculture	Quantile-SFA	hqer_induced	True	18	-2864.939437966082	hqer_induced sampler prototype	1.0
	WDI_agriculture	HQER-SFA	hqer_induced	True	11	-2836.446849110972	hqer_induced sampler prototype	4.0
	WDI_agriculture	Quantile-SFA	hqer_induced	True	30	-2915.151353827181	hqer_induced sampler prototype	5.0
	WDI_agriculture	HQER-SFA	hqer_induced	True	9	-2893.595864742647	hqer_induced sampler prototype	4.0
	WDI_agriculture	Quantile-SFA	hqer_induced	True	19	-2923.2008051844973	hqer_induced sampler prototype	5.0
	WDI_agriculture	HQER-SFA	hqer_induced	True	11	-2900.7165568747605	hqer_induced sampler prototype	4.0
	WDI_agriculture	Quantile-SFA	hqer_induced	True	22	-2899.7278604021303	hqer_induced sampler prototype	1.0
	WDI_agriculture	HQER-SFA	hqer_induced	True	7	-2872.430814552133	hqer_induced sampler prototype	2.0
	WDI_agriculture	Quantile-SFA	hqer_induced	True	21	-2875.949807633724	hqer_induced sampler prototype	5.0
	WDI_agriculture	HQER-SFA	hqer_induced	True	8	-2854.319577312757	hqer_induced sampler prototype	4.0
	WDI_agriculture	Quantile-SFA	hqer_induced	True	30	-2821.5555226228576	hqer_induced sampler prototype	5.0
	WDI_agriculture	HQER-SFA	hqer_induced	True	10	-2803.390476280621	hqer_induced sampler prototype	4.0
	WDI_agriculture	Quantile-SFA	hqer_induced	True	34	-2848.5953044058383	hqer_induced sampler prototype	3.0
	WDI_agriculture	HQER-SFA	hqer_induced	True	8	-2822.603021215414	hqer_induced sampler prototype	4.0
	WDI_agriculture	Quantile-SFA	hqer_induced	True	24	-2875.408485746548	hqer_induced sampler prototype	1.0
	WDI_agriculture	HQER-SFA	hqer_induced	True	7	-2847.9587100248464	hqer_induced sampler prototype	2.0
	WDI_agriculture	Quantile-SFA	hqer_induced	True	23	-2900.052133277565	hqer_induced sampler prototype	3.0
	WDI_agriculture	HQER-SFA	hqer_induced	True	7	-2873.2320404406933	hqer_induced sampler prototype	2.0
	WDI_agriculture	Quantile-SFA	hqer_induced	True	26	-2876.009648193548	hqer_induced sampler prototype	1.0
	WDI_agriculture	HQER-SFA	hqer_induced	True	12	-2844.59919781899	hqer_induced sampler prototype	4.0
	WDI_agriculture	Quantile-SFA	hqer_induced	True	25	-2895.340422667522	hqer_induced sampler prototype	3.0
	WDI_agriculture	HQER-SFA	hqer_induced	True	14	-2863.054119213517	hqer_induced sampler prototype	2.0
	WDI_agriculture	Quantile-SFA	hqer_induced	True	15	-2891.885714286452	hqer_induced sampler prototype	5.0
	WDI_agriculture	HQER-SFA	hqer_induced	True	13	-2868.1938982343427	hqer_induced sampler prototype	2.0
	WDI_agriculture	Quantile-SFA	hqer_induced	True	19	-2884.3000383467147	hqer_induced sampler prototype	5.0
	WDI_agriculture	HQER-SFA	hqer_induced	True	13	-2849.661541961957	hqer_induced sampler prototype	2.0
	WDI_agriculture	Quantile-SFA	hqer_induced	True	22	-2911.6294953431616	hqer_induced sampler prototype	5.0
	WDI_agriculture	HQER-SFA	hqer_induced	True	9	-2882.175720624989	hqer_induced sampler prototype	2.0
	WDI_agriculture	Quantile-SFA	hqer_induced	True	26	-2880.5149477559	hqer_induced sampler prototype	3.0
	WDI_agriculture	HQER-SFA	hqer_induced	True	8	-2859.055081834674	hqer_induced sampler prototype	4.0
	WDI_agriculture	Quantile-SFA	hqer_induced	True	21	-2870.9253876003186	hqer_induced sampler prototype	5.0
	WDI_agriculture	HQER-SFA	hqer_induced	True	8	-2835.875225428174	hqer_induced sampler prototype	2.0
	WDI_agriculture	Quantile-SFA	hqer_induced	True	21	-2893.9781835292097	hqer_induced sampler prototype	5.0
	WDI_agriculture	HQER-SFA	hqer_induced	True	9	-2861.3060483198246	hqer_induced sampler prototype	4.0
	WDI_agriculture	Quantile-SFA	hqer_induced	True	21	-2943.4979499527626	hqer_induced sampler prototype	5.0
	WDI_agriculture	HQER-SFA	hqer_induced	True	15	-2912.983653798104	hqer_induced sampler prototype	2.0
	WDI_agriculture	Quantile-SFA	hqer_induced	True	29	-2891.451960818865	hqer_induced sampler prototype	1.0
	WDI_agriculture	HQER-SFA	hqer_induced	True	14	-2865.31315220663	hqer_induced sampler prototype	4.0
	WDI_agriculture	Quantile-SFA	hqer_induced	True	24	-2879.9951709530405	hqer_induced sampler prototype	1.0
	WDI_agriculture	HQER-SFA	hqer_induced	True	7	-2851.4034919484475	hqer_induced sampler prototype	2.0
	WDI_agriculture	Quantile-SFA	hqer_induced	True	23	-2880.2330034222505	hqer_induced sampler prototype	3.0
	WDI_agriculture	HQER-SFA	hqer_induced	True	7	-2854.7990417636565	hqer_induced sampler prototype	2.0
	WDI_agriculture	Quantile-SFA	hqer_induced	True	21	-2882.627275583064	hqer_induced sampler prototype	5.0
	WDI_agriculture	HQER-SFA	hqer_induced	True	15	-2856.30597221498	hqer_induced sampler prototype	4.0
	WDI_agriculture	Quantile-SFA	hqer_induced	True	24	-2887.011832437732	hqer_induced sampler prototype	5.0
	WDI_agriculture	HQER-SFA	hqer_induced	True	6	-2858.095787722979	hqer_induced sampler prototype	2.0
	WDI_agriculture	Quantile-SFA	hqer_induced	True	19	-2817.3463243079923	hqer_induced sampler prototype	1.0
	WDI_agriculture	HQER-SFA	hqer_induced	True	5	-2788.0605232404423	hqer_induced sampler prototype	4.0
	WDI_agriculture	Quantile-SFA	hqer_induced	True	19	-2900.140104215658	hqer_induced sampler prototype	5.0
	WDI_agriculture	HQER-SFA	hqer_induced	True	18	-2870.6723088337794	hqer_induced sampler prototype	4.0
	WDI_agriculture	Quantile-SFA	hqer_induced	True	26	-2942.165639654921	hqer_induced sampler prototype	1.0
	WDI_agriculture	HQER-SFA	hqer_induced	True	8	-2914.8534319192854	hqer_induced sampler prototype	4.0
	WDI_agriculture	Quantile-SFA	hqer_induced	True	35	-2918.135214944319	hqer_induced sampler prototype	1.0
	WDI_agriculture	HQER-SFA	hqer_induced	True	8	-2889.1998168562686	hqer_induced sampler prototype	2.0
	WDI_agriculture	Quantile-SFA	hqer_induced	True	20	-2935.8261519612324	hqer_induced sampler prototype	5.0
	WDI_agriculture	HQER-SFA	hqer_induced	True	22	-2903.9313915469834	hqer_induced sampler prototype	2.0
	WDI_agriculture	Quantile-SFA	hqer_induced	True	32	-2850.1386809045907	hqer_induced sampler prototype	3.0
	WDI_agriculture	HQER-SFA	hqer_induced	True	13	-2835.9720855933983	hqer_induced sampler prototype	4.0
	WDI_agriculture	Quantile-SFA	hqer_induced	True	32	-2931.4007778086525	hqer_induced sampler prototype	1.0
	WDI_agriculture	HQER-SFA	hqer_induced	True	8	-2898.3388412542727	hqer_induced sampler prototype	2.0
	WDI_agriculture	Quantile-SFA	hqer_induced	True	21	-2890.0202057024794	hqer_induced sampler prototype	3.0
	WDI_agriculture	HQER-SFA	hqer_induced	True	8	-2859.820097630519	hqer_induced sampler prototype	4.0
	WDI_agriculture	Quantile-SFA	hqer_induced	True	20	-2847.509128390927	hqer_induced sampler prototype	5.0
	WDI_agriculture	HQER-SFA	hqer_induced	True	11	-2820.877268252854	hqer_induced sampler prototype	4.0
	WDI_agriculture	Quantile-SFA	hqer_induced	True	16	-2865.817219816321	hqer_induced sampler prototype	5.0
	WDI_agriculture	HQER-SFA	hqer_induced	True	9	-2842.2897291322306	hqer_induced sampler prototype	2.0
	WDI_agriculture	Quantile-SFA	hqer_induced	True	29	-2871.7818489703004	hqer_induced sampler prototype	5.0
	WDI_agriculture	HQER-SFA	hqer_induced	True	12	-2845.247272904705	hqer_induced sampler prototype	4.0
	WDI_agriculture	Quantile-SFA	hqer_induced	True	23	-2871.1283658710363	hqer_induced sampler prototype	1.0
	WDI_agriculture	HQER-SFA	hqer_induced	True	4	-2840.226145726004	hqer_induced sampler prototype	2.0
	WDI_agriculture	Quantile-SFA	hqer_induced	True	30	-2857.39594228549	hqer_induced sampler prototype	3.0
	WDI_agriculture	HQER-SFA	hqer_induced	True	16	-2833.7062587923447	hqer_induced sampler prototype	4.0
	WDI_agriculture	Quantile-SFA	hqer_induced	True	26	-2864.8515799946954	hqer_induced sampler prototype	3.0
	WDI_agriculture	HQER-SFA	hqer_induced	True	12	-2844.490260654268	hqer_induced sampler prototype	2.0
	WDI_agriculture	Quantile-SFA	hqer_induced	True	21	-2930.183770132923	hqer_induced sampler prototype	5.0
	WDI_agriculture	HQER-SFA	hqer_induced	True	20	-2897.8031206023534	hqer_induced sampler prototype	2.0
	WDI_agriculture	Quantile-SFA	hqer_induced	True	28	-2803.9776157298074	hqer_induced sampler prototype	5.0
	WDI_agriculture	HQER-SFA	hqer_induced	True	10	-2775.608996629582	hqer_induced sampler prototype	4.0
	WDI_agriculture	Quantile-SFA	hqer_induced	True	19	-2912.117840316522	hqer_induced sampler prototype	5.0
	WDI_agriculture	HQER-SFA	hqer_induced	True	7	-2877.1718353955184	hqer_induced sampler prototype	2.0
	WDI_agriculture	Quantile-SFA	hqer_induced	True	30	-2888.890979255369	hqer_induced sampler prototype	1.0
	WDI_agriculture	HQER-SFA	hqer_induced	True	8	-2852.60356989277	hqer_induced sampler prototype	2.0
	WDI_agriculture	Quantile-SFA	hqer_induced	True	25	-2926.991479821218	hqer_induced sampler prototype	1.0
	WDI_agriculture	HQER-SFA	hqer_induced	True	5	-2901.839382748868	hqer_induced sampler prototype	2.0
	WDI_agriculture	Quantile-SFA	hqer_induced	True	23	-2905.0098657333274	hqer_induced sampler prototype	3.0
	WDI_agriculture	HQER-SFA	hqer_induced	True	8	-2872.4093269111686	hqer_induced sampler prototype	2.0
	WDI_agriculture	Quantile-SFA	hqer_induced	True	24	-2852.953701383884	hqer_induced sampler prototype	3.0
	WDI_agriculture	HQER-SFA	hqer_induced	True	14	-2823.5668990012227	hqer_induced sampler prototype	4.0
	WDI_agriculture	Quantile-SFA	hqer_induced	True	24	-2905.2021315105667	hqer_induced sampler prototype	3.0
	WDI_agriculture	HQER-SFA	hqer_induced	True	10	-2873.4552571519744	hqer_induced sampler prototype	4.0
	WDI_agriculture	Quantile-SFA	hqer_induced	True	42	-2875.428938654519	hqer_induced sampler prototype	3.0
	WDI_agriculture	HQER-SFA	hqer_induced	True	17	-2854.6316863239485	hqer_induced sampler prototype	2.0
	WDI_agriculture	Quantile-SFA	hqer_induced	True	22	-2899.7910154980545	hqer_induced sampler prototype	5.0
	WDI_agriculture	HQER-SFA	hqer_induced	True	8	-2866.083796023824	hqer_induced sampler prototype	2.0
	WDI_agriculture	Quantile-SFA	hqer_induced	True	20	-2923.414430774723	hqer_induced sampler prototype	1.0
	WDI_agriculture	HQER-SFA	hqer_induced	True	15	-2884.74064276627	hqer_induced sampler prototype	4.0
	WDI_agriculture	Quantile-SFA	hqer_induced	True	22	-2900.0429647632486	hqer_induced sampler prototype	5.0
	WDI_agriculture	HQER-SFA	hqer_induced	True	14	-2874.502647272295	hqer_induced sampler prototype	4.0
	WDI_agriculture	Quantile-SFA	hqer_induced	True	26	-2903.989605574849	hqer_induced sampler prototype	5.0
	WDI_agriculture	HQER-SFA	hqer_induced	True	18	-2868.9930585213096	hqer_induced sampler prototype	4.0
	WDI_agriculture	Quantile-SFA	hqer_induced	True	18	-2952.773364976213	hqer_induced sampler prototype	5.0
	WDI_agriculture	HQER-SFA	hqer_induced	True	7	-2921.8538108315715	hqer_induced sampler prototype	2.0

experiment	dataset	model	scenario	converged	n_iter	loglik	warning_message	best_start_id
	WDI_agriculture	Quantile-SFA	hqer_induced	True	22	-2885.6973241147216	hqer_induced sampler prototype	3.0
	WDI_agriculture	HQER-SFA	hqer_induced	True	8	-2842.481152698492	hqer_induced sampler prototype	4.0
	WDI_agriculture	Quantile-SFA	hqer_induced	True	29	-2835.1956321950474	hqer_induced sampler prototype	3.0
	WDI_agriculture	HQER-SFA	hqer_induced	True	11	-2823.248483645313	hqer_induced sampler prototype	2.0
	WDI_agriculture	Quantile-SFA	hqer_induced	True	27	-2829.483488318393	hqer_induced sampler prototype	1.0
	WDI_agriculture	HQER-SFA	hqer_induced	True	7	-2803.32149702913	hqer_induced sampler prototype	4.0
	WDI_agriculture	Quantile-SFA	hqer_induced	True	33	-2871.833923695226	hqer_induced sampler prototype	1.0
	WDI_agriculture	HQER-SFA	hqer_induced	True	11	-2839.225862900733	hqer_induced sampler prototype	4.0
	WDI_agriculture	Quantile-SFA	hqer_induced	True	27	-2809.387208328378	hqer_induced sampler prototype	5.0
	WDI_agriculture	HQER-SFA	hqer_induced	True	8	-2784.3811918981883	hqer_induced sampler prototype	2.0
	WDI_agriculture	Quantile-SFA	hqer_induced	True	23	-2895.837525903038	hqer_induced sampler prototype	3.0
	WDI_agriculture	HQER-SFA	hqer_induced	True	12	-2874.1275164043373	hqer_induced sampler prototype	4.0
	WDI_agriculture	Quantile-SFA	hqer_induced	True	23	-2934.999474286603	hqer_induced sampler prototype	3.0
	WDI_agriculture	HQER-SFA	hqer_induced	True	8	-2894.513741693482	hqer_induced sampler prototype	4.0
	WDI_agriculture	Quantile-SFA	hqer_induced	True	19	-2883.676028708128	hqer_induced sampler prototype	5.0
	WDI_agriculture	HQER-SFA	hqer_induced	True	11	-2847.140684440593	hqer_induced sampler prototype	4.0
	WDI_agriculture	Quantile-SFA	hqer_induced	True	19	-2873.4663249592204	hqer_induced sampler prototype	1.0
	WDI_agriculture	HQER-SFA	hqer_induced	True	19	-2842.713938266233	hqer_induced sampler prototype	4.0
	WDI_agriculture	Quantile-SFA	hqer_induced	True	22	-2860.4771554300137	hqer_induced sampler prototype	3.0
	WDI_agriculture	HQER-SFA	hqer_induced	True	5	-2827.3583409277	hqer_induced sampler prototype	2.0
	WDI_agriculture	Quantile-SFA	hqer_induced	True	18	-2844.6033158929085	hqer_induced sampler prototype	5.0
	WDI_agriculture	HQER-SFA	hqer_induced	True	14	-2819.450813191721	hqer_induced sampler prototype	2.0
	WDI_agriculture	Quantile-SFA	hqer_induced	True	20	-2925.892635502725	hqer_induced sampler prototype	5.0
	WDI_agriculture	HQER-SFA	hqer_induced	True	4	-2891.785801135599	hqer_induced sampler prototype	2.0
	WDI_agriculture	Quantile-SFA	hqer_induced	True	25	-2895.3519977014384	hqer_induced sampler prototype	5.0
	WDI_agriculture	HQER-SFA	hqer_induced	True	15	-2863.8232787862507	hqer_induced sampler prototype	4.0
	WDI_agriculture	Quantile-SFA	hqer_induced	True	27	-2891.7437033509723	hqer_induced sampler prototype	3.0
	WDI_agriculture	HQER-SFA	hqer_induced	True	17	-2866.4407312808016	hqer_induced sampler prototype	2.0
	WDI_agriculture	Quantile-SFA	hqer_induced	True	19	-2861.038151014165	hqer_induced sampler prototype	3.0
	WDI_agriculture	HQER-SFA	hqer_induced	True	3	-2828.947175503986	hqer_induced sampler prototype	2.0
	WDI_agriculture	Quantile-SFA	hqer_induced	True	23	-2909.413805177121	hqer_induced sampler prototype	5.0
	WDI_agriculture	HQER-SFA	hqer_induced	True	11	-2881.042153792273	hqer_induced sampler prototype	4.0
	WDI_agriculture	Quantile-SFA	hqer_induced	True	18	-2851.05985326207	hqer_induced sampler prototype	5.0
	WDI_agriculture	HQER-SFA	hqer_induced	True	10	-2827.327621452455	hqer_induced sampler prototype	4.0
	WDI_agriculture	Quantile-SFA	hqer_induced	True	21	-2915.1654278191104	hqer_induced sampler prototype	3.0
	WDI_agriculture	HQER-SFA	hqer_induced	True	10	-2876.7797406179243	hqer_induced sampler prototype	4.0
	WDI_agriculture	Quantile-SFA	hqer_induced	True	25	-2938.9086557797154	hqer_induced sampler prototype	5.0
	WDI_agriculture	HQER-SFA	hqer_induced	True	8	-2907.5736335243964	hqer_induced sampler prototype	4.0
	WDI_agriculture	Quantile-SFA	hqer_induced	True	27	-2917.461962439424	hqer_induced sampler prototype	3.0
	WDI_agriculture	HQER-SFA	hqer_induced	True	11	-2891.735589575585	hqer_induced sampler prototype	2.0
	WDI_agriculture	Quantile-SFA	hqer_induced	True	25	-2899.300349729042	hqer_induced sampler prototype	3.0
	WDI_agriculture	HQER-SFA	hqer_induced	True	13	-2870.5090333067374	hqer_induced sampler prototype	4.0
	WDI_agriculture	Quantile-SFA	hqer_induced	True	24	-2883.9528535136888	hqer_induced sampler prototype	1.0
	WDI_agriculture	HQER-SFA	hqer_induced	True	7	-2856.8515872074936	hqer_induced sampler prototype	2.0
	WDI_agriculture	Quantile-SFA	hqer_induced	True	27	-2866.2084529591466	hqer_induced sampler prototype	5.0
	WDI_agriculture	HQER-SFA	hqer_induced	True	9	-2832.837914628556	hqer_induced sampler prototype	2.0
	WDI_agriculture	Quantile-SFA	hqer_induced	True	24	-2855.043197532148	hqer_induced sampler prototype	3.0
	WDI_agriculture	HQER-SFA	hqer_induced	True	12	-2825.3088127598203	hqer_induced sampler prototype	2.0
	WDI_agriculture	Quantile-SFA	hqer_induced	True	23	-2966.99130552022	hqer_induced sampler prototype	3.0
	WDI_agriculture	HQER-SFA	hqer_induced	True	22	-2923.142139917675	hqer_induced sampler prototype	4.0
	WDI_agriculture	Quantile-SFA	hqer_induced	True	25	-2941.267798962489	hqer_induced sampler prototype	5.0
	WDI_agriculture	HQER-SFA	hqer_induced	True	12	-2903.818717133445	hqer_induced sampler prototype	2.0
	WDI_agriculture	Quantile-SFA	hqer_induced	True	17	-2898.1983285249744	hqer_induced sampler prototype	5.0
	WDI_agriculture	HQER-SFA	hqer_induced	True	7	-2866.9782817966243	hqer_induced sampler prototype	4.0
	WDI_agriculture	Quantile-SFA	hqer_induced	True	18	-2889.107768298201	hqer_induced sampler prototype	5.0
	WDI_agriculture	HQER-SFA	hqer_induced	True	14	-2862.473104636212	hqer_induced sampler prototype	2.0
	WDI_agriculture	Quantile-SFA	hqer_induced	True	37	-2893.3333891793427	hqer_induced sampler prototype	3.0
	WDI_agriculture	HQER-SFA	hqer_induced	True	13	-2863.6077138761	hqer_induced sampler prototype	2.0
	WDI_agriculture	Quantile-SFA	hqer_induced	True	11	-2851.239099168089	hqer_induced sampler prototype	5.0
	WDI_agriculture	HQER-SFA	hqer_induced	True	4	-2828.862404247381	hqer_induced sampler prototype	2.0
	WDI_agriculture	Quantile-SFA	hqer_induced	True	15	-2862.4819727533654	hqer_induced sampler prototype	5.0
	WDI_agriculture	HQER-SFA	hqer_induced	True	17	-2839.1111799766977	hqer_induced sampler prototype	2.0
	WDI_agriculture	Quantile-SFA	hqer_induced	True	25	-2876.6422453812816	hqer_induced sampler prototype	1.0
	WDI_agriculture	HQER-SFA	hqer_induced	True	10	-2842.0978131201487	hqer_induced sampler prototype	4.0
	WDI_agriculture	Quantile-SFA	hqer_induced	True	23	-2900.706146125194	hqer_induced sampler prototype	5.0
	WDI_agriculture	HQER-SFA	hqer_induced	True	9	-2875.0476918144404	hqer_induced sampler prototype	4.0
	WDI_agriculture	Quantile-SFA	hqer_induced	True	17	-2831.4440407702805	hqer_induced sampler prototype	5.0
	WDI_agriculture	HQER-SFA	hqer_induced	True	10	-2801.843700327001	hqer_induced sampler prototype	4.0
	WDI_agriculture	Quantile-SFA	hqer_induced	True	47	-2906.014625581527	hqer_induced sampler prototype	3.0
	WDI_agriculture	HQER-SFA	hqer_induced	True	12	-2876.6023263306743	hqer_induced sampler prototype	2.0
	WDI_agriculture	Quantile-SFA	hqer_induced	True	19	-2827.858422575164	hqer_induced sampler prototype	1.0
	WDI_agriculture	HQER-SFA	hqer_induced	True	7	-2806.7685952121665	hqer_induced sampler prototype	2.0
	WDI_agriculture	Quantile-SFA	hqer_induced	True	26	-2836.9260521402493	hqer_induced sampler prototype	1.0
	WDI_agriculture	HQER-SFA	hqer_induced	True	10	-2806.4662942012897	hqer_induced sampler prototype	4.0
	WDI_agriculture	Quantile-SFA	hqer_induced	True	22	-2915.6616467703034	hqer_induced sampler prototype	5.0
	WDI_agriculture	HQER-SFA	hqer_induced	True	18	-2886.690974400144	hqer_induced sampler prototype	2.0
	WDI_agriculture	Quantile-SFA	hqer_induced	True	29	-2936.743820252294	hqer_induced sampler prototype	3.0
	WDI_agriculture	HQER-SFA	hqer_induced	True	8	-2918.180446515174	hqer_induced sampler prototype	4.0
	WDI_agriculture	Quantile-SFA	hqer_induced	True	23	-2895.152376581256	hqer_induced sampler prototype	3.0
	WDI_agriculture	HQER-SFA	hqer_induced	True	21	-2858.7458138093834	hqer_induced sampler prototype	4.0
	WDI_agriculture	Quantile-SFA	hqer_induced	True	23	-2840.017385007834	hqer_induced sampler prototype	1.0
	WDI_agriculture	HQER-SFA	hqer_induced	True	12	-2810.6606662339627	hqer_induced sampler prototype	4.0
	WDI_agriculture	Quantile-SFA	hqer_induced	True	29	-2837.7934608397018	hqer_induced sampler prototype	5.0
	WDI_agriculture	HQER-SFA	hqer_induced	True	8	-2803.5527786763505	hqer_induced sampler prototype	2.0
	WDI_agriculture	Quantile-SFA	hqer_induced	True	18	-2897.226687132973	hqer_induced sampler prototype	5.0
	WDI_agriculture	HQER-SFA	hqer_induced	True	4	-2863.5903089526128	hqer_induced sampler prototype	2.0
	WDI_agriculture	Quantile-SFA	hqer_induced	True	23	-2823.58871406379	hqer_induced sampler prototype	3.0
	WDI_agriculture	HQER-SFA	hqer_induced	True	9	-2796.3221856077407	hqer_induced sampler prototype	4.0
	WDI_agriculture	Quantile-SFA	hqer_induced	True	19	-2898.3641596172006	hqer_induced sampler prototype	5.0
	WDI_agriculture	HQER-SFA	hqer_induced	True	10	-2854.0889381973275	hqer_induced sampler prototype	4.0
	WDI_agriculture	Quantile-SFA	hqer_induced	True	34	-2900.47108196323	hqer_induced sampler prototype	3.0
	WDI_agriculture	HQER-SFA	hqer_induced	True	6	-2864.669796721817	hqer_induced sampler prototype	2.0
	WDI_agriculture	Quantile-SFA	hqer_induced	True	25	-2838.2655730016027	hqer_induced sampler prototype	1.0
	WDI_agriculture	HQER-SFA	hqer_induced	True	6	-2809.197850953475	hqer_induced sampler prototype	4.0
	WDI_agriculture	Quantile-SFA	hqer_induced	True	30	-2891.751391593756	hqer_induced sampler prototype	1.0
	WDI_agriculture	HQER-SFA	hqer_induced	True	8	-2869.812467198342	hqer_induced sampler prototype	2.0
	WDI_agriculture	Quantile-SFA	hqer_induced	True	26	-2892.344825734002	hqer_induced sampler prototype	3.0
	WDI_agriculture	HQER-SFA	hqer_induced	True	8	-2861.5018273156243	hqer_induced sampler prototype	4.0
	WDI_agriculture	Quantile-SFA	hqer_induced	True	23	-2833.888731950974	hqer_induced sampler prototype	5.0
	WDI_agriculture	HQER-SFA	hqer_induced	True	7	-2807.613036889634	hqer_induced sampler prototype	2.0
	WDI_agriculture	Quantile-SFA	hqer_induced	True	23	-2862.2602335928577	hqer_induced sampler prototype	1.0
	WDI_agriculture	HQER-SFA	hqer_induced	True	8	-2834.5594492940068	hqer_induced sampler prototype	2.0
	WDI_agriculture	Quantile-SFA	hqer_induced	True	26	-2948.9008838303653	hqer_induced sampler prototype	5.0

experiment	dataset	model	scenario	converged	n_iter	loglik	warning_message	best_start_id
	WDI_agriculture	HQER-SFA	hqer_induced	True	9	-2915.791867177204	hqer_induced sampler prototype	2.0
	WDI_agriculture	Quantile-SFA	hqer_induced	True	35	-2904.409014489177	hqer_induced sampler prototype	3.0
	WDI_agriculture	HQER-SFA	hqer_induced	True	12	-2879.146047216376	hqer_induced sampler prototype	4.0
	WDI_agriculture	Quantile-SFA	hqer_induced	True	26	-2898.710585365025	hqer_induced sampler prototype	1.0
	WDI_agriculture	HQER-SFA	hqer_induced	True	10	-2877.9145546350865	hqer_induced sampler prototype	4.0
	WDI_agriculture	Quantile-SFA	hqer_induced	True	17	-2858.3464207088746	hqer_induced sampler prototype	5.0
	WDI_agriculture	HQER-SFA	hqer_induced	True	7	-2823.8280687659435	hqer_induced sampler prototype	4.0
	WDI_agriculture	Quantile-SFA	hqer_induced	True	20	-2878.0045298052787	hqer_induced sampler prototype	3.0
	WDI_agriculture	HQER-SFA	hqer_induced	True	7	-2847.984028606468	hqer_induced sampler prototype	2.0
	WDI_agriculture	Quantile-SFA	hqer_induced	True	19	-2888.7177948291965	hqer_induced sampler prototype	3.0
	WDI_agriculture	HQER-SFA	hqer_induced	True	3	-2852.8010360669978	hqer_induced sampler prototype	2.0
	WDI_agriculture	Quantile-SFA	hqer_induced	True	39	-2846.362375658099	hqer_induced sampler prototype	1.0
	WDI_agriculture	HQER-SFA	hqer_induced	True	3	-2814.4330072149296	hqer_induced sampler prototype	2.0
	WDI_agriculture	Quantile-SFA	hqer_induced	True	24	-2925.7415722816586	hqer_induced sampler prototype	3.0
	WDI_agriculture	HQER-SFA	hqer_induced	True	10	-2899.8396794505825	hqer_induced sampler prototype	4.0
	WDI_agriculture	Quantile-SFA	hqer_induced	True	22	-2877.482127608211	hqer_induced sampler prototype	5.0
	WDI_agriculture	HQER-SFA	hqer_induced	True	12	-2856.5772882288147	hqer_induced sampler prototype	4.0
	WDI_agriculture	Quantile-SFA	hqer_induced	True	26	-2884.122179596238	hqer_induced sampler prototype	5.0
	WDI_agriculture	HQER-SFA	hqer_induced	True	7	-2855.79291184917	hqer_induced sampler prototype	2.0
	WDI_agriculture	Quantile-SFA	hqer_induced	True	30	-2903.772235830297	hqer_induced sampler prototype	5.0
	WDI_agriculture	HQER-SFA	hqer_induced	True	7	-2872.7208207549866	hqer_induced sampler prototype	2.0
	WDI_agriculture	Quantile-SFA	hqer_induced	True	30	-2883.222750354784	hqer_induced sampler prototype	5.0
	WDI_agriculture	HQER-SFA	hqer_induced	True	8	-2858.3451678842703	hqer_induced sampler prototype	4.0
	WDI_agriculture	Quantile-SFA	hqer_induced	True	27	-2895.937835718293	hqer_induced sampler prototype	3.0
	WDI_agriculture	HQER-SFA	hqer_induced	True	15	-2862.353956978752	hqer_induced sampler prototype	2.0
	WDI_agriculture	Quantile-SFA	hqer_induced	True	30	-2924.097630383875	hqer_induced sampler prototype	5.0
	WDI_agriculture	HQER-SFA	hqer_induced	True	7	-2896.3280158602065	hqer_induced sampler prototype	2.0
	WDI_agriculture	Quantile-SFA	hqer_induced	True	28	-2845.1087492595907	hqer_induced sampler prototype	3.0
	WDI_agriculture	HQER-SFA	hqer_induced	True	12	-2815.1225703254017	hqer_induced sampler prototype	2.0
	WDI_agriculture	Quantile-SFA	hqer_induced	True	30	-2914.349622960539	hqer_induced sampler prototype	3.0
	WDI_agriculture	HQER-SFA	hqer_induced	True	11	-2884.4757027815326	hqer_induced sampler prototype	4.0
	WDI_agriculture	Quantile-SFA	hqer_induced	True	29	-2866.8268297204727	hqer_induced sampler prototype	3.0
	WDI_agriculture	HQER-SFA	hqer_induced	True	9	-2838.971759266284	hqer_induced sampler prototype	4.0
	WDI_agriculture	Quantile-SFA	hqer_induced	True	28	-2893.4079022124342	hqer_induced sampler prototype	5.0
	WDI_agriculture	HQER-SFA	hqer_induced	True	7	-2863.0789811266186	hqer_induced sampler prototype	2.0
	WDI_agriculture	Quantile-SFA	hqer_induced	True	23	-2876.9685487861943	hqer_induced sampler prototype	5.0
	WDI_agriculture	HQER-SFA	hqer_induced	True	10	-2849.466452873815	hqer_induced sampler prototype	4.0
	WDI_agriculture	Quantile-SFA	hqer_induced	True	25	-2902.653498456792	hqer_induced sampler prototype	1.0
	WDI_agriculture	HQER-SFA	hqer_induced	True	8	-2872.2250040643767	hqer_induced sampler prototype	4.0
	WDI_agriculture	Quantile-SFA	hqer_induced	True	31	-2836.9204417113256	hqer_induced sampler prototype	3.0
	WDI_agriculture	HQER-SFA	hqer_induced	True	10	-2804.5234006874243	hqer_induced sampler prototype	4.0
	WDI_agriculture	Quantile-SFA	hqer_induced	True	22	-2878.7526930606223	hqer_induced sampler prototype	5.0
	WDI_agriculture	HQER-SFA	hqer_induced	True	15	-2843.535751441307	hqer_induced sampler prototype	4.0
	WDI_agriculture	Quantile-SFA	hqer_induced	True	33	-2865.461375953318	hqer_induced sampler prototype	3.0
	WDI_agriculture	HQER-SFA	hqer_induced	True	7	-2832.073926628448	hqer_induced sampler prototype	4.0
	WDI_agriculture	Quantile-SFA	hqer_induced	True	21	-2911.694117913364	hqer_induced sampler prototype	1.0
	WDI_agriculture	HQER-SFA	hqer_induced	True	22	-2874.5519265821945	hqer_induced sampler prototype	2.0
	WDI_agriculture	Quantile-SFA	hqer_induced	True	20	-2944.8090155524537	hqer_induced sampler prototype	1.0
	WDI_agriculture	HQER-SFA	hqer_induced	True	7	-2905.7473013539334	hqer_induced sampler prototype	2.0
	WDI_agriculture	Quantile-SFA	hqer_induced	True	27	-2891.3378213482174	hqer_induced sampler prototype	1.0
	WDI_agriculture	HQER-SFA	hqer_induced	True	7	-2852.205329894669	hqer_induced sampler prototype	4.0
	WDI_agriculture	Quantile-SFA	hqer_induced	True	23	-2843.4512283439817	hqer_induced sampler prototype	1.0
	WDI_agriculture	HQER-SFA	hqer_induced	True	14	-2828.0503531533723	hqer_induced sampler prototype	2.0
	WDI_agriculture	Quantile-SFA	hqer_induced	True	25	-2908.1613870605947	hqer_induced sampler prototype	3.0
	WDI_agriculture	HQER-SFA	hqer_induced	True	7	-2874.024685775551	hqer_induced sampler prototype	2.0
	WDI_agriculture	Quantile-SFA	hqer_induced	True	25	-2911.3105938763265	hqer_induced sampler prototype	3.0
	WDI_agriculture	HQER-SFA	hqer_induced	True	9	-2883.186814264993	hqer_induced sampler prototype	2.0
	WDI_agriculture	Quantile-SFA	hqer_induced	True	23	-2888.6753109373794	hqer_induced sampler prototype	3.0
	WDI_agriculture	HQER-SFA	hqer_induced	True	5	-2861.9553327252024	hqer_induced sampler prototype	2.0
	WDI_agriculture	Quantile-SFA	hqer_induced	True	19	-2938.1345101967468	hqer_induced sampler prototype	5.0
	WDI_agriculture	HQER-SFA	hqer_induced	True	7	-2901.240583764053	hqer_induced sampler prototype	4.0
	WDI_agriculture	Quantile-SFA	hqer_induced	True	20	-2903.0410746358207	hqer_induced sampler prototype	5.0
	WDI_agriculture	HQER-SFA	hqer_induced	True	8	-2871.4892481412207	hqer_induced sampler prototype	4.0
	WDI_agriculture	Quantile-SFA	hqer_induced	True	26	-2897.7365641214938	hqer_induced sampler prototype	3.0
	WDI_agriculture	HQER-SFA	hqer_induced	True	8	-2861.8184917465587	hqer_induced sampler prototype	4.0
	WDI_agriculture	Quantile-SFA	hqer_induced	True	32	-2886.613301842036	hqer_induced sampler prototype	5.0
	WDI_agriculture	HQER-SFA	hqer_induced	True	9	-2852.875964473384	hqer_induced sampler prototype	4.0
	WDI_agriculture	Quantile-SFA	hqer_induced	True	21	-2926.019282131497	hqer_induced sampler prototype	5.0
	WDI_agriculture	HQER-SFA	hqer_induced	True	12	-2894.1667912044168	hqer_induced sampler prototype	4.0
	WDI_agriculture	Quantile-SFA	hqer_induced	True	25	-2914.4719680430826	hqer_induced sampler prototype	5.0
	WDI_agriculture	HQER-SFA	hqer_induced	True	10	-2883.4664312928007	hqer_induced sampler prototype	4.0
	WDI_agriculture	Quantile-SFA	hqer_induced	True	28	-2854.3922262042297	hqer_induced sampler prototype	3.0
	WDI_agriculture	HQER-SFA	hqer_induced	True	8	-2826.2041618834232	hqer_induced sampler prototype	2.0
	WDI_agriculture	Quantile-SFA	hqer_induced	True	19	-2936.543164385348	hqer_induced sampler prototype	3.0
	WDI_agriculture	HQER-SFA	hqer_induced	True	8	-2905.874431973949	hqer_induced sampler prototype	2.0
	WDI_agriculture	Quantile-SFA	hqer_induced	True	26	-2899.302683453762	hqer_induced sampler prototype	3.0
	WDI_agriculture	HQER-SFA	hqer_induced	True	8	-2871.4344011564663	hqer_induced sampler prototype	2.0
	WDI_agriculture	Quantile-SFA	hqer_induced	True	31	-2928.9339133049843	hqer_induced sampler prototype	5.0
	WDI_agriculture	HQER-SFA	hqer_induced	True	10	-2894.470230842018	hqer_induced sampler prototype	4.0
	WDI_agriculture	Quantile-SFA	hqer_induced	True	21	-2914.1209806842435	hqer_induced sampler prototype	3.0
	WDI_agriculture	HQER-SFA	hqer_induced	True	11	-2884.245878713068	hqer_induced sampler prototype	4.0
	WDI_agriculture	Quantile-SFA	hqer_induced	True	25	-2884.9386066414067	hqer_induced sampler prototype	5.0
	WDI_agriculture	HQER-SFA	hqer_induced	True	7	-2864.8559427596724	hqer_induced sampler prototype	2.0
	WDI_agriculture	Quantile-SFA	hqer_induced	True	29	-2872.541973575745	hqer_induced sampler prototype	1.0
	WDI_agriculture	HQER-SFA	hqer_induced	True	12	-2847.7382003955563	hqer_induced sampler prototype	2.0
	WDI_agriculture	Quantile-SFA	hqer_induced	True	32	-2910.3617282718988	hqer_induced sampler prototype	1.0
	WDI_agriculture	HQER-SFA	hqer_induced	True	8	-2886.7524343529967	hqer_induced sampler prototype	4.0
	WDI_agriculture	Quantile-SFA	hqer_induced	True	25	-2850.0350993930574	hqer_induced sampler prototype	5.0
	WDI_agriculture	HQER-SFA	hqer_induced	True	9	-2819.7023538045323	hqer_induced sampler prototype	4.0
	WDI_agriculture	Quantile-SFA	hqer_induced	True	25	-2906.5311719336787	hqer_induced sampler prototype	3.0
	WDI_agriculture	HQER-SFA	hqer_induced	True	7	-2878.6769975160614	hqer_induced sampler prototype	2.0
	WDI_agriculture	Quantile-SFA	hqer_induced	True	20	-2929.7547042410256	hqer_induced sampler prototype	1.0
	WDI_agriculture	HQER-SFA	hqer_induced	True	8	-2899.0901277339262	hqer_induced sampler prototype	2.0
	WDI_agriculture	Quantile-SFA	hqer_induced	True	32	-2902.062534037233	hqer_induced sampler prototype	1.0
	WDI_agriculture	HQER-SFA	hqer_induced	True	10	-2873.668036838951	hqer_induced sampler prototype	4.0
	WDI_agriculture	Quantile-SFA	hqer_induced	True	17	-2858.243232816219	hqer_induced sampler prototype	3.0
	WDI_agriculture	HQER-SFA	hqer_induced	True	7	-2823.9638569259987	hqer_induced sampler prototype	2.0
	WDI_agriculture	Quantile-SFA	hqer_induced	True	20	-2923.699451409849	hqer_induced sampler prototype	5.0
	WDI_agriculture	HQER-SFA	hqer_induced	True	9	-2894.572215264382	hqer_induced sampler prototype	2.0
	WDI_agriculture	Quantile-SFA	hqer_induced	True	25	-2871.305093531103	hqer_induced sampler prototype	5.0
	WDI_agriculture	HQER-SFA	hqer_induced	True	17	-2844.113219901621	hqer_induced sampler prototype	4.0
	WDI_agriculture	Quantile-SFA	hqer_induced	True	22	-2918.3283566062764	hqer_induced sampler prototype	1.0
	WDI_agriculture	HQER-SFA	hqer_induced	True	9	-2887.927041330648	hqer_induced sampler prototype	2.0

experiment	dataset	model	scenario	converged	n__iter	loglik	warning__message	best__start__id
	WDI_agriculture	Quantile-SFA	hqer__induced	True	26	-2864.9454443641785	hqer__induced sampler prototype	5.0
	WDI_agriculture	HQER-SFA	hqer__induced	True	7	-2835.0457870764067	hqer__induced sampler prototype	2.0
	WDI_agriculture	Quantile-SFA	hqer__induced	True	29	-2867.125801314316	hqer__induced sampler prototype	1.0
	WDI_agriculture	HQER-SFA	hqer__induced	True	13	-2840.393034206253	hqer__induced sampler prototype	4.0
	WDI_agriculture	Quantile-SFA	hqer__induced	True	26	-2881.9979395889054	hqer__induced sampler prototype	1.0
	WDI_agriculture	HQER-SFA	hqer__induced	True	14	-2859.150391361517	hqer__induced sampler prototype	4.0
	WDI_agriculture	Quantile-SFA	hqer__induced	True	25	-2905.273151894329	hqer__induced sampler prototype	3.0
	WDI_agriculture	HQER-SFA	hqer__induced	True	7	-2882.7345606224308	hqer__induced sampler prototype	2.0
	WDI_agriculture	Quantile-SFA	hqer__induced	True	26	-2881.5636893436044	hqer__induced sampler prototype	3.0
	WDI_agriculture	HQER-SFA	hqer__induced	True	15	-2846.917865479004	hqer__induced sampler prototype	4.0
	WDI_agriculture	Quantile-SFA	hqer__induced	True	20	-2904.75456354659	hqer__induced sampler prototype	5.0
	WDI_agriculture	HQER-SFA	hqer__induced	True	12	-2875.0800068942467	hqer__induced sampler prototype	2.0
	WDI_agriculture	Quantile-SFA	hqer__induced	True	27	-2917.453067409609	hqer__induced sampler prototype	5.0
	WDI_agriculture	HQER-SFA	hqer__induced	True	7	-2890.540141892878	hqer__induced sampler prototype	2.0
	WDI_agriculture	Quantile-SFA	hqer__induced	True	20	-2930.5510725007875	hqer__induced sampler prototype	5.0
	WDI_agriculture	HQER-SFA	hqer__induced	True	7	-2897.152815071323	hqer__induced sampler prototype	4.0
	WDI_agriculture	Quantile-SFA	hqer__induced	True	32	-2847.7617321724624	hqer__induced sampler prototype	3.0
	WDI_agriculture	HQER-SFA	hqer__induced	True	8	-2833.4933100600447	hqer__induced sampler prototype	4.0
	WDI_agriculture	Quantile-SFA	hqer__induced	True	30	-2878.961092700763	hqer__induced sampler prototype	5.0
	WDI_agriculture	HQER-SFA	hqer__induced	True	10	-2859.0414401647067	hqer__induced sampler prototype	4.0
	WDI_agriculture	Quantile-SFA	hqer__induced	True	33	-2915.766837779672	hqer__induced sampler prototype	3.0
	WDI_agriculture	HQER-SFA	hqer__induced	True	16	-2887.42136318518	hqer__induced sampler prototype	4.0
	WDI_agriculture	Quantile-SFA	hqer__induced	True	25	-2880.705416743629	hqer__induced sampler prototype	3.0
	WDI_agriculture	HQER-SFA	hqer__induced	True	11	-2858.6241766112144	hqer__induced sampler prototype	4.0
	WDI_agriculture	Quantile-SFA	hqer__induced	True	29	-2852.404470127717	hqer__induced sampler prototype	3.0
	WDI_agriculture	HQER-SFA	hqer__induced	True	9	-2816.704606038144	hqer__induced sampler prototype	4.0
	WDI_agriculture	Quantile-SFA	hqer__induced	True	16	-2926.722231734546	hqer__induced sampler prototype	5.0
	WDI_agriculture	HQER-SFA	hqer__induced	True	9	-2896.3932724704227	hqer__induced sampler prototype	2.0
	WDI_agriculture	Quantile-SFA	hqer__induced	True	21	-2913.737537916252	hqer__induced sampler prototype	5.0
	WDI_agriculture	HQER-SFA	hqer__induced	True	6	-2889.533519644023	hqer__induced sampler prototype	2.0
				True	41		negative source domain; cross-domain transfer risk	4.0
				True	41		negative source domain; cross-domain transfer risk	4.0
				True	76		negative source domain; cross-domain transfer risk	4.0
				True	20		negative source domain; cross-domain transfer risk	4.0
				True	22		negative source domain; cross-domain transfer risk	4.0
				True	11		negative source domain; cross-domain transfer risk	4.0
				True	19		negative source domain; cross-domain transfer risk	4.0
				True	19		negative source domain; cross-domain transfer risk	4.0
				True	27		negative source domain; cross-domain transfer risk	4.0
				True	24		negative source domain; cross-domain transfer risk	4.0
				True	22		negative source domain; cross-domain transfer risk	4.0
				True	9		negative source domain; cross-domain transfer risk	4.0
				True	34		negative source domain; cross-domain transfer risk	4.0
				True	34		negative source domain; cross-domain transfer risk	4.0
				True	31		negative source domain; cross-domain transfer risk	4.0
				True	18		negative source domain; cross-domain transfer risk	4.0
				True	29		negative source domain; cross-domain transfer risk	4.0
				True	10		negative source domain; cross-domain transfer risk	4.0
				True	25		negative source domain; cross-domain transfer risk	4.0
				True	25		negative source domain; cross-domain transfer risk	4.0
				True	14		negative source domain; cross-domain transfer risk	4.0
				True	15		negative source domain; cross-domain transfer risk	4.0
				True	24		negative source domain; cross-domain transfer risk	4.0
				True	9		negative source domain; cross-domain transfer risk	4.0
				True	62		negative source domain; cross-domain transfer risk	4.0
				True	62		negative source domain; cross-domain transfer risk	4.0
				True	31		negative source domain; cross-domain transfer risk	4.0
				True	11		negative source domain; cross-domain transfer risk	4.0
				True	29		negative source domain; cross-domain transfer risk	4.0
				True	14		negative source domain; cross-domain transfer risk	4.0
				True	23		negative source domain; cross-domain transfer risk	4.0
				True	23		negative source domain; cross-domain transfer risk	4.0
				True	13		negative source domain; cross-domain transfer risk	4.0
				True	22		negative source domain; cross-domain transfer risk	4.0
				True	29		negative source domain; cross-domain transfer risk	4.0
				True	10		negative source domain; cross-domain transfer risk	4.0
				True	9		negative source domain; cross-domain transfer risk	4.0
				True	9		negative source domain; cross-domain transfer risk	4.0
				True	12		negative source domain; cross-domain transfer risk	4.0
				True	18		negative source domain; cross-domain transfer risk	4.0
				True	25		negative source domain; cross-domain transfer risk	4.0
				True	11		negative source domain; cross-domain transfer risk	4.0
				True	49		negative source domain; cross-domain transfer risk	4.0
				True	49		negative source domain; cross-domain transfer risk	4.0
				True	15		negative source domain; cross-domain transfer risk	4.0
				True	38		negative source domain; cross-domain transfer risk	4.0
				True	32		negative source domain; cross-domain transfer risk	4.0
				True	15		negative source domain; cross-domain transfer risk	4.0
				True	29		negative source domain; cross-domain transfer risk	4.0
				True	29		negative source domain; cross-domain transfer risk	4.0
				True	20		negative source domain; cross-domain transfer risk	4.0
				True	30		negative source domain; cross-domain transfer risk	4.0
				True	30		negative source domain; cross-domain transfer risk	4.0
				True	11		negative source domain; cross-domain transfer risk	4.0
				True	52		negative source domain; cross-domain transfer risk	4.0
				True	52		negative source domain; cross-domain transfer risk	4.0
				True	30		negative source domain; cross-domain transfer risk	4.0
				True	28		negative source domain; cross-domain transfer risk	4.0
				True	23		negative source domain; cross-domain transfer risk	4.0
				True	13		negative source domain; cross-domain transfer risk	4.0
				True	14		negative source domain; cross-domain transfer risk	4.0
				True	14		negative source domain; cross-domain transfer risk	4.0
				True	17		negative source domain; cross-domain transfer risk	4.0
				True	22		negative source domain; cross-domain transfer risk	4.0
				True	20		negative source domain; cross-domain transfer risk	4.0
				True	10		negative source domain; cross-domain transfer risk	4.0
				True	44		negative source domain; cross-domain transfer risk	4.0
				True	44		negative source domain; cross-domain transfer risk	4.0
				True	17		negative source domain; cross-domain transfer risk	4.0
				True	25		negative source domain; cross-domain transfer risk	4.0
				True	30		negative source domain; cross-domain transfer risk	4.0
				True	9		negative source domain; cross-domain transfer risk	4.0
				True	55		negative source domain; cross-domain transfer risk	4.0

experiment	dataset	model	scenario	converged	n_iter	loglik	warning_message	best_start_id
				True	55		negative source domain; cross-domain transfer risk	4.0
				True	18		negative source domain; cross-domain transfer risk	4.0
				True	21		negative source domain; cross-domain transfer risk	4.0
				True	30		negative source domain; cross-domain transfer risk	4.0
				True	11		negative source domain; cross-domain transfer risk	4.0
				True	63		negative source domain; cross-domain transfer risk	4.0
				True	63		negative source domain; cross-domain transfer risk	4.0
				True	52		negative source domain; cross-domain transfer risk	4.0
				True	14		negative source domain; cross-domain transfer risk	4.0
				True	20		negative source domain; cross-domain transfer risk	4.0
				True	9		negative source domain; cross-domain transfer risk	4.0
				True	35		negative source domain; cross-domain transfer risk	4.0
				True	35		negative source domain; cross-domain transfer risk	4.0
				True	10		negative source domain; cross-domain transfer risk	4.0
				True	36		negative source domain; cross-domain transfer risk	4.0
				True	28		negative source domain; cross-domain transfer risk	4.0
				True	10		negative source domain; cross-domain transfer risk	4.0
				True	11		negative source domain; cross-domain transfer risk	4.0
				True	11		negative source domain; cross-domain transfer risk	4.0
				True	11		negative source domain; cross-domain transfer risk	4.0
				True	15		negative source domain; cross-domain transfer risk	4.0
				True	33		negative source domain; cross-domain transfer risk	4.0
				True	9		negative source domain; cross-domain transfer risk	4.0
				True	23		negative source domain; cross-domain transfer risk	4.0
				True	23		negative source domain; cross-domain transfer risk	4.0
				True	22		negative source domain; cross-domain transfer risk	4.0
				True	34		negative source domain; cross-domain transfer risk	4.0
				True	21		negative source domain; cross-domain transfer risk	4.0
				True	11		negative source domain; cross-domain transfer risk	4.0
				True	22		negative source domain; cross-domain transfer risk	4.0
				True	22		negative source domain; cross-domain transfer risk	4.0
				True	12		negative source domain; cross-domain transfer risk	4.0
				True	21		negative source domain; cross-domain transfer risk	4.0
				True	26		negative source domain; cross-domain transfer risk	4.0
				True	10		negative source domain; cross-domain transfer risk	4.0
				True	44		negative source domain; cross-domain transfer risk	4.0
				True	44		negative source domain; cross-domain transfer risk	4.0
				True	17		negative source domain; cross-domain transfer risk	4.0
				True	25		negative source domain; cross-domain transfer risk	4.0
				True	39		negative source domain; cross-domain transfer risk	4.0
				True	14		negative source domain; cross-domain transfer risk	4.0
				True	17		negative source domain; cross-domain transfer risk	4.0
				True	17		negative source domain; cross-domain transfer risk	4.0
				True	14		negative source domain; cross-domain transfer risk	4.0
				True	13		negative source domain; cross-domain transfer risk	4.0
				True	29		negative source domain; cross-domain transfer risk	4.0
				True	10		negative source domain; cross-domain transfer risk	4.0
				True	19		negative source domain; cross-domain transfer risk	4.0
				True	19		negative source domain; cross-domain transfer risk	4.0
				True	17		negative source domain; cross-domain transfer risk	4.0
				True	27		negative source domain; cross-domain transfer risk	4.0
				True	28		negative source domain; cross-domain transfer risk	4.0
				True	12		negative source domain; cross-domain transfer risk	4.0
				True	26		negative source domain; cross-domain transfer risk	4.0
				True	26		negative source domain; cross-domain transfer risk	4.0
				True	20		negative source domain; cross-domain transfer risk	4.0
				True	24		negative source domain; cross-domain transfer risk	4.0
				True	31		negative source domain; cross-domain transfer risk	4.0
				True	11		negative source domain; cross-domain transfer risk	4.0
				True	29		negative source domain; cross-domain transfer risk	4.0
				True	29		negative source domain; cross-domain transfer risk	4.0
				True	22		negative source domain; cross-domain transfer risk	4.0
				True	22		negative source domain; cross-domain transfer risk	4.0
				True	26		negative source domain; cross-domain transfer risk	4.0
				True	11		negative source domain; cross-domain transfer risk	4.0
				True	15		negative source domain; cross-domain transfer risk	4.0
				True	15		negative source domain; cross-domain transfer risk	4.0
				True	39		negative source domain; cross-domain transfer risk	4.0
				True	19		negative source domain; cross-domain transfer risk	4.0
				True	38		negative source domain; cross-domain transfer risk	4.0
				True	14		negative source domain; cross-domain transfer risk	3.0
				True	36		negative source domain; cross-domain transfer risk	4.0
				True	36		negative source domain; cross-domain transfer risk	4.0
				True	50		negative source domain; cross-domain transfer risk	4.0
				True	25		negative source domain; cross-domain transfer risk	4.0
				True	34		negative source domain; cross-domain transfer risk	4.0
				True	15		negative source domain; cross-domain transfer risk	4.0
				True	44		negative source domain; cross-domain transfer risk	4.0
				True	44		negative source domain; cross-domain transfer risk	4.0
				True	12		negative source domain; cross-domain transfer risk	4.0
				True	48		negative source domain; cross-domain transfer risk	4.0
				True	33		negative source domain; cross-domain transfer risk	4.0
				True	15		negative source domain; cross-domain transfer risk	4.0
				True	33		negative source domain; cross-domain transfer risk	4.0
				True	33		negative source domain; cross-domain transfer risk	4.0
				True	57		negative source domain; cross-domain transfer risk	4.0
				True	23		negative source domain; cross-domain transfer risk	4.0
				True	37		negative source domain; cross-domain transfer risk	4.0
				True	10		negative source domain; cross-domain transfer risk	4.0
				True	48		negative source domain; cross-domain transfer risk	4.0
				True	48		negative source domain; cross-domain transfer risk	4.0
				True	38		negative source domain; cross-domain transfer risk	4.0
				True	13		negative source domain; cross-domain transfer risk	4.0
				True	32		negative source domain; cross-domain transfer risk	4.0
				True	10		negative source domain; cross-domain transfer risk	4.0
				True	21		negative source domain; cross-domain transfer risk	4.0
				True	21		negative source domain; cross-domain transfer risk	4.0
				True	12		negative source domain; cross-domain transfer risk	4.0
				True	12		negative source domain; cross-domain transfer risk	4.0
				True	26		negative source domain; cross-domain transfer risk	4.0
				True	11		negative source domain; cross-domain transfer risk	4.0
				True	22		negative source domain; cross-domain transfer risk	4.0
				True	22		negative source domain; cross-domain transfer risk	4.0

experiment	dataset	model	scenario	converged	n_iter	loglik	warning_message	best_start_id
				True	27		negative source domain; cross-domain transfer risk	4.0
				True	12		negative source domain; cross-domain transfer risk	4.0
				True	29		negative source domain; cross-domain transfer risk	4.0
				True	10		negative source domain; cross-domain transfer risk	4.0
				True	12		negative source domain; cross-domain transfer risk	4.0
				True	12		negative source domain; cross-domain transfer risk	4.0
				True	14		negative source domain; cross-domain transfer risk	4.0
				True	19		negative source domain; cross-domain transfer risk	4.0
				True	22		negative source domain; cross-domain transfer risk	4.0
				True	11		negative source domain; cross-domain transfer risk	4.0
				True	23		negative source domain; cross-domain transfer risk	4.0
				True	23		negative source domain; cross-domain transfer risk	4.0
				True	19		negative source domain; cross-domain transfer risk	4.0
				True	43		negative source domain; cross-domain transfer risk	4.0
				True	28		negative source domain; cross-domain transfer risk	4.0
				True	11		negative source domain; cross-domain transfer risk	4.0
				True	28		negative source domain; cross-domain transfer risk	4.0
				True	28		negative source domain; cross-domain transfer risk	4.0
				True	21		negative source domain; cross-domain transfer risk	4.0
				True	24		negative source domain; cross-domain transfer risk	4.0
				True	25		negative source domain; cross-domain transfer risk	4.0
				True	11		negative source domain; cross-domain transfer risk	4.0
				True	49		negative source domain; cross-domain transfer risk	4.0
				True	49		negative source domain; cross-domain transfer risk	4.0
				True	52		negative source domain; cross-domain transfer risk	4.0
				True	33		negative source domain; cross-domain transfer risk	4.0
				True	30		negative source domain; cross-domain transfer risk	4.0
				True	10		negative source domain; cross-domain transfer risk	4.0
				True	22		negative source domain; cross-domain transfer risk	4.0
				True	22		negative source domain; cross-domain transfer risk	4.0
				True	10		negative source domain; cross-domain transfer risk	4.0
				True	11		negative source domain; cross-domain transfer risk	4.0
				True	25		negative source domain; cross-domain transfer risk	4.0
				True	14		negative source domain; cross-domain transfer risk	1.0
				True	28		negative source domain; cross-domain transfer risk	4.0
				True	28		negative source domain; cross-domain transfer risk	4.0
				True	16		negative source domain; cross-domain transfer risk	4.0
				True	41		negative source domain; cross-domain transfer risk	4.0
				True	31		negative source domain; cross-domain transfer risk	4.0
				True	11		negative source domain; cross-domain transfer risk	4.0
				True	25		negative source domain; cross-domain transfer risk	4.0
				True	25		negative source domain; cross-domain transfer risk	4.0
				True	47		negative source domain; cross-domain transfer risk	4.0
				True	30		negative source domain; cross-domain transfer risk	4.0
				True	28		negative source domain; cross-domain transfer risk	4.0
				True	9		negative source domain; cross-domain transfer risk	4.0
				True	94		negative source domain; cross-domain transfer risk	4.0
				True	94		negative source domain; cross-domain transfer risk	4.0
				True	52		negative source domain; cross-domain transfer risk	4.0
				True	40		negative source domain; cross-domain transfer risk	4.0
				True	26		negative source domain; cross-domain transfer risk	4.0
				True	10		negative source domain; cross-domain transfer risk	4.0
				True	12		negative source domain; cross-domain transfer risk	4.0
				True	12		negative source domain; cross-domain transfer risk	4.0
				True	38		negative source domain; cross-domain transfer risk	4.0
				True	14		negative source domain; cross-domain transfer risk	4.0
				True	21		negative source domain; cross-domain transfer risk	4.0
				True	12		negative source domain; cross-domain transfer risk	4.0
				True	13		negative source domain; cross-domain transfer risk	4.0
				True	13		negative source domain; cross-domain transfer risk	4.0
				True	12		negative source domain; cross-domain transfer risk	4.0
				True	45		negative source domain; cross-domain transfer risk	4.0
				True	24		negative source domain; cross-domain transfer risk	4.0
				True	9		negative source domain; cross-domain transfer risk	4.0
				True	49		negative source domain; cross-domain transfer risk	4.0
				True	49		negative source domain; cross-domain transfer risk	4.0
				True	57		negative source domain; cross-domain transfer risk	4.0
				True	35		negative source domain; cross-domain transfer risk	4.0
				True	30		negative source domain; cross-domain transfer risk	4.0
				True	10		negative source domain; cross-domain transfer risk	4.0
				True	22		negative source domain; cross-domain transfer risk	4.0
				True	22		negative source domain; cross-domain transfer risk	4.0
				True	28		negative source domain; cross-domain transfer risk	4.0
				True	51		negative source domain; cross-domain transfer risk	4.0
				True	30		negative source domain; cross-domain transfer risk	4.0
				True	13		negative source domain; cross-domain transfer risk	4.0
				True	35		negative source domain; cross-domain transfer risk	4.0
				True	35		negative source domain; cross-domain transfer risk	4.0
				True	30		negative source domain; cross-domain transfer risk	4.0
				True	32		negative source domain; cross-domain transfer risk	4.0
				True	23		negative source domain; cross-domain transfer risk	4.0
				True	17		negative source domain; cross-domain transfer risk	2.0
				True	26		negative source domain; cross-domain transfer risk	4.0
				True	26		negative source domain; cross-domain transfer risk	4.0
				True	24		negative source domain; cross-domain transfer risk	4.0
				True	40		negative source domain; cross-domain transfer risk	4.0
				True	18		negative source domain; cross-domain transfer risk	4.0
				True	13		negative source domain; cross-domain transfer risk	4.0
				True	34		negative source domain; cross-domain transfer risk	4.0
				True	34		negative source domain; cross-domain transfer risk	4.0
				True	53		negative source domain; cross-domain transfer risk	4.0
				True	30		negative source domain; cross-domain transfer risk	4.0
				True	30		negative source domain; cross-domain transfer risk	4.0
				True	9		negative source domain; cross-domain transfer risk	4.0
				True	32		negative source domain; cross-domain transfer risk	4.0
				True	32		negative source domain; cross-domain transfer risk	4.0
				True	23		negative source domain; cross-domain transfer risk	4.0
				True	25		negative source domain; cross-domain transfer risk	4.0
				True	26		negative source domain; cross-domain transfer risk	4.0
				True	15		negative source domain; cross-domain transfer risk	4.0
				True	35		negative source domain; cross-domain transfer risk	4.0
				True	35		negative source domain; cross-domain transfer risk	4.0
				True	11		negative source domain; cross-domain transfer risk	4.0

experiment	dataset	model	scenario	converged	n_iter	loglik	warning_message	best_start_id
				True	46		negative source domain; cross-domain transfer risk	4.0
				True	28		negative source domain; cross-domain transfer risk	4.0
				True	9		negative source domain; cross-domain transfer risk	4.0
				True	20		negative source domain; cross-domain transfer risk	4.0
				True	20		negative source domain; cross-domain transfer risk	4.0
				True	25		negative source domain; cross-domain transfer risk	4.0
				True	21		negative source domain; cross-domain transfer risk	4.0
				True	30		negative source domain; cross-domain transfer risk	4.0
				True	9		negative source domain; cross-domain transfer risk	4.0
				True	77		negative source domain; cross-domain transfer risk	4.0
				True	77		negative source domain; cross-domain transfer risk	4.0
				True	33		negative source domain; cross-domain transfer risk	4.0
				True	25		negative source domain; cross-domain transfer risk	4.0
				True	22		negative source domain; cross-domain transfer risk	4.0
				True	10		negative source domain; cross-domain transfer risk	4.0
				True	25		negative source domain; cross-domain transfer risk	4.0
				True	25		negative source domain; cross-domain transfer risk	4.0
				True	19		negative source domain; cross-domain transfer risk	4.0
				True	40		negative source domain; cross-domain transfer risk	4.0
				True	34		negative source domain; cross-domain transfer risk	4.0
				True	11		negative source domain; cross-domain transfer risk	4.0
				True	52		negative source domain; cross-domain transfer risk	4.0
				True	52		negative source domain; cross-domain transfer risk	4.0
				True	37		negative source domain; cross-domain transfer risk	4.0
				True	24		negative source domain; cross-domain transfer risk	4.0
				True	33		negative source domain; cross-domain transfer risk	4.0
				True	17		negative source domain; cross-domain transfer risk	1.0
				True	14		negative source domain; cross-domain transfer risk	4.0
				True	14		negative source domain; cross-domain transfer risk	4.0
				True	16		negative source domain; cross-domain transfer risk	4.0
				True	11		negative source domain; cross-domain transfer risk	4.0
				True	24		negative source domain; cross-domain transfer risk	4.0
				True	10		negative source domain; cross-domain transfer risk	4.0
				True	26		negative source domain; cross-domain transfer risk	4.0
				True	26		negative source domain; cross-domain transfer risk	4.0
				True	28		negative source domain; cross-domain transfer risk	4.0
				True	14		negative source domain; cross-domain transfer risk	4.0
				True	23		negative source domain; cross-domain transfer risk	4.0
				True	10		negative source domain; cross-domain transfer risk	4.0
				True	53		negative source domain; cross-domain transfer risk	4.0
				True	53		negative source domain; cross-domain transfer risk	4.0
				True	21		negative source domain; cross-domain transfer risk	4.0
				True	15		negative source domain; cross-domain transfer risk	4.0
				True	27		negative source domain; cross-domain transfer risk	4.0
				True	9		negative source domain; cross-domain transfer risk	4.0
				True	75		negative source domain; cross-domain transfer risk	4.0
				True	75		negative source domain; cross-domain transfer risk	4.0
				True	25		negative source domain; cross-domain transfer risk	4.0
				True	21		negative source domain; cross-domain transfer risk	4.0
				True	37		negative source domain; cross-domain transfer risk	4.0
				True	10		negative source domain; cross-domain transfer risk	4.0
				True	9		negative source domain; cross-domain transfer risk	4.0
				True	9		negative source domain; cross-domain transfer risk	4.0
				True	20		negative source domain; cross-domain transfer risk	4.0
				True	12		negative source domain; cross-domain transfer risk	4.0
				True	23		negative source domain; cross-domain transfer risk	4.0
				True	12		negative source domain; cross-domain transfer risk	4.0
				True	10		negative source domain; cross-domain transfer risk	4.0
				True	10		negative source domain; cross-domain transfer risk	4.0
				True	22		negative source domain; cross-domain transfer risk	4.0
				True	14		negative source domain; cross-domain transfer risk	4.0
				True	24		negative source domain; cross-domain transfer risk	4.0
				True	10		negative source domain; cross-domain transfer risk	4.0
				True	18		negative source domain; cross-domain transfer risk	4.0
				True	18		negative source domain; cross-domain transfer risk	4.0
				True	14		negative source domain; cross-domain transfer risk	4.0
				True	18		negative source domain; cross-domain transfer risk	4.0
				True	22		negative source domain; cross-domain transfer risk	4.0
				True	12		negative source domain; cross-domain transfer risk	4.0
				True	24		negative source domain; cross-domain transfer risk	4.0
				True	24		negative source domain; cross-domain transfer risk	4.0
				True	31		negative source domain; cross-domain transfer risk	4.0
				True	25		negative source domain; cross-domain transfer risk	4.0
				True	25		negative source domain; cross-domain transfer risk	4.0
				True	12		negative source domain; cross-domain transfer risk	4.0
				True	20		negative source domain; cross-domain transfer risk	4.0
				True	20		negative source domain; cross-domain transfer risk	4.0
				True	35		negative source domain; cross-domain transfer risk	4.0
				True	13		negative source domain; cross-domain transfer risk	4.0
				True	32		negative source domain; cross-domain transfer risk	4.0
				True	9		negative source domain; cross-domain transfer risk	4.0
				True	23		negative source domain; cross-domain transfer risk	4.0
				True	23		negative source domain; cross-domain transfer risk	4.0
				True	20		negative source domain; cross-domain transfer risk	4.0
				True	21		negative source domain; cross-domain transfer risk	4.0
				True	38		negative source domain; cross-domain transfer risk	4.0
				True	10		negative source domain; cross-domain transfer risk	4.0
				True	22		negative source domain; cross-domain transfer risk	4.0
				True	22		negative source domain; cross-domain transfer risk	4.0
				True	22		negative source domain; cross-domain transfer risk	4.0
				True	25		negative source domain; cross-domain transfer risk	4.0
				True	34		negative source domain; cross-domain transfer risk	4.0
				True	13		negative source domain; cross-domain transfer risk	4.0
				True	37		negative source domain; cross-domain transfer risk	4.0
				True	37		negative source domain; cross-domain transfer risk	4.0
				True	32		negative source domain; cross-domain transfer risk	4.0
				True	25		negative source domain; cross-domain transfer risk	4.0
				True	25		negative source domain; cross-domain transfer risk	4.0
				True	10		negative source domain; cross-domain transfer risk	4.0
				True	17		negative source domain; cross-domain transfer risk	4.0
				True	17		negative source domain; cross-domain transfer risk	4.0
				True	15		negative source domain; cross-domain transfer risk	4.0
				True	32		negative source domain; cross-domain transfer risk	4.0

experiment	dataset	model	scenario	converged	n__iter	loglik	warning__message	best__start__id
				True	32		negative source domain; cross-domain transfer risk	4.0
				True	13		negative source domain; cross-domain transfer risk	1.0
				True	38		negative source domain; cross-domain transfer risk	4.0
				True	38		negative source domain; cross-domain transfer risk	4.0
				True	29		negative source domain; cross-domain transfer risk	4.0
				True	17		negative source domain; cross-domain transfer risk	4.0
				True	31		negative source domain; cross-domain transfer risk	4.0
				True	9		negative source domain; cross-domain transfer risk	4.0
				True	18		negative source domain; cross-domain transfer risk	4.0
				True	18		negative source domain; cross-domain transfer risk	4.0
				True	21		negative source domain; cross-domain transfer risk	4.0
				True	24		negative source domain; cross-domain transfer risk	4.0
				True	25		negative source domain; cross-domain transfer risk	4.0
				True	12		negative source domain; cross-domain transfer risk	4.0
				True	86		negative source domain; cross-domain transfer risk	3.0
				True	86		negative source domain; cross-domain transfer risk	3.0
				True	9		negative source domain; cross-domain transfer risk	4.0
				True	43		negative source domain; cross-domain transfer risk	4.0
				True	19		negative source domain; cross-domain transfer risk	4.0
				True	9		negative source domain; cross-domain transfer risk	4.0
				True	13		negative source domain; cross-domain transfer risk	4.0
				True	13		negative source domain; cross-domain transfer risk	4.0
				True	14		negative source domain; cross-domain transfer risk	4.0
				True	28		negative source domain; cross-domain transfer risk	4.0
				True	36		negative source domain; cross-domain transfer risk	4.0
				True	9		negative source domain; cross-domain transfer risk	4.0
				True	35		negative source domain; cross-domain transfer risk	4.0
				True	35		negative source domain; cross-domain transfer risk	4.0
				True	24		negative source domain; cross-domain transfer risk	4.0
				True	30		negative source domain; cross-domain transfer risk	4.0
				True	22		negative source domain; cross-domain transfer risk	4.0
				True	12		negative source domain; cross-domain transfer risk	4.0
				True	41		negative source domain; cross-domain transfer risk	4.0
				True	41		negative source domain; cross-domain transfer risk	4.0
				True	47		negative source domain; cross-domain transfer risk	4.0
				True	15		negative source domain; cross-domain transfer risk	4.0
				True	24		negative source domain; cross-domain transfer risk	4.0
				True	13		negative source domain; cross-domain transfer risk	4.0
				True	18		negative source domain; cross-domain transfer risk	4.0
				True	18		negative source domain; cross-domain transfer risk	4.0
				True	28		negative source domain; cross-domain transfer risk	4.0
				True	20		negative source domain; cross-domain transfer risk	4.0
				True	31		negative source domain; cross-domain transfer risk	4.0
				True	11		negative source domain; cross-domain transfer risk	4.0
				True	54		negative source domain; cross-domain transfer risk	4.0
				True	54		negative source domain; cross-domain transfer risk	4.0
				True	19		negative source domain; cross-domain transfer risk	4.0
				True	13		negative source domain; cross-domain transfer risk	4.0
				True	31		negative source domain; cross-domain transfer risk	4.0
				True	10		negative source domain; cross-domain transfer risk	4.0
				True	29		negative source domain; cross-domain transfer risk	4.0
				True	29		negative source domain; cross-domain transfer risk	4.0
				True	16		negative source domain; cross-domain transfer risk	4.0
				True	11		negative source domain; cross-domain transfer risk	4.0
				True	24		negative source domain; cross-domain transfer risk	4.0
				True	14		negative source domain; cross-domain transfer risk	3.0
				True	15		negative source domain; cross-domain transfer risk	4.0
				True	15		negative source domain; cross-domain transfer risk	4.0
				True	42		negative source domain; cross-domain transfer risk	4.0
				True	31		negative source domain; cross-domain transfer risk	4.0
				True	25		negative source domain; cross-domain transfer risk	4.0
				True	13		negative source domain; cross-domain transfer risk	4.0
				True	55		negative source domain; cross-domain transfer risk	4.0
				True	55		negative source domain; cross-domain transfer risk	4.0
				True	12		negative source domain; cross-domain transfer risk	4.0
				True	23		negative source domain; cross-domain transfer risk	4.0
				True	24		negative source domain; cross-domain transfer risk	4.0
				True	13		negative source domain; cross-domain transfer risk	4.0
				True	24		negative source domain; cross-domain transfer risk	4.0
				True	24		negative source domain; cross-domain transfer risk	4.0
				True	13		negative source domain; cross-domain transfer risk	4.0
				True	14		negative source domain; cross-domain transfer risk	4.0
				True	24		negative source domain; cross-domain transfer risk	4.0
				True	13		negative source domain; cross-domain transfer risk	4.0
				True	22		negative source domain; cross-domain transfer risk	4.0
				True	22		negative source domain; cross-domain transfer risk	4.0
				True	26		negative source domain; cross-domain transfer risk	4.0
				True	20		negative source domain; cross-domain transfer risk	4.0
				True	33		negative source domain; cross-domain transfer risk	4.0
				True	15		negative source domain; cross-domain transfer risk	4.0
				True	15		negative source domain; cross-domain transfer risk	4.0
				True	15		negative source domain; cross-domain transfer risk	4.0
				True	14		negative source domain; cross-domain transfer risk	4.0
				True	19		negative source domain; cross-domain transfer risk	4.0
				True	23		negative source domain; cross-domain transfer risk	4.0
				True	13		negative source domain; cross-domain transfer risk	4.0
				True	44		negative source domain; cross-domain transfer risk	4.0
				True	44		negative source domain; cross-domain transfer risk	4.0
				True	21		negative source domain; cross-domain transfer risk	4.0
				True	23		negative source domain; cross-domain transfer risk	4.0
				True	39		negative source domain; cross-domain transfer risk	4.0
				True	13		negative source domain; cross-domain transfer risk	4.0
				True	39		negative source domain; cross-domain transfer risk	4.0
				True	39		negative source domain; cross-domain transfer risk	4.0
				True	16		negative source domain; cross-domain transfer risk	4.0
				True	13		negative source domain; cross-domain transfer risk	4.0
				True	27		negative source domain; cross-domain transfer risk	4.0
				True	10		negative source domain; cross-domain transfer risk	4.0
				True	29		negative source domain; cross-domain transfer risk	4.0
				True	29		negative source domain; cross-domain transfer risk	4.0
				True	24		negative source domain; cross-domain transfer risk	4.0
				True	31		negative source domain; cross-domain transfer risk	4.0
				True	19		negative source domain; cross-domain transfer risk	4.0

experiment	dataset	model	scenario	converged	n__iter	loglik	warning__message	best__start__id
				True	13		negative source domain; cross-domain transfer risk	4.0
				True	31		negative source domain; cross-domain transfer risk	4.0
				True	31		negative source domain; cross-domain transfer risk	4.0
				True	15		negative source domain; cross-domain transfer risk	4.0
				True	21		negative source domain; cross-domain transfer risk	4.0
				True	26		negative source domain; cross-domain transfer risk	4.0
				True	10		negative source domain; cross-domain transfer risk	4.0
				True	12		negative source domain; cross-domain transfer risk	4.0
				True	12		negative source domain; cross-domain transfer risk	4.0
				True	15		negative source domain; cross-domain transfer risk	4.0
				True	14		negative source domain; cross-domain transfer risk	4.0
				True	14		negative source domain; cross-domain transfer risk	4.0
				True	10		negative source domain; cross-domain transfer risk	4.0
				True	25		negative source domain; cross-domain transfer risk	4.0
				True	25		negative source domain; cross-domain transfer risk	4.0
				True	16		negative source domain; cross-domain transfer risk	4.0
				True	16		negative source domain; cross-domain transfer risk	4.0
				True	21		negative source domain; cross-domain transfer risk	4.0
				True	10		negative source domain; cross-domain transfer risk	4.0
				True	21		negative source domain; cross-domain transfer risk	4.0
				True	21		negative source domain; cross-domain transfer risk	4.0
				True	14		negative source domain; cross-domain transfer risk	4.0
				True	12		negative source domain; cross-domain transfer risk	4.0
				True	26		negative source domain; cross-domain transfer risk	4.0
				True	17		negative source domain; cross-domain transfer risk	3.0
				True	25		negative source domain; cross-domain transfer risk	4.0
				True	25		negative source domain; cross-domain transfer risk	4.0
				True	22		negative source domain; cross-domain transfer risk	4.0
				True	15		negative source domain; cross-domain transfer risk	4.0
				True	30		negative source domain; cross-domain transfer risk	4.0
				True	10		negative source domain; cross-domain transfer risk	4.0
				True	27		negative source domain; cross-domain transfer risk	4.0
				True	27		negative source domain; cross-domain transfer risk	4.0
				True	38		negative source domain; cross-domain transfer risk	4.0
				True	31		negative source domain; cross-domain transfer risk	4.0
				True	22		negative source domain; cross-domain transfer risk	4.0
				True	14		negative source domain; cross-domain transfer risk	4.0
				True	10		negative source domain; cross-domain transfer risk	4.0
				True	10		negative source domain; cross-domain transfer risk	4.0
				True	18		negative source domain; cross-domain transfer risk	4.0
				True	14		negative source domain; cross-domain transfer risk	4.0
				True	25		negative source domain; cross-domain transfer risk	4.0
				True	11		negative source domain; cross-domain transfer risk	4.0
				True	24		negative source domain; cross-domain transfer risk	4.0
				True	24		negative source domain; cross-domain transfer risk	4.0
				True	24		negative source domain; cross-domain transfer risk	4.0
				True	17		negative source domain; cross-domain transfer risk	4.0
				True	28		negative source domain; cross-domain transfer risk	4.0
				True	17		negative source domain; cross-domain transfer risk	2.0
				True	75		negative source domain; cross-domain transfer risk	4.0
				True	75		negative source domain; cross-domain transfer risk	4.0
				True	33		negative source domain; cross-domain transfer risk	4.0
				True	40		negative source domain; cross-domain transfer risk	4.0
				True	32		negative source domain; cross-domain transfer risk	4.0
				True	11		negative source domain; cross-domain transfer risk	4.0
				True	28		negative source domain; cross-domain transfer risk	4.0
				True	28		negative source domain; cross-domain transfer risk	4.0
				True	15		negative source domain; cross-domain transfer risk	4.0
				True	28		negative source domain; cross-domain transfer risk	4.0
				True	38		negative source domain; cross-domain transfer risk	4.0
				True	11		negative source domain; cross-domain transfer risk	4.0
				True	9		negative source domain; cross-domain transfer risk	4.0
				True	9		negative source domain; cross-domain transfer risk	4.0
				True	14		negative source domain; cross-domain transfer risk	4.0
				True	32		negative source domain; cross-domain transfer risk	4.0
				True	25		negative source domain; cross-domain transfer risk	4.0
				True	10		negative source domain; cross-domain transfer risk	4.0
				True	26		negative source domain; cross-domain transfer risk	4.0
				True	26		negative source domain; cross-domain transfer risk	4.0
				True	30		negative source domain; cross-domain transfer risk	4.0
				True	15		negative source domain; cross-domain transfer risk	4.0
				True	30		negative source domain; cross-domain transfer risk	4.0
				True	10		negative source domain; cross-domain transfer risk	4.0
				True	64		negative source domain; cross-domain transfer risk	4.0
				True	64		negative source domain; cross-domain transfer risk	4.0
				True	49		negative source domain; cross-domain transfer risk	4.0
				True	30		negative source domain; cross-domain transfer risk	4.0
				True	31		negative source domain; cross-domain transfer risk	4.0
				True	10		negative source domain; cross-domain transfer risk	4.0
				True	24		negative source domain; cross-domain transfer risk	4.0
				True	24		negative source domain; cross-domain transfer risk	4.0
				True	12		negative source domain; cross-domain transfer risk	4.0
				True	35		negative source domain; cross-domain transfer risk	4.0
				True	29		negative source domain; cross-domain transfer risk	4.0
				True	9		negative source domain; cross-domain transfer risk	4.0
				True	9		negative source domain; cross-domain transfer risk	4.0
				True	9		negative source domain; cross-domain transfer risk	4.0
				True	44		negative source domain; cross-domain transfer risk	4.0
				True	18		negative source domain; cross-domain transfer risk	4.0
				True	18		negative source domain; cross-domain transfer risk	4.0
				True	10		negative source domain; cross-domain transfer risk	4.0
				True	10		negative source domain; cross-domain transfer risk	4.0
				True	10		negative source domain; cross-domain transfer risk	4.0
				True	10		negative source domain; cross-domain transfer risk	4.0
				True	21		negative source domain; cross-domain transfer risk	4.0
				True	35		negative source domain; cross-domain transfer risk	4.0
				True	12		negative source domain; cross-domain transfer risk	4.0
				True	12		negative source domain; cross-domain transfer risk	4.0
				True	12		negative source domain; cross-domain transfer risk	4.0
				True	49		negative source domain; cross-domain transfer risk	4.0
				True	15		negative source domain; cross-domain transfer risk	4.0
				True	22		negative source domain; cross-domain transfer risk	4.0
				True	9		negative source domain; cross-domain transfer risk	4.0

experiment	dataset	model	scenario	converged	n__iter	loglik	warning__message	best__start__id
				True	24		negative source domain; cross-domain transfer risk	4.0
				True	24		negative source domain; cross-domain transfer risk	4.0
				True	22		negative source domain; cross-domain transfer risk	4.0
				True	29		negative source domain; cross-domain transfer risk	4.0
				True	21		negative source domain; cross-domain transfer risk	4.0
				True	12		negative source domain; cross-domain transfer risk	4.0
				True	34		negative source domain; cross-domain transfer risk	4.0
				True	34		negative source domain; cross-domain transfer risk	4.0
				True	49		negative source domain; cross-domain transfer risk	4.0
				True	26		negative source domain; cross-domain transfer risk	5.0
				True	42		negative source domain; cross-domain transfer risk	4.0
				True	14		negative source domain; cross-domain transfer risk	4.0

S13 R = 300 paired statistical checks

Source CSV: `tables/s13_r300_paired_statistical_checks.csv`. Rows displayed: 244; columns displayed: 15. The table is split into panels to keep all cells inside the page boundary.

Panel 1 of 3

Columns shown: `metric`, `group`, `n_pairs`, `mean_difference`, `median_difference`, `bootstrap_ci_lower`, `bootstrap_ci_upper`, `wilcoxon_statistic`, `wilcoxon_pvalue`.

metric	group	n_pairs	mean_difference	median_difference	bootstrap_ci_lower	bootstrap_ci_upper	wilcoxon_statistic	wilcoxon_pvalue
beta_error	overall	18000	0.0103722342240322	-2.768184002394959e-05	0.0081138544797158	0.0126475239123667	77351841.0	1.6119131562712197e-07
beta_error	dataset_source=WDI_agriculture; pseudo_model_source=HQER-SFA; error_scenario=ald; sample_size_label=large	300	-0.1823632083050322	-0.1080884788149786	-0.2212821908209202	-0.1397803183608819	9369.0	1.610000273404498e-18
beta_error	dataset_source=WDI_agriculture; pseudo_model_source=HQER-SFA; error_scenario=ald; sample_size_label=medium	300	-0.267593057197004	-0.1167823573671962	-0.31246791708059	-0.2203101898264398	6821.0	1.1118201525961575e-25
beta_error	dataset_source=WDI_agriculture; pseudo_model_source=HQER-SFA; error_scenario=hqer_induced; sample_size_label=large	300	0.5108871703128701	0.5073881262543571	0.4999993691249969	0.5222287375961386	0.0	6.114136859304472e-51
beta_error	dataset_source=WDI_agriculture; pseudo_model_source=HQER-SFA; error_scenario=hqer_induced; sample_size_label=medium	300	0.4246294969800824	0.4408539616727562	0.4054707467181467	0.4428385425161812	117.0	1.970268843691161e-50
beta_error	dataset_source=WDI_agriculture; pseudo_model_source=HQER-SFA; error_scenario=normal; sample_size_label=large	300	0.4515680456702547	0.4592338508992571	0.4391434868872353	0.4641540055721379	0.0	6.114136859304472e-51
beta_error	dataset_source=WDI_agriculture; pseudo_model_source=HQER-SFA; error_scenario=normal; sample_size_label=medium	300	0.3868790007727782	0.4009839203687934	0.3643044529772068	0.4076158137499708	260.0	8.167558394144293e-50
beta_error	dataset_source=WDI_agriculture; pseudo_model_source=HQER-SFA; error_scenario=skewed; sample_size_label=large	300	-0.257000045164817	-0.2495562581174209	-0.2724933585509357	-0.2428598666476862	66.0	1.18393662523322e-50
beta_error	dataset_source=WDI_agriculture; pseudo_model_source=HQER-SFA; error_scenario=skewed; sample_size_label=medium	300	-0.2866807433172212	-0.2835463667431727	-0.3110828335509331	-0.2638911486242806	557.0	1.5213794573643938e-48
beta_error	dataset_source=WDI_agriculture; pseudo_model_source=HQER-SFA; error_scenario=student_t; sample_size_label=large	300	0.0003493549781314	0.0001602690212969	-0.0058287957654053	0.0062506945850844	22266.0	0.8374519224360457
beta_error	dataset_source=WDI_agriculture; pseudo_model_source=HQER-SFA; error_scenario=student_t; sample_size_label=medium	300	0.0015125798315008	0.0004865268504494	-0.0050865733530536	0.008507812273434	21306.0	0.3989164067359962
beta_error	dataset_source=WDI_agriculture; pseudo_model_source=Quantile-SFA; error_scenario=ald; sample_size_label=large	300	0.0008540203104377	-3.998695964865448e-05	-0.0002203229547679	0.0019454744779413	21296.0	0.3952093949775773
beta_error	dataset_source=WDI_agriculture; pseudo_model_source=Quantile-SFA; error_scenario=ald; sample_size_label=medium	300	0.0046680644358353	0.0004231366264306	-5.537956100316711e-05	0.0106388129167734	18953.0	0.0160264174054928
beta_error	dataset_source=WDI_agriculture; pseudo_model_source=Quantile-SFA; error_scenario=hqer_induced; sample_size_label=large	300	0.0001148436304515	8.586828805945412e-05	-0.00157876025188	0.00156495808983	21437.0	0.449383941345956
beta_error	dataset_source=WDI_agriculture; pseudo_model_source=Quantile-SFA; error_scenario=hqer_induced; sample_size_label=medium	300	0.0031642775291903	0.0002938842749391	-0.0008853115771165	0.0074584425482425	19191.0	0.0244462577159569
beta_error	dataset_source=WDI_agriculture; pseudo_model_source=Quantile-SFA; error_scenario=normal; sample_size_label=large	300	-0.0015417960898557	-0.0003332986712857	-0.0044247321542905	0.0013632863176805	21671.0	0.5479522460537845
beta_error	dataset_source=WDI_agriculture; pseudo_model_source=Quantile-SFA; error_scenario=normal; sample_size_label=medium	300	-0.0052230456092582	-0.0006641781759619	-0.0107141337101937	0.0003228452884071	18931.0	0.0153953217168606
beta_error	dataset_source=WDI_agriculture; pseudo_model_source=Quantile-SFA; error_scenario=skewed; sample_size_label=large	300	0.0032375909898372	0.000556323022271	-0.0009796960103072	0.007699744931815	20726.0	0.2189741116685059
beta_error	dataset_source=WDI_agriculture; pseudo_model_source=Quantile-SFA; error_scenario=skewed; sample_size_label=medium	300	0.0012913334279168	0.0001953640204648	-0.001756853164417	0.0047262878803481	20915.0	0.2697776369184339
beta_error	dataset_source=WDI_agriculture; pseudo_model_source=Quantile-SFA; error_scenario=student_t; sample_size_label=large	300	-0.0001637638787638	-0.0001894558270728	-0.002829766438722	0.0025993672260131	21931.0	0.6687018500257207
beta_error	dataset_source=WDI_agriculture; pseudo_model_source=Quantile-SFA; error_scenario=student_t; sample_size_label=medium	300	0.0041169628263945	0.0002214607389841	0.0011645540882985	0.0071605874311986	20078.0	0.0968775530692839
beta_error	dataset_source=WDI_industry; pseudo_model_source=HQER-SFA; error_scenario=ald; sample_size_label=large	300	2.7043771622439228e-05	-5.363180957365047e-05	-0.0005147796324925	0.0005914210814634	22217.0	0.8120834803515267
beta_error	dataset_source=WDI_industry; pseudo_model_source=HQER-SFA; error_scenario=ald; sample_size_label=medium	300	0.0001664900820101	0.0003915865299252	-0.0009764197863859	0.0012791816411646	21984.0	0.6945520843661003
beta_error	dataset_source=WDI_industry; pseudo_model_source=HQER-SFA; error_scenario=hqer_induced; sample_size_label=large	300	0.001713477126726	0.0013379893031955	0.0012257640211099	0.0021822675988985	11798.0	7.69844830955258e-13
beta_error	dataset_source=WDI_industry; pseudo_model_source=HQER-SFA; error_scenario=hqer_induced; sample_size_label=medium	300	-0.0001165944925085	0.0002752259317887	-0.0011706921919597	0.0009564561507197	21730.0	0.5743911673155322
beta_error	dataset_source=WDI_industry; pseudo_model_source=HQER-SFA; error_scenario=normal; sample_size_label=large	300	0.0015903251863108	0.0014448365422799	0.0006268579221699	0.0025639859450189	17523.0	0.0007814877048777
beta_error	dataset_source=WDI_industry; pseudo_model_source=HQER-SFA; error_scenario=normal; sample_size_label=medium	300	0.002249378347609	0.0014012641890501	0.0005053873721279	0.0039927040011787	18216.0	0.0037504604219759
beta_error	dataset_source=WDI_industry; pseudo_model_source=HQER-SFA; error_scenario=skewed; sample_size_label=large	300	-0.0200684379304774	-0.0181646585700199	-0.0214122861669053	-0.0187969813646643	60.0	1.114994117775391e-50
beta_error	dataset_source=WDI_industry; pseudo_model_source=HQER-SFA; error_scenario=skewed; sample_size_label=medium	300	-0.0229346954408349	-0.0194117908126125	-0.0248257732653071	-0.0211369850801996	18.0	7.322993782895607e-51
beta_error	dataset_source=WDI_industry; pseudo_model_source=HQER-SFA; error_scenario=student_t; sample_size_label=large	300	-0.0003528471310606	-8.700781298659997e-05	-0.0006621889941269	-3.76424281169196e-05	19652.0	0.0519587547293379
beta_error	dataset_source=WDI_industry; pseudo_model_source=HQER-SFA; error_scenario=student_t; sample_size_label=medium	300	-0.0013624404538719	-0.0003875515999846	-0.0019597844056736	-0.0007986375177237	16012.0	1.2765226941733866e-05
beta_error	dataset_source=WDI_industry; pseudo_model_source=Quantile-SFA; error_scenario=ald; sample_size_label=large	300	1.6780663529896526e-05	-1.0990142660200278e-05	-0.0001292769652998	0.0001617789593128	21833.0	0.6219413993770058
beta_error	dataset_source=WDI_industry; pseudo_model_source=Quantile-SFA; error_scenario=ald; sample_size_label=medium	300	7.010469101659445e-05	-8.17344044201293e-07	-0.000333921422916	0.0004173548213589	22453.0	0.9356025724728956
beta_error	dataset_source=WDI_industry; pseudo_model_source=Quantile-SFA; error_scenario=hqer_induced; sample_size_label=large	300	0.0001293823917373	4.95414166631486e-05	-1.1837695182696058e-05	0.0002649365225941	19701.0	0.0560187051622888
beta_error	dataset_source=WDI_industry; pseudo_model_source=Quantile-SFA; error_scenario=hqer_induced; sample_size_label=medium	300	0.0003841384465282	2.941274444629924e-05	-0.0001007768355526	0.0009784939959956	21255.0	0.380229621820012
beta_error	dataset_source=WDI_industry; pseudo_model_source=Quantile-SFA; error_scenario=normal; sample_size_label=large	300	-1.4812281276923382e-05	-7.300245984299784e-06	-0.0002719826038502	0.0002480692400609	22371.0	0.8923522375455399
beta_error	dataset_source=WDI_industry; pseudo_model_source=Quantile-SFA; error_scenario=normal; sample_size_label=medium	300	0.0003018969975391	-5.474662309150336e-06	-7.861816532867928e-05	0.0006907844288573	22410.0	0.9128906099416242
beta_error	dataset_source=WDI_industry; pseudo_model_source=Quantile-SFA; error_scenario=skewed; sample_size_label=large	300	-0.0005331721979271	-8.001980224744965e-05	-0.0007990930708718	-0.0002593936288444	16356.0	3.544452708897018e-05

metric	group	n_pairs	mean_difference	median_difference	bootstrap_ci_lower	bootstrap_ci_upper	wilcoxon_statistic	wilcoxon_pvalue
beta_error	dataset_source=WDI_industry; pseudo_model_source=Quantile-SFA; error_scenario=skewed; sample_size_label=medium	300	-0.000571149328664	-0.000123759876711	-0.0011922716961875	8.007350196301307e-05	16525.0	5.7475157777670514e-05
beta_error	dataset_source=WDI_industry; pseudo_model_source=Quantile-SFA; error_scenario=student_t; sample_size_label=large	300	-2.949336543262998e-05	-7.530888714298717e-06	-7.163118377318384e-05	1.2663111421637562e-05	21640.0	0.5343058200301174
beta_error	dataset_source=WDI_industry; pseudo_model_source=Quantile-SFA; error_scenario=student_t; sample_size_label=medium	300	-7.712642134439127e-05	-1.706083783085087e-05	-0.0001925812535571	3.644356501271093e-05	19411.0	0.035401013161192
beta_error	dataset_source=front41Data; pseudo_model_source=HQER-SFA; error_scenario=ald; sample_size_label=medium	300	-0.0434731133614975	-0.0385390383365328	-0.0481894451724828	-0.0393977187806483	1654.0	5.339283194898734e-44
beta_error	dataset_source=front41Data; pseudo_model_source=HQER-SFA; error_scenario=ald; sample_size_label=small	300	-0.0358047897612751	-0.0207810832175566	-0.04434492614984	-0.0276682285089967	10630.0	1.9711846430609805e-15
beta_error	dataset_source=front41Data; pseudo_model_source=HQER-SFA; error_scenario=hqer_induced; sample_size_label=medium	300	0.0066166841241902	0.0059400558687047	0.0046508280835952	0.0084165152605888	12248.0	6.54890267888311e-12
beta_error	dataset_source=front41Data; pseudo_model_source=HQER-SFA; error_scenario=hqer_induced; sample_size_label=small	300	-0.0124216316568782	-0.0019635768367968	-0.0172675471246247	-0.0076508966598172	17155.0	0.0003133675858035
beta_error	dataset_source=front41Data; pseudo_model_source=HQER-SFA; error_scenario=normal; sample_size_label=medium	300	0.0064974792200514	0.0037765647303497	0.0051104920142312	0.0080477212706228	10743.0	3.603573272796746e-15
beta_error	dataset_source=front41Data; pseudo_model_source=HQER-SFA; error_scenario=normal; sample_size_label=small	300	0.0039568197359663	0.0027626543239173	0.000342700917202	0.007609473437856	19116.0	0.0214528848668656
beta_error	dataset_source=front41Data; pseudo_model_source=HQER-SFA; error_scenario=skewed; sample_size_label=medium	300	-0.019981294999148	-0.0170772586821148	-0.0221084429369983	-0.018186278472398	1332.0	2.6132206495684402e-45
beta_error	dataset_source=front41Data; pseudo_model_source=HQER-SFA; error_scenario=skewed; sample_size_label=small	300	-0.0326780882053096	-0.020318161996157	-0.0375403304759495	-0.0278293230406484	6121.0	7.286189914845539e-28
beta_error	dataset_source=front41Data; pseudo_model_source=HQER-SFA; error_scenario=student_t; sample_size_label=medium	300	-0.0001327245112702	-3.006987276880056e-05	-0.0004291510130423	0.000134871528673	20867.0	0.2561679802245096
beta_error	dataset_source=front41Data; pseudo_model_source=HQER-SFA; error_scenario=student_t; sample_size_label=small	300	-0.0044192735504952	-7.347145504184999e-05	-0.0078911603181652	-0.0012605056586641	20296.0	0.1297186001651179
beta_error	dataset_source=front41Data; pseudo_model_source=Quantile-SFA; error_scenario=ald; sample_size_label=medium	300	-5.668277938660524e-05	-1.856740587805224e-05	-0.0004513812274325	0.0003167276385437	21477.0	0.4654865766796486
beta_error	dataset_source=front41Data; pseudo_model_source=Quantile-SFA; error_scenario=ald; sample_size_label=small	300	-0.0001426308717241	-6.286949934850711e-06	-0.0017824808194225	0.0014835571017295	21079.0	0.319972664155052
beta_error	dataset_source=front41Data; pseudo_model_source=Quantile-SFA; error_scenario=hqer_induced; sample_size_label=medium	300	7.928133608885046e-05	3.194691517848826e-06	-0.0002958673425831	0.0004774970952311	22563.0	0.9938981970538948
beta_error	dataset_source=front41Data; pseudo_model_source=Quantile-SFA; error_scenario=hqer_induced; sample_size_label=small	300	0.0005877074963941	3.868924066549595e-06	-0.000719361371917	0.0019548367903014	21851.0	0.630423629064728
beta_error	dataset_source=front41Data; pseudo_model_source=Quantile-SFA; error_scenario=normal; sample_size_label=medium	300	0.000711098236548	4.739003885601174e-06	7.201295186106523e-05	0.001419652712392	21359.0	0.4189105178329285
beta_error	dataset_source=front41Data; pseudo_model_source=Quantile-SFA; error_scenario=normal; sample_size_label=small	300	7.468158284774477e-05	1.4572260215549653e-05	-0.0010170070096004	0.001096686189051	21081.0	0.3206202695103319
beta_error	dataset_source=front41Data; pseudo_model_source=Quantile-SFA; error_scenario=skewed; sample_size_label=medium	300	-1.5621025429103327e-05	-1.9117361944400585e-05	-0.0003820287735079	0.0003756759727652	19835.0	0.0684884471253598
beta_error	dataset_source=front41Data; pseudo_model_source=Quantile-SFA; error_scenario=skewed; sample_size_label=small	300	0.0001118428875772	-1.259124232390156e-05	-0.001649495093124	0.0019604211031301	21577.0	0.5071116231757165
beta_error	dataset_source=front41Data; pseudo_model_source=Quantile-SFA; error_scenario=student_t; sample_size_label=medium	300	0.000147077703402	8.938342568648797e-06	-2.939464603099424e-05	0.0003407955751791	19665.0	0.0530111283319706
beta_error	dataset_source=front41Data; pseudo_model_source=Quantile-SFA; error_scenario=student_t; sample_size_label=small	300	-0.0006180989536781	-1.2931280318203424e-05	-0.0014304346560103	0.0001962659738085	20034.0	0.0911344434043255
y_rmse	overall	18000	0.0085274533024821	-7.9518466811479e-06	0.0071717723962207	0.0100218493846847	74409677.0	3.095030170149956e-21
y_rmse	dataset_source=WDI_agriculture; pseudo_model_source=HQER-SFA; error_scenario=ald; sample_size_label=large	300	-0.114125584249184	-0.0421040137640151	-0.1408662211608601	-0.0893623163600825	11133.0	2.7697864216783263e-14
y_rmse	dataset_source=WDI_agriculture; pseudo_model_source=HQER-SFA; error_scenario=ald; sample_size_label=medium	300	-0.1518014482351284	-0.0433015932191447	-0.1826160155841425	-0.1188681657143386	10026.0	7.13299524450916e-17
y_rmse	dataset_source=WDI_agriculture; pseudo_model_source=HQER-SFA; error_scenario=hqer_induced; sample_size_label=large	300	0.3542409259761455	0.3467552910240433	0.3476810205621156	0.3607537516272465	0.0	6.114136859304472e-51
y_rmse	dataset_source=WDI_agriculture; pseudo_model_source=HQER-SFA; error_scenario=hqer_induced; sample_size_label=medium	300	0.3421026564294921	0.3400896810381907	0.3309548346113503	0.3522117511599935	0.0	6.114136859304472e-51
y_rmse	dataset_source=WDI_agriculture; pseudo_model_source=HQER-SFA; error_scenario=normal; sample_size_label=large	300	0.2128941389238251	0.207888153904593	0.2065420696901232	0.2192235960218607	0.0	6.114136859304472e-51
y_rmse	dataset_source=WDI_agriculture; pseudo_model_source=HQER-SFA; error_scenario=normal; sample_size_label=medium	300	0.212317950901314	0.1973959926103698	0.201542112191278	0.2230288962418551	1.0	6.175750667302515e-51
y_rmse	dataset_source=WDI_agriculture; pseudo_model_source=HQER-SFA; error_scenario=skewed; sample_size_label=large	300	-0.1197657069494218	-0.1165274921833475	-0.1272201613688281	-0.1124670488020039	64.0	1.1604967471045638e-50
y_rmse	dataset_source=WDI_agriculture; pseudo_model_source=HQER-SFA; error_scenario=skewed; sample_size_label=medium	300	-0.1262247597148308	-0.1269894732351892	-0.1359873388377845	-0.116303872683939	522.0	1.0800248668858544e-48
y_rmse	dataset_source=WDI_agriculture; pseudo_model_source=HQER-SFA; error_scenario=student_t; sample_size_label=large	300	-0.0008522104979365	-1.9920883796986644e-05	-0.0015759077097703	-0.0002101929320816	20549.0	0.1779910595602486
y_rmse	dataset_source=WDI_agriculture; pseudo_model_source=HQER-SFA; error_scenario=student_t; sample_size_label=medium	300	-6.011443028394959e-05	1.1110372682754388e-05	-0.0020645620900345	0.0019065935473066	22182.0	0.7940815401282426
y_rmse	dataset_source=WDI_agriculture; pseudo_model_source=Quantile-SFA; error_scenario=ald; sample_size_label=large	300	0.0003516431934481	0.0001038513082757	-0.0006680550800807	0.0013133252106175	20616.0	0.1927758935014809
y_rmse	dataset_source=WDI_agriculture; pseudo_model_source=Quantile-SFA; error_scenario=ald; sample_size_label=medium	300	0.002150471551576	4.356011552164585e-05	-0.0019028952169425	0.005887942405907	21647.0	0.5373722143327444
y_rmse	dataset_source=WDI_agriculture; pseudo_model_source=Quantile-SFA; error_scenario=hqer_induced; sample_size_label=large	300	0.0002125524591423	0.0001438911907265	-0.0014089147391478	0.001517885490806	18848.0	0.0132069740304227
y_rmse	dataset_source=WDI_agriculture; pseudo_model_source=Quantile-SFA; error_scenario=hqer_induced; sample_size_label=medium	300	0.0005215817805357	5.2548289342535526e-05	-0.0019227606889306	0.003307108554537	21696.0	0.559081487711892
y_rmse	dataset_source=WDI_agriculture; pseudo_model_source=Quantile-SFA; error_scenario=normal; sample_size_label=large	300	0.0004554404386456	-0.0002919106949738	-0.0023924999270305	0.0035630294676811	21956.0	0.6808487477443097
y_rmse	dataset_source=WDI_agriculture; pseudo_model_source=Quantile-SFA; error_scenario=normal; sample_size_label=medium	300	-0.0013700913905733	-0.0002057392856478	-0.0069234265802288	0.0040736155542815	20782.0	0.2332532613226736
y_rmse	dataset_source=WDI_agriculture; pseudo_model_source=Quantile-SFA; error_scenario=skewed; sample_size_label=large	300	0.001845633913931	0.0002046198898411	-0.0026975395489093	0.006123785242157	21672.0	0.5483953049770857
y_rmse	dataset_source=WDI_agriculture; pseudo_model_source=Quantile-SFA; error_scenario=skewed; sample_size_label=medium	300	0.0032397117152351	4.0755279745008366e-05	-0.0006320084486587	0.0077578378326536	21588.0	0.5118068634904493
y_rmse	dataset_source=WDI_agriculture; pseudo_model_source=Quantile-SFA; error_scenario=student_t; sample_size_label=large	300	0.0003313615121376	3.769628894201293e-05	-0.0019293907913614	0.0027760690397409	22207.0	0.8069294736408692
y_rmse	dataset_source=WDI_agriculture; pseudo_model_source=Quantile-SFA; error_scenario=student_t; sample_size_label=medium	300	0.0020602556523806	0.0002379817904372	-0.001166406734312	0.0052812501362287	19438.0	0.036997988868337
y_rmse	dataset_source=WDI_industry; pseudo_model_source=HQER-SFA; error_scenario=ald; sample_size_label=large	300	-0.0001746260983925	-8.637522458904145e-05	-0.0002147493035934	-0.0001403805774926	7406.0	6.2991138524857574e-24
y_rmse	dataset_source=WDI_industry; pseudo_model_source=HQER-SFA; error_scenario=ald; sample_size_label=medium	300	-0.0002073246408246	-2.096070951482232e-05	-0.0003001380009889	-0.0001184540356417	18146.0	0.0032298397025131
y_rmse	dataset_source=WDI_industry; pseudo_model_source=HQER-SFA; error_scenario=hqer_induced; sample_size_label=large	300	-4.635769020290836e-06	9.145424795825584e-06	-2.2447936187759816e-05	1.3217606172673013e-05	19880.0	0.0731563051615295

metric	group	n_pairs	mean_difference	median_difference	bootstrap_ci_lower	bootstrap_ci_upper	wilcoxon_statistic	wilcoxon_pvalue
y_rmse	dataset_source=WDI_industry; pseudo_model_source=HQER-SFA; error_scenario=hqer_induced; sample_size_label=medium	300	3.4003906481615735e-05	5.086282225114092e-05	-2.550174332605898e-05	9.363336025967776e-05	15575.0	3.244555996263272e-06
y_rmse	dataset_source=WDI_industry; pseudo_model_source=HQER-SFA; error_scenario=normal; sample_size_label=large	300	-0.000291631066462	-0.00043898604718	-0.0005801431138987	-2.9545446549277004e-06	18855.0	0.0133802865082061
y_rmse	dataset_source=WDI_industry; pseudo_model_source=HQER-SFA; error_scenario=normal; sample_size_label=medium	300	-0.0006207915931696	-0.0001035124614942	-0.0011654165341147	-7.493185646692983e-05	19738.0	0.0592565674613555
y_rmse	dataset_source=WDI_industry; pseudo_model_source=HQER-SFA; error_scenario=skewed; sample_size_label=large	300	-0.0096568457282768	-0.0086143208884142	-0.0102594236612512	-0.0090411346025956	49.0	9.988162155835289e-51
y_rmse	dataset_source=WDI_industry; pseudo_model_source=HQER-SFA; error_scenario=skewed; sample_size_label=medium	300	-0.0106973897323794	-0.0090457012721553	-0.0116499803689572	-0.0097703838769511	41.0	9.219701819861368e-51
y_rmse	dataset_source=WDI_industry; pseudo_model_source=HQER-SFA; error_scenario=student_t; sample_size_label=large	300	-0.0001447956022419	-2.9945396562910956e-05	-0.0002274432526554	-6.291703294570405e-05	17454.0	0.0006612651928233
y_rmse	dataset_source=WDI_industry; pseudo_model_source=HQER-SFA; error_scenario=student_t; sample_size_label=medium	300	-0.0003678621785399	-6.644936745241192e-05	-0.000574624311816	-0.0001962523173432	17069.0	0.0002510546779667
y_rmse	dataset_source=WDI_industry; pseudo_model_source=Quantile-SFA; error_scenario=ald; sample_size_label=large	300	-1.438687929061766e-06	-4.551445419997968e-07	-4.689091093653638e-06	1.7869245705785093e-06	22005.0	0.7048958015392736
y_rmse	dataset_source=WDI_industry; pseudo_model_source=Quantile-SFA; error_scenario=ald; sample_size_label=medium	300	-4.425585117590984e-06	-1.731419738693063e-07	-3.4644101168837334e-05	1.9908590277704577e-05	22568.0	0.9965511319788062
y_rmse	dataset_source=WDI_industry; pseudo_model_source=Quantile-SFA; error_scenario=hqer_induced; sample_size_label=large	300	2.7880610562998804e-06	1.622692118868896e-06	-1.4985264290500454e-06	7.072391958502566e-06	18238.0	0.0039291878266526
y_rmse	dataset_source=WDI_industry; pseudo_model_source=Quantile-SFA; error_scenario=hqer_induced; sample_size_label=medium	300	2.612242674871053e-05	2.242752992986929e-06	4.730059682604454e-06	5.189537640326501e-05	19373.0	0.0332533054961461
y_rmse	dataset_source=WDI_industry; pseudo_model_source=Quantile-SFA; error_scenario=normal; sample_size_label=large	300	2.51006924185108e-05	1.1004871384584505e-06	-6.403121970574638e-05	0.0001153359871626	21647.0	0.5373722143327444
y_rmse	dataset_source=WDI_industry; pseudo_model_source=Quantile-SFA; error_scenario=normal; sample_size_label=medium	300	-1.9361035276268072e-05	-7.744141992532816e-07	-0.0001776765160075	0.0001448422941038	22309.0	0.8598550648587513
y_rmse	dataset_source=WDI_industry; pseudo_model_source=Quantile-SFA; error_scenario=skewed; sample_size_label=large	300	-0.0002054412754169	-2.761853861296681e-05	-0.0003149024353111	-0.0001034424483428	16353.0	3.513785752334666e-05
y_rmse	dataset_source=WDI_industry; pseudo_model_source=Quantile-SFA; error_scenario=skewed; sample_size_label=medium	300	-0.0002176662294566	-4.413291561734811e-05	-0.0004724469798361	4.394586014446271e-05	16267.0	2.7345585744483577e-05
y_rmse	dataset_source=WDI_industry; pseudo_model_source=Quantile-SFA; error_scenario=student_t; sample_size_label=large	300	-5.019485385756214e-06	-1.897238658121747e-07	-1.874210067191741e-05	8.499080633234535e-06	22128.0	0.7665241827263213
y_rmse	dataset_source=WDI_industry; pseudo_model_source=Quantile-SFA; error_scenario=student_t; sample_size_label=medium	300	5.267828664624422e-06	-2.7273267615979258e-06	-3.236769475250372e-05	4.718798777030697e-05	21713.0	0.5667115388457795
y_rmse	dataset_source=front41Data; pseudo_model_source=HQER-SFA; error_scenario=ald; sample_size_label=medium	300	-0.0371890288195609	-0.0380708493024034	-0.0399920040109947	-0.0342160045498146	797.0	1.5714386414364314e-47
y_rmse	dataset_source=front41Data; pseudo_model_source=HQER-SFA; error_scenario=ald; sample_size_label=small	300	-0.0257592106125457	-0.0163977319240941	-0.0331527238905115	-0.0189950581416035	12333.0	9.71638681037942e-12
y_rmse	dataset_source=front41Data; pseudo_model_source=HQER-SFA; error_scenario=hqer_induced; sample_size_label=medium	300	-0.0087554397320313	-0.0079036714883863	-0.009705650468301	-0.0077954841515431	3158.0	3.848769283509739e-38
y_rmse	dataset_source=front41Data; pseudo_model_source=HQER-SFA; error_scenario=hqer_induced; sample_size_label=small	300	-0.0050951515573311	-0.0024735633601615	-0.0084134353472585	-0.0015157142252443	17217.0	0.0003669747580955
y_rmse	dataset_source=front41Data; pseudo_model_source=HQER-SFA; error_scenario=normal; sample_size_label=medium	300	0.0021841197846033	0.0007830910424725	0.0015890712026976	0.0028477599515221	12899.0	1.2408453579672689e-10
y_rmse	dataset_source=front41Data; pseudo_model_source=HQER-SFA; error_scenario=normal; sample_size_label=small	300	0.0067371172030021	0.0024530670564734	0.0046442134065977	0.009090505936138	13186.0	4.28177399391141e-10
y_rmse	dataset_source=front41Data; pseudo_model_source=HQER-SFA; error_scenario=skewed; sample_size_label=medium	300	-0.008701037610501	-0.0076270715602123	-0.0094897298738377	-0.0078887746321716	1514.0	1.4461689952361204e-44
y_rmse	dataset_source=front41Data; pseudo_model_source=HQER-SFA; error_scenario=skewed; sample_size_label=small	300	-0.0071003048480723	-0.0060239652218978	-0.009064604089571	-0.0052390536562253	11301.0	6.533136569859882e-14
y_rmse	dataset_source=front41Data; pseudo_model_source=HQER-SFA; error_scenario=student_t; sample_size_label=medium	300	3.703250603943109e-05	-3.312045393361052e-07	-6.994378604774543e-05	0.0001441837995723	22310.0	0.8603774862031808
y_rmse	dataset_source=front41Data; pseudo_model_source=HQER-SFA; error_scenario=student_t; sample_size_label=small	300	4.935849694390223e-05	0.0001716598665762	-0.0017983987292385	0.0017660823981774	18771.0	0.0114275902751115
y_rmse	dataset_source=front41Data; pseudo_model_source=Quantile-SFA; error_scenario=ald; sample_size_label=medium	300	4.369021762214006e-05	4.990597398640828e-06	-0.000231294696294	0.0002942198556019	22570.0	0.9976123182675266
y_rmse	dataset_source=front41Data; pseudo_model_source=Quantile-SFA; error_scenario=ald; sample_size_label=small	300	-0.0001516392629346	-3.1724432605906294e-07	-0.0013380676626521	0.0011750591372901	21571.0	0.5045601535508075
y_rmse	dataset_source=front41Data; pseudo_model_source=Quantile-SFA; error_scenario=hqer_induced; sample_size_label=medium	300	-7.52234907695822e-05	6.07787959247541e-06	-0.0003586728298268	0.0002320315855078	21704.0	0.5626658826920315
y_rmse	dataset_source=front41Data; pseudo_model_source=Quantile-SFA; error_scenario=hqer_induced; sample_size_label=small	300	-0.0004639327390928	-8.446090052016686e-06	-0.0012449264639408	0.0002787206788303	21575.0	0.5062603808391177
y_rmse	dataset_source=front41Data; pseudo_model_source=Quantile-SFA; error_scenario=normal; sample_size_label=medium	300	0.0002568338264137	5.7316485448100085e-06	-9.64553232285133e-05	0.0006196647266835	21123.0	0.3344176727176439
y_rmse	dataset_source=front41Data; pseudo_model_source=Quantile-SFA; error_scenario=normal; sample_size_label=small	300	-0.0002030544773763	5.054555957603735e-07	-0.0008910128453635	0.0004764614143431	22431.0	0.9239747740004504
y_rmse	dataset_source=front41Data; pseudo_model_source=Quantile-SFA; error_scenario=skewed; sample_size_label=medium	300	4.148533567031089e-06	-1.0607993857561504e-06	-7.779486901041859e-05	8.984942119781739e-05	20632.0	0.1964364542421509
y_rmse	dataset_source=front41Data; pseudo_model_source=Quantile-SFA; error_scenario=skewed; sample_size_label=small	300	0.000211644095501	-2.6051449629957424e-06	-0.0005951108453498	0.0010048227390893	21730.0	0.5743911673155322
y_rmse	dataset_source=front41Data; pseudo_model_source=Quantile-SFA; error_scenario=student_t; sample_size_label=medium	300	6.51796391051603e-05	3.460696498196192e-06	-3.5340889078581654e-05	0.0001683146977684	19803.0	0.0653203009181748
y_rmse	dataset_source=front41Data; pseudo_model_source=Quantile-SFA; error_scenario=student_t; sample_size_label=small	300	-0.0004463401915806	-3.332081133344822e-06	-0.0010289174477553	9.070278957358251e-05	19819.0	0.066888932262148
TE_RMSE	overall	18000	0.023050371721949	1.0097540997788812e-09	0.0215670624312731	0.024526306692525	64310348.0	1.023634271486158e-126
TE_RMSE	dataset_source=WDI_agriculture; pseudo_model_source=HQER-SFA; error_scenario=ald; sample_size_label=large	300	-0.0920915683481272	-0.0348896988451939	-0.1180349060376314	-0.0683721653214431	6781.0	8.390252797147122e-26
TE_RMSE	dataset_source=WDI_agriculture; pseudo_model_source=HQER-SFA; error_scenario=ald; sample_size_label=medium	300	-0.1352412909916377	-0.0344925447425477	-0.1644558249815011	-0.1080673899067598	4886.0	6.0594283473702e-32
TE_RMSE	dataset_source=WDI_agriculture; pseudo_model_source=HQER-SFA; error_scenario=hqer_induced; sample_size_label=large	300	0.3930495457139085	0.3932591634828642	0.391617211242239	0.3944040474031504	0.0	6.114136859304472e-51
TE_RMSE	dataset_source=WDI_agriculture; pseudo_model_source=HQER-SFA; error_scenario=hqer_induced; sample_size_label=medium	300	0.3935360277820591	0.3968817517996833	0.3888508739970409	0.3974551689628525	1.0	6.175750667302515e-51
TE_RMSE	dataset_source=WDI_agriculture; pseudo_model_source=HQER-SFA; error_scenario=normal; sample_size_label=large	300	0.3300308356460594	0.3309489093687828	0.3278496529619191	0.3319942493561871	0.0	6.114136859304472e-51
TE_RMSE	dataset_source=WDI_agriculture; pseudo_model_source=HQER-SFA; error_scenario=normal; sample_size_label=medium	300	0.3328897580494691	0.3360685874198236	0.329307275521368	0.3365216790621875	0.0	6.114136859304472e-51
TE_RMSE	dataset_source=WDI_agriculture; pseudo_model_source=HQER-SFA; error_scenario=skewed; sample_size_label=large	300	0.0816834063352104	0.0895303937127817	0.0764217922990813	0.086724944634847	492.0	8.048245904539049e-49
TE_RMSE	dataset_source=WDI_agriculture; pseudo_model_source=HQER-SFA; error_scenario=skewed; sample_size_label=medium	300	0.087372579646275	0.0948298510468625	0.0791956836859705	0.0957016341156724	2285.0	1.728788927294877e-41
TE_RMSE	dataset_source=WDI_agriculture; pseudo_model_source=HQER-SFA; error_scenario=student_t; sample_size_label=large	300	-0.0005464330263048	9.249827714795378e-06	-0.0023173536429694	0.0012505955978514	16568.0	6.487229157620727e-05

metric	group	n_pairs	mean_difference	median_difference	bootstrap_ci_lower	bootstrap_ci_upper	wilcoxon_statistic	wilcoxon_pvalue
TE_RMSE	dataset_source=WDI_agriculture; pseudo_model_source=HQER-SFA; error_scenario=student_t; sample_size_label=medium	300	0.0011981066329088	2.217178978120016e-05	-0.0030683118237816	0.0054041507737819	17059.0	0.0002446161128115
TE_RMSE	dataset_source=WDI_agriculture; pseudo_model_source=Quantile-SFA; error_scenario=ald; sample_size_label=large	300	-3.972958037103402e-06	1.3027145473049062e-05	-9.698056825754792e-05	8.605206769631621e-05	20894.0	0.2637635619351184
TE_RMSE	dataset_source=WDI_agriculture; pseudo_model_source=Quantile-SFA; error_scenario=ald; sample_size_label=medium	300	0.001159411908023	-6.25885210696342e-06	-0.0003263858553498	0.0036821365249549	21723.0	0.5712230054796337
TE_RMSE	dataset_source=WDI_agriculture; pseudo_model_source=Quantile-SFA; error_scenario=hqer_induced; sample_size_label=large	300	-6.38261723559062e-05	2.282092737448949e-06	-0.0001508084991989	1.019306058001028e-05	22106.0	0.7553790761844071
TE_RMSE	dataset_source=WDI_agriculture; pseudo_model_source=Quantile-SFA; error_scenario=hqer_induced; sample_size_label=medium	300	6.900788343317652e-05	4.065324651890412e-05	-0.000238415300125	0.0003525657028925	19883.0	0.0734765324046218
TE_RMSE	dataset_source=WDI_agriculture; pseudo_model_source=Quantile-SFA; error_scenario=normal; sample_size_label=large	300	-0.0020395380267571	-0.0003648694324642	-0.0052819705427638	0.0001051574027728	20539.0	0.1758587830903553
TE_RMSE	dataset_source=WDI_agriculture; pseudo_model_source=Quantile-SFA; error_scenario=normal; sample_size_label=medium	300	-0.0006548270000153	-0.0005570955916595	-0.0057920025797182	0.0040011532303769	19013.0	0.0178645755532563
TE_RMSE	dataset_source=WDI_agriculture; pseudo_model_source=Quantile-SFA; error_scenario=skewed; sample_size_label=large	300	-0.0009088131875015	-0.0013233981979868	-0.0057651729858863	0.0043823902485451	17669.0	0.0011055930137834
TE_RMSE	dataset_source=WDI_agriculture; pseudo_model_source=Quantile-SFA; error_scenario=skewed; sample_size_label=medium	300	-0.0030541742351566	-7.251275853620287e-05	-0.0059699960515793	-0.0008481046960449	20140.0	0.1054573377782093
TE_RMSE	dataset_source=WDI_agriculture; pseudo_model_source=Quantile-SFA; error_scenario=student_t; sample_size_label=large	300	-0.0033835470134133	-0.0017971568379493	-0.0086409902955199	0.001101782369601	17552.0	0.0008378305719853
TE_RMSE	dataset_source=WDI_agriculture; pseudo_model_source=Quantile-SFA; error_scenario=student_t; sample_size_label=medium	300	-0.0006363824465866	-7.70449801027473e-05	-0.0048257463492296	0.0035449908472232	21232.0	0.3719812183672014
TE_RMSE	dataset_source=WDI_industry; pseudo_model_source=HQER-SFA; error_scenario=ald; sample_size_label=large	300	6.950176294438319e-11	2.6721564891363236e-10	-6.468871840718468e-10	7.056050126113404e-10	20817.0	0.2425063016669958
TE_RMSE	dataset_source=WDI_industry; pseudo_model_source=HQER-SFA; error_scenario=ald; sample_size_label=medium	300	-9.921582853668806e-10	-6.519434506135902e-10	-2.092291268174633e-09	-1.1429118363665249e-10	19634.0	0.0505305222691591
TE_RMSE	dataset_source=WDI_industry; pseudo_model_source=HQER-SFA; error_scenario=hqer_induced; sample_size_label=large	300	7.418857943137605e-10	9.430641007696083e-10	3.5207821926011105e-10	1.102925703133292e-09	15235.0	1.056565177742579e-06
TE_RMSE	dataset_source=WDI_industry; pseudo_model_source=HQER-SFA; error_scenario=hqer_induced; sample_size_label=medium	300	9.289639676483368e-10	6.84085699545478e-10	3.5173242910578834e-10	1.5302094263361027e-09	18060.0	0.0026806921296976
TE_RMSE	dataset_source=WDI_industry; pseudo_model_source=HQER-SFA; error_scenario=normal; sample_size_label=large	300	2.4743884661955483e-08	2.5009886899310385e-08	2.2217560744420527e-08	2.7164503817551865e-08	1815.0	2.3724994817737075e-43
TE_RMSE	dataset_source=WDI_industry; pseudo_model_source=HQER-SFA; error_scenario=normal; sample_size_label=medium	300	1.892504881953241e-08	1.3114853451498544e-08	1.5511909347898366e-08	2.2439790349616737e-08	7747.0	6.183191192434286e-23
TE_RMSE	dataset_source=WDI_industry; pseudo_model_source=HQER-SFA; error_scenario=skewed; sample_size_label=large	300	2.91117986382534e-08	1.7240759301109798e-08	2.3960792160612523e-08	3.5200827123587785e-08	6674.0	3.9376165121914976e-26
TE_RMSE	dataset_source=WDI_industry; pseudo_model_source=HQER-SFA; error_scenario=skewed; sample_size_label=medium	300	1.8402544735794204e-08	6.479633513772898e-10	1.1992492785613551e-08	2.6625733677013272e-08	18473.0	0.0063813261558519
TE_RMSE	dataset_source=WDI_industry; pseudo_model_source=HQER-SFA; error_scenario=student_t; sample_size_label=large	300	-6.100190477244712e-09	-4.596871952533554e-09	-7.627215042150965e-09	-4.593848593778653e-09	8271.0	1.872290134958495e-21
TE_RMSE	dataset_source=WDI_industry; pseudo_model_source=HQER-SFA; error_scenario=student_t; sample_size_label=medium	300	-6.7171457017340774e-09	-4.789400050164705e-09	-8.761823061622976e-09	-4.7664747107262556e-09	12960.0	1.6193520942160228e-10
TE_RMSE	dataset_source=WDI_industry; pseudo_model_source=Quantile-SFA; error_scenario=ald; sample_size_label=large	300	-1.2201641965322214e-10	-9.39560235169168e-12	-3.257177002182153e-10	8.987336963123721e-11	21543.0	0.4927433622718821
TE_RMSE	dataset_source=WDI_industry; pseudo_model_source=Quantile-SFA; error_scenario=ald; sample_size_label=medium	300	3.1339977046388835e-10	1.498679999545338e-10	-1.4700252716088234e-10	7.870396798592925e-10	20233.0	0.1194449006090014
TE_RMSE	dataset_source=WDI_industry; pseudo_model_source=Quantile-SFA; error_scenario=hqer_induced; sample_size_label=large	300	9.942105009634143e-15	2.6026651320232297e-11	-1.732262102924093e-10	1.7598523035141267e-10	22345.0	0.8786991298249158
TE_RMSE	dataset_source=WDI_industry; pseudo_model_source=Quantile-SFA; error_scenario=hqer_induced; sample_size_label=medium	300	2.1006432242981488e-10	3.880649274146109e-12	-1.7055081238254167e-10	5.775297795568984e-10	22190.0	0.7981869845406863
TE_RMSE	dataset_source=WDI_industry; pseudo_model_source=Quantile-SFA; error_scenario=normal; sample_size_label=large	300	8.742772771945055e-11	4.4209200536493576e-11	-1.1478450995452738e-10	2.814682182909408e-10	22260.0	0.8343359212379892
TE_RMSE	dataset_source=WDI_industry; pseudo_model_source=Quantile-SFA; error_scenario=normal; sample_size_label=medium	300	1.4953978994906395e-10	1.1215899910210768e-10	-4.53765040006257e-10	7.15874394876618e-10	20510.0	0.1697829531311139
TE_RMSE	dataset_source=WDI_industry; pseudo_model_source=Quantile-SFA; error_scenario=skewed; sample_size_label=large	300	2.2213967882864227e-09	1.0641794494226087e-09	8.64909735859287e-10	3.7759523902593816e-09	17345.0	0.0005058479859775
TE_RMSE	dataset_source=WDI_industry; pseudo_model_source=Quantile-SFA; error_scenario=skewed; sample_size_label=medium	300	1.2993510365572058e-09	7.152430210599636e-11	-1.0286013380573243e-09	3.900227870947195e-09	21571.0	0.5045601535508075
TE_RMSE	dataset_source=WDI_industry; pseudo_model_source=Quantile-SFA; error_scenario=student_t; sample_size_label=large	300	-3.0588025620494524e-10	-2.2087629815426e-10	-3.897677073764921e-10	-2.2654232334495815e-10	9130.0	3.8688277252102376e-19
TE_RMSE	dataset_source=WDI_industry; pseudo_model_source=Quantile-SFA; error_scenario=student_t; sample_size_label=medium	300	-3.112164270157221e-10	-2.928819495956469e-10	-5.746085893051112e-10	-5.7646404069515646e-11	19141.0	0.0224131410140629
TE_RMSE	dataset_source=front41Data; pseudo_model_source=HQER-SFA; error_scenario=ald; sample_size_label=medium	300	0.0002893731341777	9.486617498499772e-10	2.1884598755463984e-10	0.0008681180200654	17202.0	0.0003532707778404
TE_RMSE	dataset_source=front41Data; pseudo_model_source=HQER-SFA; error_scenario=ald; sample_size_label=small	300	7.979513019090722e-10	4.776634990322215e-10	-2.227561535051575e-09	3.739130523646867e-09	21906.0	0.6566410614036347
TE_RMSE	dataset_source=front41Data; pseudo_model_source=HQER-SFA; error_scenario=hqer_induced; sample_size_label=medium	300	1.3833800518424829e-08	1.4066171649326777e-08	1.2539505483791756e-08	1.5049052685677778e-08	1622.0	3.9641753450900097e-44
TE_RMSE	dataset_source=front41Data; pseudo_model_source=HQER-SFA; error_scenario=hqer_induced; sample_size_label=small	300	1.7885047980450522e-08	1.3242433448412694e-08	1.2265458190403173e-08	2.3462909686126036e-08	13166.0	3.9322446667594087e-10
TE_RMSE	dataset_source=front41Data; pseudo_model_source=HQER-SFA; error_scenario=normal; sample_size_label=medium	300	2.5371215088253648e-08	2.1345933000349596e-08	2.1844854444255253e-08	2.9230810323946716e-08	4957.0	1.05894355078768e-31
TE_RMSE	dataset_source=front41Data; pseudo_model_source=HQER-SFA; error_scenario=normal; sample_size_label=small	300	3.86772218551971e-08	3.0734488502826014e-08	2.788739584450029e-08	5.028972161555892e-08	11952.0	1.6176615508552774e-12
TE_RMSE	dataset_source=front41Data; pseudo_model_source=HQER-SFA; error_scenario=skewed; sample_size_label=medium	300	1.6703578680811463e-08	5.666476003696186e-10	6.228836341843486e-09	2.7478507734141818e-08	20463.0	0.1602721743371558
TE_RMSE	dataset_source=front41Data; pseudo_model_source=HQER-SFA; error_scenario=skewed; sample_size_label=small	300	0.0003684310964766	-4.892170047537947e-09	2.16792702958761e-09	0.0011052649802831	21044.0	0.3087781395198553
TE_RMSE	dataset_source=front41Data; pseudo_model_source=HQER-SFA; error_scenario=student_t; sample_size_label=medium	300	-3.3846031148703403e-09	-2.549768601117774e-09	-5.871445089488299e-09	-1.2852689875633012e-09	16006.0	1.2534283176724403e-05
TE_RMSE	dataset_source=front41Data; pseudo_model_source=HQER-SFA; error_scenario=student_t; sample_size_label=small	300	-1.6210028602747785e-08	-6.660693954718688e-09	-3.6507394057818936e-08	4.655655185501734e-09	20063.0	0.0948880053130008
TE_RMSE	dataset_source=front41Data; pseudo_model_source=Quantile-SFA; error_scenario=ald; sample_size_label=medium	300	9.8969192528775e-11	2.9065675213879594e-10	-1.0123168766385318e-09	1.222865663771365e-09	21771.0	0.5931125314002996
TE_RMSE	dataset_source=front41Data; pseudo_model_source=Quantile-SFA; error_scenario=ald; sample_size_label=small	300	2.385692369997564e-09	8.658460493721211e-10	-4.137253768985981e-09	8.213704516198243e-09	19869.0	0.0719918936166513
TE_RMSE	dataset_source=front41Data; pseudo_model_source=Quantile-SFA; error_scenario=hqer_induced; sample_size_label=medium	300	-2.670157171526706e-10	4.945739824624429e-11	-1.484853677032512e-09	9.027574608573101e-10	22230.0	0.818795848232952
TE_RMSE	dataset_source=front41Data; pseudo_model_source=Quantile-SFA; error_scenario=hqer_induced; sample_size_label=small	300	1.3636524910191443e-09	-2.7551484704790847e-10	-4.898662431930198e-09	7.537554395787258e-09	22550.0	0.9870009551541612
TE_RMSE	dataset_source=front41Data; pseudo_model_source=Quantile-SFA; error_scenario=normal; sample_size_label=medium	300	-6.554072494215543e-11	2.053568998877653e-10	-1.2381400677456455e-09	9.410434280047891e-10	21528.0	0.4864744910927828
TE_RMSE	dataset_source=front41Data; pseudo_model_source=Quantile-SFA; error_scenario=normal; sample_size_label=small	300	3.958946327905592e-09	9.219892488365743e-10	-5.0151151815306865e-09	1.1843641808168048e-08	21034.0	0.305627847869512

metric	group	n_pairs	mean_difference	median_difference	bootstrap_ci_lower	bootstrap_ci_upper	wilcoxon_statistic	wilcoxon_pvalue
TE_RMSE	dataset_source=front41Data; pseudo_model_source=Quantile-SFA; error_scenario=skewed; sample_size_label=medium	300	-1.91296510345292e-10	1.3312544989985129e-10	-2.8024630745627358e-09	2.47456324562924e-09	22238.0	0.8229331807089993
TE_RMSE	dataset_source=front41Data; pseudo_model_source=Quantile-SFA; error_scenario=skewed; sample_size_label=small	300	5.484509343935129e-09	-7.338809004941992e-10	-1.883443098853188e-08	3.329143509274607e-08	22252.0	0.8301853043352345
TE_RMSE	dataset_source=front41Data; pseudo_model_source=Quantile-SFA; error_scenario=student_t; sample_size_label=medium	300	-3.111167997763934e-10	-6.136169992260188e-10	-1.1068337839919307e-09	4.9839900303876e-10	19000.0	0.017451335433555
TE_RMSE	dataset_source=front41Data; pseudo_model_source=Quantile-SFA; error_scenario=student_t; sample_size_label=small	300	4.107639396642993e-09	-6.188153999636681e-10	-1.4493002696141605e-08	2.678021871737636e-08	22180.0	0.7930560649355277
TE_rank_corr	overall	18000	0.0022273623162616	0.0004365569962254	0.0018012156719444	0.0026346865070891	70564179.0	1.0637198803386404e-50
TE_rank_corr	dataset_source=WDI_agriculture; pseudo_model_source=HQER-SFA; error_scenario=ald; sample_size_label=large	300	-0.0032794510477548	-0.0030309238086661	-0.0045466708036842	-0.0019551536776612	15638.0	3.972749447988575e-06
TE_rank_corr	dataset_source=WDI_agriculture; pseudo_model_source=HQER-SFA; error_scenario=ald; sample_size_label=medium	300	7.867401544772131e-05	-0.0007936881456033	-0.0027309416922604	0.003026653567126	22560.0	0.9923064666021126
TE_rank_corr	dataset_source=WDI_agriculture; pseudo_model_source=HQER-SFA; error_scenario=hqer_induced; sample_size_label=large	300	0.0009631333228383	0.0008842519790197	0.0007909955366726	0.001162646582625	9212.0	6.327937836953204e-19
TE_rank_corr	dataset_source=WDI_agriculture; pseudo_model_source=HQER-SFA; error_scenario=hqer_induced; sample_size_label=medium	300	0.0008333052723953	0.0007728419550977	0.0004670531850067	0.0012307576371571	16492.0	5.234788093192218e-05
TE_rank_corr	dataset_source=WDI_agriculture; pseudo_model_source=HQER-SFA; error_scenario=normal; sample_size_label=large	300	0.0020773519888974	0.0018070812787172	0.0017147585483537	0.0024706766324872	8567.0	1.218963335899485e-20
TE_rank_corr	dataset_source=WDI_agriculture; pseudo_model_source=HQER-SFA; error_scenario=normal; sample_size_label=medium	300	0.0026343240407833	0.0021586577861891	0.0018388152474704	0.0034934264628792	13110.0	3.0951944690795225e-10
TE_rank_corr	dataset_source=WDI_agriculture; pseudo_model_source=HQER-SFA; error_scenario=skewed; sample_size_label=large	300	0.0051786373664706	0.0047089677093988	0.0036238903976605	0.0068466197701349	13834.0	6.155741058831097e-09
TE_rank_corr	dataset_source=WDI_agriculture; pseudo_model_source=HQER-SFA; error_scenario=skewed; sample_size_label=medium	300	0.0064735832396763	0.0058425686284509	0.003651489365856	0.0096728182648918	16767.0	0.000112456593823
TE_rank_corr	dataset_source=WDI_agriculture; pseudo_model_source=HQER-SFA; error_scenario=student_t; sample_size_label=large	300	0.0024401148295022	0.0017413592426617	0.0015941833096772	0.0033650161587413	15272.0	1.1966314962500444e-06
TE_rank_corr	dataset_source=WDI_agriculture; pseudo_model_source=HQER-SFA; error_scenario=student_t; sample_size_label=medium	300	0.003805941539627	0.0032399314880015	0.0021700360632897	0.005641399939634	16627.0	7.64982984203895e-05
TE_rank_corr	dataset_source=WDI_agriculture; pseudo_model_source=Quantile-SFA; error_scenario=ald; sample_size_label=large	300	-0.0003299176923786	-0.0003892337286723	-0.0004456624753884	-0.0002147445667656	15160.0	8.194596048994518e-07
TE_rank_corr	dataset_source=WDI_agriculture; pseudo_model_source=Quantile-SFA; error_scenario=ald; sample_size_label=medium	300	0.0007818570235225	-0.0001367972572933	-0.0001900288267763	0.0024687077772856	21873.0	0.6408572348120046
TE_rank_corr	dataset_source=WDI_agriculture; pseudo_model_source=Quantile-SFA; error_scenario=hqer_induced; sample_size_label=large	300	0.0001440349509952	0.0001305116252673	3.536684401000173e-05	0.000252144875979	18709.0	0.0101529999776339
TE_rank_corr	dataset_source=WDI_agriculture; pseudo_model_source=Quantile-SFA; error_scenario=hqer_induced; sample_size_label=medium	300	0.0008159309705276	0.0003158483279096	0.0004741558105611	0.0011839381273416	16976.0	0.0001968484186664
TE_rank_corr	dataset_source=WDI_agriculture; pseudo_model_source=Quantile-SFA; error_scenario=normal; sample_size_label=large	300	8.579165192356093e-05	9.530470546298409e-05	5.005862861553025e-05	0.000121925874507	12747.0	6.34691824760484e-11
TE_rank_corr	dataset_source=WDI_agriculture; pseudo_model_source=Quantile-SFA; error_scenario=normal; sample_size_label=medium	300	0.0018666292011574	9.824533717389672e-05	1.5714086023394455e-05	0.0054158482459361	18738.0	0.0107325188167282
TE_rank_corr	dataset_source=WDI_agriculture; pseudo_model_source=Quantile-SFA; error_scenario=skewed; sample_size_label=large	300	0.0015039870681265	0.0001623828322049	0.0001521389097699	0.0037245603332947	9450.0	2.5955944102117014e-18
TE_rank_corr	dataset_source=WDI_agriculture; pseudo_model_source=Quantile-SFA; error_scenario=skewed; sample_size_label=medium	300	0.000819569280932	0.0001747932224613	0.0004660846295755	0.0012045129235992	15014.0	4.962277594057618e-07
TE_rank_corr	dataset_source=WDI_agriculture; pseudo_model_source=Quantile-SFA; error_scenario=student_t; sample_size_label=large	300	0.0001518852353814	-0.000109088780731	-0.0005039479768128	0.0011595553143759	8491.0	7.562709374714457e-21
TE_rank_corr	dataset_source=WDI_agriculture; pseudo_model_source=Quantile-SFA; error_scenario=student_t; sample_size_label=medium	300	0.0011559952915574	-2.796434491258992e-05	0.0005825578299628	0.0018018570444262	22177.0	0.7915185234486262
TE_rank_corr	dataset_source=WDI_industry; pseudo_model_source=HQER-SFA; error_scenario=ald; sample_size_label=large	300	-0.00378284998763	-0.0039468145761659	-0.0050807717589489	-0.0026236755721552	13368.0	9.21977504330648e-10
TE_rank_corr	dataset_source=WDI_industry; pseudo_model_source=HQER-SFA; error_scenario=ald; sample_size_label=medium	300	-0.0041549251054616	-0.0041216473757756	-0.0065624743513249	-0.0014774381674535	17401.0	0.0005808546289285
TE_rank_corr	dataset_source=WDI_industry; pseudo_model_source=HQER-SFA; error_scenario=hqer_induced; sample_size_label=large	300	-0.0031869577305434	-0.0034419137820227	-0.0042621896719499	-0.0020709974795946	14415.0	5.7597636604620946e-08
TE_rank_corr	dataset_source=WDI_industry; pseudo_model_source=HQER-SFA; error_scenario=hqer_induced; sample_size_label=medium	300	-0.0022739286987072	-0.0024583136861449	-0.0043070104817334	-0.0002590528335168	19614.0	0.0489823252775158
TE_rank_corr	dataset_source=WDI_industry; pseudo_model_source=HQER-SFA; error_scenario=normal; sample_size_label=large	300	0.0140636473962071	0.0129671274186366	0.0129779938567291	0.0151365249758783	442.0	4.925284518695849e-49
TE_rank_corr	dataset_source=WDI_industry; pseudo_model_source=HQER-SFA; error_scenario=normal; sample_size_label=medium	300	0.0122733638605541	0.0121841402923796	0.0103560609486775	0.0142012081146076	6911.0	2.0893182648599428e-25
TE_rank_corr	dataset_source=WDI_industry; pseudo_model_source=HQER-SFA; error_scenario=skewed; sample_size_label=large	300	-0.0068439257900555	-0.008165945055656	-0.0085690882002458	-0.0049257450884922	12054.0	2.630381092875396e-12
TE_rank_corr	dataset_source=WDI_industry; pseudo_model_source=HQER-SFA; error_scenario=skewed; sample_size_label=medium	300	-0.0063207306569755	-0.0057479122408609	-0.0099205222528443	-0.0027272323620695	17136.0	0.0002984676567171
TE_rank_corr	dataset_source=WDI_industry; pseudo_model_source=HQER-SFA; error_scenario=student_t; sample_size_label=large	300	0.0053489394423622	0.0044375822978215	0.0043998048422106	0.0062551769515297	7663.0	3.53931174272432e-23
TE_rank_corr	dataset_source=WDI_industry; pseudo_model_source=HQER-SFA; error_scenario=student_t; sample_size_label=medium	300	0.0060760235334527	0.0053814361224096	0.004381073328129	0.0077945735080242	13933.0	9.103673517280953e-09
TE_rank_corr	dataset_source=WDI_industry; pseudo_model_source=Quantile-SFA; error_scenario=ald; sample_size_label=large	300	-0.0051555663408909	-0.0048787693212863	-0.0063126243811764	-0.0041213127743899	10364.0	4.660452043545022e-16
TE_rank_corr	dataset_source=WDI_industry; pseudo_model_source=Quantile-SFA; error_scenario=ald; sample_size_label=medium	300	-0.0025752221168802	-0.0027136438164242	-0.0047139041545421	-0.0003670605345531	19367.0	0.0329245975571845
TE_rank_corr	dataset_source=WDI_industry; pseudo_model_source=Quantile-SFA; error_scenario=hqer_induced; sample_size_label=large	300	-0.0028439326693266	-0.0029866939462664	-0.0039198373588325	-0.0017684841604852	15620.0	3.7500886376221695e-06
TE_rank_corr	dataset_source=WDI_industry; pseudo_model_source=Quantile-SFA; error_scenario=hqer_induced; sample_size_label=medium	300	-0.002672727848011	-0.0040275151713633	-0.0048880197438664	-0.0005294904989352	18909.0	0.0147862047991478
TE_rank_corr	dataset_source=WDI_industry; pseudo_model_source=Quantile-SFA; error_scenario=normal; sample_size_label=large	300	0.0147523300241696	0.0135691880108147	0.0137151821511955	0.0158455580917323	396.0	3.1318492065907637e-49
TE_rank_corr	dataset_source=WDI_industry; pseudo_model_source=Quantile-SFA; error_scenario=normal; sample_size_label=medium	300	0.0157461662061488	0.0157022976876783	0.0138436556860611	0.0176876811037286	3882.0	1.7830339450349791e-35
TE_rank_corr	dataset_source=WDI_industry; pseudo_model_source=Quantile-SFA; error_scenario=skewed; sample_size_label=large	300	-0.0073282923491674	-0.0097332139041609	-0.0095861521601117	-0.0049531120120407	11820.0	8.565374301461972e-13
TE_rank_corr	dataset_source=WDI_industry; pseudo_model_source=Quantile-SFA; error_scenario=skewed; sample_size_label=medium	300	-0.0068610783742097	-0.0080937547825519	-0.0104213984090492	-0.0030187245251809	16908.0	0.0001643977590188
TE_rank_corr	dataset_source=WDI_industry; pseudo_model_source=Quantile-SFA; error_scenario=student_t; sample_size_label=large	300	0.0067314161617179	0.0067734850417821	0.0057667313635599	0.0078053993815686	6201.0	1.308492346403012e-27
TE_rank_corr	dataset_source=WDI_industry; pseudo_model_source=Quantile-SFA; error_scenario=student_t; sample_size_label=medium	300	0.0045549558430211	0.0042339598143084	0.002809961593827	0.0063229340370578	15681.0	4.557110078253001e-06
TE_rank_corr	dataset_source=front41Data; pseudo_model_source=HQER-SFA; error_scenario=ald; sample_size_label=medium	300	-0.0075417721927749	-0.0074457316457398	-0.0100571574840788	-0.0048068214124334	14318.0	4.0055085213726074e-08
TE_rank_corr	dataset_source=front41Data; pseudo_model_source=HQER-SFA; error_scenario=ald; sample_size_label=small	300	-0.0054918275184668	-0.0024561825961538	-0.0130471303313357	0.0018577002065014	20731.0	0.2202229160604253

metric	group	n_pairs	mean_difference	median_difference	bootstrap_ci_lower	bootstrap_ci_upper	wilcoxon_statistic	wilcoxon_pvalue
TE_rank_corr	dataset_source=front41Data; pseudo_model_source=HQER-SFA; error_scenario=hqer_induced; sample_size_label=medium	300	0.0065906223373502	0.0070088341339172	0.0048320849848317	0.008172687105471	12376.0	1.18484722295009e-11
TE_rank_corr	dataset_source=front41Data; pseudo_model_source=HQER-SFA; error_scenario=hqer_induced; sample_size_label=small	300	0.0030783772142094	0.0043048780503981	-0.0022270642332801	0.0077716366523093	20162.0	0.1086428559697299
TE_rank_corr	dataset_source=front41Data; pseudo_model_source=HQER-SFA; error_scenario=normal; sample_size_label=medium	300	0.0138098235804826	0.0122953257891102	0.0118728117899578	0.0156812305156416	5315.0	1.7087492231694508e-30
TE_rank_corr	dataset_source=front41Data; pseudo_model_source=HQER-SFA; error_scenario=normal; sample_size_label=small	300	0.0156004215766621	0.0134864363467863	0.0107366587951628	0.02023651011158	13889.0	7.654482890141129e-09
TE_rank_corr	dataset_source=front41Data; pseudo_model_source=HQER-SFA; error_scenario=skewed; sample_size_label=medium	300	-0.0070889255500374	-0.0069440901720697	-0.0108884234678208	-0.0035915095351221	16543.0	6.0468781567973295e-05
TE_rank_corr	dataset_source=front41Data; pseudo_model_source=HQER-SFA; error_scenario=skewed; sample_size_label=small	300	0.0059978291837478	0.008069271913576	-0.0032212531076961	0.0158783075930796	20834.0	0.2470925849158998
TE_rank_corr	dataset_source=front41Data; pseudo_model_source=HQER-SFA; error_scenario=student_t; sample_size_label=medium	300	0.0061186687854572	0.0051899926689939	0.0041338337883733	0.0080934385573513	14187.0	2.4368548220574483e-08
TE_rank_corr	dataset_source=front41Data; pseudo_model_source=HQER-SFA; error_scenario=student_t; sample_size_label=small	300	0.0062295187495572	0.0012468329884235	0.0005349585741693	0.0121781993468475	20545.0	0.1771358510554002
TE_rank_corr	dataset_source=front41Data; pseudo_model_source=Quantile-SFA; error_scenario=ald; sample_size_label=medium	300	-0.0018361288867149	-0.0007788430375314	-0.0033710208361747	-0.0003737048503003	19718.0	0.0574875984649828
TE_rank_corr	dataset_source=front41Data; pseudo_model_source=Quantile-SFA; error_scenario=ald; sample_size_label=small	300	0.0026119443301752	0.0026083417335071	-0.0019652471863538	0.007489983558224	20901.0	0.2657578917765782
TE_rank_corr	dataset_source=front41Data; pseudo_model_source=Quantile-SFA; error_scenario=hqer_induced; sample_size_label=medium	300	0.0026728855723869	0.0027386706927531	0.0011601094040507	0.0042077940244743	17691.0	0.0011640365195859
TE_rank_corr	dataset_source=front41Data; pseudo_model_source=Quantile-SFA; error_scenario=hqer_induced; sample_size_label=small	300	0.0003664567433507	-0.0002994921643827	-0.0043676167669486	0.0054854348442593	22381.0	0.8976121681946732
TE_rank_corr	dataset_source=front41Data; pseudo_model_source=Quantile-SFA; error_scenario=normal; sample_size_label=medium	300	0.0158327960685964	0.0149342172428359	0.0141006522556095	0.0176153808599473	3221.0	6.626269652848489e-38
TE_rank_corr	dataset_source=front41Data; pseudo_model_source=Quantile-SFA; error_scenario=normal; sample_size_label=small	300	0.0123857037654805	0.0085837862455815	0.0073173243266267	0.0174331029835107	16242.0	2.540849044156716e-05
TE_rank_corr	dataset_source=front41Data; pseudo_model_source=Quantile-SFA; error_scenario=skewed; sample_size_label=medium	300	-0.0087444581675615	-0.0093248491339111	-0.0119674583747024	-0.005137197684643	15258.0	1.1416516112453206e-06
TE_rank_corr	dataset_source=front41Data; pseudo_model_source=Quantile-SFA; error_scenario=skewed; sample_size_label=small	300	0.0022480920922218	-0.0032630000515934	-0.006857041814167	0.0119216587786019	21793.0	0.6032720402272262
TE_rank_corr	dataset_source=front41Data; pseudo_model_source=Quantile-SFA; error_scenario=student_t; sample_size_label=medium	300	0.0081803054569249	0.0076132093293094	0.0064464560120084	0.0100590565612921	10873.0	7.164197153037136e-15
TE_rank_corr	dataset_source=front41Data; pseudo_model_source=Quantile-SFA; error_scenario=student_t; sample_size_label=small	300	0.0088733234852459	0.0085399219507839	0.0038401000562187	0.0138934275772256	17285.0	0.0004355655497647

Panel 2 of 3

Columns shown: `metric, group, wilcoxon_method, n_positive, n_negative, n_zero, positive_difference_direction`.

metric	group	wilcoxon_method	n_positive	n_negative	n_zero	positive_difference_direction
beta_error	overall	Wilcoxon signed-rank normal approximation	8658	9342	0	Positive values favor HQER-SFA.
beta_error	dataset_source=WDI_agriculture; pseudo_model_source=HQER-SFA; error_scenario=ald; sample_size_label=large	Wilcoxon signed-rank normal approximation	83	217	0	Positive values favor HQER-SFA.
beta_error	dataset_source=WDI_agriculture; pseudo_model_source=HQER-SFA; error_scenario=ald; sample_size_label=medium	Wilcoxon signed-rank normal approximation	81	219	0	Positive values favor HQER-SFA.
beta_error	dataset_source=WDI_agriculture; pseudo_model_source=HQER-SFA; error_scenario=hqer_induced; sample_size_label=large	Wilcoxon signed-rank normal approximation	300	0	0	Positive values favor HQER-SFA.
beta_error	dataset_source=WDI_agriculture; pseudo_model_source=HQER-SFA; error_scenario=hqer_induced; sample_size_label=medium	Wilcoxon signed-rank normal approximation	297	3	0	Positive values favor HQER-SFA.
beta_error	dataset_source=WDI_agriculture; pseudo_model_source=HQER-SFA; error_scenario=normal; sample_size_label=large	Wilcoxon signed-rank normal approximation	300	0	0	Positive values favor HQER-SFA.
beta_error	dataset_source=WDI_agriculture; pseudo_model_source=HQER-SFA; error_scenario=normal; sample_size_label=medium	Wilcoxon signed-rank normal approximation	289	11	0	Positive values favor HQER-SFA.
beta_error	dataset_source=WDI_agriculture; pseudo_model_source=HQER-SFA; error_scenario=skewed; sample_size_label=large	Wilcoxon signed-rank normal approximation	6	294	0	Positive values favor HQER-SFA.
beta_error	dataset_source=WDI_agriculture; pseudo_model_source=HQER-SFA; error_scenario=skewed; sample_size_label=medium	Wilcoxon signed-rank normal approximation	20	280	0	Positive values favor HQER-SFA.
beta_error	dataset_source=WDI_agriculture; pseudo_model_source=HQER-SFA; error_scenario=student_t; sample_size_label=large	Wilcoxon signed-rank normal approximation	159	141	0	Positive values favor HQER-SFA.
beta_error	dataset_source=WDI_agriculture; pseudo_model_source=HQER-SFA; error_scenario=student_t; sample_size_label=medium	Wilcoxon signed-rank normal approximation	161	139	0	Positive values favor HQER-SFA.
beta_error	dataset_source=WDI_agriculture; pseudo_model_source=Quantile-SFA; error_scenario=ald; sample_size_label=large	Wilcoxon signed-rank normal approximation	149	151	0	Positive values favor HQER-SFA.
beta_error	dataset_source=WDI_agriculture; pseudo_model_source=Quantile-SFA; error_scenario=ald; sample_size_label=medium	Wilcoxon signed-rank normal approximation	169	131	0	Positive values favor HQER-SFA.
beta_error	dataset_source=WDI_agriculture; pseudo_model_source=Quantile-SFA; error_scenario=hqer_induced; sample_size_label=large	Wilcoxon signed-rank normal approximation	157	143	0	Positive values favor HQER-SFA.
beta_error	dataset_source=WDI_agriculture; pseudo_model_source=Quantile-SFA; error_scenario=hqer_induced; sample_size_label=medium	Wilcoxon signed-rank normal approximation	167	133	0	Positive values favor HQER-SFA.
beta_error	dataset_source=WDI_agriculture; pseudo_model_source=Quantile-SFA; error_scenario=normal; sample_size_label=large	Wilcoxon signed-rank normal approximation	146	154	0	Positive values favor HQER-SFA.
beta_error	dataset_source=WDI_agriculture; pseudo_model_source=Quantile-SFA; error_scenario=normal; sample_size_label=medium	Wilcoxon signed-rank normal approximation	130	170	0	Positive values favor HQER-SFA.
beta_error	dataset_source=WDI_agriculture; pseudo_model_source=Quantile-SFA; error_scenario=skewed; sample_size_label=large	Wilcoxon signed-rank normal approximation	164	136	0	Positive values favor HQER-SFA.
beta_error	dataset_source=WDI_agriculture; pseudo_model_source=Quantile-SFA; error_scenario=skewed; sample_size_label=medium	Wilcoxon signed-rank normal approximation	155	145	0	Positive values favor HQER-SFA.
beta_error	dataset_source=WDI_agriculture; pseudo_model_source=Quantile-SFA; error_scenario=student_t; sample_size_label=large	Wilcoxon signed-rank normal approximation	147	153	0	Positive values favor HQER-SFA.
beta_error	dataset_source=WDI_agriculture; pseudo_model_source=Quantile-SFA; error_scenario=student_t; sample_size_label=medium	Wilcoxon signed-rank normal approximation	157	143	0	Positive values favor HQER-SFA.
beta_error	dataset_source=WDI_industry; pseudo_model_source=HQER-SFA; error_scenario=ald; sample_size_label=large	Wilcoxon signed-rank normal approximation	147	153	0	Positive values favor HQER-SFA.

metric	group	wilcoxon_method	n_positive	n_negative	n_zero	positive_difference_direction
beta_error	dataset_source=WDI_industry; pseudo_model_source=HQER-SFA; error_scenario=ald; sample_size_label=medium	Wilcoxon signed-rank normal approximation	165	135	0	Positive values favor HQER-SFA.
beta_error	dataset_source=WDI_industry; pseudo_model_source=HQER-SFA; error_scenario=hqer_induced; sample_size_label=large	Wilcoxon signed-rank normal approximation	207	93	0	Positive values favor HQER-SFA.
beta_error	dataset_source=WDI_industry; pseudo_model_source=HQER-SFA; error_scenario=hqer_induced; sample_size_label=medium	Wilcoxon signed-rank normal approximation	161	139	0	Positive values favor HQER-SFA.
beta_error	dataset_source=WDI_industry; pseudo_model_source=HQER-SFA; error_scenario=normal; sample_size_label=large	Wilcoxon signed-rank normal approximation	176	124	0	Positive values favor HQER-SFA.
beta_error	dataset_source=WDI_industry; pseudo_model_source=HQER-SFA; error_scenario=normal; sample_size_label=medium	Wilcoxon signed-rank normal approximation	173	127	0	Positive values favor HQER-SFA.
beta_error	dataset_source=WDI_industry; pseudo_model_source=HQER-SFA; error_scenario=skewed; sample_size_label=large	Wilcoxon signed-rank normal approximation	4	296	0	Positive values favor HQER-SFA.
beta_error	dataset_source=WDI_industry; pseudo_model_source=HQER-SFA; error_scenario=skewed; sample_size_label=medium	Wilcoxon signed-rank normal approximation	3	297	0	Positive values favor HQER-SFA.
beta_error	dataset_source=WDI_industry; pseudo_model_source=HQER-SFA; error_scenario=student_t; sample_size_label=large	Wilcoxon signed-rank normal approximation	136	164	0	Positive values favor HQER-SFA.
beta_error	dataset_source=WDI_industry; pseudo_model_source=HQER-SFA; error_scenario=student_t; sample_size_label=medium	Wilcoxon signed-rank normal approximation	119	181	0	Positive values favor HQER-SFA.
beta_error	dataset_source=WDI_industry; pseudo_model_source=Quantile-SFA; error_scenario=ald; sample_size_label=large	Wilcoxon signed-rank normal approximation	149	151	0	Positive values favor HQER-SFA.
beta_error	dataset_source=WDI_industry; pseudo_model_source=Quantile-SFA; error_scenario=ald; sample_size_label=medium	Wilcoxon signed-rank normal approximation	150	150	0	Positive values favor HQER-SFA.
beta_error	dataset_source=WDI_industry; pseudo_model_source=Quantile-SFA; error_scenario=hqer_induced; sample_size_label=large	Wilcoxon signed-rank normal approximation	165	135	0	Positive values favor HQER-SFA.
beta_error	dataset_source=WDI_industry; pseudo_model_source=Quantile-SFA; error_scenario=hqer_induced; sample_size_label=medium	Wilcoxon signed-rank normal approximation	159	141	0	Positive values favor HQER-SFA.
beta_error	dataset_source=WDI_industry; pseudo_model_source=Quantile-SFA; error_scenario=normal; sample_size_label=large	Wilcoxon signed-rank normal approximation	144	156	0	Positive values favor HQER-SFA.
beta_error	dataset_source=WDI_industry; pseudo_model_source=Quantile-SFA; error_scenario=normal; sample_size_label=medium	Wilcoxon signed-rank normal approximation	146	154	0	Positive values favor HQER-SFA.
beta_error	dataset_source=WDI_industry; pseudo_model_source=Quantile-SFA; error_scenario=skewed; sample_size_label=large	Wilcoxon signed-rank normal approximation	110	190	0	Positive values favor HQER-SFA.
beta_error	dataset_source=WDI_industry; pseudo_model_source=Quantile-SFA; error_scenario=skewed; sample_size_label=medium	Wilcoxon signed-rank normal approximation	117	183	0	Positive values favor HQER-SFA.
beta_error	dataset_source=WDI_industry; pseudo_model_source=Quantile-SFA; error_scenario=student_t; sample_size_label=large	Wilcoxon signed-rank normal approximation	144	156	0	Positive values favor HQER-SFA.
beta_error	dataset_source=WDI_industry; pseudo_model_source=Quantile-SFA; error_scenario=student_t; sample_size_label=medium	Wilcoxon signed-rank normal approximation	135	165	0	Positive values favor HQER-SFA.
beta_error	dataset_source=front41Data; pseudo_model_source=HQER-SFA; error_scenario=ald; sample_size_label=medium	Wilcoxon signed-rank normal approximation	31	269	0	Positive values favor HQER-SFA.
beta_error	dataset_source=front41Data; pseudo_model_source=HQER-SFA; error_scenario=ald; sample_size_label=small	Wilcoxon signed-rank normal approximation	97	203	0	Positive values favor HQER-SFA.
beta_error	dataset_source=front41Data; pseudo_model_source=HQER-SFA; error_scenario=hqer_induced; sample_size_label=medium	Wilcoxon signed-rank normal approximation	198	102	0	Positive values favor HQER-SFA.
beta_error	dataset_source=front41Data; pseudo_model_source=HQER-SFA; error_scenario=hqer_induced; sample_size_label=small	Wilcoxon signed-rank normal approximation	135	165	0	Positive values favor HQER-SFA.
beta_error	dataset_source=front41Data; pseudo_model_source=HQER-SFA; error_scenario=normal; sample_size_label=medium	Wilcoxon signed-rank normal approximation	217	83	0	Positive values favor HQER-SFA.
beta_error	dataset_source=front41Data; pseudo_model_source=HQER-SFA; error_scenario=normal; sample_size_label=small	Wilcoxon signed-rank normal approximation	174	126	0	Positive values favor HQER-SFA.
beta_error	dataset_source=front41Data; pseudo_model_source=HQER-SFA; error_scenario=skewed; sample_size_label=medium	Wilcoxon signed-rank normal approximation	25	275	0	Positive values favor HQER-SFA.
beta_error	dataset_source=front41Data; pseudo_model_source=HQER-SFA; error_scenario=skewed; sample_size_label=small	Wilcoxon signed-rank normal approximation	66	234	0	Positive values favor HQER-SFA.
beta_error	dataset_source=front41Data; pseudo_model_source=HQER-SFA; error_scenario=student_t; sample_size_label=medium	Wilcoxon signed-rank normal approximation	138	162	0	Positive values favor HQER-SFA.
beta_error	dataset_source=front41Data; pseudo_model_source=HQER-SFA; error_scenario=student_t; sample_size_label=small	Wilcoxon signed-rank normal approximation	141	159	0	Positive values favor HQER-SFA.
beta_error	dataset_source=front41Data; pseudo_model_source=Quantile-SFA; error_scenario=ald; sample_size_label=medium	Wilcoxon signed-rank normal approximation	137	163	0	Positive values favor HQER-SFA.
beta_error	dataset_source=front41Data; pseudo_model_source=Quantile-SFA; error_scenario=ald; sample_size_label=small	Wilcoxon signed-rank normal approximation	145	155	0	Positive values favor HQER-SFA.
beta_error	dataset_source=front41Data; pseudo_model_source=Quantile-SFA; error_scenario=hqer_induced; sample_size_label=medium	Wilcoxon signed-rank normal approximation	152	148	0	Positive values favor HQER-SFA.
beta_error	dataset_source=front41Data; pseudo_model_source=Quantile-SFA; error_scenario=hqer_induced; sample_size_label=small	Wilcoxon signed-rank normal approximation	152	148	0	Positive values favor HQER-SFA.
beta_error	dataset_source=front41Data; pseudo_model_source=Quantile-SFA; error_scenario=normal; sample_size_label=medium	Wilcoxon signed-rank normal approximation	159	141	0	Positive values favor HQER-SFA.
beta_error	dataset_source=front41Data; pseudo_model_source=Quantile-SFA; error_scenario=normal; sample_size_label=small	Wilcoxon signed-rank normal approximation	160	140	0	Positive values favor HQER-SFA.
beta_error	dataset_source=front41Data; pseudo_model_source=Quantile-SFA; error_scenario=skewed; sample_size_label=medium	Wilcoxon signed-rank normal approximation	135	165	0	Positive values favor HQER-SFA.
beta_error	dataset_source=front41Data; pseudo_model_source=Quantile-SFA; error_scenario=skewed; sample_size_label=small	Wilcoxon signed-rank normal approximation	146	154	0	Positive values favor HQER-SFA.
beta_error	dataset_source=front41Data; pseudo_model_source=Quantile-SFA; error_scenario=student_t; sample_size_label=medium	Wilcoxon signed-rank normal approximation	160	140	0	Positive values favor HQER-SFA.
beta_error	dataset_source=front41Data; pseudo_model_source=Quantile-SFA; error_scenario=student_t; sample_size_label=small	Wilcoxon signed-rank normal approximation	143	157	0	Positive values favor HQER-SFA.
y_rmse	overall	Wilcoxon signed-rank normal approximation	8404	9596	0	Positive values favor HQER-SFA.
y_rmse	dataset_source=WDI_agriculture; pseudo_model_source=HQER-SFA; error_scenario=ald; sample_size_label=large	Wilcoxon signed-rank normal approximation	99	201	0	Positive values favor HQER-SFA.
y_rmse	dataset_source=WDI_agriculture; pseudo_model_source=HQER-SFA; error_scenario=ald; sample_size_label=medium	Wilcoxon signed-rank normal approximation	91	209	0	Positive values favor HQER-SFA.
y_rmse	dataset_source=WDI_agriculture; pseudo_model_source=HQER-SFA; error_scenario=hqer_induced; sample_size_label=large	Wilcoxon signed-rank normal approximation	300	0	0	Positive values favor HQER-SFA.
y_rmse	dataset_source=WDI_agriculture; pseudo_model_source=HQER-SFA; error_scenario=hqer_induced; sample_size_label=medium	Wilcoxon signed-rank normal approximation	300	0	0	Positive values favor HQER-SFA.
y_rmse	dataset_source=WDI_agriculture; pseudo_model_source=HQER-SFA; error_scenario=normal; sample_size_label=large	Wilcoxon signed-rank normal approximation	300	0	0	Positive values favor HQER-SFA.
y_rmse	dataset_source=WDI_agriculture; pseudo_model_source=HQER-SFA; error_scenario=normal; sample_size_label=medium	Wilcoxon signed-rank normal approximation	299	1	0	Positive values favor HQER-SFA.
y_rmse	dataset_source=WDI_agriculture; pseudo_model_source=HQER-SFA; error_scenario=skewed; sample_size_label=large	Wilcoxon signed-rank normal approximation	6	294	0	Positive values favor HQER-SFA.
y_rmse	dataset_source=WDI_agriculture; pseudo_model_source=HQER-SFA; error_scenario=skewed; sample_size_label=medium	Wilcoxon signed-rank normal approximation	20	280	0	Positive values favor HQER-SFA.

metric	group	wilcoxon_method	n_positive	n_negative	n_zero	positive_difference_direction
y_rmse	dataset_source=WDI_agriculture; pseudo_model_source=HQER-SFA; error_scenario=student_t; sample_size_label=large	Wilcoxon signed-rank normal approximation	146	154	0	Positive values favor HQER-SFA.
y_rmse	dataset_source=WDI_agriculture; pseudo_model_source=HQER-SFA; error_scenario=student_t; sample_size_label=medium	Wilcoxon signed-rank normal approximation	152	148	0	Positive values favor HQER-SFA.
y_rmse	dataset_source=WDI_agriculture; pseudo_model_source=Quantile-SFA; error_scenario=ald; sample_size_label=large	Wilcoxon signed-rank normal approximation	159	141	0	Positive values favor HQER-SFA.
y_rmse	dataset_source=WDI_agriculture; pseudo_model_source=Quantile-SFA; error_scenario=ald; sample_size_label=medium	Wilcoxon signed-rank normal approximation	155	145	0	Positive values favor HQER-SFA.
y_rmse	dataset_source=WDI_agriculture; pseudo_model_source=Quantile-SFA; error_scenario=hqer_induced; sample_size_label=large	Wilcoxon signed-rank normal approximation	166	134	0	Positive values favor HQER-SFA.
y_rmse	dataset_source=WDI_agriculture; pseudo_model_source=Quantile-SFA; error_scenario=hqer_induced; sample_size_label=medium	Wilcoxon signed-rank normal approximation	157	143	0	Positive values favor HQER-SFA.
y_rmse	dataset_source=WDI_agriculture; pseudo_model_source=Quantile-SFA; error_scenario=normal; sample_size_label=large	Wilcoxon signed-rank normal approximation	139	161	0	Positive values favor HQER-SFA.
y_rmse	dataset_source=WDI_agriculture; pseudo_model_source=Quantile-SFA; error_scenario=normal; sample_size_label=medium	Wilcoxon signed-rank normal approximation	139	161	0	Positive values favor HQER-SFA.
y_rmse	dataset_source=WDI_agriculture; pseudo_model_source=Quantile-SFA; error_scenario=skewed; sample_size_label=large	Wilcoxon signed-rank normal approximation	153	147	0	Positive values favor HQER-SFA.
y_rmse	dataset_source=WDI_agriculture; pseudo_model_source=Quantile-SFA; error_scenario=skewed; sample_size_label=medium	Wilcoxon signed-rank normal approximation	155	145	0	Positive values favor HQER-SFA.
y_rmse	dataset_source=WDI_agriculture; pseudo_model_source=Quantile-SFA; error_scenario=student_t; sample_size_label=large	Wilcoxon signed-rank normal approximation	152	148	0	Positive values favor HQER-SFA.
y_rmse	dataset_source=WDI_agriculture; pseudo_model_source=Quantile-SFA; error_scenario=student_t; sample_size_label=medium	Wilcoxon signed-rank normal approximation	157	143	0	Positive values favor HQER-SFA.
y_rmse	dataset_source=WDI_industry; pseudo_model_source=HQER-SFA; error_scenario=ald; sample_size_label=large	Wilcoxon signed-rank normal approximation	73	227	0	Positive values favor HQER-SFA.
y_rmse	dataset_source=WDI_industry; pseudo_model_source=HQER-SFA; error_scenario=ald; sample_size_label=medium	Wilcoxon signed-rank normal approximation	136	164	0	Positive values favor HQER-SFA.
y_rmse	dataset_source=WDI_industry; pseudo_model_source=HQER-SFA; error_scenario=hqer_induced; sample_size_label=large	Wilcoxon signed-rank normal approximation	173	127	0	Positive values favor HQER-SFA.
y_rmse	dataset_source=WDI_industry; pseudo_model_source=HQER-SFA; error_scenario=hqer_induced; sample_size_label=medium	Wilcoxon signed-rank normal approximation	197	103	0	Positive values favor HQER-SFA.
y_rmse	dataset_source=WDI_industry; pseudo_model_source=HQER-SFA; error_scenario=normal; sample_size_label=large	Wilcoxon signed-rank normal approximation	121	179	0	Positive values favor HQER-SFA.
y_rmse	dataset_source=WDI_industry; pseudo_model_source=HQER-SFA; error_scenario=normal; sample_size_label=medium	Wilcoxon signed-rank normal approximation	141	159	0	Positive values favor HQER-SFA.
y_rmse	dataset_source=WDI_industry; pseudo_model_source=HQER-SFA; error_scenario=skewed; sample_size_label=large	Wilcoxon signed-rank normal approximation	4	296	0	Positive values favor HQER-SFA.
y_rmse	dataset_source=WDI_industry; pseudo_model_source=HQER-SFA; error_scenario=skewed; sample_size_label=medium	Wilcoxon signed-rank normal approximation	4	296	0	Positive values favor HQER-SFA.
y_rmse	dataset_source=WDI_industry; pseudo_model_source=HQER-SFA; error_scenario=student_t; sample_size_label=large	Wilcoxon signed-rank normal approximation	131	169	0	Positive values favor HQER-SFA.
y_rmse	dataset_source=WDI_industry; pseudo_model_source=HQER-SFA; error_scenario=student_t; sample_size_label=medium	Wilcoxon signed-rank normal approximation	127	173	0	Positive values favor HQER-SFA.
y_rmse	dataset_source=WDI_industry; pseudo_model_source=Quantile-SFA; error_scenario=ald; sample_size_label=large	Wilcoxon signed-rank normal approximation	142	158	0	Positive values favor HQER-SFA.
y_rmse	dataset_source=WDI_industry; pseudo_model_source=Quantile-SFA; error_scenario=ald; sample_size_label=medium	Wilcoxon signed-rank normal approximation	147	153	0	Positive values favor HQER-SFA.
y_rmse	dataset_source=WDI_industry; pseudo_model_source=Quantile-SFA; error_scenario=hqer_induced; sample_size_label=large	Wilcoxon signed-rank normal approximation	179	121	0	Positive values favor HQER-SFA.
y_rmse	dataset_source=WDI_industry; pseudo_model_source=Quantile-SFA; error_scenario=hqer_induced; sample_size_label=medium	Wilcoxon signed-rank normal approximation	165	135	0	Positive values favor HQER-SFA.
y_rmse	dataset_source=WDI_industry; pseudo_model_source=Quantile-SFA; error_scenario=normal; sample_size_label=large	Wilcoxon signed-rank normal approximation	152	148	0	Positive values favor HQER-SFA.
y_rmse	dataset_source=WDI_industry; pseudo_model_source=Quantile-SFA; error_scenario=normal; sample_size_label=medium	Wilcoxon signed-rank normal approximation	148	152	0	Positive values favor HQER-SFA.
y_rmse	dataset_source=WDI_industry; pseudo_model_source=Quantile-SFA; error_scenario=skewed; sample_size_label=large	Wilcoxon signed-rank normal approximation	109	191	0	Positive values favor HQER-SFA.
y_rmse	dataset_source=WDI_industry; pseudo_model_source=Quantile-SFA; error_scenario=skewed; sample_size_label=medium	Wilcoxon signed-rank normal approximation	113	187	0	Positive values favor HQER-SFA.
y_rmse	dataset_source=WDI_industry; pseudo_model_source=Quantile-SFA; error_scenario=student_t; sample_size_label=large	Wilcoxon signed-rank normal approximation	148	152	0	Positive values favor HQER-SFA.
y_rmse	dataset_source=WDI_industry; pseudo_model_source=Quantile-SFA; error_scenario=student_t; sample_size_label=medium	Wilcoxon signed-rank normal approximation	141	159	0	Positive values favor HQER-SFA.
y_rmse	dataset_source=front41Data; pseudo_model_source=HQER-SFA; error_scenario=ald; sample_size_label=medium	Wilcoxon signed-rank normal approximation	18	282	0	Positive values favor HQER-SFA.
y_rmse	dataset_source=front41Data; pseudo_model_source=HQER-SFA; error_scenario=ald; sample_size_label=small	Wilcoxon signed-rank normal approximation	96	204	0	Positive values favor HQER-SFA.
y_rmse	dataset_source=front41Data; pseudo_model_source=HQER-SFA; error_scenario=hqer_induced; sample_size_label=medium	Wilcoxon signed-rank normal approximation	39	261	0	Positive values favor HQER-SFA.
y_rmse	dataset_source=front41Data; pseudo_model_source=HQER-SFA; error_scenario=hqer_induced; sample_size_label=small	Wilcoxon signed-rank normal approximation	121	179	0	Positive values favor HQER-SFA.
y_rmse	dataset_source=front41Data; pseudo_model_source=HQER-SFA; error_scenario=normal; sample_size_label=medium	Wilcoxon signed-rank normal approximation	196	104	0	Positive values favor HQER-SFA.
y_rmse	dataset_source=front41Data; pseudo_model_source=HQER-SFA; error_scenario=normal; sample_size_label=small	Wilcoxon signed-rank normal approximation	199	101	0	Positive values favor HQER-SFA.
y_rmse	dataset_source=front41Data; pseudo_model_source=HQER-SFA; error_scenario=skewed; sample_size_label=medium	Wilcoxon signed-rank normal approximation	25	275	0	Positive values favor HQER-SFA.
y_rmse	dataset_source=front41Data; pseudo_model_source=HQER-SFA; error_scenario=skewed; sample_size_label=small	Wilcoxon signed-rank normal approximation	91	209	0	Positive values favor HQER-SFA.
y_rmse	dataset_source=front41Data; pseudo_model_source=HQER-SFA; error_scenario=student_t; sample_size_label=medium	Wilcoxon signed-rank normal approximation	149	151	0	Positive values favor HQER-SFA.
y_rmse	dataset_source=front41Data; pseudo_model_source=HQER-SFA; error_scenario=student_t; sample_size_label=small	Wilcoxon signed-rank normal approximation	174	126	0	Positive values favor HQER-SFA.
y_rmse	dataset_source=front41Data; pseudo_model_source=Quantile-SFA; error_scenario=ald; sample_size_label=medium	Wilcoxon signed-rank normal approximation	153	147	0	Positive values favor HQER-SFA.
y_rmse	dataset_source=front41Data; pseudo_model_source=Quantile-SFA; error_scenario=ald; sample_size_label=small	Wilcoxon signed-rank normal approximation	149	151	0	Positive values favor HQER-SFA.
y_rmse	dataset_source=front41Data; pseudo_model_source=Quantile-SFA; error_scenario=hqer_induced; sample_size_label=medium	Wilcoxon signed-rank normal approximation	155	145	0	Positive values favor HQER-SFA.
y_rmse	dataset_source=front41Data; pseudo_model_source=Quantile-SFA; error_scenario=hqer_induced; sample_size_label=small	Wilcoxon signed-rank normal approximation	147	153	0	Positive values favor HQER-SFA.
y_rmse	dataset_source=front41Data; pseudo_model_source=Quantile-SFA; error_scenario=normal; sample_size_label=medium	Wilcoxon signed-rank normal approximation	162	138	0	Positive values favor HQER-SFA.
y_rmse	dataset_source=front41Data; pseudo_model_source=Quantile-SFA; error_scenario=normal; sample_size_label=small	Wilcoxon signed-rank normal approximation	150	150	0	Positive values favor HQER-SFA.

metric	group	wilcoxon_method	n_positive	n_negative	n_zero	positive_difference_direction
y_rmse	dataset_source=front41Data; pseudo_model_source=Quantile-SFA; error_scenario=skewed; sample_size_label=medium	Wilcoxon signed-rank normal approximation	141	159	0	Positive values favor HQER-SFA.
y_rmse	dataset_source=front41Data; pseudo_model_source=Quantile-SFA; error_scenario=skewed; sample_size_label=small	Wilcoxon signed-rank normal approximation	140	160	0	Positive values favor HQER-SFA.
y_rmse	dataset_source=front41Data; pseudo_model_source=Quantile-SFA; error_scenario=student_t; sample_size_label=medium	Wilcoxon signed-rank normal approximation	159	141	0	Positive values favor HQER-SFA.
y_rmse	dataset_source=front41Data; pseudo_model_source=Quantile-SFA; error_scenario=student_t; sample_size_label=small	Wilcoxon signed-rank normal approximation	144	156	0	Positive values favor HQER-SFA.
TE_RMSE	overall	Wilcoxon signed-rank normal approximation	10163	7837	0	Positive values favor HQER-SFA.
TE_RMSE	dataset_source=WDI_agriculture; pseudo_model_source=HQER-SFA; error_scenario=ald; sample_size_label=large	Wilcoxon signed-rank normal approximation	69	231	0	Positive values favor HQER-SFA.
TE_RMSE	dataset_source=WDI_agriculture; pseudo_model_source=HQER-SFA; error_scenario=ald; sample_size_label=medium	Wilcoxon signed-rank normal approximation	62	238	0	Positive values favor HQER-SFA.
TE_RMSE	dataset_source=WDI_agriculture; pseudo_model_source=HQER-SFA; error_scenario=hqer_induced; sample_size_label=large	Wilcoxon signed-rank normal approximation	300	0	0	Positive values favor HQER-SFA.
TE_RMSE	dataset_source=WDI_agriculture; pseudo_model_source=HQER-SFA; error_scenario=hqer_induced; sample_size_label=medium	Wilcoxon signed-rank normal approximation	299	1	0	Positive values favor HQER-SFA.
TE_RMSE	dataset_source=WDI_agriculture; pseudo_model_source=HQER-SFA; error_scenario=normal; sample_size_label=large	Wilcoxon signed-rank normal approximation	300	0	0	Positive values favor HQER-SFA.
TE_RMSE	dataset_source=WDI_agriculture; pseudo_model_source=HQER-SFA; error_scenario=normal; sample_size_label=medium	Wilcoxon signed-rank normal approximation	300	0	0	Positive values favor HQER-SFA.
TE_RMSE	dataset_source=WDI_agriculture; pseudo_model_source=HQER-SFA; error_scenario=skewed; sample_size_label=large	Wilcoxon signed-rank normal approximation	286	14	0	Positive values favor HQER-SFA.
TE_RMSE	dataset_source=WDI_agriculture; pseudo_model_source=HQER-SFA; error_scenario=skewed; sample_size_label=medium	Wilcoxon signed-rank normal approximation	259	41	0	Positive values favor HQER-SFA.
TE_RMSE	dataset_source=WDI_agriculture; pseudo_model_source=HQER-SFA; error_scenario=student_t; sample_size_label=large	Wilcoxon signed-rank normal approximation	193	107	0	Positive values favor HQER-SFA.
TE_RMSE	dataset_source=WDI_agriculture; pseudo_model_source=HQER-SFA; error_scenario=student_t; sample_size_label=medium	Wilcoxon signed-rank normal approximation	184	116	0	Positive values favor HQER-SFA.
TE_RMSE	dataset_source=WDI_agriculture; pseudo_model_source=Quantile-SFA; error_scenario=ald; sample_size_label=large	Wilcoxon signed-rank normal approximation	163	137	0	Positive values favor HQER-SFA.
TE_RMSE	dataset_source=WDI_agriculture; pseudo_model_source=Quantile-SFA; error_scenario=ald; sample_size_label=medium	Wilcoxon signed-rank normal approximation	147	153	0	Positive values favor HQER-SFA.
TE_RMSE	dataset_source=WDI_agriculture; pseudo_model_source=Quantile-SFA; error_scenario=hqer_induced; sample_size_label=large	Wilcoxon signed-rank normal approximation	155	145	0	Positive values favor HQER-SFA.
TE_RMSE	dataset_source=WDI_agriculture; pseudo_model_source=Quantile-SFA; error_scenario=hqer_induced; sample_size_label=medium	Wilcoxon signed-rank normal approximation	170	130	0	Positive values favor HQER-SFA.
TE_RMSE	dataset_source=WDI_agriculture; pseudo_model_source=Quantile-SFA; error_scenario=normal; sample_size_label=large	Wilcoxon signed-rank normal approximation	145	155	0	Positive values favor HQER-SFA.
TE_RMSE	dataset_source=WDI_agriculture; pseudo_model_source=Quantile-SFA; error_scenario=normal; sample_size_label=medium	Wilcoxon signed-rank normal approximation	132	168	0	Positive values favor HQER-SFA.
TE_RMSE	dataset_source=WDI_agriculture; pseudo_model_source=Quantile-SFA; error_scenario=skewed; sample_size_label=large	Wilcoxon signed-rank normal approximation	128	172	0	Positive values favor HQER-SFA.
TE_RMSE	dataset_source=WDI_agriculture; pseudo_model_source=Quantile-SFA; error_scenario=skewed; sample_size_label=medium	Wilcoxon signed-rank normal approximation	139	161	0	Positive values favor HQER-SFA.
TE_RMSE	dataset_source=WDI_agriculture; pseudo_model_source=Quantile-SFA; error_scenario=student_t; sample_size_label=large	Wilcoxon signed-rank normal approximation	122	178	0	Positive values favor HQER-SFA.
TE_RMSE	dataset_source=WDI_agriculture; pseudo_model_source=Quantile-SFA; error_scenario=student_t; sample_size_label=medium	Wilcoxon signed-rank normal approximation	142	158	0	Positive values favor HQER-SFA.
TE_RMSE	dataset_source=WDI_industry; pseudo_model_source=HQER-SFA; error_scenario=ald; sample_size_label=large	Wilcoxon signed-rank normal approximation	157	143	0	Positive values favor HQER-SFA.
TE_RMSE	dataset_source=WDI_industry; pseudo_model_source=HQER-SFA; error_scenario=ald; sample_size_label=medium	Wilcoxon signed-rank normal approximation	128	172	0	Positive values favor HQER-SFA.
TE_RMSE	dataset_source=WDI_industry; pseudo_model_source=HQER-SFA; error_scenario=hqer_induced; sample_size_label=large	Wilcoxon signed-rank normal approximation	190	110	0	Positive values favor HQER-SFA.
TE_RMSE	dataset_source=WDI_industry; pseudo_model_source=HQER-SFA; error_scenario=hqer_induced; sample_size_label=medium	Wilcoxon signed-rank normal approximation	171	129	0	Positive values favor HQER-SFA.
TE_RMSE	dataset_source=WDI_industry; pseudo_model_source=HQER-SFA; error_scenario=normal; sample_size_label=large	Wilcoxon signed-rank normal approximation	277	23	0	Positive values favor HQER-SFA.
TE_RMSE	dataset_source=WDI_industry; pseudo_model_source=HQER-SFA; error_scenario=normal; sample_size_label=medium	Wilcoxon signed-rank normal approximation	225	75	0	Positive values favor HQER-SFA.
TE_RMSE	dataset_source=WDI_industry; pseudo_model_source=HQER-SFA; error_scenario=skewed; sample_size_label=large	Wilcoxon signed-rank normal approximation	232	68	0	Positive values favor HQER-SFA.
TE_RMSE	dataset_source=WDI_industry; pseudo_model_source=HQER-SFA; error_scenario=skewed; sample_size_label=medium	Wilcoxon signed-rank normal approximation	158	142	0	Positive values favor HQER-SFA.
TE_RMSE	dataset_source=WDI_industry; pseudo_model_source=HQER-SFA; error_scenario=student_t; sample_size_label=large	Wilcoxon signed-rank normal approximation	74	226	0	Positive values favor HQER-SFA.
TE_RMSE	dataset_source=WDI_industry; pseudo_model_source=HQER-SFA; error_scenario=student_t; sample_size_label=medium	Wilcoxon signed-rank normal approximation	104	196	0	Positive values favor HQER-SFA.
TE_RMSE	dataset_source=WDI_industry; pseudo_model_source=Quantile-SFA; error_scenario=ald; sample_size_label=large	Wilcoxon signed-rank normal approximation	149	151	0	Positive values favor HQER-SFA.
TE_RMSE	dataset_source=WDI_industry; pseudo_model_source=Quantile-SFA; error_scenario=ald; sample_size_label=medium	Wilcoxon signed-rank normal approximation	166	134	0	Positive values favor HQER-SFA.
TE_RMSE	dataset_source=WDI_industry; pseudo_model_source=Quantile-SFA; error_scenario=hqer_induced; sample_size_label=large	Wilcoxon signed-rank normal approximation	155	145	0	Positive values favor HQER-SFA.
TE_RMSE	dataset_source=WDI_industry; pseudo_model_source=Quantile-SFA; error_scenario=hqer_induced; sample_size_label=medium	Wilcoxon signed-rank normal approximation	150	150	0	Positive values favor HQER-SFA.
TE_RMSE	dataset_source=WDI_industry; pseudo_model_source=Quantile-SFA; error_scenario=normal; sample_size_label=large	Wilcoxon signed-rank normal approximation	154	146	0	Positive values favor HQER-SFA.
TE_RMSE	dataset_source=WDI_industry; pseudo_model_source=Quantile-SFA; error_scenario=normal; sample_size_label=medium	Wilcoxon signed-rank normal approximation	155	145	0	Positive values favor HQER-SFA.
TE_RMSE	dataset_source=WDI_industry; pseudo_model_source=Quantile-SFA; error_scenario=skewed; sample_size_label=large	Wilcoxon signed-rank normal approximation	169	131	0	Positive values favor HQER-SFA.
TE_RMSE	dataset_source=WDI_industry; pseudo_model_source=Quantile-SFA; error_scenario=skewed; sample_size_label=medium	Wilcoxon signed-rank normal approximation	154	146	0	Positive values favor HQER-SFA.
TE_RMSE	dataset_source=WDI_industry; pseudo_model_source=Quantile-SFA; error_scenario=student_t; sample_size_label=large	Wilcoxon signed-rank normal approximation	73	227	0	Positive values favor HQER-SFA.
TE_RMSE	dataset_source=WDI_industry; pseudo_model_source=Quantile-SFA; error_scenario=student_t; sample_size_label=medium	Wilcoxon signed-rank normal approximation	121	179	0	Positive values favor HQER-SFA.
TE_RMSE	dataset_source=front41Data; pseudo_model_source=HQER-SFA; error_scenario=ald; sample_size_label=medium	Wilcoxon signed-rank normal approximation	181	119	0	Positive values favor HQER-SFA.
TE_RMSE	dataset_source=front41Data; pseudo_model_source=HQER-SFA; error_scenario=ald; sample_size_label=small	Wilcoxon signed-rank normal approximation	153	147	0	Positive values favor HQER-SFA.
TE_RMSE	dataset_source=front41Data; pseudo_model_source=HQER-SFA; error_scenario=hqer_induced; sample_size_label=medium	Wilcoxon signed-rank normal approximation	266	34	0	Positive values favor HQER-SFA.

metric	group	wilcoxon_method	n_positive	n_negative	n_zero	positive_difference_direction
TE_RMSE	dataset_source=front41Data; pseudo_model_source=HQER-SFA; error_scenario=hqer_induced; sample_size_label=small	Wilcoxon signed-rank normal approximation	197	103	0	Positive values favor HQER-SFA.
TE_RMSE	dataset_source=front41Data; pseudo_model_source=HQER-SFA; error_scenario=normal; sample_size_label=medium	Wilcoxon signed-rank normal approximation	243	57	0	Positive values favor HQER-SFA.
TE_RMSE	dataset_source=front41Data; pseudo_model_source=HQER-SFA; error_scenario=normal; sample_size_label=small	Wilcoxon signed-rank normal approximation	205	95	0	Positive values favor HQER-SFA.
TE_RMSE	dataset_source=front41Data; pseudo_model_source=HQER-SFA; error_scenario=skewed; sample_size_label=medium	Wilcoxon signed-rank normal approximation	152	148	0	Positive values favor HQER-SFA.
TE_RMSE	dataset_source=front41Data; pseudo_model_source=HQER-SFA; error_scenario=skewed; sample_size_label=small	Wilcoxon signed-rank normal approximation	144	156	0	Positive values favor HQER-SFA.
TE_RMSE	dataset_source=front41Data; pseudo_model_source=HQER-SFA; error_scenario=student_t; sample_size_label=medium	Wilcoxon signed-rank normal approximation	118	182	0	Positive values favor HQER-SFA.
TE_RMSE	dataset_source=front41Data; pseudo_model_source=HQER-SFA; error_scenario=student_t; sample_size_label=small	Wilcoxon signed-rank normal approximation	141	159	0	Positive values favor HQER-SFA.
TE_RMSE	dataset_source=front41Data; pseudo_model_source=Quantile-SFA; error_scenario=ald; sample_size_label=medium	Wilcoxon signed-rank normal approximation	159	141	0	Positive values favor HQER-SFA.
TE_RMSE	dataset_source=front41Data; pseudo_model_source=Quantile-SFA; error_scenario=ald; sample_size_label=small	Wilcoxon signed-rank normal approximation	159	141	0	Positive values favor HQER-SFA.
TE_RMSE	dataset_source=front41Data; pseudo_model_source=Quantile-SFA; error_scenario=hqer_induced; sample_size_label=medium	Wilcoxon signed-rank normal approximation	152	148	0	Positive values favor HQER-SFA.
TE_RMSE	dataset_source=front41Data; pseudo_model_source=Quantile-SFA; error_scenario=hqer_induced; sample_size_label=small	Wilcoxon signed-rank normal approximation	148	152	0	Positive values favor HQER-SFA.
TE_RMSE	dataset_source=front41Data; pseudo_model_source=Quantile-SFA; error_scenario=normal; sample_size_label=medium	Wilcoxon signed-rank normal approximation	162	138	0	Positive values favor HQER-SFA.
TE_RMSE	dataset_source=front41Data; pseudo_model_source=Quantile-SFA; error_scenario=normal; sample_size_label=small	Wilcoxon signed-rank normal approximation	158	142	0	Positive values favor HQER-SFA.
TE_RMSE	dataset_source=front41Data; pseudo_model_source=Quantile-SFA; error_scenario=skewed; sample_size_label=medium	Wilcoxon signed-rank normal approximation	154	146	0	Positive values favor HQER-SFA.
TE_RMSE	dataset_source=front41Data; pseudo_model_source=Quantile-SFA; error_scenario=skewed; sample_size_label=small	Wilcoxon signed-rank normal approximation	141	159	0	Positive values favor HQER-SFA.
TE_RMSE	dataset_source=front41Data; pseudo_model_source=Quantile-SFA; error_scenario=student_t; sample_size_label=medium	Wilcoxon signed-rank normal approximation	130	170	0	Positive values favor HQER-SFA.
TE_RMSE	dataset_source=front41Data; pseudo_model_source=Quantile-SFA; error_scenario=student_t; sample_size_label=small	Wilcoxon signed-rank normal approximation	143	157	0	Positive values favor HQER-SFA.
TE_rank_corr	overall	Wilcoxon signed-rank normal approximation	10041	7959	0	Positive values favor HQER-SFA.
TE_rank_corr	dataset_source=WDI_agriculture; pseudo_model_source=HQER-SFA; error_scenario=ald; sample_size_label=large	Wilcoxon signed-rank normal approximation	117	183	0	Positive values favor HQER-SFA.
TE_rank_corr	dataset_source=WDI_agriculture; pseudo_model_source=HQER-SFA; error_scenario=ald; sample_size_label=medium	Wilcoxon signed-rank normal approximation	143	157	0	Positive values favor HQER-SFA.
TE_rank_corr	dataset_source=WDI_agriculture; pseudo_model_source=HQER-SFA; error_scenario=hqer_induced; sample_size_label=large	Wilcoxon signed-rank normal approximation	216	84	0	Positive values favor HQER-SFA.
TE_rank_corr	dataset_source=WDI_agriculture; pseudo_model_source=HQER-SFA; error_scenario=hqer_induced; sample_size_label=medium	Wilcoxon signed-rank normal approximation	185	115	0	Positive values favor HQER-SFA.
TE_rank_corr	dataset_source=WDI_agriculture; pseudo_model_source=HQER-SFA; error_scenario=normal; sample_size_label=large	Wilcoxon signed-rank normal approximation	218	82	0	Positive values favor HQER-SFA.
TE_rank_corr	dataset_source=WDI_agriculture; pseudo_model_source=HQER-SFA; error_scenario=normal; sample_size_label=medium	Wilcoxon signed-rank normal approximation	198	102	0	Positive values favor HQER-SFA.
TE_rank_corr	dataset_source=WDI_agriculture; pseudo_model_source=HQER-SFA; error_scenario=skewed; sample_size_label=large	Wilcoxon signed-rank normal approximation	185	115	0	Positive values favor HQER-SFA.
TE_rank_corr	dataset_source=WDI_agriculture; pseudo_model_source=HQER-SFA; error_scenario=skewed; sample_size_label=medium	Wilcoxon signed-rank normal approximation	184	116	0	Positive values favor HQER-SFA.
TE_rank_corr	dataset_source=WDI_agriculture; pseudo_model_source=HQER-SFA; error_scenario=student_t; sample_size_label=large	Wilcoxon signed-rank normal approximation	179	121	0	Positive values favor HQER-SFA.
TE_rank_corr	dataset_source=WDI_agriculture; pseudo_model_source=HQER-SFA; error_scenario=student_t; sample_size_label=medium	Wilcoxon signed-rank normal approximation	168	132	0	Positive values favor HQER-SFA.
TE_rank_corr	dataset_source=WDI_agriculture; pseudo_model_source=Quantile-SFA; error_scenario=ald; sample_size_label=large	Wilcoxon signed-rank normal approximation	117	183	0	Positive values favor HQER-SFA.
TE_rank_corr	dataset_source=WDI_agriculture; pseudo_model_source=Quantile-SFA; error_scenario=ald; sample_size_label=medium	Wilcoxon signed-rank normal approximation	143	157	0	Positive values favor HQER-SFA.
TE_rank_corr	dataset_source=WDI_agriculture; pseudo_model_source=Quantile-SFA; error_scenario=hqer_induced; sample_size_label=large	Wilcoxon signed-rank normal approximation	168	132	0	Positive values favor HQER-SFA.
TE_rank_corr	dataset_source=WDI_agriculture; pseudo_model_source=Quantile-SFA; error_scenario=hqer_induced; sample_size_label=medium	Wilcoxon signed-rank normal approximation	176	124	0	Positive values favor HQER-SFA.
TE_rank_corr	dataset_source=WDI_agriculture; pseudo_model_source=Quantile-SFA; error_scenario=normal; sample_size_label=large	Wilcoxon signed-rank normal approximation	211	89	0	Positive values favor HQER-SFA.
TE_rank_corr	dataset_source=WDI_agriculture; pseudo_model_source=Quantile-SFA; error_scenario=normal; sample_size_label=medium	Wilcoxon signed-rank normal approximation	175	125	0	Positive values favor HQER-SFA.
TE_rank_corr	dataset_source=WDI_agriculture; pseudo_model_source=Quantile-SFA; error_scenario=skewed; sample_size_label=large	Wilcoxon signed-rank normal approximation	216	84	0	Positive values favor HQER-SFA.
TE_rank_corr	dataset_source=WDI_agriculture; pseudo_model_source=Quantile-SFA; error_scenario=skewed; sample_size_label=medium	Wilcoxon signed-rank normal approximation	192	108	0	Positive values favor HQER-SFA.
TE_rank_corr	dataset_source=WDI_agriculture; pseudo_model_source=Quantile-SFA; error_scenario=student_t; sample_size_label=large	Wilcoxon signed-rank normal approximation	74	226	0	Positive values favor HQER-SFA.
TE_rank_corr	dataset_source=WDI_agriculture; pseudo_model_source=Quantile-SFA; error_scenario=student_t; sample_size_label=medium	Wilcoxon signed-rank normal approximation	144	156	0	Positive values favor HQER-SFA.
TE_rank_corr	dataset_source=WDI_industry; pseudo_model_source=HQER-SFA; error_scenario=ald; sample_size_label=large	Wilcoxon signed-rank normal approximation	98	202	0	Positive values favor HQER-SFA.
TE_rank_corr	dataset_source=WDI_industry; pseudo_model_source=HQER-SFA; error_scenario=ald; sample_size_label=medium	Wilcoxon signed-rank normal approximation	123	177	0	Positive values favor HQER-SFA.
TE_rank_corr	dataset_source=WDI_industry; pseudo_model_source=HQER-SFA; error_scenario=hqer_induced; sample_size_label=large	Wilcoxon signed-rank normal approximation	113	187	0	Positive values favor HQER-SFA.
TE_rank_corr	dataset_source=WDI_industry; pseudo_model_source=HQER-SFA; error_scenario=hqer_induced; sample_size_label=medium	Wilcoxon signed-rank normal approximation	138	162	0	Positive values favor HQER-SFA.
TE_rank_corr	dataset_source=WDI_industry; pseudo_model_source=HQER-SFA; error_scenario=normal; sample_size_label=large	Wilcoxon signed-rank normal approximation	286	14	0	Positive values favor HQER-SFA.
TE_rank_corr	dataset_source=WDI_industry; pseudo_model_source=HQER-SFA; error_scenario=normal; sample_size_label=medium	Wilcoxon signed-rank normal approximation	226	74	0	Positive values favor HQER-SFA.
TE_rank_corr	dataset_source=WDI_industry; pseudo_model_source=HQER-SFA; error_scenario=skewed; sample_size_label=large	Wilcoxon signed-rank normal approximation	94	206	0	Positive values favor HQER-SFA.
TE_rank_corr	dataset_source=WDI_industry; pseudo_model_source=HQER-SFA; error_scenario=skewed; sample_size_label=medium	Wilcoxon signed-rank normal approximation	126	174	0	Positive values favor HQER-SFA.
TE_rank_corr	dataset_source=WDI_industry; pseudo_model_source=HQER-SFA; error_scenario=student_t; sample_size_label=large	Wilcoxon signed-rank normal approximation	220	80	0	Positive values favor HQER-SFA.
TE_rank_corr	dataset_source=WDI_industry; pseudo_model_source=HQER-SFA; error_scenario=student_t; sample_size_label=medium	Wilcoxon signed-rank normal approximation	190	110	0	Positive values favor HQER-SFA.

metric	group	wilcoxon_method	n_positive	n_negative	n_zero	positive_difference_direction
TE_rank_corr	dataset_source=WDI_industry; pseudo_model_source=Quantile-SFA; error_scenario=ald; sample_size_label=large	Wilcoxon signed-rank normal approximation	90	210	0	Positive values favor HQER-SFA.
TE_rank_corr	dataset_source=WDI_industry; pseudo_model_source=Quantile-SFA; error_scenario=ald; sample_size_label=medium	Wilcoxon signed-rank normal approximation	138	162	0	Positive values favor HQER-SFA.
TE_rank_corr	dataset_source=WDI_industry; pseudo_model_source=Quantile-SFA; error_scenario=hqer_induced; sample_size_label=large	Wilcoxon signed-rank normal approximation	119	181	0	Positive values favor HQER-SFA.
TE_rank_corr	dataset_source=WDI_industry; pseudo_model_source=Quantile-SFA; error_scenario=hqer_induced; sample_size_label=medium	Wilcoxon signed-rank normal approximation	127	173	0	Positive values favor HQER-SFA.
TE_rank_corr	dataset_source=WDI_industry; pseudo_model_source=Quantile-SFA; error_scenario=normal; sample_size_label=large	Wilcoxon signed-rank normal approximation	290	10	0	Positive values favor HQER-SFA.
TE_rank_corr	dataset_source=WDI_industry; pseudo_model_source=Quantile-SFA; error_scenario=normal; sample_size_label=medium	Wilcoxon signed-rank normal approximation	252	48	0	Positive values favor HQER-SFA.
TE_rank_corr	dataset_source=WDI_industry; pseudo_model_source=Quantile-SFA; error_scenario=skewed; sample_size_label=large	Wilcoxon signed-rank normal approximation	93	207	0	Positive values favor HQER-SFA.
TE_rank_corr	dataset_source=WDI_industry; pseudo_model_source=Quantile-SFA; error_scenario=skewed; sample_size_label=medium	Wilcoxon signed-rank normal approximation	126	174	0	Positive values favor HQER-SFA.
TE_rank_corr	dataset_source=WDI_industry; pseudo_model_source=Quantile-SFA; error_scenario=student_t; sample_size_label=large	Wilcoxon signed-rank normal approximation	238	62	0	Positive values favor HQER-SFA.
TE_rank_corr	dataset_source=WDI_industry; pseudo_model_source=Quantile-SFA; error_scenario=student_t; sample_size_label=medium	Wilcoxon signed-rank normal approximation	190	110	0	Positive values favor HQER-SFA.
TE_rank_corr	dataset_source=front41Data; pseudo_model_source=HQER-SFA; error_scenario=ald; sample_size_label=medium	Wilcoxon signed-rank normal approximation	102	198	0	Positive values favor HQER-SFA.
TE_rank_corr	dataset_source=front41Data; pseudo_model_source=HQER-SFA; error_scenario=ald; sample_size_label=small	Wilcoxon signed-rank normal approximation	145	155	0	Positive values favor HQER-SFA.
TE_rank_corr	dataset_source=front41Data; pseudo_model_source=HQER-SFA; error_scenario=hqer_induced; sample_size_label=medium	Wilcoxon signed-rank normal approximation	191	109	0	Positive values favor HQER-SFA.
TE_rank_corr	dataset_source=front41Data; pseudo_model_source=HQER-SFA; error_scenario=hqer_induced; sample_size_label=small	Wilcoxon signed-rank normal approximation	169	131	0	Positive values favor HQER-SFA.
TE_rank_corr	dataset_source=front41Data; pseudo_model_source=HQER-SFA; error_scenario=normal; sample_size_label=medium	Wilcoxon signed-rank normal approximation	246	54	0	Positive values favor HQER-SFA.
TE_rank_corr	dataset_source=front41Data; pseudo_model_source=HQER-SFA; error_scenario=normal; sample_size_label=small	Wilcoxon signed-rank normal approximation	187	113	0	Positive values favor HQER-SFA.
TE_rank_corr	dataset_source=front41Data; pseudo_model_source=HQER-SFA; error_scenario=skewed; sample_size_label=medium	Wilcoxon signed-rank normal approximation	117	183	0	Positive values favor HQER-SFA.
TE_rank_corr	dataset_source=front41Data; pseudo_model_source=HQER-SFA; error_scenario=skewed; sample_size_label=small	Wilcoxon signed-rank normal approximation	158	142	0	Positive values favor HQER-SFA.
TE_rank_corr	dataset_source=front41Data; pseudo_model_source=HQER-SFA; error_scenario=student_t; sample_size_label=medium	Wilcoxon signed-rank normal approximation	191	109	0	Positive values favor HQER-SFA.
TE_rank_corr	dataset_source=front41Data; pseudo_model_source=HQER-SFA; error_scenario=student_t; sample_size_label=small	Wilcoxon signed-rank normal approximation	157	143	0	Positive values favor HQER-SFA.
TE_rank_corr	dataset_source=front41Data; pseudo_model_source=Quantile-SFA; error_scenario=ald; sample_size_label=medium	Wilcoxon signed-rank normal approximation	140	160	0	Positive values favor HQER-SFA.
TE_rank_corr	dataset_source=front41Data; pseudo_model_source=Quantile-SFA; error_scenario=ald; sample_size_label=small	Wilcoxon signed-rank normal approximation	157	143	0	Positive values favor HQER-SFA.
TE_rank_corr	dataset_source=front41Data; pseudo_model_source=Quantile-SFA; error_scenario=hqer_induced; sample_size_label=medium	Wilcoxon signed-rank normal approximation	175	125	0	Positive values favor HQER-SFA.
TE_rank_corr	dataset_source=front41Data; pseudo_model_source=Quantile-SFA; error_scenario=hqer_induced; sample_size_label=small	Wilcoxon signed-rank normal approximation	150	150	0	Positive values favor HQER-SFA.
TE_rank_corr	dataset_source=front41Data; pseudo_model_source=Quantile-SFA; error_scenario=normal; sample_size_label=medium	Wilcoxon signed-rank normal approximation	258	42	0	Positive values favor HQER-SFA.
TE_rank_corr	dataset_source=front41Data; pseudo_model_source=Quantile-SFA; error_scenario=normal; sample_size_label=small	Wilcoxon signed-rank normal approximation	179	121	0	Positive values favor HQER-SFA.
TE_rank_corr	dataset_source=front41Data; pseudo_model_source=Quantile-SFA; error_scenario=skewed; sample_size_label=medium	Wilcoxon signed-rank normal approximation	113	187	0	Positive values favor HQER-SFA.
TE_rank_corr	dataset_source=front41Data; pseudo_model_source=Quantile-SFA; error_scenario=skewed; sample_size_label=small	Wilcoxon signed-rank normal approximation	141	159	0	Positive values favor HQER-SFA.
TE_rank_corr	dataset_source=front41Data; pseudo_model_source=Quantile-SFA; error_scenario=student_t; sample_size_label=medium	Wilcoxon signed-rank normal approximation	206	94	0	Positive values favor HQER-SFA.
TE_rank_corr	dataset_source=front41Data; pseudo_model_source=Quantile-SFA; error_scenario=student_t; sample_size_label=small	Wilcoxon signed-rank normal approximation	173	127	0	Positive values favor HQER-SFA.

Panel 3 of 3

Columns shown: `metric`, `group`, `interpretation`.

metric	group	interpretation
beta_error	overall	Positive paired differences favor HQER-SFA under this design.
beta_error	dataset_source=WDI_agriculture; pseudo_model_source=HQER-SFA; error_scenario=ald; sample_size_label=large	Negative paired differences favor Quantile-SFA under this design.
beta_error	dataset_source=WDI_agriculture; pseudo_model_source=HQER-SFA; error_scenario=ald; sample_size_label=medium	Negative paired differences favor Quantile-SFA under this design.
beta_error	dataset_source=WDI_agriculture; pseudo_model_source=HQER-SFA; error_scenario=hqer_induced; sample_size_label=large	Positive paired differences favor HQER-SFA under this design.
beta_error	dataset_source=WDI_agriculture; pseudo_model_source=HQER-SFA; error_scenario=hqer_induced; sample_size_label=medium	Positive paired differences favor HQER-SFA under this design.
beta_error	dataset_source=WDI_agriculture; pseudo_model_source=HQER-SFA; error_scenario=normal; sample_size_label=large	Positive paired differences favor HQER-SFA under this design.
beta_error	dataset_source=WDI_agriculture; pseudo_model_source=HQER-SFA; error_scenario=normal; sample_size_label=medium	Positive paired differences favor HQER-SFA under this design.
beta_error	dataset_source=WDI_agriculture; pseudo_model_source=HQER-SFA; error_scenario=skewed; sample_size_label=large	Negative paired differences favor Quantile-SFA under this design.
beta_error	dataset_source=WDI_agriculture; pseudo_model_source=HQER-SFA; error_scenario=skewed; sample_size_label=medium	Negative paired differences favor Quantile-SFA under this design.
beta_error	dataset_source=WDI_agriculture; pseudo_model_source=HQER-SFA; error_scenario=student_t; sample_size_label=large	The paired evidence is mixed or not clearly separated from zero.

metric	group	interpretation
beta_error	dataset_source=WDI_agriculture; pseudo_model_source=HQER-SFA; error_scenario=student_t; sample_size_label=medium	The paired evidence is mixed or not clearly separated from zero.
beta_error	dataset_source=WDI_agriculture; pseudo_model_source=Quantile-SFA; error_scenario=ald; sample_size_label=large	The paired evidence is mixed or not clearly separated from zero.
beta_error	dataset_source=WDI_agriculture; pseudo_model_source=Quantile-SFA; error_scenario=ald; sample_size_label=medium	The paired evidence is mixed or not clearly separated from zero.
beta_error	dataset_source=WDI_agriculture; pseudo_model_source=Quantile-SFA; error_scenario=hqer_induced; sample_size_label=large	The paired evidence is mixed or not clearly separated from zero.
beta_error	dataset_source=WDI_agriculture; pseudo_model_source=Quantile-SFA; error_scenario=hqer_induced; sample_size_label=medium	The paired evidence is mixed or not clearly separated from zero.
beta_error	dataset_source=WDI_agriculture; pseudo_model_source=Quantile-SFA; error_scenario=normal; sample_size_label=large	The paired evidence is mixed or not clearly separated from zero.
beta_error	dataset_source=WDI_agriculture; pseudo_model_source=Quantile-SFA; error_scenario=normal; sample_size_label=medium	The paired evidence is mixed or not clearly separated from zero.
beta_error	dataset_source=WDI_agriculture; pseudo_model_source=Quantile-SFA; error_scenario=skewed; sample_size_label=large	The paired evidence is mixed or not clearly separated from zero.
beta_error	dataset_source=WDI_agriculture; pseudo_model_source=Quantile-SFA; error_scenario=skewed; sample_size_label=medium	The paired evidence is mixed or not clearly separated from zero.
beta_error	dataset_source=WDI_agriculture; pseudo_model_source=Quantile-SFA; error_scenario=student_t; sample_size_label=large	The paired evidence is mixed or not clearly separated from zero.
beta_error	dataset_source=WDI_agriculture; pseudo_model_source=Quantile-SFA; error_scenario=student_t; sample_size_label=medium	The paired evidence is mixed or not clearly separated from zero.
beta_error	dataset_source=WDI_industry; pseudo_model_source=HQER-SFA; error_scenario=ald; sample_size_label=large	The paired evidence is mixed or not clearly separated from zero.
beta_error	dataset_source=WDI_industry; pseudo_model_source=HQER-SFA; error_scenario=ald; sample_size_label=medium	The paired evidence is mixed or not clearly separated from zero.
beta_error	dataset_source=WDI_industry; pseudo_model_source=HQER-SFA; error_scenario=hqer_induced; sample_size_label=large	Positive paired differences favor HQER-SFA under this design.
beta_error	dataset_source=WDI_industry; pseudo_model_source=HQER-SFA; error_scenario=hqer_induced; sample_size_label=medium	The paired evidence is mixed or not clearly separated from zero.
beta_error	dataset_source=WDI_industry; pseudo_model_source=HQER-SFA; error_scenario=normal; sample_size_label=large	Positive paired differences favor HQER-SFA under this design.
beta_error	dataset_source=WDI_industry; pseudo_model_source=HQER-SFA; error_scenario=normal; sample_size_label=medium	Positive paired differences favor HQER-SFA under this design.
beta_error	dataset_source=WDI_industry; pseudo_model_source=HQER-SFA; error_scenario=skewed; sample_size_label=large	Negative paired differences favor Quantile-SFA under this design.
beta_error	dataset_source=WDI_industry; pseudo_model_source=HQER-SFA; error_scenario=skewed; sample_size_label=medium	Negative paired differences favor Quantile-SFA under this design.
beta_error	dataset_source=WDI_industry; pseudo_model_source=HQER-SFA; error_scenario=student_t; sample_size_label=large	The paired evidence is mixed or not clearly separated from zero.
beta_error	dataset_source=WDI_industry; pseudo_model_source=HQER-SFA; error_scenario=student_t; sample_size_label=medium	Negative paired differences favor Quantile-SFA under this design.
beta_error	dataset_source=WDI_industry; pseudo_model_source=Quantile-SFA; error_scenario=ald; sample_size_label=large	The paired evidence is mixed or not clearly separated from zero.
beta_error	dataset_source=WDI_industry; pseudo_model_source=Quantile-SFA; error_scenario=ald; sample_size_label=medium	The paired evidence is mixed or not clearly separated from zero.
beta_error	dataset_source=WDI_industry; pseudo_model_source=Quantile-SFA; error_scenario=hqer_induced; sample_size_label=large	The paired evidence is mixed or not clearly separated from zero.
beta_error	dataset_source=WDI_industry; pseudo_model_source=Quantile-SFA; error_scenario=hqer_induced; sample_size_label=medium	The paired evidence is mixed or not clearly separated from zero.
beta_error	dataset_source=WDI_industry; pseudo_model_source=Quantile-SFA; error_scenario=normal; sample_size_label=large	The paired evidence is mixed or not clearly separated from zero.
beta_error	dataset_source=WDI_industry; pseudo_model_source=Quantile-SFA; error_scenario=normal; sample_size_label=medium	The paired evidence is mixed or not clearly separated from zero.
beta_error	dataset_source=WDI_industry; pseudo_model_source=Quantile-SFA; error_scenario=skewed; sample_size_label=large	Negative paired differences favor Quantile-SFA under this design.
beta_error	dataset_source=WDI_industry; pseudo_model_source=Quantile-SFA; error_scenario=skewed; sample_size_label=medium	The paired evidence is mixed or not clearly separated from zero.
beta_error	dataset_source=WDI_industry; pseudo_model_source=Quantile-SFA; error_scenario=student_t; sample_size_label=large	The paired evidence is mixed or not clearly separated from zero.
beta_error	dataset_source=WDI_industry; pseudo_model_source=Quantile-SFA; error_scenario=student_t; sample_size_label=medium	The paired evidence is mixed or not clearly separated from zero.
beta_error	dataset_source=front41Data; pseudo_model_source=HQER-SFA; error_scenario=ald; sample_size_label=medium	Negative paired differences favor Quantile-SFA under this design.
beta_error	dataset_source=front41Data; pseudo_model_source=HQER-SFA; error_scenario=ald; sample_size_label=small	Negative paired differences favor Quantile-SFA under this design.
beta_error	dataset_source=front41Data; pseudo_model_source=HQER-SFA; error_scenario=hqer_induced; sample_size_label=medium	Positive paired differences favor HQER-SFA under this design.
beta_error	dataset_source=front41Data; pseudo_model_source=HQER-SFA; error_scenario=hqer_induced; sample_size_label=small	Negative paired differences favor Quantile-SFA under this design.
beta_error	dataset_source=front41Data; pseudo_model_source=HQER-SFA; error_scenario=normal; sample_size_label=medium	Positive paired differences favor HQER-SFA under this design.
beta_error	dataset_source=front41Data; pseudo_model_source=HQER-SFA; error_scenario=normal; sample_size_label=small	Positive paired differences favor HQER-SFA under this design.
beta_error	dataset_source=front41Data; pseudo_model_source=HQER-SFA; error_scenario=skewed; sample_size_label=medium	Negative paired differences favor Quantile-SFA under this design.
beta_error	dataset_source=front41Data; pseudo_model_source=HQER-SFA; error_scenario=skewed; sample_size_label=small	Negative paired differences favor Quantile-SFA under this design.
beta_error	dataset_source=front41Data; pseudo_model_source=HQER-SFA; error_scenario=student_t; sample_size_label=medium	The paired evidence is mixed or not clearly separated from zero.
beta_error	dataset_source=front41Data; pseudo_model_source=HQER-SFA; error_scenario=student_t; sample_size_label=small	The paired evidence is mixed or not clearly separated from zero.
beta_error	dataset_source=front41Data; pseudo_model_source=Quantile-SFA; error_scenario=ald; sample_size_label=medium	The paired evidence is mixed or not clearly separated from zero.
beta_error	dataset_source=front41Data; pseudo_model_source=Quantile-SFA; error_scenario=ald; sample_size_label=small	The paired evidence is mixed or not clearly separated from zero.
beta_error	dataset_source=front41Data; pseudo_model_source=Quantile-SFA; error_scenario=hqer_induced; sample_size_label=medium	The paired evidence is mixed or not clearly separated from zero.
beta_error	dataset_source=front41Data; pseudo_model_source=Quantile-SFA; error_scenario=hqer_induced; sample_size_label=small	The paired evidence is mixed or not clearly separated from zero.
beta_error	dataset_source=front41Data; pseudo_model_source=Quantile-SFA; error_scenario=normal; sample_size_label=medium	The paired evidence is mixed or not clearly separated from zero.
beta_error	dataset_source=front41Data; pseudo_model_source=Quantile-SFA; error_scenario=normal; sample_size_label=small	The paired evidence is mixed or not clearly separated from zero.
beta_error	dataset_source=front41Data; pseudo_model_source=Quantile-SFA; error_scenario=skewed; sample_size_label=medium	The paired evidence is mixed or not clearly separated from zero.

metric	group	interpretation
beta_error	dataset_source=front41Data; pseudo_model_source=Quantile-SFA; error_scenario=skewed; sample_size_label=small	The paired evidence is mixed or not clearly separated from zero.
beta_error	dataset_source=front41Data; pseudo_model_source=Quantile-SFA; error_scenario=student_t; sample_size_label=medium	The paired evidence is mixed or not clearly separated from zero.
beta_error	dataset_source=front41Data; pseudo_model_source=Quantile-SFA; error_scenario=student_t; sample_size_label=small	The paired evidence is mixed or not clearly separated from zero.
y_rmse	overall	Positive paired differences favor HQER-SFA under this design.
y_rmse	dataset_source=WDI_agriculture; pseudo_model_source=HQER-SFA; error_scenario=ald; sample_size_label=large	Negative paired differences favor Quantile-SFA under this design.
y_rmse	dataset_source=WDI_agriculture; pseudo_model_source=HQER-SFA; error_scenario=ald; sample_size_label=medium	Negative paired differences favor Quantile-SFA under this design.
y_rmse	dataset_source=WDI_agriculture; pseudo_model_source=HQER-SFA; error_scenario=hqer_induced; sample_size_label=large	Positive paired differences favor HQER-SFA under this design.
y_rmse	dataset_source=WDI_agriculture; pseudo_model_source=HQER-SFA; error_scenario=hqer_induced; sample_size_label=medium	Positive paired differences favor HQER-SFA under this design.
y_rmse	dataset_source=WDI_agriculture; pseudo_model_source=HQER-SFA; error_scenario=normal; sample_size_label=large	Positive paired differences favor HQER-SFA under this design.
y_rmse	dataset_source=WDI_agriculture; pseudo_model_source=HQER-SFA; error_scenario=normal; sample_size_label=medium	Positive paired differences favor HQER-SFA under this design.
y_rmse	dataset_source=WDI_agriculture; pseudo_model_source=HQER-SFA; error_scenario=skewed; sample_size_label=large	Negative paired differences favor Quantile-SFA under this design.
y_rmse	dataset_source=WDI_agriculture; pseudo_model_source=HQER-SFA; error_scenario=skewed; sample_size_label=medium	Negative paired differences favor Quantile-SFA under this design.
y_rmse	dataset_source=WDI_agriculture; pseudo_model_source=HQER-SFA; error_scenario=student_t; sample_size_label=large	The paired evidence is mixed or not clearly separated from zero.
y_rmse	dataset_source=WDI_agriculture; pseudo_model_source=HQER-SFA; error_scenario=student_t; sample_size_label=medium	The paired evidence is mixed or not clearly separated from zero.
y_rmse	dataset_source=WDI_agriculture; pseudo_model_source=Quantile-SFA; error_scenario=ald; sample_size_label=large	The paired evidence is mixed or not clearly separated from zero.
y_rmse	dataset_source=WDI_agriculture; pseudo_model_source=Quantile-SFA; error_scenario=ald; sample_size_label=medium	The paired evidence is mixed or not clearly separated from zero.
y_rmse	dataset_source=WDI_agriculture; pseudo_model_source=Quantile-SFA; error_scenario=hqer_induced; sample_size_label=large	The paired evidence is mixed or not clearly separated from zero.
y_rmse	dataset_source=WDI_agriculture; pseudo_model_source=Quantile-SFA; error_scenario=hqer_induced; sample_size_label=medium	The paired evidence is mixed or not clearly separated from zero.
y_rmse	dataset_source=WDI_agriculture; pseudo_model_source=Quantile-SFA; error_scenario=normal; sample_size_label=large	The paired evidence is mixed or not clearly separated from zero.
y_rmse	dataset_source=WDI_agriculture; pseudo_model_source=Quantile-SFA; error_scenario=normal; sample_size_label=medium	The paired evidence is mixed or not clearly separated from zero.
y_rmse	dataset_source=WDI_agriculture; pseudo_model_source=Quantile-SFA; error_scenario=skewed; sample_size_label=large	The paired evidence is mixed or not clearly separated from zero.
y_rmse	dataset_source=WDI_agriculture; pseudo_model_source=Quantile-SFA; error_scenario=skewed; sample_size_label=medium	The paired evidence is mixed or not clearly separated from zero.
y_rmse	dataset_source=WDI_agriculture; pseudo_model_source=Quantile-SFA; error_scenario=student_t; sample_size_label=large	The paired evidence is mixed or not clearly separated from zero.
y_rmse	dataset_source=WDI_agriculture; pseudo_model_source=Quantile-SFA; error_scenario=student_t; sample_size_label=medium	The paired evidence is mixed or not clearly separated from zero.
y_rmse	dataset_source=WDI_industry; pseudo_model_source=HQER-SFA; error_scenario=ald; sample_size_label=large	Negative paired differences favor Quantile-SFA under this design.
y_rmse	dataset_source=WDI_industry; pseudo_model_source=HQER-SFA; error_scenario=ald; sample_size_label=medium	Negative paired differences favor Quantile-SFA under this design.
y_rmse	dataset_source=WDI_industry; pseudo_model_source=HQER-SFA; error_scenario=hqer_induced; sample_size_label=large	The paired evidence is mixed or not clearly separated from zero.
y_rmse	dataset_source=WDI_industry; pseudo_model_source=HQER-SFA; error_scenario=hqer_induced; sample_size_label=medium	The paired evidence is mixed or not clearly separated from zero.
y_rmse	dataset_source=WDI_industry; pseudo_model_source=HQER-SFA; error_scenario=normal; sample_size_label=large	Negative paired differences favor Quantile-SFA under this design.
y_rmse	dataset_source=WDI_industry; pseudo_model_source=HQER-SFA; error_scenario=normal; sample_size_label=medium	The paired evidence is mixed or not clearly separated from zero.
y_rmse	dataset_source=WDI_industry; pseudo_model_source=HQER-SFA; error_scenario=skewed; sample_size_label=large	Negative paired differences favor Quantile-SFA under this design.
y_rmse	dataset_source=WDI_industry; pseudo_model_source=HQER-SFA; error_scenario=skewed; sample_size_label=medium	Negative paired differences favor Quantile-SFA under this design.
y_rmse	dataset_source=WDI_industry; pseudo_model_source=HQER-SFA; error_scenario=student_t; sample_size_label=large	Negative paired differences favor Quantile-SFA under this design.
y_rmse	dataset_source=WDI_industry; pseudo_model_source=HQER-SFA; error_scenario=student_t; sample_size_label=medium	Negative paired differences favor Quantile-SFA under this design.
y_rmse	dataset_source=WDI_industry; pseudo_model_source=Quantile-SFA; error_scenario=ald; sample_size_label=large	The paired evidence is mixed or not clearly separated from zero.
y_rmse	dataset_source=WDI_industry; pseudo_model_source=Quantile-SFA; error_scenario=ald; sample_size_label=medium	The paired evidence is mixed or not clearly separated from zero.
y_rmse	dataset_source=WDI_industry; pseudo_model_source=Quantile-SFA; error_scenario=hqer_induced; sample_size_label=large	The paired evidence is mixed or not clearly separated from zero.
y_rmse	dataset_source=WDI_industry; pseudo_model_source=Quantile-SFA; error_scenario=hqer_induced; sample_size_label=medium	Positive paired differences favor HQER-SFA under this design.
y_rmse	dataset_source=WDI_industry; pseudo_model_source=Quantile-SFA; error_scenario=normal; sample_size_label=large	The paired evidence is mixed or not clearly separated from zero.
y_rmse	dataset_source=WDI_industry; pseudo_model_source=Quantile-SFA; error_scenario=normal; sample_size_label=medium	The paired evidence is mixed or not clearly separated from zero.
y_rmse	dataset_source=WDI_industry; pseudo_model_source=Quantile-SFA; error_scenario=skewed; sample_size_label=large	Negative paired differences favor Quantile-SFA under this design.
y_rmse	dataset_source=WDI_industry; pseudo_model_source=Quantile-SFA; error_scenario=skewed; sample_size_label=medium	The paired evidence is mixed or not clearly separated from zero.
y_rmse	dataset_source=WDI_industry; pseudo_model_source=Quantile-SFA; error_scenario=student_t; sample_size_label=large	The paired evidence is mixed or not clearly separated from zero.
y_rmse	dataset_source=WDI_industry; pseudo_model_source=Quantile-SFA; error_scenario=student_t; sample_size_label=medium	The paired evidence is mixed or not clearly separated from zero.
y_rmse	dataset_source=front41Data; pseudo_model_source=HQER-SFA; error_scenario=ald; sample_size_label=medium	Negative paired differences favor Quantile-SFA under this design.
y_rmse	dataset_source=front41Data; pseudo_model_source=HQER-SFA; error_scenario=ald; sample_size_label=small	Negative paired differences favor Quantile-SFA under this design.
y_rmse	dataset_source=front41Data; pseudo_model_source=HQER-SFA; error_scenario=hqer_induced; sample_size_label=medium	Negative paired differences favor Quantile-SFA under this design.
y_rmse	dataset_source=front41Data; pseudo_model_source=HQER-SFA; error_scenario=hqer_induced; sample_size_label=small	Negative paired differences favor Quantile-SFA under this design.

metric	group	interpretation
y_rmse	dataset_source=front41Data; pseudo_model_source=HQER-SFA; error_scenario=normal; sample_size_label=medium	Positive paired differences favor HQER-SFA under this design.
y_rmse	dataset_source=front41Data; pseudo_model_source=HQER-SFA; error_scenario=normal; sample_size_label=small	Positive paired differences favor HQER-SFA under this design.
y_rmse	dataset_source=front41Data; pseudo_model_source=HQER-SFA; error_scenario=skewed; sample_size_label=medium	Negative paired differences favor Quantile-SFA under this design.
y_rmse	dataset_source=front41Data; pseudo_model_source=HQER-SFA; error_scenario=skewed; sample_size_label=small	Negative paired differences favor Quantile-SFA under this design.
y_rmse	dataset_source=front41Data; pseudo_model_source=HQER-SFA; error_scenario=student_t; sample_size_label=medium	The paired evidence is mixed or not clearly separated from zero.
y_rmse	dataset_source=front41Data; pseudo_model_source=HQER-SFA; error_scenario=student_t; sample_size_label=small	The paired evidence is mixed or not clearly separated from zero.
y_rmse	dataset_source=front41Data; pseudo_model_source=Quantile-SFA; error_scenario=ald; sample_size_label=medium	The paired evidence is mixed or not clearly separated from zero.
y_rmse	dataset_source=front41Data; pseudo_model_source=Quantile-SFA; error_scenario=ald; sample_size_label=small	The paired evidence is mixed or not clearly separated from zero.
y_rmse	dataset_source=front41Data; pseudo_model_source=Quantile-SFA; error_scenario=hqer_induced; sample_size_label=medium	The paired evidence is mixed or not clearly separated from zero.
y_rmse	dataset_source=front41Data; pseudo_model_source=Quantile-SFA; error_scenario=hqer_induced; sample_size_label=small	The paired evidence is mixed or not clearly separated from zero.
y_rmse	dataset_source=front41Data; pseudo_model_source=Quantile-SFA; error_scenario=normal; sample_size_label=medium	The paired evidence is mixed or not clearly separated from zero.
y_rmse	dataset_source=front41Data; pseudo_model_source=Quantile-SFA; error_scenario=normal; sample_size_label=small	The paired evidence is mixed or not clearly separated from zero.
y_rmse	dataset_source=front41Data; pseudo_model_source=Quantile-SFA; error_scenario=skewed; sample_size_label=medium	The paired evidence is mixed or not clearly separated from zero.
y_rmse	dataset_source=front41Data; pseudo_model_source=Quantile-SFA; error_scenario=skewed; sample_size_label=small	The paired evidence is mixed or not clearly separated from zero.
y_rmse	dataset_source=front41Data; pseudo_model_source=Quantile-SFA; error_scenario=student_t; sample_size_label=medium	The paired evidence is mixed or not clearly separated from zero.
y_rmse	dataset_source=front41Data; pseudo_model_source=Quantile-SFA; error_scenario=student_t; sample_size_label=small	The paired evidence is mixed or not clearly separated from zero.
TE_RMSE	overall	Positive paired differences favor HQER-SFA under this design.
TE_RMSE	dataset_source=WDI_agriculture; pseudo_model_source=HQER-SFA; error_scenario=ald; sample_size_label=large	Negative paired differences favor Quantile-SFA under this design.
TE_RMSE	dataset_source=WDI_agriculture; pseudo_model_source=HQER-SFA; error_scenario=ald; sample_size_label=medium	Negative paired differences favor Quantile-SFA under this design.
TE_RMSE	dataset_source=WDI_agriculture; pseudo_model_source=HQER-SFA; error_scenario=hqer_induced; sample_size_label=large	Positive paired differences favor HQER-SFA under this design.
TE_RMSE	dataset_source=WDI_agriculture; pseudo_model_source=HQER-SFA; error_scenario=hqer_induced; sample_size_label=medium	Positive paired differences favor HQER-SFA under this design.
TE_RMSE	dataset_source=WDI_agriculture; pseudo_model_source=HQER-SFA; error_scenario=normal; sample_size_label=large	Positive paired differences favor HQER-SFA under this design.
TE_RMSE	dataset_source=WDI_agriculture; pseudo_model_source=HQER-SFA; error_scenario=normal; sample_size_label=medium	Positive paired differences favor HQER-SFA under this design.
TE_RMSE	dataset_source=WDI_agriculture; pseudo_model_source=HQER-SFA; error_scenario=skewed; sample_size_label=large	Positive paired differences favor HQER-SFA under this design.
TE_RMSE	dataset_source=WDI_agriculture; pseudo_model_source=HQER-SFA; error_scenario=skewed; sample_size_label=medium	Positive paired differences favor HQER-SFA under this design.
TE_RMSE	dataset_source=WDI_agriculture; pseudo_model_source=HQER-SFA; error_scenario=student_t; sample_size_label=large	The paired evidence is mixed or not clearly separated from zero.
TE_RMSE	dataset_source=WDI_agriculture; pseudo_model_source=HQER-SFA; error_scenario=student_t; sample_size_label=medium	The paired evidence is mixed or not clearly separated from zero.
TE_RMSE	dataset_source=WDI_agriculture; pseudo_model_source=Quantile-SFA; error_scenario=ald; sample_size_label=large	The paired evidence is mixed or not clearly separated from zero.
TE_RMSE	dataset_source=WDI_agriculture; pseudo_model_source=Quantile-SFA; error_scenario=ald; sample_size_label=medium	The paired evidence is mixed or not clearly separated from zero.
TE_RMSE	dataset_source=WDI_agriculture; pseudo_model_source=Quantile-SFA; error_scenario=hqer_induced; sample_size_label=large	The paired evidence is mixed or not clearly separated from zero.
TE_RMSE	dataset_source=WDI_agriculture; pseudo_model_source=Quantile-SFA; error_scenario=hqer_induced; sample_size_label=medium	The paired evidence is mixed or not clearly separated from zero.
TE_RMSE	dataset_source=WDI_agriculture; pseudo_model_source=Quantile-SFA; error_scenario=normal; sample_size_label=large	The paired evidence is mixed or not clearly separated from zero.
TE_RMSE	dataset_source=WDI_agriculture; pseudo_model_source=Quantile-SFA; error_scenario=normal; sample_size_label=medium	The paired evidence is mixed or not clearly separated from zero.
TE_RMSE	dataset_source=WDI_agriculture; pseudo_model_source=Quantile-SFA; error_scenario=skewed; sample_size_label=large	The paired evidence is mixed or not clearly separated from zero.
TE_RMSE	dataset_source=WDI_agriculture; pseudo_model_source=Quantile-SFA; error_scenario=skewed; sample_size_label=medium	The paired evidence is mixed or not clearly separated from zero.
TE_RMSE	dataset_source=WDI_agriculture; pseudo_model_source=Quantile-SFA; error_scenario=student_t; sample_size_label=large	The paired evidence is mixed or not clearly separated from zero.
TE_RMSE	dataset_source=WDI_agriculture; pseudo_model_source=Quantile-SFA; error_scenario=student_t; sample_size_label=medium	The paired evidence is mixed or not clearly separated from zero.
TE_RMSE	dataset_source=WDI_industry; pseudo_model_source=HQER-SFA; error_scenario=ald; sample_size_label=large	The paired evidence is mixed or not clearly separated from zero.
TE_RMSE	dataset_source=WDI_industry; pseudo_model_source=HQER-SFA; error_scenario=ald; sample_size_label=medium	The paired evidence is mixed or not clearly separated from zero.
TE_RMSE	dataset_source=WDI_industry; pseudo_model_source=HQER-SFA; error_scenario=hqer_induced; sample_size_label=large	Positive paired differences favor HQER-SFA under this design.
TE_RMSE	dataset_source=WDI_industry; pseudo_model_source=HQER-SFA; error_scenario=hqer_induced; sample_size_label=medium	Positive paired differences favor HQER-SFA under this design.
TE_RMSE	dataset_source=WDI_industry; pseudo_model_source=HQER-SFA; error_scenario=normal; sample_size_label=large	Positive paired differences favor HQER-SFA under this design.
TE_RMSE	dataset_source=WDI_industry; pseudo_model_source=HQER-SFA; error_scenario=normal; sample_size_label=medium	Positive paired differences favor HQER-SFA under this design.
TE_RMSE	dataset_source=WDI_industry; pseudo_model_source=HQER-SFA; error_scenario=skewed; sample_size_label=large	Positive paired differences favor HQER-SFA under this design.
TE_RMSE	dataset_source=WDI_industry; pseudo_model_source=HQER-SFA; error_scenario=skewed; sample_size_label=medium	Positive paired differences favor HQER-SFA under this design.
TE_RMSE	dataset_source=WDI_industry; pseudo_model_source=HQER-SFA; error_scenario=student_t; sample_size_label=large	Negative paired differences favor Quantile-SFA under this design.
TE_RMSE	dataset_source=WDI_industry; pseudo_model_source=HQER-SFA; error_scenario=student_t; sample_size_label=medium	Negative paired differences favor Quantile-SFA under this design.
TE_RMSE	dataset_source=WDI_industry; pseudo_model_source=Quantile-SFA; error_scenario=ald; sample_size_label=large	The paired evidence is mixed or not clearly separated from zero.

metric	group	interpretation
TE_RMSE	dataset_source=WDI_industry; pseudo_model_source=Quantile-SFA; error_scenario=ald; sample_size_label=medium	The paired evidence is mixed or not clearly separated from zero.
TE_RMSE	dataset_source=WDI_industry; pseudo_model_source=Quantile-SFA; error_scenario=hqer_induced; sample_size_label=large	The paired evidence is mixed or not clearly separated from zero.
TE_RMSE	dataset_source=WDI_industry; pseudo_model_source=Quantile-SFA; error_scenario=hqer_induced; sample_size_label=medium	The paired evidence is mixed or not clearly separated from zero.
TE_RMSE	dataset_source=WDI_industry; pseudo_model_source=Quantile-SFA; error_scenario=normal; sample_size_label=large	The paired evidence is mixed or not clearly separated from zero.
TE_RMSE	dataset_source=WDI_industry; pseudo_model_source=Quantile-SFA; error_scenario=normal; sample_size_label=medium	The paired evidence is mixed or not clearly separated from zero.
TE_RMSE	dataset_source=WDI_industry; pseudo_model_source=Quantile-SFA; error_scenario=skewed; sample_size_label=large	Positive paired differences favor HQER-SFA under this design.
TE_RMSE	dataset_source=WDI_industry; pseudo_model_source=Quantile-SFA; error_scenario=skewed; sample_size_label=medium	The paired evidence is mixed or not clearly separated from zero.
TE_RMSE	dataset_source=WDI_industry; pseudo_model_source=Quantile-SFA; error_scenario=student_t; sample_size_label=large	Negative paired differences favor Quantile-SFA under this design.
TE_RMSE	dataset_source=WDI_industry; pseudo_model_source=Quantile-SFA; error_scenario=student_t; sample_size_label=medium	Negative paired differences favor Quantile-SFA under this design.
TE_RMSE	dataset_source=front41Data; pseudo_model_source=HQER-SFA; error_scenario=ald; sample_size_label=medium	Positive paired differences favor HQER-SFA under this design.
TE_RMSE	dataset_source=front41Data; pseudo_model_source=HQER-SFA; error_scenario=ald; sample_size_label=small	The paired evidence is mixed or not clearly separated from zero.
TE_RMSE	dataset_source=front41Data; pseudo_model_source=HQER-SFA; error_scenario=hqer_induced; sample_size_label=medium	Positive paired differences favor HQER-SFA under this design.
TE_RMSE	dataset_source=front41Data; pseudo_model_source=HQER-SFA; error_scenario=hqer_induced; sample_size_label=small	Positive paired differences favor HQER-SFA under this design.
TE_RMSE	dataset_source=front41Data; pseudo_model_source=HQER-SFA; error_scenario=normal; sample_size_label=medium	Positive paired differences favor HQER-SFA under this design.
TE_RMSE	dataset_source=front41Data; pseudo_model_source=HQER-SFA; error_scenario=normal; sample_size_label=small	Positive paired differences favor HQER-SFA under this design.
TE_RMSE	dataset_source=front41Data; pseudo_model_source=HQER-SFA; error_scenario=skewed; sample_size_label=medium	The paired evidence is mixed or not clearly separated from zero.
TE_RMSE	dataset_source=front41Data; pseudo_model_source=HQER-SFA; error_scenario=skewed; sample_size_label=small	The paired evidence is mixed or not clearly separated from zero.
TE_RMSE	dataset_source=front41Data; pseudo_model_source=HQER-SFA; error_scenario=student_t; sample_size_label=medium	Negative paired differences favor Quantile-SFA under this design.
TE_RMSE	dataset_source=front41Data; pseudo_model_source=HQER-SFA; error_scenario=student_t; sample_size_label=small	The paired evidence is mixed or not clearly separated from zero.
TE_RMSE	dataset_source=front41Data; pseudo_model_source=Quantile-SFA; error_scenario=ald; sample_size_label=medium	The paired evidence is mixed or not clearly separated from zero.
TE_RMSE	dataset_source=front41Data; pseudo_model_source=Quantile-SFA; error_scenario=ald; sample_size_label=small	The paired evidence is mixed or not clearly separated from zero.
TE_RMSE	dataset_source=front41Data; pseudo_model_source=Quantile-SFA; error_scenario=hqer_induced; sample_size_label=medium	The paired evidence is mixed or not clearly separated from zero.
TE_RMSE	dataset_source=front41Data; pseudo_model_source=Quantile-SFA; error_scenario=hqer_induced; sample_size_label=small	The paired evidence is mixed or not clearly separated from zero.
TE_RMSE	dataset_source=front41Data; pseudo_model_source=Quantile-SFA; error_scenario=normal; sample_size_label=medium	The paired evidence is mixed or not clearly separated from zero.
TE_RMSE	dataset_source=front41Data; pseudo_model_source=Quantile-SFA; error_scenario=normal; sample_size_label=small	The paired evidence is mixed or not clearly separated from zero.
TE_RMSE	dataset_source=front41Data; pseudo_model_source=Quantile-SFA; error_scenario=skewed; sample_size_label=medium	The paired evidence is mixed or not clearly separated from zero.
TE_RMSE	dataset_source=front41Data; pseudo_model_source=Quantile-SFA; error_scenario=skewed; sample_size_label=small	The paired evidence is mixed or not clearly separated from zero.
TE_RMSE	dataset_source=front41Data; pseudo_model_source=Quantile-SFA; error_scenario=student_t; sample_size_label=medium	The paired evidence is mixed or not clearly separated from zero.
TE_RMSE	dataset_source=front41Data; pseudo_model_source=Quantile-SFA; error_scenario=student_t; sample_size_label=small	The paired evidence is mixed or not clearly separated from zero.
TE_rank_corr	overall	Positive paired differences favor HQER-SFA under this design.
TE_rank_corr	dataset_source=WDI_agriculture; pseudo_model_source=HQER-SFA; error_scenario=ald; sample_size_label=large	Negative paired differences favor Quantile-SFA under this design.
TE_rank_corr	dataset_source=WDI_agriculture; pseudo_model_source=HQER-SFA; error_scenario=ald; sample_size_label=medium	The paired evidence is mixed or not clearly separated from zero.
TE_rank_corr	dataset_source=WDI_agriculture; pseudo_model_source=HQER-SFA; error_scenario=hqer_induced; sample_size_label=large	Positive paired differences favor HQER-SFA under this design.
TE_rank_corr	dataset_source=WDI_agriculture; pseudo_model_source=HQER-SFA; error_scenario=hqer_induced; sample_size_label=medium	Positive paired differences favor HQER-SFA under this design.
TE_rank_corr	dataset_source=WDI_agriculture; pseudo_model_source=HQER-SFA; error_scenario=normal; sample_size_label=large	Positive paired differences favor HQER-SFA under this design.
TE_rank_corr	dataset_source=WDI_agriculture; pseudo_model_source=HQER-SFA; error_scenario=normal; sample_size_label=medium	Positive paired differences favor HQER-SFA under this design.
TE_rank_corr	dataset_source=WDI_agriculture; pseudo_model_source=HQER-SFA; error_scenario=skewed; sample_size_label=large	Positive paired differences favor HQER-SFA under this design.
TE_rank_corr	dataset_source=WDI_agriculture; pseudo_model_source=HQER-SFA; error_scenario=skewed; sample_size_label=medium	Positive paired differences favor HQER-SFA under this design.
TE_rank_corr	dataset_source=WDI_agriculture; pseudo_model_source=HQER-SFA; error_scenario=student_t; sample_size_label=large	Positive paired differences favor HQER-SFA under this design.
TE_rank_corr	dataset_source=WDI_agriculture; pseudo_model_source=HQER-SFA; error_scenario=student_t; sample_size_label=medium	Positive paired differences favor HQER-SFA under this design.
TE_rank_corr	dataset_source=WDI_agriculture; pseudo_model_source=Quantile-SFA; error_scenario=ald; sample_size_label=large	Negative paired differences favor Quantile-SFA under this design.
TE_rank_corr	dataset_source=WDI_agriculture; pseudo_model_source=Quantile-SFA; error_scenario=ald; sample_size_label=medium	The paired evidence is mixed or not clearly separated from zero.
TE_rank_corr	dataset_source=WDI_agriculture; pseudo_model_source=Quantile-SFA; error_scenario=hqer_induced; sample_size_label=large	Positive paired differences favor HQER-SFA under this design.
TE_rank_corr	dataset_source=WDI_agriculture; pseudo_model_source=Quantile-SFA; error_scenario=hqer_induced; sample_size_label=medium	Positive paired differences favor HQER-SFA under this design.
TE_rank_corr	dataset_source=WDI_agriculture; pseudo_model_source=Quantile-SFA; error_scenario=normal; sample_size_label=large	Positive paired differences favor HQER-SFA under this design.
TE_rank_corr	dataset_source=WDI_agriculture; pseudo_model_source=Quantile-SFA; error_scenario=normal; sample_size_label=medium	Positive paired differences favor HQER-SFA under this design.
TE_rank_corr	dataset_source=WDI_agriculture; pseudo_model_source=Quantile-SFA; error_scenario=skewed; sample_size_label=large	Positive paired differences favor HQER-SFA under this design.
TE_rank_corr	dataset_source=WDI_agriculture; pseudo_model_source=Quantile-SFA; error_scenario=skewed; sample_size_label=medium	Positive paired differences favor HQER-SFA under this design.

metric	group	interpretation
TE_rank_corr	dataset_source=WDI_agriculture; pseudo_model_source=Quantile-SFA; error_scenario=student_t; sample_size_label=large	The paired evidence is mixed or not clearly separated from zero.
TE_rank_corr	dataset_source=WDI_agriculture; pseudo_model_source=Quantile-SFA; error_scenario=student_t; sample_size_label=medium	The paired evidence is mixed or not clearly separated from zero.
TE_rank_corr	dataset_source=WDI_industry; pseudo_model_source=HQER-SFA; error_scenario=ald; sample_size_label=large	Negative paired differences favor Quantile-SFA under this design.
TE_rank_corr	dataset_source=WDI_industry; pseudo_model_source=HQER-SFA; error_scenario=ald; sample_size_label=medium	Negative paired differences favor Quantile-SFA under this design.
TE_rank_corr	dataset_source=WDI_industry; pseudo_model_source=HQER-SFA; error_scenario=hqer_induced; sample_size_label=large	Negative paired differences favor Quantile-SFA under this design.
TE_rank_corr	dataset_source=WDI_industry; pseudo_model_source=HQER-SFA; error_scenario=hqer_induced; sample_size_label=medium	Negative paired differences favor Quantile-SFA under this design.
TE_rank_corr	dataset_source=WDI_industry; pseudo_model_source=HQER-SFA; error_scenario=normal; sample_size_label=large	Positive paired differences favor HQER-SFA under this design.
TE_rank_corr	dataset_source=WDI_industry; pseudo_model_source=HQER-SFA; error_scenario=normal; sample_size_label=medium	Positive paired differences favor HQER-SFA under this design.
TE_rank_corr	dataset_source=WDI_industry; pseudo_model_source=HQER-SFA; error_scenario=skewed; sample_size_label=large	Negative paired differences favor Quantile-SFA under this design.
TE_rank_corr	dataset_source=WDI_industry; pseudo_model_source=HQER-SFA; error_scenario=skewed; sample_size_label=medium	Negative paired differences favor Quantile-SFA under this design.
TE_rank_corr	dataset_source=WDI_industry; pseudo_model_source=HQER-SFA; error_scenario=student_t; sample_size_label=large	Positive paired differences favor HQER-SFA under this design.
TE_rank_corr	dataset_source=WDI_industry; pseudo_model_source=HQER-SFA; error_scenario=student_t; sample_size_label=medium	Positive paired differences favor HQER-SFA under this design.
TE_rank_corr	dataset_source=WDI_industry; pseudo_model_source=Quantile-SFA; error_scenario=ald; sample_size_label=large	Negative paired differences favor Quantile-SFA under this design.
TE_rank_corr	dataset_source=WDI_industry; pseudo_model_source=Quantile-SFA; error_scenario=ald; sample_size_label=medium	Negative paired differences favor Quantile-SFA under this design.
TE_rank_corr	dataset_source=WDI_industry; pseudo_model_source=Quantile-SFA; error_scenario=hqer_induced; sample_size_label=large	Negative paired differences favor Quantile-SFA under this design.
TE_rank_corr	dataset_source=WDI_industry; pseudo_model_source=Quantile-SFA; error_scenario=hqer_induced; sample_size_label=medium	Negative paired differences favor Quantile-SFA under this design.
TE_rank_corr	dataset_source=WDI_industry; pseudo_model_source=Quantile-SFA; error_scenario=normal; sample_size_label=large	Positive paired differences favor HQER-SFA under this design.
TE_rank_corr	dataset_source=WDI_industry; pseudo_model_source=Quantile-SFA; error_scenario=normal; sample_size_label=medium	Positive paired differences favor HQER-SFA under this design.
TE_rank_corr	dataset_source=WDI_industry; pseudo_model_source=Quantile-SFA; error_scenario=skewed; sample_size_label=large	Negative paired differences favor Quantile-SFA under this design.
TE_rank_corr	dataset_source=WDI_industry; pseudo_model_source=Quantile-SFA; error_scenario=skewed; sample_size_label=medium	Negative paired differences favor Quantile-SFA under this design.
TE_rank_corr	dataset_source=WDI_industry; pseudo_model_source=Quantile-SFA; error_scenario=student_t; sample_size_label=large	Positive paired differences favor HQER-SFA under this design.
TE_rank_corr	dataset_source=WDI_industry; pseudo_model_source=Quantile-SFA; error_scenario=student_t; sample_size_label=medium	Positive paired differences favor HQER-SFA under this design.
TE_rank_corr	dataset_source=front41Data; pseudo_model_source=HQER-SFA; error_scenario=ald; sample_size_label=medium	Negative paired differences favor Quantile-SFA under this design.
TE_rank_corr	dataset_source=front41Data; pseudo_model_source=HQER-SFA; error_scenario=ald; sample_size_label=small	The paired evidence is mixed or not clearly separated from zero.
TE_rank_corr	dataset_source=front41Data; pseudo_model_source=HQER-SFA; error_scenario=hqer_induced; sample_size_label=medium	Positive paired differences favor HQER-SFA under this design.
TE_rank_corr	dataset_source=front41Data; pseudo_model_source=HQER-SFA; error_scenario=hqer_induced; sample_size_label=small	The paired evidence is mixed or not clearly separated from zero.
TE_rank_corr	dataset_source=front41Data; pseudo_model_source=HQER-SFA; error_scenario=normal; sample_size_label=medium	Positive paired differences favor HQER-SFA under this design.
TE_rank_corr	dataset_source=front41Data; pseudo_model_source=HQER-SFA; error_scenario=normal; sample_size_label=small	Positive paired differences favor HQER-SFA under this design.
TE_rank_corr	dataset_source=front41Data; pseudo_model_source=HQER-SFA; error_scenario=skewed; sample_size_label=medium	Negative paired differences favor Quantile-SFA under this design.
TE_rank_corr	dataset_source=front41Data; pseudo_model_source=HQER-SFA; error_scenario=skewed; sample_size_label=small	The paired evidence is mixed or not clearly separated from zero.
TE_rank_corr	dataset_source=front41Data; pseudo_model_source=HQER-SFA; error_scenario=student_t; sample_size_label=medium	Positive paired differences favor HQER-SFA under this design.
TE_rank_corr	dataset_source=front41Data; pseudo_model_source=HQER-SFA; error_scenario=student_t; sample_size_label=small	The paired evidence is mixed or not clearly separated from zero.
TE_rank_corr	dataset_source=front41Data; pseudo_model_source=Quantile-SFA; error_scenario=ald; sample_size_label=medium	The paired evidence is mixed or not clearly separated from zero.
TE_rank_corr	dataset_source=front41Data; pseudo_model_source=Quantile-SFA; error_scenario=ald; sample_size_label=small	The paired evidence is mixed or not clearly separated from zero.
TE_rank_corr	dataset_source=front41Data; pseudo_model_source=Quantile-SFA; error_scenario=hqer_induced; sample_size_label=medium	Positive paired differences favor HQER-SFA under this design.
TE_rank_corr	dataset_source=front41Data; pseudo_model_source=Quantile-SFA; error_scenario=hqer_induced; sample_size_label=small	The paired evidence is mixed or not clearly separated from zero.
TE_rank_corr	dataset_source=front41Data; pseudo_model_source=Quantile-SFA; error_scenario=normal; sample_size_label=medium	Positive paired differences favor HQER-SFA under this design.
TE_rank_corr	dataset_source=front41Data; pseudo_model_source=Quantile-SFA; error_scenario=normal; sample_size_label=small	Positive paired differences favor HQER-SFA under this design.
TE_rank_corr	dataset_source=front41Data; pseudo_model_source=Quantile-SFA; error_scenario=skewed; sample_size_label=medium	Negative paired differences favor Quantile-SFA under this design.
TE_rank_corr	dataset_source=front41Data; pseudo_model_source=Quantile-SFA; error_scenario=skewed; sample_size_label=small	The paired evidence is mixed or not clearly separated from zero.
TE_rank_corr	dataset_source=front41Data; pseudo_model_source=Quantile-SFA; error_scenario=student_t; sample_size_label=medium	Positive paired differences favor HQER-SFA under this design.
TE_rank_corr	dataset_source=front41Data; pseudo_model_source=Quantile-SFA; error_scenario=student_t; sample_size_label=small	Positive paired differences favor HQER-SFA under this design.
